# Comprehensive analysis of β-catenin target genes in colorectal carcinoma cell lines with deregulated Wnt/β-catenin signaling

**DOI:** 10.1186/1471-2164-15-74

**Published:** 2014-01-28

**Authors:** Andreas Herbst, Vindi Jurinovic, Stefan Krebs, Susanne E Thieme, Helmut Blum, Burkhard Göke, Frank T Kolligs

**Affiliations:** 1 Department of Medicine II, University of Munich, Marchioninistrasse 15, 81377 Munich, Germany; 2 Institute for Medical Informatics, Biometry and Epidemiology, 81377 Munich, Germany; 3 Laboratory for Functional Genome Analysis (LAFUGA), Gene Center, University of Munich, 81377 Munich, Germany

**Keywords:** Colorectal cancer, β-catenin, Target genes, DLD1, SW480, LS174T, DNA microarray

## Abstract

**Background:**

Deregulation of Wnt/β-catenin signaling is a hallmark of the majority of sporadic forms of colorectal cancer and results in increased stability of the protein β-catenin. β-catenin is then shuttled into the nucleus where it activates the transcription of its target genes, including the proto-oncogenes MYC and CCND1 as well as the genes encoding the basic helix-loop-helix (bHLH) proteins ASCL2 and ITF-2B. To identify genes commonly regulated by β-catenin in colorectal cancer cell lines, we analyzed β-catenin target gene expression in two non-isogenic cell lines, DLD1 and SW480, using DNA microarrays and compared these genes to β-catenin target genes published in the PubMed database and DNA microarray data presented in the Gene Expression Omnibus (GEO) database.

**Results:**

Treatment of DLD1 and SW480 cells with β-catenin siRNA resulted in differential expression of 1501 and 2389 genes, respectively. 335 of these genes were regulated in the same direction in both cell lines. Comparison of these data with published β-catenin target genes for the colon carcinoma cell line LS174T revealed 193 genes that are regulated similarly in all three cell lines. The overlapping gene set includes confirmed β-catenin target genes like AXIN2, MYC, and ASCL2. We also identified 11 Kyoto Encyclopedia of Genes and Genomes (KEGG) pathways that are regulated similarly in DLD1 and SW480 cells and one pathway – the steroid biosynthesis pathway – was regulated in all three cell lines.

**Conclusions:**

Based on the large number of potential β-catenin target genes found to be similarly regulated in DLD1, SW480 and LS174T cells as well as the large overlap with confirmed β-catenin target genes, we conclude that DLD1 and SW480 colon carcinoma cell lines are suitable model systems to study Wnt/β-catenin signaling and associated colorectal carcinogenesis. Furthermore, the confirmed and the newly identified potential β-catenin target genes are useful starting points for further studies.

## Background

The majority of sporadic forms of colorectal cancer are characterized by deregulation of Wnt/β-catenin signaling resulting in increased transcriptional activity of the protein β-catenin. In colon cells, a protein complex consisting of the protein Adenomatous polyposis coli (APC), the scaffolding protein Axin, casein kinase 1 (CK-1) and glycogen synthase kinase 3β (GSK-3β) tightly controls the transcriptional activity of the β-catenin protein by targeting it for proteasomal degradation. Stimulation of colon cells with Wnt proteins results in the disruption of the APC protein complex and stabilization of the β-catenin protein. β-catenin is then transported into the nucleus where it recruits cofactors of the TCF/LEF family to activate the transcription of its target genes [1]. In colorectal tumor cells, the tumor suppressor gene APC is frequently affected by loss of heterozygosity (LOH) as well as mutations, resulting in inactivation of the APC protein complex targeting β-catenin for degradation [2,3]. Consequently, β-catenin activity is deregulated and promotes aberrant transcription of its target genes [1].

β-catenin target genes have been implicated in regulating different cellular processes including proliferation (e.g., MYC, CCND1, PPARD), stem cell fate (ASCL2), survival (ABCB1, BIRC5), differentiation (ID2, ITF2, ENC1), migration (MMP7, MMP14), and angiogenesis (VEGF) [4-18]. While these β-catenin functions play an important role in embryonic development and tissue homeostasis, they can also contribute to the initiation and progression of colon cancer. In particular, deregulation of genes involved in proliferation and migration is frequently observed in colorectal carcinomas [5-7,14-16].

Genes encoding members of the basic helix-loop-helix (bHLH) family of proteins, like ASCL2, HATH1, and ITF2, represent one of the largest groups of genes on the list of previously published β-catenin target genes [8,19,20]. Members of the bHLH protein family are characterized by the presence of a basic DNA binding domain and a HLH protein-protein interaction domain. Based on their primary sequence, bHLH proteins are grouped into seven classes and are known to form homo- and heterodimers to regulate their functions [21]. For example, the class I bHLH protein ITF-2B encoded by the gene ITF2 is known to interact with class II and class V bHLH proteins [22].

The colon carcinoma cell lines, DLD1 and SW480, are frequently used as model systems to study colorectal carcinogenesis in general and β-catenin function in particular. To identify target genes that are commonly regulated by β-catenin, we chose these non-isogenic colorectal cancer cell lines for our experiments. While DLD1 cells are derived from a microsatellite instable (MSI) tumor, SW480 tumor cells are characterized by chromosomal instability (CIN) [23]. In both cell lines the APC gene is affected by LOH on chromosome 5 and contains inactivating mutations in the remaining APC allele [24]. In contrast, the MSI colon carcinoma cell line LS174T –that was used for validation purposes– expresses wild-type APC and a mutated β-catenin protein [23,24].

Here, we analyzed the gene expression profiles of DLD1 and SW480 cells after down regulation of β-catenin expression and compared the potential β-catenin target genes that are similarly regulated in DLD1 and SW480 cells with a comparable data set published for LS174T cells in the Gene Expression Omnibus (GEO) database as well as confirmed β-catenin target genes found in the PubMed database. We also identified 11 Kyoto Encyclopedia of Genes and Genomes (KEGG) pathways that are regulated similarly in DLD1 and SW480 cells. In addition, one KEGG pathway was regulated in all three cell lines. Based on the large number of genes that are regulated in a similar way in DLD1, SW480, and LS174T cells as well as the large overlap with previously published β-catenin target genes, we conclude that DLD1 and SW480 are good model systems to study β-catenin target genes and signaling pathways. Furthermore, we were able to confirm a large number of previously published β-catenin target genes and identified many new potential β-catenin target genes. Therefore, this data is a useful resource for further studies to elucidate the role of β-catenin signaling in colorectal cancer.

## Results

### Identification of β-catenin target genes in DLD1 and SW480 colon carcinoma cells

**Figure 1:**
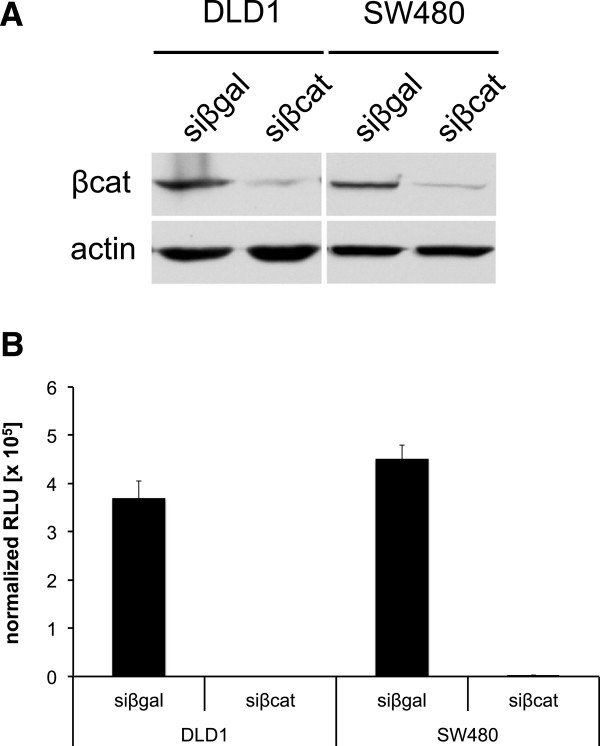
**Validation of β-catenin siRNA.** DLD1 and SW480 cells were treated with β-catenin siRNA (siβcat) or β-galactosidase siRNA (siβgal) and the protein expression levels of β-catenin were determined by immuno detection using an anti-β-catenin antibody. Actin was used as a loading control **(A)**. The activity of the β-catenin dependent reporter gene 8×TOPflash was determined in DLD1 and SW480 cells after treatment of these cells with β-catenin siRNA or β-galactosidase siRNA. The experiments were performed in duplicates with error bars representing standard deviation **(B)**.

To test the efficacy of the β-catenin siRNA used for our DNA microarray experiments, DLD1 and SW480 cells were treated with this β-catenin siRNA and β-catenin protein expression was analyzed using immuno detection. Since we observed the strongest down regulation of β-catenin protein after siRNA treatment for 48 hrs, cells were treated for this period of time. Compared to DLD1 and SW480 cells treated with control siRNA (siβgal), treatment with β-catenin siRNA (siβcat) attenuated β-catenin protein expression levels in both cell lines (Figure 1A). Similarly, treatment of DLD1 and SW480 cells with β-catenin siRNA strongly reduced the activity of the β-catenin dependent 8×TOPflash reporter gene construct, confirming the efficacy of the β-catenin siRNA (Figure 1B).

**Figure 2:**
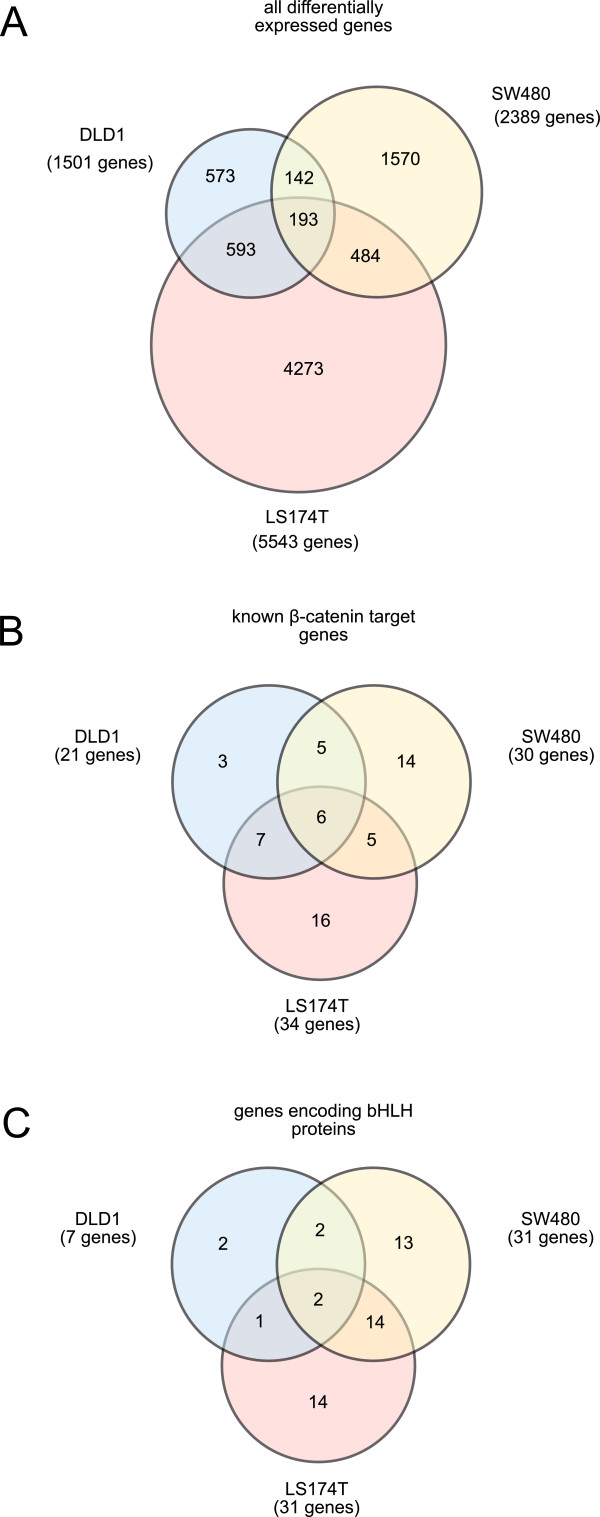
**Identification of β-catenin target genes.** DLD1 and SW480 cells were treated with β-catenin siRNA and differentially expressed genes were identified using a DNA microarray. The corresponding data for LS174T cells was published earlier and used for comparison. The Venn-like diagrams display the number of differentially expressed genes when analyzing all differentially expressed genes **(A)**, previously described β-catenin target genes listed in Table 1**(B)**, or 96 genes encoding basic helix-loop-helix (bHLH) proteins (PFAM family PF00010) **(C)**. The names of the genes corresponding to the seven sections of each Venn-like diagram are listed in separate tabs in the Additional files 1, 2 and 3.

To identify commonly regulated β-catenin target genes in colorectal cancer cell lines, we analyzed the expression of β-catenin target genes in DLD1 and SW480 cells after siRNA treatment of these cells using DNA microarrays. Treatment of DLD1 and SW480 cells with siRNA targeting β-catenin resulted in differential expression of 1501 and 2389 genes, respectively (Figure 2A and Additional file 1, tab “DLD1 complete” and “SW480_complete”). The intersecting set comprised 335 genes, which are regulated in the same direction in both cell lines (Figure 2A and Additional file 1, tab “335 target genes”). We compared the lists of potential β-catenin target genes obtained for DLD1 and SW480 cells to a previously published data set containing differentially expressed genes after treatment of the colon carcinoma cell line LS174T with β-catenin shRNA. Out of the 5543 differentially expressed genes in LS174T cells (Additional file 1, tab “LS174T_complete”), 786 genes and 677 genes were regulated similarly in DLD1 and SW480 cells, respectively. Furthermore, we identified 193 genes that are regulated in the same direction in all three cell lines (Figure 2A and Additional file 1, tab “DLD1_SW480_LS174T_only”).

Out of the 193 genes that are up or down regulated in DLD1, SW480, and LS174T cells, 4 genes (ASCL2, AXIN2, MYC, and S100A6) have previously been described as β-catenin target genes (compare Table 1 and Additional file 1, tab “DLD1_SW480_LS174T_only”; This tab also contains the false discovery rate (FDR) as well as the fold change (FC) for each gene). In addition, we identified the genes ABCB1, CD44, FGF18, and HEF1 (NEDD9) as potential β-catenin target genes in DLD1 and SW480 cells (Additional file 1, tab “335 target genes”; This tab also contains the false discovery rate (FDR) as well as the fold change (FC) for each gene).

**Table 1:** β-catenin target genes in human colon or colon carcinoma cells (including colon carcinoma cell lines)

**Gene**	**Protein**	**Direct regulation**	**Method to study direct regulation**	**Induction/Reduction**	**Reference**
**ABCB1**	ABC multidrug transporter	Yes	EMSA, RGA w/ mutated reporter gene	Up	[9]
**ADAM10**	Disintegrin and metalloproteinase domain-containing protein 1a	Not analyzed	RGA	Up	[25]
**ALEX1**	Armadillo repeat-containing X-linked protein 1	Not analyzed	RGA, ChIP for CREB; activation mediated by CREB (CRE) und E boxes within the ALEX1 promoter	Up	[26]
**ASCL2**	Achaete-scute homolog 2	Not analyzed	qRT-PCR	Up	[8]
AXIN2*	Axin-2	Not analyzed	RGA	Up	[27-29]
**BAMBI**	BMP and activin membrane-bound inhibitor	Yes	RGA w/ mutated reporter gene		[30]
**BCL2L2**	Bcl-2-like protein 2; BCLW	Yes	ChIP	Up	[31]
BIRC5*	Survivin, Baculoviral IAP repeat-containing protein 5	Yes	EMSA, RGA w/ mutated reporter gene	Up	[10]
**BMI1**	Polycomb complex protein BMI-1	Not analyzed	RGA	Up	[32]
BMP4*	Bone morphogenetic protein 4	Not analyzed	Northern blot	Up	[33]
CCND1*	Cyclin-D1	Yes	EMSA	Up	[5,6]
CD44*	CD44 antigen	Not analyzed	–	Up	[34,35]
**CDKN2A**	p16INK4a	Yes	ChIP	Up	[36]
**CDX1**	Homeobox protein Cdx-1	Yes	RGA w/ mutated reporter gene	Up	[37]
CLDN1*	Claudin-1	Yes	RGA	Up	[38]
**COX2**	cyclooxygenase-2	Yes	EMSA, RGA w/ mutated reporter gene	Up	[39]
DKK1*	Dickkopf-1	Yes	EMSA	Up	[40]
DKK4*	Dickkopf-4	Not analyzed	RGA, siRNA/qRT-PCR	Up	[41,42]
**DNMT1**	DNA methyltransferase 1	Not analyzed	RGA	Up	[43]
EDN1*	endothelin-1	Yes	ChIP	Up	[44]
EFNB1*	Ephrin-B1	Not analyzed	Northern blot	Down	[45]
**ENC1**	ectodermal-neural cortex 1	Yes	RGA w/ mutated reporter gene	Up	[13]
**EPHB2**	Ephrin type-B receptors	Not analyzed	Northern blot	Up	[45]
**EPHB3**	Ephrin type-B receptors	Not analyzed	Northern blot	Up	[45]
FGF18*	Fibroblast growth factor 18	Yes	EMSA	Up	[45,46]
**FGFBP**	Fibroblast growth factor-binding protein	Yes	RGA w/ mutated reporter gene	Up	[47]
FRA1*	Fos-related antigen 1	Yes	EMSA	Up	[48]
**FSCN1**	Fascin1	Yes	ChIP	Up	[49]
GAST*	Gastrin	Not analyzed	RGA	Up	[50]
HATH1*	human ortholog of atonal1	Not analyzed	RGA	Down	[19]
**HEF1 (NEDD9)**	human enhancer of filamentation 1	Yes	ChIP	Up	[51]
**HES1**	Hes1 (Hairy and enhancer of split 1)	Yes	RGA w/ mutated reporter gene	Up	[52]
ID2*	DNA-binding protein inhibitor ID-2	Yes	EMSA	Up	[11,12]
ITF2 (TCF4)*	Immunoglobulin transcription factor 2	Not analyzed	RGA	Up	[20]
JAG1*	Jagged-1	Yes	ChIP	Up	[53]
JUN*	Transcription factor AP-1	Yes	EMSA	Up	[48]
L1CAM*	L1 neuronal cell adhesion molecule	Yes	EMSA	Up	[54]
**LAMC2**	Laminin subunit gamma-2	Yes	EMSA, RGA w/ mutated reporter gene	Up	[55]
LEF1*	Lymphoid enhancer-binding factor 1	Yes	DNAse footprint, ChIP	Up	[56-58]
LGR5*	Leucine-rich repeat-containing G-protein coupled receptor 5	Not analyzed	–	Up	[59]
**MENA**	ENAH, Mammalian enabled homologue	Yes	ChIP	Up	[60]
MET*	Hepatocyte growth factor receptor	Not analyzed	–	Up	[61]
**MMP14**	Matrix metalloproteinase-14 (old name: MT1-MMP)	Yes	EMSA, RGA w/ mutated reporter gene	Up	[16]
MMP7*	Matrilysin	Not analyzed	RGA	Up	[14,15]
**MYB**	Transcriptional activator Myb	Not analyzed	Northern blot	Up	[62]
MYC*	Myc proto-oncogene protein	Yes	EMSA	Up	[4]
MYCBP*	c-myc binding protein	Not analyzed	RGA	Up	[63]
NOS2*	Nitric Oxide Synthase 2	Yes	EMSA	Up	[64]
**NOTCH2**	NOTCH2 protein	Yes	EMSA, RGA w/ mutated reporter gene	Up	[65]
NRCAM*	Neuronal cell adhesion molecule	Not analyzed	RGA	Up	[66]
PLAU*	urokinase plasminogen activator	Yes	EMSA	Up	[67]
**PLAUR**	urokinase-type plasminogen activator receptor	Not analyzed	–	Up	[48]
PPARD*	PPARdelta	Yes	EMSA	Up	[7]
**S100A4**	Protein S100-A4	Yes	Chip, EMSA, RGA w/ mutated reporter gene	Up	[68]
**S100A6**	Protein S100-A6	Not analyzed	RGA	Up	[69]
**SGK1**	Serum/glucocorticoid-regulated kinase 1	Yes	ChIP	Up	[70]
**SMC3**	Structural maintenance of chromosomes protein 3	Yes	EMSA, RGA w/ mutated reporter gene	Up	[71]
SOX9*	Transcription factor Sox-9	Not analyzed	Northern blot	Up	[72]
**SP5**	Transcription factor Sp5	Yes	EMSA	Up	[73]
**SRSF3 (SRp20)**	Serine/arginine-rich splicing factor 3	Not	RGA	Up	[74]
**SUZ12**	Polycomb protein SUZ12	Yes	ChIP, EMSA	Up	[75]
TCF1*	Transcription factor 7	Not analyzed	RGA	Up	[76]
TIAM1*	TIAM-1	Not analyzed	Northern blot	Up	[77]
**TN-C**	Tenascin-C	Yes	ChIP	Up	[78]
VEGF*	Vascular endothelial growth factor	Not analyzed	RGA	Up	[17]
**YAP**	Yes-associated protein	Yes	ChIP	Up	[79]

Based on a PubMed search, we identified 66 β-catenin target genes. Nusse et al. have previously reported 33 of these genes on their web site. (http://www.stanford.edu/group/nusselab/cgi-bin/wnt/target_genes; these genes are marked with an asterisk.). The gene and protein names follow the naming convention of the Swiss-Prot database (http://www.uniprot.org). A regulation of a given target gene by β-catenin had to be confirmed by chromatin immune precipitation (ChIP), electrophoretic mobility shift assay (EMSA), or reporter gene assays (RGA) with mutated TCF binding sites to be classified as “direct”.

Based on a literature search in the PubMed database, we identified 33 genes that are regulated in a β-catenin dependent manner in addition to the 33 β-catenin target genes published by Roel Nusse. (All these genes are differentially regulated either in normal colon, colon carcinoma cells, or colon carcinoma cell lines.) 38 out of these 66 genes are directly regulated by β-catenin as demonstrated by ChIP, EMSA, or RGA with mutated (canonical) TCF4 binding sites (Table 1). When we re-analyzed the microarray data focusing our analysis only on the list of these 66 previously published β-catenin target genes, we identified 21 (DLD1), 30 (SW480), and 34 (LS174T) of these genes as differentially regulated in the three cell lines, respectively (Figure 2B and Additional file 2). The genes ASCL2, AXIN2, MYC, NOTCH2, S100A6, and SP5 were regulated in DLD1, SW480, and LS174T cells. Moreover, we found that the genes ABCB1, CD44, FGF18, HEF1 (NEDD9), and ITF2 (TCF4) were also differentially regulated in DLD1 and SW480 cells (see Additional file 2, “DLD1_SW480_LS174T_only” and tab “DLD1_SW480_complete”). Thus, our second analysis using a limited number of genes confirmed our first microarray analysis using all differentially expressed genes and identified the genes ITF2 (TCF4), NOTCH2 and SP5 as β-catenin target genes in addition to ABCB1, ASCL2, AXIN2, CD44, FGF18, MYC, HEF1 (NEDD9), and S100A6 found in the first analysis.

Due to the large number of genes encoding members of the basic helix-loop-helix (bHLH) family of proteins among the list of β-catenin target genes (6 out 66 genes; Table 1), we were interested in identifying more members of this family of genes in our microarray data set.

When we re-analyzed the microarray data limiting our analysis to the 96 bHLH proteins listed in the PFAM database, we identified the genes ASCL2 and MYC as differentially regulated in DLD1, SW480, and LS174T cells (see Additional file 3, tab “DLD1_SW480_LS174T_only”). In DLD1 and SW480 cells, the genes ID1, and ITF2 (TCF4) were also differentially regulated (Figure 2C and Additional file 3, tab “DLD1_SW480_complete”). Taken together, out of the six β-catenin target genes belonging to the bHLH family of proteins (Table 1), three genes (ASCL2, ITF2 (TCF4), MYC) are commonly regulated in DLD1 *and* SW480 cells.

**Figure 3:**
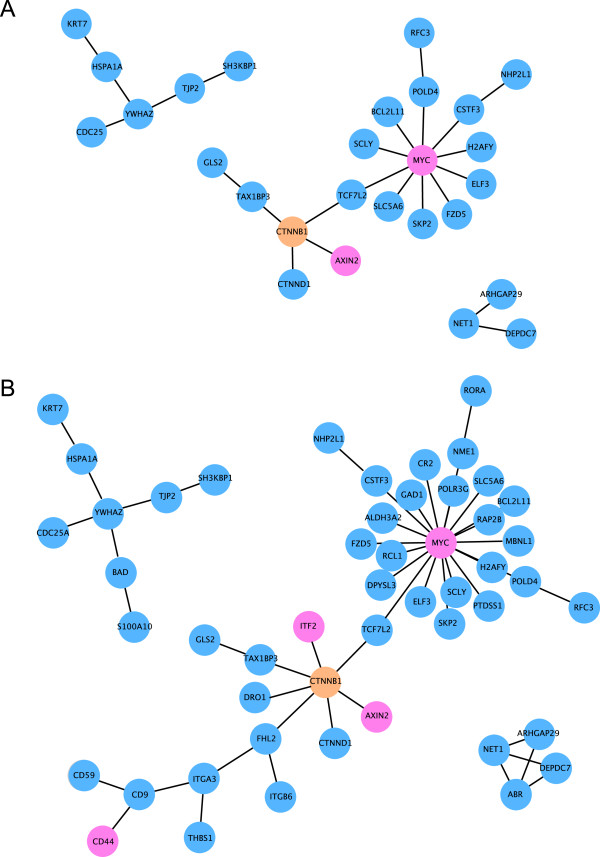
**Identification of known interactions between differentially expressed β-catenin target genes in DLD1, SW480, and LS174T cells.** Using the software package “Cytoscape” in combination with the “MiMI” plugin, we identified known interactions between the 193 differentially expressed β-catenin target genes in DLD1, SW480, and LS174T cells **(A)** and the 335 differentially expressed β-catenin target genes in DLD1 and SW480 cells **(B)**. Only gene networks are displayed that contain three or more nodes. The genes highlighted in pink color have been previously described as β-catenin target genes (see Table 1).

Using the software package “Cytoscape” in combination with the “Michigan Molecular Interactions” (MiMI) plugin, we searched the list of 193 genes that are differentially regulated in DLD1, SW480, and LS174T cells for known interactions. We identified three networks that contained three or more nodes (genes) (Figure 3A). The largest network centered on β-catenin comprised 18 genes, while the second largest network with 6 genes contained the gene YWHAZ encoding the 14-3-3 protein isoforms ξ/δ at its center. The smallest network contained the three nodes NET1, ARHGAP29, and DEPDC7 (Figure 3A). When we focused our analysis on the list of 335 genes that are differentially regulated in DLD1 and SW480 cells, we identified the same three networks that we found in the intersection of DLD1, SW480, and LS174T cells. However, the network centered on β-catenin was increased to 36 genes, while the network containing the gene YWHAZ comprised two additional nodes (8 in total). The smallest network contained the four nodes NET1, ARHGAP29, ABR and DEPDC7 (Figure 3B). Interestingly, both panels highlight the β-catenin target gene MYC as a major player with 10 and 19 directly regulated genes, respectively (Figure 3A and B).

Apart from identifying potential β-catenin target genes, we were interested to find out if particular signaling pathways or cellular functions are regulated by β-catenin. We used the gene set enrichment software (GSEA) software [80,81] to identify pathways associated with β-catenin siRNA treatment. In DLD1 and SW480 cells treated with control siRNA 2 KEGG pathways were enriched. These pathways comprised the glycine, serine and threonine metabolism as well as the renin angiotensin system. After down regulation of β-catenin expression levels, the following 9 KEGG pathways were enriched in DLD1 and SW480 cells: endocytosis, insulin signaling pathway, lysosome fc gamma r mediated phagocytosis, apoptosis, regulation of actin cytoskeleton, adipocytokine signaling, glycerolipid metabolism, lysosome and aldosterone regulated sodium reabsorption (Table 2). In addition, the steroid hormone biosynthesis pathway was enriched in DLD1, SW480, *and* LS174T cells after treatment with β-catenin siRNA (Table 2). Apart from the KEGG pathway database, we also used the Biocarta pathway database in the gene set enrichment analysis. Down regulation of β-catenin resulted in the enrichment of the M-calpain pathway, the Creb pathway, and the IGF1R pathway in DLD1 and SW480 cells (Additional files 4 and 5).

**Table 2:** Identification of signaling pathways enriched in more than one cell line

**Treatment**	**Intersection**	**Enriched KEGG pathways**	**Biocarta pathways**
β-catenin RNAi	DLD1/SW480	Endocytosis	mCalpain pathway
		Insulin signaling pathway	CREB pathway
		Lysosome	IGF1R pathway
		FC gamma R mediated phagocytosis	
		Apoptosis	
		Regulation of actin cytoskeleton	
		Glycerolipid metabolism	
		Focal adhesion	
		Aldosterone regulated sodium reabsorption	
	DLD1/LS174T	PPAR signaling pathway	–
		Metabolism of xenobiotics by cytochrome P450	
		Butanoate metabolism	
	SW480/LS174T	Complement and coagulation cascades	
		Histidine metabolism	
		Ether lipid metabolism	
		NOD like receptor signaling pathway	
	DLD1/SW480/LS174T	Steroid hormone biosynthesis	–
β-galactosidase RNAi	DLD1/SW480	Glycine serine and threonine metabolism	–
		Renin angiotensin system	
	DLD1/LS174T	DNA replication	ATRBRCA pathway
		Base excision repair	
		Mismatch repair	
		Homologous recombination	
		Cell cycle	
		Nucleotide excision repair	
		Pyrimidine metabolism	
		RNA polymerase	
		RNA degradation	
		Spliceosome	
	SW480/LS174T	Basal cell carcinoma	–
	DLD1/SW480/LS174T	–	

Gene set enrichment analyses were performed after treatment of DLD1, SW480, and LS174T cells with β-catenin RNAi or β-galactosidase RNAi using the KEGG and the Biocarta pathway database, respectively. Pathways that were enriched in more than one cell line were identified using a Biovenn software package.

## Discussion

β-catenin plays a crucial role in embryogenesis, tissue homeostasis and carcinogenesis. Because of its role in regulating homeostasis of the intestinal tract as well as being a key player in sporadic forms of colorectal cancer, inhibition of β-catenin is not a suitable strategy to treat patients with colorectal cancer. For this reason, identifying and characterizing β-catenin target genes could result in better understanding of colorectal carcinogenesis and development of new therapies. Here, we used two non-isogenic colorectal cancer cell lines, DLD1, and SW480, and identified 335 commonly regulated β-catenin target genes. 193 of these genes were also differentially regulated in LS174T cells. (Genes that are differentially expressed due to the genetic background of a given cell should be deregulated to a similar extent irrespective of the siRNA treatment and hence should not appear as being differentially regulated in the DNA microarray experiment.) Compared to the number of differentially regulated genes in LS174T *and* DLD1 cells (786 genes) as well as in LS174T *and* SW480 cells (677 genes), the number of commonly regulated genes in DLD1 *and* SW480 (335 genes) was low, resulting in a low number of commonly regulated genes in DLD1, SW480, *and* LS174T cells (193 genes). While DLD1 and SW480 cells both express a mutated form of APC, they differ with respect to the underlying genetic disturbance: DLD1 cells show microsatellite instability (MSI), whereas SW480 cells are derived from a tumor with chromosomal instability (CIN). However, SW480 cells (CIN) share a lot of commonly regulated genes with LS174T cells (MSI), suggesting that other factors apart from genetic instability contribute to the observed differences in gene expression between DLD1 and SW480 cells. Looking at the mutational status, DLD1, SW480, and LS174T cells differ with respect to the status of the genes APC, PI3K, and TP53, but they are identical with respect to status of the genes KRAS and PTEN: DLD1 (APC: Mutant; TP53: WT; PI3K: Mutant; KRAS: Mutant; PTEN: WT), SW480 (APC: Mutant; TP53: Mutant; PI3K: WT; KRAS: Mutant; PTEN: WT), and LS174T cells (APC: WT; TP53: Mutant; PI3K: Mutant; KRAS: Mutant; PTEN: WT) [82,83].

Deregulation of PI3K/Akt signaling (as observed in DLD1 and LS174T) has been implicated in the phosphorylation of β-catenin at Ser552 [84], thereby promoting nuclear accumulation and enhancing β-catenin dependent transcriptional activity even in the absence of aberrant Wnt signaling [85,86]. This observation suggests that in particular LS174T cells that express wild-type APC could benefit from a mutation in the PI3K gene. But even DLD1 cells (with mutant APC) could profit from PI3K mutations: Deming et al. demonstrated that expression of a dominant active form of PI3K in *Apc*^Min/+^ mice resulted in an increased tumor number and size and implicated the CCND1 gene as one transcriptional target that contributes to the observed phenotype of these mice [87]. In our experiments, CCND1 mRNA expression levels were up regulated in LS174T cells, but not in DLD1 cells.

The tumor suppressor p53 has been implicated in down-regulating β-catenin expression and/or activity by Siah-1 dependent (and GSK-3β independent) degradation [88,89], reduction of TCF4 mRNA and protein levels [90] as well as reduction of β-catenin mRNA by p53 dependent regulation of miRNA miR-34 [91]. Accordingly, inactivation of the transcriptional activity of p53 by mutations –as observed in SW480 cells– should result in increased β-catenin mRNA and protein levels and transcriptional activity as well as increased mRNA and protein levels of TCF4 (TCF7L2). Interestingly, LS174T cells showed a strong increase in the fold change value for CTNNB1 (encoding β-catenin) (4,56 versus 1,59 (DLD1) and 1,18 (SW480), respectively). All three cell lines showed slightly reduced fold change values for TCF4 (TCF7L2). The effect of p53 inactivation on the development of intestinal adenomas was also analyzed in *Apc*^Min/+^ mice: Despite the fact that p53 protein expression is deregulated as a consequence of APC loss (and probably other factors) and p53 is known to regulate β-catenin expression via a negative feedback loop, loss of p53 did not promote the initial steps of intestinal neoplasia in *Apc*^Min/+^ mice [92], suggesting that p53 has only a limited role in this mouse model.

Apart from regulating β-catenin protein expression levels via proteasomal degradation, (nuclear) APC has been implicated in affecting β-catenin activity by recruiting co-repressors of β-catenin to promoter regions, as well as sequestering and enhancing the nuclear export of β-catenin [93-96]. Mutated forms of APC still show a nuclear localization, but they are more frequently observed in the cytoplasm when compared to wild-type APC, suggesting that mutation of APC is not only contributing to higher β-catenin protein expression levels, but also contribute to enhanced β-catenin activity [97]. When Zeineldin et al. generated mice expressing APC with mutated nuclear localization signals (mNLS) and compared these *Apc*^mNLS/mNLS^ mice with *Apc*^+/+^ mice, they found up regulation of the mRNA for the genes AXIN2, MYC, and CCDN1 as well as down regulation of HATH1 mRNA in response to stimulation with Wnt [98]. Accordingly, we expected an increase in the fold change values for AXIN2, MYC, and CCDN1 transcripts as well as a reduction of HATH1 transcripts in DLD1 and SW480 cells (APC mutated) compared to LS174T (APC wild-type). In our experiments, DLD1 and SW480 showed a modest increase in the fold change values for MYC (see Additional file 1, tab “DLD1_SW480_LS174T_only”) compared to LS174T cells. However, LS174T (and SW480) cells responded with a higher fold change for AXIN2 compared to DLD1 cells. CCND1 transcript levels were increased only in LS174T cells, whereas HATH1 transcript levels did not changed in any of three cell lines.

The differential expression of the genes AXIN2 and CCND1 in the three colorectal cancer cell lines highlights the fact that the genetic background of a given cell has a major impact on the expression of a specific gene. However, linking a specific somatic mutation in a tumor suppressor gene or proto oncogene, e.g. APC, PI3K, or TP53, to these differences in expression is difficult because the expression of a specific gene is affected by multiple transcription factors (along with their cofactors and corepressors). Therefore, we are not able to attribute the observed differences in the gene expression profiles for DLD1 and SW480 to a single mutation.

Compared to LS174T cells, the total number of differentially regulated β-catenin target genes was much lower in DLD1 and SW480 cells. This could be either specific to the genetic background of the cell lines used or due to different experimental approaches. While we transiently transfected DLD1 and SW480 cells with β-catenin siRNA and analyzed the gene expression 48 hours after transfection, Mokry et al. [99] stably transfected LS174T cells with a doxycycline inducible expression vector encoding β-catenin shRNA and stimulated the cells for 72 hours with doxycycline before analyzing the gene expression profile. Both research groups used Affymetrix Human Genome U133 Plus 2.0 DNA microarrays for their experiments. The longer incubation period used to identify potential β-catenin target genes in LS174T cells might contribute to secondary effects like the differential expression of indirect β-catenin target genes, thus explaining why the absolute number of differentially regulated genes was higher in LS174T cells when compared to DLD1 and SW480 cells. For this reason, we were particularly interested in genes that are up and down regulated in the non-isogenic colorectal cancer cell lines DLD1 *and* SW480 or DLD1, SW480, *and* LS174T cells, assuming that these genes are commonly and directly regulated by β-catenin.

Remarkably, the number of β-catenin regulated genes was very similar in the three cell lines when focusing on the list of 66 previously described β-catenin target genes. Here, DLD1, SW480 and LS174T cells differentially regulated 21, 30 and 34 of these genes, respectively. There are at least two reasons why the number of detected β-catenin target genes did not include (almost) all 66 β-catenin target genes presented in Table 1. First, the detection of β-catenin target genes is dependent on the cellular context. DLD1, SW480, and LS174T represent colorectal cancer cell lines. Therefore, the expression pattern of β-catenin target genes in these cells is likely to differ from β-catenin target genes found in healthy colon cells, colonic adenomas, or metastatic colonic cells that are also listed in Table 1. Second, the gene expression profile was analyzed 48 and 72 hours after beginning with the RNAi treatment, respectively. Different periods of time influence the identification of β-catenin target genes as well, since some of these genes might be regulated only for a short period of time immediately after induction of β-catenin activity. This phenomenon has been described by Van de Wetering et al. (2002): They transfected LS174T cells with doxycycline (Dox) inducible expression vectors encoding dnTCFs and analyzed the changes in gene expression using DNA microarrays. 11 h after treatment with Dox, 2411 genes where differentially regulated. After an incubation period of 23 h, they identified 1199 differentially regulated genes. Comparison of the two sets of genes revealed that the expression of 1971 genes was abrogated between 11 and 23 h after Dox treatment, while 759 additional genes appeared in this time frame [62]. Similarly, another group analyzed the gene expression profiles of several known β-catenin target genes in a time-course experiment and classified these genes according to their expression profiles [100]. Based on this criterion, they identified genes that were “immediately up (or down) regulated”, “early up (down)”, or “late up (down)” in response to the activation of the Wnt/β-catenin signaling cascade. When comparing their results to the regulation of the same genes in different cellular contexts, this group found that the regulation of these genes was similar, but not necessarily identical in different cell lines. This observation suggests that the expression levels and activities of a given transcription factor and its cofactors (or co-repressors) influence the starting point and duration of the gene expression of a specific target gene. Therefore, it is recommendable to identify target genes of a given transcription factor in different cell lines at least at the same time points, better yet in a time-course experiment.

Using the KEGG pathway database, we identified 11 signaling pathways or cellular functions in DLD1 *and* SW480 cells that are differentially regulated in response to treatment of the colorectal cancer cell lines with β-catenin siRNA. In addition, the steroid hormone biosynthesis pathway was similarly regulated in all three cell lines analyzed. Whereas the steroid hormone biosynthesis pathway [101,102] as well as the endocytosis pathway [103,104], the insulin signaling pathway [105,106], apoptosis [107,108], regulation of the actin cytoskeleton [49], and focal adhesion [109], have been associated with Wnt/β-catenin signaling, there are no publications in the PubMed database linking β-catenin to the remaining six pathways in colorectal cancer cells. Interestingly, the list of KEGG pathways/functions did not include the canonical Wnt signaling pathway. This was probably due to the definition of the “KEGG Wnt signaling pathway” that summarized the canonical, the planar cell polarity (PCP), and the Wnt/Ca^2+^ signaling pathway under one database entry, despite different functions of these signaling pathways. While the canonical Wnt pathway has been implicated in regulating the activity of the β-catenin protein, PCP signaling results in remodeling of the cytoskeleton that is a prerequisite for migration and cell polarization via the GTPases RHOA and RAC1. Wnt/Ca^2+^ signaling, however, plays a role in the regulation of cell adhesion and cell movements during gastrulation and results in the activation of protein kinase (PKC), calcium calmodulin mediated kinase II (CAMKII) and calcineurin [110].

Furthermore, all three Biocarta pathways that were enriched in DLD1 *and* SW480 cells have been associated with Wnt/β-catenin signaling based on PubMed publications: the m-calpain pathway [111,112], the Creb pathway [26,113], and the IGF1R pathway [114,115].

Since 6 out of the 66 previously described β-catenin target genes encode proteins that belong to the bHLH protein family (Table 1), we focused our analysis on this group of genes. Our analysis revealed that the genes ASCL2, ID1, ITF2, and MYC were regulated in a β-catenin dependent manner in DLD1 and SW480 cells. Interestingly, the proteins ASCL2 and ITF-2B as well as ID1 and ITF-2B are known to interact with each other [116,117], suggesting that these bHLH proteins form a functional network in colorectal carcinoma cells.

## Conclusions

In conclusion, by comparing potential β-catenin target genes in DLD1 and SW480 cells with 1) corresponding data from LS174T cells and 2) a list of previously identified β-catenin target genes, we found and confirmed a large number of β-catenin target genes, suggesting that DLD1 and SW480 (as well as LS174T cells) are suitable for identifying β-catenin target genes as well as β-catenin dependent signaling pathways and functions. Therefore, the presented list of commonly regulated genes in DLD1 and SW480 cells as well as the annotated list of previously published β-catenin target genes are useful resources for further studies.

## Methods

### Cell culture

The colon carcinoma cell lines DLD1 and SW480 were obtained from ATCC (Manassas, VA, USA) and were cultured in DMEM medium supplemented with 10% calf serum (PAA, Pasching, Austria). Short interfering RNAs targeting β-catenin (cat. no. D-003482-04) and β-galactosidase (cat. no. D-012539-01) were purchased from Dharmacon (Lafayette, CO, USA) as siGenome siRNAs. Cells were transfected with these siRNAs using Lipofectamine 2000 (Invitrogen, Carlsbad, CA, USA).

#### Immuno detection

Cell lysates were prepared with reporter gene lysis buffer (Promega, Mannheim, Germany) supplemented with protease inhibitor cocktail I (Calbiochem, Darmstadt, Germany). Equal amounts of protein were mixed with 2× SDS loading buffer (0.35 M Tris, pH 6.8; 30% (v/v) glycerol; 10% (w/v) SDS; 100 mM Dithiothreitol; 0.01% (w/v) bromphenol blue), separated by electrophoresis in discontinuous SDS-polyacrylamide gels and transferred to Immobilon-P membranes (Millipore, Billerica, MA, USA). Antibodies specific for β-catenin (BD Transduction Labs, San Jose, CA, USA), β-actin (MP Biomedicals, Eschwege, Germany) and the secondary horseradish peroxidase-conjugated goat anti-mouse antibody (GE Healthcare, Freiburg, Germany) were used for immuno detection. Blots were subjected to Supersignal West Dura chemiluminescence substrate (Thermo Scientific, Rockford, IL, USA) and exposed to Fuji Medical x-ray film SuperRX (FujiFilm).

#### Reporter gene assays

For reporter gene assays, 10^5^ DLD1 or SW480 cells were seeded per well (12 well plate). Cells were transfected with the indicated siRNAs using Lipofectamine 2000 (Life Technologies). 24 hours later, the 8×TOPflash reporter gene construct (a generous gift from Randall T. Moon, University of Washington) as well as the plasmid pCH110 encoding β-galactosidase (GE Healthcare, Freiburg, Germany) were transfected into these cells. 72 hours after the siRNA transfection, cells were harvested, lysed with reporter lysis buffer (Promega) and luciferase activities were measured using a luciferase assay reagent (Promega) and a luminometer (Orion II, Berthold Detection Systems, Wildbad, Germany). β-galactosidase activity was determined by standard methods using o-Nitrophenyl β-D-galactopyranoside (ONPG, Sigma-Aldrich, Taufkirchen, Germany) as a control for transfection efficiency.

#### Microarray analysis

DLD1 and SW480 cells were harvested 48 h after siRNA transfection using TRIzol (Life Technologies, Darmstadt, Germany). Total RNA was isolated according to the manufacturer’s instructions and reverse transcribed using SuperScript II reverse transcriptase (Life Technologies) according to the instructions provided by the manufacturer. cDNA was amplified and labeled with biotin using kits from Affymetrix. Labeled probes were hybridized to Human Genome U133 Plus 2.0 microarrays (Affymetrix), washed, stained and scanned using equipment from Affymetrix. The microarray analysis was performed in triplicates for each cell line (see also the corresponding CEL files that are part of the accompanying GEO submission).

#### Identification of validated β-catenin target genes

We were particularly interested in β-catenin target genes that are regulated in *normal colon or colon carcinoma cells or colon carcinoma cell lines.* For this reason, the PubMed database was searched using the phrase “beta catenin AND target AND (colorectal OR colon OR intestinal OR intestine)”. 591 articles were identified (as of June 1^st^, 2012) and publications were screened for the identification and characterization of β-catenin target genes. We were able to identify 66 published β-catenin target genes that matched our criteria (Table 1). 33 out of the 66 genes have been published earlier by Nusse et al. on their web site http://www.stanford.edu/group/nusselab/cgi-bin/wnt/target_genes (last update: October 2010). These genes are marked with an asterisk after the gene name (Table 1).

#### Statistical analysis

For evaluation purposes, we compared genes differentially expressed in DLD1 and SW480 cells after treatment with β-catenin siRNA to a previously published data set containing differentially expressed genes after treatment of the colon carcinoma cell line LS174T with β-catenin shRNA (http://www.ncbi.nlm.nih.gov/geo/query/acc.cgi?acc=GSE18560). The software package R (version 2.14.1) was used for the statistical analysis of the microarray data. Microarray data were preprocessed using the *rma* function from the R package “affy”. The expression matrix was filtered to contain only those probe sets that can be assigned to a gene name. Log2 intensity values were used for all subsequent analyses. Differentially expressed genes were identified with the R package “limma”. Genes were called “differentially expressed” if the corrected p value (according to Benjamini-Hochberg) was lower than 0.05. We considered a gene to be significant only if all of its significant probe sets were regulated in the same direction. Genes with significant probe sets that were differently regulated were not considered significant because we could not determine if the gene is up- or down regulated.

#### Gene set enrichment analysis (GSEA)

The gene set enrichment analysis was performed using the GSEA software [80,81]. Due to the small sample size of our data sets, we used gene set permutation for the GSEA analysis. Probe sets belonging to the same gene were collapsed to a gene symbol and 1000 permutations were performed to find differentially expressed gene sets. If our data sets contained less than 15 genes of a certain gene set, the gene set was filtered out. After this restriction, 138 out of 217 Biocarta gene sets and 175 out of 186 KEGG gene sets were analyzed. Gene sets with a false discover rate (FDR) lower than 0.25 were considered statistically significant. The corresponding files containing the data of the GSEA analysis are part of the Additional files. The names of the directories containing the files were composed of the term ‘GSEA’, the name of the cell line, e.g. DLD1, SW480, or LS174T, and the pathway database (KEGG or Biocarta). Please use the files with the name ‘index.html’ in the corresponding directories to start exploring the data.

KEGG and Biocarta pathways that are regulated similarly in two or three cell lines were identified using the Biovenn software (http://www.cmbi.ru.nl/cdd/biovenn/) [118].

#### Network visualization

Gene symbols of β-catenin target genes common to two or more cell lines were entered in the query function of the “Michigan molecular interaction” (MiMI) plugin (version 3.11) in the software package “Cytoscape” (version 2.83) [119,120]. Using the human database entries to analyze interactions between all molecule types and in all data sources, we searched for “Interactions among query genes”. Only networks that contained three or more nodes were displayed.

### Availability of supporting data

The data sets supporting the results of this article are available NCBI's Gene Expression Omnibus (GEO) repository [121]. The unique identifier for this dataset is GSE44097. The corresponding information can be found at the following web site: http://www.ncbi.nlm.nih.gov/geo/query/acc.cgi?acc=GSE44097.

## Competing interests

The authors declare that they have no competing interests.

## Authors’ contributions

AH analyzed the data, generated the figures and drafted the manuscript. VJ performed the statistical analysis. ST and SK performed the DNA microarray experiment. HB, BG and FTK participated in the design and coordination of the study. All authors read and approved the final manuscript.

## Supplementary Material

Additional file 1: Table S1 Results of the DNA microarray analysis (all genes). DLD1, SW480, and LS174T cells were treated with siRNA or shRNA targeting beta-catenin. Differentially expressed genes were identified using DNA microarrays as described in Materials and Methods. Using the Biovenn software, genes that are regulated similarly in two or three cell lines were identified and listed under separate tabs. Accordingly, genes that are differentially expressed in only one cell line were listed in a separate tab labeled with the name of the cell line. Click here for file

Additional file 2: Table S2 Results of the DNA microarray analysis (focusing on known β-catenin target genes). DLD1, SW480, and LS174T cells were treated with siRNA or shRNA targeting β-catenin. Microarray data was re-analyzed focusing our analysis only on the list of these 66 previously published β-catenin target genes. Using the Biovenn software, genes that are regulated similarly in two or three cell lines were identified and listed under separate tabs. Accordingly, genes that are differentially expressed in only one cell line were listed in a separate tab labeled with the name of the cell line. Click here for file

Additional file 3: Table S3 Results of the DNA microarray analysis (focusing on genes encoding bHLH proteins). DLD1, SW480, and LS174T cells were treated with siRNA or shRNA targeting beta-catenin. Microarray data was re-analyzed focusing our analysis only on the 96 bHLH proteins listed in the PFAM database. Using the Biovenn software, genes that are regulated similarly in two or three cell lines were identified and listed under separate tabs. Accordingly, genes that are differentially expressed in only one cell line were listed in a separate tab labeled with the name of the cell line. Click here for file

Additional file 4 **GSEA analysis using the Biocarta pathway database.** This zipped file contains confirming data of the GSEA analysis. The names of the directories containing the files were composed of the term ‘GSEA’, the name of the cell line, e.g. DLD1, SW480, or LS174T, and the pathway database (Biocarta). Please use a web browser to view the files with the name ‘index.html’ in the corresponding directories to start exploring the data. Click here for file

Additional file 5 **GSEA analysis using the KEGG pathway database.** This zipped file contains confirming data of the GSEA analysis. The names of the directories containing the files were composed of the term ‘GSEA’, the name of the cell line, e.g. DLD1, SW480, or LS174T, and the pathway database (KEGG). Please use a web browser to view the files with the name ‘index.html’ in the corresponding directories to start exploring the data. Click here for file
