# Supplementary material for: Comprehensive analysis of β-catenin target genes in colorectal carcinoma cell lines with deregulated Wnt/β-catenin signaling
Source: BMC Genomics. 2014 Jan 28;15:74. doi: 10.1186/1471-2164-15-74 (PMC3909937; doi:10.1186/1471-2164-15-74)
Supplement: Additional file 4 — GSEA analysis using the Biocarta pathway database. This zipped file contains confirming data of the GSEA analysis. The names of the directories containing the files were composed of the term ‘GSEA’, the name of the cell line, e.g. DLD1, SW480, or LS174T, and the pathway database (Biocarta). Please use a web browser to view the files with the name ‘index.html’ in the corresponding directories to start exploring the data. [file 1471-2164-15-74-S4.zip › DLD1_Biocarta/BIOCARTA_ACTINY_PATHWAY.html]

Details for gene set BIOCARTA\_ACTINY\_PATHWAY[GSEA]

|  || Dataset | DLD1\_collapsed\_to\_symbols.class.cls#bg\_versus\_b |
| Phenotype | class.cls#bg\_versus\_b |
| Upregulated in class | b |
| GeneSet | BIOCARTA\_ACTINY\_PATHWAY |
| Enrichment Score (ES) | -0.56551164 |
| Normalized Enrichment Score (NES) | -1.5491564 |
| Nominal p-value | 0.043378994 |
| FDR q-value | 0.26447752 |
| FWER p-Value | 0.919 |
Table: GSEA Results Summary

  

Fig 1: Enrichment plot: BIOCARTA\_ACTINY\_PATHWAY      
 Profile of the Running ES Score & Positions of GeneSet Members on the Rank Ordered List

  

| PROBE | GENE SYMBOL | GENE\_TITLE | RANK IN GENE LIST | RANK METRIC SCORE | RUNNING ES | CORE ENRICHMENT || 1 | WASF1 | WASF1 Entrez,  Source | WAS protein family, member 1 | 3130 | 0.087 | -0.0731 | No |
| 2 | WASF3 | WASF3 Entrez,  Source | WAS protein family, member 3 | 7200 | 0.032 | -0.2496 | No |
| 3 | PSMA7 | PSMA7 Entrez,  Source | proteasome (prosome, macropain) subunit, alpha type, 7 | 10087 | 0.006 | -0.3914 | No |
| 4 | ABI2 | ABI2 Entrez,  Source | abl interactor 2 | 10898 | -0.001 | -0.4317 | No |
| 5 | ACTR2 | ACTR2 Entrez,  Source | ARP2 actin-related protein 2 homolog (yeast) | 13514 | -0.027 | -0.5381 | Yes |
| 6 | ARPC2 | ARPC2 Entrez,  Source | actin related protein 2/3 complex, subunit 2, 34kDa | 13578 | -0.028 | -0.5133 | Yes |
| 7 | ACTR3 | ACTR3 Entrez,  Source | ARP3 actin-related protein 3 homolog (yeast) | 13954 | -0.032 | -0.5000 | Yes |
| 8 | NCKAP1 | NCKAP1 Entrez,  Source | NCK-associated protein 1 | 14681 | -0.042 | -0.4953 | Yes |
| 9 | RAC1 | RAC1 Entrez,  Source | ras-related C3 botulinum toxin substrate 1 (rho family, small GTP binding protein Rac1) | 14979 | -0.046 | -0.4643 | Yes |
| 10 | NTRK1 | NTRK1 Entrez,  Source | neurotrophic tyrosine kinase, receptor, type 1 | 15212 | -0.049 | -0.4267 | Yes |
| 11 | PIR | PIR Entrez,  Source | pirin (iron-binding nuclear protein) | 15583 | -0.055 | -0.3904 | Yes |
| 12 | ARPC5 | ARPC5 Entrez,  Source | actin related protein 2/3 complex, subunit 5, 16kDa | 15899 | -0.060 | -0.3460 | Yes |
| 13 | WASF2 | WASF2 Entrez,  Source | WAS protein family, member 2 | 16189 | -0.065 | -0.2950 | Yes |
| 14 | ACTA1 | ACTA1 Entrez,  Source | actin, alpha 1, skeletal muscle | 16287 | -0.067 | -0.2323 | Yes |
| 15 | NCK1 | NCK1 Entrez,  Source | NCK adaptor protein 1 | 16345 | -0.069 | -0.1661 | Yes |
| 16 | ARPC3 | ARPC3 Entrez,  Source | actin related protein 2/3 complex, subunit 3, 21kDa | 16471 | -0.072 | -0.1005 | Yes |
| 17 | WASL | WASL Entrez,  Source | Wiskott-Aldrich syndrome-like | 16739 | -0.077 | -0.0364 | Yes |
| 18 | ARPC4 | ARPC4 Entrez,  Source | actin related protein 2/3 complex, subunit 4, 20kDa | 17054 | -0.085 | 0.0328 | Yes |
| 19 | ARPC1A | ARPC1A Entrez,  Source | actin related protein 2/3 complex, subunit 1A, 41kDa | 17416 | -0.095 | 0.1095 | Yes |
Table: GSEA details [plain text format]

  

Fig 2: BIOCARTA\_ACTINY\_PATHWAY      
 Blue-Pink O' Gram in the Space of the Analyzed GeneSet

  

Fig 3: BIOCARTA\_ACTINY\_PATHWAY: Random ES distribution      
 Gene set null distribution of ES for **BIOCARTA\_ACTINY\_PATHWAY**

  
