# Supplementary material for: Comprehensive analysis of β-catenin target genes in colorectal carcinoma cell lines with deregulated Wnt/β-catenin signaling
Source: BMC Genomics. 2014 Jan 28;15:74. doi: 10.1186/1471-2164-15-74 (PMC3909937; doi:10.1186/1471-2164-15-74)
Supplement: Additional file 4 — GSEA analysis using the Biocarta pathway database. This zipped file contains confirming data of the GSEA analysis. The names of the directories containing the files were composed of the term ‘GSEA’, the name of the cell line, e.g. DLD1, SW480, or LS174T, and the pathway database (Biocarta). Please use a web browser to view the files with the name ‘index.html’ in the corresponding directories to start exploring the data. [file 1471-2164-15-74-S4.zip › DLD1_Biocarta/BIOCARTA_ATM_PATHWAY.html]

Details for gene set BIOCARTA\_ATM\_PATHWAY[GSEA]

|  || Dataset | DLD1\_collapsed\_to\_symbols.class.cls#bg\_versus\_b |
| Phenotype | class.cls#bg\_versus\_b |
| Upregulated in class | bg |
| GeneSet | BIOCARTA\_ATM\_PATHWAY |
| Enrichment Score (ES) | 0.5665913 |
| Normalized Enrichment Score (NES) | 1.5347906 |
| Nominal p-value | 0.041516244 |
| FDR q-value | 0.42416486 |
| FWER p-Value | 0.905 |
Table: GSEA Results Summary

  

Fig 1: Enrichment plot: BIOCARTA\_ATM\_PATHWAY      
 Profile of the Running ES Score & Positions of GeneSet Members on the Rank Ordered List

  

| PROBE | GENE SYMBOL | GENE\_TITLE | RANK IN GENE LIST | RANK METRIC SCORE | RUNNING ES | CORE ENRICHMENT || 1 | MRE11A | MRE11A Entrez,  Source | MRE11 meiotic recombination 11 homolog A (S. cerevisiae) | 550 | 0.195 | 0.0980 | Yes |
| 2 | BRCA1 | BRCA1 Entrez,  Source | breast cancer 1, early onset | 674 | 0.182 | 0.2091 | Yes |
| 3 | TP53 | TP53 Entrez,  Source | tumor protein p53 (Li-Fraumeni syndrome) | 877 | 0.163 | 0.3043 | Yes |
| 4 | RAD51 | RAD51 Entrez,  Source | RAD51 homolog (RecA homolog, E. coli) (S. cerevisiae) | 987 | 0.156 | 0.3993 | Yes |
| 5 | CHEK1 | CHEK1 Entrez,  Source | CHK1 checkpoint homolog (S. pombe) | 2393 | 0.102 | 0.3936 | Yes |
| 6 | CHEK2 | CHEK2 Entrez,  Source | CHK2 checkpoint homolog (S. pombe) | 2615 | 0.097 | 0.4450 | Yes |
| 7 | RBBP8 | RBBP8 Entrez,  Source | retinoblastoma binding protein 8 | 2620 | 0.097 | 0.5074 | Yes |
| 8 | JUN | JUN Entrez,  Source | jun oncogene | 2676 | 0.096 | 0.5666 | Yes |
| 9 | TP73 | TP73 Entrez,  Source | tumor protein p73 | 4248 | 0.067 | 0.5296 | No |
| 10 | NBN | NBN Entrez,  Source | nibrin | 5987 | 0.044 | 0.4693 | No |
| 11 | ABL1 | ABL1 Entrez,  Source | v-abl Abelson murine leukemia viral oncogene homolog 1 | 7530 | 0.029 | 0.4088 | No |
| 12 | ATM | ATM Entrez,  Source | ataxia telangiectasia mutated (includes complementation groups A, C and D) | 7603 | 0.028 | 0.4231 | No |
| 13 | MDM2 | MDM2 Entrez,  Source | Mdm2, transformed 3T3 cell double minute 2, p53 binding protein (mouse) | 7751 | 0.026 | 0.4327 | No |
| 14 | NFKB1 | NFKB1 Entrez,  Source | nuclear factor of kappa light polypeptide gene enhancer in B-cells 1 (p105) | 8386 | 0.021 | 0.4135 | No |
| 15 | RAD50 | RAD50 Entrez,  Source | RAD50 homolog (S. cerevisiae) | 8390 | 0.020 | 0.4266 | No |
| 16 | MAPK8 | MAPK8 Entrez,  Source | mitogen-activated protein kinase 8 | 9088 | 0.015 | 0.4005 | No |
| 17 | GADD45A | GADD45A Entrez,  Source | growth arrest and DNA-damage-inducible, alpha | 10698 | 0.001 | 0.3185 | No |
| 18 | RELA | RELA Entrez,  Source | v-rel reticuloendotheliosis viral oncogene homolog A, nuclear factor of kappa light polypeptide gene enhancer in B-cells 3, p65 (avian) | 11371 | -0.006 | 0.2877 | No |
| 19 | CDKN1A | CDKN1A Entrez,  Source | cyclin-dependent kinase inhibitor 1A (p21, Cip1) | 17512 | -0.098 | 0.0367 | No |
| 20 | NFKBIA | NFKBIA Entrez,  Source | nuclear factor of kappa light polypeptide gene enhancer in B-cells inhibitor, alpha | 17726 | -0.105 | 0.0937 | No |
Table: GSEA details [plain text format]

  

Fig 2: BIOCARTA\_ATM\_PATHWAY      
 Blue-Pink O' Gram in the Space of the Analyzed GeneSet

  

Fig 3: BIOCARTA\_ATM\_PATHWAY: Random ES distribution      
 Gene set null distribution of ES for **BIOCARTA\_ATM\_PATHWAY**

  
