# Supplementary material for: Comprehensive analysis of β-catenin target genes in colorectal carcinoma cell lines with deregulated Wnt/β-catenin signaling
Source: BMC Genomics. 2014 Jan 28;15:74. doi: 10.1186/1471-2164-15-74 (PMC3909937; doi:10.1186/1471-2164-15-74)
Supplement: Additional file 4 — GSEA analysis using the Biocarta pathway database. This zipped file contains confirming data of the GSEA analysis. The names of the directories containing the files were composed of the term ‘GSEA’, the name of the cell line, e.g. DLD1, SW480, or LS174T, and the pathway database (Biocarta). Please use a web browser to view the files with the name ‘index.html’ in the corresponding directories to start exploring the data. [file 1471-2164-15-74-S4.zip › DLD1_Biocarta/BIOCARTA_BIOPEPTIDES_PATHWAY.html]

Details for gene set BIOCARTA\_BIOPEPTIDES\_PATHWAY[GSEA]

|  || Dataset | DLD1\_collapsed\_to\_symbols.class.cls#bg\_versus\_b |
| Phenotype | class.cls#bg\_versus\_b |
| Upregulated in class | b |
| GeneSet | BIOCARTA\_BIOPEPTIDES\_PATHWAY |
| Enrichment Score (ES) | -0.45756596 |
| Normalized Enrichment Score (NES) | -1.4757465 |
| Nominal p-value | 0.036637932 |
| FDR q-value | 0.3315513 |
| FWER p-Value | 0.981 |
Table: GSEA Results Summary

  

Fig 1: Enrichment plot: BIOCARTA\_BIOPEPTIDES\_PATHWAY      
 Profile of the Running ES Score & Positions of GeneSet Members on the Rank Ordered List

  

| PROBE | GENE SYMBOL | GENE\_TITLE | RANK IN GENE LIST | RANK METRIC SCORE | RUNNING ES | CORE ENRICHMENT || 1 | PLCG1 | PLCG1 Entrez,  Source | phospholipase C, gamma 1 | 2043 | 0.111 | -0.0652 | No |
| 2 | F2 | F2 Entrez,  Source | coagulation factor II (thrombin) | 2689 | 0.096 | -0.0643 | No |
| 3 | CAMK2A | CAMK2A Entrez,  Source | calcium/calmodulin-dependent protein kinase (CaM kinase) II alpha | 2780 | 0.094 | -0.0356 | No |
| 4 | KNG1 | KNG1 Entrez,  Source | kininogen 1 | 3036 | 0.088 | -0.0173 | No |
| 5 | AGT | AGT Entrez,  Source | angiotensinogen (serpin peptidase inhibitor, clade A, member 8) | 3678 | 0.077 | -0.0230 | No |
| 6 | GNAI1 | GNAI1 Entrez,  Source | guanine nucleotide binding protein (G protein), alpha inhibiting activity polypeptide 1 | 6049 | 0.044 | -0.1289 | No |
| 7 | CDK5 | CDK5 Entrez,  Source | cyclin-dependent kinase 5 | 7655 | 0.027 | -0.2015 | No |
| 8 | MAPT | MAPT Entrez,  Source | microtubule-associated protein tau | 8557 | 0.019 | -0.2408 | No |
| 9 | MAPK8 | MAPK8 Entrez,  Source | mitogen-activated protein kinase 8 | 9088 | 0.015 | -0.2627 | No |
| 10 | CALM1 | CALM1 Entrez,  Source | calmodulin 1 (phosphorylase kinase, delta) | 10081 | 0.006 | -0.3114 | No |
| 11 | FYN | FYN Entrez,  Source | FYN oncogene related to SRC, FGR, YES | 10299 | 0.004 | -0.3211 | No |
| 12 | HRAS | HRAS Entrez,  Source | v-Ha-ras Harvey rat sarcoma viral oncogene homolog | 10612 | 0.001 | -0.3366 | No |
| 13 | STAT4 | STAT4 Entrez,  Source | signal transducer and activator of transcription 4 | 10764 | 0.000 | -0.3443 | No |
| 14 | MAPK14 | MAPK14 Entrez,  Source | mitogen-activated protein kinase 14 | 11391 | -0.006 | -0.3743 | No |
| 15 | CAMK2B | CAMK2B Entrez,  Source | calcium/calmodulin-dependent protein kinase (CaM kinase) II beta | 11597 | -0.007 | -0.3822 | No |
| 16 | AGTR2 | AGTR2 Entrez,  Source | angiotensin II receptor, type 2 | 12136 | -0.013 | -0.4052 | No |
| 17 | SHC1 | SHC1 Entrez,  Source | SHC (Src homology 2 domain containing) transforming protein 1 | 12661 | -0.018 | -0.4255 | No |
| 18 | MAP2K2 | MAP2K2 Entrez,  Source | mitogen-activated protein kinase kinase 2 | 12979 | -0.022 | -0.4340 | No |
| 19 | GNB1 | GNB1 Entrez,  Source | guanine nucleotide binding protein (G protein), beta polypeptide 1 | 13439 | -0.027 | -0.4481 | Yes |
| 20 | STAT6 | STAT6 Entrez,  Source | signal transducer and activator of transcription 6, interleukin-4 induced | 13482 | -0.027 | -0.4407 | Yes |
| 21 | MAP2K1 | MAP2K1 Entrez,  Source | mitogen-activated protein kinase kinase 1 | 13627 | -0.028 | -0.4381 | Yes |
| 22 | RAF1 | RAF1 Entrez,  Source | v-raf-1 murine leukemia viral oncogene homolog 1 | 13629 | -0.028 | -0.4280 | Yes |
| 23 | GRB2 | GRB2 Entrez,  Source | growth factor receptor-bound protein 2 | 13726 | -0.030 | -0.4225 | Yes |
| 24 | CALM2 | CALM2 Entrez,  Source | calmodulin 2 (phosphorylase kinase, delta) | 14035 | -0.033 | -0.4265 | Yes |
| 25 | STAT3 | STAT3 Entrez,  Source | signal transducer and activator of transcription 3 (acute-phase response factor) | 14210 | -0.035 | -0.4228 | Yes |
| 26 | STAT2 | STAT2 Entrez,  Source | signal transducer and activator of transcription 2, 113kDa | 14745 | -0.043 | -0.4351 | Yes |
| 27 | STAT1 | STAT1 Entrez,  Source | signal transducer and activator of transcription 1, 91kDa | 14882 | -0.045 | -0.4262 | Yes |
| 28 | PRKCA | PRKCA Entrez,  Source | protein kinase C, alpha | 15308 | -0.051 | -0.4300 | Yes |
| 29 | SOS1 | SOS1 Entrez,  Source | son of sevenless homolog 1 (Drosophila) | 15485 | -0.053 | -0.4202 | Yes |
| 30 | MAPK1 | MAPK1 Entrez,  Source | mitogen-activated protein kinase 1 | 15755 | -0.058 | -0.4135 | Yes |
| 31 | STAT5B | STAT5B Entrez,  Source | signal transducer and activator of transcription 5B | 16215 | -0.066 | -0.4136 | Yes |
| 32 | GNGT1 | GNGT1 Entrez,  Source | guanine nucleotide binding protein (G protein), gamma transducing activity polypeptide 1 | 16603 | -0.074 | -0.4071 | Yes |
| 33 | CAMK2G | CAMK2G Entrez,  Source | calcium/calmodulin-dependent protein kinase (CaM kinase) II gamma | 17043 | -0.085 | -0.3996 | Yes |
| 34 | PTK2B | PTK2B Entrez,  Source | PTK2B protein tyrosine kinase 2 beta | 17225 | -0.090 | -0.3771 | Yes |
| 35 | CALM3 | CALM3 Entrez,  Source | calmodulin 3 (phosphorylase kinase, delta) | 17333 | -0.092 | -0.3498 | Yes |
| 36 | GNA11 | GNA11 Entrez,  Source | guanine nucleotide binding protein (G protein), alpha 11 (Gq class) | 17489 | -0.097 | -0.3232 | Yes |
| 37 | STAT5A | STAT5A Entrez,  Source | signal transducer and activator of transcription 5A | 17665 | -0.103 | -0.2958 | Yes |
| 38 | MAPK3 | MAPK3 Entrez,  Source | mitogen-activated protein kinase 3 | 18647 | -0.156 | -0.2907 | Yes |
| 39 | CAMK2D | CAMK2D Entrez,  Source | calcium/calmodulin-dependent protein kinase (CaM kinase) II delta | 18920 | -0.185 | -0.2391 | Yes |
| 40 | JAK2 | JAK2 Entrez,  Source | Janus kinase 2 (a protein tyrosine kinase) | 19059 | -0.209 | -0.1721 | Yes |
| 41 | MYLK | MYLK Entrez,  Source | myosin, light chain kinase | 19507 | -0.557 | 0.0025 | Yes |
Table: GSEA details [plain text format]

  

Fig 2: BIOCARTA\_BIOPEPTIDES\_PATHWAY      
 Blue-Pink O' Gram in the Space of the Analyzed GeneSet

  

Fig 3: BIOCARTA\_BIOPEPTIDES\_PATHWAY: Random ES distribution      
 Gene set null distribution of ES for **BIOCARTA\_BIOPEPTIDES\_PATHWAY**

  
