# Supplementary material for: Comprehensive analysis of β-catenin target genes in colorectal carcinoma cell lines with deregulated Wnt/β-catenin signaling
Source: BMC Genomics. 2014 Jan 28;15:74. doi: 10.1186/1471-2164-15-74 (PMC3909937; doi:10.1186/1471-2164-15-74)
Supplement: Additional file 4 — GSEA analysis using the Biocarta pathway database. This zipped file contains confirming data of the GSEA analysis. The names of the directories containing the files were composed of the term ‘GSEA’, the name of the cell line, e.g. DLD1, SW480, or LS174T, and the pathway database (Biocarta). Please use a web browser to view the files with the name ‘index.html’ in the corresponding directories to start exploring the data. [file 1471-2164-15-74-S4.zip › DLD1_Biocarta/BIOCARTA_CREB_PATHWAY.html]

Details for gene set BIOCARTA\_CREB\_PATHWAY[GSEA]

|  || Dataset | DLD1\_collapsed\_to\_symbols.class.cls#bg\_versus\_b |
| Phenotype | class.cls#bg\_versus\_b |
| Upregulated in class | b |
| GeneSet | BIOCARTA\_CREB\_PATHWAY |
| Enrichment Score (ES) | -0.61708355 |
| Normalized Enrichment Score (NES) | -1.8377911 |
| Nominal p-value | 0.0021978023 |
| FDR q-value | 0.18077312 |
| FWER p-Value | 0.249 |
Table: GSEA Results Summary

  

Fig 1: Enrichment plot: BIOCARTA\_CREB\_PATHWAY      
 Profile of the Running ES Score & Positions of GeneSet Members on the Rank Ordered List

  

| PROBE | GENE SYMBOL | GENE\_TITLE | RANK IN GENE LIST | RANK METRIC SCORE | RUNNING ES | CORE ENRICHMENT || 1 | CAMK2A | CAMK2A Entrez,  Source | calcium/calmodulin-dependent protein kinase (CaM kinase) II alpha | 2780 | 0.094 | -0.0954 | No |
| 2 | RPS6KA5 | RPS6KA5 Entrez,  Source | ribosomal protein S6 kinase, 90kDa, polypeptide 5 | 6426 | 0.040 | -0.2623 | No |
| 3 | PIK3R1 | PIK3R1 Entrez,  Source | phosphoinositide-3-kinase, regulatory subunit 1 (p85 alpha) | 8011 | 0.024 | -0.3314 | No |
| 4 | ADCY1 | ADCY1 Entrez,  Source | adenylate cyclase 1 (brain) | 9619 | 0.010 | -0.4087 | No |
| 5 | HRAS | HRAS Entrez,  Source | v-Ha-ras Harvey rat sarcoma viral oncogene homolog | 10612 | 0.001 | -0.4588 | No |
| 6 | MAPK14 | MAPK14 Entrez,  Source | mitogen-activated protein kinase 14 | 11391 | -0.006 | -0.4958 | No |
| 7 | CAMK2B | CAMK2B Entrez,  Source | calcium/calmodulin-dependent protein kinase (CaM kinase) II beta | 11597 | -0.007 | -0.5026 | No |
| 8 | AKT1 | AKT1 Entrez,  Source | v-akt murine thymoma viral oncogene homolog 1 | 11660 | -0.008 | -0.5017 | No |
| 9 | PRKAR2A | PRKAR2A Entrez,  Source | protein kinase, cAMP-dependent, regulatory, type II, alpha | 12398 | -0.016 | -0.5316 | No |
| 10 | PRKACG | PRKACG Entrez,  Source | protein kinase, cAMP-dependent, catalytic, gamma | 13683 | -0.029 | -0.5828 | No |
| 11 | GRB2 | GRB2 Entrez,  Source | growth factor receptor-bound protein 2 | 13726 | -0.030 | -0.5702 | No |
| 12 | PRKAR1A | PRKAR1A Entrez,  Source | protein kinase, cAMP-dependent, regulatory, type I, alpha (tissue specific extinguisher 1) | 14125 | -0.034 | -0.5734 | No |
| 13 | RAC1 | RAC1 Entrez,  Source | ras-related C3 botulinum toxin substrate 1 (rho family, small GTP binding protein Rac1) | 14979 | -0.046 | -0.5941 | Yes |
| 14 | PRKCA | PRKCA Entrez,  Source | protein kinase C, alpha | 15308 | -0.051 | -0.5856 | Yes |
| 15 | SOS1 | SOS1 Entrez,  Source | son of sevenless homolog 1 (Drosophila) | 15485 | -0.053 | -0.5681 | Yes |
| 16 | GNAS | GNAS Entrez,  Source | GNAS complex locus | 15494 | -0.053 | -0.5419 | Yes |
| 17 | MAPK1 | MAPK1 Entrez,  Source | mitogen-activated protein kinase 1 | 15755 | -0.058 | -0.5263 | Yes |
| 18 | CREB1 | CREB1 Entrez,  Source | cAMP responsive element binding protein 1 | 15936 | -0.061 | -0.5052 | Yes |
| 19 | CAMK2G | CAMK2G Entrez,  Source | calcium/calmodulin-dependent protein kinase (CaM kinase) II gamma | 17043 | -0.085 | -0.5196 | Yes |
| 20 | RPS6KA1 | RPS6KA1 Entrez,  Source | ribosomal protein S6 kinase, 90kDa, polypeptide 1 | 17464 | -0.096 | -0.4929 | Yes |
| 21 | PIK3CA | PIK3CA Entrez,  Source | phosphoinositide-3-kinase, catalytic, alpha polypeptide | 17601 | -0.101 | -0.4494 | Yes |
| 22 | PRKACB | PRKACB Entrez,  Source | protein kinase, cAMP-dependent, catalytic, beta | 17727 | -0.105 | -0.4034 | Yes |
| 23 | MAPK3 | MAPK3 Entrez,  Source | mitogen-activated protein kinase 3 | 18647 | -0.156 | -0.3725 | Yes |
| 24 | CAMK2D | CAMK2D Entrez,  Source | calcium/calmodulin-dependent protein kinase (CaM kinase) II delta | 18920 | -0.185 | -0.2941 | Yes |
| 25 | PRKAR2B | PRKAR2B Entrez,  Source | protein kinase, cAMP-dependent, regulatory, type II, beta | 19526 | -0.654 | 0.0015 | Yes |
Table: GSEA details [plain text format]

  

Fig 2: BIOCARTA\_CREB\_PATHWAY      
 Blue-Pink O' Gram in the Space of the Analyzed GeneSet

  

Fig 3: BIOCARTA\_CREB\_PATHWAY: Random ES distribution      
 Gene set null distribution of ES for **BIOCARTA\_CREB\_PATHWAY**

  
