# Supplementary material for: Comprehensive analysis of β-catenin target genes in colorectal carcinoma cell lines with deregulated Wnt/β-catenin signaling
Source: BMC Genomics. 2014 Jan 28;15:74. doi: 10.1186/1471-2164-15-74 (PMC3909937; doi:10.1186/1471-2164-15-74)
Supplement: Additional file 4 — GSEA analysis using the Biocarta pathway database. This zipped file contains confirming data of the GSEA analysis. The names of the directories containing the files were composed of the term ‘GSEA’, the name of the cell line, e.g. DLD1, SW480, or LS174T, and the pathway database (Biocarta). Please use a web browser to view the files with the name ‘index.html’ in the corresponding directories to start exploring the data. [file 1471-2164-15-74-S4.zip › DLD1_Biocarta/BIOCARTA_CTCF_PATHWAY.html]

Details for gene set BIOCARTA\_CTCF\_PATHWAY[GSEA]

|  || Dataset | DLD1\_collapsed\_to\_symbols.class.cls#bg\_versus\_b |
| Phenotype | class.cls#bg\_versus\_b |
| Upregulated in class | bg |
| GeneSet | BIOCARTA\_CTCF\_PATHWAY |
| Enrichment Score (ES) | 0.5192487 |
| Normalized Enrichment Score (NES) | 1.455062 |
| Nominal p-value | 0.045725647 |
| FDR q-value | 0.6001818 |
| FWER p-Value | 0.986 |
Table: GSEA Results Summary

  

Fig 1: Enrichment plot: BIOCARTA\_CTCF\_PATHWAY      
 Profile of the Running ES Score & Positions of GeneSet Members on the Rank Ordered List

  

| PROBE | GENE SYMBOL | GENE\_TITLE | RANK IN GENE LIST | RANK METRIC SCORE | RUNNING ES | CORE ENRICHMENT || 1 | MYC | MYC Entrez,  Source | v-myc myelocytomatosis viral oncogene homolog (avian) | 109 | 0.326 | 0.1859 | Yes |
| 2 | TGFB2 | TGFB2 Entrez,  Source | transforming growth factor, beta 2 | 165 | 0.290 | 0.3530 | Yes |
| 3 | TP53 | TP53 Entrez,  Source | tumor protein p53 (Li-Fraumeni syndrome) | 877 | 0.163 | 0.4123 | Yes |
| 4 | TGFB1 | TGFB1 Entrez,  Source | transforming growth factor, beta 1 (Camurati-Engelmann disease) | 1587 | 0.126 | 0.4502 | Yes |
| 5 | TGFBR3 | TGFBR3 Entrez,  Source | transforming growth factor, beta receptor III (betaglycan, 300kDa) | 2187 | 0.107 | 0.4825 | Yes |
| 6 | TGFBR2 | TGFBR2 Entrez,  Source | transforming growth factor, beta receptor II (70/80kDa) | 2589 | 0.098 | 0.5192 | Yes |
| 7 | TGFB3 | TGFB3 Entrez,  Source | transforming growth factor, beta 3 | 4247 | 0.067 | 0.4739 | No |
| 8 | CD79A | CD79A Entrez,  Source | CD79a molecule, immunoglobulin-associated alpha | 4817 | 0.059 | 0.4792 | No |
| 9 | SMAD1 | SMAD1 Entrez,  Source | SMAD, mothers against DPP homolog 1 (Drosophila) | 6106 | 0.043 | 0.4386 | No |
| 10 | SMAD5 | SMAD5 Entrez,  Source | SMAD, mothers against DPP homolog 5 (Drosophila) | 7539 | 0.028 | 0.3820 | No |
| 11 | RPS6KB1 | RPS6KB1 Entrez,  Source | ribosomal protein S6 kinase, 70kDa, polypeptide 1 | 7741 | 0.027 | 0.3872 | No |
| 12 | MDM2 | MDM2 Entrez,  Source | Mdm2, transformed 3T3 cell double minute 2, p53 binding protein (mouse) | 7751 | 0.026 | 0.4023 | No |
| 13 | CDKN2A | CDKN2A Entrez,  Source | cyclin-dependent kinase inhibitor 2A (melanoma, p16, inhibits CDK4) | 7824 | 0.026 | 0.4137 | No |
| 14 | PIK3R1 | PIK3R1 Entrez,  Source | phosphoinositide-3-kinase, regulatory subunit 1 (p85 alpha) | 8011 | 0.024 | 0.4183 | No |
| 15 | TGFBR1 | TGFBR1 Entrez,  Source | transforming growth factor, beta receptor I (activin A receptor type II-like kinase, 53kDa) | 8576 | 0.019 | 0.4006 | No |
| 16 | CD79B | CD79B Entrez,  Source | CD79b molecule, immunoglobulin-associated beta | 9875 | 0.008 | 0.3388 | No |
| 17 | PIK3CG | PIK3CG Entrez,  Source | phosphoinositide-3-kinase, catalytic, gamma polypeptide | 12525 | -0.017 | 0.2132 | No |
| 18 | PPP2CA | PPP2CA Entrez,  Source | protein phosphatase 2 (formerly 2A), catalytic subunit, alpha isoform | 13586 | -0.028 | 0.1753 | No |
| 19 | PTEN | PTEN Entrez,  Source | phosphatase and tensin homolog (mutated in multiple advanced cancers 1) | 14181 | -0.035 | 0.1654 | No |
| 20 | SMAD4 | SMAD4 Entrez,  Source | SMAD, mothers against DPP homolog 4 (Drosophila) | 14222 | -0.036 | 0.1842 | No |
| 21 | CDKN1B | CDKN1B Entrez,  Source | cyclin-dependent kinase inhibitor 1B (p27, Kip1) | 15291 | -0.050 | 0.1591 | No |
| 22 | PIK3CA | PIK3CA Entrez,  Source | phosphoinositide-3-kinase, catalytic, alpha polypeptide | 17601 | -0.101 | 0.1001 | No |
Table: GSEA details [plain text format]

  

Fig 2: BIOCARTA\_CTCF\_PATHWAY      
 Blue-Pink O' Gram in the Space of the Analyzed GeneSet

  

Fig 3: BIOCARTA\_CTCF\_PATHWAY: Random ES distribution      
 Gene set null distribution of ES for **BIOCARTA\_CTCF\_PATHWAY**

  
