# Supplementary material for: Comprehensive analysis of β-catenin target genes in colorectal carcinoma cell lines with deregulated Wnt/β-catenin signaling
Source: BMC Genomics. 2014 Jan 28;15:74. doi: 10.1186/1471-2164-15-74 (PMC3909937; doi:10.1186/1471-2164-15-74)
Supplement: Additional file 4 — GSEA analysis using the Biocarta pathway database. This zipped file contains confirming data of the GSEA analysis. The names of the directories containing the files were composed of the term ‘GSEA’, the name of the cell line, e.g. DLD1, SW480, or LS174T, and the pathway database (Biocarta). Please use a web browser to view the files with the name ‘index.html’ in the corresponding directories to start exploring the data. [file 1471-2164-15-74-S4.zip › DLD1_Biocarta/BIOCARTA_CXCR4_PATHWAY.html]

Details for gene set BIOCARTA\_CXCR4\_PATHWAY[GSEA]

|  || Dataset | DLD1\_collapsed\_to\_symbols.class.cls#bg\_versus\_b |
| Phenotype | class.cls#bg\_versus\_b |
| Upregulated in class | b |
| GeneSet | BIOCARTA\_CXCR4\_PATHWAY |
| Enrichment Score (ES) | -0.5205442 |
| Normalized Enrichment Score (NES) | -1.5390769 |
| Nominal p-value | 0.034246575 |
| FDR q-value | 0.26254442 |
| FWER p-Value | 0.933 |
Table: GSEA Results Summary

  

Fig 1: Enrichment plot: BIOCARTA\_CXCR4\_PATHWAY      
 Profile of the Running ES Score & Positions of GeneSet Members on the Rank Ordered List

  

| PROBE | GENE SYMBOL | GENE\_TITLE | RANK IN GENE LIST | RANK METRIC SCORE | RUNNING ES | CORE ENRICHMENT || 1 | PLCG1 | PLCG1 Entrez,  Source | phospholipase C, gamma 1 | 2043 | 0.111 | -0.0246 | No |
| 2 | CXCL12 | CXCL12 Entrez,  Source | chemokine (C-X-C motif) ligand 12 (stromal cell-derived factor 1) | 4038 | 0.071 | -0.0760 | No |
| 3 | GNAI1 | GNAI1 Entrez,  Source | guanine nucleotide binding protein (G protein), alpha inhibiting activity polypeptide 1 | 6049 | 0.044 | -0.1474 | No |
| 4 | PIK3R1 | PIK3R1 Entrez,  Source | phosphoinositide-3-kinase, regulatory subunit 1 (p85 alpha) | 8011 | 0.024 | -0.2305 | No |
| 5 | NFKB1 | NFKB1 Entrez,  Source | nuclear factor of kappa light polypeptide gene enhancer in B-cells 1 (p105) | 8386 | 0.021 | -0.2349 | No |
| 6 | HRAS | HRAS Entrez,  Source | v-Ha-ras Harvey rat sarcoma viral oncogene homolog | 10612 | 0.001 | -0.3479 | No |
| 7 | CXCR4 | CXCR4 Entrez,  Source | chemokine (C-X-C motif) receptor 4 | 11308 | -0.005 | -0.3799 | No |
| 8 | RELA | RELA Entrez,  Source | v-rel reticuloendotheliosis viral oncogene homolog A, nuclear factor of kappa light polypeptide gene enhancer in B-cells 3, p65 (avian) | 11371 | -0.006 | -0.3791 | No |
| 9 | GNB1 | GNB1 Entrez,  Source | guanine nucleotide binding protein (G protein), beta polypeptide 1 | 13439 | -0.027 | -0.4658 | No |
| 10 | MAP2K1 | MAP2K1 Entrez,  Source | mitogen-activated protein kinase kinase 1 | 13627 | -0.028 | -0.4550 | No |
| 11 | RAF1 | RAF1 Entrez,  Source | v-raf-1 murine leukemia viral oncogene homolog 1 | 13629 | -0.028 | -0.4346 | No |
| 12 | PRKCA | PRKCA Entrez,  Source | protein kinase C, alpha | 15308 | -0.051 | -0.4841 | Yes |
| 13 | MAPK1 | MAPK1 Entrez,  Source | mitogen-activated protein kinase 1 | 15755 | -0.058 | -0.4654 | Yes |
| 14 | PTK2 | PTK2 Entrez,  Source | PTK2 protein tyrosine kinase 2 | 16116 | -0.064 | -0.4377 | Yes |
| 15 | GNGT1 | GNGT1 Entrez,  Source | guanine nucleotide binding protein (G protein), gamma transducing activity polypeptide 1 | 16603 | -0.074 | -0.4091 | Yes |
| 16 | CRK | CRK Entrez,  Source | v-crk sarcoma virus CT10 oncogene homolog (avian) | 16851 | -0.080 | -0.3644 | Yes |
| 17 | PIK3C2G | PIK3C2G Entrez,  Source | phosphoinositide-3-kinase, class 2, gamma polypeptide | 16878 | -0.081 | -0.3077 | Yes |
| 18 | PXN | PXN Entrez,  Source | paxillin | 17157 | -0.088 | -0.2588 | Yes |
| 19 | PTK2B | PTK2B Entrez,  Source | PTK2B protein tyrosine kinase 2 beta | 17225 | -0.090 | -0.1979 | Yes |
| 20 | GNAQ | GNAQ Entrez,  Source | guanine nucleotide binding protein (G protein), q polypeptide | 17284 | -0.091 | -0.1353 | Yes |
| 21 | BCAR1 | BCAR1 Entrez,  Source | breast cancer anti-estrogen resistance 1 | 17344 | -0.093 | -0.0716 | Yes |
| 22 | PIK3CA | PIK3CA Entrez,  Source | phosphoinositide-3-kinase, catalytic, alpha polypeptide | 17601 | -0.101 | -0.0122 | Yes |
| 23 | MAPK3 | MAPK3 Entrez,  Source | mitogen-activated protein kinase 3 | 18647 | -0.156 | 0.0465 | Yes |
Table: GSEA details [plain text format]

  

Fig 2: BIOCARTA\_CXCR4\_PATHWAY      
 Blue-Pink O' Gram in the Space of the Analyzed GeneSet

  

Fig 3: BIOCARTA\_CXCR4\_PATHWAY: Random ES distribution      
 Gene set null distribution of ES for **BIOCARTA\_CXCR4\_PATHWAY**

  
