# Supplementary material for: Comprehensive analysis of β-catenin target genes in colorectal carcinoma cell lines with deregulated Wnt/β-catenin signaling
Source: BMC Genomics. 2014 Jan 28;15:74. doi: 10.1186/1471-2164-15-74 (PMC3909937; doi:10.1186/1471-2164-15-74)
Supplement: Additional file 4 — GSEA analysis using the Biocarta pathway database. This zipped file contains confirming data of the GSEA analysis. The names of the directories containing the files were composed of the term ‘GSEA’, the name of the cell line, e.g. DLD1, SW480, or LS174T, and the pathway database (Biocarta). Please use a web browser to view the files with the name ‘index.html’ in the corresponding directories to start exploring the data. [file 1471-2164-15-74-S4.zip › DLD1_Biocarta/BIOCARTA_ECM_PATHWAY.html]

Details for gene set BIOCARTA\_ECM\_PATHWAY[GSEA]

|  || Dataset | DLD1\_collapsed\_to\_symbols.class.cls#bg\_versus\_b |
| Phenotype | class.cls#bg\_versus\_b |
| Upregulated in class | b |
| GeneSet | BIOCARTA\_ECM\_PATHWAY |
| Enrichment Score (ES) | -0.6595016 |
| Normalized Enrichment Score (NES) | -1.8263716 |
| Nominal p-value | 0.0021459227 |
| FDR q-value | 0.13294135 |
| FWER p-Value | 0.268 |
Table: GSEA Results Summary

  

Fig 1: Enrichment plot: BIOCARTA\_ECM\_PATHWAY      
 Profile of the Running ES Score & Positions of GeneSet Members on the Rank Ordered List

  

| PROBE | GENE SYMBOL | GENE\_TITLE | RANK IN GENE LIST | RANK METRIC SCORE | RUNNING ES | CORE ENRICHMENT || 1 | PIK3R1 | PIK3R1 Entrez,  Source | phosphoinositide-3-kinase, regulatory subunit 1 (p85 alpha) | 8011 | 0.024 | -0.3976 | No |
| 2 | PFN1 | PFN1 Entrez,  Source | profilin 1 | 8523 | 0.019 | -0.4136 | No |
| 3 | FYN | FYN Entrez,  Source | FYN oncogene related to SRC, FGR, YES | 10299 | 0.004 | -0.5024 | No |
| 4 | HRAS | HRAS Entrez,  Source | v-Ha-ras Harvey rat sarcoma viral oncogene homolog | 10612 | 0.001 | -0.5176 | No |
| 5 | ARHGAP5 | ARHGAP5 Entrez,  Source | Rho GTPase activating protein 5 | 10925 | -0.001 | -0.5328 | No |
| 6 | TLN1 | TLN1 Entrez,  Source | talin 1 | 12261 | -0.014 | -0.5938 | No |
| 7 | PIK3CG | PIK3CG Entrez,  Source | phosphoinositide-3-kinase, catalytic, gamma polypeptide | 12525 | -0.017 | -0.5984 | No |
| 8 | SHC1 | SHC1 Entrez,  Source | SHC (Src homology 2 domain containing) transforming protein 1 | 12661 | -0.018 | -0.5958 | No |
| 9 | RHOA | RHOA Entrez,  Source | ras homolog gene family, member A | 13129 | -0.023 | -0.6077 | No |
| 10 | DIAPH1 | DIAPH1 Entrez,  Source | diaphanous homolog 1 (Drosophila) | 13325 | -0.025 | -0.6045 | No |
| 11 | MAP2K1 | MAP2K1 Entrez,  Source | mitogen-activated protein kinase kinase 1 | 13627 | -0.028 | -0.6051 | No |
| 12 | RAF1 | RAF1 Entrez,  Source | v-raf-1 murine leukemia viral oncogene homolog 1 | 13629 | -0.028 | -0.5904 | No |
| 13 | ROCK1 | ROCK1 Entrez,  Source | Rho-associated, coiled-coil containing protein kinase 1 | 13714 | -0.029 | -0.5794 | No |
| 14 | ITGB1 | ITGB1 Entrez,  Source | integrin, beta 1 (fibronectin receptor, beta polypeptide, antigen CD29 includes MDF2, MSK12) | 14535 | -0.040 | -0.6008 | No |
| 15 | SRC | SRC Entrez,  Source | v-src sarcoma (Schmidt-Ruppin A-2) viral oncogene homolog (avian) | 15682 | -0.056 | -0.6302 | Yes |
| 16 | MAPK1 | MAPK1 Entrez,  Source | mitogen-activated protein kinase 1 | 15755 | -0.058 | -0.6038 | Yes |
| 17 | PTK2 | PTK2 Entrez,  Source | PTK2 protein tyrosine kinase 2 | 16116 | -0.064 | -0.5889 | Yes |
| 18 | PXN | PXN Entrez,  Source | paxillin | 17157 | -0.088 | -0.5965 | Yes |
| 19 | PIK3CA | PIK3CA Entrez,  Source | phosphoinositide-3-kinase, catalytic, alpha polypeptide | 17601 | -0.101 | -0.5667 | Yes |
| 20 | MAPK3 | MAPK3 Entrez,  Source | mitogen-activated protein kinase 3 | 18647 | -0.156 | -0.5391 | Yes |
| 21 | MYL2 | MYL2 Entrez,  Source | myosin, light chain 2, regulatory, cardiac, slow | 18730 | -0.164 | -0.4580 | Yes |
| 22 | GSN | GSN Entrez,  Source | gelsolin (amyloidosis, Finnish type) | 19432 | -0.405 | -0.2831 | Yes |
| 23 | MYLK | MYLK Entrez,  Source | myosin, light chain kinase | 19507 | -0.557 | 0.0025 | Yes |
Table: GSEA details [plain text format]

  

Fig 2: BIOCARTA\_ECM\_PATHWAY      
 Blue-Pink O' Gram in the Space of the Analyzed GeneSet

  

Fig 3: BIOCARTA\_ECM\_PATHWAY: Random ES distribution      
 Gene set null distribution of ES for **BIOCARTA\_ECM\_PATHWAY**

  
