# Supplementary material for: Comprehensive analysis of β-catenin target genes in colorectal carcinoma cell lines with deregulated Wnt/β-catenin signaling
Source: BMC Genomics. 2014 Jan 28;15:74. doi: 10.1186/1471-2164-15-74 (PMC3909937; doi:10.1186/1471-2164-15-74)
Supplement: Additional file 4 — GSEA analysis using the Biocarta pathway database. This zipped file contains confirming data of the GSEA analysis. The names of the directories containing the files were composed of the term ‘GSEA’, the name of the cell line, e.g. DLD1, SW480, or LS174T, and the pathway database (Biocarta). Please use a web browser to view the files with the name ‘index.html’ in the corresponding directories to start exploring the data. [file 1471-2164-15-74-S4.zip › DLD1_Biocarta/BIOCARTA_EDG1_PATHWAY.html]

Details for gene set BIOCARTA\_EDG1\_PATHWAY[GSEA]

|  || Dataset | DLD1\_collapsed\_to\_symbols.class.cls#bg\_versus\_b |
| Phenotype | class.cls#bg\_versus\_b |
| Upregulated in class | b |
| GeneSet | BIOCARTA\_EDG1\_PATHWAY |
| Enrichment Score (ES) | -0.5587951 |
| Normalized Enrichment Score (NES) | -1.5871704 |
| Nominal p-value | 0.027484143 |
| FDR q-value | 0.24637096 |
| FWER p-Value | 0.864 |
Table: GSEA Results Summary

  

Fig 1: Enrichment plot: BIOCARTA\_EDG1\_PATHWAY      
 Profile of the Running ES Score & Positions of GeneSet Members on the Rank Ordered List

  

| PROBE | GENE SYMBOL | GENE\_TITLE | RANK IN GENE LIST | RANK METRIC SCORE | RUNNING ES | CORE ENRICHMENT || 1 | SMPD2 | SMPD2 Entrez,  Source | sphingomyelin phosphodiesterase 2, neutral membrane (neutral sphingomyelinase) | 5606 | 0.049 | -0.2524 | No |
| 2 | SPHK1 | SPHK1 Entrez,  Source | sphingosine kinase 1 | 5617 | 0.048 | -0.2185 | No |
| 3 | GNAI1 | GNAI1 Entrez,  Source | guanine nucleotide binding protein (G protein), alpha inhibiting activity polypeptide 1 | 6049 | 0.044 | -0.2094 | No |
| 4 | PIK3R1 | PIK3R1 Entrez,  Source | phosphoinositide-3-kinase, regulatory subunit 1 (p85 alpha) | 8011 | 0.024 | -0.2927 | No |
| 5 | PDGFRA | PDGFRA Entrez,  Source | platelet-derived growth factor receptor, alpha polypeptide | 9441 | 0.011 | -0.3577 | No |
| 6 | ADCY1 | ADCY1 Entrez,  Source | adenylate cyclase 1 (brain) | 9619 | 0.010 | -0.3596 | No |
| 7 | ITGB3 | ITGB3 Entrez,  Source | integrin, beta 3 (platelet glycoprotein IIIa, antigen CD61) | 10205 | 0.005 | -0.3861 | No |
| 8 | AKT1 | AKT1 Entrez,  Source | v-akt murine thymoma viral oncogene homolog 1 | 11660 | -0.008 | -0.4548 | No |
| 9 | PIK3CG | PIK3CG Entrez,  Source | phosphoinositide-3-kinase, catalytic, gamma polypeptide | 12525 | -0.017 | -0.4869 | No |
| 10 | RHOA | RHOA Entrez,  Source | ras homolog gene family, member A | 13129 | -0.023 | -0.5013 | No |
| 11 | PLCB1 | PLCB1 Entrez,  Source | phospholipase C, beta 1 (phosphoinositide-specific) | 13331 | -0.025 | -0.4934 | No |
| 12 | GNB1 | GNB1 Entrez,  Source | guanine nucleotide binding protein (G protein), beta polypeptide 1 | 13439 | -0.027 | -0.4800 | No |
| 13 | RAC1 | RAC1 Entrez,  Source | ras-related C3 botulinum toxin substrate 1 (rho family, small GTP binding protein Rac1) | 14979 | -0.046 | -0.5261 | Yes |
| 14 | PRKCA | PRKCA Entrez,  Source | protein kinase C, alpha | 15308 | -0.051 | -0.5068 | Yes |
| 15 | ITGAV | ITGAV Entrez,  Source | integrin, alpha V (vitronectin receptor, alpha polypeptide, antigen CD51) | 15309 | -0.051 | -0.4707 | Yes |
| 16 | SRC | SRC Entrez,  Source | v-src sarcoma (Schmidt-Ruppin A-2) viral oncogene homolog (avian) | 15682 | -0.056 | -0.4496 | Yes |
| 17 | MAPK1 | MAPK1 Entrez,  Source | mitogen-activated protein kinase 1 | 15755 | -0.058 | -0.4122 | Yes |
| 18 | PTK2 | PTK2 Entrez,  Source | PTK2 protein tyrosine kinase 2 | 16116 | -0.064 | -0.3849 | Yes |
| 19 | GNGT1 | GNGT1 Entrez,  Source | guanine nucleotide binding protein (G protein), gamma transducing activity polypeptide 1 | 16603 | -0.074 | -0.3569 | Yes |
| 20 | PIK3CA | PIK3CA Entrez,  Source | phosphoinositide-3-kinase, catalytic, alpha polypeptide | 17601 | -0.101 | -0.3360 | Yes |
| 21 | PDGFA | PDGFA Entrez,  Source | platelet-derived growth factor alpha polypeptide | 18367 | -0.135 | -0.2788 | Yes |
| 22 | MAPK3 | MAPK3 Entrez,  Source | mitogen-activated protein kinase 3 | 18647 | -0.156 | -0.1819 | Yes |
| 23 | ASAH1 | ASAH1 Entrez,  Source | N-acylsphingosine amidohydrolase (acid ceramidase) 1 | 18681 | -0.159 | -0.0701 | Yes |
| 24 | SMPD1 | SMPD1 Entrez,  Source | sphingomyelin phosphodiesterase 1, acid lysosomal (acid sphingomyelinase) | 18700 | -0.161 | 0.0438 | Yes |
Table: GSEA details [plain text format]

  

Fig 2: BIOCARTA\_EDG1\_PATHWAY      
 Blue-Pink O' Gram in the Space of the Analyzed GeneSet

  

Fig 3: BIOCARTA\_EDG1\_PATHWAY: Random ES distribution      
 Gene set null distribution of ES for **BIOCARTA\_EDG1\_PATHWAY**

  
