# Supplementary material for: Comprehensive analysis of β-catenin target genes in colorectal carcinoma cell lines with deregulated Wnt/β-catenin signaling
Source: BMC Genomics. 2014 Jan 28;15:74. doi: 10.1186/1471-2164-15-74 (PMC3909937; doi:10.1186/1471-2164-15-74)
Supplement: Additional file 4 — GSEA analysis using the Biocarta pathway database. This zipped file contains confirming data of the GSEA analysis. The names of the directories containing the files were composed of the term ‘GSEA’, the name of the cell line, e.g. DLD1, SW480, or LS174T, and the pathway database (Biocarta). Please use a web browser to view the files with the name ‘index.html’ in the corresponding directories to start exploring the data. [file 1471-2164-15-74-S4.zip › DLD1_Biocarta/BIOCARTA_ERK5_PATHWAY.html]

Details for gene set BIOCARTA\_ERK5\_PATHWAY[GSEA]

|  || Dataset | DLD1\_collapsed\_to\_symbols.class.cls#bg\_versus\_b |
| Phenotype | class.cls#bg\_versus\_b |
| Upregulated in class | b |
| GeneSet | BIOCARTA\_ERK5\_PATHWAY |
| Enrichment Score (ES) | -0.54432446 |
| Normalized Enrichment Score (NES) | -1.4666648 |
| Nominal p-value | 0.1010989 |
| FDR q-value | 0.32696444 |
| FWER p-Value | 0.986 |
Table: GSEA Results Summary

  

Fig 1: Enrichment plot: BIOCARTA\_ERK5\_PATHWAY      
 Profile of the Running ES Score & Positions of GeneSet Members on the Rank Ordered List

  

| PROBE | GENE SYMBOL | GENE\_TITLE | RANK IN GENE LIST | RANK METRIC SCORE | RUNNING ES | CORE ENRICHMENT || 1 | PLCG1 | PLCG1 Entrez,  Source | phospholipase C, gamma 1 | 2043 | 0.111 | 0.0056 | No |
| 2 | PIK3R1 | PIK3R1 Entrez,  Source | phosphoinositide-3-kinase, regulatory subunit 1 (p85 alpha) | 8011 | 0.024 | -0.2760 | No |
| 3 | HRAS | HRAS Entrez,  Source | v-Ha-ras Harvey rat sarcoma viral oncogene homolog | 10612 | 0.001 | -0.4077 | No |
| 4 | AKT1 | AKT1 Entrez,  Source | v-akt murine thymoma viral oncogene homolog 1 | 11660 | -0.008 | -0.4533 | No |
| 5 | PIK3CG | PIK3CG Entrez,  Source | phosphoinositide-3-kinase, catalytic, gamma polypeptide | 12525 | -0.017 | -0.4807 | No |
| 6 | SHC1 | SHC1 Entrez,  Source | SHC (Src homology 2 domain containing) transforming protein 1 | 12661 | -0.018 | -0.4695 | No |
| 7 | MAPK7 | MAPK7 Entrez,  Source | mitogen-activated protein kinase 7 | 13441 | -0.027 | -0.4831 | No |
| 8 | GRB2 | GRB2 Entrez,  Source | growth factor receptor-bound protein 2 | 13726 | -0.030 | -0.4683 | No |
| 9 | NTRK1 | NTRK1 Entrez,  Source | neurotrophic tyrosine kinase, receptor, type 1 | 15212 | -0.049 | -0.4956 | Yes |
| 10 | MAPK1 | MAPK1 Entrez,  Source | mitogen-activated protein kinase 1 | 15755 | -0.058 | -0.4661 | Yes |
| 11 | CREB1 | CREB1 Entrez,  Source | cAMP responsive element binding protein 1 | 15936 | -0.061 | -0.4152 | Yes |
| 12 | MEF2A | MEF2A Entrez,  Source | MADS box transcription enhancer factor 2, polypeptide A (myocyte enhancer factor 2A) | 16103 | -0.064 | -0.3604 | Yes |
| 13 | MEF2D | MEF2D Entrez,  Source | MADS box transcription enhancer factor 2, polypeptide D (myocyte enhancer factor 2D) | 16891 | -0.081 | -0.3205 | Yes |
| 14 | RPS6KA1 | RPS6KA1 Entrez,  Source | ribosomal protein S6 kinase, 90kDa, polypeptide 1 | 17464 | -0.096 | -0.2542 | Yes |
| 15 | PIK3CA | PIK3CA Entrez,  Source | phosphoinositide-3-kinase, catalytic, alpha polypeptide | 17601 | -0.101 | -0.1613 | Yes |
| 16 | MEF2C | MEF2C Entrez,  Source | MADS box transcription enhancer factor 2, polypeptide C (myocyte enhancer factor 2C) | 17808 | -0.108 | -0.0651 | Yes |
| 17 | MAPK3 | MAPK3 Entrez,  Source | mitogen-activated protein kinase 3 | 18647 | -0.156 | 0.0465 | Yes |
Table: GSEA details [plain text format]

  

Fig 2: BIOCARTA\_ERK5\_PATHWAY      
 Blue-Pink O' Gram in the Space of the Analyzed GeneSet

  

Fig 3: BIOCARTA\_ERK5\_PATHWAY: Random ES distribution      
 Gene set null distribution of ES for **BIOCARTA\_ERK5\_PATHWAY**

  
