# Supplementary material for: Comprehensive analysis of β-catenin target genes in colorectal carcinoma cell lines with deregulated Wnt/β-catenin signaling
Source: BMC Genomics. 2014 Jan 28;15:74. doi: 10.1186/1471-2164-15-74 (PMC3909937; doi:10.1186/1471-2164-15-74)
Supplement: Additional file 4 — GSEA analysis using the Biocarta pathway database. This zipped file contains confirming data of the GSEA analysis. The names of the directories containing the files were composed of the term ‘GSEA’, the name of the cell line, e.g. DLD1, SW480, or LS174T, and the pathway database (Biocarta). Please use a web browser to view the files with the name ‘index.html’ in the corresponding directories to start exploring the data. [file 1471-2164-15-74-S4.zip › DLD1_Biocarta/BIOCARTA_FAS_PATHWAY.html]

Details for gene set BIOCARTA\_FAS\_PATHWAY[GSEA]

|  || Dataset | DLD1\_collapsed\_to\_symbols.class.cls#bg\_versus\_b |
| Phenotype | class.cls#bg\_versus\_b |
| Upregulated in class | bg |
| GeneSet | BIOCARTA\_FAS\_PATHWAY |
| Enrichment Score (ES) | 0.42353126 |
| Normalized Enrichment Score (NES) | 1.298548 |
| Nominal p-value | 0.1484375 |
| FDR q-value | 0.536249 |
| FWER p-Value | 1.0 |
Table: GSEA Results Summary

  

Fig 1: Enrichment plot: BIOCARTA\_FAS\_PATHWAY      
 Profile of the Running ES Score & Positions of GeneSet Members on the Rank Ordered List

  

| PROBE | GENE SYMBOL | GENE\_TITLE | RANK IN GENE LIST | RANK METRIC SCORE | RUNNING ES | CORE ENRICHMENT || 1 | FASLG | FASLG Entrez,  Source | Fas ligand (TNF superfamily, member 6) | 315 | 0.234 | 0.1013 | Yes |
| 2 | FAS | FAS Entrez,  Source | Fas (TNF receptor superfamily, member 6) | 1068 | 0.151 | 0.1386 | Yes |
| 3 | DFFB | DFFB Entrez,  Source | DNA fragmentation factor, 40kDa, beta polypeptide (caspase-activated DNase) | 1215 | 0.143 | 0.2032 | Yes |
| 4 | LMNB1 | LMNB1 Entrez,  Source | lamin B1 | 1312 | 0.138 | 0.2678 | Yes |
| 5 | PARP1 | PARP1 Entrez,  Source | poly (ADP-ribose) polymerase family, member 1 | 2214 | 0.107 | 0.2753 | Yes |
| 6 | JUN | JUN Entrez,  Source | jun oncogene | 2676 | 0.096 | 0.2999 | Yes |
| 7 | FAF1 | FAF1 Entrez,  Source | Fas (TNFRSF6) associated factor 1 | 3268 | 0.084 | 0.3119 | Yes |
| 8 | PTPN13 | PTPN13 Entrez,  Source | protein tyrosine phosphatase, non-receptor type 13 (APO-1/CD95 (Fas)-associated phosphatase) | 3360 | 0.082 | 0.3485 | Yes |
| 9 | PRKDC | PRKDC Entrez,  Source | protein kinase, DNA-activated, catalytic polypeptide | 3456 | 0.080 | 0.3841 | Yes |
| 10 | CASP6 | CASP6 Entrez,  Source | caspase 6, apoptosis-related cysteine peptidase | 3722 | 0.076 | 0.4087 | Yes |
| 11 | DFFA | DFFA Entrez,  Source | DNA fragmentation factor, 45kDa, alpha polypeptide | 4362 | 0.065 | 0.4089 | Yes |
| 12 | MAP3K1 | MAP3K1 Entrez,  Source | mitogen-activated protein kinase kinase kinase 1 | 4699 | 0.061 | 0.4222 | Yes |
| 13 | DAXX | DAXX Entrez,  Source | death-associated protein 6 | 5201 | 0.054 | 0.4235 | Yes |
| 14 | RIPK2 | RIPK2 Entrez,  Source | receptor-interacting serine-threonine kinase 2 | 6020 | 0.044 | 0.4037 | No |
| 15 | ARHGDIB | ARHGDIB Entrez,  Source | Rho GDP dissociation inhibitor (GDI) beta | 7894 | 0.025 | 0.3205 | No |
| 16 | FADD | FADD Entrez,  Source | Fas (TNFRSF6)-associated via death domain | 8168 | 0.023 | 0.3178 | No |
| 17 | LMNB2 | LMNB2 Entrez,  Source | lamin B2 | 9001 | 0.015 | 0.2830 | No |
| 18 | MAPK8 | MAPK8 Entrez,  Source | mitogen-activated protein kinase 8 | 9088 | 0.015 | 0.2861 | No |
| 19 | CASP8 | CASP8 Entrez,  Source | caspase 8, apoptosis-related cysteine peptidase | 10101 | 0.006 | 0.2372 | No |
| 20 | RB1 | RB1 Entrez,  Source | retinoblastoma 1 (including osteosarcoma) | 11565 | -0.007 | 0.1659 | No |
| 21 | CASP3 | CASP3 Entrez,  Source | caspase 3, apoptosis-related cysteine peptidase | 13271 | -0.025 | 0.0910 | No |
| 22 | PAK2 | PAK2 Entrez,  Source | p21 (CDKN1A)-activated kinase 2 | 13948 | -0.032 | 0.0726 | No |
| 23 | MAP3K7 | MAP3K7 Entrez,  Source | mitogen-activated protein kinase kinase kinase 7 | 14015 | -0.033 | 0.0858 | No |
| 24 | MAP2K4 | MAP2K4 Entrez,  Source | mitogen-activated protein kinase kinase 4 | 14064 | -0.034 | 0.1001 | No |
| 25 | LMNA | LMNA Entrez,  Source | lamin A/C | 14301 | -0.037 | 0.1064 | No |
| 26 | SPTAN1 | SPTAN1 Entrez,  Source | spectrin, alpha, non-erythrocytic 1 (alpha-fodrin) | 15555 | -0.054 | 0.0696 | No |
| 27 | CFLAR | CFLAR Entrez,  Source | CASP8 and FADD-like apoptosis regulator | 15664 | -0.056 | 0.0924 | No |
| 28 | CASP7 | CASP7 Entrez,  Source | caspase 7, apoptosis-related cysteine peptidase | 15821 | -0.059 | 0.1140 | No |
| 29 | PAK1 | PAK1 Entrez,  Source | p21/Cdc42/Rac1-activated kinase 1 (STE20 homolog, yeast) | 16291 | -0.067 | 0.1238 | No |
| 30 | CASP10 | CASP10 Entrez,  Source | caspase 10, apoptosis-related cysteine peptidase | 17090 | -0.086 | 0.1263 | No |
Table: GSEA details [plain text format]

  

Fig 2: BIOCARTA\_FAS\_PATHWAY      
 Blue-Pink O' Gram in the Space of the Analyzed GeneSet

  

Fig 3: BIOCARTA\_FAS\_PATHWAY: Random ES distribution      
 Gene set null distribution of ES for **BIOCARTA\_FAS\_PATHWAY**

  
