# Supplementary material for: Comprehensive analysis of β-catenin target genes in colorectal carcinoma cell lines with deregulated Wnt/β-catenin signaling
Source: BMC Genomics. 2014 Jan 28;15:74. doi: 10.1186/1471-2164-15-74 (PMC3909937; doi:10.1186/1471-2164-15-74)
Supplement: Additional file 4 — GSEA analysis using the Biocarta pathway database. This zipped file contains confirming data of the GSEA analysis. The names of the directories containing the files were composed of the term ‘GSEA’, the name of the cell line, e.g. DLD1, SW480, or LS174T, and the pathway database (Biocarta). Please use a web browser to view the files with the name ‘index.html’ in the corresponding directories to start exploring the data. [file 1471-2164-15-74-S4.zip › DLD1_Biocarta/BIOCARTA_G1_PATHWAY.html]

Details for gene set BIOCARTA\_G1\_PATHWAY[GSEA]

|  || Dataset | DLD1\_collapsed\_to\_symbols.class.cls#bg\_versus\_b |
| Phenotype | class.cls#bg\_versus\_b |
| Upregulated in class | bg |
| GeneSet | BIOCARTA\_G1\_PATHWAY |
| Enrichment Score (ES) | 0.45084462 |
| Normalized Enrichment Score (NES) | 1.3295119 |
| Nominal p-value | 0.11923077 |
| FDR q-value | 0.53279746 |
| FWER p-Value | 0.999 |
Table: GSEA Results Summary

  

Fig 1: Enrichment plot: BIOCARTA\_G1\_PATHWAY      
 Profile of the Running ES Score & Positions of GeneSet Members on the Rank Ordered List

  

| PROBE | GENE SYMBOL | GENE\_TITLE | RANK IN GENE LIST | RANK METRIC SCORE | RUNNING ES | CORE ENRICHMENT || 1 | CDK6 | CDK6 Entrez,  Source | cyclin-dependent kinase 6 | 95 | 0.339 | 0.0945 | Yes |
| 2 | TGFB2 | TGFB2 Entrez,  Source | transforming growth factor, beta 2 | 165 | 0.290 | 0.1760 | Yes |
| 3 | CDC25A | CDC25A Entrez,  Source | cell division cycle 25A | 345 | 0.225 | 0.2329 | Yes |
| 4 | CDK4 | CDK4 Entrez,  Source | cyclin-dependent kinase 4 | 602 | 0.190 | 0.2755 | Yes |
| 5 | CCNE1 | CCNE1 Entrez,  Source | cyclin E1 | 722 | 0.178 | 0.3217 | Yes |
| 6 | TP53 | TP53 Entrez,  Source | tumor protein p53 (Li-Fraumeni syndrome) | 877 | 0.163 | 0.3617 | Yes |
| 7 | SKP2 | SKP2 Entrez,  Source | S-phase kinase-associated protein 2 (p45) | 1031 | 0.153 | 0.3987 | Yes |
| 8 | CDK2 | CDK2 Entrez,  Source | cyclin-dependent kinase 2 | 1250 | 0.141 | 0.4291 | Yes |
| 9 | TGFB1 | TGFB1 Entrez,  Source | transforming growth factor, beta 1 (Camurati-Engelmann disease) | 1587 | 0.126 | 0.4490 | Yes |
| 10 | E2F1 | E2F1 Entrez,  Source | E2F transcription factor 1 | 2635 | 0.097 | 0.4238 | Yes |
| 11 | ATR | ATR Entrez,  Source | ataxia telangiectasia and Rad3 related | 2660 | 0.096 | 0.4508 | Yes |
| 12 | TFDP1 | TFDP1 Entrez,  Source | transcription factor Dp-1 | 3284 | 0.084 | 0.4435 | No |
| 13 | CCND1 | CCND1 Entrez,  Source | cyclin D1 | 3816 | 0.074 | 0.4381 | No |
| 14 | TGFB3 | TGFB3 Entrez,  Source | transforming growth factor, beta 3 | 4247 | 0.067 | 0.4358 | No |
| 15 | ABL1 | ABL1 Entrez,  Source | v-abl Abelson murine leukemia viral oncogene homolog 1 | 7530 | 0.029 | 0.2761 | No |
| 16 | ATM | ATM Entrez,  Source | ataxia telangiectasia mutated (includes complementation groups A, C and D) | 7603 | 0.028 | 0.2806 | No |
| 17 | CDKN2A | CDKN2A Entrez,  Source | cyclin-dependent kinase inhibitor 2A (melanoma, p16, inhibits CDK4) | 7824 | 0.026 | 0.2769 | No |
| 18 | RB1 | RB1 Entrez,  Source | retinoblastoma 1 (including osteosarcoma) | 11565 | -0.007 | 0.0876 | No |
| 19 | CCNA1 | CCNA1 Entrez,  Source | cyclin A1 | 12830 | -0.020 | 0.0288 | No |
| 20 | SMAD4 | SMAD4 Entrez,  Source | SMAD, mothers against DPP homolog 4 (Drosophila) | 14222 | -0.036 | -0.0320 | No |
| 21 | CDKN1B | CDKN1B Entrez,  Source | cyclin-dependent kinase inhibitor 1B (p27, Kip1) | 15291 | -0.050 | -0.0719 | No |
| 22 | GSK3B | GSK3B Entrez,  Source | glycogen synthase kinase 3 beta | 15827 | -0.059 | -0.0820 | No |
| 23 | HDAC1 | HDAC1 Entrez,  Source | histone deacetylase 1 | 16495 | -0.072 | -0.0950 | No |
| 24 | CDKN1A | CDKN1A Entrez,  Source | cyclin-dependent kinase inhibitor 1A (p21, Cip1) | 17512 | -0.098 | -0.1183 | No |
| 25 | SMAD3 | SMAD3 Entrez,  Source | SMAD, mothers against DPP homolog 3 (Drosophila) | 17945 | -0.113 | -0.1071 | No |
| 26 | CDKN2B | CDKN2B Entrez,  Source | cyclin-dependent kinase inhibitor 2B (p15, inhibits CDK4) | 19522 | -0.646 | 0.0017 | No |
Table: GSEA details [plain text format]

  

Fig 2: BIOCARTA\_G1\_PATHWAY      
 Blue-Pink O' Gram in the Space of the Analyzed GeneSet

  

Fig 3: BIOCARTA\_G1\_PATHWAY: Random ES distribution      
 Gene set null distribution of ES for **BIOCARTA\_G1\_PATHWAY**

  
