# Supplementary material for: Comprehensive analysis of β-catenin target genes in colorectal carcinoma cell lines with deregulated Wnt/β-catenin signaling
Source: BMC Genomics. 2014 Jan 28;15:74. doi: 10.1186/1471-2164-15-74 (PMC3909937; doi:10.1186/1471-2164-15-74)
Supplement: Additional file 4 — GSEA analysis using the Biocarta pathway database. This zipped file contains confirming data of the GSEA analysis. The names of the directories containing the files were composed of the term ‘GSEA’, the name of the cell line, e.g. DLD1, SW480, or LS174T, and the pathway database (Biocarta). Please use a web browser to view the files with the name ‘index.html’ in the corresponding directories to start exploring the data. [file 1471-2164-15-74-S4.zip › DLD1_Biocarta/BIOCARTA_G2_PATHWAY.html]

Details for gene set BIOCARTA\_G2\_PATHWAY[GSEA]

|  || Dataset | DLD1\_collapsed\_to\_symbols.class.cls#bg\_versus\_b |
| Phenotype | class.cls#bg\_versus\_b |
| Upregulated in class | bg |
| GeneSet | BIOCARTA\_G2\_PATHWAY |
| Enrichment Score (ES) | 0.42460865 |
| Normalized Enrichment Score (NES) | 1.2229109 |
| Nominal p-value | 0.20185184 |
| FDR q-value | 0.63484097 |
| FWER p-Value | 1.0 |
Table: GSEA Results Summary

  

Fig 1: Enrichment plot: BIOCARTA\_G2\_PATHWAY      
 Profile of the Running ES Score & Positions of GeneSet Members on the Rank Ordered List

  

| PROBE | GENE SYMBOL | GENE\_TITLE | RANK IN GENE LIST | RANK METRIC SCORE | RUNNING ES | CORE ENRICHMENT || 1 | CDC25A | CDC25A Entrez,  Source | cell division cycle 25A | 345 | 0.225 | 0.1053 | Yes |
| 2 | BRCA1 | BRCA1 Entrez,  Source | breast cancer 1, early onset | 674 | 0.182 | 0.1877 | Yes |
| 3 | TP53 | TP53 Entrez,  Source | tumor protein p53 (Li-Fraumeni syndrome) | 877 | 0.163 | 0.2665 | Yes |
| 4 | MYT1 | MYT1 Entrez,  Source | myelin transcription factor 1 | 1005 | 0.155 | 0.3445 | Yes |
| 5 | CHEK1 | CHEK1 Entrez,  Source | CHK1 checkpoint homolog (S. pombe) | 2393 | 0.102 | 0.3294 | Yes |
| 6 | CHEK2 | CHEK2 Entrez,  Source | CHK2 checkpoint homolog (S. pombe) | 2615 | 0.097 | 0.3711 | Yes |
| 7 | ATR | ATR Entrez,  Source | ataxia telangiectasia and Rad3 related | 2660 | 0.096 | 0.4214 | Yes |
| 8 | PRKDC | PRKDC Entrez,  Source | protein kinase, DNA-activated, catalytic polypeptide | 3456 | 0.080 | 0.4246 | Yes |
| 9 | WEE1 | WEE1 Entrez,  Source | WEE1 homolog (S. pombe) | 4545 | 0.063 | 0.4033 | No |
| 10 | PLK1 | PLK1 Entrez,  Source | polo-like kinase 1 (Drosophila) | 5594 | 0.049 | 0.3763 | No |
| 11 | YWHAH | YWHAH Entrez,  Source | tyrosine 3-monooxygenase/tryptophan 5-monooxygenase activation protein, eta polypeptide | 7503 | 0.029 | 0.2944 | No |
| 12 | ATM | ATM Entrez,  Source | ataxia telangiectasia mutated (includes complementation groups A, C and D) | 7603 | 0.028 | 0.3045 | No |
| 13 | MDM2 | MDM2 Entrez,  Source | Mdm2, transformed 3T3 cell double minute 2, p53 binding protein (mouse) | 7751 | 0.026 | 0.3114 | No |
| 14 | EP300 | EP300 Entrez,  Source | E1A binding protein p300 | 7847 | 0.026 | 0.3205 | No |
| 15 | CDC25C | CDC25C Entrez,  Source | cell division cycle 25C | 8918 | 0.016 | 0.2746 | No |
| 16 | CCNB1 | CCNB1 Entrez,  Source | cyclin B1 | 9414 | 0.012 | 0.2556 | No |
| 17 | GADD45A | GADD45A Entrez,  Source | growth arrest and DNA-damage-inducible, alpha | 10698 | 0.001 | 0.1902 | No |
| 18 | YWHAQ | YWHAQ Entrez,  Source | tyrosine 3-monooxygenase/tryptophan 5-monooxygenase activation protein, theta polypeptide | 11296 | -0.005 | 0.1623 | No |
| 19 | CDC25B | CDC25B Entrez,  Source | cell division cycle 25B | 13410 | -0.026 | 0.0684 | No |
| 20 | CDC34 | CDC34 Entrez,  Source | cell division cycle 34 | 15799 | -0.058 | -0.0219 | No |
| 21 | RPS6KA1 | RPS6KA1 Entrez,  Source | ribosomal protein S6 kinase, 90kDa, polypeptide 1 | 17464 | -0.096 | -0.0543 | No |
| 22 | CDKN1A | CDKN1A Entrez,  Source | cyclin-dependent kinase inhibitor 1A (p21, Cip1) | 17512 | -0.098 | -0.0033 | No |
| 23 | CDKN2D | CDKN2D Entrez,  Source | cyclin-dependent kinase inhibitor 2D (p19, inhibits CDK4) | 19006 | -0.197 | 0.0282 | No |
Table: GSEA details [plain text format]

  

Fig 2: BIOCARTA\_G2\_PATHWAY      
 Blue-Pink O' Gram in the Space of the Analyzed GeneSet

  

Fig 3: BIOCARTA\_G2\_PATHWAY: Random ES distribution      
 Gene set null distribution of ES for **BIOCARTA\_G2\_PATHWAY**

  
