# Supplementary material for: Comprehensive analysis of β-catenin target genes in colorectal carcinoma cell lines with deregulated Wnt/β-catenin signaling
Source: BMC Genomics. 2014 Jan 28;15:74. doi: 10.1186/1471-2164-15-74 (PMC3909937; doi:10.1186/1471-2164-15-74)
Supplement: Additional file 4 — GSEA analysis using the Biocarta pathway database. This zipped file contains confirming data of the GSEA analysis. The names of the directories containing the files were composed of the term ‘GSEA’, the name of the cell line, e.g. DLD1, SW480, or LS174T, and the pathway database (Biocarta). Please use a web browser to view the files with the name ‘index.html’ in the corresponding directories to start exploring the data. [file 1471-2164-15-74-S4.zip › DLD1_Biocarta/BIOCARTA_IL10_PATHWAY.html]

Details for gene set BIOCARTA\_IL10\_PATHWAY[GSEA]

|  || Dataset | DLD1\_collapsed\_to\_symbols.class.cls#bg\_versus\_b |
| Phenotype | class.cls#bg\_versus\_b |
| Upregulated in class | b |
| GeneSet | BIOCARTA\_IL10\_PATHWAY |
| Enrichment Score (ES) | -0.6390696 |
| Normalized Enrichment Score (NES) | -1.7116228 |
| Nominal p-value | 0.014675053 |
| FDR q-value | 0.18470582 |
| FWER p-Value | 0.561 |
Table: GSEA Results Summary

  

Fig 1: Enrichment plot: BIOCARTA\_IL10\_PATHWAY      
 Profile of the Running ES Score & Positions of GeneSet Members on the Rank Ordered List

  

| PROBE | GENE SYMBOL | GENE\_TITLE | RANK IN GENE LIST | RANK METRIC SCORE | RUNNING ES | CORE ENRICHMENT || 1 | IL10 | IL10 Entrez,  Source | interleukin 10 | 5449 | 0.051 | -0.2339 | No |
| 2 | STAT4 | STAT4 Entrez,  Source | signal transducer and activator of transcription 4 | 10764 | 0.000 | -0.5058 | No |
| 3 | TNF | TNF Entrez,  Source | tumor necrosis factor (TNF superfamily, member 2) | 11808 | -0.010 | -0.5507 | No |
| 4 | IL6 | IL6 Entrez,  Source | interleukin 6 (interferon, beta 2) | 12018 | -0.012 | -0.5509 | No |
| 5 | STAT6 | STAT6 Entrez,  Source | signal transducer and activator of transcription 6, interleukin-4 induced | 13482 | -0.027 | -0.6019 | No |
| 6 | STAT3 | STAT3 Entrez,  Source | signal transducer and activator of transcription 3 (acute-phase response factor) | 14210 | -0.035 | -0.6076 | Yes |
| 7 | STAT2 | STAT2 Entrez,  Source | signal transducer and activator of transcription 2, 113kDa | 14745 | -0.043 | -0.5971 | Yes |
| 8 | STAT1 | STAT1 Entrez,  Source | signal transducer and activator of transcription 1, 91kDa | 14882 | -0.045 | -0.5644 | Yes |
| 9 | JAK1 | JAK1 Entrez,  Source | Janus kinase 1 (a protein tyrosine kinase) | 15741 | -0.057 | -0.5574 | Yes |
| 10 | BLVRB | BLVRB Entrez,  Source | biliverdin reductase B (flavin reductase (NADPH)) | 15900 | -0.060 | -0.5119 | Yes |
| 11 | STAT5B | STAT5B Entrez,  Source | signal transducer and activator of transcription 5B | 16215 | -0.066 | -0.4694 | Yes |
| 12 | IL10RA | IL10RA Entrez,  Source | interleukin 10 receptor, alpha | 16917 | -0.082 | -0.4326 | Yes |
| 13 | STAT5A | STAT5A Entrez,  Source | signal transducer and activator of transcription 5A | 17665 | -0.103 | -0.3795 | Yes |
| 14 | BLVRA | BLVRA Entrez,  Source | biliverdin reductase A | 17763 | -0.106 | -0.2901 | Yes |
| 15 | IL1A | IL1A Entrez,  Source | interleukin 1, alpha | 17802 | -0.108 | -0.1963 | Yes |
| 16 | HMOX1 | HMOX1 Entrez,  Source | heme oxygenase (decycling) 1 | 18615 | -0.154 | -0.1012 | Yes |
| 17 | IL10RB | IL10RB Entrez,  Source | interleukin 10 receptor, beta | 18766 | -0.168 | 0.0404 | Yes |
Table: GSEA details [plain text format]

  

Fig 2: BIOCARTA\_IL10\_PATHWAY      
 Blue-Pink O' Gram in the Space of the Analyzed GeneSet

  

Fig 3: BIOCARTA\_IL10\_PATHWAY: Random ES distribution      
 Gene set null distribution of ES for **BIOCARTA\_IL10\_PATHWAY**

  
