# Supplementary material for: Comprehensive analysis of β-catenin target genes in colorectal carcinoma cell lines with deregulated Wnt/β-catenin signaling
Source: BMC Genomics. 2014 Jan 28;15:74. doi: 10.1186/1471-2164-15-74 (PMC3909937; doi:10.1186/1471-2164-15-74)
Supplement: Additional file 4 — GSEA analysis using the Biocarta pathway database. This zipped file contains confirming data of the GSEA analysis. The names of the directories containing the files were composed of the term ‘GSEA’, the name of the cell line, e.g. DLD1, SW480, or LS174T, and the pathway database (Biocarta). Please use a web browser to view the files with the name ‘index.html’ in the corresponding directories to start exploring the data. [file 1471-2164-15-74-S4.zip › DLD1_Biocarta/BIOCARTA_MCALPAIN_PATHWAY.html]

Details for gene set BIOCARTA\_MCALPAIN\_PATHWAY[GSEA]

|  || Dataset | DLD1\_collapsed\_to\_symbols.class.cls#bg\_versus\_b |
| Phenotype | class.cls#bg\_versus\_b |
| Upregulated in class | b |
| GeneSet | BIOCARTA\_MCALPAIN\_PATHWAY |
| Enrichment Score (ES) | -0.7057824 |
| Normalized Enrichment Score (NES) | -2.001039 |
| Nominal p-value | 0.0 |
| FDR q-value | 0.046758827 |
| FWER p-Value | 0.036 |
Table: GSEA Results Summary

  

Fig 1: Enrichment plot: BIOCARTA\_MCALPAIN\_PATHWAY      
 Profile of the Running ES Score & Positions of GeneSet Members on the Rank Ordered List

  

| PROBE | GENE SYMBOL | GENE\_TITLE | RANK IN GENE LIST | RANK METRIC SCORE | RUNNING ES | CORE ENRICHMENT || 1 | CAPNS2 | CAPNS2 Entrez,  Source | calpain, small subunit 2 | 2609 | 0.097 | -0.1007 | No |
| 2 | CXCR3 | CXCR3 Entrez,  Source | chemokine (C-X-C motif) receptor 3 | 5049 | 0.056 | -0.2067 | No |
| 3 | HRAS | HRAS Entrez,  Source | v-Ha-ras Harvey rat sarcoma viral oncogene homolog | 10612 | 0.001 | -0.4910 | No |
| 4 | TLN1 | TLN1 Entrez,  Source | talin 1 | 12261 | -0.014 | -0.5705 | No |
| 5 | PRKAR2A | PRKAR2A Entrez,  Source | protein kinase, cAMP-dependent, regulatory, type II, alpha | 12398 | -0.016 | -0.5722 | No |
| 6 | PRKACG | PRKACG Entrez,  Source | protein kinase, cAMP-dependent, catalytic, gamma | 13683 | -0.029 | -0.6281 | No |
| 7 | PRKAR1A | PRKAR1A Entrez,  Source | protein kinase, cAMP-dependent, regulatory, type I, alpha (tissue specific extinguisher 1) | 14125 | -0.034 | -0.6390 | No |
| 8 | ITGB1 | ITGB1 Entrez,  Source | integrin, beta 1 (fibronectin receptor, beta polypeptide, antigen CD29 includes MDF2, MSK12) | 14535 | -0.040 | -0.6466 | No |
| 9 | CAPN1 | CAPN1 Entrez,  Source | calpain 1, (mu/I) large subunit | 15693 | -0.057 | -0.6866 | Yes |
| 10 | MAPK1 | MAPK1 Entrez,  Source | mitogen-activated protein kinase 1 | 15755 | -0.058 | -0.6702 | Yes |
| 11 | PTK2 | PTK2 Entrez,  Source | PTK2 protein tyrosine kinase 2 | 16116 | -0.064 | -0.6669 | Yes |
| 12 | ACTA1 | ACTA1 Entrez,  Source | actin, alpha 1, skeletal muscle | 16287 | -0.067 | -0.6528 | Yes |
| 13 | EGFR | EGFR Entrez,  Source | epidermal growth factor receptor (erythroblastic leukemia viral (v-erb-b) oncogene homolog, avian) | 16395 | -0.070 | -0.6346 | Yes |
| 14 | CAPNS1 | CAPNS1 Entrez,  Source | calpain, small subunit 1 | 16736 | -0.077 | -0.6258 | Yes |
| 15 | PXN | PXN Entrez,  Source | paxillin | 17157 | -0.088 | -0.6176 | Yes |
| 16 | PRKACB | PRKACB Entrez,  Source | protein kinase, cAMP-dependent, catalytic, beta | 17727 | -0.105 | -0.6112 | Yes |
| 17 | MAPK3 | MAPK3 Entrez,  Source | mitogen-activated protein kinase 3 | 18647 | -0.156 | -0.6054 | Yes |
| 18 | MYL2 | MYL2 Entrez,  Source | myosin, light chain 2, regulatory, cardiac, slow | 18730 | -0.164 | -0.5541 | Yes |
| 19 | CAPN2 | CAPN2 Entrez,  Source | calpain 2, (m/II) large subunit | 19066 | -0.210 | -0.5002 | Yes |
| 20 | EGF | EGF Entrez,  Source | epidermal growth factor (beta-urogastrone) | 19379 | -0.340 | -0.4009 | Yes |
| 21 | MYLK | MYLK Entrez,  Source | myosin, light chain kinase | 19507 | -0.557 | -0.2189 | Yes |
| 22 | PRKAR2B | PRKAR2B Entrez,  Source | protein kinase, cAMP-dependent, regulatory, type II, beta | 19526 | -0.654 | 0.0015 | Yes |
Table: GSEA details [plain text format]

  

Fig 2: BIOCARTA\_MCALPAIN\_PATHWAY      
 Blue-Pink O' Gram in the Space of the Analyzed GeneSet

  

Fig 3: BIOCARTA\_MCALPAIN\_PATHWAY: Random ES distribution      
 Gene set null distribution of ES for **BIOCARTA\_MCALPAIN\_PATHWAY**

  
