# Supplementary material for: Comprehensive analysis of β-catenin target genes in colorectal carcinoma cell lines with deregulated Wnt/β-catenin signaling
Source: BMC Genomics. 2014 Jan 28;15:74. doi: 10.1186/1471-2164-15-74 (PMC3909937; doi:10.1186/1471-2164-15-74)
Supplement: Additional file 4 — GSEA analysis using the Biocarta pathway database. This zipped file contains confirming data of the GSEA analysis. The names of the directories containing the files were composed of the term ‘GSEA’, the name of the cell line, e.g. DLD1, SW480, or LS174T, and the pathway database (Biocarta). Please use a web browser to view the files with the name ‘index.html’ in the corresponding directories to start exploring the data. [file 1471-2164-15-74-S4.zip › DLD1_Biocarta/BIOCARTA_MET_PATHWAY.html]

Details for gene set BIOCARTA\_MET\_PATHWAY[GSEA]

|  || Dataset | DLD1\_collapsed\_to\_symbols.class.cls#bg\_versus\_b |
| Phenotype | class.cls#bg\_versus\_b |
| Upregulated in class | b |
| GeneSet | BIOCARTA\_MET\_PATHWAY |
| Enrichment Score (ES) | -0.46762082 |
| Normalized Enrichment Score (NES) | -1.4499559 |
| Nominal p-value | 0.07926829 |
| FDR q-value | 0.32239473 |
| FWER p-Value | 0.994 |
Table: GSEA Results Summary

  

Fig 1: Enrichment plot: BIOCARTA\_MET\_PATHWAY      
 Profile of the Running ES Score & Positions of GeneSet Members on the Rank Ordered List

  

| PROBE | GENE SYMBOL | GENE\_TITLE | RANK IN GENE LIST | RANK METRIC SCORE | RUNNING ES | CORE ENRICHMENT || 1 | JUN | JUN Entrez,  Source | jun oncogene | 2676 | 0.096 | -0.0875 | No |
| 2 | ELK1 | ELK1 Entrez,  Source | ELK1, member of ETS oncogene family | 3873 | 0.073 | -0.1109 | No |
| 3 | MAP4K1 | MAP4K1 Entrez,  Source | mitogen-activated protein kinase kinase kinase kinase 1 | 5113 | 0.055 | -0.1461 | No |
| 4 | MET | MET Entrez,  Source | met proto-oncogene (hepatocyte growth factor receptor) | 7103 | 0.033 | -0.2311 | No |
| 5 | CRKL | CRKL Entrez,  Source | v-crk sarcoma virus CT10 oncogene homolog (avian)-like | 7544 | 0.028 | -0.2390 | No |
| 6 | PIK3R1 | PIK3R1 Entrez,  Source | phosphoinositide-3-kinase, regulatory subunit 1 (p85 alpha) | 8011 | 0.024 | -0.2504 | No |
| 7 | PTPN11 | PTPN11 Entrez,  Source | protein tyrosine phosphatase, non-receptor type 11 (Noonan syndrome 1) | 8544 | 0.019 | -0.2677 | No |
| 8 | MAPK8 | MAPK8 Entrez,  Source | mitogen-activated protein kinase 8 | 9088 | 0.015 | -0.2878 | No |
| 9 | RASA1 | RASA1 Entrez,  Source | RAS p21 protein activator (GTPase activating protein) 1 | 10292 | 0.004 | -0.3473 | No |
| 10 | FOS | FOS Entrez,  Source | v-fos FBJ murine osteosarcoma viral oncogene homolog | 10374 | 0.003 | -0.3497 | No |
| 11 | HRAS | HRAS Entrez,  Source | v-Ha-ras Harvey rat sarcoma viral oncogene homolog | 10612 | 0.001 | -0.3611 | No |
| 12 | DOCK1 | DOCK1 Entrez,  Source | dedicator of cytokinesis 1 | 11949 | -0.011 | -0.4239 | No |
| 13 | PIK3CG | PIK3CG Entrez,  Source | phosphoinositide-3-kinase, catalytic, gamma polypeptide | 12525 | -0.017 | -0.4446 | No |
| 14 | HGF | HGF Entrez,  Source | hepatocyte growth factor (hepapoietin A; scatter factor) | 12976 | -0.022 | -0.4564 | Yes |
| 15 | MAP2K2 | MAP2K2 Entrez,  Source | mitogen-activated protein kinase kinase 2 | 12979 | -0.022 | -0.4452 | Yes |
| 16 | RAP1A | RAP1A Entrez,  Source | RAP1A, member of RAS oncogene family | 13062 | -0.023 | -0.4377 | Yes |
| 17 | MAP2K1 | MAP2K1 Entrez,  Source | mitogen-activated protein kinase kinase 1 | 13627 | -0.028 | -0.4519 | Yes |
| 18 | RAF1 | RAF1 Entrez,  Source | v-raf-1 murine leukemia viral oncogene homolog 1 | 13629 | -0.028 | -0.4373 | Yes |
| 19 | GRB2 | GRB2 Entrez,  Source | growth factor receptor-bound protein 2 | 13726 | -0.030 | -0.4270 | Yes |
| 20 | PTEN | PTEN Entrez,  Source | phosphatase and tensin homolog (mutated in multiple advanced cancers 1) | 14181 | -0.035 | -0.4321 | Yes |
| 21 | STAT3 | STAT3 Entrez,  Source | signal transducer and activator of transcription 3 (acute-phase response factor) | 14210 | -0.035 | -0.4153 | Yes |
| 22 | ITGB1 | ITGB1 Entrez,  Source | integrin, beta 1 (fibronectin receptor, beta polypeptide, antigen CD29 includes MDF2, MSK12) | 14535 | -0.040 | -0.4114 | Yes |
| 23 | SOS1 | SOS1 Entrez,  Source | son of sevenless homolog 1 (Drosophila) | 15485 | -0.053 | -0.4326 | Yes |
| 24 | SRC | SRC Entrez,  Source | v-src sarcoma (Schmidt-Ruppin A-2) viral oncogene homolog (avian) | 15682 | -0.056 | -0.4135 | Yes |
| 25 | MAPK1 | MAPK1 Entrez,  Source | mitogen-activated protein kinase 1 | 15755 | -0.058 | -0.3873 | Yes |
| 26 | PTK2 | PTK2 Entrez,  Source | PTK2 protein tyrosine kinase 2 | 16116 | -0.064 | -0.3726 | Yes |
| 27 | ACTA1 | ACTA1 Entrez,  Source | actin, alpha 1, skeletal muscle | 16287 | -0.067 | -0.3465 | Yes |
| 28 | PAK1 | PAK1 Entrez,  Source | p21/Cdc42/Rac1-activated kinase 1 (STE20 homolog, yeast) | 16291 | -0.067 | -0.3118 | Yes |
| 29 | CRK | CRK Entrez,  Source | v-crk sarcoma virus CT10 oncogene homolog (avian) | 16851 | -0.080 | -0.2992 | Yes |
| 30 | PXN | PXN Entrez,  Source | paxillin | 17157 | -0.088 | -0.2695 | Yes |
| 31 | PTK2B | PTK2B Entrez,  Source | PTK2B protein tyrosine kinase 2 beta | 17225 | -0.090 | -0.2266 | Yes |
| 32 | RAPGEF1 | RAPGEF1 Entrez,  Source | Rap guanine nucleotide exchange factor (GEF) 1 | 17321 | -0.092 | -0.1839 | Yes |
| 33 | PIK3CA | PIK3CA Entrez,  Source | phosphoinositide-3-kinase, catalytic, alpha polypeptide | 17601 | -0.101 | -0.1461 | Yes |
| 34 | MAPK3 | MAPK3 Entrez,  Source | mitogen-activated protein kinase 3 | 18647 | -0.156 | -0.1190 | Yes |
| 35 | GAB1 | GAB1 Entrez,  Source | GRB2-associated binding protein 1 | 19342 | -0.320 | 0.0110 | Yes |
Table: GSEA details [plain text format]

  

Fig 2: BIOCARTA\_MET\_PATHWAY      
 Blue-Pink O' Gram in the Space of the Analyzed GeneSet

  

Fig 3: BIOCARTA\_MET\_PATHWAY: Random ES distribution      
 Gene set null distribution of ES for **BIOCARTA\_MET\_PATHWAY**

  
