# Supplementary material for: Comprehensive analysis of β-catenin target genes in colorectal carcinoma cell lines with deregulated Wnt/β-catenin signaling
Source: BMC Genomics. 2014 Jan 28;15:74. doi: 10.1186/1471-2164-15-74 (PMC3909937; doi:10.1186/1471-2164-15-74)
Supplement: Additional file 4 — GSEA analysis using the Biocarta pathway database. This zipped file contains confirming data of the GSEA analysis. The names of the directories containing the files were composed of the term ‘GSEA’, the name of the cell line, e.g. DLD1, SW480, or LS174T, and the pathway database (Biocarta). Please use a web browser to view the files with the name ‘index.html’ in the corresponding directories to start exploring the data. [file 1471-2164-15-74-S4.zip › DLD1_Biocarta/BIOCARTA_MPR_PATHWAY.html]

Details for gene set BIOCARTA\_MPR\_PATHWAY[GSEA]

|  || Dataset | DLD1\_collapsed\_to\_symbols.class.cls#bg\_versus\_b |
| Phenotype | class.cls#bg\_versus\_b |
| Upregulated in class | b |
| GeneSet | BIOCARTA\_MPR\_PATHWAY |
| Enrichment Score (ES) | -0.58300173 |
| Normalized Enrichment Score (NES) | -1.807638 |
| Nominal p-value | 0.004310345 |
| FDR q-value | 0.120846644 |
| FWER p-Value | 0.311 |
Table: GSEA Results Summary

  

Fig 1: Enrichment plot: BIOCARTA\_MPR\_PATHWAY      
 Profile of the Running ES Score & Positions of GeneSet Members on the Rank Ordered List

  

| PROBE | GENE SYMBOL | GENE\_TITLE | RANK IN GENE LIST | RANK METRIC SCORE | RUNNING ES | CORE ENRICHMENT || 1 | MYT1 | MYT1 Entrez,  Source | myelin transcription factor 1 | 1005 | 0.155 | 0.0173 | No |
| 2 | GNAI1 | GNAI1 Entrez,  Source | guanine nucleotide binding protein (G protein), alpha inhibiting activity polypeptide 1 | 6049 | 0.044 | -0.2216 | No |
| 3 | CDC25C | CDC25C Entrez,  Source | cell division cycle 25C | 8918 | 0.016 | -0.3613 | No |
| 4 | CCNB1 | CCNB1 Entrez,  Source | cyclin B1 | 9414 | 0.012 | -0.3814 | No |
| 5 | ADCY1 | ADCY1 Entrez,  Source | adenylate cyclase 1 (brain) | 9619 | 0.010 | -0.3874 | No |
| 6 | HRAS | HRAS Entrez,  Source | v-Ha-ras Harvey rat sarcoma viral oncogene homolog | 10612 | 0.001 | -0.4377 | No |
| 7 | PAQR5 | PAQR5 Entrez,  Source | progestin and adipoQ receptor family member V | 11377 | -0.006 | -0.4743 | No |
| 8 | PGR | PGR Entrez,  Source | progesterone receptor | 11894 | -0.010 | -0.4961 | No |
| 9 | PRKAR2A | PRKAR2A Entrez,  Source | protein kinase, cAMP-dependent, regulatory, type II, alpha | 12398 | -0.016 | -0.5149 | No |
| 10 | PIN1 | PIN1 Entrez,  Source | protein (peptidylprolyl cis/trans isomerase) NIMA-interacting 1 | 13316 | -0.025 | -0.5506 | No |
| 11 | GNB1 | GNB1 Entrez,  Source | guanine nucleotide binding protein (G protein), beta polypeptide 1 | 13439 | -0.027 | -0.5451 | No |
| 12 | ACTR2 | ACTR2 Entrez,  Source | ARP2 actin-related protein 2 homolog (yeast) | 13514 | -0.027 | -0.5368 | No |
| 13 | ARPC2 | ARPC2 Entrez,  Source | actin related protein 2/3 complex, subunit 2, 34kDa | 13578 | -0.028 | -0.5276 | No |
| 14 | PRKACG | PRKACG Entrez,  Source | protein kinase, cAMP-dependent, catalytic, gamma | 13683 | -0.029 | -0.5200 | No |
| 15 | ACTR3 | ACTR3 Entrez,  Source | ARP3 actin-related protein 3 homolog (yeast) | 13954 | -0.032 | -0.5195 | No |
| 16 | PRKAR1A | PRKAR1A Entrez,  Source | protein kinase, cAMP-dependent, regulatory, type I, alpha (tissue specific extinguisher 1) | 14125 | -0.034 | -0.5129 | No |
| 17 | GNAS | GNAS Entrez,  Source | GNAS complex locus | 15494 | -0.053 | -0.5593 | Yes |
| 18 | SRC | SRC Entrez,  Source | v-src sarcoma (Schmidt-Ruppin A-2) viral oncogene homolog (avian) | 15682 | -0.056 | -0.5438 | Yes |
| 19 | CAP1 | CAP1 Entrez,  Source | CAP, adenylate cyclase-associated protein 1 (yeast) | 15748 | -0.058 | -0.5216 | Yes |
| 20 | MAPK1 | MAPK1 Entrez,  Source | mitogen-activated protein kinase 1 | 15755 | -0.058 | -0.4962 | Yes |
| 21 | ARPC5 | ARPC5 Entrez,  Source | actin related protein 2/3 complex, subunit 5, 16kDa | 15899 | -0.060 | -0.4768 | Yes |
| 22 | ACTA1 | ACTA1 Entrez,  Source | actin, alpha 1, skeletal muscle | 16287 | -0.067 | -0.4667 | Yes |
| 23 | ARPC3 | ARPC3 Entrez,  Source | actin related protein 2/3 complex, subunit 3, 21kDa | 16471 | -0.072 | -0.4443 | Yes |
| 24 | GNGT1 | GNGT1 Entrez,  Source | guanine nucleotide binding protein (G protein), gamma transducing activity polypeptide 1 | 16603 | -0.074 | -0.4180 | Yes |
| 25 | ARPC4 | ARPC4 Entrez,  Source | actin related protein 2/3 complex, subunit 4, 20kDa | 17054 | -0.085 | -0.4033 | Yes |
| 26 | PAQR7 | PAQR7 Entrez,  Source | progestin and adipoQ receptor family member VII | 17209 | -0.089 | -0.3716 | Yes |
| 27 | ARPC1A | ARPC1A Entrez,  Source | actin related protein 2/3 complex, subunit 1A, 41kDa | 17416 | -0.095 | -0.3401 | Yes |
| 28 | RPS6KA1 | RPS6KA1 Entrez,  Source | ribosomal protein S6 kinase, 90kDa, polypeptide 1 | 17464 | -0.096 | -0.2996 | Yes |
| 29 | PRKACB | PRKACB Entrez,  Source | protein kinase, cAMP-dependent, catalytic, beta | 17727 | -0.105 | -0.2664 | Yes |
| 30 | MAPK3 | MAPK3 Entrez,  Source | mitogen-activated protein kinase 3 | 18647 | -0.156 | -0.2441 | Yes |
| 31 | PRKAR2B | PRKAR2B Entrez,  Source | protein kinase, cAMP-dependent, regulatory, type II, beta | 19526 | -0.654 | 0.0015 | Yes |
Table: GSEA details [plain text format]

  

Fig 2: BIOCARTA\_MPR\_PATHWAY      
 Blue-Pink O' Gram in the Space of the Analyzed GeneSet

  

Fig 3: BIOCARTA\_MPR\_PATHWAY: Random ES distribution      
 Gene set null distribution of ES for **BIOCARTA\_MPR\_PATHWAY**

  
