# Supplementary material for: Comprehensive analysis of β-catenin target genes in colorectal carcinoma cell lines with deregulated Wnt/β-catenin signaling
Source: BMC Genomics. 2014 Jan 28;15:74. doi: 10.1186/1471-2164-15-74 (PMC3909937; doi:10.1186/1471-2164-15-74)
Supplement: Additional file 4 — GSEA analysis using the Biocarta pathway database. This zipped file contains confirming data of the GSEA analysis. The names of the directories containing the files were composed of the term ‘GSEA’, the name of the cell line, e.g. DLD1, SW480, or LS174T, and the pathway database (Biocarta). Please use a web browser to view the files with the name ‘index.html’ in the corresponding directories to start exploring the data. [file 1471-2164-15-74-S4.zip › DLD1_Biocarta/BIOCARTA_NOS1_PATHWAY.html]

Details for gene set BIOCARTA\_NOS1\_PATHWAY[GSEA]

|  || Dataset | DLD1\_collapsed\_to\_symbols.class.cls#bg\_versus\_b |
| Phenotype | class.cls#bg\_versus\_b |
| Upregulated in class | b |
| GeneSet | BIOCARTA\_NOS1\_PATHWAY |
| Enrichment Score (ES) | -0.5477985 |
| Normalized Enrichment Score (NES) | -1.4567802 |
| Nominal p-value | 0.072463766 |
| FDR q-value | 0.32755095 |
| FWER p-Value | 0.99 |
Table: GSEA Results Summary

  

Fig 1: Enrichment plot: BIOCARTA\_NOS1\_PATHWAY      
 Profile of the Running ES Score & Positions of GeneSet Members on the Rank Ordered List

  

| PROBE | GENE SYMBOL | GENE\_TITLE | RANK IN GENE LIST | RANK METRIC SCORE | RUNNING ES | CORE ENRICHMENT || 1 | GRIN2D | GRIN2D Entrez,  Source | glutamate receptor, ionotropic, N-methyl D-aspartate 2D | 2504 | 0.100 | -0.0587 | No |
| 2 | GRIN2B | GRIN2B Entrez,  Source | glutamate receptor, ionotropic, N-methyl D-aspartate 2B | 3355 | 0.082 | -0.0447 | No |
| 3 | PPP3CC | PPP3CC Entrez,  Source | protein phosphatase 3 (formerly 2B), catalytic subunit, gamma isoform (calcineurin A gamma) | 6968 | 0.034 | -0.2059 | No |
| 4 | GRIN1 | GRIN1 Entrez,  Source | glutamate receptor, ionotropic, N-methyl D-aspartate 1 | 9796 | 0.009 | -0.3447 | No |
| 5 | CALM1 | CALM1 Entrez,  Source | calmodulin 1 (phosphorylase kinase, delta) | 10081 | 0.006 | -0.3551 | No |
| 6 | PPP3CB | PPP3CB Entrez,  Source | protein phosphatase 3 (formerly 2B), catalytic subunit, beta isoform (calcineurin A beta) | 10615 | 0.001 | -0.3814 | No |
| 7 | GRIN2A | GRIN2A Entrez,  Source | glutamate receptor, ionotropic, N-methyl D-aspartate 2A | 11110 | -0.003 | -0.4046 | No |
| 8 | DLG4 | DLG4 Entrez,  Source | discs, large homolog 4 (Drosophila) | 11543 | -0.007 | -0.4218 | No |
| 9 | PRKAR2A | PRKAR2A Entrez,  Source | protein kinase, cAMP-dependent, regulatory, type II, alpha | 12398 | -0.016 | -0.4546 | No |
| 10 | NOS1 | NOS1 Entrez,  Source | nitric oxide synthase 1 (neuronal) | 12897 | -0.021 | -0.4655 | No |
| 11 | PRKACG | PRKACG Entrez,  Source | protein kinase, cAMP-dependent, catalytic, gamma | 13683 | -0.029 | -0.4853 | No |
| 12 | CALM2 | CALM2 Entrez,  Source | calmodulin 2 (phosphorylase kinase, delta) | 14035 | -0.033 | -0.4801 | No |
| 13 | PRKAR1A | PRKAR1A Entrez,  Source | protein kinase, cAMP-dependent, regulatory, type I, alpha (tissue specific extinguisher 1) | 14125 | -0.034 | -0.4607 | No |
| 14 | PRKCA | PRKCA Entrez,  Source | protein kinase C, alpha | 15308 | -0.051 | -0.4858 | Yes |
| 15 | PPP3CA | PPP3CA Entrez,  Source | protein phosphatase 3 (formerly 2B), catalytic subunit, alpha isoform (calcineurin A alpha) | 15858 | -0.060 | -0.4724 | Yes |
| 16 | CALM3 | CALM3 Entrez,  Source | calmodulin 3 (phosphorylase kinase, delta) | 17333 | -0.092 | -0.4833 | Yes |
| 17 | GRIN2C | GRIN2C Entrez,  Source | glutamate receptor, ionotropic, N-methyl D-aspartate 2C | 17456 | -0.096 | -0.4224 | Yes |
| 18 | PRKACB | PRKACB Entrez,  Source | protein kinase, cAMP-dependent, catalytic, beta | 17727 | -0.105 | -0.3629 | Yes |
| 19 | PRKAR2B | PRKAR2B Entrez,  Source | protein kinase, cAMP-dependent, regulatory, type II, beta | 19526 | -0.654 | 0.0015 | Yes |
Table: GSEA details [plain text format]

  

Fig 2: BIOCARTA\_NOS1\_PATHWAY      
 Blue-Pink O' Gram in the Space of the Analyzed GeneSet

  

Fig 3: BIOCARTA\_NOS1\_PATHWAY: Random ES distribution      
 Gene set null distribution of ES for **BIOCARTA\_NOS1\_PATHWAY**

  
