# Supplementary material for: Comprehensive analysis of β-catenin target genes in colorectal carcinoma cell lines with deregulated Wnt/β-catenin signaling
Source: BMC Genomics. 2014 Jan 28;15:74. doi: 10.1186/1471-2164-15-74 (PMC3909937; doi:10.1186/1471-2164-15-74)
Supplement: Additional file 4 — GSEA analysis using the Biocarta pathway database. This zipped file contains confirming data of the GSEA analysis. The names of the directories containing the files were composed of the term ‘GSEA’, the name of the cell line, e.g. DLD1, SW480, or LS174T, and the pathway database (Biocarta). Please use a web browser to view the files with the name ‘index.html’ in the corresponding directories to start exploring the data. [file 1471-2164-15-74-S4.zip › DLD1_Biocarta/BIOCARTA_NTHI_PATHWAY.html]

Details for gene set BIOCARTA\_NTHI\_PATHWAY[GSEA]

|  || Dataset | DLD1\_collapsed\_to\_symbols.class.cls#bg\_versus\_b |
| Phenotype | class.cls#bg\_versus\_b |
| Upregulated in class | bg |
| GeneSet | BIOCARTA\_NTHI\_PATHWAY |
| Enrichment Score (ES) | 0.4170794 |
| Normalized Enrichment Score (NES) | 1.1922178 |
| Nominal p-value | 0.2215909 |
| FDR q-value | 0.6861586 |
| FWER p-Value | 1.0 |
Table: GSEA Results Summary

  

Fig 1: Enrichment plot: BIOCARTA\_NTHI\_PATHWAY      
 Profile of the Running ES Score & Positions of GeneSet Members on the Rank Ordered List

  

| PROBE | GENE SYMBOL | GENE\_TITLE | RANK IN GENE LIST | RANK METRIC SCORE | RUNNING ES | CORE ENRICHMENT || 1 | IL8 | IL8 Entrez,  Source | interleukin 8 | 76 | 0.373 | 0.2341 | Yes |
| 2 | MAP2K6 | MAP2K6 Entrez,  Source | mitogen-activated protein kinase kinase 6 | 171 | 0.288 | 0.4129 | Yes |
| 3 | TLR2 | TLR2 Entrez,  Source | toll-like receptor 2 | 2368 | 0.103 | 0.3660 | Yes |
| 4 | TGFBR2 | TGFBR2 Entrez,  Source | transforming growth factor, beta receptor II (70/80kDa) | 2589 | 0.098 | 0.4171 | Yes |
| 5 | EP300 | EP300 Entrez,  Source | E1A binding protein p300 | 7847 | 0.026 | 0.1643 | No |
| 6 | NFKB1 | NFKB1 Entrez,  Source | nuclear factor of kappa light polypeptide gene enhancer in B-cells 1 (p105) | 8386 | 0.021 | 0.1498 | No |
| 7 | TGFBR1 | TGFBR1 Entrez,  Source | transforming growth factor, beta receptor I (activin A receptor type II-like kinase, 53kDa) | 8576 | 0.019 | 0.1524 | No |
| 8 | CREBBP | CREBBP Entrez,  Source | CREB binding protein (Rubinstein-Taybi syndrome) | 9573 | 0.010 | 0.1080 | No |
| 9 | MAP3K14 | MAP3K14 Entrez,  Source | mitogen-activated protein kinase kinase kinase 14 | 10379 | 0.003 | 0.0690 | No |
| 10 | IL1B | IL1B Entrez,  Source | interleukin 1, beta | 10746 | 0.000 | 0.0504 | No |
| 11 | NR3C1 | NR3C1 Entrez,  Source | nuclear receptor subfamily 3, group C, member 1 (glucocorticoid receptor) | 11013 | -0.002 | 0.0383 | No |
| 12 | RELA | RELA Entrez,  Source | v-rel reticuloendotheliosis viral oncogene homolog A, nuclear factor of kappa light polypeptide gene enhancer in B-cells 3, p65 (avian) | 11371 | -0.006 | 0.0235 | No |
| 13 | MAPK14 | MAPK14 Entrez,  Source | mitogen-activated protein kinase 14 | 11391 | -0.006 | 0.0262 | No |
| 14 | TNF | TNF Entrez,  Source | tumor necrosis factor (TNF superfamily, member 2) | 11808 | -0.010 | 0.0110 | No |
| 15 | MAPK11 | MAPK11 Entrez,  Source | mitogen-activated protein kinase 11 | 13146 | -0.023 | -0.0425 | No |
| 16 | MAP3K7 | MAP3K7 Entrez,  Source | mitogen-activated protein kinase kinase kinase 7 | 14015 | -0.033 | -0.0659 | No |
| 17 | SMAD4 | SMAD4 Entrez,  Source | SMAD, mothers against DPP homolog 4 (Drosophila) | 14222 | -0.036 | -0.0538 | No |
| 18 | IKBKB | IKBKB Entrez,  Source | inhibitor of kappa light polypeptide gene enhancer in B-cells, kinase beta | 15118 | -0.048 | -0.0690 | No |
| 19 | CHUK | CHUK Entrez,  Source | conserved helix-loop-helix ubiquitous kinase | 15639 | -0.056 | -0.0600 | No |
| 20 | DUSP1 | DUSP1 Entrez,  Source | dual specificity phosphatase 1 | 17085 | -0.086 | -0.0792 | No |
| 21 | MYD88 | MYD88 Entrez,  Source | myeloid differentiation primary response gene (88) | 17695 | -0.104 | -0.0441 | No |
| 22 | NFKBIA | NFKBIA Entrez,  Source | nuclear factor of kappa light polypeptide gene enhancer in B-cells inhibitor, alpha | 17726 | -0.105 | 0.0214 | No |
| 23 | SMAD3 | SMAD3 Entrez,  Source | SMAD, mothers against DPP homolog 3 (Drosophila) | 17945 | -0.113 | 0.0825 | No |
Table: GSEA details [plain text format]

  

Fig 2: BIOCARTA\_NTHI\_PATHWAY      
 Blue-Pink O' Gram in the Space of the Analyzed GeneSet

  

Fig 3: BIOCARTA\_NTHI\_PATHWAY: Random ES distribution      
 Gene set null distribution of ES for **BIOCARTA\_NTHI\_PATHWAY**

  
