# Supplementary material for: Comprehensive analysis of β-catenin target genes in colorectal carcinoma cell lines with deregulated Wnt/β-catenin signaling
Source: BMC Genomics. 2014 Jan 28;15:74. doi: 10.1186/1471-2164-15-74 (PMC3909937; doi:10.1186/1471-2164-15-74)
Supplement: Additional file 4 — GSEA analysis using the Biocarta pathway database. This zipped file contains confirming data of the GSEA analysis. The names of the directories containing the files were composed of the term ‘GSEA’, the name of the cell line, e.g. DLD1, SW480, or LS174T, and the pathway database (Biocarta). Please use a web browser to view the files with the name ‘index.html’ in the corresponding directories to start exploring the data. [file 1471-2164-15-74-S4.zip › DLD1_Biocarta/BIOCARTA_P38MAPK_PATHWAY.html]

Details for gene set BIOCARTA\_P38MAPK\_PATHWAY[GSEA]

|  || Dataset | DLD1\_collapsed\_to\_symbols.class.cls#bg\_versus\_b |
| Phenotype | class.cls#bg\_versus\_b |
| Upregulated in class | bg |
| GeneSet | BIOCARTA\_P38MAPK\_PATHWAY |
| Enrichment Score (ES) | 0.4092563 |
| Normalized Enrichment Score (NES) | 1.3157235 |
| Nominal p-value | 0.08565737 |
| FDR q-value | 0.52764386 |
| FWER p-Value | 1.0 |
Table: GSEA Results Summary

  

Fig 1: Enrichment plot: BIOCARTA\_P38MAPK\_PATHWAY      
 Profile of the Running ES Score & Positions of GeneSet Members on the Rank Ordered List

  

| PROBE | GENE SYMBOL | GENE\_TITLE | RANK IN GENE LIST | RANK METRIC SCORE | RUNNING ES | CORE ENRICHMENT || 1 | PLA2G4A | PLA2G4A Entrez,  Source | phospholipase A2, group IVA (cytosolic, calcium-dependent) | 19 | 0.505 | 0.1489 | Yes |
| 2 | MYC | MYC Entrez,  Source | v-myc myelocytomatosis viral oncogene homolog (avian) | 109 | 0.326 | 0.2412 | Yes |
| 3 | TGFB2 | TGFB2 Entrez,  Source | transforming growth factor, beta 2 | 165 | 0.290 | 0.3242 | Yes |
| 4 | MAP2K6 | MAP2K6 Entrez,  Source | mitogen-activated protein kinase kinase 6 | 171 | 0.288 | 0.4093 | Yes |
| 5 | TGFB1 | TGFB1 Entrez,  Source | transforming growth factor, beta 1 (Camurati-Engelmann disease) | 1587 | 0.126 | 0.3743 | No |
| 6 | ELK1 | ELK1 Entrez,  Source | ELK1, member of ETS oncogene family | 3873 | 0.073 | 0.2789 | No |
| 7 | TGFB3 | TGFB3 Entrez,  Source | transforming growth factor, beta 3 | 4247 | 0.067 | 0.2797 | No |
| 8 | MAP3K1 | MAP3K1 Entrez,  Source | mitogen-activated protein kinase kinase kinase 1 | 4699 | 0.061 | 0.2746 | No |
| 9 | DAXX | DAXX Entrez,  Source | death-associated protein 6 | 5201 | 0.054 | 0.2649 | No |
| 10 | DDIT3 | DDIT3 Entrez,  Source | DNA-damage-inducible transcript 3 | 5270 | 0.052 | 0.2770 | No |
| 11 | HSPB2 | HSPB2 Entrez,  Source | heat shock 27kDa protein 2 | 5347 | 0.052 | 0.2884 | No |
| 12 | TRAF2 | TRAF2 Entrez,  Source | TNF receptor-associated factor 2 | 6319 | 0.041 | 0.2507 | No |
| 13 | RPS6KA5 | RPS6KA5 Entrez,  Source | ribosomal protein S6 kinase, 90kDa, polypeptide 5 | 6426 | 0.040 | 0.2570 | No |
| 14 | TGFBR1 | TGFBR1 Entrez,  Source | transforming growth factor, beta receptor I (activin A receptor type II-like kinase, 53kDa) | 8576 | 0.019 | 0.1526 | No |
| 15 | HMGN1 | HMGN1 Entrez,  Source | high-mobility group nucleosome binding domain 1 | 9540 | 0.011 | 0.1064 | No |
| 16 | MAPKAPK5 | MAPKAPK5 Entrez,  Source | mitogen-activated protein kinase-activated protein kinase 5 | 10557 | 0.002 | 0.0549 | No |
| 17 | MAPKAPK2 | MAPKAPK2 Entrez,  Source | mitogen-activated protein kinase-activated protein kinase 2 | 10581 | 0.002 | 0.0542 | No |
| 18 | HRAS | HRAS Entrez,  Source | v-Ha-ras Harvey rat sarcoma viral oncogene homolog | 10612 | 0.001 | 0.0530 | No |
| 19 | MAPK14 | MAPK14 Entrez,  Source | mitogen-activated protein kinase 14 | 11391 | -0.006 | 0.0149 | No |
| 20 | SHC1 | SHC1 Entrez,  Source | SHC (Src homology 2 domain containing) transforming protein 1 | 12661 | -0.018 | -0.0447 | No |
| 21 | GRB2 | GRB2 Entrez,  Source | growth factor receptor-bound protein 2 | 13726 | -0.030 | -0.0905 | No |
| 22 | MAP3K7 | MAP3K7 Entrez,  Source | mitogen-activated protein kinase kinase kinase 7 | 14015 | -0.033 | -0.0955 | No |
| 23 | MAP2K4 | MAP2K4 Entrez,  Source | mitogen-activated protein kinase kinase 4 | 14064 | -0.034 | -0.0880 | No |
| 24 | MAX | MAX Entrez,  Source | MYC associated factor X | 14120 | -0.034 | -0.0807 | No |
| 25 | STAT1 | STAT1 Entrez,  Source | signal transducer and activator of transcription 1, 91kDa | 14882 | -0.045 | -0.1064 | No |
| 26 | RIPK1 | RIPK1 Entrez,  Source | receptor (TNFRSF)-interacting serine-threonine kinase 1 | 14971 | -0.046 | -0.0974 | No |
| 27 | RAC1 | RAC1 Entrez,  Source | ras-related C3 botulinum toxin substrate 1 (rho family, small GTP binding protein Rac1) | 14979 | -0.046 | -0.0841 | No |
| 28 | CREB1 | CREB1 Entrez,  Source | cAMP responsive element binding protein 1 | 15936 | -0.061 | -0.1151 | No |
| 29 | MEF2A | MEF2A Entrez,  Source | MADS box transcription enhancer factor 2, polypeptide A (myocyte enhancer factor 2A) | 16103 | -0.064 | -0.1046 | No |
| 30 | MAP3K9 | MAP3K9 Entrez,  Source | mitogen-activated protein kinase kinase kinase 9 | 16260 | -0.067 | -0.0928 | No |
| 31 | RAPGEF2 | RAPGEF2 Entrez,  Source | Rap guanine nucleotide exchange factor (GEF) 2 | 16355 | -0.069 | -0.0771 | No |
| 32 | TRADD | TRADD Entrez,  Source | TNFRSF1A-associated via death domain | 16595 | -0.074 | -0.0674 | No |
| 33 | MKNK1 | MKNK1 Entrez,  Source | MAP kinase interacting serine/threonine kinase 1 | 16649 | -0.075 | -0.0478 | No |
| 34 | MEF2D | MEF2D Entrez,  Source | MADS box transcription enhancer factor 2, polypeptide D (myocyte enhancer factor 2D) | 16891 | -0.081 | -0.0362 | No |
| 35 | CDC42 | CDC42 Entrez,  Source | cell division cycle 42 (GTP binding protein, 25kDa) | 17370 | -0.093 | -0.0329 | No |
| 36 | ATF2 | ATF2 Entrez,  Source | activating transcription factor 2 | 17497 | -0.098 | -0.0105 | No |
| 37 | HSPB1 | HSPB1 Entrez,  Source | heat shock 27kDa protein 1 | 17671 | -0.103 | 0.0112 | No |
| 38 | MEF2C | MEF2C Entrez,  Source | MADS box transcription enhancer factor 2, polypeptide C (myocyte enhancer factor 2C) | 17808 | -0.108 | 0.0362 | No |
| 39 | MAP3K5 | MAP3K5 Entrez,  Source | mitogen-activated protein kinase kinase kinase 5 | 18885 | -0.180 | 0.0344 | No |
Table: GSEA details [plain text format]

  

Fig 2: BIOCARTA\_P38MAPK\_PATHWAY      
 Blue-Pink O' Gram in the Space of the Analyzed GeneSet

  

Fig 3: BIOCARTA\_P38MAPK\_PATHWAY: Random ES distribution      
 Gene set null distribution of ES for **BIOCARTA\_P38MAPK\_PATHWAY**

  
