# Supplementary material for: Comprehensive analysis of β-catenin target genes in colorectal carcinoma cell lines with deregulated Wnt/β-catenin signaling
Source: BMC Genomics. 2014 Jan 28;15:74. doi: 10.1186/1471-2164-15-74 (PMC3909937; doi:10.1186/1471-2164-15-74)
Supplement: Additional file 4 — GSEA analysis using the Biocarta pathway database. This zipped file contains confirming data of the GSEA analysis. The names of the directories containing the files were composed of the term ‘GSEA’, the name of the cell line, e.g. DLD1, SW480, or LS174T, and the pathway database (Biocarta). Please use a web browser to view the files with the name ‘index.html’ in the corresponding directories to start exploring the data. [file 1471-2164-15-74-S4.zip › DLD1_Biocarta/BIOCARTA_P53HYPOXIA_PATHWAY.html]

Details for gene set BIOCARTA\_P53HYPOXIA\_PATHWAY[GSEA]

|  || Dataset | DLD1\_collapsed\_to\_symbols.class.cls#bg\_versus\_b |
| Phenotype | class.cls#bg\_versus\_b |
| Upregulated in class | bg |
| GeneSet | BIOCARTA\_P53HYPOXIA\_PATHWAY |
| Enrichment Score (ES) | 0.4152237 |
| Normalized Enrichment Score (NES) | 1.1804098 |
| Nominal p-value | 0.243083 |
| FDR q-value | 0.68088555 |
| FWER p-Value | 1.0 |
Table: GSEA Results Summary

  

Fig 1: Enrichment plot: BIOCARTA\_P53HYPOXIA\_PATHWAY      
 Profile of the Running ES Score & Positions of GeneSet Members on the Rank Ordered List

  

| PROBE | GENE SYMBOL | GENE\_TITLE | RANK IN GENE LIST | RANK METRIC SCORE | RUNNING ES | CORE ENRICHMENT || 1 | ABCB1 | ABCB1 Entrez,  Source | ATP-binding cassette, sub-family B (MDR/TAP), member 1 | 40 | 0.422 | 0.2347 | Yes |
| 2 | IGFBP3 | IGFBP3 Entrez,  Source | insulin-like growth factor binding protein 3 | 309 | 0.235 | 0.3527 | Yes |
| 3 | TP53 | TP53 Entrez,  Source | tumor protein p53 (Li-Fraumeni syndrome) | 877 | 0.163 | 0.4152 | Yes |
| 4 | NFKBIB | NFKBIB Entrez,  Source | nuclear factor of kappa light polypeptide gene enhancer in B-cells inhibitor, beta | 5218 | 0.053 | 0.2230 | No |
| 5 | RPA1 | RPA1 Entrez,  Source | replication protein A1, 70kDa | 5886 | 0.045 | 0.2143 | No |
| 6 | ATM | ATM Entrez,  Source | ataxia telangiectasia mutated (includes complementation groups A, C and D) | 7603 | 0.028 | 0.1420 | No |
| 7 | MDM2 | MDM2 Entrez,  Source | Mdm2, transformed 3T3 cell double minute 2, p53 binding protein (mouse) | 7751 | 0.026 | 0.1493 | No |
| 8 | EP300 | EP300 Entrez,  Source | E1A binding protein p300 | 7847 | 0.026 | 0.1588 | No |
| 9 | HIC1 | HIC1 Entrez,  Source | hypermethylated in cancer 1 | 8341 | 0.021 | 0.1453 | No |
| 10 | TAF1 | TAF1 Entrez,  Source | TAF1 RNA polymerase II, TATA box binding protein (TBP)-associated factor, 250kDa | 8969 | 0.016 | 0.1220 | No |
| 11 | MAPK8 | MAPK8 Entrez,  Source | mitogen-activated protein kinase 8 | 9088 | 0.015 | 0.1243 | No |
| 12 | BAX | BAX Entrez,  Source | BCL2-associated X protein | 9096 | 0.015 | 0.1323 | No |
| 13 | HSP90AA1 | HSP90AA1 Entrez,  Source | heat shock protein 90kDa alpha (cytosolic), class A member 1 | 10425 | 0.003 | 0.0659 | No |
| 14 | GADD45A | GADD45A Entrez,  Source | growth arrest and DNA-damage-inducible, alpha | 10698 | 0.001 | 0.0524 | No |
| 15 | HIF1A | HIF1A Entrez,  Source | hypoxia-inducible factor 1, alpha subunit (basic helix-loop-helix transcription factor) | 11503 | -0.007 | 0.0149 | No |
| 16 | AKT1 | AKT1 Entrez,  Source | v-akt murine thymoma viral oncogene homolog 1 | 11660 | -0.008 | 0.0115 | No |
| 17 | CSNK1D | CSNK1D Entrez,  Source | casein kinase 1, delta | 14608 | -0.041 | -0.1166 | No |
| 18 | CSNK1A1 | CSNK1A1 Entrez,  Source | casein kinase 1, alpha 1 | 14829 | -0.044 | -0.1032 | No |
| 19 | NQO1 | NQO1 Entrez,  Source | NAD(P)H dehydrogenase, quinone 1 | 16101 | -0.064 | -0.1325 | No |
| 20 | CDKN1A | CDKN1A Entrez,  Source | cyclin-dependent kinase inhibitor 1A (p21, Cip1) | 17512 | -0.098 | -0.1497 | No |
| 21 | FHL2 | FHL2 Entrez,  Source | four and a half LIM domains 2 | 18309 | -0.131 | -0.1169 | No |
| 22 | HSPA1A | HSPA1A Entrez,  Source | heat shock 70kDa protein 1A | 19344 | -0.322 | 0.0109 | No |
Table: GSEA details [plain text format]

  

Fig 2: BIOCARTA\_P53HYPOXIA\_PATHWAY      
 Blue-Pink O' Gram in the Space of the Analyzed GeneSet

  

Fig 3: BIOCARTA\_P53HYPOXIA\_PATHWAY: Random ES distribution      
 Gene set null distribution of ES for **BIOCARTA\_P53HYPOXIA\_PATHWAY**

  
