# Supplementary material for: Comprehensive analysis of β-catenin target genes in colorectal carcinoma cell lines with deregulated Wnt/β-catenin signaling
Source: BMC Genomics. 2014 Jan 28;15:74. doi: 10.1186/1471-2164-15-74 (PMC3909937; doi:10.1186/1471-2164-15-74)
Supplement: Additional file 4 — GSEA analysis using the Biocarta pathway database. This zipped file contains confirming data of the GSEA analysis. The names of the directories containing the files were composed of the term ‘GSEA’, the name of the cell line, e.g. DLD1, SW480, or LS174T, and the pathway database (Biocarta). Please use a web browser to view the files with the name ‘index.html’ in the corresponding directories to start exploring the data. [file 1471-2164-15-74-S4.zip › DLD1_Biocarta/BIOCARTA_PML_PATHWAY.html]

Details for gene set BIOCARTA\_PML\_PATHWAY[GSEA]

|  || Dataset | DLD1\_collapsed\_to\_symbols.class.cls#bg\_versus\_b |
| Phenotype | class.cls#bg\_versus\_b |
| Upregulated in class | bg |
| GeneSet | BIOCARTA\_PML\_PATHWAY |
| Enrichment Score (ES) | 0.5454654 |
| Normalized Enrichment Score (NES) | 1.441074 |
| Nominal p-value | 0.067736186 |
| FDR q-value | 0.54890925 |
| FWER p-Value | 0.99 |
Table: GSEA Results Summary

  

Fig 1: Enrichment plot: BIOCARTA\_PML\_PATHWAY      
 Profile of the Running ES Score & Positions of GeneSet Members on the Rank Ordered List

  

| PROBE | GENE SYMBOL | GENE\_TITLE | RANK IN GENE LIST | RANK METRIC SCORE | RUNNING ES | CORE ENRICHMENT || 1 | TNFRSF1B | TNFRSF1B Entrez,  Source | tumor necrosis factor receptor superfamily, member 1B | 198 | 0.277 | 0.1913 | Yes |
| 2 | FASLG | FASLG Entrez,  Source | Fas ligand (TNF superfamily, member 6) | 315 | 0.234 | 0.3549 | Yes |
| 3 | TP53 | TP53 Entrez,  Source | tumor protein p53 (Li-Fraumeni syndrome) | 877 | 0.163 | 0.4447 | Yes |
| 4 | FAS | FAS Entrez,  Source | Fas (TNF receptor superfamily, member 6) | 1068 | 0.151 | 0.5444 | Yes |
| 5 | PAX3 | PAX3 Entrez,  Source | paired box gene 3 (Waardenburg syndrome 1) | 2470 | 0.100 | 0.5455 | Yes |
| 6 | DAXX | DAXX Entrez,  Source | death-associated protein 6 | 5201 | 0.054 | 0.4447 | No |
| 7 | RARA | RARA Entrez,  Source | retinoic acid receptor, alpha | 5559 | 0.049 | 0.4621 | No |
| 8 | PML | PML Entrez,  Source | promyelocytic leukemia | 8267 | 0.022 | 0.3393 | No |
| 9 | SUMO1 | SUMO1 Entrez,  Source | SMT3 suppressor of mif two 3 homolog 1 (S. cerevisiae) | 8592 | 0.019 | 0.3365 | No |
| 10 | SIRT1 | SIRT1 Entrez,  Source | sirtuin (silent mating type information regulation 2 homolog) 1 (S. cerevisiae) | 9264 | 0.013 | 0.3117 | No |
| 11 | CREBBP | CREBBP Entrez,  Source | CREB binding protein (Rubinstein-Taybi syndrome) | 9573 | 0.010 | 0.3035 | No |
| 12 | HRAS | HRAS Entrez,  Source | v-Ha-ras Harvey rat sarcoma viral oncogene homolog | 10612 | 0.001 | 0.2513 | No |
| 13 | RB1 | RB1 Entrez,  Source | retinoblastoma 1 (including osteosarcoma) | 11565 | -0.007 | 0.2078 | No |
| 14 | TNF | TNF Entrez,  Source | tumor necrosis factor (TNF superfamily, member 2) | 11808 | -0.010 | 0.2024 | No |
| 15 | PRAM1 | PRAM1 Entrez,  Source | PML-RARA regulated adaptor molecule 1 | 15492 | -0.053 | 0.0526 | No |
| 16 | SP100 | SP100 Entrez,  Source | SP100 nuclear antigen | 17422 | -0.095 | 0.0227 | No |
| 17 | TNFRSF1A | TNFRSF1A Entrez,  Source | tumor necrosis factor receptor superfamily, member 1A | 18084 | -0.119 | 0.0753 | No |
Table: GSEA details [plain text format]

  

Fig 2: BIOCARTA\_PML\_PATHWAY      
 Blue-Pink O' Gram in the Space of the Analyzed GeneSet

  

Fig 3: BIOCARTA\_PML\_PATHWAY: Random ES distribution      
 Gene set null distribution of ES for **BIOCARTA\_PML\_PATHWAY**

  
