# Supplementary material for: Comprehensive analysis of β-catenin target genes in colorectal carcinoma cell lines with deregulated Wnt/β-catenin signaling
Source: BMC Genomics. 2014 Jan 28;15:74. doi: 10.1186/1471-2164-15-74 (PMC3909937; doi:10.1186/1471-2164-15-74)
Supplement: Additional file 4 — GSEA analysis using the Biocarta pathway database. This zipped file contains confirming data of the GSEA analysis. The names of the directories containing the files were composed of the term ‘GSEA’, the name of the cell line, e.g. DLD1, SW480, or LS174T, and the pathway database (Biocarta). Please use a web browser to view the files with the name ‘index.html’ in the corresponding directories to start exploring the data. [file 1471-2164-15-74-S4.zip › DLD1_Biocarta/BIOCARTA_RAS_PATHWAY.html]

Details for gene set BIOCARTA\_RAS\_PATHWAY[GSEA]

|  || Dataset | DLD1\_collapsed\_to\_symbols.class.cls#bg\_versus\_b |
| Phenotype | class.cls#bg\_versus\_b |
| Upregulated in class | b |
| GeneSet | BIOCARTA\_RAS\_PATHWAY |
| Enrichment Score (ES) | -0.59007347 |
| Normalized Enrichment Score (NES) | -1.6866643 |
| Nominal p-value | 0.012096774 |
| FDR q-value | 0.19160521 |
| FWER p-Value | 0.637 |
Table: GSEA Results Summary

  

Fig 1: Enrichment plot: BIOCARTA\_RAS\_PATHWAY      
 Profile of the Running ES Score & Positions of GeneSet Members on the Rank Ordered List

  

| PROBE | GENE SYMBOL | GENE\_TITLE | RANK IN GENE LIST | RANK METRIC SCORE | RUNNING ES | CORE ENRICHMENT || 1 | ELK1 | ELK1 Entrez,  Source | ELK1, member of ETS oncogene family | 3873 | 0.073 | -0.1464 | No |
| 2 | PIK3R1 | PIK3R1 Entrez,  Source | phosphoinositide-3-kinase, regulatory subunit 1 (p85 alpha) | 8011 | 0.024 | -0.3411 | No |
| 3 | NFKB1 | NFKB1 Entrez,  Source | nuclear factor of kappa light polypeptide gene enhancer in B-cells 1 (p105) | 8386 | 0.021 | -0.3457 | No |
| 4 | BCL2L1 | BCL2L1 Entrez,  Source | BCL2-like 1 | 10051 | 0.006 | -0.4265 | No |
| 5 | HRAS | HRAS Entrez,  Source | v-Ha-ras Harvey rat sarcoma viral oncogene homolog | 10612 | 0.001 | -0.4543 | No |
| 6 | RELA | RELA Entrez,  Source | v-rel reticuloendotheliosis viral oncogene homolog A, nuclear factor of kappa light polypeptide gene enhancer in B-cells 3, p65 (avian) | 11371 | -0.006 | -0.4891 | No |
| 7 | AKT1 | AKT1 Entrez,  Source | v-akt murine thymoma viral oncogene homolog 1 | 11660 | -0.008 | -0.4981 | No |
| 8 | RALA | RALA Entrez,  Source | v-ral simian leukemia viral oncogene homolog A (ras related) | 11797 | -0.009 | -0.4984 | No |
| 9 | PIK3CG | PIK3CG Entrez,  Source | phosphoinositide-3-kinase, catalytic, gamma polypeptide | 12525 | -0.017 | -0.5236 | No |
| 10 | RHOA | RHOA Entrez,  Source | ras homolog gene family, member A | 13129 | -0.023 | -0.5381 | No |
| 11 | MAP2K1 | MAP2K1 Entrez,  Source | mitogen-activated protein kinase kinase 1 | 13627 | -0.028 | -0.5434 | No |
| 12 | RAF1 | RAF1 Entrez,  Source | v-raf-1 murine leukemia viral oncogene homolog 1 | 13629 | -0.028 | -0.5234 | No |
| 13 | RALBP1 | RALBP1 Entrez,  Source | ralA binding protein 1 | 14725 | -0.042 | -0.5494 | Yes |
| 14 | RAC1 | RAC1 Entrez,  Source | ras-related C3 botulinum toxin substrate 1 (rho family, small GTP binding protein Rac1) | 14979 | -0.046 | -0.5299 | Yes |
| 15 | CHUK | CHUK Entrez,  Source | conserved helix-loop-helix ubiquitous kinase | 15639 | -0.056 | -0.5240 | Yes |
| 16 | RALGDS | RALGDS Entrez,  Source | ral guanine nucleotide dissociation stimulator | 16930 | -0.082 | -0.5320 | Yes |
| 17 | CDC42 | CDC42 Entrez,  Source | cell division cycle 42 (GTP binding protein, 25kDa) | 17370 | -0.093 | -0.4883 | Yes |
| 18 | PIK3CA | PIK3CA Entrez,  Source | phosphoinositide-3-kinase, catalytic, alpha polypeptide | 17601 | -0.101 | -0.4286 | Yes |
| 19 | CASP9 | CASP9 Entrez,  Source | caspase 9, apoptosis-related cysteine peptidase | 18088 | -0.119 | -0.3691 | Yes |
| 20 | MAPK3 | MAPK3 Entrez,  Source | mitogen-activated protein kinase 3 | 18647 | -0.156 | -0.2871 | Yes |
| 21 | BAD | BAD Entrez,  Source | BCL2-antagonist of cell death | 19150 | -0.230 | -0.1500 | Yes |
| 22 | PLD1 | PLD1 Entrez,  Source | phospholipase D1, phosphatidylcholine-specific | 19198 | -0.241 | 0.0183 | Yes |
Table: GSEA details [plain text format]

  

Fig 2: BIOCARTA\_RAS\_PATHWAY      
 Blue-Pink O' Gram in the Space of the Analyzed GeneSet

  

Fig 3: BIOCARTA\_RAS\_PATHWAY: Random ES distribution      
 Gene set null distribution of ES for **BIOCARTA\_RAS\_PATHWAY**

  
