# Supplementary material for: Comprehensive analysis of β-catenin target genes in colorectal carcinoma cell lines with deregulated Wnt/β-catenin signaling
Source: BMC Genomics. 2014 Jan 28;15:74. doi: 10.1186/1471-2164-15-74 (PMC3909937; doi:10.1186/1471-2164-15-74)
Supplement: Additional file 4 — GSEA analysis using the Biocarta pathway database. This zipped file contains confirming data of the GSEA analysis. The names of the directories containing the files were composed of the term ‘GSEA’, the name of the cell line, e.g. DLD1, SW480, or LS174T, and the pathway database (Biocarta). Please use a web browser to view the files with the name ‘index.html’ in the corresponding directories to start exploring the data. [file 1471-2164-15-74-S4.zip › DLD1_Biocarta/BIOCARTA_RHO_PATHWAY.html]

Details for gene set BIOCARTA\_RHO\_PATHWAY[GSEA]

|  || Dataset | DLD1\_collapsed\_to\_symbols.class.cls#bg\_versus\_b |
| Phenotype | class.cls#bg\_versus\_b |
| Upregulated in class | b |
| GeneSet | BIOCARTA\_RHO\_PATHWAY |
| Enrichment Score (ES) | -0.57683045 |
| Normalized Enrichment Score (NES) | -1.7745221 |
| Nominal p-value | 0.0 |
| FDR q-value | 0.13283503 |
| FWER p-Value | 0.405 |
Table: GSEA Results Summary

  

Fig 1: Enrichment plot: BIOCARTA\_RHO\_PATHWAY      
 Profile of the Running ES Score & Positions of GeneSet Members on the Rank Ordered List

  

| PROBE | GENE SYMBOL | GENE\_TITLE | RANK IN GENE LIST | RANK METRIC SCORE | RUNNING ES | CORE ENRICHMENT || 1 | ARHGAP4 | ARHGAP4 Entrez,  Source | Rho GTPase activating protein 4 | 2315 | 0.104 | -0.0761 | No |
| 2 | ARHGAP6 | ARHGAP6 Entrez,  Source | Rho GTPase activating protein 6 | 3204 | 0.085 | -0.0867 | No |
| 3 | PFN1 | PFN1 Entrez,  Source | profilin 1 | 8523 | 0.019 | -0.3511 | No |
| 4 | PIP5K1A | PIP5K1A Entrez,  Source | phosphatidylinositol-4-phosphate 5-kinase, type I, alpha | 8995 | 0.016 | -0.3689 | No |
| 5 | ARHGAP5 | ARHGAP5 Entrez,  Source | Rho GTPase activating protein 5 | 10925 | -0.001 | -0.4671 | No |
| 6 | ARHGEF11 | ARHGEF11 Entrez,  Source | Rho guanine nucleotide exchange factor (GEF) 11 | 11587 | -0.007 | -0.4980 | No |
| 7 | ARHGEF1 | ARHGEF1 Entrez,  Source | Rho guanine nucleotide exchange factor (GEF) 1 | 11793 | -0.009 | -0.5046 | No |
| 8 | TLN1 | TLN1 Entrez,  Source | talin 1 | 12261 | -0.014 | -0.5227 | No |
| 9 | CFL1 | CFL1 Entrez,  Source | cofilin 1 (non-muscle) | 12491 | -0.017 | -0.5277 | No |
| 10 | ARHGEF5 | ARHGEF5 Entrez,  Source | Rho guanine nucleotide exchange factor (GEF) 5 | 12550 | -0.017 | -0.5237 | No |
| 11 | OPHN1 | OPHN1 Entrez,  Source | oligophrenin 1 | 13007 | -0.022 | -0.5380 | No |
| 12 | RHOA | RHOA Entrez,  Source | ras homolog gene family, member A | 13129 | -0.023 | -0.5348 | No |
| 13 | PPP1R12B | PPP1R12B Entrez,  Source | protein phosphatase 1, regulatory (inhibitor) subunit 12B | 13235 | -0.024 | -0.5303 | No |
| 14 | DIAPH1 | DIAPH1 Entrez,  Source | diaphanous homolog 1 (Drosophila) | 13325 | -0.025 | -0.5245 | No |
| 15 | ACTR2 | ACTR2 Entrez,  Source | ARP2 actin-related protein 2 homolog (yeast) | 13514 | -0.027 | -0.5230 | No |
| 16 | ARPC2 | ARPC2 Entrez,  Source | actin related protein 2/3 complex, subunit 2, 34kDa | 13578 | -0.028 | -0.5149 | No |
| 17 | ROCK1 | ROCK1 Entrez,  Source | Rho-associated, coiled-coil containing protein kinase 1 | 13714 | -0.029 | -0.5098 | No |
| 18 | ACTR3 | ACTR3 Entrez,  Source | ARP3 actin-related protein 3 homolog (yeast) | 13954 | -0.032 | -0.5089 | No |
| 19 | LIMK1 | LIMK1 Entrez,  Source | LIM domain kinase 1 | 15282 | -0.050 | -0.5564 | Yes |
| 20 | SRC | SRC Entrez,  Source | v-src sarcoma (Schmidt-Ruppin A-2) viral oncogene homolog (avian) | 15682 | -0.056 | -0.5538 | Yes |
| 21 | ARPC5 | ARPC5 Entrez,  Source | actin related protein 2/3 complex, subunit 5, 16kDa | 15899 | -0.060 | -0.5403 | Yes |
| 22 | BAIAP2 | BAIAP2 Entrez,  Source | BAI1-associated protein 2 | 16126 | -0.064 | -0.5257 | Yes |
| 23 | ARPC3 | ARPC3 Entrez,  Source | actin related protein 2/3 complex, subunit 3, 21kDa | 16471 | -0.072 | -0.5141 | Yes |
| 24 | VCL | VCL Entrez,  Source | vinculin | 16580 | -0.074 | -0.4896 | Yes |
| 25 | ARPC4 | ARPC4 Entrez,  Source | actin related protein 2/3 complex, subunit 4, 20kDa | 17054 | -0.085 | -0.4793 | Yes |
| 26 | ARHGAP1 | ARHGAP1 Entrez,  Source | Rho GTPase activating protein 1 | 17083 | -0.086 | -0.4458 | Yes |
| 27 | ARPC1A | ARPC1A Entrez,  Source | actin related protein 2/3 complex, subunit 1A, 41kDa | 17416 | -0.095 | -0.4242 | Yes |
| 28 | MYL2 | MYL2 Entrez,  Source | myosin, light chain 2, regulatory, cardiac, slow | 18730 | -0.164 | -0.4246 | Yes |
| 29 | PIP5K1B | PIP5K1B Entrez,  Source | phosphatidylinositol-4-phosphate 5-kinase, type I, beta | 18907 | -0.183 | -0.3590 | Yes |
| 30 | GSN | GSN Entrez,  Source | gelsolin (amyloidosis, Finnish type) | 19432 | -0.405 | -0.2206 | Yes |
| 31 | MYLK | MYLK Entrez,  Source | myosin, light chain kinase | 19507 | -0.557 | 0.0025 | Yes |
Table: GSEA details [plain text format]

  

Fig 2: BIOCARTA\_RHO\_PATHWAY      
 Blue-Pink O' Gram in the Space of the Analyzed GeneSet

  

Fig 3: BIOCARTA\_RHO\_PATHWAY: Random ES distribution      
 Gene set null distribution of ES for **BIOCARTA\_RHO\_PATHWAY**

  
