# Supplementary material for: Comprehensive analysis of β-catenin target genes in colorectal carcinoma cell lines with deregulated Wnt/β-catenin signaling
Source: BMC Genomics. 2014 Jan 28;15:74. doi: 10.1186/1471-2164-15-74 (PMC3909937; doi:10.1186/1471-2164-15-74)
Supplement: Additional file 4 — GSEA analysis using the Biocarta pathway database. This zipped file contains confirming data of the GSEA analysis. The names of the directories containing the files were composed of the term ‘GSEA’, the name of the cell line, e.g. DLD1, SW480, or LS174T, and the pathway database (Biocarta). Please use a web browser to view the files with the name ‘index.html’ in the corresponding directories to start exploring the data. [file 1471-2164-15-74-S4.zip › DLD1_Biocarta/BIOCARTA_STATHMIN_PATHWAY.html]

Details for gene set BIOCARTA\_STATHMIN\_PATHWAY[GSEA]

|  || Dataset | DLD1\_collapsed\_to\_symbols.class.cls#bg\_versus\_b |
| Phenotype | class.cls#bg\_versus\_b |
| Upregulated in class | b |
| GeneSet | BIOCARTA\_STATHMIN\_PATHWAY |
| Enrichment Score (ES) | -0.5413153 |
| Normalized Enrichment Score (NES) | -1.4337798 |
| Nominal p-value | 0.07939914 |
| FDR q-value | 0.33333072 |
| FWER p-Value | 0.999 |
Table: GSEA Results Summary

  

Fig 1: Enrichment plot: BIOCARTA\_STATHMIN\_PATHWAY      
 Profile of the Running ES Score & Positions of GeneSet Members on the Rank Ordered List

  

| PROBE | GENE SYMBOL | GENE\_TITLE | RANK IN GENE LIST | RANK METRIC SCORE | RUNNING ES | CORE ENRICHMENT || 1 | CD3E | CD3E Entrez,  Source | CD3e molecule, epsilon (CD3-TCR complex) | 2097 | 0.109 | -0.0360 | No |
| 2 | CAMK2A | CAMK2A Entrez,  Source | calcium/calmodulin-dependent protein kinase (CaM kinase) II alpha | 2780 | 0.094 | -0.0098 | No |
| 3 | CD3D | CD3D Entrez,  Source | CD3d molecule, delta (CD3-TCR complex) | 5170 | 0.054 | -0.0969 | No |
| 4 | CAMK4 | CAMK4 Entrez,  Source | calcium/calmodulin-dependent protein kinase IV | 5439 | 0.051 | -0.0776 | No |
| 5 | CCNB1 | CCNB1 Entrez,  Source | cyclin B1 | 9414 | 0.012 | -0.2733 | No |
| 6 | CD2 | CD2 Entrez,  Source | CD2 molecule | 9595 | 0.010 | -0.2759 | No |
| 7 | CAMK2B | CAMK2B Entrez,  Source | calcium/calmodulin-dependent protein kinase (CaM kinase) II beta | 11597 | -0.007 | -0.3735 | No |
| 8 | CD247 | CD247 Entrez,  Source | CD247 molecule | 12348 | -0.015 | -0.4019 | No |
| 9 | PRKAR2A | PRKAR2A Entrez,  Source | protein kinase, cAMP-dependent, regulatory, type II, alpha | 12398 | -0.016 | -0.3942 | No |
| 10 | PRKACG | PRKACG Entrez,  Source | protein kinase, cAMP-dependent, catalytic, gamma | 13683 | -0.029 | -0.4410 | No |
| 11 | MAPK13 | MAPK13 Entrez,  Source | mitogen-activated protein kinase 13 | 13693 | -0.029 | -0.4224 | No |
| 12 | PRKAR1A | PRKAR1A Entrez,  Source | protein kinase, cAMP-dependent, regulatory, type I, alpha (tissue specific extinguisher 1) | 14125 | -0.034 | -0.4221 | No |
| 13 | CD3G | CD3G Entrez,  Source | CD3g molecule, gamma (CD3-TCR complex) | 14989 | -0.046 | -0.4362 | No |
| 14 | CAMK2G | CAMK2G Entrez,  Source | calcium/calmodulin-dependent protein kinase (CaM kinase) II gamma | 17043 | -0.085 | -0.4862 | Yes |
| 15 | PRKACB | PRKACB Entrez,  Source | protein kinase, cAMP-dependent, catalytic, beta | 17727 | -0.105 | -0.4528 | Yes |
| 16 | CAMK2D | CAMK2D Entrez,  Source | calcium/calmodulin-dependent protein kinase (CaM kinase) II delta | 18920 | -0.185 | -0.3935 | Yes |
| 17 | PRKAR2B | PRKAR2B Entrez,  Source | protein kinase, cAMP-dependent, regulatory, type II, beta | 19526 | -0.654 | 0.0015 | Yes |
Table: GSEA details [plain text format]

  

Fig 2: BIOCARTA\_STATHMIN\_PATHWAY      
 Blue-Pink O' Gram in the Space of the Analyzed GeneSet

  

Fig 3: BIOCARTA\_STATHMIN\_PATHWAY: Random ES distribution      
 Gene set null distribution of ES for **BIOCARTA\_STATHMIN\_PATHWAY**

  
