# Supplementary material for: Comprehensive analysis of β-catenin target genes in colorectal carcinoma cell lines with deregulated Wnt/β-catenin signaling
Source: BMC Genomics. 2014 Jan 28;15:74. doi: 10.1186/1471-2164-15-74 (PMC3909937; doi:10.1186/1471-2164-15-74)
Supplement: Additional file 4 — GSEA analysis using the Biocarta pathway database. This zipped file contains confirming data of the GSEA analysis. The names of the directories containing the files were composed of the term ‘GSEA’, the name of the cell line, e.g. DLD1, SW480, or LS174T, and the pathway database (Biocarta). Please use a web browser to view the files with the name ‘index.html’ in the corresponding directories to start exploring the data. [file 1471-2164-15-74-S4.zip › DLD1_Biocarta/BIOCARTA_TOB1_PATHWAY.html]

Details for gene set BIOCARTA\_TOB1\_PATHWAY[GSEA]

|  || Dataset | DLD1\_collapsed\_to\_symbols.class.cls#bg\_versus\_b |
| Phenotype | class.cls#bg\_versus\_b |
| Upregulated in class | bg |
| GeneSet | BIOCARTA\_TOB1\_PATHWAY |
| Enrichment Score (ES) | 0.46548906 |
| Normalized Enrichment Score (NES) | 1.2701762 |
| Nominal p-value | 0.17636022 |
| FDR q-value | 0.575677 |
| FWER p-Value | 1.0 |
Table: GSEA Results Summary

  

Fig 1: Enrichment plot: BIOCARTA\_TOB1\_PATHWAY      
 Profile of the Running ES Score & Positions of GeneSet Members on the Rank Ordered List

  

| PROBE | GENE SYMBOL | GENE\_TITLE | RANK IN GENE LIST | RANK METRIC SCORE | RUNNING ES | CORE ENRICHMENT || 1 | TGFB2 | TGFB2 Entrez,  Source | transforming growth factor, beta 2 | 165 | 0.290 | 0.1790 | Yes |
| 2 | IFNG | IFNG Entrez,  Source | interferon, gamma | 571 | 0.193 | 0.2832 | Yes |
| 3 | TGFB1 | TGFB1 Entrez,  Source | transforming growth factor, beta 1 (Camurati-Engelmann disease) | 1587 | 0.126 | 0.3131 | Yes |
| 4 | CD3E | CD3E Entrez,  Source | CD3e molecule, epsilon (CD3-TCR complex) | 2097 | 0.109 | 0.3579 | Yes |
| 5 | TGFBR3 | TGFBR3 Entrez,  Source | transforming growth factor, beta receptor III (betaglycan, 300kDa) | 2187 | 0.107 | 0.4228 | Yes |
| 6 | TGFBR2 | TGFBR2 Entrez,  Source | transforming growth factor, beta receptor II (70/80kDa) | 2589 | 0.098 | 0.4655 | Yes |
| 7 | TGFB3 | TGFB3 Entrez,  Source | transforming growth factor, beta 3 | 4247 | 0.067 | 0.4242 | No |
| 8 | CD3D | CD3D Entrez,  Source | CD3d molecule, delta (CD3-TCR complex) | 5170 | 0.054 | 0.4119 | No |
| 9 | IL2RA | IL2RA Entrez,  Source | interleukin 2 receptor, alpha | 5186 | 0.054 | 0.4459 | No |
| 10 | CD28 | CD28 Entrez,  Source | CD28 molecule | 7039 | 0.033 | 0.3726 | No |
| 11 | TRA@ | TRA@ Entrez,  Source | T cell receptor alpha locus | 8121 | 0.023 | 0.3322 | No |
| 12 | TGFBR1 | TGFBR1 Entrez,  Source | transforming growth factor, beta receptor I (activin A receptor type II-like kinase, 53kDa) | 8576 | 0.019 | 0.3213 | No |
| 13 | IL2 | IL2 Entrez,  Source | interleukin 2 | 11037 | -0.002 | 0.1970 | No |
| 14 | IL4 | IL4 Entrez,  Source | interleukin 4 | 12028 | -0.012 | 0.1540 | No |
| 15 | CD247 | CD247 Entrez,  Source | CD247 molecule | 12348 | -0.015 | 0.1476 | No |
| 16 | TOB2 | TOB2 Entrez,  Source | transducer of ERBB2, 2 | 13788 | -0.030 | 0.0935 | No |
| 17 | SMAD4 | SMAD4 Entrez,  Source | SMAD, mothers against DPP homolog 4 (Drosophila) | 14222 | -0.036 | 0.0944 | No |
| 18 | CD3G | CD3G Entrez,  Source | CD3g molecule, gamma (CD3-TCR complex) | 14989 | -0.046 | 0.0850 | No |
| 19 | SMAD3 | SMAD3 Entrez,  Source | SMAD, mothers against DPP homolog 3 (Drosophila) | 17945 | -0.113 | 0.0070 | No |
| 20 | TOB1 | TOB1 Entrez,  Source | transducer of ERBB2, 1 | 18026 | -0.116 | 0.0783 | No |
Table: GSEA details [plain text format]

  

Fig 2: BIOCARTA\_TOB1\_PATHWAY      
 Blue-Pink O' Gram in the Space of the Analyzed GeneSet

  

Fig 3: BIOCARTA\_TOB1\_PATHWAY: Random ES distribution      
 Gene set null distribution of ES for **BIOCARTA\_TOB1\_PATHWAY**

  
