# Supplementary material for: Comprehensive analysis of β-catenin target genes in colorectal carcinoma cell lines with deregulated Wnt/β-catenin signaling
Source: BMC Genomics. 2014 Jan 28;15:74. doi: 10.1186/1471-2164-15-74 (PMC3909937; doi:10.1186/1471-2164-15-74)
Supplement: Additional file 4 — GSEA analysis using the Biocarta pathway database. This zipped file contains confirming data of the GSEA analysis. The names of the directories containing the files were composed of the term ‘GSEA’, the name of the cell line, e.g. DLD1, SW480, or LS174T, and the pathway database (Biocarta). Please use a web browser to view the files with the name ‘index.html’ in the corresponding directories to start exploring the data. [file 1471-2164-15-74-S4.zip › DLD1_Biocarta/gsea_report_for_b_1355836171296.html]

Report for b 1355836171296 [GSEA]

| GS  follow link to MSigDB | GS DETAILS | SIZE | ES | NES | NOM p-val | FDR q-val | FWER p-val | RANK AT MAX | LEADING EDGE || 1 | BIOCARTA\_MCALPAIN\_PATHWAY | Details ... | 22 | -0.71 | -2.00 | 0.000 | 0.047 | 0.036 | 3865 | tags=64%, list=20%, signal=79% |
| 2 | BIOCARTA\_CREB\_PATHWAY | Details ... | 25 | -0.62 | -1.84 | 0.002 | 0.181 | 0.249 | 4579 | tags=52%, list=23%, signal=68% |
| 3 | BIOCARTA\_ECM\_PATHWAY | Details ... | 23 | -0.66 | -1.83 | 0.002 | 0.133 | 0.268 | 3876 | tags=39%, list=20%, signal=49% |
| 4 | BIOCARTA\_MPR\_PATHWAY | Details ... | 31 | -0.58 | -1.81 | 0.004 | 0.121 | 0.311 | 4064 | tags=48%, list=21%, signal=61% |
| 5 | BIOCARTA\_RHO\_PATHWAY | Details ... | 31 | -0.58 | -1.77 | 0.000 | 0.133 | 0.405 | 4276 | tags=42%, list=22%, signal=54% |
| 6 | BIOCARTA\_IL10\_PATHWAY | Details ... | 17 | -0.64 | -1.71 | 0.015 | 0.185 | 0.561 | 5348 | tags=71%, list=27%, signal=97% |
| 7 | BIOCARTA\_RAS\_PATHWAY | Details ... | 22 | -0.59 | -1.69 | 0.012 | 0.192 | 0.637 | 2628 | tags=32%, list=13%, signal=37% |
| 8 | BIOCARTA\_IGF1R\_PATHWAY | Details ... | 22 | -0.60 | -1.66 | 0.013 | 0.203 | 0.697 | 2094 | tags=27%, list=11%, signal=31% |
| 9 | BIOCARTA\_CDC42RAC\_PATHWAY | Details ... | 15 | -0.63 | -1.66 | 0.027 | 0.186 | 0.714 | 6429 | tags=87%, list=33%, signal=129% |
| 10 | BIOCARTA\_IL3\_PATHWAY | Details ... | 15 | -0.65 | -1.65 | 0.015 | 0.178 | 0.729 | 6179 | tags=73%, list=32%, signal=107% |
| 11 | BIOCARTA\_EDG1\_PATHWAY | Details ... | 24 | -0.56 | -1.59 | 0.027 | 0.246 | 0.864 | 4579 | tags=50%, list=23%, signal=65% |
| 12 | BIOCARTA\_SPRY\_PATHWAY | Details ... | 18 | -0.59 | -1.55 | 0.038 | 0.278 | 0.916 | 4073 | tags=50%, list=21%, signal=63% |
| 13 | BIOCARTA\_ACTINY\_PATHWAY | Details ... | 19 | -0.57 | -1.55 | 0.043 | 0.264 | 0.919 | 6044 | tags=79%, list=31%, signal=114% |
| 14 | BIOCARTA\_CXCR4\_PATHWAY | Details ... | 23 | -0.52 | -1.54 | 0.034 | 0.263 | 0.933 | 4250 | tags=52%, list=22%, signal=67% |
| 15 | BIOCARTA\_HER2\_PATHWAY | Details ... | 22 | -0.53 | -1.52 | 0.042 | 0.267 | 0.943 | 5931 | tags=59%, list=30%, signal=85% |
| 16 | BIOCARTA\_BIOPEPTIDES\_PATHWAY | Details ... | 41 | -0.46 | -1.48 | 0.037 | 0.332 | 0.981 | 6119 | tags=56%, list=31%, signal=81% |
| 17 | BIOCARTA\_ERK5\_PATHWAY | Details ... | 17 | -0.54 | -1.47 | 0.101 | 0.327 | 0.986 | 4346 | tags=53%, list=22%, signal=68% |
| 18 | BIOCARTA\_NOS1\_PATHWAY | Details ... | 19 | -0.55 | -1.46 | 0.072 | 0.328 | 0.990 | 2225 | tags=21%, list=11%, signal=24% |
| 19 | BIOCARTA\_MET\_PATHWAY | Details ... | 35 | -0.47 | -1.45 | 0.079 | 0.322 | 0.994 | 6582 | tags=63%, list=34%, signal=95% |
| 20 | BIOCARTA\_STATHMIN\_PATHWAY | Details ... | 17 | -0.54 | -1.43 | 0.079 | 0.333 | 0.999 | 2515 | tags=24%, list=13%, signal=27% |
| 21 | BIOCARTA\_GCR\_PATHWAY |  | 19 | -0.52 | -1.42 | 0.099 | 0.332 | 0.999 | 2955 | tags=21%, list=15%, signal=25% |
| 22 | BIOCARTA\_GPCR\_PATHWAY |  | 32 | -0.46 | -1.40 | 0.074 | 0.356 | 0.999 | 4250 | tags=34%, list=22%, signal=44% |
| 23 | BIOCARTA\_BAD\_PATHWAY |  | 25 | -0.49 | -1.40 | 0.075 | 0.346 | 0.999 | 2094 | tags=32%, list=11%, signal=36% |
| 24 | BIOCARTA\_GH\_PATHWAY |  | 26 | -0.48 | -1.39 | 0.099 | 0.343 | 1.000 | 4250 | tags=46%, list=22%, signal=59% |
| 25 | BIOCARTA\_PPARA\_PATHWAY |  | 52 | -0.41 | -1.38 | 0.070 | 0.338 | 1.000 | 2903 | tags=27%, list=15%, signal=32% |
| 26 | BIOCARTA\_CHREBP2\_PATHWAY |  | 41 | -0.42 | -1.37 | 0.079 | 0.347 | 1.000 | 3056 | tags=24%, list=16%, signal=29% |
| 27 | BIOCARTA\_AT1R\_PATHWAY |  | 31 | -0.45 | -1.36 | 0.093 | 0.357 | 1.000 | 5931 | tags=68%, list=30%, signal=97% |
| 28 | BIOCARTA\_IL22BP\_PATHWAY |  | 16 | -0.51 | -1.34 | 0.146 | 0.382 | 1.000 | 6076 | tags=69%, list=31%, signal=100% |
| 29 | BIOCARTA\_TPO\_PATHWAY |  | 23 | -0.46 | -1.32 | 0.150 | 0.405 | 1.000 | 5931 | tags=57%, list=30%, signal=81% |
| 30 | BIOCARTA\_EGF\_PATHWAY |  | 30 | -0.43 | -1.29 | 0.166 | 0.439 | 1.000 | 6076 | tags=57%, list=31%, signal=82% |
| 31 | BIOCARTA\_NO1\_PATHWAY |  | 28 | -0.43 | -1.28 | 0.138 | 0.442 | 1.000 | 2225 | tags=21%, list=11%, signal=24% |
| 32 | BIOCARTA\_DEATH\_PATHWAY |  | 32 | -0.42 | -1.28 | 0.157 | 0.431 | 1.000 | 4587 | tags=47%, list=23%, signal=61% |
| 33 | BIOCARTA\_PYK2\_PATHWAY |  | 26 | -0.43 | -1.27 | 0.169 | 0.435 | 1.000 | 5931 | tags=62%, list=30%, signal=88% |
| 34 | BIOCARTA\_MAL\_PATHWAY |  | 18 | -0.45 | -1.27 | 0.169 | 0.423 | 1.000 | 6579 | tags=67%, list=34%, signal=100% |
| 35 | BIOCARTA\_INTEGRIN\_PATHWAY |  | 37 | -0.40 | -1.25 | 0.166 | 0.448 | 1.000 | 6579 | tags=62%, list=34%, signal=93% |
| 36 | BIOCARTA\_CERAMIDE\_PATHWAY |  | 21 | -0.45 | -1.25 | 0.194 | 0.438 | 1.000 | 1474 | tags=19%, list=8%, signal=21% |
| 37 | BIOCARTA\_UCALPAIN\_PATHWAY |  | 16 | -0.46 | -1.23 | 0.228 | 0.448 | 1.000 | 5023 | tags=56%, list=26%, signal=76% |
| 38 | BIOCARTA\_CCR3\_PATHWAY |  | 22 | -0.43 | -1.21 | 0.216 | 0.485 | 1.000 | 6429 | tags=77%, list=33%, signal=115% |
| 39 | BIOCARTA\_PGC1A\_PATHWAY |  | 22 | -0.42 | -1.19 | 0.232 | 0.494 | 1.000 | 3700 | tags=41%, list=19%, signal=50% |
| 40 | BIOCARTA\_NFKB\_PATHWAY |  | 22 | -0.42 | -1.18 | 0.273 | 0.511 | 1.000 | 1863 | tags=23%, list=10%, signal=25% |
| 41 | BIOCARTA\_SHH\_PATHWAY |  | 15 | -0.45 | -1.17 | 0.256 | 0.521 | 1.000 | 1831 | tags=20%, list=9%, signal=22% |
| 42 | BIOCARTA\_RAC1\_PATHWAY |  | 23 | -0.40 | -1.15 | 0.270 | 0.549 | 1.000 | 828 | tags=13%, list=4%, signal=14% |
| 43 | BIOCARTA\_IL6\_PATHWAY |  | 22 | -0.41 | -1.15 | 0.282 | 0.538 | 1.000 | 911 | tags=14%, list=5%, signal=14% |
| 44 | BIOCARTA\_IL1R\_PATHWAY |  | 31 | -0.37 | -1.13 | 0.271 | 0.562 | 1.000 | 1863 | tags=19%, list=10%, signal=21% |
| 45 | BIOCARTA\_CK1\_PATHWAY |  | 16 | -0.42 | -1.12 | 0.336 | 0.575 | 1.000 | 32 | tags=6%, list=0%, signal=6% |
| 46 | BIOCARTA\_IL7\_PATHWAY |  | 17 | -0.43 | -1.11 | 0.312 | 0.564 | 1.000 | 4061 | tags=41%, list=21%, signal=52% |
| 47 | BIOCARTA\_PDGF\_PATHWAY |  | 31 | -0.36 | -1.11 | 0.293 | 0.562 | 1.000 | 6076 | tags=52%, list=31%, signal=75% |
| 48 | BIOCARTA\_ERK\_PATHWAY |  | 27 | -0.38 | -1.11 | 0.325 | 0.554 | 1.000 | 4298 | tags=41%, list=22%, signal=52% |
| 49 | BIOCARTA\_MYOSIN\_PATHWAY |  | 30 | -0.35 | -1.07 | 0.353 | 0.621 | 1.000 | 991 | tags=10%, list=5%, signal=11% |
| 50 | BIOCARTA\_GLEEVEC\_PATHWAY |  | 23 | -0.38 | -1.06 | 0.368 | 0.622 | 1.000 | 1957 | tags=22%, list=10%, signal=24% |
| 51 | BIOCARTA\_VIP\_PATHWAY |  | 25 | -0.36 | -1.06 | 0.378 | 0.614 | 1.000 | 2274 | tags=24%, list=12%, signal=27% |
| 52 | BIOCARTA\_NFAT\_PATHWAY |  | 52 | -0.31 | -1.05 | 0.359 | 0.618 | 1.000 | 3874 | tags=25%, list=20%, signal=31% |
| 53 | BIOCARTA\_MEF2D\_PATHWAY |  | 18 | -0.39 | -1.05 | 0.366 | 0.608 | 1.000 | 4250 | tags=39%, list=22%, signal=50% |
| 54 | BIOCARTA\_TFF\_PATHWAY |  | 21 | -0.38 | -1.04 | 0.371 | 0.606 | 1.000 | 4073 | tags=43%, list=21%, signal=54% |
| 55 | BIOCARTA\_CSK\_PATHWAY |  | 22 | -0.36 | -1.03 | 0.432 | 0.626 | 1.000 | 32 | tags=5%, list=0%, signal=5% |
| 56 | BIOCARTA\_PTEN\_PATHWAY |  | 17 | -0.39 | -1.03 | 0.426 | 0.618 | 1.000 | 5832 | tags=65%, list=30%, signal=92% |
| 57 | BIOCARTA\_AKT\_PATHWAY |  | 19 | -0.37 | -1.02 | 0.421 | 0.619 | 1.000 | 4440 | tags=37%, list=23%, signal=48% |
| 58 | BIOCARTA\_EPO\_PATHWAY |  | 19 | -0.37 | -1.01 | 0.450 | 0.627 | 1.000 | 5931 | tags=47%, list=30%, signal=68% |
| 59 | BIOCARTA\_CALCINEURIN\_PATHWAY |  | 17 | -0.37 | -1.01 | 0.427 | 0.625 | 1.000 | 4250 | tags=35%, list=22%, signal=45% |
| 60 | BIOCARTA\_NKCELLS\_PATHWAY |  | 17 | -0.37 | -0.99 | 0.460 | 0.641 | 1.000 | 5023 | tags=53%, list=26%, signal=71% |
| 61 | BIOCARTA\_TGFB\_PATHWAY |  | 18 | -0.37 | -0.99 | 0.466 | 0.633 | 1.000 | 2126 | tags=22%, list=11%, signal=25% |
| 62 | BIOCARTA\_EIF4\_PATHWAY |  | 22 | -0.33 | -0.96 | 0.481 | 0.677 | 1.000 | 4250 | tags=36%, list=22%, signal=46% |
| 63 | BIOCARTA\_41BB\_PATHWAY |  | 17 | -0.36 | -0.96 | 0.534 | 0.679 | 1.000 | 4440 | tags=41%, list=23%, signal=53% |
| 64 | BIOCARTA\_KERATINOCYTE\_PATHWAY |  | 44 | -0.29 | -0.93 | 0.540 | 0.708 | 1.000 | 1901 | tags=18%, list=10%, signal=20% |
| 65 | BIOCARTA\_IL2RB\_PATHWAY |  | 38 | -0.29 | -0.93 | 0.549 | 0.701 | 1.000 | 4073 | tags=34%, list=21%, signal=43% |
| 66 | BIOCARTA\_NDKDYNAMIN\_PATHWAY |  | 17 | -0.33 | -0.88 | 0.615 | 0.798 | 1.000 | 4007 | tags=35%, list=20%, signal=44% |
| 67 | BIOCARTA\_MAPK\_PATHWAY |  | 85 | -0.23 | -0.86 | 0.741 | 0.818 | 1.000 | 4676 | tags=34%, list=24%, signal=45% |
| 68 | BIOCARTA\_CHEMICAL\_PATHWAY |  | 21 | -0.30 | -0.84 | 0.666 | 0.844 | 1.000 | 4676 | tags=38%, list=24%, signal=50% |
| 69 | BIOCARTA\_BARRESTIN\_SRC\_PATHWAY |  | 15 | -0.32 | -0.82 | 0.686 | 0.875 | 1.000 | 6579 | tags=67%, list=34%, signal=100% |
| 70 | BIOCARTA\_AMI\_PATHWAY |  | 19 | -0.30 | -0.80 | 0.705 | 0.893 | 1.000 | 2840 | tags=26%, list=15%, signal=31% |
| 71 | BIOCARTA\_INSULIN\_PATHWAY |  | 22 | -0.29 | -0.79 | 0.739 | 0.892 | 1.000 | 4073 | tags=23%, list=21%, signal=29% |
| 72 | BIOCARTA\_PROTEASOME\_PATHWAY |  | 26 | -0.27 | -0.77 | 0.798 | 0.922 | 1.000 | 14332 | tags=100%, list=73%, signal=374% |
| 73 | BIOCARTA\_AGR\_PATHWAY |  | 35 | -0.25 | -0.76 | 0.820 | 0.921 | 1.000 | 5039 | tags=46%, list=26%, signal=61% |
| 74 | BIOCARTA\_PTDINS\_PATHWAY |  | 23 | -0.27 | -0.76 | 0.795 | 0.910 | 1.000 | 5641 | tags=39%, list=29%, signal=55% |
| 75 | BIOCARTA\_IL2\_PATHWAY |  | 22 | -0.26 | -0.75 | 0.828 | 0.918 | 1.000 | 4073 | tags=32%, list=21%, signal=40% |
| 76 | BIOCARTA\_HIVNEF\_PATHWAY |  | 55 | -0.22 | -0.74 | 0.915 | 0.919 | 1.000 | 4003 | tags=27%, list=20%, signal=34% |
| 77 | BIOCARTA\_TOLL\_PATHWAY |  | 34 | -0.23 | -0.70 | 0.912 | 0.949 | 1.000 | 1863 | tags=15%, list=10%, signal=16% |
| 78 | BIOCARTA\_CCR5\_PATHWAY |  | 15 | -0.25 | -0.64 | 0.914 | 0.996 | 1.000 | 2333 | tags=20%, list=12%, signal=23% |
| 79 | BIOCARTA\_FMLP\_PATHWAY |  | 34 | -0.20 | -0.62 | 0.976 | 1.000 | 1.000 | 3971 | tags=26%, list=20%, signal=33% |
| 80 | BIOCARTA\_CD40\_PATHWAY |  | 15 | -0.23 | -0.60 | 0.907 | 1.000 | 1.000 | 4440 | tags=27%, list=23%, signal=34% |
| 81 | BIOCARTA\_TALL1\_PATHWAY |  | 15 | -0.24 | -0.60 | 0.941 | 0.992 | 1.000 | 2173 | tags=13%, list=11%, signal=15% |
| 82 | BIOCARTA\_IGF1\_PATHWAY |  | 21 | -0.21 | -0.59 | 0.951 | 0.983 | 1.000 | 4073 | tags=19%, list=21%, signal=24% |
| 83 | BIOCARTA\_CARM\_ER\_PATHWAY |  | 32 | -0.19 | -0.58 | 0.974 | 0.976 | 1.000 | 166 | tags=3%, list=1%, signal=3% |
| 84 | BIOCARTA\_MTOR\_PATHWAY |  | 21 | -0.20 | -0.56 | 0.970 | 0.974 | 1.000 | 2909 | tags=19%, list=15%, signal=22% |
Table: Gene sets enriched in phenotype **b (3 samples)**[plain text format]****

  
