# Supplementary material for: Comprehensive analysis of β-catenin target genes in colorectal carcinoma cell lines with deregulated Wnt/β-catenin signaling
Source: BMC Genomics. 2014 Jan 28;15:74. doi: 10.1186/1471-2164-15-74 (PMC3909937; doi:10.1186/1471-2164-15-74)
Supplement: Additional file 4 — GSEA analysis using the Biocarta pathway database. This zipped file contains confirming data of the GSEA analysis. The names of the directories containing the files were composed of the term ‘GSEA’, the name of the cell line, e.g. DLD1, SW480, or LS174T, and the pathway database (Biocarta). Please use a web browser to view the files with the name ‘index.html’ in the corresponding directories to start exploring the data. [file 1471-2164-15-74-S4.zip › DLD1_Biocarta/gsea_report_for_bg_1355836171296.html]

Report for bg 1355836171296 [GSEA]

| GS  follow link to MSigDB | GS DETAILS | SIZE | ES | NES | NOM p-val | FDR q-val | FWER p-val | RANK AT MAX | LEADING EDGE || 1 | BIOCARTA\_ATRBRCA\_PATHWAY | Details ... | 21 | 0.69 | 1.96 | 0.002 | 0.022 | 0.030 | 4225 | tags=67%, list=22%, signal=85% |
| 2 | BIOCARTA\_ARF\_PATHWAY | Details ... | 17 | 0.61 | 1.67 | 0.007 | 0.267 | 0.531 | 2635 | tags=41%, list=13%, signal=48% |
| 3 | BIOCARTA\_WNT\_PATHWAY | Details ... | 25 | 0.53 | 1.55 | 0.025 | 0.495 | 0.869 | 109 | tags=12%, list=1%, signal=12% |
| 4 | BIOCARTA\_ATM\_PATHWAY | Details ... | 20 | 0.57 | 1.53 | 0.042 | 0.424 | 0.905 | 2676 | tags=40%, list=14%, signal=46% |
| 5 | BIOCARTA\_CTCF\_PATHWAY | Details ... | 22 | 0.52 | 1.46 | 0.046 | 0.600 | 0.986 | 2589 | tags=27%, list=13%, signal=31% |
| 6 | BIOCARTA\_PML\_PATHWAY | Details ... | 17 | 0.55 | 1.44 | 0.068 | 0.549 | 0.990 | 2470 | tags=29%, list=13%, signal=34% |
| 7 | BIOCARTA\_NKT\_PATHWAY | Details ... | 27 | 0.47 | 1.41 | 0.061 | 0.564 | 0.994 | 1587 | tags=19%, list=8%, signal=20% |
| 8 | BIOCARTA\_P53\_PATHWAY | Details ... | 16 | 0.55 | 1.41 | 0.066 | 0.497 | 0.994 | 2635 | tags=38%, list=13%, signal=43% |
| 9 | BIOCARTA\_IL17\_PATHWAY | Details ... | 16 | 0.52 | 1.37 | 0.104 | 0.551 | 0.997 | 2097 | tags=25%, list=11%, signal=28% |
| 10 | BIOCARTA\_INTRINSIC\_PATHWAY | Details ... | 23 | 0.48 | 1.37 | 0.087 | 0.507 | 0.998 | 3525 | tags=43%, list=18%, signal=53% |
| 11 | BIOCARTA\_TEL\_PATHWAY | Details ... | 18 | 0.50 | 1.37 | 0.093 | 0.461 | 0.998 | 2121 | tags=22%, list=11%, signal=25% |
| 12 | BIOCARTA\_G1\_PATHWAY | Details ... | 26 | 0.45 | 1.33 | 0.119 | 0.533 | 0.999 | 2660 | tags=42%, list=14%, signal=49% |
| 13 | BIOCARTA\_P38MAPK\_PATHWAY | Details ... | 39 | 0.41 | 1.32 | 0.086 | 0.528 | 1.000 | 171 | tags=10%, list=1%, signal=10% |
| 14 | BIOCARTA\_FAS\_PATHWAY | Details ... | 30 | 0.42 | 1.30 | 0.148 | 0.536 | 1.000 | 5201 | tags=43%, list=27%, signal=59% |
| 15 | BIOCARTA\_TOB1\_PATHWAY | Details ... | 20 | 0.47 | 1.27 | 0.176 | 0.576 | 1.000 | 2589 | tags=30%, list=13%, signal=35% |
| 16 | BIOCARTA\_IL12\_PATHWAY | Details ... | 21 | 0.44 | 1.23 | 0.191 | 0.643 | 1.000 | 3692 | tags=33%, list=19%, signal=41% |
| 17 | BIOCARTA\_G2\_PATHWAY | Details ... | 23 | 0.42 | 1.22 | 0.202 | 0.635 | 1.000 | 3456 | tags=35%, list=18%, signal=42% |
| 18 | BIOCARTA\_NTHI\_PATHWAY | Details ... | 23 | 0.42 | 1.19 | 0.222 | 0.686 | 1.000 | 2589 | tags=17%, list=13%, signal=20% |
| 19 | BIOCARTA\_P53HYPOXIA\_PATHWAY | Details ... | 22 | 0.42 | 1.18 | 0.243 | 0.681 | 1.000 | 877 | tags=14%, list=4%, signal=14% |
| 20 | BIOCARTA\_ACH\_PATHWAY | Details ... | 15 | 0.45 | 1.14 | 0.325 | 0.776 | 1.000 | 806 | tags=20%, list=4%, signal=21% |
| 21 | BIOCARTA\_RACCYCD\_PATHWAY |  | 26 | 0.36 | 1.06 | 0.373 | 0.976 | 1.000 | 1250 | tags=15%, list=6%, signal=16% |
| 22 | BIOCARTA\_FCER1\_PATHWAY |  | 37 | 0.33 | 1.05 | 0.384 | 0.946 | 1.000 | 3873 | tags=24%, list=20%, signal=30% |
| 23 | BIOCARTA\_GSK3\_PATHWAY |  | 27 | 0.35 | 1.04 | 0.439 | 0.957 | 1.000 | 99 | tags=7%, list=1%, signal=7% |
| 24 | BIOCARTA\_CARDIACEGF\_PATHWAY |  | 17 | 0.38 | 1.03 | 0.420 | 0.934 | 1.000 | 3678 | tags=35%, list=19%, signal=43% |
| 25 | BIOCARTA\_ETS\_PATHWAY |  | 18 | 0.38 | 1.02 | 0.450 | 0.935 | 1.000 | 6856 | tags=50%, list=35%, signal=77% |
| 26 | BIOCARTA\_LAIR\_PATHWAY |  | 16 | 0.39 | 1.02 | 0.453 | 0.907 | 1.000 | 4630 | tags=31%, list=24%, signal=41% |
| 27 | BIOCARTA\_CELLCYCLE\_PATHWAY |  | 22 | 0.34 | 0.96 | 0.515 | 1.000 | 1.000 | 1250 | tags=23%, list=6%, signal=24% |
| 28 | BIOCARTA\_SPPA\_PATHWAY |  | 19 | 0.35 | 0.94 | 0.533 | 1.000 | 1.000 | 19 | tags=5%, list=0%, signal=5% |
| 29 | BIOCARTA\_ALK\_PATHWAY |  | 36 | 0.30 | 0.94 | 0.555 | 1.000 | 1.000 | 4247 | tags=33%, list=22%, signal=43% |
| 30 | BIOCARTA\_TID\_PATHWAY |  | 19 | 0.33 | 0.92 | 0.585 | 1.000 | 1.000 | 1577 | tags=26%, list=8%, signal=29% |
| 31 | BIOCARTA\_INFLAM\_PATHWAY |  | 29 | 0.30 | 0.91 | 0.585 | 1.000 | 1.000 | 571 | tags=10%, list=3%, signal=11% |
| 32 | BIOCARTA\_NO2IL12\_PATHWAY |  | 15 | 0.34 | 0.88 | 0.618 | 1.000 | 1.000 | 6471 | tags=53%, list=33%, signal=80% |
| 33 | BIOCARTA\_HCMV\_PATHWAY |  | 16 | 0.32 | 0.85 | 0.683 | 1.000 | 1.000 | 171 | tags=6%, list=1%, signal=6% |
| 34 | BIOCARTA\_CDMAC\_PATHWAY |  | 15 | 0.33 | 0.85 | 0.664 | 1.000 | 1.000 | 109 | tags=7%, list=1%, signal=7% |
| 35 | BIOCARTA\_CYTOKINE\_PATHWAY |  | 21 | 0.29 | 0.84 | 0.700 | 1.000 | 1.000 | 571 | tags=10%, list=3%, signal=10% |
| 36 | BIOCARTA\_HDAC\_PATHWAY |  | 28 | 0.28 | 0.84 | 0.684 | 1.000 | 1.000 | 2121 | tags=14%, list=11%, signal=16% |
| 37 | BIOCARTA\_COMP\_PATHWAY |  | 15 | 0.31 | 0.82 | 0.695 | 1.000 | 1.000 | 5210 | tags=47%, list=27%, signal=64% |
| 38 | BIOCARTA\_RELA\_PATHWAY |  | 16 | 0.31 | 0.81 | 0.730 | 1.000 | 1.000 | 198 | tags=6%, list=1%, signal=6% |
| 39 | BIOCARTA\_STEM\_PATHWAY |  | 15 | 0.31 | 0.80 | 0.738 | 1.000 | 1.000 | 76 | tags=7%, list=0%, signal=7% |
| 40 | BIOCARTA\_MITOCHONDRIA\_PATHWAY |  | 18 | 0.30 | 0.79 | 0.738 | 1.000 | 1.000 | 4996 | tags=39%, list=26%, signal=52% |
| 41 | BIOCARTA\_TNFR2\_PATHWAY |  | 18 | 0.29 | 0.78 | 0.742 | 1.000 | 1.000 | 4932 | tags=33%, list=25%, signal=45% |
| 42 | BIOCARTA\_TNFR1\_PATHWAY |  | 29 | 0.25 | 0.76 | 0.825 | 1.000 | 1.000 | 4699 | tags=24%, list=24%, signal=32% |
| 43 | BIOCARTA\_TCR\_PATHWAY |  | 43 | 0.22 | 0.74 | 0.886 | 1.000 | 1.000 | 3873 | tags=16%, list=20%, signal=20% |
| 44 | BIOCARTA\_CASPASE\_PATHWAY |  | 22 | 0.26 | 0.73 | 0.850 | 1.000 | 1.000 | 4923 | tags=32%, list=25%, signal=42% |
| 45 | BIOCARTA\_NGF\_PATHWAY |  | 17 | 0.27 | 0.71 | 0.847 | 1.000 | 1.000 | 3873 | tags=24%, list=20%, signal=29% |
| 46 | BIOCARTA\_VEGF\_PATHWAY |  | 27 | 0.24 | 0.71 | 0.880 | 1.000 | 1.000 | 4471 | tags=26%, list=23%, signal=34% |
| 47 | BIOCARTA\_CTLA4\_PATHWAY |  | 20 | 0.25 | 0.71 | 0.872 | 1.000 | 1.000 | 2097 | tags=10%, list=11%, signal=11% |
| 48 | BIOCARTA\_HSP27\_PATHWAY |  | 15 | 0.28 | 0.71 | 0.850 | 0.989 | 1.000 | 1068 | tags=13%, list=5%, signal=14% |
| 49 | BIOCARTA\_PAR1\_PATHWAY |  | 35 | 0.21 | 0.70 | 0.912 | 0.978 | 1.000 | 770 | tags=6%, list=4%, signal=6% |
| 50 | BIOCARTA\_IGF1MTOR\_PATHWAY |  | 19 | 0.24 | 0.65 | 0.940 | 0.999 | 1.000 | 4310 | tags=21%, list=22%, signal=27% |
| 51 | BIOCARTA\_STRESS\_PATHWAY |  | 24 | 0.22 | 0.64 | 0.927 | 0.987 | 1.000 | 171 | tags=4%, list=1%, signal=4% |
| 52 | BIOCARTA\_BCR\_PATHWAY |  | 33 | 0.19 | 0.60 | 0.974 | 0.996 | 1.000 | 4817 | tags=24%, list=25%, signal=32% |
| 53 | BIOCARTA\_DC\_PATHWAY |  | 22 | 0.18 | 0.52 | 0.989 | 1.000 | 1.000 | 2368 | tags=14%, list=12%, signal=15% |
| 54 | BIOCARTA\_TH1TH2\_PATHWAY |  | 19 | 0.19 | 0.52 | 0.989 | 0.988 | 1.000 | 7039 | tags=37%, list=36%, signal=58% |
Table: Gene sets enriched in phenotype **bg (3 samples)**[plain text format]****

  
