# Supplementary material for: Comprehensive analysis of β-catenin target genes in colorectal carcinoma cell lines with deregulated Wnt/β-catenin signaling
Source: BMC Genomics. 2014 Jan 28;15:74. doi: 10.1186/1471-2164-15-74 (PMC3909937; doi:10.1186/1471-2164-15-74)
Supplement: Additional file 5 — GSEA analysis using the KEGG pathway database. This zipped file contains confirming data of the GSEA analysis. The names of the directories containing the files were composed of the term ‘GSEA’, the name of the cell line, e.g. DLD1, SW480, or LS174T, and the pathway database (KEGG). Please use a web browser to view the files with the name ‘index.html’ in the corresponding directories to start exploring the data. [file 1471-2164-15-74-S5.zip › GSEA KEGG SW480/gsea_report_for_0_1358419519343.html]

Report for 0 1358419519343 [GSEA]

| GS  follow link to MSigDB | GS DETAILS | SIZE | ES | NES | NOM p-val | FDR q-val | FWER p-val | RANK AT MAX | LEADING EDGE || 1 | KEGG\_SYSTEMIC\_LUPUS\_ERYTHEMATOSUS | Details ... | 103 | -0.58 | -2.25 | 0.000 | 0.000 | 0.000 | 3420 | tags=44%, list=17%, signal=53% |
| 2 | KEGG\_NEUROACTIVE\_LIGAND\_RECEPTOR\_INTERACTION | Details ... | 250 | -0.39 | -1.72 | 0.000 | 0.111 | 0.261 | 5793 | tags=49%, list=30%, signal=68% |
| 3 | KEGG\_AUTOIMMUNE\_THYROID\_DISEASE | Details ... | 48 | -0.50 | -1.70 | 0.003 | 0.106 | 0.358 | 3322 | tags=42%, list=17%, signal=50% |
| 4 | KEGG\_OLFACTORY\_TRANSDUCTION | Details ... | 112 | -0.43 | -1.69 | 0.003 | 0.086 | 0.379 | 6628 | tags=54%, list=34%, signal=82% |
| 5 | KEGG\_ASTHMA | Details ... | 27 | -0.55 | -1.65 | 0.009 | 0.097 | 0.488 | 4900 | tags=59%, list=25%, signal=79% |
| 6 | KEGG\_BASAL\_CELL\_CARCINOMA | Details ... | 53 | -0.47 | -1.65 | 0.004 | 0.082 | 0.498 | 4496 | tags=45%, list=23%, signal=59% |
| 7 | KEGG\_GLYCINE\_SERINE\_AND\_THREONINE\_METABOLISM | Details ... | 31 | -0.51 | -1.60 | 0.017 | 0.114 | 0.671 | 4495 | tags=45%, list=23%, signal=59% |
| 8 | KEGG\_ALLOGRAFT\_REJECTION | Details ... | 34 | -0.48 | -1.52 | 0.027 | 0.212 | 0.899 | 3322 | tags=38%, list=17%, signal=46% |
| 9 | KEGG\_CYSTEINE\_AND\_METHIONINE\_METABOLISM | Details ... | 30 | -0.49 | -1.51 | 0.045 | 0.191 | 0.904 | 4738 | tags=50%, list=24%, signal=66% |
| 10 | KEGG\_RENIN\_ANGIOTENSIN\_SYSTEM | Details ... | 16 | -0.57 | -1.51 | 0.049 | 0.186 | 0.923 | 5518 | tags=69%, list=28%, signal=96% |
| 11 | KEGG\_ALANINE\_ASPARTATE\_AND\_GLUTAMATE\_METABOLISM | Details ... | 31 | -0.48 | -1.49 | 0.031 | 0.186 | 0.936 | 2975 | tags=32%, list=15%, signal=38% |
| 12 | KEGG\_WNT\_SIGNALING\_PATHWAY | Details ... | 144 | -0.36 | -1.47 | 0.006 | 0.205 | 0.961 | 2228 | tags=17%, list=11%, signal=19% |
| 13 | KEGG\_NITROGEN\_METABOLISM | Details ... | 23 | -0.51 | -1.47 | 0.063 | 0.197 | 0.970 | 3959 | tags=43%, list=20%, signal=54% |
| 14 | KEGG\_HEMATOPOIETIC\_CELL\_LINEAGE | Details ... | 82 | -0.36 | -1.35 | 0.059 | 0.419 | 1.000 | 5656 | tags=46%, list=29%, signal=65% |
| 15 | KEGG\_REGULATION\_OF\_AUTOPHAGY | Details ... | 32 | -0.43 | -1.33 | 0.122 | 0.440 | 1.000 | 3300 | tags=31%, list=17%, signal=38% |
| 16 | KEGG\_HEDGEHOG\_SIGNALING\_PATHWAY | Details ... | 53 | -0.38 | -1.29 | 0.114 | 0.503 | 1.000 | 4496 | tags=38%, list=23%, signal=49% |
| 17 | KEGG\_CYTOKINE\_CYTOKINE\_RECEPTOR\_INTERACTION | Details ... | 243 | -0.29 | -1.29 | 0.035 | 0.485 | 1.000 | 5387 | tags=40%, list=28%, signal=55% |
| 18 | KEGG\_TYPE\_I\_DIABETES\_MELLITUS | Details ... | 40 | -0.38 | -1.26 | 0.142 | 0.565 | 1.000 | 5110 | tags=43%, list=26%, signal=57% |
| 19 | KEGG\_ARGININE\_AND\_PROLINE\_METABOLISM | Details ... | 51 | -0.36 | -1.25 | 0.152 | 0.566 | 1.000 | 3629 | tags=31%, list=19%, signal=38% |
| 20 | KEGG\_GRAFT\_VERSUS\_HOST\_DISEASE | Details ... | 36 | -0.39 | -1.24 | 0.167 | 0.544 | 1.000 | 4121 | tags=36%, list=21%, signal=46% |
| 21 | KEGG\_BETA\_ALANINE\_METABOLISM |  | 22 | -0.43 | -1.23 | 0.186 | 0.543 | 1.000 | 2912 | tags=32%, list=15%, signal=37% |
| 22 | KEGG\_MELANOGENESIS |  | 98 | -0.30 | -1.18 | 0.166 | 0.707 | 1.000 | 4935 | tags=32%, list=25%, signal=42% |
| 23 | KEGG\_PRIMARY\_IMMUNODEFICIENCY |  | 35 | -0.37 | -1.17 | 0.239 | 0.688 | 1.000 | 4339 | tags=34%, list=22%, signal=44% |
| 24 | KEGG\_BUTANOATE\_METABOLISM |  | 31 | -0.38 | -1.17 | 0.238 | 0.665 | 1.000 | 2045 | tags=19%, list=10%, signal=22% |
| 25 | KEGG\_LONG\_TERM\_DEPRESSION |  | 65 | -0.32 | -1.15 | 0.249 | 0.710 | 1.000 | 6389 | tags=38%, list=33%, signal=57% |
| 26 | KEGG\_PROXIMAL\_TUBULE\_BICARBONATE\_RECLAMATION |  | 23 | -0.39 | -1.14 | 0.275 | 0.715 | 1.000 | 3631 | tags=35%, list=19%, signal=43% |
| 27 | KEGG\_GLYCOSPHINGOLIPID\_BIOSYNTHESIS\_GANGLIO\_SERIES |  | 15 | -0.45 | -1.14 | 0.304 | 0.703 | 1.000 | 1247 | tags=27%, list=6%, signal=28% |
| 28 | KEGG\_MELANOMA |  | 71 | -0.31 | -1.13 | 0.229 | 0.682 | 1.000 | 757 | tags=10%, list=4%, signal=10% |
| 29 | KEGG\_PRIMARY\_BILE\_ACID\_BIOSYNTHESIS |  | 16 | -0.42 | -1.09 | 0.380 | 0.800 | 1.000 | 963 | tags=19%, list=5%, signal=20% |
| 30 | KEGG\_TYROSINE\_METABOLISM |  | 38 | -0.34 | -1.09 | 0.337 | 0.795 | 1.000 | 1370 | tags=18%, list=7%, signal=20% |
| 31 | KEGG\_GLYOXYLATE\_AND\_DICARBOXYLATE\_METABOLISM |  | 15 | -0.42 | -1.07 | 0.405 | 0.811 | 1.000 | 3303 | tags=27%, list=17%, signal=32% |
| 32 | KEGG\_NATURAL\_KILLER\_CELL\_MEDIATED\_CYTOTOXICITY |  | 125 | -0.27 | -1.07 | 0.301 | 0.787 | 1.000 | 3301 | tags=21%, list=17%, signal=25% |
| 33 | KEGG\_VALINE\_LEUCINE\_AND\_ISOLEUCINE\_DEGRADATION |  | 42 | -0.32 | -1.07 | 0.347 | 0.778 | 1.000 | 348 | tags=7%, list=2%, signal=7% |
| 34 | KEGG\_INTESTINAL\_IMMUNE\_NETWORK\_FOR\_IGA\_PRODUCTION |  | 45 | -0.32 | -1.06 | 0.345 | 0.793 | 1.000 | 5110 | tags=42%, list=26%, signal=57% |
| 35 | KEGG\_CALCIUM\_SIGNALING\_PATHWAY |  | 170 | -0.25 | -1.05 | 0.329 | 0.807 | 1.000 | 5437 | tags=39%, list=28%, signal=53% |
| 36 | KEGG\_TRYPTOPHAN\_METABOLISM |  | 37 | -0.32 | -1.04 | 0.379 | 0.795 | 1.000 | 3841 | tags=35%, list=20%, signal=44% |
| 37 | KEGG\_PANTOTHENATE\_AND\_COA\_BIOSYNTHESIS |  | 16 | -0.40 | -1.03 | 0.426 | 0.801 | 1.000 | 5587 | tags=44%, list=29%, signal=61% |
| 38 | KEGG\_PHENYLALANINE\_METABOLISM |  | 17 | -0.38 | -1.02 | 0.443 | 0.810 | 1.000 | 2181 | tags=29%, list=11%, signal=33% |
| 39 | KEGG\_TASTE\_TRANSDUCTION |  | 44 | -0.30 | -1.01 | 0.435 | 0.827 | 1.000 | 5138 | tags=43%, list=26%, signal=58% |
| 40 | KEGG\_JAK\_STAT\_SIGNALING\_PATHWAY |  | 149 | -0.24 | -1.00 | 0.441 | 0.836 | 1.000 | 3694 | tags=25%, list=19%, signal=30% |
| 41 | KEGG\_ABC\_TRANSPORTERS |  | 44 | -0.29 | -0.99 | 0.465 | 0.850 | 1.000 | 2873 | tags=27%, list=15%, signal=32% |
| 42 | KEGG\_DRUG\_METABOLISM\_CYTOCHROME\_P450 |  | 57 | -0.28 | -0.98 | 0.478 | 0.865 | 1.000 | 5601 | tags=39%, list=29%, signal=54% |
| 43 | KEGG\_ANTIGEN\_PROCESSING\_AND\_PRESENTATION |  | 77 | -0.26 | -0.97 | 0.513 | 0.867 | 1.000 | 3384 | tags=22%, list=17%, signal=27% |
| 44 | KEGG\_TERPENOID\_BACKBONE\_BIOSYNTHESIS |  | 15 | -0.37 | -0.97 | 0.502 | 0.860 | 1.000 | 2045 | tags=20%, list=10%, signal=22% |
| 45 | KEGG\_SELENOAMINO\_ACID\_METABOLISM |  | 21 | -0.34 | -0.97 | 0.506 | 0.849 | 1.000 | 6611 | tags=52%, list=34%, signal=79% |
| 46 | KEGG\_MATURITY\_ONSET\_DIABETES\_OF\_THE\_YOUNG |  | 19 | -0.34 | -0.95 | 0.512 | 0.867 | 1.000 | 2779 | tags=26%, list=14%, signal=31% |
| 47 | KEGG\_VIRAL\_MYOCARDITIS |  | 67 | -0.26 | -0.95 | 0.567 | 0.859 | 1.000 | 4604 | tags=28%, list=24%, signal=37% |
| 48 | KEGG\_GAP\_JUNCTION |  | 75 | -0.26 | -0.94 | 0.550 | 0.862 | 1.000 | 5138 | tags=32%, list=26%, signal=43% |
| 49 | KEGG\_EPITHELIAL\_CELL\_SIGNALING\_IN\_HELICOBACTER\_PYLORI\_INFECTION |  | 62 | -0.27 | -0.94 | 0.549 | 0.853 | 1.000 | 4748 | tags=26%, list=24%, signal=34% |
| 50 | KEGG\_RETINOL\_METABOLISM |  | 45 | -0.27 | -0.91 | 0.638 | 0.927 | 1.000 | 4917 | tags=42%, list=25%, signal=56% |
| 51 | KEGG\_RIBOSOME |  | 78 | -0.24 | -0.89 | 0.672 | 0.956 | 1.000 | 11857 | tags=83%, list=61%, signal=211% |
| 52 | KEGG\_PORPHYRIN\_AND\_CHLOROPHYLL\_METABOLISM |  | 28 | -0.29 | -0.89 | 0.616 | 0.941 | 1.000 | 4008 | tags=29%, list=20%, signal=36% |
| 53 | KEGG\_CYTOSOLIC\_DNA\_SENSING\_PATHWAY |  | 50 | -0.26 | -0.88 | 0.649 | 0.928 | 1.000 | 4228 | tags=28%, list=22%, signal=36% |
| 54 | KEGG\_ARACHIDONIC\_ACID\_METABOLISM |  | 50 | -0.26 | -0.88 | 0.662 | 0.914 | 1.000 | 6263 | tags=50%, list=32%, signal=73% |
| 55 | KEGG\_PROPANOATE\_METABOLISM |  | 31 | -0.28 | -0.88 | 0.635 | 0.898 | 1.000 | 379 | tags=6%, list=2%, signal=7% |
| 56 | KEGG\_PROTEIN\_EXPORT |  | 19 | -0.32 | -0.88 | 0.633 | 0.888 | 1.000 | 3162 | tags=16%, list=16%, signal=19% |
| 57 | KEGG\_CARDIAC\_MUSCLE\_CONTRACTION |  | 70 | -0.24 | -0.88 | 0.687 | 0.882 | 1.000 | 3790 | tags=21%, list=19%, signal=26% |
| 58 | KEGG\_STEROID\_BIOSYNTHESIS |  | 16 | -0.33 | -0.86 | 0.640 | 0.906 | 1.000 | 6168 | tags=38%, list=32%, signal=55% |
| 59 | KEGG\_GLUTATHIONE\_METABOLISM |  | 44 | -0.25 | -0.85 | 0.719 | 0.914 | 1.000 | 5732 | tags=34%, list=29%, signal=48% |
| 60 | KEGG\_GLYCOSPHINGOLIPID\_BIOSYNTHESIS\_LACTO\_AND\_NEOLACTO\_SERIES |  | 26 | -0.28 | -0.82 | 0.716 | 0.961 | 1.000 | 2271 | tags=23%, list=12%, signal=26% |
| 61 | KEGG\_T\_CELL\_RECEPTOR\_SIGNALING\_PATHWAY |  | 106 | -0.21 | -0.82 | 0.815 | 0.947 | 1.000 | 5191 | tags=26%, list=27%, signal=36% |
| 62 | KEGG\_CHEMOKINE\_SIGNALING\_PATHWAY |  | 172 | -0.19 | -0.80 | 0.923 | 0.974 | 1.000 | 5166 | tags=27%, list=26%, signal=37% |
| 63 | KEGG\_AMINOACYL\_TRNA\_BIOSYNTHESIS |  | 32 | -0.26 | -0.80 | 0.761 | 0.960 | 1.000 | 8560 | tags=47%, list=44%, signal=83% |
| 64 | KEGG\_VASCULAR\_SMOOTH\_MUSCLE\_CONTRACTION |  | 108 | -0.20 | -0.79 | 0.890 | 0.950 | 1.000 | 5138 | tags=31%, list=26%, signal=41% |
| 65 | KEGG\_GLYCOSAMINOGLYCAN\_BIOSYNTHESIS\_HEPARAN\_SULFATE |  | 25 | -0.26 | -0.78 | 0.799 | 0.952 | 1.000 | 4981 | tags=28%, list=25%, signal=38% |
| 66 | KEGG\_PURINE\_METABOLISM |  | 149 | -0.19 | -0.77 | 0.951 | 0.951 | 1.000 | 6690 | tags=38%, list=34%, signal=58% |
| 67 | KEGG\_RIBOFLAVIN\_METABOLISM |  | 16 | -0.29 | -0.77 | 0.766 | 0.948 | 1.000 | 4172 | tags=25%, list=21%, signal=32% |
| 68 | KEGG\_LINOLEIC\_ACID\_METABOLISM |  | 26 | -0.25 | -0.75 | 0.820 | 0.952 | 1.000 | 6033 | tags=50%, list=31%, signal=72% |
| 69 | KEGG\_GLYCOSAMINOGLYCAN\_DEGRADATION |  | 21 | -0.26 | -0.74 | 0.813 | 0.947 | 1.000 | 1708 | tags=14%, list=9%, signal=16% |
| 70 | KEGG\_MTOR\_SIGNALING\_PATHWAY |  | 46 | -0.22 | -0.72 | 0.894 | 0.957 | 1.000 | 2925 | tags=13%, list=15%, signal=15% |
| 71 | KEGG\_RNA\_POLYMERASE |  | 28 | -0.24 | -0.72 | 0.862 | 0.945 | 1.000 | 8861 | tags=54%, list=45%, signal=98% |
| 72 | KEGG\_ENDOMETRIAL\_CANCER |  | 51 | -0.20 | -0.68 | 0.952 | 0.970 | 1.000 | 2228 | tags=12%, list=11%, signal=13% |
| 73 | KEGG\_NICOTINATE\_AND\_NICOTINAMIDE\_METABOLISM |  | 21 | -0.24 | -0.68 | 0.896 | 0.958 | 1.000 | 5089 | tags=38%, list=26%, signal=51% |
| 74 | KEGG\_ALZHEIMERS\_DISEASE |  | 148 | -0.14 | -0.56 | 1.000 | 0.989 | 1.000 | 5567 | tags=20%, list=28%, signal=28% |
Table: Gene sets enriched in phenotype **0 (3 samples)**[plain text format]****

  
