# Supplementary material for: Comprehensive analysis of β-catenin target genes in colorectal carcinoma cell lines with deregulated Wnt/β-catenin signaling
Source: BMC Genomics. 2014 Jan 28;15:74. doi: 10.1186/1471-2164-15-74 (PMC3909937; doi:10.1186/1471-2164-15-74)
Supplement: Additional file 5 — GSEA analysis using the KEGG pathway database. This zipped file contains confirming data of the GSEA analysis. The names of the directories containing the files were composed of the term ‘GSEA’, the name of the cell line, e.g. DLD1, SW480, or LS174T, and the pathway database (KEGG). Please use a web browser to view the files with the name ‘index.html’ in the corresponding directories to start exploring the data. [file 1471-2164-15-74-S5.zip › GSEA KEGG SW480/gsea_report_for_1_1358419519343.html]

Report for 1 1358419519343 [GSEA]

| GS  follow link to MSigDB | GS DETAILS | SIZE | ES | NES | NOM p-val | FDR q-val | FWER p-val | RANK AT MAX | LEADING EDGE || 1 | KEGG\_APOPTOSIS | Details ... | 81 | 0.51 | 2.02 | 0.000 | 0.020 | 0.013 | 2096 | tags=31%, list=11%, signal=34% |
| 2 | KEGG\_FRUCTOSE\_AND\_MANNOSE\_METABOLISM | Details ... | 34 | 0.59 | 1.93 | 0.000 | 0.033 | 0.044 | 3541 | tags=41%, list=18%, signal=50% |
| 3 | KEGG\_DNA\_REPLICATION | Details ... | 34 | 0.58 | 1.90 | 0.003 | 0.035 | 0.071 | 5403 | tags=65%, list=28%, signal=89% |
| 4 | KEGG\_SMALL\_CELL\_LUNG\_CANCER | Details ... | 82 | 0.47 | 1.86 | 0.000 | 0.040 | 0.104 | 2487 | tags=29%, list=13%, signal=33% |
| 5 | KEGG\_ECM\_RECEPTOR\_INTERACTION | Details ... | 80 | 0.47 | 1.84 | 0.000 | 0.037 | 0.120 | 1473 | tags=20%, list=8%, signal=22% |
| 6 | KEGG\_PROTEASOME | Details ... | 42 | 0.53 | 1.80 | 0.003 | 0.047 | 0.178 | 6165 | tags=55%, list=32%, signal=80% |
| 7 | KEGG\_PATHOGENIC\_ESCHERICHIA\_COLI\_INFECTION | Details ... | 42 | 0.53 | 1.80 | 0.002 | 0.041 | 0.183 | 4578 | tags=48%, list=23%, signal=62% |
| 8 | KEGG\_FOCAL\_ADHESION | Details ... | 187 | 0.40 | 1.76 | 0.000 | 0.048 | 0.238 | 1473 | tags=16%, list=8%, signal=17% |
| 9 | KEGG\_NUCLEOTIDE\_EXCISION\_REPAIR | Details ... | 43 | 0.50 | 1.75 | 0.007 | 0.045 | 0.253 | 5403 | tags=63%, list=28%, signal=87% |
| 10 | KEGG\_COMPLEMENT\_AND\_COAGULATION\_CASCADES | Details ... | 65 | 0.46 | 1.74 | 0.000 | 0.046 | 0.277 | 797 | tags=17%, list=4%, signal=18% |
| 11 | KEGG\_MISMATCH\_REPAIR | Details ... | 22 | 0.57 | 1.70 | 0.023 | 0.063 | 0.386 | 4089 | tags=45%, list=21%, signal=57% |
| 12 | KEGG\_AMINO\_SUGAR\_AND\_NUCLEOTIDE\_SUGAR\_METABOLISM | Details ... | 44 | 0.50 | 1.69 | 0.007 | 0.064 | 0.414 | 3827 | tags=48%, list=20%, signal=59% |
| 13 | KEGG\_ENDOCYTOSIS | Details ... | 156 | 0.39 | 1.68 | 0.000 | 0.061 | 0.426 | 3377 | tags=29%, list=17%, signal=35% |
| 14 | KEGG\_UBIQUITIN\_MEDIATED\_PROTEOLYSIS | Details ... | 121 | 0.40 | 1.67 | 0.000 | 0.062 | 0.459 | 3572 | tags=30%, list=18%, signal=36% |
| 15 | KEGG\_LYSOSOME | Details ... | 114 | 0.41 | 1.66 | 0.000 | 0.063 | 0.488 | 3810 | tags=33%, list=19%, signal=41% |
| 16 | KEGG\_ETHER\_LIPID\_METABOLISM | Details ... | 27 | 0.51 | 1.62 | 0.024 | 0.083 | 0.610 | 1765 | tags=22%, list=9%, signal=24% |
| 17 | KEGG\_PANCREATIC\_CANCER | Details ... | 69 | 0.42 | 1.57 | 0.018 | 0.110 | 0.738 | 3863 | tags=35%, list=20%, signal=43% |
| 18 | KEGG\_GLYCEROLIPID\_METABOLISM | Details ... | 43 | 0.45 | 1.55 | 0.025 | 0.123 | 0.783 | 1581 | tags=23%, list=8%, signal=25% |
| 19 | KEGG\_HOMOLOGOUS\_RECOMBINATION | Details ... | 26 | 0.50 | 1.53 | 0.041 | 0.131 | 0.823 | 4219 | tags=42%, list=22%, signal=54% |
| 20 | KEGG\_HISTIDINE\_METABOLISM | Details ... | 27 | 0.48 | 1.53 | 0.043 | 0.131 | 0.834 | 3132 | tags=37%, list=16%, signal=44% |
| 21 | KEGG\_NON\_SMALL\_CELL\_LUNG\_CANCER |  | 52 | 0.43 | 1.50 | 0.010 | 0.146 | 0.875 | 3705 | tags=33%, list=19%, signal=40% |
| 22 | KEGG\_PEROXISOME |  | 71 | 0.39 | 1.49 | 0.020 | 0.150 | 0.897 | 4122 | tags=41%, list=21%, signal=52% |
| 23 | KEGG\_REGULATION\_OF\_ACTIN\_CYTOSKELETON |  | 196 | 0.34 | 1.49 | 0.000 | 0.145 | 0.898 | 3339 | tags=28%, list=17%, signal=33% |
| 24 | KEGG\_FC\_GAMMA\_R\_MEDIATED\_PHAGOCYTOSIS |  | 85 | 0.37 | 1.48 | 0.014 | 0.149 | 0.912 | 2317 | tags=21%, list=12%, signal=24% |
| 25 | KEGG\_BASE\_EXCISION\_REPAIR |  | 33 | 0.45 | 1.47 | 0.055 | 0.153 | 0.925 | 2306 | tags=24%, list=12%, signal=27% |
| 26 | KEGG\_STEROID\_HORMONE\_BIOSYNTHESIS |  | 42 | 0.42 | 1.45 | 0.044 | 0.169 | 0.947 | 1390 | tags=19%, list=7%, signal=20% |
| 27 | KEGG\_ADIPOCYTOKINE\_SIGNALING\_PATHWAY |  | 66 | 0.39 | 1.44 | 0.043 | 0.177 | 0.959 | 1873 | tags=18%, list=10%, signal=20% |
| 28 | KEGG\_PRION\_DISEASES |  | 35 | 0.42 | 1.42 | 0.076 | 0.188 | 0.966 | 2654 | tags=26%, list=14%, signal=30% |
| 29 | KEGG\_ADHERENS\_JUNCTION |  | 73 | 0.36 | 1.40 | 0.042 | 0.211 | 0.987 | 3766 | tags=32%, list=19%, signal=39% |
| 30 | KEGG\_THYROID\_CANCER |  | 29 | 0.44 | 1.39 | 0.089 | 0.218 | 0.992 | 2654 | tags=28%, list=14%, signal=32% |
| 31 | KEGG\_INSULIN\_SIGNALING\_PATHWAY |  | 130 | 0.32 | 1.38 | 0.030 | 0.222 | 0.994 | 4394 | tags=29%, list=22%, signal=37% |
| 32 | KEGG\_ALDOSTERONE\_REGULATED\_SODIUM\_REABSORPTION |  | 40 | 0.40 | 1.35 | 0.076 | 0.245 | 0.998 | 1907 | tags=20%, list=10%, signal=22% |
| 33 | KEGG\_NEUROTROPHIN\_SIGNALING\_PATHWAY |  | 121 | 0.32 | 1.35 | 0.029 | 0.248 | 0.998 | 4341 | tags=34%, list=22%, signal=43% |
| 34 | KEGG\_GLIOMA |  | 63 | 0.36 | 1.34 | 0.068 | 0.249 | 0.998 | 3706 | tags=37%, list=19%, signal=45% |
| 35 | KEGG\_GLYCOSAMINOGLYCAN\_BIOSYNTHESIS\_KERATAN\_SULFATE |  | 15 | 0.51 | 1.34 | 0.132 | 0.246 | 0.998 | 3552 | tags=53%, list=18%, signal=65% |
| 36 | KEGG\_PROSTATE\_CANCER |  | 87 | 0.33 | 1.33 | 0.069 | 0.245 | 0.998 | 3525 | tags=28%, list=18%, signal=34% |
| 37 | KEGG\_NOD\_LIKE\_RECEPTOR\_SIGNALING\_PATHWAY |  | 51 | 0.37 | 1.33 | 0.080 | 0.243 | 0.998 | 1609 | tags=20%, list=8%, signal=21% |
| 38 | KEGG\_GLYCOSYLPHOSPHATIDYLINOSITOL\_GPI\_ANCHOR\_BIOSYNTHESIS |  | 24 | 0.43 | 1.31 | 0.148 | 0.272 | 1.000 | 3177 | tags=42%, list=16%, signal=50% |
| 39 | KEGG\_VEGF\_SIGNALING\_PATHWAY |  | 70 | 0.34 | 1.29 | 0.057 | 0.281 | 1.000 | 3251 | tags=26%, list=17%, signal=31% |
| 40 | KEGG\_HYPERTROPHIC\_CARDIOMYOPATHY\_HCM |  | 82 | 0.33 | 1.28 | 0.084 | 0.291 | 1.000 | 2483 | tags=21%, list=13%, signal=24% |
| 41 | KEGG\_O\_GLYCAN\_BIOSYNTHESIS |  | 28 | 0.42 | 1.28 | 0.134 | 0.295 | 1.000 | 2532 | tags=32%, list=13%, signal=37% |
| 42 | KEGG\_DILATED\_CARDIOMYOPATHY |  | 89 | 0.32 | 1.27 | 0.108 | 0.299 | 1.000 | 2483 | tags=18%, list=13%, signal=20% |
| 43 | KEGG\_RENAL\_CELL\_CARCINOMA |  | 68 | 0.34 | 1.27 | 0.094 | 0.293 | 1.000 | 3525 | tags=31%, list=18%, signal=38% |
| 44 | KEGG\_CELL\_ADHESION\_MOLECULES\_CAMS |  | 124 | 0.30 | 1.26 | 0.063 | 0.308 | 1.000 | 1341 | tags=11%, list=7%, signal=12% |
| 45 | KEGG\_CHRONIC\_MYELOID\_LEUKEMIA |  | 72 | 0.32 | 1.22 | 0.147 | 0.358 | 1.000 | 3995 | tags=31%, list=20%, signal=38% |
| 46 | KEGG\_PENTOSE\_AND\_GLUCURONATE\_INTERCONVERSIONS |  | 15 | 0.44 | 1.22 | 0.225 | 0.368 | 1.000 | 2620 | tags=40%, list=13%, signal=46% |
| 47 | KEGG\_BLADDER\_CANCER |  | 40 | 0.36 | 1.21 | 0.197 | 0.377 | 1.000 | 2654 | tags=20%, list=14%, signal=23% |
| 48 | KEGG\_PPAR\_SIGNALING\_PATHWAY |  | 66 | 0.32 | 1.20 | 0.146 | 0.377 | 1.000 | 1915 | tags=20%, list=10%, signal=22% |
| 49 | KEGG\_PYRIMIDINE\_METABOLISM |  | 88 | 0.30 | 1.20 | 0.156 | 0.375 | 1.000 | 4493 | tags=38%, list=23%, signal=48% |
| 50 | KEGG\_AXON\_GUIDANCE |  | 127 | 0.28 | 1.19 | 0.144 | 0.377 | 1.000 | 2758 | tags=20%, list=14%, signal=24% |
| 51 | KEGG\_PATHWAYS\_IN\_CANCER |  | 315 | 0.25 | 1.19 | 0.066 | 0.381 | 1.000 | 2487 | tags=19%, list=13%, signal=22% |
| 52 | KEGG\_ARRHYTHMOGENIC\_RIGHT\_VENTRICULAR\_CARDIOMYOPATHY\_ARVC |  | 73 | 0.31 | 1.18 | 0.175 | 0.396 | 1.000 | 2483 | tags=18%, list=13%, signal=20% |
| 53 | KEGG\_GLYCOLYSIS\_GLUCONEOGENESIS |  | 60 | 0.32 | 1.17 | 0.213 | 0.395 | 1.000 | 3540 | tags=27%, list=18%, signal=32% |
| 54 | KEGG\_OOCYTE\_MEIOSIS |  | 106 | 0.29 | 1.17 | 0.187 | 0.390 | 1.000 | 4328 | tags=33%, list=22%, signal=42% |
| 55 | KEGG\_ERBB\_SIGNALING\_PATHWAY |  | 85 | 0.30 | 1.16 | 0.199 | 0.401 | 1.000 | 3995 | tags=29%, list=20%, signal=37% |
| 56 | KEGG\_VIBRIO\_CHOLERAE\_INFECTION |  | 50 | 0.32 | 1.16 | 0.223 | 0.407 | 1.000 | 1907 | tags=16%, list=10%, signal=18% |
| 57 | KEGG\_COLORECTAL\_CANCER |  | 61 | 0.32 | 1.16 | 0.224 | 0.402 | 1.000 | 2414 | tags=21%, list=12%, signal=24% |
| 58 | KEGG\_GALACTOSE\_METABOLISM |  | 25 | 0.37 | 1.15 | 0.267 | 0.398 | 1.000 | 3965 | tags=44%, list=20%, signal=55% |
| 59 | KEGG\_MAPK\_SIGNALING\_PATHWAY |  | 253 | 0.25 | 1.14 | 0.171 | 0.426 | 1.000 | 2004 | tags=18%, list=10%, signal=20% |
| 60 | KEGG\_TYPE\_II\_DIABETES\_MELLITUS |  | 44 | 0.33 | 1.13 | 0.244 | 0.422 | 1.000 | 860 | tags=7%, list=4%, signal=7% |
| 61 | KEGG\_LEUKOCYTE\_TRANSENDOTHELIAL\_MIGRATION |  | 105 | 0.28 | 1.13 | 0.238 | 0.426 | 1.000 | 3795 | tags=28%, list=19%, signal=34% |
| 62 | KEGG\_GLYCEROPHOSPHOLIPID\_METABOLISM |  | 63 | 0.29 | 1.10 | 0.273 | 0.473 | 1.000 | 1765 | tags=17%, list=9%, signal=19% |
| 63 | KEGG\_P53\_SIGNALING\_PATHWAY |  | 64 | 0.29 | 1.10 | 0.279 | 0.475 | 1.000 | 3698 | tags=31%, list=19%, signal=38% |
| 64 | KEGG\_INOSITOL\_PHOSPHATE\_METABOLISM |  | 46 | 0.31 | 1.10 | 0.311 | 0.468 | 1.000 | 1484 | tags=15%, list=8%, signal=16% |
| 65 | KEGG\_DORSO\_VENTRAL\_AXIS\_FORMATION |  | 23 | 0.36 | 1.09 | 0.347 | 0.470 | 1.000 | 1429 | tags=22%, list=7%, signal=23% |
| 66 | KEGG\_BASAL\_TRANSCRIPTION\_FACTORS |  | 33 | 0.34 | 1.08 | 0.321 | 0.489 | 1.000 | 4540 | tags=42%, list=23%, signal=55% |
| 67 | KEGG\_SNARE\_INTERACTIONS\_IN\_VESICULAR\_TRANSPORT |  | 34 | 0.33 | 1.07 | 0.338 | 0.500 | 1.000 | 4136 | tags=35%, list=21%, signal=45% |
| 68 | KEGG\_TIGHT\_JUNCTION |  | 124 | 0.25 | 1.07 | 0.309 | 0.494 | 1.000 | 2050 | tags=16%, list=10%, signal=18% |
| 69 | KEGG\_PHOSPHATIDYLINOSITOL\_SIGNALING\_SYSTEM |  | 67 | 0.27 | 1.04 | 0.373 | 0.553 | 1.000 | 4135 | tags=33%, list=21%, signal=41% |
| 70 | KEGG\_PENTOSE\_PHOSPHATE\_PATHWAY |  | 26 | 0.34 | 1.03 | 0.387 | 0.563 | 1.000 | 3540 | tags=50%, list=18%, signal=61% |
| 71 | KEGG\_DRUG\_METABOLISM\_OTHER\_ENZYMES |  | 36 | 0.30 | 1.01 | 0.445 | 0.617 | 1.000 | 2620 | tags=25%, list=13%, signal=29% |
| 72 | KEGG\_SPHINGOLIPID\_METABOLISM |  | 30 | 0.31 | 0.99 | 0.483 | 0.662 | 1.000 | 1598 | tags=20%, list=8%, signal=22% |
| 73 | KEGG\_TGF\_BETA\_SIGNALING\_PATHWAY |  | 82 | 0.25 | 0.98 | 0.495 | 0.678 | 1.000 | 1608 | tags=12%, list=8%, signal=13% |
| 74 | KEGG\_LYSINE\_DEGRADATION |  | 43 | 0.28 | 0.96 | 0.502 | 0.709 | 1.000 | 3300 | tags=26%, list=17%, signal=31% |
| 75 | KEGG\_NOTCH\_SIGNALING\_PATHWAY |  | 41 | 0.28 | 0.96 | 0.518 | 0.706 | 1.000 | 733 | tags=10%, list=4%, signal=10% |
| 76 | KEGG\_TOLL\_LIKE\_RECEPTOR\_SIGNALING\_PATHWAY |  | 96 | 0.24 | 0.96 | 0.546 | 0.703 | 1.000 | 3360 | tags=22%, list=17%, signal=26% |
| 77 | KEGG\_LONG\_TERM\_POTENTIATION |  | 67 | 0.25 | 0.95 | 0.533 | 0.697 | 1.000 | 4328 | tags=36%, list=22%, signal=46% |
| 78 | KEGG\_RIG\_I\_LIKE\_RECEPTOR\_SIGNALING\_PATHWAY |  | 64 | 0.25 | 0.93 | 0.568 | 0.737 | 1.000 | 3360 | tags=25%, list=17%, signal=30% |
| 79 | KEGG\_CELL\_CYCLE |  | 113 | 0.23 | 0.93 | 0.605 | 0.746 | 1.000 | 3995 | tags=28%, list=20%, signal=35% |
| 80 | KEGG\_FC\_EPSILON\_RI\_SIGNALING\_PATHWAY |  | 73 | 0.24 | 0.89 | 0.636 | 0.821 | 1.000 | 2118 | tags=15%, list=11%, signal=17% |
| 81 | KEGG\_GNRH\_SIGNALING\_PATHWAY |  | 96 | 0.22 | 0.89 | 0.684 | 0.822 | 1.000 | 3706 | tags=25%, list=19%, signal=31% |
| 82 | KEGG\_RNA\_DEGRADATION |  | 51 | 0.25 | 0.87 | 0.689 | 0.850 | 1.000 | 5913 | tags=45%, list=30%, signal=64% |
| 83 | KEGG\_BIOSYNTHESIS\_OF\_UNSATURATED\_FATTY\_ACIDS |  | 19 | 0.31 | 0.86 | 0.640 | 0.853 | 1.000 | 5414 | tags=47%, list=28%, signal=65% |
| 84 | KEGG\_B\_CELL\_RECEPTOR\_SIGNALING\_PATHWAY |  | 71 | 0.23 | 0.86 | 0.704 | 0.846 | 1.000 | 2118 | tags=17%, list=11%, signal=19% |
| 85 | KEGG\_METABOLISM\_OF\_XENOBIOTICS\_BY\_CYTOCHROME\_P450 |  | 56 | 0.24 | 0.85 | 0.734 | 0.858 | 1.000 | 2753 | tags=21%, list=14%, signal=25% |
| 86 | KEGG\_AMYOTROPHIC\_LATERAL\_SCLEROSIS\_ALS |  | 49 | 0.24 | 0.85 | 0.736 | 0.856 | 1.000 | 1978 | tags=18%, list=10%, signal=20% |
| 87 | KEGG\_LEISHMANIA\_INFECTION |  | 62 | 0.23 | 0.85 | 0.749 | 0.853 | 1.000 | 3874 | tags=31%, list=20%, signal=38% |
| 88 | KEGG\_ONE\_CARBON\_POOL\_BY\_FOLATE |  | 16 | 0.31 | 0.84 | 0.698 | 0.861 | 1.000 | 4892 | tags=56%, list=25%, signal=75% |
| 89 | KEGG\_ACUTE\_MYELOID\_LEUKEMIA |  | 56 | 0.22 | 0.79 | 0.832 | 0.940 | 1.000 | 1570 | tags=13%, list=8%, signal=14% |
| 90 | KEGG\_ALPHA\_LINOLENIC\_ACID\_METABOLISM |  | 16 | 0.28 | 0.77 | 0.783 | 0.962 | 1.000 | 1651 | tags=13%, list=8%, signal=14% |
| 91 | KEGG\_FATTY\_ACID\_METABOLISM |  | 39 | 0.23 | 0.76 | 0.848 | 0.959 | 1.000 | 3388 | tags=28%, list=17%, signal=34% |
| 92 | KEGG\_SPLICEOSOME |  | 93 | 0.19 | 0.76 | 0.940 | 0.959 | 1.000 | 15872 | tags=100%, list=81%, signal=528% |
| 93 | KEGG\_N\_GLYCAN\_BIOSYNTHESIS |  | 41 | 0.22 | 0.75 | 0.875 | 0.955 | 1.000 | 4509 | tags=32%, list=23%, signal=41% |
| 94 | KEGG\_VASOPRESSIN\_REGULATED\_WATER\_REABSORPTION |  | 44 | 0.22 | 0.75 | 0.851 | 0.946 | 1.000 | 3687 | tags=23%, list=19%, signal=28% |
| 95 | KEGG\_PYRUVATE\_METABOLISM |  | 40 | 0.22 | 0.73 | 0.912 | 0.959 | 1.000 | 1416 | tags=10%, list=7%, signal=11% |
| 96 | KEGG\_STARCH\_AND\_SUCROSE\_METABOLISM |  | 34 | 0.20 | 0.66 | 0.915 | 1.000 | 1.000 | 2620 | tags=26%, list=13%, signal=31% |
| 97 | KEGG\_PROGESTERONE\_MEDIATED\_OOCYTE\_MATURATION |  | 82 | 0.16 | 0.62 | 0.993 | 1.000 | 1.000 | 4307 | tags=24%, list=22%, signal=31% |
| 98 | KEGG\_PARKINSONS\_DISEASE |  | 105 | 0.14 | 0.58 | 1.000 | 1.000 | 1.000 | 1680 | tags=5%, list=9%, signal=5% |
| 99 | KEGG\_OXIDATIVE\_PHOSPHORYLATION |  | 109 | 0.12 | 0.50 | 1.000 | 1.000 | 1.000 | 6344 | tags=44%, list=32%, signal=65% |
| 100 | KEGG\_CITRATE\_CYCLE\_TCA\_CYCLE |  | 29 | 0.16 | 0.50 | 0.993 | 1.000 | 1.000 | 3793 | tags=21%, list=19%, signal=26% |
| 101 | KEGG\_HUNTINGTONS\_DISEASE |  | 163 | 0.10 | 0.45 | 1.000 | 0.999 | 1.000 | 5190 | tags=23%, list=27%, signal=31% |
Table: Gene sets enriched in phenotype **1 (3 samples)**[plain text format]****

  
