# Supplementary material for: Comprehensive analysis of β-catenin target genes in colorectal carcinoma cell lines with deregulated Wnt/β-catenin signaling
Source: BMC Genomics. 2014 Jan 28;15:74. doi: 10.1186/1471-2164-15-74 (PMC3909937; doi:10.1186/1471-2164-15-74)
Supplement: Additional file 5 — GSEA analysis using the KEGG pathway database. This zipped file contains confirming data of the GSEA analysis. The names of the directories containing the files were composed of the term ‘GSEA’, the name of the cell line, e.g. DLD1, SW480, or LS174T, and the pathway database (KEGG). Please use a web browser to view the files with the name ‘index.html’ in the corresponding directories to start exploring the data. [file 1471-2164-15-74-S5.zip › GSEA KEGG SW480/KEGG_ALANINE_ASPARTATE_AND_GLUTAMATE_METABOLISM.html]

Details for gene set KEGG\_ALANINE\_ASPARTATE\_AND\_GLUTAMATE\_METABOLISM[GSEA]

|  || Dataset | SW480\_collapsed\_to\_symbols.class.cls#b\_versus\_bg.class.cls#b\_versus\_bg\_repos |
| Phenotype | class.cls#b\_versus\_bg\_repos |
| Upregulated in class | 0 |
| GeneSet | KEGG\_ALANINE\_ASPARTATE\_AND\_GLUTAMATE\_METABOLISM |
| Enrichment Score (ES) | -0.48417723 |
| Normalized Enrichment Score (NES) | -1.4940019 |
| Nominal p-value | 0.031141868 |
| FDR q-value | 0.18572994 |
| FWER p-Value | 0.936 |
Table: GSEA Results Summary

  

Fig 1: Enrichment plot: KEGG\_ALANINE\_ASPARTATE\_AND\_GLUTAMATE\_METABOLISM      
 Profile of the Running ES Score & Positions of GeneSet Members on the Rank Ordered List

  

| PROBE | GENE SYMBOL | GENE\_TITLE | RANK IN GENE LIST | RANK METRIC SCORE | RUNNING ES | CORE ENRICHMENT || 1 | ASL | ASL Entrez,  Source | argininosuccinate lyase | 563 | 0.258 | 0.0334 | No |
| 2 | GOT1 | GOT1 Entrez,  Source | glutamic-oxaloacetic transaminase 1, soluble (aspartate aminotransferase 1) | 1517 | 0.147 | 0.0201 | No |
| 3 | ACY3 | ACY3 Entrez,  Source | aspartoacylase (aminocyclase) 3 | 1616 | 0.139 | 0.0486 | No |
| 4 | GLS | GLS Entrez,  Source | glutaminase | 2685 | 0.092 | 0.0163 | No |
| 5 | ADSS | ADSS Entrez,  Source | adenylosuccinate synthase | 3876 | 0.058 | -0.0305 | No |
| 6 | NIT2 | NIT2 Entrez,  Source | nitrilase family, member 2 | 3988 | 0.056 | -0.0227 | No |
| 7 | AGXT | AGXT Entrez,  Source | alanine-glyoxylate aminotransferase (oxalosis I; hyperoxaluria I; glycolicaciduria; serine-pyruvate aminotransferase) | 6269 | 0.016 | -0.1357 | No |
| 8 | GPT | GPT Entrez,  Source | glutamic-pyruvate transaminase (alanine aminotransferase) | 7952 | -0.006 | -0.2203 | No |
| 9 | GLUL | GLUL Entrez,  Source | glutamate-ammonia ligase (glutamine synthetase) | 7969 | -0.007 | -0.2195 | No |
| 10 | ALDH5A1 | ALDH5A1 Entrez,  Source | aldehyde dehydrogenase 5 family, member A1 (succinate-semialdehyde dehydrogenase) | 8150 | -0.009 | -0.2266 | No |
| 11 | DDO | DDO Entrez,  Source | D-aspartate oxidase | 9367 | -0.023 | -0.2834 | No |
| 12 | ALDH4A1 | ALDH4A1 Entrez,  Source | aldehyde dehydrogenase 4 family, member A1 | 9929 | -0.029 | -0.3051 | No |
| 13 | ADSSL1 | ADSSL1 Entrez,  Source | adenylosuccinate synthase like 1 | 11010 | -0.042 | -0.3502 | No |
| 14 | GOT2 | GOT2 Entrez,  Source | glutamic-oxaloacetic transaminase 2, mitochondrial (aspartate aminotransferase 2) | 11404 | -0.047 | -0.3589 | No |
| 15 | CPS1 | CPS1 Entrez,  Source | carbamoyl-phosphate synthetase 1, mitochondrial | 11677 | -0.050 | -0.3607 | No |
| 16 | ADSL | ADSL Entrez,  Source | adenylosuccinate lyase | 13247 | -0.070 | -0.4242 | No |
| 17 | CAD | CAD Entrez,  Source | carbamoyl-phosphate synthetase 2, aspartate transcarbamylase, and dihydroorotase | 13590 | -0.074 | -0.4238 | No |
| 18 | GFPT1 | GFPT1 Entrez,  Source | glutamine-fructose-6-phosphate transaminase 1 | 14458 | -0.086 | -0.4475 | No |
| 19 | GAD2 | GAD2 Entrez,  Source | glutamate decarboxylase 2 (pancreatic islets and brain, 65kDa) | 14849 | -0.091 | -0.4454 | No |
| 20 | GPT2 | GPT2 Entrez,  Source | glutamic pyruvate transaminase (alanine aminotransferase) 2 | 15214 | -0.098 | -0.4405 | No |
| 21 | AGXT2 | AGXT2 Entrez,  Source | alanine-glyoxylate aminotransferase 2 | 15866 | -0.109 | -0.4475 | No |
| 22 | GLUD2 | GLUD2 Entrez,  Source | glutamate dehydrogenase 2 | 16583 | -0.122 | -0.4546 | Yes |
| 23 | PPAT | PPAT Entrez,  Source | phosphoribosyl pyrophosphate amidotransferase | 16724 | -0.126 | -0.4313 | Yes |
| 24 | ASPA | ASPA Entrez,  Source | aspartoacylase (Canavan disease) | 17201 | -0.139 | -0.4222 | Yes |
| 25 | GLUD1 | GLUD1 Entrez,  Source | glutamate dehydrogenase 1 | 17237 | -0.140 | -0.3902 | Yes |
| 26 | ASNS | ASNS Entrez,  Source | asparagine synthetase | 18792 | -0.216 | -0.4175 | Yes |
| 27 | ASS1 | ASS1 Entrez,  Source | argininosuccinate synthetase 1 | 19141 | -0.271 | -0.3699 | Yes |
| 28 | GFPT2 | GFPT2 Entrez,  Source | glutamine-fructose-6-phosphate transaminase 2 | 19235 | -0.298 | -0.3025 | Yes |
| 29 | GAD1 | GAD1 Entrez,  Source | glutamate decarboxylase 1 (brain, 67kDa) | 19253 | -0.305 | -0.2296 | Yes |
| 30 | GLS2 | GLS2 Entrez,  Source | glutaminase 2 (liver, mitochondrial) | 19265 | -0.312 | -0.1546 | Yes |
| 31 | ABAT | ABAT Entrez,  Source | 4-aminobutyrate aminotransferase | 19529 | -0.701 | 0.0014 | Yes |
Table: GSEA details [plain text format]

  

Fig 2: KEGG\_ALANINE\_ASPARTATE\_AND\_GLUTAMATE\_METABOLISM      
 Blue-Pink O' Gram in the Space of the Analyzed GeneSet

  

Fig 3: KEGG\_ALANINE\_ASPARTATE\_AND\_GLUTAMATE\_METABOLISM: Random ES distribution      
 Gene set null distribution of ES for **KEGG\_ALANINE\_ASPARTATE\_AND\_GLUTAMATE\_METABOLISM**

  
