# Supplementary material for: Comprehensive analysis of β-catenin target genes in colorectal carcinoma cell lines with deregulated Wnt/β-catenin signaling
Source: BMC Genomics. 2014 Jan 28;15:74. doi: 10.1186/1471-2164-15-74 (PMC3909937; doi:10.1186/1471-2164-15-74)
Supplement: Additional file 5 — GSEA analysis using the KEGG pathway database. This zipped file contains confirming data of the GSEA analysis. The names of the directories containing the files were composed of the term ‘GSEA’, the name of the cell line, e.g. DLD1, SW480, or LS174T, and the pathway database (KEGG). Please use a web browser to view the files with the name ‘index.html’ in the corresponding directories to start exploring the data. [file 1471-2164-15-74-S5.zip › GSEA KEGG SW480/KEGG_ALLOGRAFT_REJECTION.html]

Details for gene set KEGG\_ALLOGRAFT\_REJECTION[GSEA]

|  || Dataset | SW480\_collapsed\_to\_symbols.class.cls#b\_versus\_bg.class.cls#b\_versus\_bg\_repos |
| Phenotype | class.cls#b\_versus\_bg\_repos |
| Upregulated in class | 0 |
| GeneSet | KEGG\_ALLOGRAFT\_REJECTION |
| Enrichment Score (ES) | -0.48358285 |
| Normalized Enrichment Score (NES) | -1.5165803 |
| Nominal p-value | 0.026595745 |
| FDR q-value | 0.21153134 |
| FWER p-Value | 0.899 |
Table: GSEA Results Summary

  

Fig 1: Enrichment plot: KEGG\_ALLOGRAFT\_REJECTION      
 Profile of the Running ES Score & Positions of GeneSet Members on the Rank Ordered List

  

| PROBE | GENE SYMBOL | GENE\_TITLE | RANK IN GENE LIST | RANK METRIC SCORE | RUNNING ES | CORE ENRICHMENT || 1 | HLA-A | HLA-A Entrez,  Source | major histocompatibility complex, class I, A | 1218 | 0.168 | -0.0191 | No |
| 2 | HLA-F | HLA-F Entrez,  Source | major histocompatibility complex, class I, F | 1341 | 0.159 | 0.0155 | No |
| 3 | HLA-DMA | HLA-DMA Entrez,  Source | major histocompatibility complex, class II, DM alpha | 2247 | 0.110 | -0.0027 | No |
| 4 | HLA-G | HLA-G Entrez,  Source | HLA-G histocompatibility antigen, class I, G | 2852 | 0.087 | -0.0113 | No |
| 5 | HLA-DRB4 | HLA-DRB4 Entrez,  Source | major histocompatibility complex, class II, DR beta 4 | 3022 | 0.082 | 0.0011 | No |
| 6 | HLA-C | HLA-C Entrez,  Source | major histocompatibility complex, class I, C | 3264 | 0.074 | 0.0079 | No |
| 7 | HLA-B | HLA-B Entrez,  Source | major histocompatibility complex, class I, B | 3267 | 0.074 | 0.0269 | No |
| 8 | HLA-DRA | HLA-DRA Entrez,  Source | major histocompatibility complex, class II, DR alpha | 3456 | 0.069 | 0.0350 | No |
| 9 | FAS | FAS Entrez,  Source | Fas (TNF receptor superfamily, member 6) | 3978 | 0.056 | 0.0228 | No |
| 10 | CD86 | CD86 Entrez,  Source | CD86 molecule | 6293 | 0.015 | -0.0919 | No |
| 11 | HLA-E | HLA-E Entrez,  Source | major histocompatibility complex, class I, E | 6630 | 0.011 | -0.1063 | No |
| 12 | IFNG | IFNG Entrez,  Source | interferon, gamma | 6964 | 0.006 | -0.1217 | No |
| 13 | HLA-DPA1 | HLA-DPA1 Entrez,  Source | major histocompatibility complex, class II, DP alpha 1 | 7007 | 0.006 | -0.1224 | No |
| 14 | IL12B | IL12B Entrez,  Source | interleukin 12B (natural killer cell stimulatory factor 2, cytotoxic lymphocyte maturation factor 2, p40) | 7747 | -0.004 | -0.1593 | No |
| 15 | HLA-DQA1 | HLA-DQA1 Entrez,  Source | major histocompatibility complex, class II, DQ alpha 1 | 8795 | -0.016 | -0.2088 | No |
| 16 | CD28 | CD28 Entrez,  Source | CD28 molecule | 9542 | -0.025 | -0.2406 | No |
| 17 | HLA-DOA | HLA-DOA Entrez,  Source | major histocompatibility complex, class II, DO alpha | 11429 | -0.047 | -0.3251 | No |
| 18 | CD40 | CD40 Entrez,  Source | CD40 molecule, TNF receptor superfamily member 5 | 12929 | -0.066 | -0.3850 | No |
| 19 | IL2 | IL2 Entrez,  Source | interleukin 2 | 14448 | -0.085 | -0.4408 | No |
| 20 | CD40LG | CD40LG Entrez,  Source | CD40 ligand (TNF superfamily, member 5, hyper-IgM syndrome) | 14658 | -0.088 | -0.4288 | No |
| 21 | HLA-DPB1 | HLA-DPB1 Entrez,  Source | major histocompatibility complex, class II, DP beta 1 | 15437 | -0.101 | -0.4427 | No |
| 22 | HLA-DRB1 | HLA-DRB1 Entrez,  Source | major histocompatibility complex, class II, DR beta 1 | 16236 | -0.116 | -0.4539 | Yes |
| 23 | PRF1 | PRF1 Entrez,  Source | perforin 1 (pore forming protein) | 16257 | -0.116 | -0.4250 | Yes |
| 24 | CD80 | CD80 Entrez,  Source | CD80 molecule | 16726 | -0.126 | -0.4166 | Yes |
| 25 | IL5 | IL5 Entrez,  Source | interleukin 5 (colony-stimulating factor, eosinophil) | 16732 | -0.126 | -0.3845 | Yes |
| 26 | HLA-DMB | HLA-DMB Entrez,  Source | major histocompatibility complex, class II, DM beta | 17061 | -0.134 | -0.3668 | Yes |
| 27 | TNF | TNF Entrez,  Source | tumor necrosis factor (TNF superfamily, member 2) | 17349 | -0.143 | -0.3446 | Yes |
| 28 | HLA-DQB1 | HLA-DQB1 Entrez,  Source | major histocompatibility complex, class II, DQ beta 1 | 17788 | -0.158 | -0.3265 | Yes |
| 29 | IL12A | IL12A Entrez,  Source | interleukin 12A (natural killer cell stimulatory factor 1, cytotoxic lymphocyte maturation factor 1, p35) | 18214 | -0.177 | -0.3029 | Yes |
| 30 | HLA-DOB | HLA-DOB Entrez,  Source | major histocompatibility complex, class II, DO beta | 18287 | -0.181 | -0.2602 | Yes |
| 31 | IL4 | IL4 Entrez,  Source | interleukin 4 | 18404 | -0.187 | -0.2180 | Yes |
| 32 | FASLG | FASLG Entrez,  Source | Fas ligand (TNF superfamily, member 6) | 18956 | -0.237 | -0.1853 | Yes |
| 33 | IL10 | IL10 Entrez,  Source | interleukin 10 | 19415 | -0.420 | -0.1010 | Yes |
| 34 | GZMB | GZMB Entrez,  Source | granzyme B (granzyme 2, cytotoxic T-lymphocyte-associated serine esterase 1) | 19419 | -0.421 | 0.0070 | Yes |
Table: GSEA details [plain text format]

  

Fig 2: KEGG\_ALLOGRAFT\_REJECTION      
 Blue-Pink O' Gram in the Space of the Analyzed GeneSet

  

Fig 3: KEGG\_ALLOGRAFT\_REJECTION: Random ES distribution      
 Gene set null distribution of ES for **KEGG\_ALLOGRAFT\_REJECTION**

  
