# Supplementary material for: Comprehensive analysis of β-catenin target genes in colorectal carcinoma cell lines with deregulated Wnt/β-catenin signaling
Source: BMC Genomics. 2014 Jan 28;15:74. doi: 10.1186/1471-2164-15-74 (PMC3909937; doi:10.1186/1471-2164-15-74)
Supplement: Additional file 5 — GSEA analysis using the KEGG pathway database. This zipped file contains confirming data of the GSEA analysis. The names of the directories containing the files were composed of the term ‘GSEA’, the name of the cell line, e.g. DLD1, SW480, or LS174T, and the pathway database (KEGG). Please use a web browser to view the files with the name ‘index.html’ in the corresponding directories to start exploring the data. [file 1471-2164-15-74-S5.zip › GSEA KEGG SW480/KEGG_AMINO_SUGAR_AND_NUCLEOTIDE_SUGAR_METABOLISM.html]

Details for gene set KEGG\_AMINO\_SUGAR\_AND\_NUCLEOTIDE\_SUGAR\_METABOLISM[GSEA]

|  || Dataset | SW480\_collapsed\_to\_symbols.class.cls#b\_versus\_bg.class.cls#b\_versus\_bg\_repos |
| Phenotype | class.cls#b\_versus\_bg\_repos |
| Upregulated in class | 1 |
| GeneSet | KEGG\_AMINO\_SUGAR\_AND\_NUCLEOTIDE\_SUGAR\_METABOLISM |
| Enrichment Score (ES) | 0.49657795 |
| Normalized Enrichment Score (NES) | 1.6884279 |
| Nominal p-value | 0.0074074073 |
| FDR q-value | 0.063776545 |
| FWER p-Value | 0.414 |
Table: GSEA Results Summary

  

Fig 1: Enrichment plot: KEGG\_AMINO\_SUGAR\_AND\_NUCLEOTIDE\_SUGAR\_METABOLISM      
 Profile of the Running ES Score & Positions of GeneSet Members on the Rank Ordered List

  

| PROBE | GENE SYMBOL | GENE\_TITLE | RANK IN GENE LIST | RANK METRIC SCORE | RUNNING ES | CORE ENRICHMENT || 1 | FPGT | FPGT Entrez,  Source | fucose-1-phosphate guanylyltransferase | 54 | 0.636 | 0.1328 | Yes |
| 2 | GNE | GNE Entrez,  Source | glucosamine (UDP-N-acetyl)-2-epimerase/N-acetylmannosamine kinase | 269 | 0.361 | 0.1987 | Yes |
| 3 | GMPPA | GMPPA Entrez,  Source | GDP-mannose pyrophosphorylase A | 703 | 0.233 | 0.2263 | Yes |
| 4 | GMPPB | GMPPB Entrez,  Source | GDP-mannose pyrophosphorylase B | 726 | 0.229 | 0.2739 | Yes |
| 5 | GALE | GALE Entrez,  Source | UDP-galactose-4-epimerase | 897 | 0.203 | 0.3085 | Yes |
| 6 | MPI | MPI Entrez,  Source | mannose phosphate isomerase | 1476 | 0.149 | 0.3106 | Yes |
| 7 | PGM3 | PGM3 Entrez,  Source | phosphoglucomutase 3 | 1523 | 0.146 | 0.3394 | Yes |
| 8 | GNPDA2 | GNPDA2 Entrez,  Source | glucosamine-6-phosphate deaminase 2 | 1549 | 0.144 | 0.3687 | Yes |
| 9 | HEXB | HEXB Entrez,  Source | hexosaminidase B (beta polypeptide) | 1884 | 0.126 | 0.3785 | Yes |
| 10 | HEXA | HEXA Entrez,  Source | hexosaminidase A (alpha polypeptide) | 2127 | 0.115 | 0.3906 | Yes |
| 11 | PMM1 | PMM1 Entrez,  Source | phosphomannomutase 1 | 2153 | 0.114 | 0.4135 | Yes |
| 12 | PGM1 | PGM1 Entrez,  Source | phosphoglucomutase 1 | 2506 | 0.099 | 0.4167 | Yes |
| 13 | UXS1 | UXS1 Entrez,  Source | UDP-glucuronate decarboxylase 1 | 2538 | 0.098 | 0.4361 | Yes |
| 14 | GMDS | GMDS Entrez,  Source | GDP-mannose 4,6-dehydratase | 2813 | 0.089 | 0.4409 | Yes |
| 15 | NAGK | NAGK Entrez,  Source | N-acetylglucosamine kinase | 2937 | 0.084 | 0.4526 | Yes |
| 16 | GALK2 | GALK2 Entrez,  Source | galactokinase 2 | 3019 | 0.082 | 0.4659 | Yes |
| 17 | GALT | GALT Entrez,  Source | galactose-1-phosphate uridylyltransferase | 3291 | 0.074 | 0.4677 | Yes |
| 18 | CMAS | CMAS Entrez,  Source | cytidine monophosphate N-acetylneuraminic acid synthetase | 3306 | 0.073 | 0.4826 | Yes |
| 19 | PGM2 | PGM2 Entrez,  Source | phosphoglucomutase 2 | 3540 | 0.067 | 0.4849 | Yes |
| 20 | AMDHD2 | AMDHD2 Entrez,  Source | amidohydrolase domain containing 2 | 3648 | 0.064 | 0.4930 | Yes |
| 21 | GALK1 | GALK1 Entrez,  Source | galactokinase 1 | 3827 | 0.060 | 0.4966 | Yes |
| 22 | CYB5R1 | CYB5R1 Entrez,  Source | cytochrome b5 reductase 1 | 4635 | 0.042 | 0.4642 | No |
| 23 | CYB5R3 | CYB5R3 Entrez,  Source | cytochrome b5 reductase 3 | 5229 | 0.031 | 0.4405 | No |
| 24 | UGP2 | UGP2 Entrez,  Source | UDP-glucose pyrophosphorylase 2 | 5473 | 0.028 | 0.4339 | No |
| 25 | FUK | FUK Entrez,  Source | fucokinase | 7118 | 0.004 | 0.3505 | No |
| 26 | NANS | NANS Entrez,  Source | N-acetylneuraminic acid synthase (sialic acid synthase) | 7517 | -0.001 | 0.3303 | No |
| 27 | HK1 | HK1 Entrez,  Source | hexokinase 1 | 7823 | -0.005 | 0.3157 | No |
| 28 | GPI | GPI Entrez,  Source | glucose phosphate isomerase | 8418 | -0.012 | 0.2878 | No |
| 29 | GNPDA1 | GNPDA1 Entrez,  Source | glucosamine-6-phosphate deaminase 1 | 9263 | -0.021 | 0.2491 | No |
| 30 | CHIA | CHIA Entrez,  Source | chitinase, acidic | 9363 | -0.023 | 0.2489 | No |
| 31 | HK2 | HK2 Entrez,  Source | hexokinase 2 | 9703 | -0.027 | 0.2372 | No |
| 32 | NANP | NANP Entrez,  Source | N-acetylneuraminic acid phosphatase | 9831 | -0.028 | 0.2366 | No |
| 33 | RENBP | RENBP Entrez,  Source | renin binding protein | 10223 | -0.033 | 0.2236 | No |
| 34 | PMM2 | PMM2 Entrez,  Source | phosphomannomutase 2 | 10589 | -0.037 | 0.2127 | No |
| 35 | TSTA3 | TSTA3 Entrez,  Source | tissue specific transplantation antigen P35B | 11956 | -0.054 | 0.1542 | No |
| 36 | NPL | NPL Entrez,  Source | N-acetylneuraminate pyruvate lyase (dihydrodipicolinate synthase) | 12804 | -0.064 | 0.1245 | No |
| 37 | HK3 | HK3 Entrez,  Source | hexokinase 3 (white cell) | 13624 | -0.075 | 0.0984 | No |
| 38 | GFPT1 | GFPT1 Entrez,  Source | glutamine-fructose-6-phosphate transaminase 1 | 14458 | -0.086 | 0.0740 | No |
| 39 | GCK | GCK Entrez,  Source | glucokinase (hexokinase 4, maturity onset diabetes of the young 2) | 14730 | -0.090 | 0.0792 | No |
| 40 | UAP1 | UAP1 Entrez,  Source | UDP-N-acteylglucosamine pyrophosphorylase 1 | 15305 | -0.099 | 0.0708 | No |
| 41 | GNPNAT1 | GNPNAT1 Entrez,  Source | glucosamine-phosphate N-acetyltransferase 1 | 16494 | -0.121 | 0.0356 | No |
| 42 | CHIT1 | CHIT1 Entrez,  Source | chitinase 1 (chitotriosidase) | 16567 | -0.122 | 0.0580 | No |
| 43 | UGDH | UGDH Entrez,  Source | UDP-glucose dehydrogenase | 17478 | -0.148 | 0.0428 | No |
| 44 | GFPT2 | GFPT2 Entrez,  Source | glutamine-fructose-6-phosphate transaminase 2 | 19235 | -0.298 | 0.0165 | No |
Table: GSEA details [plain text format]

  

Fig 2: KEGG\_AMINO\_SUGAR\_AND\_NUCLEOTIDE\_SUGAR\_METABOLISM      
 Blue-Pink O' Gram in the Space of the Analyzed GeneSet

  

Fig 3: KEGG\_AMINO\_SUGAR\_AND\_NUCLEOTIDE\_SUGAR\_METABOLISM: Random ES distribution      
 Gene set null distribution of ES for **KEGG\_AMINO\_SUGAR\_AND\_NUCLEOTIDE\_SUGAR\_METABOLISM**

  
