# Supplementary material for: Comprehensive analysis of β-catenin target genes in colorectal carcinoma cell lines with deregulated Wnt/β-catenin signaling
Source: BMC Genomics. 2014 Jan 28;15:74. doi: 10.1186/1471-2164-15-74 (PMC3909937; doi:10.1186/1471-2164-15-74)
Supplement: Additional file 5 — GSEA analysis using the KEGG pathway database. This zipped file contains confirming data of the GSEA analysis. The names of the directories containing the files were composed of the term ‘GSEA’, the name of the cell line, e.g. DLD1, SW480, or LS174T, and the pathway database (KEGG). Please use a web browser to view the files with the name ‘index.html’ in the corresponding directories to start exploring the data. [file 1471-2164-15-74-S5.zip › GSEA KEGG SW480/KEGG_APOPTOSIS.html]

Details for gene set KEGG\_APOPTOSIS[GSEA]

|  || Dataset | SW480\_collapsed\_to\_symbols.class.cls#b\_versus\_bg.class.cls#b\_versus\_bg\_repos |
| Phenotype | class.cls#b\_versus\_bg\_repos |
| Upregulated in class | 1 |
| GeneSet | KEGG\_APOPTOSIS |
| Enrichment Score (ES) | 0.51419365 |
| Normalized Enrichment Score (NES) | 2.017362 |
| Nominal p-value | 0.0 |
| FDR q-value | 0.020340495 |
| FWER p-Value | 0.013 |
Table: GSEA Results Summary

  

Fig 1: Enrichment plot: KEGG\_APOPTOSIS      
 Profile of the Running ES Score & Positions of GeneSet Members on the Rank Ordered List

  

| PROBE | GENE SYMBOL | GENE\_TITLE | RANK IN GENE LIST | RANK METRIC SCORE | RUNNING ES | CORE ENRICHMENT || 1 | IL1RAP | IL1RAP Entrez,  Source | interleukin 1 receptor accessory protein | 18 | 0.817 | 0.0829 | Yes |
| 2 | PRKAR2B | PRKAR2B Entrez,  Source | protein kinase, cAMP-dependent, regulatory, type II, beta | 66 | 0.601 | 0.1422 | Yes |
| 3 | PRKX | PRKX Entrez,  Source | protein kinase, X-linked | 207 | 0.397 | 0.1758 | Yes |
| 4 | TNFSF10 | TNFSF10 Entrez,  Source | tumor necrosis factor (ligand) superfamily, member 10 | 325 | 0.328 | 0.2034 | Yes |
| 5 | PPP3CA | PPP3CA Entrez,  Source | protein phosphatase 3 (formerly 2B), catalytic subunit, alpha isoform (calcineurin A alpha) | 396 | 0.304 | 0.2310 | Yes |
| 6 | BIRC3 | BIRC3 Entrez,  Source | baculoviral IAP repeat-containing 3 | 455 | 0.289 | 0.2577 | Yes |
| 7 | BIRC2 | BIRC2 Entrez,  Source | baculoviral IAP repeat-containing 2 | 572 | 0.255 | 0.2779 | Yes |
| 8 | APAF1 | APAF1 Entrez,  Source | apoptotic peptidase activating factor | 584 | 0.253 | 0.3033 | Yes |
| 9 | IRAK2 | IRAK2 Entrez,  Source | interleukin-1 receptor-associated kinase 2 | 721 | 0.229 | 0.3199 | Yes |
| 10 | IKBKB | IKBKB Entrez,  Source | inhibitor of kappa light polypeptide gene enhancer in B-cells, kinase beta | 860 | 0.208 | 0.3341 | Yes |
| 11 | AKT3 | AKT3 Entrez,  Source | v-akt murine thymoma viral oncogene homolog 3 (protein kinase B, gamma) | 870 | 0.207 | 0.3549 | Yes |
| 12 | PRKAR1A | PRKAR1A Entrez,  Source | protein kinase, cAMP-dependent, regulatory, type I, alpha (tissue specific extinguisher 1) | 873 | 0.206 | 0.3759 | Yes |
| 13 | CFLAR | CFLAR Entrez,  Source | CASP8 and FADD-like apoptosis regulator | 961 | 0.194 | 0.3913 | Yes |
| 14 | IKBKG | IKBKG Entrez,  Source | inhibitor of kappa light polypeptide gene enhancer in B-cells, kinase gamma | 1169 | 0.172 | 0.3984 | Yes |
| 15 | TNFRSF1A | TNFRSF1A Entrez,  Source | tumor necrosis factor receptor superfamily, member 1A | 1236 | 0.167 | 0.4121 | Yes |
| 16 | TRADD | TRADD Entrez,  Source | TNFRSF1A-associated via death domain | 1245 | 0.166 | 0.4287 | Yes |
| 17 | BAD | BAD Entrez,  Source | BCL2-antagonist of cell death | 1250 | 0.166 | 0.4456 | Yes |
| 18 | RIPK1 | RIPK1 Entrez,  Source | receptor (TNFRSF)-interacting serine-threonine kinase 1 | 1275 | 0.164 | 0.4612 | Yes |
| 19 | CAPN2 | CAPN2 Entrez,  Source | calpain 2, (m/II) large subunit | 1413 | 0.154 | 0.4699 | Yes |
| 20 | MAP3K14 | MAP3K14 Entrez,  Source | mitogen-activated protein kinase kinase kinase 14 | 1528 | 0.146 | 0.4790 | Yes |
| 21 | FADD | FADD Entrez,  Source | Fas (TNFRSF6)-associated via death domain | 1711 | 0.133 | 0.4833 | Yes |
| 22 | IL1A | IL1A Entrez,  Source | interleukin 1, alpha | 1817 | 0.129 | 0.4912 | Yes |
| 23 | BID | BID Entrez,  Source | BH3 interacting domain death agonist | 1919 | 0.125 | 0.4988 | Yes |
| 24 | PPP3CC | PPP3CC Entrez,  Source | protein phosphatase 3 (formerly 2B), catalytic subunit, gamma isoform (calcineurin A gamma) | 1978 | 0.122 | 0.5083 | Yes |
| 25 | CASP8 | CASP8 Entrez,  Source | caspase 8, apoptosis-related cysteine peptidase | 2096 | 0.116 | 0.5142 | Yes |
| 26 | CHP | CHP Entrez,  Source | - | 2515 | 0.099 | 0.5029 | No |
| 27 | CAPN1 | CAPN1 Entrez,  Source | calpain 1, (mu/I) large subunit | 2789 | 0.089 | 0.4980 | No |
| 28 | ATM | ATM Entrez,  Source | ataxia telangiectasia mutated (includes complementation groups A, C and D) | 3015 | 0.082 | 0.4949 | No |
| 29 | PIK3CB | PIK3CB Entrez,  Source | phosphoinositide-3-kinase, catalytic, beta polypeptide | 3241 | 0.075 | 0.4910 | No |
| 30 | NFKBIA | NFKBIA Entrez,  Source | nuclear factor of kappa light polypeptide gene enhancer in B-cells inhibitor, alpha | 3360 | 0.072 | 0.4923 | No |
| 31 | AKT2 | AKT2 Entrez,  Source | v-akt murine thymoma viral oncogene homolog 2 | 3525 | 0.067 | 0.4908 | No |
| 32 | PRKACB | PRKACB Entrez,  Source | protein kinase, cAMP-dependent, catalytic, beta | 3675 | 0.063 | 0.4896 | No |
| 33 | CHUK | CHUK Entrez,  Source | conserved helix-loop-helix ubiquitous kinase | 3863 | 0.059 | 0.4861 | No |
| 34 | FAS | FAS Entrez,  Source | Fas (TNF receptor superfamily, member 6) | 3978 | 0.056 | 0.4860 | No |
| 35 | IRAK4 | IRAK4 Entrez,  Source | interleukin-1 receptor-associated kinase 4 | 4286 | 0.049 | 0.4752 | No |
| 36 | CASP7 | CASP7 Entrez,  Source | caspase 7, apoptosis-related cysteine peptidase | 4480 | 0.045 | 0.4700 | No |
| 37 | BAX | BAX Entrez,  Source | BCL2-associated X protein | 4534 | 0.044 | 0.4718 | No |
| 38 | IRAK1 | IRAK1 Entrez,  Source | interleukin-1 receptor-associated kinase 1 | 4935 | 0.036 | 0.4550 | No |
| 39 | MYD88 | MYD88 Entrez,  Source | myeloid differentiation primary response gene (88) | 5125 | 0.033 | 0.4487 | No |
| 40 | PPP3CB | PPP3CB Entrez,  Source | protein phosphatase 3 (formerly 2B), catalytic subunit, beta isoform (calcineurin A beta) | 5210 | 0.032 | 0.4477 | No |
| 41 | RELA | RELA Entrez,  Source | v-rel reticuloendotheliosis viral oncogene homolog A, nuclear factor of kappa light polypeptide gene enhancer in B-cells 3, p65 (avian) | 5505 | 0.027 | 0.4353 | No |
| 42 | TNFRSF10B | TNFRSF10B Entrez,  Source | tumor necrosis factor receptor superfamily, member 10b | 5581 | 0.026 | 0.4341 | No |
| 43 | TRAF2 | TRAF2 Entrez,  Source | TNF receptor-associated factor 2 | 5841 | 0.022 | 0.4231 | No |
| 44 | AKT1 | AKT1 Entrez,  Source | v-akt murine thymoma viral oncogene homolog 1 | 6295 | 0.015 | 0.4014 | No |
| 45 | CASP3 | CASP3 Entrez,  Source | caspase 3, apoptosis-related cysteine peptidase | 6322 | 0.015 | 0.4016 | No |
| 46 | PIK3R1 | PIK3R1 Entrez,  Source | phosphoinositide-3-kinase, regulatory subunit 1 (p85 alpha) | 6402 | 0.014 | 0.3989 | No |
| 47 | TNFRSF10A | TNFRSF10A Entrez,  Source | tumor necrosis factor receptor superfamily, member 10a | 7040 | 0.005 | 0.3667 | No |
| 48 | ENDOD1 | ENDOD1 Entrez,  Source | endonuclease domain containing 1 | 7563 | -0.001 | 0.3401 | No |
| 49 | DFFA | DFFA Entrez,  Source | DNA fragmentation factor, 45kDa, alpha polypeptide | 7599 | -0.002 | 0.3385 | No |
| 50 | BCL2L1 | BCL2L1 Entrez,  Source | BCL2-like 1 | 7937 | -0.006 | 0.3218 | No |
| 51 | NTRK1 | NTRK1 Entrez,  Source | neurotrophic tyrosine kinase, receptor, type 1 | 7954 | -0.006 | 0.3216 | No |
| 52 | PRKAR2A | PRKAR2A Entrez,  Source | protein kinase, cAMP-dependent, regulatory, type II, alpha | 8036 | -0.007 | 0.3182 | No |
| 53 | BCL2 | BCL2 Entrez,  Source | B-cell CLL/lymphoma 2 | 8283 | -0.010 | 0.3066 | No |
| 54 | PRKACA | PRKACA Entrez,  Source | protein kinase, cAMP-dependent, catalytic, alpha | 8583 | -0.014 | 0.2927 | No |
| 55 | CYCS | CYCS Entrez,  Source | cytochrome c, somatic | 8696 | -0.015 | 0.2885 | No |
| 56 | NFKB1 | NFKB1 Entrez,  Source | nuclear factor of kappa light polypeptide gene enhancer in B-cells 1 (p105) | 8939 | -0.018 | 0.2779 | No |
| 57 | ENDOG | ENDOG Entrez,  Source | endonuclease G | 9105 | -0.020 | 0.2715 | No |
| 58 | CASP6 | CASP6 Entrez,  Source | caspase 6, apoptosis-related cysteine peptidase | 9438 | -0.024 | 0.2568 | No |
| 59 | PIK3R3 | PIK3R3 Entrez,  Source | phosphoinositide-3-kinase, regulatory subunit 3 (p55, gamma) | 10110 | -0.031 | 0.2256 | No |
| 60 | PIK3R5 | PIK3R5 Entrez,  Source | phosphoinositide-3-kinase, regulatory subunit 5, p101 | 10124 | -0.031 | 0.2281 | No |
| 61 | PIK3CG | PIK3CG Entrez,  Source | phosphoinositide-3-kinase, catalytic, gamma polypeptide | 10795 | -0.039 | 0.1978 | No |
| 62 | IL3RA | IL3RA Entrez,  Source | interleukin 3 receptor, alpha (low affinity) | 11277 | -0.046 | 0.1777 | No |
| 63 | PIK3CA | PIK3CA Entrez,  Source | phosphoinositide-3-kinase, catalytic, alpha polypeptide | 11588 | -0.049 | 0.1669 | No |
| 64 | IL3 | IL3 Entrez,  Source | interleukin 3 (colony-stimulating factor, multiple) | 13294 | -0.071 | 0.0866 | No |
| 65 | TNFRSF10D | TNFRSF10D Entrez,  Source | tumor necrosis factor receptor superfamily, member 10d, decoy with truncated death domain | 13530 | -0.074 | 0.0821 | No |
| 66 | IRAK3 | IRAK3 Entrez,  Source | interleukin-1 receptor-associated kinase 3 | 13851 | -0.078 | 0.0736 | No |
| 67 | IL1R1 | IL1R1 Entrez,  Source | interleukin 1 receptor, type I | 14058 | -0.080 | 0.0713 | No |
| 68 | CSF2RB | CSF2RB Entrez,  Source | colony stimulating factor 2 receptor, beta, low-affinity (granulocyte-macrophage) | 14232 | -0.083 | 0.0709 | No |
| 69 | PIK3R2 | PIK3R2 Entrez,  Source | phosphoinositide-3-kinase, regulatory subunit 2 (p85 beta) | 14488 | -0.086 | 0.0666 | No |
| 70 | PPP3R2 | PPP3R2 Entrez,  Source | protein phosphatase 3 (formerly 2B), regulatory subunit B, 19kDa, beta isoform (calcineurin B, type II) | 14956 | -0.093 | 0.0521 | No |
| 71 | CASP10 | CASP10 Entrez,  Source | caspase 10, apoptosis-related cysteine peptidase | 14975 | -0.093 | 0.0608 | No |
| 72 | CASP9 | CASP9 Entrez,  Source | caspase 9, apoptosis-related cysteine peptidase | 15133 | -0.096 | 0.0626 | No |
| 73 | TP53 | TP53 Entrez,  Source | tumor protein p53 (Li-Fraumeni syndrome) | 15395 | -0.100 | 0.0595 | No |
| 74 | IL1B | IL1B Entrez,  Source | interleukin 1, beta | 15552 | -0.103 | 0.0620 | No |
| 75 | PPP3R1 | PPP3R1 Entrez,  Source | protein phosphatase 3 (formerly 2B), regulatory subunit B, 19kDa, alpha isoform (calcineurin B, type I) | 16469 | -0.120 | 0.0273 | No |
| 76 | PIK3CD | PIK3CD Entrez,  Source | phosphoinositide-3-kinase, catalytic, delta polypeptide | 16633 | -0.124 | 0.0317 | No |
| 77 | TNFRSF10C | TNFRSF10C Entrez,  Source | tumor necrosis factor receptor superfamily, member 10c, decoy without an intracellular domain | 16696 | -0.125 | 0.0413 | No |
| 78 | TNF | TNF Entrez,  Source | tumor necrosis factor (TNF superfamily, member 2) | 17349 | -0.143 | 0.0226 | No |
| 79 | PRKACG | PRKACG Entrez,  Source | protein kinase, cAMP-dependent, catalytic, gamma | 18249 | -0.178 | -0.0053 | No |
| 80 | FASLG | FASLG Entrez,  Source | Fas ligand (TNF superfamily, member 6) | 18956 | -0.237 | -0.0172 | No |
| 81 | DFFB | DFFB Entrez,  Source | DNA fragmentation factor, 40kDa, beta polypeptide (caspase-activated DNase) | 19457 | -0.468 | 0.0051 | No |
Table: GSEA details [plain text format]

  

Fig 2: KEGG\_APOPTOSIS      
 Blue-Pink O' Gram in the Space of the Analyzed GeneSet

  

Fig 3: KEGG\_APOPTOSIS: Random ES distribution      
 Gene set null distribution of ES for **KEGG\_APOPTOSIS**

  
