# Supplementary material for: Comprehensive analysis of β-catenin target genes in colorectal carcinoma cell lines with deregulated Wnt/β-catenin signaling
Source: BMC Genomics. 2014 Jan 28;15:74. doi: 10.1186/1471-2164-15-74 (PMC3909937; doi:10.1186/1471-2164-15-74)
Supplement: Additional file 5 — GSEA analysis using the KEGG pathway database. This zipped file contains confirming data of the GSEA analysis. The names of the directories containing the files were composed of the term ‘GSEA’, the name of the cell line, e.g. DLD1, SW480, or LS174T, and the pathway database (KEGG). Please use a web browser to view the files with the name ‘index.html’ in the corresponding directories to start exploring the data. [file 1471-2164-15-74-S5.zip › GSEA KEGG SW480/KEGG_ARGININE_AND_PROLINE_METABOLISM.html]

Details for gene set KEGG\_ARGININE\_AND\_PROLINE\_METABOLISM[GSEA]

|  || Dataset | SW480\_collapsed\_to\_symbols.class.cls#b\_versus\_bg.class.cls#b\_versus\_bg\_repos |
| Phenotype | class.cls#b\_versus\_bg\_repos |
| Upregulated in class | 0 |
| GeneSet | KEGG\_ARGININE\_AND\_PROLINE\_METABOLISM |
| Enrichment Score (ES) | -0.35723722 |
| Normalized Enrichment Score (NES) | -1.2450628 |
| Nominal p-value | 0.15239726 |
| FDR q-value | 0.5659799 |
| FWER p-Value | 1.0 |
Table: GSEA Results Summary

  

Fig 1: Enrichment plot: KEGG\_ARGININE\_AND\_PROLINE\_METABOLISM      
 Profile of the Running ES Score & Positions of GeneSet Members on the Rank Ordered List

  

| PROBE | GENE SYMBOL | GENE\_TITLE | RANK IN GENE LIST | RANK METRIC SCORE | RUNNING ES | CORE ENRICHMENT || 1 | ASL | ASL Entrez,  Source | argininosuccinate lyase | 563 | 0.258 | 0.0132 | No |
| 2 | ALDH3A2 | ALDH3A2 Entrez,  Source | aldehyde dehydrogenase 3 family, member A2 | 1015 | 0.188 | 0.0209 | No |
| 3 | P4HA2 | P4HA2 Entrez,  Source | procollagen-proline, 2-oxoglutarate 4-dioxygenase (proline 4-hydroxylase), alpha polypeptide II | 1021 | 0.188 | 0.0513 | No |
| 4 | ABP1 | ABP1 Entrez,  Source | amiloride binding protein 1 (amine oxidase (copper-containing)) | 1132 | 0.175 | 0.0743 | No |
| 5 | NOS3 | NOS3 Entrez,  Source | nitric oxide synthase 3 (endothelial cell) | 1155 | 0.173 | 0.1016 | No |
| 6 | GOT1 | GOT1 Entrez,  Source | glutamic-oxaloacetic transaminase 1, soluble (aspartate aminotransferase 1) | 1517 | 0.147 | 0.1070 | No |
| 7 | GLS | GLS Entrez,  Source | glutaminase | 2685 | 0.092 | 0.0623 | No |
| 8 | ALDH9A1 | ALDH9A1 Entrez,  Source | aldehyde dehydrogenase 9 family, member A1 | 3008 | 0.082 | 0.0592 | No |
| 9 | ALDH7A1 | ALDH7A1 Entrez,  Source | aldehyde dehydrogenase 7 family, member A1 | 3132 | 0.078 | 0.0657 | No |
| 10 | SMS | SMS Entrez,  Source | spermine synthase | 3365 | 0.072 | 0.0656 | No |
| 11 | ARG1 | ARG1 Entrez,  Source | arginase, liver | 3544 | 0.067 | 0.0673 | No |
| 12 | SAT1 | SAT1 Entrez,  Source | spermidine/spermine N1-acetyltransferase 1 | 3665 | 0.063 | 0.0715 | No |
| 13 | LAP3 | LAP3 Entrez,  Source | leucine aminopeptidase 3 | 3847 | 0.059 | 0.0719 | No |
| 14 | SAT2 | SAT2 Entrez,  Source | spermidine/spermine N1-acetyltransferase 2 | 5301 | 0.030 | 0.0023 | No |
| 15 | PYCR2 | PYCR2 Entrez,  Source | pyrroline-5-carboxylate reductase family, member 2 | 5530 | 0.027 | -0.0050 | No |
| 16 | PYCRL | PYCRL Entrez,  Source | pyrroline-5-carboxylate reductase-like | 5916 | 0.021 | -0.0214 | No |
| 17 | GLUL | GLUL Entrez,  Source | glutamate-ammonia ligase (glutamine synthetase) | 7969 | -0.007 | -0.1255 | No |
| 18 | MAOA | MAOA Entrez,  Source | monoamine oxidase A | 9475 | -0.024 | -0.1987 | No |
| 19 | ALDH4A1 | ALDH4A1 Entrez,  Source | aldehyde dehydrogenase 4 family, member A1 | 9929 | -0.029 | -0.2172 | No |
| 20 | P4HA1 | P4HA1 Entrez,  Source | procollagen-proline, 2-oxoglutarate 4-dioxygenase (proline 4-hydroxylase), alpha polypeptide I | 10201 | -0.032 | -0.2258 | No |
| 21 | GOT2 | GOT2 Entrez,  Source | glutamic-oxaloacetic transaminase 2, mitochondrial (aspartate aminotransferase 2) | 11404 | -0.047 | -0.2797 | No |
| 22 | OAT | OAT Entrez,  Source | ornithine aminotransferase (gyrate atrophy) | 11539 | -0.049 | -0.2786 | No |
| 23 | CPS1 | CPS1 Entrez,  Source | carbamoyl-phosphate synthetase 1, mitochondrial | 11677 | -0.050 | -0.2774 | No |
| 24 | OTC | OTC Entrez,  Source | ornithine carbamoyltransferase | 11689 | -0.050 | -0.2698 | No |
| 25 | ALDH2 | ALDH2 Entrez,  Source | aldehyde dehydrogenase 2 family (mitochondrial) | 12077 | -0.055 | -0.2806 | No |
| 26 | AMD1 | AMD1 Entrez,  Source | adenosylmethionine decarboxylase 1 | 12172 | -0.056 | -0.2762 | No |
| 27 | GATM | GATM Entrez,  Source | glycine amidinotransferase (L-arginine:glycine amidinotransferase) | 12375 | -0.059 | -0.2769 | No |
| 28 | DAO | DAO Entrez,  Source | D-amino-acid oxidase | 12517 | -0.061 | -0.2743 | No |
| 29 | ADC | ADC Entrez,  Source | arginine decarboxylase | 12784 | -0.064 | -0.2775 | No |
| 30 | PRODH | PRODH Entrez,  Source | proline dehydrogenase (oxidase) 1 | 13367 | -0.071 | -0.2956 | No |
| 31 | NOS1 | NOS1 Entrez,  Source | nitric oxide synthase 1 (neuronal) | 13991 | -0.079 | -0.3146 | No |
| 32 | PRODH2 | PRODH2 Entrez,  Source | proline dehydrogenase (oxidase) 2 | 14152 | -0.082 | -0.3094 | No |
| 33 | AGMAT | AGMAT Entrez,  Source | agmatine ureohydrolase (agmatinase) | 14205 | -0.082 | -0.2986 | No |
| 34 | CKM | CKM Entrez,  Source | creatine kinase, muscle | 14579 | -0.087 | -0.3035 | No |
| 35 | ACY1 | ACY1 Entrez,  Source | aminoacylase 1 | 15012 | -0.094 | -0.3103 | No |
| 36 | GAMT | GAMT Entrez,  Source | guanidinoacetate N-methyltransferase | 15929 | -0.110 | -0.3393 | Yes |
| 37 | ALDH18A1 | ALDH18A1 Entrez,  Source | aldehyde dehydrogenase 18 family, member A1 | 16104 | -0.113 | -0.3297 | Yes |
| 38 | P4HA3 | P4HA3 Entrez,  Source | procollagen-proline, 2-oxoglutarate 4-dioxygenase (proline 4-hydroxylase), alpha polypeptide III | 16354 | -0.118 | -0.3231 | Yes |
| 39 | GLUD2 | GLUD2 Entrez,  Source | glutamate dehydrogenase 2 | 16583 | -0.122 | -0.3148 | Yes |
| 40 | ALDH1B1 | ALDH1B1 Entrez,  Source | aldehyde dehydrogenase 1 family, member B1 | 16646 | -0.124 | -0.2977 | Yes |
| 41 | NAGS | NAGS Entrez,  Source | N-acetylglutamate synthase | 16745 | -0.126 | -0.2821 | Yes |
| 42 | GLUD1 | GLUD1 Entrez,  Source | glutamate dehydrogenase 1 | 17237 | -0.140 | -0.2844 | Yes |
| 43 | SRM | SRM Entrez,  Source | spermidine synthase | 17505 | -0.148 | -0.2738 | Yes |
| 44 | ODC1 | ODC1 Entrez,  Source | ornithine decarboxylase 1 | 17872 | -0.161 | -0.2662 | Yes |
| 45 | PYCR1 | PYCR1 Entrez,  Source | pyrroline-5-carboxylate reductase 1 | 18712 | -0.209 | -0.2750 | Yes |
| 46 | MAOB | MAOB Entrez,  Source | monoamine oxidase B | 18778 | -0.215 | -0.2432 | Yes |
| 47 | ASS1 | ASS1 Entrez,  Source | argininosuccinate synthetase 1 | 19141 | -0.271 | -0.2175 | Yes |
| 48 | ARG2 | ARG2 Entrez,  Source | arginase, type II | 19145 | -0.271 | -0.1732 | Yes |
| 49 | GLS2 | GLS2 Entrez,  Source | glutaminase 2 (liver, mitochondrial) | 19265 | -0.312 | -0.1283 | Yes |
| 50 | CKB | CKB Entrez,  Source | creatine kinase, brain | 19426 | -0.430 | -0.0661 | Yes |
| 51 | CKMT2 | CKMT2 Entrez,  Source | creatine kinase, mitochondrial 2 (sarcomeric) | 19440 | -0.445 | 0.0059 | Yes |
Table: GSEA details [plain text format]

  

Fig 2: KEGG\_ARGININE\_AND\_PROLINE\_METABOLISM      
 Blue-Pink O' Gram in the Space of the Analyzed GeneSet

  

Fig 3: KEGG\_ARGININE\_AND\_PROLINE\_METABOLISM: Random ES distribution      
 Gene set null distribution of ES for **KEGG\_ARGININE\_AND\_PROLINE\_METABOLISM**

  
