# Supplementary material for: Comprehensive analysis of β-catenin target genes in colorectal carcinoma cell lines with deregulated Wnt/β-catenin signaling
Source: BMC Genomics. 2014 Jan 28;15:74. doi: 10.1186/1471-2164-15-74 (PMC3909937; doi:10.1186/1471-2164-15-74)
Supplement: Additional file 5 — GSEA analysis using the KEGG pathway database. This zipped file contains confirming data of the GSEA analysis. The names of the directories containing the files were composed of the term ‘GSEA’, the name of the cell line, e.g. DLD1, SW480, or LS174T, and the pathway database (KEGG). Please use a web browser to view the files with the name ‘index.html’ in the corresponding directories to start exploring the data. [file 1471-2164-15-74-S5.zip › GSEA KEGG SW480/KEGG_ASTHMA.html]

Details for gene set KEGG\_ASTHMA[GSEA]

|  || Dataset | SW480\_collapsed\_to\_symbols.class.cls#b\_versus\_bg.class.cls#b\_versus\_bg\_repos |
| Phenotype | class.cls#b\_versus\_bg\_repos |
| Upregulated in class | 0 |
| GeneSet | KEGG\_ASTHMA |
| Enrichment Score (ES) | -0.55480736 |
| Normalized Enrichment Score (NES) | -1.6523446 |
| Nominal p-value | 0.008695652 |
| FDR q-value | 0.097093046 |
| FWER p-Value | 0.488 |
Table: GSEA Results Summary

  

Fig 1: Enrichment plot: KEGG\_ASTHMA      
 Profile of the Running ES Score & Positions of GeneSet Members on the Rank Ordered List

  

| PROBE | GENE SYMBOL | GENE\_TITLE | RANK IN GENE LIST | RANK METRIC SCORE | RUNNING ES | CORE ENRICHMENT || 1 | HLA-DMA | HLA-DMA Entrez,  Source | major histocompatibility complex, class II, DM alpha | 2247 | 0.110 | -0.0801 | No |
| 2 | HLA-DRB4 | HLA-DRB4 Entrez,  Source | major histocompatibility complex, class II, DR beta 4 | 3022 | 0.082 | -0.0937 | No |
| 3 | HLA-DRA | HLA-DRA Entrez,  Source | major histocompatibility complex, class II, DR alpha | 3456 | 0.069 | -0.0938 | No |
| 4 | HLA-DPA1 | HLA-DPA1 Entrez,  Source | major histocompatibility complex, class II, DP alpha 1 | 7007 | 0.006 | -0.2737 | No |
| 5 | HLA-DQA1 | HLA-DQA1 Entrez,  Source | major histocompatibility complex, class II, DQ alpha 1 | 8795 | -0.016 | -0.3601 | No |
| 6 | FCER1G | FCER1G Entrez,  Source | Fc fragment of IgE, high affinity I, receptor for; gamma polypeptide | 10454 | -0.035 | -0.4338 | No |
| 7 | HLA-DOA | HLA-DOA Entrez,  Source | major histocompatibility complex, class II, DO alpha | 11429 | -0.047 | -0.4686 | No |
| 8 | PRG2 | PRG2 Entrez,  Source | proteoglycan 2, bone marrow (natural killer cell activator, eosinophil granule major basic protein) | 12100 | -0.056 | -0.4852 | No |
| 9 | CCL11 | CCL11 Entrez,  Source | chemokine (C-C motif) ligand 11 | 12119 | -0.056 | -0.4683 | No |
| 10 | CD40 | CD40 Entrez,  Source | CD40 molecule, TNF receptor superfamily member 5 | 12929 | -0.066 | -0.4888 | No |
| 11 | IL3 | IL3 Entrez,  Source | interleukin 3 (colony-stimulating factor, multiple) | 13294 | -0.071 | -0.4850 | No |
| 12 | CD40LG | CD40LG Entrez,  Source | CD40 ligand (TNF superfamily, member 5, hyper-IgM syndrome) | 14658 | -0.088 | -0.5266 | Yes |
| 13 | RNASE3 | RNASE3 Entrez,  Source | ribonuclease, RNase A family, 3 (eosinophil cationic protein) | 15123 | -0.096 | -0.5199 | Yes |
| 14 | HLA-DPB1 | HLA-DPB1 Entrez,  Source | major histocompatibility complex, class II, DP beta 1 | 15437 | -0.101 | -0.5038 | Yes |
| 15 | HLA-DRB1 | HLA-DRB1 Entrez,  Source | major histocompatibility complex, class II, DR beta 1 | 16236 | -0.116 | -0.5077 | Yes |
| 16 | MS4A2 | MS4A2 Entrez,  Source | membrane-spanning 4-domains, subfamily A, member 2 (Fc fragment of IgE, high affinity I, receptor for; beta polypeptide) | 16316 | -0.117 | -0.4744 | Yes |
| 17 | IL13 | IL13 Entrez,  Source | interleukin 13 | 16498 | -0.121 | -0.4452 | Yes |
| 18 | IL5 | IL5 Entrez,  Source | interleukin 5 (colony-stimulating factor, eosinophil) | 16732 | -0.126 | -0.4169 | Yes |
| 19 | EPX | EPX Entrez,  Source | eosinophil peroxidase | 16760 | -0.127 | -0.3779 | Yes |
| 20 | HLA-DMB | HLA-DMB Entrez,  Source | major histocompatibility complex, class II, DM beta | 17061 | -0.134 | -0.3505 | Yes |
| 21 | TNF | TNF Entrez,  Source | tumor necrosis factor (TNF superfamily, member 2) | 17349 | -0.143 | -0.3195 | Yes |
| 22 | FCER1A | FCER1A Entrez,  Source | Fc fragment of IgE, high affinity I, receptor for; alpha polypeptide | 17730 | -0.156 | -0.2894 | Yes |
| 23 | HLA-DQB1 | HLA-DQB1 Entrez,  Source | major histocompatibility complex, class II, DQ beta 1 | 17788 | -0.158 | -0.2420 | Yes |
| 24 | HLA-DOB | HLA-DOB Entrez,  Source | major histocompatibility complex, class II, DO beta | 18287 | -0.181 | -0.2100 | Yes |
| 25 | IL4 | IL4 Entrez,  Source | interleukin 4 | 18404 | -0.187 | -0.1563 | Yes |
| 26 | IL9 | IL9 Entrez,  Source | interleukin 9 | 19077 | -0.256 | -0.1092 | Yes |
| 27 | IL10 | IL10 Entrez,  Source | interleukin 10 | 19415 | -0.420 | 0.0072 | Yes |
Table: GSEA details [plain text format]

  

Fig 2: KEGG\_ASTHMA      
 Blue-Pink O' Gram in the Space of the Analyzed GeneSet

  

Fig 3: KEGG\_ASTHMA: Random ES distribution      
 Gene set null distribution of ES for **KEGG\_ASTHMA**

  
