# Supplementary material for: Comprehensive analysis of β-catenin target genes in colorectal carcinoma cell lines with deregulated Wnt/β-catenin signaling
Source: BMC Genomics. 2014 Jan 28;15:74. doi: 10.1186/1471-2164-15-74 (PMC3909937; doi:10.1186/1471-2164-15-74)
Supplement: Additional file 5 — GSEA analysis using the KEGG pathway database. This zipped file contains confirming data of the GSEA analysis. The names of the directories containing the files were composed of the term ‘GSEA’, the name of the cell line, e.g. DLD1, SW480, or LS174T, and the pathway database (KEGG). Please use a web browser to view the files with the name ‘index.html’ in the corresponding directories to start exploring the data. [file 1471-2164-15-74-S5.zip › GSEA KEGG SW480/KEGG_AUTOIMMUNE_THYROID_DISEASE.html]

Details for gene set KEGG\_AUTOIMMUNE\_THYROID\_DISEASE[GSEA]

|  || Dataset | SW480\_collapsed\_to\_symbols.class.cls#b\_versus\_bg.class.cls#b\_versus\_bg\_repos |
| Phenotype | class.cls#b\_versus\_bg\_repos |
| Upregulated in class | 0 |
| GeneSet | KEGG\_AUTOIMMUNE\_THYROID\_DISEASE |
| Enrichment Score (ES) | -0.50498414 |
| Normalized Enrichment Score (NES) | -1.6956568 |
| Nominal p-value | 0.0033500837 |
| FDR q-value | 0.10582073 |
| FWER p-Value | 0.358 |
Table: GSEA Results Summary

  

Fig 1: Enrichment plot: KEGG\_AUTOIMMUNE\_THYROID\_DISEASE      
 Profile of the Running ES Score & Positions of GeneSet Members on the Rank Ordered List

  

| PROBE | GENE SYMBOL | GENE\_TITLE | RANK IN GENE LIST | RANK METRIC SCORE | RUNNING ES | CORE ENRICHMENT || 1 | HLA-A | HLA-A Entrez,  Source | major histocompatibility complex, class I, A | 1218 | 0.168 | -0.0322 | No |
| 2 | HLA-F | HLA-F Entrez,  Source | major histocompatibility complex, class I, F | 1341 | 0.159 | -0.0098 | No |
| 3 | HLA-DMA | HLA-DMA Entrez,  Source | major histocompatibility complex, class II, DM alpha | 2247 | 0.110 | -0.0365 | No |
| 4 | HLA-G | HLA-G Entrez,  Source | HLA-G histocompatibility antigen, class I, G | 2852 | 0.087 | -0.0518 | No |
| 5 | HLA-DRB4 | HLA-DRB4 Entrez,  Source | major histocompatibility complex, class II, DR beta 4 | 3022 | 0.082 | -0.0458 | No |
| 6 | HLA-C | HLA-C Entrez,  Source | major histocompatibility complex, class I, C | 3264 | 0.074 | -0.0448 | No |
| 7 | HLA-B | HLA-B Entrez,  Source | major histocompatibility complex, class I, B | 3267 | 0.074 | -0.0315 | No |
| 8 | HLA-DRA | HLA-DRA Entrez,  Source | major histocompatibility complex, class II, DR alpha | 3456 | 0.069 | -0.0287 | No |
| 9 | FAS | FAS Entrez,  Source | Fas (TNF receptor superfamily, member 6) | 3978 | 0.056 | -0.0453 | No |
| 10 | TG | TG Entrez,  Source | thyroglobulin | 4306 | 0.049 | -0.0533 | No |
| 11 | CD86 | CD86 Entrez,  Source | CD86 molecule | 6293 | 0.015 | -0.1524 | No |
| 12 | HLA-E | HLA-E Entrez,  Source | major histocompatibility complex, class I, E | 6630 | 0.011 | -0.1677 | No |
| 13 | HLA-DPA1 | HLA-DPA1 Entrez,  Source | major histocompatibility complex, class II, DP alpha 1 | 7007 | 0.006 | -0.1859 | No |
| 14 | IFNA2 | IFNA2 Entrez,  Source | interferon, alpha 2 | 7881 | -0.006 | -0.2297 | No |
| 15 | HLA-DQA1 | HLA-DQA1 Entrez,  Source | major histocompatibility complex, class II, DQ alpha 1 | 8795 | -0.016 | -0.2736 | No |
| 16 | CD28 | CD28 Entrez,  Source | CD28 molecule | 9542 | -0.025 | -0.3074 | No |
| 17 | TPO | TPO Entrez,  Source | thyroid peroxidase | 9693 | -0.027 | -0.3103 | No |
| 18 | TSHR | TSHR Entrez,  Source | thyroid stimulating hormone receptor | 10594 | -0.037 | -0.3498 | No |
| 19 | HLA-DOA | HLA-DOA Entrez,  Source | major histocompatibility complex, class II, DO alpha | 11429 | -0.047 | -0.3840 | No |
| 20 | IFNA6 | IFNA6 Entrez,  Source | interferon, alpha 6 | 11757 | -0.051 | -0.3916 | No |
| 21 | IFNA8 | IFNA8 Entrez,  Source | interferon, alpha 8 | 12021 | -0.055 | -0.3952 | No |
| 22 | CD40 | CD40 Entrez,  Source | CD40 molecule, TNF receptor superfamily member 5 | 12929 | -0.066 | -0.4299 | No |
| 23 | IFNA21 | IFNA21 Entrez,  Source | interferon, alpha 21 | 12989 | -0.066 | -0.4210 | No |
| 24 | CTLA4 | CTLA4 Entrez,  Source | cytotoxic T-lymphocyte-associated protein 4 | 14367 | -0.084 | -0.4765 | No |
| 25 | IL2 | IL2 Entrez,  Source | interleukin 2 | 14448 | -0.085 | -0.4652 | No |
| 26 | CD40LG | CD40LG Entrez,  Source | CD40 ligand (TNF superfamily, member 5, hyper-IgM syndrome) | 14658 | -0.088 | -0.4600 | No |
| 27 | TSHB | TSHB Entrez,  Source | thyroid stimulating hormone, beta | 15248 | -0.098 | -0.4726 | No |
| 28 | HLA-DPB1 | HLA-DPB1 Entrez,  Source | major histocompatibility complex, class II, DP beta 1 | 15437 | -0.101 | -0.4641 | No |
| 29 | HLA-DRB1 | HLA-DRB1 Entrez,  Source | major histocompatibility complex, class II, DR beta 1 | 16236 | -0.116 | -0.4842 | Yes |
| 30 | PRF1 | PRF1 Entrez,  Source | perforin 1 (pore forming protein) | 16257 | -0.116 | -0.4643 | Yes |
| 31 | IFNA10 | IFNA10 Entrez,  Source | interferon, alpha 10 | 16258 | -0.116 | -0.4434 | Yes |
| 32 | IFNA5 | IFNA5 Entrez,  Source | interferon, alpha 5 | 16708 | -0.126 | -0.4439 | Yes |
| 33 | CD80 | CD80 Entrez,  Source | CD80 molecule | 16726 | -0.126 | -0.4221 | Yes |
| 34 | IL5 | IL5 Entrez,  Source | interleukin 5 (colony-stimulating factor, eosinophil) | 16732 | -0.126 | -0.3997 | Yes |
| 35 | IFNA7 | IFNA7 Entrez,  Source | interferon, alpha 7 | 17022 | -0.133 | -0.3906 | Yes |
| 36 | HLA-DMB | HLA-DMB Entrez,  Source | major histocompatibility complex, class II, DM beta | 17061 | -0.134 | -0.3684 | Yes |
| 37 | HLA-DQB1 | HLA-DQB1 Entrez,  Source | major histocompatibility complex, class II, DQ beta 1 | 17788 | -0.158 | -0.3772 | Yes |
| 38 | CGA | CGA Entrez,  Source | glycoprotein hormones, alpha polypeptide | 17947 | -0.164 | -0.3558 | Yes |
| 39 | IFNA1 | IFNA1 Entrez,  Source | interferon, alpha 1 | 18074 | -0.169 | -0.3318 | Yes |
| 40 | IFNA17 | IFNA17 Entrez,  Source | interferon, alpha 17 | 18148 | -0.173 | -0.3044 | Yes |
| 41 | HLA-DOB | HLA-DOB Entrez,  Source | major histocompatibility complex, class II, DO beta | 18287 | -0.181 | -0.2790 | Yes |
| 42 | IFNA14 | IFNA14 Entrez,  Source | interferon, alpha 14 | 18299 | -0.181 | -0.2471 | Yes |
| 43 | IFNA16 | IFNA16 Entrez,  Source | interferon, alpha 16 | 18386 | -0.186 | -0.2180 | Yes |
| 44 | IL4 | IL4 Entrez,  Source | interleukin 4 | 18404 | -0.187 | -0.1852 | Yes |
| 45 | FASLG | FASLG Entrez,  Source | Fas ligand (TNF superfamily, member 6) | 18956 | -0.237 | -0.1708 | Yes |
| 46 | IFNA4 | IFNA4 Entrez,  Source | interferon, alpha 4 | 19182 | -0.280 | -0.1320 | Yes |
| 47 | IL10 | IL10 Entrez,  Source | interleukin 10 | 19415 | -0.420 | -0.0685 | Yes |
| 48 | GZMB | GZMB Entrez,  Source | granzyme B (granzyme 2, cytotoxic T-lymphocyte-associated serine esterase 1) | 19419 | -0.421 | 0.0070 | Yes |
Table: GSEA details [plain text format]

  

Fig 2: KEGG\_AUTOIMMUNE\_THYROID\_DISEASE      
 Blue-Pink O' Gram in the Space of the Analyzed GeneSet

  

Fig 3: KEGG\_AUTOIMMUNE\_THYROID\_DISEASE: Random ES distribution      
 Gene set null distribution of ES for **KEGG\_AUTOIMMUNE\_THYROID\_DISEASE**

  
