# Supplementary material for: Comprehensive analysis of β-catenin target genes in colorectal carcinoma cell lines with deregulated Wnt/β-catenin signaling
Source: BMC Genomics. 2014 Jan 28;15:74. doi: 10.1186/1471-2164-15-74 (PMC3909937; doi:10.1186/1471-2164-15-74)
Supplement: Additional file 5 — GSEA analysis using the KEGG pathway database. This zipped file contains confirming data of the GSEA analysis. The names of the directories containing the files were composed of the term ‘GSEA’, the name of the cell line, e.g. DLD1, SW480, or LS174T, and the pathway database (KEGG). Please use a web browser to view the files with the name ‘index.html’ in the corresponding directories to start exploring the data. [file 1471-2164-15-74-S5.zip › GSEA KEGG SW480/KEGG_BASAL_CELL_CARCINOMA.html]

Details for gene set KEGG\_BASAL\_CELL\_CARCINOMA[GSEA]

|  || Dataset | SW480\_collapsed\_to\_symbols.class.cls#b\_versus\_bg.class.cls#b\_versus\_bg\_repos |
| Phenotype | class.cls#b\_versus\_bg\_repos |
| Upregulated in class | 0 |
| GeneSet | KEGG\_BASAL\_CELL\_CARCINOMA |
| Enrichment Score (ES) | -0.4673117 |
| Normalized Enrichment Score (NES) | -1.6498972 |
| Nominal p-value | 0.003552398 |
| FDR q-value | 0.08228908 |
| FWER p-Value | 0.498 |
Table: GSEA Results Summary

  

Fig 1: Enrichment plot: KEGG\_BASAL\_CELL\_CARCINOMA      
 Profile of the Running ES Score & Positions of GeneSet Members on the Rank Ordered List

  

| PROBE | GENE SYMBOL | GENE\_TITLE | RANK IN GENE LIST | RANK METRIC SCORE | RUNNING ES | CORE ENRICHMENT || 1 | FZD1 | FZD1 Entrez,  Source | frizzled homolog 1 (Drosophila) | 128 | 0.470 | 0.0609 | No |
| 2 | FZD7 | FZD7 Entrez,  Source | frizzled homolog 7 (Drosophila) | 1144 | 0.175 | 0.0339 | No |
| 3 | TCF7L2 | TCF7L2 Entrez,  Source | transcription factor 7-like 2 (T-cell specific, HMG-box) | 1570 | 0.143 | 0.0326 | No |
| 4 | WNT7A | WNT7A Entrez,  Source | wingless-type MMTV integration site family, member 7A | 1917 | 0.125 | 0.0328 | No |
| 5 | WNT9A | WNT9A Entrez,  Source | wingless-type MMTV integration site family, member 9A | 1959 | 0.123 | 0.0483 | No |
| 6 | GLI3 | GLI3 Entrez,  Source | GLI-Kruppel family member GLI3 (Greig cephalopolysyndactyly syndrome) | 1967 | 0.122 | 0.0654 | No |
| 7 | GLI2 | GLI2 Entrez,  Source | GLI-Kruppel family member GLI2 | 3882 | 0.058 | -0.0243 | No |
| 8 | FZD2 | FZD2 Entrez,  Source | frizzled homolog 2 (Drosophila) | 3910 | 0.058 | -0.0174 | No |
| 9 | DVL2 | DVL2 Entrez,  Source | dishevelled, dsh homolog 2 (Drosophila) | 4810 | 0.039 | -0.0580 | No |
| 10 | FZD3 | FZD3 Entrez,  Source | frizzled homolog 3 (Drosophila) | 5233 | 0.031 | -0.0751 | No |
| 11 | WNT5A | WNT5A Entrez,  Source | wingless-type MMTV integration site family, member 5A | 5570 | 0.026 | -0.0886 | No |
| 12 | DVL3 | DVL3 Entrez,  Source | dishevelled, dsh homolog 3 (Drosophila) | 5885 | 0.021 | -0.1017 | No |
| 13 | WNT16 | WNT16 Entrez,  Source | wingless-type MMTV integration site family, member 16 | 5995 | 0.020 | -0.1044 | No |
| 14 | GSK3B | GSK3B Entrez,  Source | glycogen synthase kinase 3 beta | 6824 | 0.008 | -0.1457 | No |
| 15 | WNT10A | WNT10A Entrez,  Source | wingless-type MMTV integration site family, member 10A | 8181 | -0.009 | -0.2139 | No |
| 16 | FZD4 | FZD4 Entrez,  Source | frizzled homolog 4 (Drosophila) | 8655 | -0.014 | -0.2361 | No |
| 17 | WNT3 | WNT3 Entrez,  Source | wingless-type MMTV integration site family, member 3 | 9581 | -0.025 | -0.2799 | No |
| 18 | APC | APC Entrez,  Source | adenomatosis polyposis coli | 9658 | -0.026 | -0.2801 | No |
| 19 | APC2 | APC2 Entrez,  Source | adenomatosis polyposis coli 2 | 9797 | -0.028 | -0.2832 | No |
| 20 | STK36 | STK36 Entrez,  Source | serine/threonine kinase 36 (fused homolog, Drosophila) | 10512 | -0.036 | -0.3147 | No |
| 21 | BMP4 | BMP4 Entrez,  Source | bone morphogenetic protein 4 | 11384 | -0.047 | -0.3526 | No |
| 22 | WNT1 | WNT1 Entrez,  Source | wingless-type MMTV integration site family, member 1 | 11756 | -0.051 | -0.3643 | No |
| 23 | DVL1 | DVL1 Entrez,  Source | dishevelled, dsh homolog 1 (Drosophila) | 11895 | -0.053 | -0.3638 | No |
| 24 | FZD6 | FZD6 Entrez,  Source | frizzled homolog 6 (Drosophila) | 12007 | -0.054 | -0.3616 | No |
| 25 | LEF1 | LEF1 Entrez,  Source | lymphoid enhancer-binding factor 1 | 12073 | -0.055 | -0.3570 | No |
| 26 | TCF7 | TCF7 Entrez,  Source | transcription factor 7 (T-cell specific, HMG-box) | 12964 | -0.066 | -0.3932 | No |
| 27 | GLI1 | GLI1 Entrez,  Source | glioma-associated oncogene homolog 1 (zinc finger protein) | 13632 | -0.075 | -0.4167 | No |
| 28 | WNT9B | WNT9B Entrez,  Source | wingless-type MMTV integration site family, member 9B | 13833 | -0.078 | -0.4158 | No |
| 29 | WNT8B | WNT8B Entrez,  Source | wingless-type MMTV integration site family, member 8B | 13981 | -0.079 | -0.4119 | No |
| 30 | WNT10B | WNT10B Entrez,  Source | wingless-type MMTV integration site family, member 10B | 15062 | -0.095 | -0.4537 | Yes |
| 31 | BMP2 | BMP2 Entrez,  Source | bone morphogenetic protein 2 | 15195 | -0.097 | -0.4465 | Yes |
| 32 | TP53 | TP53 Entrez,  Source | tumor protein p53 (Li-Fraumeni syndrome) | 15395 | -0.100 | -0.4424 | Yes |
| 33 | FZD5 | FZD5 Entrez,  Source | frizzled homolog 5 (Drosophila) | 15583 | -0.103 | -0.4371 | Yes |
| 34 | SMO | SMO Entrez,  Source | smoothened homolog (Drosophila) | 15857 | -0.109 | -0.4355 | Yes |
| 35 | WNT2B | WNT2B Entrez,  Source | wingless-type MMTV integration site family, member 2B | 16011 | -0.111 | -0.4274 | Yes |
| 36 | WNT4 | WNT4 Entrez,  Source | wingless-type MMTV integration site family, member 4 | 16063 | -0.112 | -0.4139 | Yes |
| 37 | WNT8A | WNT8A Entrez,  Source | wingless-type MMTV integration site family, member 8A | 16353 | -0.118 | -0.4118 | Yes |
| 38 | FZD8 | FZD8 Entrez,  Source | frizzled homolog 8 (Drosophila) | 16521 | -0.121 | -0.4029 | Yes |
| 39 | SUFU | SUFU Entrez,  Source | suppressor of fused homolog (Drosophila) | 16780 | -0.127 | -0.3979 | Yes |
| 40 | TCF7L1 | TCF7L1 Entrez,  Source | transcription factor 7-like 1 (T-cell specific, HMG-box) | 17330 | -0.143 | -0.4056 | Yes |
| 41 | PTCH2 | PTCH2 Entrez,  Source | patched homolog 2 (Drosophila) | 17468 | -0.147 | -0.3915 | Yes |
| 42 | WNT2 | WNT2 Entrez,  Source | wingless-type MMTV integration site family member 2 | 17510 | -0.149 | -0.3723 | Yes |
| 43 | FZD9 | FZD9 Entrez,  Source | frizzled homolog 9 (Drosophila) | 17528 | -0.149 | -0.3517 | Yes |
| 44 | AXIN1 | AXIN1 Entrez,  Source | axin 1 | 17530 | -0.149 | -0.3304 | Yes |
| 45 | HHIP | HHIP Entrez,  Source | hedgehog interacting protein | 18621 | -0.202 | -0.3573 | Yes |
| 46 | PTCH1 | PTCH1 Entrez,  Source | patched homolog 1 (Drosophila) | 18816 | -0.219 | -0.3359 | Yes |
| 47 | CTNNB1 | CTNNB1 Entrez,  Source | catenin (cadherin-associated protein), beta 1, 88kDa | 18943 | -0.235 | -0.3086 | Yes |
| 48 | SHH | SHH Entrez,  Source | sonic hedgehog homolog (Drosophila) | 19038 | -0.250 | -0.2776 | Yes |
| 49 | FZD10 | FZD10 Entrez,  Source | frizzled homolog 10 (Drosophila) | 19260 | -0.308 | -0.2448 | Yes |
| 50 | WNT11 | WNT11 Entrez,  Source | wingless-type MMTV integration site family, member 11 | 19383 | -0.388 | -0.1954 | Yes |
| 51 | AXIN2 | AXIN2 Entrez,  Source | axin 2 (conductin, axil) | 19400 | -0.409 | -0.1375 | Yes |
| 52 | WNT5B | WNT5B Entrez,  Source | wingless-type MMTV integration site family, member 5B | 19464 | -0.482 | -0.0716 | Yes |
| 53 | WNT6 | WNT6 Entrez,  Source | wingless-type MMTV integration site family, member 6 | 19488 | -0.532 | 0.0035 | Yes |
Table: GSEA details [plain text format]

  

Fig 2: KEGG\_BASAL\_CELL\_CARCINOMA      
 Blue-Pink O' Gram in the Space of the Analyzed GeneSet

  

Fig 3: KEGG\_BASAL\_CELL\_CARCINOMA: Random ES distribution      
 Gene set null distribution of ES for **KEGG\_BASAL\_CELL\_CARCINOMA**

  
