# Supplementary material for: Comprehensive analysis of β-catenin target genes in colorectal carcinoma cell lines with deregulated Wnt/β-catenin signaling
Source: BMC Genomics. 2014 Jan 28;15:74. doi: 10.1186/1471-2164-15-74 (PMC3909937; doi:10.1186/1471-2164-15-74)
Supplement: Additional file 5 — GSEA analysis using the KEGG pathway database. This zipped file contains confirming data of the GSEA analysis. The names of the directories containing the files were composed of the term ‘GSEA’, the name of the cell line, e.g. DLD1, SW480, or LS174T, and the pathway database (KEGG). Please use a web browser to view the files with the name ‘index.html’ in the corresponding directories to start exploring the data. [file 1471-2164-15-74-S5.zip › GSEA KEGG SW480/KEGG_COMPLEMENT_AND_COAGULATION_CASCADES.html]

Details for gene set KEGG\_COMPLEMENT\_AND\_COAGULATION\_CASCADES[GSEA]

|  || Dataset | SW480\_collapsed\_to\_symbols.class.cls#b\_versus\_bg.class.cls#b\_versus\_bg\_repos |
| Phenotype | class.cls#b\_versus\_bg\_repos |
| Upregulated in class | 1 |
| GeneSet | KEGG\_COMPLEMENT\_AND\_COAGULATION\_CASCADES |
| Enrichment Score (ES) | 0.46464682 |
| Normalized Enrichment Score (NES) | 1.7443404 |
| Nominal p-value | 0.0 |
| FDR q-value | 0.04572646 |
| FWER p-Value | 0.277 |
Table: GSEA Results Summary

  

Fig 1: Enrichment plot: KEGG\_COMPLEMENT\_AND\_COAGULATION\_CASCADES      
 Profile of the Running ES Score & Positions of GeneSet Members on the Rank Ordered List

  

| PROBE | GENE SYMBOL | GENE\_TITLE | RANK IN GENE LIST | RANK METRIC SCORE | RUNNING ES | CORE ENRICHMENT || 1 | F13A1 | F13A1 Entrez,  Source | coagulation factor XIII, A1 polypeptide | 19 | 0.814 | 0.0867 | Yes |
| 2 | PLAU | PLAU Entrez,  Source | plasminogen activator, urokinase | 58 | 0.625 | 0.1522 | Yes |
| 3 | PLAT | PLAT Entrez,  Source | plasminogen activator, tissue | 115 | 0.488 | 0.2019 | Yes |
| 4 | F8 | F8 Entrez,  Source | coagulation factor VIII, procoagulant component (hemophilia A) | 123 | 0.474 | 0.2527 | Yes |
| 5 | F3 | F3 Entrez,  Source | coagulation factor III (thromboplastin, tissue factor) | 136 | 0.457 | 0.3013 | Yes |
| 6 | SERPINA5 | SERPINA5 Entrez,  Source | serpin peptidase inhibitor, clade A (alpha-1 antiproteinase, antitrypsin), member 5 | 202 | 0.399 | 0.3409 | Yes |
| 7 | PROS1 | PROS1 Entrez,  Source | protein S (alpha) | 283 | 0.350 | 0.3746 | Yes |
| 8 | SERPINA1 | SERPINA1 Entrez,  Source | serpin peptidase inhibitor, clade A (alpha-1 antiproteinase, antitrypsin), member 1 | 366 | 0.315 | 0.4044 | Yes |
| 9 | PLAUR | PLAUR Entrez,  Source | plasminogen activator, urokinase receptor | 437 | 0.292 | 0.4323 | Yes |
| 10 | CD59 | CD59 Entrez,  Source | CD59 molecule, complement regulatory protein | 586 | 0.253 | 0.4520 | Yes |
| 11 | SERPINE1 | SERPINE1 Entrez,  Source | serpin peptidase inhibitor, clade E (nexin, plasminogen activator inhibitor type 1), member 1 | 797 | 0.218 | 0.4646 | Yes |
| 12 | C5 | C5 Entrez,  Source | complement component 5 | 1313 | 0.161 | 0.4556 | No |
| 13 | PROC | PROC Entrez,  Source | protein C (inactivator of coagulation factors Va and VIIIa) | 1583 | 0.142 | 0.4571 | No |
| 14 | CR1 | CR1 Entrez,  Source | complement component (3b/4b) receptor 1 (Knops blood group) | 4144 | 0.052 | 0.3314 | No |
| 15 | C3AR1 | C3AR1 Entrez,  Source | complement component 3a receptor 1 | 5277 | 0.031 | 0.2766 | No |
| 16 | BDKRB2 | BDKRB2 Entrez,  Source | bradykinin receptor B2 | 5382 | 0.029 | 0.2744 | No |
| 17 | THBD | THBD Entrez,  Source | thrombomodulin | 5518 | 0.027 | 0.2703 | No |
| 18 | F9 | F9 Entrez,  Source | coagulation factor IX (plasma thromboplastic component, Christmas disease, hemophilia B) | 5953 | 0.020 | 0.2503 | No |
| 19 | CFI | CFI Entrez,  Source | complement factor I | 6851 | 0.008 | 0.2051 | No |
| 20 | KNG1 | KNG1 Entrez,  Source | kininogen 1 | 7434 | 0.000 | 0.1753 | No |
| 21 | MASP2 | MASP2 Entrez,  Source | mannan-binding lectin serine peptidase 2 | 7918 | -0.006 | 0.1511 | No |
| 22 | C5AR1 | C5AR1 Entrez,  Source | complement component 5a receptor 1 | 8121 | -0.008 | 0.1416 | No |
| 23 | C4BPA | C4BPA Entrez,  Source | complement component 4 binding protein, alpha | 8400 | -0.012 | 0.1286 | No |
| 24 | C1QA | C1QA Entrez,  Source | complement component 1, q subcomponent, A chain | 8440 | -0.012 | 0.1279 | No |
| 25 | MBL2 | MBL2 Entrez,  Source | mannose-binding lectin (protein C) 2, soluble (opsonic defect) | 8638 | -0.014 | 0.1194 | No |
| 26 | F12 | F12 Entrez,  Source | coagulation factor XII (Hageman factor) | 8661 | -0.014 | 0.1198 | No |
| 27 | FGG | FGG Entrez,  Source | fibrinogen gamma chain | 8972 | -0.018 | 0.1059 | No |
| 28 | BDKRB1 | BDKRB1 Entrez,  Source | bradykinin receptor B1 | 9911 | -0.029 | 0.0609 | No |
| 29 | C9 | C9 Entrez,  Source | complement component 9 | 10382 | -0.034 | 0.0405 | No |
| 30 | KLKB1 | KLKB1 Entrez,  Source | kallikrein B, plasma (Fletcher factor) 1 | 10863 | -0.040 | 0.0202 | No |
| 31 | SERPING1 | SERPING1 Entrez,  Source | serpin peptidase inhibitor, clade G (C1 inhibitor), member 1, (angioedema, hereditary) | 10870 | -0.040 | 0.0242 | No |
| 32 | F7 | F7 Entrez,  Source | coagulation factor VII (serum prothrombin conversion accelerator) | 10991 | -0.042 | 0.0226 | No |
| 33 | VWF | VWF Entrez,  Source | von Willebrand factor | 11029 | -0.043 | 0.0253 | No |
| 34 | FGB | FGB Entrez,  Source | fibrinogen beta chain | 11120 | -0.044 | 0.0254 | No |
| 35 | A2M | A2M Entrez,  Source | alpha-2-macroglobulin | 11282 | -0.046 | 0.0220 | No |
| 36 | CD46 | CD46 Entrez,  Source | CD46 molecule, complement regulatory protein | 11779 | -0.051 | 0.0021 | No |
| 37 | SERPINF2 | SERPINF2 Entrez,  Source | serpin peptidase inhibitor, clade F (alpha-2 antiplasmin, pigment epithelium derived factor), member 2 | 12102 | -0.056 | -0.0084 | No |
| 38 | C7 | C7 Entrez,  Source | complement component 7 | 12129 | -0.056 | -0.0037 | No |
| 39 | F11 | F11 Entrez,  Source | coagulation factor XI (plasma thromboplastin antecedent) | 12136 | -0.056 | 0.0020 | No |
| 40 | C1QC | C1QC Entrez,  Source | complement component 1, q subcomponent, C chain | 12967 | -0.066 | -0.0334 | No |
| 41 | C8A | C8A Entrez,  Source | complement component 8, alpha polypeptide | 12992 | -0.066 | -0.0275 | No |
| 42 | FGA | FGA Entrez,  Source | fibrinogen alpha chain | 13172 | -0.069 | -0.0293 | No |
| 43 | PLG | PLG Entrez,  Source | plasminogen | 13368 | -0.071 | -0.0316 | No |
| 44 | F2 | F2 Entrez,  Source | coagulation factor II (thrombin) | 13790 | -0.077 | -0.0449 | No |
| 45 | SERPINC1 | SERPINC1 Entrez,  Source | serpin peptidase inhibitor, clade C (antithrombin), member 1 | 13906 | -0.078 | -0.0423 | No |
| 46 | C4BPB | C4BPB Entrez,  Source | complement component 4 binding protein, beta | 14141 | -0.081 | -0.0455 | No |
| 47 | CFB | CFB Entrez,  Source | complement factor B | 14145 | -0.082 | -0.0369 | No |
| 48 | C8B | C8B Entrez,  Source | complement component 8, beta polypeptide | 14479 | -0.086 | -0.0447 | No |
| 49 | SERPIND1 | SERPIND1 Entrez,  Source | serpin peptidase inhibitor, clade D (heparin cofactor), member 1 | 14707 | -0.089 | -0.0467 | No |
| 50 | F2R | F2R Entrez,  Source | coagulation factor II (thrombin) receptor | 14863 | -0.091 | -0.0448 | No |
| 51 | MASP1 | MASP1 Entrez,  Source | mannan-binding lectin serine peptidase 1 (C4/C2 activating component of Ra-reactive factor) | 14916 | -0.092 | -0.0375 | No |
| 52 | F5 | F5 Entrez,  Source | coagulation factor V (proaccelerin, labile factor) | 15451 | -0.101 | -0.0540 | No |
| 53 | C2 | C2 Entrez,  Source | complement component 2 | 15766 | -0.107 | -0.0586 | No |
| 54 | CFD | CFD Entrez,  Source | complement factor D (adipsin) | 15961 | -0.111 | -0.0566 | No |
| 55 | C8G | C8G Entrez,  Source | complement component 8, gamma polypeptide | 16138 | -0.114 | -0.0534 | No |
| 56 | C6 | C6 Entrez,  Source | complement component 6 | 16259 | -0.116 | -0.0470 | No |
| 57 | C1QB | C1QB Entrez,  Source | complement component 1, q subcomponent, B chain | 16705 | -0.125 | -0.0563 | No |
| 58 | CFH | CFH Entrez,  Source | complement factor H | 16958 | -0.132 | -0.0551 | No |
| 59 | CD55 | CD55 Entrez,  Source | CD55 molecule, decay accelerating factor for complement (Cromer blood group) | 18578 | -0.198 | -0.1167 | No |
| 60 | F13B | F13B Entrez,  Source | coagulation factor XIII, B polypeptide | 18581 | -0.199 | -0.0954 | No |
| 61 | TFPI | TFPI Entrez,  Source | tissue factor pathway inhibitor (lipoprotein-associated coagulation inhibitor) | 18861 | -0.224 | -0.0856 | No |
| 62 | F10 | F10 Entrez,  Source | coagulation factor X | 18989 | -0.241 | -0.0661 | No |
| 63 | C1S | C1S Entrez,  Source | complement component 1, s subcomponent | 19058 | -0.253 | -0.0423 | No |
| 64 | CPB2 | CPB2 Entrez,  Source | carboxypeptidase B2 (plasma, carboxypeptidase U) | 19063 | -0.254 | -0.0151 | No |
| 65 | CR2 | CR2 Entrez,  Source | complement component (3d/Epstein Barr virus) receptor 2 | 19368 | -0.374 | 0.0096 | No |
Table: GSEA details [plain text format]

  

Fig 2: KEGG\_COMPLEMENT\_AND\_COAGULATION\_CASCADES      
 Blue-Pink O' Gram in the Space of the Analyzed GeneSet

  

Fig 3: KEGG\_COMPLEMENT\_AND\_COAGULATION\_CASCADES: Random ES distribution      
 Gene set null distribution of ES for **KEGG\_COMPLEMENT\_AND\_COAGULATION\_CASCADES**

  
