# Supplementary material for: Comprehensive analysis of β-catenin target genes in colorectal carcinoma cell lines with deregulated Wnt/β-catenin signaling
Source: BMC Genomics. 2014 Jan 28;15:74. doi: 10.1186/1471-2164-15-74 (PMC3909937; doi:10.1186/1471-2164-15-74)
Supplement: Additional file 5 — GSEA analysis using the KEGG pathway database. This zipped file contains confirming data of the GSEA analysis. The names of the directories containing the files were composed of the term ‘GSEA’, the name of the cell line, e.g. DLD1, SW480, or LS174T, and the pathway database (KEGG). Please use a web browser to view the files with the name ‘index.html’ in the corresponding directories to start exploring the data. [file 1471-2164-15-74-S5.zip › GSEA KEGG SW480/KEGG_CYSTEINE_AND_METHIONINE_METABOLISM.html]

Details for gene set KEGG\_CYSTEINE\_AND\_METHIONINE\_METABOLISM[GSEA]

|  || Dataset | SW480\_collapsed\_to\_symbols.class.cls#b\_versus\_bg.class.cls#b\_versus\_bg\_repos |
| Phenotype | class.cls#b\_versus\_bg\_repos |
| Upregulated in class | 0 |
| GeneSet | KEGG\_CYSTEINE\_AND\_METHIONINE\_METABOLISM |
| Enrichment Score (ES) | -0.49029937 |
| Normalized Enrichment Score (NES) | -1.5148833 |
| Nominal p-value | 0.044554457 |
| FDR q-value | 0.19091503 |
| FWER p-Value | 0.904 |
Table: GSEA Results Summary

  

Fig 1: Enrichment plot: KEGG\_CYSTEINE\_AND\_METHIONINE\_METABOLISM      
 Profile of the Running ES Score & Positions of GeneSet Members on the Rank Ordered List

  

| PROBE | GENE SYMBOL | GENE\_TITLE | RANK IN GENE LIST | RANK METRIC SCORE | RUNNING ES | CORE ENRICHMENT || 1 | GOT1 | GOT1 Entrez,  Source | glutamic-oxaloacetic transaminase 1, soluble (aspartate aminotransferase 1) | 1517 | 0.147 | -0.0287 | No |
| 2 | SMS | SMS Entrez,  Source | spermine synthase | 3365 | 0.072 | -0.0993 | No |
| 3 | LDHC | LDHC Entrez,  Source | lactate dehydrogenase C | 3502 | 0.068 | -0.0836 | No |
| 4 | DNMT1 | DNMT1 Entrez,  Source | DNA (cytosine-5-)-methyltransferase 1 | 3756 | 0.061 | -0.0761 | No |
| 5 | MTR | MTR Entrez,  Source | 5-methyltetrahydrofolate-homocysteine methyltransferase | 3771 | 0.061 | -0.0565 | No |
| 6 | AHCYL1 | AHCYL1 Entrez,  Source | S-adenosylhomocysteine hydrolase-like 1 | 3938 | 0.057 | -0.0459 | No |
| 7 | LDHA | LDHA Entrez,  Source | lactate dehydrogenase A | 4695 | 0.041 | -0.0708 | No |
| 8 | BHMT | BHMT Entrez,  Source | betaine-homocysteine methyltransferase | 7567 | -0.001 | -0.2174 | No |
| 9 | ADI1 | ADI1 Entrez,  Source | acireductone dioxygenase 1 | 7807 | -0.005 | -0.2281 | No |
| 10 | LDHB | LDHB Entrez,  Source | lactate dehydrogenase B | 8885 | -0.017 | -0.2774 | No |
| 11 | MAT1A | MAT1A Entrez,  Source | methionine adenosyltransferase I, alpha | 11261 | -0.045 | -0.3839 | No |
| 12 | GOT2 | GOT2 Entrez,  Source | glutamic-oxaloacetic transaminase 2, mitochondrial (aspartate aminotransferase 2) | 11404 | -0.047 | -0.3755 | No |
| 13 | TAT | TAT Entrez,  Source | tyrosine aminotransferase | 11930 | -0.054 | -0.3844 | No |
| 14 | AMD1 | AMD1 Entrez,  Source | adenosylmethionine decarboxylase 1 | 12172 | -0.056 | -0.3780 | No |
| 15 | MPST | MPST Entrez,  Source | mercaptopyruvate sulfurtransferase | 13208 | -0.069 | -0.4078 | No |
| 16 | MAT2B | MAT2B Entrez,  Source | methionine adenosyltransferase II, beta | 14820 | -0.091 | -0.4599 | Yes |
| 17 | APIP | APIP Entrez,  Source | APAF1 interacting protein | 15244 | -0.098 | -0.4488 | Yes |
| 18 | MAT2A | MAT2A Entrez,  Source | methionine adenosyltransferase II, alpha | 15313 | -0.099 | -0.4192 | Yes |
| 19 | SDS | SDS Entrez,  Source | serine dehydratase | 15387 | -0.100 | -0.3895 | Yes |
| 20 | AHCY | AHCY Entrez,  Source | S-adenosylhomocysteine hydrolase | 15529 | -0.102 | -0.3624 | Yes |
| 21 | CBS | CBS Entrez,  Source | cystathionine-beta-synthase | 15580 | -0.103 | -0.3304 | Yes |
| 22 | LDHAL6A | LDHAL6A Entrez,  Source | lactate dehydrogenase A-like 6A | 16101 | -0.113 | -0.3192 | Yes |
| 23 | DNMT3L | DNMT3L Entrez,  Source | DNA (cytosine-5-)-methyltransferase 3-like | 16699 | -0.125 | -0.3079 | Yes |
| 24 | MTAP | MTAP Entrez,  Source | methylthioadenosine phosphorylase | 17156 | -0.137 | -0.2855 | Yes |
| 25 | SRM | SRM Entrez,  Source | spermidine synthase | 17505 | -0.148 | -0.2537 | Yes |
| 26 | DNMT3B | DNMT3B Entrez,  Source | DNA (cytosine-5-)-methyltransferase 3 beta | 17751 | -0.156 | -0.2140 | Yes |
| 27 | CDO1 | CDO1 Entrez,  Source | cysteine dioxygenase, type I | 18009 | -0.167 | -0.1714 | Yes |
| 28 | DNMT3A | DNMT3A Entrez,  Source | DNA (cytosine-5-)-methyltransferase 3 alpha | 18774 | -0.214 | -0.1388 | Yes |
| 29 | CTH | CTH Entrez,  Source | cystathionase (cystathionine gamma-lyase) | 19082 | -0.257 | -0.0686 | Yes |
| 30 | LDHAL6B | LDHAL6B Entrez,  Source | lactate dehydrogenase A-like 6B | 19179 | -0.278 | 0.0193 | Yes |
Table: GSEA details [plain text format]

  

Fig 2: KEGG\_CYSTEINE\_AND\_METHIONINE\_METABOLISM      
 Blue-Pink O' Gram in the Space of the Analyzed GeneSet

  

Fig 3: KEGG\_CYSTEINE\_AND\_METHIONINE\_METABOLISM: Random ES distribution      
 Gene set null distribution of ES for **KEGG\_CYSTEINE\_AND\_METHIONINE\_METABOLISM**

  
