# Supplementary material for: Comprehensive analysis of β-catenin target genes in colorectal carcinoma cell lines with deregulated Wnt/β-catenin signaling
Source: BMC Genomics. 2014 Jan 28;15:74. doi: 10.1186/1471-2164-15-74 (PMC3909937; doi:10.1186/1471-2164-15-74)
Supplement: Additional file 5 — GSEA analysis using the KEGG pathway database. This zipped file contains confirming data of the GSEA analysis. The names of the directories containing the files were composed of the term ‘GSEA’, the name of the cell line, e.g. DLD1, SW480, or LS174T, and the pathway database (KEGG). Please use a web browser to view the files with the name ‘index.html’ in the corresponding directories to start exploring the data. [file 1471-2164-15-74-S5.zip › GSEA KEGG SW480/KEGG_CYTOKINE_CYTOKINE_RECEPTOR_INTERACTION.html]

Details for gene set KEGG\_CYTOKINE\_CYTOKINE\_RECEPTOR\_INTERACTION[GSEA]

|  || Dataset | SW480\_collapsed\_to\_symbols.class.cls#b\_versus\_bg.class.cls#b\_versus\_bg\_repos |
| Phenotype | class.cls#b\_versus\_bg\_repos |
| Upregulated in class | 0 |
| GeneSet | KEGG\_CYTOKINE\_CYTOKINE\_RECEPTOR\_INTERACTION |
| Enrichment Score (ES) | -0.29251426 |
| Normalized Enrichment Score (NES) | -1.291006 |
| Nominal p-value | 0.034638554 |
| FDR q-value | 0.48537707 |
| FWER p-Value | 1.0 |
Table: GSEA Results Summary

  

Fig 1: Enrichment plot: KEGG\_CYTOKINE\_CYTOKINE\_RECEPTOR\_INTERACTION      
 Profile of the Running ES Score & Positions of GeneSet Members on the Rank Ordered List

  

| PROBE | GENE SYMBOL | GENE\_TITLE | RANK IN GENE LIST | RANK METRIC SCORE | RUNNING ES | CORE ENRICHMENT || 1 | IL1RAP | IL1RAP Entrez,  Source | interleukin 1 receptor accessory protein | 18 | 0.817 | 0.0256 | No |
| 2 | PDGFC | PDGFC Entrez,  Source | platelet derived growth factor C | 25 | 0.776 | 0.0504 | No |
| 3 | IL15 | IL15 Entrez,  Source | interleukin 15 | 30 | 0.728 | 0.0738 | No |
| 4 | TNFSF15 | TNFSF15 Entrez,  Source | tumor necrosis factor (ligand) superfamily, member 15 | 71 | 0.588 | 0.0908 | No |
| 5 | LEPR | LEPR Entrez,  Source | leptin receptor | 72 | 0.587 | 0.1098 | No |
| 6 | IL20RA | IL20RA Entrez,  Source | interleukin 20 receptor, alpha | 213 | 0.391 | 0.1152 | No |
| 7 | TNFSF9 | TNFSF9 Entrez,  Source | tumor necrosis factor (ligand) superfamily, member 9 | 219 | 0.388 | 0.1275 | No |
| 8 | CCL2 | CCL2 Entrez,  Source | chemokine (C-C motif) ligand 2 | 264 | 0.362 | 0.1370 | No |
| 9 | TNFSF10 | TNFSF10 Entrez,  Source | tumor necrosis factor (ligand) superfamily, member 10 | 325 | 0.328 | 0.1445 | No |
| 10 | CSF2 | CSF2 Entrez,  Source | colony stimulating factor 2 (granulocyte-macrophage) | 327 | 0.328 | 0.1551 | No |
| 11 | NGFR | NGFR Entrez,  Source | nerve growth factor receptor (TNFR superfamily, member 16) | 349 | 0.321 | 0.1644 | No |
| 12 | CX3CL1 | CX3CL1 Entrez,  Source | chemokine (C-X3-C motif) ligand 1 | 488 | 0.276 | 0.1662 | No |
| 13 | TGFB2 | TGFB2 Entrez,  Source | transforming growth factor, beta 2 | 587 | 0.253 | 0.1694 | No |
| 14 | EGFR | EGFR Entrez,  Source | epidermal growth factor receptor (erythroblastic leukemia viral (v-erb-b) oncogene homolog, avian) | 652 | 0.241 | 0.1738 | No |
| 15 | CXCL2 | CXCL2 Entrez,  Source | chemokine (C-X-C motif) ligand 2 | 889 | 0.204 | 0.1682 | No |
| 16 | IL15RA | IL15RA Entrez,  Source | interleukin 15 receptor, alpha | 1091 | 0.180 | 0.1637 | No |
| 17 | LIF | LIF Entrez,  Source | leukemia inhibitory factor (cholinergic differentiation factor) | 1117 | 0.177 | 0.1681 | No |
| 18 | TGFBR2 | TGFBR2 Entrez,  Source | transforming growth factor, beta receptor II (70/80kDa) | 1151 | 0.174 | 0.1720 | No |
| 19 | IL10RB | IL10RB Entrez,  Source | interleukin 10 receptor, beta | 1153 | 0.174 | 0.1776 | No |
| 20 | ACVR1 | ACVR1 Entrez,  Source | activin A receptor, type I | 1206 | 0.169 | 0.1804 | No |
| 21 | TNFRSF14 | TNFRSF14 Entrez,  Source | tumor necrosis factor receptor superfamily, member 14 (herpesvirus entry mediator) | 1235 | 0.167 | 0.1844 | No |
| 22 | TNFRSF1A | TNFRSF1A Entrez,  Source | tumor necrosis factor receptor superfamily, member 1A | 1236 | 0.167 | 0.1898 | No |
| 23 | TNFRSF12A | TNFRSF12A Entrez,  Source | tumor necrosis factor receptor superfamily, member 12A | 1430 | 0.152 | 0.1847 | No |
| 24 | IL1A | IL1A Entrez,  Source | interleukin 1, alpha | 1817 | 0.129 | 0.1689 | No |
| 25 | IL13RA1 | IL13RA1 Entrez,  Source | interleukin 13 receptor, alpha 1 | 2006 | 0.120 | 0.1631 | No |
| 26 | TSLP | TSLP Entrez,  Source | - | 2140 | 0.115 | 0.1599 | No |
| 27 | IL23R | IL23R Entrez,  Source | interleukin 23 receptor | 2288 | 0.108 | 0.1558 | No |
| 28 | CCR6 | CCR6 Entrez,  Source | chemokine (C-C motif) receptor 6 | 2512 | 0.099 | 0.1475 | No |
| 29 | INHBC | INHBC Entrez,  Source | inhibin, beta C | 2610 | 0.095 | 0.1455 | No |
| 30 | PDGFB | PDGFB Entrez,  Source | platelet-derived growth factor beta polypeptide (simian sarcoma viral (v-sis) oncogene homolog) | 2833 | 0.087 | 0.1369 | No |
| 31 | IL7 | IL7 Entrez,  Source | interleukin 7 | 3146 | 0.078 | 0.1233 | No |
| 32 | CLCF1 | CLCF1 Entrez,  Source | cardiotrophin-like cytokine factor 1 | 3229 | 0.075 | 0.1214 | No |
| 33 | PDGFA | PDGFA Entrez,  Source | platelet-derived growth factor alpha polypeptide | 3262 | 0.074 | 0.1222 | No |
| 34 | BMPR2 | BMPR2 Entrez,  Source | bone morphogenetic protein receptor, type II (serine/threonine kinase) | 3425 | 0.070 | 0.1161 | No |
| 35 | PPBP | PPBP Entrez,  Source | pro-platelet basic protein (chemokine (C-X-C motif) ligand 7) | 3556 | 0.066 | 0.1115 | No |
| 36 | BMPR1A | BMPR1A Entrez,  Source | bone morphogenetic protein receptor, type IA | 3616 | 0.065 | 0.1106 | No |
| 37 | IFNGR2 | IFNGR2 Entrez,  Source | interferon gamma receptor 2 (interferon gamma transducer 1) | 3874 | 0.058 | 0.0991 | No |
| 38 | CCR1 | CCR1 Entrez,  Source | chemokine (C-C motif) receptor 1 | 3902 | 0.058 | 0.0996 | No |
| 39 | CXCL1 | CXCL1 Entrez,  Source | chemokine (C-X-C motif) ligand 1 (melanoma growth stimulating activity, alpha) | 3935 | 0.057 | 0.0998 | No |
| 40 | FAS | FAS Entrez,  Source | Fas (TNF receptor superfamily, member 6) | 3978 | 0.056 | 0.0995 | No |
| 41 | IL25 | IL25 Entrez,  Source | interleukin 25 | 4181 | 0.052 | 0.0907 | No |
| 42 | GH2 | GH2 Entrez,  Source | growth hormone 2 | 4369 | 0.048 | 0.0825 | No |
| 43 | TNFRSF21 | TNFRSF21 Entrez,  Source | tumor necrosis factor receptor superfamily, member 21 | 4422 | 0.047 | 0.0814 | No |
| 44 | IL22 | IL22 Entrez,  Source | interleukin 22 | 4547 | 0.044 | 0.0764 | No |
| 45 | LIFR | LIFR Entrez,  Source | leukemia inhibitory factor receptor alpha | 4657 | 0.042 | 0.0721 | No |
| 46 | IL18 | IL18 Entrez,  Source | interleukin 18 (interferon-gamma-inducing factor) | 4785 | 0.040 | 0.0668 | No |
| 47 | TNFRSF17 | TNFRSF17 Entrez,  Source | tumor necrosis factor receptor superfamily, member 17 | 4818 | 0.039 | 0.0664 | No |
| 48 | IL9R | IL9R Entrez,  Source | interleukin 9 receptor | 4821 | 0.039 | 0.0675 | No |
| 49 | IL6ST | IL6ST Entrez,  Source | interleukin 6 signal transducer (gp130, oncostatin M receptor) | 5259 | 0.031 | 0.0459 | No |
| 50 | CCL28 | CCL28 Entrez,  Source | chemokine (C-C motif) ligand 28 | 5355 | 0.029 | 0.0419 | No |
| 51 | TNFRSF10B | TNFRSF10B Entrez,  Source | tumor necrosis factor receptor superfamily, member 10b | 5581 | 0.026 | 0.0311 | No |
| 52 | CCL26 | CCL26 Entrez,  Source | chemokine (C-C motif) ligand 26 | 6099 | 0.018 | 0.0049 | No |
| 53 | IFNGR1 | IFNGR1 Entrez,  Source | interferon gamma receptor 1 | 6157 | 0.017 | 0.0026 | No |
| 54 | TNFSF14 | TNFSF14 Entrez,  Source | tumor necrosis factor (ligand) superfamily, member 14 | 6230 | 0.016 | -0.0006 | No |
| 55 | IL22RA1 | IL22RA1 Entrez,  Source | interleukin 22 receptor, alpha 1 | 6238 | 0.016 | -0.0005 | No |
| 56 | MET | MET Entrez,  Source | met proto-oncogene (hepatocyte growth factor receptor) | 6408 | 0.014 | -0.0088 | No |
| 57 | CXCL3 | CXCL3 Entrez,  Source | chemokine (C-X-C motif) ligand 3 | 6588 | 0.011 | -0.0177 | No |
| 58 | CXCL16 | CXCL16 Entrez,  Source | chemokine (C-X-C motif) ligand 16 | 6632 | 0.011 | -0.0196 | No |
| 59 | VEGFB | VEGFB Entrez,  Source | vascular endothelial growth factor B | 6773 | 0.009 | -0.0265 | No |
| 60 | IL17RA | IL17RA Entrez,  Source | interleukin 17 receptor A | 6933 | 0.007 | -0.0345 | No |
| 61 | IFNG | IFNG Entrez,  Source | interferon, gamma | 6964 | 0.006 | -0.0359 | No |
| 62 | TNFRSF10A | TNFRSF10A Entrez,  Source | tumor necrosis factor receptor superfamily, member 10a | 7040 | 0.005 | -0.0396 | No |
| 63 | IL7R | IL7R Entrez,  Source | interleukin 7 receptor | 7065 | 0.005 | -0.0407 | No |
| 64 | IL21 | IL21 Entrez,  Source | interleukin 21 | 7070 | 0.005 | -0.0408 | No |
| 65 | CCL23 | CCL23 Entrez,  Source | chemokine (C-C motif) ligand 23 | 7116 | 0.004 | -0.0430 | No |
| 66 | CCL16 | CCL16 Entrez,  Source | chemokine (C-C motif) ligand 16 | 7230 | 0.003 | -0.0487 | No |
| 67 | OSMR | OSMR Entrez,  Source | oncostatin M receptor | 7647 | -0.002 | -0.0702 | No |
| 68 | IL12B | IL12B Entrez,  Source | interleukin 12B (natural killer cell stimulatory factor 2, cytotoxic lymphocyte maturation factor 2, p40) | 7747 | -0.004 | -0.0752 | No |
| 69 | HGF | HGF Entrez,  Source | hepatocyte growth factor (hepapoietin A; scatter factor) | 7776 | -0.004 | -0.0765 | No |
| 70 | IFNA2 | IFNA2 Entrez,  Source | interferon, alpha 2 | 7881 | -0.006 | -0.0817 | No |
| 71 | CSF1 | CSF1 Entrez,  Source | colony stimulating factor 1 (macrophage) | 7981 | -0.007 | -0.0866 | No |
| 72 | CXCL5 | CXCL5 Entrez,  Source | chemokine (C-X-C motif) ligand 5 | 8038 | -0.007 | -0.0893 | No |
| 73 | TNFRSF18 | TNFRSF18 Entrez,  Source | tumor necrosis factor receptor superfamily, member 18 | 8053 | -0.007 | -0.0898 | No |
| 74 | IL2RB | IL2RB Entrez,  Source | interleukin 2 receptor, beta | 8166 | -0.009 | -0.0953 | No |
| 75 | CCL21 | CCL21 Entrez,  Source | chemokine (C-C motif) ligand 21 | 8207 | -0.009 | -0.0970 | No |
| 76 | GH1 | GH1 Entrez,  Source | growth hormone 1 | 8293 | -0.010 | -0.1011 | No |
| 77 | TNFRSF8 | TNFRSF8 Entrez,  Source | tumor necrosis factor receptor superfamily, member 8 | 8401 | -0.012 | -0.1063 | No |
| 78 | FLT1 | FLT1 Entrez,  Source | fms-related tyrosine kinase 1 (vascular endothelial growth factor/vascular permeability factor receptor) | 8576 | -0.014 | -0.1148 | No |
| 79 | IL4R | IL4R Entrez,  Source | interleukin 4 receptor | 8654 | -0.014 | -0.1184 | No |
| 80 | CCL4 | CCL4 Entrez,  Source | chemokine (C-C motif) ligand 4 | 9085 | -0.020 | -0.1400 | No |
| 81 | TNFSF13B | TNFSF13B Entrez,  Source | tumor necrosis factor (ligand) superfamily, member 13b | 9208 | -0.021 | -0.1456 | No |
| 82 | IFNAR2 | IFNAR2 Entrez,  Source | interferon (alpha, beta and omega) receptor 2 | 9234 | -0.021 | -0.1462 | No |
| 83 | CXCL6 | CXCL6 Entrez,  Source | chemokine (C-X-C motif) ligand 6 (granulocyte chemotactic protein 2) | 9239 | -0.021 | -0.1457 | No |
| 84 | TNFRSF11A | TNFRSF11A Entrez,  Source | tumor necrosis factor receptor superfamily, member 11a, NFKB activator | 9549 | -0.025 | -0.1609 | No |
| 85 | ACVR1B | ACVR1B Entrez,  Source | activin A receptor, type IB | 9591 | -0.025 | -0.1622 | No |
| 86 | IL11 | IL11 Entrez,  Source | interleukin 11 | 9643 | -0.026 | -0.1640 | No |
| 87 | CCL18 | CCL18 Entrez,  Source | chemokine (C-C motif) ligand 18 (pulmonary and activation-regulated) | 9666 | -0.026 | -0.1643 | No |
| 88 | TPO | TPO Entrez,  Source | thyroid peroxidase | 9693 | -0.027 | -0.1648 | No |
| 89 | MPL | MPL Entrez,  Source | myeloproliferative leukemia virus oncogene | 9747 | -0.027 | -0.1667 | No |
| 90 | FLT4 | FLT4 Entrez,  Source | fms-related tyrosine kinase 4 | 9780 | -0.028 | -0.1674 | No |
| 91 | IL6 | IL6 Entrez,  Source | interleukin 6 (interferon, beta 2) | 9785 | -0.028 | -0.1668 | No |
| 92 | CCL27 | CCL27 Entrez,  Source | chemokine (C-C motif) ligand 27 | 10048 | -0.030 | -0.1793 | No |
| 93 | CSF1R | CSF1R Entrez,  Source | colony stimulating factor 1 receptor, formerly McDonough feline sarcoma viral (v-fms) oncogene homolog | 10135 | -0.032 | -0.1828 | No |
| 94 | CCL7 | CCL7 Entrez,  Source | chemokine (C-C motif) ligand 7 | 10232 | -0.033 | -0.1867 | No |
| 95 | EDA2R | EDA2R Entrez,  Source | ectodysplasin A2 receptor | 10276 | -0.033 | -0.1878 | No |
| 96 | CXCL14 | CXCL14 Entrez,  Source | chemokine (C-X-C motif) ligand 14 | 10353 | -0.034 | -0.1906 | No |
| 97 | ACVR2A | ACVR2A Entrez,  Source | activin A receptor, type IIA | 10420 | -0.035 | -0.1929 | No |
| 98 | CXCR3 | CXCR3 Entrez,  Source | chemokine (C-X-C motif) receptor 3 | 10530 | -0.036 | -0.1974 | No |
| 99 | CCR2 | CCR2 Entrez,  Source | chemokine (C-C motif) receptor 2 | 10543 | -0.036 | -0.1969 | No |
| 100 | IL8 | IL8 Entrez,  Source | interleukin 8 | 10620 | -0.037 | -0.1996 | No |
| 101 | CXCL10 | CXCL10 Entrez,  Source | chemokine (C-X-C motif) ligand 10 | 10844 | -0.040 | -0.2098 | No |
| 102 | CXCL9 | CXCL9 Entrez,  Source | chemokine (C-X-C motif) ligand 9 | 10913 | -0.041 | -0.2120 | No |
| 103 | TNFRSF13C | TNFRSF13C Entrez,  Source | tumor necrosis factor receptor superfamily, member 13C | 11014 | -0.042 | -0.2158 | No |
| 104 | LTBR | LTBR Entrez,  Source | lymphotoxin beta receptor (TNFR superfamily, member 3) | 11228 | -0.045 | -0.2254 | No |
| 105 | TNFRSF4 | TNFRSF4 Entrez,  Source | tumor necrosis factor receptor superfamily, member 4 | 11266 | -0.045 | -0.2258 | No |
| 106 | IL3RA | IL3RA Entrez,  Source | interleukin 3 receptor, alpha (low affinity) | 11277 | -0.046 | -0.2249 | No |
| 107 | KIT | KIT Entrez,  Source | v-kit Hardy-Zuckerman 4 feline sarcoma viral oncogene homolog | 11285 | -0.046 | -0.2238 | No |
| 108 | IL17RB | IL17RB Entrez,  Source | interleukin 17 receptor B | 11294 | -0.046 | -0.2227 | No |
| 109 | CCR9 | CCR9 Entrez,  Source | chemokine (C-C motif) receptor 9 | 11413 | -0.047 | -0.2273 | No |
| 110 | IFNAR1 | IFNAR1 Entrez,  Source | interferon (alpha, beta and omega) receptor 1 | 11463 | -0.048 | -0.2283 | No |
| 111 | CCR8 | CCR8 Entrez,  Source | chemokine (C-C motif) receptor 8 | 11537 | -0.049 | -0.2305 | No |
| 112 | LTB | LTB Entrez,  Source | lymphotoxin beta (TNF superfamily, member 3) | 11666 | -0.050 | -0.2355 | No |
| 113 | CCL24 | CCL24 Entrez,  Source | chemokine (C-C motif) ligand 24 | 11740 | -0.051 | -0.2376 | No |
| 114 | IFNA6 | IFNA6 Entrez,  Source | interferon, alpha 6 | 11757 | -0.051 | -0.2368 | No |
| 115 | IL22RA2 | IL22RA2 Entrez,  Source | interleukin 22 receptor, alpha 2 | 11759 | -0.051 | -0.2352 | No |
| 116 | IL17B | IL17B Entrez,  Source | interleukin 17B | 11769 | -0.051 | -0.2340 | No |
| 117 | ACVRL1 | ACVRL1 Entrez,  Source | activin A receptor type II-like 1 | 11885 | -0.053 | -0.2382 | No |
| 118 | CCR10 | CCR10 Entrez,  Source | chemokine (C-C motif) receptor 10 | 11957 | -0.054 | -0.2401 | No |
| 119 | IFNA8 | IFNA8 Entrez,  Source | interferon, alpha 8 | 12021 | -0.055 | -0.2416 | No |
| 120 | CCL11 | CCL11 Entrez,  Source | chemokine (C-C motif) ligand 11 | 12119 | -0.056 | -0.2448 | No |
| 121 | XCL1 | XCL1 Entrez,  Source | chemokine (C motif) ligand 1 | 12639 | -0.062 | -0.2697 | No |
| 122 | TNFSF12 | TNFSF12 Entrez,  Source | tumor necrosis factor (ligand) superfamily, member 12 | 12752 | -0.063 | -0.2734 | No |
| 123 | IFNB1 | IFNB1 Entrez,  Source | interferon, beta 1, fibroblast | 12765 | -0.064 | -0.2720 | No |
| 124 | EPO | EPO Entrez,  Source | erythropoietin | 12821 | -0.064 | -0.2727 | No |
| 125 | KDR | KDR Entrez,  Source | kinase insert domain receptor (a type III receptor tyrosine kinase) | 12913 | -0.065 | -0.2753 | No |
| 126 | CD40 | CD40 Entrez,  Source | CD40 molecule, TNF receptor superfamily member 5 | 12929 | -0.066 | -0.2740 | No |
| 127 | TNFRSF1B | TNFRSF1B Entrez,  Source | tumor necrosis factor receptor superfamily, member 1B | 12942 | -0.066 | -0.2725 | No |
| 128 | BMP7 | BMP7 Entrez,  Source | bone morphogenetic protein 7 (osteogenic protein 1) | 12982 | -0.066 | -0.2723 | No |
| 129 | IFNA21 | IFNA21 Entrez,  Source | interferon, alpha 21 | 12989 | -0.066 | -0.2705 | No |
| 130 | CCL22 | CCL22 Entrez,  Source | chemokine (C-C motif) ligand 22 | 13082 | -0.068 | -0.2731 | No |
| 131 | CCL19 | CCL19 Entrez,  Source | chemokine (C-C motif) ligand 19 | 13151 | -0.069 | -0.2744 | No |
| 132 | PRLR | PRLR Entrez,  Source | prolactin receptor | 13287 | -0.070 | -0.2791 | No |
| 133 | IL3 | IL3 Entrez,  Source | interleukin 3 (colony-stimulating factor, multiple) | 13294 | -0.071 | -0.2771 | No |
| 134 | INHBB | INHBB Entrez,  Source | inhibin, beta B (activin AB beta polypeptide) | 13309 | -0.071 | -0.2755 | No |
| 135 | TGFB1 | TGFB1 Entrez,  Source | transforming growth factor, beta 1 (Camurati-Engelmann disease) | 13436 | -0.072 | -0.2797 | No |
| 136 | IL21R | IL21R Entrez,  Source | interleukin 21 receptor | 13479 | -0.073 | -0.2795 | No |
| 137 | TNFRSF10D | TNFRSF10D Entrez,  Source | tumor necrosis factor receptor superfamily, member 10d, decoy with truncated death domain | 13530 | -0.074 | -0.2797 | No |
| 138 | CRLF2 | CRLF2 Entrez,  Source | cytokine receptor-like factor 2 | 13588 | -0.074 | -0.2803 | No |
| 139 | GDF5 | GDF5 Entrez,  Source | growth differentiation factor 5 (cartilage-derived morphogenetic protein-1) | 13619 | -0.075 | -0.2794 | No |
| 140 | TNFRSF13B | TNFRSF13B Entrez,  Source | tumor necrosis factor receptor superfamily, member 13B | 13649 | -0.075 | -0.2785 | No |
| 141 | TNFSF11 | TNFSF11 Entrez,  Source | tumor necrosis factor (ligand) superfamily, member 11 | 13694 | -0.076 | -0.2783 | No |
| 142 | GHR | GHR Entrez,  Source | growth hormone receptor | 13857 | -0.078 | -0.2842 | No |
| 143 | IFNK | IFNK Entrez,  Source | interferon, kappa | 13888 | -0.078 | -0.2832 | No |
| 144 | CSF3R | CSF3R Entrez,  Source | colony stimulating factor 3 receptor (granulocyte) | 13993 | -0.080 | -0.2860 | No |
| 145 | IL1R1 | IL1R1 Entrez,  Source | interleukin 1 receptor, type I | 14058 | -0.080 | -0.2867 | No |
| 146 | FLT3 | FLT3 Entrez,  Source | fms-related tyrosine kinase 3 | 14171 | -0.082 | -0.2899 | Yes |
| 147 | IL26 | IL26 Entrez,  Source | interleukin 26 | 14172 | -0.082 | -0.2872 | Yes |
| 148 | TGFBR1 | TGFBR1 Entrez,  Source | transforming growth factor, beta receptor I (activin A receptor type II-like kinase, 53kDa) | 14193 | -0.082 | -0.2856 | Yes |
| 149 | EPOR | EPOR Entrez,  Source | erythropoietin receptor | 14204 | -0.082 | -0.2834 | Yes |
| 150 | CSF2RB | CSF2RB Entrez,  Source | colony stimulating factor 2 receptor, beta, low-affinity (granulocyte-macrophage) | 14232 | -0.083 | -0.2821 | Yes |
| 151 | CX3CR1 | CX3CR1 Entrez,  Source | chemokine (C-X3-C motif) receptor 1 | 14238 | -0.083 | -0.2797 | Yes |
| 152 | INHBA | INHBA Entrez,  Source | inhibin, beta A (activin A, activin AB alpha polypeptide) | 14258 | -0.083 | -0.2780 | Yes |
| 153 | IL28A | IL28A Entrez,  Source | interleukin 28A (interferon, lambda 2) | 14289 | -0.083 | -0.2769 | Yes |
| 154 | PF4 | PF4 Entrez,  Source | platelet factor 4 (chemokine (C-X-C motif) ligand 4) | 14392 | -0.085 | -0.2794 | Yes |
| 155 | IL2 | IL2 Entrez,  Source | interleukin 2 | 14448 | -0.085 | -0.2795 | Yes |
| 156 | CXCL12 | CXCL12 Entrez,  Source | chemokine (C-X-C motif) ligand 12 (stromal cell-derived factor 1) | 14474 | -0.086 | -0.2780 | Yes |
| 157 | CCL17 | CCL17 Entrez,  Source | chemokine (C-C motif) ligand 17 | 14541 | -0.087 | -0.2786 | Yes |
| 158 | LTA | LTA Entrez,  Source | lymphotoxin alpha (TNF superfamily, member 1) | 14593 | -0.087 | -0.2784 | Yes |
| 159 | CCL13 | CCL13 Entrez,  Source | chemokine (C-C motif) ligand 13 | 14605 | -0.088 | -0.2761 | Yes |
| 160 | CD40LG | CD40LG Entrez,  Source | CD40 ligand (TNF superfamily, member 5, hyper-IgM syndrome) | 14658 | -0.088 | -0.2759 | Yes |
| 161 | IL28RA | IL28RA Entrez,  Source | interleukin 28 receptor, alpha (interferon, lambda receptor) | 14731 | -0.090 | -0.2768 | Yes |
| 162 | TNFSF13 | TNFSF13 Entrez,  Source | tumor necrosis factor (ligand) superfamily, member 13 | 14860 | -0.091 | -0.2804 | Yes |
| 163 | PDGFRA | PDGFRA Entrez,  Source | platelet-derived growth factor receptor, alpha polypeptide | 14940 | -0.093 | -0.2815 | Yes |
| 164 | CXCL11 | CXCL11 Entrez,  Source | chemokine (C-X-C motif) ligand 11 | 14991 | -0.094 | -0.2811 | Yes |
| 165 | CCL20 | CCL20 Entrez,  Source | chemokine (C-C motif) ligand 20 | 15050 | -0.094 | -0.2810 | Yes |
| 166 | BMP2 | BMP2 Entrez,  Source | bone morphogenetic protein 2 | 15195 | -0.097 | -0.2853 | Yes |
| 167 | TGFB3 | TGFB3 Entrez,  Source | transforming growth factor, beta 3 | 15224 | -0.098 | -0.2836 | Yes |
| 168 | CSF2RA | CSF2RA Entrez,  Source | colony stimulating factor 2 receptor, alpha, low-affinity (granulocyte-macrophage) | 15267 | -0.098 | -0.2826 | Yes |
| 169 | IL10RA | IL10RA Entrez,  Source | interleukin 10 receptor, alpha | 15300 | -0.099 | -0.2810 | Yes |
| 170 | CCL5 | CCL5 Entrez,  Source | chemokine (C-C motif) ligand 5 | 15330 | -0.099 | -0.2793 | Yes |
| 171 | CCL25 | CCL25 Entrez,  Source | chemokine (C-C motif) ligand 25 | 15333 | -0.099 | -0.2762 | Yes |
| 172 | CCR4 | CCR4 Entrez,  Source | chemokine (C-C motif) receptor 4 | 15352 | -0.100 | -0.2739 | Yes |
| 173 | PDGFRB | PDGFRB Entrez,  Source | platelet-derived growth factor receptor, beta polypeptide | 15354 | -0.100 | -0.2707 | Yes |
| 174 | IL5RA | IL5RA Entrez,  Source | interleukin 5 receptor, alpha | 15488 | -0.102 | -0.2743 | Yes |
| 175 | CCL8 | CCL8 Entrez,  Source | chemokine (C-C motif) ligand 8 | 15522 | -0.102 | -0.2727 | Yes |
| 176 | IL1B | IL1B Entrez,  Source | interleukin 1, beta | 15552 | -0.103 | -0.2709 | Yes |
| 177 | XCR1 | XCR1 Entrez,  Source | chemokine (C motif) receptor 1 | 15662 | -0.105 | -0.2731 | Yes |
| 178 | TNFSF8 | TNFSF8 Entrez,  Source | tumor necrosis factor (ligand) superfamily, member 8 | 15710 | -0.106 | -0.2721 | Yes |
| 179 | TNFSF18 | TNFSF18 Entrez,  Source | tumor necrosis factor (ligand) superfamily, member 18 | 15855 | -0.109 | -0.2760 | Yes |
| 180 | CNTFR | CNTFR Entrez,  Source | ciliary neurotrophic factor receptor | 15864 | -0.109 | -0.2729 | Yes |
| 181 | OSM | OSM Entrez,  Source | oncostatin M | 15887 | -0.109 | -0.2705 | Yes |
| 182 | CTF1 | CTF1 Entrez,  Source | cardiotrophin 1 | 15899 | -0.109 | -0.2676 | Yes |
| 183 | IFNW1 | IFNW1 Entrez,  Source | interferon, omega 1 | 15983 | -0.111 | -0.2683 | Yes |
| 184 | IFNA10 | IFNA10 Entrez,  Source | interferon, alpha 10 | 16258 | -0.116 | -0.2787 | Yes |
| 185 | IL29 | IL29 Entrez,  Source | interleukin 29 (interferon, lambda 1) | 16307 | -0.117 | -0.2774 | Yes |
| 186 | EDA | EDA Entrez,  Source | ectodysplasin A | 16309 | -0.117 | -0.2736 | Yes |
| 187 | AMH | AMH Entrez,  Source | anti-Mullerian hormone | 16369 | -0.118 | -0.2728 | Yes |
| 188 | IL13 | IL13 Entrez,  Source | interleukin 13 | 16498 | -0.121 | -0.2755 | Yes |
| 189 | LEP | LEP Entrez,  Source | leptin (obesity homolog, mouse) | 16530 | -0.121 | -0.2732 | Yes |
| 190 | IL20 | IL20 Entrez,  Source | interleukin 20 | 16555 | -0.122 | -0.2705 | Yes |
| 191 | CXCR6 | CXCR6 Entrez,  Source | chemokine (C-X-C motif) receptor 6 | 16585 | -0.122 | -0.2680 | Yes |
| 192 | FLT3LG | FLT3LG Entrez,  Source | fms-related tyrosine kinase 3 ligand | 16636 | -0.124 | -0.2666 | Yes |
| 193 | TNFRSF25 | TNFRSF25 Entrez,  Source | tumor necrosis factor receptor superfamily, member 25 | 16657 | -0.124 | -0.2636 | Yes |
| 194 | IL12RB1 | IL12RB1 Entrez,  Source | interleukin 12 receptor, beta 1 | 16663 | -0.125 | -0.2598 | Yes |
| 195 | FIGF | FIGF Entrez,  Source | c-fos induced growth factor (vascular endothelial growth factor D) | 16676 | -0.125 | -0.2564 | Yes |
| 196 | TNFRSF10C | TNFRSF10C Entrez,  Source | tumor necrosis factor receptor superfamily, member 10c, decoy without an intracellular domain | 16696 | -0.125 | -0.2533 | Yes |
| 197 | XCL2 | XCL2 Entrez,  Source | chemokine (C motif) ligand 2 | 16704 | -0.125 | -0.2496 | Yes |
| 198 | IFNA5 | IFNA5 Entrez,  Source | interferon, alpha 5 | 16708 | -0.126 | -0.2457 | Yes |
| 199 | AMHR2 | AMHR2 Entrez,  Source | anti-Mullerian hormone receptor, type II | 16729 | -0.126 | -0.2427 | Yes |
| 200 | IL5 | IL5 Entrez,  Source | interleukin 5 (colony-stimulating factor, eosinophil) | 16732 | -0.126 | -0.2387 | Yes |
| 201 | IL23A | IL23A Entrez,  Source | interleukin 23, alpha subunit p19 | 16829 | -0.128 | -0.2395 | Yes |
| 202 | IL11RA | IL11RA Entrez,  Source | interleukin 11 receptor, alpha | 16989 | -0.132 | -0.2434 | Yes |
| 203 | IFNA7 | IFNA7 Entrez,  Source | interferon, alpha 7 | 17022 | -0.133 | -0.2408 | Yes |
| 204 | TNF | TNF Entrez,  Source | tumor necrosis factor (TNF superfamily, member 2) | 17349 | -0.143 | -0.2530 | Yes |
| 205 | CSF3 | CSF3 Entrez,  Source | colony stimulating factor 3 (granulocyte) | 17402 | -0.145 | -0.2510 | Yes |
| 206 | IL2RA | IL2RA Entrez,  Source | interleukin 2 receptor, alpha | 17457 | -0.147 | -0.2490 | Yes |
| 207 | EGF | EGF Entrez,  Source | epidermal growth factor (beta-urogastrone) | 17577 | -0.151 | -0.2503 | Yes |
| 208 | PRL | PRL Entrez,  Source | prolactin | 17664 | -0.153 | -0.2498 | Yes |
| 209 | IL18R1 | IL18R1 Entrez,  Source | interleukin 18 receptor 1 | 17876 | -0.162 | -0.2555 | Yes |
| 210 | IFNA1 | IFNA1 Entrez,  Source | interferon, alpha 1 | 18074 | -0.169 | -0.2602 | Yes |
| 211 | IFNA17 | IFNA17 Entrez,  Source | interferon, alpha 17 | 18148 | -0.173 | -0.2583 | Yes |
| 212 | IL12A | IL12A Entrez,  Source | interleukin 12A (natural killer cell stimulatory factor 1, cytotoxic lymphocyte maturation factor 1, p35) | 18214 | -0.177 | -0.2560 | Yes |
| 213 | IFNA14 | IFNA14 Entrez,  Source | interferon, alpha 14 | 18299 | -0.181 | -0.2544 | Yes |
| 214 | IL18RAP | IL18RAP Entrez,  Source | interleukin 18 receptor accessory protein | 18322 | -0.182 | -0.2497 | Yes |
| 215 | VEGFC | VEGFC Entrez,  Source | vascular endothelial growth factor C | 18328 | -0.182 | -0.2440 | Yes |
| 216 | PF4V1 | PF4V1 Entrez,  Source | platelet factor 4 variant 1 | 18332 | -0.183 | -0.2383 | Yes |
| 217 | IFNA16 | IFNA16 Entrez,  Source | interferon, alpha 16 | 18386 | -0.186 | -0.2350 | Yes |
| 218 | IL4 | IL4 Entrez,  Source | interleukin 4 | 18404 | -0.187 | -0.2298 | Yes |
| 219 | IL1R2 | IL1R2 Entrez,  Source | interleukin 1 receptor, type II | 18544 | -0.197 | -0.2306 | Yes |
| 220 | IL17A | IL17A Entrez,  Source | interleukin 17A | 18660 | -0.205 | -0.2299 | Yes |
| 221 | CCL1 | CCL1 Entrez,  Source | chemokine (C-C motif) ligand 1 | 18663 | -0.205 | -0.2234 | Yes |
| 222 | TNFSF4 | TNFSF4 Entrez,  Source | tumor necrosis factor (ligand) superfamily, member 4 (tax-transcriptionally activated glycoprotein 1, 34kDa) | 18710 | -0.209 | -0.2190 | Yes |
| 223 | CXCL13 | CXCL13 Entrez,  Source | chemokine (C-X-C motif) ligand 13 (B-cell chemoattractant) | 18762 | -0.213 | -0.2147 | Yes |
| 224 | FASLG | FASLG Entrez,  Source | Fas ligand (TNF superfamily, member 6) | 18956 | -0.237 | -0.2170 | Yes |
| 225 | IL2RG | IL2RG Entrez,  Source | interleukin 2 receptor, gamma (severe combined immunodeficiency) | 19052 | -0.252 | -0.2137 | Yes |
| 226 | IL9 | IL9 Entrez,  Source | interleukin 9 | 19077 | -0.256 | -0.2067 | Yes |
| 227 | KITLG | KITLG Entrez,  Source | KIT ligand | 19098 | -0.259 | -0.1993 | Yes |
| 228 | IL19 | IL19 Entrez,  Source | interleukin 19 | 19135 | -0.269 | -0.1925 | Yes |
| 229 | IFNA4 | IFNA4 Entrez,  Source | interferon, alpha 4 | 19182 | -0.280 | -0.1858 | Yes |
| 230 | TNFRSF9 | TNFRSF9 Entrez,  Source | tumor necrosis factor receptor superfamily, member 9 | 19230 | -0.297 | -0.1786 | Yes |
| 231 | CCR7 | CCR7 Entrez,  Source | chemokine (C-C motif) receptor 7 | 19234 | -0.298 | -0.1691 | Yes |
| 232 | ACVR2B | ACVR2B Entrez,  Source | activin A receptor, type IIB | 19305 | -0.326 | -0.1621 | Yes |
| 233 | IL12RB2 | IL12RB2 Entrez,  Source | interleukin 12 receptor, beta 2 | 19311 | -0.330 | -0.1517 | Yes |
| 234 | IL24 | IL24 Entrez,  Source | interleukin 24 | 19318 | -0.335 | -0.1411 | Yes |
| 235 | CCR3 | CCR3 Entrez,  Source | chemokine (C-C motif) receptor 3 | 19395 | -0.401 | -0.1320 | Yes |
| 236 | IL10 | IL10 Entrez,  Source | interleukin 10 | 19415 | -0.420 | -0.1194 | Yes |
| 237 | CXCR4 | CXCR4 Entrez,  Source | chemokine (C-X-C motif) receptor 4 | 19423 | -0.428 | -0.1059 | Yes |
| 238 | BMPR1B | BMPR1B Entrez,  Source | bone morphogenetic protein receptor, type IB | 19433 | -0.437 | -0.0922 | Yes |
| 239 | IL6R | IL6R Entrez,  Source | interleukin 6 receptor | 19442 | -0.446 | -0.0781 | Yes |
| 240 | EDAR | EDAR Entrez,  Source | ectodysplasin A receptor | 19471 | -0.487 | -0.0638 | Yes |
| 241 | INHBE | INHBE Entrez,  Source | inhibin, beta E | 19500 | -0.569 | -0.0468 | Yes |
| 242 | TNFRSF19 | TNFRSF19 Entrez,  Source | tumor necrosis factor receptor superfamily, member 19 | 19521 | -0.649 | -0.0268 | Yes |
| 243 | TNFRSF11B | TNFRSF11B Entrez,  Source | tumor necrosis factor receptor superfamily, member 11b (osteoprotegerin) | 19544 | -0.881 | 0.0006 | Yes |
Table: GSEA details [plain text format]

  

Fig 2: KEGG\_CYTOKINE\_CYTOKINE\_RECEPTOR\_INTERACTION      
 Blue-Pink O' Gram in the Space of the Analyzed GeneSet

  

Fig 3: KEGG\_CYTOKINE\_CYTOKINE\_RECEPTOR\_INTERACTION: Random ES distribution      
 Gene set null distribution of ES for **KEGG\_CYTOKINE\_CYTOKINE\_RECEPTOR\_INTERACTION**

  
