# Supplementary material for: Comprehensive analysis of β-catenin target genes in colorectal carcinoma cell lines with deregulated Wnt/β-catenin signaling
Source: BMC Genomics. 2014 Jan 28;15:74. doi: 10.1186/1471-2164-15-74 (PMC3909937; doi:10.1186/1471-2164-15-74)
Supplement: Additional file 5 — GSEA analysis using the KEGG pathway database. This zipped file contains confirming data of the GSEA analysis. The names of the directories containing the files were composed of the term ‘GSEA’, the name of the cell line, e.g. DLD1, SW480, or LS174T, and the pathway database (KEGG). Please use a web browser to view the files with the name ‘index.html’ in the corresponding directories to start exploring the data. [file 1471-2164-15-74-S5.zip › GSEA KEGG SW480/KEGG_DNA_REPLICATION.html]

Details for gene set KEGG\_DNA\_REPLICATION[GSEA]

|  || Dataset | SW480\_collapsed\_to\_symbols.class.cls#b\_versus\_bg.class.cls#b\_versus\_bg\_repos |
| Phenotype | class.cls#b\_versus\_bg\_repos |
| Upregulated in class | 1 |
| GeneSet | KEGG\_DNA\_REPLICATION |
| Enrichment Score (ES) | 0.57629526 |
| Normalized Enrichment Score (NES) | 1.8959306 |
| Nominal p-value | 0.0025188916 |
| FDR q-value | 0.03490825 |
| FWER p-Value | 0.071 |
Table: GSEA Results Summary

  

Fig 1: Enrichment plot: KEGG\_DNA\_REPLICATION      
 Profile of the Running ES Score & Positions of GeneSet Members on the Rank Ordered List

  

| PROBE | GENE SYMBOL | GENE\_TITLE | RANK IN GENE LIST | RANK METRIC SCORE | RUNNING ES | CORE ENRICHMENT || 1 | POLD4 | POLD4 Entrez,  Source | polymerase (DNA-directed), delta 4 | 621 | 0.247 | 0.0909 | Yes |
| 2 | PRIM1 | PRIM1 Entrez,  Source | primase, polypeptide 1, 49kDa | 1360 | 0.158 | 0.1315 | Yes |
| 3 | RPA1 | RPA1 Entrez,  Source | replication protein A1, 70kDa | 1399 | 0.155 | 0.2065 | Yes |
| 4 | POLE2 | POLE2 Entrez,  Source | polymerase (DNA directed), epsilon 2 (p59 subunit) | 1885 | 0.126 | 0.2442 | Yes |
| 5 | LIG1 | LIG1 Entrez,  Source | ligase I, DNA, ATP-dependent | 2306 | 0.107 | 0.2760 | Yes |
| 6 | MCM5 | MCM5 Entrez,  Source | MCM5 minichromosome maintenance deficient 5, cell division cycle 46 (S. cerevisiae) | 2591 | 0.096 | 0.3091 | Yes |
| 7 | RNASEH2A | RNASEH2A Entrez,  Source | ribonuclease H2, subunit A | 2597 | 0.095 | 0.3562 | Yes |
| 8 | RFC2 | RFC2 Entrez,  Source | replication factor C (activator 1) 2, 40kDa | 2739 | 0.091 | 0.3940 | Yes |
| 9 | RNASEH2C | RNASEH2C Entrez,  Source | ribonuclease H2, subunit C | 3254 | 0.075 | 0.4048 | Yes |
| 10 | RPA3 | RPA3 Entrez,  Source | replication protein A3, 14kDa | 3777 | 0.061 | 0.4082 | Yes |
| 11 | RFC5 | RFC5 Entrez,  Source | replication factor C (activator 1) 5, 36.5kDa | 3791 | 0.060 | 0.4376 | Yes |
| 12 | POLE3 | POLE3 Entrez,  Source | polymerase (DNA directed), epsilon 3 (p17 subunit) | 3901 | 0.058 | 0.4607 | Yes |
| 13 | RNASEH2B | RNASEH2B Entrez,  Source | ribonuclease H2, subunit B | 4006 | 0.055 | 0.4830 | Yes |
| 14 | POLD3 | POLD3 Entrez,  Source | polymerase (DNA-directed), delta 3, accessory subunit | 4089 | 0.054 | 0.5054 | Yes |
| 15 | POLA1 | POLA1 Entrez,  Source | polymerase (DNA directed), alpha 1 | 4424 | 0.047 | 0.5115 | Yes |
| 16 | MCM4 | MCM4 Entrez,  Source | MCM4 minichromosome maintenance deficient 4 (S. cerevisiae) | 4682 | 0.041 | 0.5188 | Yes |
| 17 | PCNA | PCNA Entrez,  Source | proliferating cell nuclear antigen | 4937 | 0.036 | 0.5239 | Yes |
| 18 | MCM7 | MCM7 Entrez,  Source | MCM7 minichromosome maintenance deficient 7 (S. cerevisiae) | 5140 | 0.033 | 0.5299 | Yes |
| 19 | FEN1 | FEN1 Entrez,  Source | flap structure-specific endonuclease 1 | 5176 | 0.032 | 0.5441 | Yes |
| 20 | RFC4 | RFC4 Entrez,  Source | replication factor C (activator 1) 4, 37kDa | 5300 | 0.030 | 0.5528 | Yes |
| 21 | MCM6 | MCM6 Entrez,  Source | MCM6 minichromosome maintenance deficient 6 (MIS5 homolog, S. pombe) (S. cerevisiae) | 5367 | 0.029 | 0.5639 | Yes |
| 22 | RFC1 | RFC1 Entrez,  Source | replication factor C (activator 1) 1, 145kDa | 5403 | 0.029 | 0.5763 | Yes |
| 23 | POLA2 | POLA2 Entrez,  Source | polymerase (DNA directed), alpha 2 (70kD subunit) | 6003 | 0.019 | 0.5553 | No |
| 24 | MCM2 | MCM2 Entrez,  Source | MCM2 minichromosome maintenance deficient 2, mitotin (S. cerevisiae) | 6778 | 0.009 | 0.5200 | No |
| 25 | POLD2 | POLD2 Entrez,  Source | polymerase (DNA directed), delta 2, regulatory subunit 50kDa | 7071 | 0.005 | 0.5073 | No |
| 26 | POLE | POLE Entrez,  Source | polymerase (DNA directed), epsilon | 7481 | -0.000 | 0.4866 | No |
| 27 | RFC3 | RFC3 Entrez,  Source | replication factor C (activator 1) 3, 38kDa | 7608 | -0.002 | 0.4811 | No |
| 28 | RPA2 | RPA2 Entrez,  Source | replication protein A2, 32kDa | 7783 | -0.004 | 0.4743 | No |
| 29 | MCM3 | MCM3 Entrez,  Source | MCM3 minichromosome maintenance deficient 3 (S. cerevisiae) | 8188 | -0.009 | 0.4581 | No |
| 30 | SSBP1 | SSBP1 Entrez,  Source | single-stranded DNA binding protein 1 | 8689 | -0.015 | 0.4399 | No |
| 31 | RNASEH1 | RNASEH1 Entrez,  Source | ribonuclease H1 | 9150 | -0.020 | 0.4265 | No |
| 32 | POLD1 | POLD1 Entrez,  Source | polymerase (DNA directed), delta 1, catalytic subunit 125kDa | 9782 | -0.028 | 0.4078 | No |
| 33 | POLE4 | POLE4 Entrez,  Source | polymerase (DNA-directed), epsilon 4 (p12 subunit) | 11351 | -0.046 | 0.3506 | No |
| 34 | RPA4 | RPA4 Entrez,  Source | replication protein A4, 34kDa | 17245 | -0.140 | 0.1184 | No |
Table: GSEA details [plain text format]

  

Fig 2: KEGG\_DNA\_REPLICATION      
 Blue-Pink O' Gram in the Space of the Analyzed GeneSet

  

Fig 3: KEGG\_DNA\_REPLICATION: Random ES distribution      
 Gene set null distribution of ES for **KEGG\_DNA\_REPLICATION**

  
