# Supplementary material for: Comprehensive analysis of β-catenin target genes in colorectal carcinoma cell lines with deregulated Wnt/β-catenin signaling
Source: BMC Genomics. 2014 Jan 28;15:74. doi: 10.1186/1471-2164-15-74 (PMC3909937; doi:10.1186/1471-2164-15-74)
Supplement: Additional file 5 — GSEA analysis using the KEGG pathway database. This zipped file contains confirming data of the GSEA analysis. The names of the directories containing the files were composed of the term ‘GSEA’, the name of the cell line, e.g. DLD1, SW480, or LS174T, and the pathway database (KEGG). Please use a web browser to view the files with the name ‘index.html’ in the corresponding directories to start exploring the data. [file 1471-2164-15-74-S5.zip › GSEA KEGG SW480/KEGG_ECM_RECEPTOR_INTERACTION.html]

Details for gene set KEGG\_ECM\_RECEPTOR\_INTERACTION[GSEA]

|  || Dataset | SW480\_collapsed\_to\_symbols.class.cls#b\_versus\_bg.class.cls#b\_versus\_bg\_repos |
| Phenotype | class.cls#b\_versus\_bg\_repos |
| Upregulated in class | 1 |
| GeneSet | KEGG\_ECM\_RECEPTOR\_INTERACTION |
| Enrichment Score (ES) | 0.4737841 |
| Normalized Enrichment Score (NES) | 1.838049 |
| Nominal p-value | 0.0 |
| FDR q-value | 0.03720878 |
| FWER p-Value | 0.12 |
Table: GSEA Results Summary

  

Fig 1: Enrichment plot: KEGG\_ECM\_RECEPTOR\_INTERACTION      
 Profile of the Running ES Score & Positions of GeneSet Members on the Rank Ordered List

  

| PROBE | GENE SYMBOL | GENE\_TITLE | RANK IN GENE LIST | RANK METRIC SCORE | RUNNING ES | CORE ENRICHMENT || 1 | ITGB8 | ITGB8 Entrez,  Source | integrin, beta 8 | 1 | 1.743 | 0.1363 | Yes |
| 2 | TNC | TNC Entrez,  Source | tenascin C (hexabrachion) | 5 | 0.981 | 0.2128 | Yes |
| 3 | THBS1 | THBS1 Entrez,  Source | thrombospondin 1 | 52 | 0.639 | 0.2604 | Yes |
| 4 | COL6A1 | COL6A1 Entrez,  Source | collagen, type VI, alpha 1 | 126 | 0.471 | 0.2936 | Yes |
| 5 | ITGA3 | ITGA3 Entrez,  Source | integrin, alpha 3 (antigen CD49C, alpha 3 subunit of VLA-3 receptor) | 134 | 0.458 | 0.3290 | Yes |
| 6 | FN1 | FN1 Entrez,  Source | fibronectin 1 | 145 | 0.442 | 0.3631 | Yes |
| 7 | ITGB4 | ITGB4 Entrez,  Source | integrin, beta 4 | 201 | 0.401 | 0.3916 | Yes |
| 8 | COL6A3 | COL6A3 Entrez,  Source | collagen, type VI, alpha 3 | 347 | 0.322 | 0.4093 | Yes |
| 9 | LAMA3 | LAMA3 Entrez,  Source | laminin, alpha 3 | 514 | 0.269 | 0.4218 | Yes |
| 10 | SDC3 | SDC3 Entrez,  Source | syndecan 3 (N-syndecan) | 533 | 0.264 | 0.4416 | Yes |
| 11 | SDC4 | SDC4 Entrez,  Source | syndecan 4 (amphiglycan, ryudocan) | 795 | 0.218 | 0.4453 | Yes |
| 12 | ITGA6 | ITGA6 Entrez,  Source | integrin, alpha 6 | 1088 | 0.180 | 0.4444 | Yes |
| 13 | ITGB5 | ITGB5 Entrez,  Source | integrin, beta 5 | 1286 | 0.163 | 0.4470 | Yes |
| 14 | HMMR | HMMR Entrez,  Source | hyaluronan-mediated motility receptor (RHAMM) | 1342 | 0.159 | 0.4566 | Yes |
| 15 | LAMB1 | LAMB1 Entrez,  Source | laminin, beta 1 | 1397 | 0.155 | 0.4660 | Yes |
| 16 | COL4A1 | COL4A1 Entrez,  Source | collagen, type IV, alpha 1 | 1473 | 0.149 | 0.4738 | Yes |
| 17 | ITGA2 | ITGA2 Entrez,  Source | integrin, alpha 2 (CD49B, alpha 2 subunit of VLA-2 receptor) | 1768 | 0.131 | 0.4689 | No |
| 18 | ITGAV | ITGAV Entrez,  Source | integrin, alpha V (vitronectin receptor, alpha polypeptide, antigen CD51) | 2073 | 0.117 | 0.4625 | No |
| 19 | ITGB1 | ITGB1 Entrez,  Source | integrin, beta 1 (fibronectin receptor, beta polypeptide, antigen CD29 includes MDF2, MSK12) | 2321 | 0.107 | 0.4581 | No |
| 20 | ITGA10 | ITGA10 Entrez,  Source | integrin, alpha 10 | 2418 | 0.103 | 0.4613 | No |
| 21 | LAMA5 | LAMA5 Entrez,  Source | laminin, alpha 5 | 2487 | 0.100 | 0.4656 | No |
| 22 | COMP | COMP Entrez,  Source | cartilage oligomeric matrix protein | 2825 | 0.088 | 0.4552 | No |
| 23 | ITGB6 | ITGB6 Entrez,  Source | integrin, beta 6 | 2948 | 0.084 | 0.4555 | No |
| 24 | SPP1 | SPP1 Entrez,  Source | secreted phosphoprotein 1 (osteopontin, bone sialoprotein I, early T-lymphocyte activation 1) | 3129 | 0.078 | 0.4524 | No |
| 25 | LAMB2 | LAMB2 Entrez,  Source | laminin, beta 2 (laminin S) | 5499 | 0.027 | 0.3329 | No |
| 26 | DAG1 | DAG1 Entrez,  Source | dystroglycan 1 (dystrophin-associated glycoprotein 1) | 5918 | 0.021 | 0.3130 | No |
| 27 | CD47 | CD47 Entrez,  Source | CD47 molecule | 6084 | 0.018 | 0.3060 | No |
| 28 | GP1BA | GP1BA Entrez,  Source | glycoprotein Ib (platelet), alpha polypeptide | 6380 | 0.014 | 0.2919 | No |
| 29 | ITGA7 | ITGA7 Entrez,  Source | integrin, alpha 7 | 6518 | 0.012 | 0.2858 | No |
| 30 | COL5A2 | COL5A2 Entrez,  Source | collagen, type V, alpha 2 | 6595 | 0.011 | 0.2828 | No |
| 31 | VTN | VTN Entrez,  Source | vitronectin | 6767 | 0.009 | 0.2747 | No |
| 32 | SV2B | SV2B Entrez,  Source | synaptic vesicle glycoprotein 2B | 7412 | 0.000 | 0.2417 | No |
| 33 | COL1A1 | COL1A1 Entrez,  Source | collagen, type I, alpha 1 | 8461 | -0.012 | 0.1889 | No |
| 34 | CHAD | CHAD Entrez,  Source | chondroadherin | 8843 | -0.017 | 0.1706 | No |
| 35 | ITGA8 | ITGA8 Entrez,  Source | integrin, alpha 8 | 9908 | -0.029 | 0.1182 | No |
| 36 | LAMC1 | LAMC1 Entrez,  Source | laminin, gamma 1 (formerly LAMB2) | 9927 | -0.029 | 0.1196 | No |
| 37 | ITGA4 | ITGA4 Entrez,  Source | integrin, alpha 4 (antigen CD49D, alpha 4 subunit of VLA-4 receptor) | 10572 | -0.037 | 0.0894 | No |
| 38 | THBS3 | THBS3 Entrez,  Source | thrombospondin 3 | 10680 | -0.038 | 0.0869 | No |
| 39 | COL6A2 | COL6A2 Entrez,  Source | collagen, type VI, alpha 2 | 10684 | -0.038 | 0.0897 | No |
| 40 | COL11A2 | COL11A2 Entrez,  Source | collagen, type XI, alpha 2 | 10911 | -0.041 | 0.0813 | No |
| 41 | GP5 | GP5 Entrez,  Source | glycoprotein V (platelet) | 11007 | -0.042 | 0.0797 | No |
| 42 | VWF | VWF Entrez,  Source | von Willebrand factor | 11029 | -0.043 | 0.0820 | No |
| 43 | ITGA5 | ITGA5 Entrez,  Source | integrin, alpha 5 (fibronectin receptor, alpha polypeptide) | 11109 | -0.043 | 0.0813 | No |
| 44 | LAMB3 | LAMB3 Entrez,  Source | laminin, beta 3 | 11110 | -0.043 | 0.0847 | No |
| 45 | COL5A3 | COL5A3 Entrez,  Source | collagen, type V, alpha 3 | 11626 | -0.050 | 0.0622 | No |
| 46 | ITGB3 | ITGB3 Entrez,  Source | integrin, beta 3 (platelet glycoprotein IIIa, antigen CD61) | 11699 | -0.051 | 0.0624 | No |
| 47 | COL11A1 | COL11A1 Entrez,  Source | collagen, type XI, alpha 1 | 11776 | -0.051 | 0.0625 | No |
| 48 | GP9 | GP9 Entrez,  Source | glycoprotein IX (platelet) | 11791 | -0.052 | 0.0659 | No |
| 49 | LAMB4 | LAMB4 Entrez,  Source | laminin, beta 4 | 12018 | -0.055 | 0.0585 | No |
| 50 | SDC1 | SDC1 Entrez,  Source | syndecan 1 | 12054 | -0.055 | 0.0610 | No |
| 51 | HSPG2 | HSPG2 Entrez,  Source | heparan sulfate proteoglycan 2 (perlecan) | 12078 | -0.055 | 0.0642 | No |
| 52 | THBS4 | THBS4 Entrez,  Source | thrombospondin 4 | 12206 | -0.057 | 0.0621 | No |
| 53 | LAMC2 | LAMC2 Entrez,  Source | laminin, gamma 2 | 12750 | -0.063 | 0.0392 | No |
| 54 | COL3A1 | COL3A1 Entrez,  Source | collagen, type III, alpha 1 (Ehlers-Danlos syndrome type IV, autosomal dominant) | 13010 | -0.067 | 0.0311 | No |
| 55 | CD44 | CD44 Entrez,  Source | CD44 molecule (Indian blood group) | 13533 | -0.074 | 0.0100 | No |
| 56 | LAMA4 | LAMA4 Entrez,  Source | laminin, alpha 4 | 14214 | -0.082 | -0.0184 | No |
| 57 | COL4A6 | COL4A6 Entrez,  Source | collagen, type IV, alpha 6 | 14340 | -0.084 | -0.0183 | No |
| 58 | LAMA1 | LAMA1 Entrez,  Source | laminin, alpha 1 | 14411 | -0.085 | -0.0152 | No |
| 59 | TNR | TNR Entrez,  Source | tenascin R (restrictin, janusin) | 14441 | -0.085 | -0.0100 | No |
| 60 | ITGA2B | ITGA2B Entrez,  Source | integrin, alpha 2b (platelet glycoprotein IIb of IIb/IIIa complex, antigen CD41) | 15161 | -0.097 | -0.0394 | No |
| 61 | COL1A2 | COL1A2 Entrez,  Source | collagen, type I, alpha 2 | 15172 | -0.097 | -0.0323 | No |
| 62 | RELN | RELN Entrez,  Source | reelin | 15378 | -0.100 | -0.0350 | No |
| 63 | SV2C | SV2C Entrez,  Source | synaptic vesicle glycoprotein 2C | 15383 | -0.100 | -0.0274 | No |
| 64 | ITGB7 | ITGB7 Entrez,  Source | integrin, beta 7 | 15809 | -0.108 | -0.0408 | No |
| 65 | ITGA11 | ITGA11 Entrez,  Source | integrin, alpha 11 | 15849 | -0.109 | -0.0343 | No |
| 66 | SDC2 | SDC2 Entrez,  Source | syndecan 2 (heparan sulfate proteoglycan 1, cell surface-associated, fibroglycan) | 15854 | -0.109 | -0.0260 | No |
| 67 | COL4A4 | COL4A4 Entrez,  Source | collagen, type IV, alpha 4 | 16048 | -0.112 | -0.0272 | No |
| 68 | LAMA2 | LAMA2 Entrez,  Source | laminin, alpha 2 (merosin, congenital muscular dystrophy) | 16160 | -0.114 | -0.0239 | No |
| 69 | GP6 | GP6 Entrez,  Source | glycoprotein VI (platelet) | 16872 | -0.129 | -0.0503 | No |
| 70 | CD36 | CD36 Entrez,  Source | CD36 molecule (thrombospondin receptor) | 17746 | -0.156 | -0.0829 | No |
| 71 | TNXB | TNXB Entrez,  Source | tenascin XB | 17771 | -0.157 | -0.0719 | No |
| 72 | IBSP | IBSP Entrez,  Source | integrin-binding sialoprotein (bone sialoprotein, bone sialoprotein II) | 17851 | -0.161 | -0.0634 | No |
| 73 | LAMC3 | LAMC3 Entrez,  Source | laminin, gamma 3 | 17882 | -0.162 | -0.0522 | No |
| 74 | ITGA9 | ITGA9 Entrez,  Source | integrin, alpha 9 | 18035 | -0.168 | -0.0469 | No |
| 75 | SV2A | SV2A Entrez,  Source | synaptic vesicle glycoprotein 2A | 18270 | -0.179 | -0.0449 | No |
| 76 | COL4A2 | COL4A2 Entrez,  Source | collagen, type IV, alpha 2 | 18730 | -0.211 | -0.0520 | No |
| 77 | TNN | TNN Entrez,  Source | tenascin N | 18831 | -0.221 | -0.0399 | No |
| 78 | COL5A1 | COL5A1 Entrez,  Source | collagen, type V, alpha 1 | 18981 | -0.240 | -0.0287 | No |
| 79 | THBS2 | THBS2 Entrez,  Source | thrombospondin 2 | 19096 | -0.259 | -0.0143 | No |
| 80 | COL2A1 | COL2A1 Entrez,  Source | collagen, type II, alpha 1 (primary osteoarthritis, spondyloepiphyseal dysplasia, congenital) | 19469 | -0.485 | 0.0045 | No |
Table: GSEA details [plain text format]

  

Fig 2: KEGG\_ECM\_RECEPTOR\_INTERACTION      
 Blue-Pink O' Gram in the Space of the Analyzed GeneSet

  

Fig 3: KEGG\_ECM\_RECEPTOR\_INTERACTION: Random ES distribution      
 Gene set null distribution of ES for **KEGG\_ECM\_RECEPTOR\_INTERACTION**

  
