# Supplementary material for: Comprehensive analysis of β-catenin target genes in colorectal carcinoma cell lines with deregulated Wnt/β-catenin signaling
Source: BMC Genomics. 2014 Jan 28;15:74. doi: 10.1186/1471-2164-15-74 (PMC3909937; doi:10.1186/1471-2164-15-74)
Supplement: Additional file 5 — GSEA analysis using the KEGG pathway database. This zipped file contains confirming data of the GSEA analysis. The names of the directories containing the files were composed of the term ‘GSEA’, the name of the cell line, e.g. DLD1, SW480, or LS174T, and the pathway database (KEGG). Please use a web browser to view the files with the name ‘index.html’ in the corresponding directories to start exploring the data. [file 1471-2164-15-74-S5.zip › GSEA KEGG SW480/KEGG_ENDOCYTOSIS.html]

Details for gene set KEGG\_ENDOCYTOSIS[GSEA]

|  || Dataset | SW480\_collapsed\_to\_symbols.class.cls#b\_versus\_bg.class.cls#b\_versus\_bg\_repos |
| Phenotype | class.cls#b\_versus\_bg\_repos |
| Upregulated in class | 1 |
| GeneSet | KEGG\_ENDOCYTOSIS |
| Enrichment Score (ES) | 0.388319 |
| Normalized Enrichment Score (NES) | 1.6839209 |
| Nominal p-value | 0.0 |
| FDR q-value | 0.06065565 |
| FWER p-Value | 0.426 |
Table: GSEA Results Summary

  

Fig 1: Enrichment plot: KEGG\_ENDOCYTOSIS      
 Profile of the Running ES Score & Positions of GeneSet Members on the Rank Ordered List

  

| PROBE | GENE SYMBOL | GENE\_TITLE | RANK IN GENE LIST | RANK METRIC SCORE | RUNNING ES | CORE ENRICHMENT || 1 | PSD3 | PSD3 Entrez,  Source | pleckstrin and Sec7 domain containing 3 | 37 | 0.707 | 0.0472 | Yes |
| 2 | ADRB2 | ADRB2 Entrez,  Source | adrenergic, beta-2-, receptor, surface | 42 | 0.694 | 0.0951 | Yes |
| 3 | ARF6 | ARF6 Entrez,  Source | ADP-ribosylation factor 6 | 208 | 0.395 | 0.1140 | Yes |
| 4 | ZFYVE20 | ZFYVE20 Entrez,  Source | zinc finger, FYVE domain containing 20 | 250 | 0.367 | 0.1374 | Yes |
| 5 | PARD6B | PARD6B Entrez,  Source | par-6 partitioning defective 6 homolog beta (C. elegans) | 420 | 0.297 | 0.1493 | Yes |
| 6 | SH3KBP1 | SH3KBP1 Entrez,  Source | SH3-domain kinase binding protein 1 | 464 | 0.286 | 0.1669 | Yes |
| 7 | DNM3 | DNM3 Entrez,  Source | dynamin 3 | 490 | 0.276 | 0.1848 | Yes |
| 8 | EGFR | EGFR Entrez,  Source | epidermal growth factor receptor (erythroblastic leukemia viral (v-erb-b) oncogene homolog, avian) | 652 | 0.241 | 0.1932 | Yes |
| 9 | GIT2 | GIT2 Entrez,  Source | G protein-coupled receptor kinase interactor 2 | 666 | 0.240 | 0.2091 | Yes |
| 10 | STAM2 | STAM2 Entrez,  Source | signal transducing adaptor molecule (SH3 domain and ITAM motif) 2 | 716 | 0.230 | 0.2226 | Yes |
| 11 | ARRB1 | ARRB1 Entrez,  Source | arrestin, beta 1 | 852 | 0.209 | 0.2301 | Yes |
| 12 | VPS4B | VPS4B Entrez,  Source | vacuolar protein sorting 4 homolog B (S. cerevisiae) | 855 | 0.208 | 0.2445 | Yes |
| 13 | HSPA2 | HSPA2 Entrez,  Source | heat shock 70kDa protein 2 | 932 | 0.200 | 0.2544 | Yes |
| 14 | VPS28 | VPS28 Entrez,  Source | vacuolar protein sorting 28 homolog (S. cerevisiae) | 1005 | 0.189 | 0.2638 | Yes |
| 15 | ARFGAP3 | ARFGAP3 Entrez,  Source | ADP-ribosylation factor GTPase activating protein 3 | 1051 | 0.185 | 0.2743 | Yes |
| 16 | NEDD4 | NEDD4 Entrez,  Source | neural precursor cell expressed, developmentally down-regulated 4 | 1057 | 0.184 | 0.2868 | Yes |
| 17 | HLA-A | HLA-A Entrez,  Source | major histocompatibility complex, class I, A | 1218 | 0.168 | 0.2903 | Yes |
| 18 | PSD4 | PSD4 Entrez,  Source | pleckstrin and Sec7 domain containing 4 | 1305 | 0.162 | 0.2971 | Yes |
| 19 | HSPA1A | HSPA1A Entrez,  Source | heat shock 70kDa protein 1A | 1322 | 0.160 | 0.3073 | Yes |
| 20 | HLA-F | HLA-F Entrez,  Source | major histocompatibility complex, class I, F | 1341 | 0.159 | 0.3175 | Yes |
| 21 | EPS15 | EPS15 Entrez,  Source | epidermal growth factor receptor pathway substrate 15 | 1348 | 0.159 | 0.3281 | Yes |
| 22 | IGF1R | IGF1R Entrez,  Source | insulin-like growth factor 1 receptor | 1352 | 0.158 | 0.3390 | Yes |
| 23 | VPS25 | VPS25 Entrez,  Source | vacuolar protein sorting 25 homolog (S. cerevisiae) | 1489 | 0.148 | 0.3423 | Yes |
| 24 | NEDD4L | NEDD4L Entrez,  Source | neural precursor cell expressed, developmentally down-regulated 4-like | 1500 | 0.147 | 0.3520 | Yes |
| 25 | PLD1 | PLD1 Entrez,  Source | phospholipase D1, phosphatidylcholine-specific | 1765 | 0.131 | 0.3475 | Yes |
| 26 | SH3GLB1 | SH3GLB1 Entrez,  Source | SH3-domain GRB2-like endophilin B1 | 1822 | 0.129 | 0.3535 | Yes |
| 27 | LDLR | LDLR Entrez,  Source | low density lipoprotein receptor (familial hypercholesterolemia) | 2193 | 0.112 | 0.3422 | Yes |
| 28 | IQSEC2 | IQSEC2 Entrez,  Source | IQ motif and Sec7 domain 2 | 2219 | 0.111 | 0.3486 | Yes |
| 29 | LDLRAP1 | LDLRAP1 Entrez,  Source | low density lipoprotein receptor adaptor protein 1 | 2241 | 0.110 | 0.3551 | Yes |
| 30 | CHMP4A | CHMP4A Entrez,  Source | chromatin modifying protein 4A | 2438 | 0.102 | 0.3521 | Yes |
| 31 | VPS37C | VPS37C Entrez,  Source | vacuolar protein sorting 37 homolog C (S. cerevisiae) | 2474 | 0.101 | 0.3573 | Yes |
| 32 | EEA1 | EEA1 Entrez,  Source | early endosome antigen 1, 162kD | 2639 | 0.094 | 0.3554 | Yes |
| 33 | RAB31 | RAB31 Entrez,  Source | RAB31, member RAS oncogene family | 2683 | 0.093 | 0.3596 | Yes |
| 34 | CHMP2A | CHMP2A Entrez,  Source | chromatin modifying protein 2A | 2782 | 0.089 | 0.3608 | Yes |
| 35 | HLA-G | HLA-G Entrez,  Source | HLA-G histocompatibility antigen, class I, G | 2852 | 0.087 | 0.3632 | Yes |
| 36 | SMURF2 | SMURF2 Entrez,  Source | SMAD specific E3 ubiquitin protein ligase 2 | 3018 | 0.082 | 0.3604 | Yes |
| 37 | CLTB | CLTB Entrez,  Source | clathrin, light chain (Lcb) | 3213 | 0.076 | 0.3557 | Yes |
| 38 | RABEP1 | RABEP1 Entrez,  Source | rabaptin, RAB GTPase binding effector protein 1 | 3250 | 0.075 | 0.3590 | Yes |
| 39 | CDC42 | CDC42 Entrez,  Source | cell division cycle 42 (GTP binding protein, 25kDa) | 3251 | 0.075 | 0.3642 | Yes |
| 40 | HLA-C | HLA-C Entrez,  Source | major histocompatibility complex, class I, C | 3264 | 0.074 | 0.3687 | Yes |
| 41 | HLA-B | HLA-B Entrez,  Source | major histocompatibility complex, class I, B | 3267 | 0.074 | 0.3738 | Yes |
| 42 | RAB5C | RAB5C Entrez,  Source | RAB5C, member RAS oncogene family | 3321 | 0.073 | 0.3761 | Yes |
| 43 | VPS36 | VPS36 Entrez,  Source | vacuolar protein sorting 36 (yeast) | 3357 | 0.072 | 0.3793 | Yes |
| 44 | EHD2 | EHD2 Entrez,  Source | EH-domain containing 2 | 3361 | 0.072 | 0.3841 | Yes |
| 45 | ITCH | ITCH Entrez,  Source | itchy homolog E3 ubiquitin protein ligase (mouse) | 3377 | 0.072 | 0.3883 | Yes |
| 46 | USP8 | USP8 Entrez,  Source | ubiquitin specific peptidase 8 | 3834 | 0.059 | 0.3689 | No |
| 47 | SMAP1 | SMAP1 Entrez,  Source | stromal membrane-associated protein 1 | 3852 | 0.059 | 0.3722 | No |
| 48 | STAMBP | STAMBP Entrez,  Source | STAM binding protein | 3939 | 0.057 | 0.3717 | No |
| 49 | FGFR2 | FGFR2 Entrez,  Source | ffer syndrome, Jackson-Weiss syndrome) | 4101 | 0.053 | 0.3671 | No |
| 50 | CLTA | CLTA Entrez,  Source | clathrin, light chain (Lca) | 4214 | 0.051 | 0.3648 | No |
| 51 | EHD1 | EHD1 Entrez,  Source | EH-domain containing 1 | 4581 | 0.043 | 0.3490 | No |
| 52 | RAB11A | RAB11A Entrez,  Source | RAB11A, member RAS oncogene family | 4751 | 0.040 | 0.3431 | No |
| 53 | GRK1 | GRK1 Entrez,  Source | G protein-coupled receptor kinase 1 | 4788 | 0.039 | 0.3439 | No |
| 54 | CHMP4B | CHMP4B Entrez,  Source | chromatin modifying protein 4B | 4840 | 0.038 | 0.3440 | No |
| 55 | AP2S1 | AP2S1 Entrez,  Source | adaptor-related protein complex 2, sigma 1 subunit | 4870 | 0.038 | 0.3451 | No |
| 56 | MDM2 | MDM2 Entrez,  Source | Mdm2, transformed 3T3 cell double minute 2, p53 binding protein (mouse) | 4876 | 0.038 | 0.3475 | No |
| 57 | CHMP6 | CHMP6 Entrez,  Source | chromatin modifying protein 6 | 4898 | 0.037 | 0.3490 | No |
| 58 | DNM1 | DNM1 Entrez,  Source | dynamin 1 | 4936 | 0.036 | 0.3496 | No |
| 59 | SH3GL1 | SH3GL1 Entrez,  Source | SH3-domain GRB2-like 1 | 4942 | 0.036 | 0.3518 | No |
| 60 | EHD4 | EHD4 Entrez,  Source | EH-domain containing 4 | 4965 | 0.036 | 0.3532 | No |
| 61 | HGS | HGS Entrez,  Source | hepatocyte growth factor-regulated tyrosine kinase substrate | 5048 | 0.035 | 0.3514 | No |
| 62 | SNF8 | SNF8 Entrez,  Source | SNF8, ESCRT-II complex subunit, homolog (S. cerevisiae) | 5049 | 0.035 | 0.3538 | No |
| 63 | TSG101 | TSG101 Entrez,  Source | tumor susceptibility gene 101 | 5071 | 0.034 | 0.3550 | No |
| 64 | GRK5 | GRK5 Entrez,  Source | G protein-coupled receptor kinase 5 | 5410 | 0.029 | 0.3396 | No |
| 65 | EPN2 | EPN2 Entrez,  Source | epsin 2 | 5561 | 0.026 | 0.3337 | No |
| 66 | PDCD6IP | PDCD6IP Entrez,  Source | programmed cell death 6 interacting protein | 5594 | 0.026 | 0.3338 | No |
| 67 | VPS4A | VPS4A Entrez,  Source | vacuolar protein sorting 4 homolog A (S. cerevisiae) | 5723 | 0.024 | 0.3288 | No |
| 68 | DNM2 | DNM2 Entrez,  Source | dynamin 2 | 5818 | 0.022 | 0.3256 | No |
| 69 | HSPA6 | HSPA6 Entrez,  Source | heat shock 70kDa protein 6 (HSP70B') | 5860 | 0.022 | 0.3249 | No |
| 70 | VPS37A | VPS37A Entrez,  Source | vacuolar protein sorting 37 homolog A (S. cerevisiae) | 5932 | 0.020 | 0.3227 | No |
| 71 | HSPA8 | HSPA8 Entrez,  Source | heat shock 70kDa protein 8 | 6368 | 0.014 | 0.3013 | No |
| 72 | MET | MET Entrez,  Source | met proto-oncogene (hepatocyte growth factor receptor) | 6408 | 0.014 | 0.3002 | No |
| 73 | CLTC | CLTC Entrez,  Source | clathrin, heavy chain (Hc) | 6436 | 0.013 | 0.2997 | No |
| 74 | IQSEC1 | IQSEC1 Entrez,  Source | IQ motif and Sec7 domain 1 | 6544 | 0.012 | 0.2950 | No |
| 75 | PRKCI | PRKCI Entrez,  Source | protein kinase C, iota | 6623 | 0.011 | 0.2918 | No |
| 76 | HLA-E | HLA-E Entrez,  Source | major histocompatibility complex, class I, E | 6630 | 0.011 | 0.2922 | No |
| 77 | AP2A1 | AP2A1 Entrez,  Source | adaptor-related protein complex 2, alpha 1 subunit | 6888 | 0.007 | 0.2795 | No |
| 78 | DNAJC6 | DNAJC6 Entrez,  Source | DnaJ (Hsp40) homolog, subfamily C, member 6 | 6979 | 0.006 | 0.2752 | No |
| 79 | PRKCZ | PRKCZ Entrez,  Source | protein kinase C, zeta | 7109 | 0.004 | 0.2689 | No |
| 80 | FAM125B | FAM125B Entrez,  Source | family with sequence similarity 125, member B | 7241 | 0.002 | 0.2623 | No |
| 81 | SMURF1 | SMURF1 Entrez,  Source | SMAD specific E3 ubiquitin protein ligase 1 | 7246 | 0.002 | 0.2623 | No |
| 82 | FGFR3 | FGFR3 Entrez,  Source | fibroblast growth factor receptor 3 (achondroplasia, thanatophoric dwarfism) | 7529 | -0.001 | 0.2478 | No |
| 83 | CHMP1B | CHMP1B Entrez,  Source | chromatin modifying protein 1B | 7533 | -0.001 | 0.2477 | No |
| 84 | HRAS | HRAS Entrez,  Source | v-Ha-ras Harvey rat sarcoma viral oncogene homolog | 7537 | -0.001 | 0.2476 | No |
| 85 | STAM | STAM Entrez,  Source | signal transducing adaptor molecule (SH3 domain and ITAM motif) 1 | 7649 | -0.002 | 0.2421 | No |
| 86 | NTRK1 | NTRK1 Entrez,  Source | neurotrophic tyrosine kinase, receptor, type 1 | 7954 | -0.006 | 0.2268 | No |
| 87 | CHMP2B | CHMP2B Entrez,  Source | chromatin modifying protein 2B | 8060 | -0.008 | 0.2220 | No |
| 88 | AP2A2 | AP2A2 Entrez,  Source | adaptor-related protein complex 2, alpha 2 subunit | 8072 | -0.008 | 0.2219 | No |
| 89 | IL2RB | IL2RB Entrez,  Source | interleukin 2 receptor, beta | 8166 | -0.009 | 0.2177 | No |
| 90 | SH3GL2 | SH3GL2 Entrez,  Source | SH3-domain GRB2-like 2 | 8265 | -0.010 | 0.2134 | No |
| 91 | DNM1L | DNM1L Entrez,  Source | dynamin 1-like | 8323 | -0.011 | 0.2112 | No |
| 92 | RAB11FIP5 | RAB11FIP5 Entrez,  Source | RAB11 family interacting protein 5 (class I) | 8383 | -0.011 | 0.2089 | No |
| 93 | FLT1 | FLT1 Entrez,  Source | fms-related tyrosine kinase 1 (vascular endothelial growth factor/vascular permeability factor receptor) | 8576 | -0.014 | 0.2000 | No |
| 94 | ERBB3 | ERBB3 Entrez,  Source | v-erb-b2 erythroblastic leukemia viral oncogene homolog 3 (avian) | 8640 | -0.014 | 0.1977 | No |
| 95 | AP2M1 | AP2M1 Entrez,  Source | adaptor-related protein complex 2, mu 1 subunit | 8845 | -0.017 | 0.1884 | No |
| 96 | TFRC | TFRC Entrez,  Source | transferrin receptor (p90, CD71) | 9093 | -0.020 | 0.1770 | No |
| 97 | PARD3 | PARD3 Entrez,  Source | par-3 partitioning defective 3 homolog (C. elegans) | 9168 | -0.021 | 0.1746 | No |
| 98 | VPS37B | VPS37B Entrez,  Source | vacuolar protein sorting 37 homolog B (S. cerevisiae) | 9242 | -0.021 | 0.1724 | No |
| 99 | AP2B1 | AP2B1 Entrez,  Source | adaptor-related protein complex 2, beta 1 subunit | 9462 | -0.024 | 0.1627 | No |
| 100 | CBL | CBL Entrez,  Source | Cas-Br-M (murine) ecotropic retroviral transforming sequence | 9608 | -0.026 | 0.1570 | No |
| 101 | PIP5K1C | PIP5K1C Entrez,  Source | phosphatidylinositol-4-phosphate 5-kinase, type I, gamma | 9760 | -0.027 | 0.1511 | No |
| 102 | RAB5A | RAB5A Entrez,  Source | RAB5A, member RAS oncogene family | 9879 | -0.029 | 0.1470 | No |
| 103 | CHMP5 | CHMP5 Entrez,  Source | chromatin modifying protein 5 | 9906 | -0.029 | 0.1477 | No |
| 104 | RUFY1 | RUFY1 Entrez,  Source | RUN and FYVE domain containing 1 | 9925 | -0.029 | 0.1488 | No |
| 105 | CSF1R | CSF1R Entrez,  Source | colony stimulating factor 1 receptor, formerly McDonough feline sarcoma viral (v-fms) oncogene homolog | 10135 | -0.032 | 0.1402 | No |
| 106 | RAB5B | RAB5B Entrez,  Source | RAB5B, member RAS oncogene family | 10316 | -0.034 | 0.1333 | No |
| 107 | TRAF6 | TRAF6 Entrez,  Source | TNF receptor-associated factor 6 | 10318 | -0.034 | 0.1355 | No |
| 108 | WWP1 | WWP1 Entrez,  Source | WW domain containing E3 ubiquitin protein ligase 1 | 10386 | -0.034 | 0.1345 | No |
| 109 | RET | RET Entrez,  Source | ret proto-oncogene (multiple endocrine neoplasia and medullary thyroid carcinoma 1, Hirschsprung disease) | 10485 | -0.036 | 0.1319 | No |
| 110 | CBLB | CBLB Entrez,  Source | Cas-Br-M (murine) ecotropic retroviral transforming sequence b | 10489 | -0.036 | 0.1342 | No |
| 111 | RAB22A | RAB22A Entrez,  Source | RAB22A, member RAS oncogene family | 10520 | -0.036 | 0.1352 | No |
| 112 | HSPA1L | HSPA1L Entrez,  Source | heat shock 70kDa protein 1-like | 10566 | -0.037 | 0.1354 | No |
| 113 | RAB11B | RAB11B Entrez,  Source | RAB11B, member RAS oncogene family | 10597 | -0.037 | 0.1364 | No |
| 114 | EHD3 | EHD3 Entrez,  Source | EH-domain containing 3 | 10806 | -0.040 | 0.1284 | No |
| 115 | GRK4 | GRK4 Entrez,  Source | G protein-coupled receptor kinase 4 | 10849 | -0.040 | 0.1291 | No |
| 116 | ARFGAP1 | ARFGAP1 Entrez,  Source | ADP-ribosylation factor GTPase activating protein 1 | 11017 | -0.042 | 0.1234 | No |
| 117 | CLTCL1 | CLTCL1 Entrez,  Source | clathrin, heavy chain-like 1 | 11208 | -0.045 | 0.1167 | No |
| 118 | KIT | KIT Entrez,  Source | v-kit Hardy-Zuckerman 4 feline sarcoma viral oncogene homolog | 11285 | -0.046 | 0.1159 | No |
| 119 | CHMP4C | CHMP4C Entrez,  Source | chromatin modifying protein 4C | 11598 | -0.049 | 0.1033 | No |
| 120 | FGFR4 | FGFR4 Entrez,  Source | fibroblast growth factor receptor 4 | 11796 | -0.052 | 0.0967 | No |
| 121 | SH3GLB2 | SH3GLB2 Entrez,  Source | SH3-domain GRB2-like endophilin B2 | 12264 | -0.057 | 0.0766 | No |
| 122 | RAB4A | RAB4A Entrez,  Source | RAB4A, member RAS oncogene family | 12345 | -0.058 | 0.0765 | No |
| 123 | ADRBK2 | ADRBK2 Entrez,  Source | adrenergic, beta, receptor kinase 2 | 12619 | -0.062 | 0.0668 | No |
| 124 | KDR | KDR Entrez,  Source | kinase insert domain receptor (a type III receptor tyrosine kinase) | 12913 | -0.065 | 0.0562 | No |
| 125 | RAB11FIP4 | RAB11FIP4 Entrez,  Source | RAB11 family interacting protein 4 (class II) | 13188 | -0.069 | 0.0469 | No |
| 126 | CBLC | CBLC Entrez,  Source | Cas-Br-M (murine) ecotropic retroviral transforming sequence c | 13387 | -0.072 | 0.0416 | No |
| 127 | DAB2 | DAB2 Entrez,  Source | disabled homolog 2, mitogen-responsive phosphoprotein (Drosophila) | 13481 | -0.073 | 0.0419 | No |
| 128 | PARD6A | PARD6A Entrez,  Source | par-6 partitioning defective 6 homolog alpha (C.elegans) | 13589 | -0.074 | 0.0415 | No |
| 129 | SH3GL3 | SH3GL3 Entrez,  Source | SH3-domain GRB2-like 3 | 13842 | -0.078 | 0.0339 | No |
| 130 | EPN1 | EPN1 Entrez,  Source | epsin 1 | 14093 | -0.081 | 0.0266 | No |
| 131 | PLD2 | PLD2 Entrez,  Source | phospholipase D2 | 14235 | -0.083 | 0.0251 | No |
| 132 | RAB11FIP3 | RAB11FIP3 Entrez,  Source | RAB11 family interacting protein 3 (class II) | 14348 | -0.084 | 0.0252 | No |
| 133 | PARD6G | PARD6G Entrez,  Source | par-6 partitioning defective 6 homolog gamma (C. elegans) | 14681 | -0.089 | 0.0142 | No |
| 134 | VPS37D | VPS37D Entrez,  Source | vacuolar protein sorting 37 homolog D (S. cerevisiae) | 14687 | -0.089 | 0.0202 | No |
| 135 | ADRBK1 | ADRBK1 Entrez,  Source | adrenergic, beta, receptor kinase 1 | 14710 | -0.089 | 0.0252 | No |
| 136 | F2R | F2R Entrez,  Source | coagulation factor II (thrombin) receptor | 14863 | -0.091 | 0.0237 | No |
| 137 | PDGFRA | PDGFRA Entrez,  Source | platelet-derived growth factor receptor, alpha polypeptide | 14940 | -0.093 | 0.0263 | No |
| 138 | GRK6 | GRK6 Entrez,  Source | G protein-coupled receptor kinase 6 | 14965 | -0.093 | 0.0315 | No |
| 139 | EPN3 | EPN3 Entrez,  Source | epsin 3 | 14981 | -0.093 | 0.0372 | No |
| 140 | GIT1 | GIT1 Entrez,  Source | G protein-coupled receptor kinase interactor 1 | 15156 | -0.097 | 0.0349 | No |
| 141 | SRC | SRC Entrez,  Source | v-src sarcoma (Schmidt-Ruppin A-2) viral oncogene homolog (avian) | 15539 | -0.103 | 0.0224 | No |
| 142 | PSD | PSD Entrez,  Source | pleckstrin and Sec7 domain containing | 15672 | -0.105 | 0.0228 | No |
| 143 | RNF41 | RNF41 Entrez,  Source | ring finger protein 41 | 15913 | -0.110 | 0.0181 | No |
| 144 | PSD2 | PSD2 Entrez,  Source | pleckstrin and Sec7 domain containing 2 | 16387 | -0.119 | 0.0019 | No |
| 145 | ERBB4 | ERBB4 Entrez,  Source | v-erb-a erythroblastic leukemia viral oncogene homolog 4 (avian) | 16491 | -0.121 | 0.0050 | No |
| 146 | ADRB3 | ADRB3 Entrez,  Source | adrenergic, beta-3-, receptor | 16541 | -0.122 | 0.0109 | No |
| 147 | ADRB1 | ADRB1 Entrez,  Source | adrenergic, beta-1-, receptor | 16547 | -0.122 | 0.0191 | No |
| 148 | ARRB2 | ARRB2 Entrez,  Source | arrestin, beta 2 | 17265 | -0.141 | -0.0081 | No |
| 149 | IL2RA | IL2RA Entrez,  Source | interleukin 2 receptor, alpha | 17457 | -0.147 | -0.0077 | No |
| 150 | RAB11FIP1 | RAB11FIP1 Entrez,  Source | RAB11 family interacting protein 1 (class I) | 17534 | -0.149 | -0.0013 | No |
| 151 | EGF | EGF Entrez,  Source | epidermal growth factor (beta-urogastrone) | 17577 | -0.151 | 0.0070 | No |
| 152 | PIP5K1A | PIP5K1A Entrez,  Source | phosphatidylinositol-4-phosphate 5-kinase, type I, alpha | 18375 | -0.186 | -0.0212 | No |
| 153 | RAB11FIP2 | RAB11FIP2 Entrez,  Source | RAB11 family interacting protein 2 (class I) | 18677 | -0.206 | -0.0224 | No |
| 154 | IL2RG | IL2RG Entrez,  Source | interleukin 2 receptor, gamma (severe combined immunodeficiency) | 19052 | -0.252 | -0.0242 | No |
| 155 | PIP5K1B | PIP5K1B Entrez,  Source | phosphatidylinositol-4-phosphate 5-kinase, type I, beta | 19224 | -0.294 | -0.0126 | No |
| 156 | CXCR4 | CXCR4 Entrez,  Source | chemokine (C-X-C motif) receptor 4 | 19423 | -0.428 | 0.0069 | No |
Table: GSEA details [plain text format]

  

Fig 2: KEGG\_ENDOCYTOSIS      
 Blue-Pink O' Gram in the Space of the Analyzed GeneSet

  

Fig 3: KEGG\_ENDOCYTOSIS: Random ES distribution      
 Gene set null distribution of ES for **KEGG\_ENDOCYTOSIS**

  
