# Supplementary material for: Comprehensive analysis of β-catenin target genes in colorectal carcinoma cell lines with deregulated Wnt/β-catenin signaling
Source: BMC Genomics. 2014 Jan 28;15:74. doi: 10.1186/1471-2164-15-74 (PMC3909937; doi:10.1186/1471-2164-15-74)
Supplement: Additional file 5 — GSEA analysis using the KEGG pathway database. This zipped file contains confirming data of the GSEA analysis. The names of the directories containing the files were composed of the term ‘GSEA’, the name of the cell line, e.g. DLD1, SW480, or LS174T, and the pathway database (KEGG). Please use a web browser to view the files with the name ‘index.html’ in the corresponding directories to start exploring the data. [file 1471-2164-15-74-S5.zip › GSEA KEGG SW480/KEGG_ETHER_LIPID_METABOLISM.html]

Details for gene set KEGG\_ETHER\_LIPID\_METABOLISM[GSEA]

|  || Dataset | SW480\_collapsed\_to\_symbols.class.cls#b\_versus\_bg.class.cls#b\_versus\_bg\_repos |
| Phenotype | class.cls#b\_versus\_bg\_repos |
| Upregulated in class | 1 |
| GeneSet | KEGG\_ETHER\_LIPID\_METABOLISM |
| Enrichment Score (ES) | 0.5141038 |
| Normalized Enrichment Score (NES) | 1.6190417 |
| Nominal p-value | 0.024271844 |
| FDR q-value | 0.08266333 |
| FWER p-Value | 0.61 |
Table: GSEA Results Summary

  

Fig 1: Enrichment plot: KEGG\_ETHER\_LIPID\_METABOLISM      
 Profile of the Running ES Score & Positions of GeneSet Members on the Rank Ordered List

  

| PROBE | GENE SYMBOL | GENE\_TITLE | RANK IN GENE LIST | RANK METRIC SCORE | RUNNING ES | CORE ENRICHMENT || 1 | PLA2G7 | PLA2G7 Entrez,  Source | phospholipase A2, group VII (platelet-activating factor acetylhydrolase, plasma) | 50 | 0.646 | 0.2269 | Yes |
| 2 | PLA2G4A | PLA2G4A Entrez,  Source | phospholipase A2, group IVA (cytosolic, calcium-dependent) | 217 | 0.389 | 0.3565 | Yes |
| 3 | PPAP2B | PPAP2B Entrez,  Source | phosphatidic acid phosphatase type 2B | 665 | 0.240 | 0.4188 | Yes |
| 4 | PPAP2C | PPAP2C Entrez,  Source | phosphatidic acid phosphatase type 2C | 1353 | 0.158 | 0.4399 | Yes |
| 5 | PLA2G4B | PLA2G4B Entrez,  Source | phospholipase A2, group IVB (cytosolic) | 1651 | 0.137 | 0.4733 | Yes |
| 6 | PLD1 | PLD1 Entrez,  Source | phospholipase D1, phosphatidylcholine-specific | 1765 | 0.131 | 0.5141 | Yes |
| 7 | PAFAH1B1 | PAFAH1B1 Entrez,  Source | platelet-activating factor acetylhydrolase, isoform Ib, alpha subunit 45kDa | 3161 | 0.077 | 0.4702 | No |
| 8 | AGPS | AGPS Entrez,  Source | alkylglycerone phosphate synthase | 4548 | 0.044 | 0.4149 | No |
| 9 | PLA2G2F | PLA2G2F Entrez,  Source | phospholipase A2, group IIF | 4675 | 0.041 | 0.4231 | No |
| 10 | ENPP2 | ENPP2 Entrez,  Source | ectonucleotide pyrophosphatase/phosphodiesterase 2 (autotaxin) | 4747 | 0.040 | 0.4338 | No |
| 11 | PLA2G2A | PLA2G2A Entrez,  Source | phospholipase A2, group IIA (platelets, synovial fluid) | 5011 | 0.035 | 0.4328 | No |
| 12 | ENPP6 | ENPP6 Entrez,  Source | ectonucleotide pyrophosphatase/phosphodiesterase 6 | 5678 | 0.024 | 0.4073 | No |
| 13 | PPAP2A | PPAP2A Entrez,  Source | phosphatidic acid phosphatase type 2A | 6185 | 0.017 | 0.3874 | No |
| 14 | PAFAH1B3 | PAFAH1B3 Entrez,  Source | platelet-activating factor acetylhydrolase, isoform Ib, gamma subunit 29kDa | 7069 | 0.005 | 0.3438 | No |
| 15 | PLA2G1B | PLA2G1B Entrez,  Source | phospholipase A2, group IB (pancreas) | 8420 | -0.012 | 0.2789 | No |
| 16 | CHPT1 | CHPT1 Entrez,  Source | choline phosphotransferase 1 | 8800 | -0.016 | 0.2652 | No |
| 17 | PAFAH2 | PAFAH2 Entrez,  Source | platelet-activating factor acetylhydrolase 2, 40kDa | 10445 | -0.035 | 0.1935 | No |
| 18 | PLA2G3 | PLA2G3 Entrez,  Source | phospholipase A2, group III | 11141 | -0.044 | 0.1735 | No |
| 19 | PLA2G12A | PLA2G12A Entrez,  Source | phospholipase A2, group XIIA | 11876 | -0.053 | 0.1546 | No |
| 20 | PAFAH1B2 | PAFAH1B2 Entrez,  Source | platelet-activating factor acetylhydrolase, isoform Ib, beta subunit 30kDa | 12710 | -0.063 | 0.1344 | No |
| 21 | PLA2G10 | PLA2G10 Entrez,  Source | phospholipase A2, group X | 13525 | -0.073 | 0.1188 | No |
| 22 | PLA2G5 | PLA2G5 Entrez,  Source | phospholipase A2, group V | 13571 | -0.074 | 0.1427 | No |
| 23 | PLA2G12B | PLA2G12B Entrez,  Source | phospholipase A2, group XIIB | 13707 | -0.076 | 0.1628 | No |
| 24 | PLD2 | PLD2 Entrez,  Source | phospholipase D2 | 14235 | -0.083 | 0.1652 | No |
| 25 | PLA2G6 | PLA2G6 Entrez,  Source | phospholipase A2, group VI (cytosolic, calcium-independent) | 14506 | -0.086 | 0.1820 | No |
| 26 | PLA2G2D | PLA2G2D Entrez,  Source | phospholipase A2, group IID | 15035 | -0.094 | 0.1884 | No |
| 27 | PLA2G2E | PLA2G2E Entrez,  Source | phospholipase A2, group IIE | 16515 | -0.121 | 0.1557 | No |
Table: GSEA details [plain text format]

  

Fig 2: KEGG\_ETHER\_LIPID\_METABOLISM      
 Blue-Pink O' Gram in the Space of the Analyzed GeneSet

  

Fig 3: KEGG\_ETHER\_LIPID\_METABOLISM: Random ES distribution      
 Gene set null distribution of ES for **KEGG\_ETHER\_LIPID\_METABOLISM**

  
