# Supplementary material for: Comprehensive analysis of β-catenin target genes in colorectal carcinoma cell lines with deregulated Wnt/β-catenin signaling
Source: BMC Genomics. 2014 Jan 28;15:74. doi: 10.1186/1471-2164-15-74 (PMC3909937; doi:10.1186/1471-2164-15-74)
Supplement: Additional file 5 — GSEA analysis using the KEGG pathway database. This zipped file contains confirming data of the GSEA analysis. The names of the directories containing the files were composed of the term ‘GSEA’, the name of the cell line, e.g. DLD1, SW480, or LS174T, and the pathway database (KEGG). Please use a web browser to view the files with the name ‘index.html’ in the corresponding directories to start exploring the data. [file 1471-2164-15-74-S5.zip › GSEA KEGG SW480/KEGG_FOCAL_ADHESION.html]

Details for gene set KEGG\_FOCAL\_ADHESION[GSEA]

|  || Dataset | SW480\_collapsed\_to\_symbols.class.cls#b\_versus\_bg.class.cls#b\_versus\_bg\_repos |
| Phenotype | class.cls#b\_versus\_bg\_repos |
| Upregulated in class | 1 |
| GeneSet | KEGG\_FOCAL\_ADHESION |
| Enrichment Score (ES) | 0.39886826 |
| Normalized Enrichment Score (NES) | 1.7608098 |
| Nominal p-value | 0.0 |
| FDR q-value | 0.047916736 |
| FWER p-Value | 0.238 |
Table: GSEA Results Summary

  

Fig 1: Enrichment plot: KEGG\_FOCAL\_ADHESION      
 Profile of the Running ES Score & Positions of GeneSet Members on the Rank Ordered List

  

| PROBE | GENE SYMBOL | GENE\_TITLE | RANK IN GENE LIST | RANK METRIC SCORE | RUNNING ES | CORE ENRICHMENT || 1 | ITGB8 | ITGB8 Entrez,  Source | integrin, beta 8 | 1 | 1.743 | 0.0744 | Yes |
| 2 | TNC | TNC Entrez,  Source | tenascin C (hexabrachion) | 5 | 0.981 | 0.1162 | Yes |
| 3 | PDGFC | PDGFC Entrez,  Source | platelet derived growth factor C | 25 | 0.776 | 0.1483 | Yes |
| 4 | THBS1 | THBS1 Entrez,  Source | thrombospondin 1 | 52 | 0.639 | 0.1743 | Yes |
| 5 | CAV2 | CAV2 Entrez,  Source | caveolin 2 | 75 | 0.585 | 0.1981 | Yes |
| 6 | RAC2 | RAC2 Entrez,  Source | ras-related C3 botulinum toxin substrate 2 (rho family, small GTP binding protein Rac2) | 119 | 0.477 | 0.2163 | Yes |
| 7 | COL6A1 | COL6A1 Entrez,  Source | collagen, type VI, alpha 1 | 126 | 0.471 | 0.2361 | Yes |
| 8 | ITGA3 | ITGA3 Entrez,  Source | integrin, alpha 3 (antigen CD49C, alpha 3 subunit of VLA-3 receptor) | 134 | 0.458 | 0.2553 | Yes |
| 9 | FN1 | FN1 Entrez,  Source | fibronectin 1 | 145 | 0.442 | 0.2737 | Yes |
| 10 | ITGB4 | ITGB4 Entrez,  Source | integrin, beta 4 | 201 | 0.401 | 0.2880 | Yes |
| 11 | CAV1 | CAV1 Entrez,  Source | caveolin 1, caveolae protein, 22kDa | 216 | 0.390 | 0.3039 | Yes |
| 12 | COL6A3 | COL6A3 Entrez,  Source | collagen, type VI, alpha 3 | 347 | 0.322 | 0.3109 | Yes |
| 13 | BIRC3 | BIRC3 Entrez,  Source | baculoviral IAP repeat-containing 3 | 455 | 0.289 | 0.3177 | Yes |
| 14 | LAMA3 | LAMA3 Entrez,  Source | laminin, alpha 3 | 514 | 0.269 | 0.3262 | Yes |
| 15 | BIRC2 | BIRC2 Entrez,  Source | baculoviral IAP repeat-containing 2 | 572 | 0.255 | 0.3342 | Yes |
| 16 | PXN | PXN Entrez,  Source | paxillin | 632 | 0.245 | 0.3416 | Yes |
| 17 | EGFR | EGFR Entrez,  Source | epidermal growth factor receptor (erythroblastic leukemia viral (v-erb-b) oncogene homolog, avian) | 652 | 0.241 | 0.3509 | Yes |
| 18 | VCL | VCL Entrez,  Source | vinculin | 772 | 0.222 | 0.3542 | Yes |
| 19 | AKT3 | AKT3 Entrez,  Source | v-akt murine thymoma viral oncogene homolog 3 (protein kinase B, gamma) | 870 | 0.207 | 0.3581 | Yes |
| 20 | SOS2 | SOS2 Entrez,  Source | son of sevenless homolog 2 (Drosophila) | 905 | 0.202 | 0.3650 | Yes |
| 21 | ITGA6 | ITGA6 Entrez,  Source | integrin, alpha 6 | 1088 | 0.180 | 0.3633 | Yes |
| 22 | SHC1 | SHC1 Entrez,  Source | SHC (Src homology 2 domain containing) transforming protein 1 | 1163 | 0.173 | 0.3668 | Yes |
| 23 | MYLK | MYLK Entrez,  Source | myosin, light chain kinase | 1183 | 0.171 | 0.3731 | Yes |
| 24 | BAD | BAD Entrez,  Source | BCL2-antagonist of cell death | 1250 | 0.166 | 0.3768 | Yes |
| 25 | ITGB5 | ITGB5 Entrez,  Source | integrin, beta 5 | 1286 | 0.163 | 0.3820 | Yes |
| 26 | IGF1R | IGF1R Entrez,  Source | insulin-like growth factor 1 receptor | 1352 | 0.158 | 0.3854 | Yes |
| 27 | LAMB1 | LAMB1 Entrez,  Source | laminin, beta 1 | 1397 | 0.155 | 0.3898 | Yes |
| 28 | CAPN2 | CAPN2 Entrez,  Source | calpain 2, (m/II) large subunit | 1413 | 0.154 | 0.3956 | Yes |
| 29 | COL4A1 | COL4A1 Entrez,  Source | collagen, type IV, alpha 1 | 1473 | 0.149 | 0.3989 | Yes |
| 30 | JUN | JUN Entrez,  Source | jun oncogene | 1725 | 0.133 | 0.3916 | No |
| 31 | ITGA2 | ITGA2 Entrez,  Source | integrin, alpha 2 (CD49B, alpha 2 subunit of VLA-2 receptor) | 1768 | 0.131 | 0.3950 | No |
| 32 | PRKCA | PRKCA Entrez,  Source | protein kinase C, alpha | 1907 | 0.125 | 0.3932 | No |
| 33 | PARVA | PARVA Entrez,  Source | parvin, alpha | 2022 | 0.120 | 0.3924 | No |
| 34 | ITGAV | ITGAV Entrez,  Source | integrin, alpha V (vitronectin receptor, alpha polypeptide, antigen CD51) | 2073 | 0.117 | 0.3949 | No |
| 35 | ITGB1 | ITGB1 Entrez,  Source | integrin, beta 1 (fibronectin receptor, beta polypeptide, antigen CD29 includes MDF2, MSK12) | 2321 | 0.107 | 0.3867 | No |
| 36 | BRAF | BRAF Entrez,  Source | v-raf murine sarcoma viral oncogene homolog B1 | 2414 | 0.103 | 0.3863 | No |
| 37 | ROCK2 | ROCK2 Entrez,  Source | Rho-associated, coiled-coil containing protein kinase 2 | 2416 | 0.103 | 0.3907 | No |
| 38 | ITGA10 | ITGA10 Entrez,  Source | integrin, alpha 10 | 2418 | 0.103 | 0.3950 | No |
| 39 | LAMA5 | LAMA5 Entrez,  Source | laminin, alpha 5 | 2487 | 0.100 | 0.3958 | No |
| 40 | COMP | COMP Entrez,  Source | cartilage oligomeric matrix protein | 2825 | 0.088 | 0.3821 | No |
| 41 | PDGFB | PDGFB Entrez,  Source | platelet-derived growth factor beta polypeptide (simian sarcoma viral (v-sis) oncogene homolog) | 2833 | 0.087 | 0.3855 | No |
| 42 | CCND3 | CCND3 Entrez,  Source | cyclin D3 | 2857 | 0.087 | 0.3881 | No |
| 43 | ACTN1 | ACTN1 Entrez,  Source | actinin, alpha 1 | 2936 | 0.085 | 0.3876 | No |
| 44 | ITGB6 | ITGB6 Entrez,  Source | integrin, beta 6 | 2948 | 0.084 | 0.3907 | No |
| 45 | PPP1R12A | PPP1R12A Entrez,  Source | protein phosphatase 1, regulatory (inhibitor) subunit 12A | 3049 | 0.081 | 0.3890 | No |
| 46 | SPP1 | SPP1 Entrez,  Source | secreted phosphoprotein 1 (osteopontin, bone sialoprotein I, early T-lymphocyte activation 1) | 3129 | 0.078 | 0.3882 | No |
| 47 | PIK3CB | PIK3CB Entrez,  Source | phosphoinositide-3-kinase, catalytic, beta polypeptide | 3241 | 0.075 | 0.3857 | No |
| 48 | CDC42 | CDC42 Entrez,  Source | cell division cycle 42 (GTP binding protein, 25kDa) | 3251 | 0.075 | 0.3884 | No |
| 49 | PDGFA | PDGFA Entrez,  Source | platelet-derived growth factor alpha polypeptide | 3262 | 0.074 | 0.3911 | No |
| 50 | PPP1CB | PPP1CB Entrez,  Source | protein phosphatase 1, catalytic subunit, beta isoform | 3286 | 0.074 | 0.3930 | No |
| 51 | RHOA | RHOA Entrez,  Source | ras homolog gene family, member A | 3339 | 0.072 | 0.3935 | No |
| 52 | FLNA | FLNA Entrez,  Source | filamin A, alpha (actin binding protein 280) | 3398 | 0.071 | 0.3935 | No |
| 53 | RAP1A | RAP1A Entrez,  Source | RAP1A, member of RAS oncogene family | 3447 | 0.070 | 0.3940 | No |
| 54 | AKT2 | AKT2 Entrez,  Source | v-akt murine thymoma viral oncogene homolog 2 | 3525 | 0.067 | 0.3929 | No |
| 55 | FLNB | FLNB Entrez,  Source | filamin B, beta (actin binding protein 278) | 3555 | 0.066 | 0.3942 | No |
| 56 | SHC4 | SHC4 Entrez,  Source | SHC (Src homology 2 domain containing) family, member 4 | 3570 | 0.066 | 0.3963 | No |
| 57 | CRKL | CRKL Entrez,  Source | v-crk sarcoma virus CT10 oncogene homolog (avian)-like | 3875 | 0.058 | 0.3831 | No |
| 58 | MAPK8 | MAPK8 Entrez,  Source | mitogen-activated protein kinase 8 | 4307 | 0.049 | 0.3629 | No |
| 59 | PTK2 | PTK2 Entrez,  Source | PTK2 protein tyrosine kinase 2 | 4313 | 0.049 | 0.3648 | No |
| 60 | PPP1CC | PPP1CC Entrez,  Source | protein phosphatase 1, catalytic subunit, gamma isoform | 4328 | 0.049 | 0.3661 | No |
| 61 | RAPGEF1 | RAPGEF1 Entrez,  Source | Rap guanine nucleotide exchange factor (GEF) 1 | 4341 | 0.048 | 0.3676 | No |
| 62 | VAV2 | VAV2 Entrez,  Source | vav 2 oncogene | 4538 | 0.044 | 0.3593 | No |
| 63 | BCAR1 | BCAR1 Entrez,  Source | breast cancer anti-estrogen resistance 1 | 5032 | 0.035 | 0.3354 | No |
| 64 | PTEN | PTEN Entrez,  Source | phosphatase and tensin homolog (mutated in multiple advanced cancers 1) | 5203 | 0.032 | 0.3280 | No |
| 65 | ARHGAP5 | ARHGAP5 Entrez,  Source | Rho GTPase activating protein 5 | 5422 | 0.028 | 0.3179 | No |
| 66 | LAMB2 | LAMB2 Entrez,  Source | laminin, beta 2 (laminin S) | 5499 | 0.027 | 0.3152 | No |
| 67 | GRB2 | GRB2 Entrez,  Source | growth factor receptor-bound protein 2 | 5557 | 0.026 | 0.3133 | No |
| 68 | PRKCG | PRKCG Entrez,  Source | protein kinase C, gamma | 5651 | 0.025 | 0.3096 | No |
| 69 | PPP1CA | PPP1CA Entrez,  Source | protein phosphatase 1, catalytic subunit, alpha isoform | 5908 | 0.021 | 0.2972 | No |
| 70 | ILK | ILK Entrez,  Source | integrin-linked kinase | 6056 | 0.019 | 0.2905 | No |
| 71 | ACTB | ACTB Entrez,  Source | actin, beta | 6245 | 0.016 | 0.2814 | No |
| 72 | AKT1 | AKT1 Entrez,  Source | v-akt murine thymoma viral oncogene homolog 1 | 6295 | 0.015 | 0.2796 | No |
| 73 | MAP2K1 | MAP2K1 Entrez,  Source | mitogen-activated protein kinase kinase 1 | 6310 | 0.015 | 0.2795 | No |
| 74 | CRK | CRK Entrez,  Source | v-crk sarcoma virus CT10 oncogene homolog (avian) | 6353 | 0.014 | 0.2779 | No |
| 75 | PIK3R1 | PIK3R1 Entrez,  Source | phosphoinositide-3-kinase, regulatory subunit 1 (p85 alpha) | 6402 | 0.014 | 0.2760 | No |
| 76 | MET | MET Entrez,  Source | met proto-oncogene (hepatocyte growth factor receptor) | 6408 | 0.014 | 0.2763 | No |
| 77 | ITGA7 | ITGA7 Entrez,  Source | integrin, alpha 7 | 6518 | 0.012 | 0.2712 | No |
| 78 | ACTG1 | ACTG1 Entrez,  Source | actin, gamma 1 | 6584 | 0.011 | 0.2684 | No |
| 79 | COL5A2 | COL5A2 Entrez,  Source | collagen, type V, alpha 2 | 6595 | 0.011 | 0.2683 | No |
| 80 | PAK4 | PAK4 Entrez,  Source | p21(CDKN1A)-activated kinase 4 | 6628 | 0.011 | 0.2671 | No |
| 81 | VTN | VTN Entrez,  Source | vitronectin | 6767 | 0.009 | 0.2604 | No |
| 82 | VEGFB | VEGFB Entrez,  Source | vascular endothelial growth factor B | 6773 | 0.009 | 0.2605 | No |
| 83 | GSK3B | GSK3B Entrez,  Source | glycogen synthase kinase 3 beta | 6824 | 0.008 | 0.2583 | No |
| 84 | SHC3 | SHC3 Entrez,  Source | SHC (Src homology 2 domain containing) transforming protein 3 | 6862 | 0.008 | 0.2567 | No |
| 85 | DOCK1 | DOCK1 Entrez,  Source | dedicator of cytokinesis 1 | 7132 | 0.004 | 0.2430 | No |
| 86 | MYL2 | MYL2 Entrez,  Source | myosin, light chain 2, regulatory, cardiac, slow | 7222 | 0.003 | 0.2385 | No |
| 87 | CCND1 | CCND1 Entrez,  Source | cyclin D1 | 7482 | -0.000 | 0.2251 | No |
| 88 | TLN2 | TLN2 Entrez,  Source | talin 2 | 7536 | -0.001 | 0.2225 | No |
| 89 | HRAS | HRAS Entrez,  Source | v-Ha-ras Harvey rat sarcoma viral oncogene homolog | 7537 | -0.001 | 0.2225 | No |
| 90 | PGF | PGF Entrez,  Source | placental growth factor, vascular endothelial growth factor-related protein | 7746 | -0.004 | 0.2119 | No |
| 91 | HGF | HGF Entrez,  Source | hepatocyte growth factor (hepapoietin A; scatter factor) | 7776 | -0.004 | 0.2106 | No |
| 92 | TLN1 | TLN1 Entrez,  Source | talin 1 | 8187 | -0.009 | 0.1898 | No |
| 93 | BCL2 | BCL2 Entrez,  Source | B-cell CLL/lymphoma 2 | 8283 | -0.010 | 0.1854 | No |
| 94 | VASP | VASP Entrez,  Source | vasodilator-stimulated phosphoprotein | 8345 | -0.011 | 0.1827 | No |
| 95 | COL1A1 | COL1A1 Entrez,  Source | collagen, type I, alpha 1 | 8461 | -0.012 | 0.1773 | No |
| 96 | PARVB | PARVB Entrez,  Source | parvin, beta | 8485 | -0.013 | 0.1766 | No |
| 97 | FLT1 | FLT1 Entrez,  Source | fms-related tyrosine kinase 1 (vascular endothelial growth factor/vascular permeability factor receptor) | 8576 | -0.014 | 0.1725 | No |
| 98 | MAPK9 | MAPK9 Entrez,  Source | mitogen-activated protein kinase 9 | 8669 | -0.015 | 0.1684 | No |
| 99 | RAC3 | RAC3 Entrez,  Source | ras-related C3 botulinum toxin substrate 3 (rho family, small GTP binding protein Rac3) | 8838 | -0.017 | 0.1605 | No |
| 100 | CHAD | CHAD Entrez,  Source | chondroadherin | 8843 | -0.017 | 0.1610 | No |
| 101 | RAF1 | RAF1 Entrez,  Source | v-raf-1 murine leukemia viral oncogene homolog 1 | 9036 | -0.019 | 0.1519 | No |
| 102 | PDPK1 | PDPK1 Entrez,  Source | 3-phosphoinositide dependent protein kinase-1 | 9057 | -0.019 | 0.1517 | No |
| 103 | ACTN4 | ACTN4 Entrez,  Source | actinin, alpha 4 | 9236 | -0.021 | 0.1434 | No |
| 104 | MYLPF | MYLPF Entrez,  Source | - | 9361 | -0.023 | 0.1380 | No |
| 105 | RAC1 | RAC1 Entrez,  Source | ras-related C3 botulinum toxin substrate 1 (rho family, small GTP binding protein Rac1) | 9406 | -0.023 | 0.1367 | No |
| 106 | DIAPH1 | DIAPH1 Entrez,  Source | diaphanous homolog 1 (Drosophila) | 9659 | -0.026 | 0.1248 | No |
| 107 | ZYX | ZYX Entrez,  Source | zyxin | 9694 | -0.027 | 0.1242 | No |
| 108 | PIP5K1C | PIP5K1C Entrez,  Source | phosphatidylinositol-4-phosphate 5-kinase, type I, gamma | 9760 | -0.027 | 0.1220 | No |
| 109 | FLT4 | FLT4 Entrez,  Source | fms-related tyrosine kinase 4 | 9780 | -0.028 | 0.1222 | No |
| 110 | MYL9 | MYL9 Entrez,  Source | myosin, light chain 9, regulatory | 9812 | -0.028 | 0.1217 | No |
| 111 | ITGA8 | ITGA8 Entrez,  Source | integrin, alpha 8 | 9908 | -0.029 | 0.1181 | No |
| 112 | LAMC1 | LAMC1 Entrez,  Source | laminin, gamma 1 (formerly LAMB2) | 9927 | -0.029 | 0.1184 | No |
| 113 | MAPK1 | MAPK1 Entrez,  Source | mitogen-activated protein kinase 1 | 10004 | -0.030 | 0.1158 | No |
| 114 | ELK1 | ELK1 Entrez,  Source | ELK1, member of ETS oncogene family | 10053 | -0.031 | 0.1146 | No |
| 115 | PIK3R3 | PIK3R3 Entrez,  Source | phosphoinositide-3-kinase, regulatory subunit 3 (p55, gamma) | 10110 | -0.031 | 0.1130 | No |
| 116 | PIK3R5 | PIK3R5 Entrez,  Source | phosphoinositide-3-kinase, regulatory subunit 5, p101 | 10124 | -0.031 | 0.1137 | No |
| 117 | PAK7 | PAK7 Entrez,  Source | p21(CDKN1A)-activated kinase 7 | 10370 | -0.034 | 0.1025 | No |
| 118 | ITGA4 | ITGA4 Entrez,  Source | integrin, alpha 4 (antigen CD49D, alpha 4 subunit of VLA-4 receptor) | 10572 | -0.037 | 0.0937 | No |
| 119 | PAK2 | PAK2 Entrez,  Source | p21 (CDKN1A)-activated kinase 2 | 10573 | -0.037 | 0.0953 | No |
| 120 | MAPK3 | MAPK3 Entrez,  Source | mitogen-activated protein kinase 3 | 10580 | -0.037 | 0.0965 | No |
| 121 | THBS3 | THBS3 Entrez,  Source | thrombospondin 3 | 10680 | -0.038 | 0.0930 | No |
| 122 | COL6A2 | COL6A2 Entrez,  Source | collagen, type VI, alpha 2 | 10684 | -0.038 | 0.0945 | No |
| 123 | PIK3CG | PIK3CG Entrez,  Source | phosphoinositide-3-kinase, catalytic, gamma polypeptide | 10795 | -0.039 | 0.0905 | No |
| 124 | ERBB2 | ERBB2 Entrez,  Source | v-erb-b2 erythroblastic leukemia viral oncogene homolog 2, neuro/glioblastoma derived oncogene homolog (avian) | 10899 | -0.041 | 0.0869 | No |
| 125 | COL11A2 | COL11A2 Entrez,  Source | collagen, type XI, alpha 2 | 10911 | -0.041 | 0.0881 | No |
| 126 | VWF | VWF Entrez,  Source | von Willebrand factor | 11029 | -0.043 | 0.0839 | No |
| 127 | ITGA5 | ITGA5 Entrez,  Source | integrin, alpha 5 (fibronectin receptor, alpha polypeptide) | 11109 | -0.043 | 0.0817 | No |
| 128 | LAMB3 | LAMB3 Entrez,  Source | laminin, beta 3 | 11110 | -0.043 | 0.0835 | No |
| 129 | ROCK1 | ROCK1 Entrez,  Source | Rho-associated, coiled-coil containing protein kinase 1 | 11335 | -0.046 | 0.0740 | No |
| 130 | PIK3CA | PIK3CA Entrez,  Source | phosphoinositide-3-kinase, catalytic, alpha polypeptide | 11588 | -0.049 | 0.0631 | No |
| 131 | COL5A3 | COL5A3 Entrez,  Source | collagen, type V, alpha 3 | 11626 | -0.050 | 0.0633 | No |
| 132 | SOS1 | SOS1 Entrez,  Source | son of sevenless homolog 1 (Drosophila) | 11673 | -0.050 | 0.0630 | No |
| 133 | ITGB3 | ITGB3 Entrez,  Source | integrin, beta 3 (platelet glycoprotein IIIa, antigen CD61) | 11699 | -0.051 | 0.0639 | No |
| 134 | COL11A1 | COL11A1 Entrez,  Source | collagen, type XI, alpha 1 | 11776 | -0.051 | 0.0622 | No |
| 135 | LAMB4 | LAMB4 Entrez,  Source | laminin, beta 4 | 12018 | -0.055 | 0.0521 | No |
| 136 | CAV3 | CAV3 Entrez,  Source | caveolin 3 | 12045 | -0.055 | 0.0531 | No |
| 137 | THBS4 | THBS4 Entrez,  Source | thrombospondin 4 | 12206 | -0.057 | 0.0472 | No |
| 138 | PARVG | PARVG Entrez,  Source | parvin, gamma | 12513 | -0.061 | 0.0340 | No |
| 139 | MAPK10 | MAPK10 Entrez,  Source | mitogen-activated protein kinase 10 | 12600 | -0.062 | 0.0322 | No |
| 140 | LAMC2 | LAMC2 Entrez,  Source | laminin, gamma 2 | 12750 | -0.063 | 0.0272 | No |
| 141 | MYLK2 | MYLK2 Entrez,  Source | myosin light chain kinase 2, skeletal muscle | 12780 | -0.064 | 0.0285 | No |
| 142 | VAV1 | VAV1 Entrez,  Source | vav 1 oncogene | 12844 | -0.065 | 0.0280 | No |
| 143 | KDR | KDR Entrez,  Source | kinase insert domain receptor (a type III receptor tyrosine kinase) | 12913 | -0.065 | 0.0273 | No |
| 144 | COL3A1 | COL3A1 Entrez,  Source | collagen, type III, alpha 1 (Ehlers-Danlos syndrome type IV, autosomal dominant) | 13010 | -0.067 | 0.0251 | No |
| 145 | ACTN2 | ACTN2 Entrez,  Source | actinin, alpha 2 | 13118 | -0.068 | 0.0225 | No |
| 146 | ACTN3 | ACTN3 Entrez,  Source | actinin, alpha 3 | 13270 | -0.070 | 0.0177 | No |
| 147 | PAK1 | PAK1 Entrez,  Source | p21/Cdc42/Rac1-activated kinase 1 (STE20 homolog, yeast) | 13352 | -0.071 | 0.0166 | No |
| 148 | LAMA4 | LAMA4 Entrez,  Source | laminin, alpha 4 | 14214 | -0.082 | -0.0243 | No |
| 149 | COL4A6 | COL4A6 Entrez,  Source | collagen, type IV, alpha 6 | 14340 | -0.084 | -0.0272 | No |
| 150 | LAMA1 | LAMA1 Entrez,  Source | laminin, alpha 1 | 14411 | -0.085 | -0.0272 | No |
| 151 | TNR | TNR Entrez,  Source | tenascin R (restrictin, janusin) | 14441 | -0.085 | -0.0250 | No |
| 152 | PIK3R2 | PIK3R2 Entrez,  Source | phosphoinositide-3-kinase, regulatory subunit 2 (p85 beta) | 14488 | -0.086 | -0.0237 | No |
| 153 | VAV3 | VAV3 Entrez,  Source | vav 3 oncogene | 14742 | -0.090 | -0.0330 | No |
| 154 | PDGFRA | PDGFRA Entrez,  Source | platelet-derived growth factor receptor, alpha polypeptide | 14940 | -0.093 | -0.0392 | No |
| 155 | FYN | FYN Entrez,  Source | FYN oncogene related to SRC, FGR, YES | 14954 | -0.093 | -0.0359 | No |
| 156 | ITGA2B | ITGA2B Entrez,  Source | integrin, alpha 2b (platelet glycoprotein IIb of IIb/IIIa complex, antigen CD41) | 15161 | -0.097 | -0.0424 | No |
| 157 | COL1A2 | COL1A2 Entrez,  Source | collagen, type I, alpha 2 | 15172 | -0.097 | -0.0388 | No |
| 158 | PDGFRB | PDGFRB Entrez,  Source | platelet-derived growth factor receptor, beta polypeptide | 15354 | -0.100 | -0.0439 | No |
| 159 | RELN | RELN Entrez,  Source | reelin | 15378 | -0.100 | -0.0408 | No |
| 160 | SRC | SRC Entrez,  Source | v-src sarcoma (Schmidt-Ruppin A-2) viral oncogene homolog (avian) | 15539 | -0.103 | -0.0446 | No |
| 161 | ITGB7 | ITGB7 Entrez,  Source | integrin, beta 7 | 15809 | -0.108 | -0.0539 | No |
| 162 | ITGA11 | ITGA11 Entrez,  Source | integrin, alpha 11 | 15849 | -0.109 | -0.0513 | No |
| 163 | COL4A4 | COL4A4 Entrez,  Source | collagen, type IV, alpha 4 | 16048 | -0.112 | -0.0567 | No |
| 164 | LAMA2 | LAMA2 Entrez,  Source | laminin, alpha 2 (merosin, congenital muscular dystrophy) | 16160 | -0.114 | -0.0576 | No |
| 165 | PIK3CD | PIK3CD Entrez,  Source | phosphoinositide-3-kinase, catalytic, delta polypeptide | 16633 | -0.124 | -0.0767 | No |
| 166 | FIGF | FIGF Entrez,  Source | c-fos induced growth factor (vascular endothelial growth factor D) | 16676 | -0.125 | -0.0735 | No |
| 167 | MYL7 | MYL7 Entrez,  Source | myosin, light chain 7, regulatory | 16874 | -0.129 | -0.0781 | No |
| 168 | RASGRF1 | RASGRF1 Entrez,  Source | Ras protein-specific guanine nucleotide-releasing factor 1 | 17001 | -0.133 | -0.0790 | No |
| 169 | PAK6 | PAK6 Entrez,  Source | p21(CDKN1A)-activated kinase 6 | 17199 | -0.139 | -0.0832 | No |
| 170 | EGF | EGF Entrez,  Source | epidermal growth factor (beta-urogastrone) | 17577 | -0.151 | -0.0963 | No |
| 171 | TNXB | TNXB Entrez,  Source | tenascin XB | 17771 | -0.157 | -0.0995 | No |
| 172 | SHC2 | SHC2 Entrez,  Source | SHC (Src homology 2 domain containing) transforming protein 2 | 17786 | -0.158 | -0.0935 | No |
| 173 | IBSP | IBSP Entrez,  Source | integrin-binding sialoprotein (bone sialoprotein, bone sialoprotein II) | 17851 | -0.161 | -0.0899 | No |
| 174 | LAMC3 | LAMC3 Entrez,  Source | laminin, gamma 3 | 17882 | -0.162 | -0.0846 | No |
| 175 | ITGA9 | ITGA9 Entrez,  Source | integrin, alpha 9 | 18035 | -0.168 | -0.0852 | No |
| 176 | VEGFC | VEGFC Entrez,  Source | vascular endothelial growth factor C | 18328 | -0.182 | -0.0925 | No |
| 177 | COL4A2 | COL4A2 Entrez,  Source | collagen, type IV, alpha 2 | 18730 | -0.211 | -0.1042 | No |
| 178 | PAK3 | PAK3 Entrez,  Source | p21 (CDKN1A)-activated kinase 3 | 18790 | -0.216 | -0.0981 | No |
| 179 | PDGFD | PDGFD Entrez,  Source | platelet derived growth factor D | 18801 | -0.217 | -0.0893 | No |
| 180 | TNN | TNN Entrez,  Source | tenascin N | 18831 | -0.221 | -0.0813 | No |
| 181 | FLNC | FLNC Entrez,  Source | filamin C, gamma (actin binding protein 280) | 18936 | -0.235 | -0.0767 | No |
| 182 | CTNNB1 | CTNNB1 Entrez,  Source | catenin (cadherin-associated protein), beta 1, 88kDa | 18943 | -0.235 | -0.0669 | No |
| 183 | COL5A1 | COL5A1 Entrez,  Source | collagen, type V, alpha 1 | 18981 | -0.240 | -0.0586 | No |
| 184 | THBS2 | THBS2 Entrez,  Source | thrombospondin 2 | 19096 | -0.259 | -0.0534 | No |
| 185 | IGF1 | IGF1 Entrez,  Source | insulin-like growth factor 1 (somatomedin C) | 19223 | -0.294 | -0.0474 | No |
| 186 | COL2A1 | COL2A1 Entrez,  Source | collagen, type II, alpha 1 (primary osteoarthritis, spondyloepiphyseal dysplasia, congenital) | 19469 | -0.485 | -0.0393 | No |
| 187 | CCND2 | CCND2 Entrez,  Source | cyclin D2 | 19551 | -1.024 | 0.0003 | No |
Table: GSEA details [plain text format]

  

Fig 2: KEGG\_FOCAL\_ADHESION      
 Blue-Pink O' Gram in the Space of the Analyzed GeneSet

  

Fig 3: KEGG\_FOCAL\_ADHESION: Random ES distribution      
 Gene set null distribution of ES for **KEGG\_FOCAL\_ADHESION**

  
