# Supplementary material for: Comprehensive analysis of β-catenin target genes in colorectal carcinoma cell lines with deregulated Wnt/β-catenin signaling
Source: BMC Genomics. 2014 Jan 28;15:74. doi: 10.1186/1471-2164-15-74 (PMC3909937; doi:10.1186/1471-2164-15-74)
Supplement: Additional file 5 — GSEA analysis using the KEGG pathway database. This zipped file contains confirming data of the GSEA analysis. The names of the directories containing the files were composed of the term ‘GSEA’, the name of the cell line, e.g. DLD1, SW480, or LS174T, and the pathway database (KEGG). Please use a web browser to view the files with the name ‘index.html’ in the corresponding directories to start exploring the data. [file 1471-2164-15-74-S5.zip › GSEA KEGG SW480/KEGG_FRUCTOSE_AND_MANNOSE_METABOLISM.html]

Details for gene set KEGG\_FRUCTOSE\_AND\_MANNOSE\_METABOLISM[GSEA]

|  || Dataset | SW480\_collapsed\_to\_symbols.class.cls#b\_versus\_bg.class.cls#b\_versus\_bg\_repos |
| Phenotype | class.cls#b\_versus\_bg\_repos |
| Upregulated in class | 1 |
| GeneSet | KEGG\_FRUCTOSE\_AND\_MANNOSE\_METABOLISM |
| Enrichment Score (ES) | 0.5916772 |
| Normalized Enrichment Score (NES) | 1.9299794 |
| Nominal p-value | 0.0 |
| FDR q-value | 0.033301122 |
| FWER p-Value | 0.044 |
Table: GSEA Results Summary

  

Fig 1: Enrichment plot: KEGG\_FRUCTOSE\_AND\_MANNOSE\_METABOLISM      
 Profile of the Running ES Score & Positions of GeneSet Members on the Rank Ordered List

  

| PROBE | GENE SYMBOL | GENE\_TITLE | RANK IN GENE LIST | RANK METRIC SCORE | RUNNING ES | CORE ENRICHMENT || 1 | FPGT | FPGT Entrez,  Source | fucose-1-phosphate guanylyltransferase | 54 | 0.636 | 0.1970 | Yes |
| 2 | GMPPA | GMPPA Entrez,  Source | GDP-mannose pyrophosphorylase A | 703 | 0.233 | 0.2371 | Yes |
| 3 | GMPPB | GMPPB Entrez,  Source | GDP-mannose pyrophosphorylase B | 726 | 0.229 | 0.3077 | Yes |
| 4 | AKR1B10 | AKR1B10 Entrez,  Source | aldo-keto reductase family 1, member B10 (aldose reductase) | 805 | 0.216 | 0.3716 | Yes |
| 5 | FBP1 | FBP1 Entrez,  Source | fructose-1,6-bisphosphatase 1 | 953 | 0.195 | 0.4254 | Yes |
| 6 | MPI | MPI Entrez,  Source | mannose phosphate isomerase | 1476 | 0.149 | 0.4454 | Yes |
| 7 | PFKFB4 | PFKFB4 Entrez,  Source | 6-phosphofructo-2-kinase/fructose-2,6-biphosphatase 4 | 1514 | 0.147 | 0.4896 | Yes |
| 8 | PFKFB3 | PFKFB3 Entrez,  Source | 6-phosphofructo-2-kinase/fructose-2,6-biphosphatase 3 | 1878 | 0.126 | 0.5107 | Yes |
| 9 | PMM1 | PMM1 Entrez,  Source | phosphomannomutase 1 | 2153 | 0.114 | 0.5324 | Yes |
| 10 | PFKL | PFKL Entrez,  Source | phosphofructokinase, liver | 2479 | 0.101 | 0.5474 | Yes |
| 11 | GMDS | GMDS Entrez,  Source | GDP-mannose 4,6-dehydratase | 2813 | 0.089 | 0.5581 | Yes |
| 12 | PFKP | PFKP Entrez,  Source | phosphofructokinase, platelet | 3004 | 0.082 | 0.5743 | Yes |
| 13 | MTMR6 | MTMR6 Entrez,  Source | myotubularin related protein 6 | 3206 | 0.076 | 0.5879 | Yes |
| 14 | MTMR2 | MTMR2 Entrez,  Source | myotubularin related protein 2 | 3541 | 0.067 | 0.5917 | Yes |
| 15 | AKR1B1 | AKR1B1 Entrez,  Source | aldo-keto reductase family 1, member B1 (aldose reductase) | 3965 | 0.057 | 0.5878 | No |
| 16 | MTMR7 | MTMR7 Entrez,  Source | myotubularin related protein 7 | 4999 | 0.035 | 0.5459 | No |
| 17 | ALDOA | ALDOA Entrez,  Source | aldolase A, fructose-bisphosphate | 5003 | 0.035 | 0.5568 | No |
| 18 | MTMR1 | MTMR1 Entrez,  Source | myotubularin related protein 1 | 5693 | 0.024 | 0.5290 | No |
| 19 | TPI1 | TPI1 Entrez,  Source | triosephosphate isomerase 1 | 5859 | 0.022 | 0.5274 | No |
| 20 | FUK | FUK Entrez,  Source | fucokinase | 7118 | 0.004 | 0.4643 | No |
| 21 | ALDOB | ALDOB Entrez,  Source | aldolase B, fructose-bisphosphate | 7357 | 0.001 | 0.4524 | No |
| 22 | PFKM | PFKM Entrez,  Source | phosphofructokinase, muscle | 7597 | -0.002 | 0.4407 | No |
| 23 | HK1 | HK1 Entrez,  Source | hexokinase 1 | 7823 | -0.005 | 0.4307 | No |
| 24 | PFKFB2 | PFKFB2 Entrez,  Source | 6-phosphofructo-2-kinase/fructose-2,6-biphosphatase 2 | 7888 | -0.006 | 0.4292 | No |
| 25 | PHPT1 | PHPT1 Entrez,  Source | phosphohistidine phosphatase 1 | 8274 | -0.010 | 0.4127 | No |
| 26 | FBP2 | FBP2 Entrez,  Source | fructose-1,6-bisphosphatase 2 | 9028 | -0.019 | 0.3801 | No |
| 27 | HK2 | HK2 Entrez,  Source | hexokinase 2 | 9703 | -0.027 | 0.3539 | No |
| 28 | KHK | KHK Entrez,  Source | ketohexokinase (fructokinase) | 9746 | -0.027 | 0.3603 | No |
| 29 | PFKFB1 | PFKFB1 Entrez,  Source | 6-phosphofructo-2-kinase/fructose-2,6-biphosphatase 1 | 10040 | -0.030 | 0.3548 | No |
| 30 | PMM2 | PMM2 Entrez,  Source | phosphomannomutase 2 | 10589 | -0.037 | 0.3383 | No |
| 31 | TSTA3 | TSTA3 Entrez,  Source | tissue specific transplantation antigen P35B | 11956 | -0.054 | 0.2853 | No |
| 32 | HK3 | HK3 Entrez,  Source | hexokinase 3 (white cell) | 13624 | -0.075 | 0.2233 | No |
| 33 | SORD | SORD Entrez,  Source | sorbitol dehydrogenase | 14026 | -0.080 | 0.2279 | No |
| 34 | ALDOC | ALDOC Entrez,  Source | aldolase C, fructose-bisphosphate | 18205 | -0.176 | 0.0692 | No |
Table: GSEA details [plain text format]

  

Fig 2: KEGG\_FRUCTOSE\_AND\_MANNOSE\_METABOLISM      
 Blue-Pink O' Gram in the Space of the Analyzed GeneSet

  

Fig 3: KEGG\_FRUCTOSE\_AND\_MANNOSE\_METABOLISM: Random ES distribution      
 Gene set null distribution of ES for **KEGG\_FRUCTOSE\_AND\_MANNOSE\_METABOLISM**

  
