# Supplementary material for: Comprehensive analysis of β-catenin target genes in colorectal carcinoma cell lines with deregulated Wnt/β-catenin signaling
Source: BMC Genomics. 2014 Jan 28;15:74. doi: 10.1186/1471-2164-15-74 (PMC3909937; doi:10.1186/1471-2164-15-74)
Supplement: Additional file 5 — GSEA analysis using the KEGG pathway database. This zipped file contains confirming data of the GSEA analysis. The names of the directories containing the files were composed of the term ‘GSEA’, the name of the cell line, e.g. DLD1, SW480, or LS174T, and the pathway database (KEGG). Please use a web browser to view the files with the name ‘index.html’ in the corresponding directories to start exploring the data. [file 1471-2164-15-74-S5.zip › GSEA KEGG SW480/KEGG_GLYCEROLIPID_METABOLISM.html]

Details for gene set KEGG\_GLYCEROLIPID\_METABOLISM[GSEA]

|  || Dataset | SW480\_collapsed\_to\_symbols.class.cls#b\_versus\_bg.class.cls#b\_versus\_bg\_repos |
| Phenotype | class.cls#b\_versus\_bg\_repos |
| Upregulated in class | 1 |
| GeneSet | KEGG\_GLYCEROLIPID\_METABOLISM |
| Enrichment Score (ES) | 0.45064434 |
| Normalized Enrichment Score (NES) | 1.5469512 |
| Nominal p-value | 0.024630541 |
| FDR q-value | 0.12321461 |
| FWER p-Value | 0.783 |
Table: GSEA Results Summary

  

Fig 1: Enrichment plot: KEGG\_GLYCEROLIPID\_METABOLISM      
 Profile of the Running ES Score & Positions of GeneSet Members on the Rank Ordered List

  

| PROBE | GENE SYMBOL | GENE\_TITLE | RANK IN GENE LIST | RANK METRIC SCORE | RUNNING ES | CORE ENRICHMENT || 1 | LIPG | LIPG Entrez,  Source | lipase, endothelial | 44 | 0.676 | 0.1273 | Yes |
| 2 | MGLL | MGLL Entrez,  Source | monoglyceride lipase | 239 | 0.375 | 0.1893 | Yes |
| 3 | LPL | LPL Entrez,  Source | lipoprotein lipase | 263 | 0.362 | 0.2576 | Yes |
| 4 | PNPLA3 | PNPLA3 Entrez,  Source | patatin-like phospholipase domain containing 3 | 549 | 0.260 | 0.2929 | Yes |
| 5 | PPAP2B | PPAP2B Entrez,  Source | phosphatidic acid phosphatase type 2B | 665 | 0.240 | 0.3330 | Yes |
| 6 | ALDH3A2 | ALDH3A2 Entrez,  Source | aldehyde dehydrogenase 3 family, member A2 | 1015 | 0.188 | 0.3512 | Yes |
| 7 | MBOAT1 | MBOAT1 Entrez,  Source | membrane bound O-acyltransferase domain containing 1 | 1040 | 0.186 | 0.3856 | Yes |
| 8 | DGAT2 | DGAT2 Entrez,  Source | diacylglycerol O-acyltransferase homolog 2 (mouse) | 1070 | 0.183 | 0.4192 | Yes |
| 9 | PPAP2C | PPAP2C Entrez,  Source | phosphatidic acid phosphatase type 2C | 1353 | 0.158 | 0.4351 | Yes |
| 10 | DGKH | DGKH Entrez,  Source | diacylglycerol kinase, eta | 1581 | 0.142 | 0.4506 | Yes |
| 11 | ALDH9A1 | ALDH9A1 Entrez,  Source | aldehyde dehydrogenase 9 family, member A1 | 3008 | 0.082 | 0.3933 | No |
| 12 | DGKQ | DGKQ Entrez,  Source | diacylglycerol kinase, theta 110kDa | 3099 | 0.079 | 0.4040 | No |
| 13 | ALDH7A1 | ALDH7A1 Entrez,  Source | aldehyde dehydrogenase 7 family, member A1 | 3132 | 0.078 | 0.4173 | No |
| 14 | AKR1B1 | AKR1B1 Entrez,  Source | aldo-keto reductase family 1, member B1 (aldose reductase) | 3965 | 0.057 | 0.3855 | No |
| 15 | CEL | CEL Entrez,  Source | carboxyl ester lipase (bile salt-stimulated lipase) | 4141 | 0.052 | 0.3866 | No |
| 16 | PNLIPRP1 | PNLIPRP1 Entrez,  Source | pancreatic lipase-related protein 1 | 4232 | 0.050 | 0.3917 | No |
| 17 | GK2 | GK2 Entrez,  Source | glycerol kinase 2 | 4729 | 0.041 | 0.3740 | No |
| 18 | AKR1A1 | AKR1A1 Entrez,  Source | aldo-keto reductase family 1, member A1 (aldehyde reductase) | 5188 | 0.032 | 0.3567 | No |
| 19 | GLYCTK | GLYCTK Entrez,  Source | - | 5407 | 0.029 | 0.3510 | No |
| 20 | PPAP2A | PPAP2A Entrez,  Source | phosphatidic acid phosphatase type 2A | 6185 | 0.017 | 0.3144 | No |
| 21 | DGKI | DGKI Entrez,  Source | diacylglycerol kinase, iota | 6892 | 0.007 | 0.2796 | No |
| 22 | DAK | DAK Entrez,  Source | dihydroxyacetone kinase 2 homolog (S. cerevisiae) | 7615 | -0.002 | 0.2430 | No |
| 23 | GLA | GLA Entrez,  Source | galactosidase, alpha | 7822 | -0.005 | 0.2334 | No |
| 24 | AGPAT2 | AGPAT2 Entrez,  Source | 1-acylglycerol-3-phosphate O-acyltransferase 2 (lysophosphatidic acid acyltransferase, beta) | 7976 | -0.007 | 0.2268 | No |
| 25 | LIPF | LIPF Entrez,  Source | lipase, gastric | 8554 | -0.013 | 0.1998 | No |
| 26 | DGKG | DGKG Entrez,  Source | diacylglycerol kinase, gamma 90kDa | 8944 | -0.018 | 0.1833 | No |
| 27 | AGPAT3 | AGPAT3 Entrez,  Source | 1-acylglycerol-3-phosphate O-acyltransferase 3 | 9483 | -0.024 | 0.1604 | No |
| 28 | LIPC | LIPC Entrez,  Source | lipase, hepatic | 10207 | -0.032 | 0.1296 | No |
| 29 | DGKZ | DGKZ Entrez,  Source | diacylglycerol kinase, zeta 104kDa | 10492 | -0.036 | 0.1219 | No |
| 30 | DGKB | DGKB Entrez,  Source | diacylglycerol kinase, beta 90kDa | 11430 | -0.047 | 0.0829 | No |
| 31 | ALDH2 | ALDH2 Entrez,  Source | aldehyde dehydrogenase 2 family (mitochondrial) | 12077 | -0.055 | 0.0604 | No |
| 32 | AGPAT1 | AGPAT1 Entrez,  Source | 1-acylglycerol-3-phosphate O-acyltransferase 1 (lysophosphatidic acid acyltransferase, alpha) | 12348 | -0.058 | 0.0578 | No |
| 33 | DGKA | DGKA Entrez,  Source | diacylglycerol kinase, alpha 80kDa | 12615 | -0.062 | 0.0560 | No |
| 34 | MBOAT2 | MBOAT2 Entrez,  Source | membrane bound O-acyltransferase domain containing 2 | 13072 | -0.068 | 0.0456 | No |
| 35 | DGKE | DGKE Entrez,  Source | diacylglycerol kinase, epsilon 64kDa | 14224 | -0.083 | 0.0024 | No |
| 36 | AGPAT6 | AGPAT6 Entrez,  Source | 1-acylglycerol-3-phosphate O-acyltransferase 6 (lysophosphatidic acid acyltransferase, zeta) | 14461 | -0.086 | 0.0068 | No |
| 37 | GK | GK Entrez,  Source | glycerol kinase | 14741 | -0.090 | 0.0097 | No |
| 38 | GPAM | GPAM Entrez,  Source | glycerol-3-phosphate acyltransferase, mitochondrial | 15060 | -0.095 | 0.0115 | No |
| 39 | DGKD | DGKD Entrez,  Source | diacylglycerol kinase, delta 130kDa | 16105 | -0.113 | -0.0203 | No |
| 40 | ALDH1B1 | ALDH1B1 Entrez,  Source | aldehyde dehydrogenase 1 family, member B1 | 16646 | -0.124 | -0.0241 | No |
| 41 | PNLIP | PNLIP Entrez,  Source | pancreatic lipase | 18812 | -0.218 | -0.0932 | No |
| 42 | AGPAT4 | AGPAT4 Entrez,  Source | 1-acylglycerol-3-phosphate O-acyltransferase 4 (lysophosphatidic acid acyltransferase, delta) | 19062 | -0.254 | -0.0572 | No |
| 43 | PNLIPRP2 | PNLIPRP2 Entrez,  Source | pancreatic lipase-related protein 2 | 19425 | -0.430 | 0.0067 | No |
Table: GSEA details [plain text format]

  

Fig 2: KEGG\_GLYCEROLIPID\_METABOLISM      
 Blue-Pink O' Gram in the Space of the Analyzed GeneSet

  

Fig 3: KEGG\_GLYCEROLIPID\_METABOLISM: Random ES distribution      
 Gene set null distribution of ES for **KEGG\_GLYCEROLIPID\_METABOLISM**

  
