# Supplementary material for: Comprehensive analysis of β-catenin target genes in colorectal carcinoma cell lines with deregulated Wnt/β-catenin signaling
Source: BMC Genomics. 2014 Jan 28;15:74. doi: 10.1186/1471-2164-15-74 (PMC3909937; doi:10.1186/1471-2164-15-74)
Supplement: Additional file 5 — GSEA analysis using the KEGG pathway database. This zipped file contains confirming data of the GSEA analysis. The names of the directories containing the files were composed of the term ‘GSEA’, the name of the cell line, e.g. DLD1, SW480, or LS174T, and the pathway database (KEGG). Please use a web browser to view the files with the name ‘index.html’ in the corresponding directories to start exploring the data. [file 1471-2164-15-74-S5.zip › GSEA KEGG SW480/KEGG_GLYCINE_SERINE_AND_THREONINE_METABOLISM.html]

Details for gene set KEGG\_GLYCINE\_SERINE\_AND\_THREONINE\_METABOLISM[GSEA]

|  || Dataset | SW480\_collapsed\_to\_symbols.class.cls#b\_versus\_bg.class.cls#b\_versus\_bg\_repos |
| Phenotype | class.cls#b\_versus\_bg\_repos |
| Upregulated in class | 0 |
| GeneSet | KEGG\_GLYCINE\_SERINE\_AND\_THREONINE\_METABOLISM |
| Enrichment Score (ES) | -0.514121 |
| Normalized Enrichment Score (NES) | -1.6023695 |
| Nominal p-value | 0.017331023 |
| FDR q-value | 0.1140202 |
| FWER p-Value | 0.671 |
Table: GSEA Results Summary

  

Fig 1: Enrichment plot: KEGG\_GLYCINE\_SERINE\_AND\_THREONINE\_METABOLISM      
 Profile of the Running ES Score & Positions of GeneSet Members on the Rank Ordered List

  

| PROBE | GENE SYMBOL | GENE\_TITLE | RANK IN GENE LIST | RANK METRIC SCORE | RUNNING ES | CORE ENRICHMENT || 1 | SHMT1 | SHMT1 Entrez,  Source | serine hydroxymethyltransferase 1 (soluble) | 1987 | 0.121 | -0.0588 | No |
| 2 | ALAS1 | ALAS1 Entrez,  Source | aminolevulinate, delta-, synthase 1 | 2830 | 0.088 | -0.0708 | No |
| 3 | AMT | AMT Entrez,  Source | aminomethyltransferase | 4062 | 0.054 | -0.1146 | No |
| 4 | SRR | SRR Entrez,  Source | serine racemase | 4913 | 0.037 | -0.1451 | No |
| 5 | GLYCTK | GLYCTK Entrez,  Source | - | 5407 | 0.029 | -0.1602 | No |
| 6 | AGXT | AGXT Entrez,  Source | alanine-glyoxylate aminotransferase (oxalosis I; hyperoxaluria I; glycolicaciduria; serine-pyruvate aminotransferase) | 6269 | 0.016 | -0.1987 | No |
| 7 | PSPH | PSPH Entrez,  Source | phosphoserine phosphatase | 7397 | 0.001 | -0.2562 | No |
| 8 | BHMT | BHMT Entrez,  Source | betaine-homocysteine methyltransferase | 7567 | -0.001 | -0.2644 | No |
| 9 | GLDC | GLDC Entrez,  Source | glycine dehydrogenase (decarboxylating) | 8379 | -0.011 | -0.3019 | No |
| 10 | DLD | DLD Entrez,  Source | dihydrolipoamide dehydrogenase | 8516 | -0.013 | -0.3043 | No |
| 11 | MAOA | MAOA Entrez,  Source | monoamine oxidase A | 9475 | -0.024 | -0.3448 | No |
| 12 | PIPOX | PIPOX Entrez,  Source | pipecolic acid oxidase | 10744 | -0.039 | -0.3960 | No |
| 13 | PHGDH | PHGDH Entrez,  Source | phosphoglycerate dehydrogenase | 10794 | -0.039 | -0.3846 | No |
| 14 | CHDH | CHDH Entrez,  Source | choline dehydrogenase | 11332 | -0.046 | -0.3957 | No |
| 15 | GATM | GATM Entrez,  Source | glycine amidinotransferase (L-arginine:glycine amidinotransferase) | 12375 | -0.059 | -0.4282 | No |
| 16 | DAO | DAO Entrez,  Source | D-amino-acid oxidase | 12517 | -0.061 | -0.4140 | No |
| 17 | SARDH | SARDH Entrez,  Source | sarcosine dehydrogenase | 14427 | -0.085 | -0.4816 | Yes |
| 18 | SHMT2 | SHMT2 Entrez,  Source | serine hydroxymethyltransferase 2 (mitochondrial) | 15063 | -0.095 | -0.4805 | Yes |
| 19 | SDS | SDS Entrez,  Source | serine dehydratase | 15387 | -0.100 | -0.4616 | Yes |
| 20 | DMGDH | DMGDH Entrez,  Source | dimethylglycine dehydrogenase | 15565 | -0.103 | -0.4341 | Yes |
| 21 | CBS | CBS Entrez,  Source | cystathionine-beta-synthase | 15580 | -0.103 | -0.3981 | Yes |
| 22 | AGXT2 | AGXT2 Entrez,  Source | alanine-glyoxylate aminotransferase 2 | 15866 | -0.109 | -0.3742 | Yes |
| 23 | GAMT | GAMT Entrez,  Source | guanidinoacetate N-methyltransferase | 15929 | -0.110 | -0.3384 | Yes |
| 24 | GCAT | GCAT Entrez,  Source | glycine C-acetyltransferase (2-amino-3-ketobutyrate coenzyme A ligase) | 16169 | -0.114 | -0.3100 | Yes |
| 25 | PSAT1 | PSAT1 Entrez,  Source | phosphoserine aminotransferase 1 | 16527 | -0.121 | -0.2853 | Yes |
| 26 | ALAS2 | ALAS2 Entrez,  Source | aminolevulinate, delta-, synthase 2 (sideroblastic/hypochromic anemia) | 17914 | -0.163 | -0.2985 | Yes |
| 27 | AOC3 | AOC3 Entrez,  Source | amine oxidase, copper containing 3 (vascular adhesion protein 1) | 18312 | -0.181 | -0.2545 | Yes |
| 28 | AOC2 | AOC2 Entrez,  Source | amine oxidase, copper containing 2 (retina-specific) | 18708 | -0.209 | -0.2008 | Yes |
| 29 | MAOB | MAOB Entrez,  Source | monoamine oxidase B | 18778 | -0.215 | -0.1281 | Yes |
| 30 | GNMT | GNMT Entrez,  Source | glycine N-methyltransferase | 18796 | -0.216 | -0.0523 | Yes |
| 31 | CTH | CTH Entrez,  Source | cystathionase (cystathionine gamma-lyase) | 19082 | -0.257 | 0.0243 | Yes |
Table: GSEA details [plain text format]

  

Fig 2: KEGG\_GLYCINE\_SERINE\_AND\_THREONINE\_METABOLISM      
 Blue-Pink O' Gram in the Space of the Analyzed GeneSet

  

Fig 3: KEGG\_GLYCINE\_SERINE\_AND\_THREONINE\_METABOLISM: Random ES distribution      
 Gene set null distribution of ES for **KEGG\_GLYCINE\_SERINE\_AND\_THREONINE\_METABOLISM**

  
