# Supplementary material for: Comprehensive analysis of β-catenin target genes in colorectal carcinoma cell lines with deregulated Wnt/β-catenin signaling
Source: BMC Genomics. 2014 Jan 28;15:74. doi: 10.1186/1471-2164-15-74 (PMC3909937; doi:10.1186/1471-2164-15-74)
Supplement: Additional file 5 — GSEA analysis using the KEGG pathway database. This zipped file contains confirming data of the GSEA analysis. The names of the directories containing the files were composed of the term ‘GSEA’, the name of the cell line, e.g. DLD1, SW480, or LS174T, and the pathway database (KEGG). Please use a web browser to view the files with the name ‘index.html’ in the corresponding directories to start exploring the data. [file 1471-2164-15-74-S5.zip › GSEA KEGG SW480/KEGG_GRAFT_VERSUS_HOST_DISEASE.html]

Details for gene set KEGG\_GRAFT\_VERSUS\_HOST\_DISEASE[GSEA]

|  || Dataset | SW480\_collapsed\_to\_symbols.class.cls#b\_versus\_bg.class.cls#b\_versus\_bg\_repos |
| Phenotype | class.cls#b\_versus\_bg\_repos |
| Upregulated in class | 0 |
| GeneSet | KEGG\_GRAFT\_VERSUS\_HOST\_DISEASE |
| Enrichment Score (ES) | -0.39115366 |
| Normalized Enrichment Score (NES) | -1.2431424 |
| Nominal p-value | 0.16666667 |
| FDR q-value | 0.5435674 |
| FWER p-Value | 1.0 |
Table: GSEA Results Summary

  

Fig 1: Enrichment plot: KEGG\_GRAFT\_VERSUS\_HOST\_DISEASE      
 Profile of the Running ES Score & Positions of GeneSet Members on the Rank Ordered List

  

| PROBE | GENE SYMBOL | GENE\_TITLE | RANK IN GENE LIST | RANK METRIC SCORE | RUNNING ES | CORE ENRICHMENT || 1 | HLA-A | HLA-A Entrez,  Source | major histocompatibility complex, class I, A | 1218 | 0.168 | -0.0133 | No |
| 2 | HLA-F | HLA-F Entrez,  Source | major histocompatibility complex, class I, F | 1341 | 0.159 | 0.0268 | No |
| 3 | IL1A | IL1A Entrez,  Source | interleukin 1, alpha | 1817 | 0.129 | 0.0401 | No |
| 4 | HLA-DMA | HLA-DMA Entrez,  Source | major histocompatibility complex, class II, DM alpha | 2247 | 0.110 | 0.0501 | No |
| 5 | HLA-G | HLA-G Entrez,  Source | HLA-G histocompatibility antigen, class I, G | 2852 | 0.087 | 0.0445 | No |
| 6 | HLA-DRB4 | HLA-DRB4 Entrez,  Source | major histocompatibility complex, class II, DR beta 4 | 3022 | 0.082 | 0.0597 | No |
| 7 | HLA-C | HLA-C Entrez,  Source | major histocompatibility complex, class I, C | 3264 | 0.074 | 0.0691 | No |
| 8 | HLA-B | HLA-B Entrez,  Source | major histocompatibility complex, class I, B | 3267 | 0.074 | 0.0907 | No |
| 9 | HLA-DRA | HLA-DRA Entrez,  Source | major histocompatibility complex, class II, DR alpha | 3456 | 0.069 | 0.1012 | No |
| 10 | KIR2DL5A | KIR2DL5A Entrez,  Source | killer cell immunoglobulin-like receptor, two domains, long cytoplasmic tail, 5A | 3888 | 0.058 | 0.0961 | No |
| 11 | FAS | FAS Entrez,  Source | Fas (TNF receptor superfamily, member 6) | 3978 | 0.056 | 0.1079 | No |
| 12 | KIR3DL1 | KIR3DL1 Entrez,  Source | killer cell immunoglobulin-like receptor, three domains, long cytoplasmic tail, 1 | 5256 | 0.031 | 0.0515 | No |
| 13 | CD86 | CD86 Entrez,  Source | CD86 molecule | 6293 | 0.015 | 0.0029 | No |
| 14 | HLA-E | HLA-E Entrez,  Source | major histocompatibility complex, class I, E | 6630 | 0.011 | -0.0112 | No |
| 15 | IFNG | IFNG Entrez,  Source | interferon, gamma | 6964 | 0.006 | -0.0264 | No |
| 16 | HLA-DPA1 | HLA-DPA1 Entrez,  Source | major histocompatibility complex, class II, DP alpha 1 | 7007 | 0.006 | -0.0269 | No |
| 17 | KIR2DL3 | KIR2DL3 Entrez,  Source | killer cell immunoglobulin-like receptor, two domains, long cytoplasmic tail, 3 | 7580 | -0.002 | -0.0557 | No |
| 18 | HLA-DQA1 | HLA-DQA1 Entrez,  Source | major histocompatibility complex, class II, DQ alpha 1 | 8795 | -0.016 | -0.1132 | No |
| 19 | CD28 | CD28 Entrez,  Source | CD28 molecule | 9542 | -0.025 | -0.1442 | No |
| 20 | IL6 | IL6 Entrez,  Source | interleukin 6 (interferon, beta 2) | 9785 | -0.028 | -0.1486 | No |
| 21 | KIR2DL2 | KIR2DL2 Entrez,  Source | killer cell immunoglobulin-like receptor, two domains, long cytoplasmic tail, 2 | 9790 | -0.028 | -0.1408 | No |
| 22 | HLA-DOA | HLA-DOA Entrez,  Source | major histocompatibility complex, class II, DO alpha | 11429 | -0.047 | -0.2109 | No |
| 23 | IL2 | IL2 Entrez,  Source | interleukin 2 | 14448 | -0.085 | -0.3405 | No |
| 24 | HLA-DPB1 | HLA-DPB1 Entrez,  Source | major histocompatibility complex, class II, DP beta 1 | 15437 | -0.101 | -0.3617 | Yes |
| 25 | KIR2DL1 | KIR2DL1 Entrez,  Source | killer cell immunoglobulin-like receptor, two domains, long cytoplasmic tail, 1 | 15465 | -0.101 | -0.3335 | Yes |
| 26 | IL1B | IL1B Entrez,  Source | interleukin 1, beta | 15552 | -0.103 | -0.3079 | Yes |
| 27 | HLA-DRB1 | HLA-DRB1 Entrez,  Source | major histocompatibility complex, class II, DR beta 1 | 16236 | -0.116 | -0.3092 | Yes |
| 28 | PRF1 | PRF1 Entrez,  Source | perforin 1 (pore forming protein) | 16257 | -0.116 | -0.2763 | Yes |
| 29 | KLRD1 | KLRD1 Entrez,  Source | killer cell lectin-like receptor subfamily D, member 1 | 16703 | -0.125 | -0.2625 | Yes |
| 30 | CD80 | CD80 Entrez,  Source | CD80 molecule | 16726 | -0.126 | -0.2269 | Yes |
| 31 | HLA-DMB | HLA-DMB Entrez,  Source | major histocompatibility complex, class II, DM beta | 17061 | -0.134 | -0.2048 | Yes |
| 32 | TNF | TNF Entrez,  Source | tumor necrosis factor (TNF superfamily, member 2) | 17349 | -0.143 | -0.1777 | Yes |
| 33 | HLA-DQB1 | HLA-DQB1 Entrez,  Source | major histocompatibility complex, class II, DQ beta 1 | 17788 | -0.158 | -0.1541 | Yes |
| 34 | HLA-DOB | HLA-DOB Entrez,  Source | major histocompatibility complex, class II, DO beta | 18287 | -0.181 | -0.1270 | Yes |
| 35 | FASLG | FASLG Entrez,  Source | Fas ligand (TNF superfamily, member 6) | 18956 | -0.237 | -0.0921 | Yes |
| 36 | GZMB | GZMB Entrez,  Source | granzyme B (granzyme 2, cytotoxic T-lymphocyte-associated serine esterase 1) | 19419 | -0.421 | 0.0070 | Yes |
Table: GSEA details [plain text format]

  

Fig 2: KEGG\_GRAFT\_VERSUS\_HOST\_DISEASE      
 Blue-Pink O' Gram in the Space of the Analyzed GeneSet

  

Fig 3: KEGG\_GRAFT\_VERSUS\_HOST\_DISEASE: Random ES distribution      
 Gene set null distribution of ES for **KEGG\_GRAFT\_VERSUS\_HOST\_DISEASE**

  
