# Supplementary material for: Comprehensive analysis of β-catenin target genes in colorectal carcinoma cell lines with deregulated Wnt/β-catenin signaling
Source: BMC Genomics. 2014 Jan 28;15:74. doi: 10.1186/1471-2164-15-74 (PMC3909937; doi:10.1186/1471-2164-15-74)
Supplement: Additional file 5 — GSEA analysis using the KEGG pathway database. This zipped file contains confirming data of the GSEA analysis. The names of the directories containing the files were composed of the term ‘GSEA’, the name of the cell line, e.g. DLD1, SW480, or LS174T, and the pathway database (KEGG). Please use a web browser to view the files with the name ‘index.html’ in the corresponding directories to start exploring the data. [file 1471-2164-15-74-S5.zip › GSEA KEGG SW480/KEGG_HEDGEHOG_SIGNALING_PATHWAY.html]

Details for gene set KEGG\_HEDGEHOG\_SIGNALING\_PATHWAY[GSEA]

|  || Dataset | SW480\_collapsed\_to\_symbols.class.cls#b\_versus\_bg.class.cls#b\_versus\_bg\_repos |
| Phenotype | class.cls#b\_versus\_bg\_repos |
| Upregulated in class | 0 |
| GeneSet | KEGG\_HEDGEHOG\_SIGNALING\_PATHWAY |
| Enrichment Score (ES) | -0.37786904 |
| Normalized Enrichment Score (NES) | -1.2949698 |
| Nominal p-value | 0.11404958 |
| FDR q-value | 0.50308233 |
| FWER p-Value | 1.0 |
Table: GSEA Results Summary

  

Fig 1: Enrichment plot: KEGG\_HEDGEHOG\_SIGNALING\_PATHWAY      
 Profile of the Running ES Score & Positions of GeneSet Members on the Rank Ordered List

  

| PROBE | GENE SYMBOL | GENE\_TITLE | RANK IN GENE LIST | RANK METRIC SCORE | RUNNING ES | CORE ENRICHMENT || 1 | LRP2 | LRP2 Entrez,  Source | low density lipoprotein-related protein 2 | 164 | 0.418 | 0.0539 | No |
| 2 | PRKX | PRKX Entrez,  Source | protein kinase, X-linked | 207 | 0.397 | 0.1109 | No |
| 3 | ZIC2 | ZIC2 Entrez,  Source | Zic family member 2 (odd-paired homolog, Drosophila) | 982 | 0.191 | 0.0997 | No |
| 4 | WNT7A | WNT7A Entrez,  Source | wingless-type MMTV integration site family, member 7A | 1917 | 0.125 | 0.0704 | No |
| 5 | WNT9A | WNT9A Entrez,  Source | wingless-type MMTV integration site family, member 9A | 1959 | 0.123 | 0.0866 | No |
| 6 | GLI3 | GLI3 Entrez,  Source | GLI-Kruppel family member GLI3 (Greig cephalopolysyndactyly syndrome) | 1967 | 0.122 | 0.1044 | No |
| 7 | RAB23 | RAB23 Entrez,  Source | RAB23, member RAS oncogene family | 2943 | 0.084 | 0.0670 | No |
| 8 | CSNK1G2 | CSNK1G2 Entrez,  Source | casein kinase 1, gamma 2 | 3224 | 0.075 | 0.0639 | No |
| 9 | PRKACB | PRKACB Entrez,  Source | protein kinase, cAMP-dependent, catalytic, beta | 3675 | 0.063 | 0.0502 | No |
| 10 | GLI2 | GLI2 Entrez,  Source | GLI-Kruppel family member GLI2 | 3882 | 0.058 | 0.0483 | No |
| 11 | CSNK1D | CSNK1D Entrez,  Source | casein kinase 1, delta | 4924 | 0.037 | 0.0004 | No |
| 12 | BMP8B | BMP8B Entrez,  Source | bone morphogenetic protein 8b (osteogenic protein 2) | 4932 | 0.037 | 0.0055 | No |
| 13 | WNT5A | WNT5A Entrez,  Source | wingless-type MMTV integration site family, member 5A | 5570 | 0.026 | -0.0233 | No |
| 14 | CSNK1A1 | CSNK1A1 Entrez,  Source | casein kinase 1, alpha 1 | 5884 | 0.021 | -0.0362 | No |
| 15 | WNT16 | WNT16 Entrez,  Source | wingless-type MMTV integration site family, member 16 | 5995 | 0.020 | -0.0389 | No |
| 16 | GSK3B | GSK3B Entrez,  Source | glycogen synthase kinase 3 beta | 6824 | 0.008 | -0.0801 | No |
| 17 | WNT10A | WNT10A Entrez,  Source | wingless-type MMTV integration site family, member 10A | 8181 | -0.009 | -0.1483 | No |
| 18 | CSNK1G1 | CSNK1G1 Entrez,  Source | casein kinase 1, gamma 1 | 8466 | -0.012 | -0.1610 | No |
| 19 | PRKACA | PRKACA Entrez,  Source | protein kinase, cAMP-dependent, catalytic, alpha | 8583 | -0.014 | -0.1649 | No |
| 20 | FBXW11 | FBXW11 Entrez,  Source | F-box and WD-40 domain protein 11 | 9412 | -0.023 | -0.2039 | No |
| 21 | WNT3 | WNT3 Entrez,  Source | wingless-type MMTV integration site family, member 3 | 9581 | -0.025 | -0.2088 | No |
| 22 | STK36 | STK36 Entrez,  Source | serine/threonine kinase 36 (fused homolog, Drosophila) | 10512 | -0.036 | -0.2511 | No |
| 23 | BMP8A | BMP8A Entrez,  Source | bone morphogenetic protein 8a | 11194 | -0.044 | -0.2794 | No |
| 24 | BMP4 | BMP4 Entrez,  Source | bone morphogenetic protein 4 | 11384 | -0.047 | -0.2821 | No |
| 25 | WNT1 | WNT1 Entrez,  Source | wingless-type MMTV integration site family, member 1 | 11756 | -0.051 | -0.2935 | No |
| 26 | CSNK1G3 | CSNK1G3 Entrez,  Source | casein kinase 1, gamma 3 | 12234 | -0.057 | -0.3095 | No |
| 27 | DHH | DHH Entrez,  Source | desert hedgehog homolog (Drosophila) | 12496 | -0.060 | -0.3139 | No |
| 28 | BMP7 | BMP7 Entrez,  Source | bone morphogenetic protein 7 (osteogenic protein 1) | 12982 | -0.066 | -0.3289 | No |
| 29 | GLI1 | GLI1 Entrez,  Source | glioma-associated oncogene homolog 1 (zinc finger protein) | 13632 | -0.075 | -0.3510 | No |
| 30 | IHH | IHH Entrez,  Source | Indian hedgehog homolog (Drosophila) | 13645 | -0.075 | -0.3404 | No |
| 31 | WNT9B | WNT9B Entrez,  Source | wingless-type MMTV integration site family, member 9B | 13833 | -0.078 | -0.3384 | No |
| 32 | BMP5 | BMP5 Entrez,  Source | bone morphogenetic protein 5 | 13860 | -0.078 | -0.3282 | No |
| 33 | WNT8B | WNT8B Entrez,  Source | wingless-type MMTV integration site family, member 8B | 13981 | -0.079 | -0.3225 | No |
| 34 | WNT10B | WNT10B Entrez,  Source | wingless-type MMTV integration site family, member 10B | 15062 | -0.095 | -0.3638 | Yes |
| 35 | BMP2 | BMP2 Entrez,  Source | bone morphogenetic protein 2 | 15195 | -0.097 | -0.3560 | Yes |
| 36 | GAS1 | GAS1 Entrez,  Source | growth arrest-specific 1 | 15401 | -0.100 | -0.3516 | Yes |
| 37 | CSNK1E | CSNK1E Entrez,  Source | casein kinase 1, epsilon | 15722 | -0.106 | -0.3522 | Yes |
| 38 | SMO | SMO Entrez,  Source | smoothened homolog (Drosophila) | 15857 | -0.109 | -0.3429 | Yes |
| 39 | WNT2B | WNT2B Entrez,  Source | wingless-type MMTV integration site family, member 2B | 16011 | -0.111 | -0.3341 | Yes |
| 40 | WNT4 | WNT4 Entrez,  Source | wingless-type MMTV integration site family, member 4 | 16063 | -0.112 | -0.3200 | Yes |
| 41 | WNT8A | WNT8A Entrez,  Source | wingless-type MMTV integration site family, member 8A | 16353 | -0.118 | -0.3172 | Yes |
| 42 | SUFU | SUFU Entrez,  Source | suppressor of fused homolog (Drosophila) | 16780 | -0.127 | -0.3201 | Yes |
| 43 | PTCH2 | PTCH2 Entrez,  Source | patched homolog 2 (Drosophila) | 17468 | -0.147 | -0.3333 | Yes |
| 44 | WNT2 | WNT2 Entrez,  Source | wingless-type MMTV integration site family member 2 | 17510 | -0.149 | -0.3133 | Yes |
| 45 | PRKACG | PRKACG Entrez,  Source | protein kinase, cAMP-dependent, catalytic, gamma | 18249 | -0.178 | -0.3245 | Yes |
| 46 | HHIP | HHIP Entrez,  Source | hedgehog interacting protein | 18621 | -0.202 | -0.3135 | Yes |
| 47 | PTCH1 | PTCH1 Entrez,  Source | patched homolog 1 (Drosophila) | 18816 | -0.219 | -0.2908 | Yes |
| 48 | BTRC | BTRC Entrez,  Source | beta-transducin repeat containing | 18818 | -0.219 | -0.2582 | Yes |
| 49 | SHH | SHH Entrez,  Source | sonic hedgehog homolog (Drosophila) | 19038 | -0.250 | -0.2322 | Yes |
| 50 | BMP6 | BMP6 Entrez,  Source | bone morphogenetic protein 6 | 19313 | -0.333 | -0.1966 | Yes |
| 51 | WNT11 | WNT11 Entrez,  Source | wingless-type MMTV integration site family, member 11 | 19383 | -0.388 | -0.1423 | Yes |
| 52 | WNT5B | WNT5B Entrez,  Source | wingless-type MMTV integration site family, member 5B | 19464 | -0.482 | -0.0746 | Yes |
| 53 | WNT6 | WNT6 Entrez,  Source | wingless-type MMTV integration site family, member 6 | 19488 | -0.532 | 0.0035 | Yes |
Table: GSEA details [plain text format]

  

Fig 2: KEGG\_HEDGEHOG\_SIGNALING\_PATHWAY      
 Blue-Pink O' Gram in the Space of the Analyzed GeneSet

  

Fig 3: KEGG\_HEDGEHOG\_SIGNALING\_PATHWAY: Random ES distribution      
 Gene set null distribution of ES for **KEGG\_HEDGEHOG\_SIGNALING\_PATHWAY**

  
