# Supplementary material for: Comprehensive analysis of β-catenin target genes in colorectal carcinoma cell lines with deregulated Wnt/β-catenin signaling
Source: BMC Genomics. 2014 Jan 28;15:74. doi: 10.1186/1471-2164-15-74 (PMC3909937; doi:10.1186/1471-2164-15-74)
Supplement: Additional file 5 — GSEA analysis using the KEGG pathway database. This zipped file contains confirming data of the GSEA analysis. The names of the directories containing the files were composed of the term ‘GSEA’, the name of the cell line, e.g. DLD1, SW480, or LS174T, and the pathway database (KEGG). Please use a web browser to view the files with the name ‘index.html’ in the corresponding directories to start exploring the data. [file 1471-2164-15-74-S5.zip › GSEA KEGG SW480/KEGG_HEMATOPOIETIC_CELL_LINEAGE.html]

Details for gene set KEGG\_HEMATOPOIETIC\_CELL\_LINEAGE[GSEA]

|  || Dataset | SW480\_collapsed\_to\_symbols.class.cls#b\_versus\_bg.class.cls#b\_versus\_bg\_repos |
| Phenotype | class.cls#b\_versus\_bg\_repos |
| Upregulated in class | 0 |
| GeneSet | KEGG\_HEMATOPOIETIC\_CELL\_LINEAGE |
| Enrichment Score (ES) | -0.359586 |
| Normalized Enrichment Score (NES) | -1.3460312 |
| Nominal p-value | 0.058528427 |
| FDR q-value | 0.41850835 |
| FWER p-Value | 1.0 |
Table: GSEA Results Summary

  

Fig 1: Enrichment plot: KEGG\_HEMATOPOIETIC\_CELL\_LINEAGE      
 Profile of the Running ES Score & Positions of GeneSet Members on the Rank Ordered List

  

| PROBE | GENE SYMBOL | GENE\_TITLE | RANK IN GENE LIST | RANK METRIC SCORE | RUNNING ES | CORE ENRICHMENT || 1 | ITGA3 | ITGA3 Entrez,  Source | integrin, alpha 3 (antigen CD49C, alpha 3 subunit of VLA-3 receptor) | 134 | 0.458 | 0.0411 | No |
| 2 | CSF2 | CSF2 Entrez,  Source | colony stimulating factor 2 (granulocyte-macrophage) | 327 | 0.328 | 0.0656 | No |
| 3 | CD59 | CD59 Entrez,  Source | CD59 molecule, complement regulatory protein | 586 | 0.253 | 0.0788 | No |
| 4 | ITGA6 | ITGA6 Entrez,  Source | integrin, alpha 6 | 1088 | 0.180 | 0.0720 | No |
| 5 | CD14 | CD14 Entrez,  Source | CD14 molecule | 1287 | 0.163 | 0.0789 | No |
| 6 | ITGA2 | ITGA2 Entrez,  Source | integrin, alpha 2 (CD49B, alpha 2 subunit of VLA-2 receptor) | 1768 | 0.131 | 0.0680 | No |
| 7 | IL1A | IL1A Entrez,  Source | interleukin 1, alpha | 1817 | 0.129 | 0.0790 | No |
| 8 | HLA-DRB4 | HLA-DRB4 Entrez,  Source | major histocompatibility complex, class II, DR beta 4 | 3022 | 0.082 | 0.0258 | No |
| 9 | THPO | THPO Entrez,  Source | thrombopoietin (myeloproliferative leukemia virus oncogene ligand, megakaryocyte growth and development factor) | 3032 | 0.081 | 0.0339 | No |
| 10 | IL7 | IL7 Entrez,  Source | interleukin 7 | 3146 | 0.078 | 0.0362 | No |
| 11 | HLA-DRA | HLA-DRA Entrez,  Source | major histocompatibility complex, class II, DR alpha | 3456 | 0.069 | 0.0276 | No |
| 12 | ITGAM | ITGAM Entrez,  Source | integrin, alpha M (complement component 3 receptor 3 subunit) | 3513 | 0.068 | 0.0318 | No |
| 13 | CR1 | CR1 Entrez,  Source | complement component (3b/4b) receptor 1 (Knops blood group) | 4144 | 0.052 | 0.0049 | No |
| 14 | IL9R | IL9R Entrez,  Source | interleukin 9 receptor | 4821 | 0.039 | -0.0257 | No |
| 15 | CD9 | CD9 Entrez,  Source | CD9 molecule | 6052 | 0.019 | -0.0869 | No |
| 16 | GP1BA | GP1BA Entrez,  Source | glycoprotein Ib (platelet), alpha polypeptide | 6380 | 0.014 | -0.1023 | No |
| 17 | IL7R | IL7R Entrez,  Source | interleukin 7 receptor | 7065 | 0.005 | -0.1369 | No |
| 18 | DNTT | DNTT Entrez,  Source | deoxynucleotidyltransferase, terminal | 7837 | -0.005 | -0.1759 | No |
| 19 | CSF1 | CSF1 Entrez,  Source | colony stimulating factor 1 (macrophage) | 7981 | -0.007 | -0.1826 | No |
| 20 | IL4R | IL4R Entrez,  Source | interleukin 4 receptor | 8654 | -0.014 | -0.2156 | No |
| 21 | TFRC | TFRC Entrez,  Source | transferrin receptor (p90, CD71) | 9093 | -0.020 | -0.2360 | No |
| 22 | IL11 | IL11 Entrez,  Source | interleukin 11 | 9643 | -0.026 | -0.2615 | No |
| 23 | TPO | TPO Entrez,  Source | thyroid peroxidase | 9693 | -0.027 | -0.2612 | No |
| 24 | IL6 | IL6 Entrez,  Source | interleukin 6 (interferon, beta 2) | 9785 | -0.028 | -0.2630 | No |
| 25 | CSF1R | CSF1R Entrez,  Source | colony stimulating factor 1 receptor, formerly McDonough feline sarcoma viral (v-fms) oncogene homolog | 10135 | -0.032 | -0.2776 | No |
| 26 | ITGA4 | ITGA4 Entrez,  Source | integrin, alpha 4 (antigen CD49D, alpha 4 subunit of VLA-4 receptor) | 10572 | -0.037 | -0.2962 | No |
| 27 | CD1E | CD1E Entrez,  Source | CD1e molecule | 10888 | -0.041 | -0.3081 | No |
| 28 | GP5 | GP5 Entrez,  Source | glycoprotein V (platelet) | 11007 | -0.042 | -0.3097 | No |
| 29 | ITGA5 | ITGA5 Entrez,  Source | integrin, alpha 5 (fibronectin receptor, alpha polypeptide) | 11109 | -0.043 | -0.3103 | No |
| 30 | CD3D | CD3D Entrez,  Source | CD3d molecule, delta (CD3-TCR complex) | 11272 | -0.045 | -0.3139 | No |
| 31 | IL3RA | IL3RA Entrez,  Source | interleukin 3 receptor, alpha (low affinity) | 11277 | -0.046 | -0.3093 | No |
| 32 | KIT | KIT Entrez,  Source | v-kit Hardy-Zuckerman 4 feline sarcoma viral oncogene homolog | 11285 | -0.046 | -0.3049 | No |
| 33 | CD3G | CD3G Entrez,  Source | CD3g molecule, gamma (CD3-TCR complex) | 11625 | -0.050 | -0.3171 | No |
| 34 | ITGB3 | ITGB3 Entrez,  Source | integrin, beta 3 (platelet glycoprotein IIIa, antigen CD61) | 11699 | -0.051 | -0.3155 | No |
| 35 | GP9 | GP9 Entrez,  Source | glycoprotein IX (platelet) | 11791 | -0.052 | -0.3148 | No |
| 36 | GYPA | GYPA Entrez,  Source | glycophorin A (MNS blood group) | 12610 | -0.062 | -0.3503 | No |
| 37 | FCER2 | FCER2 Entrez,  Source | Fc fragment of IgE, low affinity II, receptor for (CD23) | 12654 | -0.062 | -0.3460 | No |
| 38 | CD4 | CD4 Entrez,  Source | CD4 molecule | 12783 | -0.064 | -0.3459 | No |
| 39 | EPO | EPO Entrez,  Source | erythropoietin | 12821 | -0.064 | -0.3411 | No |
| 40 | CD8A | CD8A Entrez,  Source | CD8a molecule | 12944 | -0.066 | -0.3404 | No |
| 41 | MME | MME Entrez,  Source | membrane metallo-endopeptidase (neutral endopeptidase, enkephalinase) | 13115 | -0.068 | -0.3420 | No |
| 42 | IL3 | IL3 Entrez,  Source | interleukin 3 (colony-stimulating factor, multiple) | 13294 | -0.071 | -0.3438 | No |
| 43 | CD19 | CD19 Entrez,  Source | CD19 molecule | 13413 | -0.072 | -0.3423 | No |
| 44 | CD44 | CD44 Entrez,  Source | CD44 molecule (Indian blood group) | 13533 | -0.074 | -0.3407 | No |
| 45 | FCGR1A | FCGR1A Entrez,  Source | Fc fragment of IgG, high affinity Ia, receptor (CD64) | 13902 | -0.078 | -0.3514 | Yes |
| 46 | CD2 | CD2 Entrez,  Source | CD2 molecule | 13913 | -0.078 | -0.3437 | Yes |
| 47 | CSF3R | CSF3R Entrez,  Source | colony stimulating factor 3 receptor (granulocyte) | 13993 | -0.080 | -0.3394 | Yes |
| 48 | IL1R1 | IL1R1 Entrez,  Source | interleukin 1 receptor, type I | 14058 | -0.080 | -0.3343 | Yes |
| 49 | FLT3 | FLT3 Entrez,  Source | fms-related tyrosine kinase 3 | 14171 | -0.082 | -0.3314 | Yes |
| 50 | EPOR | EPOR Entrez,  Source | erythropoietin receptor | 14204 | -0.082 | -0.3244 | Yes |
| 51 | CD38 | CD38 Entrez,  Source | CD38 molecule | 14469 | -0.086 | -0.3290 | Yes |
| 52 | MS4A1 | MS4A1 Entrez,  Source | membrane-spanning 4-domains, subfamily A, member 1 | 14667 | -0.089 | -0.3298 | Yes |
| 53 | ITGA2B | ITGA2B Entrez,  Source | integrin, alpha 2b (platelet glycoprotein IIb of IIb/IIIa complex, antigen CD41) | 15161 | -0.097 | -0.3450 | Yes |
| 54 | CD3E | CD3E Entrez,  Source | CD3e molecule, epsilon (CD3-TCR complex) | 15223 | -0.098 | -0.3379 | Yes |
| 55 | CSF2RA | CSF2RA Entrez,  Source | colony stimulating factor 2 receptor, alpha, low-affinity (granulocyte-macrophage) | 15267 | -0.098 | -0.3298 | Yes |
| 56 | IL5RA | IL5RA Entrez,  Source | interleukin 5 receptor, alpha | 15488 | -0.102 | -0.3305 | Yes |
| 57 | ANPEP | ANPEP Entrez,  Source | alanyl (membrane) aminopeptidase (aminopeptidase N, aminopeptidase M, microsomal aminopeptidase, CD13, p150) | 15504 | -0.102 | -0.3205 | Yes |
| 58 | IL1B | IL1B Entrez,  Source | interleukin 1, beta | 15552 | -0.103 | -0.3122 | Yes |
| 59 | CD7 | CD7 Entrez,  Source | CD7 molecule | 15682 | -0.105 | -0.3078 | Yes |
| 60 | CD5 | CD5 Entrez,  Source | CD5 molecule | 15901 | -0.109 | -0.3075 | Yes |
| 61 | HLA-DRB1 | HLA-DRB1 Entrez,  Source | major histocompatibility complex, class II, DR beta 1 | 16236 | -0.116 | -0.3125 | Yes |
| 62 | CD34 | CD34 Entrez,  Source | CD34 molecule | 16402 | -0.119 | -0.3085 | Yes |
| 63 | FLT3LG | FLT3LG Entrez,  Source | fms-related tyrosine kinase 3 ligand | 16636 | -0.124 | -0.3075 | Yes |
| 64 | IL5 | IL5 Entrez,  Source | interleukin 5 (colony-stimulating factor, eosinophil) | 16732 | -0.126 | -0.2992 | Yes |
| 65 | IL11RA | IL11RA Entrez,  Source | interleukin 11 receptor, alpha | 16989 | -0.132 | -0.2984 | Yes |
| 66 | CD1D | CD1D Entrez,  Source | CD1d molecule | 17143 | -0.137 | -0.2920 | Yes |
| 67 | TNF | TNF Entrez,  Source | tumor necrosis factor (TNF superfamily, member 2) | 17349 | -0.143 | -0.2875 | Yes |
| 68 | CSF3 | CSF3 Entrez,  Source | colony stimulating factor 3 (granulocyte) | 17402 | -0.145 | -0.2750 | Yes |
| 69 | CD1B | CD1B Entrez,  Source | CD1b molecule | 17403 | -0.145 | -0.2598 | Yes |
| 70 | IL2RA | IL2RA Entrez,  Source | interleukin 2 receptor, alpha | 17457 | -0.147 | -0.2471 | Yes |
| 71 | CD36 | CD36 Entrez,  Source | CD36 molecule (thrombospondin receptor) | 17746 | -0.156 | -0.2455 | Yes |
| 72 | CD1C | CD1C Entrez,  Source | CD1c molecule | 18292 | -0.181 | -0.2546 | Yes |
| 73 | IL4 | IL4 Entrez,  Source | interleukin 4 | 18404 | -0.187 | -0.2407 | Yes |
| 74 | CD37 | CD37 Entrez,  Source | CD37 molecule | 18520 | -0.195 | -0.2262 | Yes |
| 75 | IL1R2 | IL1R2 Entrez,  Source | interleukin 1 receptor, type II | 18544 | -0.197 | -0.2068 | Yes |
| 76 | CD55 | CD55 Entrez,  Source | CD55 molecule, decay accelerating factor for complement (Cromer blood group) | 18578 | -0.198 | -0.1877 | Yes |
| 77 | CD1A | CD1A Entrez,  Source | CD1a molecule | 18864 | -0.225 | -0.1788 | Yes |
| 78 | KITLG | KITLG Entrez,  Source | KIT ligand | 19098 | -0.259 | -0.1636 | Yes |
| 79 | CD33 | CD33 Entrez,  Source | CD33 molecule | 19363 | -0.371 | -0.1383 | Yes |
| 80 | CR2 | CR2 Entrez,  Source | complement component (3d/Epstein Barr virus) receptor 2 | 19368 | -0.374 | -0.0993 | Yes |
| 81 | IL6R | IL6R Entrez,  Source | interleukin 6 receptor | 19442 | -0.446 | -0.0562 | Yes |
| 82 | CD8B | CD8B Entrez,  Source | CD8b molecule | 19510 | -0.592 | 0.0024 | Yes |
Table: GSEA details [plain text format]

  

Fig 2: KEGG\_HEMATOPOIETIC\_CELL\_LINEAGE      
 Blue-Pink O' Gram in the Space of the Analyzed GeneSet

  

Fig 3: KEGG\_HEMATOPOIETIC\_CELL\_LINEAGE: Random ES distribution      
 Gene set null distribution of ES for **KEGG\_HEMATOPOIETIC\_CELL\_LINEAGE**

  
