# Supplementary material for: Comprehensive analysis of β-catenin target genes in colorectal carcinoma cell lines with deregulated Wnt/β-catenin signaling
Source: BMC Genomics. 2014 Jan 28;15:74. doi: 10.1186/1471-2164-15-74 (PMC3909937; doi:10.1186/1471-2164-15-74)
Supplement: Additional file 5 — GSEA analysis using the KEGG pathway database. This zipped file contains confirming data of the GSEA analysis. The names of the directories containing the files were composed of the term ‘GSEA’, the name of the cell line, e.g. DLD1, SW480, or LS174T, and the pathway database (KEGG). Please use a web browser to view the files with the name ‘index.html’ in the corresponding directories to start exploring the data. [file 1471-2164-15-74-S5.zip › GSEA KEGG SW480/KEGG_LYSOSOME.html]

Details for gene set KEGG\_LYSOSOME[GSEA]

|  || Dataset | SW480\_collapsed\_to\_symbols.class.cls#b\_versus\_bg.class.cls#b\_versus\_bg\_repos |
| Phenotype | class.cls#b\_versus\_bg\_repos |
| Upregulated in class | 1 |
| GeneSet | KEGG\_LYSOSOME |
| Enrichment Score (ES) | 0.40681204 |
| Normalized Enrichment Score (NES) | 1.662648 |
| Nominal p-value | 0.0 |
| FDR q-value | 0.06329011 |
| FWER p-Value | 0.488 |
Table: GSEA Results Summary

  

Fig 1: Enrichment plot: KEGG\_LYSOSOME      
 Profile of the Running ES Score & Positions of GeneSet Members on the Rank Ordered List

  

| PROBE | GENE SYMBOL | GENE\_TITLE | RANK IN GENE LIST | RANK METRIC SCORE | RUNNING ES | CORE ENRICHMENT || 1 | LAPTM5 | LAPTM5 Entrez,  Source | lysosomal associated multispanning membrane protein 5 | 62 | 0.609 | 0.0550 | Yes |
| 2 | GNPTAB | GNPTAB Entrez,  Source | N-acetylglucosamine-1-phosphate transferase, alpha and beta subunits | 175 | 0.414 | 0.0888 | Yes |
| 3 | AP1S2 | AP1S2 Entrez,  Source | adaptor-related protein complex 1, sigma 2 subunit | 241 | 0.373 | 0.1211 | Yes |
| 4 | ABCB9 | ABCB9 Entrez,  Source | ATP-binding cassette, sub-family B (MDR/TAP), member 9 | 297 | 0.342 | 0.1510 | Yes |
| 5 | ARSG | ARSG Entrez,  Source | arylsulfatase G | 378 | 0.312 | 0.1767 | Yes |
| 6 | AP1S3 | AP1S3 Entrez,  Source | adaptor-related protein complex 1, sigma 3 subunit | 458 | 0.288 | 0.2001 | Yes |
| 7 | AP3B2 | AP3B2 Entrez,  Source | adaptor-related protein complex 3, beta 2 subunit | 541 | 0.262 | 0.2209 | Yes |
| 8 | NAGA | NAGA Entrez,  Source | N-acetylgalactosaminidase, alpha- | 718 | 0.230 | 0.2339 | Yes |
| 9 | AGA | AGA Entrez,  Source | aspartylglucosaminidase | 766 | 0.223 | 0.2528 | Yes |
| 10 | TPP1 | TPP1 Entrez,  Source | tripeptidyl peptidase I | 771 | 0.222 | 0.2738 | Yes |
| 11 | SMPD1 | SMPD1 Entrez,  Source | sphingomyelin phosphodiesterase 1, acid lysosomal (acid sphingomyelinase) | 966 | 0.193 | 0.2823 | Yes |
| 12 | LAMP3 | LAMP3 Entrez,  Source | lysosomal-associated membrane protein 3 | 1179 | 0.172 | 0.2878 | Yes |
| 13 | ARSA | ARSA Entrez,  Source | arylsulfatase A | 1401 | 0.155 | 0.2912 | Yes |
| 14 | TCIRG1 | TCIRG1 Entrez,  Source | T-cell, immune regulator 1, ATPase, H+ transporting, lysosomal V0 subunit A3 | 1474 | 0.149 | 0.3017 | Yes |
| 15 | SLC17A5 | SLC17A5 Entrez,  Source | solute carrier family 17 (anion/sugar transporter), member 5 | 1503 | 0.147 | 0.3144 | Yes |
| 16 | NPC2 | NPC2 Entrez,  Source | Niemann-Pick disease, type C2 | 1525 | 0.146 | 0.3272 | Yes |
| 17 | CTSS | CTSS Entrez,  Source | cathepsin S | 1546 | 0.144 | 0.3400 | Yes |
| 18 | HEXB | HEXB Entrez,  Source | hexosaminidase B (beta polypeptide) | 1884 | 0.126 | 0.3347 | Yes |
| 19 | AP4M1 | AP4M1 Entrez,  Source | adaptor-related protein complex 4, mu 1 subunit | 1889 | 0.126 | 0.3465 | Yes |
| 20 | GUSB | GUSB Entrez,  Source | glucuronidase, beta | 1994 | 0.121 | 0.3527 | Yes |
| 21 | SUMF1 | SUMF1 Entrez,  Source | sulfatase modifying factor 1 | 2008 | 0.120 | 0.3636 | Yes |
| 22 | HEXA | HEXA Entrez,  Source | hexosaminidase A (alpha polypeptide) | 2127 | 0.115 | 0.3685 | Yes |
| 23 | ACP2 | ACP2 Entrez,  Source | acid phosphatase 2, lysosomal | 2170 | 0.113 | 0.3771 | Yes |
| 24 | CTSD | CTSD Entrez,  Source | cathepsin D (lysosomal aspartyl peptidase) | 2271 | 0.108 | 0.3823 | Yes |
| 25 | NEU1 | NEU1 Entrez,  Source | sialidase 1 (lysosomal sialidase) | 2475 | 0.101 | 0.3815 | Yes |
| 26 | CLN5 | CLN5 Entrez,  Source | ceroid-lipofuscinosis, neuronal 5 | 2521 | 0.099 | 0.3887 | Yes |
| 27 | GM2A | GM2A Entrez,  Source | GM2 ganglioside activator | 2702 | 0.092 | 0.3882 | Yes |
| 28 | GNS | GNS Entrez,  Source | glucosamine (N-acetyl)-6-sulfatase (Sanfilippo disease IIID) | 2714 | 0.092 | 0.3964 | Yes |
| 29 | ATP6V1H | ATP6V1H Entrez,  Source | ATPase, H+ transporting, lysosomal 50/57kDa, V1 subunit H | 3012 | 0.082 | 0.3890 | Yes |
| 30 | SLC11A2 | SLC11A2 Entrez,  Source | solute carrier family 11 (proton-coupled divalent metal ion transporters), member 2 | 3135 | 0.078 | 0.3902 | Yes |
| 31 | CLTB | CLTB Entrez,  Source | clathrin, light chain (Lcb) | 3213 | 0.076 | 0.3934 | Yes |
| 32 | GNPTG | GNPTG Entrez,  Source | N-acetylglucosamine-1-phosphate transferase, gamma subunit | 3426 | 0.070 | 0.3893 | Yes |
| 33 | AP1M1 | AP1M1 Entrez,  Source | adaptor-related protein complex 1, mu 1 subunit | 3489 | 0.068 | 0.3926 | Yes |
| 34 | CD63 | CD63 Entrez,  Source | CD63 molecule | 3499 | 0.068 | 0.3986 | Yes |
| 35 | CTSH | CTSH Entrez,  Source | cathepsin H | 3512 | 0.068 | 0.4044 | Yes |
| 36 | AP1G1 | AP1G1 Entrez,  Source | adaptor-related protein complex 1, gamma 1 subunit | 3669 | 0.063 | 0.4024 | Yes |
| 37 | ATP6V0D1 | ATP6V0D1 Entrez,  Source | ATPase, H+ transporting, lysosomal 38kDa, V0 subunit d1 | 3784 | 0.061 | 0.4024 | Yes |
| 38 | ACP5 | ACP5 Entrez,  Source | acid phosphatase 5, tartrate resistant | 3810 | 0.060 | 0.4068 | Yes |
| 39 | ASAH1 | ASAH1 Entrez,  Source | N-acylsphingosine amidohydrolase (acid ceramidase) 1 | 4142 | 0.052 | 0.3948 | No |
| 40 | CLN3 | CLN3 Entrez,  Source | ceroid-lipofuscinosis, neuronal 3, juvenile (Batten, Spielmeyer-Vogt disease) | 4195 | 0.051 | 0.3970 | No |
| 41 | CLTA | CLTA Entrez,  Source | clathrin, light chain (Lca) | 4214 | 0.051 | 0.4009 | No |
| 42 | M6PR | M6PR Entrez,  Source | mannose-6-phosphate receptor (cation dependent) | 4305 | 0.049 | 0.4010 | No |
| 43 | PPT1 | PPT1 Entrez,  Source | palmitoyl-protein thioesterase 1 (ceroid-lipofuscinosis, neuronal 1, infantile) | 4463 | 0.046 | 0.3973 | No |
| 44 | IDS | IDS Entrez,  Source | iduronate 2-sulfatase (Hunter syndrome) | 4478 | 0.045 | 0.4009 | No |
| 45 | MAN2B1 | MAN2B1 Entrez,  Source | mannosidase, alpha, class 2B, member 1 | 4580 | 0.043 | 0.3999 | No |
| 46 | AP3S1 | AP3S1 Entrez,  Source | adaptor-related protein complex 3, sigma 1 subunit | 4692 | 0.041 | 0.3981 | No |
| 47 | HGSNAT | HGSNAT Entrez,  Source | heparan-alpha-glucosaminide N-acetyltransferase | 4847 | 0.038 | 0.3938 | No |
| 48 | CTSB | CTSB Entrez,  Source | cathepsin B | 5357 | 0.029 | 0.3704 | No |
| 49 | ARSB | ARSB Entrez,  Source | arylsulfatase B | 5692 | 0.024 | 0.3556 | No |
| 50 | AP4E1 | AP4E1 Entrez,  Source | adaptor-related protein complex 4, epsilon 1 subunit | 5889 | 0.021 | 0.3475 | No |
| 51 | LAMP1 | LAMP1 Entrez,  Source | lysosomal-associated membrane protein 1 | 5926 | 0.021 | 0.3476 | No |
| 52 | CD164 | CD164 Entrez,  Source | CD164 molecule, sialomucin | 6065 | 0.019 | 0.3423 | No |
| 53 | CTNS | CTNS Entrez,  Source | cystinosis, nephropathic | 6410 | 0.014 | 0.3259 | No |
| 54 | CLTC | CLTC Entrez,  Source | clathrin, heavy chain (Hc) | 6436 | 0.013 | 0.3259 | No |
| 55 | GGA3 | GGA3 Entrez,  Source | golgi associated, gamma adaptin ear containing, ARF binding protein 3 | 6537 | 0.012 | 0.3219 | No |
| 56 | LAPTM4A | LAPTM4A Entrez,  Source | lysosomal-associated protein transmembrane 4 alpha | 6653 | 0.010 | 0.3169 | No |
| 57 | NAGLU | NAGLU Entrez,  Source | N-acetylglucosaminidase, alpha- (Sanfilippo disease IIIB) | 6677 | 0.010 | 0.3167 | No |
| 58 | GALC | GALC Entrez,  Source | galactosylceramidase | 6693 | 0.010 | 0.3169 | No |
| 59 | ENTPD4 | ENTPD4 Entrez,  Source | ectonucleoside triphosphate diphosphohydrolase 4 | 6738 | 0.009 | 0.3155 | No |
| 60 | AP4S1 | AP4S1 Entrez,  Source | adaptor-related protein complex 4, sigma 1 subunit | 6741 | 0.009 | 0.3163 | No |
| 61 | AP3D1 | AP3D1 Entrez,  Source | adaptor-related protein complex 3, delta 1 subunit | 6881 | 0.007 | 0.3099 | No |
| 62 | AP3B1 | AP3B1 Entrez,  Source | adaptor-related protein complex 3, beta 1 subunit | 6926 | 0.007 | 0.3083 | No |
| 63 | ATP6AP1 | ATP6AP1 Entrez,  Source | ATPase, H+ transporting, lysosomal accessory protein 1 | 7717 | -0.003 | 0.2680 | No |
| 64 | ABCA2 | ABCA2 Entrez,  Source | ATP-binding cassette, sub-family A (ABC1), member 2 | 7753 | -0.004 | 0.2666 | No |
| 65 | SORT1 | SORT1 Entrez,  Source | sortilin 1 | 7763 | -0.004 | 0.2665 | No |
| 66 | GGA2 | GGA2 Entrez,  Source | golgi associated, gamma adaptin ear containing, ARF binding protein 2 | 7821 | -0.005 | 0.2640 | No |
| 67 | GLA | GLA Entrez,  Source | galactosidase, alpha | 7822 | -0.005 | 0.2645 | No |
| 68 | MCOLN1 | MCOLN1 Entrez,  Source | mucolipin 1 | 8088 | -0.008 | 0.2516 | No |
| 69 | PSAP | PSAP Entrez,  Source | prosaposin (variant Gaucher disease and variant metachromatic leukodystrophy) | 8324 | -0.011 | 0.2405 | No |
| 70 | ATP6V0A4 | ATP6V0A4 Entrez,  Source | ATPase, H+ transporting, lysosomal V0 subunit a4 | 8494 | -0.013 | 0.2330 | No |
| 71 | CTSL2 | CTSL2 Entrez,  Source | cathepsin L2 | 8668 | -0.015 | 0.2255 | No |
| 72 | AP3M1 | AP3M1 Entrez,  Source | adaptor-related protein complex 3, mu 1 subunit | 8746 | -0.016 | 0.2231 | No |
| 73 | ATP6V0C | ATP6V0C Entrez,  Source | ATPase, H+ transporting, lysosomal 16kDa, V0 subunit c | 8747 | -0.016 | 0.2245 | No |
| 74 | ATP6V0A1 | ATP6V0A1 Entrez,  Source | ATPase, H+ transporting, lysosomal V0 subunit a1 | 8859 | -0.017 | 0.2205 | No |
| 75 | MANBA | MANBA Entrez,  Source | mannosidase, beta A, lysosomal | 9055 | -0.019 | 0.2123 | No |
| 76 | NPC1 | NPC1 Entrez,  Source | Niemann-Pick disease, type C1 | 9329 | -0.022 | 0.2004 | No |
| 77 | DNASE2 | DNASE2 Entrez,  Source | deoxyribonuclease II, lysosomal | 9397 | -0.023 | 0.1991 | No |
| 78 | AP1B1 | AP1B1 Entrez,  Source | adaptor-related protein complex 1, beta 1 subunit | 9502 | -0.024 | 0.1961 | No |
| 79 | IGF2R | IGF2R Entrez,  Source | insulin-like growth factor 2 receptor | 9612 | -0.026 | 0.1929 | No |
| 80 | GLB1 | GLB1 Entrez,  Source | galactosidase, beta 1 | 9626 | -0.026 | 0.1947 | No |
| 81 | CTSE | CTSE Entrez,  Source | cathepsin E | 10069 | -0.031 | 0.1749 | No |
| 82 | LAMP2 | LAMP2 Entrez,  Source | lysosomal-associated membrane protein 2 | 10087 | -0.031 | 0.1770 | No |
| 83 | CTSC | CTSC Entrez,  Source | cathepsin C | 10585 | -0.037 | 0.1550 | No |
| 84 | FUCA1 | FUCA1 Entrez,  Source | fucosidase, alpha-L- 1, tissue | 10688 | -0.038 | 0.1534 | No |
| 85 | CLTCL1 | CLTCL1 Entrez,  Source | clathrin, heavy chain-like 1 | 11208 | -0.045 | 0.1309 | No |
| 86 | CTSK | CTSK Entrez,  Source | cathepsin K (pycnodysostosis) | 11395 | -0.047 | 0.1259 | No |
| 87 | AP3M2 | AP3M2 Entrez,  Source | adaptor-related protein complex 3, mu 2 subunit | 11707 | -0.051 | 0.1147 | No |
| 88 | AP1S1 | AP1S1 Entrez,  Source | adaptor-related protein complex 1, sigma 1 subunit | 12138 | -0.056 | 0.0979 | No |
| 89 | GALNS | GALNS Entrez,  Source | galactosamine (N-acetyl)-6-sulfate sulfatase (Morquio syndrome, mucopolysaccharidosis type IVA) | 12207 | -0.057 | 0.0999 | No |
| 90 | LIPA | LIPA Entrez,  Source | lipase A, lysosomal acid, cholesterol esterase (Wolman disease) | 12349 | -0.058 | 0.0982 | No |
| 91 | AP4B1 | AP4B1 Entrez,  Source | adaptor-related protein complex 4, beta 1 subunit | 12451 | -0.060 | 0.0987 | No |
| 92 | LAPTM4B | LAPTM4B Entrez,  Source | lysosomal associated protein transmembrane 4 beta | 12695 | -0.063 | 0.0922 | No |
| 93 | ATP6V0D2 | ATP6V0D2 Entrez,  Source | ATPase, H+ transporting, lysosomal 38kDa, V0 subunit d2 | 12824 | -0.064 | 0.0918 | No |
| 94 | SGSH | SGSH Entrez,  Source | N-sulfoglucosamine sulfohydrolase (sulfamidase) | 12975 | -0.066 | 0.0904 | No |
| 95 | AP3S2 | AP3S2 Entrez,  Source | adaptor-related protein complex 3, sigma 2 subunit | 13152 | -0.069 | 0.0879 | No |
| 96 | IDUA | IDUA Entrez,  Source | iduronidase, alpha-L- | 14109 | -0.081 | 0.0465 | No |
| 97 | SLC11A1 | SLC11A1 Entrez,  Source | solute carrier family 11 (proton-coupled divalent metal ion transporters), member 1 | 14250 | -0.083 | 0.0472 | No |
| 98 | NAGPA | NAGPA Entrez,  Source | N-acetylglucosamine-1-phosphodiester alpha-N-acetylglucosaminidase | 14338 | -0.084 | 0.0508 | No |
| 99 | GGA1 | GGA1 Entrez,  Source | golgi associated, gamma adaptin ear containing, ARF binding protein 1 | 14421 | -0.085 | 0.0547 | No |
| 100 | CTSW | CTSW Entrez,  Source | cathepsin W (lymphopain) | 14477 | -0.086 | 0.0600 | No |
| 101 | CTSO | CTSO Entrez,  Source | cathepsin O | 14515 | -0.086 | 0.0664 | No |
| 102 | DNASE2B | DNASE2B Entrez,  Source | deoxyribonuclease II beta | 14564 | -0.087 | 0.0723 | No |
| 103 | ATP6V0A2 | ATP6V0A2 Entrez,  Source | ATPase, H+ transporting, lysosomal V0 subunit a2 | 14810 | -0.091 | 0.0683 | No |
| 104 | GAA | GAA Entrez,  Source | glucosidase, alpha; acid (Pompe disease, glycogen storage disease type II) | 16000 | -0.111 | 0.0178 | No |
| 105 | NAPSA | NAPSA Entrez,  Source | napsin A aspartic peptidase | 16066 | -0.112 | 0.0252 | No |
| 106 | ATP6V0B | ATP6V0B Entrez,  Source | ATPase, H+ transporting, lysosomal 21kDa, V0 subunit b | 16596 | -0.123 | 0.0097 | No |
| 107 | AP1M2 | AP1M2 Entrez,  Source | adaptor-related protein complex 1, mu 2 subunit | 16786 | -0.127 | 0.0122 | No |
| 108 | SCARB2 | SCARB2 Entrez,  Source | scavenger receptor class B, member 2 | 16856 | -0.129 | 0.0210 | No |
| 109 | CD68 | CD68 Entrez,  Source | CD68 molecule | 17149 | -0.137 | 0.0190 | No |
| 110 | CTSG | CTSG Entrez,  Source | cathepsin G | 17229 | -0.140 | 0.0283 | No |
| 111 | LGMN | LGMN Entrez,  Source | legumain | 17745 | -0.156 | 0.0167 | No |
| 112 | CTSF | CTSF Entrez,  Source | cathepsin F | 18001 | -0.167 | 0.0195 | No |
| 113 | CTSZ | CTSZ Entrez,  Source | cathepsin Z | 18966 | -0.238 | -0.0073 | No |
| 114 | HYAL1 | HYAL1 Entrez,  Source | hyaluronoglucosaminidase 1 | 19391 | -0.393 | 0.0085 | No |
Table: GSEA details [plain text format]

  

Fig 2: KEGG\_LYSOSOME      
 Blue-Pink O' Gram in the Space of the Analyzed GeneSet

  

Fig 3: KEGG\_LYSOSOME: Random ES distribution      
 Gene set null distribution of ES for **KEGG\_LYSOSOME**

  
