# Supplementary material for: Comprehensive analysis of β-catenin target genes in colorectal carcinoma cell lines with deregulated Wnt/β-catenin signaling
Source: BMC Genomics. 2014 Jan 28;15:74. doi: 10.1186/1471-2164-15-74 (PMC3909937; doi:10.1186/1471-2164-15-74)
Supplement: Additional file 5 — GSEA analysis using the KEGG pathway database. This zipped file contains confirming data of the GSEA analysis. The names of the directories containing the files were composed of the term ‘GSEA’, the name of the cell line, e.g. DLD1, SW480, or LS174T, and the pathway database (KEGG). Please use a web browser to view the files with the name ‘index.html’ in the corresponding directories to start exploring the data. [file 1471-2164-15-74-S5.zip › GSEA KEGG SW480/KEGG_MISMATCH_REPAIR.html]

Details for gene set KEGG\_MISMATCH\_REPAIR[GSEA]

|  || Dataset | SW480\_collapsed\_to\_symbols.class.cls#b\_versus\_bg.class.cls#b\_versus\_bg\_repos |
| Phenotype | class.cls#b\_versus\_bg\_repos |
| Upregulated in class | 1 |
| GeneSet | KEGG\_MISMATCH\_REPAIR |
| Enrichment Score (ES) | 0.56993335 |
| Normalized Enrichment Score (NES) | 1.6982914 |
| Nominal p-value | 0.023316063 |
| FDR q-value | 0.06318805 |
| FWER p-Value | 0.386 |
Table: GSEA Results Summary

  

Fig 1: Enrichment plot: KEGG\_MISMATCH\_REPAIR      
 Profile of the Running ES Score & Positions of GeneSet Members on the Rank Ordered List

  

| PROBE | GENE SYMBOL | GENE\_TITLE | RANK IN GENE LIST | RANK METRIC SCORE | RUNNING ES | CORE ENRICHMENT || 1 | POLD4 | POLD4 Entrez,  Source | polymerase (DNA-directed), delta 4 | 621 | 0.247 | 0.1369 | Yes |
| 2 | RPA1 | RPA1 Entrez,  Source | replication protein A1, 70kDa | 1399 | 0.155 | 0.2030 | Yes |
| 3 | MSH2 | MSH2 Entrez,  Source | mutS homolog 2, colon cancer, nonpolyposis type 1 (E. coli) | 1685 | 0.135 | 0.2804 | Yes |
| 4 | MLH1 | MLH1 Entrez,  Source | mutL homolog 1, colon cancer, nonpolyposis type 2 (E. coli) | 1938 | 0.124 | 0.3522 | Yes |
| 5 | LIG1 | LIG1 Entrez,  Source | ligase I, DNA, ATP-dependent | 2306 | 0.107 | 0.4068 | Yes |
| 6 | MSH3 | MSH3 Entrez,  Source | mutS homolog 3 (E. coli) | 2328 | 0.107 | 0.4785 | Yes |
| 7 | RFC2 | RFC2 Entrez,  Source | replication factor C (activator 1) 2, 40kDa | 2739 | 0.091 | 0.5194 | Yes |
| 8 | RPA3 | RPA3 Entrez,  Source | replication protein A3, 14kDa | 3777 | 0.061 | 0.5078 | Yes |
| 9 | RFC5 | RFC5 Entrez,  Source | replication factor C (activator 1) 5, 36.5kDa | 3791 | 0.060 | 0.5485 | Yes |
| 10 | POLD3 | POLD3 Entrez,  Source | polymerase (DNA-directed), delta 3, accessory subunit | 4089 | 0.054 | 0.5699 | Yes |
| 11 | PCNA | PCNA Entrez,  Source | proliferating cell nuclear antigen | 4937 | 0.036 | 0.5514 | No |
| 12 | RFC4 | RFC4 Entrez,  Source | replication factor C (activator 1) 4, 37kDa | 5300 | 0.030 | 0.5535 | No |
| 13 | RFC1 | RFC1 Entrez,  Source | replication factor C (activator 1) 1, 145kDa | 5403 | 0.029 | 0.5678 | No |
| 14 | MLH3 | MLH3 Entrez,  Source | mutL homolog 3 (E. coli) | 7047 | 0.005 | 0.4872 | No |
| 15 | POLD2 | POLD2 Entrez,  Source | polymerase (DNA directed), delta 2, regulatory subunit 50kDa | 7071 | 0.005 | 0.4891 | No |
| 16 | RFC3 | RFC3 Entrez,  Source | replication factor C (activator 1) 3, 38kDa | 7608 | -0.002 | 0.4630 | No |
| 17 | MSH6 | MSH6 Entrez,  Source | mutS homolog 6 (E. coli) | 7757 | -0.004 | 0.4582 | No |
| 18 | RPA2 | RPA2 Entrez,  Source | replication protein A2, 32kDa | 7783 | -0.004 | 0.4599 | No |
| 19 | SSBP1 | SSBP1 Entrez,  Source | single-stranded DNA binding protein 1 | 8689 | -0.015 | 0.4237 | No |
| 20 | EXO1 | EXO1 Entrez,  Source | exonuclease 1 | 9669 | -0.026 | 0.3915 | No |
| 21 | POLD1 | POLD1 Entrez,  Source | polymerase (DNA directed), delta 1, catalytic subunit 125kDa | 9782 | -0.028 | 0.4046 | No |
| 22 | RPA4 | RPA4 Entrez,  Source | replication protein A4, 34kDa | 17245 | -0.140 | 0.1183 | No |
Table: GSEA details [plain text format]

  

Fig 2: KEGG\_MISMATCH\_REPAIR      
 Blue-Pink O' Gram in the Space of the Analyzed GeneSet

  

Fig 3: KEGG\_MISMATCH\_REPAIR: Random ES distribution      
 Gene set null distribution of ES for **KEGG\_MISMATCH\_REPAIR**

  
