# Supplementary material for: Comprehensive analysis of β-catenin target genes in colorectal carcinoma cell lines with deregulated Wnt/β-catenin signaling
Source: BMC Genomics. 2014 Jan 28;15:74. doi: 10.1186/1471-2164-15-74 (PMC3909937; doi:10.1186/1471-2164-15-74)
Supplement: Additional file 5 — GSEA analysis using the KEGG pathway database. This zipped file contains confirming data of the GSEA analysis. The names of the directories containing the files were composed of the term ‘GSEA’, the name of the cell line, e.g. DLD1, SW480, or LS174T, and the pathway database (KEGG). Please use a web browser to view the files with the name ‘index.html’ in the corresponding directories to start exploring the data. [file 1471-2164-15-74-S5.zip › GSEA KEGG SW480/KEGG_NEUROACTIVE_LIGAND_RECEPTOR_INTERACTION.html]

Details for gene set KEGG\_NEUROACTIVE\_LIGAND\_RECEPTOR\_INTERACTION[GSEA]

|  || Dataset | SW480\_collapsed\_to\_symbols.class.cls#b\_versus\_bg.class.cls#b\_versus\_bg\_repos |
| Phenotype | class.cls#b\_versus\_bg\_repos |
| Upregulated in class | 0 |
| GeneSet | KEGG\_NEUROACTIVE\_LIGAND\_RECEPTOR\_INTERACTION |
| Enrichment Score (ES) | -0.39003026 |
| Normalized Enrichment Score (NES) | -1.7245445 |
| Nominal p-value | 0.0 |
| FDR q-value | 0.111293614 |
| FWER p-Value | 0.261 |
Table: GSEA Results Summary

  

Fig 1: Enrichment plot: KEGG\_NEUROACTIVE\_LIGAND\_RECEPTOR\_INTERACTION      
 Profile of the Running ES Score & Positions of GeneSet Members on the Rank Ordered List

  

| PROBE | GENE SYMBOL | GENE\_TITLE | RANK IN GENE LIST | RANK METRIC SCORE | RUNNING ES | CORE ENRICHMENT || 1 | ADRB2 | ADRB2 Entrez,  Source | adrenergic, beta-2-, receptor, surface | 42 | 0.694 | 0.0257 | No |
| 2 | LEPR | LEPR Entrez,  Source | leptin receptor | 72 | 0.587 | 0.0479 | No |
| 3 | ADORA2B | ADORA2B Entrez,  Source | adenosine A2b receptor | 76 | 0.584 | 0.0712 | No |
| 4 | OXTR | OXTR Entrez,  Source | oxytocin receptor | 176 | 0.414 | 0.0827 | No |
| 5 | NR3C1 | NR3C1 Entrez,  Source | nuclear receptor subfamily 3, group C, member 1 (glucocorticoid receptor) | 204 | 0.398 | 0.0974 | No |
| 6 | P2RY2 | P2RY2 Entrez,  Source | purinergic receptor P2Y, G-protein coupled, 2 | 626 | 0.246 | 0.0855 | No |
| 7 | GABRE | GABRE Entrez,  Source | gamma-aminobutyric acid (GABA) A receptor, epsilon | 925 | 0.200 | 0.0781 | No |
| 8 | HRH1 | HRH1 Entrez,  Source | histamine receptor H1 | 930 | 0.200 | 0.0859 | No |
| 9 | F2RL1 | F2RL1 Entrez,  Source | coagulation factor II (thrombin) receptor-like 1 | 1527 | 0.146 | 0.0609 | No |
| 10 | P2RX4 | P2RX4 Entrez,  Source | purinergic receptor P2X, ligand-gated ion channel, 4 | 1684 | 0.135 | 0.0583 | No |
| 11 | P2RY1 | P2RY1 Entrez,  Source | purinergic receptor P2Y, G-protein coupled, 1 | 3097 | 0.079 | -0.0117 | No |
| 12 | ADRA2B | ADRA2B Entrez,  Source | adrenergic, alpha-2B-, receptor | 3266 | 0.074 | -0.0174 | No |
| 13 | HRH4 | HRH4 Entrez,  Source | histamine receptor H4 | 3945 | 0.057 | -0.0502 | No |
| 14 | NMBR | NMBR Entrez,  Source | neuromedin B receptor | 4086 | 0.054 | -0.0553 | No |
| 15 | GRIN2C | GRIN2C Entrez,  Source | glutamate receptor, ionotropic, N-methyl D-aspartate 2C | 4098 | 0.053 | -0.0537 | No |
| 16 | MLNR | MLNR Entrez,  Source | motilin receptor | 4110 | 0.053 | -0.0521 | No |
| 17 | NTSR1 | NTSR1 Entrez,  Source | neurotensin receptor 1 (high affinity) | 4351 | 0.048 | -0.0626 | No |
| 18 | GH2 | GH2 Entrez,  Source | growth hormone 2 | 4369 | 0.048 | -0.0616 | No |
| 19 | PTGFR | PTGFR Entrez,  Source | prostaglandin F receptor (FP) | 4696 | 0.041 | -0.0768 | No |
| 20 | P2RX7 | P2RX7 Entrez,  Source | purinergic receptor P2X, ligand-gated ion channel, 7 | 4718 | 0.041 | -0.0763 | No |
| 21 | CHRNA1 | CHRNA1 Entrez,  Source | cholinergic receptor, nicotinic, alpha 1 (muscle) | 5044 | 0.035 | -0.0917 | No |
| 22 | CNR1 | CNR1 Entrez,  Source | cannabinoid receptor 1 (brain) | 5155 | 0.033 | -0.0961 | No |
| 23 | TAAR2 | TAAR2 Entrez,  Source | trace amine associated receptor 2 | 5158 | 0.032 | -0.0949 | No |
| 24 | C3AR1 | C3AR1 Entrez,  Source | complement component 3a receptor 1 | 5277 | 0.031 | -0.0998 | No |
| 25 | BDKRB2 | BDKRB2 Entrez,  Source | bradykinin receptor B2 | 5382 | 0.029 | -0.1040 | No |
| 26 | MC3R | MC3R Entrez,  Source | melanocortin 3 receptor | 5384 | 0.029 | -0.1029 | No |
| 27 | CYSLTR2 | CYSLTR2 Entrez,  Source | cysteinyl leukotriene receptor 2 | 5672 | 0.024 | -0.1168 | No |
| 28 | NPFFR2 | NPFFR2 Entrez,  Source | neuropeptide FF receptor 2 | 5763 | 0.023 | -0.1205 | No |
| 29 | GRM2 | GRM2 Entrez,  Source | glutamate receptor, metabotropic 2 | 5899 | 0.021 | -0.1266 | No |
| 30 | GABRR1 | GABRR1 Entrez,  Source | gamma-aminobutyric acid (GABA) receptor, rho 1 | 5965 | 0.020 | -0.1292 | No |
| 31 | NPY1R | NPY1R Entrez,  Source | neuropeptide Y receptor Y1 | 6017 | 0.019 | -0.1311 | No |
| 32 | P2RX3 | P2RX3 Entrez,  Source | purinergic receptor P2X, ligand-gated ion channel, 3 | 6098 | 0.018 | -0.1345 | No |
| 33 | LHCGR | LHCGR Entrez,  Source | luteinizing hormone/choriogonadotropin receptor | 6139 | 0.018 | -0.1358 | No |
| 34 | NPY5R | NPY5R Entrez,  Source | neuropeptide Y receptor Y5 | 6159 | 0.017 | -0.1361 | No |
| 35 | CHRNB1 | CHRNB1 Entrez,  Source | cholinergic receptor, nicotinic, beta 1 (muscle) | 6279 | 0.015 | -0.1417 | No |
| 36 | GABBR2 | GABBR2 Entrez,  Source | gamma-aminobutyric acid (GABA) B receptor, 2 | 6365 | 0.014 | -0.1455 | No |
| 37 | GPR35 | GPR35 Entrez,  Source | G protein-coupled receptor 35 | 6370 | 0.014 | -0.1452 | No |
| 38 | CHRNG | CHRNG Entrez,  Source | cholinergic receptor, nicotinic, gamma | 6388 | 0.014 | -0.1455 | No |
| 39 | CALCR | CALCR Entrez,  Source | calcitonin receptor | 6571 | 0.011 | -0.1544 | No |
| 40 | SCTR | SCTR Entrez,  Source | secretin receptor | 6865 | 0.008 | -0.1693 | No |
| 41 | P2RY13 | P2RY13 Entrez,  Source | purinergic receptor P2Y, G-protein coupled, 13 | 6959 | 0.006 | -0.1739 | No |
| 42 | GRM1 | GRM1 Entrez,  Source | glutamate receptor, metabotropic 1 | 7229 | 0.003 | -0.1877 | No |
| 43 | NTSR2 | NTSR2 Entrez,  Source | neurotensin receptor 2 | 7306 | 0.002 | -0.1916 | No |
| 44 | PTGER3 | PTGER3 Entrez,  Source | prostaglandin E receptor 3 (subtype EP3) | 7484 | -0.000 | -0.2007 | No |
| 45 | GALR1 | GALR1 Entrez,  Source | galanin receptor 1 | 7487 | -0.001 | -0.2008 | No |
| 46 | HTR1E | HTR1E Entrez,  Source | 5-hydroxytryptamine (serotonin) receptor 1E | 7645 | -0.002 | -0.2088 | No |
| 47 | ADORA3 | ADORA3 Entrez,  Source | adenosine A3 receptor | 7687 | -0.003 | -0.2108 | No |
| 48 | GRIK1 | GRIK1 Entrez,  Source | glutamate receptor, ionotropic, kainate 1 | 7732 | -0.004 | -0.2130 | No |
| 49 | NMUR1 | NMUR1 Entrez,  Source | neuromedin U receptor 1 | 7786 | -0.004 | -0.2155 | No |
| 50 | GABRP | GABRP Entrez,  Source | gamma-aminobutyric acid (GABA) A receptor, pi | 8066 | -0.008 | -0.2297 | No |
| 51 | C5AR1 | C5AR1 Entrez,  Source | complement component 5a receptor 1 | 8121 | -0.008 | -0.2321 | No |
| 52 | GRIA2 | GRIA2 Entrez,  Source | glutamate receptor, ionotropic, AMPA 2 | 8156 | -0.009 | -0.2336 | No |
| 53 | GLRA2 | GLRA2 Entrez,  Source | glycine receptor, alpha 2 | 8204 | -0.009 | -0.2356 | No |
| 54 | GRIN3B | GRIN3B Entrez,  Source | glutamate receptor, ionotropic, N-methyl-D-aspartate 3B | 8249 | -0.010 | -0.2375 | No |
| 55 | GH1 | GH1 Entrez,  Source | growth hormone 1 | 8293 | -0.010 | -0.2393 | No |
| 56 | MCHR2 | MCHR2 Entrez,  Source | melanin-concentrating hormone receptor 2 | 8438 | -0.012 | -0.2463 | No |
| 57 | GABRG3 | GABRG3 Entrez,  Source | gamma-aminobutyric acid (GABA) A receptor, gamma 3 | 8504 | -0.013 | -0.2491 | No |
| 58 | HTR1A | HTR1A Entrez,  Source | 5-hydroxytryptamine (serotonin) receptor 1A | 8584 | -0.014 | -0.2527 | No |
| 59 | GRIN1 | GRIN1 Entrez,  Source | glutamate receptor, ionotropic, N-methyl D-aspartate 1 | 8614 | -0.014 | -0.2536 | No |
| 60 | CALCRL | CALCRL Entrez,  Source | calcitonin receptor-like | 8687 | -0.015 | -0.2568 | No |
| 61 | ADORA1 | ADORA1 Entrez,  Source | adenosine A1 receptor | 8919 | -0.018 | -0.2680 | No |
| 62 | GRM5 | GRM5 Entrez,  Source | glutamate receptor, metabotropic 5 | 8953 | -0.018 | -0.2690 | No |
| 63 | DRD4 | DRD4 Entrez,  Source | dopamine receptor D4 | 8967 | -0.018 | -0.2689 | No |
| 64 | GABRA6 | GABRA6 Entrez,  Source | gamma-aminobutyric acid (GABA) A receptor, alpha 6 | 9020 | -0.019 | -0.2709 | No |
| 65 | PTGER1 | PTGER1 Entrez,  Source | prostaglandin E receptor 1 (subtype EP1), 42kDa | 9087 | -0.020 | -0.2735 | No |
| 66 | TAAR5 | TAAR5 Entrez,  Source | trace amine associated receptor 5 | 9151 | -0.020 | -0.2759 | No |
| 67 | PARD3 | PARD3 Entrez,  Source | par-3 partitioning defective 3 homolog (C. elegans) | 9168 | -0.021 | -0.2759 | No |
| 68 | PTAFR | PTAFR Entrez,  Source | platelet-activating factor receptor | 9195 | -0.021 | -0.2764 | No |
| 69 | F2RL2 | F2RL2 Entrez,  Source | coagulation factor II (thrombin) receptor-like 2 | 9209 | -0.021 | -0.2763 | No |
| 70 | TACR2 | TACR2 Entrez,  Source | tachykinin receptor 2 | 9224 | -0.021 | -0.2761 | No |
| 71 | GRM6 | GRM6 Entrez,  Source | glutamate receptor, metabotropic 6 | 9391 | -0.023 | -0.2838 | No |
| 72 | SSTR3 | SSTR3 Entrez,  Source | somatostatin receptor 3 | 9416 | -0.023 | -0.2841 | No |
| 73 | CCKBR | CCKBR Entrez,  Source | cholecystokinin B receptor | 9494 | -0.024 | -0.2871 | No |
| 74 | GRIN2D | GRIN2D Entrez,  Source | glutamate receptor, ionotropic, N-methyl D-aspartate 2D | 9840 | -0.028 | -0.3039 | No |
| 75 | FPR1 | FPR1 Entrez,  Source | formyl peptide receptor 1 | 9873 | -0.028 | -0.3044 | No |
| 76 | BDKRB1 | BDKRB1 Entrez,  Source | bradykinin receptor B1 | 9911 | -0.029 | -0.3051 | No |
| 77 | ADRA1D | ADRA1D Entrez,  Source | adrenergic, alpha-1D-, receptor | 9994 | -0.030 | -0.3082 | No |
| 78 | DRD2 | DRD2 Entrez,  Source | dopamine receptor D2 | 10019 | -0.030 | -0.3082 | No |
| 79 | UTS2R | UTS2R Entrez,  Source | urotensin 2 receptor | 10029 | -0.030 | -0.3074 | No |
| 80 | GLRA1 | GLRA1 Entrez,  Source | glycine receptor, alpha 1 (startle disease/hyperekplexia, stiff man syndrome) | 10093 | -0.031 | -0.3094 | No |
| 81 | PTGDR | PTGDR Entrez,  Source | prostaglandin D2 receptor (DP) | 10129 | -0.032 | -0.3100 | No |
| 82 | THRB | THRB Entrez,  Source | thyroid hormone receptor, beta (erythroblastic leukemia viral (v-erb-a) oncogene homolog 2, avian) | 10309 | -0.034 | -0.3179 | No |
| 83 | TSHR | TSHR Entrez,  Source | thyroid stimulating hormone receptor | 10594 | -0.037 | -0.3311 | No |
| 84 | PRSS1 | PRSS1 Entrez,  Source | protease, serine, 1 (trypsin 1) | 10643 | -0.038 | -0.3321 | No |
| 85 | HTR2C | HTR2C Entrez,  Source | 5-hydroxytryptamine (serotonin) receptor 2C | 10657 | -0.038 | -0.3313 | No |
| 86 | P2RY6 | P2RY6 Entrez,  Source | pyrimidinergic receptor P2Y, G-protein coupled, 6 | 10789 | -0.039 | -0.3365 | No |
| 87 | P2RY10 | P2RY10 Entrez,  Source | purinergic receptor P2Y, G-protein coupled, 10 | 10897 | -0.041 | -0.3404 | No |
| 88 | MAS1 | MAS1 Entrez,  Source | MAS1 oncogene | 11060 | -0.043 | -0.3470 | No |
| 89 | DRD3 | DRD3 Entrez,  Source | dopamine receptor D3 | 11232 | -0.045 | -0.3541 | No |
| 90 | CHRNA6 | CHRNA6 Entrez,  Source | cholinergic receptor, nicotinic, alpha 6 | 11299 | -0.046 | -0.3557 | No |
| 91 | NPY2R | NPY2R Entrez,  Source | neuropeptide Y receptor Y2 | 11312 | -0.046 | -0.3544 | No |
| 92 | GRPR | GRPR Entrez,  Source | gastrin-releasing peptide receptor | 11328 | -0.046 | -0.3533 | No |
| 93 | GHSR | GHSR Entrez,  Source | growth hormone secretagogue receptor | 11527 | -0.048 | -0.3616 | No |
| 94 | SSTR1 | SSTR1 Entrez,  Source | somatostatin receptor 1 | 11571 | -0.049 | -0.3619 | No |
| 95 | GLRA3 | GLRA3 Entrez,  Source | glycine receptor, alpha 3 | 11580 | -0.049 | -0.3603 | No |
| 96 | CSH1 | CSH1 Entrez,  Source | chorionic somatomammotropin hormone 1 (placental lactogen) | 11596 | -0.049 | -0.3591 | No |
| 97 | CHRM3 | CHRM3 Entrez,  Source | cholinergic receptor, muscarinic 3 | 11748 | -0.051 | -0.3649 | No |
| 98 | GABRA4 | GABRA4 Entrez,  Source | gamma-aminobutyric acid (GABA) A receptor, alpha 4 | 11750 | -0.051 | -0.3629 | No |
| 99 | NPFFR1 | NPFFR1 Entrez,  Source | neuropeptide FF receptor 1 | 11761 | -0.051 | -0.3613 | No |
| 100 | VIPR2 | VIPR2 Entrez,  Source | vasoactive intestinal peptide receptor 2 | 11831 | -0.052 | -0.3628 | No |
| 101 | PTGER4 | PTGER4 Entrez,  Source | prostaglandin E receptor 4 (subtype EP4) | 11852 | -0.052 | -0.3617 | No |
| 102 | GRM4 | GRM4 Entrez,  Source | glutamate receptor, metabotropic 4 | 11899 | -0.053 | -0.3620 | No |
| 103 | AVPR2 | AVPR2 Entrez,  Source | arginine vasopressin receptor 2 (nephrogenic diabetes insipidus) | 11911 | -0.053 | -0.3604 | No |
| 104 | BRS3 | BRS3 Entrez,  Source | bombesin-like receptor 3 | 11932 | -0.054 | -0.3593 | No |
| 105 | GRIK5 | GRIK5 Entrez,  Source | glutamate receptor, ionotropic, kainate 5 | 11946 | -0.054 | -0.3578 | No |
| 106 | MTNR1B | MTNR1B Entrez,  Source | melatonin receptor 1B | 12080 | -0.055 | -0.3624 | No |
| 107 | CRHR1 | CRHR1 Entrez,  Source | corticotropin releasing hormone receptor 1 | 12098 | -0.056 | -0.3611 | No |
| 108 | GALR3 | GALR3 Entrez,  Source | galanin receptor 3 | 12110 | -0.056 | -0.3594 | No |
| 109 | SSTR2 | SSTR2 Entrez,  Source | somatostatin receptor 2 | 12200 | -0.057 | -0.3618 | No |
| 110 | GRIK4 | GRIK4 Entrez,  Source | glutamate receptor, ionotropic, kainate 4 | 12435 | -0.060 | -0.3715 | No |
| 111 | HRH3 | HRH3 Entrez,  Source | histamine receptor H3 | 12507 | -0.060 | -0.3727 | No |
| 112 | GABRR2 | GABRR2 Entrez,  Source | gamma-aminobutyric acid (GABA) receptor, rho 2 | 12637 | -0.062 | -0.3769 | No |
| 113 | AVPR1A | AVPR1A Entrez,  Source | arginine vasopressin receptor 1A | 12790 | -0.064 | -0.3822 | No |
| 114 | CHRNA4 | CHRNA4 Entrez,  Source | cholinergic receptor, nicotinic, alpha 4 | 12823 | -0.064 | -0.3813 | No |
| 115 | TRHR | TRHR Entrez,  Source | thyrotropin-releasing hormone receptor | 12884 | -0.065 | -0.3818 | No |
| 116 | GCGR | GCGR Entrez,  Source | glucagon receptor | 12932 | -0.066 | -0.3815 | No |
| 117 | GABRB2 | GABRB2 Entrez,  Source | gamma-aminobutyric acid (GABA) A receptor, beta 2 | 12987 | -0.066 | -0.3817 | No |
| 118 | CHRNA9 | CHRNA9 Entrez,  Source | cholinergic receptor, nicotinic, alpha 9 | 12999 | -0.067 | -0.3796 | No |
| 119 | CHRNB2 | CHRNB2 Entrez,  Source | cholinergic receptor, nicotinic, beta 2 (neuronal) | 13037 | -0.067 | -0.3788 | No |
| 120 | GHRHR | GHRHR Entrez,  Source | growth hormone releasing hormone receptor | 13089 | -0.068 | -0.3787 | No |
| 121 | PRLR | PRLR Entrez,  Source | prolactin receptor | 13287 | -0.070 | -0.3861 | No |
| 122 | TACR3 | TACR3 Entrez,  Source | tachykinin receptor 3 | 13323 | -0.071 | -0.3850 | No |
| 123 | P2RX2 | P2RX2 Entrez,  Source | purinergic receptor P2X, ligand-gated ion channel, 2 | 13334 | -0.071 | -0.3827 | No |
| 124 | PLG | PLG Entrez,  Source | plasminogen | 13368 | -0.071 | -0.3815 | No |
| 125 | GABRB3 | GABRB3 Entrez,  Source | gamma-aminobutyric acid (GABA) A receptor, beta 3 | 13446 | -0.072 | -0.3826 | No |
| 126 | GABRG1 | GABRG1 Entrez,  Source | gamma-aminobutyric acid (GABA) A receptor, gamma 1 | 13478 | -0.073 | -0.3813 | No |
| 127 | HRH2 | HRH2 Entrez,  Source | histamine receptor H2 | 13546 | -0.074 | -0.3818 | No |
| 128 | PTGIR | PTGIR Entrez,  Source | prostaglandin I2 (prostacyclin) receptor (IP) | 13565 | -0.074 | -0.3797 | No |
| 129 | HTR7 | HTR7 Entrez,  Source | 5-hydroxytryptamine (serotonin) receptor 7 (adenylate cyclase-coupled) | 13765 | -0.077 | -0.3869 | Yes |
| 130 | F2 | F2 Entrez,  Source | coagulation factor II (thrombin) | 13790 | -0.077 | -0.3851 | Yes |
| 131 | GHR | GHR Entrez,  Source | growth hormone receptor | 13857 | -0.078 | -0.3854 | Yes |
| 132 | ADCYAP1R1 | ADCYAP1R1 Entrez,  Source | adenylate cyclase activating polypeptide 1 (pituitary) receptor type I | 13910 | -0.078 | -0.3849 | Yes |
| 133 | GIPR | GIPR Entrez,  Source | gastric inhibitory polypeptide receptor | 13928 | -0.079 | -0.3826 | Yes |
| 134 | GRIA3 | GRIA3 Entrez,  Source | glutamate receptor, ionotrophic, AMPA 3 | 13938 | -0.079 | -0.3799 | Yes |
| 135 | VIPR1 | VIPR1 Entrez,  Source | vasoactive intestinal peptide receptor 1 | 13951 | -0.079 | -0.3774 | Yes |
| 136 | SSTR4 | SSTR4 Entrez,  Source | somatostatin receptor 4 | 13974 | -0.079 | -0.3753 | Yes |
| 137 | LTB4R | LTB4R Entrez,  Source | leukotriene B4 receptor | 14003 | -0.080 | -0.3736 | Yes |
| 138 | GNRHR | GNRHR Entrez,  Source | gonadotropin-releasing hormone receptor | 14012 | -0.080 | -0.3708 | Yes |
| 139 | CHRNE | CHRNE Entrez,  Source | cholinergic receptor, nicotinic, epsilon | 14034 | -0.080 | -0.3686 | Yes |
| 140 | CHRNA2 | CHRNA2 Entrez,  Source | cholinergic receptor, nicotinic, alpha 2 (neuronal) | 14043 | -0.080 | -0.3658 | Yes |
| 141 | PRLHR | PRLHR Entrez,  Source | prolactin releasing hormone receptor | 14047 | -0.080 | -0.3627 | Yes |
| 142 | HTR4 | HTR4 Entrez,  Source | 5-hydroxytryptamine (serotonin) receptor 4 | 14121 | -0.081 | -0.3633 | Yes |
| 143 | GRIN2A | GRIN2A Entrez,  Source | glutamate receptor, ionotropic, N-methyl D-aspartate 2A | 14179 | -0.082 | -0.3629 | Yes |
| 144 | CHRM1 | CHRM1 Entrez,  Source | cholinergic receptor, muscarinic 1 | 14243 | -0.083 | -0.3628 | Yes |
| 145 | CHRND | CHRND Entrez,  Source | cholinergic receptor, nicotinic, delta | 14269 | -0.083 | -0.3608 | Yes |
| 146 | HTR5A | HTR5A Entrez,  Source | 5-hydroxytryptamine (serotonin) receptor 5A | 14328 | -0.084 | -0.3604 | Yes |
| 147 | TSPO | TSPO Entrez,  Source | translocator protein (18kDa) | 14451 | -0.085 | -0.3633 | Yes |
| 148 | MC2R | MC2R Entrez,  Source | melanocortin 2 receptor (adrenocorticotropic hormone) | 14459 | -0.086 | -0.3602 | Yes |
| 149 | GRIA4 | GRIA4 Entrez,  Source | glutamate receptor, ionotrophic, AMPA 4 | 14699 | -0.089 | -0.3690 | Yes |
| 150 | TAAR9 | TAAR9 Entrez,  Source | trace amine associated receptor 9 | 14747 | -0.090 | -0.3678 | Yes |
| 151 | P2RX1 | P2RX1 Entrez,  Source | purinergic receptor P2X, ligand-gated ion channel, 1 | 14748 | -0.090 | -0.3642 | Yes |
| 152 | TBXA2R | TBXA2R Entrez,  Source | thromboxane A2 receptor | 14764 | -0.090 | -0.3614 | Yes |
| 153 | PTGER2 | PTGER2 Entrez,  Source | prostaglandin E receptor 2 (subtype EP2), 53kDa | 14797 | -0.091 | -0.3594 | Yes |
| 154 | GRID1 | GRID1 Entrez,  Source | glutamate receptor, ionotropic, delta 1 | 14837 | -0.091 | -0.3577 | Yes |
| 155 | F2R | F2R Entrez,  Source | coagulation factor II (thrombin) receptor | 14863 | -0.091 | -0.3553 | Yes |
| 156 | MC1R | MC1R Entrez,  Source | melanocortin 1 receptor (alpha melanocyte stimulating hormone receptor) | 14891 | -0.092 | -0.3530 | Yes |
| 157 | GPR156 | GPR156 Entrez,  Source | G protein-coupled receptor 156 | 14948 | -0.093 | -0.3522 | Yes |
| 158 | ADRA2A | ADRA2A Entrez,  Source | adrenergic, alpha-2A-, receptor | 14995 | -0.094 | -0.3508 | Yes |
| 159 | TACR1 | TACR1 Entrez,  Source | tachykinin receptor 1 | 15102 | -0.095 | -0.3525 | Yes |
| 160 | GRM7 | GRM7 Entrez,  Source | glutamate receptor, metabotropic 7 | 15117 | -0.096 | -0.3493 | Yes |
| 161 | GABRG2 | GABRG2 Entrez,  Source | gamma-aminobutyric acid (GABA) A receptor, gamma 2 | 15167 | -0.097 | -0.3480 | Yes |
| 162 | GRIK2 | GRIK2 Entrez,  Source | glutamate receptor, ionotropic, kainate 2 | 15191 | -0.097 | -0.3453 | Yes |
| 163 | P2RY8 | P2RY8 Entrez,  Source | purinergic receptor P2Y, G-protein coupled, 8 | 15226 | -0.098 | -0.3431 | Yes |
| 164 | GABBR1 | GABBR1 Entrez,  Source | gamma-aminobutyric acid (GABA) B receptor, 1 | 15237 | -0.098 | -0.3397 | Yes |
| 165 | TSHB | TSHB Entrez,  Source | thyroid stimulating hormone, beta | 15248 | -0.098 | -0.3362 | Yes |
| 166 | FSHB | FSHB Entrez,  Source | follicle stimulating hormone, beta polypeptide | 15356 | -0.100 | -0.3378 | Yes |
| 167 | CHRM2 | CHRM2 Entrez,  Source | cholinergic receptor, muscarinic 2 | 15501 | -0.102 | -0.3411 | Yes |
| 168 | OPRK1 | OPRK1 Entrez,  Source | opioid receptor, kappa 1 | 15510 | -0.102 | -0.3374 | Yes |
| 169 | OPRL1 | OPRL1 Entrez,  Source | opiate receptor-like 1 | 15578 | -0.103 | -0.3367 | Yes |
| 170 | AVPR1B | AVPR1B Entrez,  Source | arginine vasopressin receptor 1B | 15598 | -0.104 | -0.3335 | Yes |
| 171 | RXFP2 | RXFP2 Entrez,  Source | relaxin/insulin-like family peptide receptor 2 | 15657 | -0.105 | -0.3323 | Yes |
| 172 | HTR2B | HTR2B Entrez,  Source | 5-hydroxytryptamine (serotonin) receptor 2B | 15714 | -0.106 | -0.3310 | Yes |
| 173 | HCRTR2 | HCRTR2 Entrez,  Source | hypocretin (orexin) receptor 2 | 15820 | -0.108 | -0.3321 | Yes |
| 174 | FSHR | FSHR Entrez,  Source | follicle stimulating hormone receptor | 15883 | -0.109 | -0.3309 | Yes |
| 175 | HTR1F | HTR1F Entrez,  Source | 5-hydroxytryptamine (serotonin) receptor 1F | 15942 | -0.110 | -0.3294 | Yes |
| 176 | EDNRB | EDNRB Entrez,  Source | endothelin receptor type B | 16031 | -0.112 | -0.3295 | Yes |
| 177 | LHB | LHB Entrez,  Source | luteinizing hormone beta polypeptide | 16053 | -0.112 | -0.3261 | Yes |
| 178 | GABRD | GABRD Entrez,  Source | gamma-aminobutyric acid (GABA) A receptor, delta | 16118 | -0.113 | -0.3248 | Yes |
| 179 | GRIK3 | GRIK3 Entrez,  Source | glutamate receptor, ionotropic, kainate 3 | 16124 | -0.114 | -0.3205 | Yes |
| 180 | GABRA1 | GABRA1 Entrez,  Source | gamma-aminobutyric acid (GABA) A receptor, alpha 1 | 16127 | -0.114 | -0.3160 | Yes |
| 181 | GRM3 | GRM3 Entrez,  Source | glutamate receptor, metabotropic 3 | 16186 | -0.115 | -0.3144 | Yes |
| 182 | SSTR5 | SSTR5 Entrez,  Source | somatostatin receptor 5 | 16199 | -0.115 | -0.3104 | Yes |
| 183 | GALR2 | GALR2 Entrez,  Source | galanin receptor 2 | 16205 | -0.115 | -0.3060 | Yes |
| 184 | RXFP1 | RXFP1 Entrez,  Source | relaxin/insulin-like family peptide receptor 1 | 16231 | -0.116 | -0.3027 | Yes |
| 185 | OPRD1 | OPRD1 Entrez,  Source | opioid receptor, delta 1 | 16267 | -0.116 | -0.2998 | Yes |
| 186 | AGTR2 | AGTR2 Entrez,  Source | angiotensin II receptor, type 2 | 16275 | -0.117 | -0.2955 | Yes |
| 187 | GRIN3A | GRIN3A Entrez,  Source | glutamate receptor, ionotropic, N-methyl-D-aspartate 3A | 16349 | -0.118 | -0.2945 | Yes |
| 188 | CHRNA10 | CHRNA10 Entrez,  Source | cholinergic receptor, nicotinic, alpha 10 | 16364 | -0.118 | -0.2905 | Yes |
| 189 | LTB4R2 | LTB4R2 Entrez,  Source | leukotriene B4 receptor 2 | 16410 | -0.119 | -0.2880 | Yes |
| 190 | CHRM5 | CHRM5 Entrez,  Source | cholinergic receptor, muscarinic 5 | 16484 | -0.121 | -0.2869 | Yes |
| 191 | LEP | LEP Entrez,  Source | leptin (obesity homolog, mouse) | 16530 | -0.121 | -0.2844 | Yes |
| 192 | ADRB3 | ADRB3 Entrez,  Source | adrenergic, beta-3-, receptor | 16541 | -0.122 | -0.2800 | Yes |
| 193 | ADRB1 | ADRB1 Entrez,  Source | adrenergic, beta-1-, receptor | 16547 | -0.122 | -0.2754 | Yes |
| 194 | P2RY4 | P2RY4 Entrez,  Source | pyrimidinergic receptor P2Y, G-protein coupled, 4 | 16556 | -0.122 | -0.2709 | Yes |
| 195 | GABRA5 | GABRA5 Entrez,  Source | gamma-aminobutyric acid (GABA) A receptor, alpha 5 | 16574 | -0.122 | -0.2668 | Yes |
| 196 | MC4R | MC4R Entrez,  Source | melanocortin 4 receptor | 16581 | -0.122 | -0.2622 | Yes |
| 197 | NMUR2 | NMUR2 Entrez,  Source | neuromedin U receptor 2 | 16607 | -0.123 | -0.2586 | Yes |
| 198 | GABRB1 | GABRB1 Entrez,  Source | gamma-aminobutyric acid (GABA) A receptor, beta 1 | 16630 | -0.124 | -0.2547 | Yes |
| 199 | ADRA1A | ADRA1A Entrez,  Source | adrenergic, alpha-1A-, receptor | 16645 | -0.124 | -0.2505 | Yes |
| 200 | HTR1D | HTR1D Entrez,  Source | 5-hydroxytryptamine (serotonin) receptor 1D | 16675 | -0.125 | -0.2469 | Yes |
| 201 | TAAR8 | TAAR8 Entrez,  Source | trace amine associated receptor 8 | 16871 | -0.129 | -0.2518 | Yes |
| 202 | GLP1R | GLP1R Entrez,  Source | glucagon-like peptide 1 receptor | 16941 | -0.131 | -0.2501 | Yes |
| 203 | GABRA2 | GABRA2 Entrez,  Source | gamma-aminobutyric acid (GABA) A receptor, alpha 2 | 17034 | -0.134 | -0.2495 | Yes |
| 204 | TRPV1 | TRPV1 Entrez,  Source | transient receptor potential cation channel, subfamily V, member 1 | 17135 | -0.136 | -0.2492 | Yes |
| 205 | HTR2A | HTR2A Entrez,  Source | 5-hydroxytryptamine (serotonin) receptor 2A | 17152 | -0.137 | -0.2445 | Yes |
| 206 | DRD1 | DRD1 Entrez,  Source | dopamine receptor D1 | 17157 | -0.137 | -0.2392 | Yes |
| 207 | CHRNB3 | CHRNB3 Entrez,  Source | cholinergic receptor, nicotinic, beta 3 | 17188 | -0.138 | -0.2352 | Yes |
| 208 | CTSG | CTSG Entrez,  Source | cathepsin G | 17229 | -0.140 | -0.2317 | Yes |
| 209 | CHRNA5 | CHRNA5 Entrez,  Source | cholinergic receptor, nicotinic, alpha 5 | 17235 | -0.140 | -0.2263 | Yes |
| 210 | MCHR1 | MCHR1 Entrez,  Source | melanin-concentrating hormone receptor 1 | 17257 | -0.140 | -0.2217 | Yes |
| 211 | PPYR1 | PPYR1 Entrez,  Source | pancreatic polypeptide receptor 1 | 17383 | -0.145 | -0.2224 | Yes |
| 212 | DRD5 | DRD5 Entrez,  Source | dopamine receptor D5 | 17506 | -0.148 | -0.2227 | Yes |
| 213 | ADRA1B | ADRA1B Entrez,  Source | adrenergic, alpha-1B-, receptor | 17525 | -0.149 | -0.2177 | Yes |
| 214 | THRA | THRA Entrez,  Source | thyroid hormone receptor, alpha (erythroblastic leukemia viral (v-erb-a) oncogene homolog, avian) | 17555 | -0.150 | -0.2131 | Yes |
| 215 | GABRQ | GABRQ Entrez,  Source | gamma-aminobutyric acid (GABA) receptor, theta | 17614 | -0.152 | -0.2100 | Yes |
| 216 | PRL | PRL Entrez,  Source | prolactin | 17664 | -0.153 | -0.2064 | Yes |
| 217 | GLRB | GLRB Entrez,  Source | glycine receptor, beta | 17698 | -0.155 | -0.2019 | Yes |
| 218 | AGTR1 | AGTR1 Entrez,  Source | angiotensin II receptor, type 1 | 17714 | -0.155 | -0.1964 | Yes |
| 219 | GLP2R | GLP2R Entrez,  Source | glucagon-like peptide 2 receptor | 17717 | -0.155 | -0.1903 | Yes |
| 220 | CHRM4 | CHRM4 Entrez,  Source | cholinergic receptor, muscarinic 4 | 17856 | -0.161 | -0.1909 | Yes |
| 221 | HCRTR1 | HCRTR1 Entrez,  Source | hypocretin (orexin) receptor 1 | 17925 | -0.164 | -0.1879 | Yes |
| 222 | CGA | CGA Entrez,  Source | glycoprotein hormones, alpha polypeptide | 17947 | -0.164 | -0.1824 | Yes |
| 223 | NPBWR2 | NPBWR2 Entrez,  Source | neuropeptides B/W receptor 2 | 17995 | -0.166 | -0.1781 | Yes |
| 224 | GPR50 | GPR50 Entrez,  Source | G protein-coupled receptor 50 | 18055 | -0.168 | -0.1744 | Yes |
| 225 | GRM8 | GRM8 Entrez,  Source | glutamate receptor, metabotropic 8 | 18060 | -0.169 | -0.1678 | Yes |
| 226 | CRHR2 | CRHR2 Entrez,  Source | corticotropin releasing hormone receptor 2 | 18143 | -0.173 | -0.1650 | Yes |
| 227 | EDNRA | EDNRA Entrez,  Source | endothelin receptor type A | 18179 | -0.175 | -0.1598 | Yes |
| 228 | CNR2 | CNR2 Entrez,  Source | cannabinoid receptor 2 (macrophage) | 18192 | -0.175 | -0.1534 | Yes |
| 229 | TAAR1 | TAAR1 Entrez,  Source | trace amine associated receptor 1 | 18229 | -0.177 | -0.1481 | Yes |
| 230 | GPR83 | GPR83 Entrez,  Source | G protein-coupled receptor 83 | 18256 | -0.179 | -0.1422 | Yes |
| 231 | MC5R | MC5R Entrez,  Source | melanocortin 5 receptor | 18264 | -0.179 | -0.1354 | Yes |
| 232 | P2RY14 | P2RY14 Entrez,  Source | purinergic receptor P2Y, G-protein coupled, 14 | 18395 | -0.187 | -0.1346 | Yes |
| 233 | HTR1B | HTR1B Entrez,  Source | 5-hydroxytryptamine (serotonin) receptor 1B | 18409 | -0.187 | -0.1277 | Yes |
| 234 | CCKAR | CCKAR Entrez,  Source | cholecystokinin A receptor | 18433 | -0.189 | -0.1213 | Yes |
| 235 | OPRM1 | OPRM1 Entrez,  Source | opioid receptor, mu 1 | 18439 | -0.190 | -0.1139 | Yes |
| 236 | P2RX5 | P2RX5 Entrez,  Source | purinergic receptor P2X, ligand-gated ion channel, 5 | 18546 | -0.197 | -0.1115 | Yes |
| 237 | GRIN2B | GRIN2B Entrez,  Source | glutamate receptor, ionotropic, N-methyl D-aspartate 2B | 18602 | -0.200 | -0.1063 | Yes |
| 238 | HTR6 | HTR6 Entrez,  Source | 5-hydroxytryptamine (serotonin) receptor 6 | 19011 | -0.245 | -0.1176 | Yes |
| 239 | CHRNA3 | CHRNA3 Entrez,  Source | cholinergic receptor, nicotinic, alpha 3 | 19037 | -0.250 | -0.1088 | Yes |
| 240 | GRIA1 | GRIA1 Entrez,  Source | glutamate receptor, ionotropic, AMPA 1 | 19083 | -0.257 | -0.1008 | Yes |
| 241 | GRID2 | GRID2 Entrez,  Source | glutamate receptor, ionotropic, delta 2 | 19092 | -0.259 | -0.0908 | Yes |
| 242 | CHRNB4 | CHRNB4 Entrez,  Source | cholinergic receptor, nicotinic, beta 4 | 19116 | -0.264 | -0.0813 | Yes |
| 243 | KISS1R | KISS1R Entrez,  Source | KISS1 receptor | 19166 | -0.276 | -0.0728 | Yes |
| 244 | MTNR1A | MTNR1A Entrez,  Source | melatonin receptor 1A | 19170 | -0.277 | -0.0618 | Yes |
| 245 | CYSLTR1 | CYSLTR1 Entrez,  Source | cysteinyl leukotriene receptor 1 | 19214 | -0.292 | -0.0522 | Yes |
| 246 | GZMA | GZMA Entrez,  Source | granzyme A (granzyme 1, cytotoxic T-lymphocyte-associated serine esterase 3) | 19245 | -0.303 | -0.0416 | Yes |
| 247 | ADRA2C | ADRA2C Entrez,  Source | adrenergic, alpha-2C-, receptor | 19274 | -0.314 | -0.0304 | Yes |
| 248 | PRSS3 | PRSS3 Entrez,  Source | protease, serine, 3 (mesotrypsin) | 19276 | -0.315 | -0.0178 | Yes |
| 249 | PRSS2 | PRSS2 Entrez,  Source | protease, serine, 2 (trypsin 2) | 19350 | -0.357 | -0.0072 | Yes |
| 250 | GABRA3 | GABRA3 Entrez,  Source | gamma-aminobutyric acid (GABA) A receptor, alpha 3 | 19437 | -0.442 | 0.0062 | Yes |
Table: GSEA details [plain text format]

  

Fig 2: KEGG\_NEUROACTIVE\_LIGAND\_RECEPTOR\_INTERACTION      
 Blue-Pink O' Gram in the Space of the Analyzed GeneSet

  

Fig 3: KEGG\_NEUROACTIVE\_LIGAND\_RECEPTOR\_INTERACTION: Random ES distribution      
 Gene set null distribution of ES for **KEGG\_NEUROACTIVE\_LIGAND\_RECEPTOR\_INTERACTION**

  
