# Supplementary material for: Comprehensive analysis of β-catenin target genes in colorectal carcinoma cell lines with deregulated Wnt/β-catenin signaling
Source: BMC Genomics. 2014 Jan 28;15:74. doi: 10.1186/1471-2164-15-74 (PMC3909937; doi:10.1186/1471-2164-15-74)
Supplement: Additional file 5 — GSEA analysis using the KEGG pathway database. This zipped file contains confirming data of the GSEA analysis. The names of the directories containing the files were composed of the term ‘GSEA’, the name of the cell line, e.g. DLD1, SW480, or LS174T, and the pathway database (KEGG). Please use a web browser to view the files with the name ‘index.html’ in the corresponding directories to start exploring the data. [file 1471-2164-15-74-S5.zip › GSEA KEGG SW480/KEGG_NITROGEN_METABOLISM.html]

Details for gene set KEGG\_NITROGEN\_METABOLISM[GSEA]

|  || Dataset | SW480\_collapsed\_to\_symbols.class.cls#b\_versus\_bg.class.cls#b\_versus\_bg\_repos |
| Phenotype | class.cls#b\_versus\_bg\_repos |
| Upregulated in class | 0 |
| GeneSet | KEGG\_NITROGEN\_METABOLISM |
| Enrichment Score (ES) | -0.5122469 |
| Normalized Enrichment Score (NES) | -1.4652333 |
| Nominal p-value | 0.0626087 |
| FDR q-value | 0.19683868 |
| FWER p-Value | 0.97 |
Table: GSEA Results Summary

  

Fig 1: Enrichment plot: KEGG\_NITROGEN\_METABOLISM      
 Profile of the Running ES Score & Positions of GeneSet Members on the Rank Ordered List

  

| PROBE | GENE SYMBOL | GENE\_TITLE | RANK IN GENE LIST | RANK METRIC SCORE | RUNNING ES | CORE ENRICHMENT || 1 | CA9 | CA9 Entrez,  Source | carbonic anhydrase IX | 1082 | 0.181 | 0.0142 | No |
| 2 | GLS | GLS Entrez,  Source | glutaminase | 2685 | 0.092 | -0.0323 | No |
| 3 | AMT | AMT Entrez,  Source | aminomethyltransferase | 4062 | 0.054 | -0.0820 | No |
| 4 | CA12 | CA12 Entrez,  Source | carbonic anhydrase XII | 6768 | 0.009 | -0.2170 | No |
| 5 | HAL | HAL Entrez,  Source | histidine ammonia-lyase | 7665 | -0.003 | -0.2619 | No |
| 6 | GLUL | GLUL Entrez,  Source | glutamate-ammonia ligase (glutamine synthetase) | 7969 | -0.007 | -0.2749 | No |
| 7 | CA7 | CA7 Entrez,  Source | carbonic anhydrase VII | 8797 | -0.016 | -0.3110 | No |
| 8 | CA2 | CA2 Entrez,  Source | carbonic anhydrase II | 10777 | -0.039 | -0.3973 | No |
| 9 | CPS1 | CPS1 Entrez,  Source | carbamoyl-phosphate synthetase 1, mitochondrial | 11677 | -0.050 | -0.4240 | No |
| 10 | CA13 | CA13 Entrez,  Source | carbonic anhydrase XIII | 13017 | -0.067 | -0.4669 | No |
| 11 | CA4 | CA4 Entrez,  Source | carbonic anhydrase IV | 13092 | -0.068 | -0.4447 | No |
| 12 | CA3 | CA3 Entrez,  Source | carbonic anhydrase III, muscle specific | 13742 | -0.076 | -0.4486 | No |
| 13 | CA5A | CA5A Entrez,  Source | carbonic anhydrase VA, mitochondrial | 14147 | -0.082 | -0.4380 | No |
| 14 | CA5B | CA5B Entrez,  Source | carbonic anhydrase VB, mitochondrial | 15599 | -0.104 | -0.4724 | Yes |
| 15 | CA14 | CA14 Entrez,  Source | carbonic anhydrase XIV | 16133 | -0.114 | -0.4560 | Yes |
| 16 | GLUD2 | GLUD2 Entrez,  Source | glutamate dehydrogenase 2 | 16583 | -0.122 | -0.4320 | Yes |
| 17 | GLUD1 | GLUD1 Entrez,  Source | glutamate dehydrogenase 1 | 17237 | -0.140 | -0.4118 | Yes |
| 18 | CA6 | CA6 Entrez,  Source | carbonic anhydrase VI | 17472 | -0.148 | -0.3671 | Yes |
| 19 | CA1 | CA1 Entrez,  Source | carbonic anhydrase I | 17728 | -0.155 | -0.3205 | Yes |
| 20 | ASNS | ASNS Entrez,  Source | asparagine synthetase | 18792 | -0.216 | -0.2920 | Yes |
| 21 | CTH | CTH Entrez,  Source | cystathionase (cystathionine gamma-lyase) | 19082 | -0.257 | -0.2081 | Yes |
| 22 | CA8 | CA8 Entrez,  Source | carbonic anhydrase VIII | 19218 | -0.293 | -0.1027 | Yes |
| 23 | GLS2 | GLS2 Entrez,  Source | glutaminase 2 (liver, mitochondrial) | 19265 | -0.312 | 0.0149 | Yes |
Table: GSEA details [plain text format]

  

Fig 2: KEGG\_NITROGEN\_METABOLISM      
 Blue-Pink O' Gram in the Space of the Analyzed GeneSet

  

Fig 3: KEGG\_NITROGEN\_METABOLISM: Random ES distribution      
 Gene set null distribution of ES for **KEGG\_NITROGEN\_METABOLISM**

  
