# Supplementary material for: Comprehensive analysis of β-catenin target genes in colorectal carcinoma cell lines with deregulated Wnt/β-catenin signaling
Source: BMC Genomics. 2014 Jan 28;15:74. doi: 10.1186/1471-2164-15-74 (PMC3909937; doi:10.1186/1471-2164-15-74)
Supplement: Additional file 5 — GSEA analysis using the KEGG pathway database. This zipped file contains confirming data of the GSEA analysis. The names of the directories containing the files were composed of the term ‘GSEA’, the name of the cell line, e.g. DLD1, SW480, or LS174T, and the pathway database (KEGG). Please use a web browser to view the files with the name ‘index.html’ in the corresponding directories to start exploring the data. [file 1471-2164-15-74-S5.zip › GSEA KEGG SW480/KEGG_NUCLEOTIDE_EXCISION_REPAIR.html]

Details for gene set KEGG\_NUCLEOTIDE\_EXCISION\_REPAIR[GSEA]

|  || Dataset | SW480\_collapsed\_to\_symbols.class.cls#b\_versus\_bg.class.cls#b\_versus\_bg\_repos |
| Phenotype | class.cls#b\_versus\_bg\_repos |
| Upregulated in class | 1 |
| GeneSet | KEGG\_NUCLEOTIDE\_EXCISION\_REPAIR |
| Enrichment Score (ES) | 0.50101286 |
| Normalized Enrichment Score (NES) | 1.7541848 |
| Nominal p-value | 0.0071770335 |
| FDR q-value | 0.045322638 |
| FWER p-Value | 0.253 |
Table: GSEA Results Summary

  

Fig 1: Enrichment plot: KEGG\_NUCLEOTIDE\_EXCISION\_REPAIR      
 Profile of the Running ES Score & Positions of GeneSet Members on the Rank Ordered List

  

| PROBE | GENE SYMBOL | GENE\_TITLE | RANK IN GENE LIST | RANK METRIC SCORE | RUNNING ES | CORE ENRICHMENT || 1 | POLD4 | POLD4 Entrez,  Source | polymerase (DNA-directed), delta 4 | 621 | 0.247 | 0.0524 | Yes |
| 2 | DDB2 | DDB2 Entrez,  Source | damage-specific DNA binding protein 2, 48kDa | 737 | 0.226 | 0.1236 | Yes |
| 3 | RPA1 | RPA1 Entrez,  Source | replication protein A1, 70kDa | 1399 | 0.155 | 0.1425 | Yes |
| 4 | GTF2H5 | GTF2H5 Entrez,  Source | general transcription factor IIH, polypeptide 5 | 1810 | 0.129 | 0.1655 | Yes |
| 5 | POLE2 | POLE2 Entrez,  Source | polymerase (DNA directed), epsilon 2 (p59 subunit) | 1885 | 0.126 | 0.2047 | Yes |
| 6 | GTF2H4 | GTF2H4 Entrez,  Source | general transcription factor IIH, polypeptide 4, 52kDa | 2104 | 0.116 | 0.2331 | Yes |
| 7 | LIG1 | LIG1 Entrez,  Source | ligase I, DNA, ATP-dependent | 2306 | 0.107 | 0.2594 | Yes |
| 8 | CCNH | CCNH Entrez,  Source | cyclin H | 2364 | 0.105 | 0.2923 | Yes |
| 9 | ERCC2 | ERCC2 Entrez,  Source | excision repair cross-complementing rodent repair deficiency, complementation group 2 (xeroderma pigmentosum D) | 2655 | 0.094 | 0.3094 | Yes |
| 10 | RFC2 | RFC2 Entrez,  Source | replication factor C (activator 1) 2, 40kDa | 2739 | 0.091 | 0.3361 | Yes |
| 11 | ERCC1 | ERCC1 Entrez,  Source | excision repair cross-complementing rodent repair deficiency, complementation group 1 (includes overlapping antisense sequence) | 2934 | 0.085 | 0.3550 | Yes |
| 12 | CUL4B | CUL4B Entrez,  Source | cullin 4B | 3217 | 0.076 | 0.3663 | Yes |
| 13 | GTF2H1 | GTF2H1 Entrez,  Source | general transcription factor IIH, polypeptide 1, 62kDa | 3659 | 0.063 | 0.3653 | Yes |
| 14 | RPA3 | RPA3 Entrez,  Source | replication protein A3, 14kDa | 3777 | 0.061 | 0.3800 | Yes |
| 15 | RFC5 | RFC5 Entrez,  Source | replication factor C (activator 1) 5, 36.5kDa | 3791 | 0.060 | 0.3999 | Yes |
| 16 | POLE3 | POLE3 Entrez,  Source | polymerase (DNA directed), epsilon 3 (p17 subunit) | 3901 | 0.058 | 0.4141 | Yes |
| 17 | ERCC5 | ERCC5 Entrez,  Source | me)) | 3936 | 0.057 | 0.4319 | Yes |
| 18 | XPC | XPC Entrez,  Source | xeroderma pigmentosum, complementation group C | 4007 | 0.055 | 0.4472 | Yes |
| 19 | POLD3 | POLD3 Entrez,  Source | polymerase (DNA-directed), delta 3, accessory subunit | 4089 | 0.054 | 0.4613 | Yes |
| 20 | RAD23A | RAD23A Entrez,  Source | RAD23 homolog A (S. cerevisiae) | 4310 | 0.049 | 0.4667 | Yes |
| 21 | XPA | XPA Entrez,  Source | xeroderma pigmentosum, complementation group A | 4355 | 0.048 | 0.4808 | Yes |
| 22 | GTF2H2 | GTF2H2 Entrez,  Source | general transcription factor IIH, polypeptide 2, 44kDa | 4392 | 0.047 | 0.4950 | Yes |
| 23 | RAD23B | RAD23B Entrez,  Source | RAD23 homolog B (S. cerevisiae) | 4664 | 0.042 | 0.4953 | Yes |
| 24 | PCNA | PCNA Entrez,  Source | proliferating cell nuclear antigen | 4937 | 0.036 | 0.4937 | Yes |
| 25 | GTF2H3 | GTF2H3 Entrez,  Source | general transcription factor IIH, polypeptide 3, 34kDa | 5180 | 0.032 | 0.4923 | Yes |
| 26 | RFC4 | RFC4 Entrez,  Source | replication factor C (activator 1) 4, 37kDa | 5300 | 0.030 | 0.4965 | Yes |
| 27 | RFC1 | RFC1 Entrez,  Source | replication factor C (activator 1) 1, 145kDa | 5403 | 0.029 | 0.5010 | Yes |
| 28 | CETN2 | CETN2 Entrez,  Source | centrin, EF-hand protein, 2 | 6760 | 0.009 | 0.4346 | No |
| 29 | POLD2 | POLD2 Entrez,  Source | polymerase (DNA directed), delta 2, regulatory subunit 50kDa | 7071 | 0.005 | 0.4202 | No |
| 30 | RBX1 | RBX1 Entrez,  Source | ring-box 1 | 7189 | 0.003 | 0.4153 | No |
| 31 | POLE | POLE Entrez,  Source | polymerase (DNA directed), epsilon | 7481 | -0.000 | 0.4005 | No |
| 32 | RFC3 | RFC3 Entrez,  Source | replication factor C (activator 1) 3, 38kDa | 7608 | -0.002 | 0.3947 | No |
| 33 | RPA2 | RPA2 Entrez,  Source | replication protein A2, 32kDa | 7783 | -0.004 | 0.3873 | No |
| 34 | ERCC3 | ERCC3 Entrez,  Source | excision repair cross-complementing rodent repair deficiency, complementation group 3 (xeroderma pigmentosum group B complementing) | 8685 | -0.015 | 0.3462 | No |
| 35 | MNAT1 | MNAT1 Entrez,  Source | menage a trois homolog 1, cyclin H assembly factor (Xenopus laevis) | 9629 | -0.026 | 0.3066 | No |
| 36 | POLD1 | POLD1 Entrez,  Source | polymerase (DNA directed), delta 1, catalytic subunit 125kDa | 9782 | -0.028 | 0.3082 | No |
| 37 | ERCC4 | ERCC4 Entrez,  Source | excision repair cross-complementing rodent repair deficiency, complementation group 4 | 11067 | -0.043 | 0.2571 | No |
| 38 | ERCC8 | ERCC8 Entrez,  Source | excision repair cross-complementing rodent repair deficiency, complementation group 8 | 11127 | -0.044 | 0.2689 | No |
| 39 | POLE4 | POLE4 Entrez,  Source | polymerase (DNA-directed), epsilon 4 (p12 subunit) | 11351 | -0.046 | 0.2734 | No |
| 40 | DDB1 | DDB1 Entrez,  Source | damage-specific DNA binding protein 1, 127kDa | 12270 | -0.057 | 0.2459 | No |
| 41 | CUL4A | CUL4A Entrez,  Source | cullin 4A | 13331 | -0.071 | 0.2158 | No |
| 42 | RPA4 | RPA4 Entrez,  Source | replication protein A4, 34kDa | 17245 | -0.140 | 0.0630 | No |
| 43 | CDK7 | CDK7 Entrez,  Source | cyclin-dependent kinase 7 (MO15 homolog, Xenopus laevis, cdk-activating kinase) | 17894 | -0.162 | 0.0852 | No |
Table: GSEA details [plain text format]

  

Fig 2: KEGG\_NUCLEOTIDE\_EXCISION\_REPAIR      
 Blue-Pink O' Gram in the Space of the Analyzed GeneSet

  

Fig 3: KEGG\_NUCLEOTIDE\_EXCISION\_REPAIR: Random ES distribution      
 Gene set null distribution of ES for **KEGG\_NUCLEOTIDE\_EXCISION\_REPAIR**

  
