# Supplementary material for: Comprehensive analysis of β-catenin target genes in colorectal carcinoma cell lines with deregulated Wnt/β-catenin signaling
Source: BMC Genomics. 2014 Jan 28;15:74. doi: 10.1186/1471-2164-15-74 (PMC3909937; doi:10.1186/1471-2164-15-74)
Supplement: Additional file 5 — GSEA analysis using the KEGG pathway database. This zipped file contains confirming data of the GSEA analysis. The names of the directories containing the files were composed of the term ‘GSEA’, the name of the cell line, e.g. DLD1, SW480, or LS174T, and the pathway database (KEGG). Please use a web browser to view the files with the name ‘index.html’ in the corresponding directories to start exploring the data. [file 1471-2164-15-74-S5.zip › GSEA KEGG SW480/KEGG_OLFACTORY_TRANSDUCTION.html]

Details for gene set KEGG\_OLFACTORY\_TRANSDUCTION[GSEA]

|  || Dataset | SW480\_collapsed\_to\_symbols.class.cls#b\_versus\_bg.class.cls#b\_versus\_bg\_repos |
| Phenotype | class.cls#b\_versus\_bg\_repos |
| Upregulated in class | 0 |
| GeneSet | KEGG\_OLFACTORY\_TRANSDUCTION |
| Enrichment Score (ES) | -0.42809394 |
| Normalized Enrichment Score (NES) | -1.688829 |
| Nominal p-value | 0.0033500837 |
| FDR q-value | 0.08572411 |
| FWER p-Value | 0.379 |
Table: GSEA Results Summary

  

Fig 1: Enrichment plot: KEGG\_OLFACTORY\_TRANSDUCTION      
 Profile of the Running ES Score & Positions of GeneSet Members on the Rank Ordered List

  

| PROBE | GENE SYMBOL | GENE\_TITLE | RANK IN GENE LIST | RANK METRIC SCORE | RUNNING ES | CORE ENRICHMENT || 1 | PRKX | PRKX Entrez,  Source | protein kinase, X-linked | 207 | 0.397 | 0.0262 | No |
| 2 | CAMK2D | CAMK2D Entrez,  Source | calcium/calmodulin-dependent protein kinase (CaM kinase) II delta | 744 | 0.225 | 0.0194 | No |
| 3 | CALM3 | CALM3 Entrez,  Source | calmodulin 3 (phosphorylase kinase, delta) | 2233 | 0.110 | -0.0469 | No |
| 4 | OR7A17 | OR7A17 Entrez,  Source | olfactory receptor, family 7, subfamily A, member 17 | 2365 | 0.105 | -0.0439 | No |
| 5 | CAMK2G | CAMK2G Entrez,  Source | calcium/calmodulin-dependent protein kinase (CaM kinase) II gamma | 3255 | 0.075 | -0.0827 | No |
| 6 | OR12D3 | OR12D3 Entrez,  Source | olfactory receptor, family 12, subfamily D, member 3 | 3311 | 0.073 | -0.0787 | No |
| 7 | OR2A4 | OR2A4 Entrez,  Source | olfactory receptor, family 2, subfamily A, member 4 | 3635 | 0.064 | -0.0894 | No |
| 8 | CLCA2 | CLCA2 Entrez,  Source | chloride channel, calcium activated, family member 2 | 3646 | 0.064 | -0.0840 | No |
| 9 | PRKACB | PRKACB Entrez,  Source | protein kinase, cAMP-dependent, catalytic, beta | 3675 | 0.063 | -0.0796 | No |
| 10 | CALM1 | CALM1 Entrez,  Source | calmodulin 1 (phosphorylase kinase, delta) | 3706 | 0.062 | -0.0754 | No |
| 11 | OR2J3 | OR2J3 Entrez,  Source | olfactory receptor, family 2, subfamily J, member 3 | 3775 | 0.061 | -0.0733 | No |
| 12 | CALM2 | CALM2 Entrez,  Source | calmodulin 2 (phosphorylase kinase, delta) | 4026 | 0.055 | -0.0810 | No |
| 13 | OR2B2 | OR2B2 Entrez,  Source | olfactory receptor, family 2, subfamily B, member 2 | 4862 | 0.038 | -0.1204 | No |
| 14 | OR5J2 | OR5J2 Entrez,  Source | olfactory receptor, family 5, subfamily J, member 2 | 5016 | 0.035 | -0.1251 | No |
| 15 | OR2W1 | OR2W1 Entrez,  Source | olfactory receptor, family 2, subfamily W, member 1 | 5052 | 0.034 | -0.1237 | No |
| 16 | OR10J1 | OR10J1 Entrez,  Source | olfactory receptor, family 10, subfamily J, member 1 | 5169 | 0.032 | -0.1267 | No |
| 17 | OR6A2 | OR6A2 Entrez,  Source | olfactory receptor, family 6, subfamily A, member 2 | 5507 | 0.027 | -0.1415 | No |
| 18 | OR2H1 | OR2H1 Entrez,  Source | olfactory receptor, family 2, subfamily H, member 1 | 5591 | 0.026 | -0.1434 | No |
| 19 | GUCA1C | GUCA1C Entrez,  Source | guanylate cyclase activator 1C | 5883 | 0.021 | -0.1564 | No |
| 20 | OR51B4 | OR51B4 Entrez,  Source | olfactory receptor, family 51, subfamily B, member 4 | 6218 | 0.016 | -0.1720 | No |
| 21 | CLCA4 | CLCA4 Entrez,  Source | chloride channel, calcium activated, family member 4 | 7124 | 0.004 | -0.2182 | No |
| 22 | OR2S2 | OR2S2 Entrez,  Source | olfactory receptor, family 2, subfamily S, member 2 | 7181 | 0.003 | -0.2208 | No |
| 23 | OR3A2 | OR3A2 Entrez,  Source | olfactory receptor, family 3, subfamily A, member 2 | 7741 | -0.004 | -0.2492 | No |
| 24 | OR7A10 | OR7A10 Entrez,  Source | olfactory receptor, family 7, subfamily A, member 10 | 7862 | -0.005 | -0.2549 | No |
| 25 | OR3A1 | OR3A1 Entrez,  Source | olfactory receptor, family 3, subfamily A, member 1 | 8296 | -0.010 | -0.2762 | No |
| 26 | CNGA4 | CNGA4 Entrez,  Source | cyclic nucleotide gated channel alpha 4 | 8434 | -0.012 | -0.2821 | No |
| 27 | OR2C3 | OR2C3 Entrez,  Source | olfactory receptor, family 2, subfamily C, member 3 | 8506 | -0.013 | -0.2846 | No |
| 28 | PRKACA | PRKACA Entrez,  Source | protein kinase, cAMP-dependent, catalytic, alpha | 8583 | -0.014 | -0.2872 | No |
| 29 | OR51I1 | OR51I1 Entrez,  Source | olfactory receptor, family 51, subfamily I, member 1 | 8596 | -0.014 | -0.2866 | No |
| 30 | OR7C1 | OR7C1 Entrez,  Source | olfactory receptor, family 7, subfamily C, member 1 | 8889 | -0.017 | -0.3000 | No |
| 31 | OR7A5 | OR7A5 Entrez,  Source | olfactory receptor, family 7, subfamily A, member 5 | 9067 | -0.019 | -0.3073 | No |
| 32 | OR2L2 | OR2L2 Entrez,  Source | olfactory receptor, family 2, subfamily L, member 2 | 9356 | -0.023 | -0.3200 | No |
| 33 | OR7D2 | OR7D2 Entrez,  Source | olfactory receptor, family 7, subfamily D, member 2 | 9543 | -0.025 | -0.3273 | No |
| 34 | PDE1C | PDE1C Entrez,  Source | phosphodiesterase 1C, calmodulin-dependent 70kDa | 9718 | -0.027 | -0.3337 | No |
| 35 | OR1J2 | OR1J2 Entrez,  Source | olfactory receptor, family 1, subfamily J, member 2 | 9737 | -0.027 | -0.3321 | No |
| 36 | OR2C1 | OR2C1 Entrez,  Source | olfactory receptor, family 2, subfamily C, member 1 | 9775 | -0.027 | -0.3315 | No |
| 37 | OR8B8 | OR8B8 Entrez,  Source | olfactory receptor, family 8, subfamily B, member 8 | 10191 | -0.032 | -0.3499 | No |
| 38 | OR10A3 | OR10A3 Entrez,  Source | olfactory receptor, family 10, subfamily A, member 3 | 10380 | -0.034 | -0.3563 | No |
| 39 | OR52D1 | OR52D1 Entrez,  Source | olfactory receptor, family 52, subfamily D, member 1 | 10392 | -0.035 | -0.3537 | No |
| 40 | OR8G2 | OR8G2 Entrez,  Source | olfactory receptor, family 8, subfamily G, member 2 | 10663 | -0.038 | -0.3641 | No |
| 41 | OR8G1 | OR8G1 Entrez,  Source | olfactory receptor, family 8, subfamily G, member 1 | 10908 | -0.041 | -0.3728 | No |
| 42 | OR1J4 | OR1J4 Entrez,  Source | olfactory receptor, family 1, subfamily J, member 4 | 11018 | -0.042 | -0.3745 | No |
| 43 | OR2F1 | OR2F1 Entrez,  Source | olfactory receptor, family 2, subfamily F, member 1 | 11383 | -0.047 | -0.3889 | No |
| 44 | OR51M1 | OR51M1 Entrez,  Source | olfactory receptor, family 51, subfamily M, member 1 | 11437 | -0.047 | -0.3872 | No |
| 45 | OR5K1 | OR5K1 Entrez,  Source | olfactory receptor, family 5, subfamily K, member 1 | 11510 | -0.048 | -0.3865 | No |
| 46 | CAMK2A | CAMK2A Entrez,  Source | calcium/calmodulin-dependent protein kinase (CaM kinase) II alpha | 11694 | -0.050 | -0.3912 | No |
| 47 | CNGB1 | CNGB1 Entrez,  Source | cyclic nucleotide gated channel beta 1 | 11866 | -0.053 | -0.3951 | No |
| 48 | OR1F1 | OR1F1 Entrez,  Source | olfactory receptor, family 1, subfamily F, member 1 | 12036 | -0.055 | -0.3987 | No |
| 49 | OR2W3 | OR2W3 Entrez,  Source | olfactory receptor, family 2, subfamily W, member 3 | 12154 | -0.056 | -0.3996 | No |
| 50 | OR51B2 | OR51B2 Entrez,  Source | olfactory receptor, family 51, subfamily B, member 2 | 12426 | -0.059 | -0.4080 | No |
| 51 | ADRBK2 | ADRBK2 Entrez,  Source | adrenergic, beta, receptor kinase 2 | 12619 | -0.062 | -0.4122 | No |
| 52 | GUCA1A | GUCA1A Entrez,  Source | guanylate cyclase activator 1A (retina) | 12930 | -0.066 | -0.4220 | Yes |
| 53 | CAMK2B | CAMK2B Entrez,  Source | calcium/calmodulin-dependent protein kinase (CaM kinase) II beta | 12956 | -0.066 | -0.4172 | Yes |
| 54 | GUCA1B | GUCA1B Entrez,  Source | guanylate cyclase activator 1B (retina) | 12977 | -0.066 | -0.4121 | Yes |
| 55 | OR2K2 | OR2K2 Entrez,  Source | olfactory receptor, family 2, subfamily K, member 2 | 13157 | -0.069 | -0.4149 | Yes |
| 56 | OR51I2 | OR51I2 Entrez,  Source | olfactory receptor, family 51, subfamily I, member 2 | 13171 | -0.069 | -0.4092 | Yes |
| 57 | GUCY2D | GUCY2D Entrez,  Source | guanylate cyclase 2D, membrane (retina-specific) | 13321 | -0.071 | -0.4103 | Yes |
| 58 | OR10H3 | OR10H3 Entrez,  Source | olfactory receptor, family 10, subfamily H, member 3 | 13362 | -0.071 | -0.4058 | Yes |
| 59 | CNGA3 | CNGA3 Entrez,  Source | cyclic nucleotide gated channel alpha 3 | 13491 | -0.073 | -0.4056 | Yes |
| 60 | CLCA1 | CLCA1 Entrez,  Source | chloride channel, calcium activated, family member 1 | 13557 | -0.074 | -0.4021 | Yes |
| 61 | OR13C4 | OR13C4 Entrez,  Source | olfactory receptor, family 13, subfamily C, member 4 | 13876 | -0.078 | -0.4112 | Yes |
| 62 | OR7E24 | OR7E24 Entrez,  Source | olfactory receptor, family 7, subfamily E, member 24 | 13878 | -0.078 | -0.4040 | Yes |
| 63 | OR1A2 | OR1A2 Entrez,  Source | olfactory receptor, family 1, subfamily A, member 2 | 14086 | -0.081 | -0.4072 | Yes |
| 64 | OR12D2 | OR12D2 Entrez,  Source | olfactory receptor, family 12, subfamily D, member 2 | 14143 | -0.082 | -0.4025 | Yes |
| 65 | OR10H1 | OR10H1 Entrez,  Source | olfactory receptor, family 10, subfamily H, member 1 | 14188 | -0.082 | -0.3972 | Yes |
| 66 | OR5V1 | OR5V1 Entrez,  Source | olfactory receptor, family 5, subfamily V, member 1 | 14283 | -0.083 | -0.3943 | Yes |
| 67 | OR1C1 | OR1C1 Entrez,  Source | olfactory receptor, family 1, subfamily C, member 1 | 14470 | -0.086 | -0.3959 | Yes |
| 68 | PRKG1 | PRKG1 Entrez,  Source | protein kinase, cGMP-dependent, type I | 14798 | -0.091 | -0.4043 | Yes |
| 69 | OR4N4 | OR4N4 Entrez,  Source | olfactory receptor, family 4, subfamily N, member 4 | 14802 | -0.091 | -0.3961 | Yes |
| 70 | OR2M4 | OR2M4 Entrez,  Source | olfactory receptor, family 2, subfamily M, member 4 | 14867 | -0.092 | -0.3909 | Yes |
| 71 | PRKG2 | PRKG2 Entrez,  Source | protein kinase, cGMP-dependent, type II | 15127 | -0.096 | -0.3953 | Yes |
| 72 | OR51B5 | OR51B5 Entrez,  Source | olfactory receptor, family 51, subfamily B, member 5 | 15486 | -0.102 | -0.4043 | Yes |
| 73 | OR1I1 | OR1I1 Entrez,  Source | olfactory receptor, family 1, subfamily I, member 1 | 15513 | -0.102 | -0.3962 | Yes |
| 74 | CALML3 | CALML3 Entrez,  Source | calmodulin-like 3 | 15533 | -0.103 | -0.3877 | Yes |
| 75 | OR11A1 | OR11A1 Entrez,  Source | olfactory receptor, family 11, subfamily A, member 1 | 15556 | -0.103 | -0.3792 | Yes |
| 76 | OR10H2 | OR10H2 Entrez,  Source | olfactory receptor, family 10, subfamily H, member 2 | 15602 | -0.104 | -0.3719 | Yes |
| 77 | OR7C2 | OR7C2 Entrez,  Source | olfactory receptor, family 7, subfamily C, member 2 | 15640 | -0.104 | -0.3642 | Yes |
| 78 | CALML6 | CALML6 Entrez,  Source | calmodulin-like 6 | 15658 | -0.105 | -0.3553 | Yes |
| 79 | OR4D1 | OR4D1 Entrez,  Source | olfactory receptor, family 4, subfamily D, member 1 | 15671 | -0.105 | -0.3462 | Yes |
| 80 | OR3A3 | OR3A3 Entrez,  Source | olfactory receptor, family 3, subfamily A, member 3 | 15747 | -0.106 | -0.3402 | Yes |
| 81 | OR5P3 | OR5P3 Entrez,  Source | olfactory receptor, family 5, subfamily P, member 3 | 16082 | -0.113 | -0.3470 | Yes |
| 82 | OR5L2 | OR5L2 Entrez,  Source | olfactory receptor, family 5, subfamily L, member 2 | 16161 | -0.114 | -0.3404 | Yes |
| 83 | OR2F2 | OR2F2 Entrez,  Source | olfactory receptor, family 2, subfamily F, member 2 | 16184 | -0.115 | -0.3309 | Yes |
| 84 | OR4M2 | OR4M2 Entrez,  Source | olfactory receptor, family 4, subfamily M, member 2 | 16447 | -0.120 | -0.3333 | Yes |
| 85 | CALML5 | CALML5 Entrez,  Source | calmodulin-like 5 | 16652 | -0.124 | -0.3322 | Yes |
| 86 | OR6B1 | OR6B1 Entrez,  Source | olfactory receptor, family 6, subfamily B, member 1 | 16713 | -0.126 | -0.3237 | Yes |
| 87 | OR2H2 | OR2H2 Entrez,  Source | olfactory receptor, family 2, subfamily H, member 2 | 16838 | -0.129 | -0.3182 | Yes |
| 88 | OR51E2 | OR51E2 Entrez,  Source | olfactory receptor, family 51, subfamily E, member 2 | 16922 | -0.131 | -0.3103 | Yes |
| 89 | OR10C1 | OR10C1 Entrez,  Source | olfactory receptor, family 10, subfamily C, member 1 | 16976 | -0.132 | -0.3008 | Yes |
| 90 | ARRB2 | ARRB2 Entrez,  Source | arrestin, beta 2 | 17265 | -0.141 | -0.3026 | Yes |
| 91 | OR5I1 | OR5I1 Entrez,  Source | olfactory receptor, family 5, subfamily I, member 1 | 17397 | -0.145 | -0.2959 | Yes |
| 92 | OR1Q1 | OR1Q1 Entrez,  Source | olfactory receptor, family 1, subfamily Q, member 1 | 17445 | -0.146 | -0.2848 | Yes |
| 93 | OR1D2 | OR1D2 Entrez,  Source | olfactory receptor, family 1, subfamily D, member 2 | 17465 | -0.147 | -0.2721 | Yes |
| 94 | OR2J2 | OR2J2 Entrez,  Source | olfactory receptor, family 2, subfamily J, member 2 | 17647 | -0.153 | -0.2673 | Yes |
| 95 | ADCY3 | ADCY3 Entrez,  Source | adenylate cyclase 3 | 17753 | -0.156 | -0.2582 | Yes |
| 96 | OR10A5 | OR10A5 Entrez,  Source | olfactory receptor, family 10, subfamily A, member 5 | 17760 | -0.157 | -0.2440 | Yes |
| 97 | OR51E1 | OR51E1 Entrez,  Source | olfactory receptor, family 51, subfamily E, member 1 | 17893 | -0.162 | -0.2357 | Yes |
| 98 | OR52A1 | OR52A1 Entrez,  Source | olfactory receptor, family 52, subfamily A, member 1 | 17912 | -0.163 | -0.2216 | Yes |
| 99 | OR2L13 | OR2L13 Entrez,  Source | olfactory receptor, family 2, subfamily L, member 13 | 18070 | -0.169 | -0.2140 | Yes |
| 100 | OR2B3 | OR2B3 Entrez,  Source | olfactory receptor, family 2, subfamily B, member 3 | 18078 | -0.170 | -0.1986 | Yes |
| 101 | OR4D2 | OR4D2 Entrez,  Source | olfactory receptor, family 4, subfamily D, member 2 | 18106 | -0.171 | -0.1842 | Yes |
| 102 | OR51B6 | OR51B6 Entrez,  Source | olfactory receptor, family 51, subfamily B, member 6 | 18166 | -0.174 | -0.1711 | Yes |
| 103 | PRKACG | PRKACG Entrez,  Source | protein kinase, cAMP-dependent, catalytic, gamma | 18249 | -0.178 | -0.1588 | Yes |
| 104 | OR8D1 | OR8D1 Entrez,  Source | olfactory receptor, family 8, subfamily D, member 1 | 18398 | -0.187 | -0.1491 | Yes |
| 105 | OR10A4 | OR10A4 Entrez,  Source | olfactory receptor, family 10, subfamily A, member 4 | 18472 | -0.192 | -0.1350 | Yes |
| 106 | OR1A1 | OR1A1 Entrez,  Source | olfactory receptor, family 1, subfamily A, member 1 | 18523 | -0.195 | -0.1196 | Yes |
| 107 | OR1G1 | OR1G1 Entrez,  Source | olfactory receptor, family 1, subfamily G, member 1 | 18855 | -0.224 | -0.1159 | Yes |
| 108 | OR5P2 | OR5P2 Entrez,  Source | olfactory receptor, family 5, subfamily P, member 2 | 18876 | -0.226 | -0.0960 | Yes |
| 109 | OR8D2 | OR8D2 Entrez,  Source | olfactory receptor, family 8, subfamily D, member 2 | 18887 | -0.227 | -0.0754 | Yes |
| 110 | PDC | PDC Entrez,  Source | phosducin | 18972 | -0.239 | -0.0576 | Yes |
| 111 | OR2B6 | OR2B6 Entrez,  Source | olfactory receptor, family 2, subfamily B, member 6 | 19344 | -0.356 | -0.0438 | Yes |
| 112 | GNAL | GNAL Entrez,  Source | guanine nucleotide binding protein (G protein), alpha activating activity polypeptide, olfactory type | 19508 | -0.590 | 0.0025 | Yes |
Table: GSEA details [plain text format]

  

Fig 2: KEGG\_OLFACTORY\_TRANSDUCTION      
 Blue-Pink O' Gram in the Space of the Analyzed GeneSet

  

Fig 3: KEGG\_OLFACTORY\_TRANSDUCTION: Random ES distribution      
 Gene set null distribution of ES for **KEGG\_OLFACTORY\_TRANSDUCTION**

  
