# Supplementary material for: Comprehensive analysis of β-catenin target genes in colorectal carcinoma cell lines with deregulated Wnt/β-catenin signaling
Source: BMC Genomics. 2014 Jan 28;15:74. doi: 10.1186/1471-2164-15-74 (PMC3909937; doi:10.1186/1471-2164-15-74)
Supplement: Additional file 5 — GSEA analysis using the KEGG pathway database. This zipped file contains confirming data of the GSEA analysis. The names of the directories containing the files were composed of the term ‘GSEA’, the name of the cell line, e.g. DLD1, SW480, or LS174T, and the pathway database (KEGG). Please use a web browser to view the files with the name ‘index.html’ in the corresponding directories to start exploring the data. [file 1471-2164-15-74-S5.zip › GSEA KEGG SW480/KEGG_PANCREATIC_CANCER.html]

Details for gene set KEGG\_PANCREATIC\_CANCER[GSEA]

|  || Dataset | SW480\_collapsed\_to\_symbols.class.cls#b\_versus\_bg.class.cls#b\_versus\_bg\_repos |
| Phenotype | class.cls#b\_versus\_bg\_repos |
| Upregulated in class | 1 |
| GeneSet | KEGG\_PANCREATIC\_CANCER |
| Enrichment Score (ES) | 0.41763455 |
| Normalized Enrichment Score (NES) | 1.5728663 |
| Nominal p-value | 0.017632242 |
| FDR q-value | 0.11047784 |
| FWER p-Value | 0.738 |
Table: GSEA Results Summary

  

Fig 1: Enrichment plot: KEGG\_PANCREATIC\_CANCER      
 Profile of the Running ES Score & Positions of GeneSet Members on the Rank Ordered List

  

| PROBE | GENE SYMBOL | GENE\_TITLE | RANK IN GENE LIST | RANK METRIC SCORE | RUNNING ES | CORE ENRICHMENT || 1 | RAC2 | RAC2 Entrez,  Source | ras-related C3 botulinum toxin substrate 2 (rho family, small GTP binding protein Rac2) | 119 | 0.477 | 0.0753 | Yes |
| 2 | TGFA | TGFA Entrez,  Source | transforming growth factor, alpha | 398 | 0.304 | 0.1129 | Yes |
| 3 | TGFB2 | TGFB2 Entrez,  Source | transforming growth factor, beta 2 | 587 | 0.253 | 0.1464 | Yes |
| 4 | EGFR | EGFR Entrez,  Source | epidermal growth factor receptor (erythroblastic leukemia viral (v-erb-b) oncogene homolog, avian) | 652 | 0.241 | 0.1842 | Yes |
| 5 | IKBKB | IKBKB Entrez,  Source | inhibitor of kappa light polypeptide gene enhancer in B-cells, kinase beta | 860 | 0.208 | 0.2091 | Yes |
| 6 | AKT3 | AKT3 Entrez,  Source | v-akt murine thymoma viral oncogene homolog 3 (protein kinase B, gamma) | 870 | 0.207 | 0.2440 | Yes |
| 7 | TGFBR2 | TGFBR2 Entrez,  Source | transforming growth factor, beta receptor II (70/80kDa) | 1151 | 0.174 | 0.2593 | Yes |
| 8 | IKBKG | IKBKG Entrez,  Source | inhibitor of kappa light polypeptide gene enhancer in B-cells, kinase gamma | 1169 | 0.172 | 0.2878 | Yes |
| 9 | BAD | BAD Entrez,  Source | BCL2-antagonist of cell death | 1250 | 0.166 | 0.3121 | Yes |
| 10 | RB1 | RB1 Entrez,  Source | retinoblastoma 1 (including osteosarcoma) | 1421 | 0.153 | 0.3294 | Yes |
| 11 | KRAS | KRAS Entrez,  Source | v-Ki-ras2 Kirsten rat sarcoma viral oncogene homolog | 1429 | 0.153 | 0.3551 | Yes |
| 12 | BRCA2 | BRCA2 Entrez,  Source | breast cancer 2, early onset | 1691 | 0.134 | 0.3646 | Yes |
| 13 | PLD1 | PLD1 Entrez,  Source | phospholipase D1, phosphatidylcholine-specific | 1765 | 0.131 | 0.3833 | Yes |
| 14 | BRAF | BRAF Entrez,  Source | v-raf murine sarcoma viral oncogene homolog B1 | 2414 | 0.103 | 0.3676 | Yes |
| 15 | STAT1 | STAT1 Entrez,  Source | signal transducer and activator of transcription 1, 91kDa | 2608 | 0.095 | 0.3739 | Yes |
| 16 | JAK1 | JAK1 Entrez,  Source | Janus kinase 1 (a protein tyrosine kinase) | 3124 | 0.079 | 0.3609 | Yes |
| 17 | RAD51 | RAD51 Entrez,  Source | RAD51 homolog (RecA homolog, E. coli) (S. cerevisiae) | 3165 | 0.077 | 0.3721 | Yes |
| 18 | PIK3CB | PIK3CB Entrez,  Source | phosphoinositide-3-kinase, catalytic, beta polypeptide | 3241 | 0.075 | 0.3810 | Yes |
| 19 | CDKN2A | CDKN2A Entrez,  Source | cyclin-dependent kinase inhibitor 2A (melanoma, p16, inhibits CDK4) | 3246 | 0.075 | 0.3935 | Yes |
| 20 | CDC42 | CDC42 Entrez,  Source | cell division cycle 42 (GTP binding protein, 25kDa) | 3251 | 0.075 | 0.4061 | Yes |
| 21 | AKT2 | AKT2 Entrez,  Source | v-akt murine thymoma viral oncogene homolog 2 | 3525 | 0.067 | 0.4036 | Yes |
| 22 | CDK6 | CDK6 Entrez,  Source | cyclin-dependent kinase 6 | 3698 | 0.062 | 0.4054 | Yes |
| 23 | SMAD3 | SMAD3 Entrez,  Source | SMAD, mothers against DPP homolog 3 (Drosophila) | 3719 | 0.062 | 0.4149 | Yes |
| 24 | CHUK | CHUK Entrez,  Source | conserved helix-loop-helix ubiquitous kinase | 3863 | 0.059 | 0.4176 | Yes |
| 25 | RALB | RALB Entrez,  Source | v-ral simian leukemia viral oncogene homolog B (ras related; GTP binding protein) | 4172 | 0.052 | 0.4107 | No |
| 26 | MAPK8 | MAPK8 Entrez,  Source | mitogen-activated protein kinase 8 | 4307 | 0.049 | 0.4121 | No |
| 27 | RELA | RELA Entrez,  Source | v-rel reticuloendotheliosis viral oncogene homolog A, nuclear factor of kappa light polypeptide gene enhancer in B-cells 3, p65 (avian) | 5505 | 0.027 | 0.3553 | No |
| 28 | E2F2 | E2F2 Entrez,  Source | E2F transcription factor 2 | 5551 | 0.026 | 0.3575 | No |
| 29 | ARAF | ARAF Entrez,  Source | v-raf murine sarcoma 3611 viral oncogene homolog | 6077 | 0.018 | 0.3337 | No |
| 30 | AKT1 | AKT1 Entrez,  Source | v-akt murine thymoma viral oncogene homolog 1 | 6295 | 0.015 | 0.3252 | No |
| 31 | MAP2K1 | MAP2K1 Entrez,  Source | mitogen-activated protein kinase kinase 1 | 6310 | 0.015 | 0.3270 | No |
| 32 | PIK3R1 | PIK3R1 Entrez,  Source | phosphoinositide-3-kinase, regulatory subunit 1 (p85 alpha) | 6402 | 0.014 | 0.3247 | No |
| 33 | VEGFB | VEGFB Entrez,  Source | vascular endothelial growth factor B | 6773 | 0.009 | 0.3072 | No |
| 34 | STAT3 | STAT3 Entrez,  Source | signal transducer and activator of transcription 3 (acute-phase response factor) | 7165 | 0.003 | 0.2877 | No |
| 35 | CCND1 | CCND1 Entrez,  Source | cyclin D1 | 7482 | -0.000 | 0.2715 | No |
| 36 | PGF | PGF Entrez,  Source | placental growth factor, vascular endothelial growth factor-related protein | 7746 | -0.004 | 0.2587 | No |
| 37 | BCL2L1 | BCL2L1 Entrez,  Source | BCL2-like 1 | 7937 | -0.006 | 0.2500 | No |
| 38 | CDK4 | CDK4 Entrez,  Source | cyclin-dependent kinase 4 | 8358 | -0.011 | 0.2303 | No |
| 39 | SMAD2 | SMAD2 Entrez,  Source | SMAD, mothers against DPP homolog 2 (Drosophila) | 8435 | -0.012 | 0.2285 | No |
| 40 | MAPK9 | MAPK9 Entrez,  Source | mitogen-activated protein kinase 9 | 8669 | -0.015 | 0.2190 | No |
| 41 | RAC3 | RAC3 Entrez,  Source | ras-related C3 botulinum toxin substrate 3 (rho family, small GTP binding protein Rac3) | 8838 | -0.017 | 0.2132 | No |
| 42 | NFKB1 | NFKB1 Entrez,  Source | nuclear factor of kappa light polypeptide gene enhancer in B-cells 1 (p105) | 8939 | -0.018 | 0.2112 | No |
| 43 | RAF1 | RAF1 Entrez,  Source | v-raf-1 murine leukemia viral oncogene homolog 1 | 9036 | -0.019 | 0.2095 | No |
| 44 | RAC1 | RAC1 Entrez,  Source | ras-related C3 botulinum toxin substrate 1 (rho family, small GTP binding protein Rac1) | 9406 | -0.023 | 0.1945 | No |
| 45 | SMAD4 | SMAD4 Entrez,  Source | SMAD, mothers against DPP homolog 4 (Drosophila) | 9636 | -0.026 | 0.1872 | No |
| 46 | MAPK1 | MAPK1 Entrez,  Source | mitogen-activated protein kinase 1 | 10004 | -0.030 | 0.1735 | No |
| 47 | PIK3R3 | PIK3R3 Entrez,  Source | phosphoinositide-3-kinase, regulatory subunit 3 (p55, gamma) | 10110 | -0.031 | 0.1734 | No |
| 48 | PIK3R5 | PIK3R5 Entrez,  Source | phosphoinositide-3-kinase, regulatory subunit 5, p101 | 10124 | -0.031 | 0.1781 | No |
| 49 | MAPK3 | MAPK3 Entrez,  Source | mitogen-activated protein kinase 3 | 10580 | -0.037 | 0.1610 | No |
| 50 | PIK3CG | PIK3CG Entrez,  Source | phosphoinositide-3-kinase, catalytic, gamma polypeptide | 10795 | -0.039 | 0.1568 | No |
| 51 | E2F1 | E2F1 Entrez,  Source | E2F transcription factor 1 | 10826 | -0.040 | 0.1621 | No |
| 52 | ERBB2 | ERBB2 Entrez,  Source | v-erb-b2 erythroblastic leukemia viral oncogene homolog 2, neuro/glioblastoma derived oncogene homolog (avian) | 10899 | -0.041 | 0.1653 | No |
| 53 | E2F3 | E2F3 Entrez,  Source | E2F transcription factor 3 | 11523 | -0.048 | 0.1416 | No |
| 54 | PIK3CA | PIK3CA Entrez,  Source | phosphoinositide-3-kinase, catalytic, alpha polypeptide | 11588 | -0.049 | 0.1468 | No |
| 55 | ARHGEF6 | ARHGEF6 Entrez,  Source | Rac/Cdc42 guanine nucleotide exchange factor (GEF) 6 | 12491 | -0.060 | 0.1108 | No |
| 56 | MAPK10 | MAPK10 Entrez,  Source | mitogen-activated protein kinase 10 | 12600 | -0.062 | 0.1157 | No |
| 57 | TGFB1 | TGFB1 Entrez,  Source | transforming growth factor, beta 1 (Camurati-Engelmann disease) | 13436 | -0.072 | 0.0852 | No |
| 58 | TGFBR1 | TGFBR1 Entrez,  Source | transforming growth factor, beta receptor I (activin A receptor type II-like kinase, 53kDa) | 14193 | -0.082 | 0.0605 | No |
| 59 | RALA | RALA Entrez,  Source | v-ral simian leukemia viral oncogene homolog A (ras related) | 14379 | -0.084 | 0.0654 | No |
| 60 | PIK3R2 | PIK3R2 Entrez,  Source | phosphoinositide-3-kinase, regulatory subunit 2 (p85 beta) | 14488 | -0.086 | 0.0745 | No |
| 61 | RALGDS | RALGDS Entrez,  Source | ral guanine nucleotide dissociation stimulator | 14647 | -0.088 | 0.0815 | No |
| 62 | CASP9 | CASP9 Entrez,  Source | caspase 9, apoptosis-related cysteine peptidase | 15133 | -0.096 | 0.0730 | No |
| 63 | TGFB3 | TGFB3 Entrez,  Source | transforming growth factor, beta 3 | 15224 | -0.098 | 0.0851 | No |
| 64 | TP53 | TP53 Entrez,  Source | tumor protein p53 (Li-Fraumeni syndrome) | 15395 | -0.100 | 0.0935 | No |
| 65 | RALBP1 | RALBP1 Entrez,  Source | ralA binding protein 1 | 16458 | -0.120 | 0.0595 | No |
| 66 | PIK3CD | PIK3CD Entrez,  Source | phosphoinositide-3-kinase, catalytic, delta polypeptide | 16633 | -0.124 | 0.0717 | No |
| 67 | FIGF | FIGF Entrez,  Source | c-fos induced growth factor (vascular endothelial growth factor D) | 16676 | -0.125 | 0.0908 | No |
| 68 | EGF | EGF Entrez,  Source | epidermal growth factor (beta-urogastrone) | 17577 | -0.151 | 0.0704 | No |
| 69 | VEGFC | VEGFC Entrez,  Source | vascular endothelial growth factor C | 18328 | -0.182 | 0.0630 | No |
Table: GSEA details [plain text format]

  

Fig 2: KEGG\_PANCREATIC\_CANCER      
 Blue-Pink O' Gram in the Space of the Analyzed GeneSet

  

Fig 3: KEGG\_PANCREATIC\_CANCER: Random ES distribution      
 Gene set null distribution of ES for **KEGG\_PANCREATIC\_CANCER**

  
