# Supplementary material for: Comprehensive analysis of β-catenin target genes in colorectal carcinoma cell lines with deregulated Wnt/β-catenin signaling
Source: BMC Genomics. 2014 Jan 28;15:74. doi: 10.1186/1471-2164-15-74 (PMC3909937; doi:10.1186/1471-2164-15-74)
Supplement: Additional file 5 — GSEA analysis using the KEGG pathway database. This zipped file contains confirming data of the GSEA analysis. The names of the directories containing the files were composed of the term ‘GSEA’, the name of the cell line, e.g. DLD1, SW480, or LS174T, and the pathway database (KEGG). Please use a web browser to view the files with the name ‘index.html’ in the corresponding directories to start exploring the data. [file 1471-2164-15-74-S5.zip › GSEA KEGG SW480/KEGG_PATHOGENIC_ESCHERICHIA_COLI_INFECTION.html]

Details for gene set KEGG\_PATHOGENIC\_ESCHERICHIA\_COLI\_INFECTION[GSEA]

|  || Dataset | SW480\_collapsed\_to\_symbols.class.cls#b\_versus\_bg.class.cls#b\_versus\_bg\_repos |
| Phenotype | class.cls#b\_versus\_bg\_repos |
| Upregulated in class | 1 |
| GeneSet | KEGG\_PATHOGENIC\_ESCHERICHIA\_COLI\_INFECTION |
| Enrichment Score (ES) | 0.52517116 |
| Normalized Enrichment Score (NES) | 1.7969385 |
| Nominal p-value | 0.0022883294 |
| FDR q-value | 0.040950265 |
| FWER p-Value | 0.183 |
Table: GSEA Results Summary

  

Fig 1: Enrichment plot: KEGG\_PATHOGENIC\_ESCHERICHIA\_COLI\_INFECTION      
 Profile of the Running ES Score & Positions of GeneSet Members on the Rank Ordered List

  

| PROBE | GENE SYMBOL | GENE\_TITLE | RANK IN GENE LIST | RANK METRIC SCORE | RUNNING ES | CORE ENRICHMENT || 1 | LY96 | LY96 Entrez,  Source | lymphocyte antigen 96 | 24 | 0.777 | 0.1933 | Yes |
| 2 | TLR4 | TLR4 Entrez,  Source | toll-like receptor 4 | 87 | 0.566 | 0.3316 | Yes |
| 3 | CLDN1 | CLDN1 Entrez,  Source | claudin 1 | 600 | 0.251 | 0.3683 | Yes |
| 4 | CD14 | CD14 Entrez,  Source | CD14 molecule | 1287 | 0.163 | 0.3740 | Yes |
| 5 | YWHAZ | YWHAZ Entrez,  Source | tyrosine 3-monooxygenase/tryptophan 5-monooxygenase activation protein, zeta polypeptide | 1398 | 0.155 | 0.4071 | Yes |
| 6 | PRKCA | PRKCA Entrez,  Source | protein kinase C, alpha | 1907 | 0.125 | 0.4124 | Yes |
| 7 | WASL | WASL Entrez,  Source | Wiskott-Aldrich syndrome-like | 2317 | 0.107 | 0.4182 | Yes |
| 8 | ITGB1 | ITGB1 Entrez,  Source | integrin, beta 1 (fibronectin receptor, beta polypeptide, antigen CD29 includes MDF2, MSK12) | 2321 | 0.107 | 0.4448 | Yes |
| 9 | ROCK2 | ROCK2 Entrez,  Source | Rho-associated, coiled-coil containing protein kinase 2 | 2416 | 0.103 | 0.4657 | Yes |
| 10 | CTTN | CTTN Entrez,  Source | cortactin | 2886 | 0.086 | 0.4632 | Yes |
| 11 | CDC42 | CDC42 Entrez,  Source | cell division cycle 42 (GTP binding protein, 25kDa) | 3251 | 0.075 | 0.4633 | Yes |
| 12 | NCK1 | NCK1 Entrez,  Source | NCK adaptor protein 1 | 3298 | 0.073 | 0.4793 | Yes |
| 13 | RHOA | RHOA Entrez,  Source | ras homolog gene family, member A | 3339 | 0.072 | 0.4954 | Yes |
| 14 | ARPC3 | ARPC3 Entrez,  Source | actin related protein 2/3 complex, subunit 3, 21kDa | 3409 | 0.071 | 0.5095 | Yes |
| 15 | NCK2 | NCK2 Entrez,  Source | NCK adaptor protein 2 | 3779 | 0.061 | 0.5058 | Yes |
| 16 | ARPC1A | ARPC1A Entrez,  Source | actin related protein 2/3 complex, subunit 1A, 41kDa | 3974 | 0.056 | 0.5100 | Yes |
| 17 | ARHGEF2 | ARHGEF2 Entrez,  Source | rho/rac guanine nucleotide exchange factor (GEF) 2 | 4324 | 0.049 | 0.5042 | Yes |
| 18 | TUBB2A | TUBB2A Entrez,  Source | tubulin, beta 2A | 4442 | 0.046 | 0.5098 | Yes |
| 19 | ARPC2 | ARPC2 Entrez,  Source | actin related protein 2/3 complex, subunit 2, 34kDa | 4472 | 0.046 | 0.5197 | Yes |
| 20 | CDH1 | CDH1 Entrez,  Source | cadherin 1, type 1, E-cadherin (epithelial) | 4578 | 0.043 | 0.5252 | Yes |
| 21 | KRT18 | KRT18 Entrez,  Source | keratin 18 | 5489 | 0.027 | 0.4854 | No |
| 22 | HCLS1 | HCLS1 Entrez,  Source | hematopoietic cell-specific Lyn substrate 1 | 5511 | 0.027 | 0.4910 | No |
| 23 | TUBB | TUBB Entrez,  Source | tubulin, beta | 5864 | 0.022 | 0.4784 | No |
| 24 | ARPC5 | ARPC5 Entrez,  Source | actin related protein 2/3 complex, subunit 5, 16kDa | 6167 | 0.017 | 0.4672 | No |
| 25 | TUBB2B | TUBB2B Entrez,  Source | tubulin, beta 2B | 6174 | 0.017 | 0.4712 | No |
| 26 | ACTB | ACTB Entrez,  Source | actin, beta | 6245 | 0.016 | 0.4716 | No |
| 27 | ARPC4 | ARPC4 Entrez,  Source | actin related protein 2/3 complex, subunit 4, 20kDa | 6332 | 0.015 | 0.4708 | No |
| 28 | ACTG1 | ACTG1 Entrez,  Source | actin, gamma 1 | 6584 | 0.011 | 0.4608 | No |
| 29 | YWHAQ | YWHAQ Entrez,  Source | tyrosine 3-monooxygenase/tryptophan 5-monooxygenase activation protein, theta polypeptide | 7449 | 0.000 | 0.4165 | No |
| 30 | TUBAL3 | TUBAL3 Entrez,  Source | tubulin, alpha-like 3 | 7562 | -0.001 | 0.4111 | No |
| 31 | TUBB3 | TUBB3 Entrez,  Source | tubulin, beta 3 | 7806 | -0.005 | 0.3999 | No |
| 32 | TUBA8 | TUBA8 Entrez,  Source | tubulin, alpha 8 | 9372 | -0.023 | 0.3254 | No |
| 33 | TUBB6 | TUBB6 Entrez,  Source | tubulin, beta 6 | 9400 | -0.023 | 0.3298 | No |
| 34 | WAS | WAS Entrez,  Source | Wiskott-Aldrich syndrome (eczema-thrombocytopenia) | 10187 | -0.032 | 0.2976 | No |
| 35 | ARPC5L | ARPC5L Entrez,  Source | actin related protein 2/3 complex, subunit 5-like | 10423 | -0.035 | 0.2942 | No |
| 36 | ROCK1 | ROCK1 Entrez,  Source | Rho-associated, coiled-coil containing protein kinase 1 | 11335 | -0.046 | 0.2591 | No |
| 37 | NCL | NCL Entrez,  Source | nucleolin | 11653 | -0.050 | 0.2554 | No |
| 38 | TLR5 | TLR5 Entrez,  Source | toll-like receptor 5 | 11772 | -0.051 | 0.2622 | No |
| 39 | FYN | FYN Entrez,  Source | FYN oncogene related to SRC, FGR, YES | 14954 | -0.093 | 0.1225 | No |
| 40 | ABL1 | ABL1 Entrez,  Source | v-abl Abelson murine leukemia viral oncogene homolog 1 | 15414 | -0.100 | 0.1241 | No |
| 41 | TUBB1 | TUBB1 Entrez,  Source | tubulin, beta 1 | 16262 | -0.116 | 0.1098 | No |
| 42 | CTNNB1 | CTNNB1 Entrez,  Source | catenin (cadherin-associated protein), beta 1, 88kDa | 18943 | -0.235 | 0.0314 | No |
Table: GSEA details [plain text format]

  

Fig 2: KEGG\_PATHOGENIC\_ESCHERICHIA\_COLI\_INFECTION      
 Blue-Pink O' Gram in the Space of the Analyzed GeneSet

  

Fig 3: KEGG\_PATHOGENIC\_ESCHERICHIA\_COLI\_INFECTION: Random ES distribution      
 Gene set null distribution of ES for **KEGG\_PATHOGENIC\_ESCHERICHIA\_COLI\_INFECTION**

  
