# Supplementary material for: Comprehensive analysis of β-catenin target genes in colorectal carcinoma cell lines with deregulated Wnt/β-catenin signaling
Source: BMC Genomics. 2014 Jan 28;15:74. doi: 10.1186/1471-2164-15-74 (PMC3909937; doi:10.1186/1471-2164-15-74)
Supplement: Additional file 5 — GSEA analysis using the KEGG pathway database. This zipped file contains confirming data of the GSEA analysis. The names of the directories containing the files were composed of the term ‘GSEA’, the name of the cell line, e.g. DLD1, SW480, or LS174T, and the pathway database (KEGG). Please use a web browser to view the files with the name ‘index.html’ in the corresponding directories to start exploring the data. [file 1471-2164-15-74-S5.zip › GSEA KEGG SW480/KEGG_PROTEASOME.html]

Details for gene set KEGG\_PROTEASOME[GSEA]

|  || Dataset | SW480\_collapsed\_to\_symbols.class.cls#b\_versus\_bg.class.cls#b\_versus\_bg\_repos |
| Phenotype | class.cls#b\_versus\_bg\_repos |
| Upregulated in class | 1 |
| GeneSet | KEGG\_PROTEASOME |
| Enrichment Score (ES) | 0.530827 |
| Normalized Enrichment Score (NES) | 1.7975047 |
| Nominal p-value | 0.0025062656 |
| FDR q-value | 0.046617676 |
| FWER p-Value | 0.178 |
Table: GSEA Results Summary

  

Fig 1: Enrichment plot: KEGG\_PROTEASOME      
 Profile of the Running ES Score & Positions of GeneSet Members on the Rank Ordered List

  

| PROBE | GENE SYMBOL | GENE\_TITLE | RANK IN GENE LIST | RANK METRIC SCORE | RUNNING ES | CORE ENRICHMENT || 1 | PSMB9 | PSMB9 Entrez,  Source | proteasome (prosome, macropain) subunit, beta type, 9 (large multifunctional peptidase 2) | 238 | 0.376 | 0.1727 | Yes |
| 2 | PSMB10 | PSMB10 Entrez,  Source | proteasome (prosome, macropain) subunit, beta type, 10 | 330 | 0.327 | 0.3284 | Yes |
| 3 | PSMB8 | PSMB8 Entrez,  Source | proteasome (prosome, macropain) subunit, beta type, 8 (large multifunctional peptidase 7) | 1315 | 0.161 | 0.3571 | Yes |
| 4 | PSME1 | PSME1 Entrez,  Source | proteasome (prosome, macropain) activator subunit 1 (PA28 alpha) | 2710 | 0.092 | 0.3307 | Yes |
| 5 | PSMA8 | PSMA8 Entrez,  Source | proteasome (prosome, macropain) subunit, alpha type, 8 | 2808 | 0.089 | 0.3693 | Yes |
| 6 | PSME2 | PSME2 Entrez,  Source | proteasome (prosome, macropain) activator subunit 2 (PA28 beta) | 3366 | 0.072 | 0.3760 | Yes |
| 7 | PSMB4 | PSMB4 Entrez,  Source | proteasome (prosome, macropain) subunit, beta type, 4 | 3439 | 0.070 | 0.4066 | Yes |
| 8 | PSMC5 | PSMC5 Entrez,  Source | proteasome (prosome, macropain) 26S subunit, ATPase, 5 | 3618 | 0.065 | 0.4291 | Yes |
| 9 | PSMD7 | PSMD7 Entrez,  Source | proteasome (prosome, macropain) 26S subunit, non-ATPase, 7 (Mov34 homolog) | 4150 | 0.052 | 0.4276 | Yes |
| 10 | PSMC3 | PSMC3 Entrez,  Source | proteasome (prosome, macropain) 26S subunit, ATPase, 3 | 4346 | 0.048 | 0.4412 | Yes |
| 11 | PSMD13 | PSMD13 Entrez,  Source | proteasome (prosome, macropain) 26S subunit, non-ATPase, 13 | 4505 | 0.045 | 0.4551 | Yes |
| 12 | PSMB2 | PSMB2 Entrez,  Source | proteasome (prosome, macropain) subunit, beta type, 2 | 4748 | 0.040 | 0.4625 | Yes |
| 13 | PSMB1 | PSMB1 Entrez,  Source | proteasome (prosome, macropain) subunit, beta type, 1 | 4874 | 0.038 | 0.4747 | Yes |
| 14 | PSMD1 | PSMD1 Entrez,  Source | proteasome (prosome, macropain) 26S subunit, non-ATPase, 1 | 4995 | 0.035 | 0.4858 | Yes |
| 15 | PSMA4 | PSMA4 Entrez,  Source | proteasome (prosome, macropain) subunit, alpha type, 4 | 5058 | 0.034 | 0.4995 | Yes |
| 16 | PSMA5 | PSMA5 Entrez,  Source | proteasome (prosome, macropain) subunit, alpha type, 5 | 5416 | 0.028 | 0.4952 | Yes |
| 17 | PSMC2 | PSMC2 Entrez,  Source | proteasome (prosome, macropain) 26S subunit, ATPase, 2 | 5536 | 0.027 | 0.5021 | Yes |
| 18 | PSMA2 | PSMA2 Entrez,  Source | proteasome (prosome, macropain) subunit, alpha type, 2 | 5724 | 0.024 | 0.5041 | Yes |
| 19 | PSMA3 | PSMA3 Entrez,  Source | proteasome (prosome, macropain) subunit, alpha type, 3 | 5777 | 0.023 | 0.5127 | Yes |
| 20 | PSMA6 | PSMA6 Entrez,  Source | proteasome (prosome, macropain) subunit, alpha type, 6 | 5839 | 0.022 | 0.5205 | Yes |
| 21 | PSMA1 | PSMA1 Entrez,  Source | proteasome (prosome, macropain) subunit, alpha type, 1 | 6041 | 0.019 | 0.5194 | Yes |
| 22 | PSMD14 | PSMD14 Entrez,  Source | proteasome (prosome, macropain) 26S subunit, non-ATPase, 14 | 6042 | 0.019 | 0.5287 | Yes |
| 23 | PSMC1 | PSMC1 Entrez,  Source | proteasome (prosome, macropain) 26S subunit, ATPase, 1 | 6165 | 0.017 | 0.5308 | Yes |
| 24 | PSMC6 | PSMC6 Entrez,  Source | proteasome (prosome, macropain) 26S subunit, ATPase, 6 | 6378 | 0.014 | 0.5268 | No |
| 25 | PSMD11 | PSMD11 Entrez,  Source | proteasome (prosome, macropain) 26S subunit, non-ATPase, 11 | 6593 | 0.011 | 0.5214 | No |
| 26 | POMP | POMP Entrez,  Source | proteasome maturation protein | 6606 | 0.011 | 0.5262 | No |
| 27 | SHFM1 | SHFM1 Entrez,  Source | split hand/foot malformation (ectrodactyly) type 1 | 6625 | 0.011 | 0.5306 | No |
| 28 | IFNG | IFNG Entrez,  Source | interferon, gamma | 6964 | 0.006 | 0.5164 | No |
| 29 | PSMB6 | PSMB6 Entrez,  Source | proteasome (prosome, macropain) subunit, beta type, 6 | 7634 | -0.002 | 0.4832 | No |
| 30 | PSMD6 | PSMD6 Entrez,  Source | proteasome (prosome, macropain) 26S subunit, non-ATPase, 6 | 7660 | -0.003 | 0.4832 | No |
| 31 | PSMB7 | PSMB7 Entrez,  Source | proteasome (prosome, macropain) subunit, beta type, 7 | 7828 | -0.005 | 0.4771 | No |
| 32 | PSMB3 | PSMB3 Entrez,  Source | proteasome (prosome, macropain) subunit, beta type, 3 | 7947 | -0.006 | 0.4741 | No |
| 33 | PSMD2 | PSMD2 Entrez,  Source | proteasome (prosome, macropain) 26S subunit, non-ATPase, 2 | 8019 | -0.007 | 0.4740 | No |
| 34 | PSMD12 | PSMD12 Entrez,  Source | proteasome (prosome, macropain) 26S subunit, non-ATPase, 12 | 8044 | -0.007 | 0.4764 | No |
| 35 | PSMD8 | PSMD8 Entrez,  Source | proteasome (prosome, macropain) 26S subunit, non-ATPase, 8 | 8080 | -0.008 | 0.4784 | No |
| 36 | PSMA7 | PSMA7 Entrez,  Source | proteasome (prosome, macropain) subunit, alpha type, 7 | 8330 | -0.011 | 0.4709 | No |
| 37 | PSME4 | PSME4 Entrez,  Source | proteasome (prosome, macropain) activator subunit 4 | 8842 | -0.017 | 0.4529 | No |
| 38 | PSMD4 | PSMD4 Entrez,  Source | proteasome (prosome, macropain) 26S subunit, non-ATPase, 4 | 8864 | -0.017 | 0.4602 | No |
| 39 | PSMD3 | PSMD3 Entrez,  Source | proteasome (prosome, macropain) 26S subunit, non-ATPase, 3 | 9776 | -0.027 | 0.4270 | No |
| 40 | PSME3 | PSME3 Entrez,  Source | proteasome (prosome, macropain) activator subunit 3 (PA28 gamma; Ki) | 10822 | -0.040 | 0.3930 | No |
| 41 | PSMB5 | PSMB5 Entrez,  Source | proteasome (prosome, macropain) subunit, beta type, 5 | 11785 | -0.051 | 0.3690 | No |
| 42 | PSMF1 | PSMF1 Entrez,  Source | proteasome (prosome, macropain) inhibitor subunit 1 (PI31) | 12427 | -0.059 | 0.3653 | No |
Table: GSEA details [plain text format]

  

Fig 2: KEGG\_PROTEASOME      
 Blue-Pink O' Gram in the Space of the Analyzed GeneSet

  

Fig 3: KEGG\_PROTEASOME: Random ES distribution      
 Gene set null distribution of ES for **KEGG\_PROTEASOME**

  
