# Supplementary material for: Comprehensive analysis of β-catenin target genes in colorectal carcinoma cell lines with deregulated Wnt/β-catenin signaling
Source: BMC Genomics. 2014 Jan 28;15:74. doi: 10.1186/1471-2164-15-74 (PMC3909937; doi:10.1186/1471-2164-15-74)
Supplement: Additional file 5 — GSEA analysis using the KEGG pathway database. This zipped file contains confirming data of the GSEA analysis. The names of the directories containing the files were composed of the term ‘GSEA’, the name of the cell line, e.g. DLD1, SW480, or LS174T, and the pathway database (KEGG). Please use a web browser to view the files with the name ‘index.html’ in the corresponding directories to start exploring the data. [file 1471-2164-15-74-S5.zip › GSEA KEGG SW480/KEGG_REGULATION_OF_AUTOPHAGY.html]

Details for gene set KEGG\_REGULATION\_OF\_AUTOPHAGY[GSEA]

|  || Dataset | SW480\_collapsed\_to\_symbols.class.cls#b\_versus\_bg.class.cls#b\_versus\_bg\_repos |
| Phenotype | class.cls#b\_versus\_bg\_repos |
| Upregulated in class | 0 |
| GeneSet | KEGG\_REGULATION\_OF\_AUTOPHAGY |
| Enrichment Score (ES) | -0.4264566 |
| Normalized Enrichment Score (NES) | -1.3263967 |
| Nominal p-value | 0.12190813 |
| FDR q-value | 0.43981043 |
| FWER p-Value | 1.0 |
Table: GSEA Results Summary

  

Fig 1: Enrichment plot: KEGG\_REGULATION\_OF\_AUTOPHAGY      
 Profile of the Running ES Score & Positions of GeneSet Members on the Rank Ordered List

  

| PROBE | GENE SYMBOL | GENE\_TITLE | RANK IN GENE LIST | RANK METRIC SCORE | RUNNING ES | CORE ENRICHMENT || 1 | ATG7 | ATG7 Entrez,  Source | ATG7 autophagy related 7 homolog (S. cerevisiae) | 1453 | 0.150 | -0.0206 | No |
| 2 | PRKAA2 | PRKAA2 Entrez,  Source | protein kinase, AMP-activated, alpha 2 catalytic subunit | 1838 | 0.128 | 0.0053 | No |
| 3 | ATG3 | ATG3 Entrez,  Source | ATG3 autophagy related 3 homolog (S. cerevisiae) | 2097 | 0.116 | 0.0337 | No |
| 4 | ATG12 | ATG12 Entrez,  Source | ATG12 autophagy related 12 homolog (S. cerevisiae) | 2809 | 0.089 | 0.0289 | No |
| 5 | PRKAA1 | PRKAA1 Entrez,  Source | protein kinase, AMP-activated, alpha 1 catalytic subunit | 3814 | 0.060 | -0.0011 | No |
| 6 | BECN1 | BECN1 Entrez,  Source | beclin 1 (coiled-coil, myosin-like BCL2 interacting protein) | 4990 | 0.035 | -0.0486 | No |
| 7 | GABARAP | GABARAP Entrez,  Source | GABA(A) receptor-associated protein | 5381 | 0.029 | -0.0583 | No |
| 8 | ULK2 | ULK2 Entrez,  Source | unc-51-like kinase 2 (C. elegans) | 5462 | 0.028 | -0.0525 | No |
| 9 | ATG4A | ATG4A Entrez,  Source | ATG4 autophagy related 4 homolog A (S. cerevisiae) | 5725 | 0.024 | -0.0574 | No |
| 10 | ATG5 | ATG5 Entrez,  Source | ATG5 autophagy related 5 homolog (S. cerevisiae) | 5761 | 0.023 | -0.0510 | No |
| 11 | ULK3 | ULK3 Entrez,  Source | unc-51-like kinase 3 (C. elegans) | 6886 | 0.007 | -0.1059 | No |
| 12 | IFNG | IFNG Entrez,  Source | interferon, gamma | 6964 | 0.006 | -0.1075 | No |
| 13 | IFNA2 | IFNA2 Entrez,  Source | interferon, alpha 2 | 7881 | -0.006 | -0.1525 | No |
| 14 | PIK3R4 | PIK3R4 Entrez,  Source | phosphoinositide-3-kinase, regulatory subunit 4, p150 | 8573 | -0.014 | -0.1830 | No |
| 15 | PIK3C3 | PIK3C3 Entrez,  Source | phosphoinositide-3-kinase, class 3 | 9192 | -0.021 | -0.2072 | No |
| 16 | INS | INS Entrez,  Source | insulin | 10390 | -0.034 | -0.2562 | No |
| 17 | ATG4D | ATG4D Entrez,  Source | ATG4 autophagy related 4 homolog D (S. cerevisiae) | 10652 | -0.038 | -0.2561 | No |
| 18 | IFNA6 | IFNA6 Entrez,  Source | interferon, alpha 6 | 11757 | -0.051 | -0.2943 | No |
| 19 | IFNA8 | IFNA8 Entrez,  Source | interferon, alpha 8 | 12021 | -0.055 | -0.2883 | No |
| 20 | IFNA21 | IFNA21 Entrez,  Source | interferon, alpha 21 | 12989 | -0.066 | -0.3140 | No |
| 21 | ATG4B | ATG4B Entrez,  Source | ATG4 autophagy related 4 homolog B (S. cerevisiae) | 13662 | -0.075 | -0.3216 | No |
| 22 | GABARAPL2 | GABARAPL2 Entrez,  Source | GABA(A) receptor-associated protein-like 2 | 13900 | -0.078 | -0.3057 | No |
| 23 | IFNA10 | IFNA10 Entrez,  Source | interferon, alpha 10 | 16258 | -0.116 | -0.3849 | Yes |
| 24 | IFNA5 | IFNA5 Entrez,  Source | interferon, alpha 5 | 16708 | -0.126 | -0.3630 | Yes |
| 25 | IFNA7 | IFNA7 Entrez,  Source | interferon, alpha 7 | 17022 | -0.133 | -0.3314 | Yes |
| 26 | ULK1 | ULK1 Entrez,  Source | unc-51-like kinase 1 (C. elegans) | 17098 | -0.135 | -0.2868 | Yes |
| 27 | GABARAPL1 | GABARAPL1 Entrez,  Source | GABA(A) receptor-associated protein like 1 | 17922 | -0.163 | -0.2706 | Yes |
| 28 | IFNA1 | IFNA1 Entrez,  Source | interferon, alpha 1 | 18074 | -0.169 | -0.2178 | Yes |
| 29 | IFNA17 | IFNA17 Entrez,  Source | interferon, alpha 17 | 18148 | -0.173 | -0.1595 | Yes |
| 30 | IFNA14 | IFNA14 Entrez,  Source | interferon, alpha 14 | 18299 | -0.181 | -0.1026 | Yes |
| 31 | IFNA16 | IFNA16 Entrez,  Source | interferon, alpha 16 | 18386 | -0.186 | -0.0403 | Yes |
| 32 | IFNA4 | IFNA4 Entrez,  Source | interferon, alpha 4 | 19182 | -0.280 | 0.0192 | Yes |
Table: GSEA details [plain text format]

  

Fig 2: KEGG\_REGULATION\_OF\_AUTOPHAGY      
 Blue-Pink O' Gram in the Space of the Analyzed GeneSet

  

Fig 3: KEGG\_REGULATION\_OF\_AUTOPHAGY: Random ES distribution      
 Gene set null distribution of ES for **KEGG\_REGULATION\_OF\_AUTOPHAGY**

  
