# Supplementary material for: Comprehensive analysis of β-catenin target genes in colorectal carcinoma cell lines with deregulated Wnt/β-catenin signaling
Source: BMC Genomics. 2014 Jan 28;15:74. doi: 10.1186/1471-2164-15-74 (PMC3909937; doi:10.1186/1471-2164-15-74)
Supplement: Additional file 5 — GSEA analysis using the KEGG pathway database. This zipped file contains confirming data of the GSEA analysis. The names of the directories containing the files were composed of the term ‘GSEA’, the name of the cell line, e.g. DLD1, SW480, or LS174T, and the pathway database (KEGG). Please use a web browser to view the files with the name ‘index.html’ in the corresponding directories to start exploring the data. [file 1471-2164-15-74-S5.zip › GSEA KEGG SW480/KEGG_SMALL_CELL_LUNG_CANCER.html]

Details for gene set KEGG\_SMALL\_CELL\_LUNG\_CANCER[GSEA]

|  || Dataset | SW480\_collapsed\_to\_symbols.class.cls#b\_versus\_bg.class.cls#b\_versus\_bg\_repos |
| Phenotype | class.cls#b\_versus\_bg\_repos |
| Upregulated in class | 1 |
| GeneSet | KEGG\_SMALL\_CELL\_LUNG\_CANCER |
| Enrichment Score (ES) | 0.47233638 |
| Normalized Enrichment Score (NES) | 1.8560222 |
| Nominal p-value | 0.0 |
| FDR q-value | 0.04000057 |
| FWER p-Value | 0.104 |
Table: GSEA Results Summary

  

Fig 1: Enrichment plot: KEGG\_SMALL\_CELL\_LUNG\_CANCER      
 Profile of the Running ES Score & Positions of GeneSet Members on the Rank Ordered List

  

| PROBE | GENE SYMBOL | GENE\_TITLE | RANK IN GENE LIST | RANK METRIC SCORE | RUNNING ES | CORE ENRICHMENT || 1 | CDKN2B | CDKN2B Entrez,  Source | cyclin-dependent kinase inhibitor 2B (p15, inhibits CDK4) | 96 | 0.548 | 0.0591 | Yes |
| 2 | ITGA3 | ITGA3 Entrez,  Source | integrin, alpha 3 (antigen CD49C, alpha 3 subunit of VLA-3 receptor) | 134 | 0.458 | 0.1107 | Yes |
| 3 | FN1 | FN1 Entrez,  Source | fibronectin 1 | 145 | 0.442 | 0.1619 | Yes |
| 4 | BIRC3 | BIRC3 Entrez,  Source | baculoviral IAP repeat-containing 3 | 455 | 0.289 | 0.1797 | Yes |
| 5 | PIAS3 | PIAS3 Entrez,  Source | protein inhibitor of activated STAT, 3 | 476 | 0.283 | 0.2118 | Yes |
| 6 | LAMA3 | LAMA3 Entrez,  Source | laminin, alpha 3 | 514 | 0.269 | 0.2413 | Yes |
| 7 | BIRC2 | BIRC2 Entrez,  Source | baculoviral IAP repeat-containing 2 | 572 | 0.255 | 0.2682 | Yes |
| 8 | APAF1 | APAF1 Entrez,  Source | apoptotic peptidase activating factor | 584 | 0.253 | 0.2972 | Yes |
| 9 | IKBKB | IKBKB Entrez,  Source | inhibitor of kappa light polypeptide gene enhancer in B-cells, kinase beta | 860 | 0.208 | 0.3074 | Yes |
| 10 | AKT3 | AKT3 Entrez,  Source | v-akt murine thymoma viral oncogene homolog 3 (protein kinase B, gamma) | 870 | 0.207 | 0.3311 | Yes |
| 11 | ITGA6 | ITGA6 Entrez,  Source | integrin, alpha 6 | 1088 | 0.180 | 0.3410 | Yes |
| 12 | CCNE2 | CCNE2 Entrez,  Source | cyclin E2 | 1131 | 0.176 | 0.3594 | Yes |
| 13 | IKBKG | IKBKG Entrez,  Source | inhibitor of kappa light polypeptide gene enhancer in B-cells, kinase gamma | 1169 | 0.172 | 0.3776 | Yes |
| 14 | LAMB1 | LAMB1 Entrez,  Source | laminin, beta 1 | 1397 | 0.155 | 0.3841 | Yes |
| 15 | RB1 | RB1 Entrez,  Source | retinoblastoma 1 (including osteosarcoma) | 1421 | 0.153 | 0.4007 | Yes |
| 16 | COL4A1 | COL4A1 Entrez,  Source | collagen, type IV, alpha 1 | 1473 | 0.149 | 0.4155 | Yes |
| 17 | PIAS1 | PIAS1 Entrez,  Source | protein inhibitor of activated STAT, 1 | 1526 | 0.146 | 0.4299 | Yes |
| 18 | ITGA2 | ITGA2 Entrez,  Source | integrin, alpha 2 (CD49B, alpha 2 subunit of VLA-2 receptor) | 1768 | 0.131 | 0.4328 | Yes |
| 19 | ITGAV | ITGAV Entrez,  Source | integrin, alpha V (vitronectin receptor, alpha polypeptide, antigen CD51) | 2073 | 0.117 | 0.4309 | Yes |
| 20 | TRAF1 | TRAF1 Entrez,  Source | TNF receptor-associated factor 1 | 2203 | 0.111 | 0.4373 | Yes |
| 21 | PTGS2 | PTGS2 Entrez,  Source | prostaglandin-endoperoxide synthase 2 (prostaglandin G/H synthase and cyclooxygenase) | 2261 | 0.109 | 0.4471 | Yes |
| 22 | RXRA | RXRA Entrez,  Source | retinoid X receptor, alpha | 2312 | 0.107 | 0.4570 | Yes |
| 23 | ITGB1 | ITGB1 Entrez,  Source | integrin, beta 1 (fibronectin receptor, beta polypeptide, antigen CD29 includes MDF2, MSK12) | 2321 | 0.107 | 0.4691 | Yes |
| 24 | LAMA5 | LAMA5 Entrez,  Source | laminin, alpha 5 | 2487 | 0.100 | 0.4723 | Yes |
| 25 | PIK3CB | PIK3CB Entrez,  Source | phosphoinositide-3-kinase, catalytic, beta polypeptide | 3241 | 0.075 | 0.4424 | No |
| 26 | TRAF5 | TRAF5 Entrez,  Source | TNF receptor-associated factor 5 | 3302 | 0.073 | 0.4479 | No |
| 27 | NFKBIA | NFKBIA Entrez,  Source | nuclear factor of kappa light polypeptide gene enhancer in B-cells inhibitor, alpha | 3360 | 0.072 | 0.4534 | No |
| 28 | AKT2 | AKT2 Entrez,  Source | v-akt murine thymoma viral oncogene homolog 2 | 3525 | 0.067 | 0.4528 | No |
| 29 | CDK6 | CDK6 Entrez,  Source | cyclin-dependent kinase 6 | 3698 | 0.062 | 0.4513 | No |
| 30 | CHUK | CHUK Entrez,  Source | conserved helix-loop-helix ubiquitous kinase | 3863 | 0.059 | 0.4497 | No |
| 31 | CDKN1B | CDKN1B Entrez,  Source | cyclin-dependent kinase inhibitor 1B (p27, Kip1) | 3995 | 0.056 | 0.4495 | No |
| 32 | TRAF4 | TRAF4 Entrez,  Source | TNF receptor-associated factor 4 | 4204 | 0.051 | 0.4448 | No |
| 33 | PTK2 | PTK2 Entrez,  Source | PTK2 protein tyrosine kinase 2 | 4313 | 0.049 | 0.4449 | No |
| 34 | PTEN | PTEN Entrez,  Source | phosphatase and tensin homolog (mutated in multiple advanced cancers 1) | 5203 | 0.032 | 0.4030 | No |
| 35 | RARB | RARB Entrez,  Source | retinoic acid receptor, beta | 5380 | 0.029 | 0.3973 | No |
| 36 | CKS1B | CKS1B Entrez,  Source | CDC28 protein kinase regulatory subunit 1B | 5469 | 0.028 | 0.3960 | No |
| 37 | LAMB2 | LAMB2 Entrez,  Source | laminin, beta 2 (laminin S) | 5499 | 0.027 | 0.3977 | No |
| 38 | RELA | RELA Entrez,  Source | v-rel reticuloendotheliosis viral oncogene homolog A, nuclear factor of kappa light polypeptide gene enhancer in B-cells 3, p65 (avian) | 5505 | 0.027 | 0.4006 | No |
| 39 | E2F2 | E2F2 Entrez,  Source | E2F transcription factor 2 | 5551 | 0.026 | 0.4014 | No |
| 40 | TRAF2 | TRAF2 Entrez,  Source | TNF receptor-associated factor 2 | 5841 | 0.022 | 0.3891 | No |
| 41 | AKT1 | AKT1 Entrez,  Source | v-akt murine thymoma viral oncogene homolog 1 | 6295 | 0.015 | 0.3676 | No |
| 42 | PIK3R1 | PIK3R1 Entrez,  Source | phosphoinositide-3-kinase, regulatory subunit 1 (p85 alpha) | 6402 | 0.014 | 0.3638 | No |
| 43 | PIAS2 | PIAS2 Entrez,  Source | protein inhibitor of activated STAT, 2 | 6637 | 0.011 | 0.3530 | No |
| 44 | CDK2 | CDK2 Entrez,  Source | cyclin-dependent kinase 2 | 6673 | 0.010 | 0.3524 | No |
| 45 | CCND1 | CCND1 Entrez,  Source | cyclin D1 | 7482 | -0.000 | 0.3110 | No |
| 46 | RXRG | RXRG Entrez,  Source | retinoid X receptor, gamma | 7857 | -0.005 | 0.2924 | No |
| 47 | MAX | MAX Entrez,  Source | MYC associated factor X | 7858 | -0.005 | 0.2930 | No |
| 48 | BCL2L1 | BCL2L1 Entrez,  Source | BCL2-like 1 | 7937 | -0.006 | 0.2897 | No |
| 49 | BCL2 | BCL2 Entrez,  Source | B-cell CLL/lymphoma 2 | 8283 | -0.010 | 0.2732 | No |
| 50 | CDK4 | CDK4 Entrez,  Source | cyclin-dependent kinase 4 | 8358 | -0.011 | 0.2707 | No |
| 51 | CYCS | CYCS Entrez,  Source | cytochrome c, somatic | 8696 | -0.015 | 0.2551 | No |
| 52 | NFKB1 | NFKB1 Entrez,  Source | nuclear factor of kappa light polypeptide gene enhancer in B-cells 1 (p105) | 8939 | -0.018 | 0.2448 | No |
| 53 | RXRB | RXRB Entrez,  Source | retinoid X receptor, beta | 9175 | -0.021 | 0.2352 | No |
| 54 | TRAF3 | TRAF3 Entrez,  Source | TNF receptor-associated factor 3 | 9293 | -0.022 | 0.2317 | No |
| 55 | LAMC1 | LAMC1 Entrez,  Source | laminin, gamma 1 (formerly LAMB2) | 9927 | -0.029 | 0.2026 | No |
| 56 | PIK3R3 | PIK3R3 Entrez,  Source | phosphoinositide-3-kinase, regulatory subunit 3 (p55, gamma) | 10110 | -0.031 | 0.1969 | No |
| 57 | PIK3R5 | PIK3R5 Entrez,  Source | phosphoinositide-3-kinase, regulatory subunit 5, p101 | 10124 | -0.031 | 0.1999 | No |
| 58 | TRAF6 | TRAF6 Entrez,  Source | TNF receptor-associated factor 6 | 10318 | -0.034 | 0.1939 | No |
| 59 | PIK3CG | PIK3CG Entrez,  Source | phosphoinositide-3-kinase, catalytic, gamma polypeptide | 10795 | -0.039 | 0.1741 | No |
| 60 | E2F1 | E2F1 Entrez,  Source | E2F transcription factor 1 | 10826 | -0.040 | 0.1772 | No |
| 61 | LAMB3 | LAMB3 Entrez,  Source | laminin, beta 3 | 11110 | -0.043 | 0.1678 | No |
| 62 | E2F3 | E2F3 Entrez,  Source | E2F transcription factor 3 | 11523 | -0.048 | 0.1523 | No |
| 63 | PIK3CA | PIK3CA Entrez,  Source | phosphoinositide-3-kinase, catalytic, alpha polypeptide | 11588 | -0.049 | 0.1547 | No |
| 64 | CCNE1 | CCNE1 Entrez,  Source | cyclin E1 | 12015 | -0.055 | 0.1393 | No |
| 65 | LAMB4 | LAMB4 Entrez,  Source | laminin, beta 4 | 12018 | -0.055 | 0.1455 | No |
| 66 | LAMC2 | LAMC2 Entrez,  Source | laminin, gamma 2 | 12750 | -0.063 | 0.1154 | No |
| 67 | PIAS4 | PIAS4 Entrez,  Source | protein inhibitor of activated STAT, 4 | 13734 | -0.076 | 0.0738 | No |
| 68 | LAMA4 | LAMA4 Entrez,  Source | laminin, alpha 4 | 14214 | -0.082 | 0.0589 | No |
| 69 | COL4A6 | COL4A6 Entrez,  Source | collagen, type IV, alpha 6 | 14340 | -0.084 | 0.0623 | No |
| 70 | LAMA1 | LAMA1 Entrez,  Source | laminin, alpha 1 | 14411 | -0.085 | 0.0686 | No |
| 71 | PIK3R2 | PIK3R2 Entrez,  Source | phosphoinositide-3-kinase, regulatory subunit 2 (p85 beta) | 14488 | -0.086 | 0.0747 | No |
| 72 | CASP9 | CASP9 Entrez,  Source | caspase 9, apoptosis-related cysteine peptidase | 15133 | -0.096 | 0.0529 | No |
| 73 | ITGA2B | ITGA2B Entrez,  Source | integrin, alpha 2b (platelet glycoprotein IIb of IIb/IIIa complex, antigen CD41) | 15161 | -0.097 | 0.0628 | No |
| 74 | TP53 | TP53 Entrez,  Source | tumor protein p53 (Li-Fraumeni syndrome) | 15395 | -0.100 | 0.0625 | No |
| 75 | COL4A4 | COL4A4 Entrez,  Source | collagen, type IV, alpha 4 | 16048 | -0.112 | 0.0422 | No |
| 76 | LAMA2 | LAMA2 Entrez,  Source | laminin, alpha 2 (merosin, congenital muscular dystrophy) | 16160 | -0.114 | 0.0498 | No |
| 77 | PIK3CD | PIK3CD Entrez,  Source | phosphoinositide-3-kinase, catalytic, delta polypeptide | 16633 | -0.124 | 0.0400 | No |
| 78 | SKP2 | SKP2 Entrez,  Source | S-phase kinase-associated protein 2 (p45) | 17303 | -0.142 | 0.0222 | No |
| 79 | LAMC3 | LAMC3 Entrez,  Source | laminin, gamma 3 | 17882 | -0.162 | 0.0115 | No |
| 80 | FHIT | FHIT Entrez,  Source | fragile histidine triad gene | 17935 | -0.164 | 0.0280 | No |
| 81 | COL4A2 | COL4A2 Entrez,  Source | collagen, type IV, alpha 2 | 18730 | -0.211 | 0.0118 | No |
| 82 | MYC | MYC Entrez,  Source | v-myc myelocytomatosis viral oncogene homolog (avian) | 19109 | -0.262 | 0.0230 | No |
Table: GSEA details [plain text format]

  

Fig 2: KEGG\_SMALL\_CELL\_LUNG\_CANCER      
 Blue-Pink O' Gram in the Space of the Analyzed GeneSet

  

Fig 3: KEGG\_SMALL\_CELL\_LUNG\_CANCER: Random ES distribution      
 Gene set null distribution of ES for **KEGG\_SMALL\_CELL\_LUNG\_CANCER**

  
