# Supplementary material for: Comprehensive analysis of β-catenin target genes in colorectal carcinoma cell lines with deregulated Wnt/β-catenin signaling
Source: BMC Genomics. 2014 Jan 28;15:74. doi: 10.1186/1471-2164-15-74 (PMC3909937; doi:10.1186/1471-2164-15-74)
Supplement: Additional file 4 — GSEA analysis using the Biocarta pathway database. This zipped file contains confirming data of the GSEA analysis. The names of the directories containing the files were composed of the term ‘GSEA’, the name of the cell line, e.g. DLD1, SW480, or LS174T, and the pathway database (Biocarta). Please use a web browser to view the files with the name ‘index.html’ in the corresponding directories to start exploring the data. [file 1471-2164-15-74-S4.zip › LS174T_Biocarta/BIOCARTA_ACH_PATHWAY.html]

Details for gene set BIOCARTA\_ACH\_PATHWAY[GSEA]

|  || Dataset | LS174T\_collapsed\_to\_symbols.class.cls#bg\_versus\_b |
| Phenotype | class.cls#bg\_versus\_b |
| Upregulated in class | bg |
| GeneSet | BIOCARTA\_ACH\_PATHWAY |
| Enrichment Score (ES) | 0.5052415 |
| Normalized Enrichment Score (NES) | 1.2992066 |
| Nominal p-value | 0.17561984 |
| FDR q-value | 0.69789857 |
| FWER p-Value | 0.999 |
Table: GSEA Results Summary

  

Fig 1: Enrichment plot: BIOCARTA\_ACH\_PATHWAY      
 Profile of the Running ES Score & Positions of GeneSet Members on the Rank Ordered List

  

| PROBE | GENE SYMBOL | GENE\_TITLE | RANK IN GENE LIST | RANK METRIC SCORE | RUNNING ES | CORE ENRICHMENT || 1 | CHRNB1 | CHRNB1 Entrez,  Source | cholinergic receptor, nicotinic, beta 1 (muscle) | 254 | 0.544 | 0.2065 | Yes |
| 2 | SRC | SRC Entrez,  Source | v-src sarcoma (Schmidt-Ruppin A-2) viral oncogene homolog (avian) | 953 | 0.307 | 0.2948 | Yes |
| 3 | TERT | TERT Entrez,  Source | telomerase reverse transcriptase | 1032 | 0.296 | 0.4103 | Yes |
| 4 | AKT1 | AKT1 Entrez,  Source | v-akt murine thymoma viral oncogene homolog 1 | 1273 | 0.266 | 0.5052 | Yes |
| 5 | PIK3R1 | PIK3R1 Entrez,  Source | phosphoinositide-3-kinase, regulatory subunit 1 (p85 alpha) | 5670 | 0.070 | 0.3086 | No |
| 6 | BAD | BAD Entrez,  Source | BCL2-antagonist of cell death | 10235 | -0.012 | 0.0800 | No |
| 7 | CHRNG | CHRNG Entrez,  Source | cholinergic receptor, nicotinic, gamma | 12869 | -0.059 | -0.0309 | No |
| 8 | YWHAH | YWHAH Entrez,  Source | tyrosine 3-monooxygenase/tryptophan 5-monooxygenase activation protein, eta polypeptide | 13683 | -0.075 | -0.0420 | No |
| 9 | RAPSN | RAPSN Entrez,  Source | receptor-associated protein of the synapse, 43kD | 13781 | -0.077 | -0.0158 | No |
| 10 | MUSK | MUSK Entrez,  Source | muscle, skeletal, receptor tyrosine kinase | 13881 | -0.080 | 0.0113 | No |
| 11 | PTK2 | PTK2 Entrez,  Source | PTK2 protein tyrosine kinase 2 | 14708 | -0.099 | 0.0091 | No |
| 12 | PTK2B | PTK2B Entrez,  Source | PTK2B protein tyrosine kinase 2 beta | 14768 | -0.101 | 0.0470 | No |
| 13 | FASLG | FASLG Entrez,  Source | Fas ligand (TNF superfamily, member 6) | 15611 | -0.125 | 0.0543 | No |
| 14 | PIK3CA | PIK3CA Entrez,  Source | phosphoinositide-3-kinase, catalytic, alpha polypeptide | 16332 | -0.149 | 0.0777 | No |
| 15 | PIK3CG | PIK3CG Entrez,  Source | phosphoinositide-3-kinase, catalytic, gamma polypeptide | 17689 | -0.216 | 0.0955 | No |
Table: GSEA details [plain text format]

  

Fig 2: BIOCARTA\_ACH\_PATHWAY      
 Blue-Pink O' Gram in the Space of the Analyzed GeneSet

  

Fig 3: BIOCARTA\_ACH\_PATHWAY: Random ES distribution      
 Gene set null distribution of ES for **BIOCARTA\_ACH\_PATHWAY**
