# Supplementary material for: Comprehensive analysis of β-catenin target genes in colorectal carcinoma cell lines with deregulated Wnt/β-catenin signaling
Source: BMC Genomics. 2014 Jan 28;15:74. doi: 10.1186/1471-2164-15-74 (PMC3909937; doi:10.1186/1471-2164-15-74)
Supplement: Additional file 4 — GSEA analysis using the Biocarta pathway database. This zipped file contains confirming data of the GSEA analysis. The names of the directories containing the files were composed of the term ‘GSEA’, the name of the cell line, e.g. DLD1, SW480, or LS174T, and the pathway database (Biocarta). Please use a web browser to view the files with the name ‘index.html’ in the corresponding directories to start exploring the data. [file 1471-2164-15-74-S4.zip › LS174T_Biocarta/BIOCARTA_ALK_PATHWAY.html]

Details for gene set BIOCARTA\_ALK\_PATHWAY[GSEA]

|  || Dataset | LS174T\_collapsed\_to\_symbols.class.cls#bg\_versus\_b |
| Phenotype | class.cls#bg\_versus\_b |
| Upregulated in class | bg |
| GeneSet | BIOCARTA\_ALK\_PATHWAY |
| Enrichment Score (ES) | 0.40516686 |
| Normalized Enrichment Score (NES) | 1.2774749 |
| Nominal p-value | 0.12793177 |
| FDR q-value | 0.66393673 |
| FWER p-Value | 0.999 |
Table: GSEA Results Summary

  

Fig 1: Enrichment plot: BIOCARTA\_ALK\_PATHWAY      
 Profile of the Running ES Score & Positions of GeneSet Members on the Rank Ordered List

  

| PROBE | GENE SYMBOL | GENE\_TITLE | RANK IN GENE LIST | RANK METRIC SCORE | RUNNING ES | CORE ENRICHMENT || 1 | CTNNB1 | CTNNB1 Entrez,  Source | catenin (cadherin-associated protein), beta 1, 88kDa | 3 | 1.322 | 0.2224 | Yes |
| 2 | FZD1 | FZD1 Entrez,  Source | frizzled homolog 1 (Drosophila) | 811 | 0.331 | 0.2368 | Yes |
| 3 | TGFBR3 | TGFBR3 Entrez,  Source | transforming growth factor, beta receptor III (betaglycan, 300kDa) | 1233 | 0.270 | 0.2607 | Yes |
| 4 | NKX2-5 | NKX2-5 Entrez,  Source | NK2 transcription factor related, locus 5 (Drosophila) | 1307 | 0.262 | 0.3010 | Yes |
| 5 | SMAD6 | SMAD6 Entrez,  Source | SMAD, mothers against DPP homolog 6 (Drosophila) | 1486 | 0.243 | 0.3327 | Yes |
| 6 | BMP4 | BMP4 Entrez,  Source | bone morphogenetic protein 4 | 1533 | 0.239 | 0.3706 | Yes |
| 7 | BMP7 | BMP7 Entrez,  Source | bone morphogenetic protein 7 (osteogenic protein 1) | 1616 | 0.230 | 0.4052 | Yes |
| 8 | WNT1 | WNT1 Entrez,  Source | wingless-type MMTV integration site family, member 1 | 3453 | 0.132 | 0.3332 | No |
| 9 | AXIN1 | AXIN1 Entrez,  Source | axin 1 | 3544 | 0.128 | 0.3501 | No |
| 10 | RFC1 | RFC1 Entrez,  Source | replication factor C (activator 1) 1, 145kDa | 5042 | 0.085 | 0.2877 | No |
| 11 | SMAD4 | SMAD4 Entrez,  Source | SMAD, mothers against DPP homolog 4 (Drosophila) | 5267 | 0.079 | 0.2895 | No |
| 12 | GATA4 | GATA4 Entrez,  Source | GATA binding protein 4 | 5448 | 0.075 | 0.2929 | No |
| 13 | BMP10 | BMP10 Entrez,  Source | bone morphogenetic protein 10 | 5656 | 0.070 | 0.2941 | No |
| 14 | BMPR1A | BMPR1A Entrez,  Source | bone morphogenetic protein receptor, type IA | 7762 | 0.030 | 0.1913 | No |
| 15 | SMAD1 | SMAD1 Entrez,  Source | SMAD, mothers against DPP homolog 1 (Drosophila) | 8264 | 0.021 | 0.1691 | No |
| 16 | BMP5 | BMP5 Entrez,  Source | bone morphogenetic protein 5 | 8364 | 0.019 | 0.1672 | No |
| 17 | TGFBR1 | TGFBR1 Entrez,  Source | transforming growth factor, beta receptor I (activin A receptor type II-like kinase, 53kDa) | 8501 | 0.016 | 0.1629 | No |
| 18 | SMAD5 | SMAD5 Entrez,  Source | SMAD, mothers against DPP homolog 5 (Drosophila) | 8797 | 0.011 | 0.1496 | No |
| 19 | ACVR1 | ACVR1 Entrez,  Source | activin A receptor, type I | 9208 | 0.004 | 0.1293 | No |
| 20 | TGFBR2 | TGFBR2 Entrez,  Source | transforming growth factor, beta receptor II (70/80kDa) | 9220 | 0.004 | 0.1295 | No |
| 21 | CHRD | CHRD Entrez,  Source | chordin | 9366 | 0.002 | 0.1223 | No |
| 22 | GSK3B | GSK3B Entrez,  Source | glycogen synthase kinase 3 beta | 11233 | -0.029 | 0.0315 | No |
| 23 | NOG | NOG Entrez,  Source | noggin | 12026 | -0.043 | -0.0018 | No |
| 24 | DVL1 | DVL1 Entrez,  Source | dishevelled, dsh homolog 1 (Drosophila) | 12081 | -0.045 | 0.0030 | No |
| 25 | TGFB1 | TGFB1 Entrez,  Source | transforming growth factor, beta 1 (Camurati-Engelmann disease) | 12328 | -0.049 | -0.0013 | No |
| 26 | APC | APC Entrez,  Source | adenomatosis polyposis coli | 12500 | -0.052 | -0.0013 | No |
| 27 | MAP3K7 | MAP3K7 Entrez,  Source | mitogen-activated protein kinase kinase kinase 7 | 12737 | -0.057 | -0.0038 | No |
| 28 | NPPA | NPPA Entrez,  Source | natriuretic peptide precursor A | 13512 | -0.072 | -0.0313 | No |
| 29 | TGFB3 | TGFB3 Entrez,  Source | transforming growth factor, beta 3 | 14231 | -0.088 | -0.0533 | No |
| 30 | BMP2 | BMP2 Entrez,  Source | bone morphogenetic protein 2 | 14775 | -0.102 | -0.0641 | No |
| 31 | NPPB | NPPB Entrez,  Source | natriuretic peptide precursor B | 15839 | -0.132 | -0.0963 | No |
| 32 | MYL2 | MYL2 Entrez,  Source | myosin, light chain 2, regulatory, cardiac, slow | 15942 | -0.136 | -0.0787 | No |
| 33 | ATF2 | ATF2 Entrez,  Source | activating transcription factor 2 | 16998 | -0.175 | -0.1032 | No |
| 34 | TGFB2 | TGFB2 Entrez,  Source | transforming growth factor, beta 2 | 18757 | -0.339 | -0.1362 | No |
| 35 | BMPR2 | BMPR2 Entrez,  Source | bone morphogenetic protein receptor, type II (serine/threonine kinase) | 19123 | -0.442 | -0.0805 | No |
| 36 | MEF2C | MEF2C Entrez,  Source | MADS box transcription enhancer factor 2, polypeptide C (myocyte enhancer factor 2C) | 19322 | -0.609 | 0.0120 | No |
Table: GSEA details [plain text format]

  

Fig 2: BIOCARTA\_ALK\_PATHWAY      
 Blue-Pink O' Gram in the Space of the Analyzed GeneSet

  

Fig 3: BIOCARTA\_ALK\_PATHWAY: Random ES distribution      
 Gene set null distribution of ES for **BIOCARTA\_ALK\_PATHWAY**
