# Supplementary material for: Comprehensive analysis of β-catenin target genes in colorectal carcinoma cell lines with deregulated Wnt/β-catenin signaling
Source: BMC Genomics. 2014 Jan 28;15:74. doi: 10.1186/1471-2164-15-74 (PMC3909937; doi:10.1186/1471-2164-15-74)
Supplement: Additional file 4 — GSEA analysis using the Biocarta pathway database. This zipped file contains confirming data of the GSEA analysis. The names of the directories containing the files were composed of the term ‘GSEA’, the name of the cell line, e.g. DLD1, SW480, or LS174T, and the pathway database (Biocarta). Please use a web browser to view the files with the name ‘index.html’ in the corresponding directories to start exploring the data. [file 1471-2164-15-74-S4.zip › LS174T_Biocarta/BIOCARTA_AMI_PATHWAY.html]

Details for gene set BIOCARTA\_AMI\_PATHWAY[GSEA]

|  || Dataset | LS174T\_collapsed\_to\_symbols.class.cls#bg\_versus\_b |
| Phenotype | class.cls#bg\_versus\_b |
| Upregulated in class | b |
| GeneSet | BIOCARTA\_AMI\_PATHWAY |
| Enrichment Score (ES) | -0.4841513 |
| Normalized Enrichment Score (NES) | -1.2720201 |
| Nominal p-value | 0.18618043 |
| FDR q-value | 1.0 |
| FWER p-Value | 1.0 |
Table: GSEA Results Summary

  

Fig 1: Enrichment plot: BIOCARTA\_AMI\_PATHWAY      
 Profile of the Running ES Score & Positions of GeneSet Members on the Rank Ordered List

  

| PROBE | GENE SYMBOL | GENE\_TITLE | RANK IN GENE LIST | RANK METRIC SCORE | RUNNING ES | CORE ENRICHMENT || 1 | FGG | FGG Entrez,  Source | fibrinogen gamma chain | 1767 | 0.218 | -0.0159 | No |
| 2 | F7 | F7 Entrez,  Source | coagulation factor VII (serum prothrombin conversion accelerator) | 2890 | 0.154 | -0.0206 | No |
| 3 | SERPINC1 | SERPINC1 Entrez,  Source | serpin peptidase inhibitor, clade C (antithrombin), member 1 | 3990 | 0.113 | -0.0382 | No |
| 4 | COL4A2 | COL4A2 Entrez,  Source | collagen, type IV, alpha 2 | 5247 | 0.079 | -0.0753 | No |
| 5 | F10 | F10 Entrez,  Source | coagulation factor X | 7250 | 0.039 | -0.1645 | No |
| 6 | COL4A1 | COL4A1 Entrez,  Source | collagen, type IV, alpha 1 | 7359 | 0.036 | -0.1576 | No |
| 7 | FGB | FGB Entrez,  Source | fibrinogen beta chain | 8450 | 0.017 | -0.2075 | No |
| 8 | COL4A6 | COL4A6 Entrez,  Source | collagen, type IV, alpha 6 | 9899 | -0.007 | -0.2793 | No |
| 9 | COL4A5 | COL4A5 Entrez,  Source | collagen, type IV, alpha 5 (Alport syndrome) | 10502 | -0.016 | -0.3045 | No |
| 10 | FGA | FGA Entrez,  Source | fibrinogen alpha chain | 13230 | -0.067 | -0.4213 | No |
| 11 | COL4A3 | COL4A3 Entrez,  Source | collagen, type IV, alpha 3 (Goodpasture antigen) | 13620 | -0.074 | -0.4158 | No |
| 12 | TFPI | TFPI Entrez,  Source | tissue factor pathway inhibitor (lipoprotein-associated coagulation inhibitor) | 14957 | -0.106 | -0.4478 | Yes |
| 13 | COL4A4 | COL4A4 Entrez,  Source | collagen, type IV, alpha 4 | 15197 | -0.113 | -0.4215 | Yes |
| 14 | PLG | PLG Entrez,  Source | plasminogen | 15854 | -0.132 | -0.4098 | Yes |
| 15 | F2R | F2R Entrez,  Source | coagulation factor II (thrombin) receptor | 16507 | -0.155 | -0.3900 | Yes |
| 16 | PROC | PROC Entrez,  Source | protein C (inactivator of coagulation factors Va and VIIIa) | 17215 | -0.186 | -0.3624 | Yes |
| 17 | F2 | F2 Entrez,  Source | coagulation factor II (thrombin) | 17557 | -0.207 | -0.3089 | Yes |
| 18 | PROS1 | PROS1 Entrez,  Source | protein S (alpha) | 18928 | -0.381 | -0.2487 | Yes |
| 19 | PLAT | PLAT Entrez,  Source | plasminogen activator, tissue | 19444 | -0.820 | 0.0057 | Yes |
Table: GSEA details [plain text format]

  

Fig 2: BIOCARTA\_AMI\_PATHWAY      
 Blue-Pink O' Gram in the Space of the Analyzed GeneSet

  

Fig 3: BIOCARTA\_AMI\_PATHWAY: Random ES distribution      
 Gene set null distribution of ES for **BIOCARTA\_AMI\_PATHWAY**
