# Supplementary material for: Comprehensive analysis of β-catenin target genes in colorectal carcinoma cell lines with deregulated Wnt/β-catenin signaling
Source: BMC Genomics. 2014 Jan 28;15:74. doi: 10.1186/1471-2164-15-74 (PMC3909937; doi:10.1186/1471-2164-15-74)
Supplement: Additional file 4 — GSEA analysis using the Biocarta pathway database. This zipped file contains confirming data of the GSEA analysis. The names of the directories containing the files were composed of the term ‘GSEA’, the name of the cell line, e.g. DLD1, SW480, or LS174T, and the pathway database (Biocarta). Please use a web browser to view the files with the name ‘index.html’ in the corresponding directories to start exploring the data. [file 1471-2164-15-74-S4.zip › LS174T_Biocarta/BIOCARTA_ARF_PATHWAY.html]

Details for gene set BIOCARTA\_ARF\_PATHWAY[GSEA]

|  || Dataset | LS174T\_collapsed\_to\_symbols.class.cls#bg\_versus\_b |
| Phenotype | class.cls#bg\_versus\_b |
| Upregulated in class | bg |
| GeneSet | BIOCARTA\_ARF\_PATHWAY |
| Enrichment Score (ES) | 0.42737994 |
| Normalized Enrichment Score (NES) | 1.1340396 |
| Nominal p-value | 0.28070176 |
| FDR q-value | 0.85730916 |
| FWER p-Value | 1.0 |
Table: GSEA Results Summary

  

Fig 1: Enrichment plot: BIOCARTA\_ARF\_PATHWAY      
 Profile of the Running ES Score & Positions of GeneSet Members on the Rank Ordered List

  

| PROBE | GENE SYMBOL | GENE\_TITLE | RANK IN GENE LIST | RANK METRIC SCORE | RUNNING ES | CORE ENRICHMENT || 1 | POLR1A | POLR1A Entrez,  Source | polymerase (RNA) I polypeptide A, 194kDa | 954 | 0.307 | 0.1020 | Yes |
| 2 | E2F1 | E2F1 Entrez,  Source | E2F transcription factor 1 | 1057 | 0.292 | 0.2403 | Yes |
| 3 | MYC | MYC Entrez,  Source | v-myc myelocytomatosis viral oncogene homolog (avian) | 1540 | 0.238 | 0.3326 | Yes |
| 4 | TP53 | TP53 Entrez,  Source | tumor protein p53 (Li-Fraumeni syndrome) | 2459 | 0.175 | 0.3714 | Yes |
| 5 | POLR1B | POLR1B Entrez,  Source | polymerase (RNA) I polypeptide B, 128kDa | 2857 | 0.155 | 0.4274 | Yes |
| 6 | TWIST1 | TWIST1 Entrez,  Source | twist homolog 1 (acrocephalosyndactyly 3; Saethre-Chotzen syndrome) (Drosophila) | 4973 | 0.086 | 0.3617 | No |
| 7 | PIK3R1 | PIK3R1 Entrez,  Source | phosphoinositide-3-kinase, regulatory subunit 1 (p85 alpha) | 5670 | 0.070 | 0.3606 | No |
| 8 | ABL1 | ABL1 Entrez,  Source | v-abl Abelson murine leukemia viral oncogene homolog 1 | 6238 | 0.058 | 0.3601 | No |
| 9 | POLR1C | POLR1C Entrez,  Source | polymerase (RNA) I polypeptide C, 30kDa | 6381 | 0.055 | 0.3798 | No |
| 10 | MDM2 | MDM2 Entrez,  Source | Mdm2, transformed 3T3 cell double minute 2, p53 binding protein (mouse) | 6876 | 0.046 | 0.3770 | No |
| 11 | RB1 | RB1 Entrez,  Source | retinoblastoma 1 (including osteosarcoma) | 7450 | 0.035 | 0.3648 | No |
| 12 | RAC1 | RAC1 Entrez,  Source | ras-related C3 botulinum toxin substrate 1 (rho family, small GTP binding protein Rac1) | 9829 | -0.005 | 0.2458 | No |
| 13 | POLR1D | POLR1D Entrez,  Source | polymerase (RNA) I polypeptide D, 16kDa | 10358 | -0.014 | 0.2256 | No |
| 14 | CDKN2A | CDKN2A Entrez,  Source | cyclin-dependent kinase inhibitor 2A (melanoma, p16, inhibits CDK4) | 12732 | -0.057 | 0.1322 | No |
| 15 | TBX2 | TBX2 Entrez,  Source | T-box 2 | 13695 | -0.076 | 0.1202 | No |
| 16 | PIK3CA | PIK3CA Entrez,  Source | phosphoinositide-3-kinase, catalytic, alpha polypeptide | 16332 | -0.149 | 0.0587 | No |
| 17 | PIK3CG | PIK3CG Entrez,  Source | phosphoinositide-3-kinase, catalytic, gamma polypeptide | 17689 | -0.216 | 0.0955 | No |
Table: GSEA details [plain text format]

  

Fig 2: BIOCARTA\_ARF\_PATHWAY      
 Blue-Pink O' Gram in the Space of the Analyzed GeneSet

  

Fig 3: BIOCARTA\_ARF\_PATHWAY: Random ES distribution      
 Gene set null distribution of ES for **BIOCARTA\_ARF\_PATHWAY**
