# Supplementary material for: Comprehensive analysis of β-catenin target genes in colorectal carcinoma cell lines with deregulated Wnt/β-catenin signaling
Source: BMC Genomics. 2014 Jan 28;15:74. doi: 10.1186/1471-2164-15-74 (PMC3909937; doi:10.1186/1471-2164-15-74)
Supplement: Additional file 4 — GSEA analysis using the Biocarta pathway database. This zipped file contains confirming data of the GSEA analysis. The names of the directories containing the files were composed of the term ‘GSEA’, the name of the cell line, e.g. DLD1, SW480, or LS174T, and the pathway database (Biocarta). Please use a web browser to view the files with the name ‘index.html’ in the corresponding directories to start exploring the data. [file 1471-2164-15-74-S4.zip › LS174T_Biocarta/BIOCARTA_ATRBRCA_PATHWAY.html]

Details for gene set BIOCARTA\_ATRBRCA\_PATHWAY[GSEA]

|  || Dataset | LS174T\_collapsed\_to\_symbols.class.cls#bg\_versus\_b |
| Phenotype | class.cls#bg\_versus\_b |
| Upregulated in class | bg |
| GeneSet | BIOCARTA\_ATRBRCA\_PATHWAY |
| Enrichment Score (ES) | 0.73102325 |
| Normalized Enrichment Score (NES) | 2.0532205 |
| Nominal p-value | 0.0 |
| FDR q-value | 0.0043898043 |
| FWER p-Value | 0.0050 |
Table: GSEA Results Summary

  

Fig 1: Enrichment plot: BIOCARTA\_ATRBRCA\_PATHWAY      
 Profile of the Running ES Score & Positions of GeneSet Members on the Rank Ordered List

  

| PROBE | GENE SYMBOL | GENE\_TITLE | RANK IN GENE LIST | RANK METRIC SCORE | RUNNING ES | CORE ENRICHMENT || 1 | FANCG | FANCG Entrez,  Source | Fanconi anemia, complementation group G | 152 | 0.639 | 0.1034 | Yes |
| 2 | FANCD2 | FANCD2 Entrez,  Source | Fanconi anemia, complementation group D2 | 199 | 0.582 | 0.2021 | Yes |
| 3 | BRCA2 | BRCA2 Entrez,  Source | breast cancer 2, early onset | 212 | 0.571 | 0.3007 | Yes |
| 4 | BRCA1 | BRCA1 Entrez,  Source | breast cancer 1, early onset | 334 | 0.485 | 0.3788 | Yes |
| 5 | RAD51 | RAD51 Entrez,  Source | RAD51 homolog (RecA homolog, E. coli) (S. cerevisiae) | 351 | 0.481 | 0.4616 | Yes |
| 6 | MRE11A | MRE11A Entrez,  Source | MRE11 meiotic recombination 11 homolog A (S. cerevisiae) | 530 | 0.404 | 0.5227 | Yes |
| 7 | RAD1 | RAD1 Entrez,  Source | RAD1 homolog (S. pombe) | 563 | 0.393 | 0.5894 | Yes |
| 8 | FANCC | FANCC Entrez,  Source | Fanconi anemia, complementation group C | 591 | 0.386 | 0.6551 | Yes |
| 9 | CHEK2 | CHEK2 Entrez,  Source | CHK2 checkpoint homolog (S. pombe) | 720 | 0.350 | 0.7094 | Yes |
| 10 | RAD9A | RAD9A Entrez,  Source | RAD9 homolog A (S. pombe) | 1218 | 0.271 | 0.7310 | Yes |
| 11 | TP53 | TP53 Entrez,  Source | tumor protein p53 (Li-Fraumeni syndrome) | 2459 | 0.175 | 0.6979 | No |
| 12 | FANCE | FANCE Entrez,  Source | Fanconi anemia, complementation group E | 2630 | 0.165 | 0.7179 | No |
| 13 | NBN | NBN Entrez,  Source | nibrin | 5319 | 0.078 | 0.5939 | No |
| 14 | TREX1 | TREX1 Entrez,  Source | three prime repair exonuclease 1 | 5706 | 0.069 | 0.5862 | No |
| 15 | HUS1 | HUS1 Entrez,  Source | HUS1 checkpoint homolog (S. pombe) | 5863 | 0.065 | 0.5896 | No |
| 16 | ATR | ATR Entrez,  Source | ataxia telangiectasia and Rad3 related | 5974 | 0.063 | 0.5949 | No |
| 17 | CHEK1 | CHEK1 Entrez,  Source | CHK1 checkpoint homolog (S. pombe) | 7054 | 0.042 | 0.5471 | No |
| 18 | RAD50 | RAD50 Entrez,  Source | RAD50 homolog (S. cerevisiae) | 10334 | -0.014 | 0.3816 | No |
| 19 | ATM | ATM Entrez,  Source | ataxia telangiectasia mutated (includes complementation groups A, C and D) | 14388 | -0.092 | 0.1901 | No |
| 20 | RAD17 | RAD17 Entrez,  Source | RAD17 homolog (S. pombe) | 17195 | -0.186 | 0.0787 | No |
| 21 | FANCF | FANCF Entrez,  Source | Fanconi anemia, complementation group F | 18033 | -0.242 | 0.0780 | No |
Table: GSEA details [plain text format]

  

Fig 2: BIOCARTA\_ATRBRCA\_PATHWAY      
 Blue-Pink O' Gram in the Space of the Analyzed GeneSet

  

Fig 3: BIOCARTA\_ATRBRCA\_PATHWAY: Random ES distribution      
 Gene set null distribution of ES for **BIOCARTA\_ATRBRCA\_PATHWAY**
