# Supplementary material for: Comprehensive analysis of β-catenin target genes in colorectal carcinoma cell lines with deregulated Wnt/β-catenin signaling
Source: BMC Genomics. 2014 Jan 28;15:74. doi: 10.1186/1471-2164-15-74 (PMC3909937; doi:10.1186/1471-2164-15-74)
Supplement: Additional file 4 — GSEA analysis using the Biocarta pathway database. This zipped file contains confirming data of the GSEA analysis. The names of the directories containing the files were composed of the term ‘GSEA’, the name of the cell line, e.g. DLD1, SW480, or LS174T, and the pathway database (Biocarta). Please use a web browser to view the files with the name ‘index.html’ in the corresponding directories to start exploring the data. [file 1471-2164-15-74-S4.zip › LS174T_Biocarta/BIOCARTA_BAD_PATHWAY.html]

Details for gene set BIOCARTA\_BAD\_PATHWAY[GSEA]

|  || Dataset | LS174T\_collapsed\_to\_symbols.class.cls#bg\_versus\_b |
| Phenotype | class.cls#bg\_versus\_b |
| Upregulated in class | b |
| GeneSet | BIOCARTA\_BAD\_PATHWAY |
| Enrichment Score (ES) | -0.42453828 |
| Normalized Enrichment Score (NES) | -1.199502 |
| Nominal p-value | 0.22862454 |
| FDR q-value | 1.0 |
| FWER p-Value | 1.0 |
Table: GSEA Results Summary

  

Fig 1: Enrichment plot: BIOCARTA\_BAD\_PATHWAY      
 Profile of the Running ES Score & Positions of GeneSet Members on the Rank Ordered List

  

| PROBE | GENE SYMBOL | GENE\_TITLE | RANK IN GENE LIST | RANK METRIC SCORE | RUNNING ES | CORE ENRICHMENT || 1 | IL3 | IL3 Entrez,  Source | interleukin 3 (colony-stimulating factor, multiple) | 1194 | 0.274 | 0.0212 | No |
| 2 | AKT1 | AKT1 Entrez,  Source | v-akt murine thymoma viral oncogene homolog 1 | 1273 | 0.266 | 0.0970 | No |
| 3 | IL3RA | IL3RA Entrez,  Source | interleukin 3 receptor, alpha (low affinity) | 2854 | 0.155 | 0.0628 | No |
| 4 | PIK3R1 | PIK3R1 Entrez,  Source | phosphoinositide-3-kinase, regulatory subunit 1 (p85 alpha) | 5670 | 0.070 | -0.0603 | No |
| 5 | MAPK1 | MAPK1 Entrez,  Source | mitogen-activated protein kinase 1 | 6467 | 0.053 | -0.0851 | No |
| 6 | BCL2 | BCL2 Entrez,  Source | B-cell CLL/lymphoma 2 | 8414 | 0.018 | -0.1794 | No |
| 7 | RPS6KA1 | RPS6KA1 Entrez,  Source | ribosomal protein S6 kinase, 90kDa, polypeptide 1 | 9693 | -0.003 | -0.2438 | No |
| 8 | BAX | BAX Entrez,  Source | BCL2-associated X protein | 9820 | -0.005 | -0.2487 | No |
| 9 | BAD | BAD Entrez,  Source | BCL2-antagonist of cell death | 10235 | -0.012 | -0.2663 | No |
| 10 | PRKAR2B | PRKAR2B Entrez,  Source | protein kinase, cAMP-dependent, regulatory, type II, beta | 10285 | -0.013 | -0.2650 | No |
| 11 | PRKAR2A | PRKAR2A Entrez,  Source | protein kinase, cAMP-dependent, regulatory, type II, alpha | 10677 | -0.019 | -0.2792 | No |
| 12 | IGF1 | IGF1 Entrez,  Source | insulin-like growth factor 1 (somatomedin C) | 11748 | -0.039 | -0.3224 | No |
| 13 | IGF1R | IGF1R Entrez,  Source | insulin-like growth factor 1 receptor | 11811 | -0.040 | -0.3137 | No |
| 14 | KITLG | KITLG Entrez,  Source | KIT ligand | 12526 | -0.053 | -0.3344 | No |
| 15 | YWHAH | YWHAH Entrez,  Source | tyrosine 3-monooxygenase/tryptophan 5-monooxygenase activation protein, eta polypeptide | 13683 | -0.075 | -0.3710 | No |
| 16 | MAPK3 | MAPK3 Entrez,  Source | mitogen-activated protein kinase 3 | 14609 | -0.097 | -0.3893 | No |
| 17 | PRKACB | PRKACB Entrez,  Source | protein kinase, cAMP-dependent, catalytic, beta | 15299 | -0.115 | -0.3899 | Yes |
| 18 | ADCY1 | ADCY1 Entrez,  Source | adenylate cyclase 1 (brain) | 15374 | -0.118 | -0.3584 | Yes |
| 19 | BCL2L1 | BCL2L1 Entrez,  Source | BCL2-like 1 | 16145 | -0.142 | -0.3551 | Yes |
| 20 | PIK3CA | PIK3CA Entrez,  Source | phosphoinositide-3-kinase, catalytic, alpha polypeptide | 16332 | -0.149 | -0.3198 | Yes |
| 21 | PRKAR1A | PRKAR1A Entrez,  Source | protein kinase, cAMP-dependent, regulatory, type I, alpha (tissue specific extinguisher 1) | 16494 | -0.155 | -0.2816 | Yes |
| 22 | CSF2RB | CSF2RB Entrez,  Source | colony stimulating factor 2 receptor, beta, low-affinity (granulocyte-macrophage) | 16919 | -0.172 | -0.2518 | Yes |
| 23 | PRKACG | PRKACG Entrez,  Source | protein kinase, cAMP-dependent, catalytic, gamma | 17288 | -0.190 | -0.2135 | Yes |
| 24 | PIK3CG | PIK3CG Entrez,  Source | phosphoinositide-3-kinase, catalytic, gamma polypeptide | 17689 | -0.216 | -0.1691 | Yes |
| 25 | KIT | KIT Entrez,  Source | v-kit Hardy-Zuckerman 4 feline sarcoma viral oncogene homolog | 19461 | -0.882 | 0.0049 | Yes |
Table: GSEA details [plain text format]

  

Fig 2: BIOCARTA\_BAD\_PATHWAY      
 Blue-Pink O' Gram in the Space of the Analyzed GeneSet

  

Fig 3: BIOCARTA\_BAD\_PATHWAY: Random ES distribution      
 Gene set null distribution of ES for **BIOCARTA\_BAD\_PATHWAY**
