# Supplementary material for: Comprehensive analysis of β-catenin target genes in colorectal carcinoma cell lines with deregulated Wnt/β-catenin signaling
Source: BMC Genomics. 2014 Jan 28;15:74. doi: 10.1186/1471-2164-15-74 (PMC3909937; doi:10.1186/1471-2164-15-74)
Supplement: Additional file 4 — GSEA analysis using the Biocarta pathway database. This zipped file contains confirming data of the GSEA analysis. The names of the directories containing the files were composed of the term ‘GSEA’, the name of the cell line, e.g. DLD1, SW480, or LS174T, and the pathway database (Biocarta). Please use a web browser to view the files with the name ‘index.html’ in the corresponding directories to start exploring the data. [file 1471-2164-15-74-S4.zip › LS174T_Biocarta/BIOCARTA_CASPASE_PATHWAY.html]

Details for gene set BIOCARTA\_CASPASE\_PATHWAY[GSEA]

|  || Dataset | LS174T\_collapsed\_to\_symbols.class.cls#bg\_versus\_b |
| Phenotype | class.cls#bg\_versus\_b |
| Upregulated in class | b |
| GeneSet | BIOCARTA\_CASPASE\_PATHWAY |
| Enrichment Score (ES) | -0.46632123 |
| Normalized Enrichment Score (NES) | -1.2732509 |
| Nominal p-value | 0.1781076 |
| FDR q-value | 1.0 |
| FWER p-Value | 1.0 |
Table: GSEA Results Summary

  

Fig 1: Enrichment plot: BIOCARTA\_CASPASE\_PATHWAY      
 Profile of the Running ES Score & Positions of GeneSet Members on the Rank Ordered List

  

| PROBE | GENE SYMBOL | GENE\_TITLE | RANK IN GENE LIST | RANK METRIC SCORE | RUNNING ES | CORE ENRICHMENT || 1 | LMNB2 | LMNB2 Entrez,  Source | lamin B2 | 620 | 0.379 | 0.0407 | No |
| 2 | CASP3 | CASP3 Entrez,  Source | caspase 3, apoptosis-related cysteine peptidase | 806 | 0.332 | 0.0948 | No |
| 3 | CASP2 | CASP2 Entrez,  Source | caspase 2, apoptosis-related cysteine peptidase (neural precursor cell expressed, developmentally down-regulated 2) | 1074 | 0.289 | 0.1364 | No |
| 4 | LMNB1 | LMNB1 Entrez,  Source | lamin B1 | 1714 | 0.222 | 0.1461 | No |
| 5 | GZMB | GZMB Entrez,  Source | granzyme B (granzyme 2, cytotoxic T-lymphocyte-associated serine esterase 1) | 2031 | 0.200 | 0.1683 | No |
| 6 | PARP1 | PARP1 Entrez,  Source | poly (ADP-ribose) polymerase family, member 1 | 2729 | 0.161 | 0.1634 | No |
| 7 | CASP6 | CASP6 Entrez,  Source | caspase 6, apoptosis-related cysteine peptidase | 4107 | 0.110 | 0.1140 | No |
| 8 | DFFB | DFFB Entrez,  Source | DNA fragmentation factor, 40kDa, beta polypeptide (caspase-activated DNase) | 4110 | 0.110 | 0.1349 | No |
| 9 | PRF1 | PRF1 Entrez,  Source | perforin 1 (pore forming protein) | 4117 | 0.110 | 0.1555 | No |
| 10 | DFFA | DFFA Entrez,  Source | DNA fragmentation factor, 45kDa, alpha polypeptide | 5650 | 0.070 | 0.0906 | No |
| 11 | CYCS | CYCS Entrez,  Source | cytochrome c, somatic | 5730 | 0.069 | 0.0997 | No |
| 12 | CASP10 | CASP10 Entrez,  Source | caspase 10, apoptosis-related cysteine peptidase | 8224 | 0.021 | -0.0238 | No |
| 13 | CASP9 | CASP9 Entrez,  Source | caspase 9, apoptosis-related cysteine peptidase | 9868 | -0.006 | -0.1068 | No |
| 14 | APAF1 | APAF1 Entrez,  Source | apoptotic peptidase activating factor | 13272 | -0.067 | -0.2681 | No |
| 15 | CASP8 | CASP8 Entrez,  Source | caspase 8, apoptosis-related cysteine peptidase | 16962 | -0.174 | -0.4237 | Yes |
| 16 | BIRC2 | BIRC2 Entrez,  Source | baculoviral IAP repeat-containing 2 | 17796 | -0.224 | -0.4235 | Yes |
| 17 | LMNA | LMNA Entrez,  Source | lamin A/C | 17916 | -0.232 | -0.3852 | Yes |
| 18 | CASP7 | CASP7 Entrez,  Source | caspase 7, apoptosis-related cysteine peptidase | 18474 | -0.290 | -0.3583 | Yes |
| 19 | CASP4 | CASP4 Entrez,  Source | caspase 4, apoptosis-related cysteine peptidase | 18913 | -0.378 | -0.3085 | Yes |
| 20 | BIRC3 | BIRC3 Entrez,  Source | baculoviral IAP repeat-containing 3 | 18952 | -0.386 | -0.2367 | Yes |
| 21 | ARHGDIB | ARHGDIB Entrez,  Source | Rho GDP dissociation inhibitor (GDI) beta | 19244 | -0.527 | -0.1507 | Yes |
| 22 | CASP1 | CASP1 Entrez,  Source | caspase 1, apoptosis-related cysteine peptidase (interleukin 1, beta, convertase) | 19457 | -0.871 | 0.0051 | Yes |
Table: GSEA details [plain text format]

  

Fig 2: BIOCARTA\_CASPASE\_PATHWAY      
 Blue-Pink O' Gram in the Space of the Analyzed GeneSet

  

Fig 3: BIOCARTA\_CASPASE\_PATHWAY: Random ES distribution      
 Gene set null distribution of ES for **BIOCARTA\_CASPASE\_PATHWAY**
