# Supplementary material for: Comprehensive analysis of β-catenin target genes in colorectal carcinoma cell lines with deregulated Wnt/β-catenin signaling
Source: BMC Genomics. 2014 Jan 28;15:74. doi: 10.1186/1471-2164-15-74 (PMC3909937; doi:10.1186/1471-2164-15-74)
Supplement: Additional file 4 — GSEA analysis using the Biocarta pathway database. This zipped file contains confirming data of the GSEA analysis. The names of the directories containing the files were composed of the term ‘GSEA’, the name of the cell line, e.g. DLD1, SW480, or LS174T, and the pathway database (Biocarta). Please use a web browser to view the files with the name ‘index.html’ in the corresponding directories to start exploring the data. [file 1471-2164-15-74-S4.zip › LS174T_Biocarta/BIOCARTA_CCR5_PATHWAY.html]

Details for gene set BIOCARTA\_CCR5\_PATHWAY[GSEA]

|  || Dataset | LS174T\_collapsed\_to\_symbols.class.cls#bg\_versus\_b |
| Phenotype | class.cls#bg\_versus\_b |
| Upregulated in class | b |
| GeneSet | BIOCARTA\_CCR5\_PATHWAY |
| Enrichment Score (ES) | -0.5491101 |
| Normalized Enrichment Score (NES) | -1.3635006 |
| Nominal p-value | 0.11491936 |
| FDR q-value | 1.0 |
| FWER p-Value | 1.0 |
Table: GSEA Results Summary

  

Fig 1: Enrichment plot: BIOCARTA\_CCR5\_PATHWAY      
 Profile of the Running ES Score & Positions of GeneSet Members on the Rank Ordered List

  

| PROBE | GENE SYMBOL | GENE\_TITLE | RANK IN GENE LIST | RANK METRIC SCORE | RUNNING ES | CORE ENRICHMENT || 1 | PRKCA | PRKCA Entrez,  Source | protein kinase C, alpha | 2447 | 0.175 | -0.0403 | No |
| 2 | PLCG1 | PLCG1 Entrez,  Source | phospholipase C, gamma 1 | 6225 | 0.058 | -0.2053 | No |
| 3 | CALM3 | CALM3 Entrez,  Source | calmodulin 3 (phosphorylase kinase, delta) | 6946 | 0.044 | -0.2206 | No |
| 4 | CALM1 | CALM1 Entrez,  Source | calmodulin 1 (phosphorylase kinase, delta) | 8204 | 0.022 | -0.2742 | No |
| 5 | JUN | JUN Entrez,  Source | jun oncogene | 9654 | -0.003 | -0.3471 | No |
| 6 | CALM2 | CALM2 Entrez,  Source | calmodulin 2 (phosphorylase kinase, delta) | 9828 | -0.005 | -0.3533 | No |
| 7 | CXCL12 | CXCL12 Entrez,  Source | chemokine (C-X-C motif) ligand 12 (stromal cell-derived factor 1) | 10959 | -0.024 | -0.3996 | No |
| 8 | MAPK14 | MAPK14 Entrez,  Source | mitogen-activated protein kinase 14 | 13882 | -0.080 | -0.5105 | Yes |
| 9 | MAPK8 | MAPK8 Entrez,  Source | mitogen-activated protein kinase 8 | 13985 | -0.082 | -0.4758 | Yes |
| 10 | PTK2B | PTK2B Entrez,  Source | PTK2B protein tyrosine kinase 2 beta | 14768 | -0.101 | -0.4667 | Yes |
| 11 | CXCR4 | CXCR4 Entrez,  Source | chemokine (C-X-C motif) receptor 4 | 15586 | -0.124 | -0.4481 | Yes |
| 12 | CCL2 | CCL2 Entrez,  Source | chemokine (C-C motif) ligand 2 | 16386 | -0.151 | -0.4157 | Yes |
| 13 | GNAQ | GNAQ Entrez,  Source | guanine nucleotide binding protein (G protein), q polypeptide | 17067 | -0.178 | -0.3639 | Yes |
| 14 | CCL4 | CCL4 Entrez,  Source | chemokine (C-C motif) ligand 4 | 17337 | -0.194 | -0.2837 | Yes |
| 15 | FOS | FOS Entrez,  Source | v-fos FBJ murine osteosarcoma viral oncogene homolog | 19443 | -0.819 | 0.0058 | Yes |
Table: GSEA details [plain text format]

  

Fig 2: BIOCARTA\_CCR5\_PATHWAY      
 Blue-Pink O' Gram in the Space of the Analyzed GeneSet

  

Fig 3: BIOCARTA\_CCR5\_PATHWAY: Random ES distribution      
 Gene set null distribution of ES for **BIOCARTA\_CCR5\_PATHWAY**
