# Supplementary material for: Comprehensive analysis of β-catenin target genes in colorectal carcinoma cell lines with deregulated Wnt/β-catenin signaling
Source: BMC Genomics. 2014 Jan 28;15:74. doi: 10.1186/1471-2164-15-74 (PMC3909937; doi:10.1186/1471-2164-15-74)
Supplement: Additional file 4 — GSEA analysis using the Biocarta pathway database. This zipped file contains confirming data of the GSEA analysis. The names of the directories containing the files were composed of the term ‘GSEA’, the name of the cell line, e.g. DLD1, SW480, or LS174T, and the pathway database (Biocarta). Please use a web browser to view the files with the name ‘index.html’ in the corresponding directories to start exploring the data. [file 1471-2164-15-74-S4.zip › LS174T_Biocarta/BIOCARTA_CELLCYCLE_PATHWAY.html]

Details for gene set BIOCARTA\_CELLCYCLE\_PATHWAY[GSEA]

|  || Dataset | LS174T\_collapsed\_to\_symbols.class.cls#bg\_versus\_b |
| Phenotype | class.cls#bg\_versus\_b |
| Upregulated in class | bg |
| GeneSet | BIOCARTA\_CELLCYCLE\_PATHWAY |
| Enrichment Score (ES) | 0.4591165 |
| Normalized Enrichment Score (NES) | 1.2875233 |
| Nominal p-value | 0.14556962 |
| FDR q-value | 0.6829746 |
| FWER p-Value | 0.999 |
Table: GSEA Results Summary

  

Fig 1: Enrichment plot: BIOCARTA\_CELLCYCLE\_PATHWAY      
 Profile of the Running ES Score & Positions of GeneSet Members on the Rank Ordered List

  

| PROBE | GENE SYMBOL | GENE\_TITLE | RANK IN GENE LIST | RANK METRIC SCORE | RUNNING ES | CORE ENRICHMENT || 1 | RBL1 | RBL1 Entrez,  Source | retinoblastoma-like 1 (p107) | 189 | 0.592 | 0.0803 | Yes |
| 2 | CDKN2C | CDKN2C Entrez,  Source | cyclin-dependent kinase inhibitor 2C (p18, inhibits CDK4) | 215 | 0.569 | 0.1655 | Yes |
| 3 | CDC25A | CDC25A Entrez,  Source | cell division cycle 25A | 319 | 0.492 | 0.2350 | Yes |
| 4 | CDK2 | CDK2 Entrez,  Source | cyclin-dependent kinase 2 | 575 | 0.390 | 0.2813 | Yes |
| 5 | CCNE1 | CCNE1 Entrez,  Source | cyclin E1 | 815 | 0.330 | 0.3193 | Yes |
| 6 | E2F1 | E2F1 Entrez,  Source | E2F transcription factor 1 | 1057 | 0.292 | 0.3513 | Yes |
| 7 | CCNB1 | CCNB1 Entrez,  Source | cyclin B1 | 1091 | 0.286 | 0.3932 | Yes |
| 8 | CCND2 | CCND2 Entrez,  Source | cyclin D2 | 1200 | 0.273 | 0.4292 | Yes |
| 9 | CCND1 | CCND1 Entrez,  Source | cyclin D1 | 1372 | 0.254 | 0.4591 | Yes |
| 10 | CDK4 | CDK4 Entrez,  Source | cyclin-dependent kinase 4 | 2764 | 0.160 | 0.4122 | No |
| 11 | CDK6 | CDK6 Entrez,  Source | cyclin-dependent kinase 6 | 2942 | 0.151 | 0.4262 | No |
| 12 | TFDP1 | TFDP1 Entrez,  Source | transcription factor Dp-1 | 2968 | 0.150 | 0.4477 | No |
| 13 | CCND3 | CCND3 Entrez,  Source | cyclin D3 | 4136 | 0.109 | 0.4046 | No |
| 14 | CDKN1A | CDKN1A Entrez,  Source | cyclin-dependent kinase inhibitor 1A (p21, Cip1) | 4270 | 0.106 | 0.4138 | No |
| 15 | CDKN2D | CDKN2D Entrez,  Source | cyclin-dependent kinase inhibitor 2D (p19, inhibits CDK4) | 7006 | 0.043 | 0.2804 | No |
| 16 | CDK7 | CDK7 Entrez,  Source | cyclin-dependent kinase 7 (MO15 homolog, Xenopus laevis, cdk-activating kinase) | 7211 | 0.039 | 0.2759 | No |
| 17 | RB1 | RB1 Entrez,  Source | retinoblastoma 1 (including osteosarcoma) | 7450 | 0.035 | 0.2690 | No |
| 18 | CCNH | CCNH Entrez,  Source | cyclin H | 7484 | 0.034 | 0.2725 | No |
| 19 | CDKN2A | CDKN2A Entrez,  Source | cyclin-dependent kinase inhibitor 2A (melanoma, p16, inhibits CDK4) | 12732 | -0.057 | 0.0126 | No |
| 20 | CDKN1B | CDKN1B Entrez,  Source | cyclin-dependent kinase inhibitor 1B (p27, Kip1) | 13610 | -0.074 | -0.0210 | No |
| 21 | CCNA1 | CCNA1 Entrez,  Source | cyclin A1 | 15403 | -0.119 | -0.0947 | No |
| 22 | CDKN2B | CDKN2B Entrez,  Source | cyclin-dependent kinase inhibitor 2B (p15, inhibits CDK4) | 19546 | -2.022 | 0.0005 | No |
Table: GSEA details [plain text format]

  

Fig 2: BIOCARTA\_CELLCYCLE\_PATHWAY      
 Blue-Pink O' Gram in the Space of the Analyzed GeneSet

  

Fig 3: BIOCARTA\_CELLCYCLE\_PATHWAY: Random ES distribution      
 Gene set null distribution of ES for **BIOCARTA\_CELLCYCLE\_PATHWAY**
