# Supplementary material for: Comprehensive analysis of β-catenin target genes in colorectal carcinoma cell lines with deregulated Wnt/β-catenin signaling
Source: BMC Genomics. 2014 Jan 28;15:74. doi: 10.1186/1471-2164-15-74 (PMC3909937; doi:10.1186/1471-2164-15-74)
Supplement: Additional file 4 — GSEA analysis using the Biocarta pathway database. This zipped file contains confirming data of the GSEA analysis. The names of the directories containing the files were composed of the term ‘GSEA’, the name of the cell line, e.g. DLD1, SW480, or LS174T, and the pathway database (Biocarta). Please use a web browser to view the files with the name ‘index.html’ in the corresponding directories to start exploring the data. [file 1471-2164-15-74-S4.zip › LS174T_Biocarta/BIOCARTA_CHREBP2_PATHWAY.html]

Details for gene set BIOCARTA\_CHREBP2\_PATHWAY[GSEA]

|  || Dataset | LS174T\_collapsed\_to\_symbols.class.cls#bg\_versus\_b |
| Phenotype | class.cls#bg\_versus\_b |
| Upregulated in class | b |
| GeneSet | BIOCARTA\_CHREBP2\_PATHWAY |
| Enrichment Score (ES) | -0.34829113 |
| Normalized Enrichment Score (NES) | -1.0863295 |
| Nominal p-value | 0.32391715 |
| FDR q-value | 1.0 |
| FWER p-Value | 1.0 |
Table: GSEA Results Summary

  

Fig 1: Enrichment plot: BIOCARTA\_CHREBP2\_PATHWAY      
 Profile of the Running ES Score & Positions of GeneSet Members on the Rank Ordered List

  

| PROBE | GENE SYMBOL | GENE\_TITLE | RANK IN GENE LIST | RANK METRIC SCORE | RUNNING ES | CORE ENRICHMENT || 1 | PPP2R3B | PPP2R3B Entrez,  Source | protein phosphatase 2 (formerly 2A), regulatory subunit B'', beta | 1094 | 0.286 | 0.0079 | No |
| 2 | YWHAE | YWHAE Entrez,  Source | tyrosine 3-monooxygenase/tryptophan 5-monooxygenase activation protein, epsilon polypeptide | 1670 | 0.226 | 0.0289 | No |
| 3 | PPP2R2D | PPP2R2D Entrez,  Source | protein phosphatase 2, regulatory subunit B, delta isoform | 1776 | 0.217 | 0.0721 | No |
| 4 | MLXIPL | MLXIPL Entrez,  Source | MLX interacting protein-like | 2412 | 0.177 | 0.0791 | No |
| 5 | PPP2R4 | PPP2R4 Entrez,  Source | protein phosphatase 2A, regulatory subunit B' (PR 53) | 2816 | 0.157 | 0.0937 | No |
| 6 | PRKACA | PRKACA Entrez,  Source | protein kinase, cAMP-dependent, catalytic, alpha | 2909 | 0.153 | 0.1231 | No |
| 7 | PPP2R1A | PPP2R1A Entrez,  Source | protein phosphatase 2 (formerly 2A), regulatory subunit A (PR 65), alpha isoform | 5933 | 0.064 | -0.0175 | No |
| 8 | YWHAQ | YWHAQ Entrez,  Source | tyrosine 3-monooxygenase/tryptophan 5-monooxygenase activation protein, theta polypeptide | 6140 | 0.060 | -0.0146 | No |
| 9 | PPP2R5E | PPP2R5E Entrez,  Source | protein phosphatase 2, regulatory subunit B (B56), epsilon isoform | 6788 | 0.047 | -0.0372 | No |
| 10 | PPP2R5C | PPP2R5C Entrez,  Source | protein phosphatase 2, regulatory subunit B (B56), gamma isoform | 6849 | 0.046 | -0.0299 | No |
| 11 | PRKAG3 | PRKAG3 Entrez,  Source | protein kinase, AMP-activated, gamma 3 non-catalytic subunit | 6968 | 0.044 | -0.0261 | No |
| 12 | PPP2R2C | PPP2R2C Entrez,  Source | protein phosphatase 2 (formerly 2A), regulatory subunit B (PR 52), gamma isoform | 7933 | 0.027 | -0.0696 | No |
| 13 | PPP2R5D | PPP2R5D Entrez,  Source | protein phosphatase 2, regulatory subunit B (B56), delta isoform | 8157 | 0.023 | -0.0759 | No |
| 14 | PPP2CA | PPP2CA Entrez,  Source | protein phosphatase 2 (formerly 2A), catalytic subunit, alpha isoform | 8170 | 0.023 | -0.0715 | No |
| 15 | YWHAB | YWHAB Entrez,  Source | tyrosine 3-monooxygenase/tryptophan 5-monooxygenase activation protein, beta polypeptide | 8732 | 0.012 | -0.0976 | No |
| 16 | FASN | FASN Entrez,  Source | fatty acid synthase | 8757 | 0.012 | -0.0963 | No |
| 17 | ACACA | ACACA Entrez,  Source | acetyl-Coenzyme A carboxylase alpha | 9023 | 0.007 | -0.1082 | No |
| 18 | PPP2R5A | PPP2R5A Entrez,  Source | protein phosphatase 2, regulatory subunit B (B56), alpha isoform | 9629 | -0.002 | -0.1387 | No |
| 19 | PPP2R2A | PPP2R2A Entrez,  Source | protein phosphatase 2 (formerly 2A), regulatory subunit B (PR 52), alpha isoform | 10274 | -0.013 | -0.1689 | No |
| 20 | PRKAR2B | PRKAR2B Entrez,  Source | protein kinase, cAMP-dependent, regulatory, type II, beta | 10285 | -0.013 | -0.1666 | No |
| 21 | PPP2R2B | PPP2R2B Entrez,  Source | protein phosphatase 2 (formerly 2A), regulatory subunit B (PR 52), beta isoform | 10446 | -0.015 | -0.1713 | No |
| 22 | PPP2R1B | PPP2R1B Entrez,  Source | protein phosphatase 2 (formerly 2A), regulatory subunit A (PR 65), beta isoform | 10533 | -0.017 | -0.1719 | No |
| 23 | PRKAR2A | PRKAR2A Entrez,  Source | protein kinase, cAMP-dependent, regulatory, type II, alpha | 10677 | -0.019 | -0.1749 | No |
| 24 | GCK | GCK Entrez,  Source | glucokinase (hexokinase 4, maturity onset diabetes of the young 2) | 11631 | -0.036 | -0.2157 | No |
| 25 | MLX | MLX Entrez,  Source | MAX-like protein X | 11819 | -0.040 | -0.2164 | No |
| 26 | YWHAG | YWHAG Entrez,  Source | tyrosine 3-monooxygenase/tryptophan 5-monooxygenase activation protein, gamma polypeptide | 12192 | -0.047 | -0.2250 | No |
| 27 | PKLR | PKLR Entrez,  Source | pyruvate kinase, liver and RBC | 12811 | -0.058 | -0.2436 | No |
| 28 | YWHAZ | YWHAZ Entrez,  Source | tyrosine 3-monooxygenase/tryptophan 5-monooxygenase activation protein, zeta polypeptide | 13167 | -0.065 | -0.2473 | No |
| 29 | YWHAH | YWHAH Entrez,  Source | tyrosine 3-monooxygenase/tryptophan 5-monooxygenase activation protein, eta polypeptide | 13683 | -0.075 | -0.2568 | No |
| 30 | PPP2CB | PPP2CB Entrez,  Source | protein phosphatase 2 (formerly 2A), catalytic subunit, beta isoform | 14372 | -0.091 | -0.2716 | No |
| 31 | PRKACB | PRKACB Entrez,  Source | protein kinase, cAMP-dependent, catalytic, beta | 15299 | -0.115 | -0.2933 | No |
| 32 | PRKAG2 | PRKAG2 Entrez,  Source | protein kinase, AMP-activated, gamma 2 non-catalytic subunit | 16373 | -0.151 | -0.3146 | Yes |
| 33 | PRKAR1A | PRKAR1A Entrez,  Source | protein kinase, cAMP-dependent, regulatory, type I, alpha (tissue specific extinguisher 1) | 16494 | -0.155 | -0.2862 | Yes |
| 34 | PRKAG1 | PRKAG1 Entrez,  Source | protein kinase, AMP-activated, gamma 1 non-catalytic subunit | 16756 | -0.165 | -0.2627 | Yes |
| 35 | PRKACG | PRKACG Entrez,  Source | protein kinase, cAMP-dependent, catalytic, gamma | 17288 | -0.190 | -0.2473 | Yes |
| 36 | PPP2R3A | PPP2R3A Entrez,  Source | protein phosphatase 2 (formerly 2A), regulatory subunit B'', alpha | 17365 | -0.195 | -0.2076 | Yes |
| 37 | PRKAB1 | PRKAB1 Entrez,  Source | protein kinase, AMP-activated, beta 1 non-catalytic subunit | 17590 | -0.210 | -0.1722 | Yes |
| 38 | PRKAA1 | PRKAA1 Entrez,  Source | protein kinase, AMP-activated, alpha 1 catalytic subunit | 18106 | -0.248 | -0.1431 | Yes |
| 39 | PRKAA2 | PRKAA2 Entrez,  Source | protein kinase, AMP-activated, alpha 2 catalytic subunit | 18544 | -0.301 | -0.0981 | Yes |
| 40 | PPP2R5B | PPP2R5B Entrez,  Source | protein phosphatase 2, regulatory subunit B (B56), beta isoform | 18623 | -0.311 | -0.0324 | Yes |
| 41 | PRKAB2 | PRKAB2 Entrez,  Source | protein kinase, AMP-activated, beta 2 non-catalytic subunit | 18845 | -0.359 | 0.0364 | Yes |
Table: GSEA details [plain text format]

  

Fig 2: BIOCARTA\_CHREBP2\_PATHWAY      
 Blue-Pink O' Gram in the Space of the Analyzed GeneSet

  

Fig 3: BIOCARTA\_CHREBP2\_PATHWAY: Random ES distribution      
 Gene set null distribution of ES for **BIOCARTA\_CHREBP2\_PATHWAY**
