# Supplementary material for: Comprehensive analysis of β-catenin target genes in colorectal carcinoma cell lines with deregulated Wnt/β-catenin signaling
Source: BMC Genomics. 2014 Jan 28;15:74. doi: 10.1186/1471-2164-15-74 (PMC3909937; doi:10.1186/1471-2164-15-74)
Supplement: Additional file 4 — GSEA analysis using the Biocarta pathway database. This zipped file contains confirming data of the GSEA analysis. The names of the directories containing the files were composed of the term ‘GSEA’, the name of the cell line, e.g. DLD1, SW480, or LS174T, and the pathway database (Biocarta). Please use a web browser to view the files with the name ‘index.html’ in the corresponding directories to start exploring the data. [file 1471-2164-15-74-S4.zip › LS174T_Biocarta/BIOCARTA_COMP_PATHWAY.html]

Details for gene set BIOCARTA\_COMP\_PATHWAY[GSEA]

|  || Dataset | LS174T\_collapsed\_to\_symbols.class.cls#bg\_versus\_b |
| Phenotype | class.cls#bg\_versus\_b |
| Upregulated in class | b |
| GeneSet | BIOCARTA\_COMP\_PATHWAY |
| Enrichment Score (ES) | -0.5474343 |
| Normalized Enrichment Score (NES) | -1.3561323 |
| Nominal p-value | 0.13267326 |
| FDR q-value | 1.0 |
| FWER p-Value | 1.0 |
Table: GSEA Results Summary

  

Fig 1: Enrichment plot: BIOCARTA\_COMP\_PATHWAY      
 Profile of the Running ES Score & Positions of GeneSet Members on the Rank Ordered List

  

| PROBE | GENE SYMBOL | GENE\_TITLE | RANK IN GENE LIST | RANK METRIC SCORE | RUNNING ES | CORE ENRICHMENT || 1 | MBL2 | MBL2 Entrez,  Source | mannose-binding lectin (protein C) 2, soluble (opsonic defect) | 3260 | 0.139 | -0.0960 | No |
| 2 | C1QB | C1QB Entrez,  Source | complement component 1, q subcomponent, B chain | 3798 | 0.119 | -0.0629 | No |
| 3 | C1QC | C1QC Entrez,  Source | complement component 1, q subcomponent, C chain | 4323 | 0.104 | -0.0368 | No |
| 4 | C7 | C7 Entrez,  Source | complement component 7 | 7308 | 0.037 | -0.1704 | No |
| 5 | C9 | C9 Entrez,  Source | complement component 9 | 10204 | -0.012 | -0.3126 | No |
| 6 | MASP1 | MASP1 Entrez,  Source | mannan-binding lectin serine peptidase 1 (C4/C2 activating component of Ra-reactive factor) | 10393 | -0.015 | -0.3148 | No |
| 7 | C8A | C8A Entrez,  Source | complement component 8, alpha polypeptide | 10564 | -0.017 | -0.3146 | No |
| 8 | C1QA | C1QA Entrez,  Source | complement component 1, q subcomponent, A chain | 11096 | -0.026 | -0.3284 | No |
| 9 | C6 | C6 Entrez,  Source | complement component 6 | 11919 | -0.041 | -0.3494 | No |
| 10 | CFD | CFD Entrez,  Source | complement factor D (adipsin) | 12034 | -0.043 | -0.3330 | No |
| 11 | MASP2 | MASP2 Entrez,  Source | mannan-binding lectin serine peptidase 2 | 13302 | -0.068 | -0.3633 | No |
| 12 | C5 | C5 Entrez,  Source | complement component 5 | 16902 | -0.171 | -0.4601 | Yes |
| 13 | C2 | C2 Entrez,  Source | complement component 2 | 16973 | -0.174 | -0.3746 | Yes |
| 14 | C1S | C1S Entrez,  Source | complement component 1, s subcomponent | 17721 | -0.218 | -0.3017 | Yes |
| 15 | CFB | CFB Entrez,  Source | complement factor B | 19423 | -0.775 | 0.0068 | Yes |
Table: GSEA details [plain text format]

  

Fig 2: BIOCARTA\_COMP\_PATHWAY      
 Blue-Pink O' Gram in the Space of the Analyzed GeneSet

  

Fig 3: BIOCARTA\_COMP\_PATHWAY: Random ES distribution      
 Gene set null distribution of ES for **BIOCARTA\_COMP\_PATHWAY**
