# Supplementary material for: Comprehensive analysis of β-catenin target genes in colorectal carcinoma cell lines with deregulated Wnt/β-catenin signaling
Source: BMC Genomics. 2014 Jan 28;15:74. doi: 10.1186/1471-2164-15-74 (PMC3909937; doi:10.1186/1471-2164-15-74)
Supplement: Additional file 4 — GSEA analysis using the Biocarta pathway database. This zipped file contains confirming data of the GSEA analysis. The names of the directories containing the files were composed of the term ‘GSEA’, the name of the cell line, e.g. DLD1, SW480, or LS174T, and the pathway database (Biocarta). Please use a web browser to view the files with the name ‘index.html’ in the corresponding directories to start exploring the data. [file 1471-2164-15-74-S4.zip › LS174T_Biocarta/BIOCARTA_DEATH_PATHWAY.html]

Details for gene set BIOCARTA\_DEATH\_PATHWAY[GSEA]

|  || Dataset | LS174T\_collapsed\_to\_symbols.class.cls#bg\_versus\_b |
| Phenotype | class.cls#bg\_versus\_b |
| Upregulated in class | b |
| GeneSet | BIOCARTA\_DEATH\_PATHWAY |
| Enrichment Score (ES) | -0.41325966 |
| Normalized Enrichment Score (NES) | -1.2267673 |
| Nominal p-value | 0.18923934 |
| FDR q-value | 1.0 |
| FWER p-Value | 1.0 |
Table: GSEA Results Summary

  

Fig 1: Enrichment plot: BIOCARTA\_DEATH\_PATHWAY      
 Profile of the Running ES Score & Positions of GeneSet Members on the Rank Ordered List

  

| PROBE | GENE SYMBOL | GENE\_TITLE | RANK IN GENE LIST | RANK METRIC SCORE | RUNNING ES | CORE ENRICHMENT || 1 | CASP3 | CASP3 Entrez,  Source | caspase 3, apoptosis-related cysteine peptidase | 806 | 0.332 | 0.0309 | No |
| 2 | TRAF2 | TRAF2 Entrez,  Source | TNF receptor-associated factor 2 | 1344 | 0.258 | 0.0596 | No |
| 3 | CFLAR | CFLAR Entrez,  Source | CASP8 and FADD-like apoptosis regulator | 1642 | 0.229 | 0.0941 | No |
| 4 | TNFRSF10B | TNFRSF10B Entrez,  Source | tumor necrosis factor receptor superfamily, member 10b | 1708 | 0.222 | 0.1391 | No |
| 5 | NFKBIA | NFKBIA Entrez,  Source | nuclear factor of kappa light polypeptide gene enhancer in B-cells inhibitor, alpha | 3603 | 0.125 | 0.0694 | No |
| 6 | BID | BID Entrez,  Source | BH3 interacting domain death agonist | 4032 | 0.112 | 0.0719 | No |
| 7 | CASP6 | CASP6 Entrez,  Source | caspase 6, apoptosis-related cysteine peptidase | 4107 | 0.110 | 0.0920 | No |
| 8 | DFFB | DFFB Entrez,  Source | DNA fragmentation factor, 40kDa, beta polypeptide (caspase-activated DNase) | 4110 | 0.110 | 0.1158 | No |
| 9 | NFKB1 | NFKB1 Entrez,  Source | nuclear factor of kappa light polypeptide gene enhancer in B-cells 1 (p105) | 4927 | 0.088 | 0.0931 | No |
| 10 | DFFA | DFFA Entrez,  Source | DNA fragmentation factor, 45kDa, alpha polypeptide | 5650 | 0.070 | 0.0715 | No |
| 11 | CYCS | CYCS Entrez,  Source | cytochrome c, somatic | 5730 | 0.069 | 0.0824 | No |
| 12 | TNFRSF10A | TNFRSF10A Entrez,  Source | tumor necrosis factor receptor superfamily, member 10a | 6145 | 0.060 | 0.0742 | No |
| 13 | TNFSF12 | TNFSF12 Entrez,  Source | tumor necrosis factor (ligand) superfamily, member 12 | 7103 | 0.041 | 0.0341 | No |
| 14 | CASP10 | CASP10 Entrez,  Source | caspase 10, apoptosis-related cysteine peptidase | 8224 | 0.021 | -0.0186 | No |
| 15 | BCL2 | BCL2 Entrez,  Source | B-cell CLL/lymphoma 2 | 8414 | 0.018 | -0.0244 | No |
| 16 | RELA | RELA Entrez,  Source | v-rel reticuloendotheliosis viral oncogene homolog A, nuclear factor of kappa light polypeptide gene enhancer in B-cells 3, p65 (avian) | 8874 | 0.010 | -0.0458 | No |
| 17 | CHUK | CHUK Entrez,  Source | conserved helix-loop-helix ubiquitous kinase | 9260 | 0.003 | -0.0648 | No |
| 18 | MAP3K14 | MAP3K14 Entrez,  Source | mitogen-activated protein kinase kinase kinase 14 | 9477 | 0.000 | -0.0758 | No |
| 19 | CASP9 | CASP9 Entrez,  Source | caspase 9, apoptosis-related cysteine peptidase | 9868 | -0.006 | -0.0945 | No |
| 20 | FADD | FADD Entrez,  Source | Fas (TNFRSF6)-associated via death domain | 10215 | -0.012 | -0.1097 | No |
| 21 | RIPK1 | RIPK1 Entrez,  Source | receptor (TNFRSF)-interacting serine-threonine kinase 1 | 12898 | -0.060 | -0.2340 | No |
| 22 | APAF1 | APAF1 Entrez,  Source | apoptotic peptidase activating factor | 13272 | -0.067 | -0.2385 | No |
| 23 | TNFRSF25 | TNFRSF25 Entrez,  Source | tumor necrosis factor receptor superfamily, member 25 | 13356 | -0.069 | -0.2277 | No |
| 24 | CASP8 | CASP8 Entrez,  Source | caspase 8, apoptosis-related cysteine peptidase | 16962 | -0.174 | -0.3746 | Yes |
| 25 | TRADD | TRADD Entrez,  Source | TNFRSF1A-associated via death domain | 17718 | -0.218 | -0.3659 | Yes |
| 26 | BIRC2 | BIRC2 Entrez,  Source | baculoviral IAP repeat-containing 2 | 17796 | -0.224 | -0.3212 | Yes |
| 27 | LMNA | LMNA Entrez,  Source | lamin A/C | 17916 | -0.232 | -0.2768 | Yes |
| 28 | SPTAN1 | SPTAN1 Entrez,  Source | spectrin, alpha, non-erythrocytic 1 (alpha-fodrin) | 18122 | -0.249 | -0.2331 | Yes |
| 29 | CASP7 | CASP7 Entrez,  Source | caspase 7, apoptosis-related cysteine peptidase | 18474 | -0.290 | -0.1881 | Yes |
| 30 | GAS2 | GAS2 Entrez,  Source | growth arrest-specific 2 | 18732 | -0.333 | -0.1288 | Yes |
| 31 | BIRC3 | BIRC3 Entrez,  Source | baculoviral IAP repeat-containing 3 | 18952 | -0.386 | -0.0561 | Yes |
| 32 | TNFSF10 | TNFSF10 Entrez,  Source | tumor necrosis factor (ligand) superfamily, member 10 | 19009 | -0.400 | 0.0280 | Yes |
Table: GSEA details [plain text format]

  

Fig 2: BIOCARTA\_DEATH\_PATHWAY      
 Blue-Pink O' Gram in the Space of the Analyzed GeneSet

  

Fig 3: BIOCARTA\_DEATH\_PATHWAY: Random ES distribution      
 Gene set null distribution of ES for **BIOCARTA\_DEATH\_PATHWAY**
