# Supplementary material for: Comprehensive analysis of β-catenin target genes in colorectal carcinoma cell lines with deregulated Wnt/β-catenin signaling
Source: BMC Genomics. 2014 Jan 28;15:74. doi: 10.1186/1471-2164-15-74 (PMC3909937; doi:10.1186/1471-2164-15-74)
Supplement: Additional file 4 — GSEA analysis using the Biocarta pathway database. This zipped file contains confirming data of the GSEA analysis. The names of the directories containing the files were composed of the term ‘GSEA’, the name of the cell line, e.g. DLD1, SW480, or LS174T, and the pathway database (Biocarta). Please use a web browser to view the files with the name ‘index.html’ in the corresponding directories to start exploring the data. [file 1471-2164-15-74-S4.zip › LS174T_Biocarta/BIOCARTA_ECM_PATHWAY.html]

Details for gene set BIOCARTA\_ECM\_PATHWAY[GSEA]

|  || Dataset | LS174T\_collapsed\_to\_symbols.class.cls#bg\_versus\_b |
| Phenotype | class.cls#bg\_versus\_b |
| Upregulated in class | b |
| GeneSet | BIOCARTA\_ECM\_PATHWAY |
| Enrichment Score (ES) | -0.40416265 |
| Normalized Enrichment Score (NES) | -1.1218601 |
| Nominal p-value | 0.27889907 |
| FDR q-value | 1.0 |
| FWER p-Value | 1.0 |
Table: GSEA Results Summary

  

Fig 1: Enrichment plot: BIOCARTA\_ECM\_PATHWAY      
 Profile of the Running ES Score & Positions of GeneSet Members on the Rank Ordered List

  

| PROBE | GENE SYMBOL | GENE\_TITLE | RANK IN GENE LIST | RANK METRIC SCORE | RUNNING ES | CORE ENRICHMENT || 1 | SRC | SRC Entrez,  Source | v-src sarcoma (Schmidt-Ruppin A-2) viral oncogene homolog (avian) | 953 | 0.307 | 0.0854 | No |
| 2 | PIK3R1 | PIK3R1 Entrez,  Source | phosphoinositide-3-kinase, regulatory subunit 1 (p85 alpha) | 5670 | 0.070 | -0.1254 | No |
| 3 | RAF1 | RAF1 Entrez,  Source | v-raf-1 murine leukemia viral oncogene homolog 1 | 5946 | 0.064 | -0.1116 | No |
| 4 | MAPK1 | MAPK1 Entrez,  Source | mitogen-activated protein kinase 1 | 6467 | 0.053 | -0.1150 | No |
| 5 | SHC1 | SHC1 Entrez,  Source | SHC (Src homology 2 domain containing) transforming protein 1 | 6609 | 0.051 | -0.1002 | No |
| 6 | PFN1 | PFN1 Entrez,  Source | profilin 1 | 8847 | 0.010 | -0.2103 | No |
| 7 | HRAS | HRAS Entrez,  Source | v-Ha-ras Harvey rat sarcoma viral oncogene homolog | 9218 | 0.004 | -0.2275 | No |
| 8 | DIAPH1 | DIAPH1 Entrez,  Source | diaphanous homolog 1 (Drosophila) | 10694 | -0.020 | -0.2944 | No |
| 9 | TLN1 | TLN1 Entrez,  Source | talin 1 | 10873 | -0.022 | -0.2938 | No |
| 10 | RHOA | RHOA Entrez,  Source | ras homolog gene family, member A | 10998 | -0.024 | -0.2895 | No |
| 11 | FYN | FYN Entrez,  Source | FYN oncogene related to SRC, FGR, YES | 11139 | -0.027 | -0.2848 | No |
| 12 | MAP2K1 | MAP2K1 Entrez,  Source | mitogen-activated protein kinase kinase 1 | 11207 | -0.028 | -0.2759 | No |
| 13 | ROCK1 | ROCK1 Entrez,  Source | Rho-associated, coiled-coil containing protein kinase 1 | 12150 | -0.046 | -0.3041 | No |
| 14 | ARHGAP5 | ARHGAP5 Entrez,  Source | Rho GTPase activating protein 5 | 12836 | -0.059 | -0.3134 | No |
| 15 | MAPK3 | MAPK3 Entrez,  Source | mitogen-activated protein kinase 3 | 14609 | -0.097 | -0.3619 | Yes |
| 16 | ITGB1 | ITGB1 Entrez,  Source | integrin, beta 1 (fibronectin receptor, beta polypeptide, antigen CD29 includes MDF2, MSK12) | 14691 | -0.099 | -0.3228 | Yes |
| 17 | PTK2 | PTK2 Entrez,  Source | PTK2 protein tyrosine kinase 2 | 14708 | -0.099 | -0.2802 | Yes |
| 18 | MYL2 | MYL2 Entrez,  Source | myosin, light chain 2, regulatory, cardiac, slow | 15942 | -0.136 | -0.2842 | Yes |
| 19 | PIK3CA | PIK3CA Entrez,  Source | phosphoinositide-3-kinase, catalytic, alpha polypeptide | 16332 | -0.149 | -0.2389 | Yes |
| 20 | MYLK | MYLK Entrez,  Source | myosin, light chain kinase | 17341 | -0.194 | -0.2058 | Yes |
| 21 | PXN | PXN Entrez,  Source | paxillin | 17550 | -0.206 | -0.1265 | Yes |
| 22 | PIK3CG | PIK3CG Entrez,  Source | phosphoinositide-3-kinase, catalytic, gamma polypeptide | 17689 | -0.216 | -0.0392 | Yes |
| 23 | GSN | GSN Entrez,  Source | gelsolin (amyloidosis, Finnish type) | 18601 | -0.309 | 0.0489 | Yes |
Table: GSEA details [plain text format]

  

Fig 2: BIOCARTA\_ECM\_PATHWAY      
 Blue-Pink O' Gram in the Space of the Analyzed GeneSet

  

Fig 3: BIOCARTA\_ECM\_PATHWAY: Random ES distribution      
 Gene set null distribution of ES for **BIOCARTA\_ECM\_PATHWAY**
