# Supplementary material for: Comprehensive analysis of β-catenin target genes in colorectal carcinoma cell lines with deregulated Wnt/β-catenin signaling
Source: BMC Genomics. 2014 Jan 28;15:74. doi: 10.1186/1471-2164-15-74 (PMC3909937; doi:10.1186/1471-2164-15-74)
Supplement: Additional file 4 — GSEA analysis using the Biocarta pathway database. This zipped file contains confirming data of the GSEA analysis. The names of the directories containing the files were composed of the term ‘GSEA’, the name of the cell line, e.g. DLD1, SW480, or LS174T, and the pathway database (Biocarta). Please use a web browser to view the files with the name ‘index.html’ in the corresponding directories to start exploring the data. [file 1471-2164-15-74-S4.zip › LS174T_Biocarta/BIOCARTA_ETS_PATHWAY.html]

Details for gene set BIOCARTA\_ETS\_PATHWAY[GSEA]

|  || Dataset | LS174T\_collapsed\_to\_symbols.class.cls#bg\_versus\_b |
| Phenotype | class.cls#bg\_versus\_b |
| Upregulated in class | bg |
| GeneSet | BIOCARTA\_ETS\_PATHWAY |
| Enrichment Score (ES) | 0.5105173 |
| Normalized Enrichment Score (NES) | 1.3626422 |
| Nominal p-value | 0.11157025 |
| FDR q-value | 0.5448531 |
| FWER p-Value | 0.998 |
Table: GSEA Results Summary

  

Fig 1: Enrichment plot: BIOCARTA\_ETS\_PATHWAY      
 Profile of the Running ES Score & Positions of GeneSet Members on the Rank Ordered List

  

| PROBE | GENE SYMBOL | GENE\_TITLE | RANK IN GENE LIST | RANK METRIC SCORE | RUNNING ES | CORE ENRICHMENT || 1 | RBL1 | RBL1 Entrez,  Source | retinoblastoma-like 1 (p107) | 189 | 0.592 | 0.1480 | Yes |
| 2 | ETS2 | ETS2 Entrez,  Source | v-ets erythroblastosis virus E26 oncogene homolog 2 (avian) | 256 | 0.543 | 0.2892 | Yes |
| 3 | E2F1 | E2F1 Entrez,  Source | E2F transcription factor 1 | 1057 | 0.292 | 0.3261 | Yes |
| 4 | DDX20 | DDX20 Entrez,  Source | DEAD (Asp-Glu-Ala-Asp) box polypeptide 20 | 1112 | 0.283 | 0.3988 | Yes |
| 5 | ETV3 | ETV3 Entrez,  Source | ets variant gene 3 | 2063 | 0.198 | 0.4031 | Yes |
| 6 | NCOR2 | NCOR2 Entrez,  Source | nuclear receptor co-repressor 2 | 2408 | 0.177 | 0.4327 | Yes |
| 7 | SIN3A | SIN3A Entrez,  Source | SIN3 homolog A, transcription regulator (yeast) | 2494 | 0.173 | 0.4743 | Yes |
| 8 | HDAC5 | HDAC5 Entrez,  Source | histone deacetylase 5 | 2989 | 0.149 | 0.4888 | Yes |
| 9 | HDAC2 | HDAC2 Entrez,  Source | histone deacetylase 2 | 3459 | 0.131 | 0.4998 | Yes |
| 10 | ETS1 | ETS1 Entrez,  Source | v-ets erythroblastosis virus E26 oncogene homolog 1 (avian) | 3859 | 0.117 | 0.5105 | Yes |
| 11 | CSF1 | CSF1 Entrez,  Source | colony stimulating factor 1 (macrophage) | 5985 | 0.063 | 0.4186 | No |
| 12 | RBL2 | RBL2 Entrez,  Source | retinoblastoma-like 2 (p130) | 6030 | 0.062 | 0.4329 | No |
| 13 | E2F4 | E2F4 Entrez,  Source | E2F transcription factor 4, p107/p130-binding | 6528 | 0.052 | 0.4214 | No |
| 14 | HRAS | HRAS Entrez,  Source | v-Ha-ras Harvey rat sarcoma viral oncogene homolog | 9218 | 0.004 | 0.2848 | No |
| 15 | JUN | JUN Entrez,  Source | jun oncogene | 9654 | -0.003 | 0.2633 | No |
| 16 | SIN3B | SIN3B Entrez,  Source | SIN3 homolog B, transcription regulator (yeast) | 11718 | -0.038 | 0.1678 | No |
| 17 | CSF1R | CSF1R Entrez,  Source | colony stimulating factor 1 receptor, formerly McDonough feline sarcoma viral (v-fms) oncogene homolog | 12716 | -0.057 | 0.1318 | No |
| 18 | FOS | FOS Entrez,  Source | v-fos FBJ murine osteosarcoma viral oncogene homolog | 19443 | -0.819 | 0.0058 | No |
Table: GSEA details [plain text format]

  

Fig 2: BIOCARTA\_ETS\_PATHWAY      
 Blue-Pink O' Gram in the Space of the Analyzed GeneSet

  

Fig 3: BIOCARTA\_ETS\_PATHWAY: Random ES distribution      
 Gene set null distribution of ES for **BIOCARTA\_ETS\_PATHWAY**
