# Supplementary material for: Comprehensive analysis of β-catenin target genes in colorectal carcinoma cell lines with deregulated Wnt/β-catenin signaling
Source: BMC Genomics. 2014 Jan 28;15:74. doi: 10.1186/1471-2164-15-74 (PMC3909937; doi:10.1186/1471-2164-15-74)
Supplement: Additional file 4 — GSEA analysis using the Biocarta pathway database. This zipped file contains confirming data of the GSEA analysis. The names of the directories containing the files were composed of the term ‘GSEA’, the name of the cell line, e.g. DLD1, SW480, or LS174T, and the pathway database (Biocarta). Please use a web browser to view the files with the name ‘index.html’ in the corresponding directories to start exploring the data. [file 1471-2164-15-74-S4.zip › LS174T_Biocarta/BIOCARTA_INFLAM_PATHWAY.html]

Details for gene set BIOCARTA\_INFLAM\_PATHWAY[GSEA]

|  || Dataset | LS174T\_collapsed\_to\_symbols.class.cls#bg\_versus\_b |
| Phenotype | class.cls#bg\_versus\_b |
| Upregulated in class | bg |
| GeneSet | BIOCARTA\_INFLAM\_PATHWAY |
| Enrichment Score (ES) | 0.38319713 |
| Normalized Enrichment Score (NES) | 1.1426144 |
| Nominal p-value | 0.2648402 |
| FDR q-value | 0.92024285 |
| FWER p-Value | 1.0 |
Table: GSEA Results Summary

  

Fig 1: Enrichment plot: BIOCARTA\_INFLAM\_PATHWAY      
 Profile of the Running ES Score & Positions of GeneSet Members on the Rank Ordered List

  

| PROBE | GENE SYMBOL | GENE\_TITLE | RANK IN GENE LIST | RANK METRIC SCORE | RUNNING ES | CORE ENRICHMENT || 1 | IL15 | IL15 Entrez,  Source | interleukin 15 | 701 | 0.353 | 0.0665 | Yes |
| 2 | IL5 | IL5 Entrez,  Source | interleukin 5 (colony-stimulating factor, eosinophil) | 805 | 0.332 | 0.1574 | Yes |
| 3 | HLA-DRB1 | HLA-DRB1 Entrez,  Source | major histocompatibility complex, class II, DR beta 1 | 1080 | 0.288 | 0.2266 | Yes |
| 4 | IFNG | IFNG Entrez,  Source | interferon, gamma | 1093 | 0.286 | 0.3089 | Yes |
| 5 | IL3 | IL3 Entrez,  Source | interleukin 3 (colony-stimulating factor, multiple) | 1194 | 0.274 | 0.3832 | Yes |
| 6 | PDGFA | PDGFA Entrez,  Source | platelet-derived growth factor alpha polypeptide | 3214 | 0.140 | 0.3205 | No |
| 7 | LTA | LTA Entrez,  Source | lymphotoxin alpha (TNF superfamily, member 1) | 3241 | 0.139 | 0.3595 | No |
| 8 | IL6 | IL6 Entrez,  Source | interleukin 6 (interferon, beta 2) | 4628 | 0.095 | 0.3160 | No |
| 9 | HLA-DRA | HLA-DRA Entrez,  Source | major histocompatibility complex, class II, DR alpha | 4941 | 0.087 | 0.3254 | No |
| 10 | CSF1 | CSF1 Entrez,  Source | colony stimulating factor 1 (macrophage) | 5985 | 0.063 | 0.2902 | No |
| 11 | IL7 | IL7 Entrez,  Source | interleukin 7 | 6993 | 0.044 | 0.2513 | No |
| 12 | IL10 | IL10 Entrez,  Source | interleukin 10 | 7354 | 0.036 | 0.2434 | No |
| 13 | TNF | TNF Entrez,  Source | tumor necrosis factor (TNF superfamily, member 2) | 8127 | 0.023 | 0.2105 | No |
| 14 | IL1A | IL1A Entrez,  Source | interleukin 1, alpha | 8201 | 0.022 | 0.2132 | No |
| 15 | IL2 | IL2 Entrez,  Source | interleukin 2 | 8748 | 0.012 | 0.1886 | No |
| 16 | CSF2 | CSF2 Entrez,  Source | colony stimulating factor 2 (granulocyte-macrophage) | 9131 | 0.006 | 0.1707 | No |
| 17 | IL12B | IL12B Entrez,  Source | interleukin 12B (natural killer cell stimulatory factor 2, cytotoxic lymphocyte maturation factor 2, p40) | 9177 | 0.005 | 0.1698 | No |
| 18 | CSF3 | CSF3 Entrez,  Source | colony stimulating factor 3 (granulocyte) | 9633 | -0.002 | 0.1471 | No |
| 19 | CD4 | CD4 Entrez,  Source | CD4 molecule | 10804 | -0.021 | 0.0934 | No |
| 20 | IL12A | IL12A Entrez,  Source | interleukin 12A (natural killer cell stimulatory factor 1, cytotoxic lymphocyte maturation factor 1, p35) | 11052 | -0.026 | 0.0881 | No |
| 21 | TGFB1 | TGFB1 Entrez,  Source | transforming growth factor, beta 1 (Camurati-Engelmann disease) | 12328 | -0.049 | 0.0371 | No |
| 22 | IL13 | IL13 Entrez,  Source | interleukin 13 | 13935 | -0.081 | -0.0217 | No |
| 23 | IL11 | IL11 Entrez,  Source | interleukin 11 | 13957 | -0.081 | 0.0008 | No |
| 24 | IL4 | IL4 Entrez,  Source | interleukin 4 | 14206 | -0.087 | 0.0134 | No |
| 25 | TGFB3 | TGFB3 Entrez,  Source | transforming growth factor, beta 3 | 14231 | -0.088 | 0.0376 | No |
| 26 | IFNB1 | IFNB1 Entrez,  Source | interferon, beta 1, fibroblast | 15572 | -0.124 | 0.0049 | No |
| 27 | IL8 | IL8 Entrez,  Source | interleukin 8 | 16383 | -0.151 | 0.0071 | No |
| 28 | IFNA1 | IFNA1 Entrez,  Source | interferon, alpha 1 | 17399 | -0.197 | 0.0122 | No |
| 29 | TGFB2 | TGFB2 Entrez,  Source | transforming growth factor, beta 2 | 18757 | -0.339 | 0.0409 | No |
Table: GSEA details [plain text format]

  

Fig 2: BIOCARTA\_INFLAM\_PATHWAY      
 Blue-Pink O' Gram in the Space of the Analyzed GeneSet

  

Fig 3: BIOCARTA\_INFLAM\_PATHWAY: Random ES distribution      
 Gene set null distribution of ES for **BIOCARTA\_INFLAM\_PATHWAY**
