# Supplementary material for: Comprehensive analysis of β-catenin target genes in colorectal carcinoma cell lines with deregulated Wnt/β-catenin signaling
Source: BMC Genomics. 2014 Jan 28;15:74. doi: 10.1186/1471-2164-15-74 (PMC3909937; doi:10.1186/1471-2164-15-74)
Supplement: Additional file 4 — GSEA analysis using the Biocarta pathway database. This zipped file contains confirming data of the GSEA analysis. The names of the directories containing the files were composed of the term ‘GSEA’, the name of the cell line, e.g. DLD1, SW480, or LS174T, and the pathway database (Biocarta). Please use a web browser to view the files with the name ‘index.html’ in the corresponding directories to start exploring the data. [file 1471-2164-15-74-S4.zip › LS174T_Biocarta/BIOCARTA_NGF_PATHWAY.html]

Details for gene set BIOCARTA\_NGF\_PATHWAY[GSEA]

|  || Dataset | LS174T\_collapsed\_to\_symbols.class.cls#bg\_versus\_b |
| Phenotype | class.cls#bg\_versus\_b |
| Upregulated in class | b |
| GeneSet | BIOCARTA\_NGF\_PATHWAY |
| Enrichment Score (ES) | -0.5410257 |
| Normalized Enrichment Score (NES) | -1.3710903 |
| Nominal p-value | 0.112426035 |
| FDR q-value | 1.0 |
| FWER p-Value | 1.0 |
Table: GSEA Results Summary

  

Fig 1: Enrichment plot: BIOCARTA\_NGF\_PATHWAY      
 Profile of the Running ES Score & Positions of GeneSet Members on the Rank Ordered List

  

| PROBE | GENE SYMBOL | GENE\_TITLE | RANK IN GENE LIST | RANK METRIC SCORE | RUNNING ES | CORE ENRICHMENT || 1 | PIK3R1 | PIK3R1 Entrez,  Source | phosphoinositide-3-kinase, regulatory subunit 1 (p85 alpha) | 5670 | 0.070 | -0.2513 | No |
| 2 | RAF1 | RAF1 Entrez,  Source | v-raf-1 murine leukemia viral oncogene homolog 1 | 5946 | 0.064 | -0.2301 | No |
| 3 | PLCG1 | PLCG1 Entrez,  Source | phospholipase C, gamma 1 | 6225 | 0.058 | -0.2120 | No |
| 4 | SHC1 | SHC1 Entrez,  Source | SHC (Src homology 2 domain containing) transforming protein 1 | 6609 | 0.051 | -0.2036 | No |
| 5 | GRB2 | GRB2 Entrez,  Source | growth factor receptor-bound protein 2 | 8140 | 0.023 | -0.2691 | No |
| 6 | CSNK2A1 | CSNK2A1 Entrez,  Source | casein kinase 2, alpha 1 polypeptide | 9044 | 0.007 | -0.3115 | No |
| 7 | ELK1 | ELK1 Entrez,  Source | ELK1, member of ETS oncogene family | 9100 | 0.006 | -0.3110 | No |
| 8 | HRAS | HRAS Entrez,  Source | v-Ha-ras Harvey rat sarcoma viral oncogene homolog | 9218 | 0.004 | -0.3147 | No |
| 9 | SOS1 | SOS1 Entrez,  Source | son of sevenless homolog 1 (Drosophila) | 9435 | 0.001 | -0.3254 | No |
| 10 | JUN | JUN Entrez,  Source | jun oncogene | 9654 | -0.003 | -0.3351 | No |
| 11 | MAP2K1 | MAP2K1 Entrez,  Source | mitogen-activated protein kinase kinase 1 | 11207 | -0.028 | -0.3989 | No |
| 12 | MAPK8 | MAPK8 Entrez,  Source | mitogen-activated protein kinase 8 | 13985 | -0.082 | -0.4955 | Yes |
| 13 | MAPK3 | MAPK3 Entrez,  Source | mitogen-activated protein kinase 3 | 14609 | -0.097 | -0.4738 | Yes |
| 14 | NGFR | NGFR Entrez,  Source | nerve growth factor receptor (TNFR superfamily, member 16) | 15731 | -0.129 | -0.4600 | Yes |
| 15 | PIK3CA | PIK3CA Entrez,  Source | phosphoinositide-3-kinase, catalytic, alpha polypeptide | 16332 | -0.149 | -0.4080 | Yes |
| 16 | PIK3CG | PIK3CG Entrez,  Source | phosphoinositide-3-kinase, catalytic, gamma polypeptide | 17689 | -0.216 | -0.3577 | Yes |
| 17 | FOS | FOS Entrez,  Source | v-fos FBJ murine osteosarcoma viral oncogene homolog | 19443 | -0.819 | 0.0058 | Yes |
Table: GSEA details [plain text format]

  

Fig 2: BIOCARTA\_NGF\_PATHWAY      
 Blue-Pink O' Gram in the Space of the Analyzed GeneSet

  

Fig 3: BIOCARTA\_NGF\_PATHWAY: Random ES distribution      
 Gene set null distribution of ES for **BIOCARTA\_NGF\_PATHWAY**
