# Supplementary material for: Comprehensive analysis of β-catenin target genes in colorectal carcinoma cell lines with deregulated Wnt/β-catenin signaling
Source: BMC Genomics. 2014 Jan 28;15:74. doi: 10.1186/1471-2164-15-74 (PMC3909937; doi:10.1186/1471-2164-15-74)
Supplement: Additional file 4 — GSEA analysis using the Biocarta pathway database. This zipped file contains confirming data of the GSEA analysis. The names of the directories containing the files were composed of the term ‘GSEA’, the name of the cell line, e.g. DLD1, SW480, or LS174T, and the pathway database (Biocarta). Please use a web browser to view the files with the name ‘index.html’ in the corresponding directories to start exploring the data. [file 1471-2164-15-74-S4.zip › LS174T_Biocarta/BIOCARTA_P53_PATHWAY.html]

Details for gene set BIOCARTA\_P53\_PATHWAY[GSEA]

|  || Dataset | LS174T\_collapsed\_to\_symbols.class.cls#bg\_versus\_b |
| Phenotype | class.cls#bg\_versus\_b |
| Upregulated in class | bg |
| GeneSet | BIOCARTA\_P53\_PATHWAY |
| Enrichment Score (ES) | 0.6755141 |
| Normalized Enrichment Score (NES) | 1.7475493 |
| Nominal p-value | 0.010799136 |
| FDR q-value | 0.06765512 |
| FWER p-Value | 0.296 |
Table: GSEA Results Summary

  

Fig 1: Enrichment plot: BIOCARTA\_P53\_PATHWAY      
 Profile of the Running ES Score & Positions of GeneSet Members on the Rank Ordered List

  

| PROBE | GENE SYMBOL | GENE\_TITLE | RANK IN GENE LIST | RANK METRIC SCORE | RUNNING ES | CORE ENRICHMENT || 1 | CDK2 | CDK2 Entrez,  Source | cyclin-dependent kinase 2 | 575 | 0.390 | 0.1360 | Yes |
| 2 | CCNE1 | CCNE1 Entrez,  Source | cyclin E1 | 815 | 0.330 | 0.2637 | Yes |
| 3 | PCNA | PCNA Entrez,  Source | proliferating cell nuclear antigen | 844 | 0.326 | 0.4005 | Yes |
| 4 | E2F1 | E2F1 Entrez,  Source | E2F transcription factor 1 | 1057 | 0.292 | 0.5133 | Yes |
| 5 | CCND1 | CCND1 Entrez,  Source | cyclin D1 | 1372 | 0.254 | 0.6050 | Yes |
| 6 | TP53 | TP53 Entrez,  Source | tumor protein p53 (Li-Fraumeni syndrome) | 2459 | 0.175 | 0.6234 | Yes |
| 7 | CDK4 | CDK4 Entrez,  Source | cyclin-dependent kinase 4 | 2764 | 0.160 | 0.6755 | Yes |
| 8 | CDKN1A | CDKN1A Entrez,  Source | cyclin-dependent kinase inhibitor 1A (p21, Cip1) | 4270 | 0.106 | 0.6432 | No |
| 9 | MDM2 | MDM2 Entrez,  Source | Mdm2, transformed 3T3 cell double minute 2, p53 binding protein (mouse) | 6876 | 0.046 | 0.5293 | No |
| 10 | RB1 | RB1 Entrez,  Source | retinoblastoma 1 (including osteosarcoma) | 7450 | 0.035 | 0.5147 | No |
| 11 | BCL2 | BCL2 Entrez,  Source | B-cell CLL/lymphoma 2 | 8414 | 0.018 | 0.4729 | No |
| 12 | TIMP3 | TIMP3 Entrez,  Source | TIMP metallopeptidase inhibitor 3 (Sorsby fundus dystrophy, pseudoinflammatory) | 8716 | 0.012 | 0.4628 | No |
| 13 | BAX | BAX Entrez,  Source | BCL2-associated X protein | 9820 | -0.005 | 0.4085 | No |
| 14 | GADD45A | GADD45A Entrez,  Source | growth arrest and DNA-damage-inducible, alpha | 12506 | -0.052 | 0.2933 | No |
| 15 | APAF1 | APAF1 Entrez,  Source | apoptotic peptidase activating factor | 13272 | -0.067 | 0.2827 | No |
| 16 | ATM | ATM Entrez,  Source | ataxia telangiectasia mutated (includes complementation groups A, C and D) | 14388 | -0.092 | 0.2645 | No |
Table: GSEA details [plain text format]

  

Fig 2: BIOCARTA\_P53\_PATHWAY      
 Blue-Pink O' Gram in the Space of the Analyzed GeneSet

  

Fig 3: BIOCARTA\_P53\_PATHWAY: Random ES distribution      
 Gene set null distribution of ES for **BIOCARTA\_P53\_PATHWAY**
