# Supplementary material for: Comprehensive analysis of β-catenin target genes in colorectal carcinoma cell lines with deregulated Wnt/β-catenin signaling
Source: BMC Genomics. 2014 Jan 28;15:74. doi: 10.1186/1471-2164-15-74 (PMC3909937; doi:10.1186/1471-2164-15-74)
Supplement: Additional file 4 — GSEA analysis using the Biocarta pathway database. This zipped file contains confirming data of the GSEA analysis. The names of the directories containing the files were composed of the term ‘GSEA’, the name of the cell line, e.g. DLD1, SW480, or LS174T, and the pathway database (Biocarta). Please use a web browser to view the files with the name ‘index.html’ in the corresponding directories to start exploring the data. [file 1471-2164-15-74-S4.zip › LS174T_Biocarta/BIOCARTA_PGC1A_PATHWAY.html]

Details for gene set BIOCARTA\_PGC1A\_PATHWAY[GSEA]

|  || Dataset | LS174T\_collapsed\_to\_symbols.class.cls#bg\_versus\_b |
| Phenotype | class.cls#bg\_versus\_b |
| Upregulated in class | b |
| GeneSet | BIOCARTA\_PGC1A\_PATHWAY |
| Enrichment Score (ES) | -0.46447664 |
| Normalized Enrichment Score (NES) | -1.2489866 |
| Nominal p-value | 0.18264015 |
| FDR q-value | 1.0 |
| FWER p-Value | 1.0 |
Table: GSEA Results Summary

  

Fig 1: Enrichment plot: BIOCARTA\_PGC1A\_PATHWAY      
 Profile of the Running ES Score & Positions of GeneSet Members on the Rank Ordered List

  

| PROBE | GENE SYMBOL | GENE\_TITLE | RANK IN GENE LIST | RANK METRIC SCORE | RUNNING ES | CORE ENRICHMENT || 1 | PPP3CB | PPP3CB Entrez,  Source | protein phosphatase 3 (formerly 2B), catalytic subunit, beta isoform (calcineurin A beta) | 2044 | 0.200 | -0.0451 | No |
| 2 | CAMK1 | CAMK1 Entrez,  Source | calcium/calmodulin-dependent protein kinase I | 2195 | 0.189 | 0.0038 | No |
| 3 | CAMK4 | CAMK4 Entrez,  Source | calcium/calmodulin-dependent protein kinase IV | 2279 | 0.184 | 0.0543 | No |
| 4 | HDAC5 | HDAC5 Entrez,  Source | histone deacetylase 5 | 2989 | 0.149 | 0.0625 | No |
| 5 | CAMK2G | CAMK2G Entrez,  Source | calcium/calmodulin-dependent protein kinase (CaM kinase) II gamma | 4577 | 0.096 | 0.0100 | No |
| 6 | CAMK1G | CAMK1G Entrez,  Source | calcium/calmodulin-dependent protein kinase IG | 4868 | 0.089 | 0.0218 | No |
| 7 | SLC2A4 | SLC2A4 Entrez,  Source | solute carrier family 2 (facilitated glucose transporter), member 4 | 5015 | 0.086 | 0.0398 | No |
| 8 | CALM3 | CALM3 Entrez,  Source | calmodulin 3 (phosphorylase kinase, delta) | 6946 | 0.044 | -0.0457 | No |
| 9 | ESRRA | ESRRA Entrez,  Source | estrogen-related receptor alpha | 7889 | 0.027 | -0.0858 | No |
| 10 | CALM1 | CALM1 Entrez,  Source | calmodulin 1 (phosphorylase kinase, delta) | 8204 | 0.022 | -0.0953 | No |
| 11 | MEF2D | MEF2D Entrez,  Source | MADS box transcription enhancer factor 2, polypeptide D (myocyte enhancer factor 2D) | 9459 | 0.000 | -0.1594 | No |
| 12 | CALM2 | CALM2 Entrez,  Source | calmodulin 2 (phosphorylase kinase, delta) | 9828 | -0.005 | -0.1766 | No |
| 13 | CAMK2B | CAMK2B Entrez,  Source | calcium/calmodulin-dependent protein kinase (CaM kinase) II beta | 10525 | -0.017 | -0.2072 | No |
| 14 | PPP3CC | PPP3CC Entrez,  Source | protein phosphatase 3 (formerly 2B), catalytic subunit, gamma isoform (calcineurin A gamma) | 11974 | -0.042 | -0.2687 | No |
| 15 | YWHAH | YWHAH Entrez,  Source | tyrosine 3-monooxygenase/tryptophan 5-monooxygenase activation protein, eta polypeptide | 13683 | -0.075 | -0.3336 | No |
| 16 | PPARA | PPARA Entrez,  Source | peroxisome proliferative activated receptor, alpha | 14380 | -0.091 | -0.3420 | No |
| 17 | CAMK2A | CAMK2A Entrez,  Source | calcium/calmodulin-dependent protein kinase (CaM kinase) II alpha | 15211 | -0.113 | -0.3508 | No |
| 18 | MEF2A | MEF2A Entrez,  Source | MADS box transcription enhancer factor 2, polypeptide A (myocyte enhancer factor 2A) | 17433 | -0.199 | -0.4051 | Yes |
| 19 | PPP3CA | PPP3CA Entrez,  Source | protein phosphatase 3 (formerly 2B), catalytic subunit, alpha isoform (calcineurin A alpha) | 18224 | -0.259 | -0.3682 | Yes |
| 20 | CAMK2D | CAMK2D Entrez,  Source | calcium/calmodulin-dependent protein kinase (CaM kinase) II delta | 18274 | -0.265 | -0.2917 | Yes |
| 21 | PPARGC1A | PPARGC1A Entrez,  Source | peroxisome proliferative activated receptor, gamma, coactivator 1, alpha | 19300 | -0.588 | -0.1688 | Yes |
| 22 | MEF2C | MEF2C Entrez,  Source | MADS box transcription enhancer factor 2, polypeptide C (myocyte enhancer factor 2C) | 19322 | -0.609 | 0.0120 | Yes |
Table: GSEA details [plain text format]

  

Fig 2: BIOCARTA\_PGC1A\_PATHWAY      
 Blue-Pink O' Gram in the Space of the Analyzed GeneSet

  

Fig 3: BIOCARTA\_PGC1A\_PATHWAY: Random ES distribution      
 Gene set null distribution of ES for **BIOCARTA\_PGC1A\_PATHWAY**
