# Supplementary material for: Comprehensive analysis of β-catenin target genes in colorectal carcinoma cell lines with deregulated Wnt/β-catenin signaling
Source: BMC Genomics. 2014 Jan 28;15:74. doi: 10.1186/1471-2164-15-74 (PMC3909937; doi:10.1186/1471-2164-15-74)
Supplement: Additional file 4 — GSEA analysis using the Biocarta pathway database. This zipped file contains confirming data of the GSEA analysis. The names of the directories containing the files were composed of the term ‘GSEA’, the name of the cell line, e.g. DLD1, SW480, or LS174T, and the pathway database (Biocarta). Please use a web browser to view the files with the name ‘index.html’ in the corresponding directories to start exploring the data. [file 1471-2164-15-74-S4.zip › LS174T_Biocarta/BIOCARTA_PPARA_PATHWAY.html]

Details for gene set BIOCARTA\_PPARA\_PATHWAY[GSEA]

|  || Dataset | LS174T\_collapsed\_to\_symbols.class.cls#bg\_versus\_b |
| Phenotype | class.cls#bg\_versus\_b |
| Upregulated in class | b |
| GeneSet | BIOCARTA\_PPARA\_PATHWAY |
| Enrichment Score (ES) | -0.53431237 |
| Normalized Enrichment Score (NES) | -1.7418873 |
| Nominal p-value | 0.0018115942 |
| FDR q-value | 0.5595862 |
| FWER p-Value | 0.414 |
Table: GSEA Results Summary

  

Fig 1: Enrichment plot: BIOCARTA\_PPARA\_PATHWAY      
 Profile of the Running ES Score & Positions of GeneSet Members on the Rank Ordered List

  

| PROBE | GENE SYMBOL | GENE\_TITLE | RANK IN GENE LIST | RANK METRIC SCORE | RUNNING ES | CORE ENRICHMENT || 1 | PTGS2 | PTGS2 Entrez,  Source | prostaglandin-endoperoxide synthase 2 (prostaglandin G/H synthase and cyclooxygenase) | 705 | 0.353 | -0.0021 | No |
| 2 | DUT | DUT Entrez,  Source | dUTP pyrophosphatase | 737 | 0.346 | 0.0296 | No |
| 3 | MRPL11 | MRPL11 Entrez,  Source | mitochondrial ribosomal protein L11 | 1322 | 0.260 | 0.0247 | No |
| 4 | MYC | MYC Entrez,  Source | v-myc myelocytomatosis viral oncogene homolog (avian) | 1540 | 0.238 | 0.0366 | No |
| 5 | NCOR2 | NCOR2 Entrez,  Source | nuclear receptor co-repressor 2 | 2408 | 0.177 | 0.0092 | No |
| 6 | PRKCA | PRKCA Entrez,  Source | protein kinase C, alpha | 2447 | 0.175 | 0.0241 | No |
| 7 | PDGFA | PDGFA Entrez,  Source | platelet-derived growth factor alpha polypeptide | 3214 | 0.140 | -0.0016 | No |
| 8 | STAT5A | STAT5A Entrez,  Source | signal transducer and activator of transcription 5A | 3589 | 0.126 | -0.0087 | No |
| 9 | NFKBIA | NFKBIA Entrez,  Source | nuclear factor of kappa light polypeptide gene enhancer in B-cells inhibitor, alpha | 3603 | 0.125 | 0.0028 | No |
| 10 | DUSP1 | DUSP1 Entrez,  Source | dual specificity phosphatase 1 | 5333 | 0.078 | -0.0784 | No |
| 11 | PIK3R1 | PIK3R1 Entrez,  Source | phosphoinositide-3-kinase, regulatory subunit 1 (p85 alpha) | 5670 | 0.070 | -0.0889 | No |
| 12 | NCOA1 | NCOA1 Entrez,  Source | nuclear receptor coactivator 1 | 5885 | 0.065 | -0.0936 | No |
| 13 | MAPK1 | MAPK1 Entrez,  Source | mitogen-activated protein kinase 1 | 6467 | 0.053 | -0.1182 | No |
| 14 | SP1 | SP1 Entrez,  Source | Sp1 transcription factor | 6491 | 0.053 | -0.1143 | No |
| 15 | HSP90AA1 | HSP90AA1 Entrez,  Source | heat shock protein 90kDa alpha (cytosolic), class A member 1 | 6603 | 0.051 | -0.1151 | No |
| 16 | RB1 | RB1 Entrez,  Source | retinoblastoma 1 (including osteosarcoma) | 7450 | 0.035 | -0.1552 | No |
| 17 | CREBBP | CREBBP Entrez,  Source | CREB binding protein (Rubinstein-Taybi syndrome) | 7598 | 0.032 | -0.1596 | No |
| 18 | TNF | TNF Entrez,  Source | tumor necrosis factor (TNF superfamily, member 2) | 8127 | 0.023 | -0.1844 | No |
| 19 | RELA | RELA Entrez,  Source | v-rel reticuloendotheliosis viral oncogene homolog A, nuclear factor of kappa light polypeptide gene enhancer in B-cells 3, p65 (avian) | 8874 | 0.010 | -0.2217 | No |
| 20 | RXRA | RXRA Entrez,  Source | retinoid X receptor, alpha | 9259 | 0.003 | -0.2411 | No |
| 21 | JUN | JUN Entrez,  Source | jun oncogene | 9654 | -0.003 | -0.2611 | No |
| 22 | APOA1 | APOA1 Entrez,  Source | apolipoprotein A-I | 9989 | -0.008 | -0.2774 | No |
| 23 | HSD17B4 | HSD17B4 Entrez,  Source | hydroxysteroid (17-beta) dehydrogenase 4 | 10097 | -0.010 | -0.2820 | No |
| 24 | PRKAR2B | PRKAR2B Entrez,  Source | protein kinase, cAMP-dependent, regulatory, type II, beta | 10285 | -0.013 | -0.2903 | No |
| 25 | PRKAR2A | PRKAR2A Entrez,  Source | protein kinase, cAMP-dependent, regulatory, type II, alpha | 10677 | -0.019 | -0.3085 | No |
| 26 | NCOR1 | NCOR1 Entrez,  Source | nuclear receptor co-repressor 1 | 11054 | -0.026 | -0.3253 | No |
| 27 | STAT5B | STAT5B Entrez,  Source | signal transducer and activator of transcription 5B | 11878 | -0.041 | -0.3636 | No |
| 28 | NR2F1 | NR2F1 Entrez,  Source | nuclear receptor subfamily 2, group F, member 1 | 11957 | -0.042 | -0.3635 | No |
| 29 | SRA1 | SRA1 Entrez,  Source | steroid receptor RNA activator 1 | 12702 | -0.056 | -0.3962 | No |
| 30 | INS | INS Entrez,  Source | insulin | 12710 | -0.056 | -0.3912 | No |
| 31 | EP300 | EP300 Entrez,  Source | E1A binding protein p300 | 12980 | -0.061 | -0.3990 | No |
| 32 | PPARA | PPARA Entrez,  Source | peroxisome proliferative activated receptor, alpha | 14380 | -0.091 | -0.4620 | No |
| 33 | MAPK3 | MAPK3 Entrez,  Source | mitogen-activated protein kinase 3 | 14609 | -0.097 | -0.4643 | No |
| 34 | PRKACB | PRKACB Entrez,  Source | protein kinase, cAMP-dependent, catalytic, beta | 15299 | -0.115 | -0.4885 | No |
| 35 | NRIP1 | NRIP1 Entrez,  Source | nuclear receptor interacting protein 1 | 16193 | -0.144 | -0.5204 | Yes |
| 36 | PIK3CA | PIK3CA Entrez,  Source | phosphoinositide-3-kinase, catalytic, alpha polypeptide | 16332 | -0.149 | -0.5131 | Yes |
| 37 | ME1 | ME1 Entrez,  Source | malic enzyme 1, NADP(+)-dependent, cytosolic | 16457 | -0.153 | -0.5047 | Yes |
| 38 | PRKAR1A | PRKAR1A Entrez,  Source | protein kinase, cAMP-dependent, regulatory, type I, alpha (tissue specific extinguisher 1) | 16494 | -0.155 | -0.4916 | Yes |
| 39 | CPT1B | CPT1B Entrez,  Source | carnitine palmitoyltransferase 1B (muscle) | 16898 | -0.171 | -0.4958 | Yes |
| 40 | CD36 | CD36 Entrez,  Source | CD36 molecule (thrombospondin receptor) | 17049 | -0.177 | -0.4864 | Yes |
| 41 | APOA2 | APOA2 Entrez,  Source | apolipoprotein A-II | 17229 | -0.187 | -0.4775 | Yes |
| 42 | PRKACG | PRKACG Entrez,  Source | protein kinase, cAMP-dependent, catalytic, gamma | 17288 | -0.190 | -0.4622 | Yes |
| 43 | PIK3CG | PIK3CG Entrez,  Source | phosphoinositide-3-kinase, catalytic, gamma polypeptide | 17689 | -0.216 | -0.4618 | Yes |
| 44 | NR0B2 | NR0B2 Entrez,  Source | nuclear receptor subfamily 0, group B, member 2 | 18423 | -0.281 | -0.4723 | Yes |
| 45 | NR1H3 | NR1H3 Entrez,  Source | nuclear receptor subfamily 1, group H, member 3 | 18477 | -0.290 | -0.4470 | Yes |
| 46 | ACOX1 | ACOX1 Entrez,  Source | acyl-Coenzyme A oxidase 1, palmitoyl | 18765 | -0.341 | -0.4289 | Yes |
| 47 | LPL | LPL Entrez,  Source | lipoprotein lipase | 18774 | -0.342 | -0.3963 | Yes |
| 48 | CITED2 | CITED2 Entrez,  Source | Cbp/p300-interacting transactivator, with Glu/Asp-rich carboxy-terminal domain, 2 | 18956 | -0.386 | -0.3684 | Yes |
| 49 | HSPA1A | HSPA1A Entrez,  Source | heat shock 70kDa protein 1A | 19162 | -0.463 | -0.3343 | Yes |
| 50 | EHHADH | EHHADH Entrez,  Source | enoyl-Coenzyme A, hydratase/3-hydroxyacyl Coenzyme A dehydrogenase | 19246 | -0.528 | -0.2876 | Yes |
| 51 | PPARGC1A | PPARGC1A Entrez,  Source | peroxisome proliferative activated receptor, gamma, coactivator 1, alpha | 19300 | -0.588 | -0.2337 | Yes |
| 52 | FABP1 | FABP1 Entrez,  Source | fatty acid binding protein 1, liver | 19554 | -2.560 | 0.0001 | Yes |
Table: GSEA details [plain text format]

  

Fig 2: BIOCARTA\_PPARA\_PATHWAY      
 Blue-Pink O' Gram in the Space of the Analyzed GeneSet

  

Fig 3: BIOCARTA\_PPARA\_PATHWAY: Random ES distribution      
 Gene set null distribution of ES for **BIOCARTA\_PPARA\_PATHWAY**
