# Supplementary material for: Comprehensive analysis of β-catenin target genes in colorectal carcinoma cell lines with deregulated Wnt/β-catenin signaling
Source: BMC Genomics. 2014 Jan 28;15:74. doi: 10.1186/1471-2164-15-74 (PMC3909937; doi:10.1186/1471-2164-15-74)
Supplement: Additional file 4 — GSEA analysis using the Biocarta pathway database. This zipped file contains confirming data of the GSEA analysis. The names of the directories containing the files were composed of the term ‘GSEA’, the name of the cell line, e.g. DLD1, SW480, or LS174T, and the pathway database (Biocarta). Please use a web browser to view the files with the name ‘index.html’ in the corresponding directories to start exploring the data. [file 1471-2164-15-74-S4.zip › LS174T_Biocarta/BIOCARTA_PROTEASOME_PATHWAY.html]

Details for gene set BIOCARTA\_PROTEASOME\_PATHWAY[GSEA]

|  || Dataset | LS174T\_collapsed\_to\_symbols.class.cls#bg\_versus\_b |
| Phenotype | class.cls#bg\_versus\_b |
| Upregulated in class | bg |
| GeneSet | BIOCARTA\_PROTEASOME\_PATHWAY |
| Enrichment Score (ES) | 0.51919603 |
| Normalized Enrichment Score (NES) | 1.5228715 |
| Nominal p-value | 0.030737706 |
| FDR q-value | 0.25125617 |
| FWER p-Value | 0.917 |
Table: GSEA Results Summary

  

Fig 1: Enrichment plot: BIOCARTA\_PROTEASOME\_PATHWAY      
 Profile of the Running ES Score & Positions of GeneSet Members on the Rank Ordered List

  

| PROBE | GENE SYMBOL | GENE\_TITLE | RANK IN GENE LIST | RANK METRIC SCORE | RUNNING ES | CORE ENRICHMENT || 1 | PSMD11 | PSMD11 Entrez,  Source | proteasome (prosome, macropain) 26S subunit, non-ATPase, 11 | 2649 | 0.164 | 0.0167 | Yes |
| 2 | PSMD8 | PSMD8 Entrez,  Source | proteasome (prosome, macropain) 26S subunit, non-ATPase, 8 | 4251 | 0.106 | 0.0327 | Yes |
| 3 | PSMB2 | PSMB2 Entrez,  Source | proteasome (prosome, macropain) subunit, beta type, 2 | 5150 | 0.082 | 0.0626 | Yes |
| 4 | PSMC2 | PSMC2 Entrez,  Source | proteasome (prosome, macropain) 26S subunit, ATPase, 2 | 5550 | 0.073 | 0.1095 | Yes |
| 5 | PSMC3 | PSMC3 Entrez,  Source | proteasome (prosome, macropain) 26S subunit, ATPase, 3 | 6066 | 0.061 | 0.1400 | Yes |
| 6 | PSMA6 | PSMA6 Entrez,  Source | proteasome (prosome, macropain) subunit, alpha type, 6 | 6111 | 0.061 | 0.1939 | Yes |
| 7 | PSMD12 | PSMD12 Entrez,  Source | proteasome (prosome, macropain) 26S subunit, non-ATPase, 12 | 6282 | 0.057 | 0.2379 | Yes |
| 8 | PSMA3 | PSMA3 Entrez,  Source | proteasome (prosome, macropain) subunit, alpha type, 3 | 6412 | 0.054 | 0.2815 | Yes |
| 9 | PSMA2 | PSMA2 Entrez,  Source | proteasome (prosome, macropain) subunit, alpha type, 2 | 6770 | 0.048 | 0.3074 | Yes |
| 10 | PSMB3 | PSMB3 Entrez,  Source | proteasome (prosome, macropain) subunit, beta type, 3 | 7041 | 0.043 | 0.3332 | Yes |
| 11 | PSMD14 | PSMD14 Entrez,  Source | proteasome (prosome, macropain) 26S subunit, non-ATPase, 14 | 7105 | 0.041 | 0.3681 | Yes |
| 12 | PSMA7 | PSMA7 Entrez,  Source | proteasome (prosome, macropain) subunit, alpha type, 7 | 7194 | 0.040 | 0.4003 | Yes |
| 13 | UBE3A | UBE3A Entrez,  Source | ubiquitin protein ligase E3A (human papilloma virus E6-associated protein, Angelman syndrome) | 7325 | 0.037 | 0.4279 | Yes |
| 14 | PSMB6 | PSMB6 Entrez,  Source | proteasome (prosome, macropain) subunit, beta type, 6 | 7674 | 0.031 | 0.4388 | Yes |
| 15 | PSMA1 | PSMA1 Entrez,  Source | proteasome (prosome, macropain) subunit, alpha type, 1 | 7825 | 0.028 | 0.4573 | Yes |
| 16 | PSMB1 | PSMB1 Entrez,  Source | proteasome (prosome, macropain) subunit, beta type, 1 | 7966 | 0.026 | 0.4742 | Yes |
| 17 | RPN1 | RPN1 Entrez,  Source | ribophorin I | 8167 | 0.023 | 0.4848 | Yes |
| 18 | PSMA4 | PSMA4 Entrez,  Source | proteasome (prosome, macropain) subunit, alpha type, 4 | 8221 | 0.021 | 0.5020 | Yes |
| 19 | PSMC6 | PSMC6 Entrez,  Source | proteasome (prosome, macropain) 26S subunit, ATPase, 6 | 8260 | 0.021 | 0.5192 | Yes |
| 20 | PSMB5 | PSMB5 Entrez,  Source | proteasome (prosome, macropain) subunit, beta type, 5 | 9214 | 0.004 | 0.4743 | No |
| 21 | PSMD6 | PSMD6 Entrez,  Source | proteasome (prosome, macropain) 26S subunit, non-ATPase, 6 | 9810 | -0.005 | 0.4486 | No |
| 22 | PSMB7 | PSMB7 Entrez,  Source | proteasome (prosome, macropain) subunit, beta type, 7 | 9823 | -0.005 | 0.4529 | No |
| 23 | UBE2A | UBE2A Entrez,  Source | ubiquitin-conjugating enzyme E2A (RAD6 homolog) | 9846 | -0.006 | 0.4571 | No |
| 24 | PSMB4 | PSMB4 Entrez,  Source | proteasome (prosome, macropain) subunit, beta type, 4 | 10070 | -0.009 | 0.4544 | No |
| 25 | RPN2 | RPN2 Entrez,  Source | ribophorin II | 10522 | -0.017 | 0.4468 | No |
| 26 | PSMA5 | PSMA5 Entrez,  Source | proteasome (prosome, macropain) subunit, alpha type, 5 | 10532 | -0.017 | 0.4620 | No |
Table: GSEA details [plain text format]

  

Fig 2: BIOCARTA\_PROTEASOME\_PATHWAY      
 Blue-Pink O' Gram in the Space of the Analyzed GeneSet

  

Fig 3: BIOCARTA\_PROTEASOME\_PATHWAY: Random ES distribution      
 Gene set null distribution of ES for **BIOCARTA\_PROTEASOME\_PATHWAY**
