# Supplementary material for: Comprehensive analysis of β-catenin target genes in colorectal carcinoma cell lines with deregulated Wnt/β-catenin signaling
Source: BMC Genomics. 2014 Jan 28;15:74. doi: 10.1186/1471-2164-15-74 (PMC3909937; doi:10.1186/1471-2164-15-74)
Supplement: Additional file 4 — GSEA analysis using the Biocarta pathway database. This zipped file contains confirming data of the GSEA analysis. The names of the directories containing the files were composed of the term ‘GSEA’, the name of the cell line, e.g. DLD1, SW480, or LS174T, and the pathway database (Biocarta). Please use a web browser to view the files with the name ‘index.html’ in the corresponding directories to start exploring the data. [file 1471-2164-15-74-S4.zip › LS174T_Biocarta/BIOCARTA_RACCYCD_PATHWAY.html]

Details for gene set BIOCARTA\_RACCYCD\_PATHWAY[GSEA]

|  || Dataset | LS174T\_collapsed\_to\_symbols.class.cls#bg\_versus\_b |
| Phenotype | class.cls#bg\_versus\_b |
| Upregulated in class | bg |
| GeneSet | BIOCARTA\_RACCYCD\_PATHWAY |
| Enrichment Score (ES) | 0.48379678 |
| Normalized Enrichment Score (NES) | 1.4217341 |
| Nominal p-value | 0.06437768 |
| FDR q-value | 0.4289293 |
| FWER p-Value | 0.994 |
Table: GSEA Results Summary

  

Fig 1: Enrichment plot: BIOCARTA\_RACCYCD\_PATHWAY      
 Profile of the Running ES Score & Positions of GeneSet Members on the Rank Ordered List

  

| PROBE | GENE SYMBOL | GENE\_TITLE | RANK IN GENE LIST | RANK METRIC SCORE | RUNNING ES | CORE ENRICHMENT || 1 | CDK2 | CDK2 Entrez,  Source | cyclin-dependent kinase 2 | 575 | 0.390 | 0.0936 | Yes |
| 2 | CCNE1 | CCNE1 Entrez,  Source | cyclin E1 | 815 | 0.330 | 0.1854 | Yes |
| 3 | E2F1 | E2F1 Entrez,  Source | E2F transcription factor 1 | 1057 | 0.292 | 0.2650 | Yes |
| 4 | AKT1 | AKT1 Entrez,  Source | v-akt murine thymoma viral oncogene homolog 1 | 1273 | 0.266 | 0.3378 | Yes |
| 5 | CCND1 | CCND1 Entrez,  Source | cyclin D1 | 1372 | 0.254 | 0.4130 | Yes |
| 6 | CDK4 | CDK4 Entrez,  Source | cyclin-dependent kinase 4 | 2764 | 0.160 | 0.3921 | Yes |
| 7 | CDK6 | CDK6 Entrez,  Source | cyclin-dependent kinase 6 | 2942 | 0.151 | 0.4307 | Yes |
| 8 | TFDP1 | TFDP1 Entrez,  Source | transcription factor Dp-1 | 2968 | 0.150 | 0.4768 | Yes |
| 9 | NFKBIA | NFKBIA Entrez,  Source | nuclear factor of kappa light polypeptide gene enhancer in B-cells inhibitor, alpha | 3603 | 0.125 | 0.4838 | Yes |
| 10 | CDKN1A | CDKN1A Entrez,  Source | cyclin-dependent kinase inhibitor 1A (p21, Cip1) | 4270 | 0.106 | 0.4830 | No |
| 11 | NFKB1 | NFKB1 Entrez,  Source | nuclear factor of kappa light polypeptide gene enhancer in B-cells 1 (p105) | 4927 | 0.088 | 0.4770 | No |
| 12 | PIK3R1 | PIK3R1 Entrez,  Source | phosphoinositide-3-kinase, regulatory subunit 1 (p85 alpha) | 5670 | 0.070 | 0.4612 | No |
| 13 | RAF1 | RAF1 Entrez,  Source | v-raf-1 murine leukemia viral oncogene homolog 1 | 5946 | 0.064 | 0.4672 | No |
| 14 | MAPK1 | MAPK1 Entrez,  Source | mitogen-activated protein kinase 1 | 6467 | 0.053 | 0.4573 | No |
| 15 | RB1 | RB1 Entrez,  Source | retinoblastoma 1 (including osteosarcoma) | 7450 | 0.035 | 0.4180 | No |
| 16 | RELA | RELA Entrez,  Source | v-rel reticuloendotheliosis viral oncogene homolog A, nuclear factor of kappa light polypeptide gene enhancer in B-cells 3, p65 (avian) | 8874 | 0.010 | 0.3482 | No |
| 17 | HRAS | HRAS Entrez,  Source | v-Ha-ras Harvey rat sarcoma viral oncogene homolog | 9218 | 0.004 | 0.3319 | No |
| 18 | CHUK | CHUK Entrez,  Source | conserved helix-loop-helix ubiquitous kinase | 9260 | 0.003 | 0.3308 | No |
| 19 | RAC1 | RAC1 Entrez,  Source | ras-related C3 botulinum toxin substrate 1 (rho family, small GTP binding protein Rac1) | 9829 | -0.005 | 0.3035 | No |
| 20 | RHOA | RHOA Entrez,  Source | ras homolog gene family, member A | 10998 | -0.024 | 0.2514 | No |
| 21 | IKBKG | IKBKG Entrez,  Source | inhibitor of kappa light polypeptide gene enhancer in B-cells, kinase gamma | 12693 | -0.056 | 0.1823 | No |
| 22 | CDKN1B | CDKN1B Entrez,  Source | cyclin-dependent kinase inhibitor 1B (p27, Kip1) | 13610 | -0.074 | 0.1588 | No |
| 23 | IKBKB | IKBKB Entrez,  Source | inhibitor of kappa light polypeptide gene enhancer in B-cells, kinase beta | 14091 | -0.085 | 0.1609 | No |
| 24 | MAPK3 | MAPK3 Entrez,  Source | mitogen-activated protein kinase 3 | 14609 | -0.097 | 0.1649 | No |
| 25 | PAK1 | PAK1 Entrez,  Source | p21/Cdc42/Rac1-activated kinase 1 (STE20 homolog, yeast) | 15808 | -0.131 | 0.1448 | No |
| 26 | PIK3CA | PIK3CA Entrez,  Source | phosphoinositide-3-kinase, catalytic, alpha polypeptide | 16332 | -0.149 | 0.1651 | No |
Table: GSEA details [plain text format]

  

Fig 2: BIOCARTA\_RACCYCD\_PATHWAY      
 Blue-Pink O' Gram in the Space of the Analyzed GeneSet

  

Fig 3: BIOCARTA\_RACCYCD\_PATHWAY: Random ES distribution      
 Gene set null distribution of ES for **BIOCARTA\_RACCYCD\_PATHWAY**
