# Supplementary material for: Comprehensive analysis of β-catenin target genes in colorectal carcinoma cell lines with deregulated Wnt/β-catenin signaling
Source: BMC Genomics. 2014 Jan 28;15:74. doi: 10.1186/1471-2164-15-74 (PMC3909937; doi:10.1186/1471-2164-15-74)
Supplement: Additional file 4 — GSEA analysis using the Biocarta pathway database. This zipped file contains confirming data of the GSEA analysis. The names of the directories containing the files were composed of the term ‘GSEA’, the name of the cell line, e.g. DLD1, SW480, or LS174T, and the pathway database (Biocarta). Please use a web browser to view the files with the name ‘index.html’ in the corresponding directories to start exploring the data. [file 1471-2164-15-74-S4.zip › LS174T_Biocarta/BIOCARTA_SPRY_PATHWAY.html]

Details for gene set BIOCARTA\_SPRY\_PATHWAY[GSEA]

|  || Dataset | LS174T\_collapsed\_to\_symbols.class.cls#bg\_versus\_b |
| Phenotype | class.cls#bg\_versus\_b |
| Upregulated in class | bg |
| GeneSet | BIOCARTA\_SPRY\_PATHWAY |
| Enrichment Score (ES) | 0.42450014 |
| Normalized Enrichment Score (NES) | 1.1407473 |
| Nominal p-value | 0.28421053 |
| FDR q-value | 0.8791689 |
| FWER p-Value | 1.0 |
Table: GSEA Results Summary

  

Fig 1: Enrichment plot: BIOCARTA\_SPRY\_PATHWAY      
 Profile of the Running ES Score & Positions of GeneSet Members on the Rank Ordered List

  

| PROBE | GENE SYMBOL | GENE\_TITLE | RANK IN GENE LIST | RANK METRIC SCORE | RUNNING ES | CORE ENRICHMENT || 1 | CBL | CBL Entrez,  Source | Cas-Br-M (murine) ecotropic retroviral transforming sequence | 553 | 0.397 | 0.1375 | Yes |
| 2 | EGFR | EGFR Entrez,  Source | epidermal growth factor receptor (erythroblastic leukemia viral (v-erb-b) oncogene homolog, avian) | 774 | 0.337 | 0.2673 | Yes |
| 3 | SRC | SRC Entrez,  Source | v-src sarcoma (Schmidt-Ruppin A-2) viral oncogene homolog (avian) | 953 | 0.307 | 0.3866 | Yes |
| 4 | SPRY4 | SPRY4 Entrez,  Source | sprouty homolog 4 (Drosophila) | 1911 | 0.208 | 0.4245 | Yes |
| 5 | RAF1 | RAF1 Entrez,  Source | v-raf-1 murine leukemia viral oncogene homolog 1 | 5946 | 0.064 | 0.2447 | No |
| 6 | MAPK1 | MAPK1 Entrez,  Source | mitogen-activated protein kinase 1 | 6467 | 0.053 | 0.2403 | No |
| 7 | SHC1 | SHC1 Entrez,  Source | SHC (Src homology 2 domain containing) transforming protein 1 | 6609 | 0.051 | 0.2542 | No |
| 8 | SPRY3 | SPRY3 Entrez,  Source | sprouty homolog 3 (Drosophila) | 8133 | 0.023 | 0.1859 | No |
| 9 | GRB2 | GRB2 Entrez,  Source | growth factor receptor-bound protein 2 | 8140 | 0.023 | 0.1952 | No |
| 10 | HRAS | HRAS Entrez,  Source | v-Ha-ras Harvey rat sarcoma viral oncogene homolog | 9218 | 0.004 | 0.1418 | No |
| 11 | SPRY1 | SPRY1 Entrez,  Source | sprouty homolog 1, antagonist of FGF signaling (Drosophila) | 9223 | 0.004 | 0.1432 | No |
| 12 | SOS1 | SOS1 Entrez,  Source | son of sevenless homolog 1 (Drosophila) | 9435 | 0.001 | 0.1327 | No |
| 13 | MAP2K1 | MAP2K1 Entrez,  Source | mitogen-activated protein kinase kinase 1 | 11207 | -0.028 | 0.0539 | No |
| 14 | RASA1 | RASA1 Entrez,  Source | RAS p21 protein activator (GTPase activating protein) 1 | 11334 | -0.030 | 0.0601 | No |
| 15 | PTPRB | PTPRB Entrez,  Source | protein tyrosine phosphatase, receptor type, B | 12193 | -0.047 | 0.0358 | No |
| 16 | MAPK3 | MAPK3 Entrez,  Source | mitogen-activated protein kinase 3 | 14609 | -0.097 | -0.0474 | No |
| 17 | EGF | EGF Entrez,  Source | epidermal growth factor (beta-urogastrone) | 18788 | -0.346 | -0.1166 | No |
| 18 | SPRY2 | SPRY2 Entrez,  Source | sprouty homolog 2 (Drosophila) | 18901 | -0.373 | 0.0335 | No |
Table: GSEA details [plain text format]

  

Fig 2: BIOCARTA\_SPRY\_PATHWAY      
 Blue-Pink O' Gram in the Space of the Analyzed GeneSet

  

Fig 3: BIOCARTA\_SPRY\_PATHWAY: Random ES distribution      
 Gene set null distribution of ES for **BIOCARTA\_SPRY\_PATHWAY**
