# Supplementary material for: Comprehensive analysis of β-catenin target genes in colorectal carcinoma cell lines with deregulated Wnt/β-catenin signaling
Source: BMC Genomics. 2014 Jan 28;15:74. doi: 10.1186/1471-2164-15-74 (PMC3909937; doi:10.1186/1471-2164-15-74)
Supplement: Additional file 4 — GSEA analysis using the Biocarta pathway database. This zipped file contains confirming data of the GSEA analysis. The names of the directories containing the files were composed of the term ‘GSEA’, the name of the cell line, e.g. DLD1, SW480, or LS174T, and the pathway database (Biocarta). Please use a web browser to view the files with the name ‘index.html’ in the corresponding directories to start exploring the data. [file 1471-2164-15-74-S4.zip › LS174T_Biocarta/BIOCARTA_TEL_PATHWAY.html]

Details for gene set BIOCARTA\_TEL\_PATHWAY[GSEA]

|  || Dataset | LS174T\_collapsed\_to\_symbols.class.cls#bg\_versus\_b |
| Phenotype | class.cls#bg\_versus\_b |
| Upregulated in class | bg |
| GeneSet | BIOCARTA\_TEL\_PATHWAY |
| Enrichment Score (ES) | 0.58891433 |
| Normalized Enrichment Score (NES) | 1.5813444 |
| Nominal p-value | 0.017582418 |
| FDR q-value | 0.2136806 |
| FWER p-Value | 0.809 |
Table: GSEA Results Summary

  

Fig 1: Enrichment plot: BIOCARTA\_TEL\_PATHWAY      
 Profile of the Running ES Score & Positions of GeneSet Members on the Rank Ordered List

  

| PROBE | GENE SYMBOL | GENE\_TITLE | RANK IN GENE LIST | RANK METRIC SCORE | RUNNING ES | CORE ENRICHMENT || 1 | EGFR | EGFR Entrez,  Source | epidermal growth factor receptor (erythroblastic leukemia viral (v-erb-b) oncogene homolog, avian) | 774 | 0.337 | 0.0866 | Yes |
| 2 | TERT | TERT Entrez,  Source | telomerase reverse transcriptase | 1032 | 0.296 | 0.1841 | Yes |
| 3 | AKT1 | AKT1 Entrez,  Source | v-akt murine thymoma viral oncogene homolog 1 | 1273 | 0.266 | 0.2712 | Yes |
| 4 | MYC | MYC Entrez,  Source | v-myc myelocytomatosis viral oncogene homolog (avian) | 1540 | 0.238 | 0.3466 | Yes |
| 5 | TNKS | TNKS Entrez,  Source | tankyrase, TRF1-interacting ankyrin-related ADP-ribose polymerase | 2138 | 0.193 | 0.3883 | Yes |
| 6 | PRKCA | PRKCA Entrez,  Source | protein kinase C, alpha | 2447 | 0.175 | 0.4379 | Yes |
| 7 | TP53 | TP53 Entrez,  Source | tumor protein p53 (Li-Fraumeni syndrome) | 2459 | 0.175 | 0.5026 | Yes |
| 8 | XRCC6 | XRCC6 Entrez,  Source | X-ray repair complementing defective repair in Chinese hamster cells 6 (Ku autoantigen, 70kDa) | 3406 | 0.133 | 0.5039 | Yes |
| 9 | TERF1 | TERF1 Entrez,  Source | telomeric repeat binding factor (NIMA-interacting) 1 | 3420 | 0.133 | 0.5529 | Yes |
| 10 | POLR2A | POLR2A Entrez,  Source | polymerase (RNA) II (DNA directed) polypeptide A, 220kDa | 3626 | 0.124 | 0.5889 | Yes |
| 11 | HSP90AA1 | HSP90AA1 Entrez,  Source | heat shock protein 90kDa alpha (cytosolic), class A member 1 | 6603 | 0.051 | 0.4555 | No |
| 12 | XRCC5 | XRCC5 Entrez,  Source | X-ray repair complementing defective repair in Chinese hamster cells 5 (double-strand-break rejoining; Ku autoantigen, 80kDa) | 6606 | 0.051 | 0.4744 | No |
| 13 | RB1 | RB1 Entrez,  Source | retinoblastoma 1 (including osteosarcoma) | 7450 | 0.035 | 0.4442 | No |
| 14 | PPP2CA | PPP2CA Entrez,  Source | protein phosphatase 2 (formerly 2A), catalytic subunit, alpha isoform | 8170 | 0.023 | 0.4159 | No |
| 15 | BCL2 | BCL2 Entrez,  Source | B-cell CLL/lymphoma 2 | 8414 | 0.018 | 0.4101 | No |
| 16 | IGF1R | IGF1R Entrez,  Source | insulin-like growth factor 1 receptor | 11811 | -0.040 | 0.2511 | No |
| 17 | KRAS | KRAS Entrez,  Source | v-Ki-ras2 Kirsten rat sarcoma viral oncogene homolog | 17251 | -0.189 | 0.0433 | No |
| 18 | TEP1 | TEP1 Entrez,  Source | telomerase-associated protein 1 | 17443 | -0.200 | 0.1081 | No |
Table: GSEA details [plain text format]

  

Fig 2: BIOCARTA\_TEL\_PATHWAY      
 Blue-Pink O' Gram in the Space of the Analyzed GeneSet

  

Fig 3: BIOCARTA\_TEL\_PATHWAY: Random ES distribution      
 Gene set null distribution of ES for **BIOCARTA\_TEL\_PATHWAY**
