# Supplementary material for: Comprehensive analysis of β-catenin target genes in colorectal carcinoma cell lines with deregulated Wnt/β-catenin signaling
Source: BMC Genomics. 2014 Jan 28;15:74. doi: 10.1186/1471-2164-15-74 (PMC3909937; doi:10.1186/1471-2164-15-74)
Supplement: Additional file 4 — GSEA analysis using the Biocarta pathway database. This zipped file contains confirming data of the GSEA analysis. The names of the directories containing the files were composed of the term ‘GSEA’, the name of the cell line, e.g. DLD1, SW480, or LS174T, and the pathway database (Biocarta). Please use a web browser to view the files with the name ‘index.html’ in the corresponding directories to start exploring the data. [file 1471-2164-15-74-S4.zip › LS174T_Biocarta/BIOCARTA_TFF_PATHWAY.html]

Details for gene set BIOCARTA\_TFF\_PATHWAY[GSEA]

|  || Dataset | LS174T\_collapsed\_to\_symbols.class.cls#bg\_versus\_b |
| Phenotype | class.cls#bg\_versus\_b |
| Upregulated in class | bg |
| GeneSet | BIOCARTA\_TFF\_PATHWAY |
| Enrichment Score (ES) | 0.55297726 |
| Normalized Enrichment Score (NES) | 1.563949 |
| Nominal p-value | 0.031185031 |
| FDR q-value | 0.2121237 |
| FWER p-Value | 0.846 |
Table: GSEA Results Summary

  

Fig 1: Enrichment plot: BIOCARTA\_TFF\_PATHWAY      
 Profile of the Running ES Score & Positions of GeneSet Members on the Rank Ordered List

  

| PROBE | GENE SYMBOL | GENE\_TITLE | RANK IN GENE LIST | RANK METRIC SCORE | RUNNING ES | CORE ENRICHMENT || 1 | CTNNB1 | CTNNB1 Entrez,  Source | catenin (cadherin-associated protein), beta 1, 88kDa | 3 | 1.322 | 0.4242 | Yes |
| 2 | EGFR | EGFR Entrez,  Source | epidermal growth factor receptor (erythroblastic leukemia viral (v-erb-b) oncogene homolog, avian) | 774 | 0.337 | 0.4931 | Yes |
| 3 | AKT1 | AKT1 Entrez,  Source | v-akt murine thymoma viral oncogene homolog 1 | 1273 | 0.266 | 0.5530 | Yes |
| 4 | GHR | GHR Entrez,  Source | growth hormone receptor | 3511 | 0.129 | 0.4800 | No |
| 5 | PIK3R1 | PIK3R1 Entrez,  Source | phosphoinositide-3-kinase, regulatory subunit 1 (p85 alpha) | 5670 | 0.070 | 0.3920 | No |
| 6 | CYCS | CYCS Entrez,  Source | cytochrome c, somatic | 5730 | 0.069 | 0.4111 | No |
| 7 | MAPK1 | MAPK1 Entrez,  Source | mitogen-activated protein kinase 1 | 6467 | 0.053 | 0.3905 | No |
| 8 | SHC1 | SHC1 Entrez,  Source | SHC (Src homology 2 domain containing) transforming protein 1 | 6609 | 0.051 | 0.3995 | No |
| 9 | GRB2 | GRB2 Entrez,  Source | growth factor receptor-bound protein 2 | 8140 | 0.023 | 0.3286 | No |
| 10 | HRAS | HRAS Entrez,  Source | v-Ha-ras Harvey rat sarcoma viral oncogene homolog | 9218 | 0.004 | 0.2747 | No |
| 11 | SOS1 | SOS1 Entrez,  Source | son of sevenless homolog 1 (Drosophila) | 9435 | 0.001 | 0.2639 | No |
| 12 | CASP9 | CASP9 Entrez,  Source | caspase 9, apoptosis-related cysteine peptidase | 9868 | -0.006 | 0.2437 | No |
| 13 | BAD | BAD Entrez,  Source | BCL2-antagonist of cell death | 10235 | -0.012 | 0.2288 | No |
| 14 | GH1 | GH1 Entrez,  Source | growth hormone 1 | 10719 | -0.020 | 0.2105 | No |
| 15 | RHOA | RHOA Entrez,  Source | ras homolog gene family, member A | 10998 | -0.024 | 0.2041 | No |
| 16 | APAF1 | APAF1 Entrez,  Source | apoptotic peptidase activating factor | 13272 | -0.067 | 0.1094 | No |
| 17 | MAPK3 | MAPK3 Entrez,  Source | mitogen-activated protein kinase 3 | 14609 | -0.097 | 0.0720 | No |
| 18 | ITGB1 | ITGB1 Entrez,  Source | integrin, beta 1 (fibronectin receptor, beta polypeptide, antigen CD29 includes MDF2, MSK12) | 14691 | -0.099 | 0.0997 | No |
| 19 | PTK2 | PTK2 Entrez,  Source | PTK2 protein tyrosine kinase 2 | 14708 | -0.099 | 0.1308 | No |
| 20 | PIK3CA | PIK3CA Entrez,  Source | phosphoinositide-3-kinase, catalytic, alpha polypeptide | 16332 | -0.149 | 0.0956 | No |
| 21 | PIK3CG | PIK3CG Entrez,  Source | phosphoinositide-3-kinase, catalytic, gamma polypeptide | 17689 | -0.216 | 0.0956 | No |
Table: GSEA details [plain text format]

  

Fig 2: BIOCARTA\_TFF\_PATHWAY      
 Blue-Pink O' Gram in the Space of the Analyzed GeneSet

  

Fig 3: BIOCARTA\_TFF\_PATHWAY: Random ES distribution      
 Gene set null distribution of ES for **BIOCARTA\_TFF\_PATHWAY**
