# Supplementary material for: Comprehensive analysis of β-catenin target genes in colorectal carcinoma cell lines with deregulated Wnt/β-catenin signaling
Source: BMC Genomics. 2014 Jan 28;15:74. doi: 10.1186/1471-2164-15-74 (PMC3909937; doi:10.1186/1471-2164-15-74)
Supplement: Additional file 4 — GSEA analysis using the Biocarta pathway database. This zipped file contains confirming data of the GSEA analysis. The names of the directories containing the files were composed of the term ‘GSEA’, the name of the cell line, e.g. DLD1, SW480, or LS174T, and the pathway database (Biocarta). Please use a web browser to view the files with the name ‘index.html’ in the corresponding directories to start exploring the data. [file 1471-2164-15-74-S4.zip › LS174T_Biocarta/gsea_report_for_b_1355840256140.html]

Report for b 1355840256140 [GSEA]

| GS  follow link to MSigDB | GS DETAILS | SIZE | ES | NES | NOM p-val | FDR q-val | FWER p-val | RANK AT MAX | LEADING EDGE || 1 | BIOCARTA\_PPARA\_PATHWAY | Details ... | 52 | -0.53 | -1.74 | 0.002 | 0.560 | 0.414 | 3365 | tags=35%, list=17%, signal=42% |
| 2 | BIOCARTA\_IGF1\_PATHWAY | Details ... | 21 | -0.56 | -1.50 | 0.055 | 1.000 | 0.975 | 120 | tags=10%, list=1%, signal=10% |
| 3 | BIOCARTA\_INSULIN\_PATHWAY | Details ... | 22 | -0.53 | -1.45 | 0.087 | 1.000 | 0.992 | 120 | tags=9%, list=1%, signal=9% |
| 4 | BIOCARTA\_NGF\_PATHWAY | Details ... | 17 | -0.54 | -1.37 | 0.112 | 1.000 | 1.000 | 5573 | tags=35%, list=28%, signal=49% |
| 5 | BIOCARTA\_CCR5\_PATHWAY | Details ... | 15 | -0.55 | -1.36 | 0.115 | 1.000 | 1.000 | 5676 | tags=53%, list=29%, signal=75% |
| 6 | BIOCARTA\_COMP\_PATHWAY | Details ... | 15 | -0.55 | -1.36 | 0.133 | 1.000 | 1.000 | 2656 | tags=27%, list=14%, signal=31% |
| 7 | BIOCARTA\_NKCELLS\_PATHWAY | Details ... | 17 | -0.52 | -1.33 | 0.122 | 1.000 | 1.000 | 4949 | tags=71%, list=25%, signal=94% |
| 8 | BIOCARTA\_TPO\_PATHWAY | Details ... | 23 | -0.47 | -1.30 | 0.163 | 1.000 | 1.000 | 3226 | tags=22%, list=16%, signal=26% |
| 9 | BIOCARTA\_CASPASE\_PATHWAY | Details ... | 22 | -0.47 | -1.27 | 0.178 | 1.000 | 1.000 | 1762 | tags=32%, list=9%, signal=35% |
| 10 | BIOCARTA\_AMI\_PATHWAY | Details ... | 19 | -0.48 | -1.27 | 0.186 | 1.000 | 1.000 | 4601 | tags=42%, list=24%, signal=55% |
| 11 | BIOCARTA\_PGC1A\_PATHWAY | Details ... | 22 | -0.46 | -1.25 | 0.183 | 1.000 | 1.000 | 2125 | tags=23%, list=11%, signal=25% |
| 12 | BIOCARTA\_MCALPAIN\_PATHWAY | Details ... | 22 | -0.45 | -1.23 | 0.198 | 1.000 | 1.000 | 4949 | tags=50%, list=25%, signal=67% |
| 13 | BIOCARTA\_DEATH\_PATHWAY | Details ... | 32 | -0.41 | -1.23 | 0.189 | 1.000 | 1.000 | 1840 | tags=25%, list=9%, signal=28% |
| 14 | BIOCARTA\_IGF1R\_PATHWAY | Details ... | 22 | -0.44 | -1.20 | 0.249 | 1.000 | 1.000 | 4949 | tags=32%, list=25%, signal=43% |
| 15 | BIOCARTA\_BAD\_PATHWAY | Details ... | 25 | -0.42 | -1.20 | 0.229 | 1.000 | 1.000 | 4259 | tags=36%, list=22%, signal=46% |
| 16 | BIOCARTA\_EPO\_PATHWAY | Details ... | 19 | -0.46 | -1.20 | 0.252 | 1.000 | 1.000 | 115 | tags=5%, list=1%, signal=5% |
| 17 | BIOCARTA\_NO2IL12\_PATHWAY | Details ... | 15 | -0.48 | -1.19 | 0.261 | 1.000 | 1.000 | 3054 | tags=27%, list=16%, signal=32% |
| 18 | BIOCARTA\_EGF\_PATHWAY | Details ... | 30 | -0.39 | -1.13 | 0.289 | 1.000 | 1.000 | 770 | tags=10%, list=4%, signal=10% |
| 19 | BIOCARTA\_ECM\_PATHWAY | Details ... | 23 | -0.40 | -1.12 | 0.279 | 1.000 | 1.000 | 4949 | tags=39%, list=25%, signal=52% |
| 20 | BIOCARTA\_CHREBP2\_PATHWAY | Details ... | 41 | -0.35 | -1.09 | 0.324 | 1.000 | 1.000 | 3185 | tags=24%, list=16%, signal=29% |
| 21 | BIOCARTA\_P38MAPK\_PATHWAY |  | 39 | -0.35 | -1.08 | 0.342 | 1.000 | 1.000 | 2560 | tags=26%, list=13%, signal=29% |
| 22 | BIOCARTA\_ERK5\_PATHWAY |  | 17 | -0.42 | -1.07 | 0.376 | 1.000 | 1.000 | 3226 | tags=24%, list=16%, signal=28% |
| 23 | BIOCARTA\_SHH\_PATHWAY |  | 15 | -0.42 | -1.06 | 0.382 | 1.000 | 1.000 | 4259 | tags=33%, list=22%, signal=43% |
| 24 | BIOCARTA\_IL10\_PATHWAY |  | 17 | -0.41 | -1.06 | 0.383 | 1.000 | 1.000 | 4066 | tags=29%, list=21%, signal=37% |
| 25 | BIOCARTA\_NO1\_PATHWAY |  | 28 | -0.37 | -1.05 | 0.384 | 1.000 | 1.000 | 3286 | tags=29%, list=17%, signal=34% |
| 26 | BIOCARTA\_IL12\_PATHWAY |  | 21 | -0.38 | -1.05 | 0.412 | 1.000 | 1.000 | 1599 | tags=14%, list=8%, signal=16% |
| 27 | BIOCARTA\_IL3\_PATHWAY |  | 15 | -0.42 | -1.04 | 0.409 | 1.000 | 1.000 | 3543 | tags=27%, list=18%, signal=33% |
| 28 | BIOCARTA\_DC\_PATHWAY |  | 22 | -0.38 | -1.03 | 0.417 | 1.000 | 1.000 | 5623 | tags=45%, list=29%, signal=64% |
| 29 | BIOCARTA\_VIP\_PATHWAY |  | 25 | -0.36 | -1.03 | 0.418 | 1.000 | 1.000 | 4259 | tags=32%, list=22%, signal=41% |
| 30 | BIOCARTA\_IL6\_PATHWAY |  | 22 | -0.38 | -1.02 | 0.409 | 1.000 | 1.000 | 766 | tags=9%, list=4%, signal=9% |
| 31 | BIOCARTA\_CARDIACEGF\_PATHWAY |  | 17 | -0.40 | -1.02 | 0.409 | 1.000 | 1.000 | 770 | tags=12%, list=4%, signal=12% |
| 32 | BIOCARTA\_TOLL\_PATHWAY |  | 34 | -0.34 | -1.02 | 0.451 | 1.000 | 1.000 | 266 | tags=6%, list=1%, signal=6% |
| 33 | BIOCARTA\_PDGF\_PATHWAY |  | 31 | -0.34 | -1.02 | 0.426 | 1.000 | 1.000 | 298 | tags=6%, list=2%, signal=7% |
| 34 | BIOCARTA\_G1\_PATHWAY |  | 26 | -0.35 | -1.00 | 0.449 | 1.000 | 1.000 | 801 | tags=8%, list=4%, signal=8% |
| 35 | BIOCARTA\_GLEEVEC\_PATHWAY |  | 23 | -0.37 | -1.00 | 0.433 | 1.000 | 1.000 | 4066 | tags=26%, list=21%, signal=33% |
| 36 | BIOCARTA\_IL2\_PATHWAY |  | 22 | -0.36 | -0.98 | 0.488 | 1.000 | 1.000 | 766 | tags=9%, list=4%, signal=9% |
| 37 | BIOCARTA\_IL22BP\_PATHWAY |  | 16 | -0.39 | -0.97 | 0.482 | 1.000 | 1.000 | 766 | tags=13%, list=4%, signal=13% |
| 38 | BIOCARTA\_GH\_PATHWAY |  | 26 | -0.33 | -0.96 | 0.507 | 1.000 | 1.000 | 3543 | tags=19%, list=18%, signal=23% |
| 39 | BIOCARTA\_CDMAC\_PATHWAY |  | 15 | -0.38 | -0.96 | 0.502 | 1.000 | 1.000 | 1979 | tags=13%, list=10%, signal=15% |
| 40 | BIOCARTA\_GPCR\_PATHWAY |  | 32 | -0.32 | -0.95 | 0.545 | 1.000 | 1.000 | 4259 | tags=22%, list=22%, signal=28% |
| 41 | BIOCARTA\_RHO\_PATHWAY |  | 31 | -0.32 | -0.95 | 0.536 | 0.993 | 1.000 | 6946 | tags=45%, list=36%, signal=70% |
| 42 | BIOCARTA\_TGFB\_PATHWAY |  | 18 | -0.37 | -0.95 | 0.527 | 0.979 | 1.000 | 7230 | tags=56%, list=37%, signal=88% |
| 43 | BIOCARTA\_STATHMIN\_PATHWAY |  | 17 | -0.37 | -0.94 | 0.525 | 0.967 | 1.000 | 4347 | tags=41%, list=22%, signal=53% |
| 44 | BIOCARTA\_HIVNEF\_PATHWAY |  | 55 | -0.29 | -0.94 | 0.548 | 0.951 | 1.000 | 3457 | tags=25%, list=18%, signal=31% |
| 45 | BIOCARTA\_RAC1\_PATHWAY |  | 23 | -0.34 | -0.94 | 0.547 | 0.935 | 1.000 | 3750 | tags=35%, list=19%, signal=43% |
| 46 | BIOCARTA\_MAPK\_PATHWAY |  | 85 | -0.26 | -0.92 | 0.581 | 0.957 | 1.000 | 2560 | tags=15%, list=13%, signal=18% |
| 47 | BIOCARTA\_LAIR\_PATHWAY |  | 16 | -0.37 | -0.91 | 0.566 | 0.960 | 1.000 | 6006 | tags=44%, list=31%, signal=63% |
| 48 | BIOCARTA\_INTRINSIC\_PATHWAY |  | 23 | -0.33 | -0.91 | 0.578 | 0.941 | 1.000 | 4361 | tags=35%, list=22%, signal=45% |
| 49 | BIOCARTA\_MET\_PATHWAY |  | 35 | -0.30 | -0.91 | 0.593 | 0.923 | 1.000 | 4949 | tags=29%, list=25%, signal=38% |
| 50 | BIOCARTA\_RAS\_PATHWAY |  | 22 | -0.33 | -0.91 | 0.580 | 0.908 | 1.000 | 3413 | tags=18%, list=17%, signal=22% |
| 51 | BIOCARTA\_CDC42RAC\_PATHWAY |  | 15 | -0.36 | -0.90 | 0.570 | 0.911 | 1.000 | 7649 | tags=60%, list=39%, signal=98% |
| 52 | BIOCARTA\_IL2RB\_PATHWAY |  | 38 | -0.29 | -0.89 | 0.665 | 0.924 | 1.000 | 766 | tags=8%, list=4%, signal=8% |
| 53 | BIOCARTA\_IL1R\_PATHWAY |  | 31 | -0.30 | -0.89 | 0.642 | 0.909 | 1.000 | 5798 | tags=42%, list=30%, signal=60% |
| 54 | BIOCARTA\_IL7\_PATHWAY |  | 17 | -0.34 | -0.88 | 0.605 | 0.898 | 1.000 | 4790 | tags=35%, list=24%, signal=47% |
| 55 | BIOCARTA\_CCR3\_PATHWAY |  | 22 | -0.32 | -0.88 | 0.637 | 0.890 | 1.000 | 5753 | tags=45%, list=29%, signal=64% |
| 56 | BIOCARTA\_TCR\_PATHWAY |  | 43 | -0.28 | -0.88 | 0.665 | 0.875 | 1.000 | 1869 | tags=9%, list=10%, signal=10% |
| 57 | BIOCARTA\_NFAT\_PATHWAY |  | 52 | -0.26 | -0.86 | 0.703 | 0.896 | 1.000 | 2270 | tags=15%, list=12%, signal=17% |
| 58 | BIOCARTA\_BCR\_PATHWAY |  | 33 | -0.28 | -0.85 | 0.681 | 0.898 | 1.000 | 1334 | tags=9%, list=7%, signal=10% |
| 59 | BIOCARTA\_NOS1\_PATHWAY |  | 19 | -0.32 | -0.85 | 0.679 | 0.897 | 1.000 | 4259 | tags=32%, list=22%, signal=40% |
| 60 | BIOCARTA\_AGR\_PATHWAY |  | 35 | -0.28 | -0.84 | 0.689 | 0.892 | 1.000 | 515 | tags=6%, list=3%, signal=6% |
| 61 | BIOCARTA\_EIF4\_PATHWAY |  | 22 | -0.31 | -0.84 | 0.727 | 0.883 | 1.000 | 3226 | tags=23%, list=16%, signal=27% |
| 62 | BIOCARTA\_IL17\_PATHWAY |  | 16 | -0.33 | -0.83 | 0.712 | 0.890 | 1.000 | 1599 | tags=19%, list=8%, signal=20% |
| 63 | BIOCARTA\_MITOCHONDRIA\_PATHWAY |  | 18 | -0.31 | -0.82 | 0.703 | 0.887 | 1.000 | 3413 | tags=28%, list=17%, signal=34% |
| 64 | BIOCARTA\_AT1R\_PATHWAY |  | 31 | -0.27 | -0.80 | 0.745 | 0.901 | 1.000 | 4949 | tags=32%, list=25%, signal=43% |
| 65 | BIOCARTA\_CALCINEURIN\_PATHWAY |  | 17 | -0.31 | -0.80 | 0.729 | 0.902 | 1.000 | 2491 | tags=18%, list=13%, signal=20% |
| 66 | BIOCARTA\_HDAC\_PATHWAY |  | 28 | -0.27 | -0.79 | 0.762 | 0.904 | 1.000 | 2125 | tags=14%, list=11%, signal=16% |
| 67 | BIOCARTA\_GCR\_PATHWAY |  | 19 | -0.29 | -0.78 | 0.752 | 0.901 | 1.000 | 1869 | tags=11%, list=10%, signal=12% |
| 68 | BIOCARTA\_ACTINY\_PATHWAY |  | 19 | -0.29 | -0.76 | 0.774 | 0.913 | 1.000 | 6946 | tags=53%, list=36%, signal=82% |
| 69 | BIOCARTA\_41BB\_PATHWAY |  | 17 | -0.30 | -0.76 | 0.775 | 0.905 | 1.000 | 5676 | tags=47%, list=29%, signal=66% |
| 70 | BIOCARTA\_TOB1\_PATHWAY |  | 20 | -0.28 | -0.75 | 0.808 | 0.903 | 1.000 | 1599 | tags=15%, list=8%, signal=16% |
| 71 | BIOCARTA\_BIOPEPTIDES\_PATHWAY |  | 41 | -0.24 | -0.75 | 0.849 | 0.892 | 1.000 | 5676 | tags=32%, list=29%, signal=45% |
| 72 | BIOCARTA\_PAR1\_PATHWAY |  | 35 | -0.24 | -0.72 | 0.870 | 0.918 | 1.000 | 4184 | tags=26%, list=21%, signal=33% |
| 73 | BIOCARTA\_KERATINOCYTE\_PATHWAY |  | 44 | -0.23 | -0.71 | 0.906 | 0.920 | 1.000 | 3961 | tags=23%, list=20%, signal=28% |
| 74 | BIOCARTA\_CXCR4\_PATHWAY |  | 23 | -0.23 | -0.66 | 0.925 | 0.961 | 1.000 | 4949 | tags=35%, list=25%, signal=47% |
| 75 | BIOCARTA\_FCER1\_PATHWAY |  | 37 | -0.21 | -0.65 | 0.948 | 0.955 | 1.000 | 1869 | tags=8%, list=10%, signal=9% |
| 76 | BIOCARTA\_PTEN\_PATHWAY |  | 17 | -0.24 | -0.63 | 0.922 | 0.960 | 1.000 | 6481 | tags=47%, list=33%, signal=70% |
| 77 | BIOCARTA\_CERAMIDE\_PATHWAY |  | 21 | -0.18 | -0.47 | 0.993 | 1.000 | 1.000 | 5573 | tags=24%, list=28%, signal=33% |
| 78 | BIOCARTA\_HCMV\_PATHWAY |  | 16 | -0.17 | -0.43 | 0.998 | 0.997 | 1.000 | 5813 | tags=38%, list=30%, signal=53% |
Table: Gene sets enriched in phenotype **b (3 samples) **[plain text format]****
