# Supplementary material for: Comprehensive analysis of β-catenin target genes in colorectal carcinoma cell lines with deregulated Wnt/β-catenin signaling
Source: BMC Genomics. 2014 Jan 28;15:74. doi: 10.1186/1471-2164-15-74 (PMC3909937; doi:10.1186/1471-2164-15-74)
Supplement: Additional file 4 — GSEA analysis using the Biocarta pathway database. This zipped file contains confirming data of the GSEA analysis. The names of the directories containing the files were composed of the term ‘GSEA’, the name of the cell line, e.g. DLD1, SW480, or LS174T, and the pathway database (Biocarta). Please use a web browser to view the files with the name ‘index.html’ in the corresponding directories to start exploring the data. [file 1471-2164-15-74-S4.zip › LS174T_Biocarta/gsea_report_for_bg_1355840256140.html]

Report for bg 1355840256140 [GSEA]

| GS  follow link to MSigDB | GS DETAILS | SIZE | ES | NES | NOM p-val | FDR q-val | FWER p-val | RANK AT MAX | LEADING EDGE || 1 | BIOCARTA\_ATRBRCA\_PATHWAY | Details ... | 21 | 0.73 | 2.05 | 0.000 | 0.004 | 0.005 | 1218 | tags=48%, list=6%, signal=51% |
| 2 | BIOCARTA\_G2\_PATHWAY | Details ... | 23 | 0.66 | 1.87 | 0.000 | 0.044 | 0.086 | 2932 | tags=39%, list=15%, signal=46% |
| 3 | BIOCARTA\_WNT\_PATHWAY | Details ... | 25 | 0.64 | 1.86 | 0.002 | 0.035 | 0.104 | 1540 | tags=28%, list=8%, signal=30% |
| 4 | BIOCARTA\_GSK3\_PATHWAY | Details ... | 27 | 0.59 | 1.80 | 0.006 | 0.045 | 0.177 | 4254 | tags=48%, list=22%, signal=61% |
| 5 | BIOCARTA\_P53\_PATHWAY | Details ... | 16 | 0.68 | 1.75 | 0.011 | 0.068 | 0.296 | 2764 | tags=44%, list=14%, signal=51% |
| 6 | BIOCARTA\_ATM\_PATHWAY | Details ... | 20 | 0.59 | 1.65 | 0.008 | 0.141 | 0.590 | 5319 | tags=55%, list=27%, signal=75% |
| 7 | BIOCARTA\_TEL\_PATHWAY | Details ... | 18 | 0.59 | 1.58 | 0.018 | 0.214 | 0.809 | 3626 | tags=56%, list=19%, signal=68% |
| 8 | BIOCARTA\_TFF\_PATHWAY | Details ... | 21 | 0.55 | 1.56 | 0.031 | 0.212 | 0.846 | 1273 | tags=14%, list=7%, signal=15% |
| 9 | BIOCARTA\_PROTEASOME\_PATHWAY | Details ... | 26 | 0.52 | 1.52 | 0.031 | 0.251 | 0.917 | 8260 | tags=73%, list=42%, signal=126% |
| 10 | BIOCARTA\_RACCYCD\_PATHWAY | Details ... | 26 | 0.48 | 1.42 | 0.064 | 0.429 | 0.994 | 3603 | tags=35%, list=18%, signal=42% |
| 11 | BIOCARTA\_ETS\_PATHWAY | Details ... | 18 | 0.51 | 1.36 | 0.112 | 0.545 | 0.998 | 3859 | tags=56%, list=20%, signal=69% |
| 12 | BIOCARTA\_ACH\_PATHWAY | Details ... | 15 | 0.51 | 1.30 | 0.176 | 0.698 | 0.999 | 1273 | tags=27%, list=7%, signal=29% |
| 13 | BIOCARTA\_CELLCYCLE\_PATHWAY | Details ... | 22 | 0.46 | 1.29 | 0.146 | 0.683 | 0.999 | 1372 | tags=41%, list=7%, signal=44% |
| 14 | BIOCARTA\_ALK\_PATHWAY | Details ... | 36 | 0.41 | 1.28 | 0.128 | 0.664 | 0.999 | 1616 | tags=19%, list=8%, signal=21% |
| 15 | BIOCARTA\_MPR\_PATHWAY | Details ... | 31 | 0.41 | 1.24 | 0.162 | 0.751 | 0.999 | 2150 | tags=19%, list=11%, signal=22% |
| 16 | BIOCARTA\_CYTOKINE\_PATHWAY | Details ... | 21 | 0.41 | 1.16 | 0.255 | 0.963 | 1.000 | 1194 | tags=24%, list=6%, signal=25% |
| 17 | BIOCARTA\_STEM\_PATHWAY | Details ... | 15 | 0.45 | 1.16 | 0.296 | 0.914 | 1.000 | 1194 | tags=20%, list=6%, signal=21% |
| 18 | BIOCARTA\_INFLAM\_PATHWAY | Details ... | 29 | 0.38 | 1.14 | 0.265 | 0.920 | 1.000 | 1194 | tags=17%, list=6%, signal=18% |
| 19 | BIOCARTA\_SPRY\_PATHWAY | Details ... | 18 | 0.42 | 1.14 | 0.284 | 0.879 | 1.000 | 1911 | tags=22%, list=10%, signal=25% |
| 20 | BIOCARTA\_ARF\_PATHWAY | Details ... | 17 | 0.43 | 1.13 | 0.281 | 0.857 | 1.000 | 2857 | tags=29%, list=15%, signal=34% |
| 21 | BIOCARTA\_PTDINS\_PATHWAY |  | 23 | 0.39 | 1.12 | 0.282 | 0.849 | 1.000 | 6559 | tags=52%, list=34%, signal=78% |
| 22 | BIOCARTA\_ERK\_PATHWAY |  | 27 | 0.38 | 1.12 | 0.293 | 0.813 | 1.000 | 1540 | tags=19%, list=8%, signal=20% |
| 23 | BIOCARTA\_EDG1\_PATHWAY |  | 24 | 0.37 | 1.10 | 0.345 | 0.855 | 1.000 | 4188 | tags=38%, list=21%, signal=48% |
| 24 | BIOCARTA\_HSP27\_PATHWAY |  | 15 | 0.42 | 1.06 | 0.398 | 0.941 | 1.000 | 5730 | tags=33%, list=29%, signal=47% |
| 25 | BIOCARTA\_SPPA\_PATHWAY |  | 19 | 0.39 | 1.06 | 0.390 | 0.904 | 1.000 | 2447 | tags=26%, list=13%, signal=30% |
| 26 | BIOCARTA\_BARRESTIN\_SRC\_PATHWAY |  | 15 | 0.42 | 1.05 | 0.403 | 0.909 | 1.000 | 4349 | tags=40%, list=22%, signal=51% |
| 27 | BIOCARTA\_MAL\_PATHWAY |  | 18 | 0.38 | 1.03 | 0.426 | 0.909 | 1.000 | 2150 | tags=17%, list=11%, signal=19% |
| 28 | BIOCARTA\_IGF1MTOR\_PATHWAY |  | 19 | 0.37 | 1.02 | 0.420 | 0.919 | 1.000 | 6157 | tags=37%, list=31%, signal=54% |
| 29 | BIOCARTA\_MEF2D\_PATHWAY |  | 18 | 0.37 | 1.00 | 0.476 | 0.951 | 1.000 | 3459 | tags=28%, list=18%, signal=34% |
| 30 | BIOCARTA\_CARM\_ER\_PATHWAY |  | 32 | 0.33 | 1.00 | 0.489 | 0.922 | 1.000 | 4383 | tags=41%, list=22%, signal=52% |
| 31 | BIOCARTA\_STRESS\_PATHWAY |  | 24 | 0.32 | 0.93 | 0.566 | 1.000 | 1.000 | 3603 | tags=25%, list=18%, signal=31% |
| 32 | BIOCARTA\_NDKDYNAMIN\_PATHWAY |  | 17 | 0.35 | 0.91 | 0.591 | 1.000 | 1.000 | 2298 | tags=24%, list=12%, signal=27% |
| 33 | BIOCARTA\_HER2\_PATHWAY |  | 22 | 0.31 | 0.88 | 0.649 | 1.000 | 1.000 | 6609 | tags=55%, list=34%, signal=82% |
| 34 | BIOCARTA\_CTLA4\_PATHWAY |  | 20 | 0.32 | 0.88 | 0.624 | 1.000 | 1.000 | 5670 | tags=45%, list=29%, signal=63% |
| 35 | BIOCARTA\_TALL1\_PATHWAY |  | 15 | 0.33 | 0.86 | 0.640 | 1.000 | 1.000 | 4927 | tags=33%, list=25%, signal=45% |
| 36 | BIOCARTA\_CHEMICAL\_PATHWAY |  | 21 | 0.31 | 0.85 | 0.692 | 1.000 | 1.000 | 4107 | tags=33%, list=21%, signal=42% |
| 37 | BIOCARTA\_P53HYPOXIA\_PATHWAY |  | 22 | 0.30 | 0.85 | 0.679 | 1.000 | 1.000 | 2459 | tags=23%, list=13%, signal=26% |
| 38 | BIOCARTA\_CK1\_PATHWAY |  | 16 | 0.32 | 0.83 | 0.719 | 1.000 | 1.000 | 88 | tags=6%, list=0%, signal=6% |
| 39 | BIOCARTA\_MTOR\_PATHWAY |  | 21 | 0.30 | 0.83 | 0.704 | 1.000 | 1.000 | 2433 | tags=19%, list=12%, signal=22% |
| 40 | BIOCARTA\_TNFR1\_PATHWAY |  | 29 | 0.27 | 0.82 | 0.729 | 1.000 | 1.000 | 2932 | tags=24%, list=15%, signal=28% |
| 41 | BIOCARTA\_FAS\_PATHWAY |  | 30 | 0.26 | 0.79 | 0.800 | 1.000 | 1.000 | 4110 | tags=37%, list=21%, signal=46% |
| 42 | BIOCARTA\_TID\_PATHWAY |  | 19 | 0.28 | 0.77 | 0.804 | 1.000 | 1.000 | 3603 | tags=32%, list=18%, signal=39% |
| 43 | BIOCARTA\_TH1TH2\_PATHWAY |  | 19 | 0.28 | 0.76 | 0.793 | 1.000 | 1.000 | 3182 | tags=21%, list=16%, signal=25% |
| 44 | BIOCARTA\_TNFR2\_PATHWAY |  | 18 | 0.28 | 0.75 | 0.793 | 1.000 | 1.000 | 5333 | tags=39%, list=27%, signal=53% |
| 45 | BIOCARTA\_CSK\_PATHWAY |  | 22 | 0.26 | 0.74 | 0.837 | 1.000 | 1.000 | 5438 | tags=36%, list=28%, signal=50% |
| 46 | BIOCARTA\_NTHI\_PATHWAY |  | 23 | 0.26 | 0.73 | 0.858 | 1.000 | 1.000 | 5333 | tags=30%, list=27%, signal=42% |
| 47 | BIOCARTA\_PML\_PATHWAY |  | 17 | 0.26 | 0.70 | 0.854 | 1.000 | 1.000 | 3839 | tags=24%, list=20%, signal=29% |
| 48 | BIOCARTA\_CREB\_PATHWAY |  | 25 | 0.23 | 0.68 | 0.921 | 1.000 | 1.000 | 2447 | tags=12%, list=13%, signal=14% |
| 49 | BIOCARTA\_CD40\_PATHWAY |  | 15 | 0.25 | 0.66 | 0.912 | 1.000 | 1.000 | 5333 | tags=33%, list=27%, signal=46% |
| 50 | BIOCARTA\_NKT\_PATHWAY |  | 27 | 0.22 | 0.65 | 0.940 | 1.000 | 1.000 | 3182 | tags=15%, list=16%, signal=18% |
| 51 | BIOCARTA\_NFKB\_PATHWAY |  | 22 | 0.21 | 0.60 | 0.957 | 1.000 | 1.000 | 3603 | tags=18%, list=18%, signal=22% |
| 52 | BIOCARTA\_UCALPAIN\_PATHWAY |  | 16 | 0.23 | 0.60 | 0.938 | 1.000 | 1.000 | 3987 | tags=25%, list=20%, signal=31% |
| 53 | BIOCARTA\_FMLP\_PATHWAY |  | 34 | 0.19 | 0.60 | 0.976 | 1.000 | 1.000 | 4927 | tags=29%, list=25%, signal=39% |
| 54 | BIOCARTA\_VEGF\_PATHWAY |  | 27 | 0.19 | 0.58 | 0.972 | 1.000 | 1.000 | 3991 | tags=26%, list=20%, signal=33% |
| 55 | BIOCARTA\_CTCF\_PATHWAY |  | 22 | 0.20 | 0.56 | 0.971 | 1.000 | 1.000 | 2459 | tags=14%, list=13%, signal=16% |
| 56 | BIOCARTA\_RELA\_PATHWAY |  | 16 | 0.21 | 0.55 | 0.976 | 1.000 | 1.000 | 4927 | tags=25%, list=25%, signal=33% |
| 57 | BIOCARTA\_MYOSIN\_PATHWAY |  | 30 | 0.17 | 0.52 | 0.996 | 1.000 | 1.000 | 4072 | tags=17%, list=21%, signal=21% |
| 58 | BIOCARTA\_INTEGRIN\_PATHWAY |  | 37 | 0.16 | 0.52 | 0.998 | 1.000 | 1.000 | 4072 | tags=16%, list=21%, signal=20% |
| 59 | BIOCARTA\_AKT\_PATHWAY |  | 19 | 0.17 | 0.45 | 0.996 | 1.000 | 1.000 | 6603 | tags=37%, list=34%, signal=56% |
| 60 | BIOCARTA\_PYK2\_PATHWAY |  | 26 | 0.14 | 0.41 | 1.000 | 0.999 | 1.000 | 6946 | tags=35%, list=36%, signal=54% |
Table: Gene sets enriched in phenotype **bg (3 samples) **[plain text format]****
