# Supplementary material for: Comprehensive analysis of β-catenin target genes in colorectal carcinoma cell lines with deregulated Wnt/β-catenin signaling
Source: BMC Genomics. 2014 Jan 28;15:74. doi: 10.1186/1471-2164-15-74 (PMC3909937; doi:10.1186/1471-2164-15-74)
Supplement: Additional file 4 — GSEA analysis using the Biocarta pathway database. This zipped file contains confirming data of the GSEA analysis. The names of the directories containing the files were composed of the term ‘GSEA’, the name of the cell line, e.g. DLD1, SW480, or LS174T, and the pathway database (Biocarta). Please use a web browser to view the files with the name ‘index.html’ in the corresponding directories to start exploring the data. [file 1471-2164-15-74-S4.zip › SW480_Biocarta/BIOCARTA_CARDIACEGF_PATHWAY.html]

Details for gene set BIOCARTA\_CARDIACEGF\_PATHWAY[GSEA]

|  || Dataset | SW480\_collapsed\_to\_symbols.class.cls#bg\_versus\_b |
| Phenotype | class.cls#bg\_versus\_b |
| Upregulated in class | bg |
| GeneSet | BIOCARTA\_CARDIACEGF\_PATHWAY |
| Enrichment Score (ES) | 0.36098185 |
| Normalized Enrichment Score (NES) | 0.97862005 |
| Nominal p-value | 0.497479 |
| FDR q-value | 0.8731029 |
| FWER p-Value | 1.0 |
Table: GSEA Results Summary

  

Fig 1: Enrichment plot: BIOCARTA\_CARDIACEGF\_PATHWAY      
 Profile of the Running ES Score & Positions of GeneSet Members on the Rank Ordered List

  

| PROBE | GENE SYMBOL | GENE\_TITLE | RANK IN GENE LIST | RANK METRIC SCORE | RUNNING ES | CORE ENRICHMENT || 1 | MYC | MYC Entrez,  Source | v-myc myelocytomatosis viral oncogene homolog (avian) | 447 | 0.262 | 0.1034 | Yes |
| 2 | AGT | AGT Entrez,  Source | angiotensinogen (serpin peptidase inhibitor, clade A, member 8) | 777 | 0.215 | 0.1904 | Yes |
| 3 | EDNRA | EDNRA Entrez,  Source | endothelin receptor type A | 1377 | 0.175 | 0.2441 | Yes |
| 4 | AGTR1 | AGTR1 Entrez,  Source | angiotensin II receptor, type 1 | 1842 | 0.155 | 0.2952 | Yes |
| 5 | EGF | EGF Entrez,  Source | epidermal growth factor (beta-urogastrone) | 1979 | 0.151 | 0.3610 | Yes |
| 6 | EDNRB | EDNRB Entrez,  Source | endothelin receptor type B | 3525 | 0.112 | 0.3359 | No |
| 7 | PLCG1 | PLCG1 Entrez,  Source | phospholipase C, gamma 1 | 4276 | 0.098 | 0.3451 | No |
| 8 | NFKB1 | NFKB1 Entrez,  Source | nuclear factor of kappa light polypeptide gene enhancer in B-cells 1 (p105) | 10617 | 0.018 | 0.0293 | No |
| 9 | ADAM12 | ADAM12 Entrez,  Source | ADAM metallopeptidase domain 12 (meltrin alpha) | 12018 | 0.001 | -0.0419 | No |
| 10 | HRAS | HRAS Entrez,  Source | v-Ha-ras Harvey rat sarcoma viral oncogene homolog | 12019 | 0.001 | -0.0413 | No |
| 11 | RELA | RELA Entrez,  Source | v-rel reticuloendotheliosis viral oncogene homolog A, nuclear factor of kappa light polypeptide gene enhancer in B-cells 3, p65 (avian) | 14051 | -0.027 | -0.1322 | No |
| 12 | RHOA | RHOA Entrez,  Source | ras homolog gene family, member A | 16217 | -0.072 | -0.2081 | No |
| 13 | EDN1 | EDN1 Entrez,  Source | endothelin 1 | 16252 | -0.073 | -0.1744 | No |
| 14 | PRKCA | PRKCA Entrez,  Source | protein kinase C, alpha | 17649 | -0.125 | -0.1854 | No |
| 15 | JUN | JUN Entrez,  Source | jun oncogene | 17831 | -0.133 | -0.1307 | No |
| 16 | FOS | FOS Entrez,  Source | v-fos FBJ murine osteosarcoma viral oncogene homolog | 18736 | -0.212 | -0.0743 | No |
| 17 | EGFR | EGFR Entrez,  Source | epidermal growth factor receptor (erythroblastic leukemia viral (v-erb-b) oncogene homolog, avian) | 18904 | -0.241 | 0.0334 | No |
Table: GSEA details [plain text format]

  

Fig 2: BIOCARTA\_CARDIACEGF\_PATHWAY      
 Blue-Pink O' Gram in the Space of the Analyzed GeneSet

  

Fig 3: BIOCARTA\_CARDIACEGF\_PATHWAY: Random ES distribution      
 Gene set null distribution of ES for **BIOCARTA\_CARDIACEGF\_PATHWAY**
