# Supplementary material for: Comprehensive analysis of β-catenin target genes in colorectal carcinoma cell lines with deregulated Wnt/β-catenin signaling
Source: BMC Genomics. 2014 Jan 28;15:74. doi: 10.1186/1471-2164-15-74 (PMC3909937; doi:10.1186/1471-2164-15-74)
Supplement: Additional file 4 — GSEA analysis using the Biocarta pathway database. This zipped file contains confirming data of the GSEA analysis. The names of the directories containing the files were composed of the term ‘GSEA’, the name of the cell line, e.g. DLD1, SW480, or LS174T, and the pathway database (Biocarta). Please use a web browser to view the files with the name ‘index.html’ in the corresponding directories to start exploring the data. [file 1471-2164-15-74-S4.zip › SW480_Biocarta/BIOCARTA_CERAMIDE_PATHWAY.html]

Details for gene set BIOCARTA\_CERAMIDE\_PATHWAY[GSEA]

|  || Dataset | SW480\_collapsed\_to\_symbols.class.cls#bg\_versus\_b |
| Phenotype | class.cls#bg\_versus\_b |
| Upregulated in class | b |
| GeneSet | BIOCARTA\_CERAMIDE\_PATHWAY |
| Enrichment Score (ES) | -0.5875271 |
| Normalized Enrichment Score (NES) | -1.7140824 |
| Nominal p-value | 0.0093896715 |
| FDR q-value | 0.1472518 |
| FWER p-Value | 0.474 |
Table: GSEA Results Summary

  

Fig 1: Enrichment plot: BIOCARTA\_CERAMIDE\_PATHWAY      
 Profile of the Running ES Score & Positions of GeneSet Members on the Rank Ordered List

  

| PROBE | GENE SYMBOL | GENE\_TITLE | RANK IN GENE LIST | RANK METRIC SCORE | RUNNING ES | CORE ENRICHMENT || 1 | NSMAF | NSMAF Entrez,  Source | neutral sphingomyelinase (N-SMase) activation associated factor | 6930 | 0.062 | -0.3158 | No |
| 2 | MAP2K4 | MAP2K4 Entrez,  Source | mitogen-activated protein kinase kinase 4 | 6974 | 0.061 | -0.2795 | No |
| 3 | MAPK3 | MAPK3 Entrez,  Source | mitogen-activated protein kinase 3 | 8976 | 0.037 | -0.3588 | No |
| 4 | MAPK1 | MAPK1 Entrez,  Source | mitogen-activated protein kinase 1 | 9552 | 0.030 | -0.3694 | No |
| 5 | RAF1 | RAF1 Entrez,  Source | v-raf-1 murine leukemia viral oncogene homolog 1 | 10520 | 0.019 | -0.4068 | No |
| 6 | NFKB1 | NFKB1 Entrez,  Source | nuclear factor of kappa light polypeptide gene enhancer in B-cells 1 (p105) | 10617 | 0.018 | -0.4005 | No |
| 7 | CYCS | CYCS Entrez,  Source | cytochrome c, somatic | 10860 | 0.015 | -0.4035 | No |
| 8 | BCL2 | BCL2 Entrez,  Source | B-cell CLL/lymphoma 2 | 11273 | 0.010 | -0.4182 | No |
| 9 | MAP2K1 | MAP2K1 Entrez,  Source | mitogen-activated protein kinase kinase 1 | 13246 | -0.015 | -0.5097 | No |
| 10 | TRAF2 | TRAF2 Entrez,  Source | TNF receptor-associated factor 2 | 13715 | -0.022 | -0.5198 | No |
| 11 | RELA | RELA Entrez,  Source | v-rel reticuloendotheliosis viral oncogene homolog A, nuclear factor of kappa light polypeptide gene enhancer in B-cells 3, p65 (avian) | 14051 | -0.027 | -0.5200 | No |
| 12 | BAX | BAX Entrez,  Source | BCL2-associated X protein | 15022 | -0.044 | -0.5418 | Yes |
| 13 | MAPK8 | MAPK8 Entrez,  Source | mitogen-activated protein kinase 8 | 15249 | -0.049 | -0.5227 | Yes |
| 14 | MAP3K1 | MAP3K1 Entrez,  Source | mitogen-activated protein kinase kinase kinase 1 | 16375 | -0.077 | -0.5320 | Yes |
| 15 | CASP8 | CASP8 Entrez,  Source | caspase 8, apoptosis-related cysteine peptidase | 17460 | -0.116 | -0.5145 | Yes |
| 16 | FADD | FADD Entrez,  Source | Fas (TNFRSF6)-associated via death domain | 17845 | -0.133 | -0.4504 | Yes |
| 17 | RIPK1 | RIPK1 Entrez,  Source | receptor (TNFRSF)-interacting serine-threonine kinase 1 | 18281 | -0.164 | -0.3696 | Yes |
| 18 | BAD | BAD Entrez,  Source | BCL2-antagonist of cell death | 18306 | -0.166 | -0.2666 | Yes |
| 19 | TRADD | TRADD Entrez,  Source | TNFRSF1A-associated via death domain | 18311 | -0.166 | -0.1625 | Yes |
| 20 | TNFRSF1A | TNFRSF1A Entrez,  Source | tumor necrosis factor receptor superfamily, member 1A | 18320 | -0.167 | -0.0581 | Yes |
| 21 | SMPD1 | SMPD1 Entrez,  Source | sphingomyelin phosphodiesterase 1, acid lysosomal (acid sphingomyelinase) | 18590 | -0.193 | 0.0494 | Yes |
Table: GSEA details [plain text format]

  

Fig 2: BIOCARTA\_CERAMIDE\_PATHWAY      
 Blue-Pink O' Gram in the Space of the Analyzed GeneSet

  

Fig 3: BIOCARTA\_CERAMIDE\_PATHWAY: Random ES distribution      
 Gene set null distribution of ES for **BIOCARTA\_CERAMIDE\_PATHWAY**
