# Supplementary material for: Comprehensive analysis of β-catenin target genes in colorectal carcinoma cell lines with deregulated Wnt/β-catenin signaling
Source: BMC Genomics. 2014 Jan 28;15:74. doi: 10.1186/1471-2164-15-74 (PMC3909937; doi:10.1186/1471-2164-15-74)
Supplement: Additional file 4 — GSEA analysis using the Biocarta pathway database. This zipped file contains confirming data of the GSEA analysis. The names of the directories containing the files were composed of the term ‘GSEA’, the name of the cell line, e.g. DLD1, SW480, or LS174T, and the pathway database (Biocarta). Please use a web browser to view the files with the name ‘index.html’ in the corresponding directories to start exploring the data. [file 1471-2164-15-74-S4.zip › SW480_Biocarta/BIOCARTA_CHEMICAL_PATHWAY.html]

Details for gene set BIOCARTA\_CHEMICAL\_PATHWAY[GSEA]

|  || Dataset | SW480\_collapsed\_to\_symbols.class.cls#bg\_versus\_b |
| Phenotype | class.cls#bg\_versus\_b |
| Upregulated in class | b |
| GeneSet | BIOCARTA\_CHEMICAL\_PATHWAY |
| Enrichment Score (ES) | -0.5447879 |
| Normalized Enrichment Score (NES) | -1.6100194 |
| Nominal p-value | 0.02173913 |
| FDR q-value | 0.17335398 |
| FWER p-Value | 0.761 |
Table: GSEA Results Summary

  

Fig 1: Enrichment plot: BIOCARTA\_CHEMICAL\_PATHWAY      
 Profile of the Running ES Score & Positions of GeneSet Members on the Rank Ordered List

  

| PROBE | GENE SYMBOL | GENE\_TITLE | RANK IN GENE LIST | RANK METRIC SCORE | RUNNING ES | CORE ENRICHMENT || 1 | TP53 | TP53 Entrez,  Source | tumor protein p53 (Li-Fraumeni syndrome) | 4161 | 0.100 | -0.1497 | No |
| 2 | CASP9 | CASP9 Entrez,  Source | caspase 9, apoptosis-related cysteine peptidase | 4423 | 0.096 | -0.1025 | No |
| 3 | EIF2S1 | EIF2S1 Entrez,  Source | eukaryotic translation initiation factor 2, subunit 1 alpha, 35kDa | 9090 | 0.035 | -0.3190 | No |
| 4 | PARP1 | PARP1 Entrez,  Source | poly (ADP-ribose) polymerase family, member 1 | 9672 | 0.029 | -0.3307 | No |
| 5 | CASP6 | CASP6 Entrez,  Source | caspase 6, apoptosis-related cysteine peptidase | 10118 | 0.024 | -0.3386 | No |
| 6 | CYCS | CYCS Entrez,  Source | cytochrome c, somatic | 10860 | 0.015 | -0.3671 | No |
| 7 | BCL2 | BCL2 Entrez,  Source | B-cell CLL/lymphoma 2 | 11273 | 0.010 | -0.3817 | No |
| 8 | TLN1 | TLN1 Entrez,  Source | talin 1 | 11369 | 0.009 | -0.3808 | No |
| 9 | BCL2L1 | BCL2L1 Entrez,  Source | BCL2-like 1 | 11619 | 0.006 | -0.3897 | No |
| 10 | CASP3 | CASP3 Entrez,  Source | caspase 3, apoptosis-related cysteine peptidase | 13234 | -0.015 | -0.4630 | No |
| 11 | AKT1 | AKT1 Entrez,  Source | v-akt murine thymoma viral oncogene homolog 1 | 13261 | -0.015 | -0.4547 | No |
| 12 | BAX | BAX Entrez,  Source | BCL2-associated X protein | 15022 | -0.044 | -0.5168 | Yes |
| 13 | CASP7 | CASP7 Entrez,  Source | caspase 7, apoptosis-related cysteine peptidase | 15076 | -0.045 | -0.4908 | Yes |
| 14 | PTK2 | PTK2 Entrez,  Source | PTK2 protein tyrosine kinase 2 | 15243 | -0.049 | -0.4685 | Yes |
| 15 | ATM | ATM Entrez,  Source | ataxia telangiectasia mutated (includes complementation groups A, C and D) | 16541 | -0.082 | -0.4831 | Yes |
| 16 | STAT1 | STAT1 Entrez,  Source | signal transducer and activator of transcription 1, 91kDa | 16948 | -0.095 | -0.4438 | Yes |
| 17 | BID | BID Entrez,  Source | BH3 interacting domain death agonist | 17637 | -0.125 | -0.4004 | Yes |
| 18 | PRKCA | PRKCA Entrez,  Source | protein kinase C, alpha | 17649 | -0.125 | -0.3219 | Yes |
| 19 | BAD | BAD Entrez,  Source | BCL2-antagonist of cell death | 18306 | -0.166 | -0.2507 | Yes |
| 20 | PXN | PXN Entrez,  Source | paxillin | 18924 | -0.245 | -0.1277 | Yes |
| 21 | APAF1 | APAF1 Entrez,  Source | apoptotic peptidase activating factor | 18972 | -0.253 | 0.0299 | Yes |
Table: GSEA details [plain text format]

  

Fig 2: BIOCARTA\_CHEMICAL\_PATHWAY      
 Blue-Pink O' Gram in the Space of the Analyzed GeneSet

  

Fig 3: BIOCARTA\_CHEMICAL\_PATHWAY: Random ES distribution      
 Gene set null distribution of ES for **BIOCARTA\_CHEMICAL\_PATHWAY**
