# Supplementary material for: Comprehensive analysis of β-catenin target genes in colorectal carcinoma cell lines with deregulated Wnt/β-catenin signaling
Source: BMC Genomics. 2014 Jan 28;15:74. doi: 10.1186/1471-2164-15-74 (PMC3909937; doi:10.1186/1471-2164-15-74)
Supplement: Additional file 4 — GSEA analysis using the Biocarta pathway database. This zipped file contains confirming data of the GSEA analysis. The names of the directories containing the files were composed of the term ‘GSEA’, the name of the cell line, e.g. DLD1, SW480, or LS174T, and the pathway database (Biocarta). Please use a web browser to view the files with the name ‘index.html’ in the corresponding directories to start exploring the data. [file 1471-2164-15-74-S4.zip › SW480_Biocarta/BIOCARTA_CSK_PATHWAY.html]

Details for gene set BIOCARTA\_CSK\_PATHWAY[GSEA]

|  || Dataset | SW480\_collapsed\_to\_symbols.class.cls#bg\_versus\_b |
| Phenotype | class.cls#bg\_versus\_b |
| Upregulated in class | bg |
| GeneSet | BIOCARTA\_CSK\_PATHWAY |
| Enrichment Score (ES) | 0.46580312 |
| Normalized Enrichment Score (NES) | 1.3747349 |
| Nominal p-value | 0.09430605 |
| FDR q-value | 0.508243 |
| FWER p-Value | 1.0 |
Table: GSEA Results Summary

  

Fig 1: Enrichment plot: BIOCARTA\_CSK\_PATHWAY      
 Profile of the Running ES Score & Positions of GeneSet Members on the Rank Ordered List

  

| PROBE | GENE SYMBOL | GENE\_TITLE | RANK IN GENE LIST | RANK METRIC SCORE | RUNNING ES | CORE ENRICHMENT || 1 | TRA@ | TRA@ Entrez,  Source | T cell receptor alpha locus | 17 | 0.784 | 0.2216 | Yes |
| 2 | ZAP70 | ZAP70 Entrez,  Source | zeta-chain (TCR) associated protein kinase 70kDa | 28 | 0.697 | 0.4188 | Yes |
| 3 | LCK | LCK Entrez,  Source | lymphocyte-specific protein tyrosine kinase | 750 | 0.218 | 0.4437 | Yes |
| 4 | PRKACG | PRKACG Entrez,  Source | protein kinase, cAMP-dependent, catalytic, gamma | 1307 | 0.178 | 0.4658 | Yes |
| 5 | HLA-DRB1 | HLA-DRB1 Entrez,  Source | major histocompatibility complex, class II, DR beta 1 | 3320 | 0.116 | 0.3956 | No |
| 6 | ADCY1 | ADCY1 Entrez,  Source | adenylate cyclase 1 (brain) | 4140 | 0.101 | 0.3822 | No |
| 7 | CD3E | CD3E Entrez,  Source | CD3e molecule, epsilon (CD3-TCR complex) | 4333 | 0.098 | 0.4001 | No |
| 8 | PTPRC | PTPRC Entrez,  Source | protein tyrosine phosphatase, receptor type, C | 6720 | 0.065 | 0.2963 | No |
| 9 | CD4 | CD4 Entrez,  Source | CD4 molecule | 6773 | 0.064 | 0.3118 | No |
| 10 | CD3G | CD3G Entrez,  Source | CD3g molecule, gamma (CD3-TCR complex) | 7931 | 0.050 | 0.2666 | No |
| 11 | CD3D | CD3D Entrez,  Source | CD3d molecule, delta (CD3-TCR complex) | 8284 | 0.045 | 0.2615 | No |
| 12 | GNGT1 | GNGT1 Entrez,  Source | guanine nucleotide binding protein (G protein), gamma transducing activity polypeptide 1 | 8498 | 0.043 | 0.2628 | No |
| 13 | CD247 | CD247 Entrez,  Source | CD247 molecule | 10783 | 0.016 | 0.1504 | No |
| 14 | GNAS | GNAS Entrez,  Source | GNAS complex locus | 11149 | 0.012 | 0.1350 | No |
| 15 | PRKAR2A | PRKAR2A Entrez,  Source | protein kinase, cAMP-dependent, regulatory, type II, alpha | 11520 | 0.007 | 0.1182 | No |
| 16 | CSK | CSK Entrez,  Source | c-src tyrosine kinase | 12161 | -0.001 | 0.0856 | No |
| 17 | GNB1 | GNB1 Entrez,  Source | guanine nucleotide binding protein (G protein), beta polypeptide 1 | 14980 | -0.043 | -0.0464 | No |
| 18 | CREBBP | CREBBP Entrez,  Source | CREB binding protein (Rubinstein-Taybi syndrome) | 15257 | -0.049 | -0.0466 | No |
| 19 | PRKACB | PRKACB Entrez,  Source | protein kinase, cAMP-dependent, catalytic, beta | 15881 | -0.063 | -0.0606 | No |
| 20 | HLA-DRA | HLA-DRA Entrez,  Source | major histocompatibility complex, class II, DR alpha | 16100 | -0.069 | -0.0522 | No |
| 21 | PRKAR1A | PRKAR1A Entrez,  Source | protein kinase, cAMP-dependent, regulatory, type I, alpha (tissue specific extinguisher 1) | 18683 | -0.206 | -0.1260 | No |
| 22 | PRKAR2B | PRKAR2B Entrez,  Source | protein kinase, cAMP-dependent, regulatory, type II, beta | 19490 | -0.601 | 0.0034 | No |
Table: GSEA details [plain text format]

  

Fig 2: BIOCARTA\_CSK\_PATHWAY      
 Blue-Pink O' Gram in the Space of the Analyzed GeneSet

  

Fig 3: BIOCARTA\_CSK\_PATHWAY: Random ES distribution      
 Gene set null distribution of ES for **BIOCARTA\_CSK\_PATHWAY**
