# Supplementary material for: Comprehensive analysis of β-catenin target genes in colorectal carcinoma cell lines with deregulated Wnt/β-catenin signaling
Source: BMC Genomics. 2014 Jan 28;15:74. doi: 10.1186/1471-2164-15-74 (PMC3909937; doi:10.1186/1471-2164-15-74)
Supplement: Additional file 4 — GSEA analysis using the Biocarta pathway database. This zipped file contains confirming data of the GSEA analysis. The names of the directories containing the files were composed of the term ‘GSEA’, the name of the cell line, e.g. DLD1, SW480, or LS174T, and the pathway database (Biocarta). Please use a web browser to view the files with the name ‘index.html’ in the corresponding directories to start exploring the data. [file 1471-2164-15-74-S4.zip › SW480_Biocarta/BIOCARTA_CTLA4_PATHWAY.html]

Details for gene set BIOCARTA\_CTLA4\_PATHWAY[GSEA]

|  || Dataset | SW480\_collapsed\_to\_symbols.class.cls#bg\_versus\_b |
| Phenotype | class.cls#bg\_versus\_b |
| Upregulated in class | bg |
| GeneSet | BIOCARTA\_CTLA4\_PATHWAY |
| Enrichment Score (ES) | 0.5463506 |
| Normalized Enrichment Score (NES) | 1.5062016 |
| Nominal p-value | 0.032312926 |
| FDR q-value | 0.64849645 |
| FWER p-Value | 0.968 |
Table: GSEA Results Summary

  

Fig 1: Enrichment plot: BIOCARTA\_CTLA4\_PATHWAY      
 Profile of the Running ES Score & Positions of GeneSet Members on the Rank Ordered List

  

| PROBE | GENE SYMBOL | GENE\_TITLE | RANK IN GENE LIST | RANK METRIC SCORE | RUNNING ES | CORE ENRICHMENT || 1 | TRA@ | TRA@ Entrez,  Source | T cell receptor alpha locus | 17 | 0.784 | 0.3844 | Yes |
| 2 | LCK | LCK Entrez,  Source | lymphocyte-specific protein tyrosine kinase | 750 | 0.218 | 0.4539 | Yes |
| 3 | CD80 | CD80 Entrez,  Source | CD80 molecule | 2830 | 0.126 | 0.4093 | Yes |
| 4 | HLA-DRB1 | HLA-DRB1 Entrez,  Source | major histocompatibility complex, class II, DR beta 1 | 3320 | 0.116 | 0.4411 | Yes |
| 5 | CD3E | CD3E Entrez,  Source | CD3e molecule, epsilon (CD3-TCR complex) | 4333 | 0.098 | 0.4373 | Yes |
| 6 | ICOS | ICOS Entrez,  Source | inducible T-cell co-stimulator | 4337 | 0.098 | 0.4851 | Yes |
| 7 | IL2 | IL2 Entrez,  Source | interleukin 2 | 5108 | 0.085 | 0.4877 | Yes |
| 8 | CTLA4 | CTLA4 Entrez,  Source | cytotoxic T-lymphocyte-associated protein 4 | 5189 | 0.084 | 0.5250 | Yes |
| 9 | ICOSLG | ICOSLG Entrez,  Source | inducible T-cell co-stimulator ligand | 5539 | 0.080 | 0.5464 | Yes |
| 10 | CD3G | CD3G Entrez,  Source | CD3g molecule, gamma (CD3-TCR complex) | 7931 | 0.050 | 0.4484 | No |
| 11 | PIK3CA | PIK3CA Entrez,  Source | phosphoinositide-3-kinase, catalytic, alpha polypeptide | 7968 | 0.049 | 0.4708 | No |
| 12 | CD3D | CD3D Entrez,  Source | CD3d molecule, delta (CD3-TCR complex) | 8284 | 0.045 | 0.4770 | No |
| 13 | CD28 | CD28 Entrez,  Source | CD28 molecule | 10014 | 0.025 | 0.4006 | No |
| 14 | PTPN11 | PTPN11 Entrez,  Source | protein tyrosine phosphatase, non-receptor type 11 (Noonan syndrome 1) | 10510 | 0.019 | 0.3848 | No |
| 15 | ITK | ITK Entrez,  Source | IL2-inducible T-cell kinase | 10562 | 0.019 | 0.3913 | No |
| 16 | CD247 | CD247 Entrez,  Source | CD247 molecule | 10783 | 0.016 | 0.3878 | No |
| 17 | PIK3R1 | PIK3R1 Entrez,  Source | phosphoinositide-3-kinase, regulatory subunit 1 (p85 alpha) | 13154 | -0.014 | 0.2732 | No |
| 18 | CD86 | CD86 Entrez,  Source | CD86 molecule | 13263 | -0.015 | 0.2752 | No |
| 19 | GRB2 | GRB2 Entrez,  Source | growth factor receptor-bound protein 2 | 13999 | -0.026 | 0.2504 | No |
| 20 | HLA-DRA | HLA-DRA Entrez,  Source | major histocompatibility complex, class II, DR alpha | 16100 | -0.069 | 0.1769 | No |
Table: GSEA details [plain text format]

  

Fig 2: BIOCARTA\_CTLA4\_PATHWAY      
 Blue-Pink O' Gram in the Space of the Analyzed GeneSet

  

Fig 3: BIOCARTA\_CTLA4\_PATHWAY: Random ES distribution      
 Gene set null distribution of ES for **BIOCARTA\_CTLA4\_PATHWAY**
