# Supplementary material for: Comprehensive analysis of β-catenin target genes in colorectal carcinoma cell lines with deregulated Wnt/β-catenin signaling
Source: BMC Genomics. 2014 Jan 28;15:74. doi: 10.1186/1471-2164-15-74 (PMC3909937; doi:10.1186/1471-2164-15-74)
Supplement: Additional file 4 — GSEA analysis using the Biocarta pathway database. This zipped file contains confirming data of the GSEA analysis. The names of the directories containing the files were composed of the term ‘GSEA’, the name of the cell line, e.g. DLD1, SW480, or LS174T, and the pathway database (Biocarta). Please use a web browser to view the files with the name ‘index.html’ in the corresponding directories to start exploring the data. [file 1471-2164-15-74-S4.zip › SW480_Biocarta/BIOCARTA_CXCR4_PATHWAY.html]

Details for gene set BIOCARTA\_CXCR4\_PATHWAY[GSEA]

|  || Dataset | SW480\_collapsed\_to\_symbols.class.cls#bg\_versus\_b |
| Phenotype | class.cls#bg\_versus\_b |
| Upregulated in class | bg |
| GeneSet | BIOCARTA\_CXCR4\_PATHWAY |
| Enrichment Score (ES) | 0.4309834 |
| Normalized Enrichment Score (NES) | 1.2347066 |
| Nominal p-value | 0.18705036 |
| FDR q-value | 0.7686498 |
| FWER p-Value | 1.0 |
Table: GSEA Results Summary

  

Fig 1: Enrichment plot: BIOCARTA\_CXCR4\_PATHWAY      
 Profile of the Running ES Score & Positions of GeneSet Members on the Rank Ordered List

  

| PROBE | GENE SYMBOL | GENE\_TITLE | RANK IN GENE LIST | RANK METRIC SCORE | RUNNING ES | CORE ENRICHMENT || 1 | GNAI1 | GNAI1 Entrez,  Source | guanine nucleotide binding protein (G protein), alpha inhibiting activity polypeptide 1 | 88 | 0.484 | 0.2279 | Yes |
| 2 | CXCR4 | CXCR4 Entrez,  Source | chemokine (C-X-C motif) receptor 4 | 133 | 0.428 | 0.4310 | Yes |
| 3 | PLCG1 | PLCG1 Entrez,  Source | phospholipase C, gamma 1 | 4276 | 0.098 | 0.2662 | No |
| 4 | CXCL12 | CXCL12 Entrez,  Source | chemokine (C-X-C motif) ligand 12 (stromal cell-derived factor 1) | 5082 | 0.086 | 0.2661 | No |
| 5 | PIK3C2G | PIK3C2G Entrez,  Source | phosphoinositide-3-kinase, class 2, gamma polypeptide | 5237 | 0.084 | 0.2984 | No |
| 6 | PIK3CA | PIK3CA Entrez,  Source | phosphoinositide-3-kinase, catalytic, alpha polypeptide | 7968 | 0.049 | 0.1824 | No |
| 7 | GNGT1 | GNGT1 Entrez,  Source | guanine nucleotide binding protein (G protein), gamma transducing activity polypeptide 1 | 8498 | 0.043 | 0.1759 | No |
| 8 | GNAQ | GNAQ Entrez,  Source | guanine nucleotide binding protein (G protein), q polypeptide | 8794 | 0.039 | 0.1795 | No |
| 9 | MAPK3 | MAPK3 Entrez,  Source | mitogen-activated protein kinase 3 | 8976 | 0.037 | 0.1878 | No |
| 10 | MAPK1 | MAPK1 Entrez,  Source | mitogen-activated protein kinase 1 | 9552 | 0.030 | 0.1728 | No |
| 11 | RAF1 | RAF1 Entrez,  Source | v-raf-1 murine leukemia viral oncogene homolog 1 | 10520 | 0.019 | 0.1325 | No |
| 12 | NFKB1 | NFKB1 Entrez,  Source | nuclear factor of kappa light polypeptide gene enhancer in B-cells 1 (p105) | 10617 | 0.018 | 0.1362 | No |
| 13 | HRAS | HRAS Entrez,  Source | v-Ha-ras Harvey rat sarcoma viral oncogene homolog | 12019 | 0.001 | 0.0650 | No |
| 14 | PIK3R1 | PIK3R1 Entrez,  Source | phosphoinositide-3-kinase, regulatory subunit 1 (p85 alpha) | 13154 | -0.014 | 0.0135 | No |
| 15 | CRK | CRK Entrez,  Source | v-crk sarcoma virus CT10 oncogene homolog (avian) | 13203 | -0.014 | 0.0179 | No |
| 16 | MAP2K1 | MAP2K1 Entrez,  Source | mitogen-activated protein kinase kinase 1 | 13246 | -0.015 | 0.0230 | No |
| 17 | RELA | RELA Entrez,  Source | v-rel reticuloendotheliosis viral oncogene homolog A, nuclear factor of kappa light polypeptide gene enhancer in B-cells 3, p65 (avian) | 14051 | -0.027 | -0.0052 | No |
| 18 | BCAR1 | BCAR1 Entrez,  Source | breast cancer anti-estrogen resistance 1 | 14524 | -0.035 | -0.0127 | No |
| 19 | GNB1 | GNB1 Entrez,  Source | guanine nucleotide binding protein (G protein), beta polypeptide 1 | 14980 | -0.043 | -0.0152 | No |
| 20 | PTK2 | PTK2 Entrez,  Source | PTK2 protein tyrosine kinase 2 | 15243 | -0.049 | -0.0052 | No |
| 21 | PTK2B | PTK2B Entrez,  Source | PTK2B protein tyrosine kinase 2 beta | 17068 | -0.100 | -0.0504 | No |
| 22 | PRKCA | PRKCA Entrez,  Source | protein kinase C, alpha | 17649 | -0.125 | -0.0200 | No |
| 23 | PXN | PXN Entrez,  Source | paxillin | 18924 | -0.245 | 0.0324 | No |
Table: GSEA details [plain text format]

  

Fig 2: BIOCARTA\_CXCR4\_PATHWAY      
 Blue-Pink O' Gram in the Space of the Analyzed GeneSet

  

Fig 3: BIOCARTA\_CXCR4\_PATHWAY: Random ES distribution      
 Gene set null distribution of ES for **BIOCARTA\_CXCR4\_PATHWAY**
