# Supplementary material for: Comprehensive analysis of β-catenin target genes in colorectal carcinoma cell lines with deregulated Wnt/β-catenin signaling
Source: BMC Genomics. 2014 Jan 28;15:74. doi: 10.1186/1471-2164-15-74 (PMC3909937; doi:10.1186/1471-2164-15-74)
Supplement: Additional file 4 — GSEA analysis using the Biocarta pathway database. This zipped file contains confirming data of the GSEA analysis. The names of the directories containing the files were composed of the term ‘GSEA’, the name of the cell line, e.g. DLD1, SW480, or LS174T, and the pathway database (Biocarta). Please use a web browser to view the files with the name ‘index.html’ in the corresponding directories to start exploring the data. [file 1471-2164-15-74-S4.zip › SW480_Biocarta/BIOCARTA_CYTOKINE_PATHWAY.html]

Details for gene set BIOCARTA\_CYTOKINE\_PATHWAY[GSEA]

|  || Dataset | SW480\_collapsed\_to\_symbols.class.cls#bg\_versus\_b |
| Phenotype | class.cls#bg\_versus\_b |
| Upregulated in class | bg |
| GeneSet | BIOCARTA\_CYTOKINE\_PATHWAY |
| Enrichment Score (ES) | 0.41229537 |
| Normalized Enrichment Score (NES) | 1.1555681 |
| Nominal p-value | 0.27845883 |
| FDR q-value | 0.86320555 |
| FWER p-Value | 1.0 |
Table: GSEA Results Summary

  

Fig 1: Enrichment plot: BIOCARTA\_CYTOKINE\_PATHWAY      
 Profile of the Running ES Score & Positions of GeneSet Members on the Rank Ordered List

  

| PROBE | GENE SYMBOL | GENE\_TITLE | RANK IN GENE LIST | RANK METRIC SCORE | RUNNING ES | CORE ENRICHMENT || 1 | IL10 | IL10 Entrez,  Source | interleukin 10 | 141 | 0.420 | 0.1250 | Yes |
| 2 | IL9 | IL9 Entrez,  Source | interleukin 9 | 479 | 0.256 | 0.1884 | Yes |
| 3 | IL17A | IL17A Entrez,  Source | interleukin 17A | 896 | 0.205 | 0.2316 | Yes |
| 4 | IL4 | IL4 Entrez,  Source | interleukin 4 | 1152 | 0.187 | 0.2775 | Yes |
| 5 | IL12A | IL12A Entrez,  Source | interleukin 12A (natural killer cell stimulatory factor 1, cytotoxic lymphocyte maturation factor 1, p35) | 1342 | 0.177 | 0.3236 | Yes |
| 6 | IFNA1 | IFNA1 Entrez,  Source | interferon, alpha 1 | 1482 | 0.169 | 0.3698 | Yes |
| 7 | TNF | TNF Entrez,  Source | tumor necrosis factor (TNF superfamily, member 2) | 2207 | 0.143 | 0.3779 | Yes |
| 8 | IL5 | IL5 Entrez,  Source | interleukin 5 (colony-stimulating factor, eosinophil) | 2824 | 0.126 | 0.3862 | Yes |
| 9 | IL13 | IL13 Entrez,  Source | interleukin 13 | 3058 | 0.121 | 0.4123 | Yes |
| 10 | IL16 | IL16 Entrez,  Source | interleukin 16 (lymphocyte chemoattractant factor) | 4717 | 0.091 | 0.3562 | No |
| 11 | LTA | LTA Entrez,  Source | lymphotoxin alpha (TNF superfamily, member 1) | 4963 | 0.087 | 0.3712 | No |
| 12 | IL2 | IL2 Entrez,  Source | interleukin 2 | 5108 | 0.085 | 0.3907 | No |
| 13 | IL3 | IL3 Entrez,  Source | interleukin 3 (colony-stimulating factor, multiple) | 6262 | 0.071 | 0.3539 | No |
| 14 | IFNB1 | IFNB1 Entrez,  Source | interferon, beta 1, fibroblast | 6791 | 0.064 | 0.3470 | No |
| 15 | IL8 | IL8 Entrez,  Source | interleukin 8 | 8936 | 0.037 | 0.2490 | No |
| 16 | IL6 | IL6 Entrez,  Source | interleukin 6 (interferon, beta 2) | 9771 | 0.028 | 0.2149 | No |
| 17 | IL12B | IL12B Entrez,  Source | interleukin 12B (natural killer cell stimulatory factor 2, cytotoxic lymphocyte maturation factor 2, p40) | 11809 | 0.004 | 0.1119 | No |
| 18 | IFNG | IFNG Entrez,  Source | interferon, gamma | 12592 | -0.006 | 0.0738 | No |
| 19 | IL18 | IL18 Entrez,  Source | interleukin 18 (interferon-gamma-inducing factor) | 14771 | -0.040 | -0.0252 | No |
| 20 | IL1A | IL1A Entrez,  Source | interleukin 1, alpha | 17739 | -0.129 | -0.1365 | No |
| 21 | IL15 | IL15 Entrez,  Source | interleukin 15 | 19526 | -0.728 | 0.0015 | No |
Table: GSEA details [plain text format]

  

Fig 2: BIOCARTA\_CYTOKINE\_PATHWAY      
 Blue-Pink O' Gram in the Space of the Analyzed GeneSet

  

Fig 3: BIOCARTA\_CYTOKINE\_PATHWAY: Random ES distribution      
 Gene set null distribution of ES for **BIOCARTA\_CYTOKINE\_PATHWAY**
