# Supplementary material for: Comprehensive analysis of β-catenin target genes in colorectal carcinoma cell lines with deregulated Wnt/β-catenin signaling
Source: BMC Genomics. 2014 Jan 28;15:74. doi: 10.1186/1471-2164-15-74 (PMC3909937; doi:10.1186/1471-2164-15-74)
Supplement: Additional file 4 — GSEA analysis using the Biocarta pathway database. This zipped file contains confirming data of the GSEA analysis. The names of the directories containing the files were composed of the term ‘GSEA’, the name of the cell line, e.g. DLD1, SW480, or LS174T, and the pathway database (Biocarta). Please use a web browser to view the files with the name ‘index.html’ in the corresponding directories to start exploring the data. [file 1471-2164-15-74-S4.zip › SW480_Biocarta/BIOCARTA_DC_PATHWAY.html]

Details for gene set BIOCARTA\_DC\_PATHWAY[GSEA]

|  || Dataset | SW480\_collapsed\_to\_symbols.class.cls#bg\_versus\_b |
| Phenotype | class.cls#bg\_versus\_b |
| Upregulated in class | bg |
| GeneSet | BIOCARTA\_DC\_PATHWAY |
| Enrichment Score (ES) | 0.4271579 |
| Normalized Enrichment Score (NES) | 1.2321467 |
| Nominal p-value | 0.17922948 |
| FDR q-value | 0.6911383 |
| FWER p-Value | 1.0 |
Table: GSEA Results Summary

  

Fig 1: Enrichment plot: BIOCARTA\_DC\_PATHWAY      
 Profile of the Running ES Score & Positions of GeneSet Members on the Rank Ordered List

  

| PROBE | GENE SYMBOL | GENE\_TITLE | RANK IN GENE LIST | RANK METRIC SCORE | RUNNING ES | CORE ENRICHMENT || 1 | IL10 | IL10 Entrez,  Source | interleukin 10 | 141 | 0.420 | 0.1137 | Yes |
| 2 | CD33 | CD33 Entrez,  Source | CD33 molecule | 193 | 0.371 | 0.2179 | Yes |
| 3 | TLR2 | TLR2 Entrez,  Source | toll-like receptor 2 | 630 | 0.233 | 0.2627 | Yes |
| 4 | IL4 | IL4 Entrez,  Source | interleukin 4 | 1152 | 0.187 | 0.2899 | Yes |
| 5 | IL12A | IL12A Entrez,  Source | interleukin 12A (natural killer cell stimulatory factor 1, cytotoxic lymphocyte maturation factor 1, p35) | 1342 | 0.177 | 0.3312 | Yes |
| 6 | IFNA1 | IFNA1 Entrez,  Source | interferon, alpha 1 | 1482 | 0.169 | 0.3729 | Yes |
| 7 | IL5 | IL5 Entrez,  Source | interleukin 5 (colony-stimulating factor, eosinophil) | 2824 | 0.126 | 0.3406 | Yes |
| 8 | IL13 | IL13 Entrez,  Source | interleukin 13 | 3058 | 0.121 | 0.3635 | Yes |
| 9 | CD5 | CD5 Entrez,  Source | CD5 molecule | 3655 | 0.109 | 0.3644 | Yes |
| 10 | CD7 | CD7 Entrez,  Source | CD7 molecule | 3874 | 0.105 | 0.3836 | Yes |
| 11 | ANPEP | ANPEP Entrez,  Source | alanyl (membrane) aminopeptidase (aminopeptidase N, aminopeptidase M, microsomal aminopeptidase, CD13, p150) | 4052 | 0.102 | 0.4040 | Yes |
| 12 | ITGAX | ITGAX Entrez,  Source | integrin, alpha X (complement component 3 receptor 4 subunit) | 4164 | 0.100 | 0.4272 | Yes |
| 13 | CD2 | CD2 Entrez,  Source | CD2 molecule | 5643 | 0.078 | 0.3741 | No |
| 14 | IL3 | IL3 Entrez,  Source | interleukin 3 (colony-stimulating factor, multiple) | 6262 | 0.071 | 0.3628 | No |
| 15 | CD40 | CD40 Entrez,  Source | CD40 molecule, TNF receptor superfamily member 5 | 6627 | 0.066 | 0.3631 | No |
| 16 | IFNB1 | IFNB1 Entrez,  Source | interferon, beta 1, fibroblast | 6791 | 0.064 | 0.3731 | No |
| 17 | TLR9 | TLR9 Entrez,  Source | toll-like receptor 9 | 7729 | 0.052 | 0.3401 | No |
| 18 | IL12B | IL12B Entrez,  Source | interleukin 12B (natural killer cell stimulatory factor 2, cytotoxic lymphocyte maturation factor 2, p40) | 11809 | 0.004 | 0.1324 | No |
| 19 | IFNG | IFNG Entrez,  Source | interferon, gamma | 12592 | -0.006 | 0.0942 | No |
| 20 | TLR7 | TLR7 Entrez,  Source | toll-like receptor 7 | 13366 | -0.017 | 0.0594 | No |
| 21 | CSF2 | CSF2 Entrez,  Source | colony stimulating factor 2 (granulocyte-macrophage) | 19229 | -0.328 | -0.1463 | No |
| 22 | TLR4 | TLR4 Entrez,  Source | toll-like receptor 4 | 19469 | -0.566 | 0.0045 | No |
Table: GSEA details [plain text format]

  

Fig 2: BIOCARTA\_DC\_PATHWAY      
 Blue-Pink O' Gram in the Space of the Analyzed GeneSet

  

Fig 3: BIOCARTA\_DC\_PATHWAY: Random ES distribution      
 Gene set null distribution of ES for **BIOCARTA\_DC\_PATHWAY**
