# Supplementary material for: Comprehensive analysis of β-catenin target genes in colorectal carcinoma cell lines with deregulated Wnt/β-catenin signaling
Source: BMC Genomics. 2014 Jan 28;15:74. doi: 10.1186/1471-2164-15-74 (PMC3909937; doi:10.1186/1471-2164-15-74)
Supplement: Additional file 4 — GSEA analysis using the Biocarta pathway database. This zipped file contains confirming data of the GSEA analysis. The names of the directories containing the files were composed of the term ‘GSEA’, the name of the cell line, e.g. DLD1, SW480, or LS174T, and the pathway database (Biocarta). Please use a web browser to view the files with the name ‘index.html’ in the corresponding directories to start exploring the data. [file 1471-2164-15-74-S4.zip › SW480_Biocarta/BIOCARTA_GCR_PATHWAY.html]

Details for gene set BIOCARTA\_GCR\_PATHWAY[GSEA]

|  || Dataset | SW480\_collapsed\_to\_symbols.class.cls#bg\_versus\_b |
| Phenotype | class.cls#bg\_versus\_b |
| Upregulated in class | b |
| GeneSet | BIOCARTA\_GCR\_PATHWAY |
| Enrichment Score (ES) | -0.62939125 |
| Normalized Enrichment Score (NES) | -1.7824726 |
| Nominal p-value | 0.015228426 |
| FDR q-value | 0.16499515 |
| FWER p-Value | 0.304 |
Table: GSEA Results Summary

  

Fig 1: Enrichment plot: BIOCARTA\_GCR\_PATHWAY      
 Profile of the Running ES Score & Positions of GeneSet Members on the Rank Ordered List

  

| PROBE | GENE SYMBOL | GENE\_TITLE | RANK IN GENE LIST | RANK METRIC SCORE | RUNNING ES | CORE ENRICHMENT || 1 | NPPA | NPPA Entrez,  Source | natriuretic peptide precursor A | 2888 | 0.125 | -0.0905 | No |
| 2 | PIK3CA | PIK3CA Entrez,  Source | phosphoinositide-3-kinase, catalytic, alpha polypeptide | 7968 | 0.049 | -0.3279 | No |
| 3 | GNGT1 | GNGT1 Entrez,  Source | guanine nucleotide binding protein (G protein), gamma transducing activity polypeptide 1 | 8498 | 0.043 | -0.3352 | No |
| 4 | CORIN | CORIN Entrez,  Source | corin, serine peptidase | 8660 | 0.041 | -0.3247 | No |
| 5 | PIK3CG | PIK3CG Entrez,  Source | phosphoinositide-3-kinase, catalytic, gamma polypeptide | 8761 | 0.039 | -0.3118 | No |
| 6 | NFKB1 | NFKB1 Entrez,  Source | nuclear factor of kappa light polypeptide gene enhancer in B-cells 1 (p105) | 10617 | 0.018 | -0.3985 | No |
| 7 | GNAS | GNAS Entrez,  Source | GNAS complex locus | 11149 | 0.012 | -0.4203 | No |
| 8 | PIK3R1 | PIK3R1 Entrez,  Source | phosphoinositide-3-kinase, regulatory subunit 1 (p85 alpha) | 13154 | -0.014 | -0.5166 | No |
| 9 | AKT1 | AKT1 Entrez,  Source | v-akt murine thymoma viral oncogene homolog 1 | 13261 | -0.015 | -0.5150 | No |
| 10 | HSP90AA1 | HSP90AA1 Entrez,  Source | heat shock protein 90kDa alpha (cytosolic), class A member 1 | 13951 | -0.025 | -0.5387 | No |
| 11 | RELA | RELA Entrez,  Source | v-rel reticuloendotheliosis viral oncogene homolog A, nuclear factor of kappa light polypeptide gene enhancer in B-cells 3, p65 (avian) | 14051 | -0.027 | -0.5313 | No |
| 12 | GNB1 | GNB1 Entrez,  Source | guanine nucleotide binding protein (G protein), beta polypeptide 1 | 14980 | -0.043 | -0.5589 | No |
| 13 | CALM2 | CALM2 Entrez,  Source | calmodulin 2 (phosphorylase kinase, delta) | 15530 | -0.055 | -0.5617 | No |
| 14 | CALM1 | CALM1 Entrez,  Source | calmodulin 1 (phosphorylase kinase, delta) | 15850 | -0.062 | -0.5495 | No |
| 15 | CALM3 | CALM3 Entrez,  Source | calmodulin 3 (phosphorylase kinase, delta) | 17323 | -0.110 | -0.5743 | Yes |
| 16 | NOS3 | NOS3 Entrez,  Source | nitric oxide synthase 3 (endothelial cell) | 18401 | -0.173 | -0.5498 | Yes |
| 17 | ANXA1 | ANXA1 Entrez,  Source | annexin A1 | 18857 | -0.234 | -0.4656 | Yes |
| 18 | NR3C1 | NR3C1 Entrez,  Source | nuclear receptor subfamily 3, group C, member 1 (glucocorticoid receptor) | 19352 | -0.398 | -0.3081 | Yes |
| 19 | ADRB2 | ADRB2 Entrez,  Source | adrenergic, beta-2-, receptor, surface | 19514 | -0.694 | 0.0021 | Yes |
Table: GSEA details [plain text format]

  

Fig 2: BIOCARTA\_GCR\_PATHWAY      
 Blue-Pink O' Gram in the Space of the Analyzed GeneSet

  

Fig 3: BIOCARTA\_GCR\_PATHWAY: Random ES distribution      
 Gene set null distribution of ES for **BIOCARTA\_GCR\_PATHWAY**
