# Supplementary material for: Comprehensive analysis of β-catenin target genes in colorectal carcinoma cell lines with deregulated Wnt/β-catenin signaling
Source: BMC Genomics. 2014 Jan 28;15:74. doi: 10.1186/1471-2164-15-74 (PMC3909937; doi:10.1186/1471-2164-15-74)
Supplement: Additional file 4 — GSEA analysis using the Biocarta pathway database. This zipped file contains confirming data of the GSEA analysis. The names of the directories containing the files were composed of the term ‘GSEA’, the name of the cell line, e.g. DLD1, SW480, or LS174T, and the pathway database (Biocarta). Please use a web browser to view the files with the name ‘index.html’ in the corresponding directories to start exploring the data. [file 1471-2164-15-74-S4.zip › SW480_Biocarta/BIOCARTA_GSK3_PATHWAY.html]

Details for gene set BIOCARTA\_GSK3\_PATHWAY[GSEA]

|  || Dataset | SW480\_collapsed\_to\_symbols.class.cls#bg\_versus\_b |
| Phenotype | class.cls#bg\_versus\_b |
| Upregulated in class | b |
| GeneSet | BIOCARTA\_GSK3\_PATHWAY |
| Enrichment Score (ES) | -0.5176488 |
| Normalized Enrichment Score (NES) | -1.5829464 |
| Nominal p-value | 0.026570048 |
| FDR q-value | 0.18497616 |
| FWER p-Value | 0.83 |
Table: GSEA Results Summary

  

Fig 1: Enrichment plot: BIOCARTA\_GSK3\_PATHWAY      
 Profile of the Running ES Score & Positions of GeneSet Members on the Rank Ordered List

  

| PROBE | GENE SYMBOL | GENE\_TITLE | RANK IN GENE LIST | RANK METRIC SCORE | RUNNING ES | CORE ENRICHMENT || 1 | GNAI1 | GNAI1 Entrez,  Source | guanine nucleotide binding protein (G protein), alpha inhibiting activity polypeptide 1 | 88 | 0.484 | 0.1355 | No |
| 2 | CTNNB1 | CTNNB1 Entrez,  Source | catenin (cadherin-associated protein), beta 1, 88kDa | 613 | 0.235 | 0.1767 | No |
| 3 | AXIN1 | AXIN1 Entrez,  Source | axin 1 | 2026 | 0.149 | 0.1475 | No |
| 4 | LEF1 | LEF1 Entrez,  Source | lymphoid enhancer-binding factor 1 | 7483 | 0.055 | -0.1159 | No |
| 5 | DVL1 | DVL1 Entrez,  Source | dishevelled, dsh homolog 1 (Drosophila) | 7661 | 0.053 | -0.1096 | No |
| 6 | WNT1 | WNT1 Entrez,  Source | wingless-type MMTV integration site family, member 1 | 7800 | 0.051 | -0.1019 | No |
| 7 | PIK3CA | PIK3CA Entrez,  Source | phosphoinositide-3-kinase, catalytic, alpha polypeptide | 7968 | 0.049 | -0.0962 | No |
| 8 | APC | APC Entrez,  Source | adenomatosis polyposis coli | 9898 | 0.026 | -0.1874 | No |
| 9 | PDPK1 | PDPK1 Entrez,  Source | 3-phosphoinositide dependent protein kinase-1 | 10499 | 0.019 | -0.2126 | No |
| 10 | NFKB1 | NFKB1 Entrez,  Source | nuclear factor of kappa light polypeptide gene enhancer in B-cells 1 (p105) | 10617 | 0.018 | -0.2134 | No |
| 11 | LBP | LBP Entrez,  Source | lipopolysaccharide binding protein | 10912 | 0.014 | -0.2243 | No |
| 12 | EIF2AK2 | EIF2AK2 Entrez,  Source | eukaryotic translation initiation factor 2-alpha kinase 2 | 11659 | 0.006 | -0.2608 | No |
| 13 | PPP2CA | PPP2CA Entrez,  Source | protein phosphatase 2 (formerly 2A), catalytic subunit, alpha isoform | 11890 | 0.003 | -0.2718 | No |
| 14 | CCND1 | CCND1 Entrez,  Source | cyclin D1 | 12074 | 0.000 | -0.2811 | No |
| 15 | GSK3B | GSK3B Entrez,  Source | glycogen synthase kinase 3 beta | 12732 | -0.008 | -0.3123 | No |
| 16 | PIK3R1 | PIK3R1 Entrez,  Source | phosphoinositide-3-kinase, regulatory subunit 1 (p85 alpha) | 13154 | -0.014 | -0.3299 | No |
| 17 | AKT1 | AKT1 Entrez,  Source | v-akt murine thymoma viral oncogene homolog 1 | 13261 | -0.015 | -0.3309 | No |
| 18 | RELA | RELA Entrez,  Source | v-rel reticuloendotheliosis viral oncogene homolog A, nuclear factor of kappa light polypeptide gene enhancer in B-cells 3, p65 (avian) | 14051 | -0.027 | -0.3635 | No |
| 19 | MYD88 | MYD88 Entrez,  Source | myeloid differentiation primary response gene (88) | 14431 | -0.033 | -0.3733 | No |
| 20 | IRAK1 | IRAK1 Entrez,  Source | interleukin-1 receptor-associated kinase 1 | 14621 | -0.036 | -0.3725 | No |
| 21 | TOLLIP | TOLLIP Entrez,  Source | toll interacting protein | 14995 | -0.044 | -0.3790 | No |
| 22 | GJA1 | GJA1 Entrez,  Source | gap junction protein, alpha 1, 43kDa (connexin 43) | 15948 | -0.065 | -0.4090 | No |
| 23 | TIRAP | TIRAP Entrez,  Source | toll-interleukin 1 receptor (TIR) domain containing adaptor protein | 16368 | -0.077 | -0.4083 | No |
| 24 | CD14 | CD14 Entrez,  Source | CD14 molecule | 18269 | -0.163 | -0.4584 | Yes |
| 25 | FZD1 | FZD1 Entrez,  Source | frizzled homolog 1 (Drosophila) | 19428 | -0.470 | -0.3817 | Yes |
| 26 | TLR4 | TLR4 Entrez,  Source | toll-like receptor 4 | 19469 | -0.566 | -0.2202 | Yes |
| 27 | LY96 | LY96 Entrez,  Source | lymphocyte antigen 96 | 19532 | -0.777 | 0.0012 | Yes |
Table: GSEA details [plain text format]

  

Fig 2: BIOCARTA\_GSK3\_PATHWAY      
 Blue-Pink O' Gram in the Space of the Analyzed GeneSet

  

Fig 3: BIOCARTA\_GSK3\_PATHWAY: Random ES distribution      
 Gene set null distribution of ES for **BIOCARTA\_GSK3\_PATHWAY**
