# Supplementary material for: Comprehensive analysis of β-catenin target genes in colorectal carcinoma cell lines with deregulated Wnt/β-catenin signaling
Source: BMC Genomics. 2014 Jan 28;15:74. doi: 10.1186/1471-2164-15-74 (PMC3909937; doi:10.1186/1471-2164-15-74)
Supplement: Additional file 4 — GSEA analysis using the Biocarta pathway database. This zipped file contains confirming data of the GSEA analysis. The names of the directories containing the files were composed of the term ‘GSEA’, the name of the cell line, e.g. DLD1, SW480, or LS174T, and the pathway database (Biocarta). Please use a web browser to view the files with the name ‘index.html’ in the corresponding directories to start exploring the data. [file 1471-2164-15-74-S4.zip › SW480_Biocarta/BIOCARTA_HSP27_PATHWAY.html]

Details for gene set BIOCARTA\_HSP27\_PATHWAY[GSEA]

|  || Dataset | SW480\_collapsed\_to\_symbols.class.cls#bg\_versus\_b |
| Phenotype | class.cls#bg\_versus\_b |
| Upregulated in class | bg |
| GeneSet | BIOCARTA\_HSP27\_PATHWAY |
| Enrichment Score (ES) | 0.3517215 |
| Normalized Enrichment Score (NES) | 0.9158782 |
| Nominal p-value | 0.5614618 |
| FDR q-value | 0.9339412 |
| FWER p-Value | 1.0 |
Table: GSEA Results Summary

  

Fig 1: Enrichment plot: BIOCARTA\_HSP27\_PATHWAY      
 Profile of the Running ES Score & Positions of GeneSet Members on the Rank Ordered List

  

| PROBE | GENE SYMBOL | GENE\_TITLE | RANK IN GENE LIST | RANK METRIC SCORE | RUNNING ES | CORE ENRICHMENT || 1 | FASLG | FASLG Entrez,  Source | Fas ligand (TNF superfamily, member 6) | 600 | 0.237 | 0.1268 | Yes |
| 2 | HSPB2 | HSPB2 Entrez,  Source | heat shock 27kDa protein 2 | 1510 | 0.168 | 0.1919 | Yes |
| 3 | ACTA1 | ACTA1 Entrez,  Source | actin, alpha 1, skeletal muscle | 1966 | 0.151 | 0.2688 | Yes |
| 4 | TNF | TNF Entrez,  Source | tumor necrosis factor (TNF superfamily, member 2) | 2207 | 0.143 | 0.3517 | Yes |
| 5 | CASP9 | CASP9 Entrez,  Source | caspase 9, apoptosis-related cysteine peptidase | 4423 | 0.096 | 0.3021 | No |
| 6 | MAPKAPK2 | MAPKAPK2 Entrez,  Source | mitogen-activated protein kinase-activated protein kinase 2 | 5223 | 0.084 | 0.3169 | No |
| 7 | DAXX | DAXX Entrez,  Source | death-associated protein 6 | 5620 | 0.079 | 0.3490 | No |
| 8 | HSPB1 | HSPB1 Entrez,  Source | heat shock 27kDa protein 1 | 10214 | 0.022 | 0.1288 | No |
| 9 | CYCS | CYCS Entrez,  Source | cytochrome c, somatic | 10860 | 0.015 | 0.1057 | No |
| 10 | BCL2 | BCL2 Entrez,  Source | B-cell CLL/lymphoma 2 | 11273 | 0.010 | 0.0915 | No |
| 11 | CASP3 | CASP3 Entrez,  Source | caspase 3, apoptosis-related cysteine peptidase | 13234 | -0.015 | 0.0009 | No |
| 12 | MAPKAPK3 | MAPKAPK3 Entrez,  Source | mitogen-activated protein kinase-activated protein kinase 3 | 15167 | -0.047 | -0.0665 | No |
| 13 | FAS | FAS Entrez,  Source | Fas (TNF receptor superfamily, member 6) | 15578 | -0.056 | -0.0503 | No |
| 14 | IL1A | IL1A Entrez,  Source | interleukin 1, alpha | 17739 | -0.129 | -0.0753 | No |
| 15 | APAF1 | APAF1 Entrez,  Source | apoptotic peptidase activating factor | 18972 | -0.253 | 0.0299 | No |
Table: GSEA details [plain text format]

  

Fig 2: BIOCARTA\_HSP27\_PATHWAY      
 Blue-Pink O' Gram in the Space of the Analyzed GeneSet

  

Fig 3: BIOCARTA\_HSP27\_PATHWAY: Random ES distribution      
 Gene set null distribution of ES for **BIOCARTA\_HSP27\_PATHWAY**
