# Supplementary material for: Comprehensive analysis of β-catenin target genes in colorectal carcinoma cell lines with deregulated Wnt/β-catenin signaling
Source: BMC Genomics. 2014 Jan 28;15:74. doi: 10.1186/1471-2164-15-74 (PMC3909937; doi:10.1186/1471-2164-15-74)
Supplement: Additional file 4 — GSEA analysis using the Biocarta pathway database. This zipped file contains confirming data of the GSEA analysis. The names of the directories containing the files were composed of the term ‘GSEA’, the name of the cell line, e.g. DLD1, SW480, or LS174T, and the pathway database (Biocarta). Please use a web browser to view the files with the name ‘index.html’ in the corresponding directories to start exploring the data. [file 1471-2164-15-74-S4.zip › SW480_Biocarta/BIOCARTA_IGF1MTOR_PATHWAY.html]

Details for gene set BIOCARTA\_IGF1MTOR\_PATHWAY[GSEA]

|  || Dataset | SW480\_collapsed\_to\_symbols.class.cls#bg\_versus\_b |
| Phenotype | class.cls#bg\_versus\_b |
| Upregulated in class | bg |
| GeneSet | BIOCARTA\_IGF1MTOR\_PATHWAY |
| Enrichment Score (ES) | 0.35653004 |
| Normalized Enrichment Score (NES) | 0.9992973 |
| Nominal p-value | 0.45826235 |
| FDR q-value | 0.9876958 |
| FWER p-Value | 1.0 |
Table: GSEA Results Summary

  

Fig 1: Enrichment plot: BIOCARTA\_IGF1MTOR\_PATHWAY      
 Profile of the Running ES Score & Positions of GeneSet Members on the Rank Ordered List

  

| PROBE | GENE SYMBOL | GENE\_TITLE | RANK IN GENE LIST | RANK METRIC SCORE | RUNNING ES | CORE ENRICHMENT || 1 | IGF1 | IGF1 Entrez,  Source | insulin-like growth factor 1 (somatomedin C) | 333 | 0.294 | 0.3565 | Yes |
| 2 | PIK3CA | PIK3CA Entrez,  Source | phosphoinositide-3-kinase, catalytic, alpha polypeptide | 7968 | 0.049 | 0.0285 | No |
| 3 | INPPL1 | INPPL1 Entrez,  Source | inositol polyphosphate phosphatase-like 1 | 8898 | 0.038 | 0.0291 | No |
| 4 | EIF2S1 | EIF2S1 Entrez,  Source | eukaryotic translation initiation factor 2, subunit 1 alpha, 35kDa | 9090 | 0.035 | 0.0643 | No |
| 5 | EIF2S3 | EIF2S3 Entrez,  Source | eukaryotic translation initiation factor 2, subunit 3 gamma, 52kDa | 9354 | 0.032 | 0.0921 | No |
| 6 | EIF4E | EIF4E Entrez,  Source | eukaryotic translation initiation factor 4E | 9694 | 0.028 | 0.1109 | No |
| 7 | EIF2S2 | EIF2S2 Entrez,  Source | eukaryotic translation initiation factor 2, subunit 2 beta, 38kDa | 10430 | 0.020 | 0.0988 | No |
| 8 | PDPK1 | PDPK1 Entrez,  Source | 3-phosphoinositide dependent protein kinase-1 | 10499 | 0.019 | 0.1200 | No |
| 9 | EIF2B5 | EIF2B5 Entrez,  Source | eukaryotic translation initiation factor 2B, subunit 5 epsilon, 82kDa | 11275 | 0.010 | 0.0933 | No |
| 10 | EIF4EBP1 | EIF4EBP1 Entrez,  Source | eukaryotic translation initiation factor 4E binding protein 1 | 11671 | 0.006 | 0.0803 | No |
| 11 | RPS6 | RPS6 Entrez,  Source | ribosomal protein S6 | 11691 | 0.005 | 0.0861 | No |
| 12 | PPP2CA | PPP2CA Entrez,  Source | protein phosphatase 2 (formerly 2A), catalytic subunit, alpha isoform | 11890 | 0.003 | 0.0794 | No |
| 13 | RPS6KB1 | RPS6KB1 Entrez,  Source | ribosomal protein S6 kinase, 70kDa, polypeptide 1 | 12289 | -0.002 | 0.0617 | No |
| 14 | GSK3B | GSK3B Entrez,  Source | glycogen synthase kinase 3 beta | 12732 | -0.008 | 0.0495 | No |
| 15 | PIK3R1 | PIK3R1 Entrez,  Source | phosphoinositide-3-kinase, regulatory subunit 1 (p85 alpha) | 13154 | -0.014 | 0.0453 | No |
| 16 | AKT1 | AKT1 Entrez,  Source | v-akt murine thymoma viral oncogene homolog 1 | 13261 | -0.015 | 0.0593 | No |
| 17 | PDK2 | PDK2 Entrez,  Source | pyruvate dehydrogenase kinase, isozyme 2 | 13331 | -0.016 | 0.0765 | No |
| 18 | PTEN | PTEN Entrez,  Source | phosphatase and tensin homolog (mutated in multiple advanced cancers 1) | 14353 | -0.032 | 0.0647 | No |
| 19 | IGF1R | IGF1R Entrez,  Source | insulin-like growth factor 1 receptor | 18204 | -0.158 | 0.0692 | No |
Table: GSEA details [plain text format]

  

Fig 2: BIOCARTA\_IGF1MTOR\_PATHWAY      
 Blue-Pink O' Gram in the Space of the Analyzed GeneSet

  

Fig 3: BIOCARTA\_IGF1MTOR\_PATHWAY: Random ES distribution      
 Gene set null distribution of ES for **BIOCARTA\_IGF1MTOR\_PATHWAY**
