# Supplementary material for: Comprehensive analysis of β-catenin target genes in colorectal carcinoma cell lines with deregulated Wnt/β-catenin signaling
Source: BMC Genomics. 2014 Jan 28;15:74. doi: 10.1186/1471-2164-15-74 (PMC3909937; doi:10.1186/1471-2164-15-74)
Supplement: Additional file 4 — GSEA analysis using the Biocarta pathway database. This zipped file contains confirming data of the GSEA analysis. The names of the directories containing the files were composed of the term ‘GSEA’, the name of the cell line, e.g. DLD1, SW480, or LS174T, and the pathway database (Biocarta). Please use a web browser to view the files with the name ‘index.html’ in the corresponding directories to start exploring the data. [file 1471-2164-15-74-S4.zip › SW480_Biocarta/BIOCARTA_IGF1R_PATHWAY.html]

Details for gene set BIOCARTA\_IGF1R\_PATHWAY[GSEA]

|  || Dataset | SW480\_collapsed\_to\_symbols.class.cls#bg\_versus\_b |
| Phenotype | class.cls#bg\_versus\_b |
| Upregulated in class | b |
| GeneSet | BIOCARTA\_IGF1R\_PATHWAY |
| Enrichment Score (ES) | -0.6951908 |
| Normalized Enrichment Score (NES) | -2.025372 |
| Nominal p-value | 0.0 |
| FDR q-value | 0.055920366 |
| FWER p-Value | 0.03 |
Table: GSEA Results Summary

  

Fig 1: Enrichment plot: BIOCARTA\_IGF1R\_PATHWAY      
 Profile of the Running ES Score & Positions of GeneSet Members on the Rank Ordered List

  

| PROBE | GENE SYMBOL | GENE\_TITLE | RANK IN GENE LIST | RANK METRIC SCORE | RUNNING ES | CORE ENRICHMENT || 1 | PRKACG | PRKACG Entrez,  Source | protein kinase, cAMP-dependent, catalytic, gamma | 1307 | 0.178 | -0.0057 | No |
| 2 | ADCY1 | ADCY1 Entrez,  Source | adenylate cyclase 1 (brain) | 4140 | 0.101 | -0.1162 | No |
| 3 | SOS1 | SOS1 Entrez,  Source | son of sevenless homolog 1 (Drosophila) | 7883 | 0.050 | -0.2905 | No |
| 4 | PIK3CA | PIK3CA Entrez,  Source | phosphoinositide-3-kinase, catalytic, alpha polypeptide | 7968 | 0.049 | -0.2779 | No |
| 5 | MAPK3 | MAPK3 Entrez,  Source | mitogen-activated protein kinase 3 | 8976 | 0.037 | -0.3169 | No |
| 6 | YWHAH | YWHAH Entrez,  Source | tyrosine 3-monooxygenase/tryptophan 5-monooxygenase activation protein, eta polypeptide | 9417 | 0.032 | -0.3285 | No |
| 7 | MAPK1 | MAPK1 Entrez,  Source | mitogen-activated protein kinase 1 | 9552 | 0.030 | -0.3251 | No |
| 8 | RAF1 | RAF1 Entrez,  Source | v-raf-1 murine leukemia viral oncogene homolog 1 | 10520 | 0.019 | -0.3680 | No |
| 9 | PRKAR2A | PRKAR2A Entrez,  Source | protein kinase, cAMP-dependent, regulatory, type II, alpha | 11520 | 0.007 | -0.4166 | No |
| 10 | HRAS | HRAS Entrez,  Source | v-Ha-ras Harvey rat sarcoma viral oncogene homolog | 12019 | 0.001 | -0.4417 | No |
| 11 | PIK3R1 | PIK3R1 Entrez,  Source | phosphoinositide-3-kinase, regulatory subunit 1 (p85 alpha) | 13154 | -0.014 | -0.4951 | No |
| 12 | MAP2K1 | MAP2K1 Entrez,  Source | mitogen-activated protein kinase kinase 1 | 13246 | -0.015 | -0.4946 | No |
| 13 | AKT1 | AKT1 Entrez,  Source | v-akt murine thymoma viral oncogene homolog 1 | 13261 | -0.015 | -0.4901 | No |
| 14 | GRB2 | GRB2 Entrez,  Source | growth factor receptor-bound protein 2 | 13999 | -0.026 | -0.5189 | No |
| 15 | RPS6KA1 | RPS6KA1 Entrez,  Source | ribosomal protein S6 kinase, 90kDa, polypeptide 1 | 15304 | -0.050 | -0.5684 | No |
| 16 | PRKACB | PRKACB Entrez,  Source | protein kinase, cAMP-dependent, catalytic, beta | 15881 | -0.063 | -0.5763 | No |
| 17 | IGF1R | IGF1R Entrez,  Source | insulin-like growth factor 1 receptor | 18204 | -0.158 | -0.6408 | Yes |
| 18 | BAD | BAD Entrez,  Source | BCL2-antagonist of cell death | 18306 | -0.166 | -0.5890 | Yes |
| 19 | SHC1 | SHC1 Entrez,  Source | SHC (Src homology 2 domain containing) transforming protein 1 | 18393 | -0.173 | -0.5341 | Yes |
| 20 | PRKAR1A | PRKAR1A Entrez,  Source | protein kinase, cAMP-dependent, regulatory, type I, alpha (tissue specific extinguisher 1) | 18683 | -0.206 | -0.4784 | Yes |
| 21 | PRKAR2B | PRKAR2B Entrez,  Source | protein kinase, cAMP-dependent, regulatory, type II, beta | 19490 | -0.601 | -0.3133 | Yes |
| 22 | IRS1 | IRS1 Entrez,  Source | insulin receptor substrate 1 | 19548 | -0.923 | 0.0004 | Yes |
Table: GSEA details [plain text format]

  

Fig 2: BIOCARTA\_IGF1R\_PATHWAY      
 Blue-Pink O' Gram in the Space of the Analyzed GeneSet

  

Fig 3: BIOCARTA\_IGF1R\_PATHWAY: Random ES distribution      
 Gene set null distribution of ES for **BIOCARTA\_IGF1R\_PATHWAY**
