# Supplementary material for: Comprehensive analysis of β-catenin target genes in colorectal carcinoma cell lines with deregulated Wnt/β-catenin signaling
Source: BMC Genomics. 2014 Jan 28;15:74. doi: 10.1186/1471-2164-15-74 (PMC3909937; doi:10.1186/1471-2164-15-74)
Supplement: Additional file 4 — GSEA analysis using the Biocarta pathway database. This zipped file contains confirming data of the GSEA analysis. The names of the directories containing the files were composed of the term ‘GSEA’, the name of the cell line, e.g. DLD1, SW480, or LS174T, and the pathway database (Biocarta). Please use a web browser to view the files with the name ‘index.html’ in the corresponding directories to start exploring the data. [file 1471-2164-15-74-S4.zip › SW480_Biocarta/BIOCARTA_IL12_PATHWAY.html]

Details for gene set BIOCARTA\_IL12\_PATHWAY[GSEA]

|  || Dataset | SW480\_collapsed\_to\_symbols.class.cls#bg\_versus\_b |
| Phenotype | class.cls#bg\_versus\_b |
| Upregulated in class | bg |
| GeneSet | BIOCARTA\_IL12\_PATHWAY |
| Enrichment Score (ES) | 0.4944915 |
| Normalized Enrichment Score (NES) | 1.4106946 |
| Nominal p-value | 0.067857146 |
| FDR q-value | 0.4916548 |
| FWER p-Value | 0.997 |
Table: GSEA Results Summary

  

Fig 1: Enrichment plot: BIOCARTA\_IL12\_PATHWAY      
 Profile of the Running ES Score & Positions of GeneSet Members on the Rank Ordered List

  

| PROBE | GENE SYMBOL | GENE\_TITLE | RANK IN GENE LIST | RANK METRIC SCORE | RUNNING ES | CORE ENRICHMENT || 1 | TRA@ | TRA@ Entrez,  Source | T cell receptor alpha locus | 17 | 0.784 | 0.3124 | Yes |
| 2 | IL12RB2 | IL12RB2 Entrez,  Source | interleukin 12 receptor, beta 2 | 245 | 0.330 | 0.4327 | Yes |
| 3 | IL12A | IL12A Entrez,  Source | interleukin 12A (natural killer cell stimulatory factor 1, cytotoxic lymphocyte maturation factor 1, p35) | 1342 | 0.177 | 0.4472 | Yes |
| 4 | IL18R1 | IL18R1 Entrez,  Source | interleukin 18 receptor 1 | 1680 | 0.162 | 0.4945 | Yes |
| 5 | IL12RB1 | IL12RB1 Entrez,  Source | interleukin 12 receptor, beta 1 | 2893 | 0.125 | 0.4822 | No |
| 6 | MAPK14 | MAPK14 Entrez,  Source | mitogen-activated protein kinase 14 | 4293 | 0.098 | 0.4499 | No |
| 7 | CD3E | CD3E Entrez,  Source | CD3e molecule, epsilon (CD3-TCR complex) | 4333 | 0.098 | 0.4869 | No |
| 8 | ETV5 | ETV5 Entrez,  Source | ets variant gene 5 (ets-related molecule) | 5547 | 0.080 | 0.4566 | No |
| 9 | CD3G | CD3G Entrez,  Source | CD3g molecule, gamma (CD3-TCR complex) | 7931 | 0.050 | 0.3545 | No |
| 10 | CD3D | CD3D Entrez,  Source | CD3d molecule, delta (CD3-TCR complex) | 8284 | 0.045 | 0.3546 | No |
| 11 | STAT4 | STAT4 Entrez,  Source | signal transducer and activator of transcription 4 | 8655 | 0.041 | 0.3520 | No |
| 12 | CXCR3 | CXCR3 Entrez,  Source | chemokine (C-X-C motif) receptor 3 | 9026 | 0.036 | 0.3475 | No |
| 13 | CD247 | CD247 Entrez,  Source | CD247 molecule | 10783 | 0.016 | 0.2639 | No |
| 14 | IL12B | IL12B Entrez,  Source | interleukin 12B (natural killer cell stimulatory factor 2, cytotoxic lymphocyte maturation factor 2, p40) | 11809 | 0.004 | 0.2130 | No |
| 15 | IFNG | IFNG Entrez,  Source | interferon, gamma | 12592 | -0.006 | 0.1755 | No |
| 16 | IL18 | IL18 Entrez,  Source | interleukin 18 (interferon-gamma-inducing factor) | 14771 | -0.040 | 0.0798 | No |
| 17 | MAPK8 | MAPK8 Entrez,  Source | mitogen-activated protein kinase 8 | 15249 | -0.049 | 0.0749 | No |
| 18 | MAP2K6 | MAP2K6 Entrez,  Source | mitogen-activated protein kinase kinase 6 | 15852 | -0.062 | 0.0690 | No |
| 19 | TYK2 | TYK2 Entrez,  Source | tyrosine kinase 2 | 16326 | -0.075 | 0.0748 | No |
| 20 | JAK2 | JAK2 Entrez,  Source | Janus kinase 2 (a protein tyrosine kinase) | 16907 | -0.094 | 0.0826 | No |
| 21 | JUN | JUN Entrez,  Source | jun oncogene | 17831 | -0.133 | 0.0883 | No |
Table: GSEA details [plain text format]

  

Fig 2: BIOCARTA\_IL12\_PATHWAY      
 Blue-Pink O' Gram in the Space of the Analyzed GeneSet

  

Fig 3: BIOCARTA\_IL12\_PATHWAY: Random ES distribution      
 Gene set null distribution of ES for **BIOCARTA\_IL12\_PATHWAY**
