# Supplementary material for: Comprehensive analysis of β-catenin target genes in colorectal carcinoma cell lines with deregulated Wnt/β-catenin signaling
Source: BMC Genomics. 2014 Jan 28;15:74. doi: 10.1186/1471-2164-15-74 (PMC3909937; doi:10.1186/1471-2164-15-74)
Supplement: Additional file 4 — GSEA analysis using the Biocarta pathway database. This zipped file contains confirming data of the GSEA analysis. The names of the directories containing the files were composed of the term ‘GSEA’, the name of the cell line, e.g. DLD1, SW480, or LS174T, and the pathway database (Biocarta). Please use a web browser to view the files with the name ‘index.html’ in the corresponding directories to start exploring the data. [file 1471-2164-15-74-S4.zip › SW480_Biocarta/BIOCARTA_IL2_PATHWAY.html]

Details for gene set BIOCARTA\_IL2\_PATHWAY[GSEA]

|  || Dataset | SW480\_collapsed\_to\_symbols.class.cls#bg\_versus\_b |
| Phenotype | class.cls#bg\_versus\_b |
| Upregulated in class | bg |
| GeneSet | BIOCARTA\_IL2\_PATHWAY |
| Enrichment Score (ES) | 0.39504015 |
| Normalized Enrichment Score (NES) | 1.1159006 |
| Nominal p-value | 0.3 |
| FDR q-value | 0.77335167 |
| FWER p-Value | 1.0 |
Table: GSEA Results Summary

  

Fig 1: Enrichment plot: BIOCARTA\_IL2\_PATHWAY      
 Profile of the Running ES Score & Positions of GeneSet Members on the Rank Ordered List

  

| PROBE | GENE SYMBOL | GENE\_TITLE | RANK IN GENE LIST | RANK METRIC SCORE | RUNNING ES | CORE ENRICHMENT || 1 | SYK | SYK Entrez,  Source | spleen tyrosine kinase | 61 | 0.559 | 0.2323 | Yes |
| 2 | IL2RG | IL2RG Entrez,  Source | interleukin 2 receptor, gamma (severe combined immunodeficiency) | 504 | 0.252 | 0.3159 | Yes |
| 3 | LCK | LCK Entrez,  Source | lymphocyte-specific protein tyrosine kinase | 750 | 0.218 | 0.3950 | Yes |
| 4 | IL2RA | IL2RA Entrez,  Source | interleukin 2 receptor, alpha | 2099 | 0.147 | 0.3879 | No |
| 5 | STAT5B | STAT5B Entrez,  Source | signal transducer and activator of transcription 5B | 3544 | 0.111 | 0.3610 | No |
| 6 | IL2 | IL2 Entrez,  Source | interleukin 2 | 5108 | 0.085 | 0.3169 | No |
| 7 | JAK3 | JAK3 Entrez,  Source | Janus kinase 3 (a protein tyrosine kinase, leukocyte) | 5694 | 0.078 | 0.3198 | No |
| 8 | CSNK2A1 | CSNK2A1 Entrez,  Source | casein kinase 2, alpha 1 polypeptide | 7178 | 0.059 | 0.2687 | No |
| 9 | SOS1 | SOS1 Entrez,  Source | son of sevenless homolog 1 (Drosophila) | 7883 | 0.050 | 0.2538 | No |
| 10 | MAPK3 | MAPK3 Entrez,  Source | mitogen-activated protein kinase 3 | 8976 | 0.037 | 0.2133 | No |
| 11 | STAT5A | STAT5A Entrez,  Source | signal transducer and activator of transcription 5A | 9383 | 0.032 | 0.2060 | No |
| 12 | ELK1 | ELK1 Entrez,  Source | ELK1, member of ETS oncogene family | 9503 | 0.031 | 0.2128 | No |
| 13 | RAF1 | RAF1 Entrez,  Source | v-raf-1 murine leukemia viral oncogene homolog 1 | 10520 | 0.019 | 0.1689 | No |
| 14 | IL2RB | IL2RB Entrez,  Source | interleukin 2 receptor, beta | 11390 | 0.009 | 0.1281 | No |
| 15 | HRAS | HRAS Entrez,  Source | v-Ha-ras Harvey rat sarcoma viral oncogene homolog | 12019 | 0.001 | 0.0964 | No |
| 16 | MAP2K1 | MAP2K1 Entrez,  Source | mitogen-activated protein kinase kinase 1 | 13246 | -0.015 | 0.0399 | No |
| 17 | GRB2 | GRB2 Entrez,  Source | growth factor receptor-bound protein 2 | 13999 | -0.026 | 0.0125 | No |
| 18 | MAPK8 | MAPK8 Entrez,  Source | mitogen-activated protein kinase 8 | 15249 | -0.049 | -0.0309 | No |
| 19 | JAK1 | JAK1 Entrez,  Source | Janus kinase 1 (a protein tyrosine kinase) | 16432 | -0.079 | -0.0583 | No |
| 20 | JUN | JUN Entrez,  Source | jun oncogene | 17831 | -0.133 | -0.0741 | No |
| 21 | SHC1 | SHC1 Entrez,  Source | SHC (Src homology 2 domain containing) transforming protein 1 | 18393 | -0.173 | -0.0300 | No |
| 22 | FOS | FOS Entrez,  Source | v-fos FBJ murine osteosarcoma viral oncogene homolog | 18736 | -0.212 | 0.0420 | No |
Table: GSEA details [plain text format]

  

Fig 2: BIOCARTA\_IL2\_PATHWAY      
 Blue-Pink O' Gram in the Space of the Analyzed GeneSet

  

Fig 3: BIOCARTA\_IL2\_PATHWAY: Random ES distribution      
 Gene set null distribution of ES for **BIOCARTA\_IL2\_PATHWAY**
