# Supplementary material for: Comprehensive analysis of β-catenin target genes in colorectal carcinoma cell lines with deregulated Wnt/β-catenin signaling
Source: BMC Genomics. 2014 Jan 28;15:74. doi: 10.1186/1471-2164-15-74 (PMC3909937; doi:10.1186/1471-2164-15-74)
Supplement: Additional file 4 — GSEA analysis using the Biocarta pathway database. This zipped file contains confirming data of the GSEA analysis. The names of the directories containing the files were composed of the term ‘GSEA’, the name of the cell line, e.g. DLD1, SW480, or LS174T, and the pathway database (Biocarta). Please use a web browser to view the files with the name ‘index.html’ in the corresponding directories to start exploring the data. [file 1471-2164-15-74-S4.zip › SW480_Biocarta/BIOCARTA_INSULIN_PATHWAY.html]

Details for gene set BIOCARTA\_INSULIN\_PATHWAY[GSEA]

|  || Dataset | SW480\_collapsed\_to\_symbols.class.cls#bg\_versus\_b |
| Phenotype | class.cls#bg\_versus\_b |
| Upregulated in class | b |
| GeneSet | BIOCARTA\_INSULIN\_PATHWAY |
| Enrichment Score (ES) | -0.64309955 |
| Normalized Enrichment Score (NES) | -1.954924 |
| Nominal p-value | 0.0022172949 |
| FDR q-value | 0.0622831 |
| FWER p-Value | 0.067 |
Table: GSEA Results Summary

  

Fig 1: Enrichment plot: BIOCARTA\_INSULIN\_PATHWAY      
 Profile of the Running ES Score & Positions of GeneSet Members on the Rank Ordered List

  

| PROBE | GENE SYMBOL | GENE\_TITLE | RANK IN GENE LIST | RANK METRIC SCORE | RUNNING ES | CORE ENRICHMENT || 1 | SRF | SRF Entrez,  Source | serum response factor (c-fos serum response element-binding transcription factor) | 7086 | 0.060 | -0.3323 | No |
| 2 | CSNK2A1 | CSNK2A1 Entrez,  Source | casein kinase 2, alpha 1 polypeptide | 7178 | 0.059 | -0.3072 | No |
| 3 | SOS1 | SOS1 Entrez,  Source | son of sevenless homolog 1 (Drosophila) | 7883 | 0.050 | -0.3177 | No |
| 4 | PIK3CA | PIK3CA Entrez,  Source | phosphoinositide-3-kinase, catalytic, alpha polypeptide | 7968 | 0.049 | -0.2970 | No |
| 5 | PIK3CG | PIK3CG Entrez,  Source | phosphoinositide-3-kinase, catalytic, gamma polypeptide | 8761 | 0.039 | -0.3176 | No |
| 6 | MAPK3 | MAPK3 Entrez,  Source | mitogen-activated protein kinase 3 | 8976 | 0.037 | -0.3099 | No |
| 7 | INS | INS Entrez,  Source | insulin | 9166 | 0.034 | -0.3020 | No |
| 8 | ELK1 | ELK1 Entrez,  Source | ELK1, member of ETS oncogene family | 9503 | 0.031 | -0.3037 | No |
| 9 | PTPN11 | PTPN11 Entrez,  Source | protein tyrosine phosphatase, non-receptor type 11 (Noonan syndrome 1) | 10510 | 0.019 | -0.3455 | No |
| 10 | SLC2A4 | SLC2A4 Entrez,  Source | solute carrier family 2 (facilitated glucose transporter), member 4 | 10513 | 0.019 | -0.3358 | No |
| 11 | RAF1 | RAF1 Entrez,  Source | v-raf-1 murine leukemia viral oncogene homolog 1 | 10520 | 0.019 | -0.3264 | No |
| 12 | HRAS | HRAS Entrez,  Source | v-Ha-ras Harvey rat sarcoma viral oncogene homolog | 12019 | 0.001 | -0.4025 | No |
| 13 | RASA1 | RASA1 Entrez,  Source | RAS p21 protein activator (GTPase activating protein) 1 | 12270 | -0.002 | -0.4144 | No |
| 14 | INSR | INSR Entrez,  Source | insulin receptor | 12565 | -0.006 | -0.4264 | No |
| 15 | PIK3R1 | PIK3R1 Entrez,  Source | phosphoinositide-3-kinase, regulatory subunit 1 (p85 alpha) | 13154 | -0.014 | -0.4496 | No |
| 16 | MAP2K1 | MAP2K1 Entrez,  Source | mitogen-activated protein kinase kinase 1 | 13246 | -0.015 | -0.4466 | No |
| 17 | GRB2 | GRB2 Entrez,  Source | growth factor receptor-bound protein 2 | 13999 | -0.026 | -0.4718 | No |
| 18 | MAPK8 | MAPK8 Entrez,  Source | mitogen-activated protein kinase 8 | 15249 | -0.049 | -0.5110 | No |
| 19 | JUN | JUN Entrez,  Source | jun oncogene | 17831 | -0.133 | -0.5758 | Yes |
| 20 | SHC1 | SHC1 Entrez,  Source | SHC (Src homology 2 domain containing) transforming protein 1 | 18393 | -0.173 | -0.5168 | Yes |
| 21 | FOS | FOS Entrez,  Source | v-fos FBJ murine osteosarcoma viral oncogene homolog | 18736 | -0.212 | -0.4265 | Yes |
| 22 | IRS1 | IRS1 Entrez,  Source | insulin receptor substrate 1 | 19548 | -0.923 | 0.0004 | Yes |
Table: GSEA details [plain text format]

  

Fig 2: BIOCARTA\_INSULIN\_PATHWAY      
 Blue-Pink O' Gram in the Space of the Analyzed GeneSet

  

Fig 3: BIOCARTA\_INSULIN\_PATHWAY: Random ES distribution      
 Gene set null distribution of ES for **BIOCARTA\_INSULIN\_PATHWAY**
