# Supplementary material for: Comprehensive analysis of β-catenin target genes in colorectal carcinoma cell lines with deregulated Wnt/β-catenin signaling
Source: BMC Genomics. 2014 Jan 28;15:74. doi: 10.1186/1471-2164-15-74 (PMC3909937; doi:10.1186/1471-2164-15-74)
Supplement: Additional file 4 — GSEA analysis using the Biocarta pathway database. This zipped file contains confirming data of the GSEA analysis. The names of the directories containing the files were composed of the term ‘GSEA’, the name of the cell line, e.g. DLD1, SW480, or LS174T, and the pathway database (Biocarta). Please use a web browser to view the files with the name ‘index.html’ in the corresponding directories to start exploring the data. [file 1471-2164-15-74-S4.zip › SW480_Biocarta/BIOCARTA_MEF2D_PATHWAY.html]

Details for gene set BIOCARTA\_MEF2D\_PATHWAY[GSEA]

|  || Dataset | SW480\_collapsed\_to\_symbols.class.cls#bg\_versus\_b |
| Phenotype | class.cls#bg\_versus\_b |
| Upregulated in class | bg |
| GeneSet | BIOCARTA\_MEF2D\_PATHWAY |
| Enrichment Score (ES) | 0.36174396 |
| Normalized Enrichment Score (NES) | 0.9808055 |
| Nominal p-value | 0.4923077 |
| FDR q-value | 0.9191355 |
| FWER p-Value | 1.0 |
Table: GSEA Results Summary

  

Fig 1: Enrichment plot: BIOCARTA\_MEF2D\_PATHWAY      
 Profile of the Running ES Score & Positions of GeneSet Members on the Rank Ordered List

  

| PROBE | GENE SYMBOL | GENE\_TITLE | RANK IN GENE LIST | RANK METRIC SCORE | RUNNING ES | CORE ENRICHMENT || 1 | TRA@ | TRA@ Entrez,  Source | T cell receptor alpha locus | 17 | 0.784 | 0.3617 | Yes |
| 2 | CABIN1 | CABIN1 Entrez,  Source | - | 9287 | 0.033 | -0.0973 | No |
| 3 | HDAC2 | HDAC2 Entrez,  Source | histone deacetylase 2 | 9687 | 0.028 | -0.1046 | No |
| 4 | NFATC1 | NFATC1 Entrez,  Source | nuclear factor of activated T-cells, cytoplasmic, calcineurin-dependent 1 | 13196 | -0.014 | -0.2775 | No |
| 5 | PPP3CB | PPP3CB Entrez,  Source | protein phosphatase 3 (formerly 2B), catalytic subunit, beta isoform (calcineurin A beta) | 14346 | -0.032 | -0.3217 | No |
| 6 | CAPNS1 | CAPNS1 Entrez,  Source | calpain, small subunit 1 | 14818 | -0.040 | -0.3271 | No |
| 7 | NFATC2 | NFATC2 Entrez,  Source | nuclear factor of activated T-cells, cytoplasmic, calcineurin-dependent 2 | 14852 | -0.041 | -0.3098 | No |
| 8 | EP300 | EP300 Entrez,  Source | E1A binding protein p300 | 14893 | -0.042 | -0.2927 | No |
| 9 | HDAC1 | HDAC1 Entrez,  Source | histone deacetylase 1 | 14971 | -0.043 | -0.2766 | No |
| 10 | CALM2 | CALM2 Entrez,  Source | calmodulin 2 (phosphorylase kinase, delta) | 15530 | -0.055 | -0.2797 | No |
| 11 | CALM1 | CALM1 Entrez,  Source | calmodulin 1 (phosphorylase kinase, delta) | 15850 | -0.062 | -0.2673 | No |
| 12 | MEF2D | MEF2D Entrez,  Source | MADS box transcription enhancer factor 2, polypeptide D (myocyte enhancer factor 2D) | 16322 | -0.075 | -0.2567 | No |
| 13 | CAPNS2 | CAPNS2 Entrez,  Source | calpain, small subunit 2 | 16999 | -0.098 | -0.2462 | No |
| 14 | CALM3 | CALM3 Entrez,  Source | calmodulin 3 (phosphorylase kinase, delta) | 17323 | -0.110 | -0.2118 | No |
| 15 | PPP3CC | PPP3CC Entrez,  Source | protein phosphatase 3 (formerly 2B), catalytic subunit, gamma isoform (calcineurin A gamma) | 17578 | -0.122 | -0.1686 | No |
| 16 | PRKCA | PRKCA Entrez,  Source | protein kinase C, alpha | 17649 | -0.125 | -0.1142 | No |
| 17 | CAPN2 | CAPN2 Entrez,  Source | calpain 2, (m/II) large subunit | 18143 | -0.154 | -0.0684 | No |
| 18 | PPP3CA | PPP3CA Entrez,  Source | protein phosphatase 3 (formerly 2B), catalytic subunit, alpha isoform (calcineurin A alpha) | 19160 | -0.304 | 0.0203 | No |
Table: GSEA details [plain text format]

  

Fig 2: BIOCARTA\_MEF2D\_PATHWAY      
 Blue-Pink O' Gram in the Space of the Analyzed GeneSet

  

Fig 3: BIOCARTA\_MEF2D\_PATHWAY: Random ES distribution      
 Gene set null distribution of ES for **BIOCARTA\_MEF2D\_PATHWAY**
