# Supplementary material for: Comprehensive analysis of β-catenin target genes in colorectal carcinoma cell lines with deregulated Wnt/β-catenin signaling
Source: BMC Genomics. 2014 Jan 28;15:74. doi: 10.1186/1471-2164-15-74 (PMC3909937; doi:10.1186/1471-2164-15-74)
Supplement: Additional file 4 — GSEA analysis using the Biocarta pathway database. This zipped file contains confirming data of the GSEA analysis. The names of the directories containing the files were composed of the term ‘GSEA’, the name of the cell line, e.g. DLD1, SW480, or LS174T, and the pathway database (Biocarta). Please use a web browser to view the files with the name ‘index.html’ in the corresponding directories to start exploring the data. [file 1471-2164-15-74-S4.zip › SW480_Biocarta/BIOCARTA_NKCELLS_PATHWAY.html]

Details for gene set BIOCARTA\_NKCELLS\_PATHWAY[GSEA]

|  || Dataset | SW480\_collapsed\_to\_symbols.class.cls#bg\_versus\_b |
| Phenotype | class.cls#bg\_versus\_b |
| Upregulated in class | bg |
| GeneSet | BIOCARTA\_NKCELLS\_PATHWAY |
| Enrichment Score (ES) | 0.35675517 |
| Normalized Enrichment Score (NES) | 0.93965596 |
| Nominal p-value | 0.54833335 |
| FDR q-value | 0.92227983 |
| FWER p-Value | 1.0 |
Table: GSEA Results Summary

  

Fig 1: Enrichment plot: BIOCARTA\_NKCELLS\_PATHWAY      
 Profile of the Running ES Score & Positions of GeneSet Members on the Rank Ordered List

  

| PROBE | GENE SYMBOL | GENE\_TITLE | RANK IN GENE LIST | RANK METRIC SCORE | RUNNING ES | CORE ENRICHMENT || 1 | SYK | SYK Entrez,  Source | spleen tyrosine kinase | 61 | 0.559 | 0.3568 | Yes |
| 2 | KLRD1 | KLRD1 Entrez,  Source | killer cell lectin-like receptor subfamily D, member 1 | 2853 | 0.125 | 0.2947 | No |
| 3 | KLRC4 | KLRC4 Entrez,  Source | killer cell lectin-like receptor subfamily C, member 4 | 5903 | 0.075 | 0.1869 | No |
| 4 | PAK1 | PAK1 Entrez,  Source | p21/Cdc42/Rac1-activated kinase 1 (STE20 homolog, yeast) | 6204 | 0.071 | 0.2174 | No |
| 5 | VAV1 | VAV1 Entrez,  Source | vav 1 oncogene | 6712 | 0.065 | 0.2332 | No |
| 6 | PIK3CA | PIK3CA Entrez,  Source | phosphoinositide-3-kinase, catalytic, alpha polypeptide | 7968 | 0.049 | 0.2007 | No |
| 7 | PTPN6 | PTPN6 Entrez,  Source | protein tyrosine phosphatase, non-receptor type 6 | 8950 | 0.037 | 0.1744 | No |
| 8 | MAPK3 | MAPK3 Entrez,  Source | mitogen-activated protein kinase 3 | 8976 | 0.037 | 0.1967 | No |
| 9 | RAC1 | RAC1 Entrez,  Source | ras-related C3 botulinum toxin substrate 1 (rho family, small GTP binding protein Rac1) | 10150 | 0.023 | 0.1516 | No |
| 10 | KLRC3 | KLRC3 Entrez,  Source | killer cell lectin-like receptor subfamily C, member 3 | 11782 | 0.004 | 0.0709 | No |
| 11 | PIK3R1 | PIK3R1 Entrez,  Source | phosphoinositide-3-kinase, regulatory subunit 1 (p85 alpha) | 13154 | -0.014 | 0.0095 | No |
| 12 | MAP2K1 | MAP2K1 Entrez,  Source | mitogen-activated protein kinase kinase 1 | 13246 | -0.015 | 0.0144 | No |
| 13 | IL18 | IL18 Entrez,  Source | interleukin 18 (interferon-gamma-inducing factor) | 14771 | -0.040 | -0.0381 | No |
| 14 | B2M | B2M Entrez,  Source | beta-2-microglobulin | 15904 | -0.064 | -0.0550 | No |
| 15 | PTK2B | PTK2B Entrez,  Source | PTK2B protein tyrosine kinase 2 beta | 17068 | -0.100 | -0.0499 | No |
| 16 | ITGB1 | ITGB1 Entrez,  Source | integrin, beta 1 (fibronectin receptor, beta polypeptide, antigen CD29 includes MDF2, MSK12) | 17235 | -0.107 | 0.0103 | No |
| 17 | HLA-A | HLA-A Entrez,  Source | major histocompatibility complex, class I, A | 18338 | -0.168 | 0.0623 | No |
Table: GSEA details [plain text format]

  

Fig 2: BIOCARTA\_NKCELLS\_PATHWAY      
 Blue-Pink O' Gram in the Space of the Analyzed GeneSet

  

Fig 3: BIOCARTA\_NKCELLS\_PATHWAY: Random ES distribution      
 Gene set null distribution of ES for **BIOCARTA\_NKCELLS\_PATHWAY**
