# Supplementary material for: Comprehensive analysis of β-catenin target genes in colorectal carcinoma cell lines with deregulated Wnt/β-catenin signaling
Source: BMC Genomics. 2014 Jan 28;15:74. doi: 10.1186/1471-2164-15-74 (PMC3909937; doi:10.1186/1471-2164-15-74)
Supplement: Additional file 4 — GSEA analysis using the Biocarta pathway database. This zipped file contains confirming data of the GSEA analysis. The names of the directories containing the files were composed of the term ‘GSEA’, the name of the cell line, e.g. DLD1, SW480, or LS174T, and the pathway database (Biocarta). Please use a web browser to view the files with the name ‘index.html’ in the corresponding directories to start exploring the data. [file 1471-2164-15-74-S4.zip › SW480_Biocarta/BIOCARTA_NKT_PATHWAY.html]

Details for gene set BIOCARTA\_NKT\_PATHWAY[GSEA]

|  || Dataset | SW480\_collapsed\_to\_symbols.class.cls#bg\_versus\_b |
| Phenotype | class.cls#bg\_versus\_b |
| Upregulated in class | bg |
| GeneSet | BIOCARTA\_NKT\_PATHWAY |
| Enrichment Score (ES) | 0.47338194 |
| Normalized Enrichment Score (NES) | 1.4300337 |
| Nominal p-value | 0.072758034 |
| FDR q-value | 0.54673463 |
| FWER p-Value | 0.995 |
Table: GSEA Results Summary

  

Fig 1: Enrichment plot: BIOCARTA\_NKT\_PATHWAY      
 Profile of the Running ES Score & Positions of GeneSet Members on the Rank Ordered List

  

| PROBE | GENE SYMBOL | GENE\_TITLE | RANK IN GENE LIST | RANK METRIC SCORE | RUNNING ES | CORE ENRICHMENT || 1 | CXCR4 | CXCR4 Entrez,  Source | chemokine (C-X-C motif) receptor 4 | 133 | 0.428 | 0.1122 | Yes |
| 2 | CCR3 | CCR3 Entrez,  Source | chemokine (C-C motif) receptor 3 | 161 | 0.401 | 0.2224 | Yes |
| 3 | IL12RB2 | IL12RB2 Entrez,  Source | interleukin 12 receptor, beta 2 | 245 | 0.330 | 0.3100 | Yes |
| 4 | CCR7 | CCR7 Entrez,  Source | chemokine (C-C motif) receptor 7 | 322 | 0.298 | 0.3889 | Yes |
| 5 | IL4 | IL4 Entrez,  Source | interleukin 4 | 1152 | 0.187 | 0.3985 | Yes |
| 6 | IL12A | IL12A Entrez,  Source | interleukin 12A (natural killer cell stimulatory factor 1, cytotoxic lymphocyte maturation factor 1, p35) | 1342 | 0.177 | 0.4380 | Yes |
| 7 | IL18R1 | IL18R1 Entrez,  Source | interleukin 18 receptor 1 | 1680 | 0.162 | 0.4657 | Yes |
| 8 | IL5 | IL5 Entrez,  Source | interleukin 5 (colony-stimulating factor, eosinophil) | 2824 | 0.126 | 0.4422 | Yes |
| 9 | IL12RB1 | IL12RB1 Entrez,  Source | interleukin 12 receptor, beta 1 | 2893 | 0.125 | 0.4734 | Yes |
| 10 | CCR4 | CCR4 Entrez,  Source | chemokine (C-C motif) receptor 4 | 4204 | 0.100 | 0.4340 | No |
| 11 | TGFB3 | TGFB3 Entrez,  Source | transforming growth factor, beta 3 | 4332 | 0.098 | 0.4547 | No |
| 12 | CD40LG | CD40LG Entrez,  Source | CD40 ligand (TNF superfamily, member 5, hyper-IgM syndrome) | 4898 | 0.088 | 0.4503 | No |
| 13 | IL2 | IL2 Entrez,  Source | interleukin 2 | 5108 | 0.085 | 0.4634 | No |
| 14 | TGFB1 | TGFB1 Entrez,  Source | transforming growth factor, beta 1 (Camurati-Engelmann disease) | 6120 | 0.072 | 0.4317 | No |
| 15 | CD4 | CD4 Entrez,  Source | CD4 molecule | 6773 | 0.064 | 0.4161 | No |
| 16 | CCR2 | CCR2 Entrez,  Source | chemokine (C-C motif) receptor 2 | 9013 | 0.036 | 0.3116 | No |
| 17 | CXCR3 | CXCR3 Entrez,  Source | chemokine (C-X-C motif) receptor 3 | 9026 | 0.036 | 0.3210 | No |
| 18 | CD28 | CD28 Entrez,  Source | CD28 molecule | 10014 | 0.025 | 0.2773 | No |
| 19 | CCL4 | CCL4 Entrez,  Source | chemokine (C-C motif) ligand 4 | 10471 | 0.020 | 0.2595 | No |
| 20 | IL4R | IL4R Entrez,  Source | interleukin 4 receptor | 10902 | 0.014 | 0.2414 | No |
| 21 | IL12B | IL12B Entrez,  Source | interleukin 12B (natural killer cell stimulatory factor 2, cytotoxic lymphocyte maturation factor 2, p40) | 11809 | 0.004 | 0.1961 | No |
| 22 | IFNG | IFNG Entrez,  Source | interferon, gamma | 12592 | -0.006 | 0.1578 | No |
| 23 | IFNGR1 | IFNGR1 Entrez,  Source | interferon gamma receptor 1 | 13399 | -0.017 | 0.1213 | No |
| 24 | CCR1 | CCR1 Entrez,  Source | chemokine (C-C motif) receptor 1 | 15654 | -0.058 | 0.0220 | No |
| 25 | IFNGR2 | IFNGR2 Entrez,  Source | interferon gamma receptor 2 (interferon gamma transducer 1) | 15682 | -0.058 | 0.0369 | No |
| 26 | TGFB2 | TGFB2 Entrez,  Source | transforming growth factor, beta 2 | 18969 | -0.253 | -0.0611 | No |
| 27 | CSF2 | CSF2 Entrez,  Source | colony stimulating factor 2 (granulocyte-macrophage) | 19229 | -0.328 | 0.0167 | No |
Table: GSEA details [plain text format]

  

Fig 2: BIOCARTA\_NKT\_PATHWAY      
 Blue-Pink O' Gram in the Space of the Analyzed GeneSet

  

Fig 3: BIOCARTA\_NKT\_PATHWAY: Random ES distribution      
 Gene set null distribution of ES for **BIOCARTA\_NKT\_PATHWAY**
