# Supplementary material for: Comprehensive analysis of β-catenin target genes in colorectal carcinoma cell lines with deregulated Wnt/β-catenin signaling
Source: BMC Genomics. 2014 Jan 28;15:74. doi: 10.1186/1471-2164-15-74 (PMC3909937; doi:10.1186/1471-2164-15-74)
Supplement: Additional file 4 — GSEA analysis using the Biocarta pathway database. This zipped file contains confirming data of the GSEA analysis. The names of the directories containing the files were composed of the term ‘GSEA’, the name of the cell line, e.g. DLD1, SW480, or LS174T, and the pathway database (Biocarta). Please use a web browser to view the files with the name ‘index.html’ in the corresponding directories to start exploring the data. [file 1471-2164-15-74-S4.zip › SW480_Biocarta/BIOCARTA_PPARA_PATHWAY.html]

Details for gene set BIOCARTA\_PPARA\_PATHWAY[GSEA]

|  || Dataset | SW480\_collapsed\_to\_symbols.class.cls#bg\_versus\_b |
| Phenotype | class.cls#bg\_versus\_b |
| Upregulated in class | b |
| GeneSet | BIOCARTA\_PPARA\_PATHWAY |
| Enrichment Score (ES) | -0.4955095 |
| Normalized Enrichment Score (NES) | -1.7817858 |
| Nominal p-value | 0.0 |
| FDR q-value | 0.13319084 |
| FWER p-Value | 0.305 |
Table: GSEA Results Summary

  

Fig 1: Enrichment plot: BIOCARTA\_PPARA\_PATHWAY      
 Profile of the Running ES Score & Positions of GeneSet Members on the Rank Ordered List

  

| PROBE | GENE SYMBOL | GENE\_TITLE | RANK IN GENE LIST | RANK METRIC SCORE | RUNNING ES | CORE ENRICHMENT || 1 | MYC | MYC Entrez,  Source | v-myc myelocytomatosis viral oncogene homolog (avian) | 447 | 0.262 | 0.0188 | No |
| 2 | PPARGC1A | PPARGC1A Entrez,  Source | peroxisome proliferative activated receptor, gamma, coactivator 1, alpha | 617 | 0.235 | 0.0476 | No |
| 3 | PRKACG | PRKACG Entrez,  Source | protein kinase, cAMP-dependent, catalytic, gamma | 1307 | 0.178 | 0.0407 | No |
| 4 | CD36 | CD36 Entrez,  Source | CD36 molecule (thrombospondin receptor) | 1810 | 0.156 | 0.0399 | No |
| 5 | TNF | TNF Entrez,  Source | tumor necrosis factor (TNF superfamily, member 2) | 2207 | 0.143 | 0.0425 | No |
| 6 | FABP1 | FABP1 Entrez,  Source | fatty acid binding protein 1, liver | 2999 | 0.122 | 0.0214 | No |
| 7 | STAT5B | STAT5B Entrez,  Source | signal transducer and activator of transcription 5B | 3544 | 0.111 | 0.0113 | No |
| 8 | NR0B2 | NR0B2 Entrez,  Source | nuclear receptor subfamily 0, group B, member 2 | 6257 | 0.071 | -0.1165 | No |
| 9 | PIK3CA | PIK3CA Entrez,  Source | phosphoinositide-3-kinase, catalytic, alpha polypeptide | 7968 | 0.049 | -0.1963 | No |
| 10 | ME1 | ME1 Entrez,  Source | malic enzyme 1, NADP(+)-dependent, cytosolic | 8326 | 0.045 | -0.2075 | No |
| 11 | PIK3CG | PIK3CG Entrez,  Source | phosphoinositide-3-kinase, catalytic, gamma polypeptide | 8761 | 0.039 | -0.2235 | No |
| 12 | APOA1 | APOA1 Entrez,  Source | apolipoprotein A-I | 8864 | 0.038 | -0.2226 | No |
| 13 | MAPK3 | MAPK3 Entrez,  Source | mitogen-activated protein kinase 3 | 8976 | 0.037 | -0.2224 | No |
| 14 | INS | INS Entrez,  Source | insulin | 9166 | 0.034 | -0.2266 | No |
| 15 | STAT5A | STAT5A Entrez,  Source | signal transducer and activator of transcription 5A | 9383 | 0.032 | -0.2326 | No |
| 16 | MAPK1 | MAPK1 Entrez,  Source | mitogen-activated protein kinase 1 | 9552 | 0.030 | -0.2364 | No |
| 17 | NR2F1 | NR2F1 Entrez,  Source | nuclear receptor subfamily 2, group F, member 1 | 9866 | 0.026 | -0.2482 | No |
| 18 | MRPL11 | MRPL11 Entrez,  Source | mitochondrial ribosomal protein L11 | 11351 | 0.009 | -0.3228 | No |
| 19 | PRKAR2A | PRKAR2A Entrez,  Source | protein kinase, cAMP-dependent, regulatory, type II, alpha | 11520 | 0.007 | -0.3303 | No |
| 20 | APOA2 | APOA2 Entrez,  Source | apolipoprotein A-II | 11652 | 0.006 | -0.3361 | No |
| 21 | PIK3R1 | PIK3R1 Entrez,  Source | phosphoinositide-3-kinase, regulatory subunit 1 (p85 alpha) | 13154 | -0.014 | -0.4108 | No |
| 22 | SP1 | SP1 Entrez,  Source | Sp1 transcription factor | 13810 | -0.023 | -0.4407 | No |
| 23 | HSP90AA1 | HSP90AA1 Entrez,  Source | heat shock protein 90kDa alpha (cytosolic), class A member 1 | 13951 | -0.025 | -0.4438 | No |
| 24 | RELA | RELA Entrez,  Source | v-rel reticuloendotheliosis viral oncogene homolog A, nuclear factor of kappa light polypeptide gene enhancer in B-cells 3, p65 (avian) | 14051 | -0.027 | -0.4446 | No |
| 25 | NCOR1 | NCOR1 Entrez,  Source | nuclear receptor co-repressor 1 | 14501 | -0.034 | -0.4621 | No |
| 26 | NCOR2 | NCOR2 Entrez,  Source | nuclear receptor co-repressor 2 | 14698 | -0.038 | -0.4661 | No |
| 27 | EP300 | EP300 Entrez,  Source | E1A binding protein p300 | 14893 | -0.042 | -0.4694 | No |
| 28 | DUT | DUT Entrez,  Source | dUTP pyrophosphatase | 15063 | -0.045 | -0.4709 | No |
| 29 | DUSP1 | DUSP1 Entrez,  Source | dual specificity phosphatase 1 | 15222 | -0.048 | -0.4713 | No |
| 30 | CREBBP | CREBBP Entrez,  Source | CREB binding protein (Rubinstein-Taybi syndrome) | 15257 | -0.049 | -0.4652 | No |
| 31 | SRA1 | SRA1 Entrez,  Source | steroid receptor RNA activator 1 | 15411 | -0.052 | -0.4647 | No |
| 32 | ACOX1 | ACOX1 Entrez,  Source | acyl-Coenzyme A oxidase 1, palmitoyl | 15434 | -0.053 | -0.4574 | No |
| 33 | NCOA1 | NCOA1 Entrez,  Source | nuclear receptor coactivator 1 | 15796 | -0.061 | -0.4662 | No |
| 34 | PRKACB | PRKACB Entrez,  Source | protein kinase, cAMP-dependent, catalytic, beta | 15881 | -0.063 | -0.4604 | No |
| 35 | HSD17B4 | HSD17B4 Entrez,  Source | hydroxysteroid (17-beta) dehydrogenase 4 | 16144 | -0.071 | -0.4626 | No |
| 36 | NFKBIA | NFKBIA Entrez,  Source | nuclear factor of kappa light polypeptide gene enhancer in B-cells inhibitor, alpha | 16196 | -0.072 | -0.4538 | No |
| 37 | PDGFA | PDGFA Entrez,  Source | platelet-derived growth factor alpha polypeptide | 16294 | -0.074 | -0.4469 | No |
| 38 | RXRA | RXRA Entrez,  Source | retinoid X receptor, alpha | 17244 | -0.107 | -0.4784 | Yes |
| 39 | PTGS2 | PTGS2 Entrez,  Source | prostaglandin-endoperoxide synthase 2 (prostaglandin G/H synthase and cyclooxygenase) | 17295 | -0.109 | -0.4636 | Yes |
| 40 | PRKCA | PRKCA Entrez,  Source | protein kinase C, alpha | 17649 | -0.125 | -0.4617 | Yes |
| 41 | CPT1B | CPT1B Entrez,  Source | carnitine palmitoyltransferase 1B (muscle) | 17683 | -0.126 | -0.4433 | Yes |
| 42 | JUN | JUN Entrez,  Source | jun oncogene | 17831 | -0.133 | -0.4297 | Yes |
| 43 | PPARA | PPARA Entrez,  Source | peroxisome proliferative activated receptor, alpha | 18063 | -0.148 | -0.4179 | Yes |
| 44 | RB1 | RB1 Entrez,  Source | retinoblastoma 1 (including osteosarcoma) | 18135 | -0.153 | -0.3972 | Yes |
| 45 | HSPA1A | HSPA1A Entrez,  Source | heat shock 70kDa protein 1A | 18234 | -0.160 | -0.3767 | Yes |
| 46 | PRKAR1A | PRKAR1A Entrez,  Source | protein kinase, cAMP-dependent, regulatory, type I, alpha (tissue specific extinguisher 1) | 18683 | -0.206 | -0.3668 | Yes |
| 47 | EHHADH | EHHADH Entrez,  Source | enoyl-Coenzyme A, hydratase/3-hydroxyacyl Coenzyme A dehydrogenase | 18864 | -0.235 | -0.3386 | Yes |
| 48 | CITED2 | CITED2 Entrez,  Source | Cbp/p300-interacting transactivator, with Glu/Asp-rich carboxy-terminal domain, 2 | 19088 | -0.285 | -0.3045 | Yes |
| 49 | LPL | LPL Entrez,  Source | lipoprotein lipase | 19293 | -0.362 | -0.2572 | Yes |
| 50 | NRIP1 | NRIP1 Entrez,  Source | nuclear receptor interacting protein 1 | 19446 | -0.500 | -0.1853 | Yes |
| 51 | NR1H3 | NR1H3 Entrez,  Source | nuclear receptor subfamily 1, group H, member 3 | 19488 | -0.595 | -0.0925 | Yes |
| 52 | PRKAR2B | PRKAR2B Entrez,  Source | protein kinase, cAMP-dependent, regulatory, type II, beta | 19490 | -0.601 | 0.0034 | Yes |
Table: GSEA details [plain text format]

  

Fig 2: BIOCARTA\_PPARA\_PATHWAY      
 Blue-Pink O' Gram in the Space of the Analyzed GeneSet

  

Fig 3: BIOCARTA\_PPARA\_PATHWAY: Random ES distribution      
 Gene set null distribution of ES for **BIOCARTA\_PPARA\_PATHWAY**
