# Supplementary material for: Comprehensive analysis of β-catenin target genes in colorectal carcinoma cell lines with deregulated Wnt/β-catenin signaling
Source: BMC Genomics. 2014 Jan 28;15:74. doi: 10.1186/1471-2164-15-74 (PMC3909937; doi:10.1186/1471-2164-15-74)
Supplement: Additional file 4 — GSEA analysis using the Biocarta pathway database. This zipped file contains confirming data of the GSEA analysis. The names of the directories containing the files were composed of the term ‘GSEA’, the name of the cell line, e.g. DLD1, SW480, or LS174T, and the pathway database (Biocarta). Please use a web browser to view the files with the name ‘index.html’ in the corresponding directories to start exploring the data. [file 1471-2164-15-74-S4.zip › SW480_Biocarta/BIOCARTA_SPPA_PATHWAY.html]

Details for gene set BIOCARTA\_SPPA\_PATHWAY[GSEA]

|  || Dataset | SW480\_collapsed\_to\_symbols.class.cls#bg\_versus\_b |
| Phenotype | class.cls#bg\_versus\_b |
| Upregulated in class | bg |
| GeneSet | BIOCARTA\_SPPA\_PATHWAY |
| Enrichment Score (ES) | 0.40565348 |
| Normalized Enrichment Score (NES) | 1.1121994 |
| Nominal p-value | 0.3050571 |
| FDR q-value | 0.7270166 |
| FWER p-Value | 1.0 |
Table: GSEA Results Summary

  

Fig 1: Enrichment plot: BIOCARTA\_SPPA\_PATHWAY      
 Profile of the Running ES Score & Positions of GeneSet Members on the Rank Ordered List

  

| PROBE | GENE SYMBOL | GENE\_TITLE | RANK IN GENE LIST | RANK METRIC SCORE | RUNNING ES | CORE ENRICHMENT || 1 | SYK | SYK Entrez,  Source | spleen tyrosine kinase | 61 | 0.559 | 0.2166 | Yes |
| 2 | GNAI1 | GNAI1 Entrez,  Source | guanine nucleotide binding protein (G protein), alpha inhibiting activity polypeptide 1 | 88 | 0.484 | 0.4057 | Yes |
| 3 | PLCB1 | PLCB1 Entrez,  Source | phospholipase C, beta 1 (phosphoinositide-specific) | 2148 | 0.145 | 0.3573 | No |
| 4 | TBXAS1 | TBXAS1 Entrez,  Source | thromboxane A synthase 1 (platelet, cytochrome P450, family 5, subfamily A) | 3177 | 0.119 | 0.3513 | No |
| 5 | PTGS1 | PTGS1 Entrez,  Source | prostaglandin-endoperoxide synthase 1 (prostaglandin G/H synthase and cyclooxygenase) | 3750 | 0.108 | 0.3644 | No |
| 6 | SRC | SRC Entrez,  Source | v-src sarcoma (Schmidt-Ruppin A-2) viral oncogene homolog (avian) | 4017 | 0.103 | 0.3911 | No |
| 7 | F2R | F2R Entrez,  Source | coagulation factor II (thrombin) receptor | 4693 | 0.091 | 0.3925 | No |
| 8 | F2 | F2 Entrez,  Source | coagulation factor II (thrombin) | 5766 | 0.077 | 0.3680 | No |
| 9 | GNGT1 | GNGT1 Entrez,  Source | guanine nucleotide binding protein (G protein), gamma transducing activity polypeptide 1 | 8498 | 0.043 | 0.2451 | No |
| 10 | MAPK3 | MAPK3 Entrez,  Source | mitogen-activated protein kinase 3 | 8976 | 0.037 | 0.2351 | No |
| 11 | MAPK1 | MAPK1 Entrez,  Source | mitogen-activated protein kinase 1 | 9552 | 0.030 | 0.2175 | No |
| 12 | RAF1 | RAF1 Entrez,  Source | v-raf-1 murine leukemia viral oncogene homolog 1 | 10520 | 0.019 | 0.1755 | No |
| 13 | HRAS | HRAS Entrez,  Source | v-Ha-ras Harvey rat sarcoma viral oncogene homolog | 12019 | 0.001 | 0.0993 | No |
| 14 | MAP2K1 | MAP2K1 Entrez,  Source | mitogen-activated protein kinase kinase 1 | 13246 | -0.015 | 0.0424 | No |
| 15 | GNB1 | GNB1 Entrez,  Source | guanine nucleotide binding protein (G protein), beta polypeptide 1 | 14980 | -0.043 | -0.0292 | No |
| 16 | PTK2 | PTK2 Entrez,  Source | PTK2 protein tyrosine kinase 2 | 15243 | -0.049 | -0.0235 | No |
| 17 | ITGB1 | ITGB1 Entrez,  Source | integrin, beta 1 (fibronectin receptor, beta polypeptide, antigen CD29 includes MDF2, MSK12) | 17235 | -0.107 | -0.0834 | No |
| 18 | PRKCA | PRKCA Entrez,  Source | protein kinase C, alpha | 17649 | -0.125 | -0.0553 | No |
| 19 | PLA2G4A | PLA2G4A Entrez,  Source | phospholipase A2, group IVA (cytosolic, calcium-dependent) | 19339 | -0.389 | 0.0111 | No |
Table: GSEA details [plain text format]

  

Fig 2: BIOCARTA\_SPPA\_PATHWAY      
 Blue-Pink O' Gram in the Space of the Analyzed GeneSet

  

Fig 3: BIOCARTA\_SPPA\_PATHWAY: Random ES distribution      
 Gene set null distribution of ES for **BIOCARTA\_SPPA\_PATHWAY**
