# Supplementary material for: Comprehensive analysis of β-catenin target genes in colorectal carcinoma cell lines with deregulated Wnt/β-catenin signaling
Source: BMC Genomics. 2014 Jan 28;15:74. doi: 10.1186/1471-2164-15-74 (PMC3909937; doi:10.1186/1471-2164-15-74)
Supplement: Additional file 4 — GSEA analysis using the Biocarta pathway database. This zipped file contains confirming data of the GSEA analysis. The names of the directories containing the files were composed of the term ‘GSEA’, the name of the cell line, e.g. DLD1, SW480, or LS174T, and the pathway database (Biocarta). Please use a web browser to view the files with the name ‘index.html’ in the corresponding directories to start exploring the data. [file 1471-2164-15-74-S4.zip › SW480_Biocarta/BIOCARTA_STRESS_PATHWAY.html]

Details for gene set BIOCARTA\_STRESS\_PATHWAY[GSEA]

|  || Dataset | SW480\_collapsed\_to\_symbols.class.cls#bg\_versus\_b |
| Phenotype | class.cls#bg\_versus\_b |
| Upregulated in class | b |
| GeneSet | BIOCARTA\_STRESS\_PATHWAY |
| Enrichment Score (ES) | -0.55148846 |
| Normalized Enrichment Score (NES) | -1.6456712 |
| Nominal p-value | 0.025171624 |
| FDR q-value | 0.14445004 |
| FWER p-Value | 0.673 |
Table: GSEA Results Summary

  

Fig 1: Enrichment plot: BIOCARTA\_STRESS\_PATHWAY      
 Profile of the Running ES Score & Positions of GeneSet Members on the Rank Ordered List

  

| PROBE | GENE SYMBOL | GENE\_TITLE | RANK IN GENE LIST | RANK METRIC SCORE | RUNNING ES | CORE ENRICHMENT || 1 | TNF | TNF Entrez,  Source | tumor necrosis factor (TNF superfamily, member 2) | 2207 | 0.143 | -0.0477 | No |
| 2 | MAPK14 | MAPK14 Entrez,  Source | mitogen-activated protein kinase 14 | 4293 | 0.098 | -0.1097 | No |
| 3 | LTA | LTA Entrez,  Source | lymphotoxin alpha (TNF superfamily, member 1) | 4963 | 0.087 | -0.1041 | No |
| 4 | MAP2K4 | MAP2K4 Entrez,  Source | mitogen-activated protein kinase kinase 4 | 6974 | 0.061 | -0.1790 | No |
| 5 | NFKB1 | NFKB1 Entrez,  Source | nuclear factor of kappa light polypeptide gene enhancer in B-cells 1 (p105) | 10617 | 0.018 | -0.3573 | No |
| 6 | CRADD | CRADD Entrez,  Source | CASP2 and RIPK1 domain containing adaptor with death domain | 12606 | -0.007 | -0.4561 | No |
| 7 | TRAF2 | TRAF2 Entrez,  Source | TNF receptor-associated factor 2 | 13715 | -0.022 | -0.5027 | No |
| 8 | RELA | RELA Entrez,  Source | v-rel reticuloendotheliosis viral oncogene homolog A, nuclear factor of kappa light polypeptide gene enhancer in B-cells 3, p65 (avian) | 14051 | -0.027 | -0.5076 | No |
| 9 | TANK | TANK Entrez,  Source | TRAF family member-associated NFKB activator | 14642 | -0.037 | -0.5210 | No |
| 10 | CASP2 | CASP2 Entrez,  Source | caspase 2, apoptosis-related cysteine peptidase (neural precursor cell expressed, developmentally down-regulated 2) | 15239 | -0.049 | -0.5293 | Yes |
| 11 | MAPK8 | MAPK8 Entrez,  Source | mitogen-activated protein kinase 8 | 15249 | -0.049 | -0.5075 | Yes |
| 12 | CHUK | CHUK Entrez,  Source | conserved helix-loop-helix ubiquitous kinase | 15693 | -0.059 | -0.5034 | Yes |
| 13 | MAP2K6 | MAP2K6 Entrez,  Source | mitogen-activated protein kinase kinase 6 | 15852 | -0.062 | -0.4831 | Yes |
| 14 | NFKBIA | NFKBIA Entrez,  Source | nuclear factor of kappa light polypeptide gene enhancer in B-cells inhibitor, alpha | 16196 | -0.072 | -0.4680 | Yes |
| 15 | MAP3K1 | MAP3K1 Entrez,  Source | mitogen-activated protein kinase kinase kinase 1 | 16375 | -0.077 | -0.4421 | Yes |
| 16 | MAP4K2 | MAP4K2 Entrez,  Source | mitogen-activated protein kinase kinase kinase kinase 2 | 16572 | -0.083 | -0.4144 | Yes |
| 17 | ATF1 | ATF1 Entrez,  Source | activating transcription factor 1 | 16755 | -0.089 | -0.3832 | Yes |
| 18 | JUN | JUN Entrez,  Source | jun oncogene | 17831 | -0.133 | -0.3779 | Yes |
| 19 | MAP3K14 | MAP3K14 Entrez,  Source | mitogen-activated protein kinase kinase kinase 14 | 18028 | -0.146 | -0.3216 | Yes |
| 20 | RIPK1 | RIPK1 Entrez,  Source | receptor (TNFRSF)-interacting serine-threonine kinase 1 | 18281 | -0.164 | -0.2597 | Yes |
| 21 | TRADD | TRADD Entrez,  Source | TNFRSF1A-associated via death domain | 18311 | -0.166 | -0.1856 | Yes |
| 22 | TNFRSF1A | TNFRSF1A Entrez,  Source | tumor necrosis factor receptor superfamily, member 1A | 18320 | -0.167 | -0.1100 | Yes |
| 23 | IKBKG | IKBKG Entrez,  Source | inhibitor of kappa light polypeptide gene enhancer in B-cells, kinase gamma | 18387 | -0.172 | -0.0349 | Yes |
| 24 | IKBKB | IKBKB Entrez,  Source | inhibitor of kappa light polypeptide gene enhancer in B-cells, kinase beta | 18696 | -0.208 | 0.0440 | Yes |
Table: GSEA details [plain text format]

  

Fig 2: BIOCARTA\_STRESS\_PATHWAY      
 Blue-Pink O' Gram in the Space of the Analyzed GeneSet

  

Fig 3: BIOCARTA\_STRESS\_PATHWAY: Random ES distribution      
 Gene set null distribution of ES for **BIOCARTA\_STRESS\_PATHWAY**
