# Supplementary material for: Comprehensive analysis of β-catenin target genes in colorectal carcinoma cell lines with deregulated Wnt/β-catenin signaling
Source: BMC Genomics. 2014 Jan 28;15:74. doi: 10.1186/1471-2164-15-74 (PMC3909937; doi:10.1186/1471-2164-15-74)
Supplement: Additional file 4 — GSEA analysis using the Biocarta pathway database. This zipped file contains confirming data of the GSEA analysis. The names of the directories containing the files were composed of the term ‘GSEA’, the name of the cell line, e.g. DLD1, SW480, or LS174T, and the pathway database (Biocarta). Please use a web browser to view the files with the name ‘index.html’ in the corresponding directories to start exploring the data. [file 1471-2164-15-74-S4.zip › SW480_Biocarta/BIOCARTA_TCR_PATHWAY.html]

Details for gene set BIOCARTA\_TCR\_PATHWAY[GSEA]

|  || Dataset | SW480\_collapsed\_to\_symbols.class.cls#bg\_versus\_b |
| Phenotype | class.cls#bg\_versus\_b |
| Upregulated in class | bg |
| GeneSet | BIOCARTA\_TCR\_PATHWAY |
| Enrichment Score (ES) | 0.341727 |
| Normalized Enrichment Score (NES) | 1.1314949 |
| Nominal p-value | 0.26968175 |
| FDR q-value | 0.8627632 |
| FWER p-Value | 1.0 |
Table: GSEA Results Summary

  

Fig 1: Enrichment plot: BIOCARTA\_TCR\_PATHWAY      
 Profile of the Running ES Score & Positions of GeneSet Members on the Rank Ordered List

  

| PROBE | GENE SYMBOL | GENE\_TITLE | RANK IN GENE LIST | RANK METRIC SCORE | RUNNING ES | CORE ENRICHMENT || 1 | TRA@ | TRA@ Entrez,  Source | T cell receptor alpha locus | 17 | 0.784 | 0.1746 | Yes |
| 2 | ZAP70 | ZAP70 Entrez,  Source | zeta-chain (TCR) associated protein kinase 70kDa | 28 | 0.697 | 0.3300 | Yes |
| 3 | LCK | LCK Entrez,  Source | lymphocyte-specific protein tyrosine kinase | 750 | 0.218 | 0.3417 | Yes |
| 4 | PTPN7 | PTPN7 Entrez,  Source | protein tyrosine phosphatase, non-receptor type 7 | 2459 | 0.135 | 0.2845 | No |
| 5 | NFATC4 | NFATC4 Entrez,  Source | nuclear factor of activated T-cells, cytoplasmic, calcineurin-dependent 4 | 4199 | 0.100 | 0.2177 | No |
| 6 | PLCG1 | PLCG1 Entrez,  Source | phospholipase C, gamma 1 | 4276 | 0.098 | 0.2358 | No |
| 7 | CD3E | CD3E Entrez,  Source | CD3e molecule, epsilon (CD3-TCR complex) | 4333 | 0.098 | 0.2548 | No |
| 8 | FYN | FYN Entrez,  Source | FYN oncogene related to SRC, FGR, YES | 4602 | 0.093 | 0.2618 | No |
| 9 | NFATC3 | NFATC3 Entrez,  Source | nuclear factor of activated T-cells, cytoplasmic, calcineurin-dependent 3 | 5704 | 0.078 | 0.2228 | No |
| 10 | VAV1 | VAV1 Entrez,  Source | vav 1 oncogene | 6712 | 0.065 | 0.1857 | No |
| 11 | MAP2K4 | MAP2K4 Entrez,  Source | mitogen-activated protein kinase kinase 4 | 6974 | 0.061 | 0.1861 | No |
| 12 | SOS1 | SOS1 Entrez,  Source | son of sevenless homolog 1 (Drosophila) | 7883 | 0.050 | 0.1508 | No |
| 13 | CD3G | CD3G Entrez,  Source | CD3g molecule, gamma (CD3-TCR complex) | 7931 | 0.050 | 0.1595 | No |
| 14 | PIK3CA | PIK3CA Entrez,  Source | phosphoinositide-3-kinase, catalytic, alpha polypeptide | 7968 | 0.049 | 0.1687 | No |
| 15 | CD3D | CD3D Entrez,  Source | CD3d molecule, delta (CD3-TCR complex) | 8284 | 0.045 | 0.1627 | No |
| 16 | PIK3CG | PIK3CG Entrez,  Source | phosphoinositide-3-kinase, catalytic, gamma polypeptide | 8761 | 0.039 | 0.1471 | No |
| 17 | MAPK3 | MAPK3 Entrez,  Source | mitogen-activated protein kinase 3 | 8976 | 0.037 | 0.1444 | No |
| 18 | ELK1 | ELK1 Entrez,  Source | ELK1, member of ETS oncogene family | 9503 | 0.031 | 0.1242 | No |
| 19 | RAC1 | RAC1 Entrez,  Source | ras-related C3 botulinum toxin substrate 1 (rho family, small GTP binding protein Rac1) | 10150 | 0.023 | 0.0963 | No |
| 20 | RAF1 | RAF1 Entrez,  Source | v-raf-1 murine leukemia viral oncogene homolog 1 | 10520 | 0.019 | 0.0817 | No |
| 21 | NFKB1 | NFKB1 Entrez,  Source | nuclear factor of kappa light polypeptide gene enhancer in B-cells 1 (p105) | 10617 | 0.018 | 0.0808 | No |
| 22 | CD247 | CD247 Entrez,  Source | CD247 molecule | 10783 | 0.016 | 0.0759 | No |
| 23 | HRAS | HRAS Entrez,  Source | v-Ha-ras Harvey rat sarcoma viral oncogene homolog | 12019 | 0.001 | 0.0128 | No |
| 24 | RASA1 | RASA1 Entrez,  Source | RAS p21 protein activator (GTPase activating protein) 1 | 12270 | -0.002 | 0.0004 | No |
| 25 | PIK3R1 | PIK3R1 Entrez,  Source | phosphoinositide-3-kinase, regulatory subunit 1 (p85 alpha) | 13154 | -0.014 | -0.0418 | No |
| 26 | NFATC1 | NFATC1 Entrez,  Source | nuclear factor of activated T-cells, cytoplasmic, calcineurin-dependent 1 | 13196 | -0.014 | -0.0407 | No |
| 27 | MAP2K1 | MAP2K1 Entrez,  Source | mitogen-activated protein kinase kinase 1 | 13246 | -0.015 | -0.0399 | No |
| 28 | GRB2 | GRB2 Entrez,  Source | growth factor receptor-bound protein 2 | 13999 | -0.026 | -0.0725 | No |
| 29 | RELA | RELA Entrez,  Source | v-rel reticuloendotheliosis viral oncogene homolog A, nuclear factor of kappa light polypeptide gene enhancer in B-cells 3, p65 (avian) | 14051 | -0.027 | -0.0691 | No |
| 30 | PPP3CB | PPP3CB Entrez,  Source | protein phosphatase 3 (formerly 2B), catalytic subunit, beta isoform (calcineurin A beta) | 14346 | -0.032 | -0.0771 | No |
| 31 | NFATC2 | NFATC2 Entrez,  Source | nuclear factor of activated T-cells, cytoplasmic, calcineurin-dependent 2 | 14852 | -0.041 | -0.0938 | No |
| 32 | MAPK8 | MAPK8 Entrez,  Source | mitogen-activated protein kinase 8 | 15249 | -0.049 | -0.1031 | No |
| 33 | CALM2 | CALM2 Entrez,  Source | calmodulin 2 (phosphorylase kinase, delta) | 15530 | -0.055 | -0.1052 | No |
| 34 | CALM1 | CALM1 Entrez,  Source | calmodulin 1 (phosphorylase kinase, delta) | 15850 | -0.062 | -0.1076 | No |
| 35 | NFKBIA | NFKBIA Entrez,  Source | nuclear factor of kappa light polypeptide gene enhancer in B-cells inhibitor, alpha | 16196 | -0.072 | -0.1092 | No |
| 36 | MAP3K1 | MAP3K1 Entrez,  Source | mitogen-activated protein kinase kinase kinase 1 | 16375 | -0.077 | -0.1011 | No |
| 37 | CALM3 | CALM3 Entrez,  Source | calmodulin 3 (phosphorylase kinase, delta) | 17323 | -0.110 | -0.1250 | No |
| 38 | PPP3CC | PPP3CC Entrez,  Source | protein phosphatase 3 (formerly 2B), catalytic subunit, gamma isoform (calcineurin A gamma) | 17578 | -0.122 | -0.1108 | No |
| 39 | PRKCA | PRKCA Entrez,  Source | protein kinase C, alpha | 17649 | -0.125 | -0.0864 | No |
| 40 | JUN | JUN Entrez,  Source | jun oncogene | 17831 | -0.133 | -0.0660 | No |
| 41 | SHC1 | SHC1 Entrez,  Source | SHC (Src homology 2 domain containing) transforming protein 1 | 18393 | -0.173 | -0.0561 | No |
| 42 | FOS | FOS Entrez,  Source | v-fos FBJ murine osteosarcoma viral oncogene homolog | 18736 | -0.212 | -0.0261 | No |
| 43 | PPP3CA | PPP3CA Entrez,  Source | protein phosphatase 3 (formerly 2B), catalytic subunit, alpha isoform (calcineurin A alpha) | 19160 | -0.304 | 0.0203 | No |
Table: GSEA details [plain text format]

  

Fig 2: BIOCARTA\_TCR\_PATHWAY      
 Blue-Pink O' Gram in the Space of the Analyzed GeneSet

  

Fig 3: BIOCARTA\_TCR\_PATHWAY: Random ES distribution      
 Gene set null distribution of ES for **BIOCARTA\_TCR\_PATHWAY**
