# Supplementary material for: Comprehensive analysis of β-catenin target genes in colorectal carcinoma cell lines with deregulated Wnt/β-catenin signaling
Source: BMC Genomics. 2014 Jan 28;15:74. doi: 10.1186/1471-2164-15-74 (PMC3909937; doi:10.1186/1471-2164-15-74)
Supplement: Additional file 4 — GSEA analysis using the Biocarta pathway database. This zipped file contains confirming data of the GSEA analysis. The names of the directories containing the files were composed of the term ‘GSEA’, the name of the cell line, e.g. DLD1, SW480, or LS174T, and the pathway database (Biocarta). Please use a web browser to view the files with the name ‘index.html’ in the corresponding directories to start exploring the data. [file 1471-2164-15-74-S4.zip › SW480_Biocarta/BIOCARTA_TH1TH2_PATHWAY.html]

Details for gene set BIOCARTA\_TH1TH2\_PATHWAY[GSEA]

|  || Dataset | SW480\_collapsed\_to\_symbols.class.cls#bg\_versus\_b |
| Phenotype | class.cls#bg\_versus\_b |
| Upregulated in class | bg |
| GeneSet | BIOCARTA\_TH1TH2\_PATHWAY |
| Enrichment Score (ES) | 0.55731416 |
| Normalized Enrichment Score (NES) | 1.5568993 |
| Nominal p-value | 0.020797227 |
| FDR q-value | 0.8775979 |
| FWER p-Value | 0.897 |
Table: GSEA Results Summary

  

Fig 1: Enrichment plot: BIOCARTA\_TH1TH2\_PATHWAY      
 Profile of the Running ES Score & Positions of GeneSet Members on the Rank Ordered List

  

| PROBE | GENE SYMBOL | GENE\_TITLE | RANK IN GENE LIST | RANK METRIC SCORE | RUNNING ES | CORE ENRICHMENT || 1 | IL12RB2 | IL12RB2 Entrez,  Source | interleukin 12 receptor, beta 2 | 245 | 0.330 | 0.1782 | Yes |
| 2 | IL4 | IL4 Entrez,  Source | interleukin 4 | 1152 | 0.187 | 0.2399 | Yes |
| 3 | IL12A | IL12A Entrez,  Source | interleukin 12A (natural killer cell stimulatory factor 1, cytotoxic lymphocyte maturation factor 1, p35) | 1342 | 0.177 | 0.3324 | Yes |
| 4 | IL18R1 | IL18R1 Entrez,  Source | interleukin 18 receptor 1 | 1680 | 0.162 | 0.4084 | Yes |
| 5 | IL2RA | IL2RA Entrez,  Source | interleukin 2 receptor, alpha | 2099 | 0.147 | 0.4719 | Yes |
| 6 | IL12RB1 | IL12RB1 Entrez,  Source | interleukin 12 receptor, beta 1 | 2893 | 0.125 | 0.5032 | Yes |
| 7 | HLA-DRB1 | HLA-DRB1 Entrez,  Source | major histocompatibility complex, class II, DR beta 1 | 3320 | 0.116 | 0.5483 | Yes |
| 8 | CD40LG | CD40LG Entrez,  Source | CD40 ligand (TNF superfamily, member 5, hyper-IgM syndrome) | 4898 | 0.088 | 0.5187 | Yes |
| 9 | IL2 | IL2 Entrez,  Source | interleukin 2 | 5108 | 0.085 | 0.5573 | Yes |
| 10 | CD40 | CD40 Entrez,  Source | CD40 molecule, TNF receptor superfamily member 5 | 6627 | 0.066 | 0.5175 | No |
| 11 | CD28 | CD28 Entrez,  Source | CD28 molecule | 10014 | 0.025 | 0.3585 | No |
| 12 | IL4R | IL4R Entrez,  Source | interleukin 4 receptor | 10902 | 0.014 | 0.3214 | No |
| 13 | IL12B | IL12B Entrez,  Source | interleukin 12B (natural killer cell stimulatory factor 2, cytotoxic lymphocyte maturation factor 2, p40) | 11809 | 0.004 | 0.2772 | No |
| 14 | IFNG | IFNG Entrez,  Source | interferon, gamma | 12592 | -0.006 | 0.2409 | No |
| 15 | CD86 | CD86 Entrez,  Source | CD86 molecule | 13263 | -0.015 | 0.2154 | No |
| 16 | IFNGR1 | IFNGR1 Entrez,  Source | interferon gamma receptor 1 | 13399 | -0.017 | 0.2184 | No |
| 17 | IL18 | IL18 Entrez,  Source | interleukin 18 (interferon-gamma-inducing factor) | 14771 | -0.040 | 0.1711 | No |
| 18 | IFNGR2 | IFNGR2 Entrez,  Source | interferon gamma receptor 2 (interferon gamma transducer 1) | 15682 | -0.058 | 0.1583 | No |
| 19 | HLA-DRA | HLA-DRA Entrez,  Source | major histocompatibility complex, class II, DR alpha | 16100 | -0.069 | 0.1769 | No |
Table: GSEA details [plain text format]

  

Fig 2: BIOCARTA\_TH1TH2\_PATHWAY      
 Blue-Pink O' Gram in the Space of the Analyzed GeneSet

  

Fig 3: BIOCARTA\_TH1TH2\_PATHWAY: Random ES distribution      
 Gene set null distribution of ES for **BIOCARTA\_TH1TH2\_PATHWAY**
