# Supplementary material for: Comprehensive analysis of β-catenin target genes in colorectal carcinoma cell lines with deregulated Wnt/β-catenin signaling
Source: BMC Genomics. 2014 Jan 28;15:74. doi: 10.1186/1471-2164-15-74 (PMC3909937; doi:10.1186/1471-2164-15-74)
Supplement: Additional file 4 — GSEA analysis using the Biocarta pathway database. This zipped file contains confirming data of the GSEA analysis. The names of the directories containing the files were composed of the term ‘GSEA’, the name of the cell line, e.g. DLD1, SW480, or LS174T, and the pathway database (Biocarta). Please use a web browser to view the files with the name ‘index.html’ in the corresponding directories to start exploring the data. [file 1471-2164-15-74-S4.zip › SW480_Biocarta/BIOCARTA_TNFR2_PATHWAY.html]

Details for gene set BIOCARTA\_TNFR2\_PATHWAY[GSEA]

|  || Dataset | SW480\_collapsed\_to\_symbols.class.cls#bg\_versus\_b |
| Phenotype | class.cls#bg\_versus\_b |
| Upregulated in class | b |
| GeneSet | BIOCARTA\_TNFR2\_PATHWAY |
| Enrichment Score (ES) | -0.5481514 |
| Normalized Enrichment Score (NES) | -1.5634885 |
| Nominal p-value | 0.036117382 |
| FDR q-value | 0.18045859 |
| FWER p-Value | 0.864 |
Table: GSEA Results Summary

  

Fig 1: Enrichment plot: BIOCARTA\_TNFR2\_PATHWAY      
 Profile of the Running ES Score & Positions of GeneSet Members on the Rank Ordered List

  

| PROBE | GENE SYMBOL | GENE\_TITLE | RANK IN GENE LIST | RANK METRIC SCORE | RUNNING ES | CORE ENRICHMENT || 1 | LTA | LTA Entrez,  Source | lymphotoxin alpha (TNF superfamily, member 1) | 4963 | 0.087 | -0.1922 | No |
| 2 | TNFRSF1B | TNFRSF1B Entrez,  Source | tumor necrosis factor receptor superfamily, member 1B | 6614 | 0.066 | -0.2302 | No |
| 3 | IKBKAP | IKBKAP Entrez,  Source | inhibitor of kappa light polypeptide gene enhancer in B-cells, kinase complex-associated protein | 8548 | 0.042 | -0.2993 | No |
| 4 | TRAF3 | TRAF3 Entrez,  Source | TNF receptor-associated factor 3 | 10263 | 0.022 | -0.3716 | No |
| 5 | NFKB1 | NFKB1 Entrez,  Source | nuclear factor of kappa light polypeptide gene enhancer in B-cells 1 (p105) | 10617 | 0.018 | -0.3770 | No |
| 6 | TRAF2 | TRAF2 Entrez,  Source | TNF receptor-associated factor 2 | 13715 | -0.022 | -0.5199 | No |
| 7 | RELA | RELA Entrez,  Source | v-rel reticuloendotheliosis viral oncogene homolog A, nuclear factor of kappa light polypeptide gene enhancer in B-cells 3, p65 (avian) | 14051 | -0.027 | -0.5180 | No |
| 8 | TANK | TANK Entrez,  Source | TRAF family member-associated NFKB activator | 14642 | -0.037 | -0.5221 | Yes |
| 9 | TNFAIP3 | TNFAIP3 Entrez,  Source | tumor necrosis factor, alpha-induced protein 3 | 14660 | -0.037 | -0.4966 | Yes |
| 10 | DUSP1 | DUSP1 Entrez,  Source | dual specificity phosphatase 1 | 15222 | -0.048 | -0.4911 | Yes |
| 11 | CHUK | CHUK Entrez,  Source | conserved helix-loop-helix ubiquitous kinase | 15693 | -0.059 | -0.4736 | Yes |
| 12 | NFKBIA | NFKBIA Entrez,  Source | nuclear factor of kappa light polypeptide gene enhancer in B-cells inhibitor, alpha | 16196 | -0.072 | -0.4485 | Yes |
| 13 | MAP3K1 | MAP3K1 Entrez,  Source | mitogen-activated protein kinase kinase kinase 1 | 16375 | -0.077 | -0.4034 | Yes |
| 14 | TRAF1 | TRAF1 Entrez,  Source | TNF receptor-associated factor 1 | 17353 | -0.111 | -0.3748 | Yes |
| 15 | MAP3K14 | MAP3K14 Entrez,  Source | mitogen-activated protein kinase kinase kinase 14 | 18028 | -0.146 | -0.3063 | Yes |
| 16 | RIPK1 | RIPK1 Entrez,  Source | receptor (TNFRSF)-interacting serine-threonine kinase 1 | 18281 | -0.164 | -0.2033 | Yes |
| 17 | IKBKG | IKBKG Entrez,  Source | inhibitor of kappa light polypeptide gene enhancer in B-cells, kinase gamma | 18387 | -0.172 | -0.0871 | Yes |
| 18 | IKBKB | IKBKB Entrez,  Source | inhibitor of kappa light polypeptide gene enhancer in B-cells, kinase beta | 18696 | -0.208 | 0.0440 | Yes |
Table: GSEA details [plain text format]

  

Fig 2: BIOCARTA\_TNFR2\_PATHWAY      
 Blue-Pink O' Gram in the Space of the Analyzed GeneSet

  

Fig 3: BIOCARTA\_TNFR2\_PATHWAY: Random ES distribution      
 Gene set null distribution of ES for **BIOCARTA\_TNFR2\_PATHWAY**
