# Supplementary material for: Comprehensive analysis of β-catenin target genes in colorectal carcinoma cell lines with deregulated Wnt/β-catenin signaling
Source: BMC Genomics. 2014 Jan 28;15:74. doi: 10.1186/1471-2164-15-74 (PMC3909937; doi:10.1186/1471-2164-15-74)
Supplement: Additional file 4 — GSEA analysis using the Biocarta pathway database. This zipped file contains confirming data of the GSEA analysis. The names of the directories containing the files were composed of the term ‘GSEA’, the name of the cell line, e.g. DLD1, SW480, or LS174T, and the pathway database (Biocarta). Please use a web browser to view the files with the name ‘index.html’ in the corresponding directories to start exploring the data. [file 1471-2164-15-74-S4.zip › SW480_Biocarta/BIOCARTA_TOLL_PATHWAY.html]

Details for gene set BIOCARTA\_TOLL\_PATHWAY[GSEA]

|  || Dataset | SW480\_collapsed\_to\_symbols.class.cls#bg\_versus\_b |
| Phenotype | class.cls#bg\_versus\_b |
| Upregulated in class | b |
| GeneSet | BIOCARTA\_TOLL\_PATHWAY |
| Enrichment Score (ES) | -0.55029273 |
| Normalized Enrichment Score (NES) | -1.7673502 |
| Nominal p-value | 0.0049261083 |
| FDR q-value | 0.12697291 |
| FWER p-Value | 0.342 |
Table: GSEA Results Summary

  

Fig 1: Enrichment plot: BIOCARTA\_TOLL\_PATHWAY      
 Profile of the Running ES Score & Positions of GeneSet Members on the Rank Ordered List

  

| PROBE | GENE SYMBOL | GENE\_TITLE | RANK IN GENE LIST | RANK METRIC SCORE | RUNNING ES | CORE ENRICHMENT || 1 | TLR2 | TLR2 Entrez,  Source | toll-like receptor 2 | 630 | 0.233 | 0.0265 | No |
| 2 | ECSIT | ECSIT Entrez,  Source | ECSIT homolog (Drosophila) | 2116 | 0.146 | -0.0126 | No |
| 3 | MAPK14 | MAPK14 Entrez,  Source | mitogen-activated protein kinase 14 | 4293 | 0.098 | -0.0992 | No |
| 4 | PGLYRP1 | PGLYRP1 Entrez,  Source | peptidoglycan recognition protein 1 | 6100 | 0.072 | -0.1734 | No |
| 5 | MAP2K4 | MAP2K4 Entrez,  Source | mitogen-activated protein kinase kinase 4 | 6974 | 0.061 | -0.2026 | No |
| 6 | TLR9 | TLR9 Entrez,  Source | toll-like receptor 9 | 7729 | 0.052 | -0.2280 | No |
| 7 | TRAF6 | TRAF6 Entrez,  Source | TNF receptor-associated factor 6 | 9238 | 0.034 | -0.2968 | No |
| 8 | ELK1 | ELK1 Entrez,  Source | ELK1, member of ETS oncogene family | 9503 | 0.031 | -0.3026 | No |
| 9 | TLR6 | TLR6 Entrez,  Source | toll-like receptor 6 | 10012 | 0.025 | -0.3223 | No |
| 10 | NFKB1 | NFKB1 Entrez,  Source | nuclear factor of kappa light polypeptide gene enhancer in B-cells 1 (p105) | 10617 | 0.018 | -0.3487 | No |
| 11 | EIF2AK2 | EIF2AK2 Entrez,  Source | eukaryotic translation initiation factor 2-alpha kinase 2 | 11659 | 0.006 | -0.4006 | No |
| 12 | TLR10 | TLR10 Entrez,  Source | toll-like receptor 10 | 13005 | -0.012 | -0.4665 | No |
| 13 | TLR7 | TLR7 Entrez,  Source | toll-like receptor 7 | 13366 | -0.017 | -0.4808 | No |
| 14 | RELA | RELA Entrez,  Source | v-rel reticuloendotheliosis viral oncogene homolog A, nuclear factor of kappa light polypeptide gene enhancer in B-cells 3, p65 (avian) | 14051 | -0.027 | -0.5090 | No |
| 15 | MYD88 | MYD88 Entrez,  Source | myeloid differentiation primary response gene (88) | 14431 | -0.033 | -0.5200 | No |
| 16 | IRAK1 | IRAK1 Entrez,  Source | interleukin-1 receptor-associated kinase 1 | 14621 | -0.036 | -0.5205 | Yes |
| 17 | MAP3K7 | MAP3K7 Entrez,  Source | mitogen-activated protein kinase kinase kinase 7 | 14672 | -0.038 | -0.5135 | Yes |
| 18 | TOLLIP | TOLLIP Entrez,  Source | toll interacting protein | 14995 | -0.044 | -0.5190 | Yes |
| 19 | MAPK8 | MAPK8 Entrez,  Source | mitogen-activated protein kinase 8 | 15249 | -0.049 | -0.5196 | Yes |
| 20 | TLR3 | TLR3 Entrez,  Source | toll-like receptor 3 | 15473 | -0.054 | -0.5174 | Yes |
| 21 | CHUK | CHUK Entrez,  Source | conserved helix-loop-helix ubiquitous kinase | 15693 | -0.059 | -0.5137 | Yes |
| 22 | MAP2K6 | MAP2K6 Entrez,  Source | mitogen-activated protein kinase kinase 6 | 15852 | -0.062 | -0.5061 | Yes |
| 23 | NFKBIA | NFKBIA Entrez,  Source | nuclear factor of kappa light polypeptide gene enhancer in B-cells inhibitor, alpha | 16196 | -0.072 | -0.5055 | Yes |
| 24 | TIRAP | TIRAP Entrez,  Source | toll-interleukin 1 receptor (TIR) domain containing adaptor protein | 16368 | -0.077 | -0.4949 | Yes |
| 25 | MAP3K1 | MAP3K1 Entrez,  Source | mitogen-activated protein kinase kinase kinase 1 | 16375 | -0.077 | -0.4758 | Yes |
| 26 | JUN | JUN Entrez,  Source | jun oncogene | 17831 | -0.133 | -0.5168 | Yes |
| 27 | MAP3K14 | MAP3K14 Entrez,  Source | mitogen-activated protein kinase kinase kinase 14 | 18028 | -0.146 | -0.4900 | Yes |
| 28 | PPARA | PPARA Entrez,  Source | peroxisome proliferative activated receptor, alpha | 18063 | -0.148 | -0.4543 | Yes |
| 29 | CD14 | CD14 Entrez,  Source | CD14 molecule | 18269 | -0.163 | -0.4236 | Yes |
| 30 | IKBKG | IKBKG Entrez,  Source | inhibitor of kappa light polypeptide gene enhancer in B-cells, kinase gamma | 18387 | -0.172 | -0.3860 | Yes |
| 31 | IKBKB | IKBKB Entrez,  Source | inhibitor of kappa light polypeptide gene enhancer in B-cells, kinase beta | 18696 | -0.208 | -0.3492 | Yes |
| 32 | FOS | FOS Entrez,  Source | v-fos FBJ murine osteosarcoma viral oncogene homolog | 18736 | -0.212 | -0.2975 | Yes |
| 33 | TLR4 | TLR4 Entrez,  Source | toll-like receptor 4 | 19469 | -0.566 | -0.1920 | Yes |
| 34 | LY96 | LY96 Entrez,  Source | lymphocyte antigen 96 | 19532 | -0.777 | 0.0012 | Yes |
Table: GSEA details [plain text format]

  

Fig 2: BIOCARTA\_TOLL\_PATHWAY      
 Blue-Pink O' Gram in the Space of the Analyzed GeneSet

  

Fig 3: BIOCARTA\_TOLL\_PATHWAY: Random ES distribution      
 Gene set null distribution of ES for **BIOCARTA\_TOLL\_PATHWAY**
