# Supplementary material for: Comprehensive analysis of β-catenin target genes in colorectal carcinoma cell lines with deregulated Wnt/β-catenin signaling
Source: BMC Genomics. 2014 Jan 28;15:74. doi: 10.1186/1471-2164-15-74 (PMC3909937; doi:10.1186/1471-2164-15-74)
Supplement: Additional file 4 — GSEA analysis using the Biocarta pathway database. This zipped file contains confirming data of the GSEA analysis. The names of the directories containing the files were composed of the term ‘GSEA’, the name of the cell line, e.g. DLD1, SW480, or LS174T, and the pathway database (Biocarta). Please use a web browser to view the files with the name ‘index.html’ in the corresponding directories to start exploring the data. [file 1471-2164-15-74-S4.zip › SW480_Biocarta/BIOCARTA_VIP_PATHWAY.html]

Details for gene set BIOCARTA\_VIP\_PATHWAY[GSEA]

|  || Dataset | SW480\_collapsed\_to\_symbols.class.cls#bg\_versus\_b |
| Phenotype | class.cls#bg\_versus\_b |
| Upregulated in class | b |
| GeneSet | BIOCARTA\_VIP\_PATHWAY |
| Enrichment Score (ES) | -0.518433 |
| Normalized Enrichment Score (NES) | -1.5646536 |
| Nominal p-value | 0.030660378 |
| FDR q-value | 0.18795145 |
| FWER p-Value | 0.862 |
Table: GSEA Results Summary

  

Fig 1: Enrichment plot: BIOCARTA\_VIP\_PATHWAY      
 Profile of the Running ES Score & Positions of GeneSet Members on the Rank Ordered List

  

| PROBE | GENE SYMBOL | GENE\_TITLE | RANK IN GENE LIST | RANK METRIC SCORE | RUNNING ES | CORE ENRICHMENT || 1 | MYC | MYC Entrez,  Source | v-myc myelocytomatosis viral oncogene homolog (avian) | 447 | 0.262 | 0.0581 | No |
| 2 | PRKACG | PRKACG Entrez,  Source | protein kinase, cAMP-dependent, catalytic, gamma | 1307 | 0.178 | 0.0694 | No |
| 3 | VIP | VIP Entrez,  Source | vasoactive intestinal peptide | 2969 | 0.122 | 0.0223 | No |
| 4 | PLCG1 | PLCG1 Entrez,  Source | phospholipase C, gamma 1 | 4276 | 0.098 | -0.0141 | No |
| 5 | VIPR2 | VIPR2 Entrez,  Source | vasoactive intestinal peptide receptor 2 | 7725 | 0.052 | -0.1744 | No |
| 6 | GNAQ | GNAQ Entrez,  Source | guanine nucleotide binding protein (G protein), q polypeptide | 8794 | 0.039 | -0.2170 | No |
| 7 | NFKB1 | NFKB1 Entrez,  Source | nuclear factor of kappa light polypeptide gene enhancer in B-cells 1 (p105) | 10617 | 0.018 | -0.3048 | No |
| 8 | PRKAR2A | PRKAR2A Entrez,  Source | protein kinase, cAMP-dependent, regulatory, type II, alpha | 11520 | 0.007 | -0.3487 | No |
| 9 | NFATC1 | NFATC1 Entrez,  Source | nuclear factor of activated T-cells, cytoplasmic, calcineurin-dependent 1 | 13196 | -0.014 | -0.4300 | No |
| 10 | RELA | RELA Entrez,  Source | v-rel reticuloendotheliosis viral oncogene homolog A, nuclear factor of kappa light polypeptide gene enhancer in B-cells 3, p65 (avian) | 14051 | -0.027 | -0.4654 | No |
| 11 | PPP3CB | PPP3CB Entrez,  Source | protein phosphatase 3 (formerly 2B), catalytic subunit, beta isoform (calcineurin A beta) | 14346 | -0.032 | -0.4706 | No |
| 12 | NFATC2 | NFATC2 Entrez,  Source | nuclear factor of activated T-cells, cytoplasmic, calcineurin-dependent 2 | 14852 | -0.041 | -0.4838 | No |
| 13 | CALM2 | CALM2 Entrez,  Source | calmodulin 2 (phosphorylase kinase, delta) | 15530 | -0.055 | -0.5014 | Yes |
| 14 | CHUK | CHUK Entrez,  Source | conserved helix-loop-helix ubiquitous kinase | 15693 | -0.059 | -0.4915 | Yes |
| 15 | CALM1 | CALM1 Entrez,  Source | calmodulin 1 (phosphorylase kinase, delta) | 15850 | -0.062 | -0.4802 | Yes |
| 16 | PRKACB | PRKACB Entrez,  Source | protein kinase, cAMP-dependent, catalytic, beta | 15881 | -0.063 | -0.4622 | Yes |
| 17 | NFKBIA | NFKBIA Entrez,  Source | nuclear factor of kappa light polypeptide gene enhancer in B-cells inhibitor, alpha | 16196 | -0.072 | -0.4560 | Yes |
| 18 | MAP3K1 | MAP3K1 Entrez,  Source | mitogen-activated protein kinase kinase kinase 1 | 16375 | -0.077 | -0.4413 | Yes |
| 19 | CALM3 | CALM3 Entrez,  Source | calmodulin 3 (phosphorylase kinase, delta) | 17323 | -0.110 | -0.4557 | Yes |
| 20 | PPP3CC | PPP3CC Entrez,  Source | protein phosphatase 3 (formerly 2B), catalytic subunit, gamma isoform (calcineurin A gamma) | 17578 | -0.122 | -0.4310 | Yes |
| 21 | PRKAR1A | PRKAR1A Entrez,  Source | protein kinase, cAMP-dependent, regulatory, type I, alpha (tissue specific extinguisher 1) | 18683 | -0.206 | -0.4239 | Yes |
| 22 | EGR3 | EGR3 Entrez,  Source | early growth response 3 | 18901 | -0.241 | -0.3604 | Yes |
| 23 | PPP3CA | PPP3CA Entrez,  Source | protein phosphatase 3 (formerly 2B), catalytic subunit, alpha isoform (calcineurin A alpha) | 19160 | -0.304 | -0.2794 | Yes |
| 24 | EGR2 | EGR2 Entrez,  Source | early growth response 2 (Krox-20 homolog, Drosophila) | 19299 | -0.366 | -0.1732 | Yes |
| 25 | PRKAR2B | PRKAR2B Entrez,  Source | protein kinase, cAMP-dependent, regulatory, type II, beta | 19490 | -0.601 | 0.0034 | Yes |
Table: GSEA details [plain text format]

  

Fig 2: BIOCARTA\_VIP\_PATHWAY      
 Blue-Pink O' Gram in the Space of the Analyzed GeneSet

  

Fig 3: BIOCARTA\_VIP\_PATHWAY: Random ES distribution      
 Gene set null distribution of ES for **BIOCARTA\_VIP\_PATHWAY**
