# Supplementary material for: Comprehensive analysis of β-catenin target genes in colorectal carcinoma cell lines with deregulated Wnt/β-catenin signaling
Source: BMC Genomics. 2014 Jan 28;15:74. doi: 10.1186/1471-2164-15-74 (PMC3909937; doi:10.1186/1471-2164-15-74)
Supplement: Additional file 4 — GSEA analysis using the Biocarta pathway database. This zipped file contains confirming data of the GSEA analysis. The names of the directories containing the files were composed of the term ‘GSEA’, the name of the cell line, e.g. DLD1, SW480, or LS174T, and the pathway database (Biocarta). Please use a web browser to view the files with the name ‘index.html’ in the corresponding directories to start exploring the data. [file 1471-2164-15-74-S4.zip › SW480_Biocarta/BIOCARTA_WNT_PATHWAY.html]

Details for gene set BIOCARTA\_WNT\_PATHWAY[GSEA]

|  || Dataset | SW480\_collapsed\_to\_symbols.class.cls#bg\_versus\_b |
| Phenotype | class.cls#bg\_versus\_b |
| Upregulated in class | bg |
| GeneSet | BIOCARTA\_WNT\_PATHWAY |
| Enrichment Score (ES) | 0.49826708 |
| Normalized Enrichment Score (NES) | 1.4577353 |
| Nominal p-value | 0.052718285 |
| FDR q-value | 0.6047143 |
| FWER p-Value | 0.99 |
Table: GSEA Results Summary

  

Fig 1: Enrichment plot: BIOCARTA\_WNT\_PATHWAY      
 Profile of the Running ES Score & Positions of GeneSet Members on the Rank Ordered List

  

| PROBE | GENE SYMBOL | GENE\_TITLE | RANK IN GENE LIST | RANK METRIC SCORE | RUNNING ES | CORE ENRICHMENT || 1 | NLK | NLK Entrez,  Source | nemo-like kinase | 266 | 0.321 | 0.1046 | Yes |
| 2 | MYC | MYC Entrez,  Source | v-myc myelocytomatosis viral oncogene homolog (avian) | 447 | 0.262 | 0.1918 | Yes |
| 3 | FRAT1 | FRAT1 Entrez,  Source | frequently rearranged in advanced T-cell lymphomas | 461 | 0.259 | 0.2865 | Yes |
| 4 | CTNNB1 | CTNNB1 Entrez,  Source | catenin (cadherin-associated protein), beta 1, 88kDa | 613 | 0.235 | 0.3656 | Yes |
| 5 | BTRC | BTRC Entrez,  Source | beta-transducin repeat containing | 738 | 0.219 | 0.4401 | Yes |
| 6 | PPARD | PPARD Entrez,  Source | peroxisome proliferative activated receptor, delta | 1148 | 0.187 | 0.4882 | Yes |
| 7 | AXIN1 | AXIN1 Entrez,  Source | axin 1 | 2026 | 0.149 | 0.4983 | Yes |
| 8 | CSNK2A1 | CSNK2A1 Entrez,  Source | casein kinase 2, alpha 1 polypeptide | 7178 | 0.059 | 0.2562 | No |
| 9 | LEF1 | LEF1 Entrez,  Source | lymphoid enhancer-binding factor 1 | 7483 | 0.055 | 0.2610 | No |
| 10 | WIF1 | WIF1 Entrez,  Source | WNT inhibitory factor 1 | 7564 | 0.054 | 0.2769 | No |
| 11 | DVL1 | DVL1 Entrez,  Source | dishevelled, dsh homolog 1 (Drosophila) | 7661 | 0.053 | 0.2916 | No |
| 12 | WNT1 | WNT1 Entrez,  Source | wingless-type MMTV integration site family, member 1 | 7800 | 0.051 | 0.3034 | No |
| 13 | APC | APC Entrez,  Source | adenomatosis polyposis coli | 9898 | 0.026 | 0.2056 | No |
| 14 | SMAD4 | SMAD4 Entrez,  Source | SMAD, mothers against DPP homolog 4 (Drosophila) | 9920 | 0.026 | 0.2140 | No |
| 15 | PPP2CA | PPP2CA Entrez,  Source | protein phosphatase 2 (formerly 2A), catalytic subunit, alpha isoform | 11890 | 0.003 | 0.1142 | No |
| 16 | CCND1 | CCND1 Entrez,  Source | cyclin D1 | 12074 | 0.000 | 0.1050 | No |
| 17 | CTBP1 | CTBP1 Entrez,  Source | C-terminal binding protein 1 | 12359 | -0.003 | 0.0915 | No |
| 18 | GSK3B | GSK3B Entrez,  Source | glycogen synthase kinase 3 beta | 12732 | -0.008 | 0.0755 | No |
| 19 | CSNK1A1 | CSNK1A1 Entrez,  Source | casein kinase 1, alpha 1 | 13672 | -0.021 | 0.0353 | No |
| 20 | CSNK1D | CSNK1D Entrez,  Source | casein kinase 1, delta | 14632 | -0.037 | -0.0003 | No |
| 21 | MAP3K7 | MAP3K7 Entrez,  Source | mitogen-activated protein kinase kinase kinase 7 | 14672 | -0.038 | 0.0116 | No |
| 22 | HDAC1 | HDAC1 Entrez,  Source | histone deacetylase 1 | 14971 | -0.043 | 0.0122 | No |
| 23 | CREBBP | CREBBP Entrez,  Source | CREB binding protein (Rubinstein-Taybi syndrome) | 15257 | -0.049 | 0.0157 | No |
| 24 | TLE1 | TLE1 Entrez,  Source | transducin-like enhancer of split 1 (E(sp1) homolog, Drosophila) | 16604 | -0.084 | -0.0223 | No |
| 25 | FZD1 | FZD1 Entrez,  Source | frizzled homolog 1 (Drosophila) | 19428 | -0.470 | 0.0066 | No |
Table: GSEA details [plain text format]

  

Fig 2: BIOCARTA\_WNT\_PATHWAY      
 Blue-Pink O' Gram in the Space of the Analyzed GeneSet

  

Fig 3: BIOCARTA\_WNT\_PATHWAY: Random ES distribution      
 Gene set null distribution of ES for **BIOCARTA\_WNT\_PATHWAY**
