# Supplementary material for: Comprehensive analysis of β-catenin target genes in colorectal carcinoma cell lines with deregulated Wnt/β-catenin signaling
Source: BMC Genomics. 2014 Jan 28;15:74. doi: 10.1186/1471-2164-15-74 (PMC3909937; doi:10.1186/1471-2164-15-74)
Supplement: Additional file 4 — GSEA analysis using the Biocarta pathway database. This zipped file contains confirming data of the GSEA analysis. The names of the directories containing the files were composed of the term ‘GSEA’, the name of the cell line, e.g. DLD1, SW480, or LS174T, and the pathway database (Biocarta). Please use a web browser to view the files with the name ‘index.html’ in the corresponding directories to start exploring the data. [file 1471-2164-15-74-S4.zip › SW480_Biocarta/gsea_report_for_b_1355838868203.html]

Report for b 1355838868203 [GSEA]

| GS  follow link to MSigDB | GS DETAILS | SIZE | ES | NES | NOM p-val | FDR q-val | FWER p-val | RANK AT MAX | LEADING EDGE || 1 | BIOCARTA\_IGF1R\_PATHWAY | Details ... | 22 | -0.70 | -2.03 | 0.000 | 0.056 | 0.030 | 1354 | tags=27%, list=7%, signal=29% |
| 2 | BIOCARTA\_INSULIN\_PATHWAY | Details ... | 22 | -0.64 | -1.95 | 0.002 | 0.062 | 0.067 | 1727 | tags=18%, list=9%, signal=20% |
| 3 | BIOCARTA\_NFKB\_PATHWAY | Details ... | 22 | -0.63 | -1.86 | 0.005 | 0.105 | 0.160 | 1819 | tags=41%, list=9%, signal=45% |
| 4 | BIOCARTA\_GCR\_PATHWAY | Details ... | 19 | -0.63 | -1.78 | 0.015 | 0.165 | 0.304 | 1157 | tags=21%, list=6%, signal=22% |
| 5 | BIOCARTA\_PPARA\_PATHWAY | Details ... | 52 | -0.50 | -1.78 | 0.000 | 0.133 | 0.305 | 2314 | tags=29%, list=12%, signal=33% |
| 6 | BIOCARTA\_TOLL\_PATHWAY | Details ... | 34 | -0.55 | -1.77 | 0.005 | 0.127 | 0.342 | 1727 | tags=26%, list=9%, signal=29% |
| 7 | BIOCARTA\_DEATH\_PATHWAY | Details ... | 32 | -0.54 | -1.76 | 0.009 | 0.118 | 0.362 | 2098 | tags=38%, list=11%, signal=42% |
| 8 | BIOCARTA\_CERAMIDE\_PATHWAY | Details ... | 21 | -0.59 | -1.71 | 0.009 | 0.147 | 0.474 | 2098 | tags=33%, list=11%, signal=37% |
| 9 | BIOCARTA\_IGF1\_PATHWAY | Details ... | 21 | -0.59 | -1.70 | 0.014 | 0.147 | 0.505 | 1727 | tags=24%, list=9%, signal=26% |
| 10 | BIOCARTA\_SPRY\_PATHWAY | Details ... | 18 | -0.60 | -1.69 | 0.017 | 0.136 | 0.517 | 1300 | tags=28%, list=7%, signal=30% |
| 11 | BIOCARTA\_MCALPAIN\_PATHWAY | Details ... | 22 | -0.57 | -1.68 | 0.016 | 0.137 | 0.561 | 2791 | tags=41%, list=14%, signal=48% |
| 12 | BIOCARTA\_NDKDYNAMIN\_PATHWAY | Details ... | 17 | -0.60 | -1.67 | 0.026 | 0.135 | 0.587 | 4028 | tags=59%, list=21%, signal=74% |
| 13 | BIOCARTA\_EIF4\_PATHWAY | Details ... | 22 | -0.56 | -1.66 | 0.013 | 0.142 | 0.642 | 10 | tags=5%, list=0%, signal=5% |
| 14 | BIOCARTA\_STRESS\_PATHWAY | Details ... | 24 | -0.55 | -1.65 | 0.025 | 0.144 | 0.673 | 4319 | tags=63%, list=22%, signal=80% |
| 15 | BIOCARTA\_CHEMICAL\_PATHWAY | Details ... | 21 | -0.54 | -1.61 | 0.022 | 0.173 | 0.761 | 4536 | tags=48%, list=23%, signal=62% |
| 16 | BIOCARTA\_RELA\_PATHWAY | Details ... | 16 | -0.58 | -1.59 | 0.031 | 0.181 | 0.803 | 4665 | tags=69%, list=24%, signal=90% |
| 17 | BIOCARTA\_GSK3\_PATHWAY | Details ... | 27 | -0.52 | -1.58 | 0.027 | 0.185 | 0.830 | 130 | tags=11%, list=1%, signal=11% |
| 18 | BIOCARTA\_TEL\_PATHWAY | Details ... | 18 | -0.56 | -1.57 | 0.035 | 0.197 | 0.859 | 2439 | tags=44%, list=12%, signal=51% |
| 19 | BIOCARTA\_VIP\_PATHWAY | Details ... | 25 | -0.52 | -1.56 | 0.031 | 0.188 | 0.862 | 4028 | tags=52%, list=21%, signal=65% |
| 20 | BIOCARTA\_TNFR2\_PATHWAY | Details ... | 18 | -0.55 | -1.56 | 0.036 | 0.180 | 0.864 | 4916 | tags=61%, list=25%, signal=82% |
| 21 | BIOCARTA\_P53\_PATHWAY |  | 16 | -0.56 | -1.55 | 0.028 | 0.188 | 0.885 | 1423 | tags=19%, list=7%, signal=20% |
| 22 | BIOCARTA\_NGF\_PATHWAY |  | 17 | -0.54 | -1.54 | 0.044 | 0.193 | 0.907 | 1727 | tags=24%, list=9%, signal=26% |
| 23 | BIOCARTA\_CREB\_PATHWAY |  | 25 | -0.49 | -1.54 | 0.025 | 0.189 | 0.910 | 2521 | tags=24%, list=13%, signal=28% |
| 24 | BIOCARTA\_TID\_PATHWAY |  | 19 | -0.53 | -1.53 | 0.050 | 0.185 | 0.917 | 1423 | tags=32%, list=7%, signal=34% |
| 25 | BIOCARTA\_ALK\_PATHWAY |  | 36 | -0.46 | -1.51 | 0.036 | 0.205 | 0.944 | 1610 | tags=22%, list=8%, signal=24% |
| 26 | BIOCARTA\_P38MAPK\_PATHWAY |  | 39 | -0.44 | -1.47 | 0.035 | 0.247 | 0.967 | 3253 | tags=36%, list=17%, signal=43% |
| 27 | BIOCARTA\_HIVNEF\_PATHWAY |  | 55 | -0.41 | -1.46 | 0.024 | 0.247 | 0.973 | 2205 | tags=29%, list=11%, signal=33% |
| 28 | BIOCARTA\_ERK\_PATHWAY |  | 27 | -0.47 | -1.46 | 0.053 | 0.240 | 0.975 | 2656 | tags=26%, list=14%, signal=30% |
| 29 | BIOCARTA\_NOS1\_PATHWAY |  | 19 | -0.50 | -1.46 | 0.080 | 0.234 | 0.975 | 4100 | tags=53%, list=21%, signal=67% |
| 30 | BIOCARTA\_CK1\_PATHWAY |  | 16 | -0.54 | -1.46 | 0.054 | 0.232 | 0.976 | 875 | tags=19%, list=4%, signal=20% |
| 31 | BIOCARTA\_IL1R\_PATHWAY |  | 31 | -0.45 | -1.44 | 0.070 | 0.241 | 0.979 | 1819 | tags=23%, list=9%, signal=25% |
| 32 | BIOCARTA\_MAPK\_PATHWAY |  | 85 | -0.37 | -1.44 | 0.036 | 0.238 | 0.981 | 3362 | tags=32%, list=17%, signal=38% |
| 33 | BIOCARTA\_INTEGRIN\_PATHWAY |  | 37 | -0.44 | -1.44 | 0.057 | 0.237 | 0.983 | 4343 | tags=46%, list=22%, signal=59% |
| 34 | BIOCARTA\_GPCR\_PATHWAY |  | 32 | -0.44 | -1.43 | 0.050 | 0.237 | 0.986 | 2521 | tags=31%, list=13%, signal=36% |
| 35 | BIOCARTA\_KERATINOCYTE\_PATHWAY |  | 44 | -0.42 | -1.43 | 0.049 | 0.233 | 0.987 | 1909 | tags=27%, list=10%, signal=30% |
| 36 | BIOCARTA\_GH\_PATHWAY |  | 26 | -0.46 | -1.42 | 0.058 | 0.230 | 0.988 | 2651 | tags=15%, list=14%, signal=18% |
| 37 | BIOCARTA\_STATHMIN\_PATHWAY |  | 17 | -0.51 | -1.40 | 0.099 | 0.250 | 0.995 | 1125 | tags=24%, list=6%, signal=25% |
| 38 | BIOCARTA\_EGF\_PATHWAY |  | 30 | -0.43 | -1.39 | 0.086 | 0.265 | 0.996 | 3183 | tags=30%, list=16%, signal=36% |
| 39 | BIOCARTA\_PROTEASOME\_PATHWAY |  | 26 | -0.45 | -1.36 | 0.101 | 0.296 | 0.997 | 9013 | tags=96%, list=46%, signal=178% |
| 40 | BIOCARTA\_ECM\_PATHWAY |  | 23 | -0.45 | -1.34 | 0.117 | 0.326 | 0.998 | 2323 | tags=22%, list=12%, signal=25% |
| 41 | BIOCARTA\_TPO\_PATHWAY |  | 23 | -0.46 | -1.33 | 0.132 | 0.323 | 0.998 | 3034 | tags=30%, list=16%, signal=36% |
| 42 | BIOCARTA\_CALCINEURIN\_PATHWAY |  | 17 | -0.48 | -1.33 | 0.149 | 0.324 | 0.998 | 2235 | tags=29%, list=11%, signal=33% |
| 43 | BIOCARTA\_MITOCHONDRIA\_PATHWAY |  | 18 | -0.47 | -1.30 | 0.128 | 0.367 | 1.000 | 2098 | tags=28%, list=11%, signal=31% |
| 44 | BIOCARTA\_PDGF\_PATHWAY |  | 31 | -0.40 | -1.29 | 0.123 | 0.360 | 1.000 | 3264 | tags=29%, list=17%, signal=35% |
| 45 | BIOCARTA\_G2\_PATHWAY |  | 23 | -0.44 | -1.29 | 0.162 | 0.365 | 1.000 | 4878 | tags=52%, list=25%, signal=69% |
| 46 | BIOCARTA\_IL2RB\_PATHWAY |  | 38 | -0.38 | -1.29 | 0.125 | 0.358 | 1.000 | 1252 | tags=16%, list=6%, signal=17% |
| 47 | BIOCARTA\_CD40\_PATHWAY |  | 15 | -0.48 | -1.28 | 0.181 | 0.352 | 1.000 | 4898 | tags=53%, list=25%, signal=71% |
| 48 | BIOCARTA\_NO1\_PATHWAY |  | 28 | -0.41 | -1.27 | 0.167 | 0.369 | 1.000 | 1157 | tags=14%, list=6%, signal=15% |
| 49 | BIOCARTA\_RACCYCD\_PATHWAY |  | 26 | -0.40 | -1.25 | 0.177 | 0.394 | 1.000 | 3997 | tags=31%, list=20%, signal=39% |
| 50 | BIOCARTA\_ATRBRCA\_PATHWAY |  | 21 | -0.42 | -1.24 | 0.181 | 0.397 | 1.000 | 3274 | tags=33%, list=17%, signal=40% |
| 51 | BIOCARTA\_AT1R\_PATHWAY |  | 31 | -0.38 | -1.23 | 0.199 | 0.410 | 1.000 | 4448 | tags=48%, list=23%, signal=63% |
| 52 | BIOCARTA\_TGFB\_PATHWAY |  | 18 | -0.44 | -1.22 | 0.210 | 0.414 | 1.000 | 1153 | tags=17%, list=6%, signal=18% |
| 53 | BIOCARTA\_TFF\_PATHWAY |  | 21 | -0.42 | -1.21 | 0.207 | 0.430 | 1.000 | 2323 | tags=24%, list=12%, signal=27% |
| 54 | BIOCARTA\_RHO\_PATHWAY |  | 31 | -0.36 | -1.20 | 0.213 | 0.441 | 1.000 | 1553 | tags=19%, list=8%, signal=21% |
| 55 | BIOCARTA\_VEGF\_PATHWAY |  | 27 | -0.38 | -1.19 | 0.216 | 0.439 | 1.000 | 3364 | tags=22%, list=17%, signal=27% |
| 56 | BIOCARTA\_AGR\_PATHWAY |  | 35 | -0.36 | -1.19 | 0.217 | 0.447 | 1.000 | 3253 | tags=29%, list=17%, signal=34% |
| 57 | BIOCARTA\_PYK2\_PATHWAY |  | 26 | -0.37 | -1.15 | 0.248 | 0.502 | 1.000 | 4309 | tags=42%, list=22%, signal=54% |
| 58 | BIOCARTA\_MET\_PATHWAY |  | 35 | -0.34 | -1.14 | 0.268 | 0.503 | 1.000 | 4343 | tags=31%, list=22%, signal=40% |
| 59 | BIOCARTA\_ETS\_PATHWAY |  | 18 | -0.40 | -1.13 | 0.285 | 0.516 | 1.000 | 1727 | tags=28%, list=9%, signal=30% |
| 60 | BIOCARTA\_CDC42RAC\_PATHWAY |  | 15 | -0.42 | -1.12 | 0.329 | 0.540 | 1.000 | 4594 | tags=53%, list=23%, signal=70% |
| 61 | BIOCARTA\_ACTINY\_PATHWAY |  | 19 | -0.38 | -1.10 | 0.344 | 0.566 | 1.000 | 4891 | tags=47%, list=25%, signal=63% |
| 62 | BIOCARTA\_BAD\_PATHWAY |  | 25 | -0.37 | -1.10 | 0.339 | 0.559 | 1.000 | 1354 | tags=16%, list=7%, signal=17% |
| 63 | BIOCARTA\_NTHI\_PATHWAY |  | 23 | -0.37 | -1.09 | 0.354 | 0.559 | 1.000 | 5127 | tags=57%, list=26%, signal=77% |
| 64 | BIOCARTA\_TNFR1\_PATHWAY |  | 29 | -0.34 | -1.09 | 0.322 | 0.553 | 1.000 | 4319 | tags=45%, list=22%, signal=57% |
| 65 | BIOCARTA\_MAL\_PATHWAY |  | 18 | -0.39 | -1.08 | 0.359 | 0.560 | 1.000 | 3341 | tags=33%, list=17%, signal=40% |
| 66 | BIOCARTA\_CCR5\_PATHWAY |  | 15 | -0.40 | -1.07 | 0.361 | 0.568 | 1.000 | 2490 | tags=40%, list=13%, signal=46% |
| 67 | BIOCARTA\_CASPASE\_PATHWAY |  | 22 | -0.36 | -1.07 | 0.366 | 0.573 | 1.000 | 2098 | tags=27%, list=11%, signal=31% |
| 68 | BIOCARTA\_HCMV\_PATHWAY |  | 16 | -0.38 | -1.06 | 0.385 | 0.573 | 1.000 | 3706 | tags=31%, list=19%, signal=39% |
| 69 | BIOCARTA\_G1\_PATHWAY |  | 26 | -0.35 | -1.06 | 0.364 | 0.566 | 1.000 | 3997 | tags=31%, list=20%, signal=39% |
| 70 | BIOCARTA\_ATM\_PATHWAY |  | 20 | -0.36 | -1.05 | 0.397 | 0.586 | 1.000 | 4878 | tags=50%, list=25%, signal=67% |
| 71 | BIOCARTA\_GLEEVEC\_PATHWAY |  | 23 | -0.34 | -1.04 | 0.366 | 0.584 | 1.000 | 4309 | tags=39%, list=22%, signal=50% |
| 72 | BIOCARTA\_INTRINSIC\_PATHWAY |  | 23 | -0.34 | -1.03 | 0.395 | 0.598 | 1.000 | 1585 | tags=17%, list=8%, signal=19% |
| 73 | BIOCARTA\_CHREBP2\_PATHWAY |  | 41 | -0.30 | -1.01 | 0.452 | 0.631 | 1.000 | 1840 | tags=15%, list=9%, signal=16% |
| 74 | BIOCARTA\_41BB\_PATHWAY |  | 17 | -0.37 | -1.01 | 0.423 | 0.626 | 1.000 | 4309 | tags=53%, list=22%, signal=68% |
| 75 | BIOCARTA\_IL22BP\_PATHWAY |  | 16 | -0.36 | -1.00 | 0.458 | 0.631 | 1.000 | 3232 | tags=31%, list=17%, signal=37% |
| 76 | BIOCARTA\_CELLCYCLE\_PATHWAY |  | 22 | -0.35 | -0.99 | 0.478 | 0.656 | 1.000 | 3248 | tags=41%, list=17%, signal=49% |
| 77 | BIOCARTA\_AKT\_PATHWAY |  | 19 | -0.34 | -0.97 | 0.507 | 0.671 | 1.000 | 1252 | tags=16%, list=6%, signal=17% |
| 78 | BIOCARTA\_AMI\_PATHWAY |  | 19 | -0.34 | -0.97 | 0.514 | 0.675 | 1.000 | 1585 | tags=21%, list=8%, signal=23% |
| 79 | BIOCARTA\_INFLAM\_PATHWAY |  | 29 | -0.30 | -0.96 | 0.484 | 0.673 | 1.000 | 589 | tags=10%, list=3%, signal=11% |
| 80 | BIOCARTA\_UCALPAIN\_PATHWAY |  | 16 | -0.35 | -0.94 | 0.502 | 0.703 | 1.000 | 3341 | tags=38%, list=17%, signal=45% |
| 81 | BIOCARTA\_EPO\_PATHWAY |  | 19 | -0.33 | -0.93 | 0.540 | 0.714 | 1.000 | 2651 | tags=21%, list=14%, signal=24% |
| 82 | BIOCARTA\_P53HYPOXIA\_PATHWAY |  | 22 | -0.31 | -0.92 | 0.573 | 0.740 | 1.000 | 1401 | tags=23%, list=7%, signal=24% |
| 83 | BIOCARTA\_MPR\_PATHWAY |  | 31 | -0.29 | -0.91 | 0.580 | 0.738 | 1.000 | 4594 | tags=45%, list=23%, signal=59% |
| 84 | BIOCARTA\_IL3\_PATHWAY |  | 15 | -0.34 | -0.91 | 0.578 | 0.743 | 1.000 | 2651 | tags=20%, list=14%, signal=23% |
| 85 | BIOCARTA\_FCER1\_PATHWAY |  | 37 | -0.28 | -0.90 | 0.608 | 0.737 | 1.000 | 2235 | tags=19%, list=11%, signal=21% |
| 86 | BIOCARTA\_SHH\_PATHWAY |  | 15 | -0.33 | -0.90 | 0.568 | 0.736 | 1.000 | 1969 | tags=20%, list=10%, signal=22% |
| 87 | BIOCARTA\_PGC1A\_PATHWAY |  | 22 | -0.30 | -0.90 | 0.607 | 0.733 | 1.000 | 4028 | tags=41%, list=21%, signal=51% |
| 88 | BIOCARTA\_LAIR\_PATHWAY |  | 16 | -0.32 | -0.87 | 0.613 | 0.782 | 1.000 | 2410 | tags=25%, list=12%, signal=28% |
| 89 | BIOCARTA\_IL6\_PATHWAY |  | 22 | -0.28 | -0.85 | 0.654 | 0.804 | 1.000 | 3811 | tags=32%, list=19%, signal=39% |
| 90 | BIOCARTA\_BIOPEPTIDES\_PATHWAY |  | 41 | -0.24 | -0.85 | 0.692 | 0.798 | 1.000 | 2656 | tags=24%, list=14%, signal=28% |
| 91 | BIOCARTA\_PTDINS\_PATHWAY |  | 23 | -0.29 | -0.84 | 0.667 | 0.808 | 1.000 | 3006 | tags=22%, list=15%, signal=26% |
| 92 | BIOCARTA\_CTCF\_PATHWAY |  | 22 | -0.29 | -0.83 | 0.700 | 0.817 | 1.000 | 1153 | tags=14%, list=6%, signal=14% |
| 93 | BIOCARTA\_BCR\_PATHWAY |  | 33 | -0.25 | -0.82 | 0.749 | 0.820 | 1.000 | 2235 | tags=21%, list=11%, signal=24% |
| 94 | BIOCARTA\_RAC1\_PATHWAY |  | 23 | -0.26 | -0.81 | 0.702 | 0.825 | 1.000 | 1767 | tags=13%, list=9%, signal=14% |
| 95 | BIOCARTA\_NFAT\_PATHWAY |  | 52 | -0.22 | -0.80 | 0.816 | 0.845 | 1.000 | 2235 | tags=13%, list=11%, signal=15% |
| 96 | BIOCARTA\_FAS\_PATHWAY |  | 30 | -0.25 | -0.78 | 0.813 | 0.855 | 1.000 | 2098 | tags=23%, list=11%, signal=26% |
| 97 | BIOCARTA\_ERK5\_PATHWAY |  | 17 | -0.26 | -0.73 | 0.816 | 0.921 | 1.000 | 4448 | tags=29%, list=23%, signal=38% |
| 98 | BIOCARTA\_FMLP\_PATHWAY |  | 34 | -0.22 | -0.71 | 0.879 | 0.933 | 1.000 | 4706 | tags=32%, list=24%, signal=43% |
| 99 | BIOCARTA\_MYOSIN\_PATHWAY |  | 30 | -0.23 | -0.71 | 0.882 | 0.925 | 1.000 | 1909 | tags=13%, list=10%, signal=15% |
| 100 | BIOCARTA\_MTOR\_PATHWAY |  | 21 | -0.24 | -0.71 | 0.854 | 0.920 | 1.000 | 14783 | tags=100%, list=76%, signal=409% |
| 101 | BIOCARTA\_CARM\_ER\_PATHWAY |  | 32 | -0.22 | -0.70 | 0.917 | 0.919 | 1.000 | 4889 | tags=44%, list=25%, signal=58% |
| 102 | BIOCARTA\_HDAC\_PATHWAY |  | 28 | -0.22 | -0.68 | 0.891 | 0.933 | 1.000 | 4706 | tags=36%, list=24%, signal=47% |
| 103 | BIOCARTA\_RAS\_PATHWAY |  | 22 | -0.23 | -0.67 | 0.899 | 0.930 | 1.000 | 3865 | tags=23%, list=20%, signal=28% |
| 104 | BIOCARTA\_PTEN\_PATHWAY |  | 17 | -0.23 | -0.65 | 0.904 | 0.944 | 1.000 | 6404 | tags=65%, list=33%, signal=96% |
| 105 | BIOCARTA\_TALL1\_PATHWAY |  | 15 | -0.22 | -0.59 | 0.910 | 0.973 | 1.000 | 15265 | tags=100%, list=78%, signal=455% |
| 106 | BIOCARTA\_PML\_PATHWAY |  | 17 | -0.19 | -0.54 | 0.977 | 0.981 | 1.000 | 4301 | tags=35%, list=22%, signal=45% |
Table: Gene sets enriched in phenotype **b (3 samples) **[plain text format]****
