# Supplementary material for: Comprehensive analysis of β-catenin target genes in colorectal carcinoma cell lines with deregulated Wnt/β-catenin signaling
Source: BMC Genomics. 2014 Jan 28;15:74. doi: 10.1186/1471-2164-15-74 (PMC3909937; doi:10.1186/1471-2164-15-74)
Supplement: Additional file 4 — GSEA analysis using the Biocarta pathway database. This zipped file contains confirming data of the GSEA analysis. The names of the directories containing the files were composed of the term ‘GSEA’, the name of the cell line, e.g. DLD1, SW480, or LS174T, and the pathway database (Biocarta). Please use a web browser to view the files with the name ‘index.html’ in the corresponding directories to start exploring the data. [file 1471-2164-15-74-S4.zip › SW480_Biocarta/gsea_report_for_bg_1355838868203.html]

Report for bg 1355838868203 [GSEA]

| GS  follow link to MSigDB | GS DETAILS | SIZE | ES | NES | NOM p-val | FDR q-val | FWER p-val | RANK AT MAX | LEADING EDGE || 1 | BIOCARTA\_TH1TH2\_PATHWAY | Details ... | 19 | 0.56 | 1.56 | 0.021 | 0.878 | 0.897 | 5108 | tags=47%, list=26%, signal=64% |
| 2 | BIOCARTA\_CTLA4\_PATHWAY | Details ... | 20 | 0.55 | 1.51 | 0.032 | 0.648 | 0.968 | 5539 | tags=45%, list=28%, signal=63% |
| 3 | BIOCARTA\_WNT\_PATHWAY | Details ... | 25 | 0.50 | 1.46 | 0.053 | 0.605 | 0.990 | 2026 | tags=28%, list=10%, signal=31% |
| 4 | BIOCARTA\_NKT\_PATHWAY | Details ... | 27 | 0.47 | 1.43 | 0.073 | 0.547 | 0.995 | 2893 | tags=33%, list=15%, signal=39% |
| 5 | BIOCARTA\_IL12\_PATHWAY | Details ... | 21 | 0.49 | 1.41 | 0.068 | 0.492 | 0.997 | 1680 | tags=19%, list=9%, signal=21% |
| 6 | BIOCARTA\_CSK\_PATHWAY | Details ... | 22 | 0.47 | 1.37 | 0.094 | 0.508 | 1.000 | 1307 | tags=18%, list=7%, signal=19% |
| 7 | BIOCARTA\_IL17\_PATHWAY | Details ... | 16 | 0.48 | 1.28 | 0.176 | 0.722 | 1.000 | 896 | tags=19%, list=5%, signal=20% |
| 8 | BIOCARTA\_CXCR4\_PATHWAY | Details ... | 23 | 0.43 | 1.23 | 0.187 | 0.769 | 1.000 | 133 | tags=9%, list=1%, signal=9% |
| 9 | BIOCARTA\_DC\_PATHWAY | Details ... | 22 | 0.43 | 1.23 | 0.179 | 0.691 | 1.000 | 4164 | tags=55%, list=21%, signal=69% |
| 10 | BIOCARTA\_CYTOKINE\_PATHWAY | Details ... | 21 | 0.41 | 1.16 | 0.278 | 0.863 | 1.000 | 3058 | tags=43%, list=16%, signal=51% |
| 11 | BIOCARTA\_TCR\_PATHWAY | Details ... | 43 | 0.34 | 1.13 | 0.270 | 0.863 | 1.000 | 750 | tags=7%, list=4%, signal=7% |
| 12 | BIOCARTA\_COMP\_PATHWAY | Details ... | 15 | 0.45 | 1.12 | 0.300 | 0.813 | 1.000 | 7427 | tags=67%, list=38%, signal=107% |
| 13 | BIOCARTA\_IL2\_PATHWAY | Details ... | 22 | 0.40 | 1.12 | 0.300 | 0.773 | 1.000 | 750 | tags=14%, list=4%, signal=14% |
| 14 | BIOCARTA\_SPPA\_PATHWAY | Details ... | 19 | 0.41 | 1.11 | 0.305 | 0.727 | 1.000 | 88 | tags=11%, list=0%, signal=11% |
| 15 | BIOCARTA\_IGF1MTOR\_PATHWAY | Details ... | 19 | 0.36 | 1.00 | 0.458 | 0.988 | 1.000 | 333 | tags=5%, list=2%, signal=5% |
| 16 | BIOCARTA\_NO2IL12\_PATHWAY | Details ... | 15 | 0.38 | 0.99 | 0.480 | 0.943 | 1.000 | 9026 | tags=67%, list=46%, signal=124% |
| 17 | BIOCARTA\_MEF2D\_PATHWAY | Details ... | 18 | 0.36 | 0.98 | 0.492 | 0.919 | 1.000 | 17 | tags=6%, list=0%, signal=6% |
| 18 | BIOCARTA\_CARDIACEGF\_PATHWAY | Details ... | 17 | 0.36 | 0.98 | 0.497 | 0.873 | 1.000 | 1979 | tags=29%, list=10%, signal=33% |
| 19 | BIOCARTA\_NKCELLS\_PATHWAY | Details ... | 17 | 0.36 | 0.94 | 0.548 | 0.922 | 1.000 | 61 | tags=6%, list=0%, signal=6% |
| 20 | BIOCARTA\_HSP27\_PATHWAY | Details ... | 15 | 0.35 | 0.92 | 0.561 | 0.934 | 1.000 | 2207 | tags=27%, list=11%, signal=30% |
| 21 | BIOCARTA\_TOB1\_PATHWAY |  | 20 | 0.32 | 0.90 | 0.618 | 0.920 | 1.000 | 2099 | tags=15%, list=11%, signal=17% |
| 22 | BIOCARTA\_IL10\_PATHWAY |  | 17 | 0.33 | 0.89 | 0.605 | 0.897 | 1.000 | 304 | tags=12%, list=2%, signal=12% |
| 23 | BIOCARTA\_STEM\_PATHWAY |  | 15 | 0.33 | 0.88 | 0.639 | 0.891 | 1.000 | 6773 | tags=60%, list=35%, signal=92% |
| 24 | BIOCARTA\_CCR3\_PATHWAY |  | 22 | 0.30 | 0.85 | 0.657 | 0.900 | 1.000 | 2148 | tags=14%, list=11%, signal=15% |
| 25 | BIOCARTA\_ACH\_PATHWAY |  | 15 | 0.33 | 0.85 | 0.672 | 0.869 | 1.000 | 4017 | tags=27%, list=21%, signal=34% |
| 26 | BIOCARTA\_ARF\_PATHWAY |  | 17 | 0.31 | 0.85 | 0.671 | 0.841 | 1.000 | 8761 | tags=65%, list=45%, signal=117% |
| 27 | BIOCARTA\_CDMAC\_PATHWAY |  | 15 | 0.32 | 0.83 | 0.720 | 0.851 | 1.000 | 2207 | tags=20%, list=11%, signal=23% |
| 28 | BIOCARTA\_HER2\_PATHWAY |  | 22 | 0.26 | 0.74 | 0.822 | 0.956 | 1.000 | 3065 | tags=14%, list=16%, signal=16% |
| 29 | BIOCARTA\_BARRESTIN\_SRC\_PATHWAY |  | 15 | 0.28 | 0.73 | 0.835 | 0.933 | 1.000 | 4140 | tags=27%, list=21%, signal=34% |
| 30 | BIOCARTA\_PAR1\_PATHWAY |  | 35 | 0.22 | 0.71 | 0.896 | 0.930 | 1.000 | 2148 | tags=11%, list=11%, signal=13% |
| 31 | BIOCARTA\_IL7\_PATHWAY |  | 17 | 0.26 | 0.70 | 0.861 | 0.904 | 1.000 | 750 | tags=12%, list=4%, signal=12% |
| 32 | BIOCARTA\_EDG1\_PATHWAY |  | 24 | 0.23 | 0.69 | 0.888 | 0.892 | 1.000 | 88 | tags=4%, list=0%, signal=4% |
Table: Gene sets enriched in phenotype **bg (3 samples) **[plain text format]****
