# Supplementary material for: Comprehensive analysis of β-catenin target genes in colorectal carcinoma cell lines with deregulated Wnt/β-catenin signaling
Source: BMC Genomics. 2014 Jan 28;15:74. doi: 10.1186/1471-2164-15-74 (PMC3909937; doi:10.1186/1471-2164-15-74)
Supplement: Additional file 4 — GSEA analysis using the Biocarta pathway database. This zipped file contains confirming data of the GSEA analysis. The names of the directories containing the files were composed of the term ‘GSEA’, the name of the cell line, e.g. DLD1, SW480, or LS174T, and the pathway database (Biocarta). Please use a web browser to view the files with the name ‘index.html’ in the corresponding directories to start exploring the data. [file 1471-2164-15-74-S4.zip › SW480_Biocarta/heat_map_corr_plot.html]

Heat map and correlation plot for SW480\_collapsed\_to\_symbols  

Fig 1: heat\_map      
 Heat Map of the top 50 features for each phenotype in SW480\_collapsed\_to\_symbols

  
  

Fig 2: Ranked Gene List Correlation Profile      
 Ranked list correlations for SW480\_collapsed\_to\_symbols

  
  
    
