# Supplementary material for: Comprehensive analysis of β-catenin target genes in colorectal carcinoma cell lines with deregulated Wnt/β-catenin signaling
Source: BMC Genomics. 2014 Jan 28;15:74. doi: 10.1186/1471-2164-15-74 (PMC3909937; doi:10.1186/1471-2164-15-74)
Supplement: Additional file 5 — GSEA analysis using the KEGG pathway database. This zipped file contains confirming data of the GSEA analysis. The names of the directories containing the files were composed of the term ‘GSEA’, the name of the cell line, e.g. DLD1, SW480, or LS174T, and the pathway database (KEGG). Please use a web browser to view the files with the name ‘index.html’ in the corresponding directories to start exploring the data. [file 1471-2164-15-74-S5.zip › GSEA KEGG SW480/KEGG_SYSTEMIC_LUPUS_ERYTHEMATOSUS.html]

Details for gene set KEGG\_SYSTEMIC\_LUPUS\_ERYTHEMATOSUS[GSEA]

|  || Dataset | SW480\_collapsed\_to\_symbols.class.cls#b\_versus\_bg.class.cls#b\_versus\_bg\_repos |
| Phenotype | class.cls#b\_versus\_bg\_repos |
| Upregulated in class | 0 |
| GeneSet | KEGG\_SYSTEMIC\_LUPUS\_ERYTHEMATOSUS |
| Enrichment Score (ES) | -0.5759725 |
| Normalized Enrichment Score (NES) | -2.2491896 |
| Nominal p-value | 0.0 |
| FDR q-value | 0.0 |
| FWER p-Value | 0.0 |
Table: GSEA Results Summary

  

Fig 1: Enrichment plot: KEGG\_SYSTEMIC\_LUPUS\_ERYTHEMATOSUS      
 Profile of the Running ES Score & Positions of GeneSet Members on the Rank Ordered List

  

| PROBE | GENE SYMBOL | GENE\_TITLE | RANK IN GENE LIST | RANK METRIC SCORE | RUNNING ES | CORE ENRICHMENT || 1 | TRIM21 | TRIM21 Entrez,  Source | tripartite motif-containing 21 | 639 | 0.243 | -0.0163 | No |
| 2 | FCGR2A | FCGR2A Entrez,  Source | Fc fragment of IgG, low affinity IIa, receptor (CD32) | 1166 | 0.172 | -0.0316 | No |
| 3 | C5 | C5 Entrez,  Source | complement component 5 | 1313 | 0.161 | -0.0281 | No |
| 4 | HIST1H3A | HIST1H3A Entrez,  Source | histone cluster 1, H3a | 1716 | 0.133 | -0.0397 | No |
| 5 | HLA-DMA | HLA-DMA Entrez,  Source | major histocompatibility complex, class II, DM alpha | 2247 | 0.110 | -0.0595 | No |
| 6 | H2AFV | H2AFV Entrez,  Source | H2A histone family, member V | 2590 | 0.096 | -0.0705 | No |
| 7 | TROVE2 | TROVE2 Entrez,  Source | TROVE domain family, member 2 | 2738 | 0.091 | -0.0719 | No |
| 8 | ACTN1 | ACTN1 Entrez,  Source | actinin, alpha 1 | 2936 | 0.085 | -0.0763 | No |
| 9 | HLA-DRB4 | HLA-DRB4 Entrez,  Source | major histocompatibility complex, class II, DR beta 4 | 3022 | 0.082 | -0.0751 | No |
| 10 | HIST1H3I | HIST1H3I Entrez,  Source | histone cluster 1, H3i | 3176 | 0.077 | -0.0777 | No |
| 11 | HIST1H2BA | HIST1H2BA Entrez,  Source | histone cluster 1, H2ba | 3299 | 0.073 | -0.0789 | No |
| 12 | HLA-DRA | HLA-DRA Entrez,  Source | major histocompatibility complex, class II, DR alpha | 3456 | 0.069 | -0.0823 | No |
| 13 | H2AFY2 | H2AFY2 Entrez,  Source | H2A histone family, member Y2 | 4178 | 0.052 | -0.1158 | No |
| 14 | H2AFY | H2AFY Entrez,  Source | H2A histone family, member Y | 4431 | 0.046 | -0.1256 | No |
| 15 | H2AFJ | H2AFJ Entrez,  Source | H2A histone family, member J | 4757 | 0.040 | -0.1396 | No |
| 16 | SNRPD1 | SNRPD1 Entrez,  Source | small nuclear ribonucleoprotein D1 polypeptide 16kDa | 5496 | 0.027 | -0.1756 | No |
| 17 | H2AFZ | H2AFZ Entrez,  Source | H2A histone family, member Z | 5650 | 0.025 | -0.1818 | No |
| 18 | HIST3H3 | HIST3H3 Entrez,  Source | histone cluster 3, H3 | 5997 | 0.020 | -0.1983 | No |
| 19 | CD86 | CD86 Entrez,  Source | CD86 molecule | 6293 | 0.015 | -0.2124 | No |
| 20 | H3F3B | H3F3B Entrez,  Source | H3 histone, family 3B (H3.3B) | 6646 | 0.011 | -0.2298 | No |
| 21 | HIST1H3B | HIST1H3B Entrez,  Source | histone cluster 1, H3b | 6671 | 0.010 | -0.2303 | No |
| 22 | IFNG | IFNG Entrez,  Source | interferon, gamma | 6964 | 0.006 | -0.2449 | No |
| 23 | HLA-DPA1 | HLA-DPA1 Entrez,  Source | major histocompatibility complex, class II, DP alpha 1 | 7007 | 0.006 | -0.2467 | No |
| 24 | HIST1H3F | HIST1H3F Entrez,  Source | histone cluster 1, H3f | 7540 | -0.001 | -0.2739 | No |
| 25 | H2AFX | H2AFX Entrez,  Source | H2A histone family, member X | 7639 | -0.002 | -0.2788 | No |
| 26 | C1QA | C1QA Entrez,  Source | complement component 1, q subcomponent, A chain | 8440 | -0.012 | -0.3191 | No |
| 27 | FCGR2B | FCGR2B Entrez,  Source | Fc fragment of IgG, low affinity IIb, receptor (CD32) | 8608 | -0.014 | -0.3267 | No |
| 28 | SNRPB | SNRPB Entrez,  Source | small nuclear ribonucleoprotein polypeptides B and B1 | 8772 | -0.016 | -0.3340 | No |
| 29 | HLA-DQA1 | HLA-DQA1 Entrez,  Source | major histocompatibility complex, class II, DQ alpha 1 | 8795 | -0.016 | -0.3341 | No |
| 30 | HIST1H3G | HIST1H3G Entrez,  Source | histone cluster 1, H3g | 8979 | -0.018 | -0.3422 | No |
| 31 | SNRPD3 | SNRPD3 Entrez,  Source | small nuclear ribonucleoprotein D3 polypeptide 18kDa | 9103 | -0.020 | -0.3472 | No |
| 32 | ACTN4 | ACTN4 Entrez,  Source | actinin, alpha 4 | 9236 | -0.021 | -0.3525 | No |
| 33 | CD28 | CD28 Entrez,  Source | CD28 molecule | 9542 | -0.025 | -0.3665 | No |
| 34 | C9 | C9 Entrez,  Source | complement component 9 | 10382 | -0.034 | -0.4073 | No |
| 35 | SSB | SSB Entrez,  Source | Sjogren syndrome antigen B (autoantigen La) | 10689 | -0.038 | -0.4204 | No |
| 36 | HLA-DOA | HLA-DOA Entrez,  Source | major histocompatibility complex, class II, DO alpha | 11429 | -0.047 | -0.4552 | No |
| 37 | C7 | C7 Entrez,  Source | complement component 7 | 12129 | -0.056 | -0.4873 | No |
| 38 | FCGR2C | FCGR2C Entrez,  Source | Fc fragment of IgG, low affinity IIb, receptor for (CD32) | 12900 | -0.065 | -0.5225 | No |
| 39 | HIST1H4A | HIST1H4A Entrez,  Source | histone cluster 1, H4a | 12918 | -0.066 | -0.5189 | No |
| 40 | CD40 | CD40 Entrez,  Source | CD40 molecule, TNF receptor superfamily member 5 | 12929 | -0.066 | -0.5149 | No |
| 41 | C1QC | C1QC Entrez,  Source | complement component 1, q subcomponent, C chain | 12967 | -0.066 | -0.5123 | No |
| 42 | C8A | C8A Entrez,  Source | complement component 8, alpha polypeptide | 12992 | -0.066 | -0.5090 | No |
| 43 | ACTN2 | ACTN2 Entrez,  Source | actinin, alpha 2 | 13118 | -0.068 | -0.5108 | No |
| 44 | ACTN3 | ACTN3 Entrez,  Source | actinin, alpha 3 | 13270 | -0.070 | -0.5138 | No |
| 45 | HIST1H4G | HIST1H4G Entrez,  Source | histone cluster 1, H4g | 13861 | -0.078 | -0.5388 | No |
| 46 | FCGR1A | FCGR1A Entrez,  Source | Fc fragment of IgG, high affinity Ia, receptor (CD64) | 13902 | -0.078 | -0.5355 | No |
| 47 | HIST1H4C | HIST1H4C Entrez,  Source | histone cluster 1, H4c | 14101 | -0.081 | -0.5402 | No |
| 48 | HIST1H4F | HIST1H4F Entrez,  Source | histone cluster 1, H4f | 14106 | -0.081 | -0.5349 | No |
| 49 | GRIN2A | GRIN2A Entrez,  Source | glutamate receptor, ionotropic, N-methyl D-aspartate 2A | 14179 | -0.082 | -0.5330 | No |
| 50 | HIST1H4E | HIST1H4E Entrez,  Source | histone cluster 1, H4e | 14249 | -0.083 | -0.5309 | No |
| 51 | HIST2H2AA3 | HIST2H2AA3 Entrez,  Source | histone cluster 2, H2aa3 | 14434 | -0.085 | -0.5345 | No |
| 52 | C8B | C8B Entrez,  Source | complement component 8, beta polypeptide | 14479 | -0.086 | -0.5309 | No |
| 53 | HIST2H2BE | HIST2H2BE Entrez,  Source | histone cluster 2, H2be | 14596 | -0.088 | -0.5309 | No |
| 54 | CD40LG | CD40LG Entrez,  Source | CD40 ligand (TNF superfamily, member 5, hyper-IgM syndrome) | 14658 | -0.088 | -0.5280 | No |
| 55 | H2BFM | H2BFM Entrez,  Source | H2B histone family, member M | 15196 | -0.097 | -0.5490 | No |
| 56 | HLA-DPB1 | HLA-DPB1 Entrez,  Source | major histocompatibility complex, class II, DP beta 1 | 15437 | -0.101 | -0.5545 | No |
| 57 | HIST1H3C | HIST1H3C Entrez,  Source | histone cluster 1, H3c | 15625 | -0.104 | -0.5570 | No |
| 58 | C2 | C2 Entrez,  Source | complement component 2 | 15766 | -0.107 | -0.5569 | No |
| 59 | C8G | C8G Entrez,  Source | complement component 8, gamma polypeptide | 16138 | -0.114 | -0.5682 | Yes |
| 60 | HLA-DRB1 | HLA-DRB1 Entrez,  Source | major histocompatibility complex, class II, DR beta 1 | 16236 | -0.116 | -0.5653 | Yes |
| 61 | C6 | C6 Entrez,  Source | complement component 6 | 16259 | -0.116 | -0.5585 | Yes |
| 62 | HIST1H2AM | HIST1H2AM Entrez,  Source | histone cluster 1, H2am | 16380 | -0.119 | -0.5566 | Yes |
| 63 | C1QB | C1QB Entrez,  Source | complement component 1, q subcomponent, B chain | 16705 | -0.125 | -0.5647 | Yes |
| 64 | CD80 | CD80 Entrez,  Source | CD80 molecule | 16726 | -0.126 | -0.5572 | Yes |
| 65 | FCGR3B | FCGR3B Entrez,  Source | Fc fragment of IgG, low affinity IIIb, receptor (CD16b) | 16930 | -0.131 | -0.5587 | Yes |
| 66 | HIST1H4L | HIST1H4L Entrez,  Source | histone cluster 1, H4l | 16974 | -0.132 | -0.5519 | Yes |
| 67 | HLA-DMB | HLA-DMB Entrez,  Source | major histocompatibility complex, class II, DM beta | 17061 | -0.134 | -0.5472 | Yes |
| 68 | HIST1H3J | HIST1H3J Entrez,  Source | histone cluster 1, H3j | 17113 | -0.136 | -0.5405 | Yes |
| 69 | HIST1H2AB | HIST1H2AB Entrez,  Source | histone cluster 1, H2ab | 17150 | -0.137 | -0.5331 | Yes |
| 70 | CTSG | CTSG Entrez,  Source | cathepsin G | 17229 | -0.140 | -0.5275 | Yes |
| 71 | HIST1H2BL | HIST1H2BL Entrez,  Source | histone cluster 1, H2bl | 17295 | -0.142 | -0.5212 | Yes |
| 72 | TNF | TNF Entrez,  Source | tumor necrosis factor (TNF superfamily, member 2) | 17349 | -0.143 | -0.5142 | Yes |
| 73 | HIST1H2AG | HIST1H2AG Entrez,  Source | histone cluster 1, H2ag | 17755 | -0.156 | -0.5244 | Yes |
| 74 | HLA-DQB1 | HLA-DQB1 Entrez,  Source | major histocompatibility complex, class II, DQ beta 1 | 17788 | -0.158 | -0.5152 | Yes |
| 75 | HIST1H3E | HIST1H3E Entrez,  Source | histone cluster 1, H3e | 17830 | -0.160 | -0.5064 | Yes |
| 76 | HIST1H2AL | HIST1H2AL Entrez,  Source | histone cluster 1, H2al | 18279 | -0.180 | -0.5172 | Yes |
| 77 | HLA-DOB | HLA-DOB Entrez,  Source | major histocompatibility complex, class II, DO beta | 18287 | -0.181 | -0.5053 | Yes |
| 78 | HIST1H2BN | HIST1H2BN Entrez,  Source | histone cluster 1, H2bn | 18304 | -0.181 | -0.4938 | Yes |
| 79 | HIST1H2AJ | HIST1H2AJ Entrez,  Source | histone cluster 1, H2aj | 18403 | -0.187 | -0.4860 | Yes |
| 80 | GRIN2B | GRIN2B Entrez,  Source | glutamate receptor, ionotropic, N-methyl D-aspartate 2B | 18602 | -0.200 | -0.4826 | Yes |
| 81 | HIST1H2BK | HIST1H2BK Entrez,  Source | histone cluster 1, H2bk | 18699 | -0.208 | -0.4733 | Yes |
| 82 | HIST1H2AI | HIST1H2AI Entrez,  Source | histone cluster 1, H2ai | 18772 | -0.214 | -0.4625 | Yes |
| 83 | HIST1H2BM | HIST1H2BM Entrez,  Source | histone cluster 1, H2bm | 18823 | -0.220 | -0.4500 | Yes |
| 84 | HIST1H2BD | HIST1H2BD Entrez,  Source | histone cluster 1, H2bd | 18886 | -0.227 | -0.4377 | Yes |
| 85 | HIST1H4B | HIST1H4B Entrez,  Source | histone cluster 1, H4b | 18947 | -0.237 | -0.4247 | Yes |
| 86 | C1S | C1S Entrez,  Source | complement component 1, s subcomponent | 19058 | -0.253 | -0.4131 | Yes |
| 87 | HIST3H2A | HIST3H2A Entrez,  Source | histone cluster 3, H2a | 19088 | -0.258 | -0.3970 | Yes |
| 88 | HIST1H2BF | HIST1H2BF Entrez,  Source | histone cluster 1, H2bf | 19132 | -0.269 | -0.3809 | Yes |
| 89 | HIST1H2BB | HIST1H2BB Entrez,  Source | histone cluster 1, H2bb | 19185 | -0.281 | -0.3645 | Yes |
| 90 | HIST1H2BG | HIST1H2BG Entrez,  Source | histone cluster 1, H2bg | 19206 | -0.289 | -0.3458 | Yes |
| 91 | HIST1H2BE | HIST1H2BE Entrez,  Source | histone cluster 1, H2be | 19236 | -0.299 | -0.3269 | Yes |
| 92 | HIST1H2AE | HIST1H2AE Entrez,  Source | histone cluster 1, H2ae | 19261 | -0.308 | -0.3072 | Yes |
| 93 | HIST1H2BC | HIST1H2BC Entrez,  Source | histone cluster 1, H2bc | 19278 | -0.317 | -0.2864 | Yes |
| 94 | HIST1H2BH | HIST1H2BH Entrez,  Source | histone cluster 1, H2bh | 19283 | -0.318 | -0.2650 | Yes |
| 95 | HIST1H2BI | HIST1H2BI Entrez,  Source | histone cluster 1, H2bi | 19293 | -0.323 | -0.2434 | Yes |
| 96 | HIST1H2AC | HIST1H2AC Entrez,  Source | histone cluster 1, H2ac | 19378 | -0.382 | -0.2217 | Yes |
| 97 | HIST1H4D | HIST1H4D Entrez,  Source | histone cluster 1, H4d | 19379 | -0.382 | -0.1957 | Yes |
| 98 | HIST1H2AK | HIST1H2AK Entrez,  Source | histone cluster 1, H2ak | 19381 | -0.384 | -0.1695 | Yes |
| 99 | IL10 | IL10 Entrez,  Source | interleukin 10 | 19415 | -0.420 | -0.1426 | Yes |
| 100 | HIST1H2BO | HIST1H2BO Entrez,  Source | histone cluster 1, H2bo | 19462 | -0.479 | -0.1124 | Yes |
| 101 | HIST1H2BJ | HIST1H2BJ Entrez,  Source | histone cluster 1, H2bj | 19480 | -0.510 | -0.0785 | Yes |
| 102 | HIST1H3H | HIST1H3H Entrez,  Source | histone cluster 1, H3h | 19501 | -0.572 | -0.0405 | Yes |
| 103 | HIST1H4H | HIST1H4H Entrez,  Source | histone cluster 1, H4h | 19520 | -0.635 | 0.0019 | Yes |
Table: GSEA details [plain text format]

  

Fig 2: KEGG\_SYSTEMIC\_LUPUS\_ERYTHEMATOSUS      
 Blue-Pink O' Gram in the Space of the Analyzed GeneSet

  

Fig 3: KEGG\_SYSTEMIC\_LUPUS\_ERYTHEMATOSUS: Random ES distribution      
 Gene set null distribution of ES for **KEGG\_SYSTEMIC\_LUPUS\_ERYTHEMATOSUS**
