# Supplementary material for: Comprehensive analysis of β-catenin target genes in colorectal carcinoma cell lines with deregulated Wnt/β-catenin signaling
Source: BMC Genomics. 2014 Jan 28;15:74. doi: 10.1186/1471-2164-15-74 (PMC3909937; doi:10.1186/1471-2164-15-74)
Supplement: Additional file 5 — GSEA analysis using the KEGG pathway database. This zipped file contains confirming data of the GSEA analysis. The names of the directories containing the files were composed of the term ‘GSEA’, the name of the cell line, e.g. DLD1, SW480, or LS174T, and the pathway database (KEGG). Please use a web browser to view the files with the name ‘index.html’ in the corresponding directories to start exploring the data. [file 1471-2164-15-74-S5.zip › GSEA KEGG SW480/KEGG_UBIQUITIN_MEDIATED_PROTEOLYSIS.html]

Details for gene set KEGG\_UBIQUITIN\_MEDIATED\_PROTEOLYSIS[GSEA]

|  || Dataset | SW480\_collapsed\_to\_symbols.class.cls#b\_versus\_bg.class.cls#b\_versus\_bg\_repos |
| Phenotype | class.cls#b\_versus\_bg\_repos |
| Upregulated in class | 1 |
| GeneSet | KEGG\_UBIQUITIN\_MEDIATED\_PROTEOLYSIS |
| Enrichment Score (ES) | 0.39959145 |
| Normalized Enrichment Score (NES) | 1.6727526 |
| Nominal p-value | 0.0 |
| FDR q-value | 0.06160734 |
| FWER p-Value | 0.459 |
Table: GSEA Results Summary

  

Fig 1: Enrichment plot: KEGG\_UBIQUITIN\_MEDIATED\_PROTEOLYSIS      
 Profile of the Running ES Score & Positions of GeneSet Members on the Rank Ordered List

  

| PROBE | GENE SYMBOL | GENE\_TITLE | RANK IN GENE LIST | RANK METRIC SCORE | RUNNING ES | CORE ENRICHMENT || 1 | UBE2L6 | UBE2L6 Entrez,  Source | ubiquitin-conjugating enzyme E2L 6 | 335 | 0.324 | 0.0199 | Yes |
| 2 | HERC3 | HERC3 Entrez,  Source | hect domain and RLD 3 | 362 | 0.317 | 0.0549 | Yes |
| 3 | FBXO4 | FBXO4 Entrez,  Source | F-box protein 4 | 453 | 0.289 | 0.0833 | Yes |
| 4 | BIRC3 | BIRC3 Entrez,  Source | baculoviral IAP repeat-containing 3 | 455 | 0.289 | 0.1164 | Yes |
| 5 | PIAS3 | PIAS3 Entrez,  Source | protein inhibitor of activated STAT, 3 | 476 | 0.283 | 0.1478 | Yes |
| 6 | BIRC2 | BIRC2 Entrez,  Source | baculoviral IAP repeat-containing 2 | 572 | 0.255 | 0.1721 | Yes |
| 7 | DDB2 | DDB2 Entrez,  Source | damage-specific DNA binding protein 2, 48kDa | 737 | 0.226 | 0.1896 | Yes |
| 8 | MID1 | MID1 Entrez,  Source | midline 1 (Opitz/BBB syndrome) | 879 | 0.205 | 0.2058 | Yes |
| 9 | FBXO2 | FBXO2 Entrez,  Source | F-box protein 2 | 906 | 0.202 | 0.2276 | Yes |
| 10 | NEDD4 | NEDD4 Entrez,  Source | neural precursor cell expressed, developmentally down-regulated 4 | 1057 | 0.184 | 0.2409 | Yes |
| 11 | KLHL13 | KLHL13 Entrez,  Source | kelch-like 13 (Drosophila) | 1357 | 0.158 | 0.2436 | Yes |
| 12 | NEDD4L | NEDD4L Entrez,  Source | neural precursor cell expressed, developmentally down-regulated 4-like | 1500 | 0.147 | 0.2532 | Yes |
| 13 | PIAS1 | PIAS1 Entrez,  Source | protein inhibitor of activated STAT, 1 | 1526 | 0.146 | 0.2686 | Yes |
| 14 | UBE2C | UBE2C Entrez,  Source | ubiquitin-conjugating enzyme E2C | 1787 | 0.130 | 0.2702 | Yes |
| 15 | CDC27 | CDC27 Entrez,  Source | cell division cycle 27 | 1988 | 0.121 | 0.2738 | Yes |
| 16 | UBE3B | UBE3B Entrez,  Source | ubiquitin protein ligase E3B | 2102 | 0.116 | 0.2812 | Yes |
| 17 | TRIM32 | TRIM32 Entrez,  Source | tripartite motif-containing 32 | 2129 | 0.115 | 0.2930 | Yes |
| 18 | FANCL | FANCL Entrez,  Source | Fanconi anemia, complementation group L | 2169 | 0.113 | 0.3040 | Yes |
| 19 | UBE2E2 | UBE2E2 Entrez,  Source | ubiquitin-conjugating enzyme E2E 2 (UBC4/5 homolog, yeast) | 2294 | 0.107 | 0.3099 | Yes |
| 20 | UBE2E3 | UBE2E3 Entrez,  Source | ubiquitin-conjugating enzyme E2E 3 (UBC4/5 homolog, yeast) | 2304 | 0.107 | 0.3217 | Yes |
| 21 | BRCA1 | BRCA1 Entrez,  Source | breast cancer 1, early onset | 2598 | 0.095 | 0.3176 | Yes |
| 22 | HERC1 | HERC1 Entrez,  Source | hect (homologous to the E6-AP (UBE3A) carboxyl terminus) domain and RCC1 (CHC1)-like domain (RLD) 1 | 2736 | 0.091 | 0.3209 | Yes |
| 23 | HERC4 | HERC4 Entrez,  Source | hect domain and RLD 4 | 2834 | 0.087 | 0.3259 | Yes |
| 24 | DET1 | DET1 Entrez,  Source | de-etiolated homolog 1 (Arabidopsis) | 2950 | 0.084 | 0.3296 | Yes |
| 25 | CUL5 | CUL5 Entrez,  Source | cullin 5 | 2982 | 0.083 | 0.3375 | Yes |
| 26 | SMURF2 | SMURF2 Entrez,  Source | SMAD specific E3 ubiquitin protein ligase 2 | 3018 | 0.082 | 0.3451 | Yes |
| 27 | UBE2W | UBE2W Entrez,  Source | ubiquitin-conjugating enzyme E2W (putative) | 3172 | 0.077 | 0.3461 | Yes |
| 28 | MAP3K1 | MAP3K1 Entrez,  Source | mitogen-activated protein kinase kinase kinase 1 | 3181 | 0.077 | 0.3545 | Yes |
| 29 | CUL4B | CUL4B Entrez,  Source | cullin 4B | 3217 | 0.076 | 0.3613 | Yes |
| 30 | TCEB2 | TCEB2 Entrez,  Source | transcription elongation factor B (SIII), polypeptide 2 (18kDa, elongin B) | 3320 | 0.073 | 0.3644 | Yes |
| 31 | HERC2 | HERC2 Entrez,  Source | hect domain and RLD 2 | 3349 | 0.072 | 0.3712 | Yes |
| 32 | CDC20 | CDC20 Entrez,  Source | CDC20 cell division cycle 20 homolog (S. cerevisiae) | 3374 | 0.072 | 0.3782 | Yes |
| 33 | ITCH | ITCH Entrez,  Source | itchy homolog E3 ubiquitin protein ligase (mouse) | 3377 | 0.072 | 0.3863 | Yes |
| 34 | RFWD2 | RFWD2 Entrez,  Source | ring finger and WD repeat domain 2 | 3465 | 0.069 | 0.3897 | Yes |
| 35 | PML | PML Entrez,  Source | promyelocytic leukemia | 3501 | 0.068 | 0.3957 | Yes |
| 36 | UBE2H | UBE2H Entrez,  Source | ubiquitin-conjugating enzyme E2H (UBC8 homolog, yeast) | 3572 | 0.066 | 0.3996 | Yes |
| 37 | WWP2 | WWP2 Entrez,  Source | WW domain containing E3 ubiquitin protein ligase 2 | 3776 | 0.061 | 0.3961 | No |
| 38 | KLHL9 | KLHL9 Entrez,  Source | kelch-like 9 (Drosophila) | 3920 | 0.058 | 0.3953 | No |
| 39 | ANAPC11 | ANAPC11 Entrez,  Source | APC11 anaphase promoting complex subunit 11 homolog (yeast) | 4281 | 0.049 | 0.3825 | No |
| 40 | UBE2E1 | UBE2E1 Entrez,  Source | ubiquitin-conjugating enzyme E2E 1 (UBC4/5 homolog, yeast) | 4476 | 0.045 | 0.3777 | No |
| 41 | MDM2 | MDM2 Entrez,  Source | Mdm2, transformed 3T3 cell double minute 2, p53 binding protein (mouse) | 4876 | 0.038 | 0.3615 | No |
| 42 | FBXW7 | FBXW7 Entrez,  Source | F-box and WD-40 domain protein 7 (archipelago homolog, Drosophila) | 4888 | 0.038 | 0.3652 | No |
| 43 | RHOBTB2 | RHOBTB2 Entrez,  Source | Rho-related BTB domain containing 2 | 4919 | 0.037 | 0.3679 | No |
| 44 | BIRC6 | BIRC6 Entrez,  Source | baculoviral IAP repeat-containing 6 (apollon) | 5334 | 0.030 | 0.3500 | No |
| 45 | UBE4B | UBE4B Entrez,  Source | ubiquitination factor E4B (UFD2 homolog, yeast) | 5361 | 0.029 | 0.3520 | No |
| 46 | UBE2Z | UBE2Z Entrez,  Source | ubiquitin-conjugating enzyme E2Z (putative) | 5399 | 0.029 | 0.3534 | No |
| 47 | UBE2I | UBE2I Entrez,  Source | ubiquitin-conjugating enzyme E2I (UBC9 homolog, yeast) | 5643 | 0.025 | 0.3437 | No |
| 48 | RNF7 | RNF7 Entrez,  Source | ring finger protein 7 | 5837 | 0.022 | 0.3363 | No |
| 49 | ANAPC5 | ANAPC5 Entrez,  Source | anaphase promoting complex subunit 5 | 5975 | 0.020 | 0.3315 | No |
| 50 | CDC16 | CDC16 Entrez,  Source | CDC16 cell division cycle 16 homolog (S. cerevisiae) | 6091 | 0.018 | 0.3277 | No |
| 51 | VHL | VHL Entrez,  Source | von Hippel-Lindau tumor suppressor | 6321 | 0.015 | 0.3176 | No |
| 52 | ANAPC10 | ANAPC10 Entrez,  Source | anaphase promoting complex subunit 10 | 6603 | 0.011 | 0.3044 | No |
| 53 | UBA2 | UBA2 Entrez,  Source | - | 6611 | 0.011 | 0.3053 | No |
| 54 | PIAS2 | PIAS2 Entrez,  Source | protein inhibitor of activated STAT, 2 | 6637 | 0.011 | 0.3052 | No |
| 55 | UBE2L3 | UBE2L3 Entrez,  Source | ubiquitin-conjugating enzyme E2L 3 | 6808 | 0.008 | 0.2975 | No |
| 56 | FZR1 | FZR1 Entrez,  Source | fizzy/cell division cycle 20 related 1 (Drosophila) | 6911 | 0.007 | 0.2930 | No |
| 57 | UBE2R2 | UBE2R2 Entrez,  Source | ubiquitin-conjugating enzyme E2R 2 | 6923 | 0.007 | 0.2932 | No |
| 58 | RBX1 | RBX1 Entrez,  Source | ring-box 1 | 7189 | 0.003 | 0.2800 | No |
| 59 | PRPF19 | PRPF19 Entrez,  Source | PRP19/PSO4 pre-mRNA processing factor 19 homolog (S. cerevisiae) | 7221 | 0.003 | 0.2787 | No |
| 60 | SMURF1 | SMURF1 Entrez,  Source | SMAD specific E3 ubiquitin protein ligase 1 | 7246 | 0.002 | 0.2777 | No |
| 61 | SIAH1 | SIAH1 Entrez,  Source | seven in absentia homolog 1 (Drosophila) | 7249 | 0.002 | 0.2778 | No |
| 62 | CUL7 | CUL7 Entrez,  Source | cullin 7 | 7569 | -0.001 | 0.2616 | No |
| 63 | UBE2D3 | UBE2D3 Entrez,  Source | ubiquitin-conjugating enzyme E2D 3 (UBC4/5 homolog, yeast) | 7628 | -0.002 | 0.2589 | No |
| 64 | HUWE1 | HUWE1 Entrez,  Source | HECT, UBA and WWE domain containing 1 | 7674 | -0.003 | 0.2569 | No |
| 65 | UBE2D1 | UBE2D1 Entrez,  Source | ubiquitin-conjugating enzyme E2D 1 (UBC4/5 homolog, yeast) | 7782 | -0.004 | 0.2519 | No |
| 66 | TRIM37 | TRIM37 Entrez,  Source | tripartite motif-containing 37 | 7790 | -0.004 | 0.2520 | No |
| 67 | SOCS3 | SOCS3 Entrez,  Source | suppressor of cytokine signaling 3 | 8055 | -0.007 | 0.2393 | No |
| 68 | UBE2D4 | UBE2D4 Entrez,  Source | ubiquitin-conjugating enzyme E2D 4 (putative) | 8167 | -0.009 | 0.2346 | No |
| 69 | TCEB1 | TCEB1 Entrez,  Source | transcription elongation factor B (SIII), polypeptide 1 (15kDa, elongin C) | 8179 | -0.009 | 0.2351 | No |
| 70 | SAE1 | SAE1 Entrez,  Source | - | 8234 | -0.010 | 0.2334 | No |
| 71 | UBE3A | UBE3A Entrez,  Source | ubiquitin protein ligase E3A (human papilloma virus E6-associated protein, Angelman syndrome) | 8359 | -0.011 | 0.2283 | No |
| 72 | UBE2A | UBE2A Entrez,  Source | ubiquitin-conjugating enzyme E2A (RAD6 homolog) | 8398 | -0.012 | 0.2276 | No |
| 73 | CUL3 | CUL3 Entrez,  Source | cullin 3 | 8441 | -0.012 | 0.2269 | No |
| 74 | STUB1 | STUB1 Entrez,  Source | STIP1 homology and U-box containing protein 1 | 8469 | -0.012 | 0.2269 | No |
| 75 | UBE2G2 | UBE2G2 Entrez,  Source | ubiquitin-conjugating enzyme E2G 2 (UBC7 homolog, yeast) | 8690 | -0.015 | 0.2173 | No |
| 76 | KEAP1 | KEAP1 Entrez,  Source | kelch-like ECH-associated protein 1 | 8726 | -0.015 | 0.2172 | No |
| 77 | CUL2 | CUL2 Entrez,  Source | cullin 2 | 8858 | -0.017 | 0.2124 | No |
| 78 | PPIL2 | PPIL2 Entrez,  Source | peptidylprolyl isomerase (cyclophilin)-like 2 | 8866 | -0.017 | 0.2140 | No |
| 79 | UBE2B | UBE2B Entrez,  Source | ubiquitin-conjugating enzyme E2B (RAD6 homolog) | 8903 | -0.017 | 0.2142 | No |
| 80 | UBE2Q2 | UBE2Q2 Entrez,  Source | ubiquitin-conjugating enzyme E2Q (putative) 2 | 9116 | -0.020 | 0.2055 | No |
| 81 | UBE2G1 | UBE2G1 Entrez,  Source | ubiquitin-conjugating enzyme E2G 1 (UBC7 homolog, yeast) | 9246 | -0.021 | 0.2014 | No |
| 82 | UBE2J2 | UBE2J2 Entrez,  Source | ubiquitin-conjugating enzyme E2, J2 (UBC6 homolog, yeast) | 9347 | -0.023 | 0.1988 | No |
| 83 | UBE4A | UBE4A Entrez,  Source | ubiquitination factor E4A (UFD2 homolog, yeast) | 9394 | -0.023 | 0.1991 | No |
| 84 | FBXW11 | FBXW11 Entrez,  Source | F-box and WD-40 domain protein 11 | 9412 | -0.023 | 0.2008 | No |
| 85 | CBL | CBL Entrez,  Source | Cas-Br-M (murine) ecotropic retroviral transforming sequence | 9608 | -0.026 | 0.1937 | No |
| 86 | TRIP12 | TRIP12 Entrez,  Source | thyroid hormone receptor interactor 12 | 9949 | -0.029 | 0.1796 | No |
| 87 | MGRN1 | MGRN1 Entrez,  Source | mahogunin, ring finger 1 | 10261 | -0.033 | 0.1674 | No |
| 88 | TRAF6 | TRAF6 Entrez,  Source | TNF receptor-associated factor 6 | 10318 | -0.034 | 0.1684 | No |
| 89 | WWP1 | WWP1 Entrez,  Source | WW domain containing E3 ubiquitin protein ligase 1 | 10386 | -0.034 | 0.1689 | No |
| 90 | CBLB | CBLB Entrez,  Source | Cas-Br-M (murine) ecotropic retroviral transforming sequence b | 10489 | -0.036 | 0.1677 | No |
| 91 | ANAPC4 | ANAPC4 Entrez,  Source | anaphase promoting complex subunit 4 | 10605 | -0.037 | 0.1660 | No |
| 92 | UBE2D2 | UBE2D2 Entrez,  Source | ubiquitin-conjugating enzyme E2D 2 (UBC4/5 homolog, yeast) | 10819 | -0.040 | 0.1596 | No |
| 93 | UBE3C | UBE3C Entrez,  Source | ubiquitin protein ligase E3C | 10867 | -0.040 | 0.1618 | No |
| 94 | UBE2NL | UBE2NL Entrez,  Source | ubiquitin-conjugating enzyme E2N-like | 10902 | -0.041 | 0.1648 | No |
| 95 | ERCC8 | ERCC8 Entrez,  Source | excision repair cross-complementing rodent repair deficiency, complementation group 8 | 11127 | -0.044 | 0.1582 | No |
| 96 | UBE2N | UBE2N Entrez,  Source | ubiquitin-conjugating enzyme E2N (UBC13 homolog, yeast) | 11160 | -0.044 | 0.1616 | No |
| 97 | ANAPC2 | ANAPC2 Entrez,  Source | anaphase promoting complex subunit 2 | 11173 | -0.044 | 0.1661 | No |
| 98 | UBE2M | UBE2M Entrez,  Source | ubiquitin-conjugating enzyme E2M (UBC12 homolog, yeast) | 11426 | -0.047 | 0.1585 | No |
| 99 | CDC34 | CDC34 Entrez,  Source | cell division cycle 34 | 11624 | -0.050 | 0.1541 | No |
| 100 | PARK2 | PARK2 Entrez,  Source | Parkinson disease (autosomal recessive, juvenile) 2, parkin | 11856 | -0.052 | 0.1482 | No |
| 101 | UBE2J1 | UBE2J1 Entrez,  Source | ubiquitin-conjugating enzyme E2, J1 (UBC6 homolog, yeast) | 12122 | -0.056 | 0.1410 | No |
| 102 | DDB1 | DDB1 Entrez,  Source | damage-specific DNA binding protein 1, 127kDa | 12270 | -0.057 | 0.1400 | No |
| 103 | CDC23 | CDC23 Entrez,  Source | CDC23 (cell division cycle 23, yeast, homolog) | 12463 | -0.060 | 0.1370 | No |
| 104 | SOCS1 | SOCS1 Entrez,  Source | suppressor of cytokine signaling 1 | 12592 | -0.062 | 0.1374 | No |
| 105 | UBE2Q1 | UBE2Q1 Entrez,  Source | ubiquitin-conjugating enzyme E2Q (putative) 1 | 12647 | -0.062 | 0.1418 | No |
| 106 | ANAPC13 | ANAPC13 Entrez,  Source | anaphase promoting complex subunit 13 | 12996 | -0.067 | 0.1315 | No |
| 107 | CUL4A | CUL4A Entrez,  Source | cullin 4A | 13331 | -0.071 | 0.1224 | No |
| 108 | CBLC | CBLC Entrez,  Source | Cas-Br-M (murine) ecotropic retroviral transforming sequence c | 13387 | -0.072 | 0.1278 | No |
| 109 | ANAPC1 | ANAPC1 Entrez,  Source | anaphase promoting complex subunit 1 | 13650 | -0.075 | 0.1229 | No |
| 110 | PIAS4 | PIAS4 Entrez,  Source | protein inhibitor of activated STAT, 4 | 13734 | -0.076 | 0.1274 | No |
| 111 | UBOX5 | UBOX5 Entrez,  Source | U-box domain containing 5 | 13996 | -0.080 | 0.1230 | No |
| 112 | SYVN1 | SYVN1 Entrez,  Source | synovial apoptosis inhibitor 1, synoviolin | 14056 | -0.080 | 0.1292 | No |
| 113 | ANAPC7 | ANAPC7 Entrez,  Source | anaphase promoting complex subunit 7 | 14799 | -0.091 | 0.1014 | No |
| 114 | CUL1 | CUL1 Entrez,  Source | cullin 1 | 15428 | -0.101 | 0.0806 | No |
| 115 | AIRE | AIRE Entrez,  Source | autoimmune regulator (autoimmune polyendocrinopathy candidiasis ectodermal dystrophy) | 16172 | -0.114 | 0.0555 | No |
| 116 | UBE2O | UBE2O Entrez,  Source | ubiquitin-conjugating enzyme E2O | 16221 | -0.115 | 0.0663 | No |
| 117 | SKP2 | SKP2 Entrez,  Source | S-phase kinase-associated protein 2 (p45) | 17303 | -0.142 | 0.0269 | No |
| 118 | RCHY1 | RCHY1 Entrez,  Source | ring finger and CHY zinc finger domain containing 1 | 17772 | -0.157 | 0.0208 | No |
| 119 | UBE2U | UBE2U Entrez,  Source | ubiquitin-conjugating enzyme E2U (putative) | 18129 | -0.173 | 0.0222 | No |
| 120 | BTRC | BTRC Entrez,  Source | beta-transducin repeat containing | 18818 | -0.219 | 0.0120 | No |
| 121 | FBXW8 | FBXW8 Entrez,  Source | F-box and WD-40 domain protein 8 | 18882 | -0.227 | 0.0347 | No |
Table: GSEA details [plain text format]

  

Fig 2: KEGG\_UBIQUITIN\_MEDIATED\_PROTEOLYSIS      
 Blue-Pink O' Gram in the Space of the Analyzed GeneSet

  

Fig 3: KEGG\_UBIQUITIN\_MEDIATED\_PROTEOLYSIS: Random ES distribution      
 Gene set null distribution of ES for **KEGG\_UBIQUITIN\_MEDIATED\_PROTEOLYSIS**
