# Supplementary material for: Comprehensive analysis of β-catenin target genes in colorectal carcinoma cell lines with deregulated Wnt/β-catenin signaling
Source: BMC Genomics. 2014 Jan 28;15:74. doi: 10.1186/1471-2164-15-74 (PMC3909937; doi:10.1186/1471-2164-15-74)
Supplement: Additional file 5 — GSEA analysis using the KEGG pathway database. This zipped file contains confirming data of the GSEA analysis. The names of the directories containing the files were composed of the term ‘GSEA’, the name of the cell line, e.g. DLD1, SW480, or LS174T, and the pathway database (KEGG). Please use a web browser to view the files with the name ‘index.html’ in the corresponding directories to start exploring the data. [file 1471-2164-15-74-S5.zip › GSEA KEGG SW480/KEGG_WNT_SIGNALING_PATHWAY.html]

Details for gene set KEGG\_WNT\_SIGNALING\_PATHWAY[GSEA]

|  || Dataset | SW480\_collapsed\_to\_symbols.class.cls#b\_versus\_bg.class.cls#b\_versus\_bg\_repos |
| Phenotype | class.cls#b\_versus\_bg\_repos |
| Upregulated in class | 0 |
| GeneSet | KEGG\_WNT\_SIGNALING\_PATHWAY |
| Enrichment Score (ES) | -0.35597807 |
| Normalized Enrichment Score (NES) | -1.4698129 |
| Nominal p-value | 0.0063391444 |
| FDR q-value | 0.20500763 |
| FWER p-Value | 0.961 |
Table: GSEA Results Summary

  

Fig 1: Enrichment plot: KEGG\_WNT\_SIGNALING\_PATHWAY      
 Profile of the Running ES Score & Positions of GeneSet Members on the Rank Ordered List

  

| PROBE | GENE SYMBOL | GENE\_TITLE | RANK IN GENE LIST | RANK METRIC SCORE | RUNNING ES | CORE ENRICHMENT || 1 | RAC2 | RAC2 Entrez,  Source | ras-related C3 botulinum toxin substrate 2 (rho family, small GTP binding protein Rac2) | 119 | 0.477 | 0.0204 | No |
| 2 | FZD1 | FZD1 Entrez,  Source | frizzled homolog 1 (Drosophila) | 128 | 0.470 | 0.0461 | No |
| 3 | DAAM1 | DAAM1 Entrez,  Source | dishevelled associated activator of morphogenesis 1 | 188 | 0.408 | 0.0657 | No |
| 4 | PRKX | PRKX Entrez,  Source | protein kinase, X-linked | 207 | 0.397 | 0.0869 | No |
| 5 | PPP3CA | PPP3CA Entrez,  Source | protein phosphatase 3 (formerly 2B), catalytic subunit, alpha isoform (calcineurin A alpha) | 396 | 0.304 | 0.0941 | No |
| 6 | SFRP5 | SFRP5 Entrez,  Source | secreted frizzled-related protein 5 | 537 | 0.264 | 0.1015 | No |
| 7 | CAMK2D | CAMK2D Entrez,  Source | calcium/calmodulin-dependent protein kinase (CaM kinase) II delta | 744 | 0.225 | 0.1034 | No |
| 8 | FZD7 | FZD7 Entrez,  Source | frizzled homolog 7 (Drosophila) | 1144 | 0.175 | 0.0926 | No |
| 9 | PRICKLE1 | PRICKLE1 Entrez,  Source | prickle homolog 1 (Drosophila) | 1306 | 0.161 | 0.0932 | No |
| 10 | TCF7L2 | TCF7L2 Entrez,  Source | transcription factor 7-like 2 (T-cell specific, HMG-box) | 1570 | 0.143 | 0.0876 | No |
| 11 | PPP2R5A | PPP2R5A Entrez,  Source | protein phosphatase 2, regulatory subunit B (B56), alpha isoform | 1592 | 0.141 | 0.0944 | No |
| 12 | PORCN | PORCN Entrez,  Source | porcupine homolog (Drosophila) | 1670 | 0.136 | 0.0980 | No |
| 13 | JUN | JUN Entrez,  Source | jun oncogene | 1725 | 0.133 | 0.1025 | No |
| 14 | FOSL1 | FOSL1 Entrez,  Source | FOS-like antigen 1 | 1851 | 0.127 | 0.1032 | No |
| 15 | PRKCA | PRKCA Entrez,  Source | protein kinase C, alpha | 1907 | 0.125 | 0.1073 | No |
| 16 | WNT7A | WNT7A Entrez,  Source | wingless-type MMTV integration site family, member 7A | 1917 | 0.125 | 0.1138 | No |
| 17 | WNT9A | WNT9A Entrez,  Source | wingless-type MMTV integration site family, member 9A | 1959 | 0.123 | 0.1185 | No |
| 18 | PPP3CC | PPP3CC Entrez,  Source | protein phosphatase 3 (formerly 2B), catalytic subunit, gamma isoform (calcineurin A gamma) | 1978 | 0.122 | 0.1243 | No |
| 19 | ROCK2 | ROCK2 Entrez,  Source | Rho-associated, coiled-coil containing protein kinase 2 | 2416 | 0.103 | 0.1075 | No |
| 20 | CHP | CHP Entrez,  Source | - | 2515 | 0.099 | 0.1080 | No |
| 21 | DKK2 | DKK2 Entrez,  Source | dickkopf homolog 2 (Xenopus laevis) | 2592 | 0.096 | 0.1094 | No |
| 22 | CCND3 | CCND3 Entrez,  Source | cyclin D3 | 2857 | 0.087 | 0.1006 | No |
| 23 | NFAT5 | NFAT5 Entrez,  Source | nuclear factor of activated T-cells 5, tonicity-responsive | 2987 | 0.083 | 0.0985 | No |
| 24 | VANGL1 | VANGL1 Entrez,  Source | vang-like 1 (van gogh, Drosophila) | 3050 | 0.081 | 0.0999 | No |
| 25 | CAMK2G | CAMK2G Entrez,  Source | calcium/calmodulin-dependent protein kinase (CaM kinase) II gamma | 3255 | 0.075 | 0.0935 | No |
| 26 | RHOA | RHOA Entrez,  Source | ras homolog gene family, member A | 3339 | 0.072 | 0.0932 | No |
| 27 | PPP2R5E | PPP2R5E Entrez,  Source | protein phosphatase 2, regulatory subunit B (B56), epsilon isoform | 3667 | 0.063 | 0.0799 | No |
| 28 | PRKACB | PRKACB Entrez,  Source | protein kinase, cAMP-dependent, catalytic, beta | 3675 | 0.063 | 0.0830 | No |
| 29 | SMAD3 | SMAD3 Entrez,  Source | SMAD, mothers against DPP homolog 3 (Drosophila) | 3719 | 0.062 | 0.0843 | No |
| 30 | FZD2 | FZD2 Entrez,  Source | frizzled homolog 2 (Drosophila) | 3910 | 0.058 | 0.0777 | No |
| 31 | CREBBP | CREBBP Entrez,  Source | CREB binding protein (Rubinstein-Taybi syndrome) | 4299 | 0.049 | 0.0604 | No |
| 32 | MAPK8 | MAPK8 Entrez,  Source | mitogen-activated protein kinase 8 | 4307 | 0.049 | 0.0628 | No |
| 33 | CACYBP | CACYBP Entrez,  Source | calcyclin binding protein | 4450 | 0.046 | 0.0580 | No |
| 34 | PPP2R1A | PPP2R1A Entrez,  Source | protein phosphatase 2 (formerly 2A), regulatory subunit A (PR 65), alpha isoform | 4642 | 0.042 | 0.0505 | No |
| 35 | EP300 | EP300 Entrez,  Source | E1A binding protein p300 | 4663 | 0.042 | 0.0518 | No |
| 36 | NFATC2 | NFATC2 Entrez,  Source | nuclear factor of activated T-cells, cytoplasmic, calcineurin-dependent 2 | 4704 | 0.041 | 0.0520 | No |
| 37 | PPP2R5C | PPP2R5C Entrez,  Source | protein phosphatase 2, regulatory subunit B (B56), gamma isoform | 4777 | 0.040 | 0.0505 | No |
| 38 | DVL2 | DVL2 Entrez,  Source | dishevelled, dsh homolog 2 (Drosophila) | 4810 | 0.039 | 0.0510 | No |
| 39 | MAP3K7 | MAP3K7 Entrez,  Source | mitogen-activated protein kinase kinase kinase 7 | 4884 | 0.038 | 0.0493 | No |
| 40 | CTNNBIP1 | CTNNBIP1 Entrez,  Source | catenin, beta interacting protein 1 | 5191 | 0.032 | 0.0354 | No |
| 41 | TBL1X | TBL1X Entrez,  Source | transducin (beta)-like 1X-linked | 5193 | 0.032 | 0.0371 | No |
| 42 | PPP3CB | PPP3CB Entrez,  Source | protein phosphatase 3 (formerly 2B), catalytic subunit, beta isoform (calcineurin A beta) | 5210 | 0.032 | 0.0380 | No |
| 43 | FZD3 | FZD3 Entrez,  Source | frizzled homolog 3 (Drosophila) | 5233 | 0.031 | 0.0386 | No |
| 44 | LRP5 | LRP5 Entrez,  Source | low density lipoprotein receptor-related protein 5 | 5344 | 0.030 | 0.0346 | No |
| 45 | CHD8 | CHD8 Entrez,  Source | chromodomain helicase DNA binding protein 8 | 5525 | 0.027 | 0.0268 | No |
| 46 | WNT5A | WNT5A Entrez,  Source | wingless-type MMTV integration site family, member 5A | 5570 | 0.026 | 0.0260 | No |
| 47 | PRKCG | PRKCG Entrez,  Source | protein kinase C, gamma | 5651 | 0.025 | 0.0232 | No |
| 48 | CSNK1A1 | CSNK1A1 Entrez,  Source | casein kinase 1, alpha 1 | 5884 | 0.021 | 0.0125 | No |
| 49 | DVL3 | DVL3 Entrez,  Source | dishevelled, dsh homolog 3 (Drosophila) | 5885 | 0.021 | 0.0136 | No |
| 50 | WNT16 | WNT16 Entrez,  Source | wingless-type MMTV integration site family, member 16 | 5995 | 0.020 | 0.0091 | No |
| 51 | TBL1XR1 | TBL1XR1 Entrez,  Source | transducin (beta)-like 1X-linked receptor 1 | 6250 | 0.016 | -0.0031 | No |
| 52 | PPP2R1B | PPP2R1B Entrez,  Source | protein phosphatase 2 (formerly 2A), regulatory subunit A (PR 65), beta isoform | 6348 | 0.014 | -0.0073 | No |
| 53 | NFATC1 | NFATC1 Entrez,  Source | nuclear factor of activated T-cells, cytoplasmic, calcineurin-dependent 1 | 6360 | 0.014 | -0.0071 | No |
| 54 | PPP2R5B | PPP2R5B Entrez,  Source | protein phosphatase 2, regulatory subunit B (B56), beta isoform | 6591 | 0.011 | -0.0183 | No |
| 55 | CXXC4 | CXXC4 Entrez,  Source | CXXC finger 4 | 6664 | 0.010 | -0.0214 | No |
| 56 | GSK3B | GSK3B Entrez,  Source | glycogen synthase kinase 3 beta | 6824 | 0.008 | -0.0292 | No |
| 57 | RBX1 | RBX1 Entrez,  Source | ring-box 1 | 7189 | 0.003 | -0.0477 | No |
| 58 | CTBP1 | CTBP1 Entrez,  Source | C-terminal binding protein 1 | 7197 | 0.003 | -0.0479 | No |
| 59 | SIAH1 | SIAH1 Entrez,  Source | seven in absentia homolog 1 (Drosophila) | 7249 | 0.002 | -0.0504 | No |
| 60 | CCND1 | CCND1 Entrez,  Source | cyclin D1 | 7482 | -0.000 | -0.0624 | No |
| 61 | PPP2CA | PPP2CA Entrez,  Source | protein phosphatase 2 (formerly 2A), catalytic subunit, alpha isoform | 7666 | -0.003 | -0.0716 | No |
| 62 | CTBP2 | CTBP2 Entrez,  Source | C-terminal binding protein 2 | 7961 | -0.006 | -0.0864 | No |
| 63 | WNT10A | WNT10A Entrez,  Source | wingless-type MMTV integration site family, member 10A | 8181 | -0.009 | -0.0972 | No |
| 64 | SMAD2 | SMAD2 Entrez,  Source | SMAD, mothers against DPP homolog 2 (Drosophila) | 8435 | -0.012 | -0.1096 | No |
| 65 | PRKACA | PRKACA Entrez,  Source | protein kinase, cAMP-dependent, catalytic, alpha | 8583 | -0.014 | -0.1164 | No |
| 66 | FZD4 | FZD4 Entrez,  Source | frizzled homolog 4 (Drosophila) | 8655 | -0.014 | -0.1192 | No |
| 67 | MAPK9 | MAPK9 Entrez,  Source | mitogen-activated protein kinase 9 | 8669 | -0.015 | -0.1191 | No |
| 68 | RAC3 | RAC3 Entrez,  Source | ras-related C3 botulinum toxin substrate 3 (rho family, small GTP binding protein Rac3) | 8838 | -0.017 | -0.1268 | No |
| 69 | PLCB3 | PLCB3 Entrez,  Source | phospholipase C, beta 3 (phosphatidylinositol-specific) | 9037 | -0.019 | -0.1360 | No |
| 70 | RAC1 | RAC1 Entrez,  Source | ras-related C3 botulinum toxin substrate 1 (rho family, small GTP binding protein Rac1) | 9406 | -0.023 | -0.1536 | No |
| 71 | FBXW11 | FBXW11 Entrez,  Source | F-box and WD-40 domain protein 11 | 9412 | -0.023 | -0.1526 | No |
| 72 | CSNK2B | CSNK2B Entrez,  Source | casein kinase 2, beta polypeptide | 9523 | -0.025 | -0.1569 | No |
| 73 | WNT3 | WNT3 Entrez,  Source | wingless-type MMTV integration site family, member 3 | 9581 | -0.025 | -0.1584 | No |
| 74 | SMAD4 | SMAD4 Entrez,  Source | SMAD, mothers against DPP homolog 4 (Drosophila) | 9636 | -0.026 | -0.1598 | No |
| 75 | APC | APC Entrez,  Source | adenomatosis polyposis coli | 9658 | -0.026 | -0.1594 | No |
| 76 | APC2 | APC2 Entrez,  Source | adenomatosis polyposis coli 2 | 9797 | -0.028 | -0.1650 | No |
| 77 | PLCB4 | PLCB4 Entrez,  Source | phospholipase C, beta 4 | 10293 | -0.033 | -0.1886 | No |
| 78 | PPP2R5D | PPP2R5D Entrez,  Source | protein phosphatase 2, regulatory subunit B (B56), delta isoform | 10404 | -0.035 | -0.1924 | No |
| 79 | PPP2CB | PPP2CB Entrez,  Source | protein phosphatase 2 (formerly 2A), catalytic subunit, beta isoform | 10978 | -0.042 | -0.2196 | No |
| 80 | SFRP2 | SFRP2 Entrez,  Source | secreted frizzled-related protein 2 | 10980 | -0.042 | -0.2173 | No |
| 81 | ROCK1 | ROCK1 Entrez,  Source | Rho-associated, coiled-coil containing protein kinase 1 | 11335 | -0.046 | -0.2330 | No |
| 82 | CAMK2A | CAMK2A Entrez,  Source | calcium/calmodulin-dependent protein kinase (CaM kinase) II alpha | 11694 | -0.050 | -0.2486 | No |
| 83 | WNT1 | WNT1 Entrez,  Source | wingless-type MMTV integration site family, member 1 | 11756 | -0.051 | -0.2489 | No |
| 84 | DVL1 | DVL1 Entrez,  Source | dishevelled, dsh homolog 1 (Drosophila) | 11895 | -0.053 | -0.2531 | No |
| 85 | PSEN1 | PSEN1 Entrez,  Source | presenilin 1 (Alzheimer disease 3) | 11926 | -0.054 | -0.2516 | No |
| 86 | WIF1 | WIF1 Entrez,  Source | WNT inhibitory factor 1 | 11992 | -0.054 | -0.2520 | No |
| 87 | FZD6 | FZD6 Entrez,  Source | frizzled homolog 6 (Drosophila) | 12007 | -0.054 | -0.2496 | No |
| 88 | LEF1 | LEF1 Entrez,  Source | lymphoid enhancer-binding factor 1 | 12073 | -0.055 | -0.2499 | No |
| 89 | TBL1Y | TBL1Y Entrez,  Source | transducin (beta)-like 1Y-linked | 12309 | -0.058 | -0.2588 | No |
| 90 | RUVBL1 | RUVBL1 Entrez,  Source | RuvB-like 1 (E. coli) | 12340 | -0.058 | -0.2571 | No |
| 91 | CSNK2A1 | CSNK2A1 Entrez,  Source | casein kinase 2, alpha 1 polypeptide | 12378 | -0.059 | -0.2558 | No |
| 92 | MAPK10 | MAPK10 Entrez,  Source | mitogen-activated protein kinase 10 | 12600 | -0.062 | -0.2637 | No |
| 93 | DAAM2 | DAAM2 Entrez,  Source | dishevelled associated activator of morphogenesis 2 | 12723 | -0.063 | -0.2665 | No |
| 94 | CAMK2B | CAMK2B Entrez,  Source | calcium/calmodulin-dependent protein kinase (CaM kinase) II beta | 12956 | -0.066 | -0.2748 | No |
| 95 | TCF7 | TCF7 Entrez,  Source | transcription factor 7 (T-cell specific, HMG-box) | 12964 | -0.066 | -0.2715 | No |
| 96 | SENP2 | SENP2 Entrez,  Source | SUMO1/sentrin/SMT3 specific peptidase 2 | 13308 | -0.071 | -0.2852 | No |
| 97 | MMP7 | MMP7 Entrez,  Source | matrix metallopeptidase 7 (matrilysin, uterine) | 13780 | -0.077 | -0.3052 | No |
| 98 | WNT9B | WNT9B Entrez,  Source | wingless-type MMTV integration site family, member 9B | 13833 | -0.078 | -0.3036 | No |
| 99 | NFATC3 | NFATC3 Entrez,  Source | nuclear factor of activated T-cells, cytoplasmic, calcineurin-dependent 3 | 13852 | -0.078 | -0.3002 | No |
| 100 | WNT8B | WNT8B Entrez,  Source | wingless-type MMTV integration site family, member 8B | 13981 | -0.079 | -0.3024 | No |
| 101 | CER1 | CER1 Entrez,  Source | cerberus 1, cysteine knot superfamily, homolog (Xenopus laevis) | 14324 | -0.084 | -0.3153 | No |
| 102 | PLCB2 | PLCB2 Entrez,  Source | phospholipase C, beta 2 | 14420 | -0.085 | -0.3155 | No |
| 103 | PPP3R2 | PPP3R2 Entrez,  Source | protein phosphatase 3 (formerly 2B), regulatory subunit B, 19kDa, beta isoform (calcineurin B, type II) | 14956 | -0.093 | -0.3379 | No |
| 104 | WNT10B | WNT10B Entrez,  Source | wingless-type MMTV integration site family, member 10B | 15062 | -0.095 | -0.3380 | No |
| 105 | SFRP1 | SFRP1 Entrez,  Source | secreted frizzled-related protein 1 | 15160 | -0.097 | -0.3377 | No |
| 106 | LRP6 | LRP6 Entrez,  Source | low density lipoprotein receptor-related protein 6 | 15254 | -0.098 | -0.3370 | No |
| 107 | NFATC4 | NFATC4 Entrez,  Source | nuclear factor of activated T-cells, cytoplasmic, calcineurin-dependent 4 | 15357 | -0.100 | -0.3367 | No |
| 108 | TP53 | TP53 Entrez,  Source | tumor protein p53 (Li-Fraumeni syndrome) | 15395 | -0.100 | -0.3330 | No |
| 109 | CUL1 | CUL1 Entrez,  Source | cullin 1 | 15428 | -0.101 | -0.3291 | No |
| 110 | FZD5 | FZD5 Entrez,  Source | frizzled homolog 5 (Drosophila) | 15583 | -0.103 | -0.3313 | No |
| 111 | CSNK1E | CSNK1E Entrez,  Source | casein kinase 1, epsilon | 15722 | -0.106 | -0.3325 | No |
| 112 | SFRP4 | SFRP4 Entrez,  Source | secreted frizzled-related protein 4 | 15777 | -0.107 | -0.3293 | No |
| 113 | WNT2B | WNT2B Entrez,  Source | wingless-type MMTV integration site family, member 2B | 16011 | -0.111 | -0.3351 | No |
| 114 | WNT4 | WNT4 Entrez,  Source | wingless-type MMTV integration site family, member 4 | 16063 | -0.112 | -0.3315 | No |
| 115 | SOX17 | SOX17 Entrez,  Source | SRY (sex determining region Y)-box 17 | 16099 | -0.113 | -0.3271 | No |
| 116 | WNT8A | WNT8A Entrez,  Source | wingless-type MMTV integration site family, member 8A | 16353 | -0.118 | -0.3335 | No |
| 117 | VANGL2 | VANGL2 Entrez,  Source | vang-like 2 (van gogh, Drosophila) | 16407 | -0.119 | -0.3296 | No |
| 118 | PPP3R1 | PPP3R1 Entrez,  Source | protein phosphatase 3 (formerly 2B), regulatory subunit B, 19kDa, alpha isoform (calcineurin B, type I) | 16469 | -0.120 | -0.3261 | No |
| 119 | FZD8 | FZD8 Entrez,  Source | frizzled homolog 8 (Drosophila) | 16521 | -0.121 | -0.3220 | No |
| 120 | PRICKLE2 | PRICKLE2 Entrez,  Source | prickle homolog 2 (Drosophila) | 17133 | -0.136 | -0.3459 | No |
| 121 | TCF7L1 | TCF7L1 Entrez,  Source | transcription factor 7-like 1 (T-cell specific, HMG-box) | 17330 | -0.143 | -0.3481 | Yes |
| 122 | PLCB1 | PLCB1 Entrez,  Source | phospholipase C, beta 1 (phosphoinositide-specific) | 17408 | -0.145 | -0.3440 | Yes |
| 123 | WNT2 | WNT2 Entrez,  Source | wingless-type MMTV integration site family member 2 | 17510 | -0.149 | -0.3409 | Yes |
| 124 | FZD9 | FZD9 Entrez,  Source | frizzled homolog 9 (Drosophila) | 17528 | -0.149 | -0.3335 | Yes |
| 125 | AXIN1 | AXIN1 Entrez,  Source | axin 1 | 17530 | -0.149 | -0.3253 | Yes |
| 126 | CSNK2A2 | CSNK2A2 Entrez,  Source | casein kinase 2, alpha prime polypeptide | 17729 | -0.155 | -0.3268 | Yes |
| 127 | FRAT2 | FRAT2 Entrez,  Source | frequently rearranged in advanced T-cell lymphomas 2 | 17907 | -0.163 | -0.3269 | Yes |
| 128 | PRKACG | PRKACG Entrez,  Source | protein kinase, cAMP-dependent, catalytic, gamma | 18249 | -0.178 | -0.3346 | Yes |
| 129 | NKD2 | NKD2 Entrez,  Source | naked cuticle homolog 2 (Drosophila) | 18282 | -0.180 | -0.3262 | Yes |
| 130 | PPARD | PPARD Entrez,  Source | peroxisome proliferative activated receptor, delta | 18408 | -0.187 | -0.3222 | Yes |
| 131 | BTRC | BTRC Entrez,  Source | beta-transducin repeat containing | 18818 | -0.219 | -0.3311 | Yes |
| 132 | CTNNB1 | CTNNB1 Entrez,  Source | catenin (cadherin-associated protein), beta 1, 88kDa | 18943 | -0.235 | -0.3244 | Yes |
| 133 | FRAT1 | FRAT1 Entrez,  Source | frequently rearranged in advanced T-cell lymphomas | 19095 | -0.259 | -0.3178 | Yes |
| 134 | MYC | MYC Entrez,  Source | v-myc myelocytomatosis viral oncogene homolog (avian) | 19109 | -0.262 | -0.3040 | Yes |
| 135 | FZD10 | FZD10 Entrez,  Source | frizzled homolog 10 (Drosophila) | 19260 | -0.308 | -0.2946 | Yes |
| 136 | NLK | NLK Entrez,  Source | nemo-like kinase | 19290 | -0.321 | -0.2783 | Yes |
| 137 | WNT11 | WNT11 Entrez,  Source | wingless-type MMTV integration site family, member 11 | 19383 | -0.388 | -0.2614 | Yes |
| 138 | AXIN2 | AXIN2 Entrez,  Source | axin 2 (conductin, axil) | 19400 | -0.409 | -0.2395 | Yes |
| 139 | WNT5B | WNT5B Entrez,  Source | wingless-type MMTV integration site family, member 5B | 19464 | -0.482 | -0.2160 | Yes |
| 140 | NKD1 | NKD1 Entrez,  Source | naked cuticle homolog 1 (Drosophila) | 19481 | -0.519 | -0.1880 | Yes |
| 141 | WNT6 | WNT6 Entrez,  Source | wingless-type MMTV integration site family, member 6 | 19488 | -0.532 | -0.1588 | Yes |
| 142 | DKK1 | DKK1 Entrez,  Source | dickkopf homolog 1 (Xenopus laevis) | 19493 | -0.554 | -0.1282 | Yes |
| 143 | CCND2 | CCND2 Entrez,  Source | cyclin D2 | 19551 | -1.024 | -0.0742 | Yes |
| 144 | DKK4 | DKK4 Entrez,  Source | dickkopf homolog 4 (Xenopus laevis) | 19556 | -1.340 | -0.0000 | Yes |
Table: GSEA details [plain text format]

  

Fig 2: KEGG\_WNT\_SIGNALING\_PATHWAY      
 Blue-Pink O' Gram in the Space of the Analyzed GeneSet

  

Fig 3: KEGG\_WNT\_SIGNALING\_PATHWAY: Random ES distribution      
 Gene set null distribution of ES for **KEGG\_WNT\_SIGNALING\_PATHWAY**
