# Supplementary material for: Comprehensive analysis of β-catenin target genes in colorectal carcinoma cell lines with deregulated Wnt/β-catenin signaling
Source: BMC Genomics. 2014 Jan 28;15:74. doi: 10.1186/1471-2164-15-74 (PMC3909937; doi:10.1186/1471-2164-15-74)
Supplement: Additional file 5 — GSEA analysis using the KEGG pathway database. This zipped file contains confirming data of the GSEA analysis. The names of the directories containing the files were composed of the term ‘GSEA’, the name of the cell line, e.g. DLD1, SW480, or LS174T, and the pathway database (KEGG). Please use a web browser to view the files with the name ‘index.html’ in the corresponding directories to start exploring the data. [file 1471-2164-15-74-S5.zip › GSEA_KEGG_DLD1/gsea_report_for_0_1358419160078.html]

Report for 0 1358419160078 [GSEA]

| GS  follow link to MSigDB | GS DETAILS | SIZE | ES | NES | NOM p-val | FDR q-val | FWER p-val | RANK AT MAX | LEADING EDGE || 1 | KEGG\_DNA\_REPLICATION | Details ... | 34 | -0.76 | -2.39 | 0.000 | 0.000 | 0.000 | 1659 | tags=71%, list=8%, signal=77% |
| 2 | KEGG\_BASE\_EXCISION\_REPAIR | Details ... | 33 | -0.64 | -1.99 | 0.000 | 0.004 | 0.009 | 2294 | tags=45%, list=12%, signal=51% |
| 3 | KEGG\_MISMATCH\_REPAIR | Details ... | 22 | -0.68 | -1.93 | 0.000 | 0.006 | 0.020 | 2293 | tags=64%, list=12%, signal=72% |
| 4 | KEGG\_HOMOLOGOUS\_RECOMBINATION | Details ... | 26 | -0.66 | -1.91 | 0.000 | 0.005 | 0.024 | 3185 | tags=58%, list=16%, signal=69% |
| 5 | KEGG\_CELL\_CYCLE | Details ... | 113 | -0.46 | -1.82 | 0.000 | 0.012 | 0.068 | 3818 | tags=40%, list=20%, signal=49% |
| 6 | KEGG\_RNA\_POLYMERASE | Details ... | 28 | -0.60 | -1.81 | 0.004 | 0.010 | 0.073 | 3815 | tags=54%, list=20%, signal=66% |
| 7 | KEGG\_NUCLEOTIDE\_EXCISION\_REPAIR | Details ... | 43 | -0.54 | -1.77 | 0.000 | 0.015 | 0.119 | 5973 | tags=65%, list=31%, signal=94% |
| 8 | KEGG\_RIBOSOME | Details ... | 78 | -0.46 | -1.70 | 0.002 | 0.034 | 0.279 | 9960 | tags=91%, list=51%, signal=185% |
| 9 | KEGG\_PYRIMIDINE\_METABOLISM | Details ... | 88 | -0.44 | -1.68 | 0.000 | 0.040 | 0.344 | 3144 | tags=36%, list=16%, signal=43% |
| 10 | KEGG\_AMINOACYL\_TRNA\_BIOSYNTHESIS | Details ... | 32 | -0.53 | -1.66 | 0.015 | 0.043 | 0.396 | 6541 | tags=66%, list=33%, signal=98% |
| 11 | KEGG\_RNA\_DEGRADATION | Details ... | 51 | -0.49 | -1.66 | 0.006 | 0.040 | 0.401 | 6339 | tags=63%, list=32%, signal=93% |
| 12 | KEGG\_GLYCINE\_SERINE\_AND\_THREONINE\_METABOLISM | Details ... | 31 | -0.54 | -1.65 | 0.002 | 0.040 | 0.436 | 2115 | tags=29%, list=11%, signal=33% |
| 13 | KEGG\_SPLICEOSOME | Details ... | 93 | -0.41 | -1.56 | 0.009 | 0.081 | 0.733 | 8173 | tags=70%, list=42%, signal=119% |
| 14 | KEGG\_ONE\_CARBON\_POOL\_BY\_FOLATE | Details ... | 16 | -0.57 | -1.54 | 0.034 | 0.090 | 0.791 | 7507 | tags=75%, list=38%, signal=122% |
| 15 | KEGG\_RENIN\_ANGIOTENSIN\_SYSTEM | Details ... | 16 | -0.59 | -1.54 | 0.027 | 0.085 | 0.796 | 4175 | tags=44%, list=21%, signal=56% |
| 16 | KEGG\_PURINE\_METABOLISM | Details ... | 149 | -0.33 | -1.36 | 0.039 | 0.317 | 0.999 | 3582 | tags=31%, list=18%, signal=38% |
| 17 | KEGG\_CYSTEINE\_AND\_METHIONINE\_METABOLISM | Details ... | 30 | -0.44 | -1.35 | 0.094 | 0.315 | 1.000 | 6849 | tags=50%, list=35%, signal=77% |
| 18 | KEGG\_ALANINE\_ASPARTATE\_AND\_GLUTAMATE\_METABOLISM | Details ... | 31 | -0.43 | -1.33 | 0.088 | 0.345 | 1.000 | 3655 | tags=39%, list=19%, signal=48% |
| 19 | KEGG\_LYSINE\_DEGRADATION | Details ... | 43 | -0.39 | -1.32 | 0.083 | 0.335 | 1.000 | 4340 | tags=35%, list=22%, signal=45% |
| 20 | KEGG\_MELANOMA | Details ... | 71 | -0.35 | -1.29 | 0.104 | 0.402 | 1.000 | 879 | tags=11%, list=4%, signal=12% |
| 21 | KEGG\_TYROSINE\_METABOLISM |  | 38 | -0.39 | -1.28 | 0.131 | 0.412 | 1.000 | 3655 | tags=29%, list=19%, signal=36% |
| 22 | KEGG\_THYROID\_CANCER |  | 29 | -0.41 | -1.28 | 0.139 | 0.395 | 1.000 | 1128 | tags=17%, list=6%, signal=18% |
| 23 | KEGG\_PRIMARY\_IMMUNODEFICIENCY |  | 35 | -0.40 | -1.27 | 0.142 | 0.382 | 1.000 | 5172 | tags=40%, list=26%, signal=54% |
| 24 | KEGG\_CYTOKINE\_CYTOKINE\_RECEPTOR\_INTERACTION |  | 243 | -0.29 | -1.26 | 0.044 | 0.388 | 1.000 | 1697 | tags=15%, list=9%, signal=16% |
| 25 | KEGG\_COLORECTAL\_CANCER |  | 61 | -0.35 | -1.24 | 0.130 | 0.417 | 1.000 | 1948 | tags=18%, list=10%, signal=20% |
| 26 | KEGG\_TRYPTOPHAN\_METABOLISM |  | 37 | -0.38 | -1.23 | 0.181 | 0.437 | 1.000 | 3289 | tags=27%, list=17%, signal=32% |
| 27 | KEGG\_P53\_SIGNALING\_PATHWAY |  | 64 | -0.35 | -1.23 | 0.139 | 0.427 | 1.000 | 4998 | tags=42%, list=26%, signal=56% |
| 28 | KEGG\_BASAL\_CELL\_CARCINOMA |  | 53 | -0.36 | -1.23 | 0.142 | 0.415 | 1.000 | 3481 | tags=34%, list=18%, signal=41% |
| 29 | KEGG\_ALPHA\_LINOLENIC\_ACID\_METABOLISM |  | 16 | -0.46 | -1.22 | 0.215 | 0.422 | 1.000 | 3243 | tags=31%, list=17%, signal=37% |
| 30 | KEGG\_GLYOXYLATE\_AND\_DICARBOXYLATE\_METABOLISM |  | 15 | -0.47 | -1.20 | 0.225 | 0.458 | 1.000 | 6802 | tags=47%, list=35%, signal=71% |
| 31 | KEGG\_NOD\_LIKE\_RECEPTOR\_SIGNALING\_PATHWAY |  | 51 | -0.35 | -1.19 | 0.208 | 0.447 | 1.000 | 438 | tags=10%, list=2%, signal=10% |
| 32 | KEGG\_OXIDATIVE\_PHOSPHORYLATION |  | 109 | -0.30 | -1.19 | 0.142 | 0.452 | 1.000 | 8669 | tags=58%, list=44%, signal=103% |
| 33 | KEGG\_ETHER\_LIPID\_METABOLISM |  | 27 | -0.40 | -1.18 | 0.231 | 0.446 | 1.000 | 924 | tags=15%, list=5%, signal=16% |
| 34 | KEGG\_ARGININE\_AND\_PROLINE\_METABOLISM |  | 51 | -0.33 | -1.15 | 0.235 | 0.521 | 1.000 | 4407 | tags=31%, list=23%, signal=40% |
| 35 | KEGG\_HUNTINGTONS\_DISEASE |  | 163 | -0.27 | -1.12 | 0.171 | 0.567 | 1.000 | 7220 | tags=42%, list=37%, signal=67% |
| 36 | KEGG\_PHENYLALANINE\_METABOLISM |  | 17 | -0.43 | -1.12 | 0.308 | 0.555 | 1.000 | 652 | tags=18%, list=3%, signal=18% |
| 37 | KEGG\_PATHWAYS\_IN\_CANCER |  | 315 | -0.25 | -1.11 | 0.183 | 0.564 | 1.000 | 2261 | tags=15%, list=12%, signal=16% |
| 38 | KEGG\_AUTOIMMUNE\_THYROID\_DISEASE |  | 48 | -0.32 | -1.11 | 0.290 | 0.567 | 1.000 | 1697 | tags=13%, list=9%, signal=14% |
| 39 | KEGG\_GRAFT\_VERSUS\_HOST\_DISEASE |  | 36 | -0.33 | -1.07 | 0.339 | 0.646 | 1.000 | 1070 | tags=8%, list=5%, signal=9% |
| 40 | KEGG\_BASAL\_TRANSCRIPTION\_FACTORS |  | 33 | -0.34 | -1.05 | 0.376 | 0.686 | 1.000 | 4576 | tags=27%, list=23%, signal=36% |
| 41 | KEGG\_PRIMARY\_BILE\_ACID\_BIOSYNTHESIS |  | 16 | -0.40 | -1.05 | 0.392 | 0.671 | 1.000 | 1891 | tags=25%, list=10%, signal=28% |
| 42 | KEGG\_ALLOGRAFT\_REJECTION |  | 34 | -0.33 | -1.05 | 0.398 | 0.668 | 1.000 | 1070 | tags=9%, list=5%, signal=9% |
| 43 | KEGG\_BETA\_ALANINE\_METABOLISM |  | 22 | -0.37 | -1.04 | 0.395 | 0.665 | 1.000 | 2980 | tags=27%, list=15%, signal=32% |
| 44 | KEGG\_TYPE\_I\_DIABETES\_MELLITUS |  | 40 | -0.31 | -1.02 | 0.404 | 0.706 | 1.000 | 1181 | tags=10%, list=6%, signal=11% |
| 45 | KEGG\_ARRHYTHMOGENIC\_RIGHT\_VENTRICULAR\_CARDIOMYOPATHY\_ARVC |  | 73 | -0.28 | -1.01 | 0.415 | 0.722 | 1.000 | 2171 | tags=15%, list=11%, signal=17% |
| 46 | KEGG\_WNT\_SIGNALING\_PATHWAY |  | 144 | -0.25 | -1.00 | 0.448 | 0.735 | 1.000 | 3520 | tags=21%, list=18%, signal=25% |
| 47 | KEGG\_CHEMOKINE\_SIGNALING\_PATHWAY |  | 172 | -0.24 | -1.00 | 0.453 | 0.721 | 1.000 | 1645 | tags=11%, list=8%, signal=12% |
| 48 | KEGG\_MAPK\_SIGNALING\_PATHWAY |  | 253 | -0.23 | -0.99 | 0.457 | 0.735 | 1.000 | 1365 | tags=9%, list=7%, signal=10% |
| 49 | KEGG\_EPITHELIAL\_CELL\_SIGNALING\_IN\_HELICOBACTER\_PYLORI\_INFECTION |  | 62 | -0.28 | -0.99 | 0.478 | 0.726 | 1.000 | 1026 | tags=8%, list=5%, signal=8% |
| 50 | KEGG\_HEDGEHOG\_SIGNALING\_PATHWAY |  | 53 | -0.28 | -0.98 | 0.506 | 0.742 | 1.000 | 3146 | tags=25%, list=16%, signal=29% |
| 51 | KEGG\_ACUTE\_MYELOID\_LEUKEMIA |  | 56 | -0.26 | -0.94 | 0.585 | 0.842 | 1.000 | 1128 | tags=9%, list=6%, signal=9% |
| 52 | KEGG\_SMALL\_CELL\_LUNG\_CANCER |  | 82 | -0.24 | -0.91 | 0.634 | 0.898 | 1.000 | 4111 | tags=29%, list=21%, signal=37% |
| 53 | KEGG\_TGF\_BETA\_SIGNALING\_PATHWAY |  | 82 | -0.24 | -0.91 | 0.641 | 0.896 | 1.000 | 1938 | tags=15%, list=10%, signal=16% |
| 54 | KEGG\_MELANOGENESIS |  | 98 | -0.23 | -0.90 | 0.662 | 0.894 | 1.000 | 3481 | tags=21%, list=18%, signal=26% |
| 55 | KEGG\_MATURITY\_ONSET\_DIABETES\_OF\_THE\_YOUNG |  | 19 | -0.33 | -0.90 | 0.593 | 0.886 | 1.000 | 2386 | tags=21%, list=12%, signal=24% |
| 56 | KEGG\_OLFACTORY\_TRANSDUCTION |  | 112 | -0.23 | -0.89 | 0.713 | 0.881 | 1.000 | 3984 | tags=21%, list=20%, signal=27% |
| 57 | KEGG\_LONG\_TERM\_DEPRESSION |  | 65 | -0.24 | -0.88 | 0.696 | 0.907 | 1.000 | 2697 | tags=15%, list=14%, signal=18% |
| 58 | KEGG\_PARKINSONS\_DISEASE |  | 105 | -0.22 | -0.86 | 0.756 | 0.929 | 1.000 | 8669 | tags=55%, list=44%, signal=99% |
| 59 | KEGG\_DILATED\_CARDIOMYOPATHY |  | 89 | -0.23 | -0.86 | 0.750 | 0.917 | 1.000 | 2171 | tags=13%, list=11%, signal=15% |
| 60 | KEGG\_SELENOAMINO\_ACID\_METABOLISM |  | 21 | -0.30 | -0.85 | 0.686 | 0.923 | 1.000 | 4607 | tags=38%, list=24%, signal=50% |
| 61 | KEGG\_VIBRIO\_CHOLERAE\_INFECTION |  | 50 | -0.25 | -0.85 | 0.723 | 0.912 | 1.000 | 2891 | tags=16%, list=15%, signal=19% |
| 62 | KEGG\_PROGESTERONE\_MEDIATED\_OOCYTE\_MATURATION |  | 82 | -0.22 | -0.83 | 0.828 | 0.928 | 1.000 | 2873 | tags=17%, list=15%, signal=20% |
| 63 | KEGG\_OOCYTE\_MEIOSIS |  | 106 | -0.22 | -0.83 | 0.833 | 0.919 | 1.000 | 2782 | tags=16%, list=14%, signal=19% |
| 64 | KEGG\_PROTEASOME |  | 42 | -0.24 | -0.81 | 0.786 | 0.947 | 1.000 | 931 | tags=5%, list=5%, signal=5% |
| 65 | KEGG\_AMYOTROPHIC\_LATERAL\_SCLEROSIS\_ALS |  | 49 | -0.22 | -0.74 | 0.875 | 1.000 | 1.000 | 1076 | tags=8%, list=6%, signal=9% |
| 66 | KEGG\_LEISHMANIA\_INFECTION |  | 62 | -0.21 | -0.74 | 0.907 | 1.000 | 1.000 | 573 | tags=5%, list=3%, signal=5% |
| 67 | KEGG\_PORPHYRIN\_AND\_CHLOROPHYLL\_METABOLISM |  | 28 | -0.24 | -0.73 | 0.847 | 1.000 | 1.000 | 2778 | tags=21%, list=14%, signal=25% |
| 68 | KEGG\_ASTHMA |  | 27 | -0.24 | -0.73 | 0.898 | 1.000 | 1.000 | 6684 | tags=33%, list=34%, signal=51% |
| 69 | KEGG\_N\_GLYCAN\_BIOSYNTHESIS |  | 41 | -0.21 | -0.70 | 0.941 | 1.000 | 1.000 | 5576 | tags=32%, list=29%, signal=44% |
| 70 | KEGG\_PROTEIN\_EXPORT |  | 19 | -0.24 | -0.67 | 0.886 | 1.000 | 1.000 | 4363 | tags=16%, list=22%, signal=20% |
| 71 | KEGG\_PENTOSE\_PHOSPHATE\_PATHWAY |  | 26 | -0.22 | -0.66 | 0.931 | 1.000 | 1.000 | 4078 | tags=23%, list=21%, signal=29% |
| 72 | KEGG\_PENTOSE\_AND\_GLUCURONATE\_INTERCONVERSIONS |  | 15 | -0.25 | -0.65 | 0.911 | 1.000 | 1.000 | 4782 | tags=33%, list=24%, signal=44% |
| 73 | KEGG\_GLYCOSYLPHOSPHATIDYLINOSITOL\_GPI\_ANCHOR\_BIOSYNTHESIS |  | 24 | -0.20 | -0.58 | 0.979 | 1.000 | 1.000 | 6037 | tags=33%, list=31%, signal=48% |
| 74 | KEGG\_GLYCOSAMINOGLYCAN\_BIOSYNTHESIS\_KERATAN\_SULFATE |  | 15 | -0.22 | -0.56 | 0.964 | 1.000 | 1.000 | 5576 | tags=33%, list=29%, signal=47% |
| 75 | KEGG\_ANTIGEN\_PROCESSING\_AND\_PRESENTATION |  | 77 | -0.15 | -0.55 | 1.000 | 0.992 | 1.000 | 1697 | tags=8%, list=9%, signal=8% |
Table: Gene sets enriched in phenotype **0 (3 samples) **[plain text format]****
