# Supplementary material for: Comprehensive analysis of β-catenin target genes in colorectal carcinoma cell lines with deregulated Wnt/β-catenin signaling
Source: BMC Genomics. 2014 Jan 28;15:74. doi: 10.1186/1471-2164-15-74 (PMC3909937; doi:10.1186/1471-2164-15-74)
Supplement: Additional file 5 — GSEA analysis using the KEGG pathway database. This zipped file contains confirming data of the GSEA analysis. The names of the directories containing the files were composed of the term ‘GSEA’, the name of the cell line, e.g. DLD1, SW480, or LS174T, and the pathway database (KEGG). Please use a web browser to view the files with the name ‘index.html’ in the corresponding directories to start exploring the data. [file 1471-2164-15-74-S5.zip › GSEA_KEGG_DLD1/gsea_report_for_1_1358419160078.html]

Report for 1 1358419160078 [GSEA]

| GS  follow link to MSigDB | GS DETAILS | SIZE | ES | NES | NOM p-val | FDR q-val | FWER p-val | RANK AT MAX | LEADING EDGE || 1 | KEGG\_SYSTEMIC\_LUPUS\_ERYTHEMATOSUS | Details ... | 103 | 0.49 | 1.85 | 0.000 | 0.179 | 0.140 | 1222 | tags=18%, list=6%, signal=20% |
| 2 | KEGG\_PPAR\_SIGNALING\_PATHWAY | Details ... | 66 | 0.50 | 1.80 | 0.000 | 0.159 | 0.225 | 2901 | tags=32%, list=15%, signal=37% |
| 3 | KEGG\_METABOLISM\_OF\_XENOBIOTICS\_BY\_CYTOCHROME\_P450 | Details ... | 56 | 0.52 | 1.76 | 0.004 | 0.164 | 0.327 | 1333 | tags=18%, list=7%, signal=19% |
| 4 | KEGG\_ENDOCYTOSIS | Details ... | 156 | 0.43 | 1.76 | 0.000 | 0.125 | 0.333 | 3474 | tags=34%, list=18%, signal=41% |
| 5 | KEGG\_GLYCOSPHINGOLIPID\_BIOSYNTHESIS\_LACTO\_AND\_NEOLACTO\_SERIES | Details ... | 26 | 0.58 | 1.72 | 0.015 | 0.136 | 0.424 | 3132 | tags=42%, list=16%, signal=50% |
| 6 | KEGG\_ERBB\_SIGNALING\_PATHWAY | Details ... | 85 | 0.45 | 1.71 | 0.000 | 0.129 | 0.471 | 4265 | tags=42%, list=22%, signal=54% |
| 7 | KEGG\_PHOSPHATIDYLINOSITOL\_SIGNALING\_SYSTEM | Details ... | 67 | 0.46 | 1.67 | 0.006 | 0.152 | 0.589 | 3541 | tags=36%, list=18%, signal=44% |
| 8 | KEGG\_GLYCEROLIPID\_METABOLISM | Details ... | 43 | 0.50 | 1.66 | 0.017 | 0.139 | 0.609 | 2436 | tags=33%, list=12%, signal=37% |
| 9 | KEGG\_INSULIN\_SIGNALING\_PATHWAY | Details ... | 130 | 0.42 | 1.65 | 0.000 | 0.132 | 0.639 | 4449 | tags=35%, list=23%, signal=46% |
| 10 | KEGG\_LYSOSOME | Details ... | 114 | 0.43 | 1.65 | 0.004 | 0.121 | 0.645 | 4659 | tags=45%, list=24%, signal=58% |
| 11 | KEGG\_VALINE\_LEUCINE\_AND\_ISOLEUCINE\_DEGRADATION | Details ... | 42 | 0.50 | 1.64 | 0.015 | 0.118 | 0.666 | 1290 | tags=17%, list=7%, signal=18% |
| 12 | KEGG\_STEROID\_HORMONE\_BIOSYNTHESIS | Details ... | 42 | 0.50 | 1.62 | 0.020 | 0.123 | 0.715 | 886 | tags=24%, list=5%, signal=25% |
| 13 | KEGG\_FC\_GAMMA\_R\_MEDIATED\_PHAGOCYTOSIS | Details ... | 85 | 0.43 | 1.61 | 0.006 | 0.119 | 0.732 | 4280 | tags=40%, list=22%, signal=51% |
| 14 | KEGG\_BUTANOATE\_METABOLISM | Details ... | 31 | 0.53 | 1.57 | 0.027 | 0.145 | 0.826 | 1290 | tags=16%, list=7%, signal=17% |
| 15 | KEGG\_INOSITOL\_PHOSPHATE\_METABOLISM | Details ... | 46 | 0.46 | 1.57 | 0.018 | 0.135 | 0.826 | 3541 | tags=37%, list=18%, signal=45% |
| 16 | KEGG\_APOPTOSIS | Details ... | 81 | 0.43 | 1.56 | 0.011 | 0.135 | 0.840 | 4585 | tags=42%, list=23%, signal=55% |
| 17 | KEGG\_TERPENOID\_BACKBONE\_BIOSYNTHESIS | Details ... | 15 | 0.59 | 1.51 | 0.065 | 0.181 | 0.926 | 2749 | tags=40%, list=14%, signal=47% |
| 18 | KEGG\_SPHINGOLIPID\_METABOLISM | Details ... | 30 | 0.50 | 1.51 | 0.039 | 0.172 | 0.928 | 3471 | tags=47%, list=18%, signal=57% |
| 19 | KEGG\_GLYCEROPHOSPHOLIPID\_METABOLISM | Details ... | 63 | 0.43 | 1.50 | 0.019 | 0.177 | 0.942 | 2637 | tags=33%, list=13%, signal=38% |
| 20 | KEGG\_ALDOSTERONE\_REGULATED\_SODIUM\_REABSORPTION | Details ... | 40 | 0.45 | 1.46 | 0.057 | 0.217 | 0.977 | 4248 | tags=50%, list=22%, signal=64% |
| 21 | KEGG\_GLYCOLYSIS\_GLUCONEOGENESIS |  | 60 | 0.42 | 1.46 | 0.024 | 0.208 | 0.977 | 1012 | tags=12%, list=5%, signal=12% |
| 22 | KEGG\_TYPE\_II\_DIABETES\_MELLITUS |  | 44 | 0.43 | 1.43 | 0.058 | 0.232 | 0.990 | 2321 | tags=25%, list=12%, signal=28% |
| 23 | KEGG\_MTOR\_SIGNALING\_PATHWAY |  | 46 | 0.44 | 1.43 | 0.057 | 0.224 | 0.990 | 2871 | tags=28%, list=15%, signal=33% |
| 24 | KEGG\_REGULATION\_OF\_ACTIN\_CYTOSKELETON |  | 196 | 0.34 | 1.42 | 0.013 | 0.232 | 0.992 | 3515 | tags=26%, list=18%, signal=31% |
| 25 | KEGG\_JAK\_STAT\_SIGNALING\_PATHWAY |  | 149 | 0.35 | 1.42 | 0.023 | 0.223 | 0.992 | 3007 | tags=25%, list=15%, signal=29% |
| 26 | KEGG\_ADIPOCYTOKINE\_SIGNALING\_PATHWAY |  | 66 | 0.39 | 1.40 | 0.057 | 0.235 | 0.996 | 3196 | tags=27%, list=16%, signal=32% |
| 27 | KEGG\_O\_GLYCAN\_BIOSYNTHESIS |  | 28 | 0.46 | 1.37 | 0.106 | 0.283 | 0.999 | 4801 | tags=61%, list=25%, signal=80% |
| 28 | KEGG\_GLYCOSPHINGOLIPID\_BIOSYNTHESIS\_GANGLIO\_SERIES |  | 15 | 0.53 | 1.36 | 0.110 | 0.282 | 0.999 | 3030 | tags=40%, list=15%, signal=47% |
| 29 | KEGG\_CALCIUM\_SIGNALING\_PATHWAY |  | 170 | 0.33 | 1.36 | 0.032 | 0.281 | 0.999 | 3833 | tags=27%, list=20%, signal=33% |
| 30 | KEGG\_FOCAL\_ADHESION |  | 187 | 0.32 | 1.34 | 0.041 | 0.306 | 1.000 | 2981 | tags=21%, list=15%, signal=24% |
| 31 | KEGG\_SNARE\_INTERACTIONS\_IN\_VESICULAR\_TRANSPORT |  | 34 | 0.43 | 1.34 | 0.127 | 0.297 | 1.000 | 3862 | tags=41%, list=20%, signal=51% |
| 32 | KEGG\_GLUTATHIONE\_METABOLISM |  | 44 | 0.41 | 1.33 | 0.079 | 0.291 | 1.000 | 3653 | tags=32%, list=19%, signal=39% |
| 33 | KEGG\_STEROID\_BIOSYNTHESIS |  | 16 | 0.50 | 1.32 | 0.135 | 0.296 | 1.000 | 4340 | tags=56%, list=22%, signal=72% |
| 34 | KEGG\_BLADDER\_CANCER |  | 40 | 0.40 | 1.30 | 0.136 | 0.327 | 1.000 | 1048 | tags=18%, list=5%, signal=18% |
| 35 | KEGG\_B\_CELL\_RECEPTOR\_SIGNALING\_PATHWAY |  | 71 | 0.36 | 1.30 | 0.109 | 0.319 | 1.000 | 4071 | tags=31%, list=21%, signal=39% |
| 36 | KEGG\_BIOSYNTHESIS\_OF\_UNSATURATED\_FATTY\_ACIDS |  | 19 | 0.48 | 1.30 | 0.167 | 0.313 | 1.000 | 2093 | tags=26%, list=11%, signal=29% |
| 37 | KEGG\_RENAL\_CELL\_CARCINOMA |  | 68 | 0.36 | 1.30 | 0.106 | 0.310 | 1.000 | 3458 | tags=25%, list=18%, signal=30% |
| 38 | KEGG\_TIGHT\_JUNCTION |  | 124 | 0.33 | 1.29 | 0.070 | 0.305 | 1.000 | 2381 | tags=23%, list=12%, signal=26% |
| 39 | KEGG\_RETINOL\_METABOLISM |  | 45 | 0.38 | 1.28 | 0.138 | 0.318 | 1.000 | 265 | tags=9%, list=1%, signal=9% |
| 40 | KEGG\_AXON\_GUIDANCE |  | 127 | 0.32 | 1.28 | 0.078 | 0.312 | 1.000 | 4577 | tags=34%, list=23%, signal=44% |
| 41 | KEGG\_PROXIMAL\_TUBULE\_BICARBONATE\_RECLAMATION |  | 23 | 0.45 | 1.26 | 0.171 | 0.338 | 1.000 | 771 | tags=13%, list=4%, signal=14% |
| 42 | KEGG\_NEUROTROPHIN\_SIGNALING\_PATHWAY |  | 121 | 0.32 | 1.25 | 0.113 | 0.353 | 1.000 | 4577 | tags=33%, list=23%, signal=43% |
| 43 | KEGG\_ARACHIDONIC\_ACID\_METABOLISM |  | 50 | 0.36 | 1.22 | 0.170 | 0.390 | 1.000 | 839 | tags=14%, list=4%, signal=15% |
| 44 | KEGG\_NON\_SMALL\_CELL\_LUNG\_CANCER |  | 52 | 0.36 | 1.21 | 0.179 | 0.402 | 1.000 | 3458 | tags=31%, list=18%, signal=37% |
| 45 | KEGG\_PYRUVATE\_METABOLISM |  | 40 | 0.37 | 1.20 | 0.178 | 0.408 | 1.000 | 3193 | tags=18%, list=16%, signal=21% |
| 46 | KEGG\_GLIOMA |  | 63 | 0.34 | 1.20 | 0.189 | 0.411 | 1.000 | 2513 | tags=22%, list=13%, signal=25% |
| 47 | KEGG\_DRUG\_METABOLISM\_OTHER\_ENZYMES |  | 36 | 0.39 | 1.19 | 0.215 | 0.417 | 1.000 | 2280 | tags=17%, list=12%, signal=19% |
| 48 | KEGG\_GLYCOSAMINOGLYCAN\_DEGRADATION |  | 21 | 0.41 | 1.16 | 0.249 | 0.474 | 1.000 | 3599 | tags=33%, list=18%, signal=41% |
| 49 | KEGG\_CITRATE\_CYCLE\_TCA\_CYCLE |  | 29 | 0.37 | 1.12 | 0.304 | 0.554 | 1.000 | 1283 | tags=7%, list=7%, signal=7% |
| 50 | KEGG\_AMINO\_SUGAR\_AND\_NUCLEOTIDE\_SUGAR\_METABOLISM |  | 44 | 0.34 | 1.11 | 0.291 | 0.568 | 1.000 | 4503 | tags=34%, list=23%, signal=44% |
| 51 | KEGG\_LONG\_TERM\_POTENTIATION |  | 67 | 0.31 | 1.11 | 0.281 | 0.569 | 1.000 | 3801 | tags=33%, list=19%, signal=41% |
| 52 | KEGG\_LEUKOCYTE\_TRANSENDOTHELIAL\_MIGRATION |  | 105 | 0.29 | 1.10 | 0.258 | 0.569 | 1.000 | 3114 | tags=22%, list=16%, signal=26% |
| 53 | KEGG\_TOLL\_LIKE\_RECEPTOR\_SIGNALING\_PATHWAY |  | 96 | 0.29 | 1.10 | 0.258 | 0.562 | 1.000 | 2711 | tags=17%, list=14%, signal=19% |
| 54 | KEGG\_DORSO\_VENTRAL\_AXIS\_FORMATION |  | 23 | 0.39 | 1.10 | 0.301 | 0.555 | 1.000 | 4545 | tags=48%, list=23%, signal=62% |
| 55 | KEGG\_GAP\_JUNCTION |  | 75 | 0.29 | 1.07 | 0.339 | 0.610 | 1.000 | 4248 | tags=35%, list=22%, signal=44% |
| 56 | KEGG\_FATTY\_ACID\_METABOLISM |  | 39 | 0.34 | 1.07 | 0.358 | 0.603 | 1.000 | 4309 | tags=33%, list=22%, signal=43% |
| 57 | KEGG\_ABC\_TRANSPORTERS |  | 44 | 0.32 | 1.06 | 0.377 | 0.622 | 1.000 | 967 | tags=14%, list=5%, signal=14% |
| 58 | KEGG\_NICOTINATE\_AND\_NICOTINAMIDE\_METABOLISM |  | 21 | 0.37 | 1.05 | 0.380 | 0.625 | 1.000 | 3820 | tags=43%, list=20%, signal=53% |
| 59 | KEGG\_HEMATOPOIETIC\_CELL\_LINEAGE |  | 82 | 0.29 | 1.05 | 0.366 | 0.616 | 1.000 | 1851 | tags=12%, list=9%, signal=13% |
| 60 | KEGG\_CYTOSOLIC\_DNA\_SENSING\_PATHWAY |  | 50 | 0.31 | 1.05 | 0.347 | 0.610 | 1.000 | 1830 | tags=18%, list=9%, signal=20% |
| 61 | KEGG\_ENDOMETRIAL\_CANCER |  | 51 | 0.31 | 1.04 | 0.373 | 0.613 | 1.000 | 3161 | tags=33%, list=16%, signal=40% |
| 62 | KEGG\_PANTOTHENATE\_AND\_COA\_BIOSYNTHESIS |  | 16 | 0.39 | 1.04 | 0.411 | 0.615 | 1.000 | 2338 | tags=25%, list=12%, signal=28% |
| 63 | KEGG\_PROSTATE\_CANCER |  | 87 | 0.28 | 1.04 | 0.371 | 0.611 | 1.000 | 4071 | tags=31%, list=21%, signal=39% |
| 64 | KEGG\_PATHOGENIC\_ESCHERICHIA\_COLI\_INFECTION |  | 42 | 0.32 | 1.03 | 0.393 | 0.614 | 1.000 | 5157 | tags=48%, list=26%, signal=65% |
| 65 | KEGG\_PANCREATIC\_CANCER |  | 69 | 0.29 | 1.03 | 0.403 | 0.607 | 1.000 | 2186 | tags=19%, list=11%, signal=21% |
| 66 | KEGG\_NITROGEN\_METABOLISM |  | 23 | 0.36 | 1.03 | 0.395 | 0.603 | 1.000 | 1572 | tags=17%, list=8%, signal=19% |
| 67 | KEGG\_COMPLEMENT\_AND\_COAGULATION\_CASCADES |  | 65 | 0.28 | 1.03 | 0.413 | 0.595 | 1.000 | 3410 | tags=31%, list=17%, signal=37% |
| 68 | KEGG\_ADHERENS\_JUNCTION |  | 73 | 0.29 | 1.02 | 0.411 | 0.601 | 1.000 | 3956 | tags=34%, list=20%, signal=43% |
| 69 | KEGG\_FRUCTOSE\_AND\_MANNOSE\_METABOLISM |  | 34 | 0.33 | 1.02 | 0.440 | 0.605 | 1.000 | 1928 | tags=15%, list=10%, signal=16% |
| 70 | KEGG\_CHRONIC\_MYELOID\_LEUKEMIA |  | 72 | 0.28 | 1.01 | 0.402 | 0.601 | 1.000 | 3061 | tags=25%, list=16%, signal=30% |
| 71 | KEGG\_GNRH\_SIGNALING\_PATHWAY |  | 96 | 0.27 | 1.00 | 0.429 | 0.625 | 1.000 | 4248 | tags=32%, list=22%, signal=41% |
| 72 | KEGG\_NOTCH\_SIGNALING\_PATHWAY |  | 41 | 0.31 | 0.99 | 0.460 | 0.639 | 1.000 | 5338 | tags=46%, list=27%, signal=64% |
| 73 | KEGG\_NATURAL\_KILLER\_CELL\_MEDIATED\_CYTOTOXICITY |  | 125 | 0.24 | 0.95 | 0.554 | 0.733 | 1.000 | 4071 | tags=26%, list=21%, signal=32% |
| 74 | KEGG\_PRION\_DISEASES |  | 35 | 0.29 | 0.94 | 0.545 | 0.741 | 1.000 | 3286 | tags=26%, list=17%, signal=31% |
| 75 | KEGG\_VASOPRESSIN\_REGULATED\_WATER\_REABSORPTION |  | 44 | 0.27 | 0.92 | 0.573 | 0.783 | 1.000 | 4758 | tags=41%, list=24%, signal=54% |
| 76 | KEGG\_RIG\_I\_LIKE\_RECEPTOR\_SIGNALING\_PATHWAY |  | 64 | 0.26 | 0.92 | 0.608 | 0.778 | 1.000 | 2961 | tags=20%, list=15%, signal=24% |
| 77 | KEGG\_VEGF\_SIGNALING\_PATHWAY |  | 70 | 0.25 | 0.91 | 0.617 | 0.797 | 1.000 | 3874 | tags=29%, list=20%, signal=36% |
| 78 | KEGG\_LINOLEIC\_ACID\_METABOLISM |  | 26 | 0.30 | 0.88 | 0.598 | 0.844 | 1.000 | 814 | tags=8%, list=4%, signal=8% |
| 79 | KEGG\_ECM\_RECEPTOR\_INTERACTION |  | 80 | 0.24 | 0.88 | 0.692 | 0.837 | 1.000 | 3039 | tags=20%, list=16%, signal=24% |
| 80 | KEGG\_PEROXISOME |  | 71 | 0.24 | 0.88 | 0.649 | 0.836 | 1.000 | 5278 | tags=35%, list=27%, signal=48% |
| 81 | KEGG\_HISTIDINE\_METABOLISM |  | 27 | 0.30 | 0.87 | 0.638 | 0.840 | 1.000 | 959 | tags=15%, list=5%, signal=16% |
| 82 | KEGG\_NEUROACTIVE\_LIGAND\_RECEPTOR\_INTERACTION |  | 250 | 0.20 | 0.87 | 0.839 | 0.843 | 1.000 | 3164 | tags=17%, list=16%, signal=20% |
| 83 | KEGG\_T\_CELL\_RECEPTOR\_SIGNALING\_PATHWAY |  | 106 | 0.22 | 0.87 | 0.753 | 0.835 | 1.000 | 4071 | tags=25%, list=21%, signal=32% |
| 84 | KEGG\_CELL\_ADHESION\_MOLECULES\_CAMS |  | 124 | 0.22 | 0.86 | 0.764 | 0.837 | 1.000 | 2307 | tags=12%, list=12%, signal=14% |
| 85 | KEGG\_GLYCOSAMINOGLYCAN\_BIOSYNTHESIS\_HEPARAN\_SULFATE |  | 25 | 0.30 | 0.85 | 0.640 | 0.840 | 1.000 | 2747 | tags=24%, list=14%, signal=28% |
| 86 | KEGG\_REGULATION\_OF\_AUTOPHAGY |  | 32 | 0.28 | 0.85 | 0.684 | 0.834 | 1.000 | 2958 | tags=25%, list=15%, signal=29% |
| 87 | KEGG\_TASTE\_TRANSDUCTION |  | 44 | 0.25 | 0.84 | 0.725 | 0.853 | 1.000 | 2818 | tags=25%, list=14%, signal=29% |
| 88 | KEGG\_FC\_EPSILON\_RI\_SIGNALING\_PATHWAY |  | 73 | 0.23 | 0.84 | 0.774 | 0.848 | 1.000 | 2390 | tags=16%, list=12%, signal=19% |
| 89 | KEGG\_CARDIAC\_MUSCLE\_CONTRACTION |  | 70 | 0.23 | 0.82 | 0.795 | 0.865 | 1.000 | 2663 | tags=16%, list=14%, signal=18% |
| 90 | KEGG\_HYPERTROPHIC\_CARDIOMYOPATHY\_HCM |  | 82 | 0.22 | 0.81 | 0.806 | 0.869 | 1.000 | 3054 | tags=22%, list=16%, signal=26% |
| 91 | KEGG\_VASCULAR\_SMOOTH\_MUSCLE\_CONTRACTION |  | 108 | 0.21 | 0.81 | 0.836 | 0.860 | 1.000 | 2272 | tags=14%, list=12%, signal=16% |
| 92 | KEGG\_VIRAL\_MYOCARDITIS |  | 67 | 0.22 | 0.79 | 0.839 | 0.893 | 1.000 | 2649 | tags=18%, list=14%, signal=21% |
| 93 | KEGG\_ALZHEIMERS\_DISEASE |  | 148 | 0.19 | 0.77 | 0.953 | 0.914 | 1.000 | 3863 | tags=20%, list=20%, signal=24% |
| 94 | KEGG\_RIBOFLAVIN\_METABOLISM |  | 16 | 0.29 | 0.76 | 0.764 | 0.919 | 1.000 | 1898 | tags=19%, list=10%, signal=21% |
| 95 | KEGG\_DRUG\_METABOLISM\_CYTOCHROME\_P450 |  | 57 | 0.21 | 0.72 | 0.914 | 0.956 | 1.000 | 295 | tags=5%, list=2%, signal=5% |
| 96 | KEGG\_PROPANOATE\_METABOLISM |  | 31 | 0.23 | 0.69 | 0.910 | 0.976 | 1.000 | 1085 | tags=10%, list=6%, signal=10% |
| 97 | KEGG\_STARCH\_AND\_SUCROSE\_METABOLISM |  | 34 | 0.22 | 0.68 | 0.924 | 0.970 | 1.000 | 2969 | tags=24%, list=15%, signal=28% |
| 98 | KEGG\_UBIQUITIN\_MEDIATED\_PROTEOLYSIS |  | 121 | 0.14 | 0.57 | 1.000 | 1.000 | 1.000 | 3873 | tags=18%, list=20%, signal=23% |
| 99 | KEGG\_GALACTOSE\_METABOLISM |  | 25 | 0.19 | 0.56 | 0.966 | 0.999 | 1.000 | 4418 | tags=32%, list=23%, signal=41% |
| 100 | KEGG\_INTESTINAL\_IMMUNE\_NETWORK\_FOR\_IGA\_PRODUCTION |  | 45 | 0.16 | 0.53 | 0.998 | 0.993 | 1.000 | 2171 | tags=9%, list=11%, signal=10% |
Table: Gene sets enriched in phenotype **1 (3 samples) **[plain text format]****
