# Supplementary material for: Comprehensive analysis of β-catenin target genes in colorectal carcinoma cell lines with deregulated Wnt/β-catenin signaling
Source: BMC Genomics. 2014 Jan 28;15:74. doi: 10.1186/1471-2164-15-74 (PMC3909937; doi:10.1186/1471-2164-15-74)
Supplement: Additional file 5 — GSEA analysis using the KEGG pathway database. This zipped file contains confirming data of the GSEA analysis. The names of the directories containing the files were composed of the term ‘GSEA’, the name of the cell line, e.g. DLD1, SW480, or LS174T, and the pathway database (KEGG). Please use a web browser to view the files with the name ‘index.html’ in the corresponding directories to start exploring the data. [file 1471-2164-15-74-S5.zip › GSEA_KEGG_DLD1/KEGG_ALANINE_ASPARTATE_AND_GLUTAMATE_METABOLISM.html]

Details for gene set KEGG\_ALANINE\_ASPARTATE\_AND\_GLUTAMATE\_METABOLISM[GSEA]

|  || Dataset | DLD1\_collapsed\_to\_symbols.class.cls#b\_versus\_bg.class.cls#b\_versus\_bg\_repos |
| Phenotype | class.cls#b\_versus\_bg\_repos |
| Upregulated in class | 0 |
| GeneSet | KEGG\_ALANINE\_ASPARTATE\_AND\_GLUTAMATE\_METABOLISM |
| Enrichment Score (ES) | -0.42735016 |
| Normalized Enrichment Score (NES) | -1.3287482 |
| Nominal p-value | 0.08801498 |
| FDR q-value | 0.34498414 |
| FWER p-Value | 1.0 |
Table: GSEA Results Summary

  

Fig 1: Enrichment plot: KEGG\_ALANINE\_ASPARTATE\_AND\_GLUTAMATE\_METABOLISM      
 Profile of the Running ES Score & Positions of GeneSet Members on the Rank Ordered List

  

| PROBE | GENE SYMBOL | GENE\_TITLE | RANK IN GENE LIST | RANK METRIC SCORE | RUNNING ES | CORE ENRICHMENT || 1 | GLUL | GLUL Entrez,  Source | glutamate-ammonia ligase (glutamine synthetase) | 522 | 0.205 | 0.0445 | No |
| 2 | ABAT | ABAT Entrez,  Source | 4-aminobutyrate aminotransferase | 564 | 0.195 | 0.1103 | No |
| 3 | GFPT1 | GFPT1 Entrez,  Source | glutamine-fructose-6-phosphate transaminase 1 | 1592 | 0.114 | 0.0973 | No |
| 4 | ASL | ASL Entrez,  Source | argininosuccinate lyase | 2180 | 0.094 | 0.0998 | No |
| 5 | ASS1 | ASS1 Entrez,  Source | argininosuccinate synthetase 1 | 2213 | 0.093 | 0.1305 | No |
| 6 | CPS1 | CPS1 Entrez,  Source | carbamoyl-phosphate synthetase 1, mitochondrial | 3621 | 0.061 | 0.0796 | No |
| 7 | GFPT2 | GFPT2 Entrez,  Source | glutamine-fructose-6-phosphate transaminase 2 | 4053 | 0.053 | 0.0761 | No |
| 8 | AGXT | AGXT Entrez,  Source | alanine-glyoxylate aminotransferase (oxalosis I; hyperoxaluria I; glycolicaciduria; serine-pyruvate aminotransferase) | 5256 | 0.037 | 0.0272 | No |
| 9 | ADSSL1 | ADSSL1 Entrez,  Source | adenylosuccinate synthase like 1 | 7234 | 0.015 | -0.0688 | No |
| 10 | GLUD2 | GLUD2 Entrez,  Source | glutamate dehydrogenase 2 | 9220 | -0.004 | -0.1692 | No |
| 11 | ALDH4A1 | ALDH4A1 Entrez,  Source | aldehyde dehydrogenase 4 family, member A1 | 9871 | -0.010 | -0.1992 | No |
| 12 | AGXT2 | AGXT2 Entrez,  Source | alanine-glyoxylate aminotransferase 2 | 9978 | -0.010 | -0.2010 | No |
| 13 | ADSS | ADSS Entrez,  Source | adenylosuccinate synthase | 10260 | -0.013 | -0.2109 | No |
| 14 | GLUD1 | GLUD1 Entrez,  Source | glutamate dehydrogenase 1 | 11084 | -0.020 | -0.2462 | No |
| 15 | GPT | GPT Entrez,  Source | glutamic-pyruvate transaminase (alanine aminotransferase) | 12018 | -0.028 | -0.2840 | No |
| 16 | NIT2 | NIT2 Entrez,  Source | nitrilase family, member 2 | 12303 | -0.031 | -0.2878 | No |
| 17 | GOT1 | GOT1 Entrez,  Source | glutamic-oxaloacetic transaminase 1, soluble (aspartate aminotransferase 1) | 12844 | -0.037 | -0.3027 | No |
| 18 | DDO | DDO Entrez,  Source | D-aspartate oxidase | 13636 | -0.045 | -0.3276 | No |
| 19 | ASPA | ASPA Entrez,  Source | aspartoacylase (Canavan disease) | 13783 | -0.047 | -0.3188 | No |
| 20 | GOT2 | GOT2 Entrez,  Source | glutamic-oxaloacetic transaminase 2, mitochondrial (aspartate aminotransferase 2) | 15903 | -0.077 | -0.4005 | Yes |
| 21 | GLS | GLS Entrez,  Source | glutaminase | 15966 | -0.078 | -0.3765 | Yes |
| 22 | GPT2 | GPT2 Entrez,  Source | glutamic pyruvate transaminase (alanine aminotransferase) 2 | 16276 | -0.084 | -0.3632 | Yes |
| 23 | ASNS | ASNS Entrez,  Source | asparagine synthetase | 16329 | -0.085 | -0.3362 | Yes |
| 24 | GAD2 | GAD2 Entrez,  Source | glutamate decarboxylase 2 (pancreatic islets and brain, 65kDa) | 16578 | -0.090 | -0.3177 | Yes |
| 25 | ALDH5A1 | ALDH5A1 Entrez,  Source | aldehyde dehydrogenase 5 family, member A1 (succinate-semialdehyde dehydrogenase) | 16666 | -0.092 | -0.2903 | Yes |
| 26 | ACY3 | ACY3 Entrez,  Source | aspartoacylase (aminocyclase) 3 | 17293 | -0.106 | -0.2856 | Yes |
| 27 | ADSL | ADSL Entrez,  Source | adenylosuccinate lyase | 18068 | -0.131 | -0.2798 | Yes |
| 28 | GAD1 | GAD1 Entrez,  Source | glutamate decarboxylase 1 (brain, 67kDa) | 18377 | -0.145 | -0.2452 | Yes |
| 29 | CAD | CAD Entrez,  Source | carbamoyl-phosphate synthetase 2, aspartate transcarbamylase, and dihydroorotase | 19096 | -0.208 | -0.2095 | Yes |
| 30 | PPAT | PPAT Entrez,  Source | phosphoribosyl pyrophosphate amidotransferase | 19279 | -0.242 | -0.1346 | Yes |
| 31 | GLS2 | GLS2 Entrez,  Source | glutaminase 2 (liver, mitochondrial) | 19518 | -0.428 | 0.0019 | Yes |
Table: GSEA details [plain text format]

  

Fig 2: KEGG\_ALANINE\_ASPARTATE\_AND\_GLUTAMATE\_METABOLISM      
 Blue-Pink O' Gram in the Space of the Analyzed GeneSet

  

Fig 3: KEGG\_ALANINE\_ASPARTATE\_AND\_GLUTAMATE\_METABOLISM: Random ES distribution      
 Gene set null distribution of ES for **KEGG\_ALANINE\_ASPARTATE\_AND\_GLUTAMATE\_METABOLISM**
