# Supplementary material for: Comprehensive analysis of β-catenin target genes in colorectal carcinoma cell lines with deregulated Wnt/β-catenin signaling
Source: BMC Genomics. 2014 Jan 28;15:74. doi: 10.1186/1471-2164-15-74 (PMC3909937; doi:10.1186/1471-2164-15-74)
Supplement: Additional file 5 — GSEA analysis using the KEGG pathway database. This zipped file contains confirming data of the GSEA analysis. The names of the directories containing the files were composed of the term ‘GSEA’, the name of the cell line, e.g. DLD1, SW480, or LS174T, and the pathway database (KEGG). Please use a web browser to view the files with the name ‘index.html’ in the corresponding directories to start exploring the data. [file 1471-2164-15-74-S5.zip › GSEA_KEGG_DLD1/KEGG_ALDOSTERONE_REGULATED_SODIUM_REABSORPTION.html]

Details for gene set KEGG\_ALDOSTERONE\_REGULATED\_SODIUM\_REABSORPTION[GSEA]

|  || Dataset | DLD1\_collapsed\_to\_symbols.class.cls#b\_versus\_bg.class.cls#b\_versus\_bg\_repos |
| Phenotype | class.cls#b\_versus\_bg\_repos |
| Upregulated in class | 1 |
| GeneSet | KEGG\_ALDOSTERONE\_REGULATED\_SODIUM\_REABSORPTION |
| Enrichment Score (ES) | 0.45126343 |
| Normalized Enrichment Score (NES) | 1.4588504 |
| Nominal p-value | 0.057086613 |
| FDR q-value | 0.21675158 |
| FWER p-Value | 0.977 |
Table: GSEA Results Summary

  

Fig 1: Enrichment plot: KEGG\_ALDOSTERONE\_REGULATED\_SODIUM\_REABSORPTION      
 Profile of the Running ES Score & Positions of GeneSet Members on the Rank Ordered List

  

| PROBE | GENE SYMBOL | GENE\_TITLE | RANK IN GENE LIST | RANK METRIC SCORE | RUNNING ES | CORE ENRICHMENT || 1 | PIK3R3 | PIK3R3 Entrez,  Source | phosphoinositide-3-kinase, regulatory subunit 3 (p55, gamma) | 247 | 0.296 | 0.0748 | Yes |
| 2 | NR3C2 | NR3C2 Entrez,  Source | nuclear receptor subfamily 3, group C, member 2 | 331 | 0.257 | 0.1463 | Yes |
| 3 | SCNN1B | SCNN1B Entrez,  Source | sodium channel, nonvoltage-gated 1, beta (Liddle syndrome) | 703 | 0.175 | 0.1791 | Yes |
| 4 | ATP1A4 | ATP1A4 Entrez,  Source | ATPase, Na+/K+ transporting, alpha 4 polypeptide | 771 | 0.169 | 0.2256 | Yes |
| 5 | MAPK3 | MAPK3 Entrez,  Source | mitogen-activated protein kinase 3 | 909 | 0.156 | 0.2646 | Yes |
| 6 | SCNN1A | SCNN1A Entrez,  Source | sodium channel, nonvoltage-gated 1 alpha | 1555 | 0.116 | 0.2657 | Yes |
| 7 | HSD11B1 | HSD11B1 Entrez,  Source | hydroxysteroid (11-beta) dehydrogenase 1 | 1662 | 0.112 | 0.2933 | Yes |
| 8 | NEDD4L | NEDD4L Entrez,  Source | neural precursor cell expressed, developmentally down-regulated 4-like | 1682 | 0.111 | 0.3251 | Yes |
| 9 | PIK3CB | PIK3CB Entrez,  Source | phosphoinositide-3-kinase, catalytic, beta polypeptide | 1862 | 0.104 | 0.3465 | Yes |
| 10 | PIK3CA | PIK3CA Entrez,  Source | phosphoinositide-3-kinase, catalytic, alpha polypeptide | 1955 | 0.101 | 0.3716 | Yes |
| 11 | FXYD4 | FXYD4 Entrez,  Source | FXYD domain containing ion transport regulator 4 | 2806 | 0.077 | 0.3509 | Yes |
| 12 | PDPK1 | PDPK1 Entrez,  Source | 3-phosphoinositide dependent protein kinase-1 | 2871 | 0.076 | 0.3701 | Yes |
| 13 | KRAS | KRAS Entrez,  Source | v-Ki-ras2 Kirsten rat sarcoma viral oncogene homolog | 2993 | 0.073 | 0.3856 | Yes |
| 14 | ATP1B3 | ATP1B3 Entrez,  Source | ATPase, Na+/K+ transporting, beta 3 polypeptide | 3057 | 0.072 | 0.4036 | Yes |
| 15 | IRS1 | IRS1 Entrez,  Source | insulin receptor substrate 1 | 3119 | 0.071 | 0.4214 | Yes |
| 16 | SFN | SFN Entrez,  Source | stratifin | 3142 | 0.070 | 0.4409 | Yes |
| 17 | SLC9A3R2 | SLC9A3R2 Entrez,  Source | solute carrier family 9 (sodium/hydrogen exchanger), member 3 regulator 2 | 3331 | 0.066 | 0.4508 | Yes |
| 18 | MAPK1 | MAPK1 Entrez,  Source | mitogen-activated protein kinase 1 | 3801 | 0.058 | 0.4439 | Yes |
| 19 | ATP1B2 | ATP1B2 Entrez,  Source | ATPase, Na+/K+ transporting, beta 2 polypeptide | 4170 | 0.052 | 0.4403 | Yes |
| 20 | PRKCA | PRKCA Entrez,  Source | protein kinase C, alpha | 4248 | 0.051 | 0.4513 | Yes |
| 21 | PIK3CD | PIK3CD Entrez,  Source | phosphoinositide-3-kinase, catalytic, delta polypeptide | 5186 | 0.037 | 0.4143 | No |
| 22 | ATP1B4 | ATP1B4 Entrez,  Source | ATPase, (Na+)/K+ transporting, beta 4 polypeptide | 6706 | 0.020 | 0.3425 | No |
| 23 | PIK3CG | PIK3CG Entrez,  Source | phosphoinositide-3-kinase, catalytic, gamma polypeptide | 7031 | 0.017 | 0.3309 | No |
| 24 | PIK3R5 | PIK3R5 Entrez,  Source | phosphoinositide-3-kinase, regulatory subunit 5, p101 | 9214 | -0.004 | 0.2202 | No |
| 25 | ATP1B1 | ATP1B1 Entrez,  Source | ATPase, Na+/K+ transporting, beta 1 polypeptide | 9325 | -0.005 | 0.2159 | No |
| 26 | PIK3R1 | PIK3R1 Entrez,  Source | phosphoinositide-3-kinase, regulatory subunit 1 (p85 alpha) | 11545 | -0.024 | 0.1093 | No |
| 27 | ATP1A3 | ATP1A3 Entrez,  Source | ATPase, Na+/K+ transporting, alpha 3 polypeptide | 11838 | -0.027 | 0.1023 | No |
| 28 | INS | INS Entrez,  Source | insulin | 12003 | -0.028 | 0.1022 | No |
| 29 | FXYD2 | FXYD2 Entrez,  Source | FXYD domain containing ion transport regulator 2 | 12021 | -0.029 | 0.1097 | No |
| 30 | IGF1 | IGF1 Entrez,  Source | insulin-like growth factor 1 (somatomedin C) | 12085 | -0.029 | 0.1151 | No |
| 31 | KCNJ1 | KCNJ1 Entrez,  Source | potassium inwardly-rectifying channel, subfamily J, member 1 | 12163 | -0.030 | 0.1200 | No |
| 32 | ATP1A1 | ATP1A1 Entrez,  Source | ATPase, Na+/K+ transporting, alpha 1 polypeptide | 13431 | -0.043 | 0.0677 | No |
| 33 | PIK3R2 | PIK3R2 Entrez,  Source | phosphoinositide-3-kinase, regulatory subunit 2 (p85 beta) | 13588 | -0.044 | 0.0728 | No |
| 34 | PRKCG | PRKCG Entrez,  Source | protein kinase C, gamma | 14320 | -0.053 | 0.0510 | No |
| 35 | INSR | INSR Entrez,  Source | insulin receptor | 14440 | -0.055 | 0.0611 | No |
| 36 | IRS2 | IRS2 Entrez,  Source | insulin receptor substrate 2 | 14745 | -0.059 | 0.0629 | No |
| 37 | IRS4 | IRS4 Entrez,  Source | insulin receptor substrate 4 | 14752 | -0.059 | 0.0799 | No |
| 38 | ATP1A2 | ATP1A2 Entrez,  Source | ATPase, Na+/K+ transporting, alpha 2 (+) polypeptide | 16407 | -0.086 | 0.0207 | No |
| 39 | SCNN1G | SCNN1G Entrez,  Source | sodium channel, nonvoltage-gated 1, gamma | 16531 | -0.089 | 0.0405 | No |
| 40 | HSD11B2 | HSD11B2 Entrez,  Source | hydroxysteroid (11-beta) dehydrogenase 2 | 19492 | -0.388 | 0.0033 | No |
Table: GSEA details [plain text format]

  

Fig 2: KEGG\_ALDOSTERONE\_REGULATED\_SODIUM\_REABSORPTION      
 Blue-Pink O' Gram in the Space of the Analyzed GeneSet

  

Fig 3: KEGG\_ALDOSTERONE\_REGULATED\_SODIUM\_REABSORPTION: Random ES distribution      
 Gene set null distribution of ES for **KEGG\_ALDOSTERONE\_REGULATED\_SODIUM\_REABSORPTION**
