# Supplementary material for: Comprehensive analysis of β-catenin target genes in colorectal carcinoma cell lines with deregulated Wnt/β-catenin signaling
Source: BMC Genomics. 2014 Jan 28;15:74. doi: 10.1186/1471-2164-15-74 (PMC3909937; doi:10.1186/1471-2164-15-74)
Supplement: Additional file 5 — GSEA analysis using the KEGG pathway database. This zipped file contains confirming data of the GSEA analysis. The names of the directories containing the files were composed of the term ‘GSEA’, the name of the cell line, e.g. DLD1, SW480, or LS174T, and the pathway database (KEGG). Please use a web browser to view the files with the name ‘index.html’ in the corresponding directories to start exploring the data. [file 1471-2164-15-74-S5.zip › GSEA_KEGG_DLD1/KEGG_AMINOACYL_TRNA_BIOSYNTHESIS.html]

Details for gene set KEGG\_AMINOACYL\_TRNA\_BIOSYNTHESIS[GSEA]

|  || Dataset | DLD1\_collapsed\_to\_symbols.class.cls#b\_versus\_bg.class.cls#b\_versus\_bg\_repos |
| Phenotype | class.cls#b\_versus\_bg\_repos |
| Upregulated in class | 0 |
| GeneSet | KEGG\_AMINOACYL\_TRNA\_BIOSYNTHESIS |
| Enrichment Score (ES) | -0.5256547 |
| Normalized Enrichment Score (NES) | -1.6569483 |
| Nominal p-value | 0.015414258 |
| FDR q-value | 0.043430135 |
| FWER p-Value | 0.396 |
Table: GSEA Results Summary

  

Fig 1: Enrichment plot: KEGG\_AMINOACYL\_TRNA\_BIOSYNTHESIS      
 Profile of the Running ES Score & Positions of GeneSet Members on the Rank Ordered List

  

| PROBE | GENE SYMBOL | GENE\_TITLE | RANK IN GENE LIST | RANK METRIC SCORE | RUNNING ES | CORE ENRICHMENT || 1 | AARS | AARS Entrez,  Source | alanyl-tRNA synthetase | 1759 | 0.107 | -0.0415 | No |
| 2 | WARS | WARS Entrez,  Source | tryptophanyl-tRNA synthetase | 5512 | 0.033 | -0.2186 | No |
| 3 | RARS | RARS Entrez,  Source | arginyl-tRNA synthetase | 7172 | 0.016 | -0.2966 | No |
| 4 | DARS2 | DARS2 Entrez,  Source | aspartyl-tRNA synthetase 2 (mitochondrial) | 9532 | -0.006 | -0.4145 | No |
| 5 | TARS | TARS Entrez,  Source | threonyl-tRNA synthetase | 9831 | -0.009 | -0.4256 | No |
| 6 | CARS | CARS Entrez,  Source | cysteinyl-tRNA synthetase | 10827 | -0.018 | -0.4686 | No |
| 7 | SARS | SARS Entrez,  Source | seryl-tRNA synthetase | 10870 | -0.018 | -0.4625 | No |
| 8 | IARS2 | IARS2 Entrez,  Source | isoleucine-tRNA synthetase 2, mitochondrial | 11147 | -0.020 | -0.4675 | No |
| 9 | QARS | QARS Entrez,  Source | glutaminyl-tRNA synthetase | 11298 | -0.022 | -0.4653 | No |
| 10 | NARS | NARS Entrez,  Source | asparaginyl-tRNA synthetase | 11932 | -0.028 | -0.4853 | No |
| 11 | HARS | HARS Entrez,  Source | histidyl-tRNA synthetase | 12547 | -0.033 | -0.5016 | No |
| 12 | HARS2 | HARS2 Entrez,  Source | histidyl-tRNA synthetase 2 | 13017 | -0.038 | -0.5083 | Yes |
| 13 | GARS | GARS Entrez,  Source | glycyl-tRNA synthetase | 13330 | -0.042 | -0.5054 | Yes |
| 14 | KARS | KARS Entrez,  Source | lysyl-tRNA synthetase | 13475 | -0.043 | -0.4932 | Yes |
| 15 | EPRS | EPRS Entrez,  Source | glutamyl-prolyl-tRNA synthetase | 13604 | -0.045 | -0.4796 | Yes |
| 16 | IARS | IARS Entrez,  Source | isoleucine-tRNA synthetase | 13710 | -0.046 | -0.4643 | Yes |
| 17 | MARS | MARS Entrez,  Source | methionine-tRNA synthetase | 14253 | -0.052 | -0.4685 | Yes |
| 18 | MTFMT | MTFMT Entrez,  Source | mitochondrial methionyl-tRNA formyltransferase | 14255 | -0.052 | -0.4450 | Yes |
| 19 | PARS2 | PARS2 Entrez,  Source | prolyl-tRNA synthetase (mitochondrial)(putative) | 14629 | -0.057 | -0.4383 | Yes |
| 20 | LARS | LARS Entrez,  Source | leucyl-tRNA synthetase | 14774 | -0.059 | -0.4189 | Yes |
| 21 | YARS | YARS Entrez,  Source | tyrosyl-tRNA synthetase | 15463 | -0.070 | -0.4226 | Yes |
| 22 | WARS2 | WARS2 Entrez,  Source | tryptophanyl tRNA synthetase 2 (mitochondrial) | 16269 | -0.084 | -0.4261 | Yes |
| 23 | YARS2 | YARS2 Entrez,  Source | tyrosyl-tRNA synthetase 2 (mitochondrial) | 16367 | -0.086 | -0.3923 | Yes |
| 24 | TARSL2 | TARSL2 Entrez,  Source | threonyl-tRNA synthetase-like 2 | 16669 | -0.092 | -0.3663 | Yes |
| 25 | FARS2 | FARS2 Entrez,  Source | phenylalanine-tRNA synthetase 2 (mitochondrial) | 16939 | -0.097 | -0.3362 | Yes |
| 26 | SARS2 | SARS2 Entrez,  Source | seryl-tRNA synthetase 2 | 17043 | -0.099 | -0.2966 | Yes |
| 27 | LARS2 | LARS2 Entrez,  Source | leucyl-tRNA synthetase 2, mitochondrial | 17318 | -0.106 | -0.2626 | Yes |
| 28 | DARS | DARS Entrez,  Source | aspartyl-tRNA synthetase | 18013 | -0.128 | -0.2402 | Yes |
| 29 | NARS2 | NARS2 Entrez,  Source | asparaginyl-tRNA synthetase 2 (mitochondrial)(putative) | 18344 | -0.143 | -0.1924 | Yes |
| 30 | VARS | VARS Entrez,  Source | valyl-tRNA synthetase | 18516 | -0.152 | -0.1323 | Yes |
| 31 | EARS2 | EARS2 Entrez,  Source | glutamyl-tRNA synthetase 2 (mitochondrial)(putative) | 18531 | -0.153 | -0.0637 | Yes |
| 32 | MARS2 | MARS2 Entrez,  Source | methionine-tRNA synthetase 2 (mitochondrial) | 19318 | -0.257 | 0.0122 | Yes |
Table: GSEA details [plain text format]

  

Fig 2: KEGG\_AMINOACYL\_TRNA\_BIOSYNTHESIS      
 Blue-Pink O' Gram in the Space of the Analyzed GeneSet

  

Fig 3: KEGG\_AMINOACYL\_TRNA\_BIOSYNTHESIS: Random ES distribution      
 Gene set null distribution of ES for **KEGG\_AMINOACYL\_TRNA\_BIOSYNTHESIS**
