# Supplementary material for: Comprehensive analysis of β-catenin target genes in colorectal carcinoma cell lines with deregulated Wnt/β-catenin signaling
Source: BMC Genomics. 2014 Jan 28;15:74. doi: 10.1186/1471-2164-15-74 (PMC3909937; doi:10.1186/1471-2164-15-74)
Supplement: Additional file 5 — GSEA analysis using the KEGG pathway database. This zipped file contains confirming data of the GSEA analysis. The names of the directories containing the files were composed of the term ‘GSEA’, the name of the cell line, e.g. DLD1, SW480, or LS174T, and the pathway database (KEGG). Please use a web browser to view the files with the name ‘index.html’ in the corresponding directories to start exploring the data. [file 1471-2164-15-74-S5.zip › GSEA_KEGG_DLD1/KEGG_APOPTOSIS.html]

Details for gene set KEGG\_APOPTOSIS[GSEA]

|  || Dataset | DLD1\_collapsed\_to\_symbols.class.cls#b\_versus\_bg.class.cls#b\_versus\_bg\_repos |
| Phenotype | class.cls#b\_versus\_bg\_repos |
| Upregulated in class | 1 |
| GeneSet | KEGG\_APOPTOSIS |
| Enrichment Score (ES) | 0.42930833 |
| Normalized Enrichment Score (NES) | 1.5609723 |
| Nominal p-value | 0.010822511 |
| FDR q-value | 0.13535863 |
| FWER p-Value | 0.84 |
Table: GSEA Results Summary

  

Fig 1: Enrichment plot: KEGG\_APOPTOSIS      
 Profile of the Running ES Score & Positions of GeneSet Members on the Rank Ordered List

  

| PROBE | GENE SYMBOL | GENE\_TITLE | RANK IN GENE LIST | RANK METRIC SCORE | RUNNING ES | CORE ENRICHMENT || 1 | PRKAR2B | PRKAR2B Entrez,  Source | protein kinase, cAMP-dependent, regulatory, type II, beta | 30 | 0.654 | 0.0998 | Yes |
| 2 | IL1R1 | IL1R1 Entrez,  Source | interleukin 1 receptor, type I | 121 | 0.407 | 0.1581 | Yes |
| 3 | PIK3R3 | PIK3R3 Entrez,  Source | phosphoinositide-3-kinase, regulatory subunit 3 (p55, gamma) | 247 | 0.296 | 0.1976 | Yes |
| 4 | BAD | BAD Entrez,  Source | BCL2-antagonist of cell death | 406 | 0.230 | 0.2251 | Yes |
| 5 | TNFSF10 | TNFSF10 Entrez,  Source | tumor necrosis factor (ligand) superfamily, member 10 | 424 | 0.225 | 0.2591 | Yes |
| 6 | CAPN2 | CAPN2 Entrez,  Source | calpain 2, (m/II) large subunit | 490 | 0.210 | 0.2883 | Yes |
| 7 | CSF2RB | CSF2RB Entrez,  Source | colony stimulating factor 2 receptor, beta, low-affinity (granulocyte-macrophage) | 763 | 0.170 | 0.3006 | Yes |
| 8 | CASP9 | CASP9 Entrez,  Source | caspase 9, apoptosis-related cysteine peptidase | 1468 | 0.119 | 0.2829 | Yes |
| 9 | TNFRSF1A | TNFRSF1A Entrez,  Source | tumor necrosis factor receptor superfamily, member 1A | 1472 | 0.119 | 0.3012 | Yes |
| 10 | IL1A | IL1A Entrez,  Source | interleukin 1, alpha | 1754 | 0.108 | 0.3035 | Yes |
| 11 | PRKACB | PRKACB Entrez,  Source | protein kinase, cAMP-dependent, catalytic, beta | 1829 | 0.105 | 0.3159 | Yes |
| 12 | NFKBIA | NFKBIA Entrez,  Source | nuclear factor of kappa light polypeptide gene enhancer in B-cells inhibitor, alpha | 1830 | 0.105 | 0.3322 | Yes |
| 13 | IL3 | IL3 Entrez,  Source | interleukin 3 (colony-stimulating factor, multiple) | 1851 | 0.104 | 0.3473 | Yes |
| 14 | MYD88 | MYD88 Entrez,  Source | myeloid differentiation primary response gene (88) | 1861 | 0.104 | 0.3629 | Yes |
| 15 | PIK3CB | PIK3CB Entrez,  Source | phosphoinositide-3-kinase, catalytic, beta polypeptide | 1862 | 0.104 | 0.3790 | Yes |
| 16 | PIK3CA | PIK3CA Entrez,  Source | phosphoinositide-3-kinase, catalytic, alpha polypeptide | 1955 | 0.101 | 0.3899 | Yes |
| 17 | APAF1 | APAF1 Entrez,  Source | apoptotic peptidase activating factor | 2096 | 0.096 | 0.3977 | Yes |
| 18 | CASP10 | CASP10 Entrez,  Source | caspase 10, apoptosis-related cysteine peptidase | 2466 | 0.086 | 0.3921 | Yes |
| 19 | TRADD | TRADD Entrez,  Source | TNFRSF1A-associated via death domain | 2961 | 0.074 | 0.3782 | Yes |
| 20 | BIRC3 | BIRC3 Entrez,  Source | baculoviral IAP repeat-containing 3 | 2975 | 0.074 | 0.3889 | Yes |
| 21 | CHP | CHP Entrez,  Source | - | 3002 | 0.073 | 0.3989 | Yes |
| 22 | IL1RAP | IL1RAP Entrez,  Source | interleukin 1 receptor accessory protein | 3287 | 0.067 | 0.3947 | Yes |
| 23 | PPP3R2 | PPP3R2 Entrez,  Source | protein phosphatase 3 (formerly 2B), regulatory subunit B, 19kDa, beta isoform (calcineurin B, type II) | 3310 | 0.067 | 0.4039 | Yes |
| 24 | PPP3CA | PPP3CA Entrez,  Source | protein phosphatase 3 (formerly 2B), catalytic subunit, alpha isoform (calcineurin A alpha) | 3698 | 0.060 | 0.3932 | Yes |
| 25 | CASP7 | CASP7 Entrez,  Source | caspase 7, apoptosis-related cysteine peptidase | 3735 | 0.059 | 0.4005 | Yes |
| 26 | PPP3R1 | PPP3R1 Entrez,  Source | protein phosphatase 3 (formerly 2B), regulatory subunit B, 19kDa, alpha isoform (calcineurin B, type I) | 3800 | 0.058 | 0.4062 | Yes |
| 27 | CAPN1 | CAPN1 Entrez,  Source | calpain 1, (mu/I) large subunit | 3863 | 0.057 | 0.4118 | Yes |
| 28 | CFLAR | CFLAR Entrez,  Source | CASP8 and FADD-like apoptosis regulator | 3892 | 0.056 | 0.4190 | Yes |
| 29 | CHUK | CHUK Entrez,  Source | conserved helix-loop-helix ubiquitous kinase | 3917 | 0.056 | 0.4264 | Yes |
| 30 | NTRK1 | NTRK1 Entrez,  Source | neurotrophic tyrosine kinase, receptor, type 1 | 4344 | 0.049 | 0.4122 | Yes |
| 31 | IRAK4 | IRAK4 Entrez,  Source | interleukin-1 receptor-associated kinase 4 | 4409 | 0.048 | 0.4164 | Yes |
| 32 | IKBKB | IKBKB Entrez,  Source | inhibitor of kappa light polypeptide gene enhancer in B-cells, kinase beta | 4438 | 0.048 | 0.4224 | Yes |
| 33 | BIRC2 | BIRC2 Entrez,  Source | baculoviral IAP repeat-containing 2 | 4489 | 0.047 | 0.4271 | Yes |
| 34 | RIPK1 | RIPK1 Entrez,  Source | receptor (TNFRSF)-interacting serine-threonine kinase 1 | 4585 | 0.046 | 0.4293 | Yes |
| 35 | PIK3CD | PIK3CD Entrez,  Source | phosphoinositide-3-kinase, catalytic, delta polypeptide | 5186 | 0.037 | 0.4043 | No |
| 36 | PRKAR1A | PRKAR1A Entrez,  Source | protein kinase, cAMP-dependent, regulatory, type I, alpha (tissue specific extinguisher 1) | 5431 | 0.034 | 0.3971 | No |
| 37 | PRKACG | PRKACG Entrez,  Source | protein kinase, cAMP-dependent, catalytic, gamma | 5873 | 0.029 | 0.3790 | No |
| 38 | IL3RA | IL3RA Entrez,  Source | interleukin 3 receptor, alpha (low affinity) | 6177 | 0.026 | 0.3674 | No |
| 39 | CASP3 | CASP3 Entrez,  Source | caspase 3, apoptosis-related cysteine peptidase | 6285 | 0.025 | 0.3658 | No |
| 40 | PIK3CG | PIK3CG Entrez,  Source | phosphoinositide-3-kinase, catalytic, gamma polypeptide | 7031 | 0.017 | 0.3301 | No |
| 41 | TNFRSF10B | TNFRSF10B Entrez,  Source | tumor necrosis factor receptor superfamily, member 10b | 7034 | 0.017 | 0.3326 | No |
| 42 | PRKAR2A | PRKAR2A Entrez,  Source | protein kinase, cAMP-dependent, regulatory, type II, alpha | 7158 | 0.016 | 0.3288 | No |
| 43 | TNF | TNF Entrez,  Source | tumor necrosis factor (TNF superfamily, member 2) | 7748 | 0.010 | 0.3000 | No |
| 44 | AKT1 | AKT1 Entrez,  Source | v-akt murine thymoma viral oncogene homolog 1 | 7896 | 0.008 | 0.2937 | No |
| 45 | RELA | RELA Entrez,  Source | v-rel reticuloendotheliosis viral oncogene homolog A, nuclear factor of kappa light polypeptide gene enhancer in B-cells 3, p65 (avian) | 8185 | 0.006 | 0.2798 | No |
| 46 | IRAK3 | IRAK3 Entrez,  Source | interleukin-1 receptor-associated kinase 3 | 8603 | 0.002 | 0.2586 | No |
| 47 | ENDOD1 | ENDOD1 Entrez,  Source | endonuclease domain containing 1 | 8793 | -0.000 | 0.2489 | No |
| 48 | IL1B | IL1B Entrez,  Source | interleukin 1, beta | 8810 | -0.000 | 0.2482 | No |
| 49 | PPP3CB | PPP3CB Entrez,  Source | protein phosphatase 3 (formerly 2B), catalytic subunit, beta isoform (calcineurin A beta) | 8941 | -0.001 | 0.2417 | No |
| 50 | MAP3K14 | MAP3K14 Entrez,  Source | mitogen-activated protein kinase kinase kinase 14 | 9177 | -0.003 | 0.2302 | No |
| 51 | PIK3R5 | PIK3R5 Entrez,  Source | phosphoinositide-3-kinase, regulatory subunit 5, p101 | 9214 | -0.004 | 0.2289 | No |
| 52 | CASP8 | CASP8 Entrez,  Source | caspase 8, apoptosis-related cysteine peptidase | 9455 | -0.006 | 0.2175 | No |
| 53 | BCL2L1 | BCL2L1 Entrez,  Source | BCL2-like 1 | 9505 | -0.006 | 0.2159 | No |
| 54 | IKBKG | IKBKG Entrez,  Source | inhibitor of kappa light polypeptide gene enhancer in B-cells, kinase gamma | 9906 | -0.010 | 0.1969 | No |
| 55 | TNFRSF10C | TNFRSF10C Entrez,  Source | tumor necrosis factor receptor superfamily, member 10c, decoy without an intracellular domain | 10110 | -0.011 | 0.1882 | No |
| 56 | BAX | BAX Entrez,  Source | BCL2-associated X protein | 10460 | -0.015 | 0.1726 | No |
| 57 | BCL2 | BCL2 Entrez,  Source | B-cell CLL/lymphoma 2 | 10693 | -0.017 | 0.1632 | No |
| 58 | AKT2 | AKT2 Entrez,  Source | v-akt murine thymoma viral oncogene homolog 2 | 11074 | -0.020 | 0.1468 | No |
| 59 | NFKB1 | NFKB1 Entrez,  Source | nuclear factor of kappa light polypeptide gene enhancer in B-cells 1 (p105) | 11170 | -0.021 | 0.1451 | No |
| 60 | FADD | FADD Entrez,  Source | Fas (TNFRSF6)-associated via death domain | 11388 | -0.023 | 0.1374 | No |
| 61 | PIK3R1 | PIK3R1 Entrez,  Source | phosphoinositide-3-kinase, regulatory subunit 1 (p85 alpha) | 11545 | -0.024 | 0.1332 | No |
| 62 | ATM | ATM Entrez,  Source | ataxia telangiectasia mutated (includes complementation groups A, C and D) | 11953 | -0.028 | 0.1166 | No |
| 63 | TNFRSF10A | TNFRSF10A Entrez,  Source | tumor necrosis factor receptor superfamily, member 10a | 12098 | -0.029 | 0.1137 | No |
| 64 | IRAK1 | IRAK1 Entrez,  Source | interleukin-1 receptor-associated kinase 1 | 12456 | -0.033 | 0.1004 | No |
| 65 | IRAK2 | IRAK2 Entrez,  Source | interleukin-1 receptor-associated kinase 2 | 12522 | -0.033 | 0.1022 | No |
| 66 | PPP3CC | PPP3CC Entrez,  Source | protein phosphatase 3 (formerly 2B), catalytic subunit, gamma isoform (calcineurin A gamma) | 12588 | -0.034 | 0.1041 | No |
| 67 | PRKACA | PRKACA Entrez,  Source | protein kinase, cAMP-dependent, catalytic, alpha | 12867 | -0.037 | 0.0956 | No |
| 68 | TRAF2 | TRAF2 Entrez,  Source | TNF receptor-associated factor 2 | 13237 | -0.041 | 0.0829 | No |
| 69 | PIK3R2 | PIK3R2 Entrez,  Source | phosphoinositide-3-kinase, regulatory subunit 2 (p85 beta) | 13588 | -0.044 | 0.0719 | No |
| 70 | BID | BID Entrez,  Source | BH3 interacting domain death agonist | 14560 | -0.056 | 0.0307 | No |
| 71 | CYCS | CYCS Entrez,  Source | cytochrome c, somatic | 14633 | -0.057 | 0.0359 | No |
| 72 | DFFA | DFFA Entrez,  Source | DNA fragmentation factor, 45kDa, alpha polypeptide | 15194 | -0.065 | 0.0173 | No |
| 73 | AKT3 | AKT3 Entrez,  Source | v-akt murine thymoma viral oncogene homolog 3 (protein kinase B, gamma) | 15572 | -0.071 | 0.0090 | No |
| 74 | CASP6 | CASP6 Entrez,  Source | caspase 6, apoptosis-related cysteine peptidase | 15834 | -0.076 | 0.0073 | No |
| 75 | ENDOG | ENDOG Entrez,  Source | endonuclease G | 17601 | -0.115 | -0.0656 | No |
| 76 | DFFB | DFFB Entrez,  Source | DNA fragmentation factor, 40kDa, beta polypeptide (caspase-activated DNase) | 18341 | -0.143 | -0.0813 | No |
| 77 | FAS | FAS Entrez,  Source | Fas (TNF receptor superfamily, member 6) | 18488 | -0.151 | -0.0655 | No |
| 78 | TP53 | TP53 Entrez,  Source | tumor protein p53 (Li-Fraumeni syndrome) | 18679 | -0.163 | -0.0500 | No |
| 79 | TNFRSF10D | TNFRSF10D Entrez,  Source | tumor necrosis factor receptor superfamily, member 10d, decoy with truncated death domain | 18702 | -0.165 | -0.0255 | No |
| 80 | PRKX | PRKX Entrez,  Source | protein kinase, X-linked | 19137 | -0.214 | -0.0147 | No |
| 81 | FASLG | FASLG Entrez,  Source | Fas ligand (TNF superfamily, member 6) | 19241 | -0.234 | 0.0162 | No |
Table: GSEA details [plain text format]

  

Fig 2: KEGG\_APOPTOSIS      
 Blue-Pink O' Gram in the Space of the Analyzed GeneSet

  

Fig 3: KEGG\_APOPTOSIS: Random ES distribution      
 Gene set null distribution of ES for **KEGG\_APOPTOSIS**
