# Supplementary material for: Comprehensive analysis of β-catenin target genes in colorectal carcinoma cell lines with deregulated Wnt/β-catenin signaling
Source: BMC Genomics. 2014 Jan 28;15:74. doi: 10.1186/1471-2164-15-74 (PMC3909937; doi:10.1186/1471-2164-15-74)
Supplement: Additional file 5 — GSEA analysis using the KEGG pathway database. This zipped file contains confirming data of the GSEA analysis. The names of the directories containing the files were composed of the term ‘GSEA’, the name of the cell line, e.g. DLD1, SW480, or LS174T, and the pathway database (KEGG). Please use a web browser to view the files with the name ‘index.html’ in the corresponding directories to start exploring the data. [file 1471-2164-15-74-S5.zip › GSEA_KEGG_DLD1/KEGG_BUTANOATE_METABOLISM.html]

Details for gene set KEGG\_BUTANOATE\_METABOLISM[GSEA]

|  || Dataset | DLD1\_collapsed\_to\_symbols.class.cls#b\_versus\_bg.class.cls#b\_versus\_bg\_repos |
| Phenotype | class.cls#b\_versus\_bg\_repos |
| Upregulated in class | 1 |
| GeneSet | KEGG\_BUTANOATE\_METABOLISM |
| Enrichment Score (ES) | 0.5285408 |
| Normalized Enrichment Score (NES) | 1.5701443 |
| Nominal p-value | 0.026970955 |
| FDR q-value | 0.14452331 |
| FWER p-Value | 0.826 |
Table: GSEA Results Summary

  

Fig 1: Enrichment plot: KEGG\_BUTANOATE\_METABOLISM      
 Profile of the Running ES Score & Positions of GeneSet Members on the Rank Ordered List

  

| PROBE | GENE SYMBOL | GENE\_TITLE | RANK IN GENE LIST | RANK METRIC SCORE | RUNNING ES | CORE ENRICHMENT || 1 | HMGCS2 | HMGCS2 Entrez,  Source | 3-hydroxy-3-methylglutaryl-Coenzyme A synthase 2 (mitochondrial) | 4 | 1.159 | 0.3012 | Yes |
| 2 | AKR1B10 | AKR1B10 Entrez,  Source | aldo-keto reductase family 1, member B10 (aldose reductase) | 31 | 0.650 | 0.4690 | Yes |
| 3 | ABAT | ABAT Entrez,  Source | 4-aminobutyrate aminotransferase | 564 | 0.195 | 0.4924 | Yes |
| 4 | ALDH3A2 | ALDH3A2 Entrez,  Source | aldehyde dehydrogenase 3 family, member A2 | 959 | 0.152 | 0.5119 | Yes |
| 5 | HMGCS1 | HMGCS1 Entrez,  Source | 3-hydroxy-3-methylglutaryl-Coenzyme A synthase 1 (soluble) | 1290 | 0.129 | 0.5285 | Yes |
| 6 | ALDH9A1 | ALDH9A1 Entrez,  Source | aldehyde dehydrogenase 9 family, member A1 | 2847 | 0.077 | 0.4688 | No |
| 7 | HMGCL | HMGCL Entrez,  Source | 3-hydroxymethyl-3-methylglutaryl-Coenzyme A lyase (hydroxymethylglutaricaciduria) | 5167 | 0.038 | 0.3598 | No |
| 8 | ACAT2 | ACAT2 Entrez,  Source | acetyl-Coenzyme A acetyltransferase 2 (acetoacetyl Coenzyme A thiolase) | 5304 | 0.036 | 0.3622 | No |
| 9 | ACADS | ACADS Entrez,  Source | acyl-Coenzyme A dehydrogenase, C-2 to C-3 short chain | 6211 | 0.026 | 0.3224 | No |
| 10 | ALDH2 | ALDH2 Entrez,  Source | aldehyde dehydrogenase 2 family (mitochondrial) | 6555 | 0.022 | 0.3106 | No |
| 11 | ACSM1 | ACSM1 Entrez,  Source | acyl-CoA synthetase medium-chain family member 1 | 6605 | 0.022 | 0.3137 | No |
| 12 | PDHA2 | PDHA2 Entrez,  Source | pyruvate dehydrogenase (lipoamide) alpha 2 | 7087 | 0.016 | 0.2933 | No |
| 13 | ALDH7A1 | ALDH7A1 Entrez,  Source | aldehyde dehydrogenase 7 family, member A1 | 7706 | 0.010 | 0.2642 | No |
| 14 | BDH1 | BDH1 Entrez,  Source | 3-hydroxybutyrate dehydrogenase, type 1 | 8157 | 0.006 | 0.2427 | No |
| 15 | OXCT2 | OXCT2 Entrez,  Source | 3-oxoacid CoA transferase 2 | 8745 | 0.000 | 0.2127 | No |
| 16 | EHHADH | EHHADH Entrez,  Source | enoyl-Coenzyme A, hydratase/3-hydroxyacyl Coenzyme A dehydrogenase | 9058 | -0.002 | 0.1973 | No |
| 17 | AACS | AACS Entrez,  Source | acetoacetyl-CoA synthetase | 9674 | -0.008 | 0.1679 | No |
| 18 | HADHA | HADHA Entrez,  Source | hydroxyacyl-Coenzyme A dehydrogenase/3-ketoacyl-Coenzyme A thiolase/enoyl-Coenzyme A hydratase (trifunctional protein), alpha subunit | 10086 | -0.011 | 0.1497 | No |
| 19 | PDHB | PDHB Entrez,  Source | pyruvate dehydrogenase (lipoamide) beta | 10740 | -0.017 | 0.1207 | No |
| 20 | ECHS1 | ECHS1 Entrez,  Source | enoyl Coenzyme A hydratase, short chain, 1, mitochondrial | 11609 | -0.025 | 0.0827 | No |
| 21 | OXCT1 | OXCT1 Entrez,  Source | 3-oxoacid CoA transferase 1 | 12507 | -0.033 | 0.0453 | No |
| 22 | BDH2 | BDH2 Entrez,  Source | 3-hydroxybutyrate dehydrogenase, type 2 | 13403 | -0.043 | 0.0106 | No |
| 23 | PDHA1 | PDHA1 Entrez,  Source | pyruvate dehydrogenase (lipoamide) alpha 1 | 13515 | -0.044 | 0.0163 | No |
| 24 | ACAT1 | ACAT1 Entrez,  Source | acetyl-Coenzyme A acetyltransferase 1 (acetoacetyl Coenzyme A thiolase) | 14314 | -0.053 | -0.0108 | No |
| 25 | GAD2 | GAD2 Entrez,  Source | glutamate decarboxylase 2 (pancreatic islets and brain, 65kDa) | 16578 | -0.090 | -0.1034 | No |
| 26 | ALDH5A1 | ALDH5A1 Entrez,  Source | aldehyde dehydrogenase 5 family, member A1 (succinate-semialdehyde dehydrogenase) | 16666 | -0.092 | -0.0840 | No |
| 27 | L2HGDH | L2HGDH Entrez,  Source | L-2-hydroxyglutarate dehydrogenase | 17374 | -0.107 | -0.0923 | No |
| 28 | HADH | HADH Entrez,  Source | hydroxyacyl-Coenzyme A dehydrogenase | 17614 | -0.115 | -0.0747 | No |
| 29 | GAD1 | GAD1 Entrez,  Source | glutamate decarboxylase 1 (brain, 67kDa) | 18377 | -0.145 | -0.0761 | No |
| 30 | ALDH1B1 | ALDH1B1 Entrez,  Source | aldehyde dehydrogenase 1 family, member B1 | 19017 | -0.196 | -0.0578 | No |
| 31 | ACSM3 | ACSM3 Entrez,  Source | acyl-CoA synthetase medium-chain family member 3 | 19450 | -0.328 | 0.0054 | No |
Table: GSEA details [plain text format]

  

Fig 2: KEGG\_BUTANOATE\_METABOLISM      
 Blue-Pink O' Gram in the Space of the Analyzed GeneSet

  

Fig 3: KEGG\_BUTANOATE\_METABOLISM: Random ES distribution      
 Gene set null distribution of ES for **KEGG\_BUTANOATE\_METABOLISM**
