# Supplementary material for: Comprehensive analysis of β-catenin target genes in colorectal carcinoma cell lines with deregulated Wnt/β-catenin signaling
Source: BMC Genomics. 2014 Jan 28;15:74. doi: 10.1186/1471-2164-15-74 (PMC3909937; doi:10.1186/1471-2164-15-74)
Supplement: Additional file 5 — GSEA analysis using the KEGG pathway database. This zipped file contains confirming data of the GSEA analysis. The names of the directories containing the files were composed of the term ‘GSEA’, the name of the cell line, e.g. DLD1, SW480, or LS174T, and the pathway database (KEGG). Please use a web browser to view the files with the name ‘index.html’ in the corresponding directories to start exploring the data. [file 1471-2164-15-74-S5.zip › GSEA_KEGG_DLD1/KEGG_CELL_CYCLE.html]

Details for gene set KEGG\_CELL\_CYCLE[GSEA]

|  || Dataset | DLD1\_collapsed\_to\_symbols.class.cls#b\_versus\_bg.class.cls#b\_versus\_bg\_repos |
| Phenotype | class.cls#b\_versus\_bg\_repos |
| Upregulated in class | 0 |
| GeneSet | KEGG\_CELL\_CYCLE |
| Enrichment Score (ES) | -0.46348897 |
| Normalized Enrichment Score (NES) | -1.8187087 |
| Nominal p-value | 0.0 |
| FDR q-value | 0.011701909 |
| FWER p-Value | 0.068 |
Table: GSEA Results Summary

  

Fig 1: Enrichment plot: KEGG\_CELL\_CYCLE      
 Profile of the Running ES Score & Positions of GeneSet Members on the Rank Ordered List

  

| PROBE | GENE SYMBOL | GENE\_TITLE | RANK IN GENE LIST | RANK METRIC SCORE | RUNNING ES | CORE ENRICHMENT || 1 | CDKN2B | CDKN2B Entrez,  Source | cyclin-dependent kinase inhibitor 2B (p15, inhibits CDK4) | 34 | 0.646 | 0.0636 | No |
| 2 | CDKN2D | CDKN2D Entrez,  Source | cyclin-dependent kinase inhibitor 2D (p19, inhibits CDK4) | 550 | 0.197 | 0.0571 | No |
| 3 | GADD45G | GADD45G Entrez,  Source | growth arrest and DNA-damage-inducible, gamma | 753 | 0.171 | 0.0640 | No |
| 4 | CDKN2C | CDKN2C Entrez,  Source | cyclin-dependent kinase inhibitor 2C (p18, inhibits CDK4) | 1434 | 0.121 | 0.0413 | No |
| 5 | SMAD3 | SMAD3 Entrez,  Source | SMAD, mothers against DPP homolog 3 (Drosophila) | 1611 | 0.113 | 0.0437 | No |
| 6 | CDKN1A | CDKN1A Entrez,  Source | cyclin-dependent kinase inhibitor 1A (p21, Cip1) | 2044 | 0.098 | 0.0314 | No |
| 7 | SMC1B | SMC1B Entrez,  Source | structural maintenance of chromosomes 1B | 2060 | 0.097 | 0.0405 | No |
| 8 | YWHAZ | YWHAZ Entrez,  Source | tyrosine 3-monooxygenase/tryptophan 5-monooxygenase activation protein, zeta polypeptide | 2561 | 0.083 | 0.0232 | No |
| 9 | FZR1 | FZR1 Entrez,  Source | fizzy/cell division cycle 20 related 1 (Drosophila) | 2858 | 0.076 | 0.0157 | No |
| 10 | HDAC1 | HDAC1 Entrez,  Source | histone deacetylase 1 | 3061 | 0.072 | 0.0126 | No |
| 11 | SFN | SFN Entrez,  Source | stratifin | 3142 | 0.070 | 0.0156 | No |
| 12 | GSK3B | GSK3B Entrez,  Source | glycogen synthase kinase 3 beta | 3729 | 0.059 | -0.0085 | No |
| 13 | CDKN1B | CDKN1B Entrez,  Source | cyclin-dependent kinase inhibitor 1B (p27, Kip1) | 4265 | 0.050 | -0.0310 | No |
| 14 | RBL2 | RBL2 Entrez,  Source | retinoblastoma-like 2 (p130) | 5119 | 0.038 | -0.0709 | No |
| 15 | SMAD4 | SMAD4 Entrez,  Source | SMAD, mothers against DPP homolog 4 (Drosophila) | 5334 | 0.036 | -0.0784 | No |
| 16 | CDC27 | CDC27 Entrez,  Source | cell division cycle 27 | 5765 | 0.030 | -0.0974 | No |
| 17 | SMAD2 | SMAD2 Entrez,  Source | SMAD, mothers against DPP homolog 2 (Drosophila) | 6104 | 0.027 | -0.1121 | No |
| 18 | CDC25B | CDC25B Entrez,  Source | cell division cycle 25B | 6146 | 0.026 | -0.1115 | No |
| 19 | CDC14B | CDC14B Entrez,  Source | CDC14 cell division cycle 14 homolog B (S. cerevisiae) | 6280 | 0.025 | -0.1159 | No |
| 20 | CCNA1 | CCNA1 Entrez,  Source | cyclin A1 | 6726 | 0.020 | -0.1367 | No |
| 21 | YWHAB | YWHAB Entrez,  Source | tyrosine 3-monooxygenase/tryptophan 5-monooxygenase activation protein, beta polypeptide | 7044 | 0.017 | -0.1513 | No |
| 22 | E2F2 | E2F2 Entrez,  Source | E2F transcription factor 2 | 7856 | 0.009 | -0.1922 | No |
| 23 | CDC16 | CDC16 Entrez,  Source | CDC16 cell division cycle 16 homolog (S. cerevisiae) | 7965 | 0.007 | -0.1970 | No |
| 24 | RB1 | RB1 Entrez,  Source | retinoblastoma 1 (including osteosarcoma) | 7991 | 0.007 | -0.1975 | No |
| 25 | YWHAQ | YWHAQ Entrez,  Source | tyrosine 3-monooxygenase/tryptophan 5-monooxygenase activation protein, theta polypeptide | 8260 | 0.005 | -0.2108 | No |
| 26 | STAG2 | STAG2 Entrez,  Source | stromal antigen 2 | 8493 | 0.003 | -0.2225 | No |
| 27 | CUL1 | CUL1 Entrez,  Source | cullin 1 | 8610 | 0.002 | -0.2283 | No |
| 28 | GADD45A | GADD45A Entrez,  Source | growth arrest and DNA-damage-inducible, alpha | 8858 | -0.001 | -0.2409 | No |
| 29 | E2F4 | E2F4 Entrez,  Source | E2F transcription factor 4, p107/p130-binding | 8961 | -0.001 | -0.2460 | No |
| 30 | YWHAE | YWHAE Entrez,  Source | tyrosine 3-monooxygenase/tryptophan 5-monooxygenase activation protein, epsilon polypeptide | 9650 | -0.008 | -0.2806 | No |
| 31 | E2F3 | E2F3 Entrez,  Source | E2F transcription factor 3 | 9752 | -0.008 | -0.2850 | No |
| 32 | CREBBP | CREBBP Entrez,  Source | CREB binding protein (Rubinstein-Taybi syndrome) | 9983 | -0.010 | -0.2958 | No |
| 33 | STAG1 | STAG1 Entrez,  Source | stromal antigen 1 | 10098 | -0.011 | -0.3005 | No |
| 34 | CCNB1 | CCNB1 Entrez,  Source | cyclin B1 | 10142 | -0.012 | -0.3015 | No |
| 35 | RBL1 | RBL1 Entrez,  Source | retinoblastoma-like 1 (p107) | 10257 | -0.013 | -0.3061 | No |
| 36 | RBX1 | RBX1 Entrez,  Source | ring-box 1 | 10483 | -0.015 | -0.3161 | No |
| 37 | CDK7 | CDK7 Entrez,  Source | cyclin-dependent kinase 7 (MO15 homolog, Xenopus laevis, cdk-activating kinase) | 10567 | -0.016 | -0.3188 | No |
| 38 | CDC25C | CDC25C Entrez,  Source | cell division cycle 25C | 10638 | -0.016 | -0.3208 | No |
| 39 | CDC14A | CDC14A Entrez,  Source | CDC14 cell division cycle 14 homolog A (S. cerevisiae) | 10978 | -0.019 | -0.3363 | No |
| 40 | CCND3 | CCND3 Entrez,  Source | cyclin D3 | 10985 | -0.019 | -0.3347 | No |
| 41 | CDC23 | CDC23 Entrez,  Source | CDC23 (cell division cycle 23, yeast, homolog) | 11058 | -0.020 | -0.3364 | No |
| 42 | BUB1 | BUB1 Entrez,  Source | BUB1 budding uninhibited by benzimidazoles 1 homolog (yeast) | 11345 | -0.022 | -0.3488 | No |
| 43 | YWHAG | YWHAG Entrez,  Source | tyrosine 3-monooxygenase/tryptophan 5-monooxygenase activation protein, gamma polypeptide | 11434 | -0.023 | -0.3510 | No |
| 44 | RAD21 | RAD21 Entrez,  Source | RAD21 homolog (S. pombe) | 11562 | -0.024 | -0.3551 | No |
| 45 | EP300 | EP300 Entrez,  Source | E1A binding protein p300 | 11709 | -0.026 | -0.3600 | No |
| 46 | CDKN2A | CDKN2A Entrez,  Source | cyclin-dependent kinase inhibitor 2A (melanoma, p16, inhibits CDK4) | 11732 | -0.026 | -0.3586 | No |
| 47 | MDM2 | MDM2 Entrez,  Source | Mdm2, transformed 3T3 cell double minute 2, p53 binding protein (mouse) | 11805 | -0.026 | -0.3596 | No |
| 48 | ANAPC13 | ANAPC13 Entrez,  Source | anaphase promoting complex subunit 13 | 11870 | -0.027 | -0.3601 | No |
| 49 | ATM | ATM Entrez,  Source | ataxia telangiectasia mutated (includes complementation groups A, C and D) | 11953 | -0.028 | -0.3615 | No |
| 50 | ABL1 | ABL1 Entrez,  Source | v-abl Abelson murine leukemia viral oncogene homolog 1 | 12026 | -0.029 | -0.3624 | No |
| 51 | YWHAH | YWHAH Entrez,  Source | tyrosine 3-monooxygenase/tryptophan 5-monooxygenase activation protein, eta polypeptide | 12053 | -0.029 | -0.3608 | No |
| 52 | ANAPC5 | ANAPC5 Entrez,  Source | anaphase promoting complex subunit 5 | 12652 | -0.035 | -0.3880 | No |
| 53 | HDAC2 | HDAC2 Entrez,  Source | histone deacetylase 2 | 12700 | -0.035 | -0.3869 | No |
| 54 | CCND2 | CCND2 Entrez,  Source | cyclin D2 | 12836 | -0.036 | -0.3901 | No |
| 55 | CDC20 | CDC20 Entrez,  Source | CDC20 cell division cycle 20 homolog (S. cerevisiae) | 13126 | -0.039 | -0.4010 | No |
| 56 | ANAPC10 | ANAPC10 Entrez,  Source | anaphase promoting complex subunit 10 | 13229 | -0.041 | -0.4021 | No |
| 57 | ESPL1 | ESPL1 Entrez,  Source | extra spindle poles like 1 (S. cerevisiae) | 13308 | -0.042 | -0.4019 | No |
| 58 | BUB3 | BUB3 Entrez,  Source | BUB3 budding uninhibited by benzimidazoles 3 homolog (yeast) | 13502 | -0.044 | -0.4075 | No |
| 59 | ZBTB17 | ZBTB17 Entrez,  Source | zinc finger and BTB domain containing 17 | 13809 | -0.047 | -0.4184 | No |
| 60 | PTTG1 | PTTG1 Entrez,  Source | pituitary tumor-transforming 1 | 13959 | -0.049 | -0.4212 | No |
| 61 | PLK1 | PLK1 Entrez,  Source | polo-like kinase 1 (Drosophila) | 13962 | -0.049 | -0.4163 | No |
| 62 | CCNB2 | CCNB2 Entrez,  Source | cyclin B2 | 14040 | -0.050 | -0.4153 | No |
| 63 | ANAPC4 | ANAPC4 Entrez,  Source | anaphase promoting complex subunit 4 | 14256 | -0.052 | -0.4210 | No |
| 64 | WEE1 | WEE1 Entrez,  Source | WEE1 homolog (S. pombe) | 15011 | -0.063 | -0.4535 | No |
| 65 | TTK | TTK Entrez,  Source | TTK protein kinase | 15075 | -0.064 | -0.4502 | No |
| 66 | TGFB3 | TGFB3 Entrez,  Source | transforming growth factor, beta 3 | 15309 | -0.067 | -0.4554 | No |
| 67 | SMC3 | SMC3 Entrez,  Source | structural maintenance of chromosomes 3 | 15391 | -0.068 | -0.4527 | No |
| 68 | CCNH | CCNH Entrez,  Source | cyclin H | 15442 | -0.069 | -0.4482 | No |
| 69 | CCND1 | CCND1 Entrez,  Source | cyclin D1 | 15740 | -0.074 | -0.4560 | Yes |
| 70 | BUB1B | BUB1B Entrez,  Source | BUB1 budding uninhibited by benzimidazoles 1 homolog beta (yeast) | 15789 | -0.075 | -0.4508 | Yes |
| 71 | ANAPC2 | ANAPC2 Entrez,  Source | anaphase promoting complex subunit 2 | 15800 | -0.075 | -0.4437 | Yes |
| 72 | GADD45B | GADD45B Entrez,  Source | growth arrest and DNA-damage-inducible, beta | 15841 | -0.076 | -0.4381 | Yes |
| 73 | TFDP2 | TFDP2 Entrez,  Source | transcription factor Dp-2 (E2F dimerization partner 2) | 15937 | -0.078 | -0.4351 | Yes |
| 74 | PRKDC | PRKDC Entrez,  Source | protein kinase, DNA-activated, catalytic polypeptide | 16100 | -0.080 | -0.4353 | Yes |
| 75 | PTTG2 | PTTG2 Entrez,  Source | pituitary tumor-transforming 2 | 16146 | -0.081 | -0.4294 | Yes |
| 76 | TFDP1 | TFDP1 Entrez,  Source | transcription factor Dp-1 | 16272 | -0.084 | -0.4273 | Yes |
| 77 | CDKN1C | CDKN1C Entrez,  Source | cyclin-dependent kinase inhibitor 1C (p57, Kip2) | 16379 | -0.086 | -0.4241 | Yes |
| 78 | SMC1A | SMC1A Entrez,  Source | structural maintenance of chromosomes 1A | 16415 | -0.086 | -0.4172 | Yes |
| 79 | CCNB3 | CCNB3 Entrez,  Source | cyclin B3 | 16685 | -0.092 | -0.4217 | Yes |
| 80 | CCNA2 | CCNA2 Entrez,  Source | cyclin A2 | 16700 | -0.092 | -0.4130 | Yes |
| 81 | ATR | ATR Entrez,  Source | ataxia telangiectasia and Rad3 related | 16896 | -0.096 | -0.4133 | Yes |
| 82 | E2F1 | E2F1 Entrez,  Source | E2F transcription factor 1 | 16921 | -0.097 | -0.4048 | Yes |
| 83 | CHEK2 | CHEK2 Entrez,  Source | CHK2 checkpoint homolog (S. pombe) | 16941 | -0.097 | -0.3959 | Yes |
| 84 | ANAPC7 | ANAPC7 Entrez,  Source | anaphase promoting complex subunit 7 | 16964 | -0.098 | -0.3872 | Yes |
| 85 | CHEK1 | CHEK1 Entrez,  Source | CHK1 checkpoint homolog (S. pombe) | 17163 | -0.102 | -0.3870 | Yes |
| 86 | PCNA | PCNA Entrez,  Source | proliferating cell nuclear antigen | 17265 | -0.105 | -0.3816 | Yes |
| 87 | CCNE2 | CCNE2 Entrez,  Source | cyclin E2 | 17297 | -0.106 | -0.3725 | Yes |
| 88 | MAD2L1 | MAD2L1 Entrez,  Source | MAD2 mitotic arrest deficient-like 1 (yeast) | 17343 | -0.107 | -0.3640 | Yes |
| 89 | CDC6 | CDC6 Entrez,  Source | CDC6 cell division cycle 6 homolog (S. cerevisiae) | 17830 | -0.122 | -0.3766 | Yes |
| 90 | DBF4 | DBF4 Entrez,  Source | DBF4 homolog (S. cerevisiae) | 17896 | -0.124 | -0.3674 | Yes |
| 91 | PKMYT1 | PKMYT1 Entrez,  Source | protein kinase, membrane associated tyrosine/threonine 1 | 17940 | -0.126 | -0.3569 | Yes |
| 92 | TGFB1 | TGFB1 Entrez,  Source | transforming growth factor, beta 1 (Camurati-Engelmann disease) | 17969 | -0.126 | -0.3455 | Yes |
| 93 | MCM7 | MCM7 Entrez,  Source | MCM7 minichromosome maintenance deficient 7 (S. cerevisiae) | 18206 | -0.137 | -0.3439 | Yes |
| 94 | MAD2L2 | MAD2L2 Entrez,  Source | MAD2 mitotic arrest deficient-like 2 (yeast) | 18286 | -0.140 | -0.3337 | Yes |
| 95 | CDK2 | CDK2 Entrez,  Source | cyclin-dependent kinase 2 | 18306 | -0.141 | -0.3204 | Yes |
| 96 | ANAPC1 | ANAPC1 Entrez,  Source | anaphase promoting complex subunit 1 | 18436 | -0.147 | -0.3121 | Yes |
| 97 | SKP2 | SKP2 Entrez,  Source | S-phase kinase-associated protein 2 (p45) | 18525 | -0.153 | -0.3012 | Yes |
| 98 | CDC7 | CDC7 Entrez,  Source | CDC7 cell division cycle 7 (S. cerevisiae) | 18608 | -0.158 | -0.2894 | Yes |
| 99 | E2F5 | E2F5 Entrez,  Source | E2F transcription factor 5, p130-binding | 18656 | -0.162 | -0.2755 | Yes |
| 100 | TP53 | TP53 Entrez,  Source | tumor protein p53 (Li-Fraumeni syndrome) | 18679 | -0.163 | -0.2601 | Yes |
| 101 | MCM4 | MCM4 Entrez,  Source | MCM4 minichromosome maintenance deficient 4 (S. cerevisiae) | 18732 | -0.167 | -0.2458 | Yes |
| 102 | MCM6 | MCM6 Entrez,  Source | MCM6 minichromosome maintenance deficient 6 (MIS5 homolog, S. pombe) (S. cerevisiae) | 18764 | -0.170 | -0.2302 | Yes |
| 103 | CCNE1 | CCNE1 Entrez,  Source | cyclin E1 | 18834 | -0.178 | -0.2157 | Yes |
| 104 | ANAPC11 | ANAPC11 Entrez,  Source | APC11 anaphase promoting complex subunit 11 homolog (yeast) | 18883 | -0.182 | -0.1998 | Yes |
| 105 | CDK4 | CDK4 Entrez,  Source | cyclin-dependent kinase 4 | 18954 | -0.190 | -0.1841 | Yes |
| 106 | MCM3 | MCM3 Entrez,  Source | MCM3 minichromosome maintenance deficient 3 (S. cerevisiae) | 19143 | -0.215 | -0.1721 | Yes |
| 107 | CDC25A | CDC25A Entrez,  Source | cell division cycle 25A | 19211 | -0.225 | -0.1528 | Yes |
| 108 | MCM5 | MCM5 Entrez,  Source | MCM5 minichromosome maintenance deficient 5, cell division cycle 46 (S. cerevisiae) | 19245 | -0.234 | -0.1307 | Yes |
| 109 | MAD1L1 | MAD1L1 Entrez,  Source | MAD1 mitotic arrest deficient-like 1 (yeast) | 19285 | -0.244 | -0.1081 | Yes |
| 110 | MCM2 | MCM2 Entrez,  Source | MCM2 minichromosome maintenance deficient 2, mitotin (S. cerevisiae) | 19296 | -0.249 | -0.0834 | Yes |
| 111 | TGFB2 | TGFB2 Entrez,  Source | transforming growth factor, beta 2 | 19391 | -0.290 | -0.0589 | Yes |
| 112 | MYC | MYC Entrez,  Source | v-myc myelocytomatosis viral oncogene homolog (avian) | 19447 | -0.326 | -0.0287 | Yes |
| 113 | CDK6 | CDK6 Entrez,  Source | cyclin-dependent kinase 6 | 19461 | -0.339 | 0.0049 | Yes |
Table: GSEA details [plain text format]

  

Fig 2: KEGG\_CELL\_CYCLE      
 Blue-Pink O' Gram in the Space of the Analyzed GeneSet

  

Fig 3: KEGG\_CELL\_CYCLE: Random ES distribution      
 Gene set null distribution of ES for **KEGG\_CELL\_CYCLE**
