# Supplementary material for: Comprehensive analysis of β-catenin target genes in colorectal carcinoma cell lines with deregulated Wnt/β-catenin signaling
Source: BMC Genomics. 2014 Jan 28;15:74. doi: 10.1186/1471-2164-15-74 (PMC3909937; doi:10.1186/1471-2164-15-74)
Supplement: Additional file 5 — GSEA analysis using the KEGG pathway database. This zipped file contains confirming data of the GSEA analysis. The names of the directories containing the files were composed of the term ‘GSEA’, the name of the cell line, e.g. DLD1, SW480, or LS174T, and the pathway database (KEGG). Please use a web browser to view the files with the name ‘index.html’ in the corresponding directories to start exploring the data. [file 1471-2164-15-74-S5.zip › GSEA_KEGG_DLD1/KEGG_CYSTEINE_AND_METHIONINE_METABOLISM.html]

Details for gene set KEGG\_CYSTEINE\_AND\_METHIONINE\_METABOLISM[GSEA]

|  || Dataset | DLD1\_collapsed\_to\_symbols.class.cls#b\_versus\_bg.class.cls#b\_versus\_bg\_repos |
| Phenotype | class.cls#b\_versus\_bg\_repos |
| Upregulated in class | 0 |
| GeneSet | KEGG\_CYSTEINE\_AND\_METHIONINE\_METABOLISM |
| Enrichment Score (ES) | -0.4429115 |
| Normalized Enrichment Score (NES) | -1.3509532 |
| Nominal p-value | 0.09351145 |
| FDR q-value | 0.31491286 |
| FWER p-Value | 1.0 |
Table: GSEA Results Summary

  

Fig 1: Enrichment plot: KEGG\_CYSTEINE\_AND\_METHIONINE\_METABOLISM      
 Profile of the Running ES Score & Positions of GeneSet Members on the Rank Ordered List

  

| PROBE | GENE SYMBOL | GENE\_TITLE | RANK IN GENE LIST | RANK METRIC SCORE | RUNNING ES | CORE ENRICHMENT || 1 | CTH | CTH Entrez,  Source | cystathionase (cystathionine gamma-lyase) | 1572 | 0.115 | -0.0123 | No |
| 2 | DNMT3B | DNMT3B Entrez,  Source | DNA (cytosine-5-)-methyltransferase 3 beta | 5337 | 0.036 | -0.1839 | No |
| 3 | ADI1 | ADI1 Entrez,  Source | acireductone dioxygenase 1 | 5859 | 0.029 | -0.1932 | No |
| 4 | SDS | SDS Entrez,  Source | serine dehydratase | 6260 | 0.025 | -0.1988 | No |
| 5 | AHCYL1 | AHCYL1 Entrez,  Source | S-adenosylhomocysteine hydrolase-like 1 | 7144 | 0.016 | -0.2347 | No |
| 6 | CBS | CBS Entrez,  Source | cystathionine-beta-synthase | 7866 | 0.008 | -0.2666 | No |
| 7 | APIP | APIP Entrez,  Source | APAF1 interacting protein | 8189 | 0.005 | -0.2798 | No |
| 8 | LDHC | LDHC Entrez,  Source | lactate dehydrogenase C | 9442 | -0.006 | -0.3406 | No |
| 9 | DNMT3A | DNMT3A Entrez,  Source | DNA (cytosine-5-)-methyltransferase 3 alpha | 9818 | -0.009 | -0.3544 | No |
| 10 | TAT | TAT Entrez,  Source | tyrosine aminotransferase | 9863 | -0.009 | -0.3510 | No |
| 11 | MAT2B | MAT2B Entrez,  Source | methionine adenosyltransferase II, beta | 10068 | -0.011 | -0.3548 | No |
| 12 | LDHA | LDHA Entrez,  Source | lactate dehydrogenase A | 10698 | -0.017 | -0.3772 | No |
| 13 | LDHB | LDHB Entrez,  Source | lactate dehydrogenase B | 10770 | -0.017 | -0.3705 | No |
| 14 | MAT2A | MAT2A Entrez,  Source | methionine adenosyltransferase II, alpha | 10867 | -0.018 | -0.3646 | No |
| 15 | AMD1 | AMD1 Entrez,  Source | adenosylmethionine decarboxylase 1 | 11851 | -0.027 | -0.3990 | No |
| 16 | DNMT3L | DNMT3L Entrez,  Source | DNA (cytosine-5-)-methyltransferase 3-like | 12709 | -0.035 | -0.4220 | Yes |
| 17 | CDO1 | CDO1 Entrez,  Source | cysteine dioxygenase, type I | 12781 | -0.036 | -0.4043 | Yes |
| 18 | GOT1 | GOT1 Entrez,  Source | glutamic-oxaloacetic transaminase 1, soluble (aspartate aminotransferase 1) | 12844 | -0.037 | -0.3857 | Yes |
| 19 | LDHAL6B | LDHAL6B Entrez,  Source | lactate dehydrogenase A-like 6B | 13635 | -0.045 | -0.3994 | Yes |
| 20 | MPST | MPST Entrez,  Source | mercaptopyruvate sulfurtransferase | 14069 | -0.050 | -0.3918 | Yes |
| 21 | SMS | SMS Entrez,  Source | spermine synthase | 14549 | -0.056 | -0.3830 | Yes |
| 22 | MAT1A | MAT1A Entrez,  Source | methionine adenosyltransferase I, alpha | 15361 | -0.068 | -0.3840 | Yes |
| 23 | GOT2 | GOT2 Entrez,  Source | glutamic-oxaloacetic transaminase 2, mitochondrial (aspartate aminotransferase 2) | 15903 | -0.077 | -0.3659 | Yes |
| 24 | LDHAL6A | LDHAL6A Entrez,  Source | lactate dehydrogenase A-like 6A | 16225 | -0.083 | -0.3331 | Yes |
| 25 | AHCY | AHCY Entrez,  Source | S-adenosylhomocysteine hydrolase | 17522 | -0.112 | -0.3331 | Yes |
| 26 | DNMT1 | DNMT1 Entrez,  Source | DNA (cytosine-5-)-methyltransferase 1 | 17581 | -0.114 | -0.2684 | Yes |
| 27 | BHMT | BHMT Entrez,  Source | betaine-homocysteine methyltransferase | 17828 | -0.122 | -0.2085 | Yes |
| 28 | MTAP | MTAP Entrez,  Source | methylthioadenosine phosphorylase | 18112 | -0.132 | -0.1443 | Yes |
| 29 | MTR | MTR Entrez,  Source | 5-methyltetrahydrofolate-homocysteine methyltransferase | 18778 | -0.172 | -0.0762 | Yes |
| 30 | SRM | SRM Entrez,  Source | spermidine synthase | 19005 | -0.195 | 0.0282 | Yes |
Table: GSEA details [plain text format]

  

Fig 2: KEGG\_CYSTEINE\_AND\_METHIONINE\_METABOLISM      
 Blue-Pink O' Gram in the Space of the Analyzed GeneSet

  

Fig 3: KEGG\_CYSTEINE\_AND\_METHIONINE\_METABOLISM: Random ES distribution      
 Gene set null distribution of ES for **KEGG\_CYSTEINE\_AND\_METHIONINE\_METABOLISM**
