# Supplementary material for: Comprehensive analysis of β-catenin target genes in colorectal carcinoma cell lines with deregulated Wnt/β-catenin signaling
Source: BMC Genomics. 2014 Jan 28;15:74. doi: 10.1186/1471-2164-15-74 (PMC3909937; doi:10.1186/1471-2164-15-74)
Supplement: Additional file 5 — GSEA analysis using the KEGG pathway database. This zipped file contains confirming data of the GSEA analysis. The names of the directories containing the files were composed of the term ‘GSEA’, the name of the cell line, e.g. DLD1, SW480, or LS174T, and the pathway database (KEGG). Please use a web browser to view the files with the name ‘index.html’ in the corresponding directories to start exploring the data. [file 1471-2164-15-74-S5.zip › GSEA_KEGG_DLD1/KEGG_ENDOCYTOSIS.html]

Details for gene set KEGG\_ENDOCYTOSIS[GSEA]

|  || Dataset | DLD1\_collapsed\_to\_symbols.class.cls#b\_versus\_bg.class.cls#b\_versus\_bg\_repos |
| Phenotype | class.cls#b\_versus\_bg\_repos |
| Upregulated in class | 1 |
| GeneSet | KEGG\_ENDOCYTOSIS |
| Enrichment Score (ES) | 0.43410733 |
| Normalized Enrichment Score (NES) | 1.7566485 |
| Nominal p-value | 0.0 |
| FDR q-value | 0.12538618 |
| FWER p-Value | 0.333 |
Table: GSEA Results Summary

  

Fig 1: Enrichment plot: KEGG\_ENDOCYTOSIS      
 Profile of the Running ES Score & Positions of GeneSet Members on the Rank Ordered List

  

| PROBE | GENE SYMBOL | GENE\_TITLE | RANK IN GENE LIST | RANK METRIC SCORE | RUNNING ES | CORE ENRICHMENT || 1 | ADRB2 | ADRB2 Entrez,  Source | adrenergic, beta-2-, receptor, surface | 14 | 0.763 | 0.0614 | Yes |
| 2 | EGF | EGF Entrez,  Source | epidermal growth factor (beta-urogastrone) | 177 | 0.340 | 0.0807 | Yes |
| 3 | HSPA1A | HSPA1A Entrez,  Source | heat shock 70kDa protein 1A | 212 | 0.322 | 0.1052 | Yes |
| 4 | EHD1 | EHD1 Entrez,  Source | EH-domain containing 1 | 287 | 0.274 | 0.1237 | Yes |
| 5 | PLD1 | PLD1 Entrez,  Source | phospholipase D1, phosphatidylcholine-specific | 358 | 0.241 | 0.1397 | Yes |
| 6 | LDLR | LDLR Entrez,  Source | low density lipoprotein receptor (familial hypercholesterolemia) | 401 | 0.231 | 0.1563 | Yes |
| 7 | CLTB | CLTB Entrez,  Source | clathrin, light chain (Lcb) | 414 | 0.228 | 0.1743 | Yes |
| 8 | PIP5K1B | PIP5K1B Entrez,  Source | phosphatidylinositol-4-phosphate 5-kinase, type I, beta | 649 | 0.183 | 0.1771 | Yes |
| 9 | PSD3 | PSD3 Entrez,  Source | pleckstrin and Sec7 domain containing 3 | 698 | 0.176 | 0.1890 | Yes |
| 10 | CHMP2B | CHMP2B Entrez,  Source | chromatin modifying protein 2B | 754 | 0.171 | 0.2000 | Yes |
| 11 | DNM2 | DNM2 Entrez,  Source | dynamin 2 | 789 | 0.168 | 0.2119 | Yes |
| 12 | RAB31 | RAB31 Entrez,  Source | RAB31, member RAS oncogene family | 802 | 0.166 | 0.2248 | Yes |
| 13 | SMURF1 | SMURF1 Entrez,  Source | SMAD specific E3 ubiquitin protein ligase 1 | 808 | 0.166 | 0.2380 | Yes |
| 14 | DNM3 | DNM3 Entrez,  Source | dynamin 3 | 851 | 0.162 | 0.2490 | Yes |
| 15 | RAB11FIP1 | RAB11FIP1 Entrez,  Source | RAB11 family interacting protein 1 (class I) | 863 | 0.161 | 0.2615 | Yes |
| 16 | CBLB | CBLB Entrez,  Source | Cas-Br-M (murine) ecotropic retroviral transforming sequence b | 913 | 0.156 | 0.2717 | Yes |
| 17 | VPS37B | VPS37B Entrez,  Source | vacuolar protein sorting 37 homolog B (S. cerevisiae) | 944 | 0.154 | 0.2826 | Yes |
| 18 | ARF6 | ARF6 Entrez,  Source | ADP-ribosylation factor 6 | 972 | 0.151 | 0.2936 | Yes |
| 19 | PARD6B | PARD6B Entrez,  Source | par-6 partitioning defective 6 homolog beta (C. elegans) | 1035 | 0.147 | 0.3023 | Yes |
| 20 | STAM2 | STAM2 Entrez,  Source | signal transducing adaptor molecule (SH3 domain and ITAM motif) 2 | 1046 | 0.146 | 0.3137 | Yes |
| 21 | RAB5B | RAB5B Entrez,  Source | RAB5B, member RAS oncogene family | 1107 | 0.141 | 0.3221 | Yes |
| 22 | CBLC | CBLC Entrez,  Source | Cas-Br-M (murine) ecotropic retroviral transforming sequence c | 1363 | 0.125 | 0.3191 | Yes |
| 23 | RAB11FIP4 | RAB11FIP4 Entrez,  Source | RAB11 family interacting protein 4 (class II) | 1389 | 0.124 | 0.3279 | Yes |
| 24 | GIT2 | GIT2 Entrez,  Source | G protein-coupled receptor kinase interactor 2 | 1428 | 0.122 | 0.3358 | Yes |
| 25 | SH3KBP1 | SH3KBP1 Entrez,  Source | SH3-domain kinase binding protein 1 | 1438 | 0.121 | 0.3452 | Yes |
| 26 | VPS25 | VPS25 Entrez,  Source | vacuolar protein sorting 25 homolog (S. cerevisiae) | 1443 | 0.121 | 0.3548 | Yes |
| 27 | EPN3 | EPN3 Entrez,  Source | epsin 3 | 1672 | 0.111 | 0.3521 | Yes |
| 28 | NEDD4L | NEDD4L Entrez,  Source | neural precursor cell expressed, developmentally down-regulated 4-like | 1682 | 0.111 | 0.3607 | Yes |
| 29 | SH3GL3 | SH3GL3 Entrez,  Source | SH3-domain GRB2-like 3 | 1941 | 0.102 | 0.3557 | Yes |
| 30 | HSPA6 | HSPA6 Entrez,  Source | heat shock 70kDa protein 6 (HSP70B') | 2002 | 0.099 | 0.3607 | Yes |
| 31 | HSPA2 | HSPA2 Entrez,  Source | heat shock 70kDa protein 2 | 2021 | 0.099 | 0.3678 | Yes |
| 32 | FLT1 | FLT1 Entrez,  Source | fms-related tyrosine kinase 1 (vascular endothelial growth factor/vascular permeability factor receptor) | 2078 | 0.097 | 0.3728 | Yes |
| 33 | HLA-E | HLA-E Entrez,  Source | major histocompatibility complex, class I, E | 2101 | 0.096 | 0.3795 | Yes |
| 34 | CDC42 | CDC42 Entrez,  Source | cell division cycle 42 (GTP binding protein, 25kDa) | 2186 | 0.093 | 0.3828 | Yes |
| 35 | ITCH | ITCH Entrez,  Source | itchy homolog E3 ubiquitin protein ligase (mouse) | 2211 | 0.093 | 0.3891 | Yes |
| 36 | ARFGAP3 | ARFGAP3 Entrez,  Source | ADP-ribosylation factor GTPase activating protein 3 | 2332 | 0.090 | 0.3902 | Yes |
| 37 | SMAP1 | SMAP1 Entrez,  Source | stromal membrane-associated protein 1 | 2464 | 0.086 | 0.3904 | Yes |
| 38 | RAB22A | RAB22A Entrez,  Source | RAB22A, member RAS oncogene family | 2475 | 0.086 | 0.3969 | Yes |
| 39 | VPS37A | VPS37A Entrez,  Source | vacuolar protein sorting 37 homolog A (S. cerevisiae) | 2614 | 0.082 | 0.3965 | Yes |
| 40 | SH3GLB2 | SH3GLB2 Entrez,  Source | SH3-domain GRB2-like endophilin B2 | 2648 | 0.081 | 0.4014 | Yes |
| 41 | ERBB3 | ERBB3 Entrez,  Source | v-erb-b2 erythroblastic leukemia viral oncogene homolog 3 (avian) | 2688 | 0.080 | 0.4059 | Yes |
| 42 | SH3GL1 | SH3GL1 Entrez,  Source | SH3-domain GRB2-like 1 | 2834 | 0.077 | 0.4047 | Yes |
| 43 | VPS4B | VPS4B Entrez,  Source | vacuolar protein sorting 4 homolog B (S. cerevisiae) | 2893 | 0.076 | 0.4079 | Yes |
| 44 | IQSEC2 | IQSEC2 Entrez,  Source | IQ motif and Sec7 domain 2 | 2895 | 0.076 | 0.4140 | Yes |
| 45 | VPS37C | VPS37C Entrez,  Source | vacuolar protein sorting 37 homolog C (S. cerevisiae) | 2906 | 0.075 | 0.4196 | Yes |
| 46 | AP2B1 | AP2B1 Entrez,  Source | adaptor-related protein complex 2, beta 1 subunit | 3028 | 0.073 | 0.4192 | Yes |
| 47 | EGFR | EGFR Entrez,  Source | epidermal growth factor receptor (erythroblastic leukemia viral (v-erb-b) oncogene homolog, avian) | 3161 | 0.070 | 0.4181 | Yes |
| 48 | RAB11A | RAB11A Entrez,  Source | RAB11A, member RAS oncogene family | 3276 | 0.067 | 0.4177 | Yes |
| 49 | CHMP1B | CHMP1B Entrez,  Source | chromatin modifying protein 1B | 3292 | 0.067 | 0.4224 | Yes |
| 50 | CHMP2A | CHMP2A Entrez,  Source | chromatin modifying protein 2A | 3372 | 0.065 | 0.4236 | Yes |
| 51 | PRKCI | PRKCI Entrez,  Source | protein kinase C, iota | 3441 | 0.064 | 0.4253 | Yes |
| 52 | CHMP6 | CHMP6 Entrez,  Source | chromatin modifying protein 6 | 3449 | 0.064 | 0.4302 | Yes |
| 53 | ERBB4 | ERBB4 Entrez,  Source | v-erb-a erythroblastic leukemia viral oncogene homolog 4 (avian) | 3474 | 0.063 | 0.4341 | Yes |
| 54 | SH3GLB1 | SH3GLB1 Entrez,  Source | SH3-domain GRB2-like endophilin B1 | 3687 | 0.060 | 0.4280 | No |
| 55 | VPS37D | VPS37D Entrez,  Source | vacuolar protein sorting 37 homolog D (S. cerevisiae) | 3719 | 0.059 | 0.4313 | No |
| 56 | SRC | SRC Entrez,  Source | v-src sarcoma (Schmidt-Ruppin A-2) viral oncogene homolog (avian) | 3874 | 0.056 | 0.4279 | No |
| 57 | CHMP5 | CHMP5 Entrez,  Source | chromatin modifying protein 5 | 3915 | 0.056 | 0.4304 | No |
| 58 | EPN1 | EPN1 Entrez,  Source | epsin 1 | 4005 | 0.054 | 0.4302 | No |
| 59 | IL2RG | IL2RG Entrez,  Source | interleukin 2 receptor, gamma (severe combined immunodeficiency) | 4059 | 0.053 | 0.4318 | No |
| 60 | PIP5K1C | PIP5K1C Entrez,  Source | phosphatidylinositol-4-phosphate 5-kinase, type I, gamma | 4280 | 0.050 | 0.4246 | No |
| 61 | NTRK1 | NTRK1 Entrez,  Source | neurotrophic tyrosine kinase, receptor, type 1 | 4344 | 0.049 | 0.4253 | No |
| 62 | TSG101 | TSG101 Entrez,  Source | tumor susceptibility gene 101 | 4391 | 0.049 | 0.4269 | No |
| 63 | VPS28 | VPS28 Entrez,  Source | vacuolar protein sorting 28 homolog (S. cerevisiae) | 4402 | 0.048 | 0.4304 | No |
| 64 | PLD2 | PLD2 Entrez,  Source | phospholipase D2 | 4461 | 0.048 | 0.4312 | No |
| 65 | RAB11FIP5 | RAB11FIP5 Entrez,  Source | RAB11 family interacting protein 5 (class I) | 4488 | 0.047 | 0.4337 | No |
| 66 | CHMP4B | CHMP4B Entrez,  Source | chromatin modifying protein 4B | 4719 | 0.044 | 0.4255 | No |
| 67 | USP8 | USP8 Entrez,  Source | ubiquitin specific peptidase 8 | 4909 | 0.041 | 0.4191 | No |
| 68 | HLA-B | HLA-B Entrez,  Source | major histocompatibility complex, class I, B | 5093 | 0.039 | 0.4128 | No |
| 69 | LDLRAP1 | LDLRAP1 Entrez,  Source | low density lipoprotein receptor adaptor protein 1 | 5164 | 0.038 | 0.4122 | No |
| 70 | HSPA1L | HSPA1L Entrez,  Source | heat shock 70kDa protein 1-like | 5251 | 0.037 | 0.4108 | No |
| 71 | EEA1 | EEA1 Entrez,  Source | early endosome antigen 1, 162kD | 5493 | 0.034 | 0.4011 | No |
| 72 | CHMP4A | CHMP4A Entrez,  Source | chromatin modifying protein 4A | 5499 | 0.033 | 0.4036 | No |
| 73 | RAB5A | RAB5A Entrez,  Source | RAB5A, member RAS oncogene family | 5639 | 0.032 | 0.3990 | No |
| 74 | PARD3 | PARD3 Entrez,  Source | par-3 partitioning defective 3 homolog (C. elegans) | 5700 | 0.031 | 0.3984 | No |
| 75 | ADRB1 | ADRB1 Entrez,  Source | adrenergic, beta-1-, receptor | 5712 | 0.031 | 0.4003 | No |
| 76 | HLA-C | HLA-C Entrez,  Source | major histocompatibility complex, class I, C | 5964 | 0.028 | 0.3897 | No |
| 77 | FGFR4 | FGFR4 Entrez,  Source | fibroblast growth factor receptor 4 | 6172 | 0.026 | 0.3811 | No |
| 78 | EPS15 | EPS15 Entrez,  Source | epidermal growth factor receptor pathway substrate 15 | 6205 | 0.026 | 0.3816 | No |
| 79 | HLA-F | HLA-F Entrez,  Source | major histocompatibility complex, class I, F | 6303 | 0.024 | 0.3786 | No |
| 80 | HLA-G | HLA-G Entrez,  Source | HLA-G histocompatibility antigen, class I, G | 6327 | 0.024 | 0.3793 | No |
| 81 | CLTC | CLTC Entrez,  Source | clathrin, heavy chain (Hc) | 6356 | 0.024 | 0.3799 | No |
| 82 | AP2A2 | AP2A2 Entrez,  Source | adaptor-related protein complex 2, alpha 2 subunit | 6501 | 0.023 | 0.3743 | No |
| 83 | TRAF6 | TRAF6 Entrez,  Source | TNF receptor-associated factor 6 | 6531 | 0.022 | 0.3746 | No |
| 84 | RAB5C | RAB5C Entrez,  Source | RAB5C, member RAS oncogene family | 6776 | 0.020 | 0.3636 | No |
| 85 | RET | RET Entrez,  Source | ret proto-oncogene (multiple endocrine neoplasia and medullary thyroid carcinoma 1, Hirschsprung disease) | 6879 | 0.018 | 0.3598 | No |
| 86 | ARFGAP1 | ARFGAP1 Entrez,  Source | ADP-ribosylation factor GTPase activating protein 1 | 6911 | 0.018 | 0.3597 | No |
| 87 | PDCD6IP | PDCD6IP Entrez,  Source | programmed cell death 6 interacting protein | 7033 | 0.017 | 0.3549 | No |
| 88 | STAMBP | STAMBP Entrez,  Source | STAM binding protein | 7103 | 0.016 | 0.3526 | No |
| 89 | PRKCZ | PRKCZ Entrez,  Source | protein kinase C, zeta | 7162 | 0.016 | 0.3509 | No |
| 90 | CLTA | CLTA Entrez,  Source | clathrin, light chain (Lca) | 7248 | 0.015 | 0.3477 | No |
| 91 | STAM | STAM Entrez,  Source | signal transducing adaptor molecule (SH3 domain and ITAM motif) 1 | 7272 | 0.014 | 0.3477 | No |
| 92 | HLA-A | HLA-A Entrez,  Source | major histocompatibility complex, class I, A | 7398 | 0.013 | 0.3423 | No |
| 93 | PSD2 | PSD2 Entrez,  Source | pleckstrin and Sec7 domain containing 2 | 7507 | 0.012 | 0.3378 | No |
| 94 | SNF8 | SNF8 Entrez,  Source | SNF8, ESCRT-II complex subunit, homolog (S. cerevisiae) | 8001 | 0.007 | 0.3129 | No |
| 95 | CXCR4 | CXCR4 Entrez,  Source | chemokine (C-X-C motif) receptor 4 | 8248 | 0.005 | 0.3006 | No |
| 96 | HGS | HGS Entrez,  Source | hepatocyte growth factor-regulated tyrosine kinase substrate | 8409 | 0.003 | 0.2927 | No |
| 97 | SMURF2 | SMURF2 Entrez,  Source | SMAD specific E3 ubiquitin protein ligase 2 | 8660 | 0.001 | 0.2799 | No |
| 98 | F2R | F2R Entrez,  Source | coagulation factor II (thrombin) receptor | 8708 | 0.001 | 0.2775 | No |
| 99 | RABEP1 | RABEP1 Entrez,  Source | rabaptin, RAB GTPase binding effector protein 1 | 8833 | -0.000 | 0.2712 | No |
| 100 | HRAS | HRAS Entrez,  Source | v-Ha-ras Harvey rat sarcoma viral oncogene homolog | 8944 | -0.001 | 0.2656 | No |
| 101 | PSD4 | PSD4 Entrez,  Source | pleckstrin and Sec7 domain containing 4 | 9066 | -0.002 | 0.2596 | No |
| 102 | AP2M1 | AP2M1 Entrez,  Source | adaptor-related protein complex 2, mu 1 subunit | 9121 | -0.003 | 0.2570 | No |
| 103 | PSD | PSD Entrez,  Source | pleckstrin and Sec7 domain containing | 9210 | -0.004 | 0.2528 | No |
| 104 | GRK1 | GRK1 Entrez,  Source | G protein-coupled receptor kinase 1 | 9445 | -0.006 | 0.2412 | No |
| 105 | WWP1 | WWP1 Entrez,  Source | WW domain containing E3 ubiquitin protein ligase 1 | 9588 | -0.007 | 0.2344 | No |
| 106 | CBL | CBL Entrez,  Source | Cas-Br-M (murine) ecotropic retroviral transforming sequence | 9785 | -0.009 | 0.2250 | No |
| 107 | PARD6A | PARD6A Entrez,  Source | par-6 partitioning defective 6 homolog alpha (C.elegans) | 9822 | -0.009 | 0.2239 | No |
| 108 | FGFR3 | FGFR3 Entrez,  Source | fibroblast growth factor receptor 3 (achondroplasia, thanatophoric dwarfism) | 9899 | -0.010 | 0.2208 | No |
| 109 | HSPA8 | HSPA8 Entrez,  Source | heat shock 70kDa protein 8 | 9964 | -0.010 | 0.2183 | No |
| 110 | PDGFRA | PDGFRA Entrez,  Source | platelet-derived growth factor receptor, alpha polypeptide | 10115 | -0.011 | 0.2115 | No |
| 111 | RAB11B | RAB11B Entrez,  Source | RAB11B, member RAS oncogene family | 10267 | -0.013 | 0.2048 | No |
| 112 | ADRB3 | ADRB3 Entrez,  Source | adrenergic, beta-3-, receptor | 10440 | -0.015 | 0.1971 | No |
| 113 | FGFR2 | FGFR2 Entrez,  Source | ffer syndrome, Jackson-Weiss syndrome) | 10540 | -0.015 | 0.1932 | No |
| 114 | PIP5K1A | PIP5K1A Entrez,  Source | phosphatidylinositol-4-phosphate 5-kinase, type I, alpha | 10561 | -0.016 | 0.1935 | No |
| 115 | VPS4A | VPS4A Entrez,  Source | vacuolar protein sorting 4 homolog A (S. cerevisiae) | 10701 | -0.017 | 0.1877 | No |
| 116 | EPN2 | EPN2 Entrez,  Source | epsin 2 | 10714 | -0.017 | 0.1884 | No |
| 117 | KDR | KDR Entrez,  Source | kinase insert domain receptor (a type III receptor tyrosine kinase) | 10881 | -0.018 | 0.1813 | No |
| 118 | GRK5 | GRK5 Entrez,  Source | G protein-coupled receptor kinase 5 | 11123 | -0.020 | 0.1706 | No |
| 119 | ZFYVE20 | ZFYVE20 Entrez,  Source | zinc finger, FYVE domain containing 20 | 11337 | -0.022 | 0.1614 | No |
| 120 | CHMP4C | CHMP4C Entrez,  Source | chromatin modifying protein 4C | 11713 | -0.026 | 0.1441 | No |
| 121 | MDM2 | MDM2 Entrez,  Source | Mdm2, transformed 3T3 cell double minute 2, p53 binding protein (mouse) | 11805 | -0.026 | 0.1416 | No |
| 122 | VPS36 | VPS36 Entrez,  Source | vacuolar protein sorting 36 (yeast) | 11816 | -0.027 | 0.1432 | No |
| 123 | AP2S1 | AP2S1 Entrez,  Source | adaptor-related protein complex 2, sigma 1 subunit | 11824 | -0.027 | 0.1450 | No |
| 124 | GIT1 | GIT1 Entrez,  Source | G protein-coupled receptor kinase interactor 1 | 12060 | -0.029 | 0.1353 | No |
| 125 | DNM1L | DNM1L Entrez,  Source | dynamin 1-like | 12136 | -0.030 | 0.1338 | No |
| 126 | MET | MET Entrez,  Source | met proto-oncogene (hepatocyte growth factor receptor) | 12453 | -0.033 | 0.1202 | No |
| 127 | AP2A1 | AP2A1 Entrez,  Source | adaptor-related protein complex 2, alpha 1 subunit | 12519 | -0.033 | 0.1195 | No |
| 128 | TFRC | TFRC Entrez,  Source | transferrin receptor (p90, CD71) | 12564 | -0.034 | 0.1200 | No |
| 129 | EHD4 | EHD4 Entrez,  Source | EH-domain containing 4 | 12702 | -0.035 | 0.1158 | No |
| 130 | ADRBK1 | ADRBK1 Entrez,  Source | adrenergic, beta, receptor kinase 1 | 12747 | -0.036 | 0.1164 | No |
| 131 | CSF1R | CSF1R Entrez,  Source | colony stimulating factor 1 receptor, formerly McDonough feline sarcoma viral (v-fms) oncogene homolog | 13242 | -0.041 | 0.0943 | No |
| 132 | EHD2 | EHD2 Entrez,  Source | EH-domain containing 2 | 13303 | -0.041 | 0.0945 | No |
| 133 | GRK6 | GRK6 Entrez,  Source | G protein-coupled receptor kinase 6 | 13316 | -0.042 | 0.0973 | No |
| 134 | DNM1 | DNM1 Entrez,  Source | dynamin 1 | 13352 | -0.042 | 0.0989 | No |
| 135 | RAB4A | RAB4A Entrez,  Source | RAB4A, member RAS oncogene family | 13449 | -0.043 | 0.0975 | No |
| 136 | RUFY1 | RUFY1 Entrez,  Source | RUN and FYVE domain containing 1 | 13662 | -0.045 | 0.0902 | No |
| 137 | RAB11FIP3 | RAB11FIP3 Entrez,  Source | RAB11 family interacting protein 3 (class II) | 13686 | -0.046 | 0.0928 | No |
| 138 | RNF41 | RNF41 Entrez,  Source | ring finger protein 41 | 13887 | -0.048 | 0.0863 | No |
| 139 | DNAJC6 | DNAJC6 Entrez,  Source | DnaJ (Hsp40) homolog, subfamily C, member 6 | 13948 | -0.049 | 0.0872 | No |
| 140 | NEDD4 | NEDD4 Entrez,  Source | neural precursor cell expressed, developmentally down-regulated 4 | 14067 | -0.050 | 0.0852 | No |
| 141 | ARRB2 | ARRB2 Entrez,  Source | arrestin, beta 2 | 14118 | -0.051 | 0.0867 | No |
| 142 | IL2RA | IL2RA Entrez,  Source | interleukin 2 receptor, alpha | 14370 | -0.054 | 0.0782 | No |
| 143 | EHD3 | EHD3 Entrez,  Source | EH-domain containing 3 | 15057 | -0.063 | 0.0480 | No |
| 144 | IL2RB | IL2RB Entrez,  Source | interleukin 2 receptor, beta | 15490 | -0.070 | 0.0314 | No |
| 145 | RAB11FIP2 | RAB11FIP2 Entrez,  Source | RAB11 family interacting protein 2 (class I) | 16567 | -0.089 | -0.0168 | No |
| 146 | FAM125B | FAM125B Entrez,  Source | family with sequence similarity 125, member B | 17159 | -0.102 | -0.0389 | No |
| 147 | ADRBK2 | ADRBK2 Entrez,  Source | adrenergic, beta, receptor kinase 2 | 17262 | -0.105 | -0.0357 | No |
| 148 | GRK4 | GRK4 Entrez,  Source | G protein-coupled receptor kinase 4 | 17298 | -0.106 | -0.0289 | No |
| 149 | IGF1R | IGF1R Entrez,  Source | insulin-like growth factor 1 receptor | 17435 | -0.109 | -0.0270 | No |
| 150 | CLTCL1 | CLTCL1 Entrez,  Source | clathrin, heavy chain-like 1 | 17664 | -0.117 | -0.0293 | No |
| 151 | IQSEC1 | IQSEC1 Entrez,  Source | IQ motif and Sec7 domain 1 | 18326 | -0.143 | -0.0517 | No |
| 152 | ARRB1 | ARRB1 Entrez,  Source | arrestin, beta 1 | 18636 | -0.161 | -0.0546 | No |
| 153 | SH3GL2 | SH3GL2 Entrez,  Source | SH3-domain GRB2-like 2 | 19091 | -0.208 | -0.0611 | No |
| 154 | PARD6G | PARD6G Entrez,  Source | par-6 partitioning defective 6 homolog gamma (C. elegans) | 19277 | -0.242 | -0.0510 | No |
| 155 | DAB2 | DAB2 Entrez,  Source | disabled homolog 2, mitogen-responsive phosphoprotein (Drosophila) | 19367 | -0.280 | -0.0327 | No |
| 156 | KIT | KIT Entrez,  Source | v-kit Hardy-Zuckerman 4 feline sarcoma viral oncogene homolog | 19539 | -0.522 | 0.0009 | No |
Table: GSEA details [plain text format]

  

Fig 2: KEGG\_ENDOCYTOSIS      
 Blue-Pink O' Gram in the Space of the Analyzed GeneSet

  

Fig 3: KEGG\_ENDOCYTOSIS: Random ES distribution      
 Gene set null distribution of ES for **KEGG\_ENDOCYTOSIS**
