# Supplementary material for: Comprehensive analysis of β-catenin target genes in colorectal carcinoma cell lines with deregulated Wnt/β-catenin signaling
Source: BMC Genomics. 2014 Jan 28;15:74. doi: 10.1186/1471-2164-15-74 (PMC3909937; doi:10.1186/1471-2164-15-74)
Supplement: Additional file 5 — GSEA analysis using the KEGG pathway database. This zipped file contains confirming data of the GSEA analysis. The names of the directories containing the files were composed of the term ‘GSEA’, the name of the cell line, e.g. DLD1, SW480, or LS174T, and the pathway database (KEGG). Please use a web browser to view the files with the name ‘index.html’ in the corresponding directories to start exploring the data. [file 1471-2164-15-74-S5.zip › GSEA_KEGG_DLD1/KEGG_ERBB_SIGNALING_PATHWAY.html]

Details for gene set KEGG\_ERBB\_SIGNALING\_PATHWAY[GSEA]

|  || Dataset | DLD1\_collapsed\_to\_symbols.class.cls#b\_versus\_bg.class.cls#b\_versus\_bg\_repos |
| Phenotype | class.cls#b\_versus\_bg\_repos |
| Upregulated in class | 1 |
| GeneSet | KEGG\_ERBB\_SIGNALING\_PATHWAY |
| Enrichment Score (ES) | 0.4529102 |
| Normalized Enrichment Score (NES) | 1.7058516 |
| Nominal p-value | 0.0 |
| FDR q-value | 0.12939082 |
| FWER p-Value | 0.471 |
Table: GSEA Results Summary

  

Fig 1: Enrichment plot: KEGG\_ERBB\_SIGNALING\_PATHWAY      
 Profile of the Running ES Score & Positions of GeneSet Members on the Rank Ordered List

  

| PROBE | GENE SYMBOL | GENE\_TITLE | RANK IN GENE LIST | RANK METRIC SCORE | RUNNING ES | CORE ENRICHMENT || 1 | EGF | EGF Entrez,  Source | epidermal growth factor (beta-urogastrone) | 177 | 0.340 | 0.0427 | Yes |
| 2 | GAB1 | GAB1 Entrez,  Source | GRB2-associated binding protein 1 | 214 | 0.320 | 0.0895 | Yes |
| 3 | PIK3R3 | PIK3R3 Entrez,  Source | phosphoinositide-3-kinase, regulatory subunit 3 (p55, gamma) | 247 | 0.296 | 0.1329 | Yes |
| 4 | BTC | BTC Entrez,  Source | betacellulin | 290 | 0.272 | 0.1721 | Yes |
| 5 | HBEGF | HBEGF Entrez,  Source | heparin-binding EGF-like growth factor | 405 | 0.230 | 0.2012 | Yes |
| 6 | BAD | BAD Entrez,  Source | BCL2-antagonist of cell death | 406 | 0.230 | 0.2362 | Yes |
| 7 | ERBB2 | ERBB2 Entrez,  Source | v-erb-b2 erythroblastic leukemia viral oncogene homolog 2, neuro/glioblastoma derived oncogene homolog (avian) | 554 | 0.197 | 0.2585 | Yes |
| 8 | CAMK2D | CAMK2D Entrez,  Source | calcium/calmodulin-dependent protein kinase (CaM kinase) II delta | 636 | 0.185 | 0.2824 | Yes |
| 9 | MAPK3 | MAPK3 Entrez,  Source | mitogen-activated protein kinase 3 | 909 | 0.156 | 0.2922 | Yes |
| 10 | CBLB | CBLB Entrez,  Source | Cas-Br-M (murine) ecotropic retroviral transforming sequence b | 913 | 0.156 | 0.3157 | Yes |
| 11 | BRAF | BRAF Entrez,  Source | v-raf murine sarcoma viral oncogene homolog B1 | 1048 | 0.146 | 0.3310 | Yes |
| 12 | CBLC | CBLC Entrez,  Source | Cas-Br-M (murine) ecotropic retroviral transforming sequence c | 1363 | 0.125 | 0.3339 | Yes |
| 13 | PIK3CB | PIK3CB Entrez,  Source | phosphoinositide-3-kinase, catalytic, beta polypeptide | 1862 | 0.104 | 0.3241 | Yes |
| 14 | STAT5A | STAT5A Entrez,  Source | signal transducer and activator of transcription 5A | 1891 | 0.103 | 0.3383 | Yes |
| 15 | PIK3CA | PIK3CA Entrez,  Source | phosphoinositide-3-kinase, catalytic, alpha polypeptide | 1955 | 0.101 | 0.3504 | Yes |
| 16 | CDKN1A | CDKN1A Entrez,  Source | cyclin-dependent kinase inhibitor 1A (p21, Cip1) | 2044 | 0.098 | 0.3607 | Yes |
| 17 | NRAS | NRAS Entrez,  Source | neuroblastoma RAS viral (v-ras) oncogene homolog | 2381 | 0.088 | 0.3569 | Yes |
| 18 | SOS2 | SOS2 Entrez,  Source | son of sevenless homolog 2 (Drosophila) | 2390 | 0.088 | 0.3699 | Yes |
| 19 | CAMK2G | CAMK2G Entrez,  Source | calcium/calmodulin-dependent protein kinase (CaM kinase) II gamma | 2513 | 0.085 | 0.3765 | Yes |
| 20 | ERBB3 | ERBB3 Entrez,  Source | v-erb-b2 erythroblastic leukemia viral oncogene homolog 3 (avian) | 2688 | 0.080 | 0.3798 | Yes |
| 21 | CRK | CRK Entrez,  Source | v-crk sarcoma virus CT10 oncogene homolog (avian) | 2705 | 0.080 | 0.3911 | Yes |
| 22 | KRAS | KRAS Entrez,  Source | v-Ki-ras2 Kirsten rat sarcoma viral oncogene homolog | 2993 | 0.073 | 0.3875 | Yes |
| 23 | EGFR | EGFR Entrez,  Source | epidermal growth factor receptor (erythroblastic leukemia viral (v-erb-b) oncogene homolog, avian) | 3161 | 0.070 | 0.3896 | Yes |
| 24 | NCK1 | NCK1 Entrez,  Source | NCK adaptor protein 1 | 3211 | 0.069 | 0.3975 | Yes |
| 25 | PAK1 | PAK1 Entrez,  Source | p21/Cdc42/Rac1-activated kinase 1 (STE20 homolog, yeast) | 3265 | 0.067 | 0.4050 | Yes |
| 26 | STAT5B | STAT5B Entrez,  Source | signal transducer and activator of transcription 5B | 3341 | 0.066 | 0.4112 | Yes |
| 27 | NCK2 | NCK2 Entrez,  Source | NCK adaptor protein 2 | 3415 | 0.065 | 0.4173 | Yes |
| 28 | PTK2 | PTK2 Entrez,  Source | PTK2 protein tyrosine kinase 2 | 3440 | 0.064 | 0.4258 | Yes |
| 29 | TGFA | TGFA Entrez,  Source | transforming growth factor, alpha | 3458 | 0.064 | 0.4346 | Yes |
| 30 | ERBB4 | ERBB4 Entrez,  Source | v-erb-a erythroblastic leukemia viral oncogene homolog 4 (avian) | 3474 | 0.063 | 0.4435 | Yes |
| 31 | GSK3B | GSK3B Entrez,  Source | glycogen synthase kinase 3 beta | 3729 | 0.059 | 0.4394 | Yes |
| 32 | MAPK1 | MAPK1 Entrez,  Source | mitogen-activated protein kinase 1 | 3801 | 0.058 | 0.4445 | Yes |
| 33 | SRC | SRC Entrez,  Source | v-src sarcoma (Schmidt-Ruppin A-2) viral oncogene homolog (avian) | 3874 | 0.056 | 0.4494 | Yes |
| 34 | SOS1 | SOS1 Entrez,  Source | son of sevenless homolog 1 (Drosophila) | 4071 | 0.053 | 0.4474 | Yes |
| 35 | PRKCA | PRKCA Entrez,  Source | protein kinase C, alpha | 4248 | 0.051 | 0.4461 | Yes |
| 36 | CDKN1B | CDKN1B Entrez,  Source | cyclin-dependent kinase inhibitor 1B (p27, Kip1) | 4265 | 0.050 | 0.4529 | Yes |
| 37 | PIK3CD | PIK3CD Entrez,  Source | phosphoinositide-3-kinase, catalytic, delta polypeptide | 5186 | 0.037 | 0.4114 | No |
| 38 | SHC3 | SHC3 Entrez,  Source | SHC (Src homology 2 domain containing) transforming protein 3 | 5209 | 0.037 | 0.4159 | No |
| 39 | PAK7 | PAK7 Entrez,  Source | p21(CDKN1A)-activated kinase 7 | 5417 | 0.034 | 0.4105 | No |
| 40 | MAP2K4 | MAP2K4 Entrez,  Source | mitogen-activated protein kinase kinase 4 | 5492 | 0.034 | 0.4118 | No |
| 41 | PAK2 | PAK2 Entrez,  Source | p21 (CDKN1A)-activated kinase 2 | 5608 | 0.032 | 0.4108 | No |
| 42 | GRB2 | GRB2 Entrez,  Source | growth factor receptor-bound protein 2 | 5830 | 0.030 | 0.4039 | No |
| 43 | RAF1 | RAF1 Entrez,  Source | v-raf-1 murine leukemia viral oncogene homolog 1 | 5927 | 0.028 | 0.4033 | No |
| 44 | MAP2K1 | MAP2K1 Entrez,  Source | mitogen-activated protein kinase kinase 1 | 5929 | 0.028 | 0.4076 | No |
| 45 | PAK4 | PAK4 Entrez,  Source | p21(CDKN1A)-activated kinase 4 | 5956 | 0.028 | 0.4105 | No |
| 46 | MAPK9 | MAPK9 Entrez,  Source | mitogen-activated protein kinase 9 | 6411 | 0.023 | 0.3907 | No |
| 47 | MAP2K2 | MAP2K2 Entrez,  Source | mitogen-activated protein kinase kinase 2 | 6577 | 0.022 | 0.3856 | No |
| 48 | SHC1 | SHC1 Entrez,  Source | SHC (Src homology 2 domain containing) transforming protein 1 | 6895 | 0.018 | 0.3721 | No |
| 49 | PIK3CG | PIK3CG Entrez,  Source | phosphoinositide-3-kinase, catalytic, gamma polypeptide | 7031 | 0.017 | 0.3677 | No |
| 50 | AKT1 | AKT1 Entrez,  Source | v-akt murine thymoma viral oncogene homolog 1 | 7896 | 0.008 | 0.3246 | No |
| 51 | CAMK2B | CAMK2B Entrez,  Source | calcium/calmodulin-dependent protein kinase (CaM kinase) II beta | 7959 | 0.007 | 0.3225 | No |
| 52 | RPS6KB2 | RPS6KB2 Entrez,  Source | ribosomal protein S6 kinase, 70kDa, polypeptide 2 | 8335 | 0.004 | 0.3039 | No |
| 53 | SHC2 | SHC2 Entrez,  Source | SHC (Src homology 2 domain containing) transforming protein 2 | 8684 | 0.001 | 0.2862 | No |
| 54 | NRG3 | NRG3 Entrez,  Source | neuregulin 3 | 8918 | -0.001 | 0.2744 | No |
| 55 | HRAS | HRAS Entrez,  Source | v-Ha-ras Harvey rat sarcoma viral oncogene homolog | 8944 | -0.001 | 0.2733 | No |
| 56 | NRG4 | NRG4 Entrez,  Source | neuregulin 4 | 9088 | -0.003 | 0.2663 | No |
| 57 | PIK3R5 | PIK3R5 Entrez,  Source | phosphoinositide-3-kinase, regulatory subunit 5, p101 | 9214 | -0.004 | 0.2605 | No |
| 58 | AREG | AREG Entrez,  Source | amphiregulin (schwannoma-derived growth factor) | 9659 | -0.008 | 0.2389 | No |
| 59 | CBL | CBL Entrez,  Source | Cas-Br-M (murine) ecotropic retroviral transforming sequence | 9785 | -0.009 | 0.2338 | No |
| 60 | ARAF | ARAF Entrez,  Source | v-raf murine sarcoma 3611 viral oncogene homolog | 10441 | -0.015 | 0.2023 | No |
| 61 | MAPK8 | MAPK8 Entrez,  Source | mitogen-activated protein kinase 8 | 10468 | -0.015 | 0.2033 | No |
| 62 | NRG1 | NRG1 Entrez,  Source | neuregulin 1 | 10592 | -0.016 | 0.1994 | No |
| 63 | EREG | EREG Entrez,  Source | epiregulin | 10781 | -0.017 | 0.1923 | No |
| 64 | AKT2 | AKT2 Entrez,  Source | v-akt murine thymoma viral oncogene homolog 2 | 11074 | -0.020 | 0.1804 | No |
| 65 | PIK3R1 | PIK3R1 Entrez,  Source | phosphoinositide-3-kinase, regulatory subunit 1 (p85 alpha) | 11545 | -0.024 | 0.1599 | No |
| 66 | SHC4 | SHC4 Entrez,  Source | SHC (Src homology 2 domain containing) family, member 4 | 11757 | -0.026 | 0.1530 | No |
| 67 | RPS6KB1 | RPS6KB1 Entrez,  Source | ribosomal protein S6 kinase, 70kDa, polypeptide 1 | 11815 | -0.027 | 0.1541 | No |
| 68 | CRKL | CRKL Entrez,  Source | v-crk sarcoma virus CT10 oncogene homolog (avian)-like | 12012 | -0.028 | 0.1483 | No |
| 69 | ABL1 | ABL1 Entrez,  Source | v-abl Abelson murine leukemia viral oncogene homolog 1 | 12026 | -0.029 | 0.1520 | No |
| 70 | MAPK10 | MAPK10 Entrez,  Source | mitogen-activated protein kinase 10 | 12835 | -0.036 | 0.1160 | No |
| 71 | PLCG2 | PLCG2 Entrez,  Source | phospholipase C, gamma 2 (phosphatidylinositol-specific) | 13226 | -0.041 | 0.1022 | No |
| 72 | ABL2 | ABL2 Entrez,  Source | v-abl Abelson murine leukemia viral oncogene homolog 2 (arg, Abelson-related gene) | 13503 | -0.044 | 0.0947 | No |
| 73 | PIK3R2 | PIK3R2 Entrez,  Source | phosphoinositide-3-kinase, regulatory subunit 2 (p85 beta) | 13588 | -0.044 | 0.0971 | No |
| 74 | PAK6 | PAK6 Entrez,  Source | p21(CDKN1A)-activated kinase 6 | 14210 | -0.052 | 0.0731 | No |
| 75 | PRKCG | PRKCG Entrez,  Source | protein kinase C, gamma | 14320 | -0.053 | 0.0755 | No |
| 76 | AKT3 | AKT3 Entrez,  Source | v-akt murine thymoma viral oncogene homolog 3 (protein kinase B, gamma) | 15572 | -0.071 | 0.0221 | No |
| 77 | ELK1 | ELK1 Entrez,  Source | ELK1, member of ETS oncogene family | 15683 | -0.073 | 0.0276 | No |
| 78 | MAP2K7 | MAP2K7 Entrez,  Source | mitogen-activated protein kinase kinase 7 | 16222 | -0.083 | 0.0126 | No |
| 79 | PAK3 | PAK3 Entrez,  Source | p21 (CDKN1A)-activated kinase 3 | 16254 | -0.083 | 0.0237 | No |
| 80 | CAMK2A | CAMK2A Entrez,  Source | calcium/calmodulin-dependent protein kinase (CaM kinase) II alpha | 16776 | -0.094 | 0.0112 | No |
| 81 | JUN | JUN Entrez,  Source | jun oncogene | 16880 | -0.096 | 0.0205 | No |
| 82 | PLCG1 | PLCG1 Entrez,  Source | phospholipase C, gamma 1 | 17513 | -0.111 | 0.0049 | No |
| 83 | NRG2 | NRG2 Entrez,  Source | neuregulin 2 | 18644 | -0.161 | -0.0286 | No |
| 84 | EIF4EBP1 | EIF4EBP1 Entrez,  Source | eukaryotic translation initiation factor 4E binding protein 1 | 18757 | -0.169 | -0.0086 | No |
| 85 | MYC | MYC Entrez,  Source | v-myc myelocytomatosis viral oncogene homolog (avian) | 19447 | -0.326 | 0.0056 | No |
Table: GSEA details [plain text format]

  

Fig 2: KEGG\_ERBB\_SIGNALING\_PATHWAY      
 Blue-Pink O' Gram in the Space of the Analyzed GeneSet

  

Fig 3: KEGG\_ERBB\_SIGNALING\_PATHWAY: Random ES distribution      
 Gene set null distribution of ES for **KEGG\_ERBB\_SIGNALING\_PATHWAY**
