# Supplementary material for: Comprehensive analysis of β-catenin target genes in colorectal carcinoma cell lines with deregulated Wnt/β-catenin signaling
Source: BMC Genomics. 2014 Jan 28;15:74. doi: 10.1186/1471-2164-15-74 (PMC3909937; doi:10.1186/1471-2164-15-74)
Supplement: Additional file 5 — GSEA analysis using the KEGG pathway database. This zipped file contains confirming data of the GSEA analysis. The names of the directories containing the files were composed of the term ‘GSEA’, the name of the cell line, e.g. DLD1, SW480, or LS174T, and the pathway database (KEGG). Please use a web browser to view the files with the name ‘index.html’ in the corresponding directories to start exploring the data. [file 1471-2164-15-74-S5.zip › GSEA_KEGG_DLD1/KEGG_FC_GAMMA_R_MEDIATED_PHAGOCYTOSIS.html]

Details for gene set KEGG\_FC\_GAMMA\_R\_MEDIATED\_PHAGOCYTOSIS[GSEA]

|  || Dataset | DLD1\_collapsed\_to\_symbols.class.cls#b\_versus\_bg.class.cls#b\_versus\_bg\_repos |
| Phenotype | class.cls#b\_versus\_bg\_repos |
| Upregulated in class | 1 |
| GeneSet | KEGG\_FC\_GAMMA\_R\_MEDIATED\_PHAGOCYTOSIS |
| Enrichment Score (ES) | 0.42788023 |
| Normalized Enrichment Score (NES) | 1.6119605 |
| Nominal p-value | 0.006451613 |
| FDR q-value | 0.119284324 |
| FWER p-Value | 0.732 |
Table: GSEA Results Summary

  

Fig 1: Enrichment plot: KEGG\_FC\_GAMMA\_R\_MEDIATED\_PHAGOCYTOSIS      
 Profile of the Running ES Score & Positions of GeneSet Members on the Rank Ordered List

  

| PROBE | GENE SYMBOL | GENE\_TITLE | RANK IN GENE LIST | RANK METRIC SCORE | RUNNING ES | CORE ENRICHMENT || 1 | SCIN | SCIN Entrez,  Source | scinderin | 116 | 0.411 | 0.0532 | Yes |
| 2 | GSN | GSN Entrez,  Source | gelsolin (amyloidosis, Finnish type) | 124 | 0.405 | 0.1112 | Yes |
| 3 | PIK3R3 | PIK3R3 Entrez,  Source | phosphoinositide-3-kinase, regulatory subunit 3 (p55, gamma) | 247 | 0.296 | 0.1475 | Yes |
| 4 | VAV3 | VAV3 Entrez,  Source | vav 3 oncogene | 342 | 0.251 | 0.1788 | Yes |
| 5 | PLD1 | PLD1 Entrez,  Source | phospholipase D1, phosphatidylcholine-specific | 358 | 0.241 | 0.2127 | Yes |
| 6 | PIP5K1B | PIP5K1B Entrez,  Source | phosphatidylinositol-4-phosphate 5-kinase, type I, beta | 649 | 0.183 | 0.2242 | Yes |
| 7 | DNM2 | DNM2 Entrez,  Source | dynamin 2 | 789 | 0.168 | 0.2412 | Yes |
| 8 | PRKCE | PRKCE Entrez,  Source | protein kinase C, epsilon | 835 | 0.163 | 0.2623 | Yes |
| 9 | DNM3 | DNM3 Entrez,  Source | dynamin 3 | 851 | 0.162 | 0.2848 | Yes |
| 10 | MAPK3 | MAPK3 Entrez,  Source | mitogen-activated protein kinase 3 | 909 | 0.156 | 0.3043 | Yes |
| 11 | PRKCD | PRKCD Entrez,  Source | protein kinase C, delta | 957 | 0.153 | 0.3238 | Yes |
| 12 | ARF6 | ARF6 Entrez,  Source | ADP-ribosylation factor 6 | 972 | 0.151 | 0.3449 | Yes |
| 13 | SYK | SYK Entrez,  Source | spleen tyrosine kinase | 990 | 0.150 | 0.3657 | Yes |
| 14 | PPAP2A | PPAP2A Entrez,  Source | phosphatidic acid phosphatase type 2A | 1177 | 0.136 | 0.3757 | Yes |
| 15 | PIK3CB | PIK3CB Entrez,  Source | phosphoinositide-3-kinase, catalytic, beta polypeptide | 1862 | 0.104 | 0.3555 | Yes |
| 16 | PIK3CA | PIK3CA Entrez,  Source | phosphoinositide-3-kinase, catalytic, alpha polypeptide | 1955 | 0.101 | 0.3653 | Yes |
| 17 | ARPC1A | ARPC1A Entrez,  Source | actin related protein 2/3 complex, subunit 1A, 41kDa | 2140 | 0.095 | 0.3694 | Yes |
| 18 | CDC42 | CDC42 Entrez,  Source | cell division cycle 42 (GTP binding protein, 25kDa) | 2186 | 0.093 | 0.3806 | Yes |
| 19 | PPAP2B | PPAP2B Entrez,  Source | phosphatidic acid phosphatase type 2B | 2436 | 0.087 | 0.3803 | Yes |
| 20 | ARPC4 | ARPC4 Entrez,  Source | actin related protein 2/3 complex, subunit 4, 20kDa | 2502 | 0.085 | 0.3891 | Yes |
| 21 | VASP | VASP Entrez,  Source | vasodilator-stimulated phosphoprotein | 2568 | 0.083 | 0.3978 | Yes |
| 22 | CRK | CRK Entrez,  Source | v-crk sarcoma virus CT10 oncogene homolog (avian) | 2705 | 0.080 | 0.4023 | Yes |
| 23 | WASL | WASL Entrez,  Source | Wiskott-Aldrich syndrome-like | 2817 | 0.077 | 0.4077 | Yes |
| 24 | ARPC3 | ARPC3 Entrez,  Source | actin related protein 2/3 complex, subunit 3, 21kDa | 3085 | 0.072 | 0.4043 | Yes |
| 25 | PAK1 | PAK1 Entrez,  Source | p21/Cdc42/Rac1-activated kinase 1 (STE20 homolog, yeast) | 3265 | 0.067 | 0.4048 | Yes |
| 26 | MARCKS | MARCKS Entrez,  Source | myristoylated alanine-rich protein kinase C substrate | 3340 | 0.066 | 0.4105 | Yes |
| 27 | WASF2 | WASF2 Entrez,  Source | WAS protein family, member 2 | 3367 | 0.065 | 0.4186 | Yes |
| 28 | SPHK2 | SPHK2 Entrez,  Source | sphingosine kinase 2 | 3471 | 0.063 | 0.4224 | Yes |
| 29 | ARPC5 | ARPC5 Entrez,  Source | actin related protein 2/3 complex, subunit 5, 16kDa | 3657 | 0.060 | 0.4215 | Yes |
| 30 | MAPK1 | MAPK1 Entrez,  Source | mitogen-activated protein kinase 1 | 3801 | 0.058 | 0.4225 | Yes |
| 31 | FCGR2A | FCGR2A Entrez,  Source | Fc fragment of IgG, low affinity IIa, receptor (CD32) | 3914 | 0.056 | 0.4248 | Yes |
| 32 | PRKCA | PRKCA Entrez,  Source | protein kinase C, alpha | 4248 | 0.051 | 0.4150 | Yes |
| 33 | LIMK1 | LIMK1 Entrez,  Source | LIM domain kinase 1 | 4274 | 0.050 | 0.4209 | Yes |
| 34 | PIP5K1C | PIP5K1C Entrez,  Source | phosphatidylinositol-4-phosphate 5-kinase, type I, gamma | 4280 | 0.050 | 0.4279 | Yes |
| 35 | PLD2 | PLD2 Entrez,  Source | phospholipase D2 | 4461 | 0.048 | 0.4255 | No |
| 36 | RAC1 | RAC1 Entrez,  Source | ras-related C3 botulinum toxin substrate 1 (rho family, small GTP binding protein Rac1) | 4577 | 0.046 | 0.4262 | No |
| 37 | PIK3CD | PIK3CD Entrez,  Source | phosphoinositide-3-kinase, catalytic, delta polypeptide | 5186 | 0.037 | 0.4003 | No |
| 38 | RAC2 | RAC2 Entrez,  Source | ras-related C3 botulinum toxin substrate 2 (rho family, small GTP binding protein Rac2) | 5484 | 0.034 | 0.3899 | No |
| 39 | CFL2 | CFL2 Entrez,  Source | cofilin 2 (muscle) | 5752 | 0.030 | 0.3806 | No |
| 40 | RAF1 | RAF1 Entrez,  Source | v-raf-1 murine leukemia viral oncogene homolog 1 | 5927 | 0.028 | 0.3758 | No |
| 41 | MAP2K1 | MAP2K1 Entrez,  Source | mitogen-activated protein kinase kinase 1 | 5929 | 0.028 | 0.3798 | No |
| 42 | ARPC2 | ARPC2 Entrez,  Source | actin related protein 2/3 complex, subunit 2, 34kDa | 5978 | 0.028 | 0.3813 | No |
| 43 | GAB2 | GAB2 Entrez,  Source | GRB2-associated binding protein 2 | 6813 | 0.019 | 0.3413 | No |
| 44 | FCGR2B | FCGR2B Entrez,  Source | Fc fragment of IgG, low affinity IIb, receptor (CD32) | 6970 | 0.018 | 0.3358 | No |
| 45 | PIK3CG | PIK3CG Entrez,  Source | phosphoinositide-3-kinase, catalytic, gamma polypeptide | 7031 | 0.017 | 0.3351 | No |
| 46 | CFL1 | CFL1 Entrez,  Source | cofilin 1 (non-muscle) | 7065 | 0.017 | 0.3358 | No |
| 47 | FCGR2C | FCGR2C Entrez,  Source | Fc fragment of IgG, low affinity IIb, receptor for (CD32) | 7119 | 0.016 | 0.3354 | No |
| 48 | MARCKSL1 | MARCKSL1 Entrez,  Source | MARCKS-like 1 | 7813 | 0.009 | 0.3011 | No |
| 49 | AKT1 | AKT1 Entrez,  Source | v-akt murine thymoma viral oncogene homolog 1 | 7896 | 0.008 | 0.2981 | No |
| 50 | RPS6KB2 | RPS6KB2 Entrez,  Source | ribosomal protein S6 kinase, 70kDa, polypeptide 2 | 8335 | 0.004 | 0.2762 | No |
| 51 | LIMK2 | LIMK2 Entrez,  Source | LIM domain kinase 2 | 8518 | 0.002 | 0.2672 | No |
| 52 | PPAP2C | PPAP2C Entrez,  Source | phosphatidic acid phosphatase type 2C | 8770 | 0.000 | 0.2543 | No |
| 53 | PIK3R5 | PIK3R5 Entrez,  Source | phosphoinositide-3-kinase, regulatory subunit 5, p101 | 9214 | -0.004 | 0.2321 | No |
| 54 | PLA2G4F | PLA2G4F Entrez,  Source | phospholipase A2, group IVF | 9804 | -0.009 | 0.2031 | No |
| 55 | FCGR1A | FCGR1A Entrez,  Source | Fc fragment of IgG, high affinity Ia, receptor (CD64) | 10104 | -0.011 | 0.1894 | No |
| 56 | DOCK2 | DOCK2 Entrez,  Source | dedicator of cytokinesis 2 | 10515 | -0.015 | 0.1705 | No |
| 57 | PIP5K1A | PIP5K1A Entrez,  Source | phosphatidylinositol-4-phosphate 5-kinase, type I, alpha | 10561 | -0.016 | 0.1705 | No |
| 58 | INPP5D | INPP5D Entrez,  Source | inositol polyphosphate-5-phosphatase, 145kDa | 10829 | -0.018 | 0.1593 | No |
| 59 | AKT2 | AKT2 Entrez,  Source | v-akt murine thymoma viral oncogene homolog 2 | 11074 | -0.020 | 0.1496 | No |
| 60 | PIK3R1 | PIK3R1 Entrez,  Source | phosphoinositide-3-kinase, regulatory subunit 1 (p85 alpha) | 11545 | -0.024 | 0.1289 | No |
| 61 | ARPC5L | ARPC5L Entrez,  Source | actin related protein 2/3 complex, subunit 5-like | 11676 | -0.025 | 0.1259 | No |
| 62 | RPS6KB1 | RPS6KB1 Entrez,  Source | ribosomal protein S6 kinase, 70kDa, polypeptide 1 | 11815 | -0.027 | 0.1226 | No |
| 63 | CRKL | CRKL Entrez,  Source | v-crk sarcoma virus CT10 oncogene homolog (avian)-like | 12012 | -0.028 | 0.1166 | No |
| 64 | DNM1L | DNM1L Entrez,  Source | dynamin 1-like | 12136 | -0.030 | 0.1146 | No |
| 65 | WASF3 | WASF3 Entrez,  Source | WAS protein family, member 3 | 12356 | -0.032 | 0.1079 | No |
| 66 | PLA2G6 | PLA2G6 Entrez,  Source | phospholipase A2, group VI (cytosolic, calcium-independent) | 13148 | -0.040 | 0.0730 | No |
| 67 | PLCG2 | PLCG2 Entrez,  Source | phospholipase C, gamma 2 (phosphatidylinositol-specific) | 13226 | -0.041 | 0.0748 | No |
| 68 | DNM1 | DNM1 Entrez,  Source | dynamin 1 | 13352 | -0.042 | 0.0745 | No |
| 69 | WAS | WAS Entrez,  Source | Wiskott-Aldrich syndrome (eczema-thrombocytopenia) | 13414 | -0.043 | 0.0775 | No |
| 70 | PIK3R2 | PIK3R2 Entrez,  Source | phosphoinositide-3-kinase, regulatory subunit 2 (p85 beta) | 13588 | -0.044 | 0.0750 | No |
| 71 | VAV2 | VAV2 Entrez,  Source | vav 2 oncogene | 13661 | -0.045 | 0.0778 | No |
| 72 | SPHK1 | SPHK1 Entrez,  Source | sphingosine kinase 1 | 13939 | -0.048 | 0.0705 | No |
| 73 | PLA2G4B | PLA2G4B Entrez,  Source | phospholipase A2, group IVB (cytosolic) | 14024 | -0.050 | 0.0733 | No |
| 74 | AMPH | AMPH Entrez,  Source | amphiphysin (Stiff-Man syndrome with breast cancer 128kDa autoantigen) | 14196 | -0.051 | 0.0720 | No |
| 75 | PRKCG | PRKCG Entrez,  Source | protein kinase C, gamma | 14320 | -0.053 | 0.0733 | No |
| 76 | HCK | HCK Entrez,  Source | hemopoietic cell kinase | 15145 | -0.065 | 0.0403 | No |
| 77 | AKT3 | AKT3 Entrez,  Source | v-akt murine thymoma viral oncogene homolog 3 (protein kinase B, gamma) | 15572 | -0.071 | 0.0287 | No |
| 78 | PLA2G4D | PLA2G4D Entrez,  Source | phospholipase A2, group IVD (cytosolic) | 15901 | -0.077 | 0.0229 | No |
| 79 | LYN | LYN Entrez,  Source | v-yes-1 Yamaguchi sarcoma viral related oncogene homolog | 16091 | -0.080 | 0.0247 | No |
| 80 | WASF1 | WASF1 Entrez,  Source | WAS protein family, member 1 | 16426 | -0.087 | 0.0200 | No |
| 81 | PTPRC | PTPRC Entrez,  Source | protein tyrosine phosphatase, receptor type, C | 16980 | -0.098 | 0.0057 | No |
| 82 | PLCG1 | PLCG1 Entrez,  Source | phospholipase C, gamma 1 | 17513 | -0.111 | -0.0056 | No |
| 83 | MYO10 | MYO10 Entrez,  Source | myosin X | 17885 | -0.124 | -0.0068 | No |
| 84 | VAV1 | VAV1 Entrez,  Source | vav 1 oncogene | 18226 | -0.138 | -0.0045 | No |
| 85 | PLA2G4A | PLA2G4A Entrez,  Source | phospholipase A2, group IVA (cytosolic, calcium-dependent) | 19537 | -0.505 | 0.0010 | No |
Table: GSEA details [plain text format]

  

Fig 2: KEGG\_FC\_GAMMA\_R\_MEDIATED\_PHAGOCYTOSIS      
 Blue-Pink O' Gram in the Space of the Analyzed GeneSet

  

Fig 3: KEGG\_FC\_GAMMA\_R\_MEDIATED\_PHAGOCYTOSIS: Random ES distribution      
 Gene set null distribution of ES for **KEGG\_FC\_GAMMA\_R\_MEDIATED\_PHAGOCYTOSIS**
