# Supplementary material for: Comprehensive analysis of β-catenin target genes in colorectal carcinoma cell lines with deregulated Wnt/β-catenin signaling
Source: BMC Genomics. 2014 Jan 28;15:74. doi: 10.1186/1471-2164-15-74 (PMC3909937; doi:10.1186/1471-2164-15-74)
Supplement: Additional file 5 — GSEA analysis using the KEGG pathway database. This zipped file contains confirming data of the GSEA analysis. The names of the directories containing the files were composed of the term ‘GSEA’, the name of the cell line, e.g. DLD1, SW480, or LS174T, and the pathway database (KEGG). Please use a web browser to view the files with the name ‘index.html’ in the corresponding directories to start exploring the data. [file 1471-2164-15-74-S5.zip › GSEA_KEGG_DLD1/KEGG_GLYCEROPHOSPHOLIPID_METABOLISM.html]

Details for gene set KEGG\_GLYCEROPHOSPHOLIPID\_METABOLISM[GSEA]

|  || Dataset | DLD1\_collapsed\_to\_symbols.class.cls#b\_versus\_bg.class.cls#b\_versus\_bg\_repos |
| Phenotype | class.cls#b\_versus\_bg\_repos |
| Upregulated in class | 1 |
| GeneSet | KEGG\_GLYCEROPHOSPHOLIPID\_METABOLISM |
| Enrichment Score (ES) | 0.4307587 |
| Normalized Enrichment Score (NES) | 1.4980619 |
| Nominal p-value | 0.019354839 |
| FDR q-value | 0.17747927 |
| FWER p-Value | 0.942 |
Table: GSEA Results Summary

  

Fig 1: Enrichment plot: KEGG\_GLYCEROPHOSPHOLIPID\_METABOLISM      
 Profile of the Running ES Score & Positions of GeneSet Members on the Rank Ordered List

  

| PROBE | GENE SYMBOL | GENE\_TITLE | RANK IN GENE LIST | RANK METRIC SCORE | RUNNING ES | CORE ENRICHMENT || 1 | PLD1 | PLD1 Entrez,  Source | phospholipase D1, phosphatidylcholine-specific | 358 | 0.241 | 0.0301 | Yes |
| 2 | MBOAT2 | MBOAT2 Entrez,  Source | membrane bound O-acyltransferase domain containing 2 | 447 | 0.220 | 0.0698 | Yes |
| 3 | DGKI | DGKI Entrez,  Source | diacylglycerol kinase, iota | 541 | 0.200 | 0.1052 | Yes |
| 4 | MBOAT1 | MBOAT1 Entrez,  Source | membrane bound O-acyltransferase domain containing 1 | 629 | 0.186 | 0.1381 | Yes |
| 5 | DGKA | DGKA Entrez,  Source | diacylglycerol kinase, alpha 80kDa | 641 | 0.184 | 0.1746 | Yes |
| 6 | GPD1L | GPD1L Entrez,  Source | glycerol-3-phosphate dehydrogenase 1-like | 811 | 0.165 | 0.1992 | Yes |
| 7 | PLA2G2A | PLA2G2A Entrez,  Source | phospholipase A2, group IIA (platelets, synovial fluid) | 814 | 0.165 | 0.2322 | Yes |
| 8 | PPAP2A | PPAP2A Entrez,  Source | phosphatidic acid phosphatase type 2A | 1177 | 0.136 | 0.2410 | Yes |
| 9 | CHKA | CHKA Entrez,  Source | choline kinase alpha | 1224 | 0.133 | 0.2653 | Yes |
| 10 | DGKE | DGKE Entrez,  Source | diacylglycerol kinase, epsilon 64kDa | 1421 | 0.122 | 0.2798 | Yes |
| 11 | AGPAT3 | AGPAT3 Entrez,  Source | 1-acylglycerol-3-phosphate O-acyltransferase 3 | 1493 | 0.118 | 0.2999 | Yes |
| 12 | CDS2 | CDS2 Entrez,  Source | CDP-diacylglycerol synthase (phosphatidate cytidylyltransferase) 2 | 1772 | 0.107 | 0.3071 | Yes |
| 13 | PCYT1A | PCYT1A Entrez,  Source | phosphate cytidylyltransferase 1, choline, alpha | 1854 | 0.104 | 0.3238 | Yes |
| 14 | PCYT2 | PCYT2 Entrez,  Source | phosphate cytidylyltransferase 2, ethanolamine | 1932 | 0.102 | 0.3403 | Yes |
| 15 | LPGAT1 | LPGAT1 Entrez,  Source | lysophosphatidylglycerol acyltransferase 1 | 2037 | 0.098 | 0.3547 | Yes |
| 16 | CDIPT | CDIPT Entrez,  Source | CDP-diacylglycerol--inositol 3-phosphatidyltransferase (phosphatidylinositol synthase) | 2236 | 0.092 | 0.3631 | Yes |
| 17 | DGKQ | DGKQ Entrez,  Source | diacylglycerol kinase, theta 110kDa | 2254 | 0.092 | 0.3806 | Yes |
| 18 | DGKD | DGKD Entrez,  Source | diacylglycerol kinase, delta 130kDa | 2333 | 0.089 | 0.3946 | Yes |
| 19 | AGPAT2 | AGPAT2 Entrez,  Source | 1-acylglycerol-3-phosphate O-acyltransferase 2 (lysophosphatidic acid acyltransferase, beta) | 2393 | 0.088 | 0.4093 | Yes |
| 20 | PPAP2B | PPAP2B Entrez,  Source | phosphatidic acid phosphatase type 2B | 2436 | 0.087 | 0.4246 | Yes |
| 21 | LCAT | LCAT Entrez,  Source | lecithin-cholesterol acyltransferase | 2637 | 0.082 | 0.4308 | Yes |
| 22 | DGKB | DGKB Entrez,  Source | diacylglycerol kinase, beta 90kDa | 3424 | 0.064 | 0.4034 | No |
| 23 | PLD2 | PLD2 Entrez,  Source | phospholipase D2 | 4461 | 0.048 | 0.3598 | No |
| 24 | ETNK1 | ETNK1 Entrez,  Source | ethanolamine kinase 1 | 4550 | 0.046 | 0.3646 | No |
| 25 | GPD1 | GPD1 Entrez,  Source | glycerol-3-phosphate dehydrogenase 1 (soluble) | 4933 | 0.041 | 0.3532 | No |
| 26 | GPD2 | GPD2 Entrez,  Source | glycerol-3-phosphate dehydrogenase 2 (mitochondrial) | 5145 | 0.038 | 0.3500 | No |
| 27 | AGPAT6 | AGPAT6 Entrez,  Source | 1-acylglycerol-3-phosphate O-acyltransferase 6 (lysophosphatidic acid acyltransferase, zeta) | 5242 | 0.037 | 0.3525 | No |
| 28 | ACHE | ACHE Entrez,  Source | acetylcholinesterase (Yt blood group) | 6579 | 0.022 | 0.2883 | No |
| 29 | AGPAT1 | AGPAT1 Entrez,  Source | 1-acylglycerol-3-phosphate O-acyltransferase 1 (lysophosphatidic acid acyltransferase, alpha) | 7127 | 0.016 | 0.2635 | No |
| 30 | PLA2G2E | PLA2G2E Entrez,  Source | phospholipase A2, group IIE | 7148 | 0.016 | 0.2656 | No |
| 31 | PLA2G1B | PLA2G1B Entrez,  Source | phospholipase A2, group IB (pancreas) | 7882 | 0.008 | 0.2297 | No |
| 32 | CDS1 | CDS1 Entrez,  Source | CDP-diacylglycerol synthase (phosphatidate cytidylyltransferase) 1 | 8021 | 0.007 | 0.2240 | No |
| 33 | ETNK2 | ETNK2 Entrez,  Source | ethanolamine kinase 2 | 8035 | 0.007 | 0.2247 | No |
| 34 | PPAP2C | PPAP2C Entrez,  Source | phosphatidic acid phosphatase type 2C | 8770 | 0.000 | 0.1871 | No |
| 35 | CHAT | CHAT Entrez,  Source | choline acetyltransferase | 9085 | -0.002 | 0.1715 | No |
| 36 | DGKH | DGKH Entrez,  Source | diacylglycerol kinase, eta | 9160 | -0.003 | 0.1683 | No |
| 37 | DGKG | DGKG Entrez,  Source | diacylglycerol kinase, gamma 90kDa | 9470 | -0.006 | 0.1537 | No |
| 38 | PLA2G12A | PLA2G12A Entrez,  Source | phospholipase A2, group XIIA | 9669 | -0.008 | 0.1451 | No |
| 39 | LYPLA2 | LYPLA2 Entrez,  Source | lysophospholipase II | 9878 | -0.010 | 0.1363 | No |
| 40 | PLA2G12B | PLA2G12B Entrez,  Source | phospholipase A2, group XIIB | 10085 | -0.011 | 0.1280 | No |
| 41 | TAZ | TAZ Entrez,  Source | tafazzin (cardiomyopathy, dilated 3A (X-linked); endocardial fibroelastosis 2; Barth syndrome) | 10088 | -0.011 | 0.1302 | No |
| 42 | PLA2G10 | PLA2G10 Entrez,  Source | phospholipase A2, group X | 10566 | -0.016 | 0.1088 | No |
| 43 | LYPLA1 | LYPLA1 Entrez,  Source | lysophospholipase I | 10739 | -0.017 | 0.1034 | No |
| 44 | PHOSPHO1 | PHOSPHO1 Entrez,  Source | phosphatase, orphan 1 | 10751 | -0.017 | 0.1063 | No |
| 45 | PGS1 | PGS1 Entrez,  Source | phosphatidylglycerophosphate synthase 1 | 11439 | -0.023 | 0.0757 | No |
| 46 | PTDSS2 | PTDSS2 Entrez,  Source | phosphatidylserine synthase 2 | 11722 | -0.026 | 0.0664 | No |
| 47 | PCYT1B | PCYT1B Entrez,  Source | phosphate cytidylyltransferase 1, choline, beta | 12501 | -0.033 | 0.0331 | No |
| 48 | PLA2G2D | PLA2G2D Entrez,  Source | phospholipase A2, group IID | 12584 | -0.034 | 0.0357 | No |
| 49 | GNPAT | GNPAT Entrez,  Source | glyceronephosphate O-acyltransferase | 12649 | -0.035 | 0.0394 | No |
| 50 | DGKZ | DGKZ Entrez,  Source | diacylglycerol kinase, zeta 104kDa | 12682 | -0.035 | 0.0448 | No |
| 51 | GPAM | GPAM Entrez,  Source | glycerol-3-phosphate acyltransferase, mitochondrial | 12843 | -0.037 | 0.0439 | No |
| 52 | CHPT1 | CHPT1 Entrez,  Source | choline phosphotransferase 1 | 12986 | -0.038 | 0.0443 | No |
| 53 | PLA2G6 | PLA2G6 Entrez,  Source | phospholipase A2, group VI (cytosolic, calcium-independent) | 13148 | -0.040 | 0.0440 | No |
| 54 | PLA2G4B | PLA2G4B Entrez,  Source | phospholipase A2, group IVB (cytosolic) | 14024 | -0.050 | 0.0091 | No |
| 55 | AGPAT4 | AGPAT4 Entrez,  Source | 1-acylglycerol-3-phosphate O-acyltransferase 4 (lysophosphatidic acid acyltransferase, delta) | 15865 | -0.077 | -0.0699 | No |
| 56 | PISD | PISD Entrez,  Source | phosphatidylserine decarboxylase | 16185 | -0.082 | -0.0699 | No |
| 57 | CRLS1 | CRLS1 Entrez,  Source | cardiolipin synthase 1 | 16239 | -0.083 | -0.0558 | No |
| 58 | PLA2G5 | PLA2G5 Entrez,  Source | phospholipase A2, group V | 16861 | -0.096 | -0.0685 | No |
| 59 | PLA2G3 | PLA2G3 Entrez,  Source | phospholipase A2, group III | 17171 | -0.102 | -0.0637 | No |
| 60 | PTDSS1 | PTDSS1 Entrez,  Source | phosphatidylserine synthase 1 | 17639 | -0.116 | -0.0644 | No |
| 61 | PEMT | PEMT Entrez,  Source | phosphatidylethanolamine N-methyltransferase | 18339 | -0.143 | -0.0715 | No |
| 62 | PLA2G2F | PLA2G2F Entrez,  Source | phospholipase A2, group IIF | 18634 | -0.160 | -0.0544 | No |
| 63 | PLA2G4A | PLA2G4A Entrez,  Source | phospholipase A2, group IVA (cytosolic, calcium-dependent) | 19537 | -0.505 | 0.0010 | No |
Table: GSEA details [plain text format]

  

Fig 2: KEGG\_GLYCEROPHOSPHOLIPID\_METABOLISM      
 Blue-Pink O' Gram in the Space of the Analyzed GeneSet

  

Fig 3: KEGG\_GLYCEROPHOSPHOLIPID\_METABOLISM: Random ES distribution      
 Gene set null distribution of ES for **KEGG\_GLYCEROPHOSPHOLIPID\_METABOLISM**
