# Supplementary material for: Comprehensive analysis of β-catenin target genes in colorectal carcinoma cell lines with deregulated Wnt/β-catenin signaling
Source: BMC Genomics. 2014 Jan 28;15:74. doi: 10.1186/1471-2164-15-74 (PMC3909937; doi:10.1186/1471-2164-15-74)
Supplement: Additional file 5 — GSEA analysis using the KEGG pathway database. This zipped file contains confirming data of the GSEA analysis. The names of the directories containing the files were composed of the term ‘GSEA’, the name of the cell line, e.g. DLD1, SW480, or LS174T, and the pathway database (KEGG). Please use a web browser to view the files with the name ‘index.html’ in the corresponding directories to start exploring the data. [file 1471-2164-15-74-S5.zip › GSEA_KEGG_DLD1/KEGG_GLYCINE_SERINE_AND_THREONINE_METABOLISM.html]

Details for gene set KEGG\_GLYCINE\_SERINE\_AND\_THREONINE\_METABOLISM[GSEA]

|  || Dataset | DLD1\_collapsed\_to\_symbols.class.cls#b\_versus\_bg.class.cls#b\_versus\_bg\_repos |
| Phenotype | class.cls#b\_versus\_bg\_repos |
| Upregulated in class | 0 |
| GeneSet | KEGG\_GLYCINE\_SERINE\_AND\_THREONINE\_METABOLISM |
| Enrichment Score (ES) | -0.5377044 |
| Normalized Enrichment Score (NES) | -1.6477834 |
| Nominal p-value | 0.0018248175 |
| FDR q-value | 0.04022376 |
| FWER p-Value | 0.436 |
Table: GSEA Results Summary

  

Fig 1: Enrichment plot: KEGG\_GLYCINE\_SERINE\_AND\_THREONINE\_METABOLISM      
 Profile of the Running ES Score & Positions of GeneSet Members on the Rank Ordered List

  

| PROBE | GENE SYMBOL | GENE\_TITLE | RANK IN GENE LIST | RANK METRIC SCORE | RUNNING ES | CORE ENRICHMENT || 1 | ALAS1 | ALAS1 Entrez,  Source | aminolevulinate, delta-, synthase 1 | 1514 | 0.117 | -0.0298 | No |
| 2 | CTH | CTH Entrez,  Source | cystathionase (cystathionine gamma-lyase) | 1572 | 0.115 | 0.0142 | No |
| 3 | AGXT | AGXT Entrez,  Source | alanine-glyoxylate aminotransferase (oxalosis I; hyperoxaluria I; glycolicaciduria; serine-pyruvate aminotransferase) | 5256 | 0.037 | -0.1596 | No |
| 4 | SDS | SDS Entrez,  Source | serine dehydratase | 6260 | 0.025 | -0.2007 | No |
| 5 | AMT | AMT Entrez,  Source | aminomethyltransferase | 7472 | 0.012 | -0.2577 | No |
| 6 | GNMT | GNMT Entrez,  Source | glycine N-methyltransferase | 7761 | 0.009 | -0.2686 | No |
| 7 | CBS | CBS Entrez,  Source | cystathionine-beta-synthase | 7866 | 0.008 | -0.2705 | No |
| 8 | MAOA | MAOA Entrez,  Source | monoamine oxidase A | 8113 | 0.006 | -0.2806 | No |
| 9 | DLD | DLD Entrez,  Source | dihydrolipoamide dehydrogenase | 9628 | -0.007 | -0.3551 | No |
| 10 | GLDC | GLDC Entrez,  Source | glycine dehydrogenase (decarboxylating) | 9843 | -0.009 | -0.3622 | No |
| 11 | AGXT2 | AGXT2 Entrez,  Source | alanine-glyoxylate aminotransferase 2 | 9978 | -0.010 | -0.3649 | No |
| 12 | GLYCTK | GLYCTK Entrez,  Source | - | 10422 | -0.014 | -0.3817 | No |
| 13 | SARDH | SARDH Entrez,  Source | sarcosine dehydrogenase | 11987 | -0.028 | -0.4503 | No |
| 14 | AOC2 | AOC2 Entrez,  Source | amine oxidase, copper containing 2 (retina-specific) | 12008 | -0.028 | -0.4397 | No |
| 15 | GATM | GATM Entrez,  Source | glycine amidinotransferase (L-arginine:glycine amidinotransferase) | 12257 | -0.031 | -0.4400 | No |
| 16 | SHMT1 | SHMT1 Entrez,  Source | serine hydroxymethyltransferase 1 (soluble) | 12778 | -0.036 | -0.4519 | No |
| 17 | ALAS2 | ALAS2 Entrez,  Source | aminolevulinate, delta-, synthase 2 (sideroblastic/hypochromic anemia) | 14009 | -0.049 | -0.4948 | Yes |
| 18 | PSAT1 | PSAT1 Entrez,  Source | phosphoserine aminotransferase 1 | 14023 | -0.049 | -0.4753 | Yes |
| 19 | PSPH | PSPH Entrez,  Source | phosphoserine phosphatase | 14573 | -0.056 | -0.4804 | Yes |
| 20 | PHGDH | PHGDH Entrez,  Source | phosphoglycerate dehydrogenase | 14754 | -0.059 | -0.4655 | Yes |
| 21 | SRR | SRR Entrez,  Source | serine racemase | 15746 | -0.074 | -0.4859 | Yes |
| 22 | GAMT | GAMT Entrez,  Source | guanidinoacetate N-methyltransferase | 16372 | -0.086 | -0.4829 | Yes |
| 23 | DAO | DAO Entrez,  Source | D-amino-acid oxidase | 17443 | -0.109 | -0.4932 | Yes |
| 24 | PIPOX | PIPOX Entrez,  Source | pipecolic acid oxidase | 17750 | -0.119 | -0.4602 | Yes |
| 25 | BHMT | BHMT Entrez,  Source | betaine-homocysteine methyltransferase | 17828 | -0.122 | -0.4144 | Yes |
| 26 | SHMT2 | SHMT2 Entrez,  Source | serine hydroxymethyltransferase 2 (mitochondrial) | 17832 | -0.122 | -0.3647 | Yes |
| 27 | DMGDH | DMGDH Entrez,  Source | dimethylglycine dehydrogenase | 18047 | -0.130 | -0.3228 | Yes |
| 28 | GCAT | GCAT Entrez,  Source | glycine C-acetyltransferase (2-amino-3-ketobutyrate coenzyme A ligase) | 18329 | -0.143 | -0.2790 | Yes |
| 29 | CHDH | CHDH Entrez,  Source | choline dehydrogenase | 18375 | -0.145 | -0.2222 | Yes |
| 30 | AOC3 | AOC3 Entrez,  Source | amine oxidase, copper containing 3 (vascular adhesion protein 1) | 18906 | -0.185 | -0.1740 | Yes |
| 31 | MAOB | MAOB Entrez,  Source | monoamine oxidase B | 19538 | -0.508 | 0.0009 | Yes |
Table: GSEA details [plain text format]

  

Fig 2: KEGG\_GLYCINE\_SERINE\_AND\_THREONINE\_METABOLISM      
 Blue-Pink O' Gram in the Space of the Analyzed GeneSet

  

Fig 3: KEGG\_GLYCINE\_SERINE\_AND\_THREONINE\_METABOLISM: Random ES distribution      
 Gene set null distribution of ES for **KEGG\_GLYCINE\_SERINE\_AND\_THREONINE\_METABOLISM**
