# Supplementary material for: Comprehensive analysis of β-catenin target genes in colorectal carcinoma cell lines with deregulated Wnt/β-catenin signaling
Source: BMC Genomics. 2014 Jan 28;15:74. doi: 10.1186/1471-2164-15-74 (PMC3909937; doi:10.1186/1471-2164-15-74)
Supplement: Additional file 5 — GSEA analysis using the KEGG pathway database. This zipped file contains confirming data of the GSEA analysis. The names of the directories containing the files were composed of the term ‘GSEA’, the name of the cell line, e.g. DLD1, SW480, or LS174T, and the pathway database (KEGG). Please use a web browser to view the files with the name ‘index.html’ in the corresponding directories to start exploring the data. [file 1471-2164-15-74-S5.zip › GSEA_KEGG_DLD1/KEGG_GLYCOSPHINGOLIPID_BIOSYNTHESIS_LACTO_AND_NEOLACTO_SERIES.html]

Details for gene set KEGG\_GLYCOSPHINGOLIPID\_BIOSYNTHESIS\_LACTO\_AND\_NEOLACTO\_SERIES[GSEA]

|  || Dataset | DLD1\_collapsed\_to\_symbols.class.cls#b\_versus\_bg.class.cls#b\_versus\_bg\_repos |
| Phenotype | class.cls#b\_versus\_bg\_repos |
| Upregulated in class | 1 |
| GeneSet | KEGG\_GLYCOSPHINGOLIPID\_BIOSYNTHESIS\_LACTO\_AND\_NEOLACTO\_SERIES |
| Enrichment Score (ES) | 0.58404666 |
| Normalized Enrichment Score (NES) | 1.7200497 |
| Nominal p-value | 0.015350877 |
| FDR q-value | 0.13550714 |
| FWER p-Value | 0.424 |
Table: GSEA Results Summary

  

Fig 1: Enrichment plot: KEGG\_GLYCOSPHINGOLIPID\_BIOSYNTHESIS\_LACTO\_AND\_NEOLACTO\_SERIES      
 Profile of the Running ES Score & Positions of GeneSet Members on the Rank Ordered List

  

| PROBE | GENE SYMBOL | GENE\_TITLE | RANK IN GENE LIST | RANK METRIC SCORE | RUNNING ES | CORE ENRICHMENT || 1 | ST3GAL4 | ST3GAL4 Entrez,  Source | ST3 beta-galactoside alpha-2,3-sialyltransferase 4 | 123 | 0.406 | 0.1471 | Yes |
| 2 | B3GNT3 | B3GNT3 Entrez,  Source | UDP-GlcNAc:betaGal beta-1,3-N-acetylglucosaminyltransferase 3 | 215 | 0.319 | 0.2632 | Yes |
| 3 | FUT3 | FUT3 Entrez,  Source | fucosyltransferase 3 (galactoside 3(4)-L-fucosyltransferase, Lewis blood group) | 318 | 0.263 | 0.3573 | Yes |
| 4 | GCNT2 | GCNT2 Entrez,  Source | glucosaminyl (N-acetyl) transferase 2, I-branching enzyme (I blood group) | 528 | 0.203 | 0.4236 | Yes |
| 5 | B3GNT5 | B3GNT5 Entrez,  Source | UDP-GlcNAc:betaGal beta-1,3-N-acetylglucosaminyltransferase 5 | 607 | 0.189 | 0.4910 | Yes |
| 6 | FUT1 | FUT1 Entrez,  Source | fucosyltransferase 1 (galactoside 2-alpha-L-fucosyltransferase, H blood group) | 970 | 0.152 | 0.5299 | Yes |
| 7 | FUT2 | FUT2 Entrez,  Source | fucosyltransferase 2 (secretor status included) | 1601 | 0.114 | 0.5406 | Yes |
| 8 | B4GALT4 | B4GALT4 Entrez,  Source | UDP-Gal:betaGlcNAc beta 1,4- galactosyltransferase, polypeptide 4 | 1873 | 0.103 | 0.5658 | Yes |
| 9 | B3GALT2 | B3GALT2 Entrez,  Source | UDP-Gal:betaGlcNAc beta 1,3-galactosyltransferase, polypeptide 2 | 2870 | 0.076 | 0.5436 | Yes |
| 10 | B4GALT1 | B4GALT1 Entrez,  Source | UDP-Gal:betaGlcNAc beta 1,4- galactosyltransferase, polypeptide 1 | 3070 | 0.072 | 0.5606 | Yes |
| 11 | B3GNT2 | B3GNT2 Entrez,  Source | UDP-GlcNAc:betaGal beta-1,3-N-acetylglucosaminyltransferase 2 | 3132 | 0.070 | 0.5840 | Yes |
| 12 | FUT5 | FUT5 Entrez,  Source | fucosyltransferase 5 (alpha (1,3) fucosyltransferase) | 3611 | 0.061 | 0.5826 | No |
| 13 | FUT6 | FUT6 Entrez,  Source | fucosyltransferase 6 (alpha (1,3) fucosyltransferase) | 6668 | 0.021 | 0.4341 | No |
| 14 | B3GNT1 | B3GNT1 Entrez,  Source | UDP-GlcNAc:betaGal beta-1,3-N-acetylglucosaminyltransferase 1 | 7916 | 0.008 | 0.3732 | No |
| 15 | FUT9 | FUT9 Entrez,  Source | fucosyltransferase 9 (alpha (1,3) fucosyltransferase) | 9028 | -0.002 | 0.3171 | No |
| 16 | ST3GAL3 | ST3GAL3 Entrez,  Source | ST3 beta-galactoside alpha-2,3-sialyltransferase 3 | 10429 | -0.015 | 0.2509 | No |
| 17 | FUT7 | FUT7 Entrez,  Source | fucosyltransferase 7 (alpha (1,3) fucosyltransferase) | 11571 | -0.024 | 0.2017 | No |
| 18 | ABO | ABO Entrez,  Source | ABO blood group (transferase A, alpha 1-3-N-acetylgalactosaminyltransferase; transferase B, alpha 1-3-galactosyltransferase) | 11731 | -0.026 | 0.2033 | No |
| 19 | B3GNT4 | B3GNT4 Entrez,  Source | UDP-GlcNAc:betaGal beta-1,3-N-acetylglucosaminyltransferase 4 | 13115 | -0.039 | 0.1474 | No |
| 20 | B3GALT1 | B3GALT1 Entrez,  Source | UDP-Gal:betaGlcNAc beta 1,3-galactosyltransferase, polypeptide 1 | 13691 | -0.046 | 0.1352 | No |
| 21 | B3GALT5 | B3GALT5 Entrez,  Source | UDP-Gal:betaGlcNAc beta 1,3-galactosyltransferase, polypeptide 5 | 13854 | -0.047 | 0.1448 | No |
| 22 | B4GALT2 | B4GALT2 Entrez,  Source | UDP-Gal:betaGlcNAc beta 1,4- galactosyltransferase, polypeptide 2 | 13982 | -0.049 | 0.1569 | No |
| 23 | B4GALT3 | B4GALT3 Entrez,  Source | UDP-Gal:betaGlcNAc beta 1,4- galactosyltransferase, polypeptide 3 | 14148 | -0.051 | 0.1677 | No |
| 24 | ST8SIA1 | ST8SIA1 Entrez,  Source | ST8 alpha-N-acetyl-neuraminide alpha-2,8-sialyltransferase 1 | 14188 | -0.051 | 0.1852 | No |
| 25 | FUT4 | FUT4 Entrez,  Source | fucosyltransferase 4 (alpha (1,3) fucosyltransferase, myeloid-specific) | 17166 | -0.102 | 0.0714 | No |
| 26 | ST3GAL6 | ST3GAL6 Entrez,  Source | ST3 beta-galactoside alpha-2,3-sialyltransferase 6 | 18160 | -0.135 | 0.0715 | No |
Table: GSEA details [plain text format]

  

Fig 2: KEGG\_GLYCOSPHINGOLIPID\_BIOSYNTHESIS\_LACTO\_AND\_NEOLACTO\_SERIES      
 Blue-Pink O' Gram in the Space of the Analyzed GeneSet

  

Fig 3: KEGG\_GLYCOSPHINGOLIPID\_BIOSYNTHESIS\_LACTO\_AND\_NEOLACTO\_SERIES: Random ES distribution      
 Gene set null distribution of ES for **KEGG\_GLYCOSPHINGOLIPID\_BIOSYNTHESIS\_LACTO\_AND\_NEOLACTO\_SERIES**
