# Supplementary material for: Comprehensive analysis of β-catenin target genes in colorectal carcinoma cell lines with deregulated Wnt/β-catenin signaling
Source: BMC Genomics. 2014 Jan 28;15:74. doi: 10.1186/1471-2164-15-74 (PMC3909937; doi:10.1186/1471-2164-15-74)
Supplement: Additional file 5 — GSEA analysis using the KEGG pathway database. This zipped file contains confirming data of the GSEA analysis. The names of the directories containing the files were composed of the term ‘GSEA’, the name of the cell line, e.g. DLD1, SW480, or LS174T, and the pathway database (KEGG). Please use a web browser to view the files with the name ‘index.html’ in the corresponding directories to start exploring the data. [file 1471-2164-15-74-S5.zip › GSEA_KEGG_DLD1/KEGG_HOMOLOGOUS_RECOMBINATION.html]

Details for gene set KEGG\_HOMOLOGOUS\_RECOMBINATION[GSEA]

|  || Dataset | DLD1\_collapsed\_to\_symbols.class.cls#b\_versus\_bg.class.cls#b\_versus\_bg\_repos |
| Phenotype | class.cls#b\_versus\_bg\_repos |
| Upregulated in class | 0 |
| GeneSet | KEGG\_HOMOLOGOUS\_RECOMBINATION |
| Enrichment Score (ES) | -0.6578353 |
| Normalized Enrichment Score (NES) | -1.9107811 |
| Nominal p-value | 0.0 |
| FDR q-value | 0.0049583972 |
| FWER p-Value | 0.024 |
Table: GSEA Results Summary

  

Fig 1: Enrichment plot: KEGG\_HOMOLOGOUS\_RECOMBINATION      
 Profile of the Running ES Score & Positions of GeneSet Members on the Rank Ordered List

  

| PROBE | GENE SYMBOL | GENE\_TITLE | RANK IN GENE LIST | RANK METRIC SCORE | RUNNING ES | CORE ENRICHMENT || 1 | POLD4 | POLD4 Entrez,  Source | polymerase (DNA-directed), delta 4 | 403 | 0.230 | 0.0534 | No |
| 2 | SHFM1 | SHFM1 Entrez,  Source | split hand/foot malformation (ectrodactyly) type 1 | 9552 | -0.007 | -0.4128 | No |
| 3 | MUS81 | MUS81 Entrez,  Source | MUS81 endonuclease homolog (S. cerevisiae) | 10687 | -0.017 | -0.4656 | No |
| 4 | RAD50 | RAD50 Entrez,  Source | RAD50 homolog (S. cerevisiae) | 11166 | -0.020 | -0.4835 | No |
| 5 | RAD52 | RAD52 Entrez,  Source | RAD52 homolog (S. cerevisiae) | 11873 | -0.027 | -0.5109 | No |
| 6 | XRCC2 | XRCC2 Entrez,  Source | X-ray repair complementing defective repair in Chinese hamster cells 2 | 12326 | -0.031 | -0.5240 | No |
| 7 | EME1 | EME1 Entrez,  Source | essential meiotic endonuclease 1 homolog 1 (S. pombe) | 12455 | -0.033 | -0.5201 | No |
| 8 | NBN | NBN Entrez,  Source | nibrin | 13569 | -0.044 | -0.5628 | No |
| 9 | RPA1 | RPA1 Entrez,  Source | replication protein A1, 70kDa | 13670 | -0.045 | -0.5534 | No |
| 10 | SSBP1 | SSBP1 Entrez,  Source | single-stranded DNA binding protein 1 | 13868 | -0.048 | -0.5482 | No |
| 11 | RPA3 | RPA3 Entrez,  Source | replication protein A3, 14kDa | 14651 | -0.057 | -0.5697 | No |
| 12 | RAD51C | RAD51C Entrez,  Source | RAD51 homolog C (S. cerevisiae) | 16373 | -0.086 | -0.6302 | Yes |
| 13 | TOP3A | TOP3A Entrez,  Source | topoisomerase (DNA) III alpha | 16624 | -0.091 | -0.6139 | Yes |
| 14 | TOP3B | TOP3B Entrez,  Source | topoisomerase (DNA) III beta | 17261 | -0.105 | -0.6127 | Yes |
| 15 | RPA4 | RPA4 Entrez,  Source | replication protein A4, 34kDa | 17899 | -0.124 | -0.6054 | Yes |
| 16 | POLD2 | POLD2 Entrez,  Source | polymerase (DNA directed), delta 2, regulatory subunit 50kDa | 18279 | -0.140 | -0.5798 | Yes |
| 17 | RAD54L | RAD54L Entrez,  Source | RAD54-like (S. cerevisiae) | 18543 | -0.154 | -0.5437 | Yes |
| 18 | RAD51 | RAD51 Entrez,  Source | RAD51 homolog (RecA homolog, E. coli) (S. cerevisiae) | 18569 | -0.156 | -0.4949 | Yes |
| 19 | RPA2 | RPA2 Entrez,  Source | replication protein A2, 32kDa | 18689 | -0.164 | -0.4482 | Yes |
| 20 | POLD3 | POLD3 Entrez,  Source | polymerase (DNA-directed), delta 3, accessory subunit | 18829 | -0.177 | -0.3983 | Yes |
| 21 | MRE11A | MRE11A Entrez,  Source | MRE11 meiotic recombination 11 homolog A (S. cerevisiae) | 19006 | -0.195 | -0.3445 | Yes |
| 22 | BRCA2 | BRCA2 Entrez,  Source | breast cancer 2, early onset | 19039 | -0.200 | -0.2819 | Yes |
| 23 | POLD1 | POLD1 Entrez,  Source | polymerase (DNA directed), delta 1, catalytic subunit 125kDa | 19075 | -0.205 | -0.2177 | Yes |
| 24 | XRCC3 | XRCC3 Entrez,  Source | X-ray repair complementing defective repair in Chinese hamster cells 3 | 19206 | -0.225 | -0.1521 | Yes |
| 25 | RAD54B | RAD54B Entrez,  Source | RAD54 homolog B (S. cerevisiae) | 19320 | -0.258 | -0.0748 | Yes |
| 26 | BLM | BLM Entrez,  Source | Bloom syndrome | 19343 | -0.270 | 0.0109 | Yes |
Table: GSEA details [plain text format]

  

Fig 2: KEGG\_HOMOLOGOUS\_RECOMBINATION      
 Blue-Pink O' Gram in the Space of the Analyzed GeneSet

  

Fig 3: KEGG\_HOMOLOGOUS\_RECOMBINATION: Random ES distribution      
 Gene set null distribution of ES for **KEGG\_HOMOLOGOUS\_RECOMBINATION**
