# Supplementary material for: Comprehensive analysis of β-catenin target genes in colorectal carcinoma cell lines with deregulated Wnt/β-catenin signaling
Source: BMC Genomics. 2014 Jan 28;15:74. doi: 10.1186/1471-2164-15-74 (PMC3909937; doi:10.1186/1471-2164-15-74)
Supplement: Additional file 5 — GSEA analysis using the KEGG pathway database. This zipped file contains confirming data of the GSEA analysis. The names of the directories containing the files were composed of the term ‘GSEA’, the name of the cell line, e.g. DLD1, SW480, or LS174T, and the pathway database (KEGG). Please use a web browser to view the files with the name ‘index.html’ in the corresponding directories to start exploring the data. [file 1471-2164-15-74-S5.zip › GSEA_KEGG_DLD1/KEGG_INOSITOL_PHOSPHATE_METABOLISM.html]

Details for gene set KEGG\_INOSITOL\_PHOSPHATE\_METABOLISM[GSEA]

|  || Dataset | DLD1\_collapsed\_to\_symbols.class.cls#b\_versus\_bg.class.cls#b\_versus\_bg\_repos |
| Phenotype | class.cls#b\_versus\_bg\_repos |
| Upregulated in class | 1 |
| GeneSet | KEGG\_INOSITOL\_PHOSPHATE\_METABOLISM |
| Enrichment Score (ES) | 0.46356866 |
| Normalized Enrichment Score (NES) | 1.5698596 |
| Nominal p-value | 0.018099548 |
| FDR q-value | 0.13520499 |
| FWER p-Value | 0.826 |
Table: GSEA Results Summary

  

Fig 1: Enrichment plot: KEGG\_INOSITOL\_PHOSPHATE\_METABOLISM      
 Profile of the Running ES Score & Positions of GeneSet Members on the Rank Ordered List

  

| PROBE | GENE SYMBOL | GENE\_TITLE | RANK IN GENE LIST | RANK METRIC SCORE | RUNNING ES | CORE ENRICHMENT || 1 | PLCE1 | PLCE1 Entrez,  Source | phospholipase C, epsilon 1 | 41 | 0.610 | 0.1606 | Yes |
| 2 | PLCD3 | PLCD3 Entrez,  Source | phospholipase C, delta 3 | 279 | 0.278 | 0.2227 | Yes |
| 3 | PIP5K1B | PIP5K1B Entrez,  Source | phosphatidylinositol-4-phosphate 5-kinase, type I, beta | 649 | 0.183 | 0.2526 | Yes |
| 4 | SYNJ1 | SYNJ1 Entrez,  Source | synaptojanin 1 | 1037 | 0.147 | 0.2719 | Yes |
| 5 | ALDH6A1 | ALDH6A1 Entrez,  Source | aldehyde dehydrogenase 6 family, member A1 | 1085 | 0.143 | 0.3076 | Yes |
| 6 | IPMK | IPMK Entrez,  Source | inositol polyphosphate multikinase | 1295 | 0.129 | 0.3312 | Yes |
| 7 | PIK3CB | PIK3CB Entrez,  Source | phosphoinositide-3-kinase, catalytic, beta polypeptide | 1862 | 0.104 | 0.3298 | Yes |
| 8 | PIK3CA | PIK3CA Entrez,  Source | phosphoinositide-3-kinase, catalytic, alpha polypeptide | 1955 | 0.101 | 0.3520 | Yes |
| 9 | PLCB3 | PLCB3 Entrez,  Source | phospholipase C, beta 3 (phosphatidylinositol-specific) | 2167 | 0.094 | 0.3662 | Yes |
| 10 | INPP5A | INPP5A Entrez,  Source | inositol polyphosphate-5-phosphatase, 40kDa | 2215 | 0.093 | 0.3885 | Yes |
| 11 | CDIPT | CDIPT Entrez,  Source | CDP-diacylglycerol--inositol 3-phosphatidyltransferase (phosphatidylinositol synthase) | 2236 | 0.092 | 0.4120 | Yes |
| 12 | ISYNA1 | ISYNA1 Entrez,  Source | - | 2461 | 0.086 | 0.4235 | Yes |
| 13 | PIK3C2G | PIK3C2G Entrez,  Source | phosphoinositide-3-kinase, class 2, gamma polypeptide | 2678 | 0.081 | 0.4339 | Yes |
| 14 | MINPP1 | MINPP1 Entrez,  Source | multiple inositol polyphosphate histidine phosphatase, 1 | 2841 | 0.077 | 0.4460 | Yes |
| 15 | SYNJ2 | SYNJ2 Entrez,  Source | synaptojanin 2 | 3032 | 0.073 | 0.4557 | Yes |
| 16 | PLCD1 | PLCD1 Entrez,  Source | phospholipase C, delta 1 | 3396 | 0.065 | 0.4544 | Yes |
| 17 | ITPK1 | ITPK1 Entrez,  Source | inositol 1,3,4-triphosphate 5/6 kinase | 3541 | 0.062 | 0.4636 | Yes |
| 18 | PIP5K1C | PIP5K1C Entrez,  Source | phosphatidylinositol-4-phosphate 5-kinase, type I, gamma | 4280 | 0.050 | 0.4391 | No |
| 19 | IMPA1 | IMPA1 Entrez,  Source | inositol(myo)-1(or 4)-monophosphatase 1 | 4583 | 0.046 | 0.4358 | No |
| 20 | ITPKA | ITPKA Entrez,  Source | inositol 1,4,5-trisphosphate 3-kinase A | 4625 | 0.045 | 0.4458 | No |
| 21 | PIK3CD | PIK3CD Entrez,  Source | phosphoinositide-3-kinase, catalytic, delta polypeptide | 5186 | 0.037 | 0.4271 | No |
| 22 | PTEN | PTEN Entrez,  Source | phosphatase and tensin homolog (mutated in multiple advanced cancers 1) | 5375 | 0.035 | 0.4268 | No |
| 23 | IPPK | IPPK Entrez,  Source | inositol 1,3,4,5,6-pentakisphosphate 2-kinase | 5782 | 0.030 | 0.4140 | No |
| 24 | OCRL | OCRL Entrez,  Source | oculocerebrorenal syndrome of Lowe | 5935 | 0.028 | 0.4138 | No |
| 25 | PLCB1 | PLCB1 Entrez,  Source | phospholipase C, beta 1 (phosphoinositide-specific) | 6225 | 0.025 | 0.4057 | No |
| 26 | PIK3C2B | PIK3C2B Entrez,  Source | phosphoinositide-3-kinase, class 2, beta polypeptide | 6917 | 0.018 | 0.3751 | No |
| 27 | PIK3CG | PIK3CG Entrez,  Source | phosphoinositide-3-kinase, catalytic, gamma polypeptide | 7031 | 0.017 | 0.3738 | No |
| 28 | PIK3C2A | PIK3C2A Entrez,  Source | phosphoinositide-3-kinase, class 2, alpha polypeptide | 7377 | 0.013 | 0.3597 | No |
| 29 | PIK3C3 | PIK3C3 Entrez,  Source | phosphoinositide-3-kinase, class 3 | 7648 | 0.011 | 0.3487 | No |
| 30 | INPPL1 | INPPL1 Entrez,  Source | inositol polyphosphate phosphatase-like 1 | 9404 | -0.005 | 0.2602 | No |
| 31 | INPP1 | INPP1 Entrez,  Source | inositol polyphosphate-1-phosphatase | 9631 | -0.007 | 0.2506 | No |
| 32 | PLCB4 | PLCB4 Entrez,  Source | phospholipase C, beta 4 | 9697 | -0.008 | 0.2494 | No |
| 33 | INPP5E | INPP5E Entrez,  Source | inositol polyphosphate-5-phosphatase, 72 kDa | 10542 | -0.015 | 0.2102 | No |
| 34 | PIP5K1A | PIP5K1A Entrez,  Source | phosphatidylinositol-4-phosphate 5-kinase, type I, alpha | 10561 | -0.016 | 0.2134 | No |
| 35 | MIOX | MIOX Entrez,  Source | myo-inositol oxygenase | 11036 | -0.020 | 0.1943 | No |
| 36 | TPI1 | TPI1 Entrez,  Source | triosephosphate isomerase 1 | 12301 | -0.031 | 0.1378 | No |
| 37 | PLCG2 | PLCG2 Entrez,  Source | phospholipase C, gamma 2 (phosphatidylinositol-specific) | 13226 | -0.041 | 0.1013 | No |
| 38 | INPP4A | INPP4A Entrez,  Source | inositol polyphosphate-4-phosphatase, type I, 107kDa | 14618 | -0.057 | 0.0452 | No |
| 39 | PLCB2 | PLCB2 Entrez,  Source | phospholipase C, beta 2 | 14920 | -0.062 | 0.0462 | No |
| 40 | ITPKB | ITPKB Entrez,  Source | inositol 1,4,5-trisphosphate 3-kinase B | 15343 | -0.068 | 0.0426 | No |
| 41 | INPP4B | INPP4B Entrez,  Source | inositol polyphosphate-4-phosphatase, type II, 105kDa | 15479 | -0.070 | 0.0543 | No |
| 42 | PLCD4 | PLCD4 Entrez,  Source | phospholipase C, delta 4 | 15794 | -0.075 | 0.0583 | No |
| 43 | PLCG1 | PLCG1 Entrez,  Source | phospholipase C, gamma 1 | 17513 | -0.111 | -0.0001 | No |
| 44 | INPP5B | INPP5B Entrez,  Source | inositol polyphosphate-5-phosphatase, 75kDa | 17560 | -0.113 | 0.0277 | No |
| 45 | IMPA2 | IMPA2 Entrez,  Source | inositol(myo)-1(or 4)-monophosphatase 2 | 18272 | -0.140 | 0.0285 | No |
| 46 | PLCZ1 | PLCZ1 Entrez,  Source | phospholipase C, zeta 1 | 18276 | -0.140 | 0.0656 | No |
Table: GSEA details [plain text format]

  

Fig 2: KEGG\_INOSITOL\_PHOSPHATE\_METABOLISM      
 Blue-Pink O' Gram in the Space of the Analyzed GeneSet

  

Fig 3: KEGG\_INOSITOL\_PHOSPHATE\_METABOLISM: Random ES distribution      
 Gene set null distribution of ES for **KEGG\_INOSITOL\_PHOSPHATE\_METABOLISM**
