# Supplementary material for: Comprehensive analysis of β-catenin target genes in colorectal carcinoma cell lines with deregulated Wnt/β-catenin signaling
Source: BMC Genomics. 2014 Jan 28;15:74. doi: 10.1186/1471-2164-15-74 (PMC3909937; doi:10.1186/1471-2164-15-74)
Supplement: Additional file 5 — GSEA analysis using the KEGG pathway database. This zipped file contains confirming data of the GSEA analysis. The names of the directories containing the files were composed of the term ‘GSEA’, the name of the cell line, e.g. DLD1, SW480, or LS174T, and the pathway database (KEGG). Please use a web browser to view the files with the name ‘index.html’ in the corresponding directories to start exploring the data. [file 1471-2164-15-74-S5.zip › GSEA_KEGG_DLD1/KEGG_INSULIN_SIGNALING_PATHWAY.html]

Details for gene set KEGG\_INSULIN\_SIGNALING\_PATHWAY[GSEA]

|  || Dataset | DLD1\_collapsed\_to\_symbols.class.cls#b\_versus\_bg.class.cls#b\_versus\_bg\_repos |
| Phenotype | class.cls#b\_versus\_bg\_repos |
| Upregulated in class | 1 |
| GeneSet | KEGG\_INSULIN\_SIGNALING\_PATHWAY |
| Enrichment Score (ES) | 0.4202642 |
| Normalized Enrichment Score (NES) | 1.650484 |
| Nominal p-value | 0.0 |
| FDR q-value | 0.13223387 |
| FWER p-Value | 0.639 |
Table: GSEA Results Summary

  

Fig 1: Enrichment plot: KEGG\_INSULIN\_SIGNALING\_PATHWAY      
 Profile of the Running ES Score & Positions of GeneSet Members on the Rank Ordered List

  

| PROBE | GENE SYMBOL | GENE\_TITLE | RANK IN GENE LIST | RANK METRIC SCORE | RUNNING ES | CORE ENRICHMENT || 1 | PRKAR2B | PRKAR2B Entrez,  Source | protein kinase, cAMP-dependent, regulatory, type II, beta | 30 | 0.654 | 0.0728 | Yes |
| 2 | PCK1 | PCK1 Entrez,  Source | phosphoenolpyruvate carboxykinase 1 (soluble) | 43 | 0.603 | 0.1408 | Yes |
| 3 | PIK3R3 | PIK3R3 Entrez,  Source | phosphoinositide-3-kinase, regulatory subunit 3 (p55, gamma) | 247 | 0.296 | 0.1641 | Yes |
| 4 | BAD | BAD Entrez,  Source | BCL2-antagonist of cell death | 406 | 0.230 | 0.1821 | Yes |
| 5 | CALML5 | CALML5 Entrez,  Source | calmodulin-like 5 | 467 | 0.216 | 0.2036 | Yes |
| 6 | SOCS2 | SOCS2 Entrez,  Source | suppressor of cytokine signaling 2 | 838 | 0.162 | 0.2030 | Yes |
| 7 | PHKB | PHKB Entrez,  Source | phosphorylase kinase, beta | 908 | 0.156 | 0.2172 | Yes |
| 8 | MAPK3 | MAPK3 Entrez,  Source | mitogen-activated protein kinase 3 | 909 | 0.156 | 0.2350 | Yes |
| 9 | CBLB | CBLB Entrez,  Source | Cas-Br-M (murine) ecotropic retroviral transforming sequence b | 913 | 0.156 | 0.2525 | Yes |
| 10 | HK2 | HK2 Entrez,  Source | hexokinase 2 | 977 | 0.151 | 0.2665 | Yes |
| 11 | BRAF | BRAF Entrez,  Source | v-raf murine sarcoma viral oncogene homolog B1 | 1048 | 0.146 | 0.2795 | Yes |
| 12 | CBLC | CBLC Entrez,  Source | Cas-Br-M (murine) ecotropic retroviral transforming sequence c | 1363 | 0.125 | 0.2775 | Yes |
| 13 | RHOQ | RHOQ Entrez,  Source | ras homolog gene family, member Q | 1826 | 0.105 | 0.2657 | Yes |
| 14 | PRKACB | PRKACB Entrez,  Source | protein kinase, cAMP-dependent, catalytic, beta | 1829 | 0.105 | 0.2775 | Yes |
| 15 | HK1 | HK1 Entrez,  Source | hexokinase 1 | 1832 | 0.105 | 0.2893 | Yes |
| 16 | PIK3CB | PIK3CB Entrez,  Source | phosphoinositide-3-kinase, catalytic, beta polypeptide | 1862 | 0.104 | 0.2996 | Yes |
| 17 | PRKAG2 | PRKAG2 Entrez,  Source | protein kinase, AMP-activated, gamma 2 non-catalytic subunit | 1953 | 0.101 | 0.3065 | Yes |
| 18 | PIK3CA | PIK3CA Entrez,  Source | phosphoinositide-3-kinase, catalytic, alpha polypeptide | 1955 | 0.101 | 0.3179 | Yes |
| 19 | SREBF1 | SREBF1 Entrez,  Source | sterol regulatory element binding transcription factor 1 | 2039 | 0.098 | 0.3248 | Yes |
| 20 | CALM3 | CALM3 Entrez,  Source | calmodulin 3 (phosphorylase kinase, delta) | 2223 | 0.092 | 0.3259 | Yes |
| 21 | PYGB | PYGB Entrez,  Source | phosphorylase, glycogen; brain | 2231 | 0.092 | 0.3360 | Yes |
| 22 | RAPGEF1 | RAPGEF1 Entrez,  Source | Rap guanine nucleotide exchange factor (GEF) 1 | 2235 | 0.092 | 0.3463 | Yes |
| 23 | PRKAA1 | PRKAA1 Entrez,  Source | protein kinase, AMP-activated, alpha 1 catalytic subunit | 2287 | 0.091 | 0.3540 | Yes |
| 24 | SLC2A4 | SLC2A4 Entrez,  Source | solute carrier family 2 (facilitated glucose transporter), member 4 | 2321 | 0.090 | 0.3625 | Yes |
| 25 | NRAS | NRAS Entrez,  Source | neuroblastoma RAS viral (v-ras) oncogene homolog | 2381 | 0.088 | 0.3695 | Yes |
| 26 | SOS2 | SOS2 Entrez,  Source | son of sevenless homolog 2 (Drosophila) | 2390 | 0.088 | 0.3791 | Yes |
| 27 | PRKAA2 | PRKAA2 Entrez,  Source | protein kinase, AMP-activated, alpha 2 catalytic subunit | 2521 | 0.084 | 0.3821 | Yes |
| 28 | CRK | CRK Entrez,  Source | v-crk sarcoma virus CT10 oncogene homolog (avian) | 2705 | 0.080 | 0.3817 | Yes |
| 29 | PPP1R3D | PPP1R3D Entrez,  Source | protein phosphatase 1, regulatory subunit 3D | 2709 | 0.080 | 0.3906 | Yes |
| 30 | PDPK1 | PDPK1 Entrez,  Source | 3-phosphoinositide dependent protein kinase-1 | 2871 | 0.076 | 0.3910 | Yes |
| 31 | MKNK1 | MKNK1 Entrez,  Source | MAP kinase interacting serine/threonine kinase 1 | 2907 | 0.075 | 0.3977 | Yes |
| 32 | KRAS | KRAS Entrez,  Source | v-Ki-ras2 Kirsten rat sarcoma viral oncogene homolog | 2993 | 0.073 | 0.4017 | Yes |
| 33 | PTPRF | PTPRF Entrez,  Source | protein tyrosine phosphatase, receptor type, F | 3041 | 0.072 | 0.4075 | Yes |
| 34 | PRKAB1 | PRKAB1 Entrez,  Source | protein kinase, AMP-activated, beta 1 non-catalytic subunit | 3054 | 0.072 | 0.4151 | Yes |
| 35 | IRS1 | IRS1 Entrez,  Source | insulin receptor substrate 1 | 3119 | 0.071 | 0.4199 | Yes |
| 36 | PRKCI | PRKCI Entrez,  Source | protein kinase C, iota | 3441 | 0.064 | 0.4106 | Yes |
| 37 | SOCS1 | SOCS1 Entrez,  Source | suppressor of cytokine signaling 1 | 3470 | 0.063 | 0.4164 | Yes |
| 38 | GSK3B | GSK3B Entrez,  Source | glycogen synthase kinase 3 beta | 3729 | 0.059 | 0.4098 | Yes |
| 39 | PPP1R3A | PPP1R3A Entrez,  Source | protein phosphatase 1, regulatory (inhibitor) subunit 3A (glycogen and sarcoplasmic reticulum binding subunit, skeletal muscle) | 3779 | 0.058 | 0.4139 | Yes |
| 40 | MAPK1 | MAPK1 Entrez,  Source | mitogen-activated protein kinase 1 | 3801 | 0.058 | 0.4194 | Yes |
| 41 | SOS1 | SOS1 Entrez,  Source | son of sevenless homolog 1 (Drosophila) | 4071 | 0.053 | 0.4116 | Yes |
| 42 | PDE3B | PDE3B Entrez,  Source | phosphodiesterase 3B, cGMP-inhibited | 4255 | 0.051 | 0.4079 | Yes |
| 43 | MKNK2 | MKNK2 Entrez,  Source | MAP kinase interacting serine/threonine kinase 2 | 4296 | 0.050 | 0.4116 | Yes |
| 44 | GYS1 | GYS1 Entrez,  Source | glycogen synthase 1 (muscle) | 4375 | 0.049 | 0.4131 | Yes |
| 45 | IKBKB | IKBKB Entrez,  Source | inhibitor of kappa light polypeptide gene enhancer in B-cells, kinase beta | 4438 | 0.048 | 0.4153 | Yes |
| 46 | TSC2 | TSC2 Entrez,  Source | tuberous sclerosis 2 | 4449 | 0.048 | 0.4203 | Yes |
| 47 | CALML3 | CALML3 Entrez,  Source | calmodulin-like 3 | 4761 | 0.043 | 0.4092 | No |
| 48 | PHKG2 | PHKG2 Entrez,  Source | phosphorylase kinase, gamma 2 (testis) | 5133 | 0.038 | 0.3944 | No |
| 49 | PIK3CD | PIK3CD Entrez,  Source | phosphoinositide-3-kinase, catalytic, delta polypeptide | 5186 | 0.037 | 0.3960 | No |
| 50 | SHC3 | SHC3 Entrez,  Source | SHC (Src homology 2 domain containing) transforming protein 3 | 5209 | 0.037 | 0.3991 | No |
| 51 | PPP1R3B | PPP1R3B Entrez,  Source | protein phosphatase 1, regulatory (inhibitor) subunit 3B | 5244 | 0.037 | 0.4015 | No |
| 52 | PTPN1 | PTPN1 Entrez,  Source | protein tyrosine phosphatase, non-receptor type 1 | 5342 | 0.035 | 0.4005 | No |
| 53 | PRKAR1A | PRKAR1A Entrez,  Source | protein kinase, cAMP-dependent, regulatory, type I, alpha (tissue specific extinguisher 1) | 5431 | 0.034 | 0.3999 | No |
| 54 | CALM2 | CALM2 Entrez,  Source | calmodulin 2 (phosphorylase kinase, delta) | 5521 | 0.033 | 0.3991 | No |
| 55 | EXOC7 | EXOC7 Entrez,  Source | exocyst complex component 7 | 5811 | 0.030 | 0.3876 | No |
| 56 | GRB2 | GRB2 Entrez,  Source | growth factor receptor-bound protein 2 | 5830 | 0.030 | 0.3901 | No |
| 57 | PRKACG | PRKACG Entrez,  Source | protein kinase, cAMP-dependent, catalytic, gamma | 5873 | 0.029 | 0.3912 | No |
| 58 | FLOT2 | FLOT2 Entrez,  Source | flotillin 2 | 5919 | 0.029 | 0.3921 | No |
| 59 | RAF1 | RAF1 Entrez,  Source | v-raf-1 murine leukemia viral oncogene homolog 1 | 5927 | 0.028 | 0.3950 | No |
| 60 | MAP2K1 | MAP2K1 Entrez,  Source | mitogen-activated protein kinase kinase 1 | 5929 | 0.028 | 0.3982 | No |
| 61 | PPARGC1A | PPARGC1A Entrez,  Source | peroxisome proliferative activated receptor, gamma, coactivator 1, alpha | 6394 | 0.024 | 0.3770 | No |
| 62 | MAPK9 | MAPK9 Entrez,  Source | mitogen-activated protein kinase 9 | 6411 | 0.023 | 0.3788 | No |
| 63 | MAP2K2 | MAP2K2 Entrez,  Source | mitogen-activated protein kinase kinase 2 | 6577 | 0.022 | 0.3728 | No |
| 64 | GCK | GCK Entrez,  Source | glucokinase (hexokinase 4, maturity onset diabetes of the young 2) | 6724 | 0.020 | 0.3676 | No |
| 65 | SHC1 | SHC1 Entrez,  Source | SHC (Src homology 2 domain containing) transforming protein 1 | 6895 | 0.018 | 0.3609 | No |
| 66 | SOCS3 | SOCS3 Entrez,  Source | suppressor of cytokine signaling 3 | 6916 | 0.018 | 0.3619 | No |
| 67 | PIK3CG | PIK3CG Entrez,  Source | phosphoinositide-3-kinase, catalytic, gamma polypeptide | 7031 | 0.017 | 0.3580 | No |
| 68 | PRKAR2A | PRKAR2A Entrez,  Source | protein kinase, cAMP-dependent, regulatory, type II, alpha | 7158 | 0.016 | 0.3533 | No |
| 69 | PRKCZ | PRKCZ Entrez,  Source | protein kinase C, zeta | 7162 | 0.016 | 0.3549 | No |
| 70 | PHKG1 | PHKG1 Entrez,  Source | phosphorylase kinase, gamma 1 (muscle) | 7331 | 0.014 | 0.3478 | No |
| 71 | LIPE | LIPE Entrez,  Source | lipase, hormone-sensitive | 7427 | 0.013 | 0.3444 | No |
| 72 | EIF4E | EIF4E Entrez,  Source | eukaryotic translation initiation factor 4E | 7454 | 0.013 | 0.3445 | No |
| 73 | TSC1 | TSC1 Entrez,  Source | tuberous sclerosis 1 | 7484 | 0.012 | 0.3444 | No |
| 74 | PPP1CB | PPP1CB Entrez,  Source | protein phosphatase 1, catalytic subunit, beta isoform | 7687 | 0.010 | 0.3352 | No |
| 75 | AKT1 | AKT1 Entrez,  Source | v-akt murine thymoma viral oncogene homolog 1 | 7896 | 0.008 | 0.3254 | No |
| 76 | ACACA | ACACA Entrez,  Source | acetyl-Coenzyme A carboxylase alpha | 8010 | 0.007 | 0.3204 | No |
| 77 | RHEB | RHEB Entrez,  Source | Ras homolog enriched in brain | 8129 | 0.006 | 0.3150 | No |
| 78 | PPP1CA | PPP1CA Entrez,  Source | protein phosphatase 1, catalytic subunit, alpha isoform | 8274 | 0.005 | 0.3081 | No |
| 79 | RPS6KB2 | RPS6KB2 Entrez,  Source | ribosomal protein S6 kinase, 70kDa, polypeptide 2 | 8335 | 0.004 | 0.3055 | No |
| 80 | SHC2 | SHC2 Entrez,  Source | SHC (Src homology 2 domain containing) transforming protein 2 | 8684 | 0.001 | 0.2877 | No |
| 81 | ACACB | ACACB Entrez,  Source | acetyl-Coenzyme A carboxylase beta | 8860 | -0.001 | 0.2787 | No |
| 82 | HRAS | HRAS Entrez,  Source | v-Ha-ras Harvey rat sarcoma viral oncogene homolog | 8944 | -0.001 | 0.2746 | No |
| 83 | TRIP10 | TRIP10 Entrez,  Source | thyroid hormone receptor interactor 10 | 8983 | -0.002 | 0.2729 | No |
| 84 | FBP2 | FBP2 Entrez,  Source | fructose-1,6-bisphosphatase 2 | 9013 | -0.002 | 0.2716 | No |
| 85 | PIK3R5 | PIK3R5 Entrez,  Source | phosphoinositide-3-kinase, regulatory subunit 5, p101 | 9214 | -0.004 | 0.2617 | No |
| 86 | CALM1 | CALM1 Entrez,  Source | calmodulin 1 (phosphorylase kinase, delta) | 9475 | -0.006 | 0.2490 | No |
| 87 | RPS6 | RPS6 Entrez,  Source | ribosomal protein S6 | 9625 | -0.007 | 0.2422 | No |
| 88 | CBL | CBL Entrez,  Source | Cas-Br-M (murine) ecotropic retroviral transforming sequence | 9785 | -0.009 | 0.2350 | No |
| 89 | G6PC2 | G6PC2 Entrez,  Source | glucose-6-phosphatase, catalytic, 2 | 10004 | -0.010 | 0.2250 | No |
| 90 | SOCS4 | SOCS4 Entrez,  Source | suppressor of cytokine signaling 4 | 10106 | -0.011 | 0.2210 | No |
| 91 | FASN | FASN Entrez,  Source | fatty acid synthase | 10368 | -0.014 | 0.2092 | No |
| 92 | ARAF | ARAF Entrez,  Source | v-raf murine sarcoma 3611 viral oncogene homolog | 10441 | -0.015 | 0.2072 | No |
| 93 | EIF4E2 | EIF4E2 Entrez,  Source | eukaryotic translation initiation factor 4E member 2 | 10464 | -0.015 | 0.2077 | No |
| 94 | MAPK8 | MAPK8 Entrez,  Source | mitogen-activated protein kinase 8 | 10468 | -0.015 | 0.2093 | No |
| 95 | INPP5D | INPP5D Entrez,  Source | inositol polyphosphate-5-phosphatase, 145kDa | 10829 | -0.018 | 0.1927 | No |
| 96 | AKT2 | AKT2 Entrez,  Source | v-akt murine thymoma viral oncogene homolog 2 | 11074 | -0.020 | 0.1824 | No |
| 97 | PRKAB2 | PRKAB2 Entrez,  Source | protein kinase, AMP-activated, beta 2 non-catalytic subunit | 11153 | -0.020 | 0.1807 | No |
| 98 | FLOT1 | FLOT1 Entrez,  Source | flotillin 1 | 11164 | -0.020 | 0.1825 | No |
| 99 | PYGL | PYGL Entrez,  Source | phosphorylase, glycogen; liver (Hers disease, glycogen storage disease type VI) | 11235 | -0.021 | 0.1813 | No |
| 100 | PPP1R3C | PPP1R3C Entrez,  Source | protein phosphatase 1, regulatory (inhibitor) subunit 3C | 11412 | -0.023 | 0.1749 | No |
| 101 | PIK3R1 | PIK3R1 Entrez,  Source | phosphoinositide-3-kinase, regulatory subunit 1 (p85 alpha) | 11545 | -0.024 | 0.1708 | No |
| 102 | SHC4 | SHC4 Entrez,  Source | SHC (Src homology 2 domain containing) family, member 4 | 11757 | -0.026 | 0.1629 | No |
| 103 | RPS6KB1 | RPS6KB1 Entrez,  Source | ribosomal protein S6 kinase, 70kDa, polypeptide 1 | 11815 | -0.027 | 0.1630 | No |
| 104 | INS | INS Entrez,  Source | insulin | 12003 | -0.028 | 0.1566 | No |
| 105 | CRKL | CRKL Entrez,  Source | v-crk sarcoma virus CT10 oncogene homolog (avian)-like | 12012 | -0.028 | 0.1594 | No |
| 106 | PKLR | PKLR Entrez,  Source | pyruvate kinase, liver and RBC | 12312 | -0.031 | 0.1475 | No |
| 107 | PPP1CC | PPP1CC Entrez,  Source | protein phosphatase 1, catalytic subunit, gamma isoform | 12534 | -0.033 | 0.1399 | No |
| 108 | G6PC | G6PC Entrez,  Source | glucose-6-phosphatase, catalytic subunit | 12761 | -0.036 | 0.1324 | No |
| 109 | MAPK10 | MAPK10 Entrez,  Source | mitogen-activated protein kinase 10 | 12835 | -0.036 | 0.1327 | No |
| 110 | PRKACA | PRKACA Entrez,  Source | protein kinase, cAMP-dependent, catalytic, alpha | 12867 | -0.037 | 0.1353 | No |
| 111 | PRKAG1 | PRKAG1 Entrez,  Source | protein kinase, AMP-activated, gamma 1 non-catalytic subunit | 13207 | -0.040 | 0.1225 | No |
| 112 | PHKA2 | PHKA2 Entrez,  Source | phosphorylase kinase, alpha 2 (liver) | 13347 | -0.042 | 0.1201 | No |
| 113 | HK3 | HK3 Entrez,  Source | hexokinase 3 (white cell) | 13411 | -0.043 | 0.1217 | No |
| 114 | PCK2 | PCK2 Entrez,  Source | phosphoenolpyruvate carboxykinase 2 (mitochondrial) | 13451 | -0.043 | 0.1246 | No |
| 115 | PIK3R2 | PIK3R2 Entrez,  Source | phosphoinositide-3-kinase, regulatory subunit 2 (p85 beta) | 13588 | -0.044 | 0.1227 | No |
| 116 | PRKAG3 | PRKAG3 Entrez,  Source | protein kinase, AMP-activated, gamma 3 non-catalytic subunit | 13919 | -0.048 | 0.1111 | No |
| 117 | CALML6 | CALML6 Entrez,  Source | calmodulin-like 6 | 14105 | -0.051 | 0.1074 | No |
| 118 | INSR | INSR Entrez,  Source | insulin receptor | 14440 | -0.055 | 0.0964 | No |
| 119 | IRS2 | IRS2 Entrez,  Source | insulin receptor substrate 2 | 14745 | -0.059 | 0.0874 | No |
| 120 | IRS4 | IRS4 Entrez,  Source | insulin receptor substrate 4 | 14752 | -0.059 | 0.0938 | No |
| 121 | AKT3 | AKT3 Entrez,  Source | v-akt murine thymoma viral oncogene homolog 3 (protein kinase B, gamma) | 15572 | -0.071 | 0.0598 | No |
| 122 | FBP1 | FBP1 Entrez,  Source | fructose-1,6-bisphosphatase 1 | 15671 | -0.073 | 0.0631 | No |
| 123 | ELK1 | ELK1 Entrez,  Source | ELK1, member of ETS oncogene family | 15683 | -0.073 | 0.0708 | No |
| 124 | GYS2 | GYS2 Entrez,  Source | glycogen synthase 2 (liver) | 17095 | -0.100 | 0.0096 | No |
| 125 | PHKA1 | PHKA1 Entrez,  Source | phosphorylase kinase, alpha 1 (muscle) | 17254 | -0.105 | 0.0134 | No |
| 126 | PYGM | PYGM Entrez,  Source | phosphorylase, glycogen; muscle (McArdle syndrome, glycogen storage disease type V) | 18261 | -0.139 | -0.0226 | No |
| 127 | PDE3A | PDE3A Entrez,  Source | phosphodiesterase 3A, cGMP-inhibited | 18583 | -0.157 | -0.0213 | No |
| 128 | EIF4EBP1 | EIF4EBP1 Entrez,  Source | eukaryotic translation initiation factor 4E binding protein 1 | 18757 | -0.169 | -0.0109 | No |
| 129 | PRKX | PRKX Entrez,  Source | protein kinase, X-linked | 19137 | -0.214 | -0.0061 | No |
| 130 | SORBS1 | SORBS1 Entrez,  Source | sorbin and SH3 domain containing 1 | 19283 | -0.243 | 0.0141 | No |
Table: GSEA details [plain text format]

  

Fig 2: KEGG\_INSULIN\_SIGNALING\_PATHWAY      
 Blue-Pink O' Gram in the Space of the Analyzed GeneSet

  

Fig 3: KEGG\_INSULIN\_SIGNALING\_PATHWAY: Random ES distribution      
 Gene set null distribution of ES for **KEGG\_INSULIN\_SIGNALING\_PATHWAY**
