# Supplementary material for: Comprehensive analysis of β-catenin target genes in colorectal carcinoma cell lines with deregulated Wnt/β-catenin signaling
Source: BMC Genomics. 2014 Jan 28;15:74. doi: 10.1186/1471-2164-15-74 (PMC3909937; doi:10.1186/1471-2164-15-74)
Supplement: Additional file 5 — GSEA analysis using the KEGG pathway database. This zipped file contains confirming data of the GSEA analysis. The names of the directories containing the files were composed of the term ‘GSEA’, the name of the cell line, e.g. DLD1, SW480, or LS174T, and the pathway database (KEGG). Please use a web browser to view the files with the name ‘index.html’ in the corresponding directories to start exploring the data. [file 1471-2164-15-74-S5.zip › GSEA_KEGG_DLD1/KEGG_LYSINE_DEGRADATION.html]

Details for gene set KEGG\_LYSINE\_DEGRADATION[GSEA]

|  || Dataset | DLD1\_collapsed\_to\_symbols.class.cls#b\_versus\_bg.class.cls#b\_versus\_bg\_repos |
| Phenotype | class.cls#b\_versus\_bg\_repos |
| Upregulated in class | 0 |
| GeneSet | KEGG\_LYSINE\_DEGRADATION |
| Enrichment Score (ES) | -0.3940613 |
| Normalized Enrichment Score (NES) | -1.3249912 |
| Nominal p-value | 0.08255159 |
| FDR q-value | 0.33455515 |
| FWER p-Value | 1.0 |
Table: GSEA Results Summary

  

Fig 1: Enrichment plot: KEGG\_LYSINE\_DEGRADATION      
 Profile of the Running ES Score & Positions of GeneSet Members on the Rank Ordered List

  

| PROBE | GENE SYMBOL | GENE\_TITLE | RANK IN GENE LIST | RANK METRIC SCORE | RUNNING ES | CORE ENRICHMENT || 1 | ALDH3A2 | ALDH3A2 Entrez,  Source | aldehyde dehydrogenase 3 family, member A2 | 959 | 0.152 | 0.0041 | No |
| 2 | SUV420H1 | SUV420H1 Entrez,  Source | suppressor of variegation 4-20 homolog 1 (Drosophila) | 1255 | 0.131 | 0.0347 | No |
| 3 | OGDH | OGDH Entrez,  Source | oxoglutarate (alpha-ketoglutarate) dehydrogenase (lipoamide) | 1283 | 0.129 | 0.0786 | No |
| 4 | ASH1L | ASH1L Entrez,  Source | ash1 (absent, small, or homeotic)-like (Drosophila) | 1733 | 0.109 | 0.0935 | No |
| 5 | ALDH9A1 | ALDH9A1 Entrez,  Source | aldehyde dehydrogenase 9 family, member A1 | 2847 | 0.077 | 0.0632 | No |
| 6 | AASS | AASS Entrez,  Source | aminoadipate-semialdehyde synthase | 3781 | 0.058 | 0.0357 | No |
| 7 | ACAT2 | ACAT2 Entrez,  Source | acetyl-Coenzyme A acetyltransferase 2 (acetoacetyl Coenzyme A thiolase) | 5304 | 0.036 | -0.0297 | No |
| 8 | SETD8 | SETD8 Entrez,  Source | SET domain containing (lysine methyltransferase) 8 | 5368 | 0.035 | -0.0207 | No |
| 9 | PLOD1 | PLOD1 Entrez,  Source | procollagen-lysine 1, 2-oxoglutarate 5-dioxygenase 1 | 5792 | 0.030 | -0.0318 | No |
| 10 | SETD1B | SETD1B Entrez,  Source | SET domain containing 1B | 5985 | 0.028 | -0.0320 | No |
| 11 | PLOD2 | PLOD2 Entrez,  Source | procollagen-lysine, 2-oxoglutarate 5-dioxygenase 2 | 6220 | 0.025 | -0.0350 | No |
| 12 | NSD1 | NSD1 Entrez,  Source | nuclear receptor binding SET domain protein 1 | 6282 | 0.025 | -0.0295 | No |
| 13 | ALDH2 | ALDH2 Entrez,  Source | aldehyde dehydrogenase 2 family (mitochondrial) | 6555 | 0.022 | -0.0358 | No |
| 14 | SETDB2 | SETDB2 Entrez,  Source | SET domain, bifurcated 2 | 6996 | 0.017 | -0.0523 | No |
| 15 | BBOX1 | BBOX1 Entrez,  Source | butyrobetaine (gamma), 2-oxoglutarate dioxygenase (gamma-butyrobetaine hydroxylase) 1 | 7428 | 0.013 | -0.0699 | No |
| 16 | ALDH7A1 | ALDH7A1 Entrez,  Source | aldehyde dehydrogenase 7 family, member A1 | 7706 | 0.010 | -0.0806 | No |
| 17 | TMLHE | TMLHE Entrez,  Source | trimethyllysine hydroxylase, epsilon | 7829 | 0.009 | -0.0837 | No |
| 18 | SETD2 | SETD2 Entrez,  Source | SET domain containing 2 | 8907 | -0.001 | -0.1386 | No |
| 19 | OGDHL | OGDHL Entrez,  Source | oxoglutarate dehydrogenase-like | 8917 | -0.001 | -0.1387 | No |
| 20 | EHMT1 | EHMT1 Entrez,  Source | euchromatic histone-lysine N-methyltransferase 1 | 9008 | -0.002 | -0.1426 | No |
| 21 | EHHADH | EHHADH Entrez,  Source | enoyl-Coenzyme A, hydratase/3-hydroxyacyl Coenzyme A dehydrogenase | 9058 | -0.002 | -0.1443 | No |
| 22 | SETD7 | SETD7 Entrez,  Source | SET domain containing (lysine methyltransferase) 7 | 9905 | -0.010 | -0.1843 | No |
| 23 | HADHA | HADHA Entrez,  Source | hydroxyacyl-Coenzyme A dehydrogenase/3-ketoacyl-Coenzyme A thiolase/enoyl-Coenzyme A hydratase (trifunctional protein), alpha subunit | 10086 | -0.011 | -0.1896 | No |
| 24 | WHSC1L1 | WHSC1L1 Entrez,  Source | Wolf-Hirschhorn syndrome candidate 1-like 1 | 10405 | -0.014 | -0.2009 | No |
| 25 | ECHS1 | ECHS1 Entrez,  Source | enoyl Coenzyme A hydratase, short chain, 1, mitochondrial | 11609 | -0.025 | -0.2539 | No |
| 26 | SUV420H2 | SUV420H2 Entrez,  Source | suppressor of variegation 4-20 homolog 2 (Drosophila) | 11988 | -0.028 | -0.2634 | No |
| 27 | PLOD3 | PLOD3 Entrez,  Source | procollagen-lysine, 2-oxoglutarate 5-dioxygenase 3 | 13762 | -0.046 | -0.3381 | No |
| 28 | ACAT1 | ACAT1 Entrez,  Source | acetyl-Coenzyme A acetyltransferase 1 (acetoacetyl Coenzyme A thiolase) | 14314 | -0.053 | -0.3478 | No |
| 29 | AASDH | AASDH Entrez,  Source | - | 15218 | -0.066 | -0.3710 | Yes |
| 30 | WHSC1 | WHSC1 Entrez,  Source | Wolf-Hirschhorn syndrome candidate 1 | 15233 | -0.066 | -0.3486 | Yes |
| 31 | AASDHPPT | AASDHPPT Entrez,  Source | aminoadipate-semialdehyde dehydrogenase-phosphopantetheinyl transferase | 15825 | -0.076 | -0.3524 | Yes |
| 32 | SETDB1 | SETDB1 Entrez,  Source | SET domain, bifurcated 1 | 16070 | -0.080 | -0.3371 | Yes |
| 33 | SETD1A | SETD1A Entrez,  Source | SET domain containing 1A | 16384 | -0.086 | -0.3230 | Yes |
| 34 | SUV39H1 | SUV39H1 Entrez,  Source | suppressor of variegation 3-9 homolog 1 (Drosophila) | 16740 | -0.093 | -0.3086 | Yes |
| 35 | EHMT2 | EHMT2 Entrez,  Source | euchromatic histone-lysine N-methyltransferase 2 | 16860 | -0.096 | -0.2813 | Yes |
| 36 | GCDH | GCDH Entrez,  Source | glutaryl-Coenzyme A dehydrogenase | 17154 | -0.102 | -0.2607 | Yes |
| 37 | DOT1L | DOT1L Entrez,  Source | DOT1-like, histone H3 methyltransferase (S. cerevisiae) | 17434 | -0.109 | -0.2369 | Yes |
| 38 | HADH | HADH Entrez,  Source | hydroxyacyl-Coenzyme A dehydrogenase | 17614 | -0.115 | -0.2059 | Yes |
| 39 | PIPOX | PIPOX Entrez,  Source | pipecolic acid oxidase | 17750 | -0.119 | -0.1711 | Yes |
| 40 | AADAT | AADAT Entrez,  Source | aminoadipate aminotransferase | 18091 | -0.132 | -0.1425 | Yes |
| 41 | SUV39H2 | SUV39H2 Entrez,  Source | suppressor of variegation 3-9 homolog 2 (Drosophila) | 18514 | -0.152 | -0.1109 | Yes |
| 42 | ALDH1B1 | ALDH1B1 Entrez,  Source | aldehyde dehydrogenase 1 family, member B1 | 19017 | -0.196 | -0.0680 | Yes |
| 43 | SETMAR | SETMAR Entrez,  Source | SET domain and mariner transposase fusion gene | 19352 | -0.273 | 0.0105 | Yes |
Table: GSEA details [plain text format]

  

Fig 2: KEGG\_LYSINE\_DEGRADATION      
 Blue-Pink O' Gram in the Space of the Analyzed GeneSet

  

Fig 3: KEGG\_LYSINE\_DEGRADATION: Random ES distribution      
 Gene set null distribution of ES for **KEGG\_LYSINE\_DEGRADATION**
