# Supplementary material for: Comprehensive analysis of β-catenin target genes in colorectal carcinoma cell lines with deregulated Wnt/β-catenin signaling
Source: BMC Genomics. 2014 Jan 28;15:74. doi: 10.1186/1471-2164-15-74 (PMC3909937; doi:10.1186/1471-2164-15-74)
Supplement: Additional file 5 — GSEA analysis using the KEGG pathway database. This zipped file contains confirming data of the GSEA analysis. The names of the directories containing the files were composed of the term ‘GSEA’, the name of the cell line, e.g. DLD1, SW480, or LS174T, and the pathway database (KEGG). Please use a web browser to view the files with the name ‘index.html’ in the corresponding directories to start exploring the data. [file 1471-2164-15-74-S5.zip › GSEA_KEGG_DLD1/KEGG_LYSOSOME.html]

Details for gene set KEGG\_LYSOSOME[GSEA]

|  || Dataset | DLD1\_collapsed\_to\_symbols.class.cls#b\_versus\_bg.class.cls#b\_versus\_bg\_repos |
| Phenotype | class.cls#b\_versus\_bg\_repos |
| Upregulated in class | 1 |
| GeneSet | KEGG\_LYSOSOME |
| Enrichment Score (ES) | 0.42640066 |
| Normalized Enrichment Score (NES) | 1.6484925 |
| Nominal p-value | 0.0040241447 |
| FDR q-value | 0.12100554 |
| FWER p-Value | 0.645 |
Table: GSEA Results Summary

  

Fig 1: Enrichment plot: KEGG\_LYSOSOME      
 Profile of the Running ES Score & Positions of GeneSet Members on the Rank Ordered List

  

| PROBE | GENE SYMBOL | GENE\_TITLE | RANK IN GENE LIST | RANK METRIC SCORE | RUNNING ES | CORE ENRICHMENT || 1 | NEU1 | NEU1 Entrez,  Source | sialidase 1 (lysosomal sialidase) | 320 | 0.261 | 0.0200 | Yes |
| 2 | GM2A | GM2A Entrez,  Source | GM2 ganglioside activator | 325 | 0.259 | 0.0561 | Yes |
| 3 | HGSNAT | HGSNAT Entrez,  Source | heparan-alpha-glucosaminide N-acetyltransferase | 393 | 0.233 | 0.0853 | Yes |
| 4 | CLTB | CLTB Entrez,  Source | clathrin, light chain (Lcb) | 414 | 0.228 | 0.1161 | Yes |
| 5 | HYAL1 | HYAL1 Entrez,  Source | hyaluronoglucosaminidase 1 | 642 | 0.184 | 0.1303 | Yes |
| 6 | ARSA | ARSA Entrez,  Source | arylsulfatase A | 780 | 0.169 | 0.1468 | Yes |
| 7 | AP1S3 | AP1S3 Entrez,  Source | adaptor-related protein complex 1, sigma 3 subunit | 800 | 0.167 | 0.1693 | Yes |
| 8 | SMPD1 | SMPD1 Entrez,  Source | sphingomyelin phosphodiesterase 1, acid lysosomal (acid sphingomyelinase) | 856 | 0.161 | 0.1890 | Yes |
| 9 | ASAH1 | ASAH1 Entrez,  Source | N-acylsphingosine amidohydrolase (acid ceramidase) 1 | 875 | 0.159 | 0.2104 | Yes |
| 10 | CLN5 | CLN5 Entrez,  Source | ceroid-lipofuscinosis, neuronal 5 | 1354 | 0.126 | 0.2034 | Yes |
| 11 | NPC1 | NPC1 Entrez,  Source | Niemann-Pick disease, type C1 | 1416 | 0.122 | 0.2174 | Yes |
| 12 | ATP6AP1 | ATP6AP1 Entrez,  Source | ATPase, H+ transporting, lysosomal accessory protein 1 | 1545 | 0.116 | 0.2270 | Yes |
| 13 | LAMP3 | LAMP3 Entrez,  Source | lysosomal-associated membrane protein 3 | 1598 | 0.114 | 0.2403 | Yes |
| 14 | GALNS | GALNS Entrez,  Source | galactosamine (N-acetyl)-6-sulfate sulfatase (Morquio syndrome, mucopolysaccharidosis type IVA) | 1847 | 0.104 | 0.2421 | Yes |
| 15 | ATP6V0D2 | ATP6V0D2 Entrez,  Source | ATPase, H+ transporting, lysosomal 38kDa, V0 subunit d2 | 2075 | 0.097 | 0.2440 | Yes |
| 16 | CTSO | CTSO Entrez,  Source | cathepsin O | 2245 | 0.092 | 0.2482 | Yes |
| 17 | CD63 | CD63 Entrez,  Source | CD63 molecule | 2299 | 0.090 | 0.2581 | Yes |
| 18 | AGA | AGA Entrez,  Source | aspartylglucosaminidase | 2344 | 0.089 | 0.2683 | Yes |
| 19 | CTSK | CTSK Entrez,  Source | cathepsin K (pycnodysostosis) | 2495 | 0.085 | 0.2725 | Yes |
| 20 | DNASE2B | DNASE2B Entrez,  Source | deoxyribonuclease II beta | 2777 | 0.078 | 0.2690 | Yes |
| 21 | AP1G1 | AP1G1 Entrez,  Source | adaptor-related protein complex 1, gamma 1 subunit | 2846 | 0.077 | 0.2762 | Yes |
| 22 | AP3S2 | AP3S2 Entrez,  Source | adaptor-related protein complex 3, sigma 2 subunit | 2898 | 0.075 | 0.2842 | Yes |
| 23 | AP1M1 | AP1M1 Entrez,  Source | adaptor-related protein complex 1, mu 1 subunit | 3001 | 0.073 | 0.2892 | Yes |
| 24 | HEXA | HEXA Entrez,  Source | hexosaminidase A (alpha polypeptide) | 3030 | 0.073 | 0.2979 | Yes |
| 25 | GALC | GALC Entrez,  Source | galactosylceramidase | 3056 | 0.072 | 0.3067 | Yes |
| 26 | SORT1 | SORT1 Entrez,  Source | sortilin 1 | 3169 | 0.070 | 0.3107 | Yes |
| 27 | ATP6V1H | ATP6V1H Entrez,  Source | ATPase, H+ transporting, lysosomal 50/57kDa, V1 subunit H | 3261 | 0.067 | 0.3155 | Yes |
| 28 | SLC17A5 | SLC17A5 Entrez,  Source | solute carrier family 17 (anion/sugar transporter), member 5 | 3421 | 0.065 | 0.3163 | Yes |
| 29 | PSAP | PSAP Entrez,  Source | prosaposin (variant Gaucher disease and variant metachromatic leukodystrophy) | 3461 | 0.064 | 0.3232 | Yes |
| 30 | LIPA | LIPA Entrez,  Source | lipase A, lysosomal acid, cholesterol esterase (Wolman disease) | 3464 | 0.064 | 0.3320 | Yes |
| 31 | IDS | IDS Entrez,  Source | iduronate 2-sulfatase (Hunter syndrome) | 3483 | 0.063 | 0.3399 | Yes |
| 32 | SCARB2 | SCARB2 Entrez,  Source | scavenger receptor class B, member 2 | 3533 | 0.062 | 0.3461 | Yes |
| 33 | GNS | GNS Entrez,  Source | glucosamine (N-acetyl)-6-sulfatase (Sanfilippo disease IIID) | 3599 | 0.061 | 0.3514 | Yes |
| 34 | CD164 | CD164 Entrez,  Source | CD164 molecule, sialomucin | 3694 | 0.060 | 0.3549 | Yes |
| 35 | SLC11A2 | SLC11A2 Entrez,  Source | solute carrier family 11 (proton-coupled divalent metal ion transporters), member 2 | 3870 | 0.056 | 0.3538 | Yes |
| 36 | AP3B2 | AP3B2 Entrez,  Source | adaptor-related protein complex 3, beta 2 subunit | 3878 | 0.056 | 0.3613 | Yes |
| 37 | ATP6V0D1 | ATP6V0D1 Entrez,  Source | ATPase, H+ transporting, lysosomal 38kDa, V0 subunit d1 | 3909 | 0.056 | 0.3676 | Yes |
| 38 | AP4S1 | AP4S1 Entrez,  Source | adaptor-related protein complex 4, sigma 1 subunit | 3962 | 0.055 | 0.3726 | Yes |
| 39 | ATP6V0A1 | ATP6V0A1 Entrez,  Source | ATPase, H+ transporting, lysosomal V0 subunit a1 | 3989 | 0.055 | 0.3790 | Yes |
| 40 | CTSZ | CTSZ Entrez,  Source | cathepsin Z | 4055 | 0.053 | 0.3831 | Yes |
| 41 | IGF2R | IGF2R Entrez,  Source | insulin-like growth factor 2 receptor | 4204 | 0.051 | 0.3826 | Yes |
| 42 | TPP1 | TPP1 Entrez,  Source | tripeptidyl peptidase I | 4271 | 0.050 | 0.3863 | Yes |
| 43 | SUMF1 | SUMF1 Entrez,  Source | sulfatase modifying factor 1 | 4277 | 0.050 | 0.3931 | Yes |
| 44 | TCIRG1 | TCIRG1 Entrez,  Source | T-cell, immune regulator 1, ATPase, H+ transporting, lysosomal V0 subunit A3 | 4282 | 0.050 | 0.3999 | Yes |
| 45 | CTSL2 | CTSL2 Entrez,  Source | cathepsin L2 | 4441 | 0.048 | 0.3984 | Yes |
| 46 | ARSB | ARSB Entrez,  Source | arylsulfatase B | 4462 | 0.048 | 0.4041 | Yes |
| 47 | CTSF | CTSF Entrez,  Source | cathepsin F | 4509 | 0.047 | 0.4083 | Yes |
| 48 | CD68 | CD68 Entrez,  Source | CD68 molecule | 4546 | 0.046 | 0.4129 | Yes |
| 49 | CLN3 | CLN3 Entrez,  Source | ceroid-lipofuscinosis, neuronal 3, juvenile (Batten, Spielmeyer-Vogt disease) | 4551 | 0.046 | 0.4192 | Yes |
| 50 | CTSE | CTSE Entrez,  Source | cathepsin E | 4602 | 0.046 | 0.4230 | Yes |
| 51 | ACP2 | ACP2 Entrez,  Source | acid phosphatase 2, lysosomal | 4659 | 0.045 | 0.4264 | Yes |
| 52 | LAMP1 | LAMP1 Entrez,  Source | lysosomal-associated membrane protein 1 | 4979 | 0.040 | 0.4156 | No |
| 53 | SGSH | SGSH Entrez,  Source | N-sulfoglucosamine sulfohydrolase (sulfamidase) | 5028 | 0.039 | 0.4187 | No |
| 54 | GNPTG | GNPTG Entrez,  Source | N-acetylglucosamine-1-phosphate transferase, gamma subunit | 5067 | 0.039 | 0.4222 | No |
| 55 | MAN2B1 | MAN2B1 Entrez,  Source | mannosidase, alpha, class 2B, member 1 | 5445 | 0.034 | 0.4076 | No |
| 56 | AP1B1 | AP1B1 Entrez,  Source | adaptor-related protein complex 1, beta 1 subunit | 5477 | 0.034 | 0.4107 | No |
| 57 | FUCA1 | FUCA1 Entrez,  Source | fucosidase, alpha-L- 1, tissue | 5629 | 0.032 | 0.4074 | No |
| 58 | ATP6V0C | ATP6V0C Entrez,  Source | ATPase, H+ transporting, lysosomal 16kDa, V0 subunit c | 5632 | 0.032 | 0.4117 | No |
| 59 | LGMN | LGMN Entrez,  Source | legumain | 5722 | 0.031 | 0.4115 | No |
| 60 | LAPTM4A | LAPTM4A Entrez,  Source | lysosomal-associated protein transmembrane 4 alpha | 5783 | 0.030 | 0.4126 | No |
| 61 | AP1S2 | AP1S2 Entrez,  Source | adaptor-related protein complex 1, sigma 2 subunit | 5936 | 0.028 | 0.4088 | No |
| 62 | AP3M1 | AP3M1 Entrez,  Source | adaptor-related protein complex 3, mu 1 subunit | 6069 | 0.027 | 0.4057 | No |
| 63 | GLB1 | GLB1 Entrez,  Source | galactosidase, beta 1 | 6137 | 0.026 | 0.4060 | No |
| 64 | AP3B1 | AP3B1 Entrez,  Source | adaptor-related protein complex 3, beta 1 subunit | 6167 | 0.026 | 0.4081 | No |
| 65 | NAGA | NAGA Entrez,  Source | N-acetylgalactosaminidase, alpha- | 6259 | 0.025 | 0.4069 | No |
| 66 | CTSH | CTSH Entrez,  Source | cathepsin H | 6338 | 0.024 | 0.4063 | No |
| 67 | CLTC | CLTC Entrez,  Source | clathrin, heavy chain (Hc) | 6356 | 0.024 | 0.4088 | No |
| 68 | NAGLU | NAGLU Entrez,  Source | N-acetylglucosaminidase, alpha- (Sanfilippo disease IIIB) | 6731 | 0.020 | 0.3924 | No |
| 69 | GNPTAB | GNPTAB Entrez,  Source | N-acetylglucosamine-1-phosphate transferase, alpha and beta subunits | 6894 | 0.018 | 0.3866 | No |
| 70 | CTSD | CTSD Entrez,  Source | cathepsin D (lysosomal aspartyl peptidase) | 6910 | 0.018 | 0.3884 | No |
| 71 | AP3D1 | AP3D1 Entrez,  Source | adaptor-related protein complex 3, delta 1 subunit | 7159 | 0.016 | 0.3778 | No |
| 72 | NPC2 | NPC2 Entrez,  Source | Niemann-Pick disease, type C2 | 7239 | 0.015 | 0.3758 | No |
| 73 | CLTA | CLTA Entrez,  Source | clathrin, light chain (Lca) | 7248 | 0.015 | 0.3775 | No |
| 74 | MANBA | MANBA Entrez,  Source | mannosidase, beta A, lysosomal | 7270 | 0.015 | 0.3784 | No |
| 75 | ATP6V0B | ATP6V0B Entrez,  Source | ATPase, H+ transporting, lysosomal 21kDa, V0 subunit b | 7369 | 0.014 | 0.3753 | No |
| 76 | GAA | GAA Entrez,  Source | glucosidase, alpha; acid (Pompe disease, glycogen storage disease type II) | 7556 | 0.012 | 0.3673 | No |
| 77 | AP1M2 | AP1M2 Entrez,  Source | adaptor-related protein complex 1, mu 2 subunit | 7816 | 0.009 | 0.3553 | No |
| 78 | ENTPD4 | ENTPD4 Entrez,  Source | ectonucleoside triphosphate diphosphohydrolase 4 | 7869 | 0.008 | 0.3538 | No |
| 79 | LAMP2 | LAMP2 Entrez,  Source | lysosomal-associated membrane protein 2 | 7941 | 0.008 | 0.3512 | No |
| 80 | HEXB | HEXB Entrez,  Source | hexosaminidase B (beta polypeptide) | 8127 | 0.006 | 0.3425 | No |
| 81 | AP3S1 | AP3S1 Entrez,  Source | adaptor-related protein complex 3, sigma 1 subunit | 8369 | 0.004 | 0.3306 | No |
| 82 | LAPTM4B | LAPTM4B Entrez,  Source | lysosomal associated protein transmembrane 4 beta | 8782 | 0.000 | 0.3095 | No |
| 83 | NAGPA | NAGPA Entrez,  Source | N-acetylglucosamine-1-phosphodiester alpha-N-acetylglucosaminidase | 9451 | -0.006 | 0.2759 | No |
| 84 | GUSB | GUSB Entrez,  Source | glucuronidase, beta | 9543 | -0.006 | 0.2721 | No |
| 85 | AP4E1 | AP4E1 Entrez,  Source | adaptor-related protein complex 4, epsilon 1 subunit | 9596 | -0.007 | 0.2705 | No |
| 86 | GGA1 | GGA1 Entrez,  Source | golgi associated, gamma adaptin ear containing, ARF binding protein 1 | 9666 | -0.008 | 0.2680 | No |
| 87 | MCOLN1 | MCOLN1 Entrez,  Source | mucolipin 1 | 9778 | -0.009 | 0.2635 | No |
| 88 | CTSB | CTSB Entrez,  Source | cathepsin B | 10214 | -0.012 | 0.2429 | No |
| 89 | CTNS | CTNS Entrez,  Source | cystinosis, nephropathic | 10219 | -0.012 | 0.2444 | No |
| 90 | SLC11A1 | SLC11A1 Entrez,  Source | solute carrier family 11 (proton-coupled divalent metal ion transporters), member 1 | 10783 | -0.017 | 0.2179 | No |
| 91 | DNASE2 | DNASE2 Entrez,  Source | deoxyribonuclease II, lysosomal | 10820 | -0.018 | 0.2185 | No |
| 92 | M6PR | M6PR Entrez,  Source | mannose-6-phosphate receptor (cation dependent) | 11029 | -0.019 | 0.2105 | No |
| 93 | IDUA | IDUA Entrez,  Source | iduronidase, alpha-L- | 11175 | -0.021 | 0.2060 | No |
| 94 | GGA3 | GGA3 Entrez,  Source | golgi associated, gamma adaptin ear containing, ARF binding protein 3 | 11716 | -0.026 | 0.1818 | No |
| 95 | GGA2 | GGA2 Entrez,  Source | golgi associated, gamma adaptin ear containing, ARF binding protein 2 | 11846 | -0.027 | 0.1789 | No |
| 96 | AP1S1 | AP1S1 Entrez,  Source | adaptor-related protein complex 1, sigma 1 subunit | 12734 | -0.035 | 0.1382 | No |
| 97 | CTSS | CTSS Entrez,  Source | cathepsin S | 12845 | -0.037 | 0.1377 | No |
| 98 | GLA | GLA Entrez,  Source | galactosidase, alpha | 13345 | -0.042 | 0.1179 | No |
| 99 | PPT1 | PPT1 Entrez,  Source | palmitoyl-protein thioesterase 1 (ceroid-lipofuscinosis, neuronal 1, infantile) | 13378 | -0.042 | 0.1222 | No |
| 100 | LAPTM5 | LAPTM5 Entrez,  Source | lysosomal associated multispanning membrane protein 5 | 13393 | -0.042 | 0.1274 | No |
| 101 | CTSW | CTSW Entrez,  Source | cathepsin W (lymphopain) | 13485 | -0.043 | 0.1288 | No |
| 102 | NAPSA | NAPSA Entrez,  Source | napsin A aspartic peptidase | 13695 | -0.046 | 0.1244 | No |
| 103 | ABCA2 | ABCA2 Entrez,  Source | ATP-binding cassette, sub-family A (ABC1), member 2 | 14068 | -0.050 | 0.1123 | No |
| 104 | ABCB9 | ABCB9 Entrez,  Source | ATP-binding cassette, sub-family B (MDR/TAP), member 9 | 14079 | -0.050 | 0.1188 | No |
| 105 | ACP5 | ACP5 Entrez,  Source | acid phosphatase 5, tartrate resistant | 15624 | -0.072 | 0.0495 | No |
| 106 | CTSG | CTSG Entrez,  Source | cathepsin G | 15817 | -0.076 | 0.0502 | No |
| 107 | CTSC | CTSC Entrez,  Source | cathepsin C | 16117 | -0.081 | 0.0462 | No |
| 108 | AP4M1 | AP4M1 Entrez,  Source | adaptor-related protein complex 4, mu 1 subunit | 16361 | -0.086 | 0.0457 | No |
| 109 | ATP6V0A2 | ATP6V0A2 Entrez,  Source | ATPase, H+ transporting, lysosomal V0 subunit a2 | 16667 | -0.092 | 0.0428 | No |
| 110 | CLTCL1 | CLTCL1 Entrez,  Source | clathrin, heavy chain-like 1 | 17664 | -0.117 | 0.0079 | No |
| 111 | AP4B1 | AP4B1 Entrez,  Source | adaptor-related protein complex 4, beta 1 subunit | 18510 | -0.152 | -0.0143 | No |
| 112 | ATP6V0A4 | ATP6V0A4 Entrez,  Source | ATPase, H+ transporting, lysosomal V0 subunit a4 | 18532 | -0.153 | 0.0061 | No |
| 113 | ARSG | ARSG Entrez,  Source | arylsulfatase G | 18533 | -0.153 | 0.0276 | No |
| 114 | AP3M2 | AP3M2 Entrez,  Source | adaptor-related protein complex 3, mu 2 subunit | 18839 | -0.178 | 0.0369 | No |
Table: GSEA details [plain text format]

  

Fig 2: KEGG\_LYSOSOME      
 Blue-Pink O' Gram in the Space of the Analyzed GeneSet

  

Fig 3: KEGG\_LYSOSOME: Random ES distribution      
 Gene set null distribution of ES for **KEGG\_LYSOSOME**
