# Supplementary material for: Comprehensive analysis of β-catenin target genes in colorectal carcinoma cell lines with deregulated Wnt/β-catenin signaling
Source: BMC Genomics. 2014 Jan 28;15:74. doi: 10.1186/1471-2164-15-74 (PMC3909937; doi:10.1186/1471-2164-15-74)
Supplement: Additional file 5 — GSEA analysis using the KEGG pathway database. This zipped file contains confirming data of the GSEA analysis. The names of the directories containing the files were composed of the term ‘GSEA’, the name of the cell line, e.g. DLD1, SW480, or LS174T, and the pathway database (KEGG). Please use a web browser to view the files with the name ‘index.html’ in the corresponding directories to start exploring the data. [file 1471-2164-15-74-S5.zip › GSEA_KEGG_DLD1/KEGG_MELANOMA.html]

Details for gene set KEGG\_MELANOMA[GSEA]

|  || Dataset | DLD1\_collapsed\_to\_symbols.class.cls#b\_versus\_bg.class.cls#b\_versus\_bg\_repos |
| Phenotype | class.cls#b\_versus\_bg\_repos |
| Upregulated in class | 0 |
| GeneSet | KEGG\_MELANOMA |
| Enrichment Score (ES) | -0.35321483 |
| Normalized Enrichment Score (NES) | -1.2885447 |
| Nominal p-value | 0.10420842 |
| FDR q-value | 0.40241486 |
| FWER p-Value | 1.0 |
Table: GSEA Results Summary

  

Fig 1: Enrichment plot: KEGG\_MELANOMA      
 Profile of the Running ES Score & Positions of GeneSet Members on the Rank Ordered List

  

| PROBE | GENE SYMBOL | GENE\_TITLE | RANK IN GENE LIST | RANK METRIC SCORE | RUNNING ES | CORE ENRICHMENT || 1 | EGF | EGF Entrez,  Source | epidermal growth factor (beta-urogastrone) | 177 | 0.340 | 0.0450 | No |
| 2 | PIK3R3 | PIK3R3 Entrez,  Source | phosphoinositide-3-kinase, regulatory subunit 3 (p55, gamma) | 247 | 0.296 | 0.0885 | No |
| 3 | BAD | BAD Entrez,  Source | BCL2-antagonist of cell death | 406 | 0.230 | 0.1169 | No |
| 4 | MAPK3 | MAPK3 Entrez,  Source | mitogen-activated protein kinase 3 | 909 | 0.156 | 0.1159 | No |
| 5 | BRAF | BRAF Entrez,  Source | v-raf murine sarcoma viral oncogene homolog B1 | 1048 | 0.146 | 0.1320 | No |
| 6 | PDGFA | PDGFA Entrez,  Source | platelet-derived growth factor alpha polypeptide | 1189 | 0.135 | 0.1463 | No |
| 7 | PIK3CB | PIK3CB Entrez,  Source | phosphoinositide-3-kinase, catalytic, beta polypeptide | 1862 | 0.104 | 0.1283 | No |
| 8 | PIK3CA | PIK3CA Entrez,  Source | phosphoinositide-3-kinase, catalytic, alpha polypeptide | 1955 | 0.101 | 0.1396 | No |
| 9 | FGF3 | FGF3 Entrez,  Source | fibroblast growth factor 3 (murine mammary tumor virus integration site (v-int-2) oncogene homolog) | 1956 | 0.101 | 0.1556 | No |
| 10 | CDKN1A | CDKN1A Entrez,  Source | cyclin-dependent kinase inhibitor 1A (p21, Cip1) | 2044 | 0.098 | 0.1667 | No |
| 11 | CDH1 | CDH1 Entrez,  Source | cadherin 1, type 1, E-cadherin (epithelial) | 2124 | 0.095 | 0.1778 | No |
| 12 | NRAS | NRAS Entrez,  Source | neuroblastoma RAS viral (v-ras) oncogene homolog | 2381 | 0.088 | 0.1786 | No |
| 13 | FGF14 | FGF14 Entrez,  Source | fibroblast growth factor 14 | 2844 | 0.077 | 0.1671 | No |
| 14 | KRAS | KRAS Entrez,  Source | v-Ki-ras2 Kirsten rat sarcoma viral oncogene homolog | 2993 | 0.073 | 0.1712 | No |
| 15 | FGF7 | FGF7 Entrez,  Source | fibroblast growth factor 7 (keratinocyte growth factor) | 3102 | 0.071 | 0.1769 | No |
| 16 | EGFR | EGFR Entrez,  Source | epidermal growth factor receptor (erythroblastic leukemia viral (v-erb-b) oncogene homolog, avian) | 3161 | 0.070 | 0.1850 | No |
| 17 | MAPK1 | MAPK1 Entrez,  Source | mitogen-activated protein kinase 1 | 3801 | 0.058 | 0.1614 | No |
| 18 | MITF | MITF Entrez,  Source | microphthalmia-associated transcription factor | 3900 | 0.056 | 0.1653 | No |
| 19 | FGF13 | FGF13 Entrez,  Source | fibroblast growth factor 13 | 3984 | 0.055 | 0.1697 | No |
| 20 | FGF5 | FGF5 Entrez,  Source | fibroblast growth factor 5 | 4593 | 0.046 | 0.1458 | No |
| 21 | FGF8 | FGF8 Entrez,  Source | fibroblast growth factor 8 (androgen-induced) | 4765 | 0.043 | 0.1439 | No |
| 22 | PIK3CD | PIK3CD Entrez,  Source | phosphoinositide-3-kinase, catalytic, delta polypeptide | 5186 | 0.037 | 0.1283 | No |
| 23 | PTEN | PTEN Entrez,  Source | phosphatase and tensin homolog (mutated in multiple advanced cancers 1) | 5375 | 0.035 | 0.1242 | No |
| 24 | RAF1 | RAF1 Entrez,  Source | v-raf-1 murine leukemia viral oncogene homolog 1 | 5927 | 0.028 | 0.1004 | No |
| 25 | MAP2K1 | MAP2K1 Entrez,  Source | mitogen-activated protein kinase kinase 1 | 5929 | 0.028 | 0.1049 | No |
| 26 | FGF12 | FGF12 Entrez,  Source | fibroblast growth factor 12 | 6234 | 0.025 | 0.0933 | No |
| 27 | FGF10 | FGF10 Entrez,  Source | fibroblast growth factor 10 | 6532 | 0.022 | 0.0816 | No |
| 28 | MAP2K2 | MAP2K2 Entrez,  Source | mitogen-activated protein kinase kinase 2 | 6577 | 0.022 | 0.0828 | No |
| 29 | HGF | HGF Entrez,  Source | hepatocyte growth factor (hepapoietin A; scatter factor) | 6580 | 0.022 | 0.0861 | No |
| 30 | PIK3CG | PIK3CG Entrez,  Source | phosphoinositide-3-kinase, catalytic, gamma polypeptide | 7031 | 0.017 | 0.0657 | No |
| 31 | E2F2 | E2F2 Entrez,  Source | E2F transcription factor 2 | 7856 | 0.009 | 0.0248 | No |
| 32 | AKT1 | AKT1 Entrez,  Source | v-akt murine thymoma viral oncogene homolog 1 | 7896 | 0.008 | 0.0241 | No |
| 33 | RB1 | RB1 Entrez,  Source | retinoblastoma 1 (including osteosarcoma) | 7991 | 0.007 | 0.0204 | No |
| 34 | HRAS | HRAS Entrez,  Source | v-Ha-ras Harvey rat sarcoma viral oncogene homolog | 8944 | -0.001 | -0.0282 | No |
| 35 | PIK3R5 | PIK3R5 Entrez,  Source | phosphoinositide-3-kinase, regulatory subunit 5, p101 | 9214 | -0.004 | -0.0414 | No |
| 36 | FGF16 | FGF16 Entrez,  Source | fibroblast growth factor 16 | 9239 | -0.004 | -0.0421 | No |
| 37 | PDGFB | PDGFB Entrez,  Source | platelet-derived growth factor beta polypeptide (simian sarcoma viral (v-sis) oncogene homolog) | 9297 | -0.004 | -0.0443 | No |
| 38 | FGF6 | FGF6 Entrez,  Source | fibroblast growth factor 6 | 9621 | -0.007 | -0.0597 | No |
| 39 | E2F3 | E2F3 Entrez,  Source | E2F transcription factor 3 | 9752 | -0.008 | -0.0650 | No |
| 40 | PDGFRA | PDGFRA Entrez,  Source | platelet-derived growth factor receptor, alpha polypeptide | 10115 | -0.011 | -0.0818 | No |
| 41 | ARAF | ARAF Entrez,  Source | v-raf murine sarcoma 3611 viral oncogene homolog | 10441 | -0.015 | -0.0961 | No |
| 42 | PDGFD | PDGFD Entrez,  Source | platelet derived growth factor D | 10802 | -0.018 | -0.1118 | No |
| 43 | AKT2 | AKT2 Entrez,  Source | v-akt murine thymoma viral oncogene homolog 2 | 11074 | -0.020 | -0.1226 | No |
| 44 | FGF23 | FGF23 Entrez,  Source | fibroblast growth factor 23 | 11143 | -0.020 | -0.1229 | No |
| 45 | PIK3R1 | PIK3R1 Entrez,  Source | phosphoinositide-3-kinase, regulatory subunit 1 (p85 alpha) | 11545 | -0.024 | -0.1396 | No |
| 46 | FGF17 | FGF17 Entrez,  Source | fibroblast growth factor 17 | 11556 | -0.024 | -0.1363 | No |
| 47 | CDKN2A | CDKN2A Entrez,  Source | cyclin-dependent kinase inhibitor 2A (melanoma, p16, inhibits CDK4) | 11732 | -0.026 | -0.1412 | No |
| 48 | FGF22 | FGF22 Entrez,  Source | fibroblast growth factor 22 | 11763 | -0.026 | -0.1386 | No |
| 49 | MDM2 | MDM2 Entrez,  Source | Mdm2, transformed 3T3 cell double minute 2, p53 binding protein (mouse) | 11805 | -0.026 | -0.1365 | No |
| 50 | PDGFRB | PDGFRB Entrez,  Source | platelet-derived growth factor receptor, beta polypeptide | 11817 | -0.027 | -0.1329 | No |
| 51 | PDGFC | PDGFC Entrez,  Source | platelet derived growth factor C | 11872 | -0.027 | -0.1313 | No |
| 52 | IGF1 | IGF1 Entrez,  Source | insulin-like growth factor 1 (somatomedin C) | 12085 | -0.029 | -0.1376 | No |
| 53 | MET | MET Entrez,  Source | met proto-oncogene (hepatocyte growth factor receptor) | 12453 | -0.033 | -0.1513 | No |
| 54 | FGF11 | FGF11 Entrez,  Source | fibroblast growth factor 11 | 12996 | -0.038 | -0.1730 | No |
| 55 | PIK3R2 | PIK3R2 Entrez,  Source | phosphoinositide-3-kinase, regulatory subunit 2 (p85 beta) | 13588 | -0.044 | -0.1963 | No |
| 56 | FGFR1 | FGFR1 Entrez,  Source | fibroblast growth factor receptor 1 (fms-related tyrosine kinase 2, Pfeiffer syndrome) | 13631 | -0.045 | -0.1913 | No |
| 57 | FGF1 | FGF1 Entrez,  Source | fibroblast growth factor 1 (acidic) | 14340 | -0.053 | -0.2192 | No |
| 58 | FGF4 | FGF4 Entrez,  Source | fibroblast growth factor 4 (heparin secretory transforming protein 1, Kaposi sarcoma oncogene) | 15240 | -0.066 | -0.2548 | No |
| 59 | AKT3 | AKT3 Entrez,  Source | v-akt murine thymoma viral oncogene homolog 3 (protein kinase B, gamma) | 15572 | -0.071 | -0.2605 | No |
| 60 | CCND1 | CCND1 Entrez,  Source | cyclin D1 | 15740 | -0.074 | -0.2572 | No |
| 61 | E2F1 | E2F1 Entrez,  Source | E2F transcription factor 1 | 16921 | -0.097 | -0.3024 | No |
| 62 | IGF1R | IGF1R Entrez,  Source | insulin-like growth factor 1 receptor | 17435 | -0.109 | -0.3115 | No |
| 63 | FGF19 | FGF19 Entrez,  Source | fibroblast growth factor 19 | 18246 | -0.138 | -0.3310 | Yes |
| 64 | TP53 | TP53 Entrez,  Source | tumor protein p53 (Li-Fraumeni syndrome) | 18679 | -0.163 | -0.3273 | Yes |
| 65 | FGF2 | FGF2 Entrez,  Source | fibroblast growth factor 2 (basic) | 18805 | -0.175 | -0.3060 | Yes |
| 66 | CDK4 | CDK4 Entrez,  Source | cyclin-dependent kinase 4 | 18954 | -0.190 | -0.2834 | Yes |
| 67 | FGF21 | FGF21 Entrez,  Source | fibroblast growth factor 21 | 19324 | -0.259 | -0.2612 | Yes |
| 68 | CDK6 | CDK6 Entrez,  Source | cyclin-dependent kinase 6 | 19461 | -0.339 | -0.2144 | Yes |
| 69 | FGF20 | FGF20 Entrez,  Source | fibroblast growth factor 20 | 19495 | -0.390 | -0.1542 | Yes |
| 70 | FGF18 | FGF18 Entrez,  Source | fibroblast growth factor 18 | 19529 | -0.463 | -0.0823 | Yes |
| 71 | FGF9 | FGF9 Entrez,  Source | fibroblast growth factor 9 (glia-activating factor) | 19541 | -0.527 | 0.0008 | Yes |
Table: GSEA details [plain text format]

  

Fig 2: KEGG\_MELANOMA      
 Blue-Pink O' Gram in the Space of the Analyzed GeneSet

  

Fig 3: KEGG\_MELANOMA: Random ES distribution      
 Gene set null distribution of ES for **KEGG\_MELANOMA**
