# Supplementary material for: Comprehensive analysis of β-catenin target genes in colorectal carcinoma cell lines with deregulated Wnt/β-catenin signaling
Source: BMC Genomics. 2014 Jan 28;15:74. doi: 10.1186/1471-2164-15-74 (PMC3909937; doi:10.1186/1471-2164-15-74)
Supplement: Additional file 5 — GSEA analysis using the KEGG pathway database. This zipped file contains confirming data of the GSEA analysis. The names of the directories containing the files were composed of the term ‘GSEA’, the name of the cell line, e.g. DLD1, SW480, or LS174T, and the pathway database (KEGG). Please use a web browser to view the files with the name ‘index.html’ in the corresponding directories to start exploring the data. [file 1471-2164-15-74-S5.zip › GSEA_KEGG_DLD1/KEGG_METABOLISM_OF_XENOBIOTICS_BY_CYTOCHROME_P450.html]

Details for gene set KEGG\_METABOLISM\_OF\_XENOBIOTICS\_BY\_CYTOCHROME\_P450[GSEA]

|  || Dataset | DLD1\_collapsed\_to\_symbols.class.cls#b\_versus\_bg.class.cls#b\_versus\_bg\_repos |
| Phenotype | class.cls#b\_versus\_bg\_repos |
| Upregulated in class | 1 |
| GeneSet | KEGG\_METABOLISM\_OF\_XENOBIOTICS\_BY\_CYTOCHROME\_P450 |
| Enrichment Score (ES) | 0.51524657 |
| Normalized Enrichment Score (NES) | 1.7588342 |
| Nominal p-value | 0.0043572984 |
| FDR q-value | 0.16358757 |
| FWER p-Value | 0.327 |
Table: GSEA Results Summary

  

Fig 1: Enrichment plot: KEGG\_METABOLISM\_OF\_XENOBIOTICS\_BY\_CYTOCHROME\_P450      
 Profile of the Running ES Score & Positions of GeneSet Members on the Rank Ordered List

  

| PROBE | GENE SYMBOL | GENE\_TITLE | RANK IN GENE LIST | RANK METRIC SCORE | RUNNING ES | CORE ENRICHMENT || 1 | AKR1C1 | AKR1C1 Entrez,  Source | aldo-keto reductase family 1, member C1 (dihydrodiol dehydrogenase 1; 20-alpha (3-alpha)-hydroxysteroid dehydrogenase) | 24 | 0.690 | 0.1248 | Yes |
| 2 | AKR1C2 | AKR1C2 Entrez,  Source | aldo-keto reductase family 1, member C2 (dihydrodiol dehydrogenase 2; bile acid binding protein; 3-alpha hydroxysteroid dehydrogenase, type III) | 37 | 0.640 | 0.2411 | Yes |
| 3 | ALDH3B2 | ALDH3B2 Entrez,  Source | aldehyde dehydrogenase 3 family, member B2 | 220 | 0.315 | 0.2893 | Yes |
| 4 | GSTA4 | GSTA4 Entrez,  Source | glutathione S-transferase A4 | 269 | 0.285 | 0.3388 | Yes |
| 5 | AKR1C3 | AKR1C3 Entrez,  Source | aldo-keto reductase family 1, member C3 (3-alpha hydroxysteroid dehydrogenase, type II) | 282 | 0.277 | 0.3888 | Yes |
| 6 | ALDH3B1 | ALDH3B1 Entrez,  Source | aldehyde dehydrogenase 3 family, member B1 | 295 | 0.271 | 0.4376 | Yes |
| 7 | CYP2S1 | CYP2S1 Entrez,  Source | cytochrome P450, family 2, subfamily S, polypeptide 1 | 433 | 0.223 | 0.4713 | Yes |
| 8 | CYP1B1 | CYP1B1 Entrez,  Source | cytochrome P450, family 1, subfamily B, polypeptide 1 | 501 | 0.208 | 0.5058 | Yes |
| 9 | AKR1C4 | AKR1C4 Entrez,  Source | aldo-keto reductase family 1, member C4 (chlordecone reductase; 3-alpha hydroxysteroid dehydrogenase, type I; dihydrodiol dehydrogenase 4) | 886 | 0.159 | 0.5151 | Yes |
| 10 | GSTA1 | GSTA1 Entrez,  Source | glutathione S-transferase A1 | 1333 | 0.126 | 0.5152 | Yes |
| 11 | UGT2B17 | UGT2B17 Entrez,  Source | UDP glucuronosyltransferase 2 family, polypeptide B17 | 2280 | 0.091 | 0.4834 | No |
| 12 | CYP2B6 | CYP2B6 Entrez,  Source | cytochrome P450, family 2, subfamily B, polypeptide 6 | 2745 | 0.079 | 0.4740 | No |
| 13 | MGST1 | MGST1 Entrez,  Source | microsomal glutathione S-transferase 1 | 3063 | 0.072 | 0.4709 | No |
| 14 | GSTK1 | GSTK1 Entrez,  Source | glutathione S-transferase kappa 1 | 3303 | 0.067 | 0.4708 | No |
| 15 | ALDH3A1 | ALDH3A1 Entrez,  Source | aldehyde dehydrogenase 3 family, memberA1 | 3597 | 0.061 | 0.4670 | No |
| 16 | MGST3 | MGST3 Entrez,  Source | microsomal glutathione S-transferase 3 | 3653 | 0.060 | 0.4751 | No |
| 17 | ADH1C | ADH1C Entrez,  Source | alcohol dehydrogenase 1C (class I), gamma polypeptide | 4309 | 0.050 | 0.4506 | No |
| 18 | GSTT1 | GSTT1 Entrez,  Source | glutathione S-transferase theta 1 | 4407 | 0.048 | 0.4545 | No |
| 19 | ALDH1A3 | ALDH1A3 Entrez,  Source | aldehyde dehydrogenase 1 family, member A3 | 4862 | 0.042 | 0.4388 | No |
| 20 | ADH1A | ADH1A Entrez,  Source | alcohol dehydrogenase 1A (class I), alpha polypeptide | 4943 | 0.041 | 0.4421 | No |
| 21 | DHDH | DHDH Entrez,  Source | dihydrodiol dehydrogenase (dimeric) | 5410 | 0.035 | 0.4245 | No |
| 22 | GSTM3 | GSTM3 Entrez,  Source | glutathione S-transferase M3 (brain) | 5938 | 0.028 | 0.4027 | No |
| 23 | GSTA3 | GSTA3 Entrez,  Source | glutathione S-transferase A3 | 5989 | 0.028 | 0.4052 | No |
| 24 | CYP3A5 | CYP3A5 Entrez,  Source | cytochrome P450, family 3, subfamily A, polypeptide 5 | 6047 | 0.027 | 0.4072 | No |
| 25 | GSTP1 | GSTP1 Entrez,  Source | glutathione S-transferase pi | 6799 | 0.019 | 0.3722 | No |
| 26 | UGT1A9 | UGT1A9 Entrez,  Source | UDP glucuronosyltransferase 1 family, polypeptide A9 | 7185 | 0.015 | 0.3553 | No |
| 27 | CYP2F1 | CYP2F1 Entrez,  Source | cytochrome P450, family 2, subfamily F, polypeptide 1 | 7220 | 0.015 | 0.3564 | No |
| 28 | EPHX1 | EPHX1 Entrez,  Source | epoxide hydrolase 1, microsomal (xenobiotic) | 7543 | 0.012 | 0.3420 | No |
| 29 | ADH1B | ADH1B Entrez,  Source | alcohol dehydrogenase IB (class I), beta polypeptide | 8201 | 0.005 | 0.3093 | No |
| 30 | GSTO2 | GSTO2 Entrez,  Source | glutathione S-transferase omega 2 | 8389 | 0.004 | 0.3003 | No |
| 31 | ADH5 | ADH5 Entrez,  Source | alcohol dehydrogenase 5 (class III), chi polypeptide | 8645 | 0.001 | 0.2875 | No |
| 32 | GSTZ1 | GSTZ1 Entrez,  Source | glutathione transferase zeta 1 (maleylacetoacetate isomerase) | 8807 | -0.000 | 0.2793 | No |
| 33 | MGST2 | MGST2 Entrez,  Source | microsomal glutathione S-transferase 2 | 8881 | -0.001 | 0.2757 | No |
| 34 | CYP2E1 | CYP2E1 Entrez,  Source | cytochrome P450, family 2, subfamily E, polypeptide 1 | 9672 | -0.008 | 0.2366 | No |
| 35 | CYP3A4 | CYP3A4 Entrez,  Source | cytochrome P450, family 3, subfamily A, polypeptide 4 | 10040 | -0.011 | 0.2198 | No |
| 36 | GSTO1 | GSTO1 Entrez,  Source | glutathione S-transferase omega 1 | 10128 | -0.012 | 0.2174 | No |
| 37 | ADH7 | ADH7 Entrez,  Source | alcohol dehydrogenase 7 (class IV), mu or sigma polypeptide | 10950 | -0.019 | 0.1788 | No |
| 38 | CYP3A7 | CYP3A7 Entrez,  Source | cytochrome P450, family 3, subfamily A, polypeptide 7 | 11079 | -0.020 | 0.1758 | No |
| 39 | ADH4 | ADH4 Entrez,  Source | alcohol dehydrogenase 4 (class II), pi polypeptide | 11579 | -0.024 | 0.1547 | No |
| 40 | UGT2B15 | UGT2B15 Entrez,  Source | UDP glucuronosyltransferase 2 family, polypeptide B15 | 11818 | -0.027 | 0.1473 | No |
| 41 | GSTM5 | GSTM5 Entrez,  Source | glutathione S-transferase M5 | 12122 | -0.029 | 0.1372 | No |
| 42 | CYP2C18 | CYP2C18 Entrez,  Source | cytochrome P450, family 2, subfamily C, polypeptide 18 | 12799 | -0.036 | 0.1091 | No |
| 43 | CYP3A43 | CYP3A43 Entrez,  Source | cytochrome P450, family 3, subfamily A, polypeptide 43 | 13489 | -0.044 | 0.0817 | No |
| 44 | CYP1A2 | CYP1A2 Entrez,  Source | cytochrome P450, family 1, subfamily A, polypeptide 2 | 13895 | -0.048 | 0.0697 | No |
| 45 | CYP2C9 | CYP2C9 Entrez,  Source | cytochrome P450, family 2, subfamily C, polypeptide 9 | 14384 | -0.054 | 0.0545 | No |
| 46 | ADH6 | ADH6 Entrez,  Source | alcohol dehydrogenase 6 (class V) | 14819 | -0.060 | 0.0432 | No |
| 47 | CYP2C19 | CYP2C19 Entrez,  Source | cytochrome P450, family 2, subfamily C, polypeptide 19 | 14980 | -0.062 | 0.0464 | No |
| 48 | UGT2B28 | UGT2B28 Entrez,  Source | UDP glucuronosyltransferase 2 family, polypeptide B28 | 15270 | -0.067 | 0.0437 | No |
| 49 | GSTT2 | GSTT2 Entrez,  Source | glutathione S-transferase theta 2 | 15655 | -0.073 | 0.0373 | No |
| 50 | GSTM2 | GSTM2 Entrez,  Source | glutathione S-transferase M2 (muscle) | 16147 | -0.081 | 0.0270 | No |
| 51 | GSTM1 | GSTM1 Entrez,  Source | glutathione S-transferase M1 | 16741 | -0.093 | 0.0136 | No |
| 52 | UGT2A3 | UGT2A3 Entrez,  Source | UDP glucuronosyltransferase 2 family, polypeptide A3 | 16986 | -0.098 | 0.0190 | No |
| 53 | GSTM4 | GSTM4 Entrez,  Source | glutathione S-transferase M4 | 17904 | -0.124 | -0.0053 | No |
| 54 | UGT2B4 | UGT2B4 Entrez,  Source | UDP glucuronosyltransferase 2 family, polypeptide B4 | 18107 | -0.132 | 0.0085 | No |
| 55 | CYP2C8 | CYP2C8 Entrez,  Source | cytochrome P450, family 2, subfamily C, polypeptide 8 | 18452 | -0.148 | 0.0179 | No |
| 56 | CYP1A1 | CYP1A1 Entrez,  Source | cytochrome P450, family 1, subfamily A, polypeptide 1 | 19122 | -0.212 | 0.0223 | No |
Table: GSEA details [plain text format]

  

Fig 2: KEGG\_METABOLISM\_OF\_XENOBIOTICS\_BY\_CYTOCHROME\_P450      
 Blue-Pink O' Gram in the Space of the Analyzed GeneSet

  

Fig 3: KEGG\_METABOLISM\_OF\_XENOBIOTICS\_BY\_CYTOCHROME\_P450: Random ES distribution      
 Gene set null distribution of ES for **KEGG\_METABOLISM\_OF\_XENOBIOTICS\_BY\_CYTOCHROME\_P450**
