# Supplementary material for: Comprehensive analysis of β-catenin target genes in colorectal carcinoma cell lines with deregulated Wnt/β-catenin signaling
Source: BMC Genomics. 2014 Jan 28;15:74. doi: 10.1186/1471-2164-15-74 (PMC3909937; doi:10.1186/1471-2164-15-74)
Supplement: Additional file 5 — GSEA analysis using the KEGG pathway database. This zipped file contains confirming data of the GSEA analysis. The names of the directories containing the files were composed of the term ‘GSEA’, the name of the cell line, e.g. DLD1, SW480, or LS174T, and the pathway database (KEGG). Please use a web browser to view the files with the name ‘index.html’ in the corresponding directories to start exploring the data. [file 1471-2164-15-74-S5.zip › GSEA_KEGG_DLD1/KEGG_MISMATCH_REPAIR.html]

Details for gene set KEGG\_MISMATCH\_REPAIR[GSEA]

|  || Dataset | DLD1\_collapsed\_to\_symbols.class.cls#b\_versus\_bg.class.cls#b\_versus\_bg\_repos |
| Phenotype | class.cls#b\_versus\_bg\_repos |
| Upregulated in class | 0 |
| GeneSet | KEGG\_MISMATCH\_REPAIR |
| Enrichment Score (ES) | -0.6773104 |
| Normalized Enrichment Score (NES) | -1.9296002 |
| Nominal p-value | 0.0 |
| FDR q-value | 0.005522692 |
| FWER p-Value | 0.02 |
Table: GSEA Results Summary

  

Fig 1: Enrichment plot: KEGG\_MISMATCH\_REPAIR      
 Profile of the Running ES Score & Positions of GeneSet Members on the Rank Ordered List

  

| PROBE | GENE SYMBOL | GENE\_TITLE | RANK IN GENE LIST | RANK METRIC SCORE | RUNNING ES | CORE ENRICHMENT || 1 | POLD4 | POLD4 Entrez,  Source | polymerase (DNA-directed), delta 4 | 403 | 0.230 | 0.0595 | No |
| 2 | MSH3 | MSH3 Entrez,  Source | mutS homolog 3 (E. coli) | 5127 | 0.038 | -0.1690 | No |
| 3 | MLH1 | MLH1 Entrez,  Source | mutL homolog 1, colon cancer, nonpolyposis type 2 (E. coli) | 13261 | -0.041 | -0.5710 | No |
| 4 | RPA1 | RPA1 Entrez,  Source | replication protein A1, 70kDa | 13670 | -0.045 | -0.5761 | No |
| 5 | SSBP1 | SSBP1 Entrez,  Source | single-stranded DNA binding protein 1 | 13868 | -0.048 | -0.5697 | No |
| 6 | RFC1 | RFC1 Entrez,  Source | replication factor C (activator 1) 1, 145kDa | 14515 | -0.056 | -0.5834 | No |
| 7 | RPA3 | RPA3 Entrez,  Source | replication protein A3, 14kDa | 14651 | -0.057 | -0.5703 | No |
| 8 | MLH3 | MLH3 Entrez,  Source | mutL homolog 3 (E. coli) | 15877 | -0.077 | -0.6063 | No |
| 9 | PCNA | PCNA Entrez,  Source | proliferating cell nuclear antigen | 17265 | -0.105 | -0.6409 | Yes |
| 10 | RPA4 | RPA4 Entrez,  Source | replication protein A4, 34kDa | 17899 | -0.124 | -0.6300 | Yes |
| 11 | RFC2 | RFC2 Entrez,  Source | replication factor C (activator 1) 2, 40kDa | 18035 | -0.129 | -0.5921 | Yes |
| 12 | MSH2 | MSH2 Entrez,  Source | mutS homolog 2, colon cancer, nonpolyposis type 1 (E. coli) | 18126 | -0.133 | -0.5505 | Yes |
| 13 | POLD2 | POLD2 Entrez,  Source | polymerase (DNA directed), delta 2, regulatory subunit 50kDa | 18279 | -0.140 | -0.5096 | Yes |
| 14 | LIG1 | LIG1 Entrez,  Source | ligase I, DNA, ATP-dependent | 18381 | -0.145 | -0.4643 | Yes |
| 15 | RFC3 | RFC3 Entrez,  Source | replication factor C (activator 1) 3, 38kDa | 18474 | -0.149 | -0.4170 | Yes |
| 16 | RPA2 | RPA2 Entrez,  Source | replication protein A2, 32kDa | 18689 | -0.164 | -0.3708 | Yes |
| 17 | RFC5 | RFC5 Entrez,  Source | replication factor C (activator 1) 5, 36.5kDa | 18762 | -0.170 | -0.3153 | Yes |
| 18 | POLD3 | POLD3 Entrez,  Source | polymerase (DNA-directed), delta 3, accessory subunit | 18829 | -0.177 | -0.2570 | Yes |
| 19 | RFC4 | RFC4 Entrez,  Source | replication factor C (activator 1) 4, 37kDa | 18960 | -0.191 | -0.1974 | Yes |
| 20 | POLD1 | POLD1 Entrez,  Source | polymerase (DNA directed), delta 1, catalytic subunit 125kDa | 19075 | -0.205 | -0.1318 | Yes |
| 21 | MSH6 | MSH6 Entrez,  Source | mutS homolog 6 (E. coli) | 19110 | -0.210 | -0.0605 | Yes |
| 22 | EXO1 | EXO1 Entrez,  Source | exonuclease 1 | 19265 | -0.239 | 0.0149 | Yes |
Table: GSEA details [plain text format]

  

Fig 2: KEGG\_MISMATCH\_REPAIR      
 Blue-Pink O' Gram in the Space of the Analyzed GeneSet

  

Fig 3: KEGG\_MISMATCH\_REPAIR: Random ES distribution      
 Gene set null distribution of ES for **KEGG\_MISMATCH\_REPAIR**
