# Supplementary material for: Comprehensive analysis of β-catenin target genes in colorectal carcinoma cell lines with deregulated Wnt/β-catenin signaling
Source: BMC Genomics. 2014 Jan 28;15:74. doi: 10.1186/1471-2164-15-74 (PMC3909937; doi:10.1186/1471-2164-15-74)
Supplement: Additional file 5 — GSEA analysis using the KEGG pathway database. This zipped file contains confirming data of the GSEA analysis. The names of the directories containing the files were composed of the term ‘GSEA’, the name of the cell line, e.g. DLD1, SW480, or LS174T, and the pathway database (KEGG). Please use a web browser to view the files with the name ‘index.html’ in the corresponding directories to start exploring the data. [file 1471-2164-15-74-S5.zip › GSEA_KEGG_DLD1/KEGG_ONE_CARBON_POOL_BY_FOLATE.html]

Details for gene set KEGG\_ONE\_CARBON\_POOL\_BY\_FOLATE[GSEA]

|  || Dataset | DLD1\_collapsed\_to\_symbols.class.cls#b\_versus\_bg.class.cls#b\_versus\_bg\_repos |
| Phenotype | class.cls#b\_versus\_bg\_repos |
| Upregulated in class | 0 |
| GeneSet | KEGG\_ONE\_CARBON\_POOL\_BY\_FOLATE |
| Enrichment Score (ES) | -0.56808525 |
| Normalized Enrichment Score (NES) | -1.5407122 |
| Nominal p-value | 0.034285713 |
| FDR q-value | 0.089705475 |
| FWER p-Value | 0.791 |
Table: GSEA Results Summary

  

Fig 1: Enrichment plot: KEGG\_ONE\_CARBON\_POOL\_BY\_FOLATE      
 Profile of the Running ES Score & Positions of GeneSet Members on the Rank Ordered List

  

| PROBE | GENE SYMBOL | GENE\_TITLE | RANK IN GENE LIST | RANK METRIC SCORE | RUNNING ES | CORE ENRICHMENT || 1 | AMT | AMT Entrez,  Source | aminomethyltransferase | 7472 | 0.012 | -0.3709 | No |
| 2 | MTHFD2L | MTHFD2L Entrez,  Source | methylenetetrahydrofolate dehydrogenase (NADP+ dependent) 2-like | 8138 | 0.006 | -0.3995 | No |
| 3 | MTHFR | MTHFR Entrez,  Source | 5,10-methylenetetrahydrofolate reductase (NADPH) | 10194 | -0.012 | -0.4935 | No |
| 4 | MTHFD2 | MTHFD2 Entrez,  Source | methylenetetrahydrofolate dehydrogenase (NADP+ dependent) 2, methenyltetrahydrofolate cyclohydrolase | 11340 | -0.022 | -0.5318 | No |
| 5 | ALDH1L1 | ALDH1L1 Entrez,  Source | aldehyde dehydrogenase 1 family, member L1 | 12051 | -0.029 | -0.5417 | Yes |
| 6 | FTCD | FTCD Entrez,  Source | formiminotransferase cyclodeaminase | 12188 | -0.030 | -0.5211 | Yes |
| 7 | SHMT1 | SHMT1 Entrez,  Source | serine hydroxymethyltransferase 1 (soluble) | 12778 | -0.036 | -0.5183 | Yes |
| 8 | MTHFS | MTHFS Entrez,  Source | 5,10-methenyltetrahydrofolate synthetase (5-formyltetrahydrofolate cyclo-ligase) | 13176 | -0.040 | -0.5019 | Yes |
| 9 | MTFMT | MTFMT Entrez,  Source | mitochondrial methionyl-tRNA formyltransferase | 14255 | -0.052 | -0.5092 | Yes |
| 10 | ATIC | ATIC Entrez,  Source | 5-aminoimidazole-4-carboxamide ribonucleotide formyltransferase/IMP cyclohydrolase | 14735 | -0.059 | -0.4798 | Yes |
| 11 | TYMS | TYMS Entrez,  Source | thymidylate synthetase | 14974 | -0.062 | -0.4347 | Yes |
| 12 | MTHFD1 | MTHFD1 Entrez,  Source | methylenetetrahydrofolate dehydrogenase (NADP+ dependent) 1, methenyltetrahydrofolate cyclohydrolase, formyltetrahydrofolate synthetase | 16968 | -0.098 | -0.4471 | Yes |
| 13 | SHMT2 | SHMT2 Entrez,  Source | serine hydroxymethyltransferase 2 (mitochondrial) | 17832 | -0.122 | -0.3792 | Yes |
| 14 | GART | GART Entrez,  Source | phosphoribosylglycinamide formyltransferase, phosphoribosylglycinamide synthetase, phosphoribosylaminoimidazole synthetase | 18647 | -0.161 | -0.2732 | Yes |
| 15 | MTR | MTR Entrez,  Source | 5-methyltetrahydrofolate-homocysteine methyltransferase | 18778 | -0.172 | -0.1222 | Yes |
| 16 | MTHFD1L | MTHFD1L Entrez,  Source | methylenetetrahydrofolate dehydrogenase (NADP+ dependent) 1-like | 18823 | -0.176 | 0.0375 | Yes |
Table: GSEA details [plain text format]

  

Fig 2: KEGG\_ONE\_CARBON\_POOL\_BY\_FOLATE      
 Blue-Pink O' Gram in the Space of the Analyzed GeneSet

  

Fig 3: KEGG\_ONE\_CARBON\_POOL\_BY\_FOLATE: Random ES distribution      
 Gene set null distribution of ES for **KEGG\_ONE\_CARBON\_POOL\_BY\_FOLATE**
