# Supplementary material for: Comprehensive analysis of β-catenin target genes in colorectal carcinoma cell lines with deregulated Wnt/β-catenin signaling
Source: BMC Genomics. 2014 Jan 28;15:74. doi: 10.1186/1471-2164-15-74 (PMC3909937; doi:10.1186/1471-2164-15-74)
Supplement: Additional file 5 — GSEA analysis using the KEGG pathway database. This zipped file contains confirming data of the GSEA analysis. The names of the directories containing the files were composed of the term ‘GSEA’, the name of the cell line, e.g. DLD1, SW480, or LS174T, and the pathway database (KEGG). Please use a web browser to view the files with the name ‘index.html’ in the corresponding directories to start exploring the data. [file 1471-2164-15-74-S5.zip › GSEA_KEGG_DLD1/KEGG_PHOSPHATIDYLINOSITOL_SIGNALING_SYSTEM.html]

Details for gene set KEGG\_PHOSPHATIDYLINOSITOL\_SIGNALING\_SYSTEM[GSEA]

|  || Dataset | DLD1\_collapsed\_to\_symbols.class.cls#b\_versus\_bg.class.cls#b\_versus\_bg\_repos |
| Phenotype | class.cls#b\_versus\_bg\_repos |
| Upregulated in class | 1 |
| GeneSet | KEGG\_PHOSPHATIDYLINOSITOL\_SIGNALING\_SYSTEM |
| Enrichment Score (ES) | 0.46184373 |
| Normalized Enrichment Score (NES) | 1.6656698 |
| Nominal p-value | 0.005988024 |
| FDR q-value | 0.15170501 |
| FWER p-Value | 0.589 |
Table: GSEA Results Summary

  

Fig 1: Enrichment plot: KEGG\_PHOSPHATIDYLINOSITOL\_SIGNALING\_SYSTEM      
 Profile of the Running ES Score & Positions of GeneSet Members on the Rank Ordered List

  

| PROBE | GENE SYMBOL | GENE\_TITLE | RANK IN GENE LIST | RANK METRIC SCORE | RUNNING ES | CORE ENRICHMENT || 1 | PLCE1 | PLCE1 Entrez,  Source | phospholipase C, epsilon 1 | 41 | 0.610 | 0.1095 | Yes |
| 2 | PIK3R3 | PIK3R3 Entrez,  Source | phosphoinositide-3-kinase, regulatory subunit 3 (p55, gamma) | 247 | 0.296 | 0.1531 | Yes |
| 3 | PLCD3 | PLCD3 Entrez,  Source | phospholipase C, delta 3 | 279 | 0.278 | 0.2024 | Yes |
| 4 | CALML5 | CALML5 Entrez,  Source | calmodulin-like 5 | 467 | 0.216 | 0.2323 | Yes |
| 5 | DGKI | DGKI Entrez,  Source | diacylglycerol kinase, iota | 541 | 0.200 | 0.2652 | Yes |
| 6 | DGKA | DGKA Entrez,  Source | diacylglycerol kinase, alpha 80kDa | 641 | 0.184 | 0.2938 | Yes |
| 7 | PIP5K1B | PIP5K1B Entrez,  Source | phosphatidylinositol-4-phosphate 5-kinase, type I, beta | 649 | 0.183 | 0.3269 | Yes |
| 8 | SYNJ1 | SYNJ1 Entrez,  Source | synaptojanin 1 | 1037 | 0.147 | 0.3339 | Yes |
| 9 | DGKE | DGKE Entrez,  Source | diacylglycerol kinase, epsilon 64kDa | 1421 | 0.122 | 0.3366 | Yes |
| 10 | CDS2 | CDS2 Entrez,  Source | CDP-diacylglycerol synthase (phosphatidate cytidylyltransferase) 2 | 1772 | 0.107 | 0.3381 | Yes |
| 11 | PIK3CB | PIK3CB Entrez,  Source | phosphoinositide-3-kinase, catalytic, beta polypeptide | 1862 | 0.104 | 0.3525 | Yes |
| 12 | PIK3CA | PIK3CA Entrez,  Source | phosphoinositide-3-kinase, catalytic, alpha polypeptide | 1955 | 0.101 | 0.3662 | Yes |
| 13 | PLCB3 | PLCB3 Entrez,  Source | phospholipase C, beta 3 (phosphatidylinositol-specific) | 2167 | 0.094 | 0.3726 | Yes |
| 14 | INPP5A | INPP5A Entrez,  Source | inositol polyphosphate-5-phosphatase, 40kDa | 2215 | 0.093 | 0.3871 | Yes |
| 15 | CALM3 | CALM3 Entrez,  Source | calmodulin 3 (phosphorylase kinase, delta) | 2223 | 0.092 | 0.4036 | Yes |
| 16 | CDIPT | CDIPT Entrez,  Source | CDP-diacylglycerol--inositol 3-phosphatidyltransferase (phosphatidylinositol synthase) | 2236 | 0.092 | 0.4198 | Yes |
| 17 | DGKQ | DGKQ Entrez,  Source | diacylglycerol kinase, theta 110kDa | 2254 | 0.092 | 0.4357 | Yes |
| 18 | DGKD | DGKD Entrez,  Source | diacylglycerol kinase, delta 130kDa | 2333 | 0.089 | 0.4480 | Yes |
| 19 | PIK3C2G | PIK3C2G Entrez,  Source | phosphoinositide-3-kinase, class 2, gamma polypeptide | 2678 | 0.081 | 0.4451 | Yes |
| 20 | SYNJ2 | SYNJ2 Entrez,  Source | synaptojanin 2 | 3032 | 0.073 | 0.4403 | Yes |
| 21 | ITPR1 | ITPR1 Entrez,  Source | inositol 1,4,5-triphosphate receptor, type 1 | 3231 | 0.068 | 0.4426 | Yes |
| 22 | PLCD1 | PLCD1 Entrez,  Source | phospholipase C, delta 1 | 3396 | 0.065 | 0.4460 | Yes |
| 23 | DGKB | DGKB Entrez,  Source | diacylglycerol kinase, beta 90kDa | 3424 | 0.064 | 0.4564 | Yes |
| 24 | ITPK1 | ITPK1 Entrez,  Source | inositol 1,3,4-triphosphate 5/6 kinase | 3541 | 0.062 | 0.4618 | Yes |
| 25 | PRKCA | PRKCA Entrez,  Source | protein kinase C, alpha | 4248 | 0.051 | 0.4349 | No |
| 26 | PIP5K1C | PIP5K1C Entrez,  Source | phosphatidylinositol-4-phosphate 5-kinase, type I, gamma | 4280 | 0.050 | 0.4425 | No |
| 27 | IMPA1 | IMPA1 Entrez,  Source | inositol(myo)-1(or 4)-monophosphatase 1 | 4583 | 0.046 | 0.4353 | No |
| 28 | ITPKA | ITPKA Entrez,  Source | inositol 1,4,5-trisphosphate 3-kinase A | 4625 | 0.045 | 0.4415 | No |
| 29 | CALML3 | CALML3 Entrez,  Source | calmodulin-like 3 | 4761 | 0.043 | 0.4425 | No |
| 30 | PIK3CD | PIK3CD Entrez,  Source | phosphoinositide-3-kinase, catalytic, delta polypeptide | 5186 | 0.037 | 0.4276 | No |
| 31 | PTEN | PTEN Entrez,  Source | phosphatase and tensin homolog (mutated in multiple advanced cancers 1) | 5375 | 0.035 | 0.4243 | No |
| 32 | CALM2 | CALM2 Entrez,  Source | calmodulin 2 (phosphorylase kinase, delta) | 5521 | 0.033 | 0.4229 | No |
| 33 | IPPK | IPPK Entrez,  Source | inositol 1,3,4,5,6-pentakisphosphate 2-kinase | 5782 | 0.030 | 0.4151 | No |
| 34 | OCRL | OCRL Entrez,  Source | oculocerebrorenal syndrome of Lowe | 5935 | 0.028 | 0.4125 | No |
| 35 | PLCB1 | PLCB1 Entrez,  Source | phospholipase C, beta 1 (phosphoinositide-specific) | 6225 | 0.025 | 0.4023 | No |
| 36 | PIK3C2B | PIK3C2B Entrez,  Source | phosphoinositide-3-kinase, class 2, beta polypeptide | 6917 | 0.018 | 0.3701 | No |
| 37 | PIK3CG | PIK3CG Entrez,  Source | phosphoinositide-3-kinase, catalytic, gamma polypeptide | 7031 | 0.017 | 0.3674 | No |
| 38 | PIK3C2A | PIK3C2A Entrez,  Source | phosphoinositide-3-kinase, class 2, alpha polypeptide | 7377 | 0.013 | 0.3522 | No |
| 39 | PIK3C3 | PIK3C3 Entrez,  Source | phosphoinositide-3-kinase, class 3 | 7648 | 0.011 | 0.3403 | No |
| 40 | CDS1 | CDS1 Entrez,  Source | CDP-diacylglycerol synthase (phosphatidate cytidylyltransferase) 1 | 8021 | 0.007 | 0.3225 | No |
| 41 | DGKH | DGKH Entrez,  Source | diacylglycerol kinase, eta | 9160 | -0.003 | 0.2647 | No |
| 42 | PIK3R5 | PIK3R5 Entrez,  Source | phosphoinositide-3-kinase, regulatory subunit 5, p101 | 9214 | -0.004 | 0.2626 | No |
| 43 | INPPL1 | INPPL1 Entrez,  Source | inositol polyphosphate phosphatase-like 1 | 9404 | -0.005 | 0.2539 | No |
| 44 | DGKG | DGKG Entrez,  Source | diacylglycerol kinase, gamma 90kDa | 9470 | -0.006 | 0.2516 | No |
| 45 | CALM1 | CALM1 Entrez,  Source | calmodulin 1 (phosphorylase kinase, delta) | 9475 | -0.006 | 0.2525 | No |
| 46 | INPP1 | INPP1 Entrez,  Source | inositol polyphosphate-1-phosphatase | 9631 | -0.007 | 0.2459 | No |
| 47 | PLCB4 | PLCB4 Entrez,  Source | phospholipase C, beta 4 | 9697 | -0.008 | 0.2440 | No |
| 48 | INPP5E | INPP5E Entrez,  Source | inositol polyphosphate-5-phosphatase, 72 kDa | 10542 | -0.015 | 0.2035 | No |
| 49 | PIP5K1A | PIP5K1A Entrez,  Source | phosphatidylinositol-4-phosphate 5-kinase, type I, alpha | 10561 | -0.016 | 0.2055 | No |
| 50 | INPP5D | INPP5D Entrez,  Source | inositol polyphosphate-5-phosphatase, 145kDa | 10829 | -0.018 | 0.1950 | No |
| 51 | PIK3R1 | PIK3R1 Entrez,  Source | phosphoinositide-3-kinase, regulatory subunit 1 (p85 alpha) | 11545 | -0.024 | 0.1627 | No |
| 52 | DGKZ | DGKZ Entrez,  Source | diacylglycerol kinase, zeta 104kDa | 12682 | -0.035 | 0.1108 | No |
| 53 | PLCG2 | PLCG2 Entrez,  Source | phospholipase C, gamma 2 (phosphatidylinositol-specific) | 13226 | -0.041 | 0.0904 | No |
| 54 | ITPR3 | ITPR3 Entrez,  Source | inositol 1,4,5-triphosphate receptor, type 3 | 13254 | -0.041 | 0.0965 | No |
| 55 | PIK3R2 | PIK3R2 Entrez,  Source | phosphoinositide-3-kinase, regulatory subunit 2 (p85 beta) | 13588 | -0.044 | 0.0875 | No |
| 56 | CALML6 | CALML6 Entrez,  Source | calmodulin-like 6 | 14105 | -0.051 | 0.0703 | No |
| 57 | PRKCG | PRKCG Entrez,  Source | protein kinase C, gamma | 14320 | -0.053 | 0.0690 | No |
| 58 | INPP4A | INPP4A Entrez,  Source | inositol polyphosphate-4-phosphatase, type I, 107kDa | 14618 | -0.057 | 0.0642 | No |
| 59 | PLCB2 | PLCB2 Entrez,  Source | phospholipase C, beta 2 | 14920 | -0.062 | 0.0600 | No |
| 60 | ITPKB | ITPKB Entrez,  Source | inositol 1,4,5-trisphosphate 3-kinase B | 15343 | -0.068 | 0.0508 | No |
| 61 | INPP4B | INPP4B Entrez,  Source | inositol polyphosphate-4-phosphatase, type II, 105kDa | 15479 | -0.070 | 0.0566 | No |
| 62 | PLCD4 | PLCD4 Entrez,  Source | phospholipase C, delta 4 | 15794 | -0.075 | 0.0543 | No |
| 63 | PLCG1 | PLCG1 Entrez,  Source | phospholipase C, gamma 1 | 17513 | -0.111 | -0.0136 | No |
| 64 | INPP5B | INPP5B Entrez,  Source | inositol polyphosphate-5-phosphatase, 75kDa | 17560 | -0.113 | 0.0047 | No |
| 65 | IMPA2 | IMPA2 Entrez,  Source | inositol(myo)-1(or 4)-monophosphatase 2 | 18272 | -0.140 | -0.0062 | No |
| 66 | PLCZ1 | PLCZ1 Entrez,  Source | phospholipase C, zeta 1 | 18276 | -0.140 | 0.0192 | No |
| 67 | ITPR2 | ITPR2 Entrez,  Source | inositol 1,4,5-triphosphate receptor, type 2 | 19314 | -0.254 | 0.0124 | No |
Table: GSEA details [plain text format]

  

Fig 2: KEGG\_PHOSPHATIDYLINOSITOL\_SIGNALING\_SYSTEM      
 Blue-Pink O' Gram in the Space of the Analyzed GeneSet

  

Fig 3: KEGG\_PHOSPHATIDYLINOSITOL\_SIGNALING\_SYSTEM: Random ES distribution      
 Gene set null distribution of ES for **KEGG\_PHOSPHATIDYLINOSITOL\_SIGNALING\_SYSTEM**
