# Supplementary material for: Comprehensive analysis of β-catenin target genes in colorectal carcinoma cell lines with deregulated Wnt/β-catenin signaling
Source: BMC Genomics. 2014 Jan 28;15:74. doi: 10.1186/1471-2164-15-74 (PMC3909937; doi:10.1186/1471-2164-15-74)
Supplement: Additional file 5 — GSEA analysis using the KEGG pathway database. This zipped file contains confirming data of the GSEA analysis. The names of the directories containing the files were composed of the term ‘GSEA’, the name of the cell line, e.g. DLD1, SW480, or LS174T, and the pathway database (KEGG). Please use a web browser to view the files with the name ‘index.html’ in the corresponding directories to start exploring the data. [file 1471-2164-15-74-S5.zip › GSEA_KEGG_DLD1/KEGG_PPAR_SIGNALING_PATHWAY.html]

Details for gene set KEGG\_PPAR\_SIGNALING\_PATHWAY[GSEA]

|  || Dataset | DLD1\_collapsed\_to\_symbols.class.cls#b\_versus\_bg.class.cls#b\_versus\_bg\_repos |
| Phenotype | class.cls#b\_versus\_bg\_repos |
| Upregulated in class | 1 |
| GeneSet | KEGG\_PPAR\_SIGNALING\_PATHWAY |
| Enrichment Score (ES) | 0.50388575 |
| Normalized Enrichment Score (NES) | 1.7994118 |
| Nominal p-value | 0.0 |
| FDR q-value | 0.15917963 |
| FWER p-Value | 0.225 |
Table: GSEA Results Summary

  

Fig 1: Enrichment plot: KEGG\_PPAR\_SIGNALING\_PATHWAY      
 Profile of the Running ES Score & Positions of GeneSet Members on the Rank Ordered List

  

| PROBE | GENE SYMBOL | GENE\_TITLE | RANK IN GENE LIST | RANK METRIC SCORE | RUNNING ES | CORE ENRICHMENT || 1 | HMGCS2 | HMGCS2 Entrez,  Source | 3-hydroxy-3-methylglutaryl-Coenzyme A synthase 2 (mitochondrial) | 4 | 1.159 | 0.1716 | Yes |
| 2 | PCK1 | PCK1 Entrez,  Source | phosphoenolpyruvate carboxykinase 1 (soluble) | 43 | 0.603 | 0.2591 | Yes |
| 3 | ANGPTL4 | ANGPTL4 Entrez,  Source | angiopoietin-like 4 | 248 | 0.295 | 0.2924 | Yes |
| 4 | PPARA | PPARA Entrez,  Source | peroxisome proliferative activated receptor, alpha | 380 | 0.236 | 0.3207 | Yes |
| 5 | GK | GK Entrez,  Source | glycerol kinase | 402 | 0.230 | 0.3537 | Yes |
| 6 | ACOX1 | ACOX1 Entrez,  Source | acyl-Coenzyme A oxidase 1, palmitoyl | 505 | 0.208 | 0.3793 | Yes |
| 7 | ACAA1 | ACAA1 Entrez,  Source | acetyl-Coenzyme A acyltransferase 1 (peroxisomal 3-oxoacyl-Coenzyme A thiolase) | 596 | 0.191 | 0.4029 | Yes |
| 8 | PPARG | PPARG Entrez,  Source | peroxisome proliferative activated receptor, gamma | 820 | 0.165 | 0.4159 | Yes |
| 9 | ACSL1 | ACSL1 Entrez,  Source | acyl-CoA synthetase long-chain family member 1 | 898 | 0.157 | 0.4352 | Yes |
| 10 | OLR1 | OLR1 Entrez,  Source | oxidised low density lipoprotein (lectin-like) receptor 1 | 1091 | 0.142 | 0.4464 | Yes |
| 11 | AQP7 | AQP7 Entrez,  Source | aquaporin 7 | 1303 | 0.128 | 0.4546 | Yes |
| 12 | UCP1 | UCP1 Entrez,  Source | uncoupling protein 1 (mitochondrial, proton carrier) | 1986 | 0.100 | 0.4344 | Yes |
| 13 | CPT1A | CPT1A Entrez,  Source | carnitine palmitoyltransferase 1A (liver) | 2022 | 0.099 | 0.4473 | Yes |
| 14 | SCD | SCD Entrez,  Source | stearoyl-CoA desaturase (delta-9-desaturase) | 2093 | 0.096 | 0.4580 | Yes |
| 15 | SLC27A1 | SLC27A1 Entrez,  Source | solute carrier family 27 (fatty acid transporter), member 1 | 2121 | 0.095 | 0.4707 | Yes |
| 16 | CPT2 | CPT2 Entrez,  Source | carnitine palmitoyltransferase II | 2267 | 0.091 | 0.4768 | Yes |
| 17 | SLC27A4 | SLC27A4 Entrez,  Source | solute carrier family 27 (fatty acid transporter), member 4 | 2435 | 0.087 | 0.4811 | Yes |
| 18 | PPARD | PPARD Entrez,  Source | peroxisome proliferative activated receptor, delta | 2653 | 0.081 | 0.4820 | Yes |
| 19 | RXRA | RXRA Entrez,  Source | retinoid X receptor, alpha | 2672 | 0.081 | 0.4931 | Yes |
| 20 | PDPK1 | PDPK1 Entrez,  Source | 3-phosphoinositide dependent protein kinase-1 | 2871 | 0.076 | 0.4942 | Yes |
| 21 | ME1 | ME1 Entrez,  Source | malic enzyme 1, NADP(+)-dependent, cytosolic | 2901 | 0.075 | 0.5039 | Yes |
| 22 | ACSL3 | ACSL3 Entrez,  Source | acyl-CoA synthetase long-chain family member 3 | 3196 | 0.069 | 0.4991 | No |
| 23 | CPT1B | CPT1B Entrez,  Source | carnitine palmitoyltransferase 1B (muscle) | 3579 | 0.062 | 0.4886 | No |
| 24 | MMP1 | MMP1 Entrez,  Source | matrix metallopeptidase 1 (interstitial collagenase) | 3925 | 0.056 | 0.4791 | No |
| 25 | ACSL5 | ACSL5 Entrez,  Source | acyl-CoA synthetase long-chain family member 5 | 4261 | 0.050 | 0.4694 | No |
| 26 | SCP2 | SCP2 Entrez,  Source | sterol carrier protein 2 | 4477 | 0.047 | 0.4654 | No |
| 27 | DBI | DBI Entrez,  Source | diazepam binding inhibitor (GABA receptor modulator, acyl-Coenzyme A binding protein) | 4939 | 0.041 | 0.4478 | No |
| 28 | GK2 | GK2 Entrez,  Source | glycerol kinase 2 | 4951 | 0.041 | 0.4532 | No |
| 29 | SLC27A2 | SLC27A2 Entrez,  Source | solute carrier family 27 (fatty acid transporter), member 2 | 4981 | 0.040 | 0.4577 | No |
| 30 | ACADL | ACADL Entrez,  Source | acyl-Coenzyme A dehydrogenase, long chain | 5373 | 0.035 | 0.4428 | No |
| 31 | APOA5 | APOA5 Entrez,  Source | apolipoprotein A-V | 6299 | 0.025 | 0.3990 | No |
| 32 | ILK | ILK Entrez,  Source | integrin-linked kinase | 6937 | 0.018 | 0.3690 | No |
| 33 | FABP3 | FABP3 Entrez,  Source | fatty acid binding protein 3, muscle and heart (mammary-derived growth inhibitor) | 6942 | 0.018 | 0.3714 | No |
| 34 | UBC | UBC Entrez,  Source | ubiquitin C | 6954 | 0.018 | 0.3734 | No |
| 35 | LPL | LPL Entrez,  Source | lipoprotein lipase | 7311 | 0.014 | 0.3573 | No |
| 36 | APOC3 | APOC3 Entrez,  Source | apolipoprotein C-III | 7349 | 0.014 | 0.3574 | No |
| 37 | CYP27A1 | CYP27A1 Entrez,  Source | cytochrome P450, family 27, subfamily A, polypeptide 1 | 7755 | 0.009 | 0.3380 | No |
| 38 | FABP7 | FABP7 Entrez,  Source | fatty acid binding protein 7, brain | 7996 | 0.007 | 0.3268 | No |
| 39 | EHHADH | EHHADH Entrez,  Source | enoyl-Coenzyme A, hydratase/3-hydroxyacyl Coenzyme A dehydrogenase | 9058 | -0.002 | 0.2727 | No |
| 40 | SLC27A6 | SLC27A6 Entrez,  Source | solute carrier family 27 (fatty acid transporter), member 6 | 9069 | -0.002 | 0.2725 | No |
| 41 | RXRB | RXRB Entrez,  Source | retinoid X receptor, beta | 9260 | -0.004 | 0.2634 | No |
| 42 | FABP4 | FABP4 Entrez,  Source | fatty acid binding protein 4, adipocyte | 9566 | -0.007 | 0.2487 | No |
| 43 | APOA1 | APOA1 Entrez,  Source | apolipoprotein A-I | 10089 | -0.011 | 0.2236 | No |
| 44 | ACOX3 | ACOX3 Entrez,  Source | acyl-Coenzyme A oxidase 3, pristanoyl | 10752 | -0.017 | 0.1922 | No |
| 45 | ACADM | ACADM Entrez,  Source | acyl-Coenzyme A dehydrogenase, C-4 to C-12 straight chain | 11735 | -0.026 | 0.1456 | No |
| 46 | FABP2 | FABP2 Entrez,  Source | fatty acid binding protein 2, intestinal | 11894 | -0.027 | 0.1416 | No |
| 47 | CPT1C | CPT1C Entrez,  Source | carnitine palmitoyltransferase 1C | 11935 | -0.028 | 0.1436 | No |
| 48 | ACSL4 | ACSL4 Entrez,  Source | acyl-CoA synthetase long-chain family member 4 | 12606 | -0.034 | 0.1143 | No |
| 49 | SCD5 | SCD5 Entrez,  Source | stearoyl-CoA desaturase 5 | 13169 | -0.040 | 0.0914 | No |
| 50 | PCK2 | PCK2 Entrez,  Source | phosphoenolpyruvate carboxykinase 2 (mitochondrial) | 13451 | -0.043 | 0.0834 | No |
| 51 | CYP8B1 | CYP8B1 Entrez,  Source | cytochrome P450, family 8, subfamily B, polypeptide 1 | 13682 | -0.046 | 0.0783 | No |
| 52 | NR1H3 | NR1H3 Entrez,  Source | nuclear receptor subfamily 1, group H, member 3 | 13838 | -0.047 | 0.0774 | No |
| 53 | CD36 | CD36 Entrez,  Source | CD36 molecule (thrombospondin receptor) | 14387 | -0.054 | 0.0572 | No |
| 54 | PLTP | PLTP Entrez,  Source | phospholipid transfer protein | 14486 | -0.055 | 0.0604 | No |
| 55 | ACSL6 | ACSL6 Entrez,  Source | acyl-CoA synthetase long-chain family member 6 | 15066 | -0.064 | 0.0401 | No |
| 56 | FABP1 | FABP1 Entrez,  Source | fatty acid binding protein 1, liver | 15399 | -0.069 | 0.0333 | No |
| 57 | RXRG | RXRG Entrez,  Source | retinoid X receptor, gamma | 15447 | -0.069 | 0.0411 | No |
| 58 | ACOX2 | ACOX2 Entrez,  Source | acyl-Coenzyme A oxidase 2, branched chain | 15688 | -0.073 | 0.0397 | No |
| 59 | FADS2 | FADS2 Entrez,  Source | fatty acid desaturase 2 | 16315 | -0.085 | 0.0201 | No |
| 60 | ADIPOQ | ADIPOQ Entrez,  Source | adiponectin, C1Q and collagen domain containing | 16952 | -0.097 | 0.0019 | No |
| 61 | APOA2 | APOA2 Entrez,  Source | apolipoprotein A-II | 17556 | -0.113 | -0.0123 | No |
| 62 | CYP7A1 | CYP7A1 Entrez,  Source | cytochrome P450, family 7, subfamily A, polypeptide 1 | 17667 | -0.117 | -0.0007 | No |
| 63 | CYP4A11 | CYP4A11 Entrez,  Source | cytochrome P450, family 4, subfamily A, polypeptide 11 | 18016 | -0.128 | 0.0005 | No |
| 64 | SLC27A5 | SLC27A5 Entrez,  Source | solute carrier family 27 (fatty acid transporter), member 5 | 18132 | -0.133 | 0.0143 | No |
| 65 | FABP6 | FABP6 Entrez,  Source | fatty acid binding protein 6, ileal (gastrotropin) | 18519 | -0.152 | 0.0171 | No |
| 66 | SORBS1 | SORBS1 Entrez,  Source | sorbin and SH3 domain containing 1 | 19283 | -0.243 | 0.0140 | No |
Table: GSEA details [plain text format]

  

Fig 2: KEGG\_PPAR\_SIGNALING\_PATHWAY      
 Blue-Pink O' Gram in the Space of the Analyzed GeneSet

  

Fig 3: KEGG\_PPAR\_SIGNALING\_PATHWAY: Random ES distribution      
 Gene set null distribution of ES for **KEGG\_PPAR\_SIGNALING\_PATHWAY**
