# Supplementary material for: Comprehensive analysis of β-catenin target genes in colorectal carcinoma cell lines with deregulated Wnt/β-catenin signaling
Source: BMC Genomics. 2014 Jan 28;15:74. doi: 10.1186/1471-2164-15-74 (PMC3909937; doi:10.1186/1471-2164-15-74)
Supplement: Additional file 5 — GSEA analysis using the KEGG pathway database. This zipped file contains confirming data of the GSEA analysis. The names of the directories containing the files were composed of the term ‘GSEA’, the name of the cell line, e.g. DLD1, SW480, or LS174T, and the pathway database (KEGG). Please use a web browser to view the files with the name ‘index.html’ in the corresponding directories to start exploring the data. [file 1471-2164-15-74-S5.zip › GSEA_KEGG_DLD1/KEGG_PURINE_METABOLISM.html]

Details for gene set KEGG\_PURINE\_METABOLISM[GSEA]

|  || Dataset | DLD1\_collapsed\_to\_symbols.class.cls#b\_versus\_bg.class.cls#b\_versus\_bg\_repos |
| Phenotype | class.cls#b\_versus\_bg\_repos |
| Upregulated in class | 0 |
| GeneSet | KEGG\_PURINE\_METABOLISM |
| Enrichment Score (ES) | -0.32980207 |
| Normalized Enrichment Score (NES) | -1.3588507 |
| Nominal p-value | 0.0390625 |
| FDR q-value | 0.31688488 |
| FWER p-Value | 0.999 |
Table: GSEA Results Summary

  

Fig 1: Enrichment plot: KEGG\_PURINE\_METABOLISM      
 Profile of the Running ES Score & Positions of GeneSet Members on the Rank Ordered List

  

| PROBE | GENE SYMBOL | GENE\_TITLE | RANK IN GENE LIST | RANK METRIC SCORE | RUNNING ES | CORE ENRICHMENT || 1 | XDH | XDH Entrez,  Source | xanthine dehydrogenase | 36 | 0.642 | 0.0494 | No |
| 2 | PDE5A | PDE5A Entrez,  Source | phosphodiesterase 5A, cGMP-specific | 150 | 0.362 | 0.0725 | No |
| 3 | PDE9A | PDE9A Entrez,  Source | phosphodiesterase 9A | 376 | 0.237 | 0.0799 | No |
| 4 | POLD4 | POLD4 Entrez,  Source | polymerase (DNA-directed), delta 4 | 403 | 0.230 | 0.0970 | No |
| 5 | ADCY9 | ADCY9 Entrez,  Source | adenylate cyclase 9 | 546 | 0.198 | 0.1055 | No |
| 6 | NT5C2 | NT5C2 Entrez,  Source | 5'-nucleotidase, cytosolic II | 622 | 0.187 | 0.1165 | No |
| 7 | NT5E | NT5E Entrez,  Source | 5'-nucleotidase, ecto (CD73) | 741 | 0.171 | 0.1242 | No |
| 8 | GUCY2C | GUCY2C Entrez,  Source | guanylate cyclase 2C (heat stable enterotoxin receptor) | 895 | 0.157 | 0.1288 | No |
| 9 | ENTPD5 | ENTPD5 Entrez,  Source | ectonucleoside triphosphate diphosphohydrolase 5 | 912 | 0.156 | 0.1404 | No |
| 10 | ADA | ADA Entrez,  Source | adenosine deaminase | 994 | 0.150 | 0.1483 | No |
| 11 | GDA | GDA Entrez,  Source | guanine deaminase | 1320 | 0.127 | 0.1417 | No |
| 12 | ENTPD3 | ENTPD3 Entrez,  Source | ectonucleoside triphosphate diphosphohydrolase 3 | 1355 | 0.125 | 0.1500 | No |
| 13 | POLR3A | POLR3A Entrez,  Source | polymerase (RNA) III (DNA directed) polypeptide A, 155kDa | 1470 | 0.119 | 0.1536 | No |
| 14 | GUCY1A3 | GUCY1A3 Entrez,  Source | guanylate cyclase 1, soluble, alpha 3 | 1797 | 0.106 | 0.1453 | No |
| 15 | ENPP1 | ENPP1 Entrez,  Source | ectonucleotide pyrophosphatase/phosphodiesterase 1 | 1898 | 0.103 | 0.1483 | No |
| 16 | PDE6B | PDE6B Entrez,  Source | phosphodiesterase 6B, cGMP-specific, rod, beta (congenital stationary night blindness 3, autosomal dominant) | 2043 | 0.098 | 0.1487 | No |
| 17 | PDE4D | PDE4D Entrez,  Source | phosphodiesterase 4D, cAMP-specific (phosphodiesterase E3 dunce homolog, Drosophila) | 2306 | 0.090 | 0.1424 | No |
| 18 | PDE6G | PDE6G Entrez,  Source | phosphodiesterase 6G, cGMP-specific, rod, gamma | 3096 | 0.071 | 0.1075 | No |
| 19 | CANT1 | CANT1 Entrez,  Source | calcium activated nucleotidase 1 | 3156 | 0.070 | 0.1100 | No |
| 20 | ENTPD2 | ENTPD2 Entrez,  Source | ectonucleoside triphosphate diphosphohydrolase 2 | 3188 | 0.069 | 0.1140 | No |
| 21 | PDE10A | PDE10A Entrez,  Source | phosphodiesterase 10A | 3268 | 0.067 | 0.1153 | No |
| 22 | ADCY6 | ADCY6 Entrez,  Source | adenylate cyclase 6 | 3411 | 0.065 | 0.1132 | No |
| 23 | NT5C | NT5C Entrez,  Source | 5', 3'-nucleotidase, cytosolic | 3820 | 0.057 | 0.0967 | No |
| 24 | NME6 | NME6 Entrez,  Source | non-metastatic cells 6, protein expressed in (nucleoside-diphosphate kinase) | 4114 | 0.053 | 0.0858 | No |
| 25 | ENTPD6 | ENTPD6 Entrez,  Source | ectonucleoside triphosphate diphosphohydrolase 6 (putative function) | 4202 | 0.051 | 0.0854 | No |
| 26 | PDE3B | PDE3B Entrez,  Source | phosphodiesterase 3B, cGMP-inhibited | 4255 | 0.051 | 0.0868 | No |
| 27 | NUDT9 | NUDT9 Entrez,  Source | nudix (nucleoside diphosphate linked moiety X)-type motif 9 | 4608 | 0.045 | 0.0723 | No |
| 28 | ADCY2 | ADCY2 Entrez,  Source | adenylate cyclase 2 (brain) | 4632 | 0.045 | 0.0747 | No |
| 29 | PDE6H | PDE6H Entrez,  Source | phosphodiesterase 6H, cGMP-specific, cone, gamma | 4777 | 0.043 | 0.0707 | No |
| 30 | AK5 | AK5 Entrez,  Source | adenylate kinase 5 | 4836 | 0.042 | 0.0711 | No |
| 31 | PAPSS1 | PAPSS1 Entrez,  Source | 3'-phosphoadenosine 5'-phosphosulfate synthase 1 | 4873 | 0.042 | 0.0726 | No |
| 32 | GUCY1B3 | GUCY1B3 Entrez,  Source | guanylate cyclase 1, soluble, beta 3 | 5110 | 0.038 | 0.0635 | No |
| 33 | NME3 | NME3 Entrez,  Source | non-metastatic cells 3, protein expressed in | 5204 | 0.037 | 0.0617 | No |
| 34 | NPR1 | NPR1 Entrez,  Source | natriuretic peptide receptor A/guanylate cyclase A (atrionatriuretic peptide receptor A) | 5435 | 0.034 | 0.0526 | No |
| 35 | PDE6C | PDE6C Entrez,  Source | phosphodiesterase 6C, cGMP-specific, cone, alpha prime | 5444 | 0.034 | 0.0549 | No |
| 36 | NME5 | NME5 Entrez,  Source | non-metastatic cells 5, protein expressed in (nucleoside-diphosphate kinase) | 5689 | 0.031 | 0.0448 | No |
| 37 | GUCY2D | GUCY2D Entrez,  Source | guanylate cyclase 2D, membrane (retina-specific) | 5709 | 0.031 | 0.0463 | No |
| 38 | PDE11A | PDE11A Entrez,  Source | phosphodiesterase 11A | 5763 | 0.030 | 0.0460 | No |
| 39 | NME7 | NME7 Entrez,  Source | non-metastatic cells 7, protein expressed in (nucleoside-diphosphate kinase) | 5876 | 0.029 | 0.0426 | No |
| 40 | ENPP3 | ENPP3 Entrez,  Source | ectonucleotide pyrophosphatase/phosphodiesterase 3 | 5898 | 0.029 | 0.0438 | No |
| 41 | PDE4A | PDE4A Entrez,  Source | phosphodiesterase 4A, cAMP-specific (phosphodiesterase E2 dunce homolog, Drosophila) | 6150 | 0.026 | 0.0329 | No |
| 42 | POLE4 | POLE4 Entrez,  Source | polymerase (DNA-directed), epsilon 4 (p12 subunit) | 6397 | 0.024 | 0.0221 | No |
| 43 | GUCY2F | GUCY2F Entrez,  Source | guanylate cyclase 2F, retinal | 6464 | 0.023 | 0.0206 | No |
| 44 | AK7 | AK7 Entrez,  Source | adenylate kinase 7 | 6888 | 0.018 | 0.0002 | No |
| 45 | IMPDH1 | IMPDH1 Entrez,  Source | IMP (inosine monophosphate) dehydrogenase 1 | 7082 | 0.016 | -0.0084 | No |
| 46 | RRM2B | RRM2B Entrez,  Source | ribonucleotide reductase M2 B (TP53 inducible) | 7149 | 0.016 | -0.0105 | No |
| 47 | ADSSL1 | ADSSL1 Entrez,  Source | adenylosuccinate synthase like 1 | 7234 | 0.015 | -0.0137 | No |
| 48 | POLR2L | POLR2L Entrez,  Source | polymerase (RNA) II (DNA directed) polypeptide L, 7.6kDa | 7269 | 0.015 | -0.0143 | No |
| 49 | AK1 | AK1 Entrez,  Source | adenylate kinase 1 | 7847 | 0.009 | -0.0433 | No |
| 50 | ENTPD4 | ENTPD4 Entrez,  Source | ectonucleoside triphosphate diphosphohydrolase 4 | 7869 | 0.008 | -0.0437 | No |
| 51 | PDE4C | PDE4C Entrez,  Source | phosphodiesterase 4C, cAMP-specific (phosphodiesterase E1 dunce homolog, Drosophila) | 8136 | 0.006 | -0.0569 | No |
| 52 | NT5C3 | NT5C3 Entrez,  Source | 5'-nucleotidase, cytosolic III | 8240 | 0.005 | -0.0618 | No |
| 53 | PDE6D | PDE6D Entrez,  Source | phosphodiesterase 6D, cGMP-specific, rod, delta | 8277 | 0.005 | -0.0633 | No |
| 54 | ADCY7 | ADCY7 Entrez,  Source | adenylate cyclase 7 | 8431 | 0.003 | -0.0710 | No |
| 55 | GUK1 | GUK1 Entrez,  Source | guanylate kinase 1 | 8496 | 0.003 | -0.0741 | No |
| 56 | NUDT2 | NUDT2 Entrez,  Source | nudix (nucleoside diphosphate linked moiety X)-type motif 2 | 8923 | -0.001 | -0.0959 | No |
| 57 | GMPR2 | GMPR2 Entrez,  Source | guanosine monophosphate reductase 2 | 9041 | -0.002 | -0.1018 | No |
| 58 | DCK | DCK Entrez,  Source | deoxycytidine kinase | 9336 | -0.005 | -0.1165 | No |
| 59 | NPR2 | NPR2 Entrez,  Source | natriuretic peptide receptor B/guanylate cyclase B (atrionatriuretic peptide receptor B) | 9454 | -0.006 | -0.1221 | No |
| 60 | POLR2J | POLR2J Entrez,  Source | polymerase (RNA) II (DNA directed) polypeptide J, 13.3kDa | 9814 | -0.009 | -0.1399 | No |
| 61 | ADCY1 | ADCY1 Entrez,  Source | adenylate cyclase 1 (brain) | 9937 | -0.010 | -0.1454 | No |
| 62 | PRUNE | PRUNE Entrez,  Source | prune homolog (Drosophila) | 9948 | -0.010 | -0.1451 | No |
| 63 | PRPS1L1 | PRPS1L1 Entrez,  Source | phosphoribosyl pyrophosphate synthetase 1-like 1 | 9990 | -0.010 | -0.1464 | No |
| 64 | ADCY8 | ADCY8 Entrez,  Source | adenylate cyclase 8 (brain) | 10229 | -0.013 | -0.1576 | No |
| 65 | ADSS | ADSS Entrez,  Source | adenylosuccinate synthase | 10260 | -0.013 | -0.1581 | No |
| 66 | PDE1B | PDE1B Entrez,  Source | phosphodiesterase 1B, calmodulin-dependent | 10324 | -0.013 | -0.1603 | No |
| 67 | AMPD1 | AMPD1 Entrez,  Source | adenosine monophosphate deaminase 1 (isoform M) | 10493 | -0.015 | -0.1678 | No |
| 68 | POLR2C | POLR2C Entrez,  Source | polymerase (RNA) II (DNA directed) polypeptide C, 33kDa | 10551 | -0.015 | -0.1695 | No |
| 69 | ENTPD1 | ENTPD1 Entrez,  Source | ectonucleoside triphosphate diphosphohydrolase 1 | 10705 | -0.017 | -0.1760 | No |
| 70 | POLR2K | POLR2K Entrez,  Source | polymerase (RNA) II (DNA directed) polypeptide K, 7.0kDa | 10758 | -0.017 | -0.1773 | No |
| 71 | PDE7B | PDE7B Entrez,  Source | phosphodiesterase 7B | 11286 | -0.022 | -0.2027 | No |
| 72 | PKM2 | PKM2 Entrez,  Source | pyruvate kinase, muscle | 11291 | -0.022 | -0.2012 | No |
| 73 | POLR3C | POLR3C Entrez,  Source | polymerase (RNA) III (DNA directed) polypeptide C (62kD) | 11371 | -0.022 | -0.2035 | No |
| 74 | PDE8A | PDE8A Entrez,  Source | phosphodiesterase 8A | 11570 | -0.024 | -0.2118 | No |
| 75 | RRM2 | RRM2 Entrez,  Source | ribonucleotide reductase M2 polypeptide | 11631 | -0.025 | -0.2129 | No |
| 76 | ADK | ADK Entrez,  Source | adenosine kinase | 11673 | -0.025 | -0.2130 | No |
| 77 | PDE4B | PDE4B Entrez,  Source | phosphodiesterase 4B, cAMP-specific (phosphodiesterase E4 dunce homolog, Drosophila) | 12264 | -0.031 | -0.2409 | No |
| 78 | PKLR | PKLR Entrez,  Source | pyruvate kinase, liver and RBC | 12312 | -0.031 | -0.2408 | No |
| 79 | POLR2A | POLR2A Entrez,  Source | polymerase (RNA) II (DNA directed) polypeptide A, 220kDa | 12338 | -0.031 | -0.2396 | No |
| 80 | ALLC | ALLC Entrez,  Source | allantoicase | 12363 | -0.032 | -0.2383 | No |
| 81 | PDE1C | PDE1C Entrez,  Source | phosphodiesterase 1C, calmodulin-dependent 70kDa | 12512 | -0.033 | -0.2433 | No |
| 82 | ADCY4 | ADCY4 Entrez,  Source | adenylate cyclase 4 | 12566 | -0.034 | -0.2434 | No |
| 83 | PDE1A | PDE1A Entrez,  Source | phosphodiesterase 1A, calmodulin-dependent | 12797 | -0.036 | -0.2523 | No |
| 84 | POLR3D | POLR3D Entrez,  Source | polymerase (RNA) III (DNA directed) polypeptide D, 44kDa | 12828 | -0.036 | -0.2510 | No |
| 85 | POLR3GL | POLR3GL Entrez,  Source | polymerase (RNA) III (DNA directed) polypeptide G (32kD) like | 12917 | -0.037 | -0.2525 | No |
| 86 | POLR2E | POLR2E Entrez,  Source | polymerase (RNA) II (DNA directed) polypeptide E, 25kDa | 13299 | -0.041 | -0.2688 | No |
| 87 | NT5C1A | NT5C1A Entrez,  Source | 5'-nucleotidase, cytosolic IA | 13317 | -0.042 | -0.2664 | No |
| 88 | DGUOK | DGUOK Entrez,  Source | deoxyguanosine kinase | 13495 | -0.044 | -0.2720 | No |
| 89 | POLR2J2 | POLR2J2 Entrez,  Source | - | 13799 | -0.047 | -0.2839 | No |
| 90 | PDE7A | PDE7A Entrez,  Source | phosphodiesterase 7A | 13812 | -0.047 | -0.2807 | No |
| 91 | PDE6A | PDE6A Entrez,  Source | phosphodiesterase 6A, cGMP-specific, rod, alpha | 13862 | -0.048 | -0.2795 | No |
| 92 | ITPA | ITPA Entrez,  Source | inosine triphosphatase (nucleoside triphosphate pyrophosphatase) | 14096 | -0.050 | -0.2874 | No |
| 93 | POLR2B | POLR2B Entrez,  Source | polymerase (RNA) II (DNA directed) polypeptide B, 140kDa | 14233 | -0.052 | -0.2903 | No |
| 94 | PRPS2 | PRPS2 Entrez,  Source | phosphoribosyl pyrophosphate synthetase 2 | 14285 | -0.052 | -0.2887 | No |
| 95 | ADCY5 | ADCY5 Entrez,  Source | adenylate cyclase 5 | 14374 | -0.054 | -0.2890 | No |
| 96 | RRM1 | RRM1 Entrez,  Source | ribonucleotide reductase M1 polypeptide | 14488 | -0.055 | -0.2904 | No |
| 97 | POLR3F | POLR3F Entrez,  Source | polymerase (RNA) III (DNA directed) polypeptide F, 39 kDa | 14726 | -0.059 | -0.2979 | No |
| 98 | ATIC | ATIC Entrez,  Source | 5-aminoimidazole-4-carboxamide ribonucleotide formyltransferase/IMP cyclohydrolase | 14735 | -0.059 | -0.2936 | No |
| 99 | PAPSS2 | PAPSS2 Entrez,  Source | 3'-phosphoadenosine 5'-phosphosulfate synthase 2 | 14951 | -0.062 | -0.2997 | No |
| 100 | NUDT5 | NUDT5 Entrez,  Source | nudix (nucleoside diphosphate linked moiety X)-type motif 5 | 15281 | -0.067 | -0.3113 | No |
| 101 | GUCY1A2 | GUCY1A2 Entrez,  Source | guanylate cyclase 1, soluble, alpha 2 | 15436 | -0.069 | -0.3137 | No |
| 102 | NME4 | NME4 Entrez,  Source | non-metastatic cells 4, protein expressed in | 15534 | -0.071 | -0.3131 | No |
| 103 | POLR2G | POLR2G Entrez,  Source | polymerase (RNA) II (DNA directed) polypeptide G | 15743 | -0.074 | -0.3178 | No |
| 104 | HPRT1 | HPRT1 Entrez,  Source | hypoxanthine phosphoribosyltransferase 1 (Lesch-Nyhan syndrome) | 15976 | -0.078 | -0.3235 | Yes |
| 105 | AMPD3 | AMPD3 Entrez,  Source | adenosine monophosphate deaminase (isoform E) | 16052 | -0.080 | -0.3210 | Yes |
| 106 | POLR2H | POLR2H Entrez,  Source | polymerase (RNA) II (DNA directed) polypeptide H | 16209 | -0.082 | -0.3225 | Yes |
| 107 | GMPR | GMPR Entrez,  Source | guanosine monophosphate reductase | 16249 | -0.083 | -0.3178 | Yes |
| 108 | AK2 | AK2 Entrez,  Source | adenylate kinase 2 | 16358 | -0.086 | -0.3166 | Yes |
| 109 | POLE | POLE Entrez,  Source | polymerase (DNA directed), epsilon | 16414 | -0.086 | -0.3125 | Yes |
| 110 | ZNRD1 | ZNRD1 Entrez,  Source | zinc ribbon domain containing 1 | 16449 | -0.087 | -0.3073 | Yes |
| 111 | POLR2F | POLR2F Entrez,  Source | polymerase (RNA) II (DNA directed) polypeptide F | 16514 | -0.088 | -0.3035 | Yes |
| 112 | GMPS | GMPS Entrez,  Source | guanine monphosphate synthetase | 16544 | -0.089 | -0.2979 | Yes |
| 113 | POLR2I | POLR2I Entrez,  Source | polymerase (RNA) II (DNA directed) polypeptide I, 14.5kDa | 16579 | -0.090 | -0.2925 | Yes |
| 114 | POLR2D | POLR2D Entrez,  Source | polymerase (RNA) II (DNA directed) polypeptide D | 16673 | -0.092 | -0.2899 | Yes |
| 115 | PNPT1 | PNPT1 Entrez,  Source | polyribonucleotide nucleotidyltransferase 1 | 16702 | -0.093 | -0.2840 | Yes |
| 116 | POLR3B | POLR3B Entrez,  Source | polymerase (RNA) III (DNA directed) polypeptide B | 16928 | -0.097 | -0.2878 | Yes |
| 117 | FHIT | FHIT Entrez,  Source | fragile histidine triad gene | 16937 | -0.097 | -0.2805 | Yes |
| 118 | APRT | APRT Entrez,  Source | adenine phosphoribosyltransferase | 17081 | -0.100 | -0.2799 | Yes |
| 119 | POLR3H | POLR3H Entrez,  Source | polymerase (RNA) III (DNA directed) polypeptide H (22.9kD) | 17084 | -0.100 | -0.2720 | Yes |
| 120 | IMPDH2 | IMPDH2 Entrez,  Source | IMP (inosine monophosphate) dehydrogenase 2 | 17252 | -0.105 | -0.2722 | Yes |
| 121 | AMPD2 | AMPD2 Entrez,  Source | adenosine monophosphate deaminase 2 (isoform L) | 17311 | -0.106 | -0.2667 | Yes |
| 122 | POLR1D | POLR1D Entrez,  Source | polymerase (RNA) I polypeptide D, 16kDa | 17721 | -0.119 | -0.2783 | Yes |
| 123 | NT5C1B | NT5C1B Entrez,  Source | 5'-nucleotidase, cytosolic IB | 17773 | -0.120 | -0.2713 | Yes |
| 124 | NT5M | NT5M Entrez,  Source | 5',3'-nucleotidase, mitochondrial | 17956 | -0.126 | -0.2706 | Yes |
| 125 | POLE3 | POLE3 Entrez,  Source | polymerase (DNA directed), epsilon 3 (p17 subunit) | 18007 | -0.128 | -0.2630 | Yes |
| 126 | ADCY3 | ADCY3 Entrez,  Source | adenylate cyclase 3 | 18065 | -0.131 | -0.2555 | Yes |
| 127 | ADSL | ADSL Entrez,  Source | adenylosuccinate lyase | 18068 | -0.131 | -0.2451 | Yes |
| 128 | POLA2 | POLA2 Entrez,  Source | polymerase (DNA directed), alpha 2 (70kD subunit) | 18235 | -0.138 | -0.2426 | Yes |
| 129 | POLD2 | POLD2 Entrez,  Source | polymerase (DNA directed), delta 2, regulatory subunit 50kDa | 18279 | -0.140 | -0.2337 | Yes |
| 130 | PDE2A | PDE2A Entrez,  Source | phosphodiesterase 2A, cGMP-stimulated | 18340 | -0.143 | -0.2253 | Yes |
| 131 | POLR1C | POLR1C Entrez,  Source | polymerase (RNA) I polypeptide C, 30kDa | 18440 | -0.147 | -0.2187 | Yes |
| 132 | PRPS1 | PRPS1 Entrez,  Source | phosphoribosyl pyrophosphate synthetase 1 | 18536 | -0.154 | -0.2113 | Yes |
| 133 | NME1 | NME1 Entrez,  Source | non-metastatic cells 1, protein (NM23A) expressed in | 18578 | -0.156 | -0.2009 | Yes |
| 134 | PAICS | PAICS Entrez,  Source | phosphoribosylaminoimidazole carboxylase, phosphoribosylaminoimidazole succinocarboxamide synthetase | 18581 | -0.156 | -0.1885 | Yes |
| 135 | PDE3A | PDE3A Entrez,  Source | phosphodiesterase 3A, cGMP-inhibited | 18583 | -0.157 | -0.1761 | Yes |
| 136 | GART | GART Entrez,  Source | phosphoribosylglycinamide formyltransferase, phosphoribosylglycinamide synthetase, phosphoribosylaminoimidazole synthetase | 18647 | -0.161 | -0.1664 | Yes |
| 137 | POLR1A | POLR1A Entrez,  Source | polymerase (RNA) I polypeptide A, 194kDa | 18719 | -0.166 | -0.1568 | Yes |
| 138 | POLD3 | POLD3 Entrez,  Source | polymerase (DNA-directed), delta 3, accessory subunit | 18829 | -0.177 | -0.1483 | Yes |
| 139 | PRIM1 | PRIM1 Entrez,  Source | primase, polypeptide 1, 49kDa | 18843 | -0.179 | -0.1346 | Yes |
| 140 | POLA1 | POLA1 Entrez,  Source | polymerase (DNA directed), alpha 1 | 18962 | -0.191 | -0.1255 | Yes |
| 141 | POLR1E | POLR1E Entrez,  Source | polymerase (RNA) I polypeptide E, 53kDa | 19019 | -0.196 | -0.1126 | Yes |
| 142 | POLD1 | POLD1 Entrez,  Source | polymerase (DNA directed), delta 1, catalytic subunit 125kDa | 19075 | -0.205 | -0.0991 | Yes |
| 143 | POLE2 | POLE2 Entrez,  Source | polymerase (DNA directed), epsilon 2 (p59 subunit) | 19106 | -0.210 | -0.0839 | Yes |
| 144 | POLR1B | POLR1B Entrez,  Source | polymerase (RNA) I polypeptide B, 128kDa | 19138 | -0.214 | -0.0684 | Yes |
| 145 | POLR3K | POLR3K Entrez,  Source | polymerase (RNA) III (DNA directed) polypeptide K, 12.3 kDa | 19162 | -0.218 | -0.0522 | Yes |
| 146 | PFAS | PFAS Entrez,  Source | phosphoribosylformylglycinamidine synthase (FGAR amidotransferase) | 19163 | -0.218 | -0.0347 | Yes |
| 147 | PDE8B | PDE8B Entrez,  Source | phosphodiesterase 8B | 19177 | -0.220 | -0.0178 | Yes |
| 148 | POLR3G | POLR3G Entrez,  Source | polymerase (RNA) III (DNA directed) polypeptide G (32kD) | 19204 | -0.224 | -0.0013 | Yes |
| 149 | PPAT | PPAT Entrez,  Source | phosphoribosyl pyrophosphate amidotransferase | 19279 | -0.242 | 0.0143 | Yes |
Table: GSEA details [plain text format]

  

Fig 2: KEGG\_PURINE\_METABOLISM      
 Blue-Pink O' Gram in the Space of the Analyzed GeneSet

  

Fig 3: KEGG\_PURINE\_METABOLISM: Random ES distribution      
 Gene set null distribution of ES for **KEGG\_PURINE\_METABOLISM**
