# Supplementary material for: Comprehensive analysis of β-catenin target genes in colorectal carcinoma cell lines with deregulated Wnt/β-catenin signaling
Source: BMC Genomics. 2014 Jan 28;15:74. doi: 10.1186/1471-2164-15-74 (PMC3909937; doi:10.1186/1471-2164-15-74)
Supplement: Additional file 5 — GSEA analysis using the KEGG pathway database. This zipped file contains confirming data of the GSEA analysis. The names of the directories containing the files were composed of the term ‘GSEA’, the name of the cell line, e.g. DLD1, SW480, or LS174T, and the pathway database (KEGG). Please use a web browser to view the files with the name ‘index.html’ in the corresponding directories to start exploring the data. [file 1471-2164-15-74-S5.zip › GSEA_KEGG_DLD1/KEGG_PYRIMIDINE_METABOLISM.html]

Details for gene set KEGG\_PYRIMIDINE\_METABOLISM[GSEA]

|  || Dataset | DLD1\_collapsed\_to\_symbols.class.cls#b\_versus\_bg.class.cls#b\_versus\_bg\_repos |
| Phenotype | class.cls#b\_versus\_bg\_repos |
| Upregulated in class | 0 |
| GeneSet | KEGG\_PYRIMIDINE\_METABOLISM |
| Enrichment Score (ES) | -0.44129643 |
| Normalized Enrichment Score (NES) | -1.6771638 |
| Nominal p-value | 0.0 |
| FDR q-value | 0.03952511 |
| FWER p-Value | 0.344 |
Table: GSEA Results Summary

  

Fig 1: Enrichment plot: KEGG\_PYRIMIDINE\_METABOLISM      
 Profile of the Running ES Score & Positions of GeneSet Members on the Rank Ordered List

  

| PROBE | GENE SYMBOL | GENE\_TITLE | RANK IN GENE LIST | RANK METRIC SCORE | RUNNING ES | CORE ENRICHMENT || 1 | CDA | CDA Entrez,  Source | cytidine deaminase | 312 | 0.265 | 0.0172 | No |
| 2 | POLD4 | POLD4 Entrez,  Source | polymerase (DNA-directed), delta 4 | 403 | 0.230 | 0.0414 | No |
| 3 | NT5C2 | NT5C2 Entrez,  Source | 5'-nucleotidase, cytosolic II | 622 | 0.187 | 0.0535 | No |
| 4 | TXNRD1 | TXNRD1 Entrez,  Source | thioredoxin reductase 1 | 694 | 0.177 | 0.0720 | No |
| 5 | NT5E | NT5E Entrez,  Source | 5'-nucleotidase, ecto (CD73) | 741 | 0.171 | 0.0911 | No |
| 6 | ENTPD5 | ENTPD5 Entrez,  Source | ectonucleoside triphosphate diphosphohydrolase 5 | 912 | 0.156 | 0.1018 | No |
| 7 | DPYS | DPYS Entrez,  Source | dihydropyrimidinase | 1212 | 0.134 | 0.1032 | No |
| 8 | ENTPD3 | ENTPD3 Entrez,  Source | ectonucleoside triphosphate diphosphohydrolase 3 | 1355 | 0.125 | 0.1116 | No |
| 9 | POLR3A | POLR3A Entrez,  Source | polymerase (RNA) III (DNA directed) polypeptide A, 155kDa | 1470 | 0.119 | 0.1206 | No |
| 10 | UPP1 | UPP1 Entrez,  Source | uridine phosphorylase 1 | 2123 | 0.095 | 0.0991 | No |
| 11 | CANT1 | CANT1 Entrez,  Source | calcium activated nucleotidase 1 | 3156 | 0.070 | 0.0548 | No |
| 12 | NT5C | NT5C Entrez,  Source | 5', 3'-nucleotidase, cytosolic | 3820 | 0.057 | 0.0279 | No |
| 13 | NME6 | NME6 Entrez,  Source | non-metastatic cells 6, protein expressed in (nucleoside-diphosphate kinase) | 4114 | 0.053 | 0.0194 | No |
| 14 | ENTPD6 | ENTPD6 Entrez,  Source | ectonucleoside triphosphate diphosphohydrolase 6 (putative function) | 4202 | 0.051 | 0.0214 | No |
| 15 | NME3 | NME3 Entrez,  Source | non-metastatic cells 3, protein expressed in | 5204 | 0.037 | -0.0254 | No |
| 16 | NME5 | NME5 Entrez,  Source | non-metastatic cells 5, protein expressed in (nucleoside-diphosphate kinase) | 5689 | 0.031 | -0.0463 | No |
| 17 | NME7 | NME7 Entrez,  Source | non-metastatic cells 7, protein expressed in (nucleoside-diphosphate kinase) | 5876 | 0.029 | -0.0523 | No |
| 18 | TK2 | TK2 Entrez,  Source | thymidine kinase 2, mitochondrial | 6065 | 0.027 | -0.0585 | No |
| 19 | DPYD | DPYD Entrez,  Source | dihydropyrimidine dehydrogenase | 6230 | 0.025 | -0.0638 | No |
| 20 | POLE4 | POLE4 Entrez,  Source | polymerase (DNA-directed), epsilon 4 (p12 subunit) | 6397 | 0.024 | -0.0694 | No |
| 21 | UPP2 | UPP2 Entrez,  Source | uridine phosphorylase 2 | 6561 | 0.022 | -0.0750 | No |
| 22 | UPB1 | UPB1 Entrez,  Source | ureidopropionase, beta | 6602 | 0.022 | -0.0743 | No |
| 23 | RRM2B | RRM2B Entrez,  Source | ribonucleotide reductase M2 B (TP53 inducible) | 7149 | 0.016 | -0.1004 | No |
| 24 | POLR2L | POLR2L Entrez,  Source | polymerase (RNA) II (DNA directed) polypeptide L, 7.6kDa | 7269 | 0.015 | -0.1047 | No |
| 25 | AK3 | AK3 Entrez,  Source | adenylate kinase 3 | 7479 | 0.012 | -0.1139 | No |
| 26 | TXNRD2 | TXNRD2 Entrez,  Source | thioredoxin reductase 2 | 7625 | 0.011 | -0.1200 | No |
| 27 | ENTPD4 | ENTPD4 Entrez,  Source | ectonucleoside triphosphate diphosphohydrolase 4 | 7869 | 0.008 | -0.1314 | No |
| 28 | NT5C3 | NT5C3 Entrez,  Source | 5'-nucleotidase, cytosolic III | 8240 | 0.005 | -0.1498 | No |
| 29 | DTYMK | DTYMK Entrez,  Source | deoxythymidylate kinase (thymidylate kinase) | 8397 | 0.003 | -0.1574 | No |
| 30 | NUDT2 | NUDT2 Entrez,  Source | nudix (nucleoside diphosphate linked moiety X)-type motif 2 | 8923 | -0.001 | -0.1842 | No |
| 31 | DCK | DCK Entrez,  Source | deoxycytidine kinase | 9336 | -0.005 | -0.2048 | No |
| 32 | POLR2J | POLR2J Entrez,  Source | polymerase (RNA) II (DNA directed) polypeptide J, 13.3kDa | 9814 | -0.009 | -0.2281 | No |
| 33 | UCK1 | UCK1 Entrez,  Source | uridine-cytidine kinase 1 | 10326 | -0.013 | -0.2527 | No |
| 34 | POLR2C | POLR2C Entrez,  Source | polymerase (RNA) II (DNA directed) polypeptide C, 33kDa | 10551 | -0.015 | -0.2623 | No |
| 35 | ENTPD1 | ENTPD1 Entrez,  Source | ectonucleoside triphosphate diphosphohydrolase 1 | 10705 | -0.017 | -0.2680 | No |
| 36 | POLR2K | POLR2K Entrez,  Source | polymerase (RNA) II (DNA directed) polypeptide K, 7.0kDa | 10758 | -0.017 | -0.2686 | No |
| 37 | DCTD | DCTD Entrez,  Source | dCMP deaminase | 11222 | -0.021 | -0.2897 | No |
| 38 | UCKL1 | UCKL1 Entrez,  Source | uridine-cytidine kinase 1-like 1 | 11280 | -0.022 | -0.2899 | No |
| 39 | POLR3C | POLR3C Entrez,  Source | polymerase (RNA) III (DNA directed) polypeptide C (62kD) | 11371 | -0.022 | -0.2918 | No |
| 40 | CTPS2 | CTPS2 Entrez,  Source | CTP synthase II | 11441 | -0.023 | -0.2924 | No |
| 41 | RRM2 | RRM2 Entrez,  Source | ribonucleotide reductase M2 polypeptide | 11631 | -0.025 | -0.2990 | No |
| 42 | POLR2A | POLR2A Entrez,  Source | polymerase (RNA) II (DNA directed) polypeptide A, 220kDa | 12338 | -0.031 | -0.3314 | No |
| 43 | POLR3D | POLR3D Entrez,  Source | polymerase (RNA) III (DNA directed) polypeptide D, 44kDa | 12828 | -0.036 | -0.3519 | No |
| 44 | POLR3GL | POLR3GL Entrez,  Source | polymerase (RNA) III (DNA directed) polypeptide G (32kD) like | 12917 | -0.037 | -0.3518 | No |
| 45 | POLR2E | POLR2E Entrez,  Source | polymerase (RNA) II (DNA directed) polypeptide E, 25kDa | 13299 | -0.041 | -0.3661 | No |
| 46 | NT5C1A | NT5C1A Entrez,  Source | 5'-nucleotidase, cytosolic IA | 13317 | -0.042 | -0.3618 | No |
| 47 | POLR2J2 | POLR2J2 Entrez,  Source | - | 13799 | -0.047 | -0.3807 | No |
| 48 | ITPA | ITPA Entrez,  Source | inosine triphosphatase (nucleoside triphosphate pyrophosphatase) | 14096 | -0.050 | -0.3895 | No |
| 49 | POLR2B | POLR2B Entrez,  Source | polymerase (RNA) II (DNA directed) polypeptide B, 140kDa | 14233 | -0.052 | -0.3900 | No |
| 50 | RRM1 | RRM1 Entrez,  Source | ribonucleotide reductase M1 polypeptide | 14488 | -0.055 | -0.3962 | No |
| 51 | POLR3F | POLR3F Entrez,  Source | polymerase (RNA) III (DNA directed) polypeptide F, 39 kDa | 14726 | -0.059 | -0.4010 | No |
| 52 | TYMS | TYMS Entrez,  Source | thymidylate synthetase | 14974 | -0.062 | -0.4059 | No |
| 53 | NME4 | NME4 Entrez,  Source | non-metastatic cells 4, protein expressed in | 15534 | -0.071 | -0.4257 | No |
| 54 | POLR2G | POLR2G Entrez,  Source | polymerase (RNA) II (DNA directed) polypeptide G | 15743 | -0.074 | -0.4271 | No |
| 55 | DUT | DUT Entrez,  Source | dUTP pyrophosphatase | 15986 | -0.079 | -0.4297 | No |
| 56 | POLR2H | POLR2H Entrez,  Source | polymerase (RNA) II (DNA directed) polypeptide H | 16209 | -0.082 | -0.4308 | Yes |
| 57 | POLE | POLE Entrez,  Source | polymerase (DNA directed), epsilon | 16414 | -0.086 | -0.4305 | Yes |
| 58 | ZNRD1 | ZNRD1 Entrez,  Source | zinc ribbon domain containing 1 | 16449 | -0.087 | -0.4213 | Yes |
| 59 | POLR2F | POLR2F Entrez,  Source | polymerase (RNA) II (DNA directed) polypeptide F | 16514 | -0.088 | -0.4136 | Yes |
| 60 | POLR2I | POLR2I Entrez,  Source | polymerase (RNA) II (DNA directed) polypeptide I, 14.5kDa | 16579 | -0.090 | -0.4056 | Yes |
| 61 | POLR2D | POLR2D Entrez,  Source | polymerase (RNA) II (DNA directed) polypeptide D | 16673 | -0.092 | -0.3989 | Yes |
| 62 | PNPT1 | PNPT1 Entrez,  Source | polyribonucleotide nucleotidyltransferase 1 | 16702 | -0.093 | -0.3888 | Yes |
| 63 | POLR3B | POLR3B Entrez,  Source | polymerase (RNA) III (DNA directed) polypeptide B | 16928 | -0.097 | -0.3882 | Yes |
| 64 | POLR3H | POLR3H Entrez,  Source | polymerase (RNA) III (DNA directed) polypeptide H (22.9kD) | 17084 | -0.100 | -0.3836 | Yes |
| 65 | TK1 | TK1 Entrez,  Source | thymidine kinase 1, soluble | 17237 | -0.104 | -0.3784 | Yes |
| 66 | UMPS | UMPS Entrez,  Source | uridine monophosphate synthetase (orotate phosphoribosyl transferase and orotidine-5'-decarboxylase) | 17538 | -0.112 | -0.3798 | Yes |
| 67 | POLR1D | POLR1D Entrez,  Source | polymerase (RNA) I polypeptide D, 16kDa | 17721 | -0.119 | -0.3743 | Yes |
| 68 | NT5C1B | NT5C1B Entrez,  Source | 5'-nucleotidase, cytosolic IB | 17773 | -0.120 | -0.3619 | Yes |
| 69 | NT5M | NT5M Entrez,  Source | 5',3'-nucleotidase, mitochondrial | 17956 | -0.126 | -0.3554 | Yes |
| 70 | POLE3 | POLE3 Entrez,  Source | polymerase (DNA directed), epsilon 3 (p17 subunit) | 18007 | -0.128 | -0.3420 | Yes |
| 71 | POLA2 | POLA2 Entrez,  Source | polymerase (DNA directed), alpha 2 (70kD subunit) | 18235 | -0.138 | -0.3364 | Yes |
| 72 | POLD2 | POLD2 Entrez,  Source | polymerase (DNA directed), delta 2, regulatory subunit 50kDa | 18279 | -0.140 | -0.3211 | Yes |
| 73 | POLR1C | POLR1C Entrez,  Source | polymerase (RNA) I polypeptide C, 30kDa | 18440 | -0.147 | -0.3109 | Yes |
| 74 | NME1 | NME1 Entrez,  Source | non-metastatic cells 1, protein (NM23A) expressed in | 18578 | -0.156 | -0.2984 | Yes |
| 75 | POLR1A | POLR1A Entrez,  Source | polymerase (RNA) I polypeptide A, 194kDa | 18719 | -0.166 | -0.2848 | Yes |
| 76 | POLD3 | POLD3 Entrez,  Source | polymerase (DNA-directed), delta 3, accessory subunit | 18829 | -0.177 | -0.2682 | Yes |
| 77 | PRIM1 | PRIM1 Entrez,  Source | primase, polypeptide 1, 49kDa | 18843 | -0.179 | -0.2465 | Yes |
| 78 | CTPS | CTPS Entrez,  Source | CTP synthase | 18943 | -0.189 | -0.2279 | Yes |
| 79 | POLA1 | POLA1 Entrez,  Source | polymerase (DNA directed), alpha 1 | 18962 | -0.191 | -0.2050 | Yes |
| 80 | UCK2 | UCK2 Entrez,  Source | uridine-cytidine kinase 2 | 19004 | -0.195 | -0.1827 | Yes |
| 81 | POLR1E | POLR1E Entrez,  Source | polymerase (RNA) I polypeptide E, 53kDa | 19019 | -0.196 | -0.1588 | Yes |
| 82 | POLD1 | POLD1 Entrez,  Source | polymerase (DNA directed), delta 1, catalytic subunit 125kDa | 19075 | -0.205 | -0.1360 | Yes |
| 83 | CAD | CAD Entrez,  Source | carbamoyl-phosphate synthetase 2, aspartate transcarbamylase, and dihydroorotase | 19096 | -0.208 | -0.1110 | Yes |
| 84 | DHODH | DHODH Entrez,  Source | dihydroorotate dehydrogenase | 19097 | -0.208 | -0.0849 | Yes |
| 85 | POLE2 | POLE2 Entrez,  Source | polymerase (DNA directed), epsilon 2 (p59 subunit) | 19106 | -0.210 | -0.0591 | Yes |
| 86 | POLR1B | POLR1B Entrez,  Source | polymerase (RNA) I polypeptide B, 128kDa | 19138 | -0.214 | -0.0339 | Yes |
| 87 | POLR3K | POLR3K Entrez,  Source | polymerase (RNA) III (DNA directed) polypeptide K, 12.3 kDa | 19162 | -0.218 | -0.0079 | Yes |
| 88 | POLR3G | POLR3G Entrez,  Source | polymerase (RNA) III (DNA directed) polypeptide G (32kD) | 19204 | -0.224 | 0.0181 | Yes |
Table: GSEA details [plain text format]

  

Fig 2: KEGG\_PYRIMIDINE\_METABOLISM      
 Blue-Pink O' Gram in the Space of the Analyzed GeneSet

  

Fig 3: KEGG\_PYRIMIDINE\_METABOLISM: Random ES distribution      
 Gene set null distribution of ES for **KEGG\_PYRIMIDINE\_METABOLISM**
