# Supplementary material for: Comprehensive analysis of β-catenin target genes in colorectal carcinoma cell lines with deregulated Wnt/β-catenin signaling
Source: BMC Genomics. 2014 Jan 28;15:74. doi: 10.1186/1471-2164-15-74 (PMC3909937; doi:10.1186/1471-2164-15-74)
Supplement: Additional file 5 — GSEA analysis using the KEGG pathway database. This zipped file contains confirming data of the GSEA analysis. The names of the directories containing the files were composed of the term ‘GSEA’, the name of the cell line, e.g. DLD1, SW480, or LS174T, and the pathway database (KEGG). Please use a web browser to view the files with the name ‘index.html’ in the corresponding directories to start exploring the data. [file 1471-2164-15-74-S5.zip › GSEA_KEGG_DLD1/KEGG_RENIN_ANGIOTENSIN_SYSTEM.html]

Details for gene set KEGG\_RENIN\_ANGIOTENSIN\_SYSTEM[GSEA]

|  || Dataset | DLD1\_collapsed\_to\_symbols.class.cls#b\_versus\_bg.class.cls#b\_versus\_bg\_repos |
| Phenotype | class.cls#b\_versus\_bg\_repos |
| Upregulated in class | 0 |
| GeneSet | KEGG\_RENIN\_ANGIOTENSIN\_SYSTEM |
| Enrichment Score (ES) | -0.5911436 |
| Normalized Enrichment Score (NES) | -1.5381883 |
| Nominal p-value | 0.026923077 |
| FDR q-value | 0.085172094 |
| FWER p-Value | 0.796 |
Table: GSEA Results Summary

  

Fig 1: Enrichment plot: KEGG\_RENIN\_ANGIOTENSIN\_SYSTEM      
 Profile of the Running ES Score & Positions of GeneSet Members on the Rank Ordered List

  

| PROBE | GENE SYMBOL | GENE\_TITLE | RANK IN GENE LIST | RANK METRIC SCORE | RUNNING ES | CORE ENRICHMENT || 1 | ACE2 | ACE2 Entrez,  Source | angiotensin I converting enzyme (peptidyl-dipeptidase A) 2 | 2601 | 0.082 | -0.0660 | No |
| 2 | LNPEP | LNPEP Entrez,  Source | leucyl/cystinyl aminopeptidase | 4989 | 0.040 | -0.1555 | No |
| 3 | NLN | NLN Entrez,  Source | neurolysin (metallopeptidase M3 family) | 6026 | 0.027 | -0.1862 | No |
| 4 | CMA1 | CMA1 Entrez,  Source | chymase 1, mast cell | 6336 | 0.024 | -0.1824 | No |
| 5 | ANPEP | ANPEP Entrez,  Source | alanyl (membrane) aminopeptidase (aminopeptidase N, aminopeptidase M, microsomal aminopeptidase, CD13, p150) | 6636 | 0.021 | -0.1804 | No |
| 6 | AGTR2 | AGTR2 Entrez,  Source | angiotensin II receptor, type 2 | 7420 | 0.013 | -0.2100 | No |
| 7 | ACE | ACE Entrez,  Source | angiotensin I converting enzyme (peptidyl-dipeptidase A) 1 | 8447 | 0.003 | -0.2600 | No |
| 8 | MME | MME Entrez,  Source | membrane metallo-endopeptidase (neutral endopeptidase, enkephalinase) | 9173 | -0.003 | -0.2944 | No |
| 9 | MAS1 | MAS1 Entrez,  Source | MAS1 oncogene | 11730 | -0.026 | -0.4043 | No |
| 10 | THOP1 | THOP1 Entrez,  Source | thimet oligopeptidase 1 | 15383 | -0.068 | -0.5355 | Yes |
| 11 | CTSG | CTSG Entrez,  Source | cathepsin G | 15817 | -0.076 | -0.4961 | Yes |
| 12 | AGT | AGT Entrez,  Source | angiotensinogen (serpin peptidase inhibitor, clade A, member 8) | 15878 | -0.077 | -0.4367 | Yes |
| 13 | CPA3 | CPA3 Entrez,  Source | carboxypeptidase A3 (mast cell) | 16541 | -0.089 | -0.3983 | Yes |
| 14 | REN | REN Entrez,  Source | renin | 16555 | -0.089 | -0.3264 | Yes |
| 15 | AGTR1 | AGTR1 Entrez,  Source | angiotensin II receptor, type 1 | 17006 | -0.099 | -0.2691 | Yes |
| 16 | ENPEP | ENPEP Entrez,  Source | glutamyl aminopeptidase (aminopeptidase A) | 19536 | -0.491 | 0.0010 | Yes |
Table: GSEA details [plain text format]

  

Fig 2: KEGG\_RENIN\_ANGIOTENSIN\_SYSTEM      
 Blue-Pink O' Gram in the Space of the Analyzed GeneSet

  

Fig 3: KEGG\_RENIN\_ANGIOTENSIN\_SYSTEM: Random ES distribution      
 Gene set null distribution of ES for **KEGG\_RENIN\_ANGIOTENSIN\_SYSTEM**
