# Supplementary material for: Comprehensive analysis of β-catenin target genes in colorectal carcinoma cell lines with deregulated Wnt/β-catenin signaling
Source: BMC Genomics. 2014 Jan 28;15:74. doi: 10.1186/1471-2164-15-74 (PMC3909937; doi:10.1186/1471-2164-15-74)
Supplement: Additional file 5 — GSEA analysis using the KEGG pathway database. This zipped file contains confirming data of the GSEA analysis. The names of the directories containing the files were composed of the term ‘GSEA’, the name of the cell line, e.g. DLD1, SW480, or LS174T, and the pathway database (KEGG). Please use a web browser to view the files with the name ‘index.html’ in the corresponding directories to start exploring the data. [file 1471-2164-15-74-S5.zip › GSEA_KEGG_DLD1/KEGG_RIBOSOME.html]

Details for gene set KEGG\_RIBOSOME[GSEA]

|  || Dataset | DLD1\_collapsed\_to\_symbols.class.cls#b\_versus\_bg.class.cls#b\_versus\_bg\_repos |
| Phenotype | class.cls#b\_versus\_bg\_repos |
| Upregulated in class | 0 |
| GeneSet | KEGG\_RIBOSOME |
| Enrichment Score (ES) | -0.45906448 |
| Normalized Enrichment Score (NES) | -1.699533 |
| Nominal p-value | 0.0019193857 |
| FDR q-value | 0.034444615 |
| FWER p-Value | 0.279 |
Table: GSEA Results Summary

  

Fig 1: Enrichment plot: KEGG\_RIBOSOME      
 Profile of the Running ES Score & Positions of GeneSet Members on the Rank Ordered List

  

| PROBE | GENE SYMBOL | GENE\_TITLE | RANK IN GENE LIST | RANK METRIC SCORE | RUNNING ES | CORE ENRICHMENT || 1 | RPL26L1 | RPL26L1 Entrez,  Source | ribosomal protein L26-like 1 | 5252 | 0.037 | -0.2501 | No |
| 2 | RPL37A | RPL37A Entrez,  Source | ribosomal protein L37a | 8063 | 0.007 | -0.3908 | No |
| 3 | RPL41 | RPL41 Entrez,  Source | ribosomal protein L41 | 8910 | -0.001 | -0.4337 | No |
| 4 | RPL39 | RPL39 Entrez,  Source | ribosomal protein L39 | 9159 | -0.003 | -0.4447 | No |
| 5 | RPS2 | RPS2 Entrez,  Source | ribosomal protein S2 | 9258 | -0.004 | -0.4475 | No |
| 6 | RPL23A | RPL23A Entrez,  Source | ribosomal protein L23a | 9395 | -0.005 | -0.4517 | No |
| 7 | RPL3L | RPL3L Entrez,  Source | ribosomal protein L3-like | 9431 | -0.006 | -0.4505 | No |
| 8 | RPL36AL | RPL36AL Entrez,  Source | ribosomal protein L36a-like | 9598 | -0.007 | -0.4552 | Yes |
| 9 | RPS6 | RPS6 Entrez,  Source | ribosomal protein S6 | 9625 | -0.007 | -0.4526 | Yes |
| 10 | RPS27L | RPS27L Entrez,  Source | ribosomal protein S27-like | 9704 | -0.008 | -0.4523 | Yes |
| 11 | RPL37 | RPL37 Entrez,  Source | ribosomal protein L37 | 9809 | -0.009 | -0.4528 | Yes |
| 12 | RPS16 | RPS16 Entrez,  Source | ribosomal protein S16 | 9864 | -0.009 | -0.4505 | Yes |
| 13 | RPLP0 | RPLP0 Entrez,  Source | ribosomal protein, large, P0 | 9973 | -0.010 | -0.4506 | Yes |
| 14 | RPL18 | RPL18 Entrez,  Source | ribosomal protein L18 | 10050 | -0.011 | -0.4486 | Yes |
| 15 | RPL32 | RPL32 Entrez,  Source | ribosomal protein L32 | 10054 | -0.011 | -0.4429 | Yes |
| 16 | RPL27A | RPL27A Entrez,  Source | ribosomal protein L27a | 10139 | -0.012 | -0.4410 | Yes |
| 17 | RPS20 | RPS20 Entrez,  Source | ribosomal protein S20 | 10157 | -0.012 | -0.4355 | Yes |
| 18 | RPLP1 | RPLP1 Entrez,  Source | ribosomal protein, large, P1 | 10181 | -0.012 | -0.4303 | Yes |
| 19 | RPS18 | RPS18 Entrez,  Source | ribosomal protein S18 | 10183 | -0.012 | -0.4238 | Yes |
| 20 | RPL6 | RPL6 Entrez,  Source | ribosomal protein L6 | 10188 | -0.012 | -0.4176 | Yes |
| 21 | RPL7 | RPL7 Entrez,  Source | ribosomal protein L7 | 10191 | -0.012 | -0.4112 | Yes |
| 22 | RPL9 | RPL9 Entrez,  Source | ribosomal protein L9 | 10199 | -0.012 | -0.4050 | Yes |
| 23 | RPL29 | RPL29 Entrez,  Source | ribosomal protein L29 | 10347 | -0.014 | -0.4052 | Yes |
| 24 | RPS27 | RPS27 Entrez,  Source | ribosomal protein S27 (metallopanstimulin 1) | 10350 | -0.014 | -0.3979 | Yes |
| 25 | RPS3 | RPS3 Entrez,  Source | ribosomal protein S3 | 10377 | -0.014 | -0.3917 | Yes |
| 26 | RPS13 | RPS13 Entrez,  Source | ribosomal protein S13 | 10432 | -0.015 | -0.3867 | Yes |
| 27 | RPL7A | RPL7A Entrez,  Source | ribosomal protein L7a | 10447 | -0.015 | -0.3796 | Yes |
| 28 | RPL17 | RPL17 Entrez,  Source | ribosomal protein L17 | 10471 | -0.015 | -0.3728 | Yes |
| 29 | RPL15 | RPL15 Entrez,  Source | ribosomal protein L15 | 10490 | -0.015 | -0.3656 | Yes |
| 30 | RPS27A | RPS27A Entrez,  Source | ribosomal protein S27a | 10529 | -0.015 | -0.3594 | Yes |
| 31 | RPS3A | RPS3A Entrez,  Source | ribosomal protein S3A | 10575 | -0.016 | -0.3533 | Yes |
| 32 | RPL31 | RPL31 Entrez,  Source | ribosomal protein L31 | 10590 | -0.016 | -0.3456 | Yes |
| 33 | RPL13 | RPL13 Entrez,  Source | ribosomal protein L13 | 10681 | -0.016 | -0.3415 | Yes |
| 34 | RPS21 | RPS21 Entrez,  Source | ribosomal protein S21 | 10760 | -0.017 | -0.3362 | Yes |
| 35 | RPL12 | RPL12 Entrez,  Source | ribosomal protein L12 | 10774 | -0.017 | -0.3276 | Yes |
| 36 | RPLP2 | RPLP2 Entrez,  Source | ribosomal protein, large, P2 | 10775 | -0.017 | -0.3183 | Yes |
| 37 | RPS15 | RPS15 Entrez,  Source | ribosomal protein S15 | 10823 | -0.018 | -0.3112 | Yes |
| 38 | RPS4X | RPS4X Entrez,  Source | ribosomal protein S4, X-linked | 10901 | -0.018 | -0.3053 | Yes |
| 39 | RPL13A | RPL13A Entrez,  Source | ribosomal protein L13a | 10946 | -0.019 | -0.2975 | Yes |
| 40 | RPS11 | RPS11 Entrez,  Source | ribosomal protein S11 | 10968 | -0.019 | -0.2884 | Yes |
| 41 | RPL27 | RPL27 Entrez,  Source | ribosomal protein L27 | 10986 | -0.019 | -0.2791 | Yes |
| 42 | RPS19 | RPS19 Entrez,  Source | ribosomal protein S19 | 10995 | -0.019 | -0.2692 | Yes |
| 43 | RPL5 | RPL5 Entrez,  Source | ribosomal protein L5 | 11099 | -0.020 | -0.2638 | Yes |
| 44 | RPS15A | RPS15A Entrez,  Source | ribosomal protein S15a | 11150 | -0.020 | -0.2555 | Yes |
| 45 | RPS23 | RPS23 Entrez,  Source | ribosomal protein S23 | 11220 | -0.021 | -0.2478 | Yes |
| 46 | RPL28 | RPL28 Entrez,  Source | ribosomal protein L28 | 11243 | -0.021 | -0.2376 | Yes |
| 47 | RPL8 | RPL8 Entrez,  Source | ribosomal protein L8 | 11343 | -0.022 | -0.2308 | Yes |
| 48 | RPL10 | RPL10 Entrez,  Source | ribosomal protein L10 | 11351 | -0.022 | -0.2193 | Yes |
| 49 | RPL38 | RPL38 Entrez,  Source | ribosomal protein L38 | 11356 | -0.022 | -0.2075 | Yes |
| 50 | RPS7 | RPS7 Entrez,  Source | ribosomal protein S7 | 11417 | -0.023 | -0.1984 | Yes |
| 51 | RPL24 | RPL24 Entrez,  Source | ribosomal protein L24 | 11453 | -0.023 | -0.1878 | Yes |
| 52 | RPS24 | RPS24 Entrez,  Source | ribosomal protein S24 | 11535 | -0.024 | -0.1792 | Yes |
| 53 | RPS12 | RPS12 Entrez,  Source | ribosomal protein S12 | 11560 | -0.024 | -0.1675 | Yes |
| 54 | RPS10 | RPS10 Entrez,  Source | ribosomal protein S10 | 11561 | -0.024 | -0.1545 | Yes |
| 55 | RPS17 | RPS17 Entrez,  Source | ribosomal protein S17 | 11565 | -0.024 | -0.1417 | Yes |
| 56 | RPL10A | RPL10A Entrez,  Source | ribosomal protein L10a | 11628 | -0.025 | -0.1316 | Yes |
| 57 | RPS29 | RPS29 Entrez,  Source | ribosomal protein S29 | 11830 | -0.027 | -0.1277 | Yes |
| 58 | RPL3 | RPL3 Entrez,  Source | ribosomal protein L3 | 11871 | -0.027 | -0.1153 | Yes |
| 59 | RPL19 | RPL19 Entrez,  Source | ribosomal protein L19 | 11928 | -0.028 | -0.1034 | Yes |
| 60 | RPS5 | RPS5 Entrez,  Source | ribosomal protein S5 | 12006 | -0.028 | -0.0922 | Yes |
| 61 | RPL35A | RPL35A Entrez,  Source | ribosomal protein L35a | 12024 | -0.029 | -0.0778 | Yes |
| 62 | RPS8 | RPS8 Entrez,  Source | ribosomal protein S8 | 12115 | -0.029 | -0.0667 | Yes |
| 63 | RPL30 | RPL30 Entrez,  Source | ribosomal protein L30 | 12229 | -0.030 | -0.0563 | Yes |
| 64 | RPL4 | RPL4 Entrez,  Source | ribosomal protein L4 | 12480 | -0.033 | -0.0515 | Yes |
| 65 | UBA52 | UBA52 Entrez,  Source | ubiquitin A-52 residue ribosomal protein fusion product 1 | 12768 | -0.036 | -0.0471 | Yes |
| 66 | RPS25 | RPS25 Entrez,  Source | ribosomal protein S25 | 13014 | -0.038 | -0.0392 | Yes |
| 67 | FAU | FAU Entrez,  Source | Finkel-Biskis-Reilly murine sarcoma virus (FBR-MuSV) ubiquitously expressed (fox derived); ribosomal protein S30 | 13036 | -0.039 | -0.0196 | Yes |
| 68 | RPL11 | RPL11 Entrez,  Source | ribosomal protein L11 | 13081 | -0.039 | -0.0010 | Yes |
| 69 | RPS9 | RPS9 Entrez,  Source | ribosomal protein S9 | 13084 | -0.039 | 0.0198 | Yes |
| 70 | RPL22 | RPL22 Entrez,  Source | ribosomal protein L22 | 13319 | -0.042 | 0.0301 | Yes |
| 71 | MRPL13 | MRPL13 Entrez,  Source | mitochondrial ribosomal protein L13 | 13427 | -0.043 | 0.0475 | Yes |
| 72 | RPL35 | RPL35 Entrez,  Source | ribosomal protein L35 | 13459 | -0.043 | 0.0690 | Yes |
| 73 | RPL36 | RPL36 Entrez,  Source | ribosomal protein L36 | 13599 | -0.045 | 0.0857 | Yes |
| 74 | RPL23 | RPL23 Entrez,  Source | ribosomal protein L23 | 13618 | -0.045 | 0.1088 | Yes |
| 75 | RPS4Y1 | RPS4Y1 Entrez,  Source | ribosomal protein S4, Y-linked 1 | 13771 | -0.046 | 0.1258 | Yes |
| 76 | RPL14 | RPL14 Entrez,  Source | ribosomal protein L14 | 13924 | -0.048 | 0.1438 | Yes |
| 77 | RPL10L | RPL10L Entrez,  Source | ribosomal protein L10-like | 16653 | -0.091 | 0.0525 | Yes |
| 78 | RPS28 | RPS28 Entrez,  Source | ribosomal protein S28 | 18864 | -0.180 | 0.0355 | Yes |
Table: GSEA details [plain text format]

  

Fig 2: KEGG\_RIBOSOME      
 Blue-Pink O' Gram in the Space of the Analyzed GeneSet

  

Fig 3: KEGG\_RIBOSOME: Random ES distribution      
 Gene set null distribution of ES for **KEGG\_RIBOSOME**
