# Supplementary material for: Comprehensive analysis of β-catenin target genes in colorectal carcinoma cell lines with deregulated Wnt/β-catenin signaling
Source: BMC Genomics. 2014 Jan 28;15:74. doi: 10.1186/1471-2164-15-74 (PMC3909937; doi:10.1186/1471-2164-15-74)
Supplement: Additional file 5 — GSEA analysis using the KEGG pathway database. This zipped file contains confirming data of the GSEA analysis. The names of the directories containing the files were composed of the term ‘GSEA’, the name of the cell line, e.g. DLD1, SW480, or LS174T, and the pathway database (KEGG). Please use a web browser to view the files with the name ‘index.html’ in the corresponding directories to start exploring the data. [file 1471-2164-15-74-S5.zip › GSEA_KEGG_DLD1/KEGG_RNA_DEGRADATION.html]

Details for gene set KEGG\_RNA\_DEGRADATION[GSEA]

|  || Dataset | DLD1\_collapsed\_to\_symbols.class.cls#b\_versus\_bg.class.cls#b\_versus\_bg\_repos |
| Phenotype | class.cls#b\_versus\_bg\_repos |
| Upregulated in class | 0 |
| GeneSet | KEGG\_RNA\_DEGRADATION |
| Enrichment Score (ES) | -0.48651174 |
| Normalized Enrichment Score (NES) | -1.6553489 |
| Nominal p-value | 0.0057142857 |
| FDR q-value | 0.040011793 |
| FWER p-Value | 0.401 |
Table: GSEA Results Summary

  

Fig 1: Enrichment plot: KEGG\_RNA\_DEGRADATION      
 Profile of the Running ES Score & Positions of GeneSet Members on the Rank Ordered List

  

| PROBE | GENE SYMBOL | GENE\_TITLE | RANK IN GENE LIST | RANK METRIC SCORE | RUNNING ES | CORE ENRICHMENT || 1 | PAPOLB | PAPOLB Entrez,  Source | poly(A) polymerase beta (testis specific) | 2085 | 0.097 | -0.0757 | No |
| 2 | CNOT4 | CNOT4 Entrez,  Source | CCR4-NOT transcription complex, subunit 4 | 2523 | 0.084 | -0.0709 | No |
| 3 | C1D | C1D Entrez,  Source | - | 4201 | 0.051 | -0.1404 | No |
| 4 | XRN1 | XRN1 Entrez,  Source | 5'-3' exoribonuclease 1 | 4217 | 0.051 | -0.1247 | No |
| 5 | DCP2 | DCP2 Entrez,  Source | DCP2 decapping enzyme homolog (S. cerevisiae) | 4447 | 0.048 | -0.1210 | No |
| 6 | DDX6 | DDX6 Entrez,  Source | DEAD (Asp-Glu-Ala-Asp) box polypeptide 6 | 5566 | 0.033 | -0.1678 | No |
| 7 | ENO2 | ENO2 Entrez,  Source | enolase 2 (gamma, neuronal) | 5577 | 0.033 | -0.1579 | No |
| 8 | LSM1 | LSM1 Entrez,  Source | LSM1 homolog, U6 small nuclear RNA associated (S. cerevisiae) | 7143 | 0.016 | -0.2330 | No |
| 9 | PAPOLG | PAPOLG Entrez,  Source | poly(A) polymerase gamma | 7477 | 0.012 | -0.2461 | No |
| 10 | CNOT3 | CNOT3 Entrez,  Source | CCR4-NOT transcription complex, subunit 3 | 8522 | 0.002 | -0.2988 | No |
| 11 | PAPOLA | PAPOLA Entrez,  Source | poly(A) polymerase alpha | 9171 | -0.003 | -0.3310 | No |
| 12 | CNOT6 | CNOT6 Entrez,  Source | CCR4-NOT transcription complex, subunit 6 | 9936 | -0.010 | -0.3669 | No |
| 13 | DCP1A | DCP1A Entrez,  Source | DCP1 decapping enzyme homolog A (S. cerevisiae) | 10029 | -0.011 | -0.3681 | No |
| 14 | CNOT8 | CNOT8 Entrez,  Source | CCR4-NOT transcription complex, subunit 8 | 10647 | -0.016 | -0.3945 | No |
| 15 | SKIV2L | SKIV2L Entrez,  Source | superkiller viralicidic activity 2-like (S. cerevisiae) | 10919 | -0.019 | -0.4024 | No |
| 16 | CNOT7 | CNOT7 Entrez,  Source | CCR4-NOT transcription complex, subunit 7 | 11007 | -0.019 | -0.4007 | No |
| 17 | HSPD1 | HSPD1 Entrez,  Source | heat shock 60kDa protein 1 (chaperonin) | 11905 | -0.027 | -0.4378 | No |
| 18 | CNOT6L | CNOT6L Entrez,  Source | CCR4-NOT transcription complex, subunit 6-like | 11955 | -0.028 | -0.4314 | No |
| 19 | EDC3 | EDC3 Entrez,  Source | enhancer of mRNA decapping 3 homolog (S. cerevisiae) | 12152 | -0.030 | -0.4319 | No |
| 20 | ENO1 | ENO1 Entrez,  Source | enolase 1, (alpha) | 13219 | -0.041 | -0.4734 | Yes |
| 21 | WDR61 | WDR61 Entrez,  Source | WD repeat domain 61 | 13243 | -0.041 | -0.4614 | Yes |
| 22 | PARN | PARN Entrez,  Source | poly(A)-specific ribonuclease (deadenylation nuclease) | 13425 | -0.043 | -0.4569 | Yes |
| 23 | LSM5 | LSM5 Entrez,  Source | LSM5 homolog, U6 small nuclear RNA associated (S. cerevisiae) | 13432 | -0.043 | -0.4434 | Yes |
| 24 | RQCD1 | RQCD1 Entrez,  Source | RCD1 required for cell differentiation1 homolog (S. pombe) | 13816 | -0.047 | -0.4479 | Yes |
| 25 | CNOT1 | CNOT1 Entrez,  Source | CCR4-NOT transcription complex, subunit 1 | 13927 | -0.048 | -0.4380 | Yes |
| 26 | ENO3 | ENO3 Entrez,  Source | enolase 3 (beta, muscle) | 13952 | -0.049 | -0.4236 | Yes |
| 27 | SKIV2L2 | SKIV2L2 Entrez,  Source | superkiller viralicidic activity 2-like 2 (S. cerevisiae) | 14063 | -0.050 | -0.4131 | Yes |
| 28 | EXOSC1 | EXOSC1 Entrez,  Source | exosome component 1 | 14293 | -0.053 | -0.4079 | Yes |
| 29 | EXOSC10 | EXOSC10 Entrez,  Source | exosome component 10 | 14325 | -0.053 | -0.3923 | Yes |
| 30 | LSM4 | LSM4 Entrez,  Source | LSM4 homolog, U6 small nuclear RNA associated (S. cerevisiae) | 14363 | -0.054 | -0.3769 | Yes |
| 31 | CNOT2 | CNOT2 Entrez,  Source | CCR4-NOT transcription complex, subunit 2 | 14472 | -0.055 | -0.3647 | Yes |
| 32 | LSM3 | LSM3 Entrez,  Source | LSM3 homolog, U6 small nuclear RNA associated (S. cerevisiae) | 14532 | -0.056 | -0.3497 | Yes |
| 33 | DCPS | DCPS Entrez,  Source | decapping enzyme, scavenger | 14921 | -0.062 | -0.3498 | Yes |
| 34 | EXOSC6 | EXOSC6 Entrez,  Source | exosome component 6 | 15081 | -0.064 | -0.3373 | Yes |
| 35 | CNOT10 | CNOT10 Entrez,  Source | CCR4-NOT transcription complex, subunit 10 | 15181 | -0.065 | -0.3214 | Yes |
| 36 | MPHOSPH6 | MPHOSPH6 Entrez,  Source | M-phase phosphoprotein 6 | 15311 | -0.067 | -0.3063 | Yes |
| 37 | DCP1B | DCP1B Entrez,  Source | DCP1 decapping enzyme homolog B (S. cerevisiae) | 15384 | -0.068 | -0.2880 | Yes |
| 38 | EDC4 | EDC4 Entrez,  Source | enhancer of mRNA decapping 4 | 15443 | -0.069 | -0.2686 | Yes |
| 39 | LSM2 | LSM2 Entrez,  Source | LSM2 homolog, U6 small nuclear RNA associated (S. cerevisiae) | 15446 | -0.069 | -0.2464 | Yes |
| 40 | EXOSC7 | EXOSC7 Entrez,  Source | exosome component 7 | 15615 | -0.072 | -0.2318 | Yes |
| 41 | LSM7 | LSM7 Entrez,  Source | LSM7 homolog, U6 small nuclear RNA associated (S. cerevisiae) | 15910 | -0.077 | -0.2219 | Yes |
| 42 | LSM6 | LSM6 Entrez,  Source | LSM6 homolog, U6 small nuclear RNA associated (S. cerevisiae) | 16216 | -0.083 | -0.2110 | Yes |
| 43 | XRN2 | XRN2 Entrez,  Source | 5'-3' exoribonuclease 2 | 16397 | -0.086 | -0.1924 | Yes |
| 44 | ZCCHC7 | ZCCHC7 Entrez,  Source | zinc finger, CCHC domain containing 7 | 16501 | -0.088 | -0.1693 | Yes |
| 45 | PNPT1 | PNPT1 Entrez,  Source | polyribonucleotide nucleotidyltransferase 1 | 16702 | -0.093 | -0.1498 | Yes |
| 46 | EXOSC3 | EXOSC3 Entrez,  Source | exosome component 3 | 17034 | -0.099 | -0.1347 | Yes |
| 47 | EXOSC8 | EXOSC8 Entrez,  Source | exosome component 8 | 17176 | -0.102 | -0.1089 | Yes |
| 48 | EXOSC9 | EXOSC9 Entrez,  Source | exosome component 9 | 17898 | -0.124 | -0.1058 | Yes |
| 49 | EXOSC2 | EXOSC2 Entrez,  Source | exosome component 2 | 18603 | -0.158 | -0.0911 | Yes |
| 50 | EXOSC4 | EXOSC4 Entrez,  Source | exosome component 4 | 18758 | -0.170 | -0.0442 | Yes |
| 51 | EXOSC5 | EXOSC5 Entrez,  Source | exosome component 5 | 19333 | -0.264 | 0.0114 | Yes |
Table: GSEA details [plain text format]

  

Fig 2: KEGG\_RNA\_DEGRADATION      
 Blue-Pink O' Gram in the Space of the Analyzed GeneSet

  

Fig 3: KEGG\_RNA\_DEGRADATION: Random ES distribution      
 Gene set null distribution of ES for **KEGG\_RNA\_DEGRADATION**
