# Supplementary material for: Comprehensive analysis of β-catenin target genes in colorectal carcinoma cell lines with deregulated Wnt/β-catenin signaling
Source: BMC Genomics. 2014 Jan 28;15:74. doi: 10.1186/1471-2164-15-74 (PMC3909937; doi:10.1186/1471-2164-15-74)
Supplement: Additional file 5 — GSEA analysis using the KEGG pathway database. This zipped file contains confirming data of the GSEA analysis. The names of the directories containing the files were composed of the term ‘GSEA’, the name of the cell line, e.g. DLD1, SW480, or LS174T, and the pathway database (KEGG). Please use a web browser to view the files with the name ‘index.html’ in the corresponding directories to start exploring the data. [file 1471-2164-15-74-S5.zip › GSEA_KEGG_DLD1/KEGG_SPHINGOLIPID_METABOLISM.html]

Details for gene set KEGG\_SPHINGOLIPID\_METABOLISM[GSEA]

|  || Dataset | DLD1\_collapsed\_to\_symbols.class.cls#b\_versus\_bg.class.cls#b\_versus\_bg\_repos |
| Phenotype | class.cls#b\_versus\_bg\_repos |
| Upregulated in class | 1 |
| GeneSet | KEGG\_SPHINGOLIPID\_METABOLISM |
| Enrichment Score (ES) | 0.50274605 |
| Normalized Enrichment Score (NES) | 1.5106529 |
| Nominal p-value | 0.03909465 |
| FDR q-value | 0.17178115 |
| FWER p-Value | 0.928 |
Table: GSEA Results Summary

  

Fig 1: Enrichment plot: KEGG\_SPHINGOLIPID\_METABOLISM      
 Profile of the Running ES Score & Positions of GeneSet Members on the Rank Ordered List

  

| PROBE | GENE SYMBOL | GENE\_TITLE | RANK IN GENE LIST | RANK METRIC SCORE | RUNNING ES | CORE ENRICHMENT || 1 | NEU1 | NEU1 Entrez,  Source | sialidase 1 (lysosomal sialidase) | 320 | 0.261 | 0.0853 | Yes |
| 2 | ARSA | ARSA Entrez,  Source | arylsulfatase A | 780 | 0.169 | 0.1276 | Yes |
| 3 | SMPD1 | SMPD1 Entrez,  Source | sphingomyelin phosphodiesterase 1, acid lysosomal (acid sphingomyelinase) | 856 | 0.161 | 0.1867 | Yes |
| 4 | ASAH1 | ASAH1 Entrez,  Source | N-acylsphingosine amidohydrolase (acid ceramidase) 1 | 875 | 0.159 | 0.2480 | Yes |
| 5 | CERK | CERK Entrez,  Source | ceramide kinase | 1043 | 0.146 | 0.2965 | Yes |
| 6 | PPAP2A | PPAP2A Entrez,  Source | phosphatidic acid phosphatase type 2A | 1177 | 0.136 | 0.3427 | Yes |
| 7 | SMPD3 | SMPD3 Entrez,  Source | sphingomyelin phosphodiesterase 3, neutral membrane (neutral sphingomyelinase II) | 1569 | 0.115 | 0.3675 | Yes |
| 8 | ENPP7 | ENPP7 Entrez,  Source | ectonucleotide pyrophosphatase/phosphodiesterase 7 | 1742 | 0.108 | 0.4008 | Yes |
| 9 | UGCG | UGCG Entrez,  Source | UDP-glucose ceramide glucosyltransferase | 2141 | 0.095 | 0.4174 | Yes |
| 10 | DEGS2 | DEGS2 Entrez,  Source | degenerative spermatocyte homolog 2, lipid desaturase (Drosophila) | 2304 | 0.090 | 0.4443 | Yes |
| 11 | PPAP2B | PPAP2B Entrez,  Source | phosphatidic acid phosphatase type 2B | 2436 | 0.087 | 0.4715 | Yes |
| 12 | SPTLC2 | SPTLC2 Entrez,  Source | serine palmitoyltransferase, long chain base subunit 2 | 2701 | 0.080 | 0.4892 | Yes |
| 13 | GALC | GALC Entrez,  Source | galactosylceramidase | 3056 | 0.072 | 0.4992 | Yes |
| 14 | SPHK2 | SPHK2 Entrez,  Source | sphingosine kinase 2 | 3471 | 0.063 | 0.5027 | Yes |
| 15 | SGPP1 | SGPP1 Entrez,  Source | sphingosine-1-phosphate phosphatase 1 | 4112 | 0.053 | 0.4905 | No |
| 16 | SGPL1 | SGPL1 Entrez,  Source | sphingosine-1-phosphate lyase 1 | 4313 | 0.050 | 0.4996 | No |
| 17 | SPTLC1 | SPTLC1 Entrez,  Source | serine palmitoyltransferase, long chain base subunit 1 | 4740 | 0.044 | 0.4948 | No |
| 18 | GLB1 | GLB1 Entrez,  Source | galactosidase, beta 1 | 6137 | 0.026 | 0.4336 | No |
| 19 | DEGS1 | DEGS1 Entrez,  Source | degenerative spermatocyte homolog 1, lipid desaturase (Drosophila) | 7277 | 0.014 | 0.3809 | No |
| 20 | NEU4 | NEU4 Entrez,  Source | sialidase 4 | 8407 | 0.003 | 0.3244 | No |
| 21 | PPAP2C | PPAP2C Entrez,  Source | phosphatidic acid phosphatase type 2C | 8770 | 0.000 | 0.3060 | No |
| 22 | NEU2 | NEU2 Entrez,  Source | sialidase 2 (cytosolic sialidase) | 11024 | -0.019 | 0.1982 | No |
| 23 | GLA | GLA Entrez,  Source | galactosidase, alpha | 13345 | -0.042 | 0.0957 | No |
| 24 | NEU3 | NEU3 Entrez,  Source | sialidase 3 (membrane sialidase) | 13607 | -0.045 | 0.0997 | No |
| 25 | SPHK1 | SPHK1 Entrez,  Source | sphingosine kinase 1 | 13939 | -0.048 | 0.1017 | No |
| 26 | SMPD2 | SMPD2 Entrez,  Source | sphingomyelin phosphodiesterase 2, neutral membrane (neutral sphingomyelinase) | 13950 | -0.049 | 0.1201 | No |
| 27 | GAL3ST1 | GAL3ST1 Entrez,  Source | galactose-3-O-sulfotransferase 1 | 14759 | -0.059 | 0.1018 | No |
| 28 | UGT8 | UGT8 Entrez,  Source | UDP glycosyltransferase 8 (UDP-galactose ceramide galactosyltransferase) | 15211 | -0.066 | 0.1043 | No |
| 29 | B4GALT6 | B4GALT6 Entrez,  Source | UDP-Gal:betaGlcNAc beta 1,4- galactosyltransferase, polypeptide 6 | 18266 | -0.139 | 0.0023 | No |
| 30 | SGPP2 | SGPP2 Entrez,  Source | sphingosine-1-phosphate phosphotase 2 | 18681 | -0.163 | 0.0448 | No |
Table: GSEA details [plain text format]

  

Fig 2: KEGG\_SPHINGOLIPID\_METABOLISM      
 Blue-Pink O' Gram in the Space of the Analyzed GeneSet

  

Fig 3: KEGG\_SPHINGOLIPID\_METABOLISM: Random ES distribution      
 Gene set null distribution of ES for **KEGG\_SPHINGOLIPID\_METABOLISM**
