# Supplementary material for: Comprehensive analysis of β-catenin target genes in colorectal carcinoma cell lines with deregulated Wnt/β-catenin signaling
Source: BMC Genomics. 2014 Jan 28;15:74. doi: 10.1186/1471-2164-15-74 (PMC3909937; doi:10.1186/1471-2164-15-74)
Supplement: Additional file 5 — GSEA analysis using the KEGG pathway database. This zipped file contains confirming data of the GSEA analysis. The names of the directories containing the files were composed of the term ‘GSEA’, the name of the cell line, e.g. DLD1, SW480, or LS174T, and the pathway database (KEGG). Please use a web browser to view the files with the name ‘index.html’ in the corresponding directories to start exploring the data. [file 1471-2164-15-74-S5.zip › GSEA_KEGG_DLD1/KEGG_SPLICEOSOME.html]

Details for gene set KEGG\_SPLICEOSOME[GSEA]

|  || Dataset | DLD1\_collapsed\_to\_symbols.class.cls#b\_versus\_bg.class.cls#b\_versus\_bg\_repos |
| Phenotype | class.cls#b\_versus\_bg\_repos |
| Upregulated in class | 0 |
| GeneSet | KEGG\_SPLICEOSOME |
| Enrichment Score (ES) | -0.40621182 |
| Normalized Enrichment Score (NES) | -1.5633659 |
| Nominal p-value | 0.009276438 |
| FDR q-value | 0.080555476 |
| FWER p-Value | 0.733 |
Table: GSEA Results Summary

  

Fig 1: Enrichment plot: KEGG\_SPLICEOSOME      
 Profile of the Running ES Score & Positions of GeneSet Members on the Rank Ordered List

  

| PROBE | GENE SYMBOL | GENE\_TITLE | RANK IN GENE LIST | RANK METRIC SCORE | RUNNING ES | CORE ENRICHMENT || 1 | HSPA1A | HSPA1A Entrez,  Source | heat shock 70kDa protein 1A | 212 | 0.322 | 0.0534 | No |
| 2 | HSPA6 | HSPA6 Entrez,  Source | heat shock 70kDa protein 6 (HSP70B') | 2002 | 0.099 | -0.0187 | No |
| 3 | HSPA2 | HSPA2 Entrez,  Source | heat shock 70kDa protein 2 | 2021 | 0.099 | 0.0001 | No |
| 4 | SLU7 | SLU7 Entrez,  Source | - | 4031 | 0.054 | -0.0924 | No |
| 5 | AQR | AQR Entrez,  Source | aquarius homolog (mouse) | 4519 | 0.047 | -0.1081 | No |
| 6 | HSPA1L | HSPA1L Entrez,  Source | heat shock 70kDa protein 1-like | 5251 | 0.037 | -0.1384 | No |
| 7 | PRPF3 | PRPF3 Entrez,  Source | PRP3 pre-mRNA processing factor 3 homolog (S. cerevisiae) | 6534 | 0.022 | -0.1998 | No |
| 8 | ZMAT2 | ZMAT2 Entrez,  Source | zinc finger, matrin type 2 | 6590 | 0.022 | -0.1983 | No |
| 9 | DHX8 | DHX8 Entrez,  Source | DEAH (Asp-Glu-Ala-His) box polypeptide 8 | 6732 | 0.020 | -0.2015 | No |
| 10 | CRNKL1 | CRNKL1 Entrez,  Source | Crn, crooked neck-like 1 (Drosophila) | 7051 | 0.017 | -0.2145 | No |
| 11 | PCBP1 | PCBP1 Entrez,  Source | poly(rC) binding protein 1 | 7459 | 0.013 | -0.2329 | No |
| 12 | RBM25 | RBM25 Entrez,  Source | RNA binding motif protein 25 | 8320 | 0.004 | -0.2763 | No |
| 13 | DDX5 | DDX5 Entrez,  Source | DEAD (Asp-Glu-Ala-Asp) box polypeptide 5 | 8404 | 0.003 | -0.2798 | No |
| 14 | SF3B1 | SF3B1 Entrez,  Source | splicing factor 3b, subunit 1, 155kDa | 8662 | 0.001 | -0.2928 | No |
| 15 | CDC40 | CDC40 Entrez,  Source | cell division cycle 40 homolog (S. cerevisiae) | 8832 | -0.000 | -0.3014 | No |
| 16 | PRPF6 | PRPF6 Entrez,  Source | PRP6 pre-mRNA processing factor 6 homolog (S. cerevisiae) | 8885 | -0.001 | -0.3039 | No |
| 17 | PRPF18 | PRPF18 Entrez,  Source | PRP18 pre-mRNA processing factor 18 homolog (S. cerevisiae) | 9039 | -0.002 | -0.3113 | No |
| 18 | THOC2 | THOC2 Entrez,  Source | THO complex 2 | 9127 | -0.003 | -0.3152 | No |
| 19 | DHX16 | DHX16 Entrez,  Source | DEAH (Asp-Glu-Ala-His) box polypeptide 16 | 9294 | -0.004 | -0.3229 | No |
| 20 | SMNDC1 | SMNDC1 Entrez,  Source | survival motor neuron domain containing 1 | 9335 | -0.005 | -0.3240 | No |
| 21 | BCAS2 | BCAS2 Entrez,  Source | breast carcinoma amplified sequence 2 | 9658 | -0.008 | -0.3390 | No |
| 22 | NCBP2 | NCBP2 Entrez,  Source | nuclear cap binding protein subunit 2, 20kDa | 9875 | -0.010 | -0.3482 | No |
| 23 | HSPA8 | HSPA8 Entrez,  Source | heat shock 70kDa protein 8 | 9964 | -0.010 | -0.3507 | No |
| 24 | PRPF40A | PRPF40A Entrez,  Source | PRP40 pre-mRNA processing factor 40 homolog A (yeast) | 10015 | -0.011 | -0.3511 | No |
| 25 | SNW1 | SNW1 Entrez,  Source | SNW domain containing 1 | 10445 | -0.015 | -0.3702 | No |
| 26 | TRA2A | TRA2A Entrez,  Source | - | 10973 | -0.019 | -0.3935 | No |
| 27 | SF3B4 | SF3B4 Entrez,  Source | splicing factor 3b, subunit 4, 49kDa | 11118 | -0.020 | -0.3969 | No |
| 28 | DDX42 | DDX42 Entrez,  Source | DEAD (Asp-Glu-Ala-Asp) box polypeptide 42 | 11292 | -0.022 | -0.4015 | No |
| 29 | SYF2 | SYF2 Entrez,  Source | SYF2 homolog, RNA splicing factor (S. cerevisiae) | 11385 | -0.023 | -0.4017 | Yes |
| 30 | CDC5L | CDC5L Entrez,  Source | CDC5 cell division cycle 5-like (S. pombe) | 11394 | -0.023 | -0.3976 | Yes |
| 31 | DHX15 | DHX15 Entrez,  Source | DEAH (Asp-Glu-Ala-His) box polypeptide 15 | 11515 | -0.024 | -0.3990 | Yes |
| 32 | SNRPG | SNRPG Entrez,  Source | small nuclear ribonucleoprotein polypeptide G | 11624 | -0.025 | -0.3996 | Yes |
| 33 | RBM17 | RBM17 Entrez,  Source | RNA binding motif protein 17 | 11668 | -0.025 | -0.3968 | Yes |
| 34 | BUD31 | BUD31 Entrez,  Source | BUD31 homolog (yeast) | 11708 | -0.026 | -0.3937 | Yes |
| 35 | PRPF38A | PRPF38A Entrez,  Source | PRP38 pre-mRNA processing factor 38 (yeast) domain containing A | 11800 | -0.026 | -0.3932 | Yes |
| 36 | RBM22 | RBM22 Entrez,  Source | RNA binding motif protein 22 | 11998 | -0.028 | -0.3976 | Yes |
| 37 | DHX38 | DHX38 Entrez,  Source | DEAH (Asp-Glu-Ala-His) box polypeptide 38 | 12022 | -0.029 | -0.3931 | Yes |
| 38 | SF3B14 | SF3B14 Entrez,  Source | - | 12170 | -0.030 | -0.3948 | Yes |
| 39 | SF3B2 | SF3B2 Entrez,  Source | splicing factor 3b, subunit 2, 145kDa | 12288 | -0.031 | -0.3946 | Yes |
| 40 | WBP11 | WBP11 Entrez,  Source | WW domain binding protein 11 | 12433 | -0.032 | -0.3956 | Yes |
| 41 | PQBP1 | PQBP1 Entrez,  Source | polyglutamine binding protein 1 | 12598 | -0.034 | -0.3972 | Yes |
| 42 | SNRPC | SNRPC Entrez,  Source | small nuclear ribonucleoprotein polypeptide C | 12601 | -0.034 | -0.3905 | Yes |
| 43 | CTNNBL1 | CTNNBL1 Entrez,  Source | catenin, beta like 1 | 12675 | -0.035 | -0.3873 | Yes |
| 44 | U2AF1 | U2AF1 Entrez,  Source | U2 small nuclear RNA auxiliary factor 1 | 12976 | -0.038 | -0.3952 | Yes |
| 45 | CCDC12 | CCDC12 Entrez,  Source | coiled-coil domain containing 12 | 13051 | -0.039 | -0.3913 | Yes |
| 46 | DDX23 | DDX23 Entrez,  Source | DEAD (Asp-Glu-Ala-Asp) box polypeptide 23 | 13142 | -0.040 | -0.3880 | Yes |
| 47 | LSM5 | LSM5 Entrez,  Source | LSM5 homolog, U6 small nuclear RNA associated (S. cerevisiae) | 13432 | -0.043 | -0.3943 | Yes |
| 48 | RBM8A | RBM8A Entrez,  Source | RNA binding motif protein 8A | 13522 | -0.044 | -0.3901 | Yes |
| 49 | PPIL1 | PPIL1 Entrez,  Source | peptidylprolyl isomerase (cyclophilin)-like 1 | 13642 | -0.045 | -0.3872 | Yes |
| 50 | RBMX | RBMX Entrez,  Source | RNA binding motif protein, X-linked | 13721 | -0.046 | -0.3821 | Yes |
| 51 | SNRPB2 | SNRPB2 Entrez,  Source | small nuclear ribonucleoprotein polypeptide B'' | 13756 | -0.046 | -0.3746 | Yes |
| 52 | SART1 | SART1 Entrez,  Source | squamous cell carcinoma antigen recognised by T cells | 13976 | -0.049 | -0.3761 | Yes |
| 53 | PLRG1 | PLRG1 Entrez,  Source | pleiotropic regulator 1 (PRL1 homolog, Arabidopsis) | 14173 | -0.051 | -0.3759 | Yes |
| 54 | U2AF2 | U2AF2 Entrez,  Source | U2 small nuclear RNA auxiliary factor 2 | 14298 | -0.053 | -0.3718 | Yes |
| 55 | DDX46 | DDX46 Entrez,  Source | DEAD (Asp-Glu-Ala-Asp) box polypeptide 46 | 14353 | -0.054 | -0.3639 | Yes |
| 56 | THOC4 | THOC4 Entrez,  Source | THO complex 4 | 14358 | -0.054 | -0.3534 | Yes |
| 57 | LSM4 | LSM4 Entrez,  Source | LSM4 homolog, U6 small nuclear RNA associated (S. cerevisiae) | 14363 | -0.054 | -0.3429 | Yes |
| 58 | NCBP1 | NCBP1 Entrez,  Source | nuclear cap binding protein subunit 1, 80kDa | 14422 | -0.054 | -0.3350 | Yes |
| 59 | LSM3 | LSM3 Entrez,  Source | LSM3 homolog, U6 small nuclear RNA associated (S. cerevisiae) | 14532 | -0.056 | -0.3295 | Yes |
| 60 | USP39 | USP39 Entrez,  Source | ubiquitin specific peptidase 39 | 14680 | -0.058 | -0.3255 | Yes |
| 61 | SNRPD2 | SNRPD2 Entrez,  Source | small nuclear ribonucleoprotein D2 polypeptide 16.5kDa | 14716 | -0.058 | -0.3156 | Yes |
| 62 | SF3A1 | SF3A1 Entrez,  Source | splicing factor 3a, subunit 1, 120kDa | 14799 | -0.060 | -0.3079 | Yes |
| 63 | PRPF8 | PRPF8 Entrez,  Source | PRP8 pre-mRNA processing factor 8 homolog (S. cerevisiae) | 14990 | -0.063 | -0.3052 | Yes |
| 64 | PPIE | PPIE Entrez,  Source | peptidylprolyl isomerase E (cyclophilin E) | 15040 | -0.063 | -0.2951 | Yes |
| 65 | LSM2 | LSM2 Entrez,  Source | LSM2 homolog, U6 small nuclear RNA associated (S. cerevisiae) | 15446 | -0.069 | -0.3021 | Yes |
| 66 | SF3B5 | SF3B5 Entrez,  Source | splicing factor 3b, subunit 5, 10kDa | 15496 | -0.070 | -0.2906 | Yes |
| 67 | SNRPE | SNRPE Entrez,  Source | small nuclear ribonucleoprotein polypeptide E | 15556 | -0.071 | -0.2794 | Yes |
| 68 | XAB2 | XAB2 Entrez,  Source | XPA binding protein 2 | 15714 | -0.074 | -0.2728 | Yes |
| 69 | SF3B3 | SF3B3 Entrez,  Source | splicing factor 3b, subunit 3, 130kDa | 15784 | -0.075 | -0.2614 | Yes |
| 70 | TXNL4A | TXNL4A Entrez,  Source | thioredoxin-like 4A | 15801 | -0.075 | -0.2471 | Yes |
| 71 | LSM7 | LSM7 Entrez,  Source | LSM7 homolog, U6 small nuclear RNA associated (S. cerevisiae) | 15910 | -0.077 | -0.2373 | Yes |
| 72 | SNRPD3 | SNRPD3 Entrez,  Source | small nuclear ribonucleoprotein D3 polypeptide 18kDa | 16046 | -0.079 | -0.2284 | Yes |
| 73 | PRPF31 | PRPF31 Entrez,  Source | PRP31 pre-mRNA processing factor 31 homolog (S. cerevisiae) | 16153 | -0.081 | -0.2176 | Yes |
| 74 | CHERP | CHERP Entrez,  Source | calcium homeostasis endoplasmic reticulum protein | 16199 | -0.082 | -0.2035 | Yes |
| 75 | ACIN1 | ACIN1 Entrez,  Source | apoptotic chromatin condensation inducer 1 | 16202 | -0.082 | -0.1872 | Yes |
| 76 | PHF5A | PHF5A Entrez,  Source | PHD finger protein 5A | 16207 | -0.082 | -0.1710 | Yes |
| 77 | SNRPB | SNRPB Entrez,  Source | small nuclear ribonucleoprotein polypeptides B and B1 | 16210 | -0.082 | -0.1547 | Yes |
| 78 | LSM6 | LSM6 Entrez,  Source | LSM6 homolog, U6 small nuclear RNA associated (S. cerevisiae) | 16216 | -0.083 | -0.1385 | Yes |
| 79 | TCERG1 | TCERG1 Entrez,  Source | transcription elongation regulator 1 | 16250 | -0.083 | -0.1235 | Yes |
| 80 | SNRPD1 | SNRPD1 Entrez,  Source | small nuclear ribonucleoprotein D1 polypeptide 16kDa | 16257 | -0.083 | -0.1072 | Yes |
| 81 | PRPF38B | PRPF38B Entrez,  Source | PRP38 pre-mRNA processing factor 38 (yeast) domain containing B | 16274 | -0.084 | -0.0913 | Yes |
| 82 | MAGOH | MAGOH Entrez,  Source | mago-nashi homolog, proliferation-associated (Drosophila) | 16409 | -0.086 | -0.0810 | Yes |
| 83 | SF3A2 | SF3A2 Entrez,  Source | splicing factor 3a, subunit 2, 66kDa | 16440 | -0.087 | -0.0652 | Yes |
| 84 | SNRPA1 | SNRPA1 Entrez,  Source | small nuclear ribonucleoprotein polypeptide A' | 16562 | -0.089 | -0.0536 | Yes |
| 85 | EFTUD2 | EFTUD2 Entrez,  Source | elongation factor Tu GTP binding domain containing 2 | 16639 | -0.091 | -0.0394 | Yes |
| 86 | THOC1 | THOC1 Entrez,  Source | THO complex 1 | 16681 | -0.092 | -0.0231 | Yes |
| 87 | SNRPF | SNRPF Entrez,  Source | small nuclear ribonucleoprotein polypeptide F | 16769 | -0.094 | -0.0089 | Yes |
| 88 | NHP2L1 | NHP2L1 Entrez,  Source | NHP2 non-histone chromosome protein 2-like 1 (S. cerevisiae) | 16888 | -0.096 | 0.0042 | Yes |
| 89 | SNRPA | SNRPA Entrez,  Source | small nuclear ribonucleoprotein polypeptide A | 17371 | -0.107 | 0.0008 | Yes |
| 90 | PRPF19 | PRPF19 Entrez,  Source | PRP19/PSO4 pre-mRNA processing factor 19 homolog (S. cerevisiae) | 18031 | -0.129 | -0.0074 | Yes |
| 91 | PRPF4 | PRPF4 Entrez,  Source | PRP4 pre-mRNA processing factor 4 homolog (yeast) | 18255 | -0.139 | 0.0089 | Yes |
| 92 | SF3A3 | SF3A3 Entrez,  Source | splicing factor 3a, subunit 3, 60kDa | 18300 | -0.141 | 0.0348 | Yes |
| 93 | PPIH | PPIH Entrez,  Source | peptidylprolyl isomerase H (cyclophilin H) | 18469 | -0.149 | 0.0558 | Yes |
Table: GSEA details [plain text format]

  

Fig 2: KEGG\_SPLICEOSOME      
 Blue-Pink O' Gram in the Space of the Analyzed GeneSet

  

Fig 3: KEGG\_SPLICEOSOME: Random ES distribution      
 Gene set null distribution of ES for **KEGG\_SPLICEOSOME**
