# Supplementary material for: Comprehensive analysis of β-catenin target genes in colorectal carcinoma cell lines with deregulated Wnt/β-catenin signaling
Source: BMC Genomics. 2014 Jan 28;15:74. doi: 10.1186/1471-2164-15-74 (PMC3909937; doi:10.1186/1471-2164-15-74)
Supplement: Additional file 5 — GSEA analysis using the KEGG pathway database. This zipped file contains confirming data of the GSEA analysis. The names of the directories containing the files were composed of the term ‘GSEA’, the name of the cell line, e.g. DLD1, SW480, or LS174T, and the pathway database (KEGG). Please use a web browser to view the files with the name ‘index.html’ in the corresponding directories to start exploring the data. [file 1471-2164-15-74-S5.zip › GSEA_KEGG_DLD1/KEGG_STEROID_HORMONE_BIOSYNTHESIS.html]

Details for gene set KEGG\_STEROID\_HORMONE\_BIOSYNTHESIS[GSEA]

|  || Dataset | DLD1\_collapsed\_to\_symbols.class.cls#b\_versus\_bg.class.cls#b\_versus\_bg\_repos |
| Phenotype | class.cls#b\_versus\_bg\_repos |
| Upregulated in class | 1 |
| GeneSet | KEGG\_STEROID\_HORMONE\_BIOSYNTHESIS |
| Enrichment Score (ES) | 0.50409967 |
| Normalized Enrichment Score (NES) | 1.6203451 |
| Nominal p-value | 0.020408163 |
| FDR q-value | 0.122985095 |
| FWER p-Value | 0.715 |
Table: GSEA Results Summary

  

Fig 1: Enrichment plot: KEGG\_STEROID\_HORMONE\_BIOSYNTHESIS      
 Profile of the Running ES Score & Positions of GeneSet Members on the Rank Ordered List

  

| PROBE | GENE SYMBOL | GENE\_TITLE | RANK IN GENE LIST | RANK METRIC SCORE | RUNNING ES | CORE ENRICHMENT || 1 | AKR1C1 | AKR1C1 Entrez,  Source | aldo-keto reductase family 1, member C1 (dihydrodiol dehydrogenase 1; 20-alpha (3-alpha)-hydroxysteroid dehydrogenase) | 24 | 0.690 | 0.1198 | Yes |
| 2 | AKR1C2 | AKR1C2 Entrez,  Source | aldo-keto reductase family 1, member C2 (dihydrodiol dehydrogenase 2; bile acid binding protein; 3-alpha hydroxysteroid dehydrogenase, type III) | 37 | 0.640 | 0.2316 | Yes |
| 3 | STS | STS Entrez,  Source | steroid sulfatase (microsomal), arylsulfatase C, isozyme S | 159 | 0.356 | 0.2880 | Yes |
| 4 | AKR1C3 | AKR1C3 Entrez,  Source | aldo-keto reductase family 1, member C3 (3-alpha hydroxysteroid dehydrogenase, type II) | 282 | 0.277 | 0.3304 | Yes |
| 5 | AKR1D1 | AKR1D1 Entrez,  Source | aldo-keto reductase family 1, member D1 (delta 4-3-ketosteroid-5-beta-reductase) | 350 | 0.246 | 0.3701 | Yes |
| 6 | CYP1B1 | CYP1B1 Entrez,  Source | cytochrome P450, family 1, subfamily B, polypeptide 1 | 501 | 0.208 | 0.3989 | Yes |
| 7 | SULT2B1 | SULT2B1 Entrez,  Source | sulfotransferase family, cytosolic, 2B, member 1 | 583 | 0.193 | 0.4285 | Yes |
| 8 | HSD17B3 | HSD17B3 Entrez,  Source | hydroxysteroid (17-beta) dehydrogenase 3 | 627 | 0.186 | 0.4589 | Yes |
| 9 | HSD17B2 | HSD17B2 Entrez,  Source | hydroxysteroid (17-beta) dehydrogenase 2 | 717 | 0.174 | 0.4849 | Yes |
| 10 | AKR1C4 | AKR1C4 Entrez,  Source | aldo-keto reductase family 1, member C4 (chlordecone reductase; 3-alpha hydroxysteroid dehydrogenase, type I; dihydrodiol dehydrogenase 4) | 886 | 0.159 | 0.5041 | Yes |
| 11 | HSD11B1 | HSD11B1 Entrez,  Source | hydroxysteroid (11-beta) dehydrogenase 1 | 1662 | 0.112 | 0.4840 | No |
| 12 | UGT2B17 | UGT2B17 Entrez,  Source | UDP glucuronosyltransferase 2 family, polypeptide B17 | 2280 | 0.091 | 0.4684 | No |
| 13 | HSD3B2 | HSD3B2 Entrez,  Source | hydroxy-delta-5-steroid dehydrogenase, 3 beta- and steroid delta-isomerase 2 | 3977 | 0.055 | 0.3911 | No |
| 14 | CYP19A1 | CYP19A1 Entrez,  Source | cytochrome P450, family 19, subfamily A, polypeptide 1 | 4644 | 0.045 | 0.3649 | No |
| 15 | HSD17B12 | HSD17B12 Entrez,  Source | hydroxysteroid (17-beta) dehydrogenase 12 | 5009 | 0.040 | 0.3532 | No |
| 16 | HSD17B7 | HSD17B7 Entrez,  Source | hydroxysteroid (17-beta) dehydrogenase 7 | 6010 | 0.028 | 0.3068 | No |
| 17 | CYP3A5 | CYP3A5 Entrez,  Source | cytochrome P450, family 3, subfamily A, polypeptide 5 | 6047 | 0.027 | 0.3097 | No |
| 18 | UGT1A9 | UGT1A9 Entrez,  Source | UDP glucuronosyltransferase 1 family, polypeptide A9 | 7185 | 0.015 | 0.2541 | No |
| 19 | CYP11A1 | CYP11A1 Entrez,  Source | cytochrome P450, family 11, subfamily A, polypeptide 1 | 8348 | 0.004 | 0.1953 | No |
| 20 | CYP17A1 | CYP17A1 Entrez,  Source | cytochrome P450, family 17, subfamily A, polypeptide 1 | 8383 | 0.004 | 0.1942 | No |
| 21 | CYP11B2 | CYP11B2 Entrez,  Source | cytochrome P450, family 11, subfamily B, polypeptide 2 | 9627 | -0.007 | 0.1318 | No |
| 22 | CYP11B1 | CYP11B1 Entrez,  Source | cytochrome P450, family 11, subfamily B, polypeptide 1 | 9716 | -0.008 | 0.1287 | No |
| 23 | HSD17B1 | HSD17B1 Entrez,  Source | hydroxysteroid (17-beta) dehydrogenase 1 | 9898 | -0.010 | 0.1211 | No |
| 24 | CYP3A4 | CYP3A4 Entrez,  Source | cytochrome P450, family 3, subfamily A, polypeptide 4 | 10040 | -0.011 | 0.1158 | No |
| 25 | COMT | COMT Entrez,  Source | catechol-O-methyltransferase | 10166 | -0.012 | 0.1115 | No |
| 26 | CYP3A7 | CYP3A7 Entrez,  Source | cytochrome P450, family 3, subfamily A, polypeptide 7 | 11079 | -0.020 | 0.0683 | No |
| 27 | UGT2B15 | UGT2B15 Entrez,  Source | UDP glucuronosyltransferase 2 family, polypeptide B15 | 11818 | -0.027 | 0.0351 | No |
| 28 | SRD5A2 | SRD5A2 Entrez,  Source | steroid-5-alpha-reductase, alpha polypeptide 2 (3-oxo-5 alpha-steroid delta 4-dehydrogenase alpha 2) | 11819 | -0.027 | 0.0398 | No |
| 29 | CYP21A2 | CYP21A2 Entrez,  Source | cytochrome P450, family 21, subfamily A, polypeptide 2 | 12357 | -0.032 | 0.0178 | No |
| 30 | CYP3A43 | CYP3A43 Entrez,  Source | cytochrome P450, family 3, subfamily A, polypeptide 43 | 13489 | -0.044 | -0.0325 | No |
| 31 | CYP7B1 | CYP7B1 Entrez,  Source | cytochrome P450, family 7, subfamily B, polypeptide 1 | 13499 | -0.044 | -0.0254 | No |
| 32 | SRD5A1 | SRD5A1 Entrez,  Source | steroid-5-alpha-reductase, alpha polypeptide 1 (3-oxo-5 alpha-steroid delta 4-dehydrogenase alpha 1) | 15209 | -0.066 | -0.1014 | No |
| 33 | UGT2B28 | UGT2B28 Entrez,  Source | UDP glucuronosyltransferase 2 family, polypeptide B28 | 15270 | -0.067 | -0.0928 | No |
| 34 | HSD17B8 | HSD17B8 Entrez,  Source | hydroxysteroid (17-beta) dehydrogenase 8 | 15424 | -0.069 | -0.0885 | No |
| 35 | SULT1E1 | SULT1E1 Entrez,  Source | sulfotransferase family 1E, estrogen-preferring, member 1 | 16143 | -0.081 | -0.1110 | No |
| 36 | UGT2A3 | UGT2A3 Entrez,  Source | UDP glucuronosyltransferase 2 family, polypeptide A3 | 16986 | -0.098 | -0.1370 | No |
| 37 | CYP7A1 | CYP7A1 Entrez,  Source | cytochrome P450, family 7, subfamily A, polypeptide 1 | 17667 | -0.117 | -0.1513 | No |
| 38 | UGT2B4 | UGT2B4 Entrez,  Source | UDP glucuronosyltransferase 2 family, polypeptide B4 | 18107 | -0.132 | -0.1506 | No |
| 39 | CYP1A1 | CYP1A1 Entrez,  Source | cytochrome P450, family 1, subfamily A, polypeptide 1 | 19122 | -0.212 | -0.1654 | No |
| 40 | HSD17B6 | HSD17B6 Entrez,  Source | hydroxysteroid (17-beta) dehydrogenase 6 | 19198 | -0.223 | -0.1300 | No |
| 41 | HSD11B2 | HSD11B2 Entrez,  Source | hydroxysteroid (11-beta) dehydrogenase 2 | 19492 | -0.388 | -0.0770 | No |
| 42 | HSD3B1 | HSD3B1 Entrez,  Source | hydroxy-delta-5-steroid dehydrogenase, 3 beta- and steroid delta-isomerase 1 | 19528 | -0.457 | 0.0014 | No |
Table: GSEA details [plain text format]

  

Fig 2: KEGG\_STEROID\_HORMONE\_BIOSYNTHESIS      
 Blue-Pink O' Gram in the Space of the Analyzed GeneSet

  

Fig 3: KEGG\_STEROID\_HORMONE\_BIOSYNTHESIS: Random ES distribution      
 Gene set null distribution of ES for **KEGG\_STEROID\_HORMONE\_BIOSYNTHESIS**
