# Supplementary material for: Comprehensive analysis of β-catenin target genes in colorectal carcinoma cell lines with deregulated Wnt/β-catenin signaling
Source: BMC Genomics. 2014 Jan 28;15:74. doi: 10.1186/1471-2164-15-74 (PMC3909937; doi:10.1186/1471-2164-15-74)
Supplement: Additional file 5 — GSEA analysis using the KEGG pathway database. This zipped file contains confirming data of the GSEA analysis. The names of the directories containing the files were composed of the term ‘GSEA’, the name of the cell line, e.g. DLD1, SW480, or LS174T, and the pathway database (KEGG). Please use a web browser to view the files with the name ‘index.html’ in the corresponding directories to start exploring the data. [file 1471-2164-15-74-S5.zip › GSEA_KEGG_DLD1/KEGG_SYSTEMIC_LUPUS_ERYTHEMATOSUS.html]

Details for gene set KEGG\_SYSTEMIC\_LUPUS\_ERYTHEMATOSUS[GSEA]

|  || Dataset | DLD1\_collapsed\_to\_symbols.class.cls#b\_versus\_bg.class.cls#b\_versus\_bg\_repos |
| Phenotype | class.cls#b\_versus\_bg\_repos |
| Upregulated in class | 1 |
| GeneSet | KEGG\_SYSTEMIC\_LUPUS\_ERYTHEMATOSUS |
| Enrichment Score (ES) | 0.48855546 |
| Normalized Enrichment Score (NES) | 1.8498956 |
| Nominal p-value | 0.0 |
| FDR q-value | 0.17904505 |
| FWER p-Value | 0.14 |
Table: GSEA Results Summary

  

Fig 1: Enrichment plot: KEGG\_SYSTEMIC\_LUPUS\_ERYTHEMATOSUS      
 Profile of the Running ES Score & Positions of GeneSet Members on the Rank Ordered List

  

| PROBE | GENE SYMBOL | GENE\_TITLE | RANK IN GENE LIST | RANK METRIC SCORE | RUNNING ES | CORE ENRICHMENT || 1 | HIST1H2BC | HIST1H2BC Entrez,  Source | histone cluster 1, H2bc | 13 | 0.804 | 0.0875 | Yes |
| 2 | HIST1H2AC | HIST1H2AC Entrez,  Source | histone cluster 1, H2ac | 74 | 0.476 | 0.1366 | Yes |
| 3 | HIST1H4H | HIST1H4H Entrez,  Source | histone cluster 1, H4h | 146 | 0.362 | 0.1727 | Yes |
| 4 | HIST2H2BE | HIST2H2BE Entrez,  Source | histone cluster 2, H2be | 155 | 0.357 | 0.2115 | Yes |
| 5 | HIST1H3H | HIST1H3H Entrez,  Source | histone cluster 1, H3h | 275 | 0.282 | 0.2362 | Yes |
| 6 | HIST1H2BD | HIST1H2BD Entrez,  Source | histone cluster 1, H2bd | 305 | 0.268 | 0.2641 | Yes |
| 7 | HIST1H2BI | HIST1H2BI Entrez,  Source | histone cluster 1, H2bi | 408 | 0.229 | 0.2840 | Yes |
| 8 | HIST1H2AL | HIST1H2AL Entrez,  Source | histone cluster 1, H2al | 422 | 0.225 | 0.3081 | Yes |
| 9 | HIST1H2AE | HIST1H2AE Entrez,  Source | histone cluster 1, H2ae | 475 | 0.214 | 0.3289 | Yes |
| 10 | HIST1H2BH | HIST1H2BH Entrez,  Source | histone cluster 1, H2bh | 480 | 0.213 | 0.3520 | Yes |
| 11 | HIST1H3E | HIST1H3E Entrez,  Source | histone cluster 1, H3e | 492 | 0.210 | 0.3744 | Yes |
| 12 | HIST1H2AG | HIST1H2AG Entrez,  Source | histone cluster 1, H2ag | 523 | 0.204 | 0.3953 | Yes |
| 13 | HIST1H2BE | HIST1H2BE Entrez,  Source | histone cluster 1, H2be | 562 | 0.195 | 0.4147 | Yes |
| 14 | H2AFJ | H2AFJ Entrez,  Source | H2A histone family, member J | 592 | 0.192 | 0.4343 | Yes |
| 15 | HIST1H2BK | HIST1H2BK Entrez,  Source | histone cluster 1, H2bk | 628 | 0.186 | 0.4528 | Yes |
| 16 | HIST1H2BF | HIST1H2BF Entrez,  Source | histone cluster 1, H2bf | 772 | 0.169 | 0.4640 | Yes |
| 17 | HIST1H2BB | HIST1H2BB Entrez,  Source | histone cluster 1, H2bb | 929 | 0.154 | 0.4729 | Yes |
| 18 | HIST1H2BJ | HIST1H2BJ Entrez,  Source | histone cluster 1, H2bj | 1047 | 0.146 | 0.4829 | Yes |
| 19 | C9 | C9 Entrez,  Source | complement component 9 | 1222 | 0.133 | 0.4886 | Yes |
| 20 | HIST1H2AJ | HIST1H2AJ Entrez,  Source | histone cluster 1, H2aj | 2113 | 0.096 | 0.4533 | No |
| 21 | HIST1H2BA | HIST1H2BA Entrez,  Source | histone cluster 1, H2ba | 2136 | 0.095 | 0.4626 | No |
| 22 | HIST1H2BO | HIST1H2BO Entrez,  Source | histone cluster 1, H2bo | 2258 | 0.091 | 0.4664 | No |
| 23 | HIST1H4D | HIST1H4D Entrez,  Source | histone cluster 1, H4d | 2642 | 0.082 | 0.4557 | No |
| 24 | C6 | C6 Entrez,  Source | complement component 6 | 2685 | 0.081 | 0.4623 | No |
| 25 | HIST1H2BG | HIST1H2BG Entrez,  Source | histone cluster 1, H2bg | 2855 | 0.076 | 0.4620 | No |
| 26 | HIST1H2AI | HIST1H2AI Entrez,  Source | histone cluster 1, H2ai | 3072 | 0.072 | 0.4588 | No |
| 27 | C1QC | C1QC Entrez,  Source | complement component 1, q subcomponent, C chain | 3074 | 0.072 | 0.4666 | No |
| 28 | HIST1H2AB | HIST1H2AB Entrez,  Source | histone cluster 1, H2ab | 3081 | 0.072 | 0.4742 | No |
| 29 | C1QA | C1QA Entrez,  Source | complement component 1, q subcomponent, A chain | 3286 | 0.067 | 0.4710 | No |
| 30 | HIST1H2AM | HIST1H2AM Entrez,  Source | histone cluster 1, H2am | 3412 | 0.065 | 0.4717 | No |
| 31 | HIST2H2AA3 | HIST2H2AA3 Entrez,  Source | histone cluster 2, H2aa3 | 3839 | 0.057 | 0.4560 | No |
| 32 | FCGR2A | FCGR2A Entrez,  Source | Fc fragment of IgG, low affinity IIa, receptor (CD32) | 3914 | 0.056 | 0.4583 | No |
| 33 | HIST1H3C | HIST1H3C Entrez,  Source | histone cluster 1, H3c | 3919 | 0.056 | 0.4643 | No |
| 34 | HIST1H3G | HIST1H3G Entrez,  Source | histone cluster 1, H3g | 4216 | 0.051 | 0.4546 | No |
| 35 | HIST1H4L | HIST1H4L Entrez,  Source | histone cluster 1, H4l | 4329 | 0.049 | 0.4543 | No |
| 36 | HIST1H4F | HIST1H4F Entrez,  Source | histone cluster 1, H4f | 4473 | 0.047 | 0.4522 | No |
| 37 | TRIM21 | TRIM21 Entrez,  Source | tripartite motif-containing 21 | 4520 | 0.047 | 0.4549 | No |
| 38 | C8G | C8G Entrez,  Source | complement component 8, gamma polypeptide | 4764 | 0.043 | 0.4472 | No |
| 39 | HLA-DRA | HLA-DRA Entrez,  Source | major histocompatibility complex, class II, DR alpha | 4768 | 0.043 | 0.4517 | No |
| 40 | HIST1H2BL | HIST1H2BL Entrez,  Source | histone cluster 1, H2bl | 4794 | 0.043 | 0.4552 | No |
| 41 | HIST1H2BM | HIST1H2BM Entrez,  Source | histone cluster 1, H2bm | 4931 | 0.041 | 0.4526 | No |
| 42 | HLA-DMB | HLA-DMB Entrez,  Source | major histocompatibility complex, class II, DM beta | 5031 | 0.039 | 0.4519 | No |
| 43 | HIST1H2BN | HIST1H2BN Entrez,  Source | histone cluster 1, H2bn | 5043 | 0.039 | 0.4556 | No |
| 44 | HLA-DRB1 | HLA-DRB1 Entrez,  Source | major histocompatibility complex, class II, DR beta 1 | 5078 | 0.039 | 0.4581 | No |
| 45 | ACTN4 | ACTN4 Entrez,  Source | actinin, alpha 4 | 5199 | 0.037 | 0.4560 | No |
| 46 | HIST1H3F | HIST1H3F Entrez,  Source | histone cluster 1, H3f | 5483 | 0.034 | 0.4452 | No |
| 47 | HIST1H3A | HIST1H3A Entrez,  Source | histone cluster 1, H3a | 5839 | 0.029 | 0.4302 | No |
| 48 | TROVE2 | TROVE2 Entrez,  Source | TROVE domain family, member 2 | 6728 | 0.020 | 0.3867 | No |
| 49 | FCGR2B | FCGR2B Entrez,  Source | Fc fragment of IgG, low affinity IIb, receptor (CD32) | 6970 | 0.018 | 0.3763 | No |
| 50 | HLA-DOB | HLA-DOB Entrez,  Source | major histocompatibility complex, class II, DO beta | 6991 | 0.017 | 0.3771 | No |
| 51 | FCGR2C | FCGR2C Entrez,  Source | Fc fragment of IgG, low affinity IIb, receptor for (CD32) | 7119 | 0.016 | 0.3724 | No |
| 52 | ACTN3 | ACTN3 Entrez,  Source | actinin, alpha 3 | 7258 | 0.015 | 0.3669 | No |
| 53 | HLA-DPB1 | HLA-DPB1 Entrez,  Source | major histocompatibility complex, class II, DP beta 1 | 7263 | 0.015 | 0.3683 | No |
| 54 | HLA-DQA1 | HLA-DQA1 Entrez,  Source | major histocompatibility complex, class II, DQ alpha 1 | 7580 | 0.011 | 0.3533 | No |
| 55 | TNF | TNF Entrez,  Source | tumor necrosis factor (TNF superfamily, member 2) | 7748 | 0.010 | 0.3457 | No |
| 56 | CD80 | CD80 Entrez,  Source | CD80 molecule | 7751 | 0.009 | 0.3467 | No |
| 57 | HLA-DMA | HLA-DMA Entrez,  Source | major histocompatibility complex, class II, DM alpha | 7752 | 0.009 | 0.3477 | No |
| 58 | HIST3H3 | HIST3H3 Entrez,  Source | histone cluster 3, H3 | 8216 | 0.005 | 0.3245 | No |
| 59 | C1QB | C1QB Entrez,  Source | complement component 1, q subcomponent, B chain | 8368 | 0.004 | 0.3171 | No |
| 60 | H3F3B | H3F3B Entrez,  Source | H3 histone, family 3B (H3.3B) | 8424 | 0.003 | 0.3147 | No |
| 61 | GRIN2A | GRIN2A Entrez,  Source | glutamate receptor, ionotropic, N-methyl D-aspartate 2A | 8446 | 0.003 | 0.3139 | No |
| 62 | HLA-DPA1 | HLA-DPA1 Entrez,  Source | major histocompatibility complex, class II, DP alpha 1 | 8607 | 0.002 | 0.3059 | No |
| 63 | HLA-DOA | HLA-DOA Entrez,  Source | major histocompatibility complex, class II, DO alpha | 8753 | 0.000 | 0.2985 | No |
| 64 | H2AFX | H2AFX Entrez,  Source | H2A histone family, member X | 8789 | -0.000 | 0.2967 | No |
| 65 | C8A | C8A Entrez,  Source | complement component 8, alpha polypeptide | 9077 | -0.002 | 0.2822 | No |
| 66 | HIST1H2AK | HIST1H2AK Entrez,  Source | histone cluster 1, H2ak | 9232 | -0.004 | 0.2747 | No |
| 67 | H2AFV | H2AFV Entrez,  Source | H2A histone family, member V | 9443 | -0.006 | 0.2645 | No |
| 68 | H2AFY | H2AFY Entrez,  Source | H2A histone family, member Y | 9720 | -0.008 | 0.2512 | No |
| 69 | HLA-DRB4 | HLA-DRB4 Entrez,  Source | major histocompatibility complex, class II, DR beta 4 | 9873 | -0.010 | 0.2445 | No |
| 70 | FCGR1A | FCGR1A Entrez,  Source | Fc fragment of IgG, high affinity Ia, receptor (CD64) | 10104 | -0.011 | 0.2339 | No |
| 71 | ACTN2 | ACTN2 Entrez,  Source | actinin, alpha 2 | 10220 | -0.012 | 0.2293 | No |
| 72 | CD40 | CD40 Entrez,  Source | CD40 molecule, TNF receptor superfamily member 5 | 10227 | -0.012 | 0.2304 | No |
| 73 | H2BFM | H2BFM Entrez,  Source | H2B histone family, member M | 10531 | -0.015 | 0.2165 | No |
| 74 | HIST1H4E | HIST1H4E Entrez,  Source | histone cluster 1, H4e | 10635 | -0.016 | 0.2130 | No |
| 75 | HIST1H4G | HIST1H4G Entrez,  Source | histone cluster 1, H4g | 10877 | -0.018 | 0.2026 | No |
| 76 | C8B | C8B Entrez,  Source | complement component 8, beta polypeptide | 11062 | -0.020 | 0.1953 | No |
| 77 | CD86 | CD86 Entrez,  Source | CD86 molecule | 11218 | -0.021 | 0.1896 | No |
| 78 | HIST1H4B | HIST1H4B Entrez,  Source | histone cluster 1, H4b | 11696 | -0.025 | 0.1679 | No |
| 79 | H2AFZ | H2AFZ Entrez,  Source | H2A histone family, member Z | 12023 | -0.029 | 0.1543 | No |
| 80 | CD28 | CD28 Entrez,  Source | CD28 molecule | 12517 | -0.033 | 0.1326 | No |
| 81 | CD40LG | CD40LG Entrez,  Source | CD40 ligand (TNF superfamily, member 5, hyper-IgM syndrome) | 13578 | -0.044 | 0.0829 | No |
| 82 | HLA-DQB1 | HLA-DQB1 Entrez,  Source | major histocompatibility complex, class II, DQ beta 1 | 13990 | -0.049 | 0.0672 | No |
| 83 | ACTN1 | ACTN1 Entrez,  Source | actinin, alpha 1 | 14062 | -0.050 | 0.0690 | No |
| 84 | IL10 | IL10 Entrez,  Source | interleukin 10 | 14107 | -0.051 | 0.0723 | No |
| 85 | FCGR3B | FCGR3B Entrez,  Source | Fc fragment of IgG, low affinity IIIb, receptor (CD16b) | 14379 | -0.054 | 0.0643 | No |
| 86 | HIST1H4A | HIST1H4A Entrez,  Source | histone cluster 1, H4a | 14771 | -0.059 | 0.0507 | No |
| 87 | C5 | C5 Entrez,  Source | complement component 5 | 14926 | -0.062 | 0.0495 | No |
| 88 | HIST1H3B | HIST1H3B Entrez,  Source | histone cluster 1, H3b | 15054 | -0.063 | 0.0500 | No |
| 89 | CTSG | CTSG Entrez,  Source | cathepsin G | 15817 | -0.076 | 0.0191 | No |
| 90 | SSB | SSB Entrez,  Source | Sjogren syndrome antigen B (autoantigen La) | 15968 | -0.078 | 0.0200 | No |
| 91 | SNRPD3 | SNRPD3 Entrez,  Source | small nuclear ribonucleoprotein D3 polypeptide 18kDa | 16046 | -0.079 | 0.0247 | No |
| 92 | GRIN2B | GRIN2B Entrez,  Source | glutamate receptor, ionotropic, N-methyl D-aspartate 2B | 16201 | -0.082 | 0.0258 | No |
| 93 | SNRPB | SNRPB Entrez,  Source | small nuclear ribonucleoprotein polypeptides B and B1 | 16210 | -0.082 | 0.0344 | No |
| 94 | SNRPD1 | SNRPD1 Entrez,  Source | small nuclear ribonucleoprotein D1 polypeptide 16kDa | 16257 | -0.083 | 0.0412 | No |
| 95 | HIST3H2A | HIST3H2A Entrez,  Source | histone cluster 3, H2a | 16439 | -0.087 | 0.0415 | No |
| 96 | HIST1H3J | HIST1H3J Entrez,  Source | histone cluster 1, H3j | 17115 | -0.101 | 0.0178 | No |
| 97 | C7 | C7 Entrez,  Source | complement component 7 | 17170 | -0.102 | 0.0263 | No |
| 98 | HIST1H3I | HIST1H3I Entrez,  Source | histone cluster 1, H3i | 17202 | -0.103 | 0.0360 | No |
| 99 | C1S | C1S Entrez,  Source | complement component 1, s subcomponent | 17313 | -0.106 | 0.0420 | No |
| 100 | H2AFY2 | H2AFY2 Entrez,  Source | H2A histone family, member Y2 | 17489 | -0.110 | 0.0451 | No |
| 101 | HIST1H4C | HIST1H4C Entrez,  Source | histone cluster 1, H4c | 17536 | -0.112 | 0.0550 | No |
| 102 | IFNG | IFNG Entrez,  Source | interferon, gamma | 18985 | -0.193 | 0.0017 | No |
| 103 | C2 | C2 Entrez,  Source | complement component 2 | 19305 | -0.251 | 0.0129 | No |
Table: GSEA details [plain text format]

  

Fig 2: KEGG\_SYSTEMIC\_LUPUS\_ERYTHEMATOSUS      
 Blue-Pink O' Gram in the Space of the Analyzed GeneSet

  

Fig 3: KEGG\_SYSTEMIC\_LUPUS\_ERYTHEMATOSUS: Random ES distribution      
 Gene set null distribution of ES for **KEGG\_SYSTEMIC\_LUPUS\_ERYTHEMATOSUS**
