# Supplementary material for: Comprehensive analysis of β-catenin target genes in colorectal carcinoma cell lines with deregulated Wnt/β-catenin signaling
Source: BMC Genomics. 2014 Jan 28;15:74. doi: 10.1186/1471-2164-15-74 (PMC3909937; doi:10.1186/1471-2164-15-74)
Supplement: Additional file 5 — GSEA analysis using the KEGG pathway database. This zipped file contains confirming data of the GSEA analysis. The names of the directories containing the files were composed of the term ‘GSEA’, the name of the cell line, e.g. DLD1, SW480, or LS174T, and the pathway database (KEGG). Please use a web browser to view the files with the name ‘index.html’ in the corresponding directories to start exploring the data. [file 1471-2164-15-74-S5.zip › GSEA_KEGG_DLD1/KEGG_TERPENOID_BACKBONE_BIOSYNTHESIS.html]

Details for gene set KEGG\_TERPENOID\_BACKBONE\_BIOSYNTHESIS[GSEA]

|  || Dataset | DLD1\_collapsed\_to\_symbols.class.cls#b\_versus\_bg.class.cls#b\_versus\_bg\_repos |
| Phenotype | class.cls#b\_versus\_bg\_repos |
| Upregulated in class | 1 |
| GeneSet | KEGG\_TERPENOID\_BACKBONE\_BIOSYNTHESIS |
| Enrichment Score (ES) | 0.58832556 |
| Normalized Enrichment Score (NES) | 1.5115764 |
| Nominal p-value | 0.064989515 |
| FDR q-value | 0.18124922 |
| FWER p-Value | 0.926 |
Table: GSEA Results Summary

  

Fig 1: Enrichment plot: KEGG\_TERPENOID\_BACKBONE\_BIOSYNTHESIS      
 Profile of the Running ES Score & Positions of GeneSet Members on the Rank Ordered List

  

| PROBE | GENE SYMBOL | GENE\_TITLE | RANK IN GENE LIST | RANK METRIC SCORE | RUNNING ES | CORE ENRICHMENT || 1 | HMGCS2 | HMGCS2 Entrez,  Source | 3-hydroxy-3-methylglutaryl-Coenzyme A synthase 2 (mitochondrial) | 4 | 1.159 | 0.4947 | Yes |
| 2 | MVD | MVD Entrez,  Source | mevalonate (diphospho) decarboxylase | 969 | 0.152 | 0.5102 | Yes |
| 3 | HMGCS1 | HMGCS1 Entrez,  Source | 3-hydroxy-3-methylglutaryl-Coenzyme A synthase 1 (soluble) | 1290 | 0.129 | 0.5489 | Yes |
| 4 | PDSS2 | PDSS2 Entrez,  Source | prenyl (decaprenyl) diphosphate synthase, subunit 2 | 2114 | 0.096 | 0.5477 | Yes |
| 5 | IDI1 | IDI1 Entrez,  Source | isopentenyl-diphosphate delta isomerase 1 | 2230 | 0.092 | 0.5812 | Yes |
| 6 | HMGCR | HMGCR Entrez,  Source | 3-hydroxy-3-methylglutaryl-Coenzyme A reductase | 2749 | 0.079 | 0.5883 | Yes |
| 7 | DHDDS | DHDDS Entrez,  Source | dehydrodolichyl diphosphate synthase | 4964 | 0.040 | 0.4922 | No |
| 8 | ACAT2 | ACAT2 Entrez,  Source | acetyl-Coenzyme A acetyltransferase 2 (acetoacetyl Coenzyme A thiolase) | 5304 | 0.036 | 0.4902 | No |
| 9 | FDPS | FDPS Entrez,  Source | farnesyl diphosphate synthase (farnesyl pyrophosphate synthetase, dimethylallyltranstransferase, geranyltranstransferase) | 5364 | 0.035 | 0.5022 | No |
| 10 | GGPS1 | GGPS1 Entrez,  Source | geranylgeranyl diphosphate synthase 1 | 8712 | 0.001 | 0.3312 | No |
| 11 | MVK | MVK Entrez,  Source | mevalonate kinase (mevalonic aciduria) | 11257 | -0.021 | 0.2101 | No |
| 12 | ACAT1 | ACAT1 Entrez,  Source | acetyl-Coenzyme A acetyltransferase 1 (acetoacetyl Coenzyme A thiolase) | 14314 | -0.053 | 0.0763 | No |
| 13 | IDI2 | IDI2 Entrez,  Source | isopentenyl-diphosphate delta isomerase 2 | 15969 | -0.078 | 0.0251 | No |
| 14 | PMVK | PMVK Entrez,  Source | phosphomevalonate kinase | 16114 | -0.081 | 0.0521 | No |
| 15 | PDSS1 | PDSS1 Entrez,  Source | prenyl (decaprenyl) diphosphate synthase, subunit 1 | 19392 | -0.290 | 0.0084 | No |
Table: GSEA details [plain text format]

  

Fig 2: KEGG\_TERPENOID\_BACKBONE\_BIOSYNTHESIS      
 Blue-Pink O' Gram in the Space of the Analyzed GeneSet

  

Fig 3: KEGG\_TERPENOID\_BACKBONE\_BIOSYNTHESIS: Random ES distribution      
 Gene set null distribution of ES for **KEGG\_TERPENOID\_BACKBONE\_BIOSYNTHESIS**
