# Supplementary material for: Comprehensive analysis of β-catenin target genes in colorectal carcinoma cell lines with deregulated Wnt/β-catenin signaling
Source: BMC Genomics. 2014 Jan 28;15:74. doi: 10.1186/1471-2164-15-74 (PMC3909937; doi:10.1186/1471-2164-15-74)
Supplement: Additional file 5 — GSEA analysis using the KEGG pathway database. This zipped file contains confirming data of the GSEA analysis. The names of the directories containing the files were composed of the term ‘GSEA’, the name of the cell line, e.g. DLD1, SW480, or LS174T, and the pathway database (KEGG). Please use a web browser to view the files with the name ‘index.html’ in the corresponding directories to start exploring the data. [file 1471-2164-15-74-S5.zip › GSEA_KEGG_DLD1/KEGG_VALINE_LEUCINE_AND_ISOLEUCINE_DEGRADATION.html]

Details for gene set KEGG\_VALINE\_LEUCINE\_AND\_ISOLEUCINE\_DEGRADATION[GSEA]

|  || Dataset | DLD1\_collapsed\_to\_symbols.class.cls#b\_versus\_bg.class.cls#b\_versus\_bg\_repos |
| Phenotype | class.cls#b\_versus\_bg\_repos |
| Upregulated in class | 1 |
| GeneSet | KEGG\_VALINE\_LEUCINE\_AND\_ISOLEUCINE\_DEGRADATION |
| Enrichment Score (ES) | 0.4950519 |
| Normalized Enrichment Score (NES) | 1.6384082 |
| Nominal p-value | 0.014522822 |
| FDR q-value | 0.11763641 |
| FWER p-Value | 0.666 |
Table: GSEA Results Summary

  

Fig 1: Enrichment plot: KEGG\_VALINE\_LEUCINE\_AND\_ISOLEUCINE\_DEGRADATION      
 Profile of the Running ES Score & Positions of GeneSet Members on the Rank Ordered List

  

| PROBE | GENE SYMBOL | GENE\_TITLE | RANK IN GENE LIST | RANK METRIC SCORE | RUNNING ES | CORE ENRICHMENT || 1 | HMGCS2 | HMGCS2 Entrez,  Source | 3-hydroxy-3-methylglutaryl-Coenzyme A synthase 2 (mitochondrial) | 4 | 1.159 | 0.3092 | Yes |
| 2 | ABAT | ABAT Entrez,  Source | 4-aminobutyrate aminotransferase | 564 | 0.195 | 0.3326 | Yes |
| 3 | ACAA1 | ACAA1 Entrez,  Source | acetyl-Coenzyme A acyltransferase 1 (peroxisomal 3-oxoacyl-Coenzyme A thiolase) | 596 | 0.191 | 0.3819 | Yes |
| 4 | ALDH3A2 | ALDH3A2 Entrez,  Source | aldehyde dehydrogenase 3 family, member A2 | 959 | 0.152 | 0.4040 | Yes |
| 5 | ALDH6A1 | ALDH6A1 Entrez,  Source | aldehyde dehydrogenase 6 family, member A1 | 1085 | 0.143 | 0.4357 | Yes |
| 6 | ACADSB | ACADSB Entrez,  Source | acyl-Coenzyme A dehydrogenase, short/branched chain | 1228 | 0.132 | 0.4637 | Yes |
| 7 | HMGCS1 | HMGCS1 Entrez,  Source | 3-hydroxy-3-methylglutaryl-Coenzyme A synthase 1 (soluble) | 1290 | 0.129 | 0.4951 | Yes |
| 8 | ALDH9A1 | ALDH9A1 Entrez,  Source | aldehyde dehydrogenase 9 family, member A1 | 2847 | 0.077 | 0.4357 | No |
| 9 | MUT | MUT Entrez,  Source | methylmalonyl Coenzyme A mutase | 2982 | 0.074 | 0.4485 | No |
| 10 | ACAA2 | ACAA2 Entrez,  Source | acetyl-Coenzyme A acyltransferase 2 (mitochondrial 3-oxoacyl-Coenzyme A thiolase) | 2999 | 0.073 | 0.4673 | No |
| 11 | BCKDHB | BCKDHB Entrez,  Source | branched chain keto acid dehydrogenase E1, beta polypeptide (maple syrup urine disease) | 3509 | 0.063 | 0.4579 | No |
| 12 | HMGCL | HMGCL Entrez,  Source | 3-hydroxymethyl-3-methylglutaryl-Coenzyme A lyase (hydroxymethylglutaricaciduria) | 5167 | 0.038 | 0.3831 | No |
| 13 | ACAT2 | ACAT2 Entrez,  Source | acetyl-Coenzyme A acetyltransferase 2 (acetoacetyl Coenzyme A thiolase) | 5304 | 0.036 | 0.3857 | No |
| 14 | ACADS | ACADS Entrez,  Source | acyl-Coenzyme A dehydrogenase, C-2 to C-3 short chain | 6211 | 0.026 | 0.3461 | No |
| 15 | ALDH2 | ALDH2 Entrez,  Source | aldehyde dehydrogenase 2 family (mitochondrial) | 6555 | 0.022 | 0.3344 | No |
| 16 | HIBADH | HIBADH Entrez,  Source | 3-hydroxyisobutyrate dehydrogenase | 6775 | 0.020 | 0.3284 | No |
| 17 | MCCC1 | MCCC1 Entrez,  Source | methylcrotonoyl-Coenzyme A carboxylase 1 (alpha) | 6808 | 0.019 | 0.3319 | No |
| 18 | HADHB | HADHB Entrez,  Source | hydroxyacyl-Coenzyme A dehydrogenase/3-ketoacyl-Coenzyme A thiolase/enoyl-Coenzyme A hydratase (trifunctional protein), beta subunit | 7650 | 0.011 | 0.2916 | No |
| 19 | ALDH7A1 | ALDH7A1 Entrez,  Source | aldehyde dehydrogenase 7 family, member A1 | 7706 | 0.010 | 0.2914 | No |
| 20 | DBT | DBT Entrez,  Source | dihydrolipoamide branched chain transacylase E2 | 8135 | 0.006 | 0.2711 | No |
| 21 | OXCT2 | OXCT2 Entrez,  Source | 3-oxoacid CoA transferase 2 | 8745 | 0.000 | 0.2400 | No |
| 22 | EHHADH | EHHADH Entrez,  Source | enoyl-Coenzyme A, hydratase/3-hydroxyacyl Coenzyme A dehydrogenase | 9058 | -0.002 | 0.2246 | No |
| 23 | HIBCH | HIBCH Entrez,  Source | 3-hydroxyisobutyryl-Coenzyme A hydrolase | 9414 | -0.005 | 0.2078 | No |
| 24 | DLD | DLD Entrez,  Source | dihydrolipoamide dehydrogenase | 9628 | -0.007 | 0.1989 | No |
| 25 | HADHA | HADHA Entrez,  Source | hydroxyacyl-Coenzyme A dehydrogenase/3-ketoacyl-Coenzyme A thiolase/enoyl-Coenzyme A hydratase (trifunctional protein), alpha subunit | 10086 | -0.011 | 0.1785 | No |
| 26 | PCCB | PCCB Entrez,  Source | propionyl Coenzyme A carboxylase, beta polypeptide | 10311 | -0.013 | 0.1706 | No |
| 27 | AUH | AUH Entrez,  Source | AU RNA binding protein/enoyl-Coenzyme A hydratase | 10388 | -0.014 | 0.1705 | No |
| 28 | BCKDHA | BCKDHA Entrez,  Source | branched chain keto acid dehydrogenase E1, alpha polypeptide | 11599 | -0.025 | 0.1150 | No |
| 29 | ECHS1 | ECHS1 Entrez,  Source | enoyl Coenzyme A hydratase, short chain, 1, mitochondrial | 11609 | -0.025 | 0.1211 | No |
| 30 | BCAT1 | BCAT1 Entrez,  Source | branched chain aminotransferase 1, cytosolic | 11720 | -0.026 | 0.1223 | No |
| 31 | ACADM | ACADM Entrez,  Source | acyl-Coenzyme A dehydrogenase, C-4 to C-12 straight chain | 11735 | -0.026 | 0.1285 | No |
| 32 | OXCT1 | OXCT1 Entrez,  Source | 3-oxoacid CoA transferase 1 | 12507 | -0.033 | 0.0978 | No |
| 33 | ACAD8 | ACAD8 Entrez,  Source | acyl-Coenzyme A dehydrogenase family, member 8 | 13348 | -0.042 | 0.0659 | No |
| 34 | IVD | IVD Entrez,  Source | isovaleryl Coenzyme A dehydrogenase | 13896 | -0.048 | 0.0507 | No |
| 35 | BCAT2 | BCAT2 Entrez,  Source | branched chain aminotransferase 2, mitochondrial | 13926 | -0.048 | 0.0620 | No |
| 36 | ACAT1 | ACAT1 Entrez,  Source | acetyl-Coenzyme A acetyltransferase 1 (acetoacetyl Coenzyme A thiolase) | 14314 | -0.053 | 0.0563 | No |
| 37 | MCEE | MCEE Entrez,  Source | methylmalonyl CoA epimerase | 14946 | -0.062 | 0.0406 | No |
| 38 | MCCC2 | MCCC2 Entrez,  Source | methylcrotonoyl-Coenzyme A carboxylase 2 (beta) | 16055 | -0.080 | 0.0050 | No |
| 39 | HADH | HADH Entrez,  Source | hydroxyacyl-Coenzyme A dehydrogenase | 17614 | -0.115 | -0.0441 | No |
| 40 | AOX1 | AOX1 Entrez,  Source | aldehyde oxidase 1 | 17872 | -0.123 | -0.0243 | No |
| 41 | ALDH1B1 | ALDH1B1 Entrez,  Source | aldehyde dehydrogenase 1 family, member B1 | 19017 | -0.196 | -0.0306 | No |
| 42 | PCCA | PCCA Entrez,  Source | propionyl Coenzyme A carboxylase, alpha polypeptide | 19164 | -0.218 | 0.0201 | No |
Table: GSEA details [plain text format]

  

Fig 2: KEGG\_VALINE\_LEUCINE\_AND\_ISOLEUCINE\_DEGRADATION      
 Blue-Pink O' Gram in the Space of the Analyzed GeneSet

  

Fig 3: KEGG\_VALINE\_LEUCINE\_AND\_ISOLEUCINE\_DEGRADATION: Random ES distribution      
 Gene set null distribution of ES for **KEGG\_VALINE\_LEUCINE\_AND\_ISOLEUCINE\_DEGRADATION**
