# Supplementary material for: Comprehensive analysis of β-catenin target genes in colorectal carcinoma cell lines with deregulated Wnt/β-catenin signaling
Source: BMC Genomics. 2014 Jan 28;15:74. doi: 10.1186/1471-2164-15-74 (PMC3909937; doi:10.1186/1471-2164-15-74)
Supplement: Additional file 5 — GSEA analysis using the KEGG pathway database. This zipped file contains confirming data of the GSEA analysis. The names of the directories containing the files were composed of the term ‘GSEA’, the name of the cell line, e.g. DLD1, SW480, or LS174T, and the pathway database (KEGG). Please use a web browser to view the files with the name ‘index.html’ in the corresponding directories to start exploring the data. [file 1471-2164-15-74-S5.zip › GSEA_LS174T_KEGG/gsea_report_for_0_1358419934281.html]

Report for 0 1358419934281 [GSEA]

| GS  follow link to MSigDB | GS DETAILS | SIZE | ES | NES | NOM p-val | FDR q-val | FWER p-val | RANK AT MAX | LEADING EDGE || 1 | KEGG\_DNA\_REPLICATION | Details ... | 34 | -0.83 | -2.55 | 0.000 | 0.000 | 0.000 | 941 | tags=71%, list=5%, signal=74% |
| 2 | KEGG\_NUCLEOTIDE\_EXCISION\_REPAIR | Details ... | 43 | -0.70 | -2.33 | 0.000 | 0.000 | 0.000 | 2160 | tags=40%, list=11%, signal=44% |
| 3 | KEGG\_CELL\_CYCLE | Details ... | 113 | -0.59 | -2.32 | 0.000 | 0.000 | 0.000 | 2970 | tags=45%, list=15%, signal=53% |
| 4 | KEGG\_HOMOLOGOUS\_RECOMBINATION | Details ... | 26 | -0.78 | -2.28 | 0.000 | 0.000 | 0.000 | 2764 | tags=69%, list=14%, signal=81% |
| 5 | KEGG\_MISMATCH\_REPAIR | Details ... | 22 | -0.77 | -2.17 | 0.000 | 0.000 | 0.001 | 2160 | tags=68%, list=11%, signal=77% |
| 6 | KEGG\_PYRIMIDINE\_METABOLISM | Details ... | 88 | -0.54 | -2.00 | 0.000 | 0.001 | 0.009 | 3146 | tags=40%, list=16%, signal=47% |
| 7 | KEGG\_BASE\_EXCISION\_REPAIR | Details ... | 33 | -0.60 | -1.89 | 0.000 | 0.005 | 0.039 | 2214 | tags=42%, list=11%, signal=48% |
| 8 | KEGG\_OOCYTE\_MEIOSIS | Details ... | 106 | -0.48 | -1.86 | 0.000 | 0.007 | 0.063 | 1672 | tags=22%, list=9%, signal=24% |
| 9 | KEGG\_SPLICEOSOME | Details ... | 93 | -0.48 | -1.85 | 0.000 | 0.007 | 0.067 | 4859 | tags=47%, list=25%, signal=63% |
| 10 | KEGG\_PURINE\_METABOLISM | Details ... | 149 | -0.40 | -1.64 | 0.000 | 0.058 | 0.459 | 3002 | tags=28%, list=15%, signal=32% |
| 11 | KEGG\_P53\_SIGNALING\_PATHWAY | Details ... | 64 | -0.44 | -1.56 | 0.009 | 0.107 | 0.730 | 2944 | tags=31%, list=15%, signal=37% |
| 12 | KEGG\_PANTOTHENATE\_AND\_COA\_BIOSYNTHESIS | Details ... | 16 | -0.60 | -1.55 | 0.051 | 0.110 | 0.762 | 2643 | tags=44%, list=14%, signal=51% |
| 13 | KEGG\_RNA\_DEGRADATION | Details ... | 51 | -0.45 | -1.53 | 0.024 | 0.119 | 0.804 | 2583 | tags=27%, list=13%, signal=32% |
| 14 | KEGG\_RNA\_POLYMERASE | Details ... | 28 | -0.50 | -1.47 | 0.046 | 0.163 | 0.909 | 6468 | tags=61%, list=33%, signal=91% |
| 15 | KEGG\_COLORECTAL\_CANCER | Details ... | 61 | -0.41 | -1.46 | 0.014 | 0.171 | 0.928 | 1903 | tags=20%, list=10%, signal=22% |
| 16 | KEGG\_GLYCOSAMINOGLYCAN\_BIOSYNTHESIS\_HEPARAN\_SULFATE | Details ... | 25 | -0.49 | -1.44 | 0.076 | 0.179 | 0.953 | 2455 | tags=36%, list=13%, signal=41% |
| 17 | KEGG\_THYROID\_CANCER | Details ... | 29 | -0.46 | -1.41 | 0.060 | 0.215 | 0.974 | 1542 | tags=17%, list=8%, signal=19% |
| 18 | KEGG\_PROSTATE\_CANCER | Details ... | 87 | -0.38 | -1.40 | 0.035 | 0.207 | 0.976 | 1374 | tags=14%, list=7%, signal=15% |
| 19 | KEGG\_PROGESTERONE\_MEDIATED\_OOCYTE\_MATURATION | Details ... | 82 | -0.38 | -1.40 | 0.035 | 0.199 | 0.978 | 1903 | tags=18%, list=10%, signal=20% |
| 20 | KEGG\_DORSO\_VENTRAL\_AXIS\_FORMATION | Details ... | 23 | -0.49 | -1.37 | 0.079 | 0.233 | 0.990 | 1842 | tags=22%, list=9%, signal=24% |
| 21 | KEGG\_BASAL\_CELL\_CARCINOMA |  | 53 | -0.40 | -1.36 | 0.050 | 0.241 | 0.996 | 959 | tags=15%, list=5%, signal=16% |
| 22 | KEGG\_LYSINE\_DEGRADATION |  | 43 | -0.40 | -1.35 | 0.086 | 0.235 | 0.997 | 2242 | tags=21%, list=11%, signal=24% |
| 23 | KEGG\_ENDOMETRIAL\_CANCER |  | 51 | -0.38 | -1.29 | 0.112 | 0.341 | 0.999 | 1542 | tags=16%, list=8%, signal=17% |
| 24 | KEGG\_PROXIMAL\_TUBULE\_BICARBONATE\_RECLAMATION |  | 23 | -0.44 | -1.27 | 0.152 | 0.355 | 0.999 | 3394 | tags=30%, list=17%, signal=37% |
| 25 | KEGG\_WNT\_SIGNALING\_PATHWAY |  | 144 | -0.30 | -1.21 | 0.125 | 0.493 | 1.000 | 4684 | tags=31%, list=24%, signal=41% |
| 26 | KEGG\_BLADDER\_CANCER |  | 40 | -0.37 | -1.19 | 0.202 | 0.538 | 1.000 | 2766 | tags=25%, list=14%, signal=29% |
| 27 | KEGG\_ADHERENS\_JUNCTION |  | 73 | -0.32 | -1.18 | 0.186 | 0.534 | 1.000 | 955 | tags=8%, list=5%, signal=9% |
| 28 | KEGG\_PARKINSONS\_DISEASE |  | 105 | -0.29 | -1.14 | 0.201 | 0.629 | 1.000 | 7667 | tags=46%, list=39%, signal=75% |
| 29 | KEGG\_TGF\_BETA\_SIGNALING\_PATHWAY |  | 82 | -0.31 | -1.14 | 0.215 | 0.620 | 1.000 | 2970 | tags=22%, list=15%, signal=26% |
| 30 | KEGG\_AMINOACYL\_TRNA\_BIOSYNTHESIS |  | 32 | -0.37 | -1.13 | 0.249 | 0.603 | 1.000 | 5805 | tags=47%, list=30%, signal=67% |
| 31 | KEGG\_GLYCOSYLPHOSPHATIDYLINOSITOL\_GPI\_ANCHOR\_BIOSYNTHESIS |  | 24 | -0.38 | -1.10 | 0.323 | 0.679 | 1.000 | 6584 | tags=54%, list=34%, signal=82% |
| 32 | KEGG\_CITRATE\_CYCLE\_TCA\_CYCLE |  | 29 | -0.35 | -1.07 | 0.352 | 0.776 | 1.000 | 5303 | tags=38%, list=27%, signal=52% |
| 33 | KEGG\_UBIQUITIN\_MEDIATED\_PROTEOLYSIS |  | 121 | -0.27 | -1.07 | 0.290 | 0.752 | 1.000 | 4713 | tags=25%, list=24%, signal=32% |
| 34 | KEGG\_PANCREATIC\_CANCER |  | 69 | -0.28 | -1.04 | 0.413 | 0.821 | 1.000 | 2944 | tags=20%, list=15%, signal=24% |
| 35 | KEGG\_ONE\_CARBON\_POOL\_BY\_FOLATE |  | 16 | -0.39 | -1.03 | 0.429 | 0.827 | 1.000 | 2069 | tags=25%, list=11%, signal=28% |
| 36 | KEGG\_PROTEASOME |  | 42 | -0.31 | -1.02 | 0.414 | 0.828 | 1.000 | 8262 | tags=60%, list=42%, signal=103% |
| 37 | KEGG\_GAP\_JUNCTION |  | 75 | -0.27 | -1.01 | 0.433 | 0.863 | 1.000 | 3216 | tags=24%, list=16%, signal=29% |
| 38 | KEGG\_ARGININE\_AND\_PROLINE\_METABOLISM |  | 51 | -0.29 | -1.01 | 0.452 | 0.841 | 1.000 | 2363 | tags=22%, list=12%, signal=24% |
| 39 | KEGG\_N\_GLYCAN\_BIOSYNTHESIS |  | 41 | -0.30 | -0.99 | 0.452 | 0.875 | 1.000 | 3824 | tags=24%, list=20%, signal=30% |
| 40 | KEGG\_LONG\_TERM\_DEPRESSION |  | 65 | -0.28 | -0.99 | 0.484 | 0.871 | 1.000 | 3037 | tags=23%, list=16%, signal=27% |
| 41 | KEGG\_NOTCH\_SIGNALING\_PATHWAY |  | 41 | -0.30 | -0.97 | 0.506 | 0.908 | 1.000 | 3959 | tags=32%, list=20%, signal=40% |
| 42 | KEGG\_TIGHT\_JUNCTION |  | 124 | -0.24 | -0.96 | 0.559 | 0.906 | 1.000 | 1275 | tags=10%, list=7%, signal=11% |
| 43 | KEGG\_MELANOMA |  | 71 | -0.27 | -0.96 | 0.532 | 0.901 | 1.000 | 4423 | tags=30%, list=23%, signal=38% |
| 44 | KEGG\_PATHWAYS\_IN\_CANCER |  | 315 | -0.22 | -0.95 | 0.612 | 0.896 | 1.000 | 1571 | tags=11%, list=8%, signal=12% |
| 45 | KEGG\_TASTE\_TRANSDUCTION |  | 44 | -0.29 | -0.95 | 0.520 | 0.893 | 1.000 | 3076 | tags=23%, list=16%, signal=27% |
| 46 | KEGG\_HUNTINGTONS\_DISEASE |  | 163 | -0.23 | -0.94 | 0.640 | 0.909 | 1.000 | 6529 | tags=36%, list=33%, signal=54% |
| 47 | KEGG\_CHRONIC\_MYELOID\_LEUKEMIA |  | 72 | -0.26 | -0.91 | 0.635 | 0.964 | 1.000 | 4423 | tags=28%, list=23%, signal=36% |
| 48 | KEGG\_VIRAL\_MYOCARDITIS |  | 67 | -0.24 | -0.88 | 0.700 | 1.000 | 1.000 | 1699 | tags=12%, list=9%, signal=13% |
| 49 | KEGG\_VASCULAR\_SMOOTH\_MUSCLE\_CONTRACTION |  | 108 | -0.23 | -0.88 | 0.755 | 1.000 | 1.000 | 3228 | tags=19%, list=17%, signal=23% |
| 50 | KEGG\_MELANOGENESIS |  | 98 | -0.22 | -0.86 | 0.784 | 1.000 | 1.000 | 1543 | tags=10%, list=8%, signal=11% |
| 51 | KEGG\_FC\_EPSILON\_RI\_SIGNALING\_PATHWAY |  | 73 | -0.23 | -0.85 | 0.771 | 1.000 | 1.000 | 4838 | tags=32%, list=25%, signal=42% |
| 52 | KEGG\_NICOTINATE\_AND\_NICOTINAMIDE\_METABOLISM |  | 21 | -0.30 | -0.84 | 0.683 | 1.000 | 1.000 | 1590 | tags=14%, list=8%, signal=16% |
| 53 | KEGG\_CYSTEINE\_AND\_METHIONINE\_METABOLISM |  | 30 | -0.27 | -0.83 | 0.715 | 1.000 | 1.000 | 5023 | tags=33%, list=26%, signal=45% |
| 54 | KEGG\_GLIOMA |  | 63 | -0.23 | -0.82 | 0.816 | 1.000 | 1.000 | 4741 | tags=30%, list=24%, signal=40% |
| 55 | KEGG\_PATHOGENIC\_ESCHERICHIA\_COLI\_INFECTION |  | 42 | -0.25 | -0.82 | 0.793 | 1.000 | 1.000 | 5 | tags=2%, list=0%, signal=2% |
| 56 | KEGG\_LONG\_TERM\_POTENTIATION |  | 67 | -0.22 | -0.81 | 0.836 | 1.000 | 1.000 | 3334 | tags=19%, list=17%, signal=23% |
| 57 | KEGG\_PRIMARY\_BILE\_ACID\_BIOSYNTHESIS |  | 16 | -0.31 | -0.81 | 0.724 | 1.000 | 1.000 | 1462 | tags=19%, list=7%, signal=20% |
| 58 | KEGG\_AXON\_GUIDANCE |  | 127 | -0.20 | -0.80 | 0.911 | 1.000 | 1.000 | 2088 | tags=12%, list=11%, signal=13% |
| 59 | KEGG\_HEDGEHOG\_SIGNALING\_PATHWAY |  | 53 | -0.23 | -0.80 | 0.823 | 1.000 | 1.000 | 3455 | tags=23%, list=18%, signal=27% |
| 60 | KEGG\_GNRH\_SIGNALING\_PATHWAY |  | 96 | -0.21 | -0.80 | 0.904 | 1.000 | 1.000 | 3228 | tags=20%, list=17%, signal=24% |
| 61 | KEGG\_GLYOXYLATE\_AND\_DICARBOXYLATE\_METABOLISM |  | 15 | -0.31 | -0.80 | 0.736 | 0.992 | 1.000 | 2069 | tags=20%, list=11%, signal=22% |
| 62 | KEGG\_APOPTOSIS |  | 81 | -0.21 | -0.79 | 0.854 | 0.982 | 1.000 | 4112 | tags=30%, list=21%, signal=37% |
| 63 | KEGG\_PRIMARY\_IMMUNODEFICIENCY |  | 35 | -0.25 | -0.79 | 0.797 | 0.971 | 1.000 | 2473 | tags=23%, list=13%, signal=26% |
| 64 | KEGG\_NON\_SMALL\_CELL\_LUNG\_CANCER |  | 52 | -0.22 | -0.77 | 0.889 | 0.991 | 1.000 | 2944 | tags=17%, list=15%, signal=20% |
| 65 | KEGG\_ACUTE\_MYELOID\_LEUKEMIA |  | 56 | -0.21 | -0.74 | 0.914 | 1.000 | 1.000 | 2968 | tags=20%, list=15%, signal=23% |
| 66 | KEGG\_BASAL\_TRANSCRIPTION\_FACTORS |  | 33 | -0.24 | -0.73 | 0.891 | 1.000 | 1.000 | 4392 | tags=27%, list=22%, signal=35% |
| 67 | KEGG\_PROTEIN\_EXPORT |  | 19 | -0.27 | -0.73 | 0.846 | 0.996 | 1.000 | 758 | tags=5%, list=4%, signal=5% |
| 68 | KEGG\_PENTOSE\_PHOSPHATE\_PATHWAY |  | 26 | -0.24 | -0.72 | 0.869 | 0.991 | 1.000 | 5894 | tags=46%, list=30%, signal=66% |
| 69 | KEGG\_PYRUVATE\_METABOLISM |  | 40 | -0.22 | -0.70 | 0.900 | 0.990 | 1.000 | 3501 | tags=20%, list=18%, signal=24% |
| 70 | KEGG\_T\_CELL\_RECEPTOR\_SIGNALING\_PATHWAY |  | 106 | -0.18 | -0.69 | 1.000 | 0.986 | 1.000 | 4996 | tags=26%, list=26%, signal=35% |
| 71 | KEGG\_STEROID\_BIOSYNTHESIS |  | 16 | -0.24 | -0.64 | 0.920 | 0.998 | 1.000 | 474 | tags=6%, list=2%, signal=6% |
| 72 | KEGG\_ALZHEIMERS\_DISEASE |  | 148 | -0.15 | -0.62 | 1.000 | 0.989 | 1.000 | 5744 | tags=24%, list=29%, signal=33% |
| 73 | KEGG\_MTOR\_SIGNALING\_PATHWAY |  | 46 | -0.18 | -0.60 | 0.991 | 0.982 | 1.000 | 1343 | tags=9%, list=7%, signal=9% |
Table: Gene sets enriched in phenotype **0 (3 samples) **[plain text format]****
