# Supplementary material for: Comprehensive analysis of β-catenin target genes in colorectal carcinoma cell lines with deregulated Wnt/β-catenin signaling
Source: BMC Genomics. 2014 Jan 28;15:74. doi: 10.1186/1471-2164-15-74 (PMC3909937; doi:10.1186/1471-2164-15-74)
Supplement: Additional file 5 — GSEA analysis using the KEGG pathway database. This zipped file contains confirming data of the GSEA analysis. The names of the directories containing the files were composed of the term ‘GSEA’, the name of the cell line, e.g. DLD1, SW480, or LS174T, and the pathway database (KEGG). Please use a web browser to view the files with the name ‘index.html’ in the corresponding directories to start exploring the data. [file 1471-2164-15-74-S5.zip › GSEA_LS174T_KEGG/gsea_report_for_1_1358419934281.html]

Report for 1 1358419934281 [GSEA]

| GS  follow link to MSigDB | GS DETAILS | SIZE | ES | NES | NOM p-val | FDR q-val | FWER p-val | RANK AT MAX | LEADING EDGE || 1 | KEGG\_METABOLISM\_OF\_XENOBIOTICS\_BY\_CYTOCHROME\_P450 | Details ... | 56 | 0.65 | 2.16 | 0.000 | 0.001 | 0.001 | 1018 | tags=27%, list=5%, signal=28% |
| 2 | KEGG\_RETINOL\_METABOLISM | Details ... | 45 | 0.68 | 2.14 | 0.000 | 0.001 | 0.001 | 1790 | tags=33%, list=9%, signal=37% |
| 3 | KEGG\_DRUG\_METABOLISM\_CYTOCHROME\_P450 | Details ... | 57 | 0.62 | 2.07 | 0.000 | 0.002 | 0.005 | 1018 | tags=23%, list=5%, signal=24% |
| 4 | KEGG\_STEROID\_HORMONE\_BIOSYNTHESIS | Details ... | 42 | 0.64 | 2.01 | 0.000 | 0.002 | 0.008 | 810 | tags=24%, list=4%, signal=25% |
| 5 | KEGG\_DRUG\_METABOLISM\_OTHER\_ENZYMES | Details ... | 36 | 0.59 | 1.80 | 0.002 | 0.034 | 0.150 | 952 | tags=19%, list=5%, signal=20% |
| 6 | KEGG\_PORPHYRIN\_AND\_CHLOROPHYLL\_METABOLISM | Details ... | 28 | 0.62 | 1.79 | 0.009 | 0.032 | 0.164 | 801 | tags=14%, list=4%, signal=15% |
| 7 | KEGG\_PENTOSE\_AND\_GLUCURONATE\_INTERCONVERSIONS | Details ... | 15 | 0.71 | 1.78 | 0.015 | 0.033 | 0.195 | 70 | tags=20%, list=0%, signal=20% |
| 8 | KEGG\_LINOLEIC\_ACID\_METABOLISM | Details ... | 26 | 0.58 | 1.68 | 0.015 | 0.077 | 0.448 | 1098 | tags=19%, list=6%, signal=20% |
| 9 | KEGG\_ARACHIDONIC\_ACID\_METABOLISM | Details ... | 50 | 0.50 | 1.63 | 0.016 | 0.105 | 0.594 | 4020 | tags=44%, list=21%, signal=55% |
| 10 | KEGG\_PHENYLALANINE\_METABOLISM | Details ... | 17 | 0.63 | 1.63 | 0.011 | 0.098 | 0.612 | 2020 | tags=41%, list=10%, signal=46% |
| 11 | KEGG\_STARCH\_AND\_SUCROSE\_METABOLISM | Details ... | 34 | 0.55 | 1.62 | 0.017 | 0.099 | 0.654 | 3171 | tags=29%, list=16%, signal=35% |
| 12 | KEGG\_NOD\_LIKE\_RECEPTOR\_SIGNALING\_PATHWAY | Details ... | 51 | 0.50 | 1.61 | 0.015 | 0.098 | 0.675 | 3173 | tags=31%, list=16%, signal=37% |
| 13 | KEGG\_COMPLEMENT\_AND\_COAGULATION\_CASCADES | Details ... | 65 | 0.47 | 1.60 | 0.009 | 0.097 | 0.702 | 3702 | tags=34%, list=19%, signal=42% |
| 14 | KEGG\_HYPERTROPHIC\_CARDIOMYOPATHY\_HCM | Details ... | 82 | 0.43 | 1.54 | 0.014 | 0.155 | 0.878 | 3962 | tags=30%, list=20%, signal=38% |
| 15 | KEGG\_PPAR\_SIGNALING\_PATHWAY | Details ... | 66 | 0.44 | 1.51 | 0.037 | 0.181 | 0.925 | 4315 | tags=41%, list=22%, signal=52% |
| 16 | KEGG\_O\_GLYCAN\_BIOSYNTHESIS | Details ... | 28 | 0.52 | 1.51 | 0.049 | 0.176 | 0.932 | 2463 | tags=29%, list=13%, signal=33% |
| 17 | KEGG\_BUTANOATE\_METABOLISM | Details ... | 31 | 0.50 | 1.50 | 0.033 | 0.176 | 0.942 | 1162 | tags=19%, list=6%, signal=21% |
| 18 | KEGG\_NITROGEN\_METABOLISM | Details ... | 23 | 0.55 | 1.50 | 0.034 | 0.169 | 0.942 | 825 | tags=22%, list=4%, signal=23% |
| 19 | KEGG\_HISTIDINE\_METABOLISM | Details ... | 27 | 0.52 | 1.49 | 0.038 | 0.164 | 0.946 | 3737 | tags=44%, list=19%, signal=55% |
| 20 | KEGG\_ETHER\_LIPID\_METABOLISM | Details ... | 27 | 0.51 | 1.47 | 0.056 | 0.180 | 0.965 | 2533 | tags=33%, list=13%, signal=38% |
| 21 | KEGG\_ABC\_TRANSPORTERS |  | 44 | 0.47 | 1.47 | 0.034 | 0.178 | 0.969 | 3355 | tags=32%, list=17%, signal=38% |
| 22 | KEGG\_GLYCOSAMINOGLYCAN\_DEGRADATION |  | 21 | 0.54 | 1.44 | 0.069 | 0.212 | 0.990 | 5462 | tags=52%, list=28%, signal=73% |
| 23 | KEGG\_GLYCOSAMINOGLYCAN\_BIOSYNTHESIS\_KERATAN\_SULFATE |  | 15 | 0.57 | 1.40 | 0.104 | 0.265 | 0.999 | 5968 | tags=53%, list=31%, signal=77% |
| 24 | KEGG\_TYROSINE\_METABOLISM |  | 38 | 0.43 | 1.32 | 0.103 | 0.415 | 1.000 | 3737 | tags=37%, list=19%, signal=45% |
| 25 | KEGG\_ENDOCYTOSIS |  | 156 | 0.33 | 1.30 | 0.055 | 0.447 | 1.000 | 3662 | tags=26%, list=19%, signal=31% |
| 26 | KEGG\_ECM\_RECEPTOR\_INTERACTION |  | 80 | 0.36 | 1.29 | 0.099 | 0.450 | 1.000 | 3135 | tags=25%, list=16%, signal=30% |
| 27 | KEGG\_DILATED\_CARDIOMYOPATHY |  | 89 | 0.36 | 1.29 | 0.087 | 0.439 | 1.000 | 4423 | tags=29%, list=23%, signal=38% |
| 28 | KEGG\_FRUCTOSE\_AND\_MANNOSE\_METABOLISM |  | 34 | 0.43 | 1.28 | 0.155 | 0.437 | 1.000 | 2829 | tags=26%, list=14%, signal=31% |
| 29 | KEGG\_CYTOSOLIC\_DNA\_SENSING\_PATHWAY |  | 50 | 0.39 | 1.28 | 0.103 | 0.423 | 1.000 | 2762 | tags=24%, list=14%, signal=28% |
| 30 | KEGG\_LYSOSOME |  | 114 | 0.34 | 1.28 | 0.083 | 0.418 | 1.000 | 4384 | tags=31%, list=22%, signal=39% |
| 31 | KEGG\_CARDIAC\_MUSCLE\_CONTRACTION |  | 70 | 0.37 | 1.27 | 0.108 | 0.415 | 1.000 | 4725 | tags=24%, list=24%, signal=32% |
| 32 | KEGG\_VASOPRESSIN\_REGULATED\_WATER\_REABSORPTION |  | 44 | 0.40 | 1.27 | 0.143 | 0.415 | 1.000 | 4257 | tags=39%, list=22%, signal=49% |
| 33 | KEGG\_REGULATION\_OF\_ACTIN\_CYTOSKELETON |  | 196 | 0.32 | 1.27 | 0.058 | 0.403 | 1.000 | 4865 | tags=33%, list=25%, signal=43% |
| 34 | KEGG\_AMINO\_SUGAR\_AND\_NUCLEOTIDE\_SUGAR\_METABOLISM |  | 44 | 0.40 | 1.26 | 0.157 | 0.407 | 1.000 | 5272 | tags=45%, list=27%, signal=62% |
| 35 | KEGG\_MAPK\_SIGNALING\_PATHWAY |  | 253 | 0.30 | 1.25 | 0.062 | 0.419 | 1.000 | 2958 | tags=18%, list=15%, signal=21% |
| 36 | KEGG\_HEMATOPOIETIC\_CELL\_LINEAGE |  | 82 | 0.36 | 1.25 | 0.147 | 0.410 | 1.000 | 2507 | tags=15%, list=13%, signal=17% |
| 37 | KEGG\_SNARE\_INTERACTIONS\_IN\_VESICULAR\_TRANSPORT |  | 34 | 0.41 | 1.25 | 0.160 | 0.400 | 1.000 | 5041 | tags=44%, list=26%, signal=59% |
| 38 | KEGG\_GLYCOSPHINGOLIPID\_BIOSYNTHESIS\_LACTO\_AND\_NEOLACTO\_SERIES |  | 26 | 0.43 | 1.22 | 0.187 | 0.445 | 1.000 | 3620 | tags=27%, list=19%, signal=33% |
| 39 | KEGG\_SYSTEMIC\_LUPUS\_ERYTHEMATOSUS |  | 103 | 0.33 | 1.21 | 0.133 | 0.466 | 1.000 | 3302 | tags=25%, list=17%, signal=30% |
| 40 | KEGG\_SPHINGOLIPID\_METABOLISM |  | 30 | 0.41 | 1.20 | 0.212 | 0.475 | 1.000 | 4088 | tags=37%, list=21%, signal=46% |
| 41 | KEGG\_SMALL\_CELL\_LUNG\_CANCER |  | 82 | 0.33 | 1.17 | 0.213 | 0.554 | 1.000 | 3224 | tags=24%, list=16%, signal=29% |
| 42 | KEGG\_GLUTATHIONE\_METABOLISM |  | 44 | 0.36 | 1.17 | 0.240 | 0.544 | 1.000 | 4722 | tags=36%, list=24%, signal=48% |
| 43 | KEGG\_RENIN\_ANGIOTENSIN\_SYSTEM |  | 16 | 0.47 | 1.16 | 0.282 | 0.541 | 1.000 | 177 | tags=13%, list=1%, signal=13% |
| 44 | KEGG\_PEROXISOME |  | 71 | 0.34 | 1.16 | 0.231 | 0.535 | 1.000 | 4315 | tags=38%, list=22%, signal=49% |
| 45 | KEGG\_CYTOKINE\_CYTOKINE\_RECEPTOR\_INTERACTION |  | 243 | 0.28 | 1.16 | 0.159 | 0.529 | 1.000 | 2498 | tags=16%, list=13%, signal=18% |
| 46 | KEGG\_GLYCEROPHOSPHOLIPID\_METABOLISM |  | 63 | 0.34 | 1.13 | 0.249 | 0.573 | 1.000 | 2625 | tags=19%, list=13%, signal=22% |
| 47 | KEGG\_ANTIGEN\_PROCESSING\_AND\_PRESENTATION |  | 77 | 0.33 | 1.13 | 0.247 | 0.572 | 1.000 | 1363 | tags=10%, list=7%, signal=11% |
| 48 | KEGG\_ALDOSTERONE\_REGULATED\_SODIUM\_REABSORPTION |  | 40 | 0.36 | 1.11 | 0.318 | 0.618 | 1.000 | 5220 | tags=40%, list=27%, signal=54% |
| 49 | KEGG\_RENAL\_CELL\_CARCINOMA |  | 68 | 0.32 | 1.10 | 0.298 | 0.618 | 1.000 | 3748 | tags=21%, list=19%, signal=25% |
| 50 | KEGG\_FATTY\_ACID\_METABOLISM |  | 39 | 0.35 | 1.09 | 0.339 | 0.641 | 1.000 | 3716 | tags=33%, list=19%, signal=41% |
| 51 | KEGG\_FOCAL\_ADHESION |  | 187 | 0.27 | 1.09 | 0.284 | 0.642 | 1.000 | 5030 | tags=29%, list=26%, signal=39% |
| 52 | KEGG\_ADIPOCYTOKINE\_SIGNALING\_PATHWAY |  | 66 | 0.32 | 1.08 | 0.327 | 0.633 | 1.000 | 3386 | tags=27%, list=17%, signal=33% |
| 53 | KEGG\_BETA\_ALANINE\_METABOLISM |  | 22 | 0.40 | 1.08 | 0.345 | 0.642 | 1.000 | 3716 | tags=32%, list=19%, signal=39% |
| 54 | KEGG\_NEUROACTIVE\_LIGAND\_RECEPTOR\_INTERACTION |  | 250 | 0.26 | 1.08 | 0.276 | 0.632 | 1.000 | 3817 | tags=24%, list=20%, signal=29% |
| 55 | KEGG\_ALPHA\_LINOLENIC\_ACID\_METABOLISM |  | 16 | 0.42 | 1.05 | 0.397 | 0.682 | 1.000 | 1098 | tags=19%, list=6%, signal=20% |
| 56 | KEGG\_VALINE\_LEUCINE\_AND\_ISOLEUCINE\_DEGRADATION |  | 42 | 0.32 | 1.02 | 0.414 | 0.761 | 1.000 | 310 | tags=7%, list=2%, signal=7% |
| 57 | KEGG\_PRION\_DISEASES |  | 35 | 0.33 | 1.02 | 0.397 | 0.749 | 1.000 | 2655 | tags=23%, list=14%, signal=26% |
| 58 | KEGG\_GLYCINE\_SERINE\_AND\_THREONINE\_METABOLISM |  | 31 | 0.35 | 1.02 | 0.432 | 0.746 | 1.000 | 2333 | tags=26%, list=12%, signal=29% |
| 59 | KEGG\_CHEMOKINE\_SIGNALING\_PATHWAY |  | 172 | 0.26 | 1.02 | 0.407 | 0.737 | 1.000 | 5030 | tags=31%, list=26%, signal=42% |
| 60 | KEGG\_GLYCOLYSIS\_GLUCONEOGENESIS |  | 60 | 0.30 | 1.01 | 0.422 | 0.752 | 1.000 | 2829 | tags=17%, list=14%, signal=19% |
| 61 | KEGG\_RIBOFLAVIN\_METABOLISM |  | 16 | 0.40 | 1.01 | 0.460 | 0.740 | 1.000 | 5542 | tags=50%, list=28%, signal=70% |
| 62 | KEGG\_RIBOSOME |  | 78 | 0.28 | 0.99 | 0.473 | 0.765 | 1.000 | 9990 | tags=74%, list=51%, signal=151% |
| 63 | KEGG\_INTESTINAL\_IMMUNE\_NETWORK\_FOR\_IGA\_PRODUCTION |  | 45 | 0.31 | 0.99 | 0.451 | 0.754 | 1.000 | 4422 | tags=29%, list=23%, signal=37% |
| 64 | KEGG\_ERBB\_SIGNALING\_PATHWAY |  | 85 | 0.28 | 0.98 | 0.488 | 0.766 | 1.000 | 4947 | tags=29%, list=25%, signal=39% |
| 65 | KEGG\_PROPANOATE\_METABOLISM |  | 31 | 0.34 | 0.98 | 0.468 | 0.759 | 1.000 | 6735 | tags=45%, list=34%, signal=69% |
| 66 | KEGG\_TYPE\_II\_DIABETES\_MELLITUS |  | 44 | 0.31 | 0.98 | 0.484 | 0.748 | 1.000 | 3273 | tags=20%, list=17%, signal=25% |
| 67 | KEGG\_LEISHMANIA\_INFECTION |  | 62 | 0.29 | 0.97 | 0.510 | 0.759 | 1.000 | 799 | tags=6%, list=4%, signal=7% |
| 68 | KEGG\_GLYCEROLIPID\_METABOLISM |  | 43 | 0.30 | 0.97 | 0.496 | 0.763 | 1.000 | 2301 | tags=19%, list=12%, signal=21% |
| 69 | KEGG\_TOLL\_LIKE\_RECEPTOR\_SIGNALING\_PATHWAY |  | 96 | 0.26 | 0.96 | 0.509 | 0.761 | 1.000 | 5844 | tags=38%, list=30%, signal=53% |
| 70 | KEGG\_INSULIN\_SIGNALING\_PATHWAY |  | 130 | 0.25 | 0.96 | 0.520 | 0.758 | 1.000 | 4686 | tags=26%, list=24%, signal=34% |
| 71 | KEGG\_MATURITY\_ONSET\_DIABETES\_OF\_THE\_YOUNG |  | 19 | 0.36 | 0.95 | 0.518 | 0.778 | 1.000 | 2719 | tags=37%, list=14%, signal=43% |
| 72 | KEGG\_VIBRIO\_CHOLERAE\_INFECTION |  | 50 | 0.29 | 0.94 | 0.551 | 0.799 | 1.000 | 7061 | tags=54%, list=36%, signal=84% |
| 73 | KEGG\_ALANINE\_ASPARTATE\_AND\_GLUTAMATE\_METABOLISM |  | 31 | 0.31 | 0.93 | 0.556 | 0.797 | 1.000 | 4851 | tags=42%, list=25%, signal=56% |
| 74 | KEGG\_GLYCOSPHINGOLIPID\_BIOSYNTHESIS\_GANGLIO\_SERIES |  | 15 | 0.37 | 0.92 | 0.561 | 0.817 | 1.000 | 5462 | tags=40%, list=28%, signal=55% |
| 75 | KEGG\_GALACTOSE\_METABOLISM |  | 25 | 0.33 | 0.92 | 0.578 | 0.810 | 1.000 | 5462 | tags=40%, list=28%, signal=55% |
| 76 | KEGG\_FC\_GAMMA\_R\_MEDIATED\_PHAGOCYTOSIS |  | 85 | 0.26 | 0.92 | 0.595 | 0.803 | 1.000 | 5354 | tags=39%, list=27%, signal=53% |
| 77 | KEGG\_ARRHYTHMOGENIC\_RIGHT\_VENTRICULAR\_CARDIOMYOPATHY\_ARVC |  | 73 | 0.26 | 0.90 | 0.657 | 0.847 | 1.000 | 4443 | tags=34%, list=23%, signal=44% |
| 78 | KEGG\_OLFACTORY\_TRANSDUCTION |  | 112 | 0.24 | 0.88 | 0.699 | 0.875 | 1.000 | 5328 | tags=35%, list=27%, signal=48% |
| 79 | KEGG\_ALLOGRAFT\_REJECTION |  | 34 | 0.29 | 0.86 | 0.636 | 0.902 | 1.000 | 45 | tags=3%, list=0%, signal=3% |
| 80 | KEGG\_GRAFT\_VERSUS\_HOST\_DISEASE |  | 36 | 0.28 | 0.86 | 0.683 | 0.902 | 1.000 | 45 | tags=3%, list=0%, signal=3% |
| 81 | KEGG\_TERPENOID\_BACKBONE\_BIOSYNTHESIS |  | 15 | 0.35 | 0.85 | 0.646 | 0.907 | 1.000 | 122 | tags=7%, list=1%, signal=7% |
| 82 | KEGG\_RIG\_I\_LIKE\_RECEPTOR\_SIGNALING\_PATHWAY |  | 64 | 0.25 | 0.85 | 0.757 | 0.903 | 1.000 | 4050 | tags=27%, list=21%, signal=33% |
| 83 | KEGG\_AMYOTROPHIC\_LATERAL\_SCLEROSIS\_ALS |  | 49 | 0.26 | 0.84 | 0.703 | 0.912 | 1.000 | 2575 | tags=14%, list=13%, signal=16% |
| 84 | KEGG\_TRYPTOPHAN\_METABOLISM |  | 37 | 0.27 | 0.84 | 0.733 | 0.905 | 1.000 | 3737 | tags=32%, list=19%, signal=40% |
| 85 | KEGG\_REGULATION\_OF\_AUTOPHAGY |  | 32 | 0.28 | 0.83 | 0.711 | 0.906 | 1.000 | 3017 | tags=25%, list=15%, signal=30% |
| 86 | KEGG\_TYPE\_I\_DIABETES\_MELLITUS |  | 40 | 0.27 | 0.82 | 0.756 | 0.917 | 1.000 | 4256 | tags=23%, list=22%, signal=29% |
| 87 | KEGG\_ASTHMA |  | 27 | 0.28 | 0.82 | 0.720 | 0.917 | 1.000 | 45 | tags=4%, list=0%, signal=4% |
| 88 | KEGG\_CALCIUM\_SIGNALING\_PATHWAY |  | 170 | 0.21 | 0.81 | 0.891 | 0.910 | 1.000 | 4858 | tags=26%, list=25%, signal=35% |
| 89 | KEGG\_JAK\_STAT\_SIGNALING\_PATHWAY |  | 149 | 0.21 | 0.81 | 0.901 | 0.907 | 1.000 | 3541 | tags=20%, list=18%, signal=24% |
| 90 | KEGG\_BIOSYNTHESIS\_OF\_UNSATURATED\_FATTY\_ACIDS |  | 19 | 0.31 | 0.80 | 0.757 | 0.922 | 1.000 | 791 | tags=16%, list=4%, signal=16% |
| 91 | KEGG\_PHOSPHATIDYLINOSITOL\_SIGNALING\_SYSTEM |  | 67 | 0.23 | 0.79 | 0.852 | 0.918 | 1.000 | 3224 | tags=24%, list=16%, signal=28% |
| 92 | KEGG\_LEUKOCYTE\_TRANSENDOTHELIAL\_MIGRATION |  | 105 | 0.21 | 0.78 | 0.907 | 0.927 | 1.000 | 4865 | tags=30%, list=25%, signal=39% |
| 93 | KEGG\_CELL\_ADHESION\_MOLECULES\_CAMS |  | 124 | 0.20 | 0.78 | 0.917 | 0.924 | 1.000 | 1748 | tags=8%, list=9%, signal=9% |
| 94 | KEGG\_SELENOAMINO\_ACID\_METABOLISM |  | 21 | 0.29 | 0.77 | 0.774 | 0.924 | 1.000 | 2580 | tags=24%, list=13%, signal=27% |
| 95 | KEGG\_AUTOIMMUNE\_THYROID\_DISEASE |  | 48 | 0.24 | 0.76 | 0.841 | 0.921 | 1.000 | 4256 | tags=21%, list=22%, signal=27% |
| 96 | KEGG\_INOSITOL\_PHOSPHATE\_METABOLISM |  | 46 | 0.24 | 0.76 | 0.887 | 0.924 | 1.000 | 2726 | tags=22%, list=14%, signal=25% |
| 97 | KEGG\_EPITHELIAL\_CELL\_SIGNALING\_IN\_HELICOBACTER\_PYLORI\_INFECTION |  | 62 | 0.22 | 0.75 | 0.900 | 0.925 | 1.000 | 5795 | tags=39%, list=30%, signal=55% |
| 98 | KEGG\_NATURAL\_KILLER\_CELL\_MEDIATED\_CYTOTOXICITY |  | 125 | 0.19 | 0.71 | 0.974 | 0.956 | 1.000 | 5030 | tags=29%, list=26%, signal=39% |
| 99 | KEGG\_VEGF\_SIGNALING\_PATHWAY |  | 70 | 0.20 | 0.69 | 0.946 | 0.961 | 1.000 | 3224 | tags=19%, list=16%, signal=22% |
| 100 | KEGG\_NEUROTROPHIN\_SIGNALING\_PATHWAY |  | 121 | 0.18 | 0.67 | 0.985 | 0.970 | 1.000 | 5923 | tags=31%, list=30%, signal=44% |
| 101 | KEGG\_B\_CELL\_RECEPTOR\_SIGNALING\_PATHWAY |  | 71 | 0.18 | 0.64 | 0.989 | 0.975 | 1.000 | 3658 | tags=18%, list=19%, signal=22% |
| 102 | KEGG\_OXIDATIVE\_PHOSPHORYLATION |  | 109 | 0.13 | 0.49 | 1.000 | 0.997 | 1.000 | 16430 | tags=99%, list=84%, signal=616% |
Table: Gene sets enriched in phenotype **1 (3 samples) **[plain text format]****
