# Supplementary material for: Comprehensive analysis of β-catenin target genes in colorectal carcinoma cell lines with deregulated Wnt/β-catenin signaling
Source: BMC Genomics. 2014 Jan 28;15:74. doi: 10.1186/1471-2164-15-74 (PMC3909937; doi:10.1186/1471-2164-15-74)
Supplement: Additional file 5 — GSEA analysis using the KEGG pathway database. This zipped file contains confirming data of the GSEA analysis. The names of the directories containing the files were composed of the term ‘GSEA’, the name of the cell line, e.g. DLD1, SW480, or LS174T, and the pathway database (KEGG). Please use a web browser to view the files with the name ‘index.html’ in the corresponding directories to start exploring the data. [file 1471-2164-15-74-S5.zip › GSEA_LS174T_KEGG/index.html]

Index for xtools.gsea.Gsea my\_analysis.Gsea.1358419934281

### GSEA Report for Dataset LS174T

#### Enrichment in phenotype: **1 (3 samples)**

- 102 / 175 gene sets are upregulated in phenotype **1**- 22 gene sets are significant at FDR < 25%- 7 gene sets are significantly enriched at nominal pvalue < 1%- 20 gene sets are significantly enriched at nominal pvalue < 5%- Snapshot of enrichment results- Detailed enrichment results in html format- Detailed enrichment results in excel format (tab delimited text)- Guide to interpret results

#### Enrichment in phenotype: **0 (3 samples)**

- 73 / 175 gene sets are upregulated in phenotype **0**- 22 gene sets are significantly enriched at FDR < 25%- 11 gene sets are significantly enriched at nominal pvalue < 1%- 17 gene sets are significantly enriched at nominal pvalue < 5%- Snapshot of enrichment results- Detailed enrichment results in html format- Detailed enrichment results in excel format (tab delimited text)- Guide to interpret results

#### Dataset details

- The dataset has 41656 native features- After collapsing features into gene symbols, there are: 19557 genes

#### Gene set details

- Gene set size filters (min=15, max=500) resulted in filtering out 11 / 186 gene sets- The remaining 175 gene sets were used in the analysis- List of gene sets used and their sizes (restricted to features in the specified dataset)

#### Gene markers for the **1** *versus* **0** comparison

- The dataset has 19557 features (genes)- # of markers for phenotype **1**: 10079 (51.5% ) with correlation area 52.3%- # of markers for phenotype **0**: 9478 (48.5% ) with correlation area 47.7%- Detailed rank ordered gene list for all features in the dataset- Heat map and gene list correlation  profile for all features in the dataset

#### Global statistics and plots

- Plot of p-values *vs.* NES- Global ES histogram

#### Other

- Parameters used for this analysis

---

Report: my\_analysis.Gsea.1358419934281.rpt   by user: Jurionovic

xtools.gsea.Gsea [Do, Jan 17, '13 11 AM 52]

Website: www.broadinstitute.org/GSEA
Questions & Suggestions: Email
