# Supplementary material for: Comprehensive analysis of β-catenin target genes in colorectal carcinoma cell lines with deregulated Wnt/β-catenin signaling
Source: BMC Genomics. 2014 Jan 28;15:74. doi: 10.1186/1471-2164-15-74 (PMC3909937; doi:10.1186/1471-2164-15-74)
Supplement: Additional file 5 — GSEA analysis using the KEGG pathway database. This zipped file contains confirming data of the GSEA analysis. The names of the directories containing the files were composed of the term ‘GSEA’, the name of the cell line, e.g. DLD1, SW480, or LS174T, and the pathway database (KEGG). Please use a web browser to view the files with the name ‘index.html’ in the corresponding directories to start exploring the data. [file 1471-2164-15-74-S5.zip › GSEA_LS174T_KEGG/KEGG_ARACHIDONIC_ACID_METABOLISM.html]

Details for gene set KEGG\_ARACHIDONIC\_ACID\_METABOLISM[GSEA]

|  || Dataset | LS174T\_collapsed\_to\_symbols.class.cls#b\_versus\_bg.class.cls#b\_versus\_bg\_repos |
| Phenotype | class.cls#b\_versus\_bg\_repos |
| Upregulated in class | 1 |
| GeneSet | KEGG\_ARACHIDONIC\_ACID\_METABOLISM |
| Enrichment Score (ES) | 0.49937442 |
| Normalized Enrichment Score (NES) | 1.6340761 |
| Nominal p-value | 0.016274864 |
| FDR q-value | 0.10529233 |
| FWER p-Value | 0.594 |
Table: GSEA Results Summary

  

Fig 1: Enrichment plot: KEGG\_ARACHIDONIC\_ACID\_METABOLISM      
 Profile of the Running ES Score & Positions of GeneSet Members on the Rank Ordered List

  

| PROBE | GENE SYMBOL | GENE\_TITLE | RANK IN GENE LIST | RANK METRIC SCORE | RUNNING ES | CORE ENRICHMENT || 1 | CYP4F2 | CYP4F2 Entrez,  Source | cytochrome P450, family 4, subfamily F, polypeptide 2 | 69 | 0.990 | 0.1066 | Yes |
| 2 | PLA2G10 | PLA2G10 Entrez,  Source | phospholipase A2, group X | 111 | 0.821 | 0.1957 | Yes |
| 3 | AKR1C3 | AKR1C3 Entrez,  Source | aldo-keto reductase family 1, member C3 (3-alpha hydroxysteroid dehydrogenase, type II) | 364 | 0.481 | 0.2363 | Yes |
| 4 | CYP2B6 | CYP2B6 Entrez,  Source | cytochrome P450, family 2, subfamily B, polypeptide 6 | 581 | 0.391 | 0.2688 | Yes |
| 5 | GPX7 | GPX7 Entrez,  Source | glutathione peroxidase 7 | 803 | 0.338 | 0.2951 | Yes |
| 6 | CYP2C18 | CYP2C18 Entrez,  Source | cytochrome P450, family 2, subfamily C, polypeptide 18 | 816 | 0.334 | 0.3316 | Yes |
| 7 | PLA2G6 | PLA2G6 Entrez,  Source | phospholipase A2, group VI (cytosolic, calcium-independent) | 1098 | 0.287 | 0.3491 | Yes |
| 8 | ALOX12B | ALOX12B Entrez,  Source | arachidonate 12-lipoxygenase, 12R type | 1145 | 0.280 | 0.3779 | Yes |
| 9 | GGT6 | GGT6 Entrez,  Source | gamma-glutamyltransferase 6 homolog (rat) | 1587 | 0.237 | 0.3816 | Yes |
| 10 | GPX3 | GPX3 Entrez,  Source | glutathione peroxidase 3 (plasma) | 1730 | 0.226 | 0.3994 | Yes |
| 11 | CYP2C8 | CYP2C8 Entrez,  Source | cytochrome P450, family 2, subfamily C, polypeptide 8 | 1790 | 0.221 | 0.4210 | Yes |
| 12 | CYP4F3 | CYP4F3 Entrez,  Source | cytochrome P450, family 4, subfamily F, polypeptide 3 | 1820 | 0.219 | 0.4439 | Yes |
| 13 | CBR1 | CBR1 Entrez,  Source | carbonyl reductase 1 | 2208 | 0.194 | 0.4457 | Yes |
| 14 | PLA2G2F | PLA2G2F Entrez,  Source | phospholipase A2, group IIF | 2425 | 0.182 | 0.4548 | Yes |
| 15 | GGT1 | GGT1 Entrez,  Source | gamma-glutamyltransferase 1 | 2580 | 0.175 | 0.4663 | Yes |
| 16 | CYP2J2 | CYP2J2 Entrez,  Source | cytochrome P450, family 2, subfamily J, polypeptide 2 | 2847 | 0.163 | 0.4708 | Yes |
| 17 | EPHX2 | EPHX2 Entrez,  Source | epoxide hydrolase 2, cytoplasmic | 3344 | 0.145 | 0.4615 | Yes |
| 18 | ALOX15B | ALOX15B Entrez,  Source | arachidonate 15-lipoxygenase, type B | 3563 | 0.138 | 0.4656 | Yes |
| 19 | CYP2E1 | CYP2E1 Entrez,  Source | cytochrome P450, family 2, subfamily E, polypeptide 1 | 3682 | 0.133 | 0.4744 | Yes |
| 20 | PTGDS | PTGDS Entrez,  Source | prostaglandin D2 synthase 21kDa (brain) | 3811 | 0.129 | 0.4822 | Yes |
| 21 | TBXAS1 | TBXAS1 Entrez,  Source | thromboxane A synthase 1 (platelet, cytochrome P450, family 5, subfamily A) | 3883 | 0.127 | 0.4927 | Yes |
| 22 | PTGS1 | PTGS1 Entrez,  Source | prostaglandin-endoperoxide synthase 1 (prostaglandin G/H synthase and cyclooxygenase) | 4020 | 0.123 | 0.4994 | Yes |
| 23 | GPX5 | GPX5 Entrez,  Source | glutathione peroxidase 5 (epididymal androgen-related protein) | 4700 | 0.103 | 0.4761 | No |
| 24 | ALOX12 | ALOX12 Entrez,  Source | arachidonate 12-lipoxygenase | 5253 | 0.090 | 0.4578 | No |
| 25 | CYP2C9 | CYP2C9 Entrez,  Source | cytochrome P450, family 2, subfamily C, polypeptide 9 | 5445 | 0.085 | 0.4574 | No |
| 26 | LTA4H | LTA4H Entrez,  Source | leukotriene A4 hydrolase | 5802 | 0.077 | 0.4477 | No |
| 27 | PLA2G12A | PLA2G12A Entrez,  Source | phospholipase A2, group XIIA | 5935 | 0.074 | 0.4492 | No |
| 28 | CBR3 | CBR3 Entrez,  Source | carbonyl reductase 3 | 6207 | 0.069 | 0.4430 | No |
| 29 | CYP4A11 | CYP4A11 Entrez,  Source | cytochrome P450, family 4, subfamily A, polypeptide 11 | 7273 | 0.048 | 0.3938 | No |
| 30 | LTC4S | LTC4S Entrez,  Source | leukotriene C4 synthase | 7661 | 0.041 | 0.3785 | No |
| 31 | GPX1 | GPX1 Entrez,  Source | glutathione peroxidase 1 | 7893 | 0.037 | 0.3707 | No |
| 32 | PTGES | PTGES Entrez,  Source | prostaglandin E synthase | 9576 | 0.008 | 0.2854 | No |
| 33 | PLA2G5 | PLA2G5 Entrez,  Source | phospholipase A2, group V | 10163 | -0.001 | 0.2555 | No |
| 34 | PLA2G12B | PLA2G12B Entrez,  Source | phospholipase A2, group XIIB | 10181 | -0.001 | 0.2548 | No |
| 35 | GPX4 | GPX4 Entrez,  Source | glutathione peroxidase 4 (phospholipid hydroperoxidase) | 10776 | -0.011 | 0.2255 | No |
| 36 | PLA2G4B | PLA2G4B Entrez,  Source | phospholipase A2, group IVB (cytosolic) | 12128 | -0.035 | 0.1602 | No |
| 37 | PTGIS | PTGIS Entrez,  Source | prostaglandin I2 (prostacyclin) synthase | 12462 | -0.041 | 0.1477 | No |
| 38 | ALOX5 | ALOX5 Entrez,  Source | arachidonate 5-lipoxygenase | 13269 | -0.057 | 0.1127 | No |
| 39 | ALOX15 | ALOX15 Entrez,  Source | arachidonate 15-lipoxygenase | 13709 | -0.066 | 0.0975 | No |
| 40 | CYP2U1 | CYP2U1 Entrez,  Source | cytochrome P450, family 2, subfamily U, polypeptide 1 | 14299 | -0.079 | 0.0761 | No |
| 41 | PLA2G3 | PLA2G3 Entrez,  Source | phospholipase A2, group III | 14302 | -0.079 | 0.0848 | No |
| 42 | PTGES2 | PTGES2 Entrez,  Source | prostaglandin E synthase 2 | 14781 | -0.092 | 0.0705 | No |
| 43 | GPX2 | GPX2 Entrez,  Source | glutathione peroxidase 2 (gastrointestinal) | 15114 | -0.100 | 0.0647 | No |
| 44 | PLA2G2D | PLA2G2D Entrez,  Source | phospholipase A2, group IID | 15256 | -0.105 | 0.0691 | No |
| 45 | PLA2G2A | PLA2G2A Entrez,  Source | phospholipase A2, group IIA (platelets, synovial fluid) | 16521 | -0.147 | 0.0206 | No |
| 46 | PLA2G2E | PLA2G2E Entrez,  Source | phospholipase A2, group IIE | 16698 | -0.155 | 0.0289 | No |
| 47 | CYP2C19 | CYP2C19 Entrez,  Source | cytochrome P450, family 2, subfamily C, polypeptide 19 | 17104 | -0.175 | 0.0276 | No |
| 48 | PLA2G4A | PLA2G4A Entrez,  Source | phospholipase A2, group IVA (cytosolic, calcium-dependent) | 17896 | -0.227 | 0.0123 | No |
| 49 | PLA2G1B | PLA2G1B Entrez,  Source | phospholipase A2, group IB (pancreas) | 18566 | -0.301 | 0.0115 | No |
| 50 | PTGS2 | PTGS2 Entrez,  Source | prostaglandin-endoperoxide synthase 2 (prostaglandin G/H synthase and cyclooxygenase) | 18851 | -0.353 | 0.0361 | No |
Table: GSEA details [plain text format]

  

Fig 2: KEGG\_ARACHIDONIC\_ACID\_METABOLISM      
 Blue-Pink O' Gram in the Space of the Analyzed GeneSet

  

Fig 3: KEGG\_ARACHIDONIC\_ACID\_METABOLISM: Random ES distribution      
 Gene set null distribution of ES for **KEGG\_ARACHIDONIC\_ACID\_METABOLISM**
