# Supplementary material for: Comprehensive analysis of β-catenin target genes in colorectal carcinoma cell lines with deregulated Wnt/β-catenin signaling
Source: BMC Genomics. 2014 Jan 28;15:74. doi: 10.1186/1471-2164-15-74 (PMC3909937; doi:10.1186/1471-2164-15-74)
Supplement: Additional file 5 — GSEA analysis using the KEGG pathway database. This zipped file contains confirming data of the GSEA analysis. The names of the directories containing the files were composed of the term ‘GSEA’, the name of the cell line, e.g. DLD1, SW480, or LS174T, and the pathway database (KEGG). Please use a web browser to view the files with the name ‘index.html’ in the corresponding directories to start exploring the data. [file 1471-2164-15-74-S5.zip › GSEA_LS174T_KEGG/KEGG_BASE_EXCISION_REPAIR.html]

Details for gene set KEGG\_BASE\_EXCISION\_REPAIR[GSEA]

|  || Dataset | LS174T\_collapsed\_to\_symbols.class.cls#b\_versus\_bg.class.cls#b\_versus\_bg\_repos |
| Phenotype | class.cls#b\_versus\_bg\_repos |
| Upregulated in class | 0 |
| GeneSet | KEGG\_BASE\_EXCISION\_REPAIR |
| Enrichment Score (ES) | -0.60372424 |
| Normalized Enrichment Score (NES) | -1.8868544 |
| Nominal p-value | 0.0 |
| FDR q-value | 0.0051208087 |
| FWER p-Value | 0.039 |
Table: GSEA Results Summary

  

Fig 1: Enrichment plot: KEGG\_BASE\_EXCISION\_REPAIR      
 Profile of the Running ES Score & Positions of GeneSet Members on the Rank Ordered List

  

| PROBE | GENE SYMBOL | GENE\_TITLE | RANK IN GENE LIST | RANK METRIC SCORE | RUNNING ES | CORE ENRICHMENT || 1 | POLD4 | POLD4 Entrez,  Source | polymerase (DNA-directed), delta 4 | 753 | 0.350 | -0.0000 | No |
| 2 | OGG1 | OGG1 Entrez,  Source | 8-oxoguanine DNA glycosylase | 869 | 0.324 | 0.0297 | No |
| 3 | NEIL1 | NEIL1 Entrez,  Source | nei endonuclease VIII-like 1 (E. coli) | 1031 | 0.298 | 0.0543 | No |
| 4 | TDG | TDG Entrez,  Source | thymine-DNA glycosylase | 1472 | 0.246 | 0.0589 | No |
| 5 | POLE4 | POLE4 Entrez,  Source | polymerase (DNA-directed), epsilon 4 (p12 subunit) | 2276 | 0.190 | 0.0387 | No |
| 6 | POLL | POLL Entrez,  Source | polymerase (DNA directed), lambda | 4589 | 0.106 | -0.0680 | No |
| 7 | PARP4 | PARP4 Entrez,  Source | poly (ADP-ribose) polymerase family, member 4 | 5133 | 0.092 | -0.0857 | No |
| 8 | SMUG1 | SMUG1 Entrez,  Source | single-strand-selective monofunctional uracil-DNA glycosylase 1 | 5219 | 0.091 | -0.0801 | No |
| 9 | PARP3 | PARP3 Entrez,  Source | poly (ADP-ribose) polymerase family, member 3 | 5356 | 0.087 | -0.0774 | No |
| 10 | APEX1 | APEX1 Entrez,  Source | APEX nuclease (multifunctional DNA repair enzyme) 1 | 7919 | 0.036 | -0.2047 | No |
| 11 | POLB | POLB Entrez,  Source | polymerase (DNA directed), beta | 8845 | 0.020 | -0.2499 | No |
| 12 | MPG | MPG Entrez,  Source | N-methylpurine-DNA glycosylase | 11622 | -0.027 | -0.3891 | No |
| 13 | MBD4 | MBD4 Entrez,  Source | methyl-CpG binding domain protein 4 | 13131 | -0.054 | -0.4604 | No |
| 14 | MUTYH | MUTYH Entrez,  Source | mutY homolog (E. coli) | 13559 | -0.063 | -0.4754 | No |
| 15 | APEX2 | APEX2 Entrez,  Source | APEX nuclease (apurinic/apyrimidinic endonuclease) 2 | 15108 | -0.100 | -0.5437 | No |
| 16 | NTHL1 | NTHL1 Entrez,  Source | nth endonuclease III-like 1 (E. coli) | 15133 | -0.101 | -0.5338 | No |
| 17 | HMGB1 | HMGB1 Entrez,  Source | high-mobility group box 1 | 15299 | -0.106 | -0.5306 | No |
| 18 | LIG3 | LIG3 Entrez,  Source | ligase III, DNA, ATP-dependent | 15945 | -0.125 | -0.5499 | No |
| 19 | PARP1 | PARP1 Entrez,  Source | poly (ADP-ribose) polymerase family, member 1 | 16827 | -0.161 | -0.5773 | No |
| 20 | UNG | UNG Entrez,  Source | uracil-DNA glycosylase | 17344 | -0.188 | -0.5830 | Yes |
| 21 | PARP2 | PARP2 Entrez,  Source | poly (ADP-ribose) polymerase family, member 2 | 17488 | -0.198 | -0.5685 | Yes |
| 22 | POLD2 | POLD2 Entrez,  Source | polymerase (DNA directed), delta 2, regulatory subunit 50kDa | 17923 | -0.230 | -0.5655 | Yes |
| 23 | XRCC1 | XRCC1 Entrez,  Source | X-ray repair complementing defective repair in Chinese hamster cells 1 | 18328 | -0.271 | -0.5564 | Yes |
| 24 | POLD1 | POLD1 Entrez,  Source | polymerase (DNA directed), delta 1, catalytic subunit 125kDa | 18666 | -0.318 | -0.5386 | Yes |
| 25 | PCNA | PCNA Entrez,  Source | proliferating cell nuclear antigen | 18712 | -0.326 | -0.5050 | Yes |
| 26 | POLE3 | POLE3 Entrez,  Source | polymerase (DNA directed), epsilon 3 (p17 subunit) | 18960 | -0.385 | -0.4752 | Yes |
| 27 | FEN1 | FEN1 Entrez,  Source | flap structure-specific endonuclease 1 | 19114 | -0.440 | -0.4346 | Yes |
| 28 | POLE | POLE Entrez,  Source | polymerase (DNA directed), epsilon | 19281 | -0.529 | -0.3849 | Yes |
| 29 | LIG1 | LIG1 Entrez,  Source | ligase I, DNA, ATP-dependent | 19298 | -0.542 | -0.3260 | Yes |
| 30 | NEIL2 | NEIL2 Entrez,  Source | nei like 2 (E. coli) | 19338 | -0.567 | -0.2656 | Yes |
| 31 | NEIL3 | NEIL3 Entrez,  Source | nei endonuclease VIII-like 3 (E. coli) | 19485 | -0.789 | -0.1862 | Yes |
| 32 | POLD3 | POLD3 Entrez,  Source | polymerase (DNA-directed), delta 3, accessory subunit | 19495 | -0.805 | -0.0980 | Yes |
| 33 | POLE2 | POLE2 Entrez,  Source | polymerase (DNA directed), epsilon 2 (p59 subunit) | 19520 | -0.918 | 0.0018 | Yes |
Table: GSEA details [plain text format]

  

Fig 2: KEGG\_BASE\_EXCISION\_REPAIR      
 Blue-Pink O' Gram in the Space of the Analyzed GeneSet

  

Fig 3: KEGG\_BASE\_EXCISION\_REPAIR: Random ES distribution      
 Gene set null distribution of ES for **KEGG\_BASE\_EXCISION\_REPAIR**
