# Supplementary material for: Comprehensive analysis of β-catenin target genes in colorectal carcinoma cell lines with deregulated Wnt/β-catenin signaling
Source: BMC Genomics. 2014 Jan 28;15:74. doi: 10.1186/1471-2164-15-74 (PMC3909937; doi:10.1186/1471-2164-15-74)
Supplement: Additional file 5 — GSEA analysis using the KEGG pathway database. This zipped file contains confirming data of the GSEA analysis. The names of the directories containing the files were composed of the term ‘GSEA’, the name of the cell line, e.g. DLD1, SW480, or LS174T, and the pathway database (KEGG). Please use a web browser to view the files with the name ‘index.html’ in the corresponding directories to start exploring the data. [file 1471-2164-15-74-S5.zip › GSEA_LS174T_KEGG/KEGG_CELL_CYCLE.html]

Details for gene set KEGG\_CELL\_CYCLE[GSEA]

|  || Dataset | LS174T\_collapsed\_to\_symbols.class.cls#b\_versus\_bg.class.cls#b\_versus\_bg\_repos |
| Phenotype | class.cls#b\_versus\_bg\_repos |
| Upregulated in class | 0 |
| GeneSet | KEGG\_CELL\_CYCLE |
| Enrichment Score (ES) | -0.5891673 |
| Normalized Enrichment Score (NES) | -2.3242662 |
| Nominal p-value | 0.0 |
| FDR q-value | 0.0 |
| FWER p-Value | 0.0 |
Table: GSEA Results Summary

  

Fig 1: Enrichment plot: KEGG\_CELL\_CYCLE      
 Profile of the Running ES Score & Positions of GeneSet Members on the Rank Ordered List

  

| PROBE | GENE SYMBOL | GENE\_TITLE | RANK IN GENE LIST | RANK METRIC SCORE | RUNNING ES | CORE ENRICHMENT || 1 | CDKN2B | CDKN2B Entrez,  Source | cyclin-dependent kinase inhibitor 2B (p15, inhibits CDK4) | 10 | 2.022 | 0.0808 | No |
| 2 | TGFB2 | TGFB2 Entrez,  Source | transforming growth factor, beta 2 | 799 | 0.339 | 0.0540 | No |
| 3 | E2F5 | E2F5 Entrez,  Source | E2F transcription factor 5, p130-binding | 1620 | 0.234 | 0.0212 | No |
| 4 | E2F3 | E2F3 Entrez,  Source | E2F transcription factor 3 | 2456 | 0.180 | -0.0145 | No |
| 5 | GADD45B | GADD45B Entrez,  Source | growth arrest and DNA-damage-inducible, beta | 2459 | 0.180 | -0.0074 | No |
| 6 | ANAPC13 | ANAPC13 Entrez,  Source | anaphase promoting complex subunit 13 | 2876 | 0.162 | -0.0223 | No |
| 7 | CCNA1 | CCNA1 Entrez,  Source | cyclin A1 | 4153 | 0.119 | -0.0831 | No |
| 8 | SMC1B | SMC1B Entrez,  Source | structural maintenance of chromosomes 1B | 4486 | 0.109 | -0.0958 | No |
| 9 | CDC16 | CDC16 Entrez,  Source | CDC16 cell division cycle 16 homolog (S. cerevisiae) | 4588 | 0.106 | -0.0967 | No |
| 10 | CCNB3 | CCNB3 Entrez,  Source | cyclin B3 | 4731 | 0.103 | -0.0999 | No |
| 11 | ATM | ATM Entrez,  Source | ataxia telangiectasia mutated (includes complementation groups A, C and D) | 5168 | 0.092 | -0.1186 | No |
| 12 | SFN | SFN Entrez,  Source | stratifin | 5220 | 0.090 | -0.1176 | No |
| 13 | TGFB3 | TGFB3 Entrez,  Source | transforming growth factor, beta 3 | 5325 | 0.088 | -0.1194 | No |
| 14 | YWHAH | YWHAH Entrez,  Source | tyrosine 3-monooxygenase/tryptophan 5-monooxygenase activation protein, eta polypeptide | 5873 | 0.075 | -0.1445 | No |
| 15 | CDKN1B | CDKN1B Entrez,  Source | cyclin-dependent kinase inhibitor 1B (p27, Kip1) | 5946 | 0.074 | -0.1452 | No |
| 16 | YWHAZ | YWHAZ Entrez,  Source | tyrosine 3-monooxygenase/tryptophan 5-monooxygenase activation protein, zeta polypeptide | 6389 | 0.065 | -0.1653 | No |
| 17 | EP300 | EP300 Entrez,  Source | E1A binding protein p300 | 6576 | 0.061 | -0.1724 | No |
| 18 | CDKN2A | CDKN2A Entrez,  Source | cyclin-dependent kinase inhibitor 2A (melanoma, p16, inhibits CDK4) | 6824 | 0.057 | -0.1828 | No |
| 19 | CDC14A | CDC14A Entrez,  Source | CDC14 cell division cycle 14 homolog A (S. cerevisiae) | 6914 | 0.055 | -0.1852 | No |
| 20 | GADD45A | GADD45A Entrez,  Source | growth arrest and DNA-damage-inducible, alpha | 7050 | 0.052 | -0.1900 | No |
| 21 | SMAD2 | SMAD2 Entrez,  Source | SMAD, mothers against DPP homolog 2 (Drosophila) | 7089 | 0.052 | -0.1899 | No |
| 22 | TGFB1 | TGFB1 Entrez,  Source | transforming growth factor, beta 1 (Camurati-Engelmann disease) | 7228 | 0.049 | -0.1950 | No |
| 23 | YWHAG | YWHAG Entrez,  Source | tyrosine 3-monooxygenase/tryptophan 5-monooxygenase activation protein, gamma polypeptide | 7364 | 0.047 | -0.2001 | No |
| 24 | GSK3B | GSK3B Entrez,  Source | glycogen synthase kinase 3 beta | 8323 | 0.029 | -0.2482 | No |
| 25 | CDC14B | CDC14B Entrez,  Source | CDC14 cell division cycle 14 homolog B (S. cerevisiae) | 9578 | 0.008 | -0.3124 | No |
| 26 | ANAPC2 | ANAPC2 Entrez,  Source | anaphase promoting complex subunit 2 | 9963 | 0.002 | -0.3320 | No |
| 27 | STAG2 | STAG2 Entrez,  Source | stromal antigen 2 | 9997 | 0.001 | -0.3337 | No |
| 28 | CDC25B | CDC25B Entrez,  Source | cell division cycle 25B | 10129 | -0.001 | -0.3404 | No |
| 29 | RBX1 | RBX1 Entrez,  Source | ring-box 1 | 10204 | -0.002 | -0.3441 | No |
| 30 | FZR1 | FZR1 Entrez,  Source | fizzy/cell division cycle 20 related 1 (Drosophila) | 10724 | -0.010 | -0.3704 | No |
| 31 | YWHAB | YWHAB Entrez,  Source | tyrosine 3-monooxygenase/tryptophan 5-monooxygenase activation protein, beta polypeptide | 10824 | -0.012 | -0.3750 | No |
| 32 | TFDP2 | TFDP2 Entrez,  Source | transcription factor Dp-2 (E2F dimerization partner 2) | 10862 | -0.013 | -0.3764 | No |
| 33 | CREBBP | CREBBP Entrez,  Source | CREB binding protein (Rubinstein-Taybi syndrome) | 11958 | -0.032 | -0.4314 | No |
| 34 | ANAPC11 | ANAPC11 Entrez,  Source | APC11 anaphase promoting complex subunit 11 homolog (yeast) | 11989 | -0.033 | -0.4316 | No |
| 35 | CCNH | CCNH Entrez,  Source | cyclin H | 12072 | -0.034 | -0.4345 | No |
| 36 | RB1 | RB1 Entrez,  Source | retinoblastoma 1 (including osteosarcoma) | 12106 | -0.035 | -0.4348 | No |
| 37 | CDK7 | CDK7 Entrez,  Source | cyclin-dependent kinase 7 (MO15 homolog, Xenopus laevis, cdk-activating kinase) | 12345 | -0.039 | -0.4454 | No |
| 38 | CHEK1 | CHEK1 Entrez,  Source | CHK1 checkpoint homolog (S. pombe) | 12502 | -0.042 | -0.4518 | No |
| 39 | CDKN2D | CDKN2D Entrez,  Source | cyclin-dependent kinase inhibitor 2D (p19, inhibits CDK4) | 12550 | -0.043 | -0.4524 | No |
| 40 | MAD1L1 | MAD1L1 Entrez,  Source | MAD1 mitotic arrest deficient-like 1 (yeast) | 12608 | -0.044 | -0.4536 | No |
| 41 | ANAPC4 | ANAPC4 Entrez,  Source | anaphase promoting complex subunit 4 | 12643 | -0.045 | -0.4535 | No |
| 42 | MDM2 | MDM2 Entrez,  Source | Mdm2, transformed 3T3 cell double minute 2, p53 binding protein (mouse) | 12680 | -0.046 | -0.4535 | No |
| 43 | HDAC1 | HDAC1 Entrez,  Source | histone deacetylase 1 | 12701 | -0.046 | -0.4527 | No |
| 44 | GADD45G | GADD45G Entrez,  Source | growth arrest and DNA-damage-inducible, gamma | 12789 | -0.048 | -0.4552 | No |
| 45 | CDC23 | CDC23 Entrez,  Source | CDC23 (cell division cycle 23, yeast, homolog) | 12882 | -0.049 | -0.4580 | No |
| 46 | E2F4 | E2F4 Entrez,  Source | E2F transcription factor 4, p107/p130-binding | 13028 | -0.052 | -0.4634 | No |
| 47 | ANAPC1 | ANAPC1 Entrez,  Source | anaphase promoting complex subunit 1 | 13231 | -0.056 | -0.4715 | No |
| 48 | ABL1 | ABL1 Entrez,  Source | v-abl Abelson murine leukemia viral oncogene homolog 1 | 13318 | -0.058 | -0.4736 | No |
| 49 | YWHAQ | YWHAQ Entrez,  Source | tyrosine 3-monooxygenase/tryptophan 5-monooxygenase activation protein, theta polypeptide | 13416 | -0.060 | -0.4762 | No |
| 50 | RBL2 | RBL2 Entrez,  Source | retinoblastoma-like 2 (p130) | 13526 | -0.062 | -0.4793 | No |
| 51 | ATR | ATR Entrez,  Source | ataxia telangiectasia and Rad3 related | 13582 | -0.063 | -0.4795 | No |
| 52 | CDC27 | CDC27 Entrez,  Source | cell division cycle 27 | 13859 | -0.070 | -0.4909 | No |
| 53 | CUL1 | CUL1 Entrez,  Source | cullin 1 | 14106 | -0.075 | -0.5006 | No |
| 54 | SMAD4 | SMAD4 Entrez,  Source | SMAD, mothers against DPP homolog 4 (Drosophila) | 14289 | -0.079 | -0.5067 | No |
| 55 | BUB3 | BUB3 Entrez,  Source | BUB3 budding uninhibited by benzimidazoles 3 homolog (yeast) | 14508 | -0.085 | -0.5146 | No |
| 56 | ANAPC5 | ANAPC5 Entrez,  Source | anaphase promoting complex subunit 5 | 15189 | -0.103 | -0.5454 | No |
| 57 | CDKN1A | CDKN1A Entrez,  Source | cyclin-dependent kinase inhibitor 1A (p21, Cip1) | 15286 | -0.106 | -0.5461 | No |
| 58 | CCND3 | CCND3 Entrez,  Source | cyclin D3 | 15420 | -0.109 | -0.5485 | No |
| 59 | RAD21 | RAD21 Entrez,  Source | RAD21 homolog (S. pombe) | 15426 | -0.109 | -0.5444 | No |
| 60 | ANAPC7 | ANAPC7 Entrez,  Source | anaphase promoting complex subunit 7 | 15479 | -0.111 | -0.5426 | No |
| 61 | ANAPC10 | ANAPC10 Entrez,  Source | anaphase promoting complex subunit 10 | 15957 | -0.126 | -0.5621 | No |
| 62 | HDAC2 | HDAC2 Entrez,  Source | histone deacetylase 2 | 16097 | -0.131 | -0.5640 | No |
| 63 | TFDP1 | TFDP1 Entrez,  Source | transcription factor Dp-1 | 16588 | -0.150 | -0.5831 | Yes |
| 64 | CDK6 | CDK6 Entrez,  Source | cyclin-dependent kinase 6 | 16614 | -0.151 | -0.5783 | Yes |
| 65 | ZBTB17 | ZBTB17 Entrez,  Source | zinc finger and BTB domain containing 17 | 16615 | -0.151 | -0.5722 | Yes |
| 66 | PRKDC | PRKDC Entrez,  Source | protein kinase, DNA-activated, catalytic polypeptide | 16624 | -0.152 | -0.5665 | Yes |
| 67 | SMC1A | SMC1A Entrez,  Source | structural maintenance of chromosomes 1A | 16683 | -0.154 | -0.5633 | Yes |
| 68 | STAG1 | STAG1 Entrez,  Source | stromal antigen 1 | 16711 | -0.156 | -0.5584 | Yes |
| 69 | CDK4 | CDK4 Entrez,  Source | cyclin-dependent kinase 4 | 16792 | -0.160 | -0.5561 | Yes |
| 70 | SMAD3 | SMAD3 Entrez,  Source | SMAD, mothers against DPP homolog 3 (Drosophila) | 16806 | -0.160 | -0.5503 | Yes |
| 71 | TP53 | TP53 Entrez,  Source | tumor protein p53 (Li-Fraumeni syndrome) | 17097 | -0.175 | -0.5582 | Yes |
| 72 | SMC3 | SMC3 Entrez,  Source | structural maintenance of chromosomes 3 | 17134 | -0.177 | -0.5529 | Yes |
| 73 | SKP2 | SKP2 Entrez,  Source | S-phase kinase-associated protein 2 (p45) | 17419 | -0.193 | -0.5598 | Yes |
| 74 | WEE1 | WEE1 Entrez,  Source | WEE1 homolog (S. pombe) | 17732 | -0.214 | -0.5672 | Yes |
| 75 | YWHAE | YWHAE Entrez,  Source | tyrosine 3-monooxygenase/tryptophan 5-monooxygenase activation protein, epsilon polypeptide | 17886 | -0.226 | -0.5660 | Yes |
| 76 | MYC | MYC Entrez,  Source | v-myc myelocytomatosis viral oncogene homolog (avian) | 18016 | -0.238 | -0.5630 | Yes |
| 77 | MAD2L2 | MAD2L2 Entrez,  Source | MAD2 mitotic arrest deficient-like 2 (yeast) | 18108 | -0.246 | -0.5578 | Yes |
| 78 | CCND1 | CCND1 Entrez,  Source | cyclin D1 | 18184 | -0.254 | -0.5514 | Yes |
| 79 | CCNB2 | CCNB2 Entrez,  Source | cyclin B2 | 18253 | -0.262 | -0.5444 | Yes |
| 80 | PTTG1 | PTTG1 Entrez,  Source | pituitary tumor-transforming 1 | 18263 | -0.263 | -0.5343 | Yes |
| 81 | DBF4 | DBF4 Entrez,  Source | DBF4 homolog (S. cerevisiae) | 18276 | -0.264 | -0.5243 | Yes |
| 82 | CCND2 | CCND2 Entrez,  Source | cyclin D2 | 18356 | -0.273 | -0.5173 | Yes |
| 83 | CCNB1 | CCNB1 Entrez,  Source | cyclin B1 | 18465 | -0.286 | -0.5114 | Yes |
| 84 | E2F1 | E2F1 Entrez,  Source | E2F transcription factor 1 | 18499 | -0.292 | -0.5013 | Yes |
| 85 | PTTG2 | PTTG2 Entrez,  Source | pituitary tumor-transforming 2 | 18589 | -0.305 | -0.4936 | Yes |
| 86 | PLK1 | PLK1 Entrez,  Source | polo-like kinase 1 (Drosophila) | 18596 | -0.306 | -0.4816 | Yes |
| 87 | MAD2L1 | MAD2L1 Entrez,  Source | MAD2 mitotic arrest deficient-like 1 (yeast) | 18616 | -0.310 | -0.4701 | Yes |
| 88 | PKMYT1 | PKMYT1 Entrez,  Source | protein kinase, membrane associated tyrosine/threonine 1 | 18637 | -0.313 | -0.4586 | Yes |
| 89 | CDC20 | CDC20 Entrez,  Source | CDC20 cell division cycle 20 homolog (S. cerevisiae) | 18657 | -0.317 | -0.4468 | Yes |
| 90 | PCNA | PCNA Entrez,  Source | proliferating cell nuclear antigen | 18712 | -0.326 | -0.4364 | Yes |
| 91 | MCM6 | MCM6 Entrez,  Source | MCM6 minichromosome maintenance deficient 6 (MIS5 homolog, S. pombe) (S. cerevisiae) | 18732 | -0.329 | -0.4242 | Yes |
| 92 | CCNE1 | CCNE1 Entrez,  Source | cyclin E1 | 18741 | -0.330 | -0.4113 | Yes |
| 93 | CHEK2 | CHEK2 Entrez,  Source | CHK2 checkpoint homolog (S. pombe) | 18836 | -0.350 | -0.4021 | Yes |
| 94 | CDK2 | CDK2 Entrez,  Source | cyclin-dependent kinase 2 | 18981 | -0.390 | -0.3938 | Yes |
| 95 | BUB1B | BUB1B Entrez,  Source | BUB1 budding uninhibited by benzimidazoles 1 homolog beta (yeast) | 18996 | -0.394 | -0.3786 | Yes |
| 96 | MCM7 | MCM7 Entrez,  Source | MCM7 minichromosome maintenance deficient 7 (S. cerevisiae) | 19014 | -0.400 | -0.3634 | Yes |
| 97 | MCM2 | MCM2 Entrez,  Source | MCM2 minichromosome maintenance deficient 2, mitotin (S. cerevisiae) | 19094 | -0.431 | -0.3501 | Yes |
| 98 | MCM4 | MCM4 Entrez,  Source | MCM4 minichromosome maintenance deficient 4 (S. cerevisiae) | 19110 | -0.438 | -0.3332 | Yes |
| 99 | ESPL1 | ESPL1 Entrez,  Source | extra spindle poles like 1 (S. cerevisiae) | 19120 | -0.442 | -0.3159 | Yes |
| 100 | CDC7 | CDC7 Entrez,  Source | CDC7 cell division cycle 7 (S. cerevisiae) | 19160 | -0.463 | -0.2993 | Yes |
| 101 | MCM3 | MCM3 Entrez,  Source | MCM3 minichromosome maintenance deficient 3 (S. cerevisiae) | 19232 | -0.489 | -0.2833 | Yes |
| 102 | CDC25A | CDC25A Entrez,  Source | cell division cycle 25A | 19237 | -0.492 | -0.2637 | Yes |
| 103 | CDC6 | CDC6 Entrez,  Source | CDC6 cell division cycle 6 homolog (S. cerevisiae) | 19241 | -0.495 | -0.2439 | Yes |
| 104 | BUB1 | BUB1 Entrez,  Source | BUB1 budding uninhibited by benzimidazoles 1 homolog (yeast) | 19278 | -0.527 | -0.2245 | Yes |
| 105 | CCNA2 | CCNA2 Entrez,  Source | cyclin A2 | 19327 | -0.557 | -0.2046 | Yes |
| 106 | CDKN2C | CDKN2C Entrez,  Source | cyclin-dependent kinase inhibitor 2C (p18, inhibits CDK4) | 19341 | -0.569 | -0.1824 | Yes |
| 107 | E2F2 | E2F2 Entrez,  Source | E2F transcription factor 2 | 19355 | -0.577 | -0.1598 | Yes |
| 108 | MCM5 | MCM5 Entrez,  Source | MCM5 minichromosome maintenance deficient 5, cell division cycle 46 (S. cerevisiae) | 19364 | -0.590 | -0.1364 | Yes |
| 109 | RBL1 | RBL1 Entrez,  Source | retinoblastoma-like 1 (p107) | 19367 | -0.592 | -0.1127 | Yes |
| 110 | CDC25C | CDC25C Entrez,  Source | cell division cycle 25C | 19398 | -0.634 | -0.0888 | Yes |
| 111 | TTK | TTK Entrez,  Source | TTK protein kinase | 19462 | -0.721 | -0.0630 | Yes |
| 112 | CCNE2 | CCNE2 Entrez,  Source | cyclin E2 | 19501 | -0.835 | -0.0313 | Yes |
| 113 | CDKN1C | CDKN1C Entrez,  Source | cyclin-dependent kinase inhibitor 1C (p57, Kip2) | 19505 | -0.847 | 0.0026 | Yes |
Table: GSEA details [plain text format]

  

Fig 2: KEGG\_CELL\_CYCLE      
 Blue-Pink O' Gram in the Space of the Analyzed GeneSet

  

Fig 3: KEGG\_CELL\_CYCLE: Random ES distribution      
 Gene set null distribution of ES for **KEGG\_CELL\_CYCLE**
