# Supplementary material for: Comprehensive analysis of β-catenin target genes in colorectal carcinoma cell lines with deregulated Wnt/β-catenin signaling
Source: BMC Genomics. 2014 Jan 28;15:74. doi: 10.1186/1471-2164-15-74 (PMC3909937; doi:10.1186/1471-2164-15-74)
Supplement: Additional file 5 — GSEA analysis using the KEGG pathway database. This zipped file contains confirming data of the GSEA analysis. The names of the directories containing the files were composed of the term ‘GSEA’, the name of the cell line, e.g. DLD1, SW480, or LS174T, and the pathway database (KEGG). Please use a web browser to view the files with the name ‘index.html’ in the corresponding directories to start exploring the data. [file 1471-2164-15-74-S5.zip › GSEA_LS174T_KEGG/KEGG_COLORECTAL_CANCER.html]

Details for gene set KEGG\_COLORECTAL\_CANCER[GSEA]

|  || Dataset | LS174T\_collapsed\_to\_symbols.class.cls#b\_versus\_bg.class.cls#b\_versus\_bg\_repos |
| Phenotype | class.cls#b\_versus\_bg\_repos |
| Upregulated in class | 0 |
| GeneSet | KEGG\_COLORECTAL\_CANCER |
| Enrichment Score (ES) | -0.40956306 |
| Normalized Enrichment Score (NES) | -1.4584221 |
| Nominal p-value | 0.013667426 |
| FDR q-value | 0.17089017 |
| FWER p-Value | 0.928 |
Table: GSEA Results Summary

  

Fig 1: Enrichment plot: KEGG\_COLORECTAL\_CANCER      
 Profile of the Running ES Score & Positions of GeneSet Members on the Rank Ordered List

  

| PROBE | GENE SYMBOL | GENE\_TITLE | RANK IN GENE LIST | RANK METRIC SCORE | RUNNING ES | CORE ENRICHMENT || 1 | FOS | FOS Entrez,  Source | v-fos FBJ murine osteosarcoma viral oncogene homolog | 113 | 0.819 | 0.0723 | No |
| 2 | TCF7L1 | TCF7L1 Entrez,  Source | transcription factor 7-like 1 (T-cell specific, HMG-box) | 461 | 0.429 | 0.0955 | No |
| 3 | TGFB2 | TGFB2 Entrez,  Source | transforming growth factor, beta 2 | 799 | 0.339 | 0.1105 | No |
| 4 | PIK3R3 | PIK3R3 Entrez,  Source | phosphoinositide-3-kinase, regulatory subunit 3 (p55, gamma) | 1292 | 0.264 | 0.1105 | No |
| 5 | PIK3CG | PIK3CG Entrez,  Source | phosphoinositide-3-kinase, catalytic, gamma polypeptide | 1867 | 0.216 | 0.1017 | No |
| 6 | TCF7L2 | TCF7L2 Entrez,  Source | transcription factor 7-like 2 (T-cell specific, HMG-box) | 2119 | 0.199 | 0.1078 | No |
| 7 | KRAS | KRAS Entrez,  Source | v-Ki-ras2 Kirsten rat sarcoma viral oncogene homolog | 2305 | 0.189 | 0.1163 | No |
| 8 | PIK3R5 | PIK3R5 Entrez,  Source | phosphoinositide-3-kinase, regulatory subunit 5, p101 | 3094 | 0.154 | 0.0906 | No |
| 9 | PIK3CA | PIK3CA Entrez,  Source | phosphoinositide-3-kinase, catalytic, alpha polypeptide | 3224 | 0.149 | 0.0982 | No |
| 10 | MAPK10 | MAPK10 Entrez,  Source | mitogen-activated protein kinase 10 | 4498 | 0.109 | 0.0433 | No |
| 11 | PIK3R2 | PIK3R2 Entrez,  Source | phosphoinositide-3-kinase, regulatory subunit 2 (p85 beta) | 4617 | 0.106 | 0.0473 | No |
| 12 | PIK3CD | PIK3CD Entrez,  Source | phosphoinositide-3-kinase, catalytic, delta polypeptide | 4686 | 0.104 | 0.0537 | No |
| 13 | MAPK3 | MAPK3 Entrez,  Source | mitogen-activated protein kinase 3 | 4947 | 0.097 | 0.0496 | No |
| 14 | RAC2 | RAC2 Entrez,  Source | ras-related C3 botulinum toxin substrate 2 (rho family, small GTP binding protein Rac2) | 5030 | 0.095 | 0.0545 | No |
| 15 | TGFB3 | TGFB3 Entrez,  Source | transforming growth factor, beta 3 | 5325 | 0.088 | 0.0478 | No |
| 16 | MLH1 | MLH1 Entrez,  Source | mutL homolog 1, colon cancer, nonpolyposis type 2 (E. coli) | 5473 | 0.085 | 0.0483 | No |
| 17 | MAPK8 | MAPK8 Entrez,  Source | mitogen-activated protein kinase 8 | 5571 | 0.082 | 0.0512 | No |
| 18 | APC | APC Entrez,  Source | adenomatosis polyposis coli | 7056 | 0.052 | -0.0199 | No |
| 19 | SMAD2 | SMAD2 Entrez,  Source | SMAD, mothers against DPP homolog 2 (Drosophila) | 7089 | 0.052 | -0.0166 | No |
| 20 | TGFB1 | TGFB1 Entrez,  Source | transforming growth factor, beta 1 (Camurati-Engelmann disease) | 7228 | 0.049 | -0.0190 | No |
| 21 | GSK3B | GSK3B Entrez,  Source | glycogen synthase kinase 3 beta | 8323 | 0.029 | -0.0724 | No |
| 22 | MAP2K1 | MAP2K1 Entrez,  Source | mitogen-activated protein kinase kinase 1 | 8349 | 0.028 | -0.0710 | No |
| 23 | RHOA | RHOA Entrez,  Source | ras homolog gene family, member A | 8558 | 0.024 | -0.0793 | No |
| 24 | MSH3 | MSH3 Entrez,  Source | mutS homolog 3 (E. coli) | 8577 | 0.024 | -0.0779 | No |
| 25 | AKT2 | AKT2 Entrez,  Source | v-akt murine thymoma viral oncogene homolog 2 | 9288 | 0.012 | -0.1132 | No |
| 26 | BAD | BAD Entrez,  Source | BCL2-antagonist of cell death | 9321 | 0.012 | -0.1137 | No |
| 27 | CASP9 | CASP9 Entrez,  Source | caspase 9, apoptosis-related cysteine peptidase | 9688 | 0.006 | -0.1319 | No |
| 28 | RAC1 | RAC1 Entrez,  Source | ras-related C3 botulinum toxin substrate 1 (rho family, small GTP binding protein Rac1) | 9727 | 0.005 | -0.1333 | No |
| 29 | BAX | BAX Entrez,  Source | BCL2-associated X protein | 9736 | 0.005 | -0.1332 | No |
| 30 | JUN | JUN Entrez,  Source | jun oncogene | 9902 | 0.003 | -0.1414 | No |
| 31 | TGFBR2 | TGFBR2 Entrez,  Source | transforming growth factor, beta receptor II (70/80kDa) | 10336 | -0.004 | -0.1632 | No |
| 32 | ARAF | ARAF Entrez,  Source | v-raf murine sarcoma 3611 viral oncogene homolog | 10578 | -0.008 | -0.1748 | No |
| 33 | TGFBR1 | TGFBR1 Entrez,  Source | transforming growth factor, beta receptor I (activin A receptor type II-like kinase, 53kDa) | 11055 | -0.016 | -0.1977 | No |
| 34 | BCL2 | BCL2 Entrez,  Source | B-cell CLL/lymphoma 2 | 11142 | -0.018 | -0.2004 | No |
| 35 | RALGDS | RALGDS Entrez,  Source | ral guanine nucleotide dissociation stimulator | 11981 | -0.033 | -0.2403 | No |
| 36 | BRAF | BRAF Entrez,  Source | v-raf murine sarcoma viral oncogene homolog B1 | 12037 | -0.033 | -0.2399 | No |
| 37 | MAPK1 | MAPK1 Entrez,  Source | mitogen-activated protein kinase 1 | 13089 | -0.053 | -0.2888 | No |
| 38 | RAC3 | RAC3 Entrez,  Source | ras-related C3 botulinum toxin substrate 3 (rho family, small GTP binding protein Rac3) | 13113 | -0.054 | -0.2848 | No |
| 39 | RAF1 | RAF1 Entrez,  Source | v-raf-1 murine leukemia viral oncogene homolog 1 | 13610 | -0.064 | -0.3042 | No |
| 40 | CYCS | CYCS Entrez,  Source | cytochrome c, somatic | 13826 | -0.069 | -0.3086 | No |
| 41 | PIK3R1 | PIK3R1 Entrez,  Source | phosphoinositide-3-kinase, regulatory subunit 1 (p85 alpha) | 13886 | -0.070 | -0.3050 | No |
| 42 | APC2 | APC2 Entrez,  Source | adenomatosis polyposis coli 2 | 14042 | -0.074 | -0.3059 | No |
| 43 | SMAD4 | SMAD4 Entrez,  Source | SMAD, mothers against DPP homolog 4 (Drosophila) | 14289 | -0.079 | -0.3110 | No |
| 44 | DCC | DCC Entrez,  Source | deleted in colorectal carcinoma | 14948 | -0.095 | -0.3356 | No |
| 45 | PIK3CB | PIK3CB Entrez,  Source | phosphoinositide-3-kinase, catalytic, beta polypeptide | 15135 | -0.101 | -0.3356 | No |
| 46 | AKT3 | AKT3 Entrez,  Source | v-akt murine thymoma viral oncogene homolog 3 (protein kinase B, gamma) | 15597 | -0.114 | -0.3483 | No |
| 47 | AXIN1 | AXIN1 Entrez,  Source | axin 1 | 16012 | -0.128 | -0.3574 | No |
| 48 | SMAD3 | SMAD3 Entrez,  Source | SMAD, mothers against DPP homolog 3 (Drosophila) | 16806 | -0.160 | -0.3828 | No |
| 49 | TP53 | TP53 Entrez,  Source | tumor protein p53 (Li-Fraumeni syndrome) | 17097 | -0.175 | -0.3810 | No |
| 50 | MAPK9 | MAPK9 Entrez,  Source | mitogen-activated protein kinase 9 | 17655 | -0.209 | -0.3896 | Yes |
| 51 | MYC | MYC Entrez,  Source | v-myc myelocytomatosis viral oncogene homolog (avian) | 18016 | -0.238 | -0.3854 | Yes |
| 52 | CCND1 | CCND1 Entrez,  Source | cyclin D1 | 18184 | -0.254 | -0.3697 | Yes |
| 53 | AKT1 | AKT1 Entrez,  Source | v-akt murine thymoma viral oncogene homolog 1 | 18283 | -0.266 | -0.3493 | Yes |
| 54 | CASP3 | CASP3 Entrez,  Source | caspase 3, apoptosis-related cysteine peptidase | 18750 | -0.332 | -0.3415 | Yes |
| 55 | MSH2 | MSH2 Entrez,  Source | mutS homolog 2, colon cancer, nonpolyposis type 1 (E. coli) | 18990 | -0.393 | -0.3163 | Yes |
| 56 | MSH6 | MSH6 Entrez,  Source | mutS homolog 6 (E. coli) | 19064 | -0.416 | -0.2804 | Yes |
| 57 | TCF7 | TCF7 Entrez,  Source | transcription factor 7 (T-cell specific, HMG-box) | 19081 | -0.424 | -0.2408 | Yes |
| 58 | LEF1 | LEF1 Entrez,  Source | lymphoid enhancer-binding factor 1 | 19108 | -0.438 | -0.2004 | Yes |
| 59 | BIRC5 | BIRC5 Entrez,  Source | baculoviral IAP repeat-containing 5 (survivin) | 19219 | -0.484 | -0.1598 | Yes |
| 60 | AXIN2 | AXIN2 Entrez,  Source | axin 2 (conductin, axil) | 19287 | -0.533 | -0.1124 | Yes |
| 61 | CTNNB1 | CTNNB1 Entrez,  Source | catenin (cadherin-associated protein), beta 1, 88kDa | 19553 | -1.322 | 0.0002 | Yes |
Table: GSEA details [plain text format]

  

Fig 2: KEGG\_COLORECTAL\_CANCER      
 Blue-Pink O' Gram in the Space of the Analyzed GeneSet

  

Fig 3: KEGG\_COLORECTAL\_CANCER: Random ES distribution      
 Gene set null distribution of ES for **KEGG\_COLORECTAL\_CANCER**
