# Supplementary material for: Comprehensive analysis of β-catenin target genes in colorectal carcinoma cell lines with deregulated Wnt/β-catenin signaling
Source: BMC Genomics. 2014 Jan 28;15:74. doi: 10.1186/1471-2164-15-74 (PMC3909937; doi:10.1186/1471-2164-15-74)
Supplement: Additional file 5 — GSEA analysis using the KEGG pathway database. This zipped file contains confirming data of the GSEA analysis. The names of the directories containing the files were composed of the term ‘GSEA’, the name of the cell line, e.g. DLD1, SW480, or LS174T, and the pathway database (KEGG). Please use a web browser to view the files with the name ‘index.html’ in the corresponding directories to start exploring the data. [file 1471-2164-15-74-S5.zip › GSEA_LS174T_KEGG/KEGG_COMPLEMENT_AND_COAGULATION_CASCADES.html]

Details for gene set KEGG\_COMPLEMENT\_AND\_COAGULATION\_CASCADES[GSEA]

|  || Dataset | LS174T\_collapsed\_to\_symbols.class.cls#b\_versus\_bg.class.cls#b\_versus\_bg\_repos |
| Phenotype | class.cls#b\_versus\_bg\_repos |
| Upregulated in class | 1 |
| GeneSet | KEGG\_COMPLEMENT\_AND\_COAGULATION\_CASCADES |
| Enrichment Score (ES) | 0.46660534 |
| Normalized Enrichment Score (NES) | 1.600853 |
| Nominal p-value | 0.009041592 |
| FDR q-value | 0.0966963 |
| FWER p-Value | 0.702 |
Table: GSEA Results Summary

  

Fig 1: Enrichment plot: KEGG\_COMPLEMENT\_AND\_COAGULATION\_CASCADES      
 Profile of the Running ES Score & Positions of GeneSet Members on the Rank Ordered List

  

| PROBE | GENE SYMBOL | GENE\_TITLE | RANK IN GENE LIST | RANK METRIC SCORE | RUNNING ES | CORE ENRICHMENT || 1 | PLAT | PLAT Entrez,  Source | plasminogen activator, tissue | 112 | 0.820 | 0.0751 | Yes |
| 2 | CFB | CFB Entrez,  Source | complement factor B | 133 | 0.775 | 0.1504 | Yes |
| 3 | BDKRB1 | BDKRB1 Entrez,  Source | bradykinin receptor B1 | 368 | 0.479 | 0.1856 | Yes |
| 4 | C4BPB | C4BPB Entrez,  Source | complement component 4 binding protein, beta | 585 | 0.391 | 0.2130 | Yes |
| 5 | BDKRB2 | BDKRB2 Entrez,  Source | bradykinin receptor B2 | 592 | 0.389 | 0.2510 | Yes |
| 6 | PROS1 | PROS1 Entrez,  Source | protein S (alpha) | 628 | 0.381 | 0.2867 | Yes |
| 7 | SERPINA1 | SERPINA1 Entrez,  Source | serpin peptidase inhibitor, clade A (alpha-1 antiproteinase, antitrypsin), member 1 | 853 | 0.326 | 0.3074 | Yes |
| 8 | SERPINF2 | SERPINF2 Entrez,  Source | serpin peptidase inhibitor, clade F (alpha-2 antiplasmin, pigment epithelium derived factor), member 2 | 912 | 0.315 | 0.3354 | Yes |
| 9 | KNG1 | KNG1 Entrez,  Source | kininogen 1 | 1075 | 0.291 | 0.3557 | Yes |
| 10 | CFI | CFI Entrez,  Source | complement factor I | 1358 | 0.256 | 0.3665 | Yes |
| 11 | PLAUR | PLAUR Entrez,  Source | plasminogen activator, urokinase receptor | 1371 | 0.255 | 0.3910 | Yes |
| 12 | F5 | F5 Entrez,  Source | coagulation factor V (proaccelerin, labile factor) | 1387 | 0.254 | 0.4153 | Yes |
| 13 | C1S | C1S Entrez,  Source | complement component 1, s subcomponent | 1835 | 0.218 | 0.4138 | Yes |
| 14 | F2 | F2 Entrez,  Source | coagulation factor II (thrombin) | 1999 | 0.207 | 0.4259 | Yes |
| 15 | C8G | C8G Entrez,  Source | complement component 8, gamma polypeptide | 2094 | 0.201 | 0.4408 | Yes |
| 16 | PROC | PROC Entrez,  Source | protein C (inactivator of coagulation factors Va and VIIIa) | 2341 | 0.186 | 0.4465 | Yes |
| 17 | C2 | C2 Entrez,  Source | complement component 2 | 2583 | 0.174 | 0.4514 | Yes |
| 18 | C5 | C5 Entrez,  Source | complement component 5 | 2654 | 0.171 | 0.4646 | Yes |
| 19 | F2R | F2R Entrez,  Source | coagulation factor II (thrombin) receptor | 3049 | 0.155 | 0.4597 | Yes |
| 20 | F3 | F3 Entrez,  Source | coagulation factor III (thromboplastin, tissue factor) | 3530 | 0.138 | 0.4487 | Yes |
| 21 | THBD | THBD Entrez,  Source | thrombomodulin | 3558 | 0.138 | 0.4609 | Yes |
| 22 | PLG | PLG Entrez,  Source | plasminogen | 3702 | 0.132 | 0.4666 | Yes |
| 23 | SERPING1 | SERPING1 Entrez,  Source | serpin peptidase inhibitor, clade G (C1 inhibitor), member 1, (angioedema, hereditary) | 4124 | 0.119 | 0.4568 | No |
| 24 | TFPI | TFPI Entrez,  Source | tissue factor pathway inhibitor (lipoprotein-associated coagulation inhibitor) | 4599 | 0.106 | 0.4429 | No |
| 25 | F13A1 | F13A1 Entrez,  Source | coagulation factor XIII, A1 polypeptide | 5273 | 0.089 | 0.4172 | No |
| 26 | C3AR1 | C3AR1 Entrez,  Source | complement component 3a receptor 1 | 5821 | 0.077 | 0.3967 | No |
| 27 | VWF | VWF Entrez,  Source | von Willebrand factor | 5960 | 0.074 | 0.3969 | No |
| 28 | C8B | C8B Entrez,  Source | complement component 8, beta polypeptide | 6124 | 0.070 | 0.3954 | No |
| 29 | MASP2 | MASP2 Entrez,  Source | mannan-binding lectin serine peptidase 2 | 6254 | 0.068 | 0.3955 | No |
| 30 | FGA | FGA Entrez,  Source | fibrinogen alpha chain | 6326 | 0.067 | 0.3984 | No |
| 31 | SERPINE1 | SERPINE1 Entrez,  Source | serpin peptidase inhibitor, clade E (nexin, plasminogen activator inhibitor type 1), member 1 | 7480 | 0.044 | 0.3436 | No |
| 32 | CFD | CFD Entrez,  Source | complement factor D (adipsin) | 7522 | 0.043 | 0.3458 | No |
| 33 | C6 | C6 Entrez,  Source | complement component 6 | 7637 | 0.041 | 0.3440 | No |
| 34 | C1QA | C1QA Entrez,  Source | complement component 1, q subcomponent, A chain | 8460 | 0.026 | 0.3044 | No |
| 35 | CD59 | CD59 Entrez,  Source | CD59 molecule, complement regulatory protein | 8560 | 0.024 | 0.3018 | No |
| 36 | KLKB1 | KLKB1 Entrez,  Source | kallikrein B, plasma (Fletcher factor) 1 | 8988 | 0.017 | 0.2816 | No |
| 37 | C8A | C8A Entrez,  Source | complement component 8, alpha polypeptide | 8992 | 0.017 | 0.2831 | No |
| 38 | MASP1 | MASP1 Entrez,  Source | mannan-binding lectin serine peptidase 1 (C4/C2 activating component of Ra-reactive factor) | 9163 | 0.015 | 0.2758 | No |
| 39 | F13B | F13B Entrez,  Source | coagulation factor XIII, B polypeptide | 9200 | 0.014 | 0.2754 | No |
| 40 | C9 | C9 Entrez,  Source | complement component 9 | 9352 | 0.012 | 0.2688 | No |
| 41 | CPB2 | CPB2 Entrez,  Source | carboxypeptidase B2 (plasma, carboxypeptidase U) | 10184 | -0.001 | 0.2263 | No |
| 42 | F11 | F11 Entrez,  Source | coagulation factor XI (plasma thromboplastin antecedent) | 10784 | -0.011 | 0.1967 | No |
| 43 | CFH | CFH Entrez,  Source | complement factor H | 10873 | -0.013 | 0.1934 | No |
| 44 | FGB | FGB Entrez,  Source | fibrinogen beta chain | 11106 | -0.017 | 0.1832 | No |
| 45 | F12 | F12 Entrez,  Source | coagulation factor XII (Hageman factor) | 12058 | -0.034 | 0.1378 | No |
| 46 | A2M | A2M Entrez,  Source | alpha-2-macroglobulin | 12169 | -0.036 | 0.1357 | No |
| 47 | C7 | C7 Entrez,  Source | complement component 7 | 12248 | -0.037 | 0.1353 | No |
| 48 | F10 | F10 Entrez,  Source | coagulation factor X | 12306 | -0.039 | 0.1362 | No |
| 49 | SERPINA5 | SERPINA5 Entrez,  Source | serpin peptidase inhibitor, clade A (alpha-1 antiproteinase, antitrypsin), member 5 | 12542 | -0.043 | 0.1284 | No |
| 50 | CD46 | CD46 Entrez,  Source | CD46 molecule, complement regulatory protein | 12805 | -0.048 | 0.1197 | No |
| 51 | SERPIND1 | SERPIND1 Entrez,  Source | serpin peptidase inhibitor, clade D (heparin cofactor), member 1 | 13202 | -0.055 | 0.1049 | No |
| 52 | CR1 | CR1 Entrez,  Source | complement component (3b/4b) receptor 1 (Knops blood group) | 13680 | -0.065 | 0.0868 | No |
| 53 | CD55 | CD55 Entrez,  Source | CD55 molecule, decay accelerating factor for complement (Cromer blood group) | 13894 | -0.070 | 0.0828 | No |
| 54 | C4BPA | C4BPA Entrez,  Source | complement component 4 binding protein, alpha | 14975 | -0.096 | 0.0369 | No |
| 55 | C1QC | C1QC Entrez,  Source | complement component 1, q subcomponent, C chain | 15233 | -0.104 | 0.0339 | No |
| 56 | SERPINC1 | SERPINC1 Entrez,  Source | serpin peptidase inhibitor, clade C (antithrombin), member 1 | 15566 | -0.113 | 0.0280 | No |
| 57 | C1QB | C1QB Entrez,  Source | complement component 1, q subcomponent, B chain | 15758 | -0.119 | 0.0299 | No |
| 58 | C5AR1 | C5AR1 Entrez,  Source | complement component 5a receptor 1 | 16093 | -0.131 | 0.0257 | No |
| 59 | MBL2 | MBL2 Entrez,  Source | mannose-binding lectin (protein C) 2, soluble (opsonic defect) | 16296 | -0.139 | 0.0290 | No |
| 60 | PLAU | PLAU Entrez,  Source | plasminogen activator, urokinase | 16526 | -0.148 | 0.0318 | No |
| 61 | F7 | F7 Entrez,  Source | coagulation factor VII (serum prothrombin conversion accelerator) | 16666 | -0.154 | 0.0398 | No |
| 62 | FGG | FGG Entrez,  Source | fibrinogen gamma chain | 17789 | -0.218 | 0.0037 | No |
| 63 | CR2 | CR2 Entrez,  Source | complement component (3d/Epstein Barr virus) receptor 2 | 17824 | -0.220 | 0.0236 | No |
| 64 | F9 | F9 Entrez,  Source | coagulation factor IX (plasma thromboplastic component, Christmas disease, hemophilia B) | 18669 | -0.319 | 0.0117 | No |
| 65 | F8 | F8 Entrez,  Source | coagulation factor VIII, procoagulant component (hemophilia A) | 18807 | -0.342 | 0.0384 | No |
Table: GSEA details [plain text format]

  

Fig 2: KEGG\_COMPLEMENT\_AND\_COAGULATION\_CASCADES      
 Blue-Pink O' Gram in the Space of the Analyzed GeneSet

  

Fig 3: KEGG\_COMPLEMENT\_AND\_COAGULATION\_CASCADES: Random ES distribution      
 Gene set null distribution of ES for **KEGG\_COMPLEMENT\_AND\_COAGULATION\_CASCADES**
