# Supplementary material for: Comprehensive analysis of β-catenin target genes in colorectal carcinoma cell lines with deregulated Wnt/β-catenin signaling
Source: BMC Genomics. 2014 Jan 28;15:74. doi: 10.1186/1471-2164-15-74 (PMC3909937; doi:10.1186/1471-2164-15-74)
Supplement: Additional file 5 — GSEA analysis using the KEGG pathway database. This zipped file contains confirming data of the GSEA analysis. The names of the directories containing the files were composed of the term ‘GSEA’, the name of the cell line, e.g. DLD1, SW480, or LS174T, and the pathway database (KEGG). Please use a web browser to view the files with the name ‘index.html’ in the corresponding directories to start exploring the data. [file 1471-2164-15-74-S5.zip › GSEA_LS174T_KEGG/KEGG_DNA_REPLICATION.html]

Details for gene set KEGG\_DNA\_REPLICATION[GSEA]

|  || Dataset | LS174T\_collapsed\_to\_symbols.class.cls#b\_versus\_bg.class.cls#b\_versus\_bg\_repos |
| Phenotype | class.cls#b\_versus\_bg\_repos |
| Upregulated in class | 0 |
| GeneSet | KEGG\_DNA\_REPLICATION |
| Enrichment Score (ES) | -0.8289616 |
| Normalized Enrichment Score (NES) | -2.5533035 |
| Nominal p-value | 0.0 |
| FDR q-value | 0.0 |
| FWER p-Value | 0.0 |
Table: GSEA Results Summary

  

Fig 1: Enrichment plot: KEGG\_DNA\_REPLICATION      
 Profile of the Running ES Score & Positions of GeneSet Members on the Rank Ordered List

  

| PROBE | GENE SYMBOL | GENE\_TITLE | RANK IN GENE LIST | RANK METRIC SCORE | RUNNING ES | CORE ENRICHMENT || 1 | POLD4 | POLD4 Entrez,  Source | polymerase (DNA-directed), delta 4 | 753 | 0.350 | -0.0132 | No |
| 2 | POLE4 | POLE4 Entrez,  Source | polymerase (DNA-directed), epsilon 4 (p12 subunit) | 2276 | 0.190 | -0.0774 | No |
| 3 | SSBP1 | SSBP1 Entrez,  Source | single-stranded DNA binding protein 1 | 12933 | -0.050 | -0.6196 | No |
| 4 | RFC1 | RFC1 Entrez,  Source | replication factor C (activator 1) 1, 145kDa | 14514 | -0.085 | -0.6944 | No |
| 5 | RNASEH2C | RNASEH2C Entrez,  Source | ribonuclease H2, subunit C | 14637 | -0.088 | -0.6943 | No |
| 6 | RPA3 | RPA3 Entrez,  Source | replication protein A3, 14kDa | 16794 | -0.160 | -0.7931 | No |
| 7 | RNASEH1 | RNASEH1 Entrez,  Source | ribonuclease H1 | 16979 | -0.169 | -0.7903 | No |
| 8 | RPA1 | RPA1 Entrez,  Source | replication protein A1, 70kDa | 17398 | -0.192 | -0.7978 | No |
| 9 | RPA4 | RPA4 Entrez,  Source | replication protein A4, 34kDa | 17533 | -0.201 | -0.7902 | No |
| 10 | POLD2 | POLD2 Entrez,  Source | polymerase (DNA directed), delta 2, regulatory subunit 50kDa | 17923 | -0.230 | -0.7935 | No |
| 11 | RNASEH2A | RNASEH2A Entrez,  Source | ribonuclease H2, subunit A | 18617 | -0.310 | -0.8065 | Yes |
| 12 | POLD1 | POLD1 Entrez,  Source | polymerase (DNA directed), delta 1, catalytic subunit 125kDa | 18666 | -0.318 | -0.7859 | Yes |
| 13 | PCNA | PCNA Entrez,  Source | proliferating cell nuclear antigen | 18712 | -0.326 | -0.7646 | Yes |
| 14 | MCM6 | MCM6 Entrez,  Source | MCM6 minichromosome maintenance deficient 6 (MIS5 homolog, S. pombe) (S. cerevisiae) | 18732 | -0.329 | -0.7418 | Yes |
| 15 | RPA2 | RPA2 Entrez,  Source | replication protein A2, 32kDa | 18880 | -0.361 | -0.7231 | Yes |
| 16 | POLE3 | POLE3 Entrez,  Source | polymerase (DNA directed), epsilon 3 (p17 subunit) | 18960 | -0.385 | -0.6993 | Yes |
| 17 | RFC2 | RFC2 Entrez,  Source | replication factor C (activator 1) 2, 40kDa | 18995 | -0.394 | -0.6725 | Yes |
| 18 | MCM7 | MCM7 Entrez,  Source | MCM7 minichromosome maintenance deficient 7 (S. cerevisiae) | 19014 | -0.400 | -0.6444 | Yes |
| 19 | MCM2 | MCM2 Entrez,  Source | MCM2 minichromosome maintenance deficient 2, mitotin (S. cerevisiae) | 19094 | -0.431 | -0.6173 | Yes |
| 20 | MCM4 | MCM4 Entrez,  Source | MCM4 minichromosome maintenance deficient 4 (S. cerevisiae) | 19110 | -0.438 | -0.5863 | Yes |
| 21 | FEN1 | FEN1 Entrez,  Source | flap structure-specific endonuclease 1 | 19114 | -0.440 | -0.5546 | Yes |
| 22 | RFC3 | RFC3 Entrez,  Source | replication factor C (activator 1) 3, 38kDa | 19190 | -0.476 | -0.5240 | Yes |
| 23 | MCM3 | MCM3 Entrez,  Source | MCM3 minichromosome maintenance deficient 3 (S. cerevisiae) | 19232 | -0.489 | -0.4907 | Yes |
| 24 | PRIM1 | PRIM1 Entrez,  Source | primase, polypeptide 1, 49kDa | 19268 | -0.519 | -0.4549 | Yes |
| 25 | RFC4 | RFC4 Entrez,  Source | replication factor C (activator 1) 4, 37kDa | 19269 | -0.520 | -0.4172 | Yes |
| 26 | POLE | POLE Entrez,  Source | polymerase (DNA directed), epsilon | 19281 | -0.529 | -0.3795 | Yes |
| 27 | LIG1 | LIG1 Entrez,  Source | ligase I, DNA, ATP-dependent | 19298 | -0.542 | -0.3410 | Yes |
| 28 | POLA2 | POLA2 Entrez,  Source | polymerase (DNA directed), alpha 2 (70kD subunit) | 19321 | -0.553 | -0.3021 | Yes |
| 29 | RNASEH2B | RNASEH2B Entrez,  Source | ribonuclease H2, subunit B | 19330 | -0.561 | -0.2619 | Yes |
| 30 | MCM5 | MCM5 Entrez,  Source | MCM5 minichromosome maintenance deficient 5, cell division cycle 46 (S. cerevisiae) | 19364 | -0.590 | -0.2209 | Yes |
| 31 | RFC5 | RFC5 Entrez,  Source | replication factor C (activator 1) 5, 36.5kDa | 19433 | -0.674 | -0.1756 | Yes |
| 32 | POLA1 | POLA1 Entrez,  Source | polymerase (DNA directed), alpha 1 | 19484 | -0.786 | -0.1212 | Yes |
| 33 | POLD3 | POLD3 Entrez,  Source | polymerase (DNA-directed), delta 3, accessory subunit | 19495 | -0.805 | -0.0634 | Yes |
| 34 | POLE2 | POLE2 Entrez,  Source | polymerase (DNA directed), epsilon 2 (p59 subunit) | 19520 | -0.918 | 0.0018 | Yes |
Table: GSEA details [plain text format]

  

Fig 2: KEGG\_DNA\_REPLICATION      
 Blue-Pink O' Gram in the Space of the Analyzed GeneSet

  

Fig 3: KEGG\_DNA\_REPLICATION: Random ES distribution      
 Gene set null distribution of ES for **KEGG\_DNA\_REPLICATION**
