# Supplementary material for: Comprehensive analysis of β-catenin target genes in colorectal carcinoma cell lines with deregulated Wnt/β-catenin signaling
Source: BMC Genomics. 2014 Jan 28;15:74. doi: 10.1186/1471-2164-15-74 (PMC3909937; doi:10.1186/1471-2164-15-74)
Supplement: Additional file 5 — GSEA analysis using the KEGG pathway database. This zipped file contains confirming data of the GSEA analysis. The names of the directories containing the files were composed of the term ‘GSEA’, the name of the cell line, e.g. DLD1, SW480, or LS174T, and the pathway database (KEGG). Please use a web browser to view the files with the name ‘index.html’ in the corresponding directories to start exploring the data. [file 1471-2164-15-74-S5.zip › GSEA_LS174T_KEGG/KEGG_DORSO_VENTRAL_AXIS_FORMATION.html]

Details for gene set KEGG\_DORSO\_VENTRAL\_AXIS\_FORMATION[GSEA]

|  || Dataset | LS174T\_collapsed\_to\_symbols.class.cls#b\_versus\_bg.class.cls#b\_versus\_bg\_repos |
| Phenotype | class.cls#b\_versus\_bg\_repos |
| Upregulated in class | 0 |
| GeneSet | KEGG\_DORSO\_VENTRAL\_AXIS\_FORMATION |
| Enrichment Score (ES) | -0.48794088 |
| Normalized Enrichment Score (NES) | -1.3682873 |
| Nominal p-value | 0.07936508 |
| FDR q-value | 0.23341702 |
| FWER p-Value | 0.99 |
Table: GSEA Results Summary

  

Fig 1: Enrichment plot: KEGG\_DORSO\_VENTRAL\_AXIS\_FORMATION      
 Profile of the Running ES Score & Positions of GeneSet Members on the Rank Ordered List

  

| PROBE | GENE SYMBOL | GENE\_TITLE | RANK IN GENE LIST | RANK METRIC SCORE | RUNNING ES | CORE ENRICHMENT || 1 | KRAS | KRAS Entrez,  Source | v-Ki-ras2 Kirsten rat sarcoma viral oncogene homolog | 2305 | 0.189 | -0.0543 | No |
| 2 | NOTCH2 | NOTCH2 Entrez,  Source | Notch homolog 2 (Drosophila) | 4139 | 0.119 | -0.1080 | No |
| 3 | MAPK3 | MAPK3 Entrez,  Source | mitogen-activated protein kinase 3 | 4947 | 0.097 | -0.1167 | No |
| 4 | FMN2 | FMN2 Entrez,  Source | formin 2 | 5254 | 0.090 | -0.1021 | No |
| 5 | CPEB1 | CPEB1 Entrez,  Source | cytoplasmic polyadenylation element binding protein 1 | 5662 | 0.080 | -0.0959 | No |
| 6 | MAP2K1 | MAP2K1 Entrez,  Source | mitogen-activated protein kinase kinase 1 | 8349 | 0.028 | -0.2238 | No |
| 7 | SPIRE2 | SPIRE2 Entrez,  Source | spire homolog 2 (Drosophila) | 9712 | 0.006 | -0.2916 | No |
| 8 | SOS1 | SOS1 Entrez,  Source | son of sevenless homolog 1 (Drosophila) | 10121 | -0.001 | -0.3123 | No |
| 9 | NOTCH3 | NOTCH3 Entrez,  Source | Notch homolog 3 (Drosophila) | 10947 | -0.014 | -0.3497 | No |
| 10 | GRB2 | GRB2 Entrez,  Source | growth factor receptor-bound protein 2 | 11416 | -0.023 | -0.3659 | No |
| 11 | PIWIL4 | PIWIL4 Entrez,  Source | piwi-like 4 (Drosophila) | 11924 | -0.032 | -0.3812 | No |
| 12 | PIWIL2 | PIWIL2 Entrez,  Source | piwi-like 2 (Drosophila) | 12762 | -0.047 | -0.4081 | No |
| 13 | MAPK1 | MAPK1 Entrez,  Source | mitogen-activated protein kinase 1 | 13089 | -0.053 | -0.4069 | No |
| 14 | ETV6 | ETV6 Entrez,  Source | ets variant gene 6 (TEL oncogene) | 14097 | -0.075 | -0.4332 | Yes |
| 15 | ETV7 | ETV7 Entrez,  Source | ets variant gene 7 (TEL2 oncogene) | 14516 | -0.085 | -0.4260 | Yes |
| 16 | NOTCH4 | NOTCH4 Entrez,  Source | Notch homolog 4 (Drosophila) | 14799 | -0.092 | -0.4094 | Yes |
| 17 | SOS2 | SOS2 Entrez,  Source | son of sevenless homolog 2 (Drosophila) | 14817 | -0.092 | -0.3791 | Yes |
| 18 | ETS1 | ETS1 Entrez,  Source | v-ets erythroblastosis virus E26 oncogene homolog 1 (avian) | 15697 | -0.117 | -0.3846 | Yes |
| 19 | SPIRE1 | SPIRE1 Entrez,  Source | spire homolog 1 (Drosophila) | 17716 | -0.213 | -0.4161 | Yes |
| 20 | PIWIL1 | PIWIL1 Entrez,  Source | piwi-like 1 (Drosophila) | 18054 | -0.241 | -0.3519 | Yes |
| 21 | EGFR | EGFR Entrez,  Source | epidermal growth factor receptor (erythroblastic leukemia viral (v-erb-b) oncogene homolog, avian) | 18782 | -0.337 | -0.2753 | Yes |
| 22 | NOTCH1 | NOTCH1 Entrez,  Source | Notch homolog 1, translocation-associated (Drosophila) | 18982 | -0.391 | -0.1537 | Yes |
| 23 | ETS2 | ETS2 Entrez,  Source | v-ets erythroblastosis virus E26 oncogene homolog 2 (avian) | 19300 | -0.543 | 0.0131 | Yes |
Table: GSEA details [plain text format]

  

Fig 2: KEGG\_DORSO\_VENTRAL\_AXIS\_FORMATION      
 Blue-Pink O' Gram in the Space of the Analyzed GeneSet

  

Fig 3: KEGG\_DORSO\_VENTRAL\_AXIS\_FORMATION: Random ES distribution      
 Gene set null distribution of ES for **KEGG\_DORSO\_VENTRAL\_AXIS\_FORMATION**
