# Supplementary material for: Comprehensive analysis of β-catenin target genes in colorectal carcinoma cell lines with deregulated Wnt/β-catenin signaling
Source: BMC Genomics. 2014 Jan 28;15:74. doi: 10.1186/1471-2164-15-74 (PMC3909937; doi:10.1186/1471-2164-15-74)
Supplement: Additional file 5 — GSEA analysis using the KEGG pathway database. This zipped file contains confirming data of the GSEA analysis. The names of the directories containing the files were composed of the term ‘GSEA’, the name of the cell line, e.g. DLD1, SW480, or LS174T, and the pathway database (KEGG). Please use a web browser to view the files with the name ‘index.html’ in the corresponding directories to start exploring the data. [file 1471-2164-15-74-S5.zip › GSEA_LS174T_KEGG/KEGG_DRUG_METABOLISM_CYTOCHROME_P450.html]

Details for gene set KEGG\_DRUG\_METABOLISM\_CYTOCHROME\_P450[GSEA]

|  || Dataset | LS174T\_collapsed\_to\_symbols.class.cls#b\_versus\_bg.class.cls#b\_versus\_bg\_repos |
| Phenotype | class.cls#b\_versus\_bg\_repos |
| Upregulated in class | 1 |
| GeneSet | KEGG\_DRUG\_METABOLISM\_CYTOCHROME\_P450 |
| Enrichment Score (ES) | 0.61649525 |
| Normalized Enrichment Score (NES) | 2.069546 |
| Nominal p-value | 0.0 |
| FDR q-value | 0.0018265351 |
| FWER p-Value | 0.0050 |
Table: GSEA Results Summary

  

Fig 1: Enrichment plot: KEGG\_DRUG\_METABOLISM\_CYTOCHROME\_P450      
 Profile of the Running ES Score & Positions of GeneSet Members on the Rank Ordered List

  

| PROBE | GENE SYMBOL | GENE\_TITLE | RANK IN GENE LIST | RANK METRIC SCORE | RUNNING ES | CORE ENRICHMENT || 1 | UGT2B28 | UGT2B28 Entrez,  Source | UDP glucuronosyltransferase 2 family, polypeptide B28 | 15 | 1.479 | 0.1065 | Yes |
| 2 | UGT2B17 | UGT2B17 Entrez,  Source | UDP glucuronosyltransferase 2 family, polypeptide B17 | 21 | 1.429 | 0.2098 | Yes |
| 3 | CYP3A5 | CYP3A5 Entrez,  Source | cytochrome P450, family 3, subfamily A, polypeptide 5 | 34 | 1.267 | 0.3011 | Yes |
| 4 | UGT2B15 | UGT2B15 Entrez,  Source | UDP glucuronosyltransferase 2 family, polypeptide B15 | 70 | 0.989 | 0.3710 | Yes |
| 5 | ADH1A | ADH1A Entrez,  Source | alcohol dehydrogenase 1A (class I), alpha polypeptide | 166 | 0.708 | 0.4175 | Yes |
| 6 | ALDH3B2 | ALDH3B2 Entrez,  Source | aldehyde dehydrogenase 3 family, member B2 | 196 | 0.656 | 0.4635 | Yes |
| 7 | FMO5 | FMO5 Entrez,  Source | flavin containing monooxygenase 5 | 258 | 0.584 | 0.5027 | Yes |
| 8 | FMO4 | FMO4 Entrez,  Source | flavin containing monooxygenase 4 | 497 | 0.419 | 0.5209 | Yes |
| 9 | CYP2B6 | CYP2B6 Entrez,  Source | cytochrome P450, family 2, subfamily B, polypeptide 6 | 581 | 0.391 | 0.5450 | Yes |
| 10 | CYP2C18 | CYP2C18 Entrez,  Source | cytochrome P450, family 2, subfamily C, polypeptide 18 | 816 | 0.334 | 0.5572 | Yes |
| 11 | ALDH3B1 | ALDH3B1 Entrez,  Source | aldehyde dehydrogenase 3 family, member B1 | 828 | 0.332 | 0.5808 | Yes |
| 12 | GSTA4 | GSTA4 Entrez,  Source | glutathione S-transferase A4 | 857 | 0.325 | 0.6029 | Yes |
| 13 | ALDH1A3 | ALDH1A3 Entrez,  Source | aldehyde dehydrogenase 1 family, member A3 | 1018 | 0.300 | 0.6165 | Yes |
| 14 | ADH1B | ADH1B Entrez,  Source | alcohol dehydrogenase IB (class I), beta polypeptide | 1618 | 0.234 | 0.6027 | No |
| 15 | CYP2C8 | CYP2C8 Entrez,  Source | cytochrome P450, family 2, subfamily C, polypeptide 8 | 1790 | 0.221 | 0.6100 | No |
| 16 | ALDH3A1 | ALDH3A1 Entrez,  Source | aldehyde dehydrogenase 3 family, memberA1 | 2020 | 0.205 | 0.6131 | No |
| 17 | GSTT1 | GSTT1 Entrez,  Source | glutathione S-transferase theta 1 | 2760 | 0.166 | 0.5873 | No |
| 18 | GSTT2 | GSTT2 Entrez,  Source | glutathione S-transferase theta 2 | 3204 | 0.150 | 0.5755 | No |
| 19 | MGST3 | MGST3 Entrez,  Source | microsomal glutathione S-transferase 3 | 3246 | 0.148 | 0.5841 | No |
| 20 | GSTZ1 | GSTZ1 Entrez,  Source | glutathione transferase zeta 1 (maleylacetoacetate isomerase) | 3500 | 0.140 | 0.5813 | No |
| 21 | CYP1A2 | CYP1A2 Entrez,  Source | cytochrome P450, family 1, subfamily A, polypeptide 2 | 3536 | 0.138 | 0.5895 | No |
| 22 | CYP2E1 | CYP2E1 Entrez,  Source | cytochrome P450, family 2, subfamily E, polypeptide 1 | 3682 | 0.133 | 0.5917 | No |
| 23 | MAOA | MAOA Entrez,  Source | monoamine oxidase A | 3737 | 0.131 | 0.5985 | No |
| 24 | GSTK1 | GSTK1 Entrez,  Source | glutathione S-transferase kappa 1 | 4722 | 0.103 | 0.5555 | No |
| 25 | UGT2A3 | UGT2A3 Entrez,  Source | UDP glucuronosyltransferase 2 family, polypeptide A3 | 4867 | 0.099 | 0.5552 | No |
| 26 | GSTO2 | GSTO2 Entrez,  Source | glutathione S-transferase omega 2 | 5234 | 0.090 | 0.5430 | No |
| 27 | CYP2C9 | CYP2C9 Entrez,  Source | cytochrome P450, family 2, subfamily C, polypeptide 9 | 5445 | 0.085 | 0.5384 | No |
| 28 | CYP2D6 | CYP2D6 Entrez,  Source | cytochrome P450, family 2, subfamily D, polypeptide 6 | 5505 | 0.084 | 0.5415 | No |
| 29 | ADH1C | ADH1C Entrez,  Source | alcohol dehydrogenase 1C (class I), gamma polypeptide | 5527 | 0.083 | 0.5464 | No |
| 30 | AOX1 | AOX1 Entrez,  Source | aldehyde oxidase 1 | 5696 | 0.079 | 0.5435 | No |
| 31 | FMO1 | FMO1 Entrez,  Source | flavin containing monooxygenase 1 | 5787 | 0.077 | 0.5445 | No |
| 32 | GSTM1 | GSTM1 Entrez,  Source | glutathione S-transferase M1 | 6132 | 0.070 | 0.5320 | No |
| 33 | CYP3A4 | CYP3A4 Entrez,  Source | cytochrome P450, family 3, subfamily A, polypeptide 4 | 6476 | 0.063 | 0.5190 | No |
| 34 | FMO2 | FMO2 Entrez,  Source | flavin containing monooxygenase 2 (non-functional) | 6876 | 0.056 | 0.5026 | No |
| 35 | GSTM2 | GSTM2 Entrez,  Source | glutathione S-transferase M2 (muscle) | 7451 | 0.045 | 0.4764 | No |
| 36 | GSTO1 | GSTO1 Entrez,  Source | glutathione S-transferase omega 1 | 7571 | 0.043 | 0.4734 | No |
| 37 | MGST1 | MGST1 Entrez,  Source | microsomal glutathione S-transferase 1 | 7715 | 0.040 | 0.4690 | No |
| 38 | MGST2 | MGST2 Entrez,  Source | microsomal glutathione S-transferase 2 | 8480 | 0.026 | 0.4317 | No |
| 39 | GSTM3 | GSTM3 Entrez,  Source | glutathione S-transferase M3 (brain) | 8860 | 0.020 | 0.4137 | No |
| 40 | MAOB | MAOB Entrez,  Source | monoamine oxidase B | 10309 | -0.004 | 0.3397 | No |
| 41 | UGT1A9 | UGT1A9 Entrez,  Source | UDP glucuronosyltransferase 1 family, polypeptide A9 | 10361 | -0.005 | 0.3374 | No |
| 42 | GSTP1 | GSTP1 Entrez,  Source | glutathione S-transferase pi | 10471 | -0.006 | 0.3322 | No |
| 43 | CYP2A6 | CYP2A6 Entrez,  Source | cytochrome P450, family 2, subfamily A, polypeptide 6 | 10879 | -0.013 | 0.3123 | No |
| 44 | ADH6 | ADH6 Entrez,  Source | alcohol dehydrogenase 6 (class V) | 11401 | -0.023 | 0.2872 | No |
| 45 | ADH7 | ADH7 Entrez,  Source | alcohol dehydrogenase 7 (class IV), mu or sigma polypeptide | 11465 | -0.024 | 0.2857 | No |
| 46 | ADH5 | ADH5 Entrez,  Source | alcohol dehydrogenase 5 (class III), chi polypeptide | 11976 | -0.032 | 0.2619 | No |
| 47 | GSTM5 | GSTM5 Entrez,  Source | glutathione S-transferase M5 | 12355 | -0.040 | 0.2454 | No |
| 48 | CYP2A13 | CYP2A13 Entrez,  Source | cytochrome P450, family 2, subfamily A, polypeptide 13 | 14053 | -0.074 | 0.1638 | No |
| 49 | GSTA1 | GSTA1 Entrez,  Source | glutathione S-transferase A1 | 14534 | -0.085 | 0.1453 | No |
| 50 | CYP3A7 | CYP3A7 Entrez,  Source | cytochrome P450, family 3, subfamily A, polypeptide 7 | 14947 | -0.095 | 0.1311 | No |
| 51 | GSTA3 | GSTA3 Entrez,  Source | glutathione S-transferase A3 | 15029 | -0.097 | 0.1340 | No |
| 52 | GSTM4 | GSTM4 Entrez,  Source | glutathione S-transferase M4 | 15912 | -0.124 | 0.0978 | No |
| 53 | CYP2C19 | CYP2C19 Entrez,  Source | cytochrome P450, family 2, subfamily C, polypeptide 19 | 17104 | -0.175 | 0.0494 | No |
| 54 | ADH4 | ADH4 Entrez,  Source | alcohol dehydrogenase 4 (class II), pi polypeptide | 17566 | -0.204 | 0.0405 | No |
| 55 | FMO3 | FMO3 Entrez,  Source | flavin containing monooxygenase 3 | 17689 | -0.212 | 0.0496 | No |
| 56 | CYP3A43 | CYP3A43 Entrez,  Source | cytochrome P450, family 3, subfamily A, polypeptide 43 | 17928 | -0.230 | 0.0541 | No |
| 57 | UGT2B4 | UGT2B4 Entrez,  Source | UDP glucuronosyltransferase 2 family, polypeptide B4 | 19031 | -0.405 | 0.0269 | No |
Table: GSEA details [plain text format]

  

Fig 2: KEGG\_DRUG\_METABOLISM\_CYTOCHROME\_P450      
 Blue-Pink O' Gram in the Space of the Analyzed GeneSet

  

Fig 3: KEGG\_DRUG\_METABOLISM\_CYTOCHROME\_P450: Random ES distribution      
 Gene set null distribution of ES for **KEGG\_DRUG\_METABOLISM\_CYTOCHROME\_P450**
