# Supplementary material for: Comprehensive analysis of β-catenin target genes in colorectal carcinoma cell lines with deregulated Wnt/β-catenin signaling
Source: BMC Genomics. 2014 Jan 28;15:74. doi: 10.1186/1471-2164-15-74 (PMC3909937; doi:10.1186/1471-2164-15-74)
Supplement: Additional file 5 — GSEA analysis using the KEGG pathway database. This zipped file contains confirming data of the GSEA analysis. The names of the directories containing the files were composed of the term ‘GSEA’, the name of the cell line, e.g. DLD1, SW480, or LS174T, and the pathway database (KEGG). Please use a web browser to view the files with the name ‘index.html’ in the corresponding directories to start exploring the data. [file 1471-2164-15-74-S5.zip › GSEA_LS174T_KEGG/KEGG_DRUG_METABOLISM_OTHER_ENZYMES.html]

Details for gene set KEGG\_DRUG\_METABOLISM\_OTHER\_ENZYMES[GSEA]

|  || Dataset | LS174T\_collapsed\_to\_symbols.class.cls#b\_versus\_bg.class.cls#b\_versus\_bg\_repos |
| Phenotype | class.cls#b\_versus\_bg\_repos |
| Upregulated in class | 1 |
| GeneSet | KEGG\_DRUG\_METABOLISM\_OTHER\_ENZYMES |
| Enrichment Score (ES) | 0.5902112 |
| Normalized Enrichment Score (NES) | 1.8039181 |
| Nominal p-value | 0.0018416207 |
| FDR q-value | 0.034119084 |
| FWER p-Value | 0.15 |
Table: GSEA Results Summary

  

Fig 1: Enrichment plot: KEGG\_DRUG\_METABOLISM\_OTHER\_ENZYMES      
 Profile of the Running ES Score & Positions of GeneSet Members on the Rank Ordered List

  

| PROBE | GENE SYMBOL | GENE\_TITLE | RANK IN GENE LIST | RANK METRIC SCORE | RUNNING ES | CORE ENRICHMENT || 1 | UGT2B28 | UGT2B28 Entrez,  Source | UDP glucuronosyltransferase 2 family, polypeptide B28 | 15 | 1.479 | 0.1396 | Yes |
| 2 | UGT2B17 | UGT2B17 Entrez,  Source | UDP glucuronosyltransferase 2 family, polypeptide B17 | 21 | 1.429 | 0.2749 | Yes |
| 3 | CYP3A5 | CYP3A5 Entrez,  Source | cytochrome P450, family 3, subfamily A, polypeptide 5 | 34 | 1.267 | 0.3946 | Yes |
| 4 | UGT2B15 | UGT2B15 Entrez,  Source | UDP glucuronosyltransferase 2 family, polypeptide B15 | 70 | 0.989 | 0.4867 | Yes |
| 5 | CDA | CDA Entrez,  Source | cytidine deaminase | 97 | 0.874 | 0.5683 | Yes |
| 6 | NAT2 | NAT2 Entrez,  Source | N-acetyltransferase 2 (arylamine N-acetyltransferase) | 616 | 0.382 | 0.5781 | Yes |
| 7 | NAT1 | NAT1 Entrez,  Source | N-acetyltransferase 1 (arylamine N-acetyltransferase) | 952 | 0.309 | 0.5902 | Yes |
| 8 | CES1 | CES1 Entrez,  Source | carboxylesterase 1 (monocyte/macrophage serine esterase 1) | 1795 | 0.221 | 0.5681 | No |
| 9 | XDH | XDH Entrez,  Source | xanthine dehydrogenase | 2493 | 0.178 | 0.5492 | No |
| 10 | CES2 | CES2 Entrez,  Source | carboxylesterase 2 (intestine, liver) | 2940 | 0.159 | 0.5415 | No |
| 11 | UGT2A3 | UGT2A3 Entrez,  Source | UDP glucuronosyltransferase 2 family, polypeptide A3 | 4867 | 0.099 | 0.4522 | No |
| 12 | TPMT | TPMT Entrez,  Source | thiopurine S-methyltransferase | 5128 | 0.092 | 0.4477 | No |
| 13 | CYP3A4 | CYP3A4 Entrez,  Source | cytochrome P450, family 3, subfamily A, polypeptide 4 | 6476 | 0.063 | 0.3847 | No |
| 14 | UCKL1 | UCKL1 Entrez,  Source | uridine-cytidine kinase 1-like 1 | 6543 | 0.062 | 0.3872 | No |
| 15 | ITPA | ITPA Entrez,  Source | inosine triphosphatase (nucleoside triphosphate pyrophosphatase) | 7246 | 0.049 | 0.3559 | No |
| 16 | UPP1 | UPP1 Entrez,  Source | uridine phosphorylase 1 | 7418 | 0.046 | 0.3515 | No |
| 17 | DPYD | DPYD Entrez,  Source | dihydropyrimidine dehydrogenase | 9709 | 0.006 | 0.2347 | No |
| 18 | IMPDH2 | IMPDH2 Entrez,  Source | IMP (inosine monophosphate) dehydrogenase 2 | 9774 | 0.005 | 0.2318 | No |
| 19 | UGT1A9 | UGT1A9 Entrez,  Source | UDP glucuronosyltransferase 1 family, polypeptide A9 | 10361 | -0.005 | 0.2023 | No |
| 20 | CYP2A6 | CYP2A6 Entrez,  Source | cytochrome P450, family 2, subfamily A, polypeptide 6 | 10879 | -0.013 | 0.1770 | No |
| 21 | HPRT1 | HPRT1 Entrez,  Source | hypoxanthine phosphoribosyltransferase 1 (Lesch-Nyhan syndrome) | 12713 | -0.046 | 0.0875 | No |
| 22 | GUSB | GUSB Entrez,  Source | glucuronidase, beta | 12719 | -0.046 | 0.0917 | No |
| 23 | UPB1 | UPB1 Entrez,  Source | ureidopropionase, beta | 12891 | -0.049 | 0.0876 | No |
| 24 | CYP2A13 | CYP2A13 Entrez,  Source | cytochrome P450, family 2, subfamily A, polypeptide 13 | 14053 | -0.074 | 0.0351 | No |
| 25 | IMPDH1 | IMPDH1 Entrez,  Source | IMP (inosine monophosphate) dehydrogenase 1 | 14405 | -0.082 | 0.0249 | No |
| 26 | TK2 | TK2 Entrez,  Source | thymidine kinase 2, mitochondrial | 14498 | -0.084 | 0.0282 | No |
| 27 | CYP3A7 | CYP3A7 Entrez,  Source | cytochrome P450, family 3, subfamily A, polypeptide 7 | 14947 | -0.095 | 0.0143 | No |
| 28 | UPP2 | UPP2 Entrez,  Source | uridine phosphorylase 2 | 16412 | -0.143 | -0.0471 | No |
| 29 | UCK2 | UCK2 Entrez,  Source | uridine-cytidine kinase 2 | 16950 | -0.167 | -0.0588 | No |
| 30 | DPYS | DPYS Entrez,  Source | dihydropyrimidinase | 17252 | -0.183 | -0.0569 | No |
| 31 | UCK1 | UCK1 Entrez,  Source | uridine-cytidine kinase 1 | 17906 | -0.228 | -0.0688 | No |
| 32 | CYP3A43 | CYP3A43 Entrez,  Source | cytochrome P450, family 3, subfamily A, polypeptide 43 | 17928 | -0.230 | -0.0480 | No |
| 33 | GMPS | GMPS Entrez,  Source | guanine monphosphate synthetase | 18003 | -0.236 | -0.0294 | No |
| 34 | UMPS | UMPS Entrez,  Source | uridine monophosphate synthetase (orotate phosphoribosyl transferase and orotidine-5'-decarboxylase) | 18198 | -0.256 | -0.0150 | No |
| 35 | UGT2B4 | UGT2B4 Entrez,  Source | UDP glucuronosyltransferase 2 family, polypeptide B4 | 19031 | -0.405 | -0.0191 | No |
| 36 | TK1 | TK1 Entrez,  Source | thymidine kinase 1, soluble | 19220 | -0.485 | 0.0172 | No |
Table: GSEA details [plain text format]

  

Fig 2: KEGG\_DRUG\_METABOLISM\_OTHER\_ENZYMES      
 Blue-Pink O' Gram in the Space of the Analyzed GeneSet

  

Fig 3: KEGG\_DRUG\_METABOLISM\_OTHER\_ENZYMES: Random ES distribution      
 Gene set null distribution of ES for **KEGG\_DRUG\_METABOLISM\_OTHER\_ENZYMES**
