# Supplementary material for: Comprehensive analysis of β-catenin target genes in colorectal carcinoma cell lines with deregulated Wnt/β-catenin signaling
Source: BMC Genomics. 2014 Jan 28;15:74. doi: 10.1186/1471-2164-15-74 (PMC3909937; doi:10.1186/1471-2164-15-74)
Supplement: Additional file 5 — GSEA analysis using the KEGG pathway database. This zipped file contains confirming data of the GSEA analysis. The names of the directories containing the files were composed of the term ‘GSEA’, the name of the cell line, e.g. DLD1, SW480, or LS174T, and the pathway database (KEGG). Please use a web browser to view the files with the name ‘index.html’ in the corresponding directories to start exploring the data. [file 1471-2164-15-74-S5.zip › GSEA_LS174T_KEGG/KEGG_GLYCOSAMINOGLYCAN_BIOSYNTHESIS_HEPARAN_SULFATE.html]

Details for gene set KEGG\_GLYCOSAMINOGLYCAN\_BIOSYNTHESIS\_HEPARAN\_SULFATE[GSEA]

|  || Dataset | LS174T\_collapsed\_to\_symbols.class.cls#b\_versus\_bg.class.cls#b\_versus\_bg\_repos |
| Phenotype | class.cls#b\_versus\_bg\_repos |
| Upregulated in class | 0 |
| GeneSet | KEGG\_GLYCOSAMINOGLYCAN\_BIOSYNTHESIS\_HEPARAN\_SULFATE |
| Enrichment Score (ES) | -0.48832384 |
| Normalized Enrichment Score (NES) | -1.4431446 |
| Nominal p-value | 0.075630255 |
| FDR q-value | 0.17890385 |
| FWER p-Value | 0.953 |
Table: GSEA Results Summary

  

Fig 1: Enrichment plot: KEGG\_GLYCOSAMINOGLYCAN\_BIOSYNTHESIS\_HEPARAN\_SULFATE      
 Profile of the Running ES Score & Positions of GeneSet Members on the Rank Ordered List

  

| PROBE | GENE SYMBOL | GENE\_TITLE | RANK IN GENE LIST | RANK METRIC SCORE | RUNNING ES | CORE ENRICHMENT || 1 | EXT1 | EXT1 Entrez,  Source | exostoses (multiple) 1 | 424 | 0.445 | 0.0852 | No |
| 2 | B3GALT6 | B3GALT6 Entrez,  Source | UDP-Gal:betaGal beta 1,3-galactosyltransferase polypeptide 6 | 2948 | 0.159 | -0.0059 | No |
| 3 | EXTL1 | EXTL1 Entrez,  Source | exostoses (multiple)-like 1 | 3154 | 0.152 | 0.0201 | No |
| 4 | HS3ST3A1 | HS3ST3A1 Entrez,  Source | heparan sulfate (glucosamine) 3-O-sulfotransferase 3A1 | 3198 | 0.150 | 0.0540 | No |
| 5 | HS3ST2 | HS3ST2 Entrez,  Source | heparan sulfate (glucosamine) 3-O-sulfotransferase 2 | 3331 | 0.146 | 0.0822 | No |
| 6 | B4GALT7 | B4GALT7 Entrez,  Source | xylosylprotein beta 1,4-galactosyltransferase, polypeptide 7 (galactosyltransferase I) | 5858 | 0.076 | -0.0289 | No |
| 7 | HS6ST3 | HS6ST3 Entrez,  Source | heparan sulfate 6-O-sulfotransferase 3 | 6948 | 0.054 | -0.0717 | No |
| 8 | NDST1 | NDST1 Entrez,  Source | N-deacetylase/N-sulfotransferase (heparan glucosaminyl) 1 | 6950 | 0.054 | -0.0587 | No |
| 9 | NDST4 | NDST4 Entrez,  Source | N-deacetylase/N-sulfotransferase (heparan glucosaminyl) 4 | 7770 | 0.039 | -0.0912 | No |
| 10 | HS3ST3B1 | HS3ST3B1 Entrez,  Source | heparan sulfate (glucosamine) 3-O-sulfotransferase 3B1 | 8043 | 0.034 | -0.0970 | No |
| 11 | HS3ST5 | HS3ST5 Entrez,  Source | heparan sulfate (glucosamine) 3-O-sulfotransferase 5 | 8125 | 0.032 | -0.0933 | No |
| 12 | B3GAT3 | B3GAT3 Entrez,  Source | beta-1,3-glucuronyltransferase 3 (glucuronosyltransferase I) | 9421 | 0.010 | -0.1572 | No |
| 13 | XYLT1 | XYLT1 Entrez,  Source | xylosyltransferase I | 10708 | -0.010 | -0.2206 | No |
| 14 | GLCE | GLCE Entrez,  Source | UDP-glucuronic acid epimerase | 13528 | -0.062 | -0.3499 | No |
| 15 | EXT2 | EXT2 Entrez,  Source | exostoses (multiple) 2 | 14518 | -0.085 | -0.3802 | No |
| 16 | NDST2 | NDST2 Entrez,  Source | N-deacetylase/N-sulfotransferase (heparan glucosaminyl) 2 | 15115 | -0.100 | -0.3866 | No |
| 17 | HS6ST1 | HS6ST1 Entrez,  Source | heparan sulfate 6-O-sulfotransferase 1 | 17103 | -0.175 | -0.4463 | Yes |
| 18 | NDST3 | NDST3 Entrez,  Source | N-deacetylase/N-sulfotransferase (heparan glucosaminyl) 3 | 17305 | -0.185 | -0.4121 | Yes |
| 19 | EXTL2 | EXTL2 Entrez,  Source | exostoses (multiple)-like 2 | 17483 | -0.198 | -0.3736 | Yes |
| 20 | XYLT2 | XYLT2 Entrez,  Source | xylosyltransferase II | 17765 | -0.216 | -0.3360 | Yes |
| 21 | EXTL3 | EXTL3 Entrez,  Source | exostoses (multiple)-like 3 | 17866 | -0.224 | -0.2873 | Yes |
| 22 | HS2ST1 | HS2ST1 Entrez,  Source | heparan sulfate 2-O-sulfotransferase 1 | 17898 | -0.227 | -0.2343 | Yes |
| 23 | HS6ST2 | HS6ST2 Entrez,  Source | heparan sulfate 6-O-sulfotransferase 2 | 17953 | -0.231 | -0.1815 | Yes |
| 24 | HS3ST1 | HS3ST1 Entrez,  Source | heparan sulfate (glucosamine) 3-O-sulfotransferase 1 | 19282 | -0.529 | -0.1223 | Yes |
| 25 | B3GAT1 | B3GAT1 Entrez,  Source | beta-1,3-glucuronyltransferase 1 (glucuronosyltransferase P) | 19340 | -0.567 | 0.0111 | Yes |
Table: GSEA details [plain text format]

  

Fig 2: KEGG\_GLYCOSAMINOGLYCAN\_BIOSYNTHESIS\_HEPARAN\_SULFATE      
 Blue-Pink O' Gram in the Space of the Analyzed GeneSet

  

Fig 3: KEGG\_GLYCOSAMINOGLYCAN\_BIOSYNTHESIS\_HEPARAN\_SULFATE: Random ES distribution      
 Gene set null distribution of ES for **KEGG\_GLYCOSAMINOGLYCAN\_BIOSYNTHESIS\_HEPARAN\_SULFATE**
