# Supplementary material for: Comprehensive analysis of β-catenin target genes in colorectal carcinoma cell lines with deregulated Wnt/β-catenin signaling
Source: BMC Genomics. 2014 Jan 28;15:74. doi: 10.1186/1471-2164-15-74 (PMC3909937; doi:10.1186/1471-2164-15-74)
Supplement: Additional file 5 — GSEA analysis using the KEGG pathway database. This zipped file contains confirming data of the GSEA analysis. The names of the directories containing the files were composed of the term ‘GSEA’, the name of the cell line, e.g. DLD1, SW480, or LS174T, and the pathway database (KEGG). Please use a web browser to view the files with the name ‘index.html’ in the corresponding directories to start exploring the data. [file 1471-2164-15-74-S5.zip › GSEA_LS174T_KEGG/KEGG_HISTIDINE_METABOLISM.html]

Details for gene set KEGG\_HISTIDINE\_METABOLISM[GSEA]

|  || Dataset | LS174T\_collapsed\_to\_symbols.class.cls#b\_versus\_bg.class.cls#b\_versus\_bg\_repos |
| Phenotype | class.cls#b\_versus\_bg\_repos |
| Upregulated in class | 1 |
| GeneSet | KEGG\_HISTIDINE\_METABOLISM |
| Enrichment Score (ES) | 0.5209918 |
| Normalized Enrichment Score (NES) | 1.491598 |
| Nominal p-value | 0.03811252 |
| FDR q-value | 0.1641402 |
| FWER p-Value | 0.946 |
Table: GSEA Results Summary

  

Fig 1: Enrichment plot: KEGG\_HISTIDINE\_METABOLISM      
 Profile of the Running ES Score & Positions of GeneSet Members on the Rank Ordered List

  

| PROBE | GENE SYMBOL | GENE\_TITLE | RANK IN GENE LIST | RANK METRIC SCORE | RUNNING ES | CORE ENRICHMENT || 1 | ABP1 | ABP1 Entrez,  Source | amiloride binding protein 1 (amine oxidase (copper-containing)) | 117 | 0.808 | 0.1569 | Yes |
| 2 | ALDH3B2 | ALDH3B2 Entrez,  Source | aldehyde dehydrogenase 3 family, member B2 | 196 | 0.656 | 0.2851 | Yes |
| 3 | ALDH3B1 | ALDH3B1 Entrez,  Source | aldehyde dehydrogenase 3 family, member B1 | 828 | 0.332 | 0.3197 | Yes |
| 4 | ALDH1A3 | ALDH1A3 Entrez,  Source | aldehyde dehydrogenase 1 family, member A3 | 1018 | 0.300 | 0.3705 | Yes |
| 5 | DDC | DDC Entrez,  Source | dopa decarboxylase (aromatic L-amino acid decarboxylase) | 1562 | 0.239 | 0.3909 | Yes |
| 6 | HNMT | HNMT Entrez,  Source | histamine N-methyltransferase | 2016 | 0.205 | 0.4091 | Yes |
| 7 | ALDH3A1 | ALDH3A1 Entrez,  Source | aldehyde dehydrogenase 3 family, memberA1 | 2020 | 0.205 | 0.4503 | Yes |
| 8 | HEMK1 | HEMK1 Entrez,  Source | HemK methyltransferase family member 1 | 2243 | 0.192 | 0.4776 | Yes |
| 9 | ACY3 | ACY3 Entrez,  Source | aspartoacylase (aminocyclase) 3 | 2578 | 0.175 | 0.4956 | Yes |
| 10 | CNDP1 | CNDP1 Entrez,  Source | carnosine dipeptidase 1 (metallopeptidase M20 family) | 3013 | 0.157 | 0.5050 | Yes |
| 11 | ALDH2 | ALDH2 Entrez,  Source | aldehyde dehydrogenase 2 family (mitochondrial) | 3716 | 0.132 | 0.4956 | Yes |
| 12 | MAOA | MAOA Entrez,  Source | monoamine oxidase A | 3737 | 0.131 | 0.5210 | Yes |
| 13 | ASPA | ASPA Entrez,  Source | aspartoacylase (Canavan disease) | 4696 | 0.104 | 0.4928 | No |
| 14 | LCMT1 | LCMT1 Entrez,  Source | leucine carboxyl methyltransferase 1 | 5604 | 0.081 | 0.4627 | No |
| 15 | FTCD | FTCD Entrez,  Source | formiminotransferase cyclodeaminase | 6109 | 0.071 | 0.4512 | No |
| 16 | ALDH3A2 | ALDH3A2 Entrez,  Source | aldehyde dehydrogenase 3 family, member A2 | 6565 | 0.062 | 0.4403 | No |
| 17 | HDC | HDC Entrez,  Source | histidine decarboxylase | 8972 | 0.018 | 0.3207 | No |
| 18 | LCMT2 | LCMT2 Entrez,  Source | leucine carboxyl methyltransferase 2 | 10148 | -0.001 | 0.2607 | No |
| 19 | MAOB | MAOB Entrez,  Source | monoamine oxidase B | 10309 | -0.004 | 0.2532 | No |
| 20 | AMDHD1 | AMDHD1 Entrez,  Source | amidohydrolase domain containing 1 | 10343 | -0.004 | 0.2524 | No |
| 21 | ALDH9A1 | ALDH9A1 Entrez,  Source | aldehyde dehydrogenase 9 family, member A1 | 11333 | -0.021 | 0.2061 | No |
| 22 | HAL | HAL Entrez,  Source | histidine ammonia-lyase | 13482 | -0.061 | 0.1084 | No |
| 23 | WBSCR22 | WBSCR22 Entrez,  Source | Williams Beuren syndrome chromosome region 22 | 14291 | -0.079 | 0.0830 | No |
| 24 | UROC1 | UROC1 Entrez,  Source | urocanase domain containing 1 | 15593 | -0.114 | 0.0393 | No |
| 25 | ALDH7A1 | ALDH7A1 Entrez,  Source | aldehyde dehydrogenase 7 family, member A1 | 17316 | -0.186 | -0.0114 | No |
| 26 | ALDH1B1 | ALDH1B1 Entrez,  Source | aldehyde dehydrogenase 1 family, member B1 | 18592 | -0.306 | -0.0151 | No |
| 27 | METTL6 | METTL6 Entrez,  Source | methyltransferase like 6 | 18673 | -0.319 | 0.0452 | No |
Table: GSEA details [plain text format]

  

Fig 2: KEGG\_HISTIDINE\_METABOLISM      
 Blue-Pink O' Gram in the Space of the Analyzed GeneSet

  

Fig 3: KEGG\_HISTIDINE\_METABOLISM: Random ES distribution      
 Gene set null distribution of ES for **KEGG\_HISTIDINE\_METABOLISM**
