# Supplementary material for: Comprehensive analysis of β-catenin target genes in colorectal carcinoma cell lines with deregulated Wnt/β-catenin signaling
Source: BMC Genomics. 2014 Jan 28;15:74. doi: 10.1186/1471-2164-15-74 (PMC3909937; doi:10.1186/1471-2164-15-74)
Supplement: Additional file 5 — GSEA analysis using the KEGG pathway database. This zipped file contains confirming data of the GSEA analysis. The names of the directories containing the files were composed of the term ‘GSEA’, the name of the cell line, e.g. DLD1, SW480, or LS174T, and the pathway database (KEGG). Please use a web browser to view the files with the name ‘index.html’ in the corresponding directories to start exploring the data. [file 1471-2164-15-74-S5.zip › GSEA_LS174T_KEGG/KEGG_HOMOLOGOUS_RECOMBINATION.html]

Details for gene set KEGG\_HOMOLOGOUS\_RECOMBINATION[GSEA]

|  || Dataset | LS174T\_collapsed\_to\_symbols.class.cls#b\_versus\_bg.class.cls#b\_versus\_bg\_repos |
| Phenotype | class.cls#b\_versus\_bg\_repos |
| Upregulated in class | 0 |
| GeneSet | KEGG\_HOMOLOGOUS\_RECOMBINATION |
| Enrichment Score (ES) | -0.7808149 |
| Normalized Enrichment Score (NES) | -2.2776926 |
| Nominal p-value | 0.0 |
| FDR q-value | 0.0 |
| FWER p-Value | 0.0 |
Table: GSEA Results Summary

  

Fig 1: Enrichment plot: KEGG\_HOMOLOGOUS\_RECOMBINATION      
 Profile of the Running ES Score & Positions of GeneSet Members on the Rank Ordered List

  

| PROBE | GENE SYMBOL | GENE\_TITLE | RANK IN GENE LIST | RANK METRIC SCORE | RUNNING ES | CORE ENRICHMENT || 1 | POLD4 | POLD4 Entrez,  Source | polymerase (DNA-directed), delta 4 | 753 | 0.350 | 0.0051 | No |
| 2 | MUS81 | MUS81 Entrez,  Source | MUS81 endonuclease homolog (S. cerevisiae) | 7774 | 0.039 | -0.3494 | No |
| 3 | TOP3B | TOP3B Entrez,  Source | topoisomerase (DNA) III beta | 9115 | 0.015 | -0.4161 | No |
| 4 | RAD50 | RAD50 Entrez,  Source | RAD50 homolog (S. cerevisiae) | 9222 | 0.014 | -0.4199 | No |
| 5 | RAD52 | RAD52 Entrez,  Source | RAD52 homolog (S. cerevisiae) | 11873 | -0.031 | -0.5517 | No |
| 6 | SSBP1 | SSBP1 Entrez,  Source | single-stranded DNA binding protein 1 | 12933 | -0.050 | -0.5996 | No |
| 7 | SHFM1 | SHFM1 Entrez,  Source | split hand/foot malformation (ectrodactyly) type 1 | 13101 | -0.053 | -0.6015 | No |
| 8 | NBN | NBN Entrez,  Source | nibrin | 14237 | -0.078 | -0.6499 | No |
| 9 | RPA3 | RPA3 Entrez,  Source | replication protein A3, 14kDa | 16794 | -0.160 | -0.7609 | Yes |
| 10 | EME1 | EME1 Entrez,  Source | essential meiotic endonuclease 1 homolog 1 (S. pombe) | 17028 | -0.171 | -0.7515 | Yes |
| 11 | RPA1 | RPA1 Entrez,  Source | replication protein A1, 70kDa | 17398 | -0.192 | -0.7464 | Yes |
| 12 | RPA4 | RPA4 Entrez,  Source | replication protein A4, 34kDa | 17533 | -0.201 | -0.7282 | Yes |
| 13 | POLD2 | POLD2 Entrez,  Source | polymerase (DNA directed), delta 2, regulatory subunit 50kDa | 17923 | -0.230 | -0.7195 | Yes |
| 14 | XRCC2 | XRCC2 Entrez,  Source | X-ray repair complementing defective repair in Chinese hamster cells 2 | 18152 | -0.251 | -0.6999 | Yes |
| 15 | TOP3A | TOP3A Entrez,  Source | topoisomerase (DNA) III alpha | 18466 | -0.286 | -0.6802 | Yes |
| 16 | POLD1 | POLD1 Entrez,  Source | polymerase (DNA directed), delta 1, catalytic subunit 125kDa | 18666 | -0.318 | -0.6507 | Yes |
| 17 | RPA2 | RPA2 Entrez,  Source | replication protein A2, 32kDa | 18880 | -0.361 | -0.6166 | Yes |
| 18 | RAD51C | RAD51C Entrez,  Source | RAD51 homolog C (S. cerevisiae) | 18886 | -0.363 | -0.5715 | Yes |
| 19 | MRE11A | MRE11A Entrez,  Source | MRE11 meiotic recombination 11 homolog A (S. cerevisiae) | 19026 | -0.404 | -0.5282 | Yes |
| 20 | XRCC3 | XRCC3 Entrez,  Source | X-ray repair complementing defective repair in Chinese hamster cells 3 | 19189 | -0.476 | -0.4772 | Yes |
| 21 | RAD51 | RAD51 Entrez,  Source | RAD51 homolog (RecA homolog, E. coli) (S. cerevisiae) | 19205 | -0.481 | -0.4180 | Yes |
| 22 | BRCA2 | BRCA2 Entrez,  Source | breast cancer 2, early onset | 19344 | -0.571 | -0.3539 | Yes |
| 23 | RAD54L | RAD54L Entrez,  Source | RAD54-like (S. cerevisiae) | 19359 | -0.584 | -0.2818 | Yes |
| 24 | BLM | BLM Entrez,  Source | Bloom syndrome | 19434 | -0.675 | -0.2015 | Yes |
| 25 | POLD3 | POLD3 Entrez,  Source | polymerase (DNA-directed), delta 3, accessory subunit | 19495 | -0.805 | -0.1041 | Yes |
| 26 | RAD54B | RAD54B Entrez,  Source | RAD54 homolog B (S. cerevisiae) | 19507 | -0.860 | 0.0025 | Yes |
Table: GSEA details [plain text format]

  

Fig 2: KEGG\_HOMOLOGOUS\_RECOMBINATION      
 Blue-Pink O' Gram in the Space of the Analyzed GeneSet

  

Fig 3: KEGG\_HOMOLOGOUS\_RECOMBINATION: Random ES distribution      
 Gene set null distribution of ES for **KEGG\_HOMOLOGOUS\_RECOMBINATION**
