# Supplementary material for: Comprehensive analysis of β-catenin target genes in colorectal carcinoma cell lines with deregulated Wnt/β-catenin signaling
Source: BMC Genomics. 2014 Jan 28;15:74. doi: 10.1186/1471-2164-15-74 (PMC3909937; doi:10.1186/1471-2164-15-74)
Supplement: Additional file 5 — GSEA analysis using the KEGG pathway database. This zipped file contains confirming data of the GSEA analysis. The names of the directories containing the files were composed of the term ‘GSEA’, the name of the cell line, e.g. DLD1, SW480, or LS174T, and the pathway database (KEGG). Please use a web browser to view the files with the name ‘index.html’ in the corresponding directories to start exploring the data. [file 1471-2164-15-74-S5.zip › GSEA_LS174T_KEGG/KEGG_HYPERTROPHIC_CARDIOMYOPATHY_HCM.html]

Details for gene set KEGG\_HYPERTROPHIC\_CARDIOMYOPATHY\_HCM[GSEA]

|  || Dataset | LS174T\_collapsed\_to\_symbols.class.cls#b\_versus\_bg.class.cls#b\_versus\_bg\_repos |
| Phenotype | class.cls#b\_versus\_bg\_repos |
| Upregulated in class | 1 |
| GeneSet | KEGG\_HYPERTROPHIC\_CARDIOMYOPATHY\_HCM |
| Enrichment Score (ES) | 0.4315181 |
| Normalized Enrichment Score (NES) | 1.5381292 |
| Nominal p-value | 0.013536379 |
| FDR q-value | 0.15534423 |
| FWER p-Value | 0.878 |
Table: GSEA Results Summary

  

Fig 1: Enrichment plot: KEGG\_HYPERTROPHIC\_CARDIOMYOPATHY\_HCM      
 Profile of the Running ES Score & Positions of GeneSet Members on the Rank Ordered List

  

| PROBE | GENE SYMBOL | GENE\_TITLE | RANK IN GENE LIST | RANK METRIC SCORE | RUNNING ES | CORE ENRICHMENT || 1 | TNNC1 | TNNC1 Entrez,  Source | troponin C type 1 (slow) | 12 | 1.732 | 0.1586 | Yes |
| 2 | CACNA2D4 | CACNA2D4 Entrez,  Source | calcium channel, voltage-dependent, alpha 2/delta subunit 4 | 478 | 0.425 | 0.1737 | Yes |
| 3 | PRKAB2 | PRKAB2 Entrez,  Source | protein kinase, AMP-activated, beta 2 non-catalytic subunit | 711 | 0.359 | 0.1948 | Yes |
| 4 | TGFB2 | TGFB2 Entrez,  Source | transforming growth factor, beta 2 | 799 | 0.339 | 0.2215 | Yes |
| 5 | CACNG8 | CACNG8 Entrez,  Source | calcium channel, voltage-dependent, gamma subunit 8 | 981 | 0.306 | 0.2403 | Yes |
| 6 | PRKAA2 | PRKAA2 Entrez,  Source | protein kinase, AMP-activated, alpha 2 catalytic subunit | 1012 | 0.301 | 0.2664 | Yes |
| 7 | ITGB6 | ITGB6 Entrez,  Source | integrin, beta 6 | 1229 | 0.271 | 0.2803 | Yes |
| 8 | PRKAA1 | PRKAA1 Entrez,  Source | protein kinase, AMP-activated, alpha 1 catalytic subunit | 1450 | 0.248 | 0.2918 | Yes |
| 9 | ITGB4 | ITGB4 Entrez,  Source | integrin, beta 4 | 1605 | 0.235 | 0.3055 | Yes |
| 10 | LMNA | LMNA Entrez,  Source | lamin A/C | 1640 | 0.232 | 0.3250 | Yes |
| 11 | ITGAV | ITGAV Entrez,  Source | integrin, alpha V (vitronectin receptor, alpha polypeptide, antigen CD51) | 1649 | 0.231 | 0.3459 | Yes |
| 12 | ITGA9 | ITGA9 Entrez,  Source | integrin, alpha 9 | 1695 | 0.228 | 0.3646 | Yes |
| 13 | CACNG2 | CACNG2 Entrez,  Source | calcium channel, voltage-dependent, gamma subunit 2 | 1843 | 0.217 | 0.3770 | Yes |
| 14 | PRKAB1 | PRKAB1 Entrez,  Source | protein kinase, AMP-activated, beta 1 non-catalytic subunit | 1966 | 0.210 | 0.3900 | Yes |
| 15 | PRKAG1 | PRKAG1 Entrez,  Source | protein kinase, AMP-activated, gamma 1 non-catalytic subunit | 2800 | 0.165 | 0.3624 | Yes |
| 16 | RYR2 | RYR2 Entrez,  Source | ryanodine receptor 2 (cardiac) | 2967 | 0.158 | 0.3684 | Yes |
| 17 | ITGA3 | ITGA3 Entrez,  Source | integrin, alpha 3 (antigen CD49C, alpha 3 subunit of VLA-3 receptor) | 3135 | 0.152 | 0.3738 | Yes |
| 18 | PRKAG2 | PRKAG2 Entrez,  Source | protein kinase, AMP-activated, gamma 2 non-catalytic subunit | 3183 | 0.151 | 0.3852 | Yes |
| 19 | TPM1 | TPM1 Entrez,  Source | tropomyosin 1 (alpha) | 3287 | 0.147 | 0.3934 | Yes |
| 20 | CACNG5 | CACNG5 Entrez,  Source | calcium channel, voltage-dependent, gamma subunit 5 | 3436 | 0.141 | 0.3988 | Yes |
| 21 | MYL2 | MYL2 Entrez,  Source | myosin, light chain 2, regulatory, cardiac, slow | 3614 | 0.136 | 0.4022 | Yes |
| 22 | CACNG3 | CACNG3 Entrez,  Source | calcium channel, voltage-dependent, gamma subunit 3 | 3635 | 0.135 | 0.4136 | Yes |
| 23 | CACNG1 | CACNG1 Entrez,  Source | calcium channel, voltage-dependent, gamma subunit 1 | 3869 | 0.127 | 0.4133 | Yes |
| 24 | CACNG6 | CACNG6 Entrez,  Source | calcium channel, voltage-dependent, gamma subunit 6 | 3956 | 0.125 | 0.4203 | Yes |
| 25 | SGCG | SGCG Entrez,  Source | sarcoglycan, gamma (35kDa dystrophin-associated glycoprotein) | 3962 | 0.125 | 0.4315 | Yes |
| 26 | CACNB2 | CACNB2 Entrez,  Source | calcium channel, voltage-dependent, beta 2 subunit | 4362 | 0.112 | 0.4214 | No |
| 27 | CACNA1S | CACNA1S Entrez,  Source | calcium channel, voltage-dependent, L type, alpha 1S subunit | 4423 | 0.111 | 0.4285 | No |
| 28 | ITGB1 | ITGB1 Entrez,  Source | integrin, beta 1 (fibronectin receptor, beta polypeptide, antigen CD29 includes MDF2, MSK12) | 4865 | 0.099 | 0.4149 | No |
| 29 | TGFB3 | TGFB3 Entrez,  Source | transforming growth factor, beta 3 | 5325 | 0.088 | 0.3994 | No |
| 30 | SLC8A1 | SLC8A1 Entrez,  Source | solute carrier family 8 (sodium/calcium exchanger), member 1 | 5523 | 0.083 | 0.3969 | No |
| 31 | CACNB4 | CACNB4 Entrez,  Source | calcium channel, voltage-dependent, beta 4 subunit | 5830 | 0.076 | 0.3882 | No |
| 32 | TPM2 | TPM2 Entrez,  Source | tropomyosin 2 (beta) | 5920 | 0.075 | 0.3905 | No |
| 33 | CACNB3 | CACNB3 Entrez,  Source | calcium channel, voltage-dependent, beta 3 subunit | 6298 | 0.067 | 0.3773 | No |
| 34 | ITGA5 | ITGA5 Entrez,  Source | integrin, alpha 5 (fibronectin receptor, alpha polypeptide) | 6530 | 0.062 | 0.3712 | No |
| 35 | ITGA8 | ITGA8 Entrez,  Source | integrin, alpha 8 | 6894 | 0.055 | 0.3577 | No |
| 36 | TGFB1 | TGFB1 Entrez,  Source | transforming growth factor, beta 1 (Camurati-Engelmann disease) | 7228 | 0.049 | 0.3451 | No |
| 37 | LAMA2 | LAMA2 Entrez,  Source | laminin, alpha 2 (merosin, congenital muscular dystrophy) | 7610 | 0.042 | 0.3294 | No |
| 38 | ITGA2B | ITGA2B Entrez,  Source | integrin, alpha 2b (platelet glycoprotein IIb of IIb/IIIa complex, antigen CD41) | 7695 | 0.040 | 0.3288 | No |
| 39 | IGF1 | IGF1 Entrez,  Source | insulin-like growth factor 1 (somatomedin C) | 7808 | 0.039 | 0.3266 | No |
| 40 | ITGB5 | ITGB5 Entrez,  Source | integrin, beta 5 | 8046 | 0.034 | 0.3175 | No |
| 41 | ACTG1 | ACTG1 Entrez,  Source | actin, gamma 1 | 8191 | 0.031 | 0.3130 | No |
| 42 | CACNG7 | CACNG7 Entrez,  Source | calcium channel, voltage-dependent, gamma subunit 7 | 8249 | 0.030 | 0.3128 | No |
| 43 | ITGA4 | ITGA4 Entrez,  Source | integrin, alpha 4 (antigen CD49D, alpha 4 subunit of VLA-4 receptor) | 8311 | 0.029 | 0.3123 | No |
| 44 | TTN | TTN Entrez,  Source | titin | 8533 | 0.025 | 0.3032 | No |
| 45 | CACNG4 | CACNG4 Entrez,  Source | calcium channel, voltage-dependent, gamma subunit 4 | 8624 | 0.023 | 0.3008 | No |
| 46 | MYBPC3 | MYBPC3 Entrez,  Source | myosin binding protein C, cardiac | 9070 | 0.016 | 0.2794 | No |
| 47 | ITGB8 | ITGB8 Entrez,  Source | integrin, beta 8 | 9618 | 0.007 | 0.2520 | No |
| 48 | ACTB | ACTB Entrez,  Source | actin, beta | 9874 | 0.003 | 0.2392 | No |
| 49 | ITGA10 | ITGA10 Entrez,  Source | integrin, alpha 10 | 10033 | 0.001 | 0.2311 | No |
| 50 | ACTC1 | ACTC1 Entrez,  Source | actin, alpha, cardiac muscle 1 | 10108 | -0.000 | 0.2274 | No |
| 51 | EMD | EMD Entrez,  Source | emerin (Emery-Dreifuss muscular dystrophy) | 10409 | -0.005 | 0.2124 | No |
| 52 | ATP2A2 | ATP2A2 Entrez,  Source | ATPase, Ca++ transporting, cardiac muscle, slow twitch 2 | 10923 | -0.014 | 0.1874 | No |
| 53 | SGCD | SGCD Entrez,  Source | sarcoglycan, delta (35kDa dystrophin-associated glycoprotein) | 10946 | -0.014 | 0.1876 | No |
| 54 | TPM3 | TPM3 Entrez,  Source | tropomyosin 3 | 11224 | -0.019 | 0.1751 | No |
| 55 | ITGA11 | ITGA11 Entrez,  Source | integrin, alpha 11 | 11271 | -0.020 | 0.1746 | No |
| 56 | TNF | TNF Entrez,  Source | tumor necrosis factor (TNF superfamily, member 2) | 11429 | -0.023 | 0.1687 | No |
| 57 | CACNB1 | CACNB1 Entrez,  Source | calcium channel, voltage-dependent, beta 1 subunit | 11453 | -0.024 | 0.1697 | No |
| 58 | MYH7 | MYH7 Entrez,  Source | myosin, heavy chain 7, cardiac muscle, beta | 11597 | -0.026 | 0.1647 | No |
| 59 | CACNA1F | CACNA1F Entrez,  Source | calcium channel, voltage-dependent, alpha 1F subunit | 11860 | -0.031 | 0.1541 | No |
| 60 | PRKAG3 | PRKAG3 Entrez,  Source | protein kinase, AMP-activated, gamma 3 non-catalytic subunit | 12588 | -0.044 | 0.1208 | No |
| 61 | SGCA | SGCA Entrez,  Source | sarcoglycan, alpha (50kDa dystrophin-associated glycoprotein) | 12813 | -0.048 | 0.1137 | No |
| 62 | ITGA6 | ITGA6 Entrez,  Source | integrin, alpha 6 | 12869 | -0.049 | 0.1154 | No |
| 63 | CACNA2D1 | CACNA2D1 Entrez,  Source | calcium channel, voltage-dependent, alpha 2/delta subunit 1 | 13069 | -0.053 | 0.1100 | No |
| 64 | SGCB | SGCB Entrez,  Source | sarcoglycan, beta (43kDa dystrophin-associated glycoprotein) | 13305 | -0.058 | 0.1033 | No |
| 65 | MYL3 | MYL3 Entrez,  Source | myosin, light chain 3, alkali; ventricular, skeletal, slow | 13439 | -0.061 | 0.1020 | No |
| 66 | DMD | DMD Entrez,  Source | dystrophin (muscular dystrophy, Duchenne and Becker types) | 13468 | -0.061 | 0.1062 | No |
| 67 | CACNA2D3 | CACNA2D3 Entrez,  Source | calcium channel, voltage-dependent, alpha 2/delta 3 subunit | 13976 | -0.072 | 0.0868 | No |
| 68 | CACNA1D | CACNA1D Entrez,  Source | calcium channel, voltage-dependent, L type, alpha 1D subunit | 13987 | -0.072 | 0.0929 | No |
| 69 | DES | DES Entrez,  Source | desmin | 14078 | -0.074 | 0.0951 | No |
| 70 | ACE | ACE Entrez,  Source | angiotensin I converting enzyme (peptidyl-dipeptidase A) 1 | 14502 | -0.084 | 0.0811 | No |
| 71 | IL6 | IL6 Entrez,  Source | interleukin 6 (interferon, beta 2) | 14928 | -0.095 | 0.0680 | No |
| 72 | TNNT2 | TNNT2 Entrez,  Source | troponin T type 2 (cardiac) | 15371 | -0.108 | 0.0552 | No |
| 73 | MYH6 | MYH6 Entrez,  Source | myosin, heavy chain 6, cardiac muscle, alpha (cardiomyopathy, hypertrophic 1) | 15396 | -0.108 | 0.0640 | No |
| 74 | ITGB3 | ITGB3 Entrez,  Source | integrin, beta 3 (platelet glycoprotein IIIa, antigen CD61) | 15569 | -0.113 | 0.0655 | No |
| 75 | ITGA2 | ITGA2 Entrez,  Source | integrin, alpha 2 (CD49B, alpha 2 subunit of VLA-2 receptor) | 15877 | -0.123 | 0.0610 | No |
| 76 | CACNA1C | CACNA1C Entrez,  Source | calcium channel, voltage-dependent, L type, alpha 1C subunit | 16330 | -0.140 | 0.0507 | No |
| 77 | TPM4 | TPM4 Entrez,  Source | tropomyosin 4 | 16410 | -0.143 | 0.0598 | No |
| 78 | ITGA7 | ITGA7 Entrez,  Source | integrin, alpha 7 | 16731 | -0.157 | 0.0577 | No |
| 79 | ITGB7 | ITGB7 Entrez,  Source | integrin, beta 7 | 17647 | -0.208 | 0.0299 | No |
| 80 | CACNA2D2 | CACNA2D2 Entrez,  Source | calcium channel, voltage-dependent, alpha 2/delta subunit 2 | 17753 | -0.216 | 0.0443 | No |
| 81 | TNNI3 | TNNI3 Entrez,  Source | troponin I type 3 (cardiac) | 17921 | -0.229 | 0.0568 | No |
| 82 | DAG1 | DAG1 Entrez,  Source | dystroglycan 1 (dystrophin-associated glycoprotein 1) | 18517 | -0.295 | 0.0534 | No |
Table: GSEA details [plain text format]

  

Fig 2: KEGG\_HYPERTROPHIC\_CARDIOMYOPATHY\_HCM      
 Blue-Pink O' Gram in the Space of the Analyzed GeneSet

  

Fig 3: KEGG\_HYPERTROPHIC\_CARDIOMYOPATHY\_HCM: Random ES distribution      
 Gene set null distribution of ES for **KEGG\_HYPERTROPHIC\_CARDIOMYOPATHY\_HCM**
