# Supplementary material for: Comprehensive analysis of β-catenin target genes in colorectal carcinoma cell lines with deregulated Wnt/β-catenin signaling
Source: BMC Genomics. 2014 Jan 28;15:74. doi: 10.1186/1471-2164-15-74 (PMC3909937; doi:10.1186/1471-2164-15-74)
Supplement: Additional file 5 — GSEA analysis using the KEGG pathway database. This zipped file contains confirming data of the GSEA analysis. The names of the directories containing the files were composed of the term ‘GSEA’, the name of the cell line, e.g. DLD1, SW480, or LS174T, and the pathway database (KEGG). Please use a web browser to view the files with the name ‘index.html’ in the corresponding directories to start exploring the data. [file 1471-2164-15-74-S5.zip › GSEA_LS174T_KEGG/KEGG_LINOLEIC_ACID_METABOLISM.html]

Details for gene set KEGG\_LINOLEIC\_ACID\_METABOLISM[GSEA]

|  || Dataset | LS174T\_collapsed\_to\_symbols.class.cls#b\_versus\_bg.class.cls#b\_versus\_bg\_repos |
| Phenotype | class.cls#b\_versus\_bg\_repos |
| Upregulated in class | 1 |
| GeneSet | KEGG\_LINOLEIC\_ACID\_METABOLISM |
| Enrichment Score (ES) | 0.58446723 |
| Normalized Enrichment Score (NES) | 1.679946 |
| Nominal p-value | 0.015037594 |
| FDR q-value | 0.07689478 |
| FWER p-Value | 0.448 |
Table: GSEA Results Summary

  

Fig 1: Enrichment plot: KEGG\_LINOLEIC\_ACID\_METABOLISM      
 Profile of the Running ES Score & Positions of GeneSet Members on the Rank Ordered List

  

| PROBE | GENE SYMBOL | GENE\_TITLE | RANK IN GENE LIST | RANK METRIC SCORE | RUNNING ES | CORE ENRICHMENT || 1 | AKR1B10 | AKR1B10 Entrez,  Source | aldo-keto reductase family 1, member B10 (aldose reductase) | 9 | 2.063 | 0.2764 | Yes |
| 2 | CYP3A5 | CYP3A5 Entrez,  Source | cytochrome P450, family 3, subfamily A, polypeptide 5 | 34 | 1.267 | 0.4453 | Yes |
| 3 | PLA2G10 | PLA2G10 Entrez,  Source | phospholipase A2, group X | 111 | 0.821 | 0.5516 | Yes |
| 4 | CYP2C18 | CYP2C18 Entrez,  Source | cytochrome P450, family 2, subfamily C, polypeptide 18 | 816 | 0.334 | 0.5604 | Yes |
| 5 | PLA2G6 | PLA2G6 Entrez,  Source | phospholipase A2, group VI (cytosolic, calcium-independent) | 1098 | 0.287 | 0.5845 | Yes |
| 6 | CYP2C8 | CYP2C8 Entrez,  Source | cytochrome P450, family 2, subfamily C, polypeptide 8 | 1790 | 0.221 | 0.5788 | No |
| 7 | PLA2G2F | PLA2G2F Entrez,  Source | phospholipase A2, group IIF | 2425 | 0.182 | 0.5707 | No |
| 8 | CYP2J2 | CYP2J2 Entrez,  Source | cytochrome P450, family 2, subfamily J, polypeptide 2 | 2847 | 0.163 | 0.5711 | No |
| 9 | CYP1A2 | CYP1A2 Entrez,  Source | cytochrome P450, family 1, subfamily A, polypeptide 2 | 3536 | 0.138 | 0.5544 | No |
| 10 | CYP2E1 | CYP2E1 Entrez,  Source | cytochrome P450, family 2, subfamily E, polypeptide 1 | 3682 | 0.133 | 0.5648 | No |
| 11 | CYP2C9 | CYP2C9 Entrez,  Source | cytochrome P450, family 2, subfamily C, polypeptide 9 | 5445 | 0.085 | 0.4860 | No |
| 12 | PLA2G12A | PLA2G12A Entrez,  Source | phospholipase A2, group XIIA | 5935 | 0.074 | 0.4710 | No |
| 13 | CYP3A4 | CYP3A4 Entrez,  Source | cytochrome P450, family 3, subfamily A, polypeptide 4 | 6476 | 0.063 | 0.4518 | No |
| 14 | PLA2G5 | PLA2G5 Entrez,  Source | phospholipase A2, group V | 10163 | -0.001 | 0.2633 | No |
| 15 | PLA2G12B | PLA2G12B Entrez,  Source | phospholipase A2, group XIIB | 10181 | -0.001 | 0.2626 | No |
| 16 | PLA2G4B | PLA2G4B Entrez,  Source | phospholipase A2, group IVB (cytosolic) | 12128 | -0.035 | 0.1677 | No |
| 17 | ALOX15 | ALOX15 Entrez,  Source | arachidonate 15-lipoxygenase | 13709 | -0.066 | 0.0956 | No |
| 18 | PLA2G3 | PLA2G3 Entrez,  Source | phospholipase A2, group III | 14302 | -0.079 | 0.0759 | No |
| 19 | CYP3A7 | CYP3A7 Entrez,  Source | cytochrome P450, family 3, subfamily A, polypeptide 7 | 14947 | -0.095 | 0.0558 | No |
| 20 | PLA2G2D | PLA2G2D Entrez,  Source | phospholipase A2, group IID | 15256 | -0.105 | 0.0540 | No |
| 21 | PLA2G2A | PLA2G2A Entrez,  Source | phospholipase A2, group IIA (platelets, synovial fluid) | 16521 | -0.147 | 0.0091 | No |
| 22 | PLA2G2E | PLA2G2E Entrez,  Source | phospholipase A2, group IIE | 16698 | -0.155 | 0.0209 | No |
| 23 | CYP2C19 | CYP2C19 Entrez,  Source | cytochrome P450, family 2, subfamily C, polypeptide 19 | 17104 | -0.175 | 0.0236 | No |
| 24 | PLA2G4A | PLA2G4A Entrez,  Source | phospholipase A2, group IVA (cytosolic, calcium-dependent) | 17896 | -0.227 | 0.0136 | No |
| 25 | CYP3A43 | CYP3A43 Entrez,  Source | cytochrome P450, family 3, subfamily A, polypeptide 43 | 17928 | -0.230 | 0.0429 | No |
| 26 | PLA2G1B | PLA2G1B Entrez,  Source | phospholipase A2, group IB (pancreas) | 18566 | -0.301 | 0.0507 | No |
Table: GSEA details [plain text format]

  

Fig 2: KEGG\_LINOLEIC\_ACID\_METABOLISM      
 Blue-Pink O' Gram in the Space of the Analyzed GeneSet

  

Fig 3: KEGG\_LINOLEIC\_ACID\_METABOLISM: Random ES distribution      
 Gene set null distribution of ES for **KEGG\_LINOLEIC\_ACID\_METABOLISM**
