# Supplementary material for: Comprehensive analysis of β-catenin target genes in colorectal carcinoma cell lines with deregulated Wnt/β-catenin signaling
Source: BMC Genomics. 2014 Jan 28;15:74. doi: 10.1186/1471-2164-15-74 (PMC3909937; doi:10.1186/1471-2164-15-74)
Supplement: Additional file 5 — GSEA analysis using the KEGG pathway database. This zipped file contains confirming data of the GSEA analysis. The names of the directories containing the files were composed of the term ‘GSEA’, the name of the cell line, e.g. DLD1, SW480, or LS174T, and the pathway database (KEGG). Please use a web browser to view the files with the name ‘index.html’ in the corresponding directories to start exploring the data. [file 1471-2164-15-74-S5.zip › GSEA_LS174T_KEGG/KEGG_NITROGEN_METABOLISM.html]

Details for gene set KEGG\_NITROGEN\_METABOLISM[GSEA]

|  || Dataset | LS174T\_collapsed\_to\_symbols.class.cls#b\_versus\_bg.class.cls#b\_versus\_bg\_repos |
| Phenotype | class.cls#b\_versus\_bg\_repos |
| Upregulated in class | 1 |
| GeneSet | KEGG\_NITROGEN\_METABOLISM |
| Enrichment Score (ES) | 0.546352 |
| Normalized Enrichment Score (NES) | 1.4958061 |
| Nominal p-value | 0.034351144 |
| FDR q-value | 0.1685566 |
| FWER p-Value | 0.942 |
Table: GSEA Results Summary

  

Fig 1: Enrichment plot: KEGG\_NITROGEN\_METABOLISM      
 Profile of the Running ES Score & Positions of GeneSet Members on the Rank Ordered List

  

| PROBE | GENE SYMBOL | GENE\_TITLE | RANK IN GENE LIST | RANK METRIC SCORE | RUNNING ES | CORE ENRICHMENT || 1 | CA1 | CA1 Entrez,  Source | carbonic anhydrase I | 11 | 1.744 | 0.2918 | Yes |
| 2 | CA13 | CA13 Entrez,  Source | carbonic anhydrase XIII | 223 | 0.626 | 0.3860 | Yes |
| 3 | CA12 | CA12 Entrez,  Source | carbonic anhydrase XII | 481 | 0.424 | 0.4439 | Yes |
| 4 | CA3 | CA3 Entrez,  Source | carbonic anhydrase III, muscle specific | 617 | 0.382 | 0.5011 | Yes |
| 5 | CTH | CTH Entrez,  Source | cystathionase (cystathionine gamma-lyase) | 825 | 0.333 | 0.5464 | Yes |
| 6 | CA7 | CA7 Entrez,  Source | carbonic anhydrase VII | 1958 | 0.210 | 0.5236 | No |
| 7 | CA6 | CA6 Entrez,  Source | carbonic anhydrase VI | 3490 | 0.140 | 0.4687 | No |
| 8 | CA5B | CA5B Entrez,  Source | carbonic anhydrase VB, mitochondrial | 3704 | 0.132 | 0.4800 | No |
| 9 | CA4 | CA4 Entrez,  Source | carbonic anhydrase IV | 4916 | 0.098 | 0.4343 | No |
| 10 | CA14 | CA14 Entrez,  Source | carbonic anhydrase XIV | 5456 | 0.085 | 0.4210 | No |
| 11 | GLS | GLS Entrez,  Source | glutaminase | 6688 | 0.059 | 0.3679 | No |
| 12 | CA5A | CA5A Entrez,  Source | carbonic anhydrase VA, mitochondrial | 8155 | 0.032 | 0.2982 | No |
| 13 | AMT | AMT Entrez,  Source | aminomethyltransferase | 11819 | -0.030 | 0.1157 | No |
| 14 | GLUL | GLUL Entrez,  Source | glutamate-ammonia ligase (glutamine synthetase) | 11906 | -0.031 | 0.1166 | No |
| 15 | GLUD1 | GLUD1 Entrez,  Source | glutamate dehydrogenase 1 | 12310 | -0.039 | 0.1024 | No |
| 16 | HAL | HAL Entrez,  Source | histidine ammonia-lyase | 13482 | -0.061 | 0.0528 | No |
| 17 | GLUD2 | GLUD2 Entrez,  Source | glutamate dehydrogenase 2 | 14354 | -0.080 | 0.0217 | No |
| 18 | CPS1 | CPS1 Entrez,  Source | carbamoyl-phosphate synthetase 1, mitochondrial | 14876 | -0.094 | 0.0107 | No |
| 19 | CA2 | CA2 Entrez,  Source | carbonic anhydrase II | 16164 | -0.133 | -0.0328 | No |
| 20 | CA8 | CA8 Entrez,  Source | carbonic anhydrase VIII | 16790 | -0.160 | -0.0380 | No |
| 21 | ASNS | ASNS Entrez,  Source | asparagine synthetase | 18293 | -0.267 | -0.0702 | No |
| 22 | GLS2 | GLS2 Entrez,  Source | glutaminase 2 (liver, mitochondrial) | 18509 | -0.294 | -0.0319 | No |
| 23 | CA9 | CA9 Entrez,  Source | carbonic anhydrase IX | 19254 | -0.510 | 0.0155 | No |
Table: GSEA details [plain text format]

  

Fig 2: KEGG\_NITROGEN\_METABOLISM      
 Blue-Pink O' Gram in the Space of the Analyzed GeneSet

  

Fig 3: KEGG\_NITROGEN\_METABOLISM: Random ES distribution      
 Gene set null distribution of ES for **KEGG\_NITROGEN\_METABOLISM**
