# Supplementary material for: Comprehensive analysis of β-catenin target genes in colorectal carcinoma cell lines with deregulated Wnt/β-catenin signaling
Source: BMC Genomics. 2014 Jan 28;15:74. doi: 10.1186/1471-2164-15-74 (PMC3909937; doi:10.1186/1471-2164-15-74)
Supplement: Additional file 5 — GSEA analysis using the KEGG pathway database. This zipped file contains confirming data of the GSEA analysis. The names of the directories containing the files were composed of the term ‘GSEA’, the name of the cell line, e.g. DLD1, SW480, or LS174T, and the pathway database (KEGG). Please use a web browser to view the files with the name ‘index.html’ in the corresponding directories to start exploring the data. [file 1471-2164-15-74-S5.zip › GSEA_LS174T_KEGG/KEGG_NOD_LIKE_RECEPTOR_SIGNALING_PATHWAY.html]

Details for gene set KEGG\_NOD\_LIKE\_RECEPTOR\_SIGNALING\_PATHWAY[GSEA]

|  || Dataset | LS174T\_collapsed\_to\_symbols.class.cls#b\_versus\_bg.class.cls#b\_versus\_bg\_repos |
| Phenotype | class.cls#b\_versus\_bg\_repos |
| Upregulated in class | 1 |
| GeneSet | KEGG\_NOD\_LIKE\_RECEPTOR\_SIGNALING\_PATHWAY |
| Enrichment Score (ES) | 0.5035124 |
| Normalized Enrichment Score (NES) | 1.6105015 |
| Nominal p-value | 0.015238095 |
| FDR q-value | 0.0975636 |
| FWER p-Value | 0.675 |
Table: GSEA Results Summary

  

Fig 1: Enrichment plot: KEGG\_NOD\_LIKE\_RECEPTOR\_SIGNALING\_PATHWAY      
 Profile of the Running ES Score & Positions of GeneSet Members on the Rank Ordered List

  

| PROBE | GENE SYMBOL | GENE\_TITLE | RANK IN GENE LIST | RANK METRIC SCORE | RUNNING ES | CORE ENRICHMENT || 1 | CASP1 | CASP1 Entrez,  Source | caspase 1, apoptosis-related cysteine peptidase (interleukin 1, beta, convertase) | 99 | 0.871 | 0.1088 | Yes |
| 2 | CASP5 | CASP5 Entrez,  Source | caspase 5, apoptosis-related cysteine peptidase | 254 | 0.589 | 0.1779 | Yes |
| 3 | IL18 | IL18 Entrez,  Source | interleukin 18 (interferon-gamma-inducing factor) | 275 | 0.563 | 0.2505 | Yes |
| 4 | CXCL1 | CXCL1 Entrez,  Source | chemokine (C-X-C motif) ligand 1 (melanoma growth stimulating activity, alpha) | 549 | 0.399 | 0.2886 | Yes |
| 5 | BIRC3 | BIRC3 Entrez,  Source | baculoviral IAP repeat-containing 3 | 604 | 0.386 | 0.3362 | Yes |
| 6 | CCL8 | CCL8 Entrez,  Source | chemokine (C-C motif) ligand 8 | 940 | 0.310 | 0.3597 | Yes |
| 7 | PYCARD | PYCARD Entrez,  Source | PYD and CARD domain containing | 1186 | 0.275 | 0.3830 | Yes |
| 8 | ERBB2IP | ERBB2IP Entrez,  Source | erbb2 interacting protein | 1524 | 0.242 | 0.3975 | Yes |
| 9 | BIRC2 | BIRC2 Entrez,  Source | baculoviral IAP repeat-containing 2 | 1760 | 0.224 | 0.4147 | Yes |
| 10 | CARD6 | CARD6 Entrez,  Source | caspase recruitment domain family, member 6 | 1778 | 0.222 | 0.4429 | Yes |
| 11 | IL1B | IL1B Entrez,  Source | interleukin 1, beta | 2213 | 0.194 | 0.4460 | Yes |
| 12 | CCL7 | CCL7 Entrez,  Source | chemokine (C-C motif) ligand 7 | 2483 | 0.179 | 0.4555 | Yes |
| 13 | CASP8 | CASP8 Entrez,  Source | caspase 8, apoptosis-related cysteine peptidase | 2594 | 0.174 | 0.4726 | Yes |
| 14 | TRIP6 | TRIP6 Entrez,  Source | thyroid hormone receptor interactor 6 | 2922 | 0.160 | 0.4767 | Yes |
| 15 | CCL2 | CCL2 Entrez,  Source | chemokine (C-C motif) ligand 2 | 3170 | 0.151 | 0.4839 | Yes |
| 16 | IL8 | IL8 Entrez,  Source | interleukin 8 | 3173 | 0.151 | 0.5035 | Yes |
| 17 | CCL5 | CCL5 Entrez,  Source | chemokine (C-C motif) ligand 5 | 3560 | 0.138 | 0.5017 | No |
| 18 | CCL11 | CCL11 Entrez,  Source | chemokine (C-C motif) ligand 11 | 4363 | 0.112 | 0.4753 | No |
| 19 | RIPK2 | RIPK2 Entrez,  Source | receptor-interacting serine-threonine kinase 2 | 4485 | 0.109 | 0.4834 | No |
| 20 | MAPK10 | MAPK10 Entrez,  Source | mitogen-activated protein kinase 10 | 4498 | 0.109 | 0.4970 | No |
| 21 | MAPK3 | MAPK3 Entrez,  Source | mitogen-activated protein kinase 3 | 4947 | 0.097 | 0.4867 | No |
| 22 | IKBKB | IKBKB Entrez,  Source | inhibitor of kappa light polypeptide gene enhancer in B-cells, kinase beta | 5465 | 0.085 | 0.4713 | No |
| 23 | MAPK8 | MAPK8 Entrez,  Source | mitogen-activated protein kinase 8 | 5571 | 0.082 | 0.4766 | No |
| 24 | MAPK14 | MAPK14 Entrez,  Source | mitogen-activated protein kinase 14 | 5674 | 0.080 | 0.4818 | No |
| 25 | TRAF6 | TRAF6 Entrez,  Source | TNF receptor-associated factor 6 | 5796 | 0.077 | 0.4857 | No |
| 26 | TNFAIP3 | TNFAIP3 Entrez,  Source | tumor necrosis factor, alpha-induced protein 3 | 5928 | 0.075 | 0.4887 | No |
| 27 | CCL13 | CCL13 Entrez,  Source | chemokine (C-C motif) ligand 13 | 6061 | 0.071 | 0.4913 | No |
| 28 | CXCL2 | CXCL2 Entrez,  Source | chemokine (C-X-C motif) ligand 2 | 6400 | 0.065 | 0.4824 | No |
| 29 | MAP3K7 | MAP3K7 Entrez,  Source | mitogen-activated protein kinase kinase kinase 7 | 6819 | 0.057 | 0.4685 | No |
| 30 | IKBKG | IKBKG Entrez,  Source | inhibitor of kappa light polypeptide gene enhancer in B-cells, kinase gamma | 6863 | 0.056 | 0.4736 | No |
| 31 | SUGT1 | SUGT1 Entrez,  Source | SGT1, suppressor of G2 allele of SKP1 (S. cerevisiae) | 7134 | 0.051 | 0.4664 | No |
| 32 | NFKBIB | NFKBIB Entrez,  Source | nuclear factor of kappa light polypeptide gene enhancer in B-cells inhibitor, beta | 9743 | 0.005 | 0.3334 | No |
| 33 | PSTPIP1 | PSTPIP1 Entrez,  Source | proline-serine-threonine phosphatase interacting protein 1 | 10021 | 0.001 | 0.3193 | No |
| 34 | CHUK | CHUK Entrez,  Source | conserved helix-loop-helix ubiquitous kinase | 10296 | -0.003 | 0.3057 | No |
| 35 | HSP90B1 | HSP90B1 Entrez,  Source | heat shock protein 90kDa beta (Grp94), member 1 | 10421 | -0.005 | 0.3001 | No |
| 36 | RELA | RELA Entrez,  Source | v-rel reticuloendotheliosis viral oncogene homolog A, nuclear factor of kappa light polypeptide gene enhancer in B-cells 3, p65 (avian) | 10682 | -0.010 | 0.2880 | No |
| 37 | TNF | TNF Entrez,  Source | tumor necrosis factor (TNF superfamily, member 2) | 11429 | -0.023 | 0.2528 | No |
| 38 | MAPK11 | MAPK11 Entrez,  Source | mitogen-activated protein kinase 11 | 11797 | -0.030 | 0.2378 | No |
| 39 | CARD9 | CARD9 Entrez,  Source | caspase recruitment domain family, member 9 | 11858 | -0.031 | 0.2388 | No |
| 40 | HSP90AB1 | HSP90AB1 Entrez,  Source | heat shock protein 90kDa alpha (cytosolic), class B member 1 | 11863 | -0.031 | 0.2426 | No |
| 41 | HSP90AA1 | HSP90AA1 Entrez,  Source | heat shock protein 90kDa alpha (cytosolic), class A member 1 | 12953 | -0.051 | 0.1934 | No |
| 42 | MAPK1 | MAPK1 Entrez,  Source | mitogen-activated protein kinase 1 | 13089 | -0.053 | 0.1934 | No |
| 43 | MEFV | MEFV Entrez,  Source | Mediterranean fever | 13700 | -0.066 | 0.1707 | No |
| 44 | CARD8 | CARD8 Entrez,  Source | caspase recruitment domain family, member 8 | 13954 | -0.071 | 0.1671 | No |
| 45 | NFKB1 | NFKB1 Entrez,  Source | nuclear factor of kappa light polypeptide gene enhancer in B-cells 1 (p105) | 14629 | -0.088 | 0.1440 | No |
| 46 | MAPK12 | MAPK12 Entrez,  Source | mitogen-activated protein kinase 12 | 14771 | -0.091 | 0.1487 | No |
| 47 | IL6 | IL6 Entrez,  Source | interleukin 6 (interferon, beta 2) | 14928 | -0.095 | 0.1532 | No |
| 48 | NFKBIA | NFKBIA Entrez,  Source | nuclear factor of kappa light polypeptide gene enhancer in B-cells inhibitor, alpha | 15953 | -0.125 | 0.1171 | No |
| 49 | MAPK13 | MAPK13 Entrez,  Source | mitogen-activated protein kinase 13 | 16657 | -0.153 | 0.1011 | No |
| 50 | PYDC1 | PYDC1 Entrez,  Source | PYD (pyrin domain) containing 1 | 16668 | -0.154 | 0.1207 | No |
| 51 | MAPK9 | MAPK9 Entrez,  Source | mitogen-activated protein kinase 9 | 17655 | -0.209 | 0.0975 | No |
Table: GSEA details [plain text format]

  

Fig 2: KEGG\_NOD\_LIKE\_RECEPTOR\_SIGNALING\_PATHWAY      
 Blue-Pink O' Gram in the Space of the Analyzed GeneSet

  

Fig 3: KEGG\_NOD\_LIKE\_RECEPTOR\_SIGNALING\_PATHWAY: Random ES distribution      
 Gene set null distribution of ES for **KEGG\_NOD\_LIKE\_RECEPTOR\_SIGNALING\_PATHWAY**
