# Supplementary material for: Comprehensive analysis of β-catenin target genes in colorectal carcinoma cell lines with deregulated Wnt/β-catenin signaling
Source: BMC Genomics. 2014 Jan 28;15:74. doi: 10.1186/1471-2164-15-74 (PMC3909937; doi:10.1186/1471-2164-15-74)
Supplement: Additional file 5 — GSEA analysis using the KEGG pathway database. This zipped file contains confirming data of the GSEA analysis. The names of the directories containing the files were composed of the term ‘GSEA’, the name of the cell line, e.g. DLD1, SW480, or LS174T, and the pathway database (KEGG). Please use a web browser to view the files with the name ‘index.html’ in the corresponding directories to start exploring the data. [file 1471-2164-15-74-S5.zip › GSEA_LS174T_KEGG/KEGG_O_GLYCAN_BIOSYNTHESIS.html]

Details for gene set KEGG\_O\_GLYCAN\_BIOSYNTHESIS[GSEA]

|  || Dataset | LS174T\_collapsed\_to\_symbols.class.cls#b\_versus\_bg.class.cls#b\_versus\_bg\_repos |
| Phenotype | class.cls#b\_versus\_bg\_repos |
| Upregulated in class | 1 |
| GeneSet | KEGG\_O\_GLYCAN\_BIOSYNTHESIS |
| Enrichment Score (ES) | 0.5169018 |
| Normalized Enrichment Score (NES) | 1.5051111 |
| Nominal p-value | 0.048780486 |
| FDR q-value | 0.17628236 |
| FWER p-Value | 0.932 |
Table: GSEA Results Summary

  

Fig 1: Enrichment plot: KEGG\_O\_GLYCAN\_BIOSYNTHESIS      
 Profile of the Running ES Score & Positions of GeneSet Members on the Rank Ordered List

  

| PROBE | GENE SYMBOL | GENE\_TITLE | RANK IN GENE LIST | RANK METRIC SCORE | RUNNING ES | CORE ENRICHMENT || 1 | GCNT3 | GCNT3 Entrez,  Source | glucosaminyl (N-acetyl) transferase 3, mucin type | 27 | 1.334 | 0.2659 | Yes |
| 2 | ST6GALNAC1 | ST6GALNAC1 Entrez,  Source | ST6 (alpha-N-acetyl-neuraminyl-2,3-beta-galactosyl-1,3)-N-acetylgalactosaminide alpha-2,6-sialyltransferase 1 | 671 | 0.369 | 0.3069 | Yes |
| 3 | GALNT5 | GALNT5 Entrez,  Source | UDP-N-acetyl-alpha-D-galactosamine:polypeptide N-acetylgalactosaminyltransferase 5 (GalNAc-T5) | 683 | 0.368 | 0.3800 | Yes |
| 4 | GALNT7 | GALNT7 Entrez,  Source | UDP-N-acetyl-alpha-D-galactosamine:polypeptide N-acetylgalactosaminyltransferase 7 (GalNAc-T7) | 868 | 0.324 | 0.4354 | Yes |
| 5 | B3GNT6 | B3GNT6 Entrez,  Source | UDP-GlcNAc:betaGal beta-1,3-N-acetylglucosaminyltransferase 6 (core 3 synthase) | 1740 | 0.225 | 0.4359 | Yes |
| 6 | GALNTL4 | GALNTL4 Entrez,  Source | UDP-N-acetyl-alpha-D-galactosamine:polypeptide N-acetylgalactosaminyltransferase-like 4 | 1804 | 0.220 | 0.4768 | Yes |
| 7 | GALNT3 | GALNT3 Entrez,  Source | UDP-N-acetyl-alpha-D-galactosamine:polypeptide N-acetylgalactosaminyltransferase 3 (GalNAc-T3) | 2314 | 0.188 | 0.4885 | Yes |
| 8 | GCNT4 | GCNT4 Entrez,  Source | glucosaminyl (N-acetyl) transferase 4, core 2 (beta-1,6-N-acetylglucosaminyltransferase) | 2463 | 0.180 | 0.5169 | Yes |
| 9 | B4GALT5 | B4GALT5 Entrez,  Source | UDP-Gal:betaGlcNAc beta 1,4- galactosyltransferase, polypeptide 5 | 3699 | 0.133 | 0.4802 | No |
| 10 | C1GALT1 | C1GALT1 Entrez,  Source | core 1 synthase, glycoprotein-N-acetylgalactosamine 3-beta-galactosyltransferase, 1 | 4502 | 0.109 | 0.4609 | No |
| 11 | GALNT2 | GALNT2 Entrez,  Source | UDP-N-acetyl-alpha-D-galactosamine:polypeptide N-acetylgalactosaminyltransferase 2 (GalNAc-T2) | 4546 | 0.108 | 0.4803 | No |
| 12 | GALNT1 | GALNT1 Entrez,  Source | UDP-N-acetyl-alpha-D-galactosamine:polypeptide N-acetylgalactosaminyltransferase 1 (GalNAc-T1) | 4557 | 0.107 | 0.5013 | No |
| 13 | WBSCR17 | WBSCR17 Entrez,  Source | Williams-Beuren syndrome chromosome region 17 | 4756 | 0.102 | 0.5116 | No |
| 14 | GALNT10 | GALNT10 Entrez,  Source | UDP-N-acetyl-alpha-D-galactosamine:polypeptide N-acetylgalactosaminyltransferase 10 (GalNAc-T10) | 6068 | 0.071 | 0.4587 | No |
| 15 | GALNT12 | GALNT12 Entrez,  Source | UDP-N-acetyl-alpha-D-galactosamine:polypeptide N-acetylgalactosaminyltransferase 12 (GalNAc-T12) | 7464 | 0.045 | 0.3963 | No |
| 16 | ST3GAL2 | ST3GAL2 Entrez,  Source | ST3 beta-galactoside alpha-2,3-sialyltransferase 2 | 8880 | 0.019 | 0.3277 | No |
| 17 | ST3GAL1 | ST3GAL1 Entrez,  Source | ST3 beta-galactoside alpha-2,3-sialyltransferase 1 | 9354 | 0.012 | 0.3058 | No |
| 18 | C1GALT1C1 | C1GALT1C1 Entrez,  Source | C1GALT1-specific chaperone 1 | 9444 | 0.010 | 0.3032 | No |
| 19 | GALNT13 | GALNT13 Entrez,  Source | UDP-N-acetyl-alpha-D-galactosamine:polypeptide N-acetylgalactosaminyltransferase 13 (GalNAc-T13) | 9655 | 0.007 | 0.2938 | No |
| 20 | GALNTL1 | GALNTL1 Entrez,  Source | UDP-N-acetyl-alpha-D-galactosamine:polypeptide N-acetylgalactosaminyltransferase-like 1 | 10845 | -0.012 | 0.2354 | No |
| 21 | GALNT8 | GALNT8 Entrez,  Source | UDP-N-acetyl-alpha-D-galactosamine:polypeptide N-acetylgalactosaminyltransferase 8 (GalNAc-T8) | 11141 | -0.018 | 0.2238 | No |
| 22 | GALNT14 | GALNT14 Entrez,  Source | UDP-N-acetyl-alpha-D-galactosamine:polypeptide N-acetylgalactosaminyltransferase 14 (GalNAc-T14) | 11732 | -0.028 | 0.1993 | No |
| 23 | GALNTL5 | GALNTL5 Entrez,  Source | UDP-N-acetyl-alpha-D-galactosamine:polypeptide N-acetylgalactosaminyltransferase-like 5 | 12547 | -0.043 | 0.1663 | No |
| 24 | GALNTL2 | GALNTL2 Entrez,  Source | UDP-N-acetyl-alpha-D-galactosamine:polypeptide N-acetylgalactosaminyltransferase-like 2 | 12780 | -0.048 | 0.1640 | No |
| 25 | GALNT9 | GALNT9 Entrez,  Source | UDP-N-acetyl-alpha-D-galactosamine:polypeptide N-acetylgalactosaminyltransferase 9 (GalNAc-T9) | 14756 | -0.091 | 0.0811 | No |
| 26 | GCNT1 | GCNT1 Entrez,  Source | glucosaminyl (N-acetyl) transferase 1, core 2 (beta-1,6-N-acetylglucosaminyltransferase) | 15820 | -0.121 | 0.0508 | No |
| 27 | GALNT6 | GALNT6 Entrez,  Source | UDP-N-acetyl-alpha-D-galactosamine:polypeptide N-acetylgalactosaminyltransferase 6 (GalNAc-T6) | 17817 | -0.220 | -0.0073 | No |
| 28 | GALNT11 | GALNT11 Entrez,  Source | UDP-N-acetyl-alpha-D-galactosamine:polypeptide N-acetylgalactosaminyltransferase 11 (GalNAc-T11) | 19204 | -0.481 | 0.0180 | No |
Table: GSEA details [plain text format]

  

Fig 2: KEGG\_O\_GLYCAN\_BIOSYNTHESIS      
 Blue-Pink O' Gram in the Space of the Analyzed GeneSet

  

Fig 3: KEGG\_O\_GLYCAN\_BIOSYNTHESIS: Random ES distribution      
 Gene set null distribution of ES for **KEGG\_O\_GLYCAN\_BIOSYNTHESIS**
