# Supplementary material for: Comprehensive analysis of β-catenin target genes in colorectal carcinoma cell lines with deregulated Wnt/β-catenin signaling
Source: BMC Genomics. 2014 Jan 28;15:74. doi: 10.1186/1471-2164-15-74 (PMC3909937; doi:10.1186/1471-2164-15-74)
Supplement: Additional file 5 — GSEA analysis using the KEGG pathway database. This zipped file contains confirming data of the GSEA analysis. The names of the directories containing the files were composed of the term ‘GSEA’, the name of the cell line, e.g. DLD1, SW480, or LS174T, and the pathway database (KEGG). Please use a web browser to view the files with the name ‘index.html’ in the corresponding directories to start exploring the data. [file 1471-2164-15-74-S5.zip › GSEA_LS174T_KEGG/KEGG_OOCYTE_MEIOSIS.html]

Details for gene set KEGG\_OOCYTE\_MEIOSIS[GSEA]

|  || Dataset | LS174T\_collapsed\_to\_symbols.class.cls#b\_versus\_bg.class.cls#b\_versus\_bg\_repos |
| Phenotype | class.cls#b\_versus\_bg\_repos |
| Upregulated in class | 0 |
| GeneSet | KEGG\_OOCYTE\_MEIOSIS |
| Enrichment Score (ES) | -0.48490843 |
| Normalized Enrichment Score (NES) | -1.8558345 |
| Nominal p-value | 0.0 |
| FDR q-value | 0.007197176 |
| FWER p-Value | 0.063 |
Table: GSEA Results Summary

  

Fig 1: Enrichment plot: KEGG\_OOCYTE\_MEIOSIS      
 Profile of the Running ES Score & Positions of GeneSet Members on the Rank Ordered List

  

| PROBE | GENE SYMBOL | GENE\_TITLE | RANK IN GENE LIST | RANK METRIC SCORE | RUNNING ES | CORE ENRICHMENT || 1 | PPP2R5B | PPP2R5B Entrez,  Source | protein phosphatase 2, regulatory subunit B (B56), beta isoform | 933 | 0.311 | -0.0282 | No |
| 2 | PGR | PGR Entrez,  Source | progesterone receptor | 1144 | 0.280 | -0.0211 | No |
| 3 | CAMK2D | CAMK2D Entrez,  Source | calcium/calmodulin-dependent protein kinase (CaM kinase) II delta | 1282 | 0.265 | -0.0114 | No |
| 4 | PPP3CA | PPP3CA Entrez,  Source | protein phosphatase 3 (formerly 2B), catalytic subunit, alpha isoform (calcineurin A alpha) | 1332 | 0.259 | 0.0026 | No |
| 5 | PRKACG | PRKACG Entrez,  Source | protein kinase, cAMP-dependent, catalytic, gamma | 2268 | 0.190 | -0.0334 | No |
| 6 | BTRC | BTRC Entrez,  Source | beta-transducin repeat containing | 2856 | 0.163 | -0.0532 | No |
| 7 | ANAPC13 | ANAPC13 Entrez,  Source | anaphase promoting complex subunit 13 | 2876 | 0.162 | -0.0439 | No |
| 8 | ADCY8 | ADCY8 Entrez,  Source | adenylate cyclase 8 (brain) | 4003 | 0.123 | -0.0939 | No |
| 9 | MOS | MOS Entrez,  Source | v-mos Moloney murine sarcoma viral oncogene homolog | 4038 | 0.122 | -0.0879 | No |
| 10 | ADCY9 | ADCY9 Entrez,  Source | adenylate cyclase 9 | 4106 | 0.120 | -0.0837 | No |
| 11 | ADCY1 | ADCY1 Entrez,  Source | adenylate cyclase 1 (brain) | 4182 | 0.118 | -0.0801 | No |
| 12 | PRKACB | PRKACB Entrez,  Source | protein kinase, cAMP-dependent, catalytic, beta | 4257 | 0.115 | -0.0766 | No |
| 13 | CAMK2A | CAMK2A Entrez,  Source | calcium/calmodulin-dependent protein kinase (CaM kinase) II alpha | 4345 | 0.113 | -0.0739 | No |
| 14 | SMC1B | SMC1B Entrez,  Source | structural maintenance of chromosomes 1B | 4486 | 0.109 | -0.0741 | No |
| 15 | CDC16 | CDC16 Entrez,  Source | CDC16 cell division cycle 16 homolog (S. cerevisiae) | 4588 | 0.106 | -0.0725 | No |
| 16 | ADCY7 | ADCY7 Entrez,  Source | adenylate cyclase 7 | 4771 | 0.102 | -0.0754 | No |
| 17 | ITPR1 | ITPR1 Entrez,  Source | inositol 1,4,5-triphosphate receptor, type 1 | 4858 | 0.099 | -0.0735 | No |
| 18 | MAPK3 | MAPK3 Entrez,  Source | mitogen-activated protein kinase 3 | 4947 | 0.097 | -0.0719 | No |
| 19 | PPP2CB | PPP2CB Entrez,  Source | protein phosphatase 2 (formerly 2A), catalytic subunit, beta isoform | 5184 | 0.091 | -0.0782 | No |
| 20 | PLCZ1 | PLCZ1 Entrez,  Source | phospholipase C, zeta 1 | 5246 | 0.090 | -0.0757 | No |
| 21 | RPS6KA3 | RPS6KA3 Entrez,  Source | ribosomal protein S6 kinase, 90kDa, polypeptide 3 | 5521 | 0.083 | -0.0844 | No |
| 22 | CPEB1 | CPEB1 Entrez,  Source | cytoplasmic polyadenylation element binding protein 1 | 5662 | 0.080 | -0.0865 | No |
| 23 | YWHAH | YWHAH Entrez,  Source | tyrosine 3-monooxygenase/tryptophan 5-monooxygenase activation protein, eta polypeptide | 5873 | 0.075 | -0.0925 | No |
| 24 | SPDYA | SPDYA Entrez,  Source | speedy homolog A (Drosophila) | 5996 | 0.073 | -0.0942 | No |
| 25 | FBXW11 | FBXW11 Entrez,  Source | F-box and WD-40 domain protein 11 | 6104 | 0.071 | -0.0952 | No |
| 26 | ADCY4 | ADCY4 Entrez,  Source | adenylate cyclase 4 | 6242 | 0.068 | -0.0979 | No |
| 27 | PPP3R1 | PPP3R1 Entrez,  Source | protein phosphatase 3 (formerly 2B), regulatory subunit B, 19kDa, alpha isoform (calcineurin B, type I) | 6333 | 0.066 | -0.0983 | No |
| 28 | YWHAZ | YWHAZ Entrez,  Source | tyrosine 3-monooxygenase/tryptophan 5-monooxygenase activation protein, zeta polypeptide | 6389 | 0.065 | -0.0970 | No |
| 29 | CHP | CHP Entrez,  Source | - | 6594 | 0.061 | -0.1036 | No |
| 30 | INS | INS Entrez,  Source | insulin | 6846 | 0.056 | -0.1129 | No |
| 31 | YWHAG | YWHAG Entrez,  Source | tyrosine 3-monooxygenase/tryptophan 5-monooxygenase activation protein, gamma polypeptide | 7364 | 0.047 | -0.1365 | No |
| 32 | PPP3CC | PPP3CC Entrez,  Source | protein phosphatase 3 (formerly 2B), catalytic subunit, gamma isoform (calcineurin A gamma) | 7582 | 0.042 | -0.1450 | No |
| 33 | IGF1R | IGF1R Entrez,  Source | insulin-like growth factor 1 receptor | 7745 | 0.040 | -0.1508 | No |
| 34 | IGF1 | IGF1 Entrez,  Source | insulin-like growth factor 1 (somatomedin C) | 7808 | 0.039 | -0.1515 | No |
| 35 | ADCY5 | ADCY5 Entrez,  Source | adenylate cyclase 5 | 8116 | 0.032 | -0.1653 | No |
| 36 | STAG3 | STAG3 Entrez,  Source | stromal antigen 3 | 8136 | 0.032 | -0.1642 | No |
| 37 | MAP2K1 | MAP2K1 Entrez,  Source | mitogen-activated protein kinase kinase 1 | 8349 | 0.028 | -0.1733 | No |
| 38 | PPP2R1B | PPP2R1B Entrez,  Source | protein phosphatase 2 (formerly 2A), regulatory subunit A (PR 65), beta isoform | 9023 | 0.017 | -0.2068 | No |
| 39 | CAMK2B | CAMK2B Entrez,  Source | calcium/calmodulin-dependent protein kinase (CaM kinase) II beta | 9031 | 0.017 | -0.2061 | No |
| 40 | PPP1CB | PPP1CB Entrez,  Source | protein phosphatase 1, catalytic subunit, beta isoform | 9701 | 0.006 | -0.2401 | No |
| 41 | CALM2 | CALM2 Entrez,  Source | calmodulin 2 (phosphorylase kinase, delta) | 9728 | 0.005 | -0.2411 | No |
| 42 | RPS6KA1 | RPS6KA1 Entrez,  Source | ribosomal protein S6 kinase, 90kDa, polypeptide 1 | 9863 | 0.003 | -0.2478 | No |
| 43 | PPP2R5A | PPP2R5A Entrez,  Source | protein phosphatase 2, regulatory subunit B (B56), alpha isoform | 9927 | 0.002 | -0.2509 | No |
| 44 | ANAPC2 | ANAPC2 Entrez,  Source | anaphase promoting complex subunit 2 | 9963 | 0.002 | -0.2526 | No |
| 45 | RBX1 | RBX1 Entrez,  Source | ring-box 1 | 10204 | -0.002 | -0.2648 | No |
| 46 | CALML6 | CALML6 Entrez,  Source | calmodulin-like 6 | 10483 | -0.006 | -0.2787 | No |
| 47 | PPP1CA | PPP1CA Entrez,  Source | protein phosphatase 1, catalytic subunit, alpha isoform | 10528 | -0.007 | -0.2805 | No |
| 48 | ITPR3 | ITPR3 Entrez,  Source | inositol 1,4,5-triphosphate receptor, type 3 | 10591 | -0.008 | -0.2832 | No |
| 49 | YWHAB | YWHAB Entrez,  Source | tyrosine 3-monooxygenase/tryptophan 5-monooxygenase activation protein, beta polypeptide | 10824 | -0.012 | -0.2943 | No |
| 50 | RPS6KA6 | RPS6KA6 Entrez,  Source | ribosomal protein S6 kinase, 90kDa, polypeptide 6 | 10844 | -0.012 | -0.2945 | No |
| 51 | CALM1 | CALM1 Entrez,  Source | calmodulin 1 (phosphorylase kinase, delta) | 11352 | -0.022 | -0.3192 | No |
| 52 | PPP2CA | PPP2CA Entrez,  Source | protein phosphatase 2 (formerly 2A), catalytic subunit, alpha isoform | 11386 | -0.023 | -0.3195 | No |
| 53 | PPP2R5D | PPP2R5D Entrez,  Source | protein phosphatase 2, regulatory subunit B (B56), delta isoform | 11399 | -0.023 | -0.3186 | No |
| 54 | ANAPC11 | ANAPC11 Entrez,  Source | APC11 anaphase promoting complex subunit 11 homolog (yeast) | 11989 | -0.033 | -0.3468 | No |
| 55 | ADCY6 | ADCY6 Entrez,  Source | adenylate cyclase 6 | 12006 | -0.033 | -0.3456 | No |
| 56 | ADCY2 | ADCY2 Entrez,  Source | adenylate cyclase 2 (brain) | 12538 | -0.043 | -0.3701 | No |
| 57 | CALM3 | CALM3 Entrez,  Source | calmodulin 3 (phosphorylase kinase, delta) | 12610 | -0.044 | -0.3710 | No |
| 58 | ANAPC4 | ANAPC4 Entrez,  Source | anaphase promoting complex subunit 4 | 12643 | -0.045 | -0.3697 | No |
| 59 | PPP2R5C | PPP2R5C Entrez,  Source | protein phosphatase 2, regulatory subunit B (B56), gamma isoform | 12707 | -0.046 | -0.3700 | No |
| 60 | PPP2R5E | PPP2R5E Entrez,  Source | protein phosphatase 2, regulatory subunit B (B56), epsilon isoform | 12768 | -0.047 | -0.3701 | No |
| 61 | CDC23 | CDC23 Entrez,  Source | CDC23 (cell division cycle 23, yeast, homolog) | 12882 | -0.049 | -0.3728 | No |
| 62 | MAPK1 | MAPK1 Entrez,  Source | mitogen-activated protein kinase 1 | 13089 | -0.053 | -0.3800 | No |
| 63 | AR | AR Entrez,  Source | androgen receptor (dihydrotestosterone receptor; testicular feminization; spinal and bulbar muscular atrophy; Kennedy disease) | 13091 | -0.053 | -0.3767 | No |
| 64 | ANAPC1 | ANAPC1 Entrez,  Source | anaphase promoting complex subunit 1 | 13231 | -0.056 | -0.3803 | No |
| 65 | YWHAQ | YWHAQ Entrez,  Source | tyrosine 3-monooxygenase/tryptophan 5-monooxygenase activation protein, theta polypeptide | 13416 | -0.060 | -0.3859 | No |
| 66 | PPP2R1A | PPP2R1A Entrez,  Source | protein phosphatase 2 (formerly 2A), regulatory subunit A (PR 65), alpha isoform | 13623 | -0.064 | -0.3924 | No |
| 67 | CDC27 | CDC27 Entrez,  Source | cell division cycle 27 | 13859 | -0.070 | -0.4001 | No |
| 68 | CALML5 | CALML5 Entrez,  Source | calmodulin-like 5 | 13997 | -0.072 | -0.4025 | No |
| 69 | CUL1 | CUL1 Entrez,  Source | cullin 1 | 14106 | -0.075 | -0.4033 | No |
| 70 | CALML3 | CALML3 Entrez,  Source | calmodulin-like 3 | 14117 | -0.075 | -0.3990 | No |
| 71 | FBXO43 | FBXO43 Entrez,  Source | F-box protein 43 | 14343 | -0.080 | -0.4055 | No |
| 72 | MAPK12 | MAPK12 Entrez,  Source | mitogen-activated protein kinase 12 | 14771 | -0.091 | -0.4216 | No |
| 73 | CAMK2G | CAMK2G Entrez,  Source | calcium/calmodulin-dependent protein kinase (CaM kinase) II gamma | 14979 | -0.096 | -0.4262 | No |
| 74 | ANAPC5 | ANAPC5 Entrez,  Source | anaphase promoting complex subunit 5 | 15189 | -0.103 | -0.4304 | No |
| 75 | PPP1CC | PPP1CC Entrez,  Source | protein phosphatase 1, catalytic subunit, gamma isoform | 15272 | -0.105 | -0.4279 | No |
| 76 | ANAPC7 | ANAPC7 Entrez,  Source | anaphase promoting complex subunit 7 | 15479 | -0.111 | -0.4315 | No |
| 77 | ANAPC10 | ANAPC10 Entrez,  Source | anaphase promoting complex subunit 10 | 15957 | -0.126 | -0.4480 | No |
| 78 | PPP3R2 | PPP3R2 Entrez,  Source | protein phosphatase 3 (formerly 2B), regulatory subunit B, 19kDa, beta isoform (calcineurin B, type II) | 16224 | -0.136 | -0.4530 | No |
| 79 | PRKACA | PRKACA Entrez,  Source | protein kinase, cAMP-dependent, catalytic, alpha | 16647 | -0.153 | -0.4650 | No |
| 80 | RPS6KA2 | RPS6KA2 Entrez,  Source | ribosomal protein S6 kinase, 90kDa, polypeptide 2 | 16682 | -0.154 | -0.4569 | No |
| 81 | SMC1A | SMC1A Entrez,  Source | structural maintenance of chromosomes 1A | 16683 | -0.154 | -0.4471 | No |
| 82 | SMC3 | SMC3 Entrez,  Source | structural maintenance of chromosomes 3 | 17134 | -0.177 | -0.4590 | No |
| 83 | PPP3CB | PPP3CB Entrez,  Source | protein phosphatase 3 (formerly 2B), catalytic subunit, beta isoform (calcineurin A beta) | 17512 | -0.200 | -0.4657 | No |
| 84 | YWHAE | YWHAE Entrez,  Source | tyrosine 3-monooxygenase/tryptophan 5-monooxygenase activation protein, epsilon polypeptide | 17886 | -0.226 | -0.4706 | Yes |
| 85 | ADCY3 | ADCY3 Entrez,  Source | adenylate cyclase 3 | 18015 | -0.238 | -0.4620 | Yes |
| 86 | MAD2L2 | MAD2L2 Entrez,  Source | MAD2 mitotic arrest deficient-like 2 (yeast) | 18108 | -0.246 | -0.4511 | Yes |
| 87 | PRKX | PRKX Entrez,  Source | protein kinase, X-linked | 18151 | -0.251 | -0.4373 | Yes |
| 88 | CCNB2 | CCNB2 Entrez,  Source | cyclin B2 | 18253 | -0.262 | -0.4259 | Yes |
| 89 | PTTG1 | PTTG1 Entrez,  Source | pituitary tumor-transforming 1 | 18263 | -0.263 | -0.4096 | Yes |
| 90 | CCNB1 | CCNB1 Entrez,  Source | cyclin B1 | 18465 | -0.286 | -0.4018 | Yes |
| 91 | PTTG2 | PTTG2 Entrez,  Source | pituitary tumor-transforming 2 | 18589 | -0.305 | -0.3887 | Yes |
| 92 | PLK1 | PLK1 Entrez,  Source | polo-like kinase 1 (Drosophila) | 18596 | -0.306 | -0.3695 | Yes |
| 93 | MAD2L1 | MAD2L1 Entrez,  Source | MAD2 mitotic arrest deficient-like 1 (yeast) | 18616 | -0.310 | -0.3508 | Yes |
| 94 | PKMYT1 | PKMYT1 Entrez,  Source | protein kinase, membrane associated tyrosine/threonine 1 | 18637 | -0.313 | -0.3320 | Yes |
| 95 | CDC20 | CDC20 Entrez,  Source | CDC20 cell division cycle 20 homolog (S. cerevisiae) | 18657 | -0.317 | -0.3128 | Yes |
| 96 | CCNE1 | CCNE1 Entrez,  Source | cyclin E1 | 18741 | -0.330 | -0.2961 | Yes |
| 97 | AURKA | AURKA Entrez,  Source | aurora kinase A | 18824 | -0.347 | -0.2783 | Yes |
| 98 | CDK2 | CDK2 Entrez,  Source | cyclin-dependent kinase 2 | 18981 | -0.390 | -0.2615 | Yes |
| 99 | SLK | SLK Entrez,  Source | STE20-like kinase (yeast) | 19009 | -0.398 | -0.2375 | Yes |
| 100 | ESPL1 | ESPL1 Entrez,  Source | extra spindle poles like 1 (S. cerevisiae) | 19120 | -0.442 | -0.2151 | Yes |
| 101 | SGOL1 | SGOL1 Entrez,  Source | shugoshin-like 1 (S. pombe) | 19170 | -0.466 | -0.1879 | Yes |
| 102 | BUB1 | BUB1 Entrez,  Source | BUB1 budding uninhibited by benzimidazoles 1 homolog (yeast) | 19278 | -0.527 | -0.1599 | Yes |
| 103 | FBXO5 | FBXO5 Entrez,  Source | F-box protein 5 | 19322 | -0.554 | -0.1269 | Yes |
| 104 | CDC25C | CDC25C Entrez,  Source | cell division cycle 25C | 19398 | -0.634 | -0.0905 | Yes |
| 105 | ITPR2 | ITPR2 Entrez,  Source | inositol 1,4,5-triphosphate receptor, type 2 | 19456 | -0.714 | -0.0480 | Yes |
| 106 | CCNE2 | CCNE2 Entrez,  Source | cyclin E2 | 19501 | -0.835 | 0.0028 | Yes |
Table: GSEA details [plain text format]

  

Fig 2: KEGG\_OOCYTE\_MEIOSIS      
 Blue-Pink O' Gram in the Space of the Analyzed GeneSet

  

Fig 3: KEGG\_OOCYTE\_MEIOSIS: Random ES distribution      
 Gene set null distribution of ES for **KEGG\_OOCYTE\_MEIOSIS**
