# Supplementary material for: Comprehensive analysis of β-catenin target genes in colorectal carcinoma cell lines with deregulated Wnt/β-catenin signaling
Source: BMC Genomics. 2014 Jan 28;15:74. doi: 10.1186/1471-2164-15-74 (PMC3909937; doi:10.1186/1471-2164-15-74)
Supplement: Additional file 5 — GSEA analysis using the KEGG pathway database. This zipped file contains confirming data of the GSEA analysis. The names of the directories containing the files were composed of the term ‘GSEA’, the name of the cell line, e.g. DLD1, SW480, or LS174T, and the pathway database (KEGG). Please use a web browser to view the files with the name ‘index.html’ in the corresponding directories to start exploring the data. [file 1471-2164-15-74-S5.zip › GSEA_LS174T_KEGG/KEGG_P53_SIGNALING_PATHWAY.html]

Details for gene set KEGG\_P53\_SIGNALING\_PATHWAY[GSEA]

|  || Dataset | LS174T\_collapsed\_to\_symbols.class.cls#b\_versus\_bg.class.cls#b\_versus\_bg\_repos |
| Phenotype | class.cls#b\_versus\_bg\_repos |
| Upregulated in class | 0 |
| GeneSet | KEGG\_P53\_SIGNALING\_PATHWAY |
| Enrichment Score (ES) | -0.44151187 |
| Normalized Enrichment Score (NES) | -1.5630163 |
| Nominal p-value | 0.00896861 |
| FDR q-value | 0.10707021 |
| FWER p-Value | 0.73 |
Table: GSEA Results Summary

  

Fig 1: Enrichment plot: KEGG\_P53\_SIGNALING\_PATHWAY      
 Profile of the Running ES Score & Positions of GeneSet Members on the Rank Ordered List

  

| PROBE | GENE SYMBOL | GENE\_TITLE | RANK IN GENE LIST | RANK METRIC SCORE | RUNNING ES | CORE ENRICHMENT || 1 | SERPINB5 | SERPINB5 Entrez,  Source | serpin peptidase inhibitor, clade B (ovalbumin), member 5 | 185 | 0.674 | 0.0498 | No |
| 2 | SESN3 | SESN3 Entrez,  Source | sestrin 3 | 332 | 0.507 | 0.0869 | No |
| 3 | TP53I3 | TP53I3 Entrez,  Source | tumor protein p53 inducible protein 3 | 735 | 0.354 | 0.0974 | No |
| 4 | CCNG2 | CCNG2 Entrez,  Source | cyclin G2 | 1085 | 0.288 | 0.1048 | No |
| 5 | GADD45B | GADD45B Entrez,  Source | growth arrest and DNA-damage-inducible, beta | 2459 | 0.180 | 0.0502 | No |
| 6 | CASP8 | CASP8 Entrez,  Source | caspase 8, apoptosis-related cysteine peptidase | 2594 | 0.174 | 0.0586 | No |
| 7 | PERP | PERP Entrez,  Source | PERP, TP53 apoptosis effector | 2972 | 0.158 | 0.0531 | No |
| 8 | THBS1 | THBS1 Entrez,  Source | thrombospondin 1 | 3031 | 0.156 | 0.0639 | No |
| 9 | CCNB3 | CCNB3 Entrez,  Source | cyclin B3 | 4731 | 0.103 | -0.0142 | No |
| 10 | ATM | ATM Entrez,  Source | ataxia telangiectasia mutated (includes complementation groups A, C and D) | 5168 | 0.092 | -0.0285 | No |
| 11 | SFN | SFN Entrez,  Source | stratifin | 5220 | 0.090 | -0.0232 | No |
| 12 | IGFBP3 | IGFBP3 Entrez,  Source | insulin-like growth factor binding protein 3 | 5377 | 0.087 | -0.0236 | No |
| 13 | CCNG1 | CCNG1 Entrez,  Source | cyclin G1 | 6070 | 0.071 | -0.0528 | No |
| 14 | APAF1 | APAF1 Entrez,  Source | apoptotic peptidase activating factor | 6284 | 0.067 | -0.0578 | No |
| 15 | CDKN2A | CDKN2A Entrez,  Source | cyclin-dependent kinase inhibitor 2A (melanoma, p16, inhibits CDK4) | 6824 | 0.057 | -0.0805 | No |
| 16 | GADD45A | GADD45A Entrez,  Source | growth arrest and DNA-damage-inducible, alpha | 7050 | 0.052 | -0.0874 | No |
| 17 | RPRM | RPRM Entrez,  Source | reprimo, TP53 dependent G2 arrest mediator candidate | 7090 | 0.052 | -0.0848 | No |
| 18 | SERPINE1 | SERPINE1 Entrez,  Source | serpin peptidase inhibitor, clade E (nexin, plasminogen activator inhibitor type 1), member 1 | 7480 | 0.044 | -0.1009 | No |
| 19 | PTEN | PTEN Entrez,  Source | phosphatase and tensin homolog (mutated in multiple advanced cancers 1) | 7592 | 0.042 | -0.1029 | No |
| 20 | RFWD2 | RFWD2 Entrez,  Source | ring finger and WD repeat domain 2 | 7722 | 0.040 | -0.1060 | No |
| 21 | IGF1 | IGF1 Entrez,  Source | insulin-like growth factor 1 (somatomedin C) | 7808 | 0.039 | -0.1069 | No |
| 22 | ZMAT3 | ZMAT3 Entrez,  Source | zinc finger, matrin type 3 | 8184 | 0.031 | -0.1234 | No |
| 23 | FAS | FAS Entrez,  Source | Fas (TNF receptor superfamily, member 6) | 8828 | 0.020 | -0.1546 | No |
| 24 | RRM2B | RRM2B Entrez,  Source | ribonucleotide reductase M2 B (TP53 inducible) | 8999 | 0.017 | -0.1618 | No |
| 25 | CD82 | CD82 Entrez,  Source | CD82 molecule | 9417 | 0.010 | -0.1823 | No |
| 26 | CASP9 | CASP9 Entrez,  Source | caspase 9, apoptosis-related cysteine peptidase | 9688 | 0.006 | -0.1957 | No |
| 27 | BAX | BAX Entrez,  Source | BCL2-associated X protein | 9736 | 0.005 | -0.1976 | No |
| 28 | BBC3 | BBC3 Entrez,  Source | BCL2 binding component 3 | 11426 | -0.023 | -0.2822 | No |
| 29 | SESN2 | SESN2 Entrez,  Source | sestrin 2 | 11633 | -0.027 | -0.2904 | No |
| 30 | RCHY1 | RCHY1 Entrez,  Source | ring finger and CHY zinc finger domain containing 1 | 12181 | -0.036 | -0.3153 | No |
| 31 | SIAH1 | SIAH1 Entrez,  Source | seven in absentia homolog 1 (Drosophila) | 12302 | -0.039 | -0.3181 | No |
| 32 | CHEK1 | CHEK1 Entrez,  Source | CHK1 checkpoint homolog (S. pombe) | 12502 | -0.042 | -0.3246 | No |
| 33 | MDM2 | MDM2 Entrez,  Source | Mdm2, transformed 3T3 cell double minute 2, p53 binding protein (mouse) | 12680 | -0.046 | -0.3296 | No |
| 34 | GADD45G | GADD45G Entrez,  Source | growth arrest and DNA-damage-inducible, gamma | 12789 | -0.048 | -0.3309 | No |
| 35 | ATR | ATR Entrez,  Source | ataxia telangiectasia and Rad3 related | 13582 | -0.063 | -0.3660 | No |
| 36 | CYCS | CYCS Entrez,  Source | cytochrome c, somatic | 13826 | -0.069 | -0.3724 | No |
| 37 | EI24 | EI24 Entrez,  Source | etoposide induced 2.4 mRNA | 14018 | -0.073 | -0.3758 | No |
| 38 | PPM1D | PPM1D Entrez,  Source | protein phosphatase 1D magnesium-dependent, delta isoform | 14651 | -0.088 | -0.4005 | No |
| 39 | TP73 | TP73 Entrez,  Source | tumor protein p73 | 14924 | -0.095 | -0.4061 | No |
| 40 | CDKN1A | CDKN1A Entrez,  Source | cyclin-dependent kinase inhibitor 1A (p21, Cip1) | 15286 | -0.106 | -0.4153 | No |
| 41 | CCND3 | CCND3 Entrez,  Source | cyclin D3 | 15420 | -0.109 | -0.4125 | No |
| 42 | BID | BID Entrez,  Source | BH3 interacting domain death agonist | 15524 | -0.112 | -0.4079 | No |
| 43 | MDM4 | MDM4 Entrez,  Source | Mdm4, transformed 3T3 cell double minute 4, p53 binding protein (mouse) | 15834 | -0.121 | -0.4131 | No |
| 44 | BAI1 | BAI1 Entrez,  Source | brain-specific angiogenesis inhibitor 1 | 16094 | -0.131 | -0.4149 | No |
| 45 | CDK6 | CDK6 Entrez,  Source | cyclin-dependent kinase 6 | 16614 | -0.151 | -0.4282 | Yes |
| 46 | CDK4 | CDK4 Entrez,  Source | cyclin-dependent kinase 4 | 16792 | -0.160 | -0.4232 | Yes |
| 47 | TP53 | TP53 Entrez,  Source | tumor protein p53 (Li-Fraumeni syndrome) | 17097 | -0.175 | -0.4235 | Yes |
| 48 | TSC2 | TSC2 Entrez,  Source | tuberous sclerosis 2 | 17123 | -0.176 | -0.4093 | Yes |
| 49 | SESN1 | SESN1 Entrez,  Source | sestrin 1 | 17478 | -0.198 | -0.4100 | Yes |
| 50 | TNFRSF10B | TNFRSF10B Entrez,  Source | tumor necrosis factor receptor superfamily, member 10b | 17848 | -0.222 | -0.4094 | Yes |
| 51 | CCND1 | CCND1 Entrez,  Source | cyclin D1 | 18184 | -0.254 | -0.4042 | Yes |
| 52 | CCNB2 | CCNB2 Entrez,  Source | cyclin B2 | 18253 | -0.262 | -0.3847 | Yes |
| 53 | STEAP3 | STEAP3 Entrez,  Source | STEAP family member 3 | 18317 | -0.269 | -0.3642 | Yes |
| 54 | CCND2 | CCND2 Entrez,  Source | cyclin D2 | 18356 | -0.273 | -0.3421 | Yes |
| 55 | CCNB1 | CCNB1 Entrez,  Source | cyclin B1 | 18465 | -0.286 | -0.3224 | Yes |
| 56 | RRM2 | RRM2 Entrez,  Source | ribonucleotide reductase M2 polypeptide | 18610 | -0.308 | -0.3027 | Yes |
| 57 | CCNE1 | CCNE1 Entrez,  Source | cyclin E1 | 18741 | -0.330 | -0.2803 | Yes |
| 58 | CASP3 | CASP3 Entrez,  Source | caspase 3, apoptosis-related cysteine peptidase | 18750 | -0.332 | -0.2515 | Yes |
| 59 | CHEK2 | CHEK2 Entrez,  Source | CHK2 checkpoint homolog (S. pombe) | 18836 | -0.350 | -0.2251 | Yes |
| 60 | CDK2 | CDK2 Entrez,  Source | cyclin-dependent kinase 2 | 18981 | -0.390 | -0.1982 | Yes |
| 61 | GTSE1 | GTSE1 Entrez,  Source | G-2 and S-phase expressed 1 | 19073 | -0.421 | -0.1658 | Yes |
| 62 | DDB2 | DDB2 Entrez,  Source | damage-specific DNA binding protein 2, 48kDa | 19346 | -0.572 | -0.1294 | Yes |
| 63 | PMAIP1 | PMAIP1 Entrez,  Source | phorbol-12-myristate-13-acetate-induced protein 1 | 19475 | -0.758 | -0.0693 | Yes |
| 64 | CCNE2 | CCNE2 Entrez,  Source | cyclin E2 | 19501 | -0.835 | 0.0028 | Yes |
Table: GSEA details [plain text format]

  

Fig 2: KEGG\_P53\_SIGNALING\_PATHWAY      
 Blue-Pink O' Gram in the Space of the Analyzed GeneSet

  

Fig 3: KEGG\_P53\_SIGNALING\_PATHWAY: Random ES distribution      
 Gene set null distribution of ES for **KEGG\_P53\_SIGNALING\_PATHWAY**
