# Supplementary material for: Comprehensive analysis of β-catenin target genes in colorectal carcinoma cell lines with deregulated Wnt/β-catenin signaling
Source: BMC Genomics. 2014 Jan 28;15:74. doi: 10.1186/1471-2164-15-74 (PMC3909937; doi:10.1186/1471-2164-15-74)
Supplement: Additional file 5 — GSEA analysis using the KEGG pathway database. This zipped file contains confirming data of the GSEA analysis. The names of the directories containing the files were composed of the term ‘GSEA’, the name of the cell line, e.g. DLD1, SW480, or LS174T, and the pathway database (KEGG). Please use a web browser to view the files with the name ‘index.html’ in the corresponding directories to start exploring the data. [file 1471-2164-15-74-S5.zip › GSEA_LS174T_KEGG/KEGG_PANTOTHENATE_AND_COA_BIOSYNTHESIS.html]

Details for gene set KEGG\_PANTOTHENATE\_AND\_COA\_BIOSYNTHESIS[GSEA]

|  || Dataset | LS174T\_collapsed\_to\_symbols.class.cls#b\_versus\_bg.class.cls#b\_versus\_bg\_repos |
| Phenotype | class.cls#b\_versus\_bg\_repos |
| Upregulated in class | 0 |
| GeneSet | KEGG\_PANTOTHENATE\_AND\_COA\_BIOSYNTHESIS |
| Enrichment Score (ES) | -0.6012554 |
| Normalized Enrichment Score (NES) | -1.5488873 |
| Nominal p-value | 0.0513347 |
| FDR q-value | 0.109857626 |
| FWER p-Value | 0.762 |
Table: GSEA Results Summary

  

Fig 1: Enrichment plot: KEGG\_PANTOTHENATE\_AND\_COA\_BIOSYNTHESIS      
 Profile of the Running ES Score & Positions of GeneSet Members on the Rank Ordered List

  

| PROBE | GENE SYMBOL | GENE\_TITLE | RANK IN GENE LIST | RANK METRIC SCORE | RUNNING ES | CORE ENRICHMENT || 1 | ENPP3 | ENPP3 Entrez,  Source | ectonucleotide pyrophosphatase/phosphodiesterase 3 | 2943 | 0.159 | -0.0784 | No |
| 2 | PANK1 | PANK1 Entrez,  Source | pantothenate kinase 1 | 3538 | 0.138 | -0.0459 | No |
| 3 | VNN2 | VNN2 Entrez,  Source | vanin 2 | 6540 | 0.062 | -0.1712 | No |
| 4 | PPCS | PPCS Entrez,  Source | phosphopantothenoylcysteine synthetase | 8305 | 0.029 | -0.2483 | No |
| 5 | COASY | COASY Entrez,  Source | Coenzyme A synthase | 8979 | 0.018 | -0.2747 | No |
| 6 | BCAT2 | BCAT2 Entrez,  Source | branched chain aminotransferase 2, mitochondrial | 9406 | 0.011 | -0.2917 | No |
| 7 | DPYD | DPYD Entrez,  Source | dihydropyrimidine dehydrogenase | 9709 | 0.006 | -0.3046 | No |
| 8 | UPB1 | UPB1 Entrez,  Source | ureidopropionase, beta | 12891 | -0.049 | -0.4448 | No |
| 9 | PANK3 | PANK3 Entrez,  Source | pantothenate kinase 3 | 15406 | -0.109 | -0.5241 | No |
| 10 | PANK2 | PANK2 Entrez,  Source | pantothenate kinase 2 (Hallervorden-Spatz syndrome) | 16915 | -0.165 | -0.5262 | Yes |
| 11 | DPYS | DPYS Entrez,  Source | dihydropyrimidinase | 17252 | -0.183 | -0.4603 | Yes |
| 12 | VNN1 | VNN1 Entrez,  Source | vanin 1 | 17877 | -0.225 | -0.3899 | Yes |
| 13 | PPCDC | PPCDC Entrez,  Source | phosphopantothenoylcysteine decarboxylase | 18112 | -0.247 | -0.2896 | Yes |
| 14 | ENPP1 | ENPP1 Entrez,  Source | ectonucleotide pyrophosphatase/phosphodiesterase 1 | 18181 | -0.254 | -0.1776 | Yes |
| 15 | BCAT1 | BCAT1 Entrez,  Source | branched chain aminotransferase 1, cytosolic | 18312 | -0.269 | -0.0619 | Yes |
| 16 | PANK4 | PANK4 Entrez,  Source | pantothenate kinase 4 | 18372 | -0.276 | 0.0606 | Yes |
Table: GSEA details [plain text format]

  

Fig 2: KEGG\_PANTOTHENATE\_AND\_COA\_BIOSYNTHESIS      
 Blue-Pink O' Gram in the Space of the Analyzed GeneSet

  

Fig 3: KEGG\_PANTOTHENATE\_AND\_COA\_BIOSYNTHESIS: Random ES distribution      
 Gene set null distribution of ES for **KEGG\_PANTOTHENATE\_AND\_COA\_BIOSYNTHESIS**
