# Supplementary material for: Comprehensive analysis of β-catenin target genes in colorectal carcinoma cell lines with deregulated Wnt/β-catenin signaling
Source: BMC Genomics. 2014 Jan 28;15:74. doi: 10.1186/1471-2164-15-74 (PMC3909937; doi:10.1186/1471-2164-15-74)
Supplement: Additional file 5 — GSEA analysis using the KEGG pathway database. This zipped file contains confirming data of the GSEA analysis. The names of the directories containing the files were composed of the term ‘GSEA’, the name of the cell line, e.g. DLD1, SW480, or LS174T, and the pathway database (KEGG). Please use a web browser to view the files with the name ‘index.html’ in the corresponding directories to start exploring the data. [file 1471-2164-15-74-S5.zip › GSEA_LS174T_KEGG/KEGG_PENTOSE_AND_GLUCURONATE_INTERCONVERSIONS.html]

Details for gene set KEGG\_PENTOSE\_AND\_GLUCURONATE\_INTERCONVERSIONS[GSEA]

|  || Dataset | LS174T\_collapsed\_to\_symbols.class.cls#b\_versus\_bg.class.cls#b\_versus\_bg\_repos |
| Phenotype | class.cls#b\_versus\_bg\_repos |
| Upregulated in class | 1 |
| GeneSet | KEGG\_PENTOSE\_AND\_GLUCURONATE\_INTERCONVERSIONS |
| Enrichment Score (ES) | 0.71096694 |
| Normalized Enrichment Score (NES) | 1.775307 |
| Nominal p-value | 0.015444015 |
| FDR q-value | 0.032571945 |
| FWER p-Value | 0.195 |
Table: GSEA Results Summary

  

Fig 1: Enrichment plot: KEGG\_PENTOSE\_AND\_GLUCURONATE\_INTERCONVERSIONS      
 Profile of the Running ES Score & Positions of GeneSet Members on the Rank Ordered List

  

| PROBE | GENE SYMBOL | GENE\_TITLE | RANK IN GENE LIST | RANK METRIC SCORE | RUNNING ES | CORE ENRICHMENT || 1 | UGT2B28 | UGT2B28 Entrez,  Source | UDP glucuronosyltransferase 2 family, polypeptide B28 | 15 | 1.479 | 0.2704 | Yes |
| 2 | UGT2B17 | UGT2B17 Entrez,  Source | UDP glucuronosyltransferase 2 family, polypeptide B17 | 21 | 1.429 | 0.5321 | Yes |
| 3 | UGT2B15 | UGT2B15 Entrez,  Source | UDP glucuronosyltransferase 2 family, polypeptide B15 | 70 | 0.989 | 0.7110 | Yes |
| 4 | UGDH | UGDH Entrez,  Source | UDP-glucose dehydrogenase | 2056 | 0.203 | 0.6466 | No |
| 5 | CRYL1 | CRYL1 Entrez,  Source | crystallin, lambda 1 | 2229 | 0.193 | 0.6731 | No |
| 6 | UGT2A3 | UGT2A3 Entrez,  Source | UDP glucuronosyltransferase 2 family, polypeptide A3 | 4867 | 0.099 | 0.5563 | No |
| 7 | UGP2 | UGP2 Entrez,  Source | UDP-glucose pyrophosphorylase 2 | 4977 | 0.096 | 0.5684 | No |
| 8 | UGT1A9 | UGT1A9 Entrez,  Source | UDP glucuronosyltransferase 1 family, polypeptide A9 | 10361 | -0.005 | 0.2938 | No |
| 9 | GUSB | GUSB Entrez,  Source | glucuronidase, beta | 12719 | -0.046 | 0.1816 | No |
| 10 | XYLB | XYLB Entrez,  Source | xylulokinase homolog (H. influenzae) | 12954 | -0.051 | 0.1790 | No |
| 11 | AKR1B1 | AKR1B1 Entrez,  Source | aldo-keto reductase family 1, member B1 (aldose reductase) | 13320 | -0.058 | 0.1709 | No |
| 12 | RPE | RPE Entrez,  Source | ribulose-5-phosphate-3-epimerase | 15470 | -0.110 | 0.0812 | No |
| 13 | DHDH | DHDH Entrez,  Source | dihydrodiol dehydrogenase (dimeric) | 16184 | -0.135 | 0.0695 | No |
| 14 | DCXR | DCXR Entrez,  Source | dicarbonyl/L-xylulose reductase | 16723 | -0.156 | 0.0706 | No |
| 15 | UGT2B4 | UGT2B4 Entrez,  Source | UDP glucuronosyltransferase 2 family, polypeptide B4 | 19031 | -0.405 | 0.0269 | No |
Table: GSEA details [plain text format]

  

Fig 2: KEGG\_PENTOSE\_AND\_GLUCURONATE\_INTERCONVERSIONS      
 Blue-Pink O' Gram in the Space of the Analyzed GeneSet

  

Fig 3: KEGG\_PENTOSE\_AND\_GLUCURONATE\_INTERCONVERSIONS: Random ES distribution      
 Gene set null distribution of ES for **KEGG\_PENTOSE\_AND\_GLUCURONATE\_INTERCONVERSIONS**
