# Supplementary material for: Comprehensive analysis of β-catenin target genes in colorectal carcinoma cell lines with deregulated Wnt/β-catenin signaling
Source: BMC Genomics. 2014 Jan 28;15:74. doi: 10.1186/1471-2164-15-74 (PMC3909937; doi:10.1186/1471-2164-15-74)
Supplement: Additional file 5 — GSEA analysis using the KEGG pathway database. This zipped file contains confirming data of the GSEA analysis. The names of the directories containing the files were composed of the term ‘GSEA’, the name of the cell line, e.g. DLD1, SW480, or LS174T, and the pathway database (KEGG). Please use a web browser to view the files with the name ‘index.html’ in the corresponding directories to start exploring the data. [file 1471-2164-15-74-S5.zip › GSEA_LS174T_KEGG/KEGG_PHENYLALANINE_METABOLISM.html]

Details for gene set KEGG\_PHENYLALANINE\_METABOLISM[GSEA]

|  || Dataset | LS174T\_collapsed\_to\_symbols.class.cls#b\_versus\_bg.class.cls#b\_versus\_bg\_repos |
| Phenotype | class.cls#b\_versus\_bg\_repos |
| Upregulated in class | 1 |
| GeneSet | KEGG\_PHENYLALANINE\_METABOLISM |
| Enrichment Score (ES) | 0.6303855 |
| Normalized Enrichment Score (NES) | 1.6295 |
| Nominal p-value | 0.011029412 |
| FDR q-value | 0.09833865 |
| FWER p-Value | 0.612 |
Table: GSEA Results Summary

  

Fig 1: Enrichment plot: KEGG\_PHENYLALANINE\_METABOLISM      
 Profile of the Running ES Score & Positions of GeneSet Members on the Rank Ordered List

  

| PROBE | GENE SYMBOL | GENE\_TITLE | RANK IN GENE LIST | RANK METRIC SCORE | RUNNING ES | CORE ENRICHMENT || 1 | ALDH3B2 | ALDH3B2 Entrez,  Source | aldehyde dehydrogenase 3 family, member B2 | 196 | 0.656 | 0.2016 | Yes |
| 2 | ALDH3B1 | ALDH3B1 Entrez,  Source | aldehyde dehydrogenase 3 family, member B1 | 828 | 0.332 | 0.2765 | Yes |
| 3 | ALDH1A3 | ALDH1A3 Entrez,  Source | aldehyde dehydrogenase 1 family, member A3 | 1018 | 0.300 | 0.3637 | Yes |
| 4 | AOC2 | AOC2 Entrez,  Source | amine oxidase, copper containing 2 (retina-specific) | 1136 | 0.281 | 0.4484 | Yes |
| 5 | NAT6 | NAT6 Entrez,  Source | N-acetyltransferase 6 | 1327 | 0.259 | 0.5223 | Yes |
| 6 | DDC | DDC Entrez,  Source | dopa decarboxylase (aromatic L-amino acid decarboxylase) | 1562 | 0.239 | 0.5876 | Yes |
| 7 | ALDH3A1 | ALDH3A1 Entrez,  Source | aldehyde dehydrogenase 3 family, memberA1 | 2020 | 0.205 | 0.6304 | Yes |
| 8 | MAOA | MAOA Entrez,  Source | monoamine oxidase A | 3737 | 0.131 | 0.5849 | No |
| 9 | PRDX6 | PRDX6 Entrez,  Source | peroxiredoxin 6 | 8022 | 0.034 | 0.3767 | No |
| 10 | PAH | PAH Entrez,  Source | phenylalanine hydroxylase | 9884 | 0.003 | 0.2824 | No |
| 11 | MAOB | MAOB Entrez,  Source | monoamine oxidase B | 10309 | -0.004 | 0.2618 | No |
| 12 | MIF | MIF Entrez,  Source | macrophage migration inhibitory factor (glycosylation-inhibiting factor) | 10502 | -0.007 | 0.2542 | No |
| 13 | TAT | TAT Entrez,  Source | tyrosine aminotransferase | 12714 | -0.046 | 0.1560 | No |
| 14 | GOT2 | GOT2 Entrez,  Source | glutamic-oxaloacetic transaminase 2, mitochondrial (aspartate aminotransferase 2) | 14535 | -0.085 | 0.0904 | No |
| 15 | GOT1 | GOT1 Entrez,  Source | glutamic-oxaloacetic transaminase 1, soluble (aspartate aminotransferase 1) | 16278 | -0.138 | 0.0458 | No |
| 16 | AOC3 | AOC3 Entrez,  Source | amine oxidase, copper containing 3 (vascular adhesion protein 1) | 17289 | -0.184 | 0.0536 | No |
| 17 | HPD | HPD Entrez,  Source | 4-hydroxyphenylpyruvate dioxygenase | 17417 | -0.193 | 0.1095 | No |
Table: GSEA details [plain text format]

  

Fig 2: KEGG\_PHENYLALANINE\_METABOLISM      
 Blue-Pink O' Gram in the Space of the Analyzed GeneSet

  

Fig 3: KEGG\_PHENYLALANINE\_METABOLISM: Random ES distribution      
 Gene set null distribution of ES for **KEGG\_PHENYLALANINE\_METABOLISM**
