# Supplementary material for: Comprehensive analysis of β-catenin target genes in colorectal carcinoma cell lines with deregulated Wnt/β-catenin signaling
Source: BMC Genomics. 2014 Jan 28;15:74. doi: 10.1186/1471-2164-15-74 (PMC3909937; doi:10.1186/1471-2164-15-74)
Supplement: Additional file 5 — GSEA analysis using the KEGG pathway database. This zipped file contains confirming data of the GSEA analysis. The names of the directories containing the files were composed of the term ‘GSEA’, the name of the cell line, e.g. DLD1, SW480, or LS174T, and the pathway database (KEGG). Please use a web browser to view the files with the name ‘index.html’ in the corresponding directories to start exploring the data. [file 1471-2164-15-74-S5.zip › GSEA_LS174T_KEGG/KEGG_PORPHYRIN_AND_CHLOROPHYLL_METABOLISM.html]

Details for gene set KEGG\_PORPHYRIN\_AND\_CHLOROPHYLL\_METABOLISM[GSEA]

|  || Dataset | LS174T\_collapsed\_to\_symbols.class.cls#b\_versus\_bg.class.cls#b\_versus\_bg\_repos |
| Phenotype | class.cls#b\_versus\_bg\_repos |
| Upregulated in class | 1 |
| GeneSet | KEGG\_PORPHYRIN\_AND\_CHLOROPHYLL\_METABOLISM |
| Enrichment Score (ES) | 0.61623216 |
| Normalized Enrichment Score (NES) | 1.7949493 |
| Nominal p-value | 0.009451796 |
| FDR q-value | 0.03187347 |
| FWER p-Value | 0.164 |
Table: GSEA Results Summary

  

Fig 1: Enrichment plot: KEGG\_PORPHYRIN\_AND\_CHLOROPHYLL\_METABOLISM      
 Profile of the Running ES Score & Positions of GeneSet Members on the Rank Ordered List

  

| PROBE | GENE SYMBOL | GENE\_TITLE | RANK IN GENE LIST | RANK METRIC SCORE | RUNNING ES | CORE ENRICHMENT || 1 | UGT2B28 | UGT2B28 Entrez,  Source | UDP glucuronosyltransferase 2 family, polypeptide B28 | 15 | 1.479 | 0.2287 | Yes |
| 2 | UGT2B17 | UGT2B17 Entrez,  Source | UDP glucuronosyltransferase 2 family, polypeptide B17 | 21 | 1.429 | 0.4500 | Yes |
| 3 | UGT2B15 | UGT2B15 Entrez,  Source | UDP glucuronosyltransferase 2 family, polypeptide B15 | 70 | 0.989 | 0.6010 | Yes |
| 4 | HMOX1 | HMOX1 Entrez,  Source | heme oxygenase (decycling) 1 | 801 | 0.339 | 0.6162 | Yes |
| 5 | ALAS1 | ALAS1 Entrez,  Source | aminolevulinate, delta-, synthase 1 | 2064 | 0.202 | 0.5830 | No |
| 6 | ALAS2 | ALAS2 Entrez,  Source | aminolevulinate, delta-, synthase 2 (sideroblastic/hypochromic anemia) | 3033 | 0.156 | 0.5576 | No |
| 7 | COX15 | COX15 Entrez,  Source | COX15 homolog, cytochrome c oxidase assembly protein (yeast) | 3668 | 0.133 | 0.5459 | No |
| 8 | BLVRB | BLVRB Entrez,  Source | biliverdin reductase B (flavin reductase (NADPH)) | 3944 | 0.125 | 0.5512 | No |
| 9 | PPOX | PPOX Entrez,  Source | protoporphyrinogen oxidase | 4043 | 0.122 | 0.5651 | No |
| 10 | UGT2A3 | UGT2A3 Entrez,  Source | UDP glucuronosyltransferase 2 family, polypeptide A3 | 4867 | 0.099 | 0.5383 | No |
| 11 | FECH | FECH Entrez,  Source | ferrochelatase (protoporphyria) | 5497 | 0.084 | 0.5191 | No |
| 12 | BLVRA | BLVRA Entrez,  Source | biliverdin reductase A | 5986 | 0.073 | 0.5055 | No |
| 13 | UROD | UROD Entrez,  Source | uroporphyrinogen decarboxylase | 7983 | 0.035 | 0.4087 | No |
| 14 | HMOX2 | HMOX2 Entrez,  Source | heme oxygenase (decycling) 2 | 8521 | 0.025 | 0.3852 | No |
| 15 | COX10 | COX10 Entrez,  Source | COX10 homolog, cytochrome c oxidase assembly protein, heme A: farnesyltransferase (yeast) | 8721 | 0.022 | 0.3783 | No |
| 16 | EPRS | EPRS Entrez,  Source | glutamyl-prolyl-tRNA synthetase | 9778 | 0.005 | 0.3250 | No |
| 17 | ALAD | ALAD Entrez,  Source | aminolevulinate, delta-, dehydratase | 10078 | 0.000 | 0.3097 | No |
| 18 | UGT1A9 | UGT1A9 Entrez,  Source | UDP glucuronosyltransferase 1 family, polypeptide A9 | 10361 | -0.005 | 0.2959 | No |
| 19 | MMAB | MMAB Entrez,  Source | methylmalonic aciduria (cobalamin deficiency) cblB type | 10376 | -0.005 | 0.2960 | No |
| 20 | FTH1 | FTH1 Entrez,  Source | ferritin, heavy polypeptide 1 | 10405 | -0.005 | 0.2953 | No |
| 21 | UROS | UROS Entrez,  Source | uroporphyrinogen III synthase (congenital erythropoietic porphyria) | 11560 | -0.026 | 0.2402 | No |
| 22 | HMBS | HMBS Entrez,  Source | hydroxymethylbilane synthase | 12238 | -0.037 | 0.2113 | No |
| 23 | GUSB | GUSB Entrez,  Source | glucuronidase, beta | 12719 | -0.046 | 0.1939 | No |
| 24 | HCCS | HCCS Entrez,  Source | holocytochrome c synthase (cytochrome c heme-lyase) | 13423 | -0.060 | 0.1673 | No |
| 25 | CP | CP Entrez,  Source | ceruloplasmin (ferroxidase) | 15797 | -0.120 | 0.0643 | No |
| 26 | EARS2 | EARS2 Entrez,  Source | glutamyl-tRNA synthetase 2 (mitochondrial)(putative) | 16056 | -0.130 | 0.0712 | No |
| 27 | CPOX | CPOX Entrez,  Source | coproporphyrinogen oxidase | 18491 | -0.290 | -0.0084 | No |
| 28 | UGT2B4 | UGT2B4 Entrez,  Source | UDP glucuronosyltransferase 2 family, polypeptide B4 | 19031 | -0.405 | 0.0269 | No |
Table: GSEA details [plain text format]

  

Fig 2: KEGG\_PORPHYRIN\_AND\_CHLOROPHYLL\_METABOLISM      
 Blue-Pink O' Gram in the Space of the Analyzed GeneSet

  

Fig 3: KEGG\_PORPHYRIN\_AND\_CHLOROPHYLL\_METABOLISM: Random ES distribution      
 Gene set null distribution of ES for **KEGG\_PORPHYRIN\_AND\_CHLOROPHYLL\_METABOLISM**
