# Supplementary material for: Comprehensive analysis of β-catenin target genes in colorectal carcinoma cell lines with deregulated Wnt/β-catenin signaling
Source: BMC Genomics. 2014 Jan 28;15:74. doi: 10.1186/1471-2164-15-74 (PMC3909937; doi:10.1186/1471-2164-15-74)
Supplement: Additional file 5 — GSEA analysis using the KEGG pathway database. This zipped file contains confirming data of the GSEA analysis. The names of the directories containing the files were composed of the term ‘GSEA’, the name of the cell line, e.g. DLD1, SW480, or LS174T, and the pathway database (KEGG). Please use a web browser to view the files with the name ‘index.html’ in the corresponding directories to start exploring the data. [file 1471-2164-15-74-S5.zip › GSEA_LS174T_KEGG/KEGG_PPAR_SIGNALING_PATHWAY.html]

Details for gene set KEGG\_PPAR\_SIGNALING\_PATHWAY[GSEA]

|  || Dataset | LS174T\_collapsed\_to\_symbols.class.cls#b\_versus\_bg.class.cls#b\_versus\_bg\_repos |
| Phenotype | class.cls#b\_versus\_bg\_repos |
| Upregulated in class | 1 |
| GeneSet | KEGG\_PPAR\_SIGNALING\_PATHWAY |
| Enrichment Score (ES) | 0.43729255 |
| Normalized Enrichment Score (NES) | 1.5109639 |
| Nominal p-value | 0.037300177 |
| FDR q-value | 0.18091558 |
| FWER p-Value | 0.925 |
Table: GSEA Results Summary

  

Fig 1: Enrichment plot: KEGG\_PPAR\_SIGNALING\_PATHWAY      
 Profile of the Running ES Score & Positions of GeneSet Members on the Rank Ordered List

  

| PROBE | GENE SYMBOL | GENE\_TITLE | RANK IN GENE LIST | RANK METRIC SCORE | RUNNING ES | CORE ENRICHMENT || 1 | FABP1 | FABP1 Entrez,  Source | fatty acid binding protein 1, liver | 2 | 2.560 | 0.1901 | Yes |
| 2 | HMGCS2 | HMGCS2 Entrez,  Source | 3-hydroxy-3-methylglutaryl-Coenzyme A synthase 2 (mitochondrial) | 122 | 0.800 | 0.2434 | Yes |
| 3 | ACOX2 | ACOX2 Entrez,  Source | acyl-Coenzyme A oxidase 2, branched chain | 172 | 0.691 | 0.2923 | Yes |
| 4 | EHHADH | EHHADH Entrez,  Source | enoyl-Coenzyme A, hydratase/3-hydroxyacyl Coenzyme A dehydrogenase | 310 | 0.528 | 0.3245 | Yes |
| 5 | LPL | LPL Entrez,  Source | lipoprotein lipase | 782 | 0.342 | 0.3257 | Yes |
| 6 | ACOX1 | ACOX1 Entrez,  Source | acyl-Coenzyme A oxidase 1, palmitoyl | 791 | 0.341 | 0.3506 | Yes |
| 7 | NR1H3 | NR1H3 Entrez,  Source | nuclear receptor subfamily 1, group H, member 3 | 1079 | 0.290 | 0.3575 | Yes |
| 8 | SLC27A1 | SLC27A1 Entrez,  Source | solute carrier family 27 (fatty acid transporter), member 1 | 1299 | 0.263 | 0.3658 | Yes |
| 9 | ACSL4 | ACSL4 Entrez,  Source | acyl-CoA synthetase long-chain family member 4 | 1548 | 0.241 | 0.3709 | Yes |
| 10 | ADIPOQ | ADIPOQ Entrez,  Source | adiponectin, C1Q and collagen domain containing | 1853 | 0.217 | 0.3715 | Yes |
| 11 | GK | GK Entrez,  Source | glycerol kinase | 1967 | 0.210 | 0.3812 | Yes |
| 12 | APOA2 | APOA2 Entrez,  Source | apolipoprotein A-II | 2327 | 0.187 | 0.3767 | Yes |
| 13 | CD36 | CD36 Entrez,  Source | CD36 molecule (thrombospondin receptor) | 2507 | 0.177 | 0.3807 | Yes |
| 14 | CPT1B | CPT1B Entrez,  Source | carnitine palmitoyltransferase 1B (muscle) | 2658 | 0.171 | 0.3857 | Yes |
| 15 | ACSL1 | ACSL1 Entrez,  Source | acyl-CoA synthetase long-chain family member 1 | 2659 | 0.171 | 0.3984 | Yes |
| 16 | CPT2 | CPT2 Entrez,  Source | carnitine palmitoyltransferase II | 2890 | 0.161 | 0.3986 | Yes |
| 17 | ME1 | ME1 Entrez,  Source | malic enzyme 1, NADP(+)-dependent, cytosolic | 3099 | 0.153 | 0.3993 | Yes |
| 18 | FABP6 | FABP6 Entrez,  Source | fatty acid binding protein 6, ileal (gastrotropin) | 3176 | 0.151 | 0.4066 | Yes |
| 19 | PLTP | PLTP Entrez,  Source | phospholipid transfer protein | 3220 | 0.149 | 0.4155 | Yes |
| 20 | CYP8B1 | CYP8B1 Entrez,  Source | cytochrome P450, family 8, subfamily B, polypeptide 1 | 3245 | 0.149 | 0.4253 | Yes |
| 21 | CPT1C | CPT1C Entrez,  Source | carnitine palmitoyltransferase 1C | 3386 | 0.144 | 0.4288 | Yes |
| 22 | FABP3 | FABP3 Entrez,  Source | fatty acid binding protein 3, muscle and heart (mammary-derived growth inhibitor) | 3594 | 0.136 | 0.4283 | Yes |
| 23 | CPT1A | CPT1A Entrez,  Source | carnitine palmitoyltransferase 1A (liver) | 3674 | 0.133 | 0.4342 | Yes |
| 24 | SCP2 | SCP2 Entrez,  Source | sterol carrier protein 2 | 3827 | 0.129 | 0.4359 | Yes |
| 25 | APOA5 | APOA5 Entrez,  Source | apolipoprotein A-V | 3999 | 0.123 | 0.4363 | Yes |
| 26 | FABP2 | FABP2 Entrez,  Source | fatty acid binding protein 2, intestinal | 4233 | 0.116 | 0.4330 | Yes |
| 27 | ACAA1 | ACAA1 Entrez,  Source | acetyl-Coenzyme A acyltransferase 1 (peroxisomal 3-oxoacyl-Coenzyme A thiolase) | 4315 | 0.114 | 0.4373 | Yes |
| 28 | PCK1 | PCK1 Entrez,  Source | phosphoenolpyruvate carboxykinase 1 (soluble) | 4630 | 0.105 | 0.4290 | No |
| 29 | PPARG | PPARG Entrez,  Source | peroxisome proliferative activated receptor, gamma | 5150 | 0.092 | 0.4092 | No |
| 30 | PPARA | PPARA Entrez,  Source | peroxisome proliferative activated receptor, alpha | 5176 | 0.091 | 0.4147 | No |
| 31 | FABP4 | FABP4 Entrez,  Source | fatty acid binding protein 4, adipocyte | 5624 | 0.081 | 0.3978 | No |
| 32 | ILK | ILK Entrez,  Source | integrin-linked kinase | 5773 | 0.077 | 0.3959 | No |
| 33 | CYP27A1 | CYP27A1 Entrez,  Source | cytochrome P450, family 27, subfamily A, polypeptide 1 | 5876 | 0.075 | 0.3963 | No |
| 34 | SLC27A4 | SLC27A4 Entrez,  Source | solute carrier family 27 (fatty acid transporter), member 4 | 6308 | 0.067 | 0.3792 | No |
| 35 | AQP7 | AQP7 Entrez,  Source | aquaporin 7 | 6660 | 0.060 | 0.3656 | No |
| 36 | ACADM | ACADM Entrez,  Source | acyl-Coenzyme A dehydrogenase, C-4 to C-12 straight chain | 6692 | 0.059 | 0.3684 | No |
| 37 | CYP4A11 | CYP4A11 Entrez,  Source | cytochrome P450, family 4, subfamily A, polypeptide 11 | 7273 | 0.048 | 0.3422 | No |
| 38 | SCD5 | SCD5 Entrez,  Source | stearoyl-CoA desaturase 5 | 7346 | 0.047 | 0.3421 | No |
| 39 | FABP7 | FABP7 Entrez,  Source | fatty acid binding protein 7, brain | 7896 | 0.037 | 0.3166 | No |
| 40 | ACOX3 | ACOX3 Entrez,  Source | acyl-Coenzyme A oxidase 3, pristanoyl | 8596 | 0.024 | 0.2825 | No |
| 41 | RXRB | RXRB Entrez,  Source | retinoid X receptor, beta | 8977 | 0.018 | 0.2643 | No |
| 42 | CYP7A1 | CYP7A1 Entrez,  Source | cytochrome P450, family 7, subfamily A, polypeptide 1 | 9152 | 0.015 | 0.2565 | No |
| 43 | ANGPTL4 | ANGPTL4 Entrez,  Source | angiopoietin-like 4 | 9174 | 0.014 | 0.2565 | No |
| 44 | APOA1 | APOA1 Entrez,  Source | apolipoprotein A-I | 9567 | 0.008 | 0.2370 | No |
| 45 | ACADL | ACADL Entrez,  Source | acyl-Coenzyme A dehydrogenase, long chain | 9625 | 0.007 | 0.2346 | No |
| 46 | ACSL3 | ACSL3 Entrez,  Source | acyl-CoA synthetase long-chain family member 3 | 10004 | 0.001 | 0.2153 | No |
| 47 | UBC | UBC Entrez,  Source | ubiquitin C | 10022 | 0.001 | 0.2145 | No |
| 48 | RXRA | RXRA Entrez,  Source | retinoid X receptor, alpha | 10297 | -0.003 | 0.2007 | No |
| 49 | GK2 | GK2 Entrez,  Source | glycerol kinase 2 | 10821 | -0.012 | 0.1747 | No |
| 50 | DBI | DBI Entrez,  Source | diazepam binding inhibitor (GABA receptor modulator, acyl-Coenzyme A binding protein) | 10866 | -0.013 | 0.1734 | No |
| 51 | RXRG | RXRG Entrez,  Source | retinoid X receptor, gamma | 11789 | -0.029 | 0.1283 | No |
| 52 | ACSL6 | ACSL6 Entrez,  Source | acyl-CoA synthetase long-chain family member 6 | 12223 | -0.037 | 0.1088 | No |
| 53 | APOC3 | APOC3 Entrez,  Source | apolipoprotein C-III | 13301 | -0.058 | 0.0579 | No |
| 54 | PDPK1 | PDPK1 Entrez,  Source | 3-phosphoinositide dependent protein kinase-1 | 13399 | -0.060 | 0.0573 | No |
| 55 | UCP1 | UCP1 Entrez,  Source | uncoupling protein 1 (mitochondrial, proton carrier) | 13500 | -0.062 | 0.0568 | No |
| 56 | SLC27A6 | SLC27A6 Entrez,  Source | solute carrier family 27 (fatty acid transporter), member 6 | 13509 | -0.062 | 0.0609 | No |
| 57 | SLC27A2 | SLC27A2 Entrez,  Source | solute carrier family 27 (fatty acid transporter), member 2 | 13689 | -0.065 | 0.0566 | No |
| 58 | SLC27A5 | SLC27A5 Entrez,  Source | solute carrier family 27 (fatty acid transporter), member 5 | 14551 | -0.086 | 0.0188 | No |
| 59 | PCK2 | PCK2 Entrez,  Source | phosphoenolpyruvate carboxykinase 2 (mitochondrial) | 16497 | -0.146 | -0.0701 | No |
| 60 | MMP1 | MMP1 Entrez,  Source | matrix metallopeptidase 1 (interstitial collagenase) | 17356 | -0.189 | -0.1001 | No |
| 61 | FADS2 | FADS2 Entrez,  Source | fatty acid desaturase 2 | 18222 | -0.259 | -0.1252 | No |
| 62 | SCD | SCD Entrez,  Source | stearoyl-CoA desaturase (delta-9-desaturase) | 18532 | -0.297 | -0.1190 | No |
| 63 | PPARD | PPARD Entrez,  Source | peroxisome proliferative activated receptor, delta | 18739 | -0.330 | -0.1050 | No |
| 64 | OLR1 | OLR1 Entrez,  Source | oxidised low density lipoprotein (lectin-like) receptor 1 | 18991 | -0.393 | -0.0887 | No |
| 65 | SORBS1 | SORBS1 Entrez,  Source | sorbin and SH3 domain containing 1 | 19413 | -0.651 | -0.0620 | No |
| 66 | ACSL5 | ACSL5 Entrez,  Source | acyl-CoA synthetase long-chain family member 5 | 19523 | -0.932 | 0.0017 | No |
Table: GSEA details [plain text format]

  

Fig 2: KEGG\_PPAR\_SIGNALING\_PATHWAY      
 Blue-Pink O' Gram in the Space of the Analyzed GeneSet

  

Fig 3: KEGG\_PPAR\_SIGNALING\_PATHWAY: Random ES distribution      
 Gene set null distribution of ES for **KEGG\_PPAR\_SIGNALING\_PATHWAY**
