# Supplementary material for: Comprehensive analysis of β-catenin target genes in colorectal carcinoma cell lines with deregulated Wnt/β-catenin signaling
Source: BMC Genomics. 2014 Jan 28;15:74. doi: 10.1186/1471-2164-15-74 (PMC3909937; doi:10.1186/1471-2164-15-74)
Supplement: Additional file 5 — GSEA analysis using the KEGG pathway database. This zipped file contains confirming data of the GSEA analysis. The names of the directories containing the files were composed of the term ‘GSEA’, the name of the cell line, e.g. DLD1, SW480, or LS174T, and the pathway database (KEGG). Please use a web browser to view the files with the name ‘index.html’ in the corresponding directories to start exploring the data. [file 1471-2164-15-74-S5.zip › GSEA_LS174T_KEGG/KEGG_PROGESTERONE_MEDIATED_OOCYTE_MATURATION.html]

Details for gene set KEGG\_PROGESTERONE\_MEDIATED\_OOCYTE\_MATURATION[GSEA]

|  || Dataset | LS174T\_collapsed\_to\_symbols.class.cls#b\_versus\_bg.class.cls#b\_versus\_bg\_repos |
| Phenotype | class.cls#b\_versus\_bg\_repos |
| Upregulated in class | 0 |
| GeneSet | KEGG\_PROGESTERONE\_MEDIATED\_OOCYTE\_MATURATION |
| Enrichment Score (ES) | -0.38033637 |
| Normalized Enrichment Score (NES) | -1.4018537 |
| Nominal p-value | 0.035377357 |
| FDR q-value | 0.1987386 |
| FWER p-Value | 0.978 |
Table: GSEA Results Summary

  

Fig 1: Enrichment plot: KEGG\_PROGESTERONE\_MEDIATED\_OOCYTE\_MATURATION      
 Profile of the Running ES Score & Positions of GeneSet Members on the Rank Ordered List

  

| PROBE | GENE SYMBOL | GENE\_TITLE | RANK IN GENE LIST | RANK METRIC SCORE | RUNNING ES | CORE ENRICHMENT || 1 | PGR | PGR Entrez,  Source | progesterone receptor | 1144 | 0.280 | -0.0334 | No |
| 2 | PIK3R3 | PIK3R3 Entrez,  Source | phosphoinositide-3-kinase, regulatory subunit 3 (p55, gamma) | 1292 | 0.264 | -0.0171 | No |
| 3 | PIK3CG | PIK3CG Entrez,  Source | phosphoinositide-3-kinase, catalytic, gamma polypeptide | 1867 | 0.216 | -0.0271 | No |
| 4 | PRKACG | PRKACG Entrez,  Source | protein kinase, cAMP-dependent, catalytic, gamma | 2268 | 0.190 | -0.0305 | No |
| 5 | KRAS | KRAS Entrez,  Source | v-Ki-ras2 Kirsten rat sarcoma viral oncogene homolog | 2305 | 0.189 | -0.0153 | No |
| 6 | ANAPC13 | ANAPC13 Entrez,  Source | anaphase promoting complex subunit 13 | 2876 | 0.162 | -0.0299 | No |
| 7 | PIK3R5 | PIK3R5 Entrez,  Source | phosphoinositide-3-kinase, regulatory subunit 5, p101 | 3094 | 0.154 | -0.0272 | No |
| 8 | PIK3CA | PIK3CA Entrez,  Source | phosphoinositide-3-kinase, catalytic, alpha polypeptide | 3224 | 0.149 | -0.0203 | No |
| 9 | ADCY8 | ADCY8 Entrez,  Source | adenylate cyclase 8 (brain) | 4003 | 0.123 | -0.0492 | No |
| 10 | MOS | MOS Entrez,  Source | v-mos Moloney murine sarcoma viral oncogene homolog | 4038 | 0.122 | -0.0398 | No |
| 11 | ADCY9 | ADCY9 Entrez,  Source | adenylate cyclase 9 | 4106 | 0.120 | -0.0324 | No |
| 12 | CCNA1 | CCNA1 Entrez,  Source | cyclin A1 | 4153 | 0.119 | -0.0241 | No |
| 13 | ADCY1 | ADCY1 Entrez,  Source | adenylate cyclase 1 (brain) | 4182 | 0.118 | -0.0149 | No |
| 14 | PRKACB | PRKACB Entrez,  Source | protein kinase, cAMP-dependent, catalytic, beta | 4257 | 0.115 | -0.0083 | No |
| 15 | MAPK10 | MAPK10 Entrez,  Source | mitogen-activated protein kinase 10 | 4498 | 0.109 | -0.0108 | No |
| 16 | CDC16 | CDC16 Entrez,  Source | CDC16 cell division cycle 16 homolog (S. cerevisiae) | 4588 | 0.106 | -0.0058 | No |
| 17 | PIK3R2 | PIK3R2 Entrez,  Source | phosphoinositide-3-kinase, regulatory subunit 2 (p85 beta) | 4617 | 0.106 | 0.0023 | No |
| 18 | PIK3CD | PIK3CD Entrez,  Source | phosphoinositide-3-kinase, catalytic, delta polypeptide | 4686 | 0.104 | 0.0082 | No |
| 19 | CCNB3 | CCNB3 Entrez,  Source | cyclin B3 | 4731 | 0.103 | 0.0152 | No |
| 20 | ADCY7 | ADCY7 Entrez,  Source | adenylate cyclase 7 | 4771 | 0.102 | 0.0224 | No |
| 21 | MAPK3 | MAPK3 Entrez,  Source | mitogen-activated protein kinase 3 | 4947 | 0.097 | 0.0222 | No |
| 22 | RPS6KA3 | RPS6KA3 Entrez,  Source | ribosomal protein S6 kinase, 90kDa, polypeptide 3 | 5521 | 0.083 | 0.0003 | No |
| 23 | MAPK8 | MAPK8 Entrez,  Source | mitogen-activated protein kinase 8 | 5571 | 0.082 | 0.0052 | No |
| 24 | CPEB1 | CPEB1 Entrez,  Source | cytoplasmic polyadenylation element binding protein 1 | 5662 | 0.080 | 0.0078 | No |
| 25 | MAPK14 | MAPK14 Entrez,  Source | mitogen-activated protein kinase 14 | 5674 | 0.080 | 0.0144 | No |
| 26 | SPDYA | SPDYA Entrez,  Source | speedy homolog A (Drosophila) | 5996 | 0.073 | 0.0045 | No |
| 27 | ADCY4 | ADCY4 Entrez,  Source | adenylate cyclase 4 | 6242 | 0.068 | -0.0019 | No |
| 28 | PDE3A | PDE3A Entrez,  Source | phosphodiesterase 3A, cGMP-inhibited | 6482 | 0.063 | -0.0085 | No |
| 29 | INS | INS Entrez,  Source | insulin | 6846 | 0.056 | -0.0220 | No |
| 30 | IGF1R | IGF1R Entrez,  Source | insulin-like growth factor 1 receptor | 7745 | 0.040 | -0.0646 | No |
| 31 | IGF1 | IGF1 Entrez,  Source | insulin-like growth factor 1 (somatomedin C) | 7808 | 0.039 | -0.0643 | No |
| 32 | ADCY5 | ADCY5 Entrez,  Source | adenylate cyclase 5 | 8116 | 0.032 | -0.0771 | No |
| 33 | MAP2K1 | MAP2K1 Entrez,  Source | mitogen-activated protein kinase kinase 1 | 8349 | 0.028 | -0.0865 | No |
| 34 | GNAI3 | GNAI3 Entrez,  Source | guanine nucleotide binding protein (G protein), alpha inhibiting activity polypeptide 3 | 8492 | 0.026 | -0.0914 | No |
| 35 | AKT2 | AKT2 Entrez,  Source | v-akt murine thymoma viral oncogene homolog 2 | 9288 | 0.012 | -0.1311 | No |
| 36 | GNAI2 | GNAI2 Entrez,  Source | guanine nucleotide binding protein (G protein), alpha inhibiting activity polypeptide 2 | 9649 | 0.007 | -0.1490 | No |
| 37 | RPS6KA1 | RPS6KA1 Entrez,  Source | ribosomal protein S6 kinase, 90kDa, polypeptide 1 | 9863 | 0.003 | -0.1596 | No |
| 38 | ANAPC2 | ANAPC2 Entrez,  Source | anaphase promoting complex subunit 2 | 9963 | 0.002 | -0.1645 | No |
| 39 | CDC25B | CDC25B Entrez,  Source | cell division cycle 25B | 10129 | -0.001 | -0.1730 | No |
| 40 | ARAF | ARAF Entrez,  Source | v-raf murine sarcoma 3611 viral oncogene homolog | 10578 | -0.008 | -0.1952 | No |
| 41 | FZR1 | FZR1 Entrez,  Source | fizzy/cell division cycle 20 related 1 (Drosophila) | 10724 | -0.010 | -0.2018 | No |
| 42 | RPS6KA6 | RPS6KA6 Entrez,  Source | ribosomal protein S6 kinase, 90kDa, polypeptide 6 | 10844 | -0.012 | -0.2068 | No |
| 43 | MAPK11 | MAPK11 Entrez,  Source | mitogen-activated protein kinase 11 | 11797 | -0.030 | -0.2530 | No |
| 44 | HSP90AB1 | HSP90AB1 Entrez,  Source | heat shock protein 90kDa alpha (cytosolic), class B member 1 | 11863 | -0.031 | -0.2535 | No |
| 45 | ANAPC11 | ANAPC11 Entrez,  Source | APC11 anaphase promoting complex subunit 11 homolog (yeast) | 11989 | -0.033 | -0.2570 | No |
| 46 | ADCY6 | ADCY6 Entrez,  Source | adenylate cyclase 6 | 12006 | -0.033 | -0.2548 | No |
| 47 | BRAF | BRAF Entrez,  Source | v-raf murine sarcoma viral oncogene homolog B1 | 12037 | -0.033 | -0.2534 | No |
| 48 | ADCY2 | ADCY2 Entrez,  Source | adenylate cyclase 2 (brain) | 12538 | -0.043 | -0.2751 | No |
| 49 | ANAPC4 | ANAPC4 Entrez,  Source | anaphase promoting complex subunit 4 | 12643 | -0.045 | -0.2764 | No |
| 50 | CDC23 | CDC23 Entrez,  Source | CDC23 (cell division cycle 23, yeast, homolog) | 12882 | -0.049 | -0.2842 | No |
| 51 | HSP90AA1 | HSP90AA1 Entrez,  Source | heat shock protein 90kDa alpha (cytosolic), class A member 1 | 12953 | -0.051 | -0.2832 | No |
| 52 | MAPK1 | MAPK1 Entrez,  Source | mitogen-activated protein kinase 1 | 13089 | -0.053 | -0.2854 | No |
| 53 | ANAPC1 | ANAPC1 Entrez,  Source | anaphase promoting complex subunit 1 | 13231 | -0.056 | -0.2875 | No |
| 54 | PDE3B | PDE3B Entrez,  Source | phosphodiesterase 3B, cGMP-inhibited | 13564 | -0.063 | -0.2989 | No |
| 55 | RAF1 | RAF1 Entrez,  Source | v-raf-1 murine leukemia viral oncogene homolog 1 | 13610 | -0.064 | -0.2955 | No |
| 56 | CDC27 | CDC27 Entrez,  Source | cell division cycle 27 | 13859 | -0.070 | -0.3019 | No |
| 57 | PIK3R1 | PIK3R1 Entrez,  Source | phosphoinositide-3-kinase, regulatory subunit 1 (p85 alpha) | 13886 | -0.070 | -0.2969 | No |
| 58 | MAPK12 | MAPK12 Entrez,  Source | mitogen-activated protein kinase 12 | 14771 | -0.091 | -0.3340 | No |
| 59 | PIK3CB | PIK3CB Entrez,  Source | phosphoinositide-3-kinase, catalytic, beta polypeptide | 15135 | -0.101 | -0.3436 | No |
| 60 | ANAPC5 | ANAPC5 Entrez,  Source | anaphase promoting complex subunit 5 | 15189 | -0.103 | -0.3370 | No |
| 61 | GNAI1 | GNAI1 Entrez,  Source | guanine nucleotide binding protein (G protein), alpha inhibiting activity polypeptide 1 | 15368 | -0.108 | -0.3364 | No |
| 62 | ANAPC7 | ANAPC7 Entrez,  Source | anaphase promoting complex subunit 7 | 15479 | -0.111 | -0.3321 | No |
| 63 | AKT3 | AKT3 Entrez,  Source | v-akt murine thymoma viral oncogene homolog 3 (protein kinase B, gamma) | 15597 | -0.114 | -0.3278 | No |
| 64 | ANAPC10 | ANAPC10 Entrez,  Source | anaphase promoting complex subunit 10 | 15957 | -0.126 | -0.3349 | No |
| 65 | PRKACA | PRKACA Entrez,  Source | protein kinase, cAMP-dependent, catalytic, alpha | 16647 | -0.153 | -0.3565 | No |
| 66 | MAPK13 | MAPK13 Entrez,  Source | mitogen-activated protein kinase 13 | 16657 | -0.153 | -0.3431 | No |
| 67 | RPS6KA2 | RPS6KA2 Entrez,  Source | ribosomal protein S6 kinase, 90kDa, polypeptide 2 | 16682 | -0.154 | -0.3304 | No |
| 68 | MAPK9 | MAPK9 Entrez,  Source | mitogen-activated protein kinase 9 | 17655 | -0.209 | -0.3615 | Yes |
| 69 | ADCY3 | ADCY3 Entrez,  Source | adenylate cyclase 3 | 18015 | -0.238 | -0.3584 | Yes |
| 70 | MAD2L2 | MAD2L2 Entrez,  Source | MAD2 mitotic arrest deficient-like 2 (yeast) | 18108 | -0.246 | -0.3409 | Yes |
| 71 | PRKX | PRKX Entrez,  Source | protein kinase, X-linked | 18151 | -0.251 | -0.3204 | Yes |
| 72 | CCNB2 | CCNB2 Entrez,  Source | cyclin B2 | 18253 | -0.262 | -0.3020 | Yes |
| 73 | AKT1 | AKT1 Entrez,  Source | v-akt murine thymoma viral oncogene homolog 1 | 18283 | -0.266 | -0.2795 | Yes |
| 74 | CCNB1 | CCNB1 Entrez,  Source | cyclin B1 | 18465 | -0.286 | -0.2630 | Yes |
| 75 | PLK1 | PLK1 Entrez,  Source | polo-like kinase 1 (Drosophila) | 18596 | -0.306 | -0.2420 | Yes |
| 76 | MAD2L1 | MAD2L1 Entrez,  Source | MAD2 mitotic arrest deficient-like 1 (yeast) | 18616 | -0.310 | -0.2150 | Yes |
| 77 | PKMYT1 | PKMYT1 Entrez,  Source | protein kinase, membrane associated tyrosine/threonine 1 | 18637 | -0.313 | -0.1878 | Yes |
| 78 | CDK2 | CDK2 Entrez,  Source | cyclin-dependent kinase 2 | 18981 | -0.390 | -0.1702 | Yes |
| 79 | CDC25A | CDC25A Entrez,  Source | cell division cycle 25A | 19237 | -0.492 | -0.1388 | Yes |
| 80 | BUB1 | BUB1 Entrez,  Source | BUB1 budding uninhibited by benzimidazoles 1 homolog (yeast) | 19278 | -0.527 | -0.0934 | Yes |
| 81 | CCNA2 | CCNA2 Entrez,  Source | cyclin A2 | 19327 | -0.557 | -0.0455 | Yes |
| 82 | CDC25C | CDC25C Entrez,  Source | cell division cycle 25C | 19398 | -0.634 | 0.0081 | Yes |
Table: GSEA details [plain text format]

  

Fig 2: KEGG\_PROGESTERONE\_MEDIATED\_OOCYTE\_MATURATION      
 Blue-Pink O' Gram in the Space of the Analyzed GeneSet

  

Fig 3: KEGG\_PROGESTERONE\_MEDIATED\_OOCYTE\_MATURATION: Random ES distribution      
 Gene set null distribution of ES for **KEGG\_PROGESTERONE\_MEDIATED\_OOCYTE\_MATURATION**
