# Supplementary material for: Comprehensive analysis of β-catenin target genes in colorectal carcinoma cell lines with deregulated Wnt/β-catenin signaling
Source: BMC Genomics. 2014 Jan 28;15:74. doi: 10.1186/1471-2164-15-74 (PMC3909937; doi:10.1186/1471-2164-15-74)
Supplement: Additional file 5 — GSEA analysis using the KEGG pathway database. This zipped file contains confirming data of the GSEA analysis. The names of the directories containing the files were composed of the term ‘GSEA’, the name of the cell line, e.g. DLD1, SW480, or LS174T, and the pathway database (KEGG). Please use a web browser to view the files with the name ‘index.html’ in the corresponding directories to start exploring the data. [file 1471-2164-15-74-S5.zip › GSEA_LS174T_KEGG/KEGG_PROSTATE_CANCER.html]

Details for gene set KEGG\_PROSTATE\_CANCER[GSEA]

|  || Dataset | LS174T\_collapsed\_to\_symbols.class.cls#b\_versus\_bg.class.cls#b\_versus\_bg\_repos |
| Phenotype | class.cls#b\_versus\_bg\_repos |
| Upregulated in class | 0 |
| GeneSet | KEGG\_PROSTATE\_CANCER |
| Enrichment Score (ES) | -0.3783793 |
| Normalized Enrichment Score (NES) | -1.4035758 |
| Nominal p-value | 0.035377357 |
| FDR q-value | 0.20696673 |
| FWER p-Value | 0.976 |
Table: GSEA Results Summary

  

Fig 1: Enrichment plot: KEGG\_PROSTATE\_CANCER      
 Profile of the Running ES Score & Positions of GeneSet Members on the Rank Ordered List

  

| PROBE | GENE SYMBOL | GENE\_TITLE | RANK IN GENE LIST | RANK METRIC SCORE | RUNNING ES | CORE ENRICHMENT || 1 | TCF7L1 | TCF7L1 Entrez,  Source | transcription factor 7-like 1 (T-cell specific, HMG-box) | 461 | 0.429 | 0.0098 | No |
| 2 | CREB3L1 | CREB3L1 Entrez,  Source | cAMP responsive element binding protein 3-like 1 | 523 | 0.409 | 0.0387 | No |
| 3 | TGFA | TGFA Entrez,  Source | transforming growth factor, alpha | 582 | 0.391 | 0.0663 | No |
| 4 | EGF | EGF Entrez,  Source | epidermal growth factor (beta-urogastrone) | 768 | 0.346 | 0.0838 | No |
| 5 | PIK3R3 | PIK3R3 Entrez,  Source | phosphoinositide-3-kinase, regulatory subunit 3 (p55, gamma) | 1292 | 0.264 | 0.0776 | No |
| 6 | PIK3CG | PIK3CG Entrez,  Source | phosphoinositide-3-kinase, catalytic, gamma polypeptide | 1867 | 0.216 | 0.0650 | No |
| 7 | CREB3 | CREB3 Entrez,  Source | cAMP responsive element binding protein 3 | 1991 | 0.208 | 0.0749 | No |
| 8 | TCF7L2 | TCF7L2 Entrez,  Source | transcription factor 7-like 2 (T-cell specific, HMG-box) | 2119 | 0.199 | 0.0840 | No |
| 9 | KRAS | KRAS Entrez,  Source | v-Ki-ras2 Kirsten rat sarcoma viral oncogene homolog | 2305 | 0.189 | 0.0892 | No |
| 10 | CREB3L4 | CREB3L4 Entrez,  Source | cAMP responsive element binding protein 3-like 4 | 2396 | 0.183 | 0.0989 | No |
| 11 | E2F3 | E2F3 Entrez,  Source | E2F transcription factor 3 | 2456 | 0.180 | 0.1099 | No |
| 12 | PDGFD | PDGFD Entrez,  Source | platelet derived growth factor D | 2863 | 0.162 | 0.1018 | No |
| 13 | NRAS | NRAS Entrez,  Source | neuroblastoma RAS viral (v-ras) oncogene homolog | 2918 | 0.160 | 0.1115 | No |
| 14 | PIK3R5 | PIK3R5 Entrez,  Source | phosphoinositide-3-kinase, regulatory subunit 5, p101 | 3094 | 0.154 | 0.1145 | No |
| 15 | PIK3CA | PIK3CA Entrez,  Source | phosphoinositide-3-kinase, catalytic, alpha polypeptide | 3224 | 0.149 | 0.1195 | No |
| 16 | PIK3R2 | PIK3R2 Entrez,  Source | phosphoinositide-3-kinase, regulatory subunit 2 (p85 beta) | 4617 | 0.106 | 0.0563 | No |
| 17 | PIK3CD | PIK3CD Entrez,  Source | phosphoinositide-3-kinase, catalytic, delta polypeptide | 4686 | 0.104 | 0.0609 | No |
| 18 | MAPK3 | MAPK3 Entrez,  Source | mitogen-activated protein kinase 3 | 4947 | 0.097 | 0.0551 | No |
| 19 | IKBKB | IKBKB Entrez,  Source | inhibitor of kappa light polypeptide gene enhancer in B-cells, kinase beta | 5465 | 0.085 | 0.0352 | No |
| 20 | CREB1 | CREB1 Entrez,  Source | cAMP responsive element binding protein 1 | 5811 | 0.077 | 0.0235 | No |
| 21 | CDKN1B | CDKN1B Entrez,  Source | cyclin-dependent kinase inhibitor 1B (p27, Kip1) | 5946 | 0.074 | 0.0224 | No |
| 22 | CREB3L3 | CREB3L3 Entrez,  Source | cAMP responsive element binding protein 3-like 3 | 6006 | 0.073 | 0.0250 | No |
| 23 | CREB3L2 | CREB3L2 Entrez,  Source | cAMP responsive element binding protein 3-like 2 | 6221 | 0.069 | 0.0194 | No |
| 24 | ERBB2 | ERBB2 Entrez,  Source | v-erb-b2 erythroblastic leukemia viral oncogene homolog 2, neuro/glioblastoma derived oncogene homolog (avian) | 6491 | 0.063 | 0.0105 | No |
| 25 | EP300 | EP300 Entrez,  Source | E1A binding protein p300 | 6576 | 0.061 | 0.0110 | No |
| 26 | INS | INS Entrez,  Source | insulin | 6846 | 0.056 | 0.0016 | No |
| 27 | IKBKG | IKBKG Entrez,  Source | inhibitor of kappa light polypeptide gene enhancer in B-cells, kinase gamma | 6863 | 0.056 | 0.0051 | No |
| 28 | PTEN | PTEN Entrez,  Source | phosphatase and tensin homolog (mutated in multiple advanced cancers 1) | 7592 | 0.042 | -0.0290 | No |
| 29 | IGF1R | IGF1R Entrez,  Source | insulin-like growth factor 1 receptor | 7745 | 0.040 | -0.0337 | No |
| 30 | IGF1 | IGF1 Entrez,  Source | insulin-like growth factor 1 (somatomedin C) | 7808 | 0.039 | -0.0338 | No |
| 31 | GSK3B | GSK3B Entrez,  Source | glycogen synthase kinase 3 beta | 8323 | 0.029 | -0.0580 | No |
| 32 | MAP2K1 | MAP2K1 Entrez,  Source | mitogen-activated protein kinase kinase 1 | 8349 | 0.028 | -0.0571 | No |
| 33 | PDGFB | PDGFB Entrez,  Source | platelet-derived growth factor beta polypeptide (simian sarcoma viral (v-sis) oncogene homolog) | 9193 | 0.014 | -0.0993 | No |
| 34 | AKT2 | AKT2 Entrez,  Source | v-akt murine thymoma viral oncogene homolog 2 | 9288 | 0.012 | -0.1031 | No |
| 35 | BAD | BAD Entrez,  Source | BCL2-antagonist of cell death | 9321 | 0.012 | -0.1038 | No |
| 36 | CASP9 | CASP9 Entrez,  Source | caspase 9, apoptosis-related cysteine peptidase | 9688 | 0.006 | -0.1221 | No |
| 37 | SOS1 | SOS1 Entrez,  Source | son of sevenless homolog 1 (Drosophila) | 10121 | -0.001 | -0.1443 | No |
| 38 | CHUK | CHUK Entrez,  Source | conserved helix-loop-helix ubiquitous kinase | 10296 | -0.003 | -0.1530 | No |
| 39 | HRAS | HRAS Entrez,  Source | v-Ha-ras Harvey rat sarcoma viral oncogene homolog | 10338 | -0.004 | -0.1547 | No |
| 40 | FGFR1 | FGFR1 Entrez,  Source | fibroblast growth factor receptor 1 (fms-related tyrosine kinase 2, Pfeiffer syndrome) | 10351 | -0.004 | -0.1550 | No |
| 41 | HSP90B1 | HSP90B1 Entrez,  Source | heat shock protein 90kDa beta (Grp94), member 1 | 10421 | -0.005 | -0.1581 | No |
| 42 | GSTP1 | GSTP1 Entrez,  Source | glutathione S-transferase pi | 10471 | -0.006 | -0.1602 | No |
| 43 | ARAF | ARAF Entrez,  Source | v-raf murine sarcoma 3611 viral oncogene homolog | 10578 | -0.008 | -0.1650 | No |
| 44 | RELA | RELA Entrez,  Source | v-rel reticuloendotheliosis viral oncogene homolog A, nuclear factor of kappa light polypeptide gene enhancer in B-cells 3, p65 (avian) | 10682 | -0.010 | -0.1695 | No |
| 45 | SRD5A2 | SRD5A2 Entrez,  Source | steroid-5-alpha-reductase, alpha polypeptide 2 (3-oxo-5 alpha-steroid delta 4-dehydrogenase alpha 2) | 10798 | -0.012 | -0.1745 | No |
| 46 | PDGFRB | PDGFRB Entrez,  Source | platelet-derived growth factor receptor, beta polypeptide | 10851 | -0.013 | -0.1762 | No |
| 47 | NKX3-1 | NKX3-1 Entrez,  Source | NK3 transcription factor related, locus 1 (Drosophila) | 11085 | -0.017 | -0.1869 | No |
| 48 | BCL2 | BCL2 Entrez,  Source | B-cell CLL/lymphoma 2 | 11142 | -0.018 | -0.1884 | No |
| 49 | GRB2 | GRB2 Entrez,  Source | growth factor receptor-bound protein 2 | 11416 | -0.023 | -0.2006 | No |
| 50 | HSP90AB1 | HSP90AB1 Entrez,  Source | heat shock protein 90kDa alpha (cytosolic), class B member 1 | 11863 | -0.031 | -0.2211 | No |
| 51 | CREBBP | CREBBP Entrez,  Source | CREB binding protein (Rubinstein-Taybi syndrome) | 11958 | -0.032 | -0.2234 | No |
| 52 | BRAF | BRAF Entrez,  Source | v-raf murine sarcoma viral oncogene homolog B1 | 12037 | -0.033 | -0.2248 | No |
| 53 | RB1 | RB1 Entrez,  Source | retinoblastoma 1 (including osteosarcoma) | 12106 | -0.035 | -0.2256 | No |
| 54 | PDGFC | PDGFC Entrez,  Source | platelet derived growth factor C | 12335 | -0.039 | -0.2342 | No |
| 55 | MDM2 | MDM2 Entrez,  Source | Mdm2, transformed 3T3 cell double minute 2, p53 binding protein (mouse) | 12680 | -0.046 | -0.2483 | No |
| 56 | HSP90AA1 | HSP90AA1 Entrez,  Source | heat shock protein 90kDa alpha (cytosolic), class A member 1 | 12953 | -0.051 | -0.2583 | No |
| 57 | MAPK1 | MAPK1 Entrez,  Source | mitogen-activated protein kinase 1 | 13089 | -0.053 | -0.2611 | No |
| 58 | AR | AR Entrez,  Source | androgen receptor (dihydrotestosterone receptor; testicular feminization; spinal and bulbar muscular atrophy; Kennedy disease) | 13091 | -0.053 | -0.2570 | No |
| 59 | PDPK1 | PDPK1 Entrez,  Source | 3-phosphoinositide dependent protein kinase-1 | 13399 | -0.060 | -0.2681 | No |
| 60 | FGFR2 | FGFR2 Entrez,  Source | ffer syndrome, Jackson-Weiss syndrome) | 13470 | -0.061 | -0.2669 | No |
| 61 | RAF1 | RAF1 Entrez,  Source | v-raf-1 murine leukemia viral oncogene homolog 1 | 13610 | -0.064 | -0.2691 | No |
| 62 | CREB5 | CREB5 Entrez,  Source | cAMP responsive element binding protein 5 | 13631 | -0.064 | -0.2651 | No |
| 63 | PIK3R1 | PIK3R1 Entrez,  Source | phosphoinositide-3-kinase, regulatory subunit 1 (p85 alpha) | 13886 | -0.070 | -0.2727 | No |
| 64 | NFKB1 | NFKB1 Entrez,  Source | nuclear factor of kappa light polypeptide gene enhancer in B-cells 1 (p105) | 14629 | -0.088 | -0.3039 | No |
| 65 | SOS2 | SOS2 Entrez,  Source | son of sevenless homolog 2 (Drosophila) | 14817 | -0.092 | -0.3063 | No |
| 66 | ATF4 | ATF4 Entrez,  Source | activating transcription factor 4 (tax-responsive enhancer element B67) | 14909 | -0.095 | -0.3036 | No |
| 67 | PIK3CB | PIK3CB Entrez,  Source | phosphoinositide-3-kinase, catalytic, beta polypeptide | 15135 | -0.101 | -0.3073 | No |
| 68 | CDKN1A | CDKN1A Entrez,  Source | cyclin-dependent kinase inhibitor 1A (p21, Cip1) | 15286 | -0.106 | -0.3067 | No |
| 69 | MAP2K2 | MAP2K2 Entrez,  Source | mitogen-activated protein kinase kinase 2 | 15495 | -0.111 | -0.3087 | No |
| 70 | AKT3 | AKT3 Entrez,  Source | v-akt murine thymoma viral oncogene homolog 3 (protein kinase B, gamma) | 15597 | -0.114 | -0.3050 | No |
| 71 | NFKBIA | NFKBIA Entrez,  Source | nuclear factor of kappa light polypeptide gene enhancer in B-cells inhibitor, alpha | 15953 | -0.125 | -0.3134 | No |
| 72 | PDGFRA | PDGFRA Entrez,  Source | platelet-derived growth factor receptor, alpha polypeptide | 16041 | -0.129 | -0.3078 | No |
| 73 | PDGFA | PDGFA Entrez,  Source | platelet-derived growth factor alpha polypeptide | 16342 | -0.140 | -0.3123 | No |
| 74 | TP53 | TP53 Entrez,  Source | tumor protein p53 (Li-Fraumeni syndrome) | 17097 | -0.175 | -0.3373 | No |
| 75 | INSRR | INSRR Entrez,  Source | insulin receptor-related receptor | 17346 | -0.188 | -0.3354 | No |
| 76 | CCND1 | CCND1 Entrez,  Source | cyclin D1 | 18184 | -0.254 | -0.3585 | Yes |
| 77 | AKT1 | AKT1 Entrez,  Source | v-akt murine thymoma viral oncogene homolog 1 | 18283 | -0.266 | -0.3428 | Yes |
| 78 | KLK3 | KLK3 Entrez,  Source | kallikrein 3, (prostate specific antigen) | 18345 | -0.272 | -0.3247 | Yes |
| 79 | E2F1 | E2F1 Entrez,  Source | E2F transcription factor 1 | 18499 | -0.292 | -0.3097 | Yes |
| 80 | CCNE1 | CCNE1 Entrez,  Source | cyclin E1 | 18741 | -0.330 | -0.2963 | Yes |
| 81 | EGFR | EGFR Entrez,  Source | epidermal growth factor receptor (erythroblastic leukemia viral (v-erb-b) oncogene homolog, avian) | 18782 | -0.337 | -0.2720 | Yes |
| 82 | CDK2 | CDK2 Entrez,  Source | cyclin-dependent kinase 2 | 18981 | -0.390 | -0.2517 | Yes |
| 83 | TCF7 | TCF7 Entrez,  Source | transcription factor 7 (T-cell specific, HMG-box) | 19081 | -0.424 | -0.2237 | Yes |
| 84 | LEF1 | LEF1 Entrez,  Source | lymphoid enhancer-binding factor 1 | 19108 | -0.438 | -0.1908 | Yes |
| 85 | E2F2 | E2F2 Entrez,  Source | E2F transcription factor 2 | 19355 | -0.577 | -0.1583 | Yes |
| 86 | CCNE2 | CCNE2 Entrez,  Source | cyclin E2 | 19501 | -0.835 | -0.1005 | Yes |
| 87 | CTNNB1 | CTNNB1 Entrez,  Source | catenin (cadherin-associated protein), beta 1, 88kDa | 19553 | -1.322 | 0.0002 | Yes |
Table: GSEA details [plain text format]

  

Fig 2: KEGG\_PROSTATE\_CANCER      
 Blue-Pink O' Gram in the Space of the Analyzed GeneSet

  

Fig 3: KEGG\_PROSTATE\_CANCER: Random ES distribution      
 Gene set null distribution of ES for **KEGG\_PROSTATE\_CANCER**
