# Supplementary material for: Comprehensive analysis of β-catenin target genes in colorectal carcinoma cell lines with deregulated Wnt/β-catenin signaling
Source: BMC Genomics. 2014 Jan 28;15:74. doi: 10.1186/1471-2164-15-74 (PMC3909937; doi:10.1186/1471-2164-15-74)
Supplement: Additional file 5 — GSEA analysis using the KEGG pathway database. This zipped file contains confirming data of the GSEA analysis. The names of the directories containing the files were composed of the term ‘GSEA’, the name of the cell line, e.g. DLD1, SW480, or LS174T, and the pathway database (KEGG). Please use a web browser to view the files with the name ‘index.html’ in the corresponding directories to start exploring the data. [file 1471-2164-15-74-S5.zip › GSEA_LS174T_KEGG/KEGG_PURINE_METABOLISM.html]

Details for gene set KEGG\_PURINE\_METABOLISM[GSEA]

|  || Dataset | LS174T\_collapsed\_to\_symbols.class.cls#b\_versus\_bg.class.cls#b\_versus\_bg\_repos |
| Phenotype | class.cls#b\_versus\_bg\_repos |
| Upregulated in class | 0 |
| GeneSet | KEGG\_PURINE\_METABOLISM |
| Enrichment Score (ES) | -0.40019056 |
| Normalized Enrichment Score (NES) | -1.6445954 |
| Nominal p-value | 0.0 |
| FDR q-value | 0.057571016 |
| FWER p-Value | 0.459 |
Table: GSEA Results Summary

  

Fig 1: Enrichment plot: KEGG\_PURINE\_METABOLISM      
 Profile of the Running ES Score & Positions of GeneSet Members on the Rank Ordered List

  

| PROBE | GENE SYMBOL | GENE\_TITLE | RANK IN GENE LIST | RANK METRIC SCORE | RUNNING ES | CORE ENRICHMENT || 1 | GUCY2C | GUCY2C Entrez,  Source | guanylate cyclase 2C (heat stable enterotoxin receptor) | 56 | 1.032 | 0.0409 | No |
| 2 | PDE4D | PDE4D Entrez,  Source | phosphodiesterase 4D, cAMP-specific (phosphodiesterase E3 dunce homolog, Drosophila) | 326 | 0.512 | 0.0488 | No |
| 3 | GDA | GDA Entrez,  Source | guanine deaminase | 649 | 0.375 | 0.0481 | No |
| 4 | POLD4 | POLD4 Entrez,  Source | polymerase (DNA-directed), delta 4 | 753 | 0.350 | 0.0576 | No |
| 5 | ADSSL1 | ADSSL1 Entrez,  Source | adenylosuccinate synthase like 1 | 807 | 0.336 | 0.0692 | No |
| 6 | AMPD3 | AMPD3 Entrez,  Source | adenosine monophosphate deaminase (isoform E) | 976 | 0.307 | 0.0735 | No |
| 7 | PDE6A | PDE6A Entrez,  Source | phosphodiesterase 6A, cGMP-specific, rod, alpha | 1187 | 0.275 | 0.0743 | No |
| 8 | NME5 | NME5 Entrez,  Source | non-metastatic cells 5, protein expressed in (nucleoside-diphosphate kinase) | 1372 | 0.255 | 0.0757 | No |
| 9 | FHIT | FHIT Entrez,  Source | fragile histidine triad gene | 1610 | 0.235 | 0.0734 | No |
| 10 | PDE9A | PDE9A Entrez,  Source | phosphodiesterase 9A | 2152 | 0.197 | 0.0539 | No |
| 11 | GMPR2 | GMPR2 Entrez,  Source | guanosine monophosphate reductase 2 | 2207 | 0.194 | 0.0594 | No |
| 12 | POLE4 | POLE4 Entrez,  Source | polymerase (DNA-directed), epsilon 4 (p12 subunit) | 2276 | 0.190 | 0.0639 | No |
| 13 | PAPSS2 | PAPSS2 Entrez,  Source | 3'-phosphoadenosine 5'-phosphosulfate synthase 2 | 2482 | 0.179 | 0.0609 | No |
| 14 | XDH | XDH Entrez,  Source | xanthine dehydrogenase | 2493 | 0.178 | 0.0680 | No |
| 15 | POLR3D | POLR3D Entrez,  Source | polymerase (RNA) III (DNA directed) polypeptide D, 44kDa | 2762 | 0.166 | 0.0612 | No |
| 16 | ENPP3 | ENPP3 Entrez,  Source | ectonucleotide pyrophosphatase/phosphodiesterase 3 | 2943 | 0.159 | 0.0587 | No |
| 17 | GUCY2F | GUCY2F Entrez,  Source | guanylate cyclase 2F, retinal | 3035 | 0.156 | 0.0606 | No |
| 18 | NT5C2 | NT5C2 Entrez,  Source | 5'-nucleotidase, cytosolic II | 3457 | 0.141 | 0.0449 | No |
| 19 | NT5C3 | NT5C3 Entrez,  Source | 5'-nucleotidase, cytosolic III | 3461 | 0.141 | 0.0507 | No |
| 20 | ENTPD3 | ENTPD3 Entrez,  Source | ectonucleoside triphosphate diphosphohydrolase 3 | 3501 | 0.140 | 0.0546 | No |
| 21 | NUDT9 | NUDT9 Entrez,  Source | nudix (nucleoside diphosphate linked moiety X)-type motif 9 | 3599 | 0.136 | 0.0554 | No |
| 22 | POLR3A | POLR3A Entrez,  Source | polymerase (RNA) III (DNA directed) polypeptide A, 155kDa | 3971 | 0.124 | 0.0416 | No |
| 23 | ADCY8 | ADCY8 Entrez,  Source | adenylate cyclase 8 (brain) | 4003 | 0.123 | 0.0452 | No |
| 24 | ADCY9 | ADCY9 Entrez,  Source | adenylate cyclase 9 | 4106 | 0.120 | 0.0450 | No |
| 25 | ADCY1 | ADCY1 Entrez,  Source | adenylate cyclase 1 (brain) | 4182 | 0.118 | 0.0462 | No |
| 26 | GUCY1A3 | GUCY1A3 Entrez,  Source | guanylate cyclase 1, soluble, alpha 3 | 4204 | 0.117 | 0.0500 | No |
| 27 | PDE1C | PDE1C Entrez,  Source | phosphodiesterase 1C, calmodulin-dependent 70kDa | 4273 | 0.115 | 0.0514 | No |
| 28 | NT5E | NT5E Entrez,  Source | 5'-nucleotidase, ecto (CD73) | 4445 | 0.110 | 0.0473 | No |
| 29 | AMPD1 | AMPD1 Entrez,  Source | adenosine monophosphate deaminase 1 (isoform M) | 4676 | 0.104 | 0.0398 | No |
| 30 | ADCY7 | ADCY7 Entrez,  Source | adenylate cyclase 7 | 4771 | 0.102 | 0.0393 | No |
| 31 | PDE10A | PDE10A Entrez,  Source | phosphodiesterase 10A | 4950 | 0.097 | 0.0342 | No |
| 32 | PDE5A | PDE5A Entrez,  Source | phosphodiesterase 5A, cGMP-specific | 6009 | 0.073 | -0.0172 | No |
| 33 | NT5C1A | NT5C1A Entrez,  Source | 5'-nucleotidase, cytosolic IA | 6198 | 0.069 | -0.0239 | No |
| 34 | ADCY4 | ADCY4 Entrez,  Source | adenylate cyclase 4 | 6242 | 0.068 | -0.0233 | No |
| 35 | ADK | ADK Entrez,  Source | adenosine kinase | 6348 | 0.066 | -0.0259 | No |
| 36 | GUCY2D | GUCY2D Entrez,  Source | guanylate cyclase 2D, membrane (retina-specific) | 6364 | 0.066 | -0.0239 | No |
| 37 | PDE3A | PDE3A Entrez,  Source | phosphodiesterase 3A, cGMP-inhibited | 6482 | 0.063 | -0.0272 | No |
| 38 | PDE4B | PDE4B Entrez,  Source | phosphodiesterase 4B, cAMP-specific (phosphodiesterase E4 dunce homolog, Drosophila) | 6584 | 0.061 | -0.0298 | No |
| 39 | NME7 | NME7 Entrez,  Source | non-metastatic cells 7, protein expressed in (nucleoside-diphosphate kinase) | 6610 | 0.061 | -0.0285 | No |
| 40 | PDE7B | PDE7B Entrez,  Source | phosphodiesterase 7B | 6670 | 0.060 | -0.0291 | No |
| 41 | NME3 | NME3 Entrez,  Source | non-metastatic cells 3, protein expressed in | 6686 | 0.059 | -0.0273 | No |
| 42 | PDE6G | PDE6G Entrez,  Source | phosphodiesterase 6G, cGMP-specific, rod, gamma | 6719 | 0.059 | -0.0265 | No |
| 43 | PKLR | PKLR Entrez,  Source | pyruvate kinase, liver and RBC | 6745 | 0.058 | -0.0253 | No |
| 44 | ADSS | ADSS Entrez,  Source | adenylosuccinate synthase | 6834 | 0.057 | -0.0274 | No |
| 45 | ITPA | ITPA Entrez,  Source | inosine triphosphatase (nucleoside triphosphate pyrophosphatase) | 7246 | 0.049 | -0.0465 | No |
| 46 | NT5C1B | NT5C1B Entrez,  Source | 5'-nucleotidase, cytosolic IB | 7287 | 0.048 | -0.0465 | No |
| 47 | AK1 | AK1 Entrez,  Source | adenylate kinase 1 | 7597 | 0.042 | -0.0607 | No |
| 48 | POLR2L | POLR2L Entrez,  Source | polymerase (RNA) II (DNA directed) polypeptide L, 7.6kDa | 7901 | 0.037 | -0.0747 | No |
| 49 | ADCY5 | ADCY5 Entrez,  Source | adenylate cyclase 5 | 8116 | 0.032 | -0.0844 | No |
| 50 | PAPSS1 | PAPSS1 Entrez,  Source | 3'-phosphoadenosine 5'-phosphosulfate synthase 1 | 8254 | 0.030 | -0.0902 | No |
| 51 | AK7 | AK7 Entrez,  Source | adenylate kinase 7 | 8258 | 0.030 | -0.0891 | No |
| 52 | PDE6H | PDE6H Entrez,  Source | phosphodiesterase 6H, cGMP-specific, cone, gamma | 8277 | 0.029 | -0.0887 | No |
| 53 | ADSL | ADSL Entrez,  Source | adenylosuccinate lyase | 8394 | 0.028 | -0.0936 | No |
| 54 | APRT | APRT Entrez,  Source | adenine phosphoribosyltransferase | 8458 | 0.026 | -0.0957 | No |
| 55 | GUK1 | GUK1 Entrez,  Source | guanylate kinase 1 | 8680 | 0.022 | -0.1061 | No |
| 56 | RRM2B | RRM2B Entrez,  Source | ribonucleotide reductase M2 B (TP53 inducible) | 8999 | 0.017 | -0.1218 | No |
| 57 | POLR1D | POLR1D Entrez,  Source | polymerase (RNA) I polypeptide D, 16kDa | 9198 | 0.014 | -0.1314 | No |
| 58 | ENTPD1 | ENTPD1 Entrez,  Source | ectonucleoside triphosphate diphosphohydrolase 1 | 9273 | 0.013 | -0.1347 | No |
| 59 | PDE11A | PDE11A Entrez,  Source | phosphodiesterase 11A | 9656 | 0.007 | -0.1541 | No |
| 60 | IMPDH2 | IMPDH2 Entrez,  Source | IMP (inosine monophosphate) dehydrogenase 2 | 9774 | 0.005 | -0.1599 | No |
| 61 | CANT1 | CANT1 Entrez,  Source | calcium activated nucleotidase 1 | 10353 | -0.004 | -0.1895 | No |
| 62 | POLR2J2 | POLR2J2 Entrez,  Source | - | 10373 | -0.005 | -0.1903 | No |
| 63 | POLR2J | POLR2J Entrez,  Source | polymerase (RNA) II (DNA directed) polypeptide J, 13.3kDa | 10628 | -0.009 | -0.2030 | No |
| 64 | ALLC | ALLC Entrez,  Source | allantoicase | 10672 | -0.010 | -0.2048 | No |
| 65 | DGUOK | DGUOK Entrez,  Source | deoxyguanosine kinase | 10731 | -0.010 | -0.2073 | No |
| 66 | PDE6B | PDE6B Entrez,  Source | phosphodiesterase 6B, cGMP-specific, rod, beta (congenital stationary night blindness 3, autosomal dominant) | 10770 | -0.011 | -0.2088 | No |
| 67 | ENTPD6 | ENTPD6 Entrez,  Source | ectonucleoside triphosphate diphosphohydrolase 6 (putative function) | 10904 | -0.013 | -0.2151 | No |
| 68 | AK5 | AK5 Entrez,  Source | adenylate kinase 5 | 11082 | -0.017 | -0.2235 | No |
| 69 | POLR2B | POLR2B Entrez,  Source | polymerase (RNA) II (DNA directed) polypeptide B, 140kDa | 11138 | -0.018 | -0.2256 | No |
| 70 | PDE1B | PDE1B Entrez,  Source | phosphodiesterase 1B, calmodulin-dependent | 11157 | -0.018 | -0.2258 | No |
| 71 | POLR3B | POLR3B Entrez,  Source | polymerase (RNA) III (DNA directed) polypeptide B | 11250 | -0.020 | -0.2297 | No |
| 72 | PKM2 | PKM2 Entrez,  Source | pyruvate kinase, muscle | 11256 | -0.020 | -0.2291 | No |
| 73 | NPR1 | NPR1 Entrez,  Source | natriuretic peptide receptor A/guanylate cyclase A (atrionatriuretic peptide receptor A) | 11617 | -0.027 | -0.2465 | No |
| 74 | ADA | ADA Entrez,  Source | adenosine deaminase | 11855 | -0.031 | -0.2574 | No |
| 75 | NME1 | NME1 Entrez,  Source | non-metastatic cells 1, protein (NM23A) expressed in | 11908 | -0.031 | -0.2587 | No |
| 76 | ENTPD2 | ENTPD2 Entrez,  Source | ectonucleoside triphosphate diphosphohydrolase 2 | 11944 | -0.032 | -0.2592 | No |
| 77 | NT5C | NT5C Entrez,  Source | 5', 3'-nucleotidase, cytosolic | 11959 | -0.032 | -0.2586 | No |
| 78 | ADCY6 | ADCY6 Entrez,  Source | adenylate cyclase 6 | 12006 | -0.033 | -0.2595 | No |
| 79 | PDE4C | PDE4C Entrez,  Source | phosphodiesterase 4C, cAMP-specific (phosphodiesterase E1 dunce homolog, Drosophila) | 12014 | -0.033 | -0.2585 | No |
| 80 | POLR3F | POLR3F Entrez,  Source | polymerase (RNA) III (DNA directed) polypeptide F, 39 kDa | 12059 | -0.034 | -0.2593 | No |
| 81 | GMPR | GMPR Entrez,  Source | guanosine monophosphate reductase | 12321 | -0.039 | -0.2711 | No |
| 82 | NUDT5 | NUDT5 Entrez,  Source | nudix (nucleoside diphosphate linked moiety X)-type motif 5 | 12427 | -0.041 | -0.2748 | No |
| 83 | POLR3H | POLR3H Entrez,  Source | polymerase (RNA) III (DNA directed) polypeptide H (22.9kD) | 12523 | -0.043 | -0.2779 | No |
| 84 | POLR2I | POLR2I Entrez,  Source | polymerase (RNA) II (DNA directed) polypeptide I, 14.5kDa | 12535 | -0.043 | -0.2766 | No |
| 85 | ADCY2 | ADCY2 Entrez,  Source | adenylate cyclase 2 (brain) | 12538 | -0.043 | -0.2749 | No |
| 86 | HPRT1 | HPRT1 Entrez,  Source | hypoxanthine phosphoribosyltransferase 1 (Lesch-Nyhan syndrome) | 12713 | -0.046 | -0.2819 | No |
| 87 | ATIC | ATIC Entrez,  Source | 5-aminoimidazole-4-carboxamide ribonucleotide formyltransferase/IMP cyclohydrolase | 13018 | -0.052 | -0.2953 | No |
| 88 | PDE1A | PDE1A Entrez,  Source | phosphodiesterase 1A, calmodulin-dependent | 13042 | -0.052 | -0.2943 | No |
| 89 | POLR2K | POLR2K Entrez,  Source | polymerase (RNA) II (DNA directed) polypeptide K, 7.0kDa | 13090 | -0.053 | -0.2945 | No |
| 90 | POLR1C | POLR1C Entrez,  Source | polymerase (RNA) I polypeptide C, 30kDa | 13175 | -0.055 | -0.2965 | No |
| 91 | PNPT1 | PNPT1 Entrez,  Source | polyribonucleotide nucleotidyltransferase 1 | 13203 | -0.055 | -0.2955 | No |
| 92 | POLR2C | POLR2C Entrez,  Source | polymerase (RNA) II (DNA directed) polypeptide C, 33kDa | 13358 | -0.059 | -0.3010 | No |
| 93 | PDE3B | PDE3B Entrez,  Source | phosphodiesterase 3B, cGMP-inhibited | 13564 | -0.063 | -0.3088 | No |
| 94 | ENTPD4 | ENTPD4 Entrez,  Source | ectonucleoside triphosphate diphosphohydrolase 4 | 13651 | -0.065 | -0.3105 | No |
| 95 | PRPS1L1 | PRPS1L1 Entrez,  Source | phosphoribosyl pyrophosphate synthetase 1-like 1 | 14028 | -0.073 | -0.3268 | No |
| 96 | POLR2E | POLR2E Entrez,  Source | polymerase (RNA) II (DNA directed) polypeptide E, 25kDa | 14089 | -0.075 | -0.3267 | No |
| 97 | POLR2H | POLR2H Entrez,  Source | polymerase (RNA) II (DNA directed) polypeptide H | 14235 | -0.078 | -0.3309 | No |
| 98 | ENTPD5 | ENTPD5 Entrez,  Source | ectonucleoside triphosphate diphosphohydrolase 5 | 14241 | -0.078 | -0.3279 | No |
| 99 | POLR2F | POLR2F Entrez,  Source | polymerase (RNA) II (DNA directed) polypeptide F | 14269 | -0.078 | -0.3259 | No |
| 100 | IMPDH1 | IMPDH1 Entrez,  Source | IMP (inosine monophosphate) dehydrogenase 1 | 14405 | -0.082 | -0.3294 | No |
| 101 | PAICS | PAICS Entrez,  Source | phosphoribosylaminoimidazole carboxylase, phosphoribosylaminoimidazole succinocarboxamide synthetase | 14787 | -0.092 | -0.3451 | No |
| 102 | PPAT | PPAT Entrez,  Source | phosphoribosyl pyrophosphate amidotransferase | 14814 | -0.092 | -0.3426 | No |
| 103 | POLR3C | POLR3C Entrez,  Source | polymerase (RNA) III (DNA directed) polypeptide C (62kD) | 15557 | -0.113 | -0.3760 | No |
| 104 | PDE4A | PDE4A Entrez,  Source | phosphodiesterase 4A, cAMP-specific (phosphodiesterase E2 dunce homolog, Drosophila) | 15824 | -0.121 | -0.3846 | No |
| 105 | POLR2A | POLR2A Entrez,  Source | polymerase (RNA) II (DNA directed) polypeptide A, 220kDa | 15930 | -0.124 | -0.3847 | No |
| 106 | PDE6C | PDE6C Entrez,  Source | phosphodiesterase 6C, cGMP-specific, cone, alpha prime | 15948 | -0.125 | -0.3803 | No |
| 107 | PDE8A | PDE8A Entrez,  Source | phosphodiesterase 8A | 16126 | -0.132 | -0.3838 | No |
| 108 | POLR1E | POLR1E Entrez,  Source | polymerase (RNA) I polypeptide E, 53kDa | 16145 | -0.133 | -0.3791 | No |
| 109 | NUDT2 | NUDT2 Entrez,  Source | nudix (nucleoside diphosphate linked moiety X)-type motif 2 | 16556 | -0.149 | -0.3939 | Yes |
| 110 | ZNRD1 | ZNRD1 Entrez,  Source | zinc ribbon domain containing 1 | 16598 | -0.150 | -0.3896 | Yes |
| 111 | POLR1B | POLR1B Entrez,  Source | polymerase (RNA) I polypeptide B, 128kDa | 16699 | -0.155 | -0.3882 | Yes |
| 112 | POLR3G | POLR3G Entrez,  Source | polymerase (RNA) III (DNA directed) polypeptide G (32kD) | 16729 | -0.157 | -0.3830 | Yes |
| 113 | NME4 | NME4 Entrez,  Source | non-metastatic cells 4, protein expressed in | 16749 | -0.158 | -0.3773 | Yes |
| 114 | POLR3GL | POLR3GL Entrez,  Source | polymerase (RNA) III (DNA directed) polypeptide G (32kD) like | 16887 | -0.164 | -0.3774 | Yes |
| 115 | POLR2G | POLR2G Entrez,  Source | polymerase (RNA) II (DNA directed) polypeptide G | 16910 | -0.165 | -0.3716 | Yes |
| 116 | AMPD2 | AMPD2 Entrez,  Source | adenosine monophosphate deaminase 2 (isoform L) | 16953 | -0.167 | -0.3667 | Yes |
| 117 | NME6 | NME6 Entrez,  Source | non-metastatic cells 6, protein expressed in (nucleoside-diphosphate kinase) | 17122 | -0.176 | -0.3679 | Yes |
| 118 | GUCY1B3 | GUCY1B3 Entrez,  Source | guanylate cyclase 1, soluble, beta 3 | 17261 | -0.183 | -0.3672 | Yes |
| 119 | GUCY1A2 | GUCY1A2 Entrez,  Source | guanylate cyclase 1, soluble, alpha 2 | 17265 | -0.183 | -0.3596 | Yes |
| 120 | AK2 | AK2 Entrez,  Source | adenylate kinase 2 | 17336 | -0.188 | -0.3552 | Yes |
| 121 | PRPS1 | PRPS1 Entrez,  Source | phosphoribosyl pyrophosphate synthetase 1 | 17364 | -0.190 | -0.3486 | Yes |
| 122 | POLR3K | POLR3K Entrez,  Source | polymerase (RNA) III (DNA directed) polypeptide K, 12.3 kDa | 17437 | -0.194 | -0.3440 | Yes |
| 123 | PDE2A | PDE2A Entrez,  Source | phosphodiesterase 2A, cGMP-stimulated | 17458 | -0.196 | -0.3368 | Yes |
| 124 | PRUNE | PRUNE Entrez,  Source | prune homolog (Drosophila) | 17502 | -0.199 | -0.3306 | Yes |
| 125 | PFAS | PFAS Entrez,  Source | phosphoribosylformylglycinamidine synthase (FGAR amidotransferase) | 17719 | -0.213 | -0.3326 | Yes |
| 126 | DCK | DCK Entrez,  Source | deoxycytidine kinase | 17890 | -0.226 | -0.3318 | Yes |
| 127 | POLD2 | POLD2 Entrez,  Source | polymerase (DNA directed), delta 2, regulatory subunit 50kDa | 17923 | -0.230 | -0.3237 | Yes |
| 128 | NT5M | NT5M Entrez,  Source | 5',3'-nucleotidase, mitochondrial | 17968 | -0.233 | -0.3161 | Yes |
| 129 | PDE6D | PDE6D Entrez,  Source | phosphodiesterase 6D, cGMP-specific, rod, delta | 17979 | -0.234 | -0.3067 | Yes |
| 130 | PRPS2 | PRPS2 Entrez,  Source | phosphoribosyl pyrophosphate synthetase 2 | 17983 | -0.235 | -0.2968 | Yes |
| 131 | GMPS | GMPS Entrez,  Source | guanine monphosphate synthetase | 18003 | -0.236 | -0.2878 | Yes |
| 132 | ADCY3 | ADCY3 Entrez,  Source | adenylate cyclase 3 | 18015 | -0.238 | -0.2783 | Yes |
| 133 | ENPP1 | ENPP1 Entrez,  Source | ectonucleotide pyrophosphatase/phosphodiesterase 1 | 18181 | -0.254 | -0.2760 | Yes |
| 134 | NPR2 | NPR2 Entrez,  Source | natriuretic peptide receptor B/guanylate cyclase B (atrionatriuretic peptide receptor B) | 18183 | -0.254 | -0.2653 | Yes |
| 135 | GART | GART Entrez,  Source | phosphoribosylglycinamide formyltransferase, phosphoribosylglycinamide synthetase, phosphoribosylaminoimidazole synthetase | 18257 | -0.262 | -0.2579 | Yes |
| 136 | POLR1A | POLR1A Entrez,  Source | polymerase (RNA) I polypeptide A, 194kDa | 18602 | -0.307 | -0.2627 | Yes |
| 137 | RRM2 | RRM2 Entrez,  Source | ribonucleotide reductase M2 polypeptide | 18610 | -0.308 | -0.2499 | Yes |
| 138 | POLD1 | POLD1 Entrez,  Source | polymerase (DNA directed), delta 1, catalytic subunit 125kDa | 18666 | -0.318 | -0.2393 | Yes |
| 139 | RRM1 | RRM1 Entrez,  Source | ribonucleotide reductase M1 polypeptide | 18889 | -0.364 | -0.2353 | Yes |
| 140 | POLE3 | POLE3 Entrez,  Source | polymerase (DNA directed), epsilon 3 (p17 subunit) | 18960 | -0.385 | -0.2226 | Yes |
| 141 | POLR2D | POLR2D Entrez,  Source | polymerase (RNA) II (DNA directed) polypeptide D | 19126 | -0.444 | -0.2122 | Yes |
| 142 | PRIM1 | PRIM1 Entrez,  Source | primase, polypeptide 1, 49kDa | 19268 | -0.519 | -0.1975 | Yes |
| 143 | POLE | POLE Entrez,  Source | polymerase (DNA directed), epsilon | 19281 | -0.529 | -0.1756 | Yes |
| 144 | POLA2 | POLA2 Entrez,  Source | polymerase (DNA directed), alpha 2 (70kD subunit) | 19321 | -0.553 | -0.1542 | Yes |
| 145 | PDE7A | PDE7A Entrez,  Source | phosphodiesterase 7A | 19418 | -0.657 | -0.1312 | Yes |
| 146 | PDE8B | PDE8B Entrez,  Source | phosphodiesterase 8B | 19469 | -0.747 | -0.1021 | Yes |
| 147 | POLA1 | POLA1 Entrez,  Source | polymerase (DNA directed), alpha 1 | 19484 | -0.786 | -0.0695 | Yes |
| 148 | POLD3 | POLD3 Entrez,  Source | polymerase (DNA-directed), delta 3, accessory subunit | 19495 | -0.805 | -0.0359 | Yes |
| 149 | POLE2 | POLE2 Entrez,  Source | polymerase (DNA directed), epsilon 2 (p59 subunit) | 19520 | -0.918 | 0.0019 | Yes |
Table: GSEA details [plain text format]

  

Fig 2: KEGG\_PURINE\_METABOLISM      
 Blue-Pink O' Gram in the Space of the Analyzed GeneSet

  

Fig 3: KEGG\_PURINE\_METABOLISM: Random ES distribution      
 Gene set null distribution of ES for **KEGG\_PURINE\_METABOLISM**
