# Supplementary material for: Comprehensive analysis of β-catenin target genes in colorectal carcinoma cell lines with deregulated Wnt/β-catenin signaling
Source: BMC Genomics. 2014 Jan 28;15:74. doi: 10.1186/1471-2164-15-74 (PMC3909937; doi:10.1186/1471-2164-15-74)
Supplement: Additional file 5 — GSEA analysis using the KEGG pathway database. This zipped file contains confirming data of the GSEA analysis. The names of the directories containing the files were composed of the term ‘GSEA’, the name of the cell line, e.g. DLD1, SW480, or LS174T, and the pathway database (KEGG). Please use a web browser to view the files with the name ‘index.html’ in the corresponding directories to start exploring the data. [file 1471-2164-15-74-S5.zip › GSEA_LS174T_KEGG/KEGG_PYRIMIDINE_METABOLISM.html]

Details for gene set KEGG\_PYRIMIDINE\_METABOLISM[GSEA]

|  || Dataset | LS174T\_collapsed\_to\_symbols.class.cls#b\_versus\_bg.class.cls#b\_versus\_bg\_repos |
| Phenotype | class.cls#b\_versus\_bg\_repos |
| Upregulated in class | 0 |
| GeneSet | KEGG\_PYRIMIDINE\_METABOLISM |
| Enrichment Score (ES) | -0.5381974 |
| Normalized Enrichment Score (NES) | -2.0009558 |
| Nominal p-value | 0.0 |
| FDR q-value | 0.001341402 |
| FWER p-Value | 0.0090 |
Table: GSEA Results Summary

  

Fig 1: Enrichment plot: KEGG\_PYRIMIDINE\_METABOLISM      
 Profile of the Running ES Score & Positions of GeneSet Members on the Rank Ordered List

  

| PROBE | GENE SYMBOL | GENE\_TITLE | RANK IN GENE LIST | RANK METRIC SCORE | RUNNING ES | CORE ENRICHMENT || 1 | CDA | CDA Entrez,  Source | cytidine deaminase | 97 | 0.874 | 0.0486 | No |
| 2 | POLD4 | POLD4 Entrez,  Source | polymerase (DNA-directed), delta 4 | 753 | 0.350 | 0.0365 | No |
| 3 | DTYMK | DTYMK Entrez,  Source | deoxythymidylate kinase (thymidylate kinase) | 1266 | 0.266 | 0.0265 | No |
| 4 | NME5 | NME5 Entrez,  Source | non-metastatic cells 5, protein expressed in (nucleoside-diphosphate kinase) | 1372 | 0.255 | 0.0368 | No |
| 5 | POLE4 | POLE4 Entrez,  Source | polymerase (DNA-directed), epsilon 4 (p12 subunit) | 2276 | 0.190 | 0.0021 | No |
| 6 | POLR3D | POLR3D Entrez,  Source | polymerase (RNA) III (DNA directed) polypeptide D, 44kDa | 2762 | 0.166 | -0.0126 | No |
| 7 | NT5C2 | NT5C2 Entrez,  Source | 5'-nucleotidase, cytosolic II | 3457 | 0.141 | -0.0396 | No |
| 8 | NT5C3 | NT5C3 Entrez,  Source | 5'-nucleotidase, cytosolic III | 3461 | 0.141 | -0.0311 | No |
| 9 | ENTPD3 | ENTPD3 Entrez,  Source | ectonucleoside triphosphate diphosphohydrolase 3 | 3501 | 0.140 | -0.0246 | No |
| 10 | POLR3A | POLR3A Entrez,  Source | polymerase (RNA) III (DNA directed) polypeptide A, 155kDa | 3971 | 0.124 | -0.0410 | No |
| 11 | NT5E | NT5E Entrez,  Source | 5'-nucleotidase, ecto (CD73) | 4445 | 0.110 | -0.0586 | No |
| 12 | TXNRD2 | TXNRD2 Entrez,  Source | thioredoxin reductase 2 | 5308 | 0.088 | -0.0974 | No |
| 13 | NT5C1A | NT5C1A Entrez,  Source | 5'-nucleotidase, cytosolic IA | 6198 | 0.069 | -0.1389 | No |
| 14 | UCKL1 | UCKL1 Entrez,  Source | uridine-cytidine kinase 1-like 1 | 6543 | 0.062 | -0.1527 | No |
| 15 | TXNRD1 | TXNRD1 Entrez,  Source | thioredoxin reductase 1 | 6583 | 0.061 | -0.1510 | No |
| 16 | NME7 | NME7 Entrez,  Source | non-metastatic cells 7, protein expressed in (nucleoside-diphosphate kinase) | 6610 | 0.061 | -0.1486 | No |
| 17 | NME3 | NME3 Entrez,  Source | non-metastatic cells 3, protein expressed in | 6686 | 0.059 | -0.1488 | No |
| 18 | ITPA | ITPA Entrez,  Source | inosine triphosphatase (nucleoside triphosphate pyrophosphatase) | 7246 | 0.049 | -0.1745 | No |
| 19 | NT5C1B | NT5C1B Entrez,  Source | 5'-nucleotidase, cytosolic IB | 7287 | 0.048 | -0.1736 | No |
| 20 | UPP1 | UPP1 Entrez,  Source | uridine phosphorylase 1 | 7418 | 0.046 | -0.1774 | No |
| 21 | POLR2L | POLR2L Entrez,  Source | polymerase (RNA) II (DNA directed) polypeptide L, 7.6kDa | 7901 | 0.037 | -0.2000 | No |
| 22 | RRM2B | RRM2B Entrez,  Source | ribonucleotide reductase M2 B (TP53 inducible) | 8999 | 0.017 | -0.2552 | No |
| 23 | POLR1D | POLR1D Entrez,  Source | polymerase (RNA) I polypeptide D, 16kDa | 9198 | 0.014 | -0.2645 | No |
| 24 | ENTPD1 | ENTPD1 Entrez,  Source | ectonucleoside triphosphate diphosphohydrolase 1 | 9273 | 0.013 | -0.2676 | No |
| 25 | AK3 | AK3 Entrez,  Source | adenylate kinase 3 | 9377 | 0.011 | -0.2722 | No |
| 26 | DPYD | DPYD Entrez,  Source | dihydropyrimidine dehydrogenase | 9709 | 0.006 | -0.2888 | No |
| 27 | CANT1 | CANT1 Entrez,  Source | calcium activated nucleotidase 1 | 10353 | -0.004 | -0.3216 | No |
| 28 | POLR2J2 | POLR2J2 Entrez,  Source | - | 10373 | -0.005 | -0.3223 | No |
| 29 | POLR2J | POLR2J Entrez,  Source | polymerase (RNA) II (DNA directed) polypeptide J, 13.3kDa | 10628 | -0.009 | -0.3348 | No |
| 30 | ENTPD6 | ENTPD6 Entrez,  Source | ectonucleoside triphosphate diphosphohydrolase 6 (putative function) | 10904 | -0.013 | -0.3481 | No |
| 31 | POLR2B | POLR2B Entrez,  Source | polymerase (RNA) II (DNA directed) polypeptide B, 140kDa | 11138 | -0.018 | -0.3590 | No |
| 32 | POLR3B | POLR3B Entrez,  Source | polymerase (RNA) III (DNA directed) polypeptide B | 11250 | -0.020 | -0.3634 | No |
| 33 | NME1 | NME1 Entrez,  Source | non-metastatic cells 1, protein (NM23A) expressed in | 11908 | -0.031 | -0.3953 | No |
| 34 | NT5C | NT5C Entrez,  Source | 5', 3'-nucleotidase, cytosolic | 11959 | -0.032 | -0.3958 | No |
| 35 | POLR3F | POLR3F Entrez,  Source | polymerase (RNA) III (DNA directed) polypeptide F, 39 kDa | 12059 | -0.034 | -0.3989 | No |
| 36 | POLR3H | POLR3H Entrez,  Source | polymerase (RNA) III (DNA directed) polypeptide H (22.9kD) | 12523 | -0.043 | -0.4200 | No |
| 37 | POLR2I | POLR2I Entrez,  Source | polymerase (RNA) II (DNA directed) polypeptide I, 14.5kDa | 12535 | -0.043 | -0.4179 | No |
| 38 | UPB1 | UPB1 Entrez,  Source | ureidopropionase, beta | 12891 | -0.049 | -0.4331 | No |
| 39 | POLR2K | POLR2K Entrez,  Source | polymerase (RNA) II (DNA directed) polypeptide K, 7.0kDa | 13090 | -0.053 | -0.4400 | No |
| 40 | POLR1C | POLR1C Entrez,  Source | polymerase (RNA) I polypeptide C, 30kDa | 13175 | -0.055 | -0.4410 | No |
| 41 | PNPT1 | PNPT1 Entrez,  Source | polyribonucleotide nucleotidyltransferase 1 | 13203 | -0.055 | -0.4390 | No |
| 42 | POLR2C | POLR2C Entrez,  Source | polymerase (RNA) II (DNA directed) polypeptide C, 33kDa | 13358 | -0.059 | -0.4433 | No |
| 43 | ENTPD4 | ENTPD4 Entrez,  Source | ectonucleoside triphosphate diphosphohydrolase 4 | 13651 | -0.065 | -0.4543 | No |
| 44 | CAD | CAD Entrez,  Source | carbamoyl-phosphate synthetase 2, aspartate transcarbamylase, and dihydroorotase | 13858 | -0.070 | -0.4606 | No |
| 45 | POLR2E | POLR2E Entrez,  Source | polymerase (RNA) II (DNA directed) polypeptide E, 25kDa | 14089 | -0.075 | -0.4678 | No |
| 46 | POLR2H | POLR2H Entrez,  Source | polymerase (RNA) II (DNA directed) polypeptide H | 14235 | -0.078 | -0.4705 | No |
| 47 | ENTPD5 | ENTPD5 Entrez,  Source | ectonucleoside triphosphate diphosphohydrolase 5 | 14241 | -0.078 | -0.4660 | No |
| 48 | POLR2F | POLR2F Entrez,  Source | polymerase (RNA) II (DNA directed) polypeptide F | 14269 | -0.078 | -0.4626 | No |
| 49 | TK2 | TK2 Entrez,  Source | thymidine kinase 2, mitochondrial | 14498 | -0.084 | -0.4691 | No |
| 50 | DCTD | DCTD Entrez,  Source | dCMP deaminase | 15157 | -0.101 | -0.4967 | No |
| 51 | POLR3C | POLR3C Entrez,  Source | polymerase (RNA) III (DNA directed) polypeptide C (62kD) | 15557 | -0.113 | -0.5102 | No |
| 52 | POLR2A | POLR2A Entrez,  Source | polymerase (RNA) II (DNA directed) polypeptide A, 220kDa | 15930 | -0.124 | -0.5217 | No |
| 53 | POLR1E | POLR1E Entrez,  Source | polymerase (RNA) I polypeptide E, 53kDa | 16145 | -0.133 | -0.5245 | No |
| 54 | UPP2 | UPP2 Entrez,  Source | uridine phosphorylase 2 | 16412 | -0.143 | -0.5294 | Yes |
| 55 | NUDT2 | NUDT2 Entrez,  Source | nudix (nucleoside diphosphate linked moiety X)-type motif 2 | 16556 | -0.149 | -0.5277 | Yes |
| 56 | ZNRD1 | ZNRD1 Entrez,  Source | zinc ribbon domain containing 1 | 16598 | -0.150 | -0.5205 | Yes |
| 57 | POLR1B | POLR1B Entrez,  Source | polymerase (RNA) I polypeptide B, 128kDa | 16699 | -0.155 | -0.5161 | Yes |
| 58 | POLR3G | POLR3G Entrez,  Source | polymerase (RNA) III (DNA directed) polypeptide G (32kD) | 16729 | -0.157 | -0.5080 | Yes |
| 59 | NME4 | NME4 Entrez,  Source | non-metastatic cells 4, protein expressed in | 16749 | -0.158 | -0.4993 | Yes |
| 60 | POLR3GL | POLR3GL Entrez,  Source | polymerase (RNA) III (DNA directed) polypeptide G (32kD) like | 16887 | -0.164 | -0.4963 | Yes |
| 61 | POLR2G | POLR2G Entrez,  Source | polymerase (RNA) II (DNA directed) polypeptide G | 16910 | -0.165 | -0.4873 | Yes |
| 62 | UCK2 | UCK2 Entrez,  Source | uridine-cytidine kinase 2 | 16950 | -0.167 | -0.4791 | Yes |
| 63 | NME6 | NME6 Entrez,  Source | non-metastatic cells 6, protein expressed in (nucleoside-diphosphate kinase) | 17122 | -0.176 | -0.4771 | Yes |
| 64 | DPYS | DPYS Entrez,  Source | dihydropyrimidinase | 17252 | -0.183 | -0.4725 | Yes |
| 65 | POLR3K | POLR3K Entrez,  Source | polymerase (RNA) III (DNA directed) polypeptide K, 12.3 kDa | 17437 | -0.194 | -0.4700 | Yes |
| 66 | CTPS2 | CTPS2 Entrez,  Source | CTP synthase II | 17797 | -0.218 | -0.4751 | Yes |
| 67 | DCK | DCK Entrez,  Source | deoxycytidine kinase | 17890 | -0.226 | -0.4659 | Yes |
| 68 | UCK1 | UCK1 Entrez,  Source | uridine-cytidine kinase 1 | 17906 | -0.228 | -0.4527 | Yes |
| 69 | POLD2 | POLD2 Entrez,  Source | polymerase (DNA directed), delta 2, regulatory subunit 50kDa | 17923 | -0.230 | -0.4395 | Yes |
| 70 | NT5M | NT5M Entrez,  Source | 5',3'-nucleotidase, mitochondrial | 17968 | -0.233 | -0.4274 | Yes |
| 71 | UMPS | UMPS Entrez,  Source | uridine monophosphate synthetase (orotate phosphoribosyl transferase and orotidine-5'-decarboxylase) | 18198 | -0.256 | -0.4235 | Yes |
| 72 | DHODH | DHODH Entrez,  Source | dihydroorotate dehydrogenase | 18208 | -0.257 | -0.4081 | Yes |
| 73 | POLR1A | POLR1A Entrez,  Source | polymerase (RNA) I polypeptide A, 194kDa | 18602 | -0.307 | -0.4095 | Yes |
| 74 | RRM2 | RRM2 Entrez,  Source | ribonucleotide reductase M2 polypeptide | 18610 | -0.308 | -0.3909 | Yes |
| 75 | POLD1 | POLD1 Entrez,  Source | polymerase (DNA directed), delta 1, catalytic subunit 125kDa | 18666 | -0.318 | -0.3742 | Yes |
| 76 | CTPS | CTPS Entrez,  Source | CTP synthase | 18671 | -0.319 | -0.3548 | Yes |
| 77 | DUT | DUT Entrez,  Source | dUTP pyrophosphatase | 18819 | -0.346 | -0.3411 | Yes |
| 78 | RRM1 | RRM1 Entrez,  Source | ribonucleotide reductase M1 polypeptide | 18889 | -0.364 | -0.3224 | Yes |
| 79 | TYMS | TYMS Entrez,  Source | thymidylate synthetase | 18930 | -0.378 | -0.3013 | Yes |
| 80 | POLE3 | POLE3 Entrez,  Source | polymerase (DNA directed), epsilon 3 (p17 subunit) | 18960 | -0.385 | -0.2791 | Yes |
| 81 | POLR2D | POLR2D Entrez,  Source | polymerase (RNA) II (DNA directed) polypeptide D | 19126 | -0.444 | -0.2603 | Yes |
| 82 | TK1 | TK1 Entrez,  Source | thymidine kinase 1, soluble | 19220 | -0.485 | -0.2354 | Yes |
| 83 | PRIM1 | PRIM1 Entrez,  Source | primase, polypeptide 1, 49kDa | 19268 | -0.519 | -0.2059 | Yes |
| 84 | POLE | POLE Entrez,  Source | polymerase (DNA directed), epsilon | 19281 | -0.529 | -0.1740 | Yes |
| 85 | POLA2 | POLA2 Entrez,  Source | polymerase (DNA directed), alpha 2 (70kD subunit) | 19321 | -0.553 | -0.1421 | Yes |
| 86 | POLA1 | POLA1 Entrez,  Source | polymerase (DNA directed), alpha 1 | 19484 | -0.786 | -0.1022 | Yes |
| 87 | POLD3 | POLD3 Entrez,  Source | polymerase (DNA-directed), delta 3, accessory subunit | 19495 | -0.805 | -0.0533 | Yes |
| 88 | POLE2 | POLE2 Entrez,  Source | polymerase (DNA directed), epsilon 2 (p59 subunit) | 19520 | -0.918 | 0.0018 | Yes |
Table: GSEA details [plain text format]

  

Fig 2: KEGG\_PYRIMIDINE\_METABOLISM      
 Blue-Pink O' Gram in the Space of the Analyzed GeneSet

  

Fig 3: KEGG\_PYRIMIDINE\_METABOLISM: Random ES distribution      
 Gene set null distribution of ES for **KEGG\_PYRIMIDINE\_METABOLISM**
