# Supplementary material for: Comprehensive analysis of β-catenin target genes in colorectal carcinoma cell lines with deregulated Wnt/β-catenin signaling
Source: BMC Genomics. 2014 Jan 28;15:74. doi: 10.1186/1471-2164-15-74 (PMC3909937; doi:10.1186/1471-2164-15-74)
Supplement: Additional file 5 — GSEA analysis using the KEGG pathway database. This zipped file contains confirming data of the GSEA analysis. The names of the directories containing the files were composed of the term ‘GSEA’, the name of the cell line, e.g. DLD1, SW480, or LS174T, and the pathway database (KEGG). Please use a web browser to view the files with the name ‘index.html’ in the corresponding directories to start exploring the data. [file 1471-2164-15-74-S5.zip › GSEA_LS174T_KEGG/KEGG_RETINOL_METABOLISM.html]

Details for gene set KEGG\_RETINOL\_METABOLISM[GSEA]

|  || Dataset | LS174T\_collapsed\_to\_symbols.class.cls#b\_versus\_bg.class.cls#b\_versus\_bg\_repos |
| Phenotype | class.cls#b\_versus\_bg\_repos |
| Upregulated in class | 1 |
| GeneSet | KEGG\_RETINOL\_METABOLISM |
| Enrichment Score (ES) | 0.6792399 |
| Normalized Enrichment Score (NES) | 2.1446056 |
| Nominal p-value | 0.0 |
| FDR q-value | 5.9302326E-4 |
| FWER p-Value | 0.0010 |
Table: GSEA Results Summary

  

Fig 1: Enrichment plot: KEGG\_RETINOL\_METABOLISM      
 Profile of the Running ES Score & Positions of GeneSet Members on the Rank Ordered List

  

| PROBE | GENE SYMBOL | GENE\_TITLE | RANK IN GENE LIST | RANK METRIC SCORE | RUNNING ES | CORE ENRICHMENT || 1 | UGT2B28 | UGT2B28 Entrez,  Source | UDP glucuronosyltransferase 2 family, polypeptide B28 | 15 | 1.479 | 0.1085 | Yes |
| 2 | UGT2B17 | UGT2B17 Entrez,  Source | UDP glucuronosyltransferase 2 family, polypeptide B17 | 21 | 1.429 | 0.2138 | Yes |
| 3 | CYP3A5 | CYP3A5 Entrez,  Source | cytochrome P450, family 3, subfamily A, polypeptide 5 | 34 | 1.267 | 0.3068 | Yes |
| 4 | UGT2B15 | UGT2B15 Entrez,  Source | UDP glucuronosyltransferase 2 family, polypeptide B15 | 70 | 0.989 | 0.3781 | Yes |
| 5 | DHRS3 | DHRS3 Entrez,  Source | dehydrogenase/reductase (SDR family) member 3 | 87 | 0.907 | 0.4443 | Yes |
| 6 | ALDH1A1 | ALDH1A1 Entrez,  Source | aldehyde dehydrogenase 1 family, member A1 | 157 | 0.724 | 0.4943 | Yes |
| 7 | ADH1A | ADH1A Entrez,  Source | alcohol dehydrogenase 1A (class I), alpha polypeptide | 166 | 0.708 | 0.5462 | Yes |
| 8 | BCMO1 | BCMO1 Entrez,  Source | beta-carotene 15,15'-monooxygenase 1 | 180 | 0.676 | 0.5955 | Yes |
| 9 | DHRS9 | DHRS9 Entrez,  Source | dehydrogenase/reductase (SDR family) member 9 | 338 | 0.499 | 0.6244 | Yes |
| 10 | CYP2B6 | CYP2B6 Entrez,  Source | cytochrome P450, family 2, subfamily B, polypeptide 6 | 581 | 0.391 | 0.6409 | Yes |
| 11 | CYP1A1 | CYP1A1 Entrez,  Source | cytochrome P450, family 1, subfamily A, polypeptide 1 | 810 | 0.335 | 0.6540 | Yes |
| 12 | CYP2C18 | CYP2C18 Entrez,  Source | cytochrome P450, family 2, subfamily C, polypeptide 18 | 816 | 0.334 | 0.6784 | Yes |
| 13 | ADH1B | ADH1B Entrez,  Source | alcohol dehydrogenase IB (class I), beta polypeptide | 1618 | 0.234 | 0.6547 | Yes |
| 14 | ALDH1A2 | ALDH1A2 Entrez,  Source | aldehyde dehydrogenase 1 family, member A2 | 1681 | 0.229 | 0.6684 | Yes |
| 15 | CYP2C8 | CYP2C8 Entrez,  Source | cytochrome P450, family 2, subfamily C, polypeptide 8 | 1790 | 0.221 | 0.6792 | Yes |
| 16 | RDH11 | RDH11 Entrez,  Source | retinol dehydrogenase 11 (all-trans/9-cis/11-cis) | 3236 | 0.149 | 0.6162 | No |
| 17 | PNPLA4 | PNPLA4 Entrez,  Source | patatin-like phospholipase domain containing 4 | 3348 | 0.145 | 0.6212 | No |
| 18 | CYP1A2 | CYP1A2 Entrez,  Source | cytochrome P450, family 1, subfamily A, polypeptide 2 | 3536 | 0.138 | 0.6218 | No |
| 19 | RDH10 | RDH10 Entrez,  Source | retinol dehydrogenase 10 (all-trans) | 4409 | 0.111 | 0.5853 | No |
| 20 | UGT2A3 | UGT2A3 Entrez,  Source | UDP glucuronosyltransferase 2 family, polypeptide A3 | 4867 | 0.099 | 0.5692 | No |
| 21 | CYP2C9 | CYP2C9 Entrez,  Source | cytochrome P450, family 2, subfamily C, polypeptide 9 | 5445 | 0.085 | 0.5459 | No |
| 22 | ADH1C | ADH1C Entrez,  Source | alcohol dehydrogenase 1C (class I), gamma polypeptide | 5527 | 0.083 | 0.5479 | No |
| 23 | RETSAT | RETSAT Entrez,  Source | retinol saturase (all-trans-retinol 13,14-reductase) | 6166 | 0.070 | 0.5204 | No |
| 24 | CYP3A4 | CYP3A4 Entrez,  Source | cytochrome P450, family 3, subfamily A, polypeptide 4 | 6476 | 0.063 | 0.5092 | No |
| 25 | CYP4A11 | CYP4A11 Entrez,  Source | cytochrome P450, family 4, subfamily A, polypeptide 11 | 7273 | 0.048 | 0.4720 | No |
| 26 | RDH16 | RDH16 Entrez,  Source | retinol dehydrogenase 16 (all-trans) | 7470 | 0.045 | 0.4653 | No |
| 27 | LRAT | LRAT Entrez,  Source | lecithin retinol acyltransferase (phosphatidylcholine--retinol O-acyltransferase) | 7643 | 0.041 | 0.4595 | No |
| 28 | RDH8 | RDH8 Entrez,  Source | retinol dehydrogenase 8 (all-trans) | 8574 | 0.024 | 0.4136 | No |
| 29 | RDH5 | RDH5 Entrez,  Source | retinol dehydrogenase 5 (11-cis/9-cis) | 10300 | -0.003 | 0.3255 | No |
| 30 | UGT1A9 | UGT1A9 Entrez,  Source | UDP glucuronosyltransferase 1 family, polypeptide A9 | 10361 | -0.005 | 0.3227 | No |
| 31 | CYP2A6 | CYP2A6 Entrez,  Source | cytochrome P450, family 2, subfamily A, polypeptide 6 | 10879 | -0.013 | 0.2972 | No |
| 32 | ADH6 | ADH6 Entrez,  Source | alcohol dehydrogenase 6 (class V) | 11401 | -0.023 | 0.2722 | No |
| 33 | ADH7 | ADH7 Entrez,  Source | alcohol dehydrogenase 7 (class IV), mu or sigma polypeptide | 11465 | -0.024 | 0.2707 | No |
| 34 | ADH5 | ADH5 Entrez,  Source | alcohol dehydrogenase 5 (class III), chi polypeptide | 11976 | -0.032 | 0.2470 | No |
| 35 | CYP2A13 | CYP2A13 Entrez,  Source | cytochrome P450, family 2, subfamily A, polypeptide 13 | 14053 | -0.074 | 0.1460 | No |
| 36 | CYP3A7 | CYP3A7 Entrez,  Source | cytochrome P450, family 3, subfamily A, polypeptide 7 | 14947 | -0.095 | 0.1073 | No |
| 37 | RPE65 | RPE65 Entrez,  Source | retinal pigment epithelium-specific protein 65kDa | 15365 | -0.108 | 0.0939 | No |
| 38 | CYP26A1 | CYP26A1 Entrez,  Source | cytochrome P450, family 26, subfamily A, polypeptide 1 | 15499 | -0.111 | 0.0953 | No |
| 39 | RDH12 | RDH12 Entrez,  Source | retinol dehydrogenase 12 (all-trans/9-cis/11-cis) | 15581 | -0.114 | 0.0995 | No |
| 40 | CYP2C19 | CYP2C19 Entrez,  Source | cytochrome P450, family 2, subfamily C, polypeptide 19 | 17104 | -0.175 | 0.0345 | No |
| 41 | CYP26B1 | CYP26B1 Entrez,  Source | cytochrome P450, family 26, subfamily B, polypeptide 1 | 17209 | -0.181 | 0.0425 | No |
| 42 | ADH4 | ADH4 Entrez,  Source | alcohol dehydrogenase 4 (class II), pi polypeptide | 17566 | -0.204 | 0.0393 | No |
| 43 | DGAT2 | DGAT2 Entrez,  Source | diacylglycerol O-acyltransferase homolog 2 (mouse) | 17690 | -0.212 | 0.0486 | No |
| 44 | CYP3A43 | CYP3A43 Entrez,  Source | cytochrome P450, family 3, subfamily A, polypeptide 43 | 17928 | -0.230 | 0.0534 | No |
| 45 | UGT2B4 | UGT2B4 Entrez,  Source | UDP glucuronosyltransferase 2 family, polypeptide B4 | 19031 | -0.405 | 0.0269 | No |
Table: GSEA details [plain text format]

  

Fig 2: KEGG\_RETINOL\_METABOLISM      
 Blue-Pink O' Gram in the Space of the Analyzed GeneSet

  

Fig 3: KEGG\_RETINOL\_METABOLISM: Random ES distribution      
 Gene set null distribution of ES for **KEGG\_RETINOL\_METABOLISM**
