# Supplementary material for: Comprehensive analysis of β-catenin target genes in colorectal carcinoma cell lines with deregulated Wnt/β-catenin signaling
Source: BMC Genomics. 2014 Jan 28;15:74. doi: 10.1186/1471-2164-15-74 (PMC3909937; doi:10.1186/1471-2164-15-74)
Supplement: Additional file 5 — GSEA analysis using the KEGG pathway database. This zipped file contains confirming data of the GSEA analysis. The names of the directories containing the files were composed of the term ‘GSEA’, the name of the cell line, e.g. DLD1, SW480, or LS174T, and the pathway database (KEGG). Please use a web browser to view the files with the name ‘index.html’ in the corresponding directories to start exploring the data. [file 1471-2164-15-74-S5.zip › GSEA_LS174T_KEGG/KEGG_RNA_DEGRADATION.html]

Details for gene set KEGG\_RNA\_DEGRADATION[GSEA]

|  || Dataset | LS174T\_collapsed\_to\_symbols.class.cls#b\_versus\_bg.class.cls#b\_versus\_bg\_repos |
| Phenotype | class.cls#b\_versus\_bg\_repos |
| Upregulated in class | 0 |
| GeneSet | KEGG\_RNA\_DEGRADATION |
| Enrichment Score (ES) | -0.4492226 |
| Normalized Enrichment Score (NES) | -1.5269263 |
| Nominal p-value | 0.023706896 |
| FDR q-value | 0.119374216 |
| FWER p-Value | 0.804 |
Table: GSEA Results Summary

  

Fig 1: Enrichment plot: KEGG\_RNA\_DEGRADATION      
 Profile of the Running ES Score & Positions of GeneSet Members on the Rank Ordered List

  

| PROBE | GENE SYMBOL | GENE\_TITLE | RANK IN GENE LIST | RANK METRIC SCORE | RUNNING ES | CORE ENRICHMENT || 1 | DCP2 | DCP2 Entrez,  Source | DCP2 decapping enzyme homolog (S. cerevisiae) | 950 | 0.309 | 0.0012 | No |
| 2 | PAPOLB | PAPOLB Entrez,  Source | poly(A) polymerase beta (testis specific) | 3083 | 0.154 | -0.0832 | No |
| 3 | XRN1 | XRN1 Entrez,  Source | 5'-3' exoribonuclease 1 | 3768 | 0.130 | -0.0972 | No |
| 4 | C1D | C1D Entrez,  Source | - | 3952 | 0.125 | -0.0864 | No |
| 5 | DDX6 | DDX6 Entrez,  Source | DEAD (Asp-Glu-Ala-Asp) box polypeptide 6 | 3985 | 0.124 | -0.0681 | No |
| 6 | SKIV2L | SKIV2L Entrez,  Source | superkiller viralicidic activity 2-like (S. cerevisiae) | 4643 | 0.105 | -0.0848 | No |
| 7 | PAPOLG | PAPOLG Entrez,  Source | poly(A) polymerase gamma | 4783 | 0.101 | -0.0755 | No |
| 8 | ENO3 | ENO3 Entrez,  Source | enolase 3 (beta, muscle) | 5177 | 0.091 | -0.0809 | No |
| 9 | ZCCHC7 | ZCCHC7 Entrez,  Source | zinc finger, CCHC domain containing 7 | 6869 | 0.056 | -0.1585 | No |
| 10 | EXOSC1 | EXOSC1 Entrez,  Source | exosome component 1 | 7860 | 0.038 | -0.2032 | No |
| 11 | CNOT4 | CNOT4 Entrez,  Source | CCR4-NOT transcription complex, subunit 4 | 8167 | 0.032 | -0.2138 | No |
| 12 | XRN2 | XRN2 Entrez,  Source | 5'-3' exoribonuclease 2 | 8286 | 0.029 | -0.2152 | No |
| 13 | CNOT8 | CNOT8 Entrez,  Source | CCR4-NOT transcription complex, subunit 8 | 8442 | 0.027 | -0.2188 | No |
| 14 | EXOSC4 | EXOSC4 Entrez,  Source | exosome component 4 | 10236 | -0.002 | -0.3103 | No |
| 15 | CNOT7 | CNOT7 Entrez,  Source | CCR4-NOT transcription complex, subunit 7 | 10691 | -0.010 | -0.3320 | No |
| 16 | WDR61 | WDR61 Entrez,  Source | WD repeat domain 61 | 11093 | -0.017 | -0.3498 | No |
| 17 | SKIV2L2 | SKIV2L2 Entrez,  Source | superkiller viralicidic activity 2-like 2 (S. cerevisiae) | 11284 | -0.021 | -0.3563 | No |
| 18 | CNOT10 | CNOT10 Entrez,  Source | CCR4-NOT transcription complex, subunit 10 | 11682 | -0.027 | -0.3722 | No |
| 19 | CNOT2 | CNOT2 Entrez,  Source | CCR4-NOT transcription complex, subunit 2 | 11690 | -0.028 | -0.3681 | No |
| 20 | LSM1 | LSM1 Entrez,  Source | LSM1 homolog, U6 small nuclear RNA associated (S. cerevisiae) | 11705 | -0.028 | -0.3643 | No |
| 21 | PARN | PARN Entrez,  Source | poly(A)-specific ribonuclease (deadenylation nuclease) | 11727 | -0.028 | -0.3608 | No |
| 22 | DCP1B | DCP1B Entrez,  Source | DCP1 decapping enzyme homolog B (S. cerevisiae) | 11872 | -0.031 | -0.3632 | No |
| 23 | EXOSC10 | EXOSC10 Entrez,  Source | exosome component 10 | 12259 | -0.038 | -0.3769 | No |
| 24 | EDC3 | EDC3 Entrez,  Source | enhancer of mRNA decapping 3 homolog (S. cerevisiae) | 12511 | -0.043 | -0.3829 | No |
| 25 | HSPD1 | HSPD1 Entrez,  Source | heat shock 60kDa protein 1 (chaperonin) | 12555 | -0.043 | -0.3781 | No |
| 26 | LSM4 | LSM4 Entrez,  Source | LSM4 homolog, U6 small nuclear RNA associated (S. cerevisiae) | 12650 | -0.045 | -0.3756 | No |
| 27 | ENO1 | ENO1 Entrez,  Source | enolase 1, (alpha) | 12718 | -0.046 | -0.3716 | No |
| 28 | CNOT6L | CNOT6L Entrez,  Source | CCR4-NOT transcription complex, subunit 6-like | 12987 | -0.051 | -0.3770 | No |
| 29 | PNPT1 | PNPT1 Entrez,  Source | polyribonucleotide nucleotidyltransferase 1 | 13203 | -0.055 | -0.3791 | No |
| 30 | DCP1A | DCP1A Entrez,  Source | DCP1 decapping enzyme homolog A (S. cerevisiae) | 13767 | -0.067 | -0.3971 | No |
| 31 | EXOSC6 | EXOSC6 Entrez,  Source | exosome component 6 | 14008 | -0.073 | -0.3976 | No |
| 32 | PAPOLA | PAPOLA Entrez,  Source | poly(A) polymerase alpha | 14203 | -0.077 | -0.3951 | No |
| 33 | EDC4 | EDC4 Entrez,  Source | enhancer of mRNA decapping 4 | 14496 | -0.084 | -0.3965 | No |
| 34 | CNOT1 | CNOT1 Entrez,  Source | CCR4-NOT transcription complex, subunit 1 | 15193 | -0.103 | -0.4156 | No |
| 35 | MPHOSPH6 | MPHOSPH6 Entrez,  Source | M-phase phosphoprotein 6 | 15262 | -0.105 | -0.4022 | No |
| 36 | ENO2 | ENO2 Entrez,  Source | enolase 2 (gamma, neuronal) | 15411 | -0.109 | -0.3922 | No |
| 37 | LSM5 | LSM5 Entrez,  Source | LSM5 homolog, U6 small nuclear RNA associated (S. cerevisiae) | 16401 | -0.143 | -0.4198 | No |
| 38 | LSM2 | LSM2 Entrez,  Source | LSM2 homolog, U6 small nuclear RNA associated (S. cerevisiae) | 16975 | -0.168 | -0.4221 | Yes |
| 39 | LSM3 | LSM3 Entrez,  Source | LSM3 homolog, U6 small nuclear RNA associated (S. cerevisiae) | 17329 | -0.187 | -0.4100 | Yes |
| 40 | CNOT6 | CNOT6 Entrez,  Source | CCR4-NOT transcription complex, subunit 6 | 17358 | -0.189 | -0.3808 | Yes |
| 41 | LSM7 | LSM7 Entrez,  Source | LSM7 homolog, U6 small nuclear RNA associated (S. cerevisiae) | 17486 | -0.198 | -0.3553 | Yes |
| 42 | EXOSC5 | EXOSC5 Entrez,  Source | exosome component 5 | 17642 | -0.208 | -0.3297 | Yes |
| 43 | RQCD1 | RQCD1 Entrez,  Source | RCD1 required for cell differentiation1 homolog (S. pombe) | 17658 | -0.209 | -0.2967 | Yes |
| 44 | DCPS | DCPS Entrez,  Source | decapping enzyme, scavenger | 17972 | -0.234 | -0.2750 | Yes |
| 45 | CNOT3 | CNOT3 Entrez,  Source | CCR4-NOT transcription complex, subunit 3 | 18025 | -0.239 | -0.2391 | Yes |
| 46 | LSM6 | LSM6 Entrez,  Source | LSM6 homolog, U6 small nuclear RNA associated (S. cerevisiae) | 18322 | -0.270 | -0.2106 | Yes |
| 47 | EXOSC8 | EXOSC8 Entrez,  Source | exosome component 8 | 18423 | -0.281 | -0.1703 | Yes |
| 48 | EXOSC9 | EXOSC9 Entrez,  Source | exosome component 9 | 18433 | -0.282 | -0.1252 | Yes |
| 49 | EXOSC2 | EXOSC2 Entrez,  Source | exosome component 2 | 18503 | -0.293 | -0.0814 | Yes |
| 50 | EXOSC7 | EXOSC7 Entrez,  Source | exosome component 7 | 18583 | -0.304 | -0.0363 | Yes |
| 51 | EXOSC3 | EXOSC3 Entrez,  Source | exosome component 3 | 19289 | -0.533 | 0.0137 | Yes |
Table: GSEA details [plain text format]

  

Fig 2: KEGG\_RNA\_DEGRADATION      
 Blue-Pink O' Gram in the Space of the Analyzed GeneSet

  

Fig 3: KEGG\_RNA\_DEGRADATION: Random ES distribution      
 Gene set null distribution of ES for **KEGG\_RNA\_DEGRADATION**
