# Supplementary material for: Comprehensive analysis of β-catenin target genes in colorectal carcinoma cell lines with deregulated Wnt/β-catenin signaling
Source: BMC Genomics. 2014 Jan 28;15:74. doi: 10.1186/1471-2164-15-74 (PMC3909937; doi:10.1186/1471-2164-15-74)
Supplement: Additional file 5 — GSEA analysis using the KEGG pathway database. This zipped file contains confirming data of the GSEA analysis. The names of the directories containing the files were composed of the term ‘GSEA’, the name of the cell line, e.g. DLD1, SW480, or LS174T, and the pathway database (KEGG). Please use a web browser to view the files with the name ‘index.html’ in the corresponding directories to start exploring the data. [file 1471-2164-15-74-S5.zip › GSEA_LS174T_KEGG/KEGG_RNA_POLYMERASE.html]

Details for gene set KEGG\_RNA\_POLYMERASE[GSEA]

|  || Dataset | LS174T\_collapsed\_to\_symbols.class.cls#b\_versus\_bg.class.cls#b\_versus\_bg\_repos |
| Phenotype | class.cls#b\_versus\_bg\_repos |
| Upregulated in class | 0 |
| GeneSet | KEGG\_RNA\_POLYMERASE |
| Enrichment Score (ES) | -0.4998835 |
| Normalized Enrichment Score (NES) | -1.474377 |
| Nominal p-value | 0.045908183 |
| FDR q-value | 0.16318129 |
| FWER p-Value | 0.909 |
Table: GSEA Results Summary

  

Fig 1: Enrichment plot: KEGG\_RNA\_POLYMERASE      
 Profile of the Running ES Score & Positions of GeneSet Members on the Rank Ordered List

  

| PROBE | GENE SYMBOL | GENE\_TITLE | RANK IN GENE LIST | RANK METRIC SCORE | RUNNING ES | CORE ENRICHMENT || 1 | POLR3D | POLR3D Entrez,  Source | polymerase (RNA) III (DNA directed) polypeptide D, 44kDa | 2762 | 0.166 | -0.0863 | No |
| 2 | POLR3A | POLR3A Entrez,  Source | polymerase (RNA) III (DNA directed) polypeptide A, 155kDa | 3971 | 0.124 | -0.1069 | No |
| 3 | POLR2L | POLR2L Entrez,  Source | polymerase (RNA) II (DNA directed) polypeptide L, 7.6kDa | 7901 | 0.037 | -0.2960 | No |
| 4 | POLR1D | POLR1D Entrez,  Source | polymerase (RNA) I polypeptide D, 16kDa | 9198 | 0.014 | -0.3577 | No |
| 5 | POLR2J2 | POLR2J2 Entrez,  Source | - | 10373 | -0.005 | -0.4162 | No |
| 6 | POLR2J | POLR2J Entrez,  Source | polymerase (RNA) II (DNA directed) polypeptide J, 13.3kDa | 10628 | -0.009 | -0.4263 | No |
| 7 | POLR2B | POLR2B Entrez,  Source | polymerase (RNA) II (DNA directed) polypeptide B, 140kDa | 11138 | -0.018 | -0.4465 | No |
| 8 | POLR3B | POLR3B Entrez,  Source | polymerase (RNA) III (DNA directed) polypeptide B | 11250 | -0.020 | -0.4456 | No |
| 9 | POLR3F | POLR3F Entrez,  Source | polymerase (RNA) III (DNA directed) polypeptide F, 39 kDa | 12059 | -0.034 | -0.4758 | No |
| 10 | POLR3H | POLR3H Entrez,  Source | polymerase (RNA) III (DNA directed) polypeptide H (22.9kD) | 12523 | -0.043 | -0.4852 | Yes |
| 11 | POLR2I | POLR2I Entrez,  Source | polymerase (RNA) II (DNA directed) polypeptide I, 14.5kDa | 12535 | -0.043 | -0.4715 | Yes |
| 12 | POLR2K | POLR2K Entrez,  Source | polymerase (RNA) II (DNA directed) polypeptide K, 7.0kDa | 13090 | -0.053 | -0.4823 | Yes |
| 13 | POLR1C | POLR1C Entrez,  Source | polymerase (RNA) I polypeptide C, 30kDa | 13175 | -0.055 | -0.4684 | Yes |
| 14 | POLR2C | POLR2C Entrez,  Source | polymerase (RNA) II (DNA directed) polypeptide C, 33kDa | 13358 | -0.059 | -0.4582 | Yes |
| 15 | POLR2E | POLR2E Entrez,  Source | polymerase (RNA) II (DNA directed) polypeptide E, 25kDa | 14089 | -0.075 | -0.4709 | Yes |
| 16 | POLR2H | POLR2H Entrez,  Source | polymerase (RNA) II (DNA directed) polypeptide H | 14235 | -0.078 | -0.4525 | Yes |
| 17 | POLR2F | POLR2F Entrez,  Source | polymerase (RNA) II (DNA directed) polypeptide F | 14269 | -0.078 | -0.4282 | Yes |
| 18 | POLR3C | POLR3C Entrez,  Source | polymerase (RNA) III (DNA directed) polypeptide C (62kD) | 15557 | -0.113 | -0.4566 | Yes |
| 19 | POLR2A | POLR2A Entrez,  Source | polymerase (RNA) II (DNA directed) polypeptide A, 220kDa | 15930 | -0.124 | -0.4344 | Yes |
| 20 | POLR1E | POLR1E Entrez,  Source | polymerase (RNA) I polypeptide E, 53kDa | 16145 | -0.133 | -0.4013 | Yes |
| 21 | ZNRD1 | ZNRD1 Entrez,  Source | zinc ribbon domain containing 1 | 16598 | -0.150 | -0.3746 | Yes |
| 22 | POLR1B | POLR1B Entrez,  Source | polymerase (RNA) I polypeptide B, 128kDa | 16699 | -0.155 | -0.3282 | Yes |
| 23 | POLR3G | POLR3G Entrez,  Source | polymerase (RNA) III (DNA directed) polypeptide G (32kD) | 16729 | -0.157 | -0.2778 | Yes |
| 24 | POLR3GL | POLR3GL Entrez,  Source | polymerase (RNA) III (DNA directed) polypeptide G (32kD) like | 16887 | -0.164 | -0.2316 | Yes |
| 25 | POLR2G | POLR2G Entrez,  Source | polymerase (RNA) II (DNA directed) polypeptide G | 16910 | -0.165 | -0.1781 | Yes |
| 26 | POLR3K | POLR3K Entrez,  Source | polymerase (RNA) III (DNA directed) polypeptide K, 12.3 kDa | 17437 | -0.194 | -0.1406 | Yes |
| 27 | POLR1A | POLR1A Entrez,  Source | polymerase (RNA) I polypeptide A, 194kDa | 18602 | -0.307 | -0.0985 | Yes |
| 28 | POLR2D | POLR2D Entrez,  Source | polymerase (RNA) II (DNA directed) polypeptide D | 19126 | -0.444 | 0.0220 | Yes |
Table: GSEA details [plain text format]

  

Fig 2: KEGG\_RNA\_POLYMERASE      
 Blue-Pink O' Gram in the Space of the Analyzed GeneSet

  

Fig 3: KEGG\_RNA\_POLYMERASE: Random ES distribution      
 Gene set null distribution of ES for **KEGG\_RNA\_POLYMERASE**
