# Supplementary material for: Comprehensive analysis of β-catenin target genes in colorectal carcinoma cell lines with deregulated Wnt/β-catenin signaling
Source: BMC Genomics. 2014 Jan 28;15:74. doi: 10.1186/1471-2164-15-74 (PMC3909937; doi:10.1186/1471-2164-15-74)
Supplement: Additional file 5 — GSEA analysis using the KEGG pathway database. This zipped file contains confirming data of the GSEA analysis. The names of the directories containing the files were composed of the term ‘GSEA’, the name of the cell line, e.g. DLD1, SW480, or LS174T, and the pathway database (KEGG). Please use a web browser to view the files with the name ‘index.html’ in the corresponding directories to start exploring the data. [file 1471-2164-15-74-S5.zip › GSEA_LS174T_KEGG/KEGG_SPLICEOSOME.html]

Details for gene set KEGG\_SPLICEOSOME[GSEA]

|  || Dataset | LS174T\_collapsed\_to\_symbols.class.cls#b\_versus\_bg.class.cls#b\_versus\_bg\_repos |
| Phenotype | class.cls#b\_versus\_bg\_repos |
| Upregulated in class | 0 |
| GeneSet | KEGG\_SPLICEOSOME |
| Enrichment Score (ES) | -0.48232174 |
| Normalized Enrichment Score (NES) | -1.8502344 |
| Nominal p-value | 0.0 |
| FDR q-value | 0.0068229428 |
| FWER p-Value | 0.067 |
Table: GSEA Results Summary

  

Fig 1: Enrichment plot: KEGG\_SPLICEOSOME      
 Profile of the Running ES Score & Positions of GeneSet Members on the Rank Ordered List

  

| PROBE | GENE SYMBOL | GENE\_TITLE | RANK IN GENE LIST | RANK METRIC SCORE | RUNNING ES | CORE ENRICHMENT || 1 | HSPA1L | HSPA1L Entrez,  Source | heat shock 70kDa protein 1-like | 237 | 0.607 | 0.0445 | No |
| 2 | HSPA1A | HSPA1A Entrez,  Source | heat shock 70kDa protein 1A | 394 | 0.463 | 0.0797 | No |
| 3 | HSPA2 | HSPA2 Entrez,  Source | heat shock 70kDa protein 2 | 5187 | 0.091 | -0.1580 | No |
| 4 | DHX16 | DHX16 Entrez,  Source | DEAH (Asp-Glu-Ala-His) box polypeptide 16 | 6512 | 0.063 | -0.2202 | No |
| 5 | PQBP1 | PQBP1 Entrez,  Source | polyglutamine binding protein 1 | 6890 | 0.056 | -0.2343 | No |
| 6 | HSPA6 | HSPA6 Entrez,  Source | heat shock 70kDa protein 6 (HSP70B') | 7288 | 0.048 | -0.2502 | No |
| 7 | SYF2 | SYF2 Entrez,  Source | SYF2 homolog, RNA splicing factor (S. cerevisiae) | 7398 | 0.046 | -0.2515 | No |
| 8 | DDX42 | DDX42 Entrez,  Source | DEAD (Asp-Glu-Ala-Asp) box polypeptide 42 | 7809 | 0.039 | -0.2690 | No |
| 9 | CDC40 | CDC40 Entrez,  Source | cell division cycle 40 homolog (S. cerevisiae) | 8209 | 0.031 | -0.2866 | No |
| 10 | CDC5L | CDC5L Entrez,  Source | CDC5 cell division cycle 5-like (S. pombe) | 8402 | 0.027 | -0.2939 | No |
| 11 | CRNKL1 | CRNKL1 Entrez,  Source | Crn, crooked neck-like 1 (Drosophila) | 8469 | 0.026 | -0.2949 | No |
| 12 | PLRG1 | PLRG1 Entrez,  Source | pleiotropic regulator 1 (PRL1 homolog, Arabidopsis) | 8532 | 0.025 | -0.2958 | No |
| 13 | RBMX | RBMX Entrez,  Source | RNA binding motif protein, X-linked | 8802 | 0.020 | -0.3077 | No |
| 14 | RBM22 | RBM22 Entrez,  Source | RNA binding motif protein 22 | 8918 | 0.019 | -0.3118 | No |
| 15 | SART1 | SART1 Entrez,  Source | squamous cell carcinoma antigen recognised by T cells | 9294 | 0.012 | -0.3299 | No |
| 16 | SF3B5 | SF3B5 Entrez,  Source | splicing factor 3b, subunit 5, 10kDa | 9912 | 0.003 | -0.3614 | No |
| 17 | DDX5 | DDX5 Entrez,  Source | DEAD (Asp-Glu-Ala-Asp) box polypeptide 5 | 10250 | -0.003 | -0.3785 | No |
| 18 | PCBP1 | PCBP1 Entrez,  Source | poly(rC) binding protein 1 | 10346 | -0.004 | -0.3829 | No |
| 19 | HSPA8 | HSPA8 Entrez,  Source | heat shock 70kDa protein 8 | 10474 | -0.006 | -0.3889 | No |
| 20 | DHX8 | DHX8 Entrez,  Source | DEAH (Asp-Glu-Ala-His) box polypeptide 8 | 10926 | -0.014 | -0.4108 | No |
| 21 | PRPF6 | PRPF6 Entrez,  Source | PRP6 pre-mRNA processing factor 6 homolog (S. cerevisiae) | 11042 | -0.016 | -0.4152 | No |
| 22 | U2AF1 | U2AF1 Entrez,  Source | U2 small nuclear RNA auxiliary factor 1 | 11061 | -0.016 | -0.4146 | No |
| 23 | SNW1 | SNW1 Entrez,  Source | SNW domain containing 1 | 11063 | -0.016 | -0.4131 | No |
| 24 | NCBP2 | NCBP2 Entrez,  Source | nuclear cap binding protein subunit 2, 20kDa | 11260 | -0.020 | -0.4213 | No |
| 25 | SLU7 | SLU7 Entrez,  Source | - | 11281 | -0.020 | -0.4204 | No |
| 26 | SNRPD2 | SNRPD2 Entrez,  Source | small nuclear ribonucleoprotein D2 polypeptide 16.5kDa | 11330 | -0.021 | -0.4209 | No |
| 27 | SF3B1 | SF3B1 Entrez,  Source | splicing factor 3b, subunit 1, 155kDa | 11786 | -0.029 | -0.4415 | No |
| 28 | SF3B2 | SF3B2 Entrez,  Source | splicing factor 3b, subunit 2, 145kDa | 11798 | -0.030 | -0.4393 | No |
| 29 | EFTUD2 | EFTUD2 Entrez,  Source | elongation factor Tu GTP binding domain containing 2 | 11812 | -0.030 | -0.4372 | No |
| 30 | BCAS2 | BCAS2 Entrez,  Source | breast carcinoma amplified sequence 2 | 11905 | -0.031 | -0.4390 | No |
| 31 | SF3B4 | SF3B4 Entrez,  Source | splicing factor 3b, subunit 4, 49kDa | 12069 | -0.034 | -0.4442 | No |
| 32 | PRPF40A | PRPF40A Entrez,  Source | PRP40 pre-mRNA processing factor 40 homolog A (yeast) | 12102 | -0.035 | -0.4426 | No |
| 33 | SF3A1 | SF3A1 Entrez,  Source | splicing factor 3a, subunit 1, 120kDa | 12475 | -0.042 | -0.4578 | No |
| 34 | SMNDC1 | SMNDC1 Entrez,  Source | survival motor neuron domain containing 1 | 12527 | -0.043 | -0.4564 | No |
| 35 | LSM4 | LSM4 Entrez,  Source | LSM4 homolog, U6 small nuclear RNA associated (S. cerevisiae) | 12650 | -0.045 | -0.4584 | No |
| 36 | NHP2L1 | NHP2L1 Entrez,  Source | NHP2 non-histone chromosome protein 2-like 1 (S. cerevisiae) | 12734 | -0.047 | -0.4584 | No |
| 37 | PRPF19 | PRPF19 Entrez,  Source | PRP19/PSO4 pre-mRNA processing factor 19 homolog (S. cerevisiae) | 12801 | -0.048 | -0.4573 | No |
| 38 | PPIE | PPIE Entrez,  Source | peptidylprolyl isomerase E (cyclophilin E) | 12916 | -0.050 | -0.4585 | No |
| 39 | PRPF8 | PRPF8 Entrez,  Source | PRP8 pre-mRNA processing factor 8 homolog (S. cerevisiae) | 13148 | -0.054 | -0.4653 | No |
| 40 | PRPF18 | PRPF18 Entrez,  Source | PRP18 pre-mRNA processing factor 18 homolog (S. cerevisiae) | 13288 | -0.057 | -0.4670 | No |
| 41 | SNRPB2 | SNRPB2 Entrez,  Source | small nuclear ribonucleoprotein polypeptide B'' | 13400 | -0.060 | -0.4672 | No |
| 42 | SNRPE | SNRPE Entrez,  Source | small nuclear ribonucleoprotein polypeptide E | 13451 | -0.061 | -0.4641 | No |
| 43 | THOC2 | THOC2 Entrez,  Source | THO complex 2 | 13515 | -0.062 | -0.4615 | No |
| 44 | TXNL4A | TXNL4A Entrez,  Source | thioredoxin-like 4A | 13544 | -0.063 | -0.4571 | No |
| 45 | DHX38 | DHX38 Entrez,  Source | DEAH (Asp-Glu-Ala-His) box polypeptide 38 | 13655 | -0.065 | -0.4567 | No |
| 46 | U2AF2 | U2AF2 Entrez,  Source | U2 small nuclear RNA auxiliary factor 2 | 13687 | -0.065 | -0.4522 | No |
| 47 | SNRPC | SNRPC Entrez,  Source | small nuclear ribonucleoprotein polypeptide C | 13820 | -0.069 | -0.4526 | No |
| 48 | BUD31 | BUD31 Entrez,  Source | BUD31 homolog (yeast) | 14321 | -0.080 | -0.4708 | No |
| 49 | PRPF38A | PRPF38A Entrez,  Source | PRP38 pre-mRNA processing factor 38 (yeast) domain containing A | 14479 | -0.084 | -0.4711 | No |
| 50 | CCDC12 | CCDC12 Entrez,  Source | coiled-coil domain containing 12 | 14699 | -0.089 | -0.4740 | Yes |
| 51 | DHX15 | DHX15 Entrez,  Source | DEAH (Asp-Glu-Ala-His) box polypeptide 15 | 14704 | -0.090 | -0.4658 | Yes |
| 52 | PPIL1 | PPIL1 Entrez,  Source | peptidylprolyl isomerase (cyclophilin)-like 1 | 14848 | -0.093 | -0.4645 | Yes |
| 53 | XAB2 | XAB2 Entrez,  Source | XPA binding protein 2 | 14887 | -0.094 | -0.4576 | Yes |
| 54 | PRPF3 | PRPF3 Entrez,  Source | PRP3 pre-mRNA processing factor 3 homolog (S. cerevisiae) | 15171 | -0.102 | -0.4626 | Yes |
| 55 | SF3A2 | SF3A2 Entrez,  Source | splicing factor 3a, subunit 2, 66kDa | 15317 | -0.106 | -0.4602 | Yes |
| 56 | RBM8A | RBM8A Entrez,  Source | RNA binding motif protein 8A | 15387 | -0.108 | -0.4536 | Yes |
| 57 | SNRPB | SNRPB Entrez,  Source | small nuclear ribonucleoprotein polypeptides B and B1 | 15405 | -0.109 | -0.4443 | Yes |
| 58 | TRA2A | TRA2A Entrez,  Source | - | 15520 | -0.112 | -0.4397 | Yes |
| 59 | SNRPD3 | SNRPD3 Entrez,  Source | small nuclear ribonucleoprotein D3 polypeptide 18kDa | 15726 | -0.118 | -0.4393 | Yes |
| 60 | SNRPG | SNRPG Entrez,  Source | small nuclear ribonucleoprotein polypeptide G | 15728 | -0.118 | -0.4283 | Yes |
| 61 | SNRPA | SNRPA Entrez,  Source | small nuclear ribonucleoprotein polypeptide A | 16009 | -0.128 | -0.4308 | Yes |
| 62 | PRPF31 | PRPF31 Entrez,  Source | PRP31 pre-mRNA processing factor 31 homolog (S. cerevisiae) | 16198 | -0.135 | -0.4278 | Yes |
| 63 | AQR | AQR Entrez,  Source | aquarius homolog (mouse) | 16219 | -0.136 | -0.4161 | Yes |
| 64 | LSM5 | LSM5 Entrez,  Source | LSM5 homolog, U6 small nuclear RNA associated (S. cerevisiae) | 16401 | -0.143 | -0.4121 | Yes |
| 65 | DDX23 | DDX23 Entrez,  Source | DEAD (Asp-Glu-Ala-Asp) box polypeptide 23 | 16411 | -0.143 | -0.3993 | Yes |
| 66 | CTNNBL1 | CTNNBL1 Entrez,  Source | catenin, beta like 1 | 16479 | -0.145 | -0.3891 | Yes |
| 67 | SNRPF | SNRPF Entrez,  Source | small nuclear ribonucleoprotein polypeptide F | 16656 | -0.153 | -0.3839 | Yes |
| 68 | RBM17 | RBM17 Entrez,  Source | RNA binding motif protein 17 | 16747 | -0.158 | -0.3738 | Yes |
| 69 | CHERP | CHERP Entrez,  Source | calcium homeostasis endoplasmic reticulum protein | 16966 | -0.168 | -0.3693 | Yes |
| 70 | SNRPA1 | SNRPA1 Entrez,  Source | small nuclear ribonucleoprotein polypeptide A' | 16973 | -0.168 | -0.3539 | Yes |
| 71 | LSM2 | LSM2 Entrez,  Source | LSM2 homolog, U6 small nuclear RNA associated (S. cerevisiae) | 16975 | -0.168 | -0.3383 | Yes |
| 72 | ACIN1 | ACIN1 Entrez,  Source | apoptotic chromatin condensation inducer 1 | 16981 | -0.169 | -0.3228 | Yes |
| 73 | WBP11 | WBP11 Entrez,  Source | WW domain binding protein 11 | 17038 | -0.172 | -0.3096 | Yes |
| 74 | THOC1 | THOC1 Entrez,  Source | THO complex 1 | 17086 | -0.174 | -0.2958 | Yes |
| 75 | NCBP1 | NCBP1 Entrez,  Source | nuclear cap binding protein subunit 1, 80kDa | 17108 | -0.175 | -0.2805 | Yes |
| 76 | PHF5A | PHF5A Entrez,  Source | PHD finger protein 5A | 17162 | -0.178 | -0.2666 | Yes |
| 77 | LSM3 | LSM3 Entrez,  Source | LSM3 homolog, U6 small nuclear RNA associated (S. cerevisiae) | 17329 | -0.187 | -0.2577 | Yes |
| 78 | SNRPD1 | SNRPD1 Entrez,  Source | small nuclear ribonucleoprotein D1 polypeptide 16kDa | 17332 | -0.187 | -0.2403 | Yes |
| 79 | DDX46 | DDX46 Entrez,  Source | DEAD (Asp-Glu-Ala-Asp) box polypeptide 46 | 17386 | -0.192 | -0.2252 | Yes |
| 80 | LSM7 | LSM7 Entrez,  Source | LSM7 homolog, U6 small nuclear RNA associated (S. cerevisiae) | 17486 | -0.198 | -0.2117 | Yes |
| 81 | ZMAT2 | ZMAT2 Entrez,  Source | zinc finger, matrin type 2 | 17496 | -0.199 | -0.1936 | Yes |
| 82 | RBM25 | RBM25 Entrez,  Source | RNA binding motif protein 25 | 17720 | -0.213 | -0.1852 | Yes |
| 83 | PRPF4 | PRPF4 Entrez,  Source | PRP4 pre-mRNA processing factor 4 homolog (yeast) | 17749 | -0.216 | -0.1665 | Yes |
| 84 | USP39 | USP39 Entrez,  Source | ubiquitin specific peptidase 39 | 17827 | -0.220 | -0.1499 | Yes |
| 85 | PRPF38B | PRPF38B Entrez,  Source | PRP38 pre-mRNA processing factor 38 (yeast) domain containing B | 18008 | -0.237 | -0.1370 | Yes |
| 86 | SF3B14 | SF3B14 Entrez,  Source | - | 18192 | -0.255 | -0.1226 | Yes |
| 87 | PPIH | PPIH Entrez,  Source | peptidylprolyl isomerase H (cyclophilin H) | 18244 | -0.261 | -0.1008 | Yes |
| 88 | LSM6 | LSM6 Entrez,  Source | LSM6 homolog, U6 small nuclear RNA associated (S. cerevisiae) | 18322 | -0.270 | -0.0796 | Yes |
| 89 | THOC4 | THOC4 Entrez,  Source | THO complex 4 | 18394 | -0.278 | -0.0572 | Yes |
| 90 | MAGOH | MAGOH Entrez,  Source | mago-nashi homolog, proliferation-associated (Drosophila) | 18430 | -0.282 | -0.0327 | Yes |
| 91 | TCERG1 | TCERG1 Entrez,  Source | transcription elongation regulator 1 | 18584 | -0.304 | -0.0122 | Yes |
| 92 | SF3A3 | SF3A3 Entrez,  Source | splicing factor 3a, subunit 3, 60kDa | 18600 | -0.307 | 0.0157 | Yes |
| 93 | SF3B3 | SF3B3 Entrez,  Source | splicing factor 3b, subunit 3, 130kDa | 18872 | -0.357 | 0.0351 | Yes |
Table: GSEA details [plain text format]

  

Fig 2: KEGG\_SPLICEOSOME      
 Blue-Pink O' Gram in the Space of the Analyzed GeneSet

  

Fig 3: KEGG\_SPLICEOSOME: Random ES distribution      
 Gene set null distribution of ES for **KEGG\_SPLICEOSOME**
