# Supplementary material for: Comprehensive analysis of β-catenin target genes in colorectal carcinoma cell lines with deregulated Wnt/β-catenin signaling
Source: BMC Genomics. 2014 Jan 28;15:74. doi: 10.1186/1471-2164-15-74 (PMC3909937; doi:10.1186/1471-2164-15-74)
Supplement: Additional file 5 — GSEA analysis using the KEGG pathway database. This zipped file contains confirming data of the GSEA analysis. The names of the directories containing the files were composed of the term ‘GSEA’, the name of the cell line, e.g. DLD1, SW480, or LS174T, and the pathway database (KEGG). Please use a web browser to view the files with the name ‘index.html’ in the corresponding directories to start exploring the data. [file 1471-2164-15-74-S5.zip › GSEA_LS174T_KEGG/KEGG_STARCH_AND_SUCROSE_METABOLISM.html]

Details for gene set KEGG\_STARCH\_AND\_SUCROSE\_METABOLISM[GSEA]

|  || Dataset | LS174T\_collapsed\_to\_symbols.class.cls#b\_versus\_bg.class.cls#b\_versus\_bg\_repos |
| Phenotype | class.cls#b\_versus\_bg\_repos |
| Upregulated in class | 1 |
| GeneSet | KEGG\_STARCH\_AND\_SUCROSE\_METABOLISM |
| Enrichment Score (ES) | 0.54734427 |
| Normalized Enrichment Score (NES) | 1.6182461 |
| Nominal p-value | 0.017274473 |
| FDR q-value | 0.098973826 |
| FWER p-Value | 0.654 |
Table: GSEA Results Summary

  

Fig 1: Enrichment plot: KEGG\_STARCH\_AND\_SUCROSE\_METABOLISM      
 Profile of the Running ES Score & Positions of GeneSet Members on the Rank Ordered List

  

| PROBE | GENE SYMBOL | GENE\_TITLE | RANK IN GENE LIST | RANK METRIC SCORE | RUNNING ES | CORE ENRICHMENT || 1 | UGT2B28 | UGT2B28 Entrez,  Source | UDP glucuronosyltransferase 2 family, polypeptide B28 | 15 | 1.479 | 0.1924 | Yes |
| 2 | UGT2B17 | UGT2B17 Entrez,  Source | UDP glucuronosyltransferase 2 family, polypeptide B17 | 21 | 1.429 | 0.3787 | Yes |
| 3 | UGT2B15 | UGT2B15 Entrez,  Source | UDP glucuronosyltransferase 2 family, polypeptide B15 | 70 | 0.989 | 0.5054 | Yes |
| 4 | GANC | GANC Entrez,  Source | glucosidase, alpha; neutral C | 491 | 0.420 | 0.5388 | Yes |
| 5 | PYGM | PYGM Entrez,  Source | phosphorylase, glycogen; muscle (McArdle syndrome, glycogen storage disease type V) | 1404 | 0.251 | 0.5249 | Yes |
| 6 | UGDH | UGDH Entrez,  Source | UDP-glucose dehydrogenase | 2056 | 0.203 | 0.5180 | Yes |
| 7 | PGM2L1 | PGM2L1 Entrez,  Source | phosphoglucomutase 2-like 1 | 2283 | 0.190 | 0.5312 | Yes |
| 8 | AGL | AGL Entrez,  Source | amylo-1, 6-glucosidase, 4-alpha-glucanotransferase (glycogen debranching enzyme, glycogen storage disease type III) | 2906 | 0.160 | 0.5203 | Yes |
| 9 | ENPP3 | ENPP3 Entrez,  Source | ectonucleotide pyrophosphatase/phosphodiesterase 3 | 2943 | 0.159 | 0.5392 | Yes |
| 10 | TREH | TREH Entrez,  Source | trehalase (brush-border membrane glycoprotein) | 3171 | 0.151 | 0.5473 | Yes |
| 11 | GAA | GAA Entrez,  Source | glucosidase, alpha; acid (Pompe disease, glycogen storage disease type II) | 4574 | 0.107 | 0.4895 | No |
| 12 | PGM1 | PGM1 Entrez,  Source | phosphoglucomutase 1 | 4693 | 0.104 | 0.4970 | No |
| 13 | UGT2A3 | UGT2A3 Entrez,  Source | UDP glucuronosyltransferase 2 family, polypeptide A3 | 4867 | 0.099 | 0.5010 | No |
| 14 | UGP2 | UGP2 Entrez,  Source | UDP-glucose pyrophosphorylase 2 | 4977 | 0.096 | 0.5080 | No |
| 15 | PYGB | PYGB Entrez,  Source | phosphorylase, glycogen; brain | 7266 | 0.049 | 0.3972 | No |
| 16 | UXS1 | UXS1 Entrez,  Source | UDP-glucuronate decarboxylase 1 | 7268 | 0.048 | 0.4034 | No |
| 17 | GCK | GCK Entrez,  Source | glucokinase (hexokinase 4, maturity onset diabetes of the young 2) | 7925 | 0.036 | 0.3746 | No |
| 18 | GYS2 | GYS2 Entrez,  Source | glycogen synthase 2 (liver) | 8032 | 0.034 | 0.3736 | No |
| 19 | HK3 | HK3 Entrez,  Source | hexokinase 3 (white cell) | 8792 | 0.021 | 0.3374 | No |
| 20 | GYS1 | GYS1 Entrez,  Source | glycogen synthase 1 (muscle) | 9348 | 0.012 | 0.3105 | No |
| 21 | HK2 | HK2 Entrez,  Source | hexokinase 2 | 9909 | 0.003 | 0.2821 | No |
| 22 | UGT1A9 | UGT1A9 Entrez,  Source | UDP glucuronosyltransferase 1 family, polypeptide A9 | 10361 | -0.005 | 0.2596 | No |
| 23 | HK1 | HK1 Entrez,  Source | hexokinase 1 | 11540 | -0.025 | 0.2026 | No |
| 24 | PYGL | PYGL Entrez,  Source | phosphorylase, glycogen; liver (Hers disease, glycogen storage disease type VI) | 11935 | -0.032 | 0.1865 | No |
| 25 | GPI | GPI Entrez,  Source | glucose phosphate isomerase | 12600 | -0.044 | 0.1583 | No |
| 26 | GUSB | GUSB Entrez,  Source | glucuronidase, beta | 12719 | -0.046 | 0.1583 | No |
| 27 | PGM2 | PGM2 Entrez,  Source | phosphoglucomutase 2 | 14035 | -0.073 | 0.1006 | No |
| 28 | G6PC2 | G6PC2 Entrez,  Source | glucose-6-phosphatase, catalytic, 2 | 14742 | -0.091 | 0.0762 | No |
| 29 | GBA3 | GBA3 Entrez,  Source | glucosidase, beta, acid 3 (cytosolic) | 16182 | -0.135 | 0.0201 | No |
| 30 | G6PC | G6PC Entrez,  Source | glucose-6-phosphatase, catalytic subunit | 16620 | -0.152 | 0.0176 | No |
| 31 | GBE1 | GBE1 Entrez,  Source | glucan (1,4-alpha-), branching enzyme 1 (glycogen branching enzyme, Andersen disease, glycogen storage disease type IV) | 16669 | -0.154 | 0.0352 | No |
| 32 | SI | SI Entrez,  Source | sucrase-isomaltase (alpha-glucosidase) | 17554 | -0.202 | 0.0164 | No |
| 33 | ENPP1 | ENPP1 Entrez,  Source | ectonucleotide pyrophosphatase/phosphodiesterase 1 | 18181 | -0.254 | 0.0175 | No |
| 34 | UGT2B4 | UGT2B4 Entrez,  Source | UDP glucuronosyltransferase 2 family, polypeptide B4 | 19031 | -0.405 | 0.0269 | No |
Table: GSEA details [plain text format]

  

Fig 2: KEGG\_STARCH\_AND\_SUCROSE\_METABOLISM      
 Blue-Pink O' Gram in the Space of the Analyzed GeneSet

  

Fig 3: KEGG\_STARCH\_AND\_SUCROSE\_METABOLISM: Random ES distribution      
 Gene set null distribution of ES for **KEGG\_STARCH\_AND\_SUCROSE\_METABOLISM**
