# Supplementary material for: Comprehensive analysis of β-catenin target genes in colorectal carcinoma cell lines with deregulated Wnt/β-catenin signaling
Source: BMC Genomics. 2014 Jan 28;15:74. doi: 10.1186/1471-2164-15-74 (PMC3909937; doi:10.1186/1471-2164-15-74)
Supplement: Additional file 5 — GSEA analysis using the KEGG pathway database. This zipped file contains confirming data of the GSEA analysis. The names of the directories containing the files were composed of the term ‘GSEA’, the name of the cell line, e.g. DLD1, SW480, or LS174T, and the pathway database (KEGG). Please use a web browser to view the files with the name ‘index.html’ in the corresponding directories to start exploring the data. [file 1471-2164-15-74-S5.zip › GSEA_LS174T_KEGG/KEGG_STEROID_HORMONE_BIOSYNTHESIS.html]

Details for gene set KEGG\_STEROID\_HORMONE\_BIOSYNTHESIS[GSEA]

|  || Dataset | LS174T\_collapsed\_to\_symbols.class.cls#b\_versus\_bg.class.cls#b\_versus\_bg\_repos |
| Phenotype | class.cls#b\_versus\_bg\_repos |
| Upregulated in class | 1 |
| GeneSet | KEGG\_STEROID\_HORMONE\_BIOSYNTHESIS |
| Enrichment Score (ES) | 0.6425038 |
| Normalized Enrichment Score (NES) | 2.0142043 |
| Nominal p-value | 0.0 |
| FDR q-value | 0.0021654961 |
| FWER p-Value | 0.0080 |
Table: GSEA Results Summary

  

Fig 1: Enrichment plot: KEGG\_STEROID\_HORMONE\_BIOSYNTHESIS      
 Profile of the Running ES Score & Positions of GeneSet Members on the Rank Ordered List

  

| PROBE | GENE SYMBOL | GENE\_TITLE | RANK IN GENE LIST | RANK METRIC SCORE | RUNNING ES | CORE ENRICHMENT || 1 | UGT2B28 | UGT2B28 Entrez,  Source | UDP glucuronosyltransferase 2 family, polypeptide B28 | 15 | 1.479 | 0.1218 | Yes |
| 2 | UGT2B17 | UGT2B17 Entrez,  Source | UDP glucuronosyltransferase 2 family, polypeptide B17 | 21 | 1.429 | 0.2399 | Yes |
| 3 | CYP3A5 | CYP3A5 Entrez,  Source | cytochrome P450, family 3, subfamily A, polypeptide 5 | 34 | 1.267 | 0.3443 | Yes |
| 4 | UGT2B15 | UGT2B15 Entrez,  Source | UDP glucuronosyltransferase 2 family, polypeptide B15 | 70 | 0.989 | 0.4245 | Yes |
| 5 | AKR1C2 | AKR1C2 Entrez,  Source | aldo-keto reductase family 1, member C2 (dihydrodiol dehydrogenase 2; bile acid binding protein; 3-alpha hydroxysteroid dehydrogenase, type III) | 195 | 0.656 | 0.4725 | Yes |
| 6 | AKR1C1 | AKR1C1 Entrez,  Source | aldo-keto reductase family 1, member C1 (dihydrodiol dehydrogenase 1; 20-alpha (3-alpha)-hydroxysteroid dehydrogenase) | 215 | 0.635 | 0.5241 | Yes |
| 7 | HSD17B2 | HSD17B2 Entrez,  Source | hydroxysteroid (17-beta) dehydrogenase 2 | 315 | 0.523 | 0.5624 | Yes |
| 8 | AKR1C3 | AKR1C3 Entrez,  Source | aldo-keto reductase family 1, member C3 (3-alpha hydroxysteroid dehydrogenase, type II) | 364 | 0.481 | 0.5998 | Yes |
| 9 | STS | STS Entrez,  Source | steroid sulfatase (microsomal), arylsulfatase C, isozyme S | 407 | 0.454 | 0.6353 | Yes |
| 10 | CYP1A1 | CYP1A1 Entrez,  Source | cytochrome P450, family 1, subfamily A, polypeptide 1 | 810 | 0.335 | 0.6425 | Yes |
| 11 | CYP17A1 | CYP17A1 Entrez,  Source | cytochrome P450, family 17, subfamily A, polypeptide 1 | 2298 | 0.189 | 0.5820 | No |
| 12 | CYP11B2 | CYP11B2 Entrez,  Source | cytochrome P450, family 11, subfamily B, polypeptide 2 | 4738 | 0.103 | 0.4655 | No |
| 13 | HSD17B8 | HSD17B8 Entrez,  Source | hydroxysteroid (17-beta) dehydrogenase 8 | 4859 | 0.099 | 0.4676 | No |
| 14 | UGT2A3 | UGT2A3 Entrez,  Source | UDP glucuronosyltransferase 2 family, polypeptide A3 | 4867 | 0.099 | 0.4754 | No |
| 15 | HSD3B2 | HSD3B2 Entrez,  Source | hydroxy-delta-5-steroid dehydrogenase, 3 beta- and steroid delta-isomerase 2 | 6122 | 0.070 | 0.4170 | No |
| 16 | CYP3A4 | CYP3A4 Entrez,  Source | cytochrome P450, family 3, subfamily A, polypeptide 4 | 6476 | 0.063 | 0.4041 | No |
| 17 | SRD5A1 | SRD5A1 Entrez,  Source | steroid-5-alpha-reductase, alpha polypeptide 1 (3-oxo-5 alpha-steroid delta 4-dehydrogenase alpha 1) | 6554 | 0.062 | 0.4053 | No |
| 18 | CYP1B1 | CYP1B1 Entrez,  Source | cytochrome P450, family 1, subfamily B, polypeptide 1 | 6737 | 0.058 | 0.4008 | No |
| 19 | CYP11B1 | CYP11B1 Entrez,  Source | cytochrome P450, family 11, subfamily B, polypeptide 1 | 6850 | 0.056 | 0.3998 | No |
| 20 | COMT | COMT Entrez,  Source | catechol-O-methyltransferase | 6989 | 0.053 | 0.3971 | No |
| 21 | HSD17B12 | HSD17B12 Entrez,  Source | hydroxysteroid (17-beta) dehydrogenase 12 | 7045 | 0.052 | 0.3987 | No |
| 22 | HSD3B1 | HSD3B1 Entrez,  Source | hydroxy-delta-5-steroid dehydrogenase, 3 beta- and steroid delta-isomerase 1 | 7602 | 0.042 | 0.3737 | No |
| 23 | HSD11B1 | HSD11B1 Entrez,  Source | hydroxysteroid (11-beta) dehydrogenase 1 | 8289 | 0.029 | 0.3409 | No |
| 24 | HSD17B1 | HSD17B1 Entrez,  Source | hydroxysteroid (17-beta) dehydrogenase 1 | 9033 | 0.017 | 0.3042 | No |
| 25 | CYP7A1 | CYP7A1 Entrez,  Source | cytochrome P450, family 7, subfamily A, polypeptide 1 | 9152 | 0.015 | 0.2994 | No |
| 26 | SULT1E1 | SULT1E1 Entrez,  Source | sulfotransferase family 1E, estrogen-preferring, member 1 | 9924 | 0.002 | 0.2601 | No |
| 27 | UGT1A9 | UGT1A9 Entrez,  Source | UDP glucuronosyltransferase 1 family, polypeptide A9 | 10361 | -0.005 | 0.2381 | No |
| 28 | SRD5A2 | SRD5A2 Entrez,  Source | steroid-5-alpha-reductase, alpha polypeptide 2 (3-oxo-5 alpha-steroid delta 4-dehydrogenase alpha 2) | 10798 | -0.012 | 0.2167 | No |
| 29 | CYP7B1 | CYP7B1 Entrez,  Source | cytochrome P450, family 7, subfamily B, polypeptide 1 | 11058 | -0.016 | 0.2048 | No |
| 30 | AKR1C4 | AKR1C4 Entrez,  Source | aldo-keto reductase family 1, member C4 (chlordecone reductase; 3-alpha hydroxysteroid dehydrogenase, type I; dihydrodiol dehydrogenase 4) | 11413 | -0.023 | 0.1886 | No |
| 31 | AKR1D1 | AKR1D1 Entrez,  Source | aldo-keto reductase family 1, member D1 (delta 4-3-ketosteroid-5-beta-reductase) | 11850 | -0.031 | 0.1688 | No |
| 32 | CYP19A1 | CYP19A1 Entrez,  Source | cytochrome P450, family 19, subfamily A, polypeptide 1 | 12396 | -0.040 | 0.1442 | No |
| 33 | HSD17B7 | HSD17B7 Entrez,  Source | hydroxysteroid (17-beta) dehydrogenase 7 | 12941 | -0.050 | 0.1205 | No |
| 34 | CYP21A2 | CYP21A2 Entrez,  Source | cytochrome P450, family 21, subfamily A, polypeptide 2 | 13156 | -0.054 | 0.1140 | No |
| 35 | HSD17B3 | HSD17B3 Entrez,  Source | hydroxysteroid (17-beta) dehydrogenase 3 | 13705 | -0.066 | 0.0914 | No |
| 36 | CYP3A7 | CYP3A7 Entrez,  Source | cytochrome P450, family 3, subfamily A, polypeptide 7 | 14947 | -0.095 | 0.0357 | No |
| 37 | SULT2B1 | SULT2B1 Entrez,  Source | sulfotransferase family, cytosolic, 2B, member 1 | 15539 | -0.112 | 0.0147 | No |
| 38 | CYP3A43 | CYP3A43 Entrez,  Source | cytochrome P450, family 3, subfamily A, polypeptide 43 | 17928 | -0.230 | -0.0886 | No |
| 39 | CYP11A1 | CYP11A1 Entrez,  Source | cytochrome P450, family 11, subfamily A, polypeptide 1 | 18514 | -0.294 | -0.0942 | No |
| 40 | UGT2B4 | UGT2B4 Entrez,  Source | UDP glucuronosyltransferase 2 family, polypeptide B4 | 19031 | -0.405 | -0.0870 | No |
| 41 | HSD11B2 | HSD11B2 Entrez,  Source | hydroxysteroid (11-beta) dehydrogenase 2 | 19425 | -0.665 | -0.0520 | No |
| 42 | HSD17B6 | HSD17B6 Entrez,  Source | hydroxysteroid (17-beta) dehydrogenase 6 | 19454 | -0.708 | 0.0052 | No |
Table: GSEA details [plain text format]

  

Fig 2: KEGG\_STEROID\_HORMONE\_BIOSYNTHESIS      
 Blue-Pink O' Gram in the Space of the Analyzed GeneSet

  

Fig 3: KEGG\_STEROID\_HORMONE\_BIOSYNTHESIS: Random ES distribution      
 Gene set null distribution of ES for **KEGG\_STEROID\_HORMONE\_BIOSYNTHESIS**
