# Supplementary material for: Comprehensive analysis of β-catenin target genes in colorectal carcinoma cell lines with deregulated Wnt/β-catenin signaling
Source: BMC Genomics. 2014 Jan 28;15:74. doi: 10.1186/1471-2164-15-74 (PMC3909937; doi:10.1186/1471-2164-15-74)
Supplement: Additional file 5 — GSEA analysis using the KEGG pathway database. This zipped file contains confirming data of the GSEA analysis. The names of the directories containing the files were composed of the term ‘GSEA’, the name of the cell line, e.g. DLD1, SW480, or LS174T, and the pathway database (KEGG). Please use a web browser to view the files with the name ‘index.html’ in the corresponding directories to start exploring the data. [file 1471-2164-15-74-S5.zip › GSEA_LS174T_KEGG/KEGG_THYROID_CANCER.html]

Details for gene set KEGG\_THYROID\_CANCER[GSEA]

|  || Dataset | LS174T\_collapsed\_to\_symbols.class.cls#b\_versus\_bg.class.cls#b\_versus\_bg\_repos |
| Phenotype | class.cls#b\_versus\_bg\_repos |
| Upregulated in class | 0 |
| GeneSet | KEGG\_THYROID\_CANCER |
| Enrichment Score (ES) | -0.45830488 |
| Normalized Enrichment Score (NES) | -1.4065524 |
| Nominal p-value | 0.060344826 |
| FDR q-value | 0.21453753 |
| FWER p-Value | 0.974 |
Table: GSEA Results Summary

  

Fig 1: Enrichment plot: KEGG\_THYROID\_CANCER      
 Profile of the Running ES Score & Positions of GeneSet Members on the Rank Ordered List

  

| PROBE | GENE SYMBOL | GENE\_TITLE | RANK IN GENE LIST | RANK METRIC SCORE | RUNNING ES | CORE ENRICHMENT || 1 | TCF7L1 | TCF7L1 Entrez,  Source | transcription factor 7-like 1 (T-cell specific, HMG-box) | 461 | 0.429 | 0.0625 | No |
| 2 | TCF7L2 | TCF7L2 Entrez,  Source | transcription factor 7-like 2 (T-cell specific, HMG-box) | 2119 | 0.199 | 0.0176 | No |
| 3 | KRAS | KRAS Entrez,  Source | v-Ki-ras2 Kirsten rat sarcoma viral oncogene homolog | 2305 | 0.189 | 0.0460 | No |
| 4 | CDH1 | CDH1 Entrez,  Source | cadherin 1, type 1, E-cadherin (epithelial) | 2701 | 0.169 | 0.0597 | No |
| 5 | NRAS | NRAS Entrez,  Source | neuroblastoma RAS viral (v-ras) oncogene homolog | 2918 | 0.160 | 0.0808 | No |
| 6 | TFG | TFG Entrez,  Source | TRK-fused gene | 3478 | 0.140 | 0.0803 | No |
| 7 | MAPK3 | MAPK3 Entrez,  Source | mitogen-activated protein kinase 3 | 4947 | 0.097 | 0.0245 | No |
| 8 | PPARG | PPARG Entrez,  Source | peroxisome proliferative activated receptor, gamma | 5150 | 0.092 | 0.0326 | No |
| 9 | NCOA4 | NCOA4 Entrez,  Source | nuclear receptor coactivator 4 | 5416 | 0.086 | 0.0363 | No |
| 10 | MAP2K1 | MAP2K1 Entrez,  Source | mitogen-activated protein kinase kinase 1 | 8349 | 0.028 | -0.1082 | No |
| 11 | RET | RET Entrez,  Source | ret proto-oncogene (multiple endocrine neoplasia and medullary thyroid carcinoma 1, Hirschsprung disease) | 8361 | 0.028 | -0.1031 | No |
| 12 | RXRB | RXRB Entrez,  Source | retinoid X receptor, beta | 8977 | 0.018 | -0.1311 | No |
| 13 | RXRA | RXRA Entrez,  Source | retinoid X receptor, alpha | 10297 | -0.003 | -0.1980 | No |
| 14 | HRAS | HRAS Entrez,  Source | v-Ha-ras Harvey rat sarcoma viral oncogene homolog | 10338 | -0.004 | -0.1992 | No |
| 15 | TPM3 | TPM3 Entrez,  Source | tropomyosin 3 | 11224 | -0.019 | -0.2406 | No |
| 16 | RXRG | RXRG Entrez,  Source | retinoid X receptor, gamma | 11789 | -0.029 | -0.2636 | No |
| 17 | TPR | TPR Entrez,  Source | translocated promoter region (to activated MET oncogene) | 11954 | -0.032 | -0.2655 | No |
| 18 | BRAF | BRAF Entrez,  Source | v-raf murine sarcoma viral oncogene homolog B1 | 12037 | -0.033 | -0.2630 | No |
| 19 | CCDC6 | CCDC6 Entrez,  Source | coiled-coil domain containing 6 | 12146 | -0.036 | -0.2614 | No |
| 20 | PAX8 | PAX8 Entrez,  Source | paired box gene 8 | 12654 | -0.045 | -0.2783 | No |
| 21 | MAPK1 | MAPK1 Entrez,  Source | mitogen-activated protein kinase 1 | 13089 | -0.053 | -0.2899 | No |
| 22 | MAP2K2 | MAP2K2 Entrez,  Source | mitogen-activated protein kinase kinase 2 | 15495 | -0.111 | -0.3908 | No |
| 23 | NTRK1 | NTRK1 Entrez,  Source | neurotrophic tyrosine kinase, receptor, type 1 | 16101 | -0.131 | -0.3954 | No |
| 24 | TP53 | TP53 Entrez,  Source | tumor protein p53 (Li-Fraumeni syndrome) | 17097 | -0.175 | -0.4113 | Yes |
| 25 | MYC | MYC Entrez,  Source | v-myc myelocytomatosis viral oncogene homolog (avian) | 18016 | -0.238 | -0.4106 | Yes |
| 26 | CCND1 | CCND1 Entrez,  Source | cyclin D1 | 18184 | -0.254 | -0.3681 | Yes |
| 27 | TCF7 | TCF7 Entrez,  Source | transcription factor 7 (T-cell specific, HMG-box) | 19081 | -0.424 | -0.3290 | Yes |
| 28 | LEF1 | LEF1 Entrez,  Source | lymphoid enhancer-binding factor 1 | 19108 | -0.438 | -0.2424 | Yes |
| 29 | CTNNB1 | CTNNB1 Entrez,  Source | catenin (cadherin-associated protein), beta 1, 88kDa | 19553 | -1.322 | 0.0002 | Yes |
Table: GSEA details [plain text format]

  

Fig 2: KEGG\_THYROID\_CANCER      
 Blue-Pink O' Gram in the Space of the Analyzed GeneSet

  

Fig 3: KEGG\_THYROID\_CANCER: Random ES distribution      
 Gene set null distribution of ES for **KEGG\_THYROID\_CANCER**
